# Machine Learning to Develop Peptide Catalysts—Successes, Limitations, and Opportunities

**DOI:** 10.1021/acscentsci.3c01284

**Published:** 2024-02-05

**Authors:** Tobias Schnitzer, Martin Schnurr, Andrew F. Zahrt, Nader Sakhaee, Scott E. Denmark, Helma Wennemers

**Affiliations:** † Laboratory of Organic Chemistry, ETH Zurich, D-CHAB, Vladimir-Prelog-Weg 3, 8093 Zurich, Switzerland; ‡ Roger Adams Laboratory, Department of Chemistry, University of Illinois, Urbana, Illinois 61801, United States

## Abstract

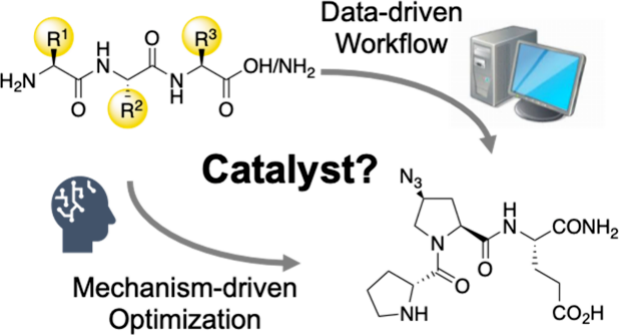

Peptides have been established as modular catalysts for various transformations. Still, the vast number of potential amino acid building blocks renders the identification of peptides with desired catalytic activity challenging. Here, we develop a machine-learning workflow for the optimization of peptide catalysts. First—in a hypothetical competition—we challenged our workflow to identify peptide catalysts for the conjugate addition reaction of aldehydes to nitroolefins and compared the performance of the predicted structures with those optimized in our laboratory. On the basis of the positive results, we established a universal training set (UTS) containing 161 catalysts to sample an *in silico* library of ∼30,000 tripeptide members. Finally, we challenged our machine learning strategy to identify a member of the library as a stereoselective catalyst for an annulation reaction that has not been catalyzed by a peptide thus far. We conclude with a comparison of data-driven versus expert-knowledge-guided peptide catalyst optimization.

## Abstract 2

Statistical learning methods were challenged to identify enantioselective peptide catalysts from a 30,000-member *in silico* library and compared with expert-knowledge-guided methods.

## Introduction

The development of efficient catalytic methods is central in chemical research. An often tedious and time-consuming step is the optimization of the catalyst structure. Linking a catalyst structure to its activity and stereoselectivity is difficult, as capturing the dynamics of a molecule is inherently challenging.^1^ Methods to describe the dynamic behavior of a catalyst alongside a strategy to implement the conformational properties in a systematic catalyst optimization are hence highly desirable.

Recent advances in data-driven approaches and mathematical modeling have succeeded in predicting the reactivity and stereoselectivity of small-molecule catalysts.^2−6^ One key aspect of the success of these endeavors is the numerical representation of the molecules of interest. In enantioselective catalysis, this means capturing the important structural elements responsible for stereoinduction. A critical molecular property for catalyst development is conformational flexibility.^1,7,8^ Although challenging, a number of studies have indicated that incorporating conformational flexibility into the numerical representation improves the predictive power of mathematical models.^9−11^

**Figure 1:**
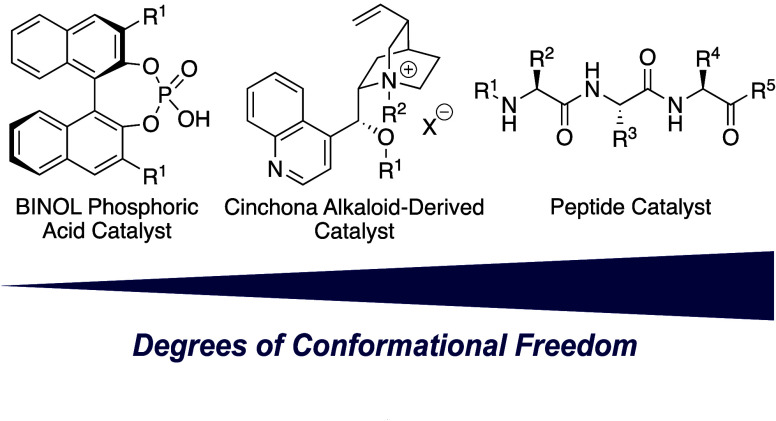
Degrees of conformational freedom of peptide catalyst in the context of other small-molecule catalysts.

So far, such mathematical models have provided impressive success in improving small-molecule catalysts.^12^ In this study, we interrogated whether conformer-dependent representations can assist in catalyst design for tripeptide catalysts. Such organocatalysts have a similar molecular weight but many more degrees of conformational freedom compared to conventional small-molecule catalysts (Figure 1). This dynamic behavior makes this catalyst family a unique challenge and allows for probing the limits of current machine-learning approaches.

Over the past two decades, peptides have been established as potent organocatalysts.^13,14^ Their structure is modular, and automated, solid-phase synthesis allows for straightforward access to a diverse array of peptides. This ease of access to many structurally and functionally different peptides makes this class of catalysts—in case their many different conformational states with different functions can be successfully represented—ideal for using machine learning and other computer-aided tools for catalyst optimization.

Previously, Sigman and Miller successfully constructed linear regression models to predict reaction outcomes for peptide-catalyzed reactions.^15^ These studies used modeling retrospectively to interrogate the reaction mechanism and identify those catalyst properties responsible for stereoinduction. In this work, we were primarily interested in: (1) using unsupervised learning to create an ideal screening set of tripeptide catalysts and (2) making *a priori* predictions and experimental validation of superior catalyst structures. Namely, we planned to use mathematical modeling to select a superior-performing catalyst from a large pool of *in silico* catalyst candidates. This approach has successfully been applied to predict synthetic catalyst performance through the application of 4D- and 3.5-QSAR concepts.^3^ These conformer-dependent, grid-based representations, known as Average Steric Occupancy (ASO) and Average Electronic Indicator Field (AEIF) descriptors, have been used to represent cinchona alkaloids, amino alcohol-transition metal complexes, and chiral Brønsted acids.^16,17^

Here, we extended these descriptors to peptide catalysis and evaluated whether they are capable of representing catalysts with greater conformational flexibility.

## Results and Discussion

**Figure 2:**
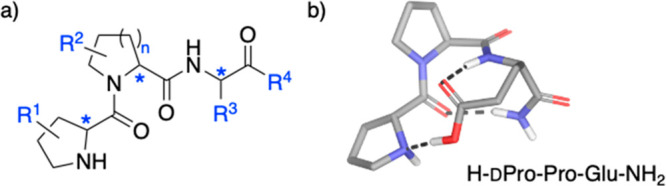
(a) Pro-Pro-Xaa-type peptide catalysts and (b) ground-state structure of H-dPro-Pro-Glu-NH_2_ (ref (7)).

As a testing ground for machine-learning-guided peptide catalyst optimization, we used Pro-Pro-Xaa-type peptides (Figure 2a). These tripeptides are powerful catalysts for stereoselective C–C bond formations that proceed through an enamine intermediate.^13,14^ Compared to other chiral amine-based catalysts, Pro-Pro-Xaa peptides stand out for their modularity. This feature allows for structural tuning and, thus, stereoselective reactions adjusted to the steric and stereoelectronic properties of the substrates of interest. As a result, human-guided catalyst optimization methods established Pro-Pro-Xaa catalysts that provide stereoselective access to a range of different addition products at catalyst loadings of as low as 0.05 mol %.^18^ Examples include aldol^19^ and conjugate addition reactions with a diverse range of electrophiles, including nitroolefins,^18,20−25^ maleimides,^26,27^ dicyanoolefins,^28^ allenamides,^29^ and vinyl triflones.^30^

These conventional catalyst development studies were guided by detailed conformational and mechanistic studies that revealed, for example, the importance of a balance between flexibility and rigidity,^7^ the *trans*/*cis* ratio of the tertiary amide,^18,31^*endo*-N-pyramidalization of the enamine intermediate,^32,33^ and the role of an internal proton donor for the performance of the tripeptide catalysts.^34^ Among the many Pro-Pro-Xaa catalysts identified for different reactions, H-dPro-Pro-Glu-NH_2_ features a rigid ground state,^7^ but all others (e.g., H-Pro-Pro-Asp-NH_2_, H-dPro-Pro-Gln-OH, H-dPro-Pro-Gln-OH) are more flexible.

### Can Machine Learning Predict an Experimentally Optimized Peptide Catalyst?

**Scheme 1:**
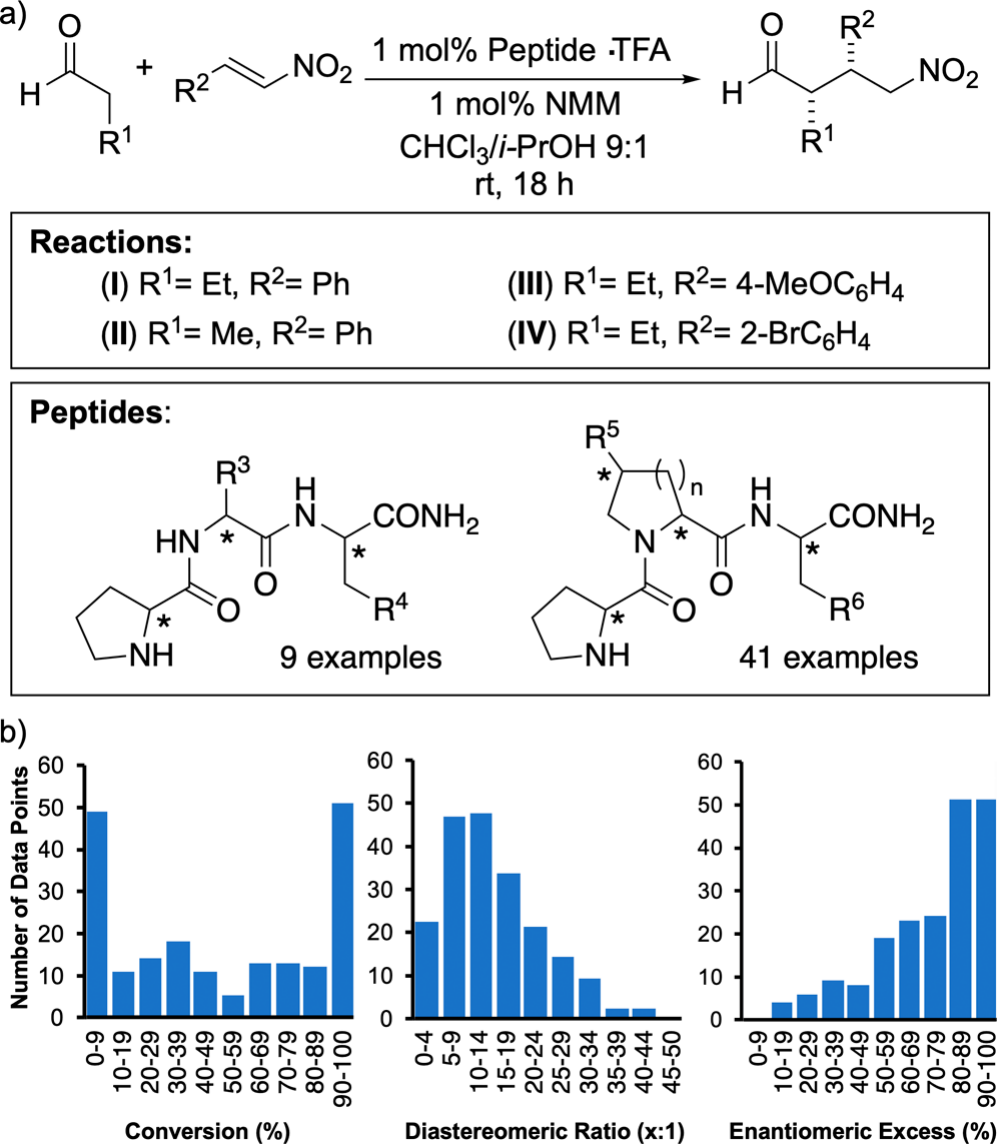
(a) Conjugate Addition Reactions and Catalysts Used for the Machine-Learning Study and (b) Distribution of Conversion to Product (left), Diastereomeric Ratio of the *syn* Diastereomers (middle), and Enantiomeric Excess of the *syn* Enantiomers in the 200 Catalytic Reactions (right)

At the outset of the study, we asked whether a machine-learning-guided catalyst optimization procedure could identify the same peptide catalysts that were previously obtained by traditional, human-guided optimization methods.^18,31^ To answer this question, we used the conjugate addition of aldehydes to β-nitroolefins as a model reaction (Scheme 1a). We chose this reaction since the so far optimal catalysts H-dPro-Pro-Glu-NH_2_, H-dPro-Pip-Glu-NH_2_ (Pip = l-piperidine-2-carboxylic acid), and H-dPro-MePro-Glu-NH_2_ (MePro = α-methyl-proline), and analogues thereof, adopt a single, well-defined conformation in the ground state (Figure 2b).^7,18,31^ This rigid structure arises from a β-turn, an intramolecular salt bridge, and two additional H-bonds.^7^ The structure becomes more flexible upon enamine formation and toggles back and forth between rigid and flexible conformations throughout the catalytic cycle.^7^ Thus, the ground-state rigidity of these tripeptides should ease the challenge of the machine-learning approach.

Specifically, we used the addition reactions of butanal to (*E*)-β-nitrostyrene (**I**), (*E*)-4-methoxy-β-nitrostyrene (**III**), and (*E*)-2-bromo-β-nitrostyrene (**IV**) and propanal to (*E*)-β-nitrostyrene (**II**), performed under previously optimized conditions (1 mol % peptide TFA salt, 1 mol % N-methylmorpholine (NMM), CHCl_3_/*i-*PrOH, 9:1, 20 °C, 18 h), as trial reactions.^18^ Fifty peptides that were available in our laboratories and that contained an N-terminal dPro residue were used to establish a data set encompassing conversion to product and dia- and enantioselectivity for each reaction. Forty one of those peptides contain an N-terminal dPro-Pro motif, and nine contain an amino acid other than Pro in the middle position. Of note, one of the optimal peptides, H-dPro-Pip-Glu-NH_2_, was not included in the set of catalysts.^18^

The 200 experiments (50 peptides × 4 reactions) provided a spread of both conversions and product stereoselectivities (Scheme 1b). Half of the experiments proceeded with conversions between 10% and 90%. In approximately 25% of the reactions, less than 10% conversion to product was observed (mainly catalysts without a Pro-Pro motif), and in 25% of the reactions, the γ-nitroaldehyde formed almost quantitatively (mostly catalysts with a Pro-Pro motif and an internal acid). The enantioselectivity ranged from 10% ee to 98% ee and the diastereoselectivity from 58:42 to 98:2 *syn*/*anti*.

**Figure 3:**
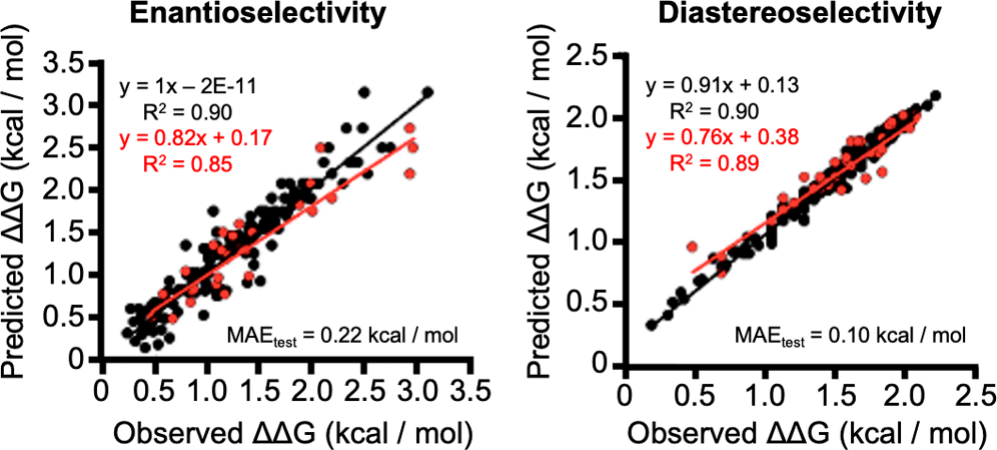
Enantioselectivity and diastereoselectivity predicted vs observed. Black points are training data; red points are the test set.

With the data set in hand, we evaluated whether our previously established ASO and AEIF descriptors can be used for peptide catalysts.^16^ These descriptors seek to capture the dynamic, steric, and electronic properties of the catalysts in 3D-space with respect to a common structural motif (in this case, the first proline residue). Because tripeptides contain many more rotational degrees of freedom compared to previously modeled small-molecule catalysts,^3,16,17,35^ they represent a more rigorous test of how well the ASO and AEIF descriptors capture the dynamic nature of catalyst scaffolds (see the Supporting Information for descriptor calculations). To validate the representation, ten catalysts (with each of their associated reactions) were left out of the training set to ensure out-of-sample catalysts in the test set (Figure 3). Nine of these catalysts were selected randomly—as the tenth catalyst, we chose the peptide which provided the highest conversion and stereoselectivity.

Two separate modeling tasks were undertaken, and Projection to Latent Structure (PLS) models were used to predict both diastereo- and enantioselectivity. These methods constructed excellent models for both enantioselectivity (MAE_test_ = 0.22 kcal/mmol) and diastereoselectivity (MAE_test_ = 0.10 kcal/mol). Further, the two most stereoselective catalysts, H-dPro-Pip-Glu-NH_2_ and H-dPro-MePro-Glu-NH_2_, were predicted to be, on average, the most enantioselective catalysts (predicted 97% ee, observed 97% ee on average for the top three reactions with these catalysts). This outcome is remarkable, as these two tripeptides are the best-performing catalysts for conjugate addition reactions between aldehydes and β-nitroolefins.^18,31^ This excellent match between the traditional, mechanism-guided and modeling-guided results indicated that the descriptors used in this case study constituted a valid representation for tripeptide catalysts that feature a well-defined ground-state conformation.

### Can Machine Learning Predict an As-Yet-Unknown Peptide Catalyst?

Next, we asked whether a machine-learning-guided procedure could identify a highly stereoselective peptide catalyst for a new reaction. This endeavor is a significantly greater challenge since most Pro-Pro-Xaa and Pro-Xaa-Yaa peptides other than H-dPro-Pro-Glu-NH_2_, and analogues thereof, cannot form an intramolecular salt bridge and feature greater conformational space.

This next stage required the construction of a large *in silico* library of tripeptide catalysts. To ensure accessibility of all potential catalysts, a database of 174 commercially available amino acids was compiled. To limit the domain space, the first residue was limited to dPro. By generating tripeptides in which any of the 174 amino acids can be present at the middle or C-terminal positions, a 30,276-member *in silico* library of potential tripeptide catalysts was constructed. A critical element of the ML workflow is to then select from this library a representative set of molecules, called a Universal Training Set (UTS).^3^ For this study, the UTS was identified by the use of unsupervised learning methods that have recently been featured in data science applied to catalysis.^17,6,36^ To achieve the most rapid avenue for the construction of a large training set, we clustered the 174 amino acids and selected representatives for peptide synthesis. This process involved calculating ASO and AEIF descriptors for the 174 individual amino acids. These descriptors were flattened, concatenated, and scaled, and the dimensionality of the resultant feature vector was reduced by removing dimensions with zero variance or with high correlation. Finally, this space was reduced to the first 20 principal components (∼87% explained variance).

**Figure 4:**
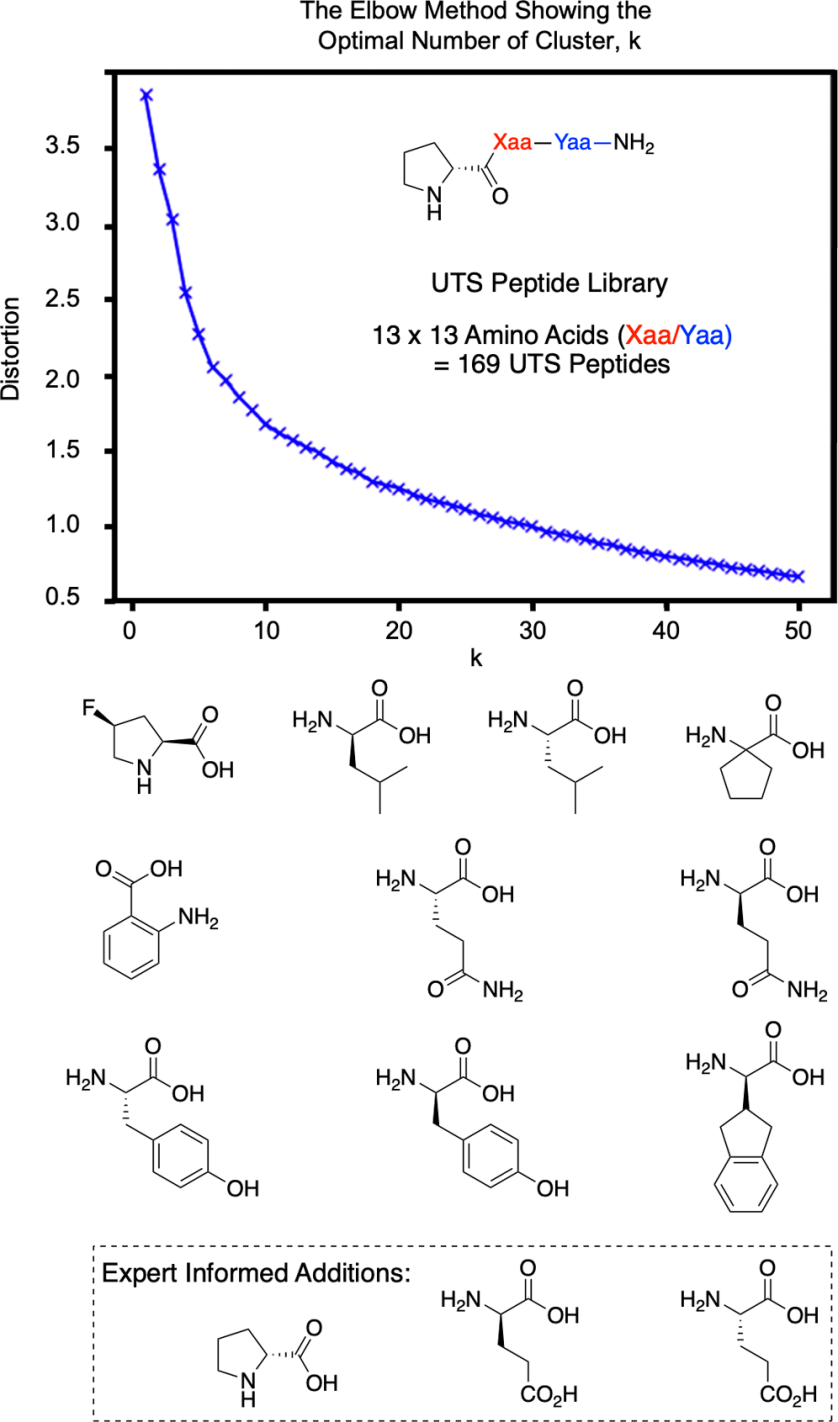
Elbow plot informing number of clusters and selected amino acids (exemplars of each cluster) for combinatorial construction of the tripeptide UTS.

The amino acid space thus created was then clustered using the *K*-means algorithm with the number of clusters set to 1–50 as informed by the elbow method.^37^ The distortion of each cluster was calculated and plotted to produce an elbow plot (Figure 4). In this analysis, distortion is a measure used to determine how much the variation between clusters changes as more clusters are selected. Small changes in distortion per cluster indicate that the variation between clusters changes very little when a new cluster is added. This outcome can be interpreted as “diminishing returns” for each new compound added to the set. Although the plot lacks distinctive elbows (indicating a clear cutoff), a sharp decline is noted until six clusters are identified. We selected a range of 6–15 to explore. Prospective training sets for this range were selected by identifying the amino acids nearest to the cluster centroids and using them as exemplars for each cluster. These sets were then qualitatively evaluated by considering at which point the cluster exemplars seemed redundant from a chemical perspective. Using this qualitative analysis, ten cluster exemplars were identified as optimal.

In addition to these ten algorithmically selected representatives, three more amino acids were added manually. The rationale for this manual addition is twofold: (1) from expert knowledge, we added amino acids that conferred high reactivity and stereoselectivity in previous studies,^18−34^ and (2) the three additional amino acids were expected to be similar to some of the algorithmically selected compounds (according to raw ASO/AEIF, dimensionality-reduced space) but may behave differently in chemical systems. For example, the algorithmically chosen glutamine (Gln) residues are related to manually chosen glutamic acid (Glu) residues, but the amide and carboxylic acid groups have different chemical properties. As such, these additions would be important for supervised methods to learn the difference between these related functionalities (see the Supporting Information).

**Scheme 2:**
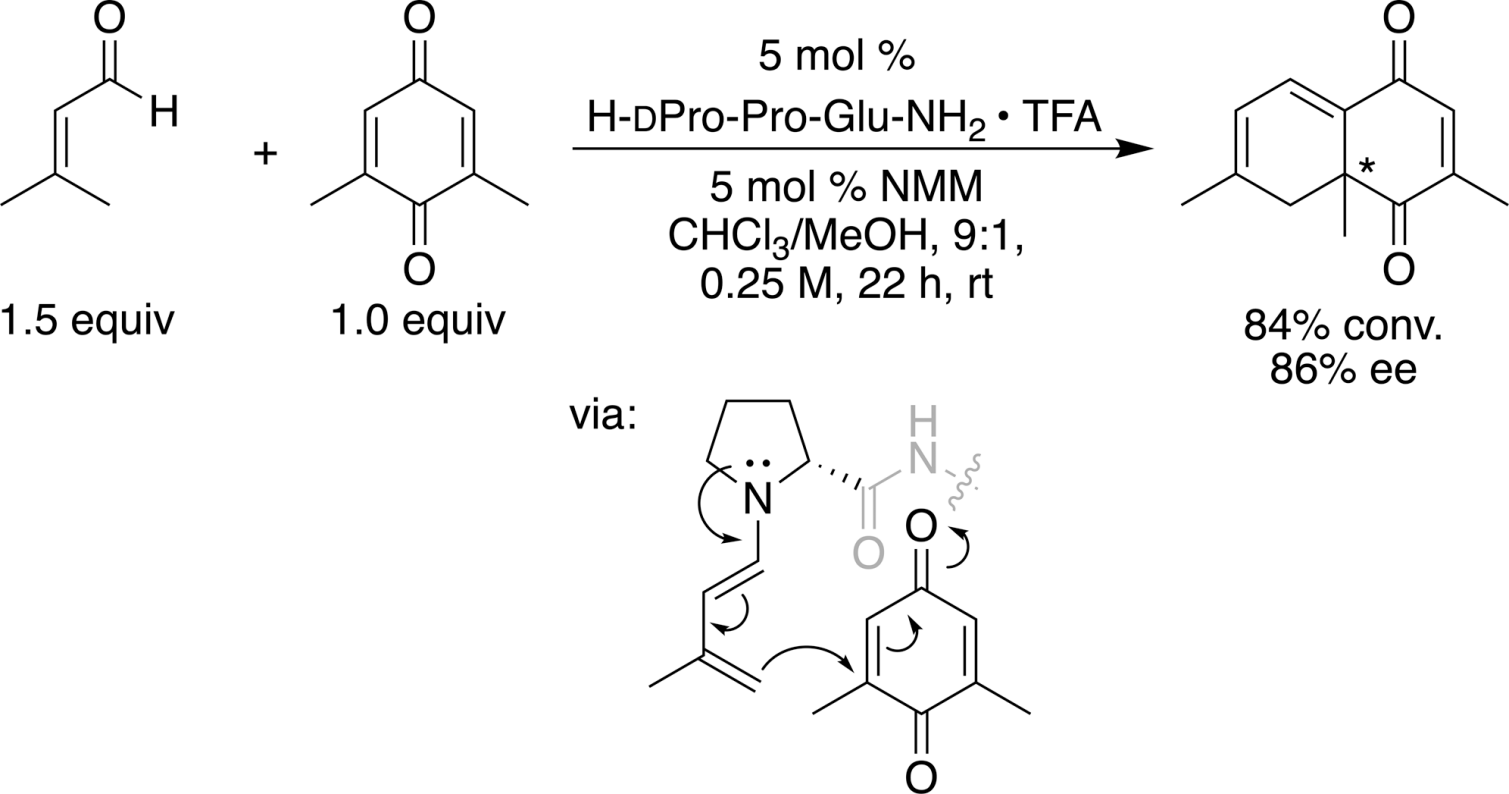
Preoptimization of the Peptide-Catalyzed Annulation Reaction

In view of the demonstrated success of the tripeptide catalysts in nitroolefin conjugate addition reactions, the chemoinformatic workflow was challenged to predict a stereoselective catalyst for a different chemical transformation. So far, the developed tripeptide catalysts focused on reactions that proceed via an enamine intermediate. Now, we focused on a reaction that relies on carbonyl activation via a dienamine species from an α,β-unsaturated aldehyde. Specifically, we selected the annulation of senecialdehyde and 2,6-dimethylquinone as a model, a reaction that can be catalyzed with chiral amines (Scheme 2).^38^ This reaction was chosen because the annulation proceeds via a dienamine, not an enamine, intermediate and therefore may require a peptide catalyst with a reactivity that has so far not been explored. Thus, this annulation reaction poses a greater challenge to the ML workflow. Furthermore, the reaction takes place at ambient conditions and yields stable UV active enantiomeric products that can be easily analyzed by chiral stationary phase with supercritical fluid chromatography. These practical considerations are important since the workflow requires high-throughput reaction analysis.

In an initial test reaction, equimolar amounts of senecialdehyde and 2,6-dimethylquinone formed in the presence of 5 mol % H-dPro-Pro-Glu-NH_2_ the annulation product quantitatively with 57% ee in MeOH. Variations of the reaction parameters (15 different solvents, concentration, and reagent stoichiometries; see the Supporting Information for details) identified CHCl_3_/MeOH (9:1), a concentration of 0.25 M, and a slight excess of aldehyde (1.5 equiv) as optimal reaction conditions. Under these conditions, 84% conversion to the product was observed at room temperature within 22 h with 86% ee.^39^

**Figure 5:**
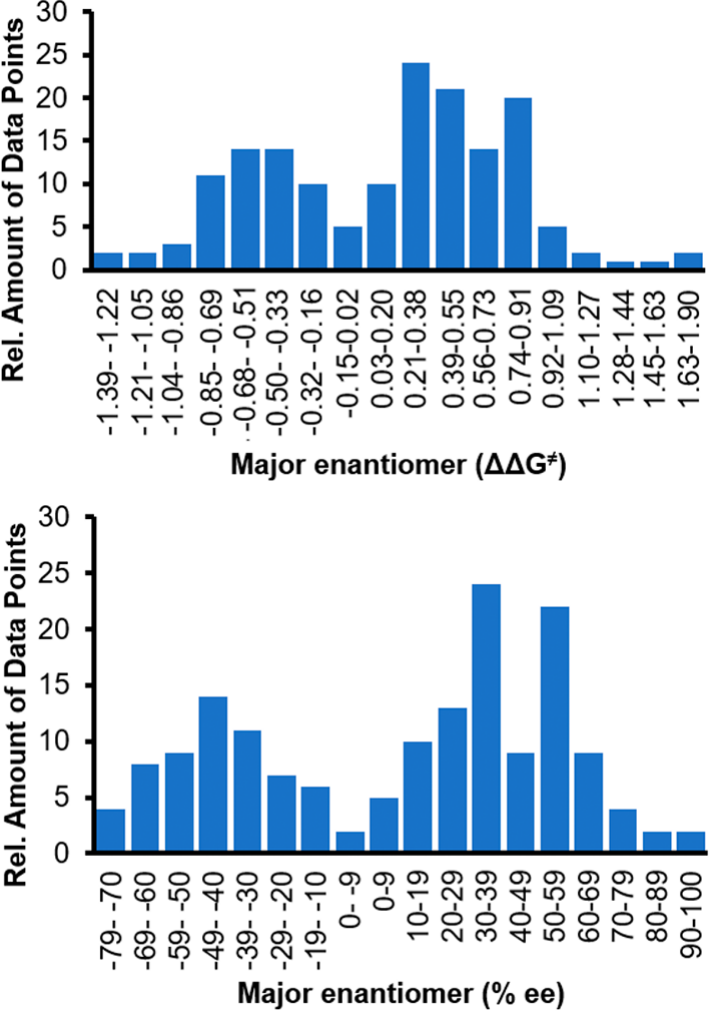
Graph depicting the distribution of selectivity values gathered from the UTS (skew = −0.08, kurtosis = −0.49).

Building on this base, we prepared 161 out of the proposed 169 peptides of the UTS by solid-phase peptide synthesis. Owing to repeated, incomplete amino acid couplings, we excluded the eight remaining peptides from the final training set. The catalytic properties of the 161-member UTS were then tested in the model reaction. The most stereoselective peptide, H-dPro-(4*S*)Flp-Glu-NH_2_,^40^ provided the product with an improved enantioselectivity of 91% ee (Δ = +5% ee). Significantly, the mechanism-agnostic selection protocols sample a wide range of reactivity space, providing a normal distribution of selectivity data (Kolmogorov–Smirnov *p*-value = 0.07315). This distribution, along with the identification of a good lead catalyst, facilitates subsequent modeling endeavors for catalyst structure refinement. These results also support the hypothesis that this set of molecules is an ideal starting point for any reaction which can be catalyzed by tripeptides, owing to the reaction- and mechanism-agnostic selection protocol. A summary of the distribution of stereoselectivities is depicted in Figure 5.

**Figure 6:**
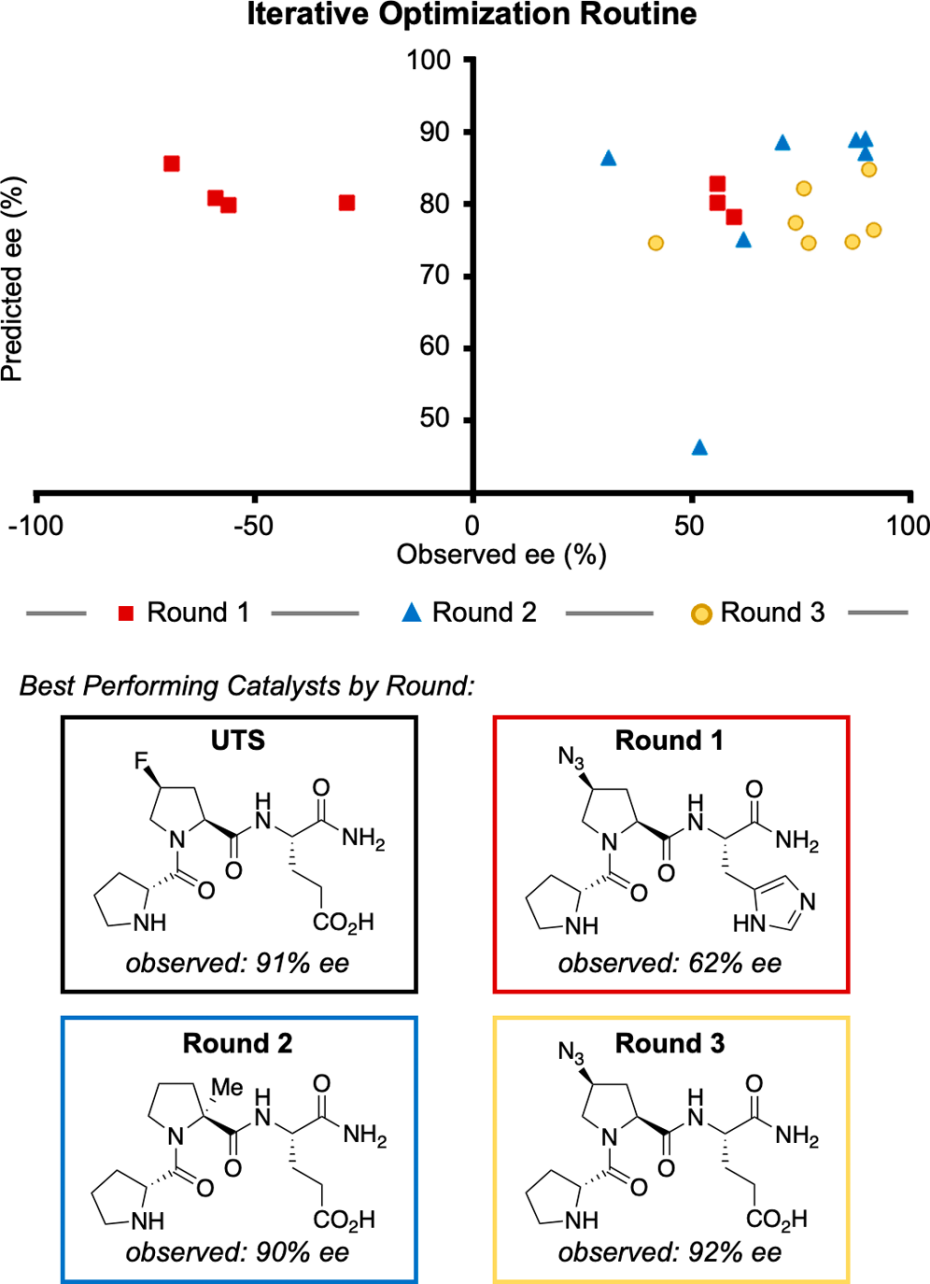
Iterative optimization process with three rounds of predictions.

With a data set exhibiting a wide range of selectivities and relatively normal distribution, a variety of machine learning methods were tested in an effort to discover a more selective catalyst (Figure 6; for a full description of the methods, see the Supporting Information). Three rounds of predictions were made. In the first round of predictions, neural networks were constructed, and catalysts were selected only on the basis of their predicted selectivity without regard to prediction confidence. These predictions can be thought of as “high-risk, high-reward” targets. Notably, the first round of predictions was poor (predicted, ∼80% ee; experimental, 62% ee). Predictive accuracy of later iterations was achieved by using simpler models and including prediction certainty as a selection criterion. This modification resulted in iterative, predictive improvement over the subsequent rounds (for an in-depth discussion, see the Supporting Information). In two additional iterations, a catalyst bearing an azide instead of a fluorine substituent, H-dPro-(4*S*)Azp-Glu-NH_2_,^40^ with marginal improvement (+1% ee) over the previous best, was identified.

Reflecting on the results of the optimization workflow, an obvious strength is in the construction of the ideal training set of tripeptide catalysts. This set of catalysts covered a diverse array of structures that produced a selectivity range from −78% to 91% ee. Most importantly, this workflow identified a superior catalyst in just one round of optimization. Further, we were able to make reasonable models, namely, those with good cross-validation scores. When attempting to implement these models in our optimization workflow, we found challenges in making accurate predictions into sparsely populated regions of chemical space. That prompted us to incorporate prediction certainty metrics into our catalyst selection process, substantially increasing the accuracy of *a priori* predictions (as evidenced by the increased predictive performance from round 1 to round 2 and round 3 targets). Even then, only modest improvements were made over the original best hit.

This study clearly identified areas for improvement in our workflow. In fine-tuning the catalyst structure to improve selectivity beyond the original best catalyst model, generalizability was particularly challenging. This behavior is unsurprising given that the entire >30,000-member chemical space of tripeptides can be only sparsely sampled experimentally. Three possible explanations for achieving only marginal improvements over the best UTS catalyst include the following: (1) the dynamic nature of peptide catalysts results in indicator fields with many high-variance dimensions, causing challenges in dimensionality reduction which make the resulting models less generalizable (for a full explanation, see the Supporting Information), (2) the combinatorial nature of the training set resulted in some degree of simple “pattern matching”, despite the whole-molecule representation, rather than finding physically meaningful correlations, negatively impacting generalization, or (3) the algorithm did indeed converge on a local maximum given the structures present in the original *in silico* library.

Each of these explanations represents opportunities for future improvements of our workflow. For point one, unsupervised methods of compressing high-variance indicator fields could provide the same spatial information in a lower-dimensional form. For point two, altering the design of the original set such that it covers the same breadth of chemical space but breaks the combinatorial design would possibly result in more general models. For point three, a possible improvement to our workflow would be the implementation of generative models, effectively yielding a mutable *in silico* library.

An additional approach to constructing generalizable models would be to calculate the descriptors incorporating known reactive intermediates (e.g., a (di)enamine) rather than just the catalyst structure. This approach may enable the model to identify more direct physicochemical relationships, thereby improving predicted accuracy into sparsely populated regions of chemical space. However, using this type of representation would require recalculation of descriptors for each new reaction to optimize. The current descriptors and the UTS are agnostic to the reaction mechanism. As such, it is currently possible to apply the existing descriptors and UTS directly to any new reaction.

## Coda: Comparison of Expert-Knowledge- versus Data-Science-Guided Approaches

It is impossible to directly compare these two approaches because an expert-knowledge-guided identification of a peptide catalyst was not carried out for the annulation reaction; nevertheless, a number of considerations can be highlighted.

(1) In the very first round of descriptor validation, PLS models gave excellent performance metrics and predicted for both diastereo- and enantioselectivity the highest-performing catalysts (H-dPro-Pip-Glu-NH_2_ and H-dPro-MePro-Glu-NH_2_) known to be the best catalysts from prior traditional optimization. These catalysts feature a single well-defined conformation. This excellent match between the mechanism-guided and modeling-guided results is striking.
(2) In prior catalyst optimization campaigns that used mechanism-based considerations, distinctly different Pro-Pro-Xaa catalysts emerged.^19,23−27,30^ These reactions include aldol and conjugate additions to vinyl triflones,^30^ maleimides,^26,27^ or disubstituted nitroolefins.^23,24^ These “manually” optimized catalysts afford the addition products with high stereoselectivities (typically 90–99% ee) and feature a residue other than Glu in the Xaa position, varied absolute configurations at the α-carbons of the amino acids, alternative coordinating moieties to COOH, substituted Pro residues, or a combination of those features. These expert-knowledge-guided catalyst optimizations built on the common Pro-Pro-Xaa scaffold. A similar approach would have likely led to the identification of the high-performing catalyst H-dPro-(4*S*)Azp-Glu-NH_2_ for the annulation reaction with less experimental overhead than the *de novo* campaign with a large training set.
(3) *De novo* approaches ideally identify a new, “out of the box” class of catalysts that is difficult to imagine by a knowledge-guided approach. In prior studies, a combinatorial split-and-mix library approach provided, for example, H-Pro-dAla-dAsp-NH_2_ as an alternative to the Pro-Pro-Xaa motif for aldol reactions.^19^ Similarly, machine learning is a powerful tool to guide to new catalysts. In this study, the data-driven approach identified potent catalysts within a “privileged” class. At the same time, the study provided a versatile algorithm and a UTS, ready for identifying tripeptide catalysts for other reactions.

## Conclusions

In conclusion, using a machine-learning workflow, we generated predictive models for a nitroolefin conjugate addition reaction, validating our molecular representation for tripeptide catalysts that feature a rigid ground state. We next used this representation in an optimization campaign to identify a yet-unknown tripeptide catalyst for an annulation reaction that proceeds via a different mechanism. The respective products were obtained in >90% enantiomeric excess. The motifs identified are closely related to the “manually” optimized H-dPro-Pro-Glu-NH_2_ catalysts for reactions with β-nitroolefins. This outcome suggests that H-dPro-Pro-Glu-NH_2_-type catalysts represent either a global performance optimum of the training set or a local maximum with the global one undiscovered.

In this regard, it is encouraging that the algorithmic selection process was able to identify a high-performing catalyst motif in the first round of experimentation. Subsequent supervised modeling yielded marginal catalyst improvements but also identified multiple opportunities for improvement of our optimization workflow. Finally, this study also yielded a large *in silico* library of tripeptides, descriptor profiles for the constituent tripeptides, and a UTS of 161 tripeptide catalysts. The combination of these elements comprises a powerful starting point for future optimization campaigns.

## Supporting Information Available

Synthetic protocols and analytical data of peptides and catalysis products, and details on the computational methods (PDF)
