# Supplementary material for: Machine Learning to Develop Peptide Catalysts—Successes, Limitations, and Opportunities
Source: ACS Cent Sci. 2024 Feb 5;10(2):367–73. doi: 10.1021/acscentsci.3c01284 (PMC10906243; doi:10.1021/acscentsci.3c01284)
Supplement: Supplementary file 1 — oc3c01284_si_001.pdf [file oc3c01284_si_001.pdf]

Machine Learning to Develop Peptide Catalysts – Successes, Limitations and Opportunities

Tobias Schnitzer,‡,1 Martin Schnurr,‡,1 Andrew F. Zahrt,‡,2 Nader Sakhaee,2 Scott E. Denmark,*,2 Helma Wennemers*,1

1Laboratory of Organic Chemistry, ETH Zürich, Vladimir-Prelog-Weg 3, 8093 Zürich, Switzerland.

2Roger Adams Laboratory, Department of Chemistry, University of Illinois, Urbana, IL, USA, 61801

e-mail: Helma.Wennemers@org.chem.ethz.ch and sdenmark@illinois.edu

Supporting Information

Table of Contents:

1. General Remarks **2**

2. Experimental Screening and Evaluation of Conjugate Addition Reactions **2**

2.1 Synthesis and Characterization of Peptide Catalysts **2**

Analytical Data of Peptides for the Conjugate Addition Reaction (**P1** – **P50**) 4

Analytical Data of Universal Training Set Peptides (**UTS-1** – **UTS-161**) 10

Analytical Data of Predicted Peptides for the Annulation Reaction 47

Analytical Data of Additional Training Set Peptides 54

2.2 Catalysis Screenings **56**

Screening of Conjugate Addition Reactions 56

Screening of the Annulation Reaction 62

3. Computational Methods ***69***

4. NMR-Spectra of Peptides ***78***

5. References ***300***

1. General Remarks

Reagents and materials were of the highest commercially available grade and used without further purification. Reactions were monitored by thin layer chromatography using Merck silica gel 60 F254 aluminium sheets. Visualization of the compounds was achieved by UV-Vis or $KMnO_4$. Flash chromatography and plug filtrations were performed using silica gel 60 (particle size 0.040 – 0.063 mm, 200 – 400 mesh) manufactured by Fluka. ^{1}H and ^{13}C NMR spectra were recorded on a Bruker DRX 400, a Bruker AV III 400 (400 MHz/100 MHz) or a Bruker AV III 600 (600 MHz/150 MHz). All spectra were recorded at 25 °C. Chemical shifts (δ) are reported in parts per million (ppm) relative to the signal of tetramethylsilane (TMS) using the residual solvent signals. SFC analyses were performed on an analytical SFC with a diode array detector ACQUITY-UPLC-PDA from Waters using chiral stationary phase columns (Trefoil, AS, AD, IA, Whelk, IC, OD, OJ) (150 mm x 30 mm) from Daicel or Waters under the reported conditions. HPLC analyses were performed on an analytical Ultimate 3000 HPLC system from Dionex with a diode array detector and chiral stationary phase columns (Daicel AD-H, Daicel AS-H, AY-H, OD-H or Daicel OJ-H). High-resolution electron ionization (HR-EI) mass spectra were measured on a Waters Micromass AutoSpec Ultima spectrometer. High-resolution MALDI spectra were acquired on a Bruker solariX 94 (ESI/MALDI-FT-ICR) and a Bruker Ultra-Flex II (MALDI-TOF) spectrometer.

2. Experimental Screening and Evaluation of Conjugate Addition Reactions

2.1 Synthesis and Characterization of Peptide Catalysts

Peptides were prepared on solid phase using Rink Amide resin. The general protocol for Fmoc/*t*Bu peptide synthesis was followed according to the general procedures below.

General Procedure A

General procedure for peptide couplings: *i*Pr_2NEt (6 equiv.) was added to a solution of Fmoc-Xaa-OH (3 equiv.) and HATU (3 equiv.) in DMF. The solution of the activated amino acid (≈ 100 mM) was added to the amino-functionalized resin, preswollen in CH_2Cl_2 and the mixture was agitated for 1 h before washing with DMF (3 ×) and CH_2Cl_2 (3 ×).

General procedure for Fmoc-deprotections: A solution of 20% piperidine in DMF was added to the resin (preswollen in CH_2Cl_2) and the reaction mixture was agitated for 10 min, drained and the piperidine treatment was repeated for 10 min. Finally, the resin was washed with DMF (3 ×) and CH_2Cl_2 (3 ×).

General procedure for side chain deprotection and cleavage of the peptides from the solid support: The peptides were deprotected and cleaved from the resin by agitating in a mixture of TFA/TIS/H_2O (95:2.5:2.5) for 1 h and a second time for 30 min. Pooling of the filtrates and removal of all volatiles at reduced pressure followed by precipitation and thorough washing with Et_2O afforded the peptides as their TFA salts. The peptides were redissolved in MeCN/H_2O 1:1, dried by lyophilisation and used without further purification.

General Procedure B

Peptide couplings: In a semi-automated parallel synthesizer, iPr_2NEt (6 equiv.) was added to a solution of Fmoc-Xaa-OH (3 equiv.) and HATU (3 equiv.) in DMF. The solution of the activated amino acid (≈

100 mM) was added to the amino-functionalized resin (250 mg scale, pre-washed with DMF) and the mixture was agitated for 30 min before washing with DMF (5x). This step was repeated once before washing with DMF (5x) and DCM (3x) and Et_2O (2x).

Fmoc-deprotections: In a semi-automated parallel synthesizer, a solution of 20% piperidine in DMF was added to the resin (pre-washed with DMF) and the reaction mixture was agitated for 10 min, drained and the piperidine treatment was repeated for 10 min. Finally, the resin was washed with DMF (5x).

Sidechain deprotection and cleavage of the peptides from the solid support: The peptides were sidechain deprotected and cleaved from the resin by shaking in a mixture of TFA/TIS/H_2O (95:2.5:2.5) for 1 h and a second time for 30 min. Pooling of the filtrates and removal of all volatiles by a stream of pressurized air followed by precipitation with –18 °C cold Et_2O (3x) afforded the peptides as their TFA-salts. The peptides were re-dissolved in MeCN/H_2O 1:1, dried by lyophilization and used without further purification.

General Procedure C (Peptides containing Aminobenzoic Acid (Abz))

Peptide couplings with aminobenzoic acid: Diisopropylcarbodiimid (DIC; 5.5 equiv.) was added to a solution of 1-hydroxybenzotriazol (HOBt; 5.5 equiv.) and aminobenzoic acid (Fmoc-Abz-OH) in DMF. The mixture was shaken for 3 h at r.t. and washed with DMF (3x) and CH_2Cl_2 (3x).

Peptide couplings after Abz: iPr_2NEt (10 equiv.) was added to a solution of Fmoc-Xaa-OH (5 equiv.) and HATU (5 equiv.) in DMF. The solution of the activated amino acid (≈ 100 mM) was added to the (amino-functionalized) resin (pre-swollen in CH_2Cl_2) and the mixture was agitated for 1 h before washing with DMF (3x) and CH_2Cl_2 (3x).

→ All other steps as described in general procedure A.

General Procedure D (Peptides containing (2*S*,4*S*)-Hydroxyproline)

Peptide couplings with (2S,4S)-hydroxyproline: iPr_2NEt (10 equiv.) was added to a ice-cold solution of Fmoc-(2*S*,4*S*)-Hyp-OH (5 equiv.) and HATU (5 equiv.) in DMF. The solution of the activated amino acid (≈ 100 mM) was added to the amino-functionalized resin pre-swollen in CH_2Cl_2 and the mixture was agitated for 1 h. This step was repeated once before washing with DMF (3x) and CH_2Cl_2 (3x).

Peptide couplings after (2S,4S)-Hydroxyproline: iPr_2NEt (10 equiv.) was added to a solution of Fmoc-Xaa-OH (5 equiv.) and HATU (5 equiv.) in DMF. The solution of the activated amino acid (≈ 100 mM) was added to the (amino-functionalized) resin (pre-swollen in CH_2Cl_2) and the mixture was agitated for 1 h before washing with DMF (3x) and CH_2Cl_2 (3x).

→ All other steps as described in general procedure A.

Analytical Data of Peptides for the Conjugate Addition Reaction (P1 – P50)

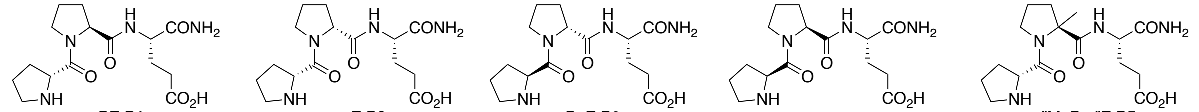

pPE **P1** | ppE **P2** | PpE **P3** | PPE **P4** | p”MePro”E **P5**

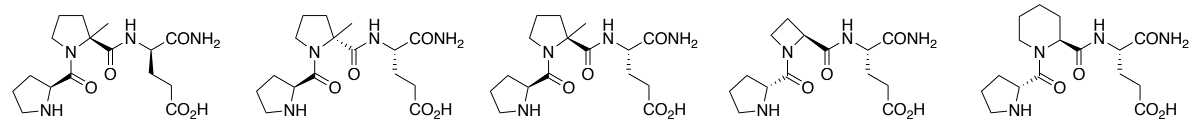

P”MePro”e **P6** | P”D-MePro”E **P7** | P”MePro”E **P8** | p4E **P9** | p6E **P10**

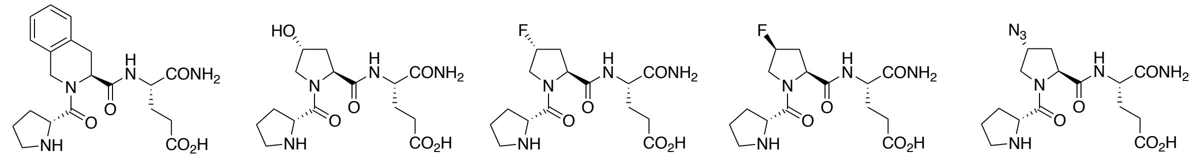

p”Tic”E **P11** | p”4R-Hyp”E **P12** | p”4R-Flp”E **P13** | p”4S-Flp”E **P14** | p”4R-Azp”E **P15**

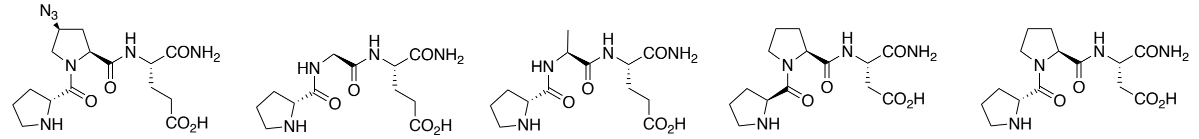

p”4S-Azp”E **P16** | pGE **P17** | pAE **P18** | PPD **P19** | pPD **P20**

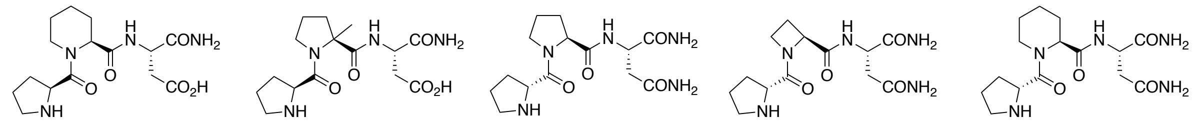

P6D **P21** | P”MePro”D **P22** | pPN **P23** | p4N **P24** | p6N **P25**

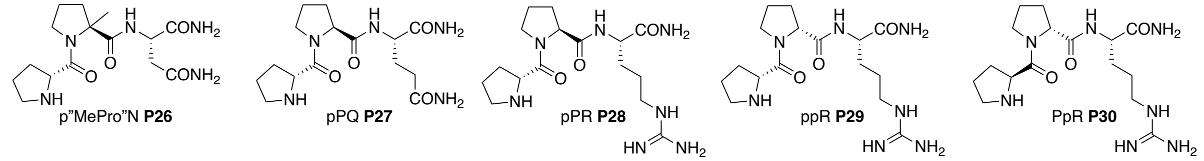

p”MePro”N **P26** | pPQ **P27** | pPR **P28** | ppR **P29** | PpR **P30**

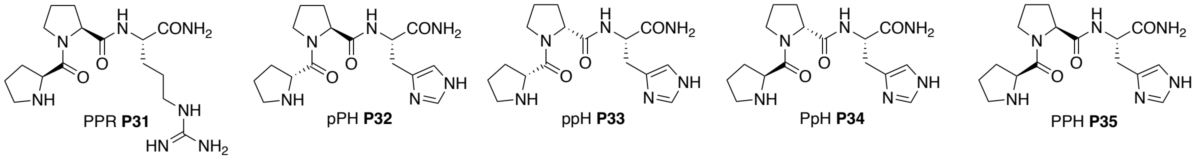

PPR **P31** | pPH **P32** | ppH **P33** | PpH **P34** | PPH **P35**

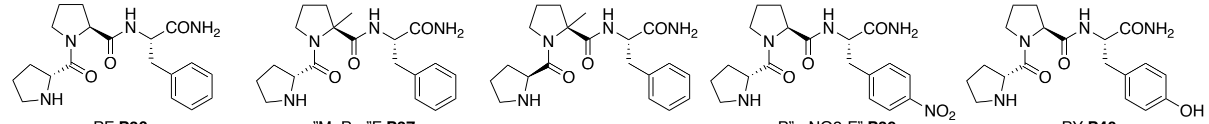

pPF **P36** | p”MePro”F **P37** | P”MePro”F **P38** | pP”p-NO2-F” **P39** | pPY **P40**

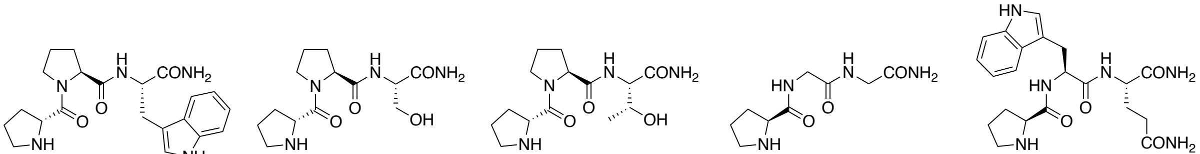

pPW **P41** | pPS **P42** | pPT **P43** | PGG **P44** | PWQ **P45**

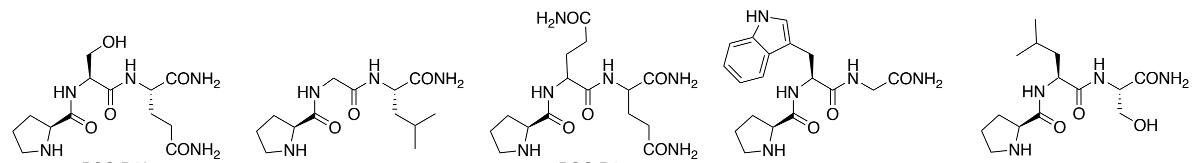

PSQ **P46** | PGL **P47** | PQQ **P48** | PWG **P49** | PLS **P50**

Peptide **P19**: analytical data as reported before.[1]
Peptide **P20**: analytical data as reported before.[2]
Peptides **P1** and **P23**: analytical data as reported before.[3]
Peptide **P5**: analytical data as reported before.[4]
Peptides **P12-P16**: analytical data as reported before.[5]
Peptides **P9**, **P10**, **P18**, and **P25**: analytical data as reported before.[6]
Peptides **P2-P4**: analytical data as reported before.[7]
Peptides **P28**, **P32**, **P36**, and **P39-P43**: analytical data as reported before.[8]
Peptide **P27**: analytical data as reported before.[9]

Analytical Data P6: The peptide was synthesized according to the general procedure A for solid phase peptide synthesis. The signals of the major conformer are reported (note, two *trans/cis* amide conformers are present in a 2:1 ratio): **^{1}H NMR** (600 MHz, H_2O+D_2O) δ = 7.90 (d, J = 7.9 Hz, 1H), 7.53 (s, 1H), 7.05 (s, 1H), 4.37 – 4.31 (m, 1H), 3.72 (ddd, J = 10.3, 6.9, 3.6 Hz, 1H), 3.59 – 3.53 (m, 1H), 3.35 – 3.25 (m, 2H), 2.53 – 2.29 (m, 3H), 2.19 – 1.83 (m, 10H), 1.50 (s, 3H). **^{13}C NMR** (151 MHz, H_2O+D_2O) δ = 177.5, 176.0, 170.6, 167.1, 68.1, 59.5, 53.2, 48.3, 46.7, 38.3, 30.4, 27.9, 25.7, 23.9, 23.5, 19.4. **HRMS** (MALDI): *m/z* calcd. for $[M + H]^+$ $C_{16}H_{27}N_4O_5^+$: 355.1976; found: 355.1995.

Analytical Data P7: The peptide was synthesized according to the general procedure A for solid phase peptide synthesis. The signals of the major conformer are reported. **^{1}H NMR** (600 MHz, H_2O+D_2O) δ = 8.06 (d, J = 7.2 Hz, 1H), 7.50 (s, 1H), 7.03 (s, 1H), 4.29 – 4.21 (m, 1H), 3.69 (dd, J = 10.3, 6.8 Hz, 1H), 3.68 – 3.58 (m, 1H), 3.41 – 3.18 (m, 2H), 2.48 (ddt, J = 13.5, 8.9, 6.8 Hz, 1H), 2.42 – 2.25 (m, 2H), 2.16 – 1.74 (m, 10H), 1.48 (s, 3H). **^{13}C NMR** (151 MHz, H_2O+D_2O) δ = 178.3, 176.2, 176.1, 167.6, 67.9, 59.5, 53.7, 48.3, 46.8, 38.7, 30.9, 28.2, 25.6, 23.9, 23.4, 19.8. **HRMS** (MALDI): *m/z* calcd. for $[M + H]^+$ $C_{16}H_{27}N_4O_5^+$: 355.1976; found: 355.1976.

Analytical Data P8: The peptide was synthesized according to the general procedure A for solid phase peptide synthesis. The signals of the major conformer are reported: **^{1}H NMR** (600 MHz, H_2O+D_2O) δ = 7.85 (d, J = 7.2 Hz, 1H), 7.47 (s, 1H), 7.02 (s, 1H), 4.28 (ddd, J = 9.7, 7.2, 5.1 Hz, 1H), 3.72 (ddd, J = 10.5, 7.3, 3.6 Hz, 1H), 3.64 – 3.57 (m, 1H), 3.31 (dd, J = 16.6, 9.3 Hz, 2H), 2.51 – 2.37 (m, 3H), 2.13 – 1.88 (m, 10H), 1.49 (s, 3H). **^{13}C NMR** (151 MHz, H_2O+D_2O) δ = 177.8, 176.1, 175.9, 167.4, 68.1, 59.6, 53.4, 48.2, 46.7, 38.5, 30.4, 27.9, 25.9, 23.9, 23.4, 19.6. **HRMS** (MALDI): *m/z* calcd. for $[M + H]^+$ $C_{16}H_{27}N_4O_5^+$: 355.1976; found: 355.1973.

Analytical Data P11: The peptide was synthesized according to the general procedure A for solid phase peptide synthesis. The signals of the major conformer are reported: **^{1}H NMR** (400 MHz, D_2O) δ = 7.33 – 7.16 (m, 4H), 4.96 (dd, J = 8.7, 6.3 Hz, 1H), 4.82 (dd, J = 5.8, 3.8 Hz, 1H), 4.76 (s, 1H), 4.59 (s, 1H), 4.18 – 4.07 (m, 1H), 3.53 – 3.30 (m, 2H), 3.30 – 3.11 (m, 2H), 2.67 – 2.52 (m, 1H), 2.14 – 1.95 (m, 3H), 1.95 – 1.79 (m, 2H), 1.79 – 1.55 (m, 2H). **^{13}C NMR** (101 MHz, D_2O) δ = 177.1, 175.6, 173.3, 170.2, 133.6, 132.3, 128.6, 127.6, 127.5, 126.3, 59.3, 56.8, 52.2, 46.5, 46.1, 31.2, 29.4, 28.1, 25.6, 23.9. **HRMS** (MALDI): *m/z* calcd. for $[M + H]^+$ $C_{20}H_{27}N_4O_5^+$: 403.1976; found: 403.1972.

Analytical Data P17: The peptide was synthesized according to the general procedure A for solid phase peptide synthesis. The signals of the major conformer are reported: **^{1}H NMR** (400 MHz, D_2O) δ = 4.46 (s, 1H), 4.37 (s, 1H), 4.04 (s, 2H), 3.43 (s, 1H), 2.97 (s, 3H), 2.50 (s, 2H), 2.09 (s, 4H). **^{13}C NMR** (101 MHz,

D_2O) δ = 177.0, 175.9, 171.0, 170.3, 59.7, 52.7, 46.4, 45.9, 42.4, 29.9, 26.1, 23.7. **HRMS** (MALDI): *m/z* calcd. for $[M + H]^+$ $C_{14}H_{20}F_3N_4O_7^+$: 413.1290; found: 413.1289.

Analytical Data P21: The peptide was synthesized according to the general procedure A for solid phase peptide synthesis. The signals of the major conformer are reported: **^{1}H NMR** (400 MHz, D_2O) δ = 5.08 (dd, J = 6.1, 3.0 Hz, 1H), 4.72 – 4.66 (m, 1H), 3.76 (d, J = 12.9 Hz, 1H), 3.56 – 3.39 (m, 2H), 3.38 – 3.25 (m, 1H), 3.08 – 2.76 (m, 2H), 2.65 – 2.47 (m, 1H), 2.24 – 1.94 (m, 4H), 1.91 – 1.81 (m, 1H), 1.81 – 1.69 (m, 3H), 1.63 – 1.38 (m, 2H). **^{13}C NMR** (101 MHz, D_2O) δ = 175.2, 174.6, 173.0, 169.8, 59.1, 54.0, 50.4, 46.9, 44.2, 35.9, 28.7, 26.4, 24.1, 24.1, 19.6. **HRMS** (MALDI): *m/z* calcd. for $[M + H]^+$ $C_{15}H_{25}N_4O_5^+$: 341.1819; found: 341.1820.

Analytical Data P22: The peptide was synthesized according to the general procedure A for solid phase peptide synthesis. The signals of the major conformer are reported: **^{1}H NMR** (400 MHz, D_2O) δ = 4.73 (dd, J = 9.0, 4.9 Hz, 1H), 4.67 (dd, J = 9.0, 6.9 Hz, 1H), 3.83 (dt, J = 10.5, 6.6 Hz, 1H), 3.72 (dt, J = 10.4, 6.8 Hz, 1H), 3.44 (qt, J = 11.5, 7.1 Hz, 2H), 3.03 – 2.92 (m, 1H), 2.78 (dd, J = 16.5, 9.0 Hz, 1H), 2.59 (ddt, J = 13.1, 9.1, 6.6 Hz, 1H), 2.27 – 1.93 (m, 7H), 1.56 (s, 3H). **^{13}C NMR** (101 MHz, D_2O) δ = 175.7, 175.4, 175.3, 168.2, 68.0, 59.6, 50.5, 48.3, 46.7, 38.8, 35.9, 28.1, 23.9, 23.3, 20.0. **HRMS** (MALDI): *m/z* calcd. for $[M + H]^+$ $C_{15}H_{25}N_4O_5^+$: 341.1819; found: 341.1819.

Analytical Data P24: The peptide was synthesized according to the general procedure A for solid phase peptide synthesis. The signals of the major conformer are reported: **^{1}H NMR** (400 MHz, D_2O) δ = 4.95 (dd, J = 9.4, 5.8 Hz, 1H), 4.77 – 4.68 (m, 1H), 4.45 (dd, J = 8.8, 6.9 Hz, 1H), 4.39 – 4.26 (m, 2H), 3.43 (qt, J = 11.7, 7.1 Hz, 2H), 3.03 – 2.64 (m, 3H), 2.57 – 2.44 (m, 1H), 2.37 (ddt, J = 11.9, 9.2, 6.1 Hz, 1H), 2.20 – 1.99 (m, 3H). **^{13}C NMR** (101 MHz, D_2O) δ = 174.7, 174.4, 171.8, 168.8, 61.2, 57.3, 50.2, 49.1, 46.5, 36.3, 28.0, 24.0, 20.5. **HRMS** (MALDI): *m/z* calcd. for $[M + H]^+$ $C_{13}H_{22}N_5O_4^+$: 312.1666; found: 312.1664.

Analytical Data P26: The peptide was synthesized according to the general procedure A for solid phase peptide synthesis. The signals of the major conformer are reported: **^{1}H NMR** (400 MHz, D_2O) δ = 4.77 – 4.72 (m, 1H), 4.67 (dd, J = 9.0, 6.8 Hz, 1H), 3.88 – 3.78 (m, 1H), 3.72 (dt, J = 13.1, 6.7 Hz, 1H), 3.44 (tdd, J = 11.6, 7.9, 4.6 Hz, 2H), 2.91 (dd, J = 15.4, 4.9 Hz, 1H), 2.74 (dd, J = 15.4, 9.3 Hz, 1H), 2.59 (ddt, J = 13.0, 9.0, 7.0 Hz, 1H), 2.24 – 1.94 (m, 7H), 1.56 (s, 3H). **^{13}C NMR** (101 MHz, D_2O) δ = 176.0, 175.5, 175.1, 168.5, 68.2, 59.8, 50.8, 48.6, 47.0, 39.1, 36.1, 28.4, 24.1, 23.6, 20.2. **HRMS** (MALDI): *m/z* calcd. for $[M + H]^+$ $C_{15}H_{25}N_5O_4^+$: 340.1979; found: 340.1980.

Analytical Data P29: The peptide was synthesized according to the general procedure A for solid phase peptide synthesis. The signals of the major conformer are reported: **^{1}H NMR** (400 MHz, D_2O) δ = 4.71 – 4.61 (m, 1H), 4.57 – 4.49 (m, 1H), 4.34 (dd, J = 9.3, 4.7 Hz, 1H), 3.76 (d, J = 1.5 Hz, 1H), 3.68 – 3.56 (m, 1H), 3.44 (q, J = 6.8 Hz, 2H), 3.24 (t, J = 6.7 Hz, 2H), 2.68 – 2.53 (m, 1H), 2.39 (ddt, J = 12.5, 8.2, 6.1 Hz, 1H), 2.20 – 1.88 (m, 7H), 1.84 – 1.60 (m, 3H). **^{13}C NMR** (101 MHz, D_2O) δ = 176.3, 174.1, 168.1, 156.8, 60.7, 59.1, 53.1, 47.7, 46.6, 40.4, 29.4, 28.3, 28.0, 24.7, 24.4, 23.8. **HRMS** (MALDI): *m/z* calcd. for $[M + H]^+$ $C_{16}H_{30}N_7O_3^+$: 368.2405; found: 368.2403.

Analytical Data P30: The peptide was synthesized according to the general procedure A for solid phase peptide synthesis. The signals of the major conformer are reported: **^{1}H NMR** (400 MHz, D_2O) δ = 4.65 (dd, J = 8.9, 6.8 Hz, 1H), 4.52 (td, J = 8.2, 4.3 Hz, 1H), 4.32 (dd, J = 9.2, 4.9 Hz, 1H), 3.75 (dt, J = 10.6, 6.4 Hz,

1H), 3.65 (dt, *J* = 10.0, 6.8 Hz, 1H), 3.55 – 3.35 (m, 2H), 3.24 (dd, *J* = 8.0, 5.7 Hz, 2H), 2.69 – 2.51 (m, 1H), 2.36 (dq, *J* = 12.6, 7.9 Hz, 1H), 2.24 – 1.86 (m, 7H), 1.85 – 1.53 (m, 3H). **^{13}C NMR** (101 MHz, D_2O) δ = 176.3, 174.4, 168.0, 156.8, 60.9, 59.2, 53.2, 47.7, 46.6, 40.4, 29.6, 28.1, 27.9, 24.4, 24.4, 23.9. **HRMS** (MALDI): *m/z* calcd. for $[M + H]^+$ $C_{16}H_{30}N_7O_3^+$: 368.2405; found: 368.2405.

Analytical Data P31: The peptide was synthesized according to the general procedure A for solid phase peptide synthesis. The signals of the major conformer are reported: **^{1}H NMR** (400 MHz, D_2O) δ = 4.67 (dd, *J* = 8.8, 5.5 Hz, 1H), 4.53 (dd, *J* = 8.3, 6.2 Hz, 1H), 4.28 (dd, *J* = 8.4, 5.8 Hz, 1H), 3.80 – 3.70 (m, 1H), 3.61 (dt, *J* = 10.1, 7.1 Hz, 1H), 3.51 – 3.36 (m, 2H), 3.24 (t, *J* = 6.9 Hz, 2H), 2.67 – 2.52 (m, 1H), 2.38 (ddt, *J* = 12.7, 8.4, 6.3 Hz, 1H), 2.20 – 1.62 (m, 10H). **^{13}C NMR** (101 MHz, D_2O) δ = 176.2, 173.8, 168.1, 156.8, 60.4, 59.1, 53.3, 47.8, 46.6, 40.5, 29.4, 28.4, 28.1, 24.6, 24.4, 23.8. **HRMS** (MALDI): *m/z* calcd. for $[M + H]^+$ $C_{16}H_{30}N_7O_3^+$: 368.2405; found: 368.2407.

Analytical Data P33: The peptide was synthesized according to the general procedure A for solid phase peptide synthesis. The signals of the major conformer are reported: **^{1}H NMR** (400 MHz, D_2O) δ = 8.66 (d, *J* = 1.4 Hz, 1H), 7.34 (d, *J* = 1.3 Hz, 1H), 4.77 – 4.74 (m, 1H), 4.63 (dd, *J* = 8.9, 6.3 Hz, 1H), 4.44 (dd, *J* = 8.1, 6.8 Hz, 1H), 3.71 (dt, *J* = 10.1, 6.2 Hz, 1H), 3.54 (dt, *J* = 10.1, 7.4 Hz, 1H), 3.48 – 3.31 (m, 3H), 3.14 (dd, *J* = 15.5, 9.8 Hz, 1H), 2.65 – 2.50 (m, 1H), 2.26 (ddt, *J* = 12.6, 8.1, 6.3 Hz, 1H), 2.17 – 1.91 (m, 5H), 1.63 (dq, *J* = 12.7, 7.3 Hz, 1H). **^{13}C NMR** (101 MHz, D_2O) δ = 174.1, 173.7, 168.0, 133.6, 128.8, 117.2, 60.6, 59.0, 51.8, 47.6, 46.6, 29.4, 28.3, 26.4, 24.6, 23.8. **HRMS** (MALDI): *m/z* calcd. for $[M + H]^+$ $C_{16}H_{25}N_6O_3^+$: 349.1983; found: 349.1986.

Analytical Data P34: The peptide was synthesized according to the general procedure A for solid phase peptide synthesis. The signals of the major conformer are reported: **^{1}H NMR** (400 MHz, D_2O) δ = 8.66 (d, *J* = 1.4 Hz, 1H), 7.35 (d, *J* = 1.4 Hz, 1H), 4.77 – 4.73 (m, 1H), 4.62 (dd, *J* = 8.9, 6.8 Hz, 1H), 4.43 (dd, *J* = 8.6, 4.8 Hz, 1H), 3.73 – 3.55 (m, 2H), 3.51 – 3.33 (m, 3H), 3.16 (dd, *J* = 15.5, 9.7 Hz, 1H), 2.63 – 2.49 (m, 1H), 2.25 (dtd, *J* = 12.9, 8.5, 6.8 Hz, 1H), 2.16 – 1.87 (m, 5H), 1.73 (ddd, *J* = 13.0, 6.5, 4.9 Hz, 1H). **^{13}C NMR** (101 MHz, D_2O) δ = 174.1, 173.9, 168.0, 133.5, 128.8, 117.2, 60.7, 59.2, 52.0, 47.6, 46.6, 29.5, 28.1, 26.2, 24.2, 23.9. **HRMS** (MALDI): *m/z* calcd. for $[M + H]^+$ $C_{16}H_{25}N_6O_3^+$: 349.1983; found: 349.1984.

Analytical Data P35: The peptide was synthesized according to the general procedure A for solid phase peptide synthesis. The signals of the major conformer are reported: **^{1}H NMR** (400 MHz, D_2O) δ = 8.61 (d, *J* = 1.4 Hz, 1H), 7.36 (d, *J* = 1.3 Hz, 1H), 4.70 – 4.60 (m, 2H), 4.47 (dd, *J* = 8.4, 6.3 Hz, 1H), 3.72 (ddd, *J* = 10.0, 7.1, 5.5 Hz, 1H), 3.60 (dt, *J* = 10.1, 7.1 Hz, 1H), 3.48 – 3.38 (m, 2H), 3.26 (qd, *J* = 15.5, 7.1 Hz, 2H), 2.63 – 2.53 (m, 1H), 2.33 (ddt, *J* = 12.7, 8.2, 6.4 Hz, 1H), 2.18 – 1.94 (m, 5H), 1.88 (dq, *J* = 13.7, 7.0 Hz, 1H). **^{13}C NMR** (101 MHz, D_2O) δ = 174.0, 173.6, 168.1, 133.6, 128.5, 117.3, 60.5, 59.1, 52.4, 47.7, 46.6, 29.4, 28.4, 26.3, 24.6, 23.8. **HRMS** (MALDI): *m/z* calcd. for $[M + H]^+$ $C_{16}H_{25}N_6O_3^+$: 349.1983; found: 349.1984.

Analytical Data P37: The peptide was synthesized according to the general procedure A for solid phase peptide synthesis. The signals of the major conformer are reported: **^{1}H NMR** (600 MHz, H_2O+D_2O) δ = 7.97 (d, *J* = 7.8 Hz, 1H), 7.29 (dd, *J* = 8.1, 6.9 Hz, 2H), 7.26 (s, 1H), 7.24 – 7.18 (m, 3H), 7.06 (s, 1H), 4.56 – 4.49 (m, 1H), 3.59 (ddd, *J* = 10.3, 7.3, 5.8 Hz, 1H), 3.52 (dt, *J* = 10.3, 7.2 Hz, 1H), 3.38 – 3.23 (m, 3H), 2.85 (dd, *J* = 14.3, 10.5 Hz, 1H), 2.49 – 2.40 (m, 1H), 1.98 (ddt, *J* = 15.4, 13.6, 7.0 Hz, 2H), 1.84 (tdd, *J* = 13.0, 11.5, 6.9 Hz, 2H), 1.70 (dt, *J* = 13.1, 7.3 Hz, 1H), 1.57 (ddt, *J* = 14.7, 12.7, 7.3 Hz, 1H), 1.45 – 1.35

(m1H), 1.34 (s, 3H). . **^{13}C NMR** (151 MHz, H_2O+D_2O) δ = 176.2, 175.7, 168.4, 137.0, 129.0, 128.7, 127.1, 67.8, 59.7, 54.4, 48.2, 46.9, 38.6, 36.2, 28.1, 23.9, 23.1, 20.1. **HRMS** (MALDI): *m/z* calcd. for $[M + H]^+$ $C_{20}H_{29}N_4O_3^+$: 373.2234; found: 373.2235.

Analytical Data P38: The peptide was synthesized according to the general procedure A for solid phase peptide synthesis. The signals of the major conformer are reported: **^{1}H NMR** (600 MHz, H_2O+D_2O) δ= 7.39 (d, *J* = 7.5 Hz, 1H), 7.36 (s, 1H), 7.33 – 7.27 (m, 2H), 7.27 – 7.18 (m, 3H), 7.01 (s, 1H), 4.43 (dd, *J* = 9.3, 7.4 Hz, 1H), 3.66 (ddd, *J* = 10.2, 8.0, 4.5 Hz, 1H), 3.53 (ddd, *J* = 10.3, 8.6, 7.2 Hz, 1H), 3.34 – 3.19 (m, 2H), 3.18 – 3.08 (m, 1H), 3.01 (dd, *J* = 14.1, 8.1 Hz, 1H), 2.40 (dddd, *J* = 13.8, 8.9, 7.9, 6.3 Hz, 1H), 2.04 – 1.73 (m, 8H), 1.38 (s, 3H). **^{13}C NMR** (151 MHz, H_2O+D_2O) δ= 175.7, 175.3, 167.6, 136.4, 129.3, 128.8, 127.2, 68.1, 59.5, 54.3, 48.2, 46.7, 38.4, 36.5, 28.0, 23.9, 23.3, 19.6. **HRMS** (MALDI): *m/z* calcd. for $[M + H]^+$ $C_{20}H_{29}N_4O_3^+$: 373.2234; found: 373.2224.

Analytical Data P44: The peptide was synthesized according to the general procedure A for solid phase peptide synthesis. The signals of the major conformer are reported: **^{1}H NMR** (400 MHz, D_2O) δ= 4.48 (dd, *J* = 8.6, 6.6 Hz, 1H), 4.15 – 4.01 (m, 2H), 3.96 (s, 2H), 3.44 (qt, *J* = 11.5, 7.1 Hz, 2H), 2.56 – 2.44 (m, 1H), 2.21 – 2.05 (m, 3H). **^{13}C NMR** (101 MHz, D_2O) δ= 174.1, 171.5, 170.4, 59.8, 46.4, 42.5, 42.0, 29.5, 23.8. **HRMS** (MALDI): *m/z* calcd. for $[M + H]^+$ $C_9H_{17}N_4O_3^+$: 229.1295; found: 229.1299.

Analytical Data P45: The peptide was synthesized according to the general procedure A for solid phase peptide synthesis. The signals of the major conformer are reported: **^{1}H NMR** (400 MHz, D_2O) δ= 7.66 (dt, *J* = 8.0, 1.0 Hz, 1H), 7.52 (dt, *J* = 8.2, 1.0 Hz, 1H), 7.32 – 7.22 (m, 2H), 7.18 (ddd, *J* = 8.0, 7.0, 1.1 Hz, 1H), 4.68 (dd, *J* = 8.5, 6.9 Hz, 1H), 4.39 (dd, *J* = 8.5, 5.8 Hz, 1H), 4.16 (dd, *J* = 9.1, 5.1 Hz, 1H), 3.49 – 3.22 (m, 4H), 2.53 – 2.39 (m, 1H), 2.24 – 2.13 (m, 2H), 2.12 – 1.92 (m, 4H), 1.78 (dtd, *J* = 14.6, 8.8, 6.1 Hz, 1H). **^{13}C NMR** (101 MHz, D_2O) δ= 177.9, 174.9, 173.1, 169.4, 136.1, 126.6, 124.6, 122.1, 119.5, 118.3, 112.0, 108.4, 59.5, 55.3, 52.6, 46.5, 30.8, 29.7, 26.8, 26.7, 23.7. **HRMS** (MALDI): *m/z* calcd. for $[M + H]^+$ $C_{21}H_{29}N_6O_4^+$: 429.2245; found: 429.2240.

Analytical Data P46: The peptide was synthesized according to the general procedure A for solid phase peptide synthesis. The signals of the major conformer are reported: **^{1}H NMR** (400 MHz, D_2O) δ= 4.53 (t, *J* = 5.8 Hz, 1H), 4.47 (dd, *J* = 8.7, 6.3 Hz, 1H), 4.37 (dd, *J* = 9.1, 5.2 Hz, 1H), 3.90 (d, *J* = 5.8 Hz, 2H), 3.44 (qt, *J* = 11.4, 7.0 Hz, 2H), 2.51 (tq, *J* = 8.7, 5.9 Hz, 1H), 2.41 (t, *J* = 7.5 Hz, 2H), 2.24 – 1.94 (m, 5H). **^{13}C NMR** (101 MHz, D_2O) δ= 177.9, 175.6, 171.4, 169.9, 60.9, 59.6, 55.7, 52.9, 46.5, 31.1, 29.7, 26.8, 23.7. **HRMS** (MALDI): *m/z* calcd. for $[M + H]^+$ $C_{13}H_{24}N_5O_5^+$: 330.1772; found: 330.1770.

Analytical Data P47: The peptide was synthesized according to the general procedure A for solid phase peptide synthesis. The signals ofl the major conformer are reported: **^{1}H NMR** (400 MHz, D_2O) δ= 4.50 – 4.43 (m, 1H), 4.33 (dd, *J* = 9.9, 4.6 Hz, 1H), 4.05 (d, *J* = 1.8 Hz, 2H), 3.44 (qt, *J* = 11.5, 7.1 Hz, 2H), 2.55 – 2.43 (m, 1H), 2.20 – 2.03 (m, 3H), 1.76 – 1.56 (m, 3H), 0.95 (d, *J* = 6.1 Hz, 3H), 0.91 (d, *J* = 6.1 Hz, 3H). **^{13}C NMR** (101 MHz, D_2O) δ= 177.6, 170.9, 170.2, 59.7, 52.3, 46.5, 42.3, 39.9, 29.6, 24.3, 23.7, 22.2, 20.5. **HRMS** (MALDI): *m/z* calcd. for $[M + H]^+$ $C_{13}H_{25}N_4O_3^+$: 285.1921; found: 285.1923.

Analytical Data P48: The peptide was synthesized according to the general procedure A for solid phase peptide synthesis. The signals of the major conformer are reported: **^{1}H NMR** (400 MHz, D_2O) δ= 4.47 – 4.30 (m, 3H), 3.43 (dddd, *J* = 13.7, 11.3, 5.9, 4.3 Hz, 2H), 2.49 (dtd, *J* = 8.5, 5.9, 2.0 Hz, 1H), 2.40 (td, *J* =

7.5, 1.5 Hz, 4H), 2.20 – 1.96 (m, 7H). **^{13}C NMR** (101 MHz, D_2O) δ = 177.8, 177.7, 175.5, 172.9, 169.6, 59.5, 53.4, 52.9, 46.5, 31.0, 30.9, 29.7, 26.9, 26.7, 23.7. **HRMS** (MALDI): *m/z* calcd. for $[M + H]^+$ $C_{15}H_{27}N_6O_5^+$: 371.2037; found: 371.2040.

Analytical Data P49: The peptide was synthesized according to the general procedure A for solid phase peptide synthesis. The signals of the major conformer are reported: **^{1}H NMR** (400 MHz, D_2O) δ = 7.67 (dt, J = 7.9, 1.0 Hz, 1H), 7.52 (dt, J = 8.2, 0.9 Hz, 1H), 7.32 – 7.24 (m, 2H), 7.19 (ddd, J = 8.0, 7.1, 1.1 Hz, 1H), 4.68 (t, J = 7.7 Hz, 1H), 4.38 – 4.31 (m, 1H), 3.82 – 3.60 (m, 2H), 3.46 – 3.34 (m, 2H), 3.31 (d, J = 7.8 Hz, 2H), 2.50 – 2.34 (m, 1H), 2.13 – 1.93 (m, 3H). **^{13}C NMR** (101 MHz, D_2O) δ = 173.8, 173.7, 169.5, 136.1, 126.6, 124.5, 122.0, 119.4, 118.3, 111.9, 108.6, 59.5, 55.3, 46.5, 42.0, 29.7, 26.8, 23.6. **HRMS** (MALDI): *m/z* calcd. for $[M + H]^+$ $C_{18}H_{24}N_5O_3^+$: 358.1874; found: 358.1872.

Analytical Data P50: The peptide was synthesized according to the general procedure A for solid phase peptide synthesis. The signals of the major conformer are reported: **^{1}H NMR** (400 MHz, D_2O) δ = 4.47 – 4.39 (m, 3H), 3.91 – 3.83 (m, 2H), 3.43 (qt, J = 11.6, 7.1 Hz, 2H), 2.55 – 2.43 (m, 1H), 2.17 – 1.99 (m, 3H), 1.74 – 1.62 (m, 3H), 0.99 – 0.94 (m, 3H), 0.92 (d, J = 6.0 Hz, 3H). **^{13}C NMR** (101 MHz, D_2O) δ = 174.4, 173.9, 169.7, 61.1, 59.5, 55.1, 52.9, 46.5, 39.5, 29.8, 24.3, 23.7, 22.0, 20.7. **HRMS** (MALDI): *m/z* calcd. for $[M + H]^+$ $C_{14}H_{27}N_4O_4^+$: 315.2027; found: 315.2028.

Analytical Data of Universal Training Set Peptides (UTS-1 – UTS-161)

H-D-Pro-D-Pro-D-Pro-NH$_2$ · TFA (UTS-1)

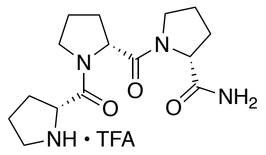

The peptide was synthesized according to the general procedure B for solid phase peptide synthesis.
^{1}H-NMR (400 MHz, D$_2$O) δ 4.81 (m, 1H), 4.66 (dd, *J* = 9.0, 5.9 Hz, 1H), 4.40 (dd, *J* = 8.6, 5.5 Hz, 1H), 3.84 (dt, *J* = 10.1, 6.8 Hz, 1H), 3.75 (ddd, *J* = 11.2, 9.9, 5.4 Hz, 1H), 3.68 (dd, *J* = 10.2, 6.8 Hz, 1H), 3.59 (dt, *J* = 10.1, 7.2 Hz, 1H), 3.44 (qt, *J* = 11.5, 7.1 Hz, 2H), 2.58 (ddd, *J* = 12.5, 8.3, 6.2 Hz, 1H), 2.44 (ddd, *J* = 12.7, 8.3, 6.3 Hz, 1H), 2.34 (ddt, *J* = 12.7, 8.5, 7.0 Hz, 1H), 2.15 – 2.02 (m, 7H), 2.02 – 1.90 (m, 2H). **^{13}C-NMR** (101 MHz, D$_2$O) δ 176.8, 171.6, 167.6, 60.3, 59.1, 59.0, 47.7, 46.6, 29.5, 28.2, 28.0, 24.6, 24.6, 23.8. **HR-MS** (ESI) m/z: calc. for $C_{15}H_{25}N_4O_3$ 309.1921 (M+H)$^+$, found 309.1921.

H-D-Pro-L-(4*S*)-Flp-D-Pro-NH$_2$ · TFA (UTS-2)

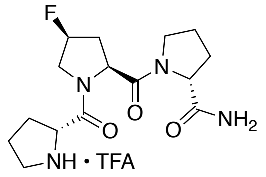

The peptide was synthesized according to the general procedure B for solid phase peptide synthesis.
^{1}H-NMR (400 MHz, CDCl$_3$) δ 5.59 – 5.32 (m, 1H), 5.04 – 4.93 (m, 1H), 4.67 (dd, *J* = 8.9, 7.1 Hz, 1H), 4.42 (ddd, *J* = 21.7, 8.7, 4.0 Hz, 1H), 4.11 (dd, *J* = 24.0, 12.7 Hz, 1H), 4.04 – 3.90 (m, 1H), 3.90 – 3.80 (m, 1H), 3.72 – 3.57 (m, 1H), 3.52 – 3.37 (m, 2H), 2.80 – 2.52 (m, 2H), 2.45 (dd, *J* = 20.8, 15.5 Hz, 1H), 2.38 – 2.24 (m, 1H), 2.19 – 1.94 (m, 6H). **^{13}C-NMR** (101 MHz, CDCl$_3$) δ 179.6, 173.3, 170.5, 95.3 (d, *J* = 176.9 Hz), 63.3, 61.8, 61.1, 56.4 (d, *J* = 23.5 Hz), 50.1, 49.1, 37.0 (d, *J* = 21.7 Hz), 32.0, 30.4, 26.8, 26.5. **HR-MS** (ESI) m/z: calc. for $C_{15}H_{24}FN_4O_3$ 327.1827 (M+H)$^+$, found 327.1818.

H-D-Pro-L-Leu-D-Pro-NH$_2$ · TFA (UTS-3)

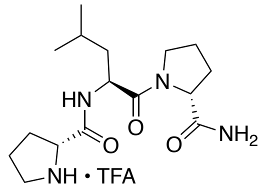

The peptide was synthesized according to the general procedure B for solid phase peptide synthesis.
^{1}H-NMR (400 MHz, D$_2$O) δ 4.70 (dd, *J* = 10.5, 3.7 Hz, 2H), 4.40 (ddd, *J* = 16.4, 8.3, 4.8 Hz, 2H), 3.92 (dt, *J* = 11.6, 6.1 Hz, 1H), 3.73 – 3.63 (m, 1H), 3.42 (qd, *J* = 9.7, 5.0 Hz, 2H), 2.48 (q, *J* = 10.3 Hz, 1H), 2.37 – 2.23 (m, 1H), 2.04 (ddd, *J* = 26.9, 10.7, 6.3 Hz, 6H), 1.65 (dd, *J* = 15.3, 4.9 Hz, 2H), 1.61 – 1.49 (m, 1H), 1.11 – 0.78 (m, 6H). **^{13}C-NMR** (101 MHz, D$_2$O) δ 177.0, 172.7, 169.5, 60.7, 59.7, 50.8, 47.7, 46.5, 38.7, 30.0, 29.5, 24.5, 24.2, 23.8, 22.4, 20.3. **HR-MS** (ESI) m/z: calc. for $C_{16}H_{29}N_4O_3$ 325.2234 (M+H)$^+$, found 325.2229.

H-D-Pro-D-Leu-D-Pro-NH$_2$ · TFA (UTS-4)

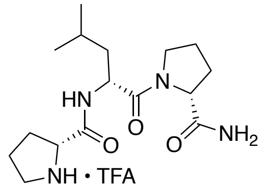

The peptide was synthesized according to the general procedure B for solid phase peptide synthesis.
^{1}H-NMR (400 MHz, D$_2$O) δ 4.68 – 4.62 (m, 1H), 4.41 (ddd, *J* = 11.4, 8.3, 5.9 Hz, 2H), 3.87 (dt, *J* = 10.1, 6.6 Hz, 1H), 3.69 (dt, *J* = 10.1, 6.9 Hz, 1H), 3.50 – 3.34 (m, 2H), 2.53 – 2.43 (m, 1H), 2.34 (ddt, *J* = 12.9, 8.5, 6.7 Hz, 1H), 2.14 – 2.01 (m, 5H), 1.95 (dq, *J* = 13.1, 6.9 Hz, 1H), 1.74 (dp, *J* = 13.1, 6.6 Hz, 1H), 1.67 – 1.60 (m, 2H), 1.01 – 0.91 (m, 6H). **^{13}C-NMR** (101 MHz, D$_2$O) δ 176.7, 172.5, 169.5, 60.4, 59.4, 50.8, 47.8, 46.5, 38.6, 29.8, 29.5, 24.7, 24.4, 23.7, 22.4, 20.3. **HR-MS** (ESI) m/z: calc. for $C_{16}H_{29}N_4O_3$ 325.2234 (M+H)$^+$, found 325.2227.

H-D-Pro-L-Gln-D-Pro-NH$_2$ · TFA (UTS-5)

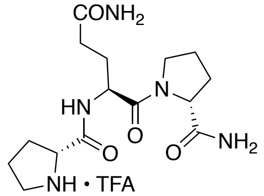

The peptide was synthesized according to the general procedure B for solid phase peptide synthesis.
^{1}H-NMR (400 MHz, D_2O) δ 4.69 (dd, *J* = 9.5, 4.5 Hz, 1H), 4.43 – 4.31 (m, 2H), 3.82 (dt, *J* = 12.1, 6.3 Hz, 1H), 3.68 (dt, *J* = 10.4, 7.0 Hz, 1H), 3.38 (ddt, *J* = 18.2, 11.4, 5.6 Hz, 2H), 2.51 – 2.41 (m, 1H), 2.37 (td, *J* = 7.2, 3.9 Hz, 2H), 2.28 (dq, *J* = 12.0, 8.0 Hz, 1H), 2.14 – 1.87 (m, 8H). **^{13}C-NMR** (101 MHz, D_2O) δ 177.5, 176.8, 171.3, 169.6, 60.6, 59.6, 51.3, 47.8, 46.4, 30.6, 29.8, 29.6, 25.7, 24.2, 23.7. **HR-MS** (ESI) m/z: calc. for $C_{15}H_{26}N_5O_4$ 340.1979 $(M+H)^+$, found 340.1980.

H-D-Pro-D-Gln-D-Pro-NH2 · TFA (UTS-6)

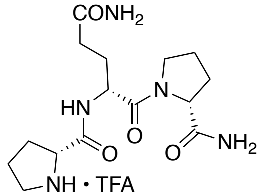

The peptide was synthesized according to the general procedure B for solid phase peptide synthesis.
^{1}H-NMR (400 MHz, D_2O) δ 4.66 (dd, *J* = 9.2, 4.8 Hz, 1H), 4.38 (td, *J* = 9.5, 5.8 Hz, 2H), 3.79 (dd, *J* = 11.1, 5.8 Hz, 1H), 3.72 – 3.62 (m, 1H), 3.38 (tq, *J* = 11.7, 6.2 Hz, 3H), 2.42 (dt, *J* = 15.5, 7.2 Hz, 3H), 2.30 (dq, *J* = 13.8, 6.9 Hz, 1H), 2.13 (td, *J* = 13.1, 7.4 Hz, 1H), 1.98 (ddq, *J* = 32.5, 12.6, 6.7 Hz, 7H). **^{13}C-NMR** (101 MHz, D_2O) δ 177.8, 176.6, 171.1, 169.5, 60.7, 59.4, 51.2, 47.9, 46.5, 30.6, 29.7, 29.5, 25.9, 24.6, 23.6. **HR-MS** (ESI) m/z: calc. for $C_{15}H_{26}N_5O_4$ 340.1979 $(M+H)^+$, found 340.1980.

H-D-Pro-L-Glu-D-Pro-NH$_2$ · TFA (UTS-7)

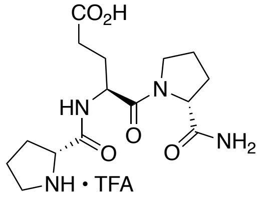

The peptide was synthesized according to the general procedure B for solid phase peptide synthesis.
^{1}H-NMR (400 MHz, D_2O) δ 4.41 (ddd, *J* = 13.5, 8.5, 5.3 Hz, 2H), 3.92 – 3.82 (m, 1H), 3.75 (dt, *J* = 10.4, 7.0 Hz, 1H), 3.43 (ddt, *J* = 18.3, 11.5, 5.6 Hz, 2H), 2.50 (tdd, *J* = 6.8, 5.9, 3.3 Hz, 3H), 2.37 – 2.28 (m, 1H), 2.20 – 2.03 (m, 7H), 2.02 – 1.90 (m, 2H). **^{13}C-NMR** (101 MHz, D_2O) δ 176.9, 171.4, 169.6, 60.6, 59.7, 51.2, 47.8, 46.5, 29.8, 29.6, 29.6, 25.2, 24.3, 23.8. **HR-MS** (ESI) m/z: calc. for $C_{15}H_{25}N_4O_5$ 341.1819 $(M+H)^+$, found 341.1820.

H-D-Pro-D-Glu-D-Pro-NH$_2$ · TFA (UTS-8)

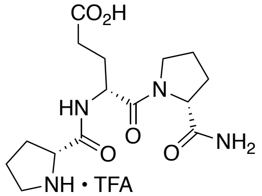

The peptide was synthesized according to the general procedure B for solid phase peptide synthesis.
^{1}H-NMR (400 MHz, D_2O) δ 4.76 (dd, *J* = 10.2, 5.1 Hz, 1H), 4.42 (ddd, *J* = 12.7, 8.4, 5.9 Hz, 2H), 3.84 (dt, *J* = 10.3, 6.5 Hz, 1H), 3.74 (dt, *J* = 10.3, 6.9 Hz, 1H), 3.43 (tdd, *J* = 11.4, 6.9, 4.5 Hz, 2H), 2.56 (td, *J* = 7.1, 5.3 Hz, 2H), 2.52 – 2.43 (m, 2H), 2.39 – 2.29 (m, 1H), 2.26 – 2.15 (m, 1H), 2.14 – 2.01 (m, 4H), 2.01 – 1.91 (m, 2H). **^{13}C-NMR** (101 MHz, D_2O) δ 177.0, 176.7, 171.2, 169.6, 60.4, 59.5, 51.2, 47.9, 46.5, 29.7, 29.6, 29.5, 25.3, 24.7, 23.7. **HR-MS** (ESI) m/z: calc. for $C_{15}H_{25}N_4O_5$ 341.1819 $(M+H)^+$, found 341.1820.

H-D-Pro-L-Tyr-D-Pro-NH$_2$ · TFA (UTS-9)

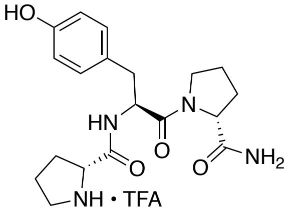

The peptide was synthesized according to the general procedure B for solid phase peptide synthesis.
^{1}H-NMR (400 MHz, D_2O) δ 7.16 – 7.11 (m, 2H), 6.85 – 6.81 (m, 2H), 4.85 (t, *J* = 8.0 Hz, 1H), 4.32 (dd, *J* = 8.6, 6.3 Hz, 1H), 4.26 (dd, *J* = 8.6, 4.1 Hz, 1H), 3.69 (dt, *J* = 10.5, 6.5 Hz, 1H), 3.37 – 3.27 (m, 2H), 3.12 (dt, *J* = 10.2, 6.9 Hz, 1H), 3.04 – 2.96 (m, 1H), 2.90 (dd, *J* = 13.5, 8.1 Hz, 1H), 2.31 (ddt, *J* = 13.3, 8.6, 6.4 Hz, 1H), 2.13 – 2.02 (m, 1H), 1.98 (dt, *J* = 13.5, 6.8 Hz, 1H), 1.93 – 1.81 (m, 3H), 1.81 – 1.68 (m, 2H). **^{13}C-NMR** (101 MHz, D_2O) δ 176.8, 171.5, 169.1, 154.6, 130.7, 127.4, 115.4,

60.4, 59.5, 53.4, 47.7, 46.4, 35.9, 29.9, 29.4, 24.0, 23.5. **HR-MS** (ESI) m/z: calc. for $C_{19}H_{27}N_4O_4$ 375.2027 $(M+H)^+$, found 375.2028.

H-D-Pro-D-Tyr-D-Pro-NH₂ · TFA (UTS-10)

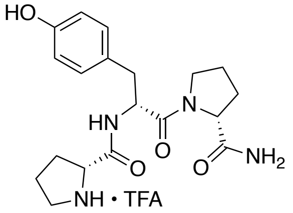

The peptide was synthesized according to the general procedure B for solid phase peptide synthesis.
^{1}**H-NMR** (400 MHz, D_2O) δ 7.20 (d, *J* = 8.5 Hz, 2H), 6.88 – 6.82 (m, 2H), 4.86 (dd, *J* = 8.8, 6.2 Hz, 2H), 4.38 – 4.25 (m, 2H), 3.79 (dt, *J* = 10.3, 6.7 Hz, 1H), 3.43 – 3.28 (m, 4H), 3.09 (dd, *J* = 14.2, 6.2 Hz, 1H), 2.94 – 2.84 (m, 1H), 2.40 (qd, *J* = 9.1, 4.0 Hz, 2H), 1.98 (tt, *J* = 12.8, 6.3 Hz, 3H), 1.74 – 1.66 (m, 2H). ^{13}**C-NMR** ^{13}C NMR (101 MHz, D_2O) δ 176.5, 171.4, 169.1, 154.6, 130.6, 127.8, 115.5, 60.4, 59.3, 53.4, 48.0, 46.4, 35.4, 29.7, 29.4, 24.5, 23.6. **HR-MS** (ESI) m/z: calc. for $C_{19}H_{27}N_4O_4$ 375.2027 $(M+H)^+$, found 375.2025.

H-D-Pro-CyLeu-D-Pro-NH₂ · TFA (UTS-11)

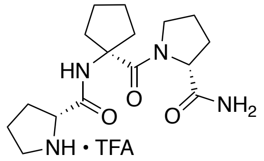

The peptide was synthesized according to the general procedure B for solid phase peptide synthesis.
^{1}**H-NMR** (400 MHz, D_2O) δ 4.45 – 4.34 (m, 2H), 3.68 (dt, *J* = 10.6, 6.4 Hz, 1H), 3.56 (dt, *J* = 10.6, 6.4 Hz, 1H), 3.43 (tdd, *J* = 11.8, 8.2, 4.3 Hz, 2H), 2.50 (dddd, *J* = 11.3, 7.9, 4.9, 2.6 Hz, 1H), 2.38 – 2.16 (m, 1H), 2.09 (qt, *J* = 6.8, 2.7 Hz, 2H), 2.04 – 1.84 (m, 4H), 1.83 – 1.68 (m, 5H). ^{13}**C-NMR** ^{13}C NMR (101 MHz, D_2O) δ 177.6, 173.5, 168.5, 67.1, 61.9, 59.6, 48.8, 46.2, 36.1, 35.3, 29.8, 28.6, 25.2, 23.7, 23.6. **HR-MS** (ESI) m/z: calc. for $C_{16}H_{26}N_4NaO_3$ 345.1897 $(M+Na)^+$, found 345.1894.

H-D-Pro-D-Ind-D-Pro-NH₂ · TFA (UTS-12)

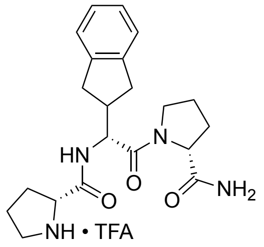

The peptide was synthesized according to the general procedure B for solid phase peptide synthesis.
^{1}**H-NMR** (400 MHz, D_2O) δ 7.37 – 7.28 (m, 2H), 7.28 – 7.22 (m, 2H), 4.75 – 4.70 (m, 1H), 4.42 (dd, *J* = 8.4, 5.8 Hz, 1H), 4.40 – 4.34 (m, 1H), 3.84 (dt, *J* = 12.0, 6.2 Hz, 1H), 3.63 (dt, *J* = 10.1, 6.9 Hz, 1H), 3.41 (dddd, *J* = 18.8, 11.7, 7.3, 3.7 Hz, 2H), 3.16 – 3.03 (m, 2H), 3.01 – 2.82 (m, 2H), 2.49 – 2.41 (m, 1H), 2.33 (ddd, *J* = 14.9, 7.3, 4.5 Hz, 1H), 2.13 – 1.93 (m, 7H). ^{13}**C-NMR** (101 MHz, D_2O) δ 176.6, 171.3, 169.5, 142.3, 142.1, 126.7, 124.7, 124.6, 60.4, 59.4, 54.7, 48.3, 46.6, 40.8, 34.8, 34.7, 29.8, 29.6, 24.7, 23.7. **HR-MS** (ESI) m/z: calc. for $C_{21}H_{29}N_4O_3$ 385.2234 $(M+H)^+$, found 385.2233.

H-D-Pro-D-Pro-L-Flp-NH₂ · TFA (UTS-13)

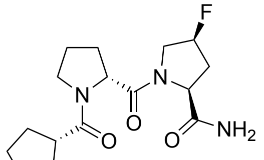

The peptide was synthesized according to the general procedure B for solid phase peptide synthesis.
^{1}**H-NMR** (400 MHz, D_2O) δ 5.35 (d, *J* = 51.9 Hz, 1H), 4.66 (t, *J* = 7.6 Hz, 1H), 4.54 (dd, *J* = 9.2, 6.1 Hz, 2H), 4.17 (dd, *J* = 24.6, 12.9 Hz, 1H), 3.85 (ddd, *J* = 38.5, 13.0, 3.9 Hz, 1H), 3.65 (td, *J* = 9.0, 4.5 Hz, 1H), 3.54 – 3.42 (m, 1H), 3.31 (ddd, *J* = 15.6, 7.6, 5.1 Hz, 2H), 2.52 – 2.38 (m, 2H), 2.35 (d, *J* = 12.3 Hz, 1H), 2.28 (dq, *J* = 12.9, 6.9 Hz, 1H), 2.09 – 1.84 (m, 6H). ^{13}**C-NMR** (101 MHz, D_2O) δ 176.2, 172.7, 167.8, 93.2 (d, *J* = 173.6 Hz), 59.2, 59.2, 59.1, 54.0, 47.7, 46.5, 35.8 (d, *J* = 21.0 Hz), 28.1, 27.6, 24.8, 23.7. **HR-MS** (ESI) m/z: calc. for $C_{15}H_{24}FN_4O_3$ 327.1827 $(M+H)^+$, found 327.1825.

H-D-Pro-D-Pro-L-Leu-NH_2 · TFA (UTS-14):

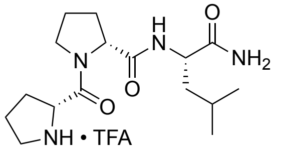

The peptide was synthesized according to the general procedure B for solid phase peptide synthesis.
^{1}H-NMR (400 MHz, D_2O) δ 4.66 – 4.58 (m, 1H), 4.51 – 4.43 (m, 1H), 4.31 (dd, *J* = 10.6, 4.0 Hz, 1H), 3.71 (ddd, *J* = 10.2, 7.2, 4.9 Hz, 1H), 3.57 (dt, *J* = 10.2, 7.2 Hz, 1H), 3.44 – 3.33 (m, 2H), 2.60 – 2.50 (m, 1H), 2.38 – 2.28 (m, 1H), 2.15 – 1.96 (m, 5H), 1.96 – 1.85 (m, 1H), 1.70 – 1.56 (m, 3H), 0.91 (d, *J* = 6.1 Hz, 3H), 0.85 (d, *J* = 5.8 Hz, 3H). **^{13}C-NMR** (101 MHz, D_2O) δ 177.5, 174.0, 168.0, 60.7, 59.0, 51.9, 47.7, 46.5, 39.7, 29.4, 28.2, 24.7, 24.4, 23.8, 22.2, 20.1. **HR-MS** (ESI) m/z: calc. for $C_{16}H_{29}N_4O_3$ 325.2234 $(M+H)^+$, found 325.2232.

H-D-Pro-D-Pro-D-Leu-NH_2 · TFA (UTS-15)

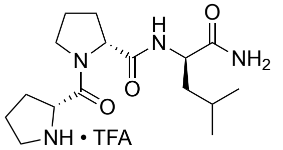

The peptide was synthesized according to the general procedure B for solid phase peptide synthesis.
^{1}H-NMR (400 MHz, D_2O) δ 4.61 (dd, *J* = 8.7, 6.2 Hz, 1H), 4.47 (dd, *J* = 8.4, 6.1 Hz, 1H), 4.25 (dd, *J* = 9.6, 5.2 Hz, 1H), 3.69 (dt, *J* = 10.1, 6.3 Hz, 1H), 3.56 (dt, *J* = 10.2, 7.1 Hz, 1H), 3.39 (tdd, *J* = 11.6, 6.9, 4.5 Hz, 2H), 2.61 – 2.49 (m, 1H), 2.37 – 2.26 (m, 1H), 2.12 – 1.96 (m, 5H), 1.90 (dq, *J* = 13.7, 6.9 Hz, 1H), 1.66 (ddd, *J* = 14.6, 10.0, 5.0 Hz, 2H), 1.55 (ddd, *J* = 13.9, 9.6, 5.3 Hz, 1H), 0.92 (d, *J* = 6.3 Hz, 3H), 0.88 (d, *J* = 6.3 Hz, 3H). **^{13}C-NMR** (101 MHz, D_2O) δ 177.4, 173.7, 168.0, 60.2, 59.0, 52.3, 47.6, 46.6, 39.7, 29.3, 28.3, 24.6, 24.3, 23.8, 22.1, 20.6. **HR-MS** (ESI) m/z: calc. for $C_{16}H_{29}N_4O_3$ 325.2234 $(M+H)^+$, found 325.2229.

H-D-Pro-D-Pro-L-Gln-NH_2 · TFA (UTS-16)

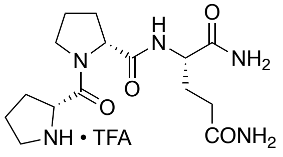

The peptide was synthesized according to the general procedure B for solid phase peptide synthesis.
^{1}H-NMR (400 MHz, D_2O) δ 4.70 – 4.62 (m, 1H), 4.50 (dd, *J* = 8.1, 6.6 Hz, 1H), 4.35 (dd, *J* = 9.7, 4.7 Hz, 1H), 3.75 (ddd, *J* = 10.2, 7.1, 5.0 Hz, 1H), 3.61 (dt, *J* = 10.1, 7.2 Hz, 1H), 3.42 (dtd, *J* = 13.8, 7.0, 4.0 Hz, 2H), 2.66 – 2.51 (m, 1H), 2.38 (q, *J* = 7.3 Hz, 3H), 2.21 (dtd, *J* = 14.3, 7.6, 4.8 Hz, 1H), 2.16 – 1.92 (m, 7H). **^{13}C-NMR** (101 MHz, D_2O) δ 177.8, 175.9, 174.0, 168.1, 60.8, 59.1, 52.8, 47.7, 46.6, 31.2, 29.3, 28.3, 26.7, 24.7, 23.8. **HR-MS** (ESI) m/z: calc. for $C_{15}H_{25}N_5NaO_4$ 362.1799 $(M+Na)^+$, found 362.1798.

H-D-Pro-D-Pro-D-Gln-NH_2 · TFA (UTS-17)

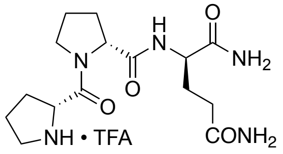

The peptide was synthesized according to the general procedure B for solid phase peptide synthesis.
^{1}H-NMR (400 MHz, D_2O) δ 4.66 (dd, *J* = 8.6, 5.6 Hz, 1H), 4.51 (dd, *J* = 8.4, 6.2 Hz, 1H), 4.29 (dd, *J* = 9.0, 5.4 Hz, 1H), 3.74 (ddd, *J* = 10.2, 7.0, 5.4 Hz, 1H), 3.62 (dt, *J* = 10.1, 7.1 Hz, 1H), 3.52 – 3.35 (m, 2H), 2.67 – 2.53 (m, 1H), 2.43 (t, *J* = 7.5 Hz, 2H), 2.41 – 2.32 (m, 1H), 2.20 – 1.90 (m, 8H). **^{13}C-NMR** (101 MHz, D_2O) δ 177.9, 175.8, 173.8, 168.1, 60.5, 59.1, 53.0, 47.7, 46.6, 31.1, 29.3, 28.3, 26.8, 24.6, 23.8. **HR-MS** (ESI) m/z: calc. for $C_{15}H_{25}N_5NaO_4$ 362.1799 $(M+Na)^+$, found 362.1798.

H-D-Pro-D-Pro-L-Glu-NH_2 · TFA (UTS-18)

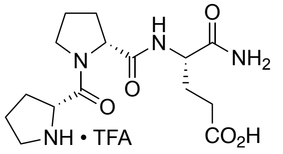

The peptide was synthesized according to the general procedure B for solid phase peptide synthesis.
^{1}H-NMR (400 MHz, D_2O) δ 4.65 – 4.58 (m, 1H), 4.49 – 4.43 (m, 1H), 4.35 (dd, *J* = 9.9, 4.7 Hz, 1H), 3.70 (ddd, *J* = 10.2, 7.1, 5.0 Hz, 1H), 3.57 (dt, *J* = 10.0, 7.1 Hz, 1H), 3.46 – 3.31 (m, 2H), 2.54 (dq, *J* = 12.0, 5.9 Hz, 1H), 2.45 (t, *J* = 7.2 Hz, 2H), 2.38 – 2.29 (m, 1H), 2.19 (dtd, *J* = 15.2, 7.7, 4.8 Hz, 1H), 2.11 – 1.87 (m, 7H). **^{13}C-NMR** (101 MHz,

D_2O) δ 177.1, 175.9, 174.0, 168.0, 60.8, 59.1, 52.6, 47.7, 46.5, 30.2, 29.3, 28.2, 26.1, 24.7, 23.8. **HR-MS** (ESI) m/z: calc. for $C_{15}H_{25}N_4O_5$ 341.1819 $(M+H)^+$, found 341.1819.

H-D-Pro-D-Pro-D-Glu-NH$_2$ · TFA (UTS-19):

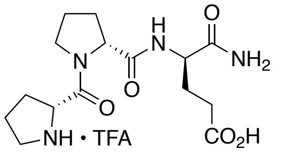

The peptide was synthesized according to the general procedure B for solid phase peptide synthesis.
^{1}H-NMR (400 MHz, D_2O) δ 4.65 (dd, *J* = 8.9, 5.7 Hz, 1H), 4.51 (dd, *J* = 8.4, 6.2 Hz, 1H), 4.34 (dd, *J* = 9.0, 5.5 Hz, 1H), 3.73 (dt, *J* = 10.2, 6.2 Hz, 1H), 3.61 (dt, *J* = 10.3, 7.1 Hz, 1H), 3.43 (qt, *J* = 11.6, 5.5 Hz, 2H), 2.65 – 2.48 (m, 3H), 2.43 – 2.31 (m, 1H), 2.21 – 1.90 (m, 8H). **^{13}C-NMR** (101 MHz, D_2O) δ 177.3, 175.8, 173.8, 168.1, 60.5, 59.1, 52.8, 47.7, 46.6, 30.0, 29.3, 28.3, 26.2, 24.7, 23.8. **HR-MS** (ESI) m/z: calc. for $C_{15}H_{25}N_4O_5$ 341.1819 $(M+H)^+$, found 341.1819.

H-D-Pro-D-Pro-L-Tyr-NH$_2$ · TFA (UTS-20):

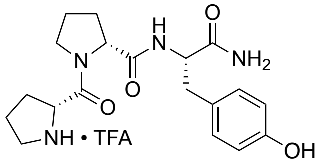

The peptide was synthesized according to the general procedure B for solid phase peptide synthesis.
^{1}H-NMR (400 MHz, D_2O) δ 7.21 – 7.14 (m, 2H), 6.89 – 6.83 (m, 2H), 4.71 (dd, *J* = 10.6, 4.9 Hz, 1H), 4.59 (dd, *J* = 8.9, 6.8 Hz, 1H), 4.40 (dd, *J* = 8.3, 6.1 Hz, 1H), 3.63 (dt, *J* = 10.2, 6.7 Hz, 1H), 3.48 (dt, *J* = 10.2, 7.1 Hz, 1H), 3.40 (ddt, *J* = 11.5, 6.7, 4.4 Hz, 2H), 3.27 (dd, *J* = 14.2, 4.9 Hz, 1H), 2.82 (dd, *J* = 14.2, 10.7 Hz, 1H), 2.53 (ddt, *J* = 13.1, 9.1, 6.2 Hz, 1H), 2.14 – 2.00 (m, 3H), 2.00 – 1.85 (m, 2H), 1.81 (dt, *J* = 12.7, 6.4 Hz, 1H), 1.36 (dq, *J* = 13.3, 6.8 Hz, 1H). **^{13}C-NMR** (101 MHz, D_2O) δ 175.9, 173.4, 168.0, 154.3, 130.5, 128.5, 115.4, 60.5, 59.0, 54.1, 47.5, 46.6, 36.4, 29.3, 28.3, 24.4, 23.8. **HR-MS** (ESI) m/z: calc. for $C_{19}H_{26}N_4NaO_4$ 397.1846 $(M+Na)^+$, found 397.1848.

H-D-Pro-D-Pro-D-Tyr-NH$_2$ · TFA (UTS-21):

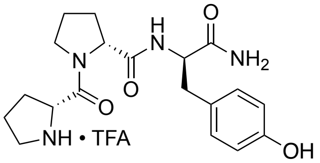

The peptide was synthesized according to the general procedure B for solid phase peptide synthesis.
^{1}H-NMR (500 MHz, D_2O) δ 7.21 – 7.10 (m, 2H), 6.88 (ddt, *J* = 9.6, 5.4, 3.0 Hz, 2H), 4.61 (dd, *J* = 8.8, 6.8 Hz, 1H), 4.51 (t, *J* = 7.4 Hz, 1H), 4.45 (dd, *J* = 8.5, 5.8 Hz, 1H), 3.68 (dt, *J* = 10.1, 6.6 Hz, 1H), 3.56 (dt, *J* = 9.9, 6.8 Hz, 1H), 3.42 (qt, *J* = 11.5, 7.1 Hz, 2H), 3.09 – 2.99 (m, 2H), 2.61 – 2.47 (m, 1H), 2.27 (dq, *J* = 13.0, 7.4 Hz, 1H), 2.14 – 1.81 (m, 6H). **^{13}C-NMR** (126 MHz, D_2O) δ 175.6, 173.3, 168.3, 154.5, 130.6, 128.0, 115.4, 60.5, 59.0, 54.7, 47.6, 46.6, 36.0, 29.1, 28.4, 24.5, 23.8. **HR-MS** (ESI) m/z: calc. for $C_{19}H_{27}N_4O_4$ 375.2027 $(M+H)^+$, found 375.2028.

H-D-Pro-D-Pro-CyLeu-NH$_2$ · TFA (UTS-22):

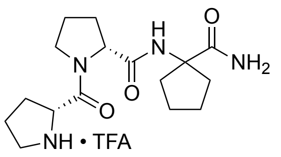

The peptide was synthesized according to the general procedure B for solid phase peptide synthesis.
^{1}H-NMR (500 MHz, D_2O) δ 4.68 – 4.61 (m, 1H), 4.46 (t, *J* = 7.4 Hz, 1H), 3.73 (ddd, *J* = 12.1, 7.1, 4.7 Hz, 1H), 3.63 – 3.53 (m, 1H), 3.51 – 3.35 (m, 2H), 2.64 – 2.52 (m, 1H), 2.33 (dq, *J* = 13.6, 6.6 Hz, 1H), 2.19 (dd, *J* = 14.3, 7.7 Hz, 1H), 2.08 (tt, *J* = 14.6, 7.1 Hz, 5H), 2.03 – 1.88 (m, 4H), 1.77 (s, 4H). **^{13}C-NMR** (126 MHz, D_2O) δ 179.3, 173.3, 167.9, 66.8, 60.5, 59.1, 47.7, 46.6, 37.3, 36.0, 29.0, 28.2, 24.7, 24.0, 23.9, 23.8. **HR-MS** (ESI) m/z: calc. for $C_{16}H_{26}N_4NaO_3$ 345.1897 $(M+Na)^+$, found 345.1899.

H-D-Pro-D-Pro-Abz-NH₂ · TFA (UTS-23):

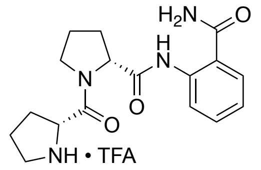

The peptide was synthesized according to the general procedure C for solid phase peptide synthesis.
^{1}H-NMR (400 MHz, D_2O) δ 7.70 (td, *J* = 8.3, 1.4 Hz, 2H), 7.65 – 7.56 (m, 1H), 7.41 (dt, *J* = 15.2, 8.1 Hz, 1H), 4.68 (dd, *J* = 8.9, 6.3 Hz, 1H), 4.62 (dd, *J* = 8.3, 4.9 Hz, 1H), 3.78 (dt, *J* = 10.7, 6.0 Hz, 1H), 3.74 – 3.66 (m, 1H), 3.53 – 3.34 (m, 2H), 2.66 – 2.54 (m, 1H), 2.42 (dt, *J* = 8.7, 5.9 Hz, 1H), 2.22 – 1.98 (m, 6H). **^{13}C-NMR** (101 MHz, D_2O) δ 172.9, 172.4, 168.5, 132.3, 128.6, 126.2, 124.4, 61.6, 59.2, 47.8, 46.6, 29.1, 28.4, 24.7, 23.8. **HR-MS** (ESI) m/z: calc. for $C_{17}H_{22}N_4NaO_3$ 353.1584 $(M+Na)^+$, found 353.1584.

H-D-Pro-D-Pro-D-Ind-NH₂ · TFA (UTS-24):

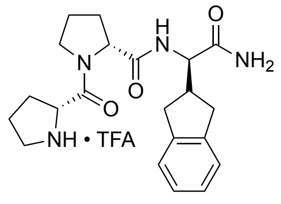

The peptide was synthesized according to the general procedure B for solid phase peptide synthesis.
^{1}H-NMR (400 MHz, D_2O) δ 7.38 – 7.28 (m, 2H), 7.24 (dd, *J* = 5.6, 3.2 Hz, 2H), 4.65 (dd, *J* = 8.9, 6.2 Hz, 1H), 4.55 (dd, *J* = 8.4, 6.0 Hz, 1H), 4.35 (d, *J* = 7.4 Hz, 1H), 3.71 (dt, *J* = 9.9, 6.3 Hz, 1H), 3.61 – 3.50 (m, 1H), 3.49 – 3.37 (m, 2H), 3.23 – 3.12 (m, 1H), 3.07 (dd, *J* = 12.3, 7.0 Hz, 1H), 2.86 (dq, *J* = 14.5, 7.3 Hz, 3H), 2.57 (dt, *J* = 13.1, 5.9 Hz, 1H), 2.41 – 2.29 (m, 1H), 2.16 – 1.98 (m, 5H), 1.93 (dp, *J* = 13.4, 6.2 Hz, 1H). **^{13}C-NMR** (101 MHz, D_2O) δ 175.7, 173.7, 168.1, 142.3, 142.1, 126.7, 124.6, 60.4, 59.1, 57.0, 47.7, 46.6, 40.9, 35.2, 29.2, 28.4, 24.6, 23.9. **HR-MS** (ESI) m/z: calc. for $C_{21}H_{29}N_4O_3$ 385.2234 $(M+H)^+$, found 385.2235.

H-D-Pro-L-(4*S*)-Flp-L-Flp-NH₂ · TFA (UTS-25):

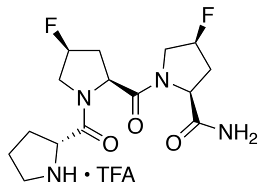

The peptide was synthesized according to the general procedure B for solid phase peptide synthesis.
^{1}H-NMR (400 MHz, D_2O) δ 5.62 – 5.47 (m, 1H), 5.47 – 5.32 (m, 1H), 4.96 – 4.88 (m, 1H), 4.73 – 4.65 (m, 2H), 4.21 – 3.77 (m, 4H), 3.53 – 3.33 (m, 2H), 2.88 – 2.35 (m, 5H), 2.21 – 2.07 (m, 2H), 2.02 (dq, *J* = 12.8, 7.4 Hz, 1H). **^{13}C-NMR** (101 MHz, D_2O) δ 175.6, 171.2, 168.1, 93.9 (d, *J* = 71.5 Hz), 92.2 (d, *J* = 73.8 Hz), 59.3, 59.1, 58.3, 54.0, 53.8, 53.7, 53.5, 46.5, 35.6 (d, *J* = 21.0 Hz), 34.9 (d, *J* = 21.7 Hz), 27.8, 23.9. **HR-MS** (ESI) m/z: calc. for $C_{15}H_{22}F_2N_4NaO_3$ 367.1552 $(M+Na)^+$, found 367.1552.

H-D-Pro-L-(4*S*)-Flp-L-Leu-NH₂ · TFA (UTS-26):

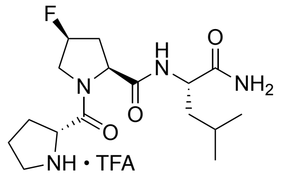

The peptide was synthesized according to the general procedure B for solid phase peptide synthesis.
^{1}H-NMR (400 MHz, D_2O) δ 5.53 (t, *J* = 3.5 Hz, 1H), 5.41 (t, *J* = 3.4 Hz, 1H), 4.75 – 4.67 (m, 2H), 4.37 (dd, *J* = 10.4, 4.5 Hz, 1H), 4.11 (dd, *J* = 23.1, 12.7 Hz, 1H), 3.93 (dtd, *J* = 37.8, 12.3, 4.2 Hz, 1H), 3.46 (qt, *J* = 11.4, 7.0 Hz, 2H), 2.68 (ddd, *J* = 14.9, 10.4, 3.6 Hz, 1H), 2.63 – 2.44 (m, 2H), 2.18 – 2.07 (m, 2H), 2.03 (dt, *J* = 12.1, 7.1 Hz, 1H), 1.76 – 1.54 (m, 3H), 0.93 (d, *J* = 6.1 Hz, 3H), 0.89 (d, *J* = 6.2 Hz, 3H). **^{13}C-NMR** (101 MHz, D_2O) δ 177.5, 172.9, 169.2, 92.8 (d, *J* = 175.3 Hz), 59.8, 59.5, 53.7 (d, *J* = 23.1 Hz), 52.1, 46.7, 39.5, 35.9 (d, *J* = 20.8 Hz), 27.8, 24.1, 24.0, 22.2, 20.3. **HR-MS** (ESI) m/z: calc. for $C_{16}H_{28}FN_4O_3$ 343.2140 $(M+H)^+$, found 343.2139.

H-D-Pro-L-(4*S*)-Flp-D-Leu-NH_2 · TFA (UTS-27):

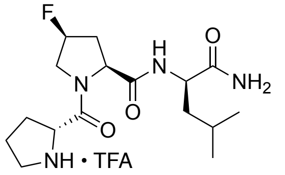

The peptide was synthesized according to the general procedure B for solid phase peptide synthesis.
^{1}H-NMR (400 MHz, D_2O) δ 5.55 – 5.42 (m, 1H), 5.42 – 5.28 (m, 1H), 4.72 (d, *J* = 10.2 Hz, 1H), 4.64 (dd, *J* = 8.8, 7.2 Hz, 1H), 4.30 (dd, *J* = 10.5, 4.1 Hz, 1H), 4.15 – 3.99 (m, 1H), 3.87 (ddd, *J* = 37.2, 12.7, 3.7 Hz, 1H), 3.40 (ddt, *J* = 18.5, 11.6, 5.8 Hz, 2H), 2.65 (ddt, *J* = 17.6, 11.0, 5.7 Hz, 1H), 2.54 (ddt, *J* = 15.7, 12.3, 5.3 Hz, 1H), 2.48 – 2.34 (m, 1H), 2.08 (q, *J* = 7.0 Hz, 2H), 2.04 – 1.94 (m, 1H), 1.73 – 1.53 (m, 3H), 0.91 (d, *J* = 5.6 Hz, 3H), 0.85 (t, *J* = 5.8 Hz, 3H). **^{13}C-NMR** (101 MHz, D_2O) δ 177.5, 172.8, 168.4, 92.8 (d, *J* = 175.7 Hz), 59.5, 59.3, 53.6 (d, *J* = 23.1 Hz), 52.1, 46.5, 39.7, 36.2 (d, *J* = 20.8 Hz), 27.7, 24.3, 23.9, 22.2, 20.1. **HR-MS** (ESI) m/z: calc. for $C_{16}H_{28}FN_4O_3$ 343.2140 $(M+H)^+$, found 343.2140.

H-D-Pro-L-(4*S*)-Flp-L-Gln-NH_2 · TFA (UTS-28):

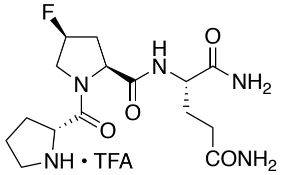

The peptide was synthesized according to the general procedure B for solid phase peptide synthesis.
^{1}H-NMR (400 MHz, D_2O) δ 5.59 – 5.33 (m, 1H), 4.77 – 4.68 (m, 2H), 4.36 (dt, *J* = 11.3, 5.1 Hz, 1H), 4.13 (dd, *J* = 23.1, 12.7 Hz, 1H), 3.92 (ddd, *J* = 37.8, 12.7, 3.5 Hz, 1H), 3.55 – 3.37 (m, 2H), 2.74 – 2.62 (m, 1H), 2.56 (ddd, *J* = 17.0, 8.2, 5.2 Hz, 2H), 2.44 – 2.35 (m, 2H), 2.24 – 2.08 (m, 3H), 2.01 (tdd, *J* = 16.6, 7.9, 5.7 Hz, 2H). **^{13}C-NMR** (101 MHz, D_2O) δ 178.0, 175.8, 172.9, 169.2, 92.9 (d, *J* = 175.1 Hz), 59.9, 59.5, 53.7 (d, *J* = 23.1 Hz), 52.9, 46.7, 35.9 (d, *J* = 20.9 Hz), 31.0, 27.8, 26.5, 24.0. **HR-MS** (ESI) m/z: calc. for $C_{15}H_{24}FN_5NaO_4$ 380.1705 $(M+Na)^+$, found 380.1703.

H-D-Pro-L-(4*S*)-Flp-D-Gln-NH_2 · TFA (UTS-29):

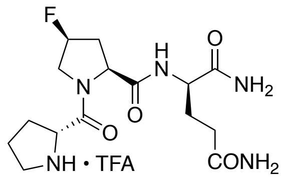

The peptide was synthesized according to the general procedure B for solid phase peptide synthesis.
^{1}H-NMR (400 MHz, D_2O) δ 5.38 (d, *J* = 28.7 Hz, 1H), 4.87 (d, *J* = 6.5 Hz, 1H), 4.72 – 4.58 (m, 2H), 4.41 (q, *J* = 5.5 Hz, 1H), 4.16 – 3.75 (m, 2H), 3.51 – 3.35 (m, 2H), 2.75 – 2.60 (m, 1H), 2.55 – 2.33 (m, 4H), 2.25 (ddd, *J* = 14.4, 7.4, 4.7 Hz, 1H), 2.17 – 1.98 (m, 4H). **^{13}C-NMR** (101 MHz, D_2O) δ 177.6, 175.5, 172.0, 169.6, 93.3 (d, *J* = 174.9 Hz), 59.7, 59.0, 53.9 (d, *J* = 23.3 Hz), 51.2, 46.4, 35.8 (d, *J* = 20.6 Hz), 30.5, 29.7, 26.0, 23.7. **HR-MS** (ESI) m/z: calc. for $C_{15}H_{25}FN_5O_4$ 358.1885 $(M+H)^+$, found 358.1884.

H-D-Pro-L-(4*S*)-Flp-L-Glu-NH_2 · TFA (UTS-30):

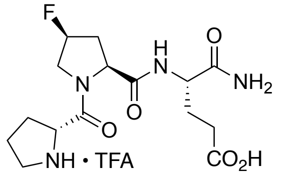

The peptide was synthesized according to the general procedure B for solid phase peptide synthesis.
^{1}H-NMR (400 MHz, D_2O) δ 5.50 (t, *J* = 3.4 Hz, 1H), 5.37 (s, 1H), 4.68 (dd, *J* = 9.6, 5.1 Hz, 2H), 4.36 (dd, *J* = 9.9, 4.8 Hz, 1H), 4.08 (dd, *J* = 23.1, 12.7 Hz, 1H), 3.87 (ddd, *J* = 37.9, 12.7, 3.6 Hz, 1H), 3.42 (qt, *J* = 11.5, 7.0 Hz, 2H), 2.63 (ddd, *J* = 14.2, 10.3, 3.5 Hz, 1H), 2.58 – 2.41 (m, 4H), 2.19 – 2.03 (m, 3H), 2.03 – 1.89 (m, 2H). **^{13}C-NMR** (101 MHz, D_2O) δ 177.4, 175.8, 172.9, 169.1, 92.8 (d, *J* = 175.1 Hz), 59.8, 59.4, 53.7 (d, *J* = 23.3 Hz), 52.7, 46.6, 35.8 (d, *J* = 21.4 Hz), 29.9, 27.7, 25.8, 23.9. **HR-MS** (ESI) m/z: calc. for $C_{15}H_{24}FN_4O_5$ 359.1725 $(M+H)^+$, found 359.1725.

H-D-Pro-L-(4*S*)-Flp-D-Glu-NH$_2$ · TFA (UTS-31):

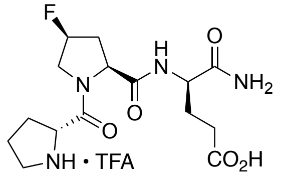

The peptide was synthesized according to the general procedure B for solid phase peptide synthesis.
^{1}H-NMR (500 MHz, D$_2$O) δ 5.57 – 5.46 (m, 1H), 5.44 (t, *J* = 3.5 Hz, 1H), 4.76 – 4.66 (m, 2H), 4.38 (ddd, *J* = 9.8, 4.8, 0.9 Hz, 1H), 4.18 – 4.07 (m, 1H), 3.99 – 3.83 (m, 1H), 3.52 – 3.37 (m, 2H), 2.67 (ddd, *J* = 14.2, 10.4, 3.7 Hz, 1H), 2.62 – 2.51 (m, 2H), 2.49 (t, *J* = 7.2 Hz, 3H), 2.19 (dtd, *J* = 15.0, 7.6, 5.1 Hz, 1H), 2.15 – 2.07 (m, 2H), 2.01 (dddt, *J* = 21.1, 13.9, 9.8, 6.6 Hz, 2H). **^{13}C-NMR** (126 MHz, D$_2$O) δ 177.4, 175.9, 172.9, 168.7, 93.0 (d, *J* = 175.0 Hz), 59.7, 59.4, 53.6 (d, *J* = 23.1 Hz), 52.9, 46.6, 36.0 (d, *J* = 21.0 Hz), 30.2, 27.7, 25.9, 23.9. **HR-MS** (ESI) m/z: calc. for $C_{15}H_{24}FN_4O_5$ 359.1725 (M+H)$^+$, found 359.1726.

H-D-Pro-L-(4*S*)-Flp-L-Tyr-NH$_2$ · TFA (UTS-32):

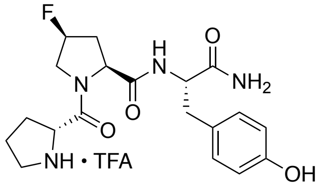

The peptide was synthesized according to the general procedure B for solid phase peptide synthesis.
^{1}H-NMR (500 MHz, D$_2$O) δ 7.20 – 7.15 (m, 2H), 6.88 – 6.83 (m, 2H), 5.45 (dt, *J* = 16.7, 3.7 Hz, 1H), 5.39 – 5.31 (m, 1H), 4.70 – 4.63 (m, 2H), 4.56 (dd, *J* = 8.3, 6.7 Hz, 1H), 4.06 (dd, *J* = 23.1, 12.7 Hz, 1H), 3.95 – 3.81 (m, 1H), 3.51 – 3.39 (m, 2H), 3.08 (dd, *J* = 14.1, 6.7 Hz, 1H), 2.98 (dd, *J* = 14.1, 8.3 Hz, 1H), 2.64 – 2.47 (m, 2H), 2.43 – 2.33 (m, 1H), 2.15 – 2.06 (m, 2H), 2.00 (dq, *J* = 13.0, 7.4 Hz, 1H). **^{13}C-NMR** (126 MHz, D$_2$O) δ 175.6, 172.5, 168.9, 154.4, 130.6, 128.2, 115.4, 92.6 (d, *J* = 175.6 Hz), 59.7, 59.4, 55.0, 53.7 (d, *J* = 23.4 Hz), 46.6, 35.9, 35.7 (d, *J* = 20.7 Hz), 27.7, 23.9. **HR-MS** (ESI) m/z: calc. for $C_{19}H_{25}FN_4NaO_4$ 415.1752 (M+Na)$^+$, found 415.1753.

H-D-Pro-L-(4*S*)-Flp-D-Tyr-NH$_2$ · TFA (UTS-33):

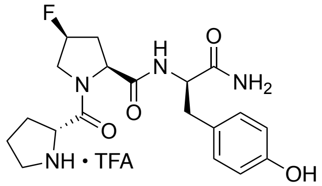

The peptide was synthesized according to the general procedure B for solid phase peptide synthesis.
^{1}H-NMR (400 MHz, D$_2$O) δ 7.24 – 7.11 (m, 2H), 6.92 – 6.80 (m, 2H), 5.38 (t, *J* = 3.8 Hz, 1H), 5.25 (t, *J* = 3.8 Hz, 1H), 4.69 – 4.58 (m, 3H), 4.01 (dd, *J* = 23.6, 12.8 Hz, 1H), 3.91 – 3.74 (m, 1H), 3.53 – 3.32 (m, 2H), 3.22 (dd, *J* = 14.2, 5.5 Hz, 1H), 2.87 (dd, *J* = 14.2, 10.1 Hz, 1H), 2.60 – 2.32 (m, 2H), 2.09 (p, *J* = 7.1 Hz, 2H), 1.99 (dt, *J* = 13.0, 7.3 Hz, 1H), 1.95 – 1.81 (m, 1H). **^{13}C-NMR** (101 MHz, D$_2$O) δ 175.9, 172.4, 168.3, 154.4, 130.4, 128.4, 115.5, 92.4 (d, *J* = 176.0 Hz), 59.5, 59.3, 54.5, 53.7 (d, *J* = 23.3 Hz), 46.6, 36.2, 35.9 (d, *J* = 20.8 Hz), 27.7, 23.9. **HR-MS** (ESI) m/z: calc. for $C_{19}H_{25}FN_4NaO_4$ 415.1752 (M+Na)$^+$, found 415.1753.

H-D-Pro-L-(4*S*)-Flp-CyLeu-NH$_2$ · TFA (UTS-34):

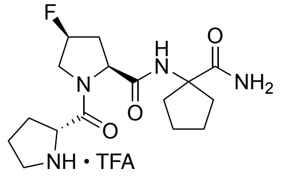

The peptide was synthesized according to the general procedure B for solid phase peptide synthesis.
^{1}H-NMR (400 MHz, D$_2$O) δ 5.53 (t, *J* = 3.7 Hz, 1H), 5.43 – 5.32 (m, 1H), 4.72 – 4.63 (m, 2H), 4.09 (ddd, *J* = 23.6, 12.5, 1.5 Hz, 1H), 3.90 (ddd, *J* = 36.7, 12.8, 3.8 Hz, 1H), 3.45 (qt, *J* = 11.6, 7.1 Hz, 2H), 2.66 (ddd, *J* = 14.6, 10.3, 3.9 Hz, 1H), 2.55 (dtd, *J* = 12.6, 6.4, 3.3 Hz, 2H), 2.46 (dd, *J* = 20.3, 15.3 Hz, 1H), 2.11 (dq, *J* = 9.9, 6.2 Hz, 4H), 2.02 – 1.89 (m, 3H), 1.83 – 1.69 (m, 5H). **^{13}C-NMR** (101 MHz, D$_2$O) δ 179.3, 172.2, 168.5, 92.8 (d, *J* = 175.9 Hz), 67.0, 59.7, 59.3, 53.7 (d, *J* = 23.3 Hz), 46.6, 36.9, 36.1, 35.8 (d, *J* = 21.4 Hz), 27.8, 23.9, 23.9, 23.8. **HR-MS** (ESI) m/z: calc. for $C_{16}H_{25}FN_4NaO_3$ 363.1803 (M+Na)$^+$, found 363.1804.

H-D-Pro-L-(4*S*)-Flp-D-Ind-NH$_2$ · TFA (UTS-35):

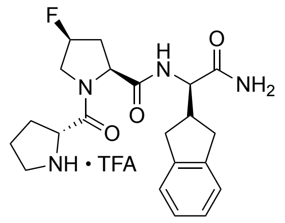

The peptide was synthesized according to the general procedure B for solid phase peptide synthesis.
^{1}H-NMR (400 MHz, D$_2$O) δ 7.26 (q, *J* = 3.9 Hz, 2H), 7.23 – 7.14 (m, 2H), 5.44 (t, *J* = 3.5 Hz, 1H), 5.31 (t, *J* = 3.5 Hz, 1H), 4.71 – 4.59 (m, 2H), 4.46 (d, *J* = 7.6 Hz, 1H), 4.04 (dd, *J* = 23.6, 12.7 Hz, 1H), 3.93 – 3.75 (m, 1H), 3.40 (qt, *J* = 11.4, 7.0 Hz, 2H), 3.09 (ddd, *J* = 15.9, 8.1, 4.6 Hz, 2H), 2.92 (dp, *J* = 14.7, 7.3 Hz, 1H), 2.76 (dt, *J* = 15.9, 6.0 Hz, 2H), 2.51 (ddt, *J* = 13.1, 8.9, 6.3 Hz, 1H), 2.15 (d, *J* = 17.4 Hz, 1H), 2.06 (p, *J* = 6.8 Hz, 2H), 2.02 – 1.93 (m, 1H). **^{13}C-NMR** (101 MHz, D$_2$O) δ 175.6, 172.6, 168.4, 142.4, 142.0, 126.6, 124.6, 124.5, 92.7 (d, *J* = 175.7 Hz), 59.5, 59.3, 56.9, 53.6 (d, *J* = 23.1 Hz), 46.5, 40.5, 36.1 (d, *J* = 20.8 Hz), 35.4, 34.8, 27.7, 23.9. **HR-MS** (ESI) m/z: calc. for $C_{21}H_{28}FN_4O_3$ 403.2140 $(M+H)^+$, found 403.2139.

H-D-Pro-L-Leu-L-(4*S*)-Flp-NH$_2$ · TFA (UTS-36):

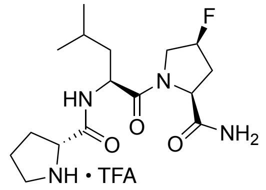

The peptide was synthesized according to the general procedure B for solid phase peptide synthesis.
^{1}H-NMR ^{1}H NMR (400 MHz, D$_2$O) δ 5.55 – 5.26 (m, 1H), 4.63 (t, *J* = 9.1 Hz, 1H), 4.59 – 4.45 (m, 1H), 4.38 (dd, *J* = 8.8, 5.4 Hz, 1H), 4.18 – 4.00 (m, 1H), 3.98 – 3.65 (m, 1H), 3.47 – 3.31 (m, 2H), 2.75 – 2.55 (m, 1H), 2.52 – 2.38 (m, 2H), 2.02 (dq, *J* = 10.1, 3.8 Hz, 3H), 1.78 – 1.52 (m, 3H), 1.04 – 0.84 (m, 6H). **^{13}C-NMR** (101 MHz, D$_2$O) δ 175.6, 173.2, 169.6, 93.3 (d, *J* = 174.7 Hz), 59.6, 58.9, 53.7 (d, *J* = 23.3 Hz), 50.6, 46.4, 38.7, 35.7 (d, *J* = 21.0 Hz), 29.9, 24.5, 23.7, 22.4, 20.1. **HR-MS** (ESI) m/z: calc. for $C_{16}H_{27}FN_4NaO_3$ 365.1959 $(M+Na)^+$, found 365.1960.

H-D-Pro-D-Leu-L-(4*S*)-Flp-NH$_2$ · TFA (UTS-37):

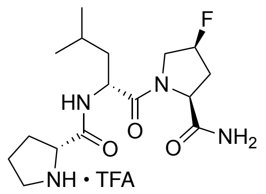

The peptide was synthesized according to the general procedure B for solid phase peptide synthesis.
^{1}H-NMR (400 MHz, D$_2$O) δ 5.45 (dd, *J* = 51.8, 28.3 Hz, 1H), 4.68 – 4.57 (m, 2H), 4.49 – 4.24 (m, 2H), 4.09 – 3.68 (m, 1H), 3.42 (qd, *J* = 10.8, 5.0 Hz, 2H), 2.67 – 2.37 (m, 3H), 2.06 (tdq, *J* = 21.1, 14.3, 7.0 Hz, 3H), 1.81 – 1.60 (m, 2H), 1.60 – 1.42 (m, 1H), 1.05 – 0.80 (m, 6H). **^{13}C-NMR** (101 MHz, D$_2$O) δ 176.0, 173.1, 169.8, 93.2 (d, *J* = 174.0 Hz), 46.5, 38.2, 35.9 (d, *J* = 20.5 Hz), 29.8, 24.4, 23.6, 22.4, 20.5. **HR-MS** (ESI) m/z: calc. for $C_{16}H_{27}FN_4NaO_3$ 365.1959 $(M+Na)^+$, found 365.1960.

H-D-Pro-L-Gln-L-(4*S*)-Flp-NH$_2$ · TFA (UTS-38):

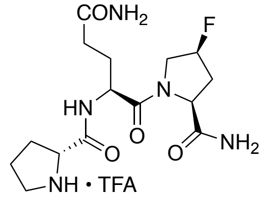

The peptide was synthesized according to the general procedure B for solid phase peptide synthesis.
^{1}H-NMR (400 MHz, D$_2$O) δ 5.45 (dd, *J* = 52.3, 28.5 Hz, 1H), 4.89 – 4.84 (m, 1H), 4.72 – 4.57 (m, 2H), 4.41 (q, *J* = 5.5 Hz, 1H), 4.19 – 3.98 (m, 2H), 3.94 – 3.73 (m, 1H), 3.52 – 3.34 (m, 2H), 2.75 – 2.60 (m, 1H), 2.60 – 2.43 (m, 3H), 2.44 – 2.33 (m, 1H), 2.25 (ddd, *J* = 14.4, 7.4, 4.7 Hz, 1H), 2.17 – 1.87 (m, 4H). **^{13}C-NMR** (101 MHz, D$_2$O) δ 177.6, 175.5, 172.0, 169.6, 93.3 (d, *J* = 174.9 Hz), 59.7, 59.0, 53.9 (d, *J* = 23.3 Hz), 51.2, 46.4, 35.8 (d, *J* = 20.6 Hz), 30.5, 29.7, 26.0, 23.7. **HR-MS** (ESI) m/z: calc. for $C_{15}H_{24}FN_5NaO_4$ 380.1705 $(M+Na)^+$, found 380.1706.

H-D-Pro-D-Gln-L-(4*S*)-Flp-NH$_2$ · TFA (UTS-39):

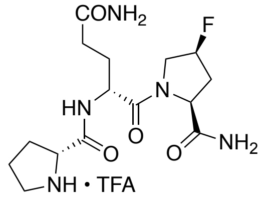

The peptide was synthesized according to the general procedure B for solid phase peptide synthesis.
^{1}H-NMR (500 MHz, D$_2$O) δ 5.56 – 5.33 (m, 1H), 4.71 – 4.61 (m, 2H), 4.49 – 4.43 (m, 1H), 4.24 (dd, *J* = 24.5, 12.9 Hz, 1H), 4.07 – 3.73 (m, 1H), 3.50 – 3.35 (m, 2H), 2.78 – 2.57 (m, 1H), 2.57 – 2.38 (m, 4H), 2.18 – 2.05 (m, 3H), 1.99 (dq, *J* = 23.5, 6.9 Hz, 2H). **^{13}C-NMR** (126 MHz, D$_2$O) δ 177.6, 175.8, 171.7, 169.8, 93.1 (d, *J* = 174.3 Hz), 59.5, 59.3, 53.9 (d, *J* = 23.2 Hz), 51.6, 46.5, 35.9 (d, *J* = 20.6 Hz), 30.5, 29.7, 25.4, 23.6. **HR-MS** (ESI) m/z: calc. for $C_{15}H_{24}FN_5NaO_4$ 380.1705 $(M+Na)^+$, found 380.1705.

H-D-Pro-L-Glu-L-(4*S*)-Flp-NH$_2$ · TFA (UTS-40):

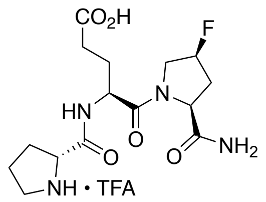

The peptide was synthesized according to the general procedure B for solid phase peptide synthesis.
^{1}H-NMR (500 MHz, D$_2$O) δ 5.56 – 5.33 (m, 1H), 4.74 – 4.63 (m, 2H), 4.46 – 4.38 (m, 1H), 4.20 – 3.73 (m, 2H), 3.51 – 3.34 (m, 2H), 2.75 – 2.37 (m, 5H), 2.32 – 2.22 (m, 1H), 2.17 – 2.05 (m, 3H), 2.05 – 1.84 (m, 1H). **^{13}C-NMR** (126 MHz, D$_2$O) δ 177.0, 175.5, 172.1, 169.6, 93.3 (d, *J* = 174.8 Hz), 59.7, 59.0, 53.8 (d, *J* = 23.1 Hz), 51.1, 46.4, 35.7 (d, *J* = 21.1 Hz), 29.8, 25.5, 23.7. **HR-MS** (ESI) m/z: calc. for $C_{15}H_{24}FN_4O_5$ 359.1725 $(M+H)^+$, found 359.1727.

H-D-Pro-D-Glu-L-(4*S*)-Flp-NH$_2$ · TFA (UTS-41):

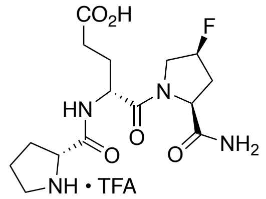

The peptide was synthesized according to the general procedure B for solid phase peptide synthesis.
^{1}H-NMR (400 MHz, D$_2$O) δ 5.58 – 5.32 (m, 1H), 4.73 (dd, *J* = 9.2, 4.6 Hz, 1H), 4.63 (dd, *J* = 8.9, 2.6 Hz, 1H), 4.44 (td, *J* = 8.7, 6.2 Hz, 1H), 4.25 (dd, *J* = 24.4, 12.8 Hz, 1H), 4.03 (ddd, *J* = 37.7, 13.0, 3.8 Hz, 1H), 3.51 – 3.33 (m, 2H), 2.77 – 2.38 (m, 5H), 2.21 – 1.89 (m, 5H). **^{13}C-NMR** (101 MHz, D$_2$O) δ 176.9, 175.9, 171.8, 169.8, 93.2 (d, *J* = 174.0 Hz), 59.5, 59.3, 53.9 (d, *J* = 23.7 Hz), 51.5, 46.5, 35.9 (d, *J* = 21.0 Hz), 29.7, 24.8, 23.6. **HR-MS** (ESI) m/z: calc. for $C_{15}H_{24}FN_4O_5$ 359.1725 $(M+H)^+$, found 359.1726.

H-D-Pro-L-Tyr-L-(4*S*)-Flp-NH$_2$ · TFA (UTS-42):

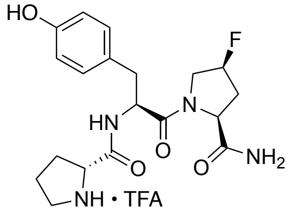

The peptide was synthesized according to the general procedure B for solid phase peptide synthesis.
^{1}H-NMR (400 MHz, D$_2$O) δ 7.29 – 7.12 (m, 2H), 6.88 (ddd, *J* = 8.5, 4.8, 2.4 Hz, 2H), 5.38 (tt, *J* = 52.6, 3.4 Hz, 1H), 4.94 (dd, *J* = 9.6, 6.0 Hz, 1H), 4.65 (dd, *J* = 10.2, 1.9 Hz, 1H), 4.38 – 4.24 (m, 1H), 4.17 – 3.95 (m, 1H), 3.94 – 3.63 (m, 1H), 3.33 (ddt, *J* = 11.7, 8.0, 5.5 Hz, 2H), 3.21 (dd, *J* = 14.1, 5.9 Hz, 1H), 3.08 – 2.87 (m, 1H), 2.67 – 2.43 (m, 1H), 2.42 – 2.15 (m, 1H), 2.15 – 1.90 (m, 1H), 1.89 – 1.72 (m, 1H), 1.66 (dq, *J* = 13.6, 6.6 Hz, 1H). **^{13}C-NMR** (101 MHz, D$_2$O) δ 175.7, 172.1, 169.0, 154.7, 130.8, 127.7, 115.6, 115.5, 93.3 (d, *J* = 174.1 Hz), 59.5, 59.2, 54.0 (d, *J* = 23.3 Hz), 52.8, 46.4, 38.0 (d, *J* = 20.6 Hz), 35.9, 29.9, 23.4. **HR-MS** (ESI) m/z: calc. for $C_{19}H_{25}FN_4NaO_4$ 415.1752 $(M+Na)^+$, found 415.1752.

H-D-Pro-D-Tyr-L-(4*S*)-Flp-NH$_2$ · TFA (UTS-43):

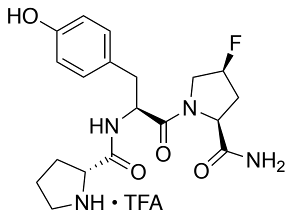

The peptide was synthesized according to the general procedure B for solid phase peptide synthesis.
^{1}H-NMR (400 MHz, D_2O) δ 7.22 – 7.13 (m, 2H), 6.92 – 6.83 (m, 2H), 5.25 – 5.05 (m, 1H), 4.75 – 4.68 (m, 1H), 4.53 (d, *J* = 10.0 Hz, 1H), 4.43 (dd, *J* = 8.8, 6.1 Hz, 1H), 3.99 (dd, *J* = 24.8, 13.0 Hz, 1H), 3.49 – 3.32 (m, 2H), 3.10 (dd, *J* = 13.0, 6.1 Hz, 1H), 2.96 (dd, *J* = 13.1, 10.1 Hz, 1H), 2.79 (ddd, *J* = 38.2, 13.0, 3.9 Hz, 1H), 2.54 – 2.33 (m, 2H), 2.23 – 1.93 (m, 4H). **^{13}C-NMR** (101 MHz, D_2O) δ 175.8, 172.5, 169.6, 154.9, 130.7, 126.9, 115.6, 92.7 (d, *J* = 173.8 Hz), 59.4, 58.9, 54.3, 53.7 (d, *J* = 23.3 Hz), 46.5, 35.7, 35.5, 29.7, 23.6. **HR-MS** (ESI) m/z: calc. for $C_{19}H_{25}FN_4NaO_4$ 415.1752 $(M+Na)^+$, found 415.1752.

H-D-Pro-CyLeu-L-(4*S*)-Flp-NH$_2$ · TFA (UTS-44):

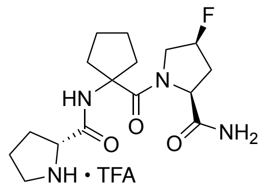

The peptide was synthesized according to the general procedure B for solid phase peptide synthesis.
^{1}H-NMR (400 MHz, D_2O) δ 5.53 – 5.32 (m, 1H), 4.74 – 4.67 (m, 1H), 4.51 – 4.42 (m, 1H), 4.08 (dd, *J* = 23.4, 13.0 Hz, 1H), 3.86 (ddd, *J* = 37.4, 13.0, 3.6 Hz, 1H), 3.51 – 3.35 (m, 2H), 2.57 – 2.36 (m, 4H), 2.16 – 1.97 (m, 5H), 1.93 – 1.61 (m, 5H). **^{13}C-NMR** (101 MHz, D_2O) δ 176.5, 174.0, 169.2, 93.7 (d, *J* = 173.0 Hz), 67.2, 60.6, 59.3, 54.1 (d, *J* = 23.5 Hz), 46.4, 36.7, 34.5 (d, *J* = 21.0 Hz), 34.2, 29.8, 23.6, 23.5, 23.4. **HR-MS** (ESI) m/z: calc. for $C_{16}H_{25}FN_4NaO_3$ 363.1803 $(M+Na)^+$, found 363.1803.

H-D-Pro-D-Ind-L-(4*S*)-Flp-NH$_2$ · TFA (UTS-45):

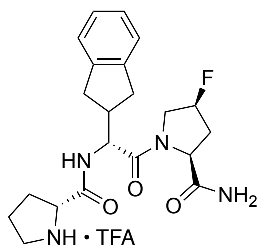

The peptide was synthesized according to the general procedure B for solid phase peptide synthesis.
^{1}H-NMR (400 MHz, D_2O) δ 7.37 – 7.20 (m, 4H), 5.53 – 5.32 (m, 1H), 4.69 – 4.60 (m, 2H), 4.40 (dd, *J* = 8.6, 5.8 Hz, 1H), 4.23 (dd, *J* = 24.8, 13.0 Hz, 1H), 3.91 (ddd, *J* = 37.7, 13.0, 3.9 Hz, 1H), 3.41 (tq, *J* = 11.8, 5.9 Hz, 2H), 3.12 (ddd, *J* = 20.7, 15.5, 7.2 Hz, 2H), 2.99 – 2.85 (m, 2H), 2.75 (dd, *J* = 15.9, 5.8 Hz, 1H), 2.66 – 2.39 (m, 3H), 2.15 – 1.93 (m, 3H). **^{13}C-NMR** (101 MHz, D_2O) δ 175.7, 172.0, 170.0, 142.0, 126.8, 124.7, 124.5, 93.1 (d, *J* = 174.1 Hz), 59.4, 59.2, 55.3, 54.3 (d, *J* = 23.8 Hz), 46.6, 40.4, 36.0 (d, *J* = 20.8 Hz), 35.1, 34.8, 29.8, 23.6. **HR-MS** (ESI) m/z: calc. for $C_{21}H_{28}FN_4O_3$ 403.2140 $(M+H)^+$, found 403.2141.

H-D-Pro-L-Leu-L-Leu-NH$_2$ · TFA (UTS-46):

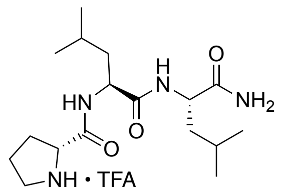

The peptide was synthesized according to the general procedure B for solid phase peptide synthesis.
^{1}H-NMR (400 MHz, D_2O) δ 4.45 – 4.26 (m, 3H), 3.40 (ddt, *J* = 14.3, 11.6, 6.0 Hz, 2H), 2.44 (dd, *J* = 9.9, 7.1 Hz, 1H), 2.04 (d, *J* = 7.3 Hz, 3H), 1.72 – 1.51 (m, 6H), 0.97 – 0.80 (m, 12H). **^{13}C-NMR** (101 MHz, D_2O) δ 177.2, 174.5, 169.7, 59.7, 52.7, 52.1, 46.5, 39.7, 39.6, 29.9, 24.3, 24.3, 23.7, 22.1, 22.0, 20.5, 20.5. **HR-MS** (ESI) m/z: calc. for $C_{17}H_{33}N_4O_3$ 341.2547 $(M+H)^+$, found 341.2549.

H-D-Pro-L-Leu-D-Leu-NH$_2$ · TFA (UTS-47):

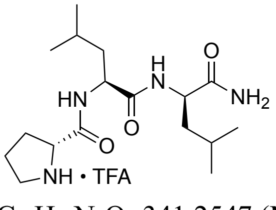

The peptide was synthesized according to the general procedure B for solid phase peptide synthesis.
^{1}H-NMR (400 MHz, D_2O) δ 4.43 – 4.32 (m, 2H), 4.32 – 4.21 (m, 1H), 3.47 – 3.31 (m, 2H), 2.44 (q, *J* = 8.4 Hz, 1H), 2.03 (s, 3H), 1.72 – 1.50 (m, 6H), 0.96 – 0.81 (m, 12H). **^{13}C-NMR** (101 MHz, D_2O) δ 177.6, 174.8, 169.6, 59.6, 52.8, 52.1, 46.5, 39.8, 39.4, 29.8, 24.7, 23.7, 22.3, 21.7, 20.9, 19.9. **HR-MS** (ESI) m/z: calc. for $C_{17}H_{33}N_4O_3$ 341.2547 $(M+H)^+$, found 341.2549.

H-D-Pro-L-Leu-L-Gln-NH$_2$ · TFA (UTS-48):

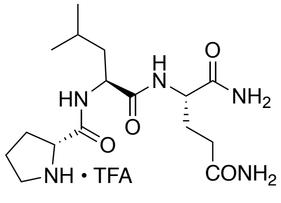

The peptide was synthesized according to the general procedure B for solid phase peptide synthesis.
^{1}H-NMR (400 MHz, D_2O) δ 4.50 – 4.37 (m, 2H), 4.33 (dd, *J* = 9.1, 5.4 Hz, 1H), 3.52 – 3.36 (m, 2H), 2.55 – 2.45 (m, 1H), 2.40 (t, *J* = 7.4 Hz, 2H), 2.20 – 1.96 (m, 5H), 1.75 – 1.58 (m, 3H), 1.03 – 0.86 (m, 6H). **^{13}C-NMR** (101 MHz, D_2O) δ 177.9, 175.7, 174.5, 169.9, 59.8, 52.9, 52.7, 46.5, 39.7, 31.1, 29.9, 26.7, 24.4, 23.8, 22.1, 20.5. **HR-MS** (ESI) m/z: calc. for $C_{16}H_{30}N_5O_4$ 356.2292 $(M+H)^+$, found 356.2291.

H-D-Pro-L-Leu-D-Gln-NH$_2$ · TFA (UTS-49):

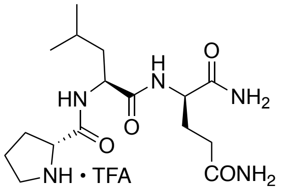

The peptide was synthesized according to the general procedure B for solid phase peptide synthesis.
^{1}H-NMR (400 MHz, D_2O) δ 4.38 (td, *J* = 9.9, 5.5 Hz, 2H), 4.27 (dd, *J* = 9.6, 4.9 Hz, 1H), 3.47 – 3.31 (m, 2H), 2.50 – 2.40 (m, 1H), 2.35 (t, *J* = 7.4 Hz, 2H), 2.14 (ddd, *J* = 14.3, 7.5, 4.9 Hz, 1H), 2.09 – 1.90 (m, 4H), 1.72 – 1.53 (m, 3H), 0.90 (dd, *J* = 19.2, 5.9 Hz, 6H). **^{13}C-NMR** (101 MHz, D_2O) δ 177.7, 175.9, 174.7, 169.8, 59.6, 53.1, 52.7, 46.5, 39.6, 31.1, 29.8, 26.4, 24.4, 23.7, 22.0, 20.5. **HR-MS** (ESI) m/z: calc. for $C_{16}H_{30}N_5O_4$ 356.2292 $(M+H)^+$, found 356.2291.

H-D-Pro-L-Leu-L-Glu-NH$_2$ · TFA (UTS-50):

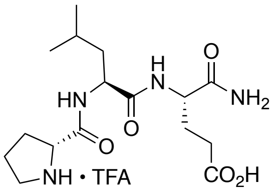

The peptide was synthesized according to the general procedure B for solid phase peptide synthesis.
^{1}H-NMR (400 MHz, D_2O) δ 4.44 – 4.29 (m, 3H), 3.47 – 3.32 (m, 2H), 2.52 – 2.38 (m, 3H), 2.17 – 1.90 (m, 5H), 1.69 – 1.55 (m, 3H), 0.89 (dd, *J* = 19.5, 6.0 Hz, 6H). **^{13}C-NMR** (101 MHz, D_2O) δ 177.5, 175.7, 174.5, 169.8, 59.7, 52.8, 52.6, 46.5, 39.5, 30.2, 29.8, 26.1, 24.3, 23.7, 22.0, 20.4. **HR-MS** (ESI) m/z: calc. for $C_{16}H_{29}N_4O_5$ 357.2132 $(M+H)^+$, found 357.2131.

H-D-Pro-L-Leu-D-Glu-NH$_2$ · TFA (UTS-51):

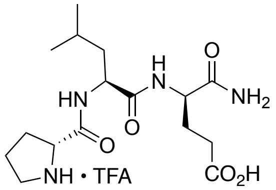

The peptide was synthesized according to the general procedure B for solid phase peptide synthesis.
^{1}H-NMR (400 MHz, D_2O) δ 4.48 – 4.30 (m, 3H), 3.43 (qt, *J* = 11.7, 7.3 Hz, 2H), 2.50 (dd, *J* = 7.9, 6.5 Hz, 3H), 2.26 – 2.14 (m, 1H), 2.14 – 1.93 (m, 4H), 1.75 – 1.57 (m, 3H), 0.94 (dd, *J* = 19.4, 5.8 Hz, 6H). **^{13}C-NMR** (101 MHz, D_2O) δ 177.1, 176.0, 174.7, 169.8, 59.7, 52.9, 52.8, 46.5, 39.7, 30.2, 29.9, 25.8, 24.4, 23.8, 21.9, 20.7. **HR-MS** (ESI) m/z: calc. for $C_{16}H_{29}N_4O_5$ 357.2132 $(M+H)^+$, found 357.2133.

H-D-Pro-L-Leu-L-Tyr-NH$_2$ · TFA (UTS-52):

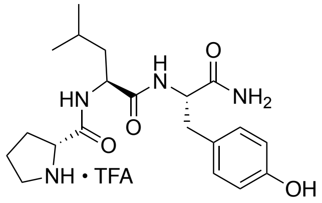

The peptide was synthesized according to the general procedure B for solid phase peptide synthesis.
^{1}H-NMR (400 MHz, D$_2$O) δ 7.22 – 7.09 (m, 2H), 6.91 – 6.78 (m, 2H), 4.59 (dd, *J* = 9.3, 6.2 Hz, 1H), 4.39 (dd, *J* = 8.7, 6.0 Hz, 1H), 4.26 (dd, *J* = 9.7, 5.1 Hz, 1H), 3.52 – 3.35 (m, 2H), 3.14 (dd, *J* = 14.0, 6.2 Hz, 1H), 2.93 (dd, *J* = 14.0, 9.3 Hz, 1H), 2.54 – 2.36 (m, 1H), 2.16 – 1.92 (m, 3H), 1.48 (tt, *J* = 12.6, 5.2 Hz, 2H), 1.43 – 1.30 (m, 1H), 0.86 (dd, *J* = 22.6, 6.1 Hz, 6H). **^{13}C-NMR** (101 MHz, D$_2$O) δ 175.6, 174.0, 169.7, 154.3, 130.6, 128.4, 115.4, 59.7, 54.5, 52.8, 46.6, 39.7, 36.0, 29.9, 24.3, 23.7, 21.9, 20.5. **HR-MS** (ESI) m/z: calc. for $C_{20}H_{31}N_4O_4$ 391.2340 (M+H)$^+$, found 391.2340.

H-D-Pro-L-Leu-D-Tyr-NH$_2$ · TFA (UTS-53):

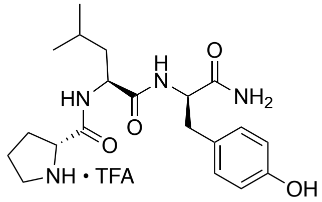

The peptide was synthesized according to the general procedure B for solid phase peptide synthesis.
^{1}H-NMR (400 MHz, D$_2$O) δ 7.20 – 7.13 (m, 2H), 6.88 – 6.81 (m, 2H), 4.61 (dd, *J* = 9.4, 5.8 Hz, 1H), 4.32 (ddd, *J* = 20.0, 8.9, 5.7 Hz, 2H), 3.46 – 3.35 (m, 2H), 3.13 (dd, *J* = 14.0, 5.8 Hz, 1H), 2.93 (dd, *J* = 14.0, 9.4 Hz, 1H), 2.38 (ddt, *J* = 13.4, 8.7, 6.6 Hz, 1H), 2.11 – 1.93 (m, 2H), 1.93 – 1.81 (m, 1H), 1.57 – 1.40 (m, 3H), 0.88 (dd, *J* = 18.8, 6.1 Hz, 6H). **^{13}C-NMR** (101 MHz, D$_2$O) δ 175.5, 173.8, 154.3, 130.6, 128.2, 115.3, 59.4, 54.3, 52.7, 46.5, 39.7, 36.2, 29.8, 24.2, 23.7, 21.8, 20.9. **HR-MS** (ESI) m/z: calc. for $C_{20}H_{31}N_4O_4$ 391.2340 (M+H)$^+$, found 391.2341.

H-D-Pro-L-Leu-CyLeu-NH$_2$ · TFA (UTS-54):

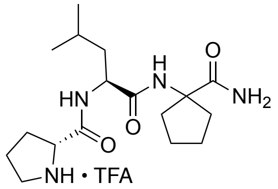

The peptide was synthesized according to the general procedure B for solid phase peptide synthesis.
^{1}H-NMR (400 MHz, D$_2$O) δ 4.46 – 4.31 (m, 2H), 3.41 (ddt, *J* = 18.4, 11.4, 6.7 Hz, 2H), 2.57 – 2.38 (m, 1H), 2.23 (dd, *J* = 14.5, 7.1 Hz, 1H), 2.07 (d, *J* = 6.0 Hz, 4H), 1.95 (t, *J* = 12.7 Hz, 2H), 1.84 – 1.70 (m, 4H), 1.61 (qd, *J* = 10.8, 5.4 Hz, 3H), 0.93 (dd, *J* = 19.0, 5.3 Hz, 6H). **^{13}C-NMR** (101 MHz, D$_2$O) δ 179.3, 174.3, 169.8, 66.9, 59.6, 52.7 46.5, 39.6, 37.4, 35.4, 29.9, 24.4, 23.8, 23.8, 21.8, 20.8. **HR-MS** (ESI) m/z: calc. for $C_{17}H_{31}N_4O_3$ 339.2391 (M+H)$^+$, found 339.2391.

H-D-Pro-L-Leu-Abz-NH$_2$ · TFA (UTS-55):

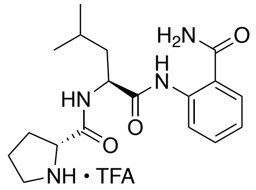

The peptide was synthesized according to the general procedure C for solid phase peptide synthesis.
^{1}H-NMR (400 MHz, D$_2$O) δ 7.87 – 7.18 (m, 4H), 4.57 – 4.20 (m, 2H), 3.56 – 3.14 (m, 2H), 3.02 (s, 2H), 2.63 – 2.42 (m, 1H), 2.11 (d, *J* = 5.4 Hz, 2H), 1.82 – 1.66 (m, 2H), 0.99 (ddd, *J* = 19.0, 17.7, 6.2 Hz, 6H). **^{13}C-NMR** (101 MHz, D$_2$O) δ 173.1, 172.9, 170.0, 134.4, 132.4, 128.6, 126.1, 124.2, 59.8, 53.4, 46.6, 39.5, 29.9, 24.5, 23.8, 22.2, 20.4. **HR-MS** (ESI) m/z: calc. for $C_{18}H_{27}N_4O_3$ 347.2078 (M+H)$^+$, found 347.2080.

H-D-Pro-L-Leu-D-Ind-NH$_2$ · TFA (UTS-56):

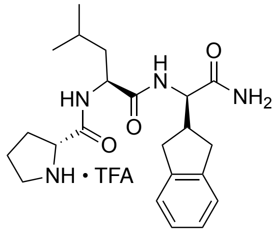

The peptide was synthesized according to the general procedure B for solid phase peptide synthesis.
^{1}H-NMR (400 MHz, D$_2$O) δ 7.26 (d, *J* = 5.0 Hz, 2H), 7.23 – 7.17 (m, 2H), 4.42 (d, *J* = 7.5 Hz, 1H), 4.39 – 4.29 (m, 2H), 3.46 – 3.31 (m, 2H), 3.17 – 3.06 (m, 2H), 2.95 (h, *J* = 7.4 Hz, 1H), 2.77 (dt, *J* = 15.9, 6.3 Hz, 2H), 2.49 – 2.36 (m, 1H), 2.08 – 1.95 (m, 3H), 1.54 (ddt, *J* = 18.5, 12.7, 5.9 Hz, 2H), 1.36 – 1.24 (m, 1H), 0.83 (dd, *J* = 21.4, 6.3 Hz, 6H). **^{13}C-NMR** (101 MHz, D$_2$O) δ 175.8, 174.6, 169.5,

142.3, 142.0, 126.8, 126.7, 124.5, 59.6, 57.2, 52.6, 46.4, 40.1, 39.7, 35.5, 34.9, 29.9, 24.3, 23.7, 21.9, 20.5. **HR-MS** (ESI) m/z: calc. for $C_{22}H_{33}N_4O_3$ 401.2547 $(M+H)^+$, found 401.2547.

H-D-Pro-D-Leu-L-Leu-NH₂ · TFA (UTS-57):

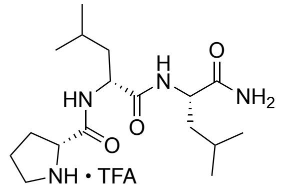

The peptide was synthesized according to the general procedure B for solid phase peptide synthesis.
^{1}H-NMR (400 MHz, D_2O) δ 4.41 – 4.31 (m, 2H), 4.31 – 4.23 (m, 1H), 3.37 (tq, *J* = 11.7, 6.0 Hz, 2H), 2.49 – 2.36 (m, 1H), 2.00 (tt, *J* = 15.2, 7.7 Hz, 3H), 1.72 – 1.50 (m, 6H), 0.98 – 0.79 (m, 12H). **^{13}C-NMR** (101 MHz, D_2O) δ 177.6, 174.7, 169.6, 59.4, 53.1, 52.0, 46.4, 39.5, 29.8, 24.4, 23.6, 22.3, 21.6, 21.2, 19.8. **HR-MS** (ESI) m/z: calc. for $C_{17}H_{33}N_4O_3$ 341.2547 $(M+H)^+$, found 341.2547.

H-D-Pro-L-Gln-L-Leu-NH₂ · TFA (UTS-58):

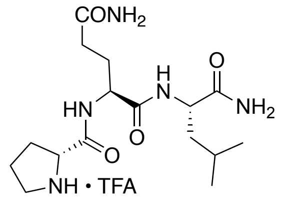

The peptide was synthesized according to the general procedure B for solid phase peptide synthesis.
^{1}H-NMR (500 MHz, D_2O) δ 4.41 (ddd, *J* = 11.9, 8.2, 5.7 Hz, 2H), 4.35 (dd, *J* = 9.9, 4.9 Hz, 1H), 3.43 (tt, *J* = 11.5, 5.5 Hz, 2H), 2.49 (d, *J* = 8.3 Hz, 1H), 2.39 (t, *J* = 7.4 Hz, 2H), 2.22 – 1.97 (m, 5H), 1.66 (tdd, *J* = 25.8, 12.0, 4.7 Hz, 3H), 0.93 (dd, *J* = 25.5, 6.1 Hz, 6H). **^{13}C-NMR** (126 MHz, D_2O) δ 177.7, 177.3, 173.0, 169.7, 59.8, 53.3, 52.2, 46.5, 39.7, 30.9, 29.7, 26.8, 24.3, 23.7, 22.1, 20.6. **HR-MS** (ESI) m/z: calc. for $C_{16}H_{30}N_5O_4$ 356.2292 $(M+H)^+$, found 356.2293.

H-D-Pro-D-Gln-L-Leu-NH₂ · TFA (UTS-59):

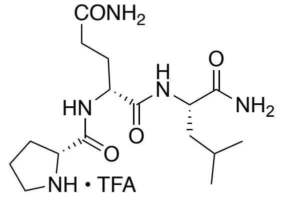

The peptide was synthesized according to the general procedure B for solid phase peptide synthesis.
^{1}H-NMR (500 MHz, D_2O) δ 4.42 (ddd, *J* = 18.3, 8.5, 6.1 Hz, 2H), 4.31 (dd, *J* = 10.3, 3.9 Hz, 1H), 3.49 – 3.35 (m, 2H), 2.53 – 2.43 (m, 1H), 2.39 (t, *J* = 7.5 Hz, 2H), 2.16 – 1.98 (m, 5H), 1.74 – 1.58 (m, 3H), 0.99 – 0.87 (m, 6H). **^{13}C-NMR** (126 MHz, D_2O) δ 177.5, 177.4, 173.2, 169.7, 59.5, 53.7, 52.2, 46.5, 39.6, 30.9, 29.7, 26.7, 24.4, 23.7, 22.2, 20.2. **HR-MS** (ESI) m/z: calc. for $C_{16}H_{30}N_5O_4$ 356.2292 $(M+H)^+$, found 356.2292.

H-D-Pro-L-Glu-L-Leu-NH₂ · TFA (UTS-60):

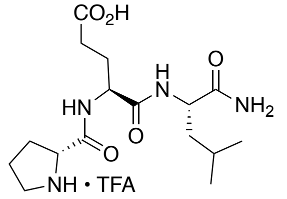

The peptide was synthesized according to the general procedure B for solid phase peptide synthesis.
^{1}H-NMR ^{1}H NMR (500 MHz, D_2O) δ 4.43 (ddd, *J* = 8.4, 5.9, 3.7 Hz, 2H), 4.35 (dd, *J* = 10.0, 4.9 Hz, 1H), 3.50 – 3.37 (m, 2H), 2.54 – 2.45 (m, 3H), 2.17 (dtd, *J* = 14.3, 7.6, 5.5 Hz, 1H), 2.13 – 2.06 (m, 3H), 2.06 – 1.98 (m, 1H), 1.74 – 1.57 (m, 3H), 0.92 (dd, *J* = 25.5, 6.3 Hz, 6H). **^{13}C-NMR** (126 MHz, D_2O) δ 177.3, 177.2, 173.1, 169.7, 59.8, 53.2, 52.3, 46.5, 39.7, 30.1, 29.8, 26.3, 24.3, 23.7, 22.1, 20.6. **HR-MS** (ESI) m/z: calc. for $C_{16}H_{29}N_4O_5$ 357.2132 $(M+H)^+$, found 357.2133.

H-D-Pro-D-Glu-L-Leu-NH₂ · TFA (UTS-61):

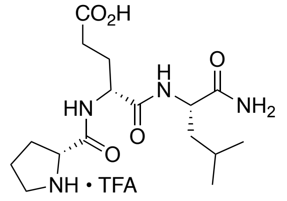

The peptide was synthesized according to the general procedure B for solid phase peptide synthesis.
^{1}H-NMR (400 MHz, D_2O) δ 4.43 (ddd, *J* = 8.2, 6.3, 4.0 Hz, 2H), 4.35 – 4.26 (m, 1H), 3.51 – 3.33 (m, 2H), 2.47 (q, *J* = 6.4 Hz, 3H), 2.18 – 1.96 (m, 5H), 1.75 – 1.56 (m, 3H), 1.01 – 0.83 (m, 6H). **^{13}C-NMR** (101 MHz, D_2O) δ 177.5, 177.0,

173.3, 169.7, 59.5, 53.6, 52.2, 46.5, 39.6, 30.0, 29.7, 26.1, 24.4, 23.7, 22.3, 20.1. **HR-MS** (ESI) m/z: calc. for $C_{16}H_{29}N_4O_5$ 357.2132 $(M+H)^+$, found 357.2133.

H-D-Pro-L-Tyr-L-Leu-NH₂ · TFA (UTS-62):

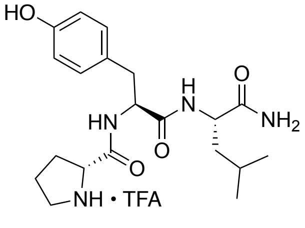

The peptide was synthesized according to the general procedure B for solid phase peptide synthesis.
¹H-NMR (400 MHz, D_2O) δ 7.23 – 7.10 (m, 2H), 6.91 – 6.80 (m, 2H), 4.76 – 4.70 (m, 1H), 4.39 – 4.27 (m, 2H), 3.42 – 3.27 (m, 2H), 3.16 (dd, *J* = 14.0, 6.4 Hz, 1H), 2.97 – 2.84 (m, 1H), 2.28 (ddt, *J* = 13.7, 8.6, 7.1 Hz, 1H), 2.05 – 1.92 (m, 1H), 1.80 (dp, *J* = 14.2, 7.2 Hz, 1H), 1.72 – 1.62 (m, 2H), 1.62 – 1.51 (m, 2H), 0.90 (dd, *J* = 21.6, 5.7 Hz, 6H). **¹³C-NMR** (101 MHz, D_2O) δ 177.0, 172.8, 169.4, 154.5, 130.6, 127.9, 115.5, 59.6, 54.9, 52.2, 46.4, 39.7, 36.4, 29.9, 24.2, 23.5, 22.1, 20.6. **HR-MS** (ESI) m/z: calc. for $C_{17}H_{31}N_4O_3$ 391.2340 $(M+H)^+$, found 391.2341.

H-D-Pro-D-Tyr-L-Leu-NH₂ · TFA (UTS-63):

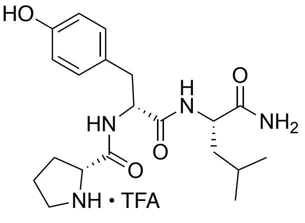

The peptide was synthesized according to the general procedure B for solid phase peptide synthesis.
¹H-NMR (400 MHz, D_2O) δ 7.20 – 7.10 (m, 2H), 6.92 – 6.81 (m, 2H), 4.50 (dd, *J* = 11.3, 5.7 Hz, 1H), 4.43 (dd, *J* = 8.8, 5.9 Hz, 1H), 4.03 – 3.94 (m, 1H), 3.49 – 3.34 (m, 2H), 3.14 (dd, *J* = 13.1, 5.7 Hz, 1H), 2.86 (dd, *J* = 13.2, 11.3 Hz, 1H), 2.53 – 2.40 (m, 1H), 2.15 – 1.94 (m, 3H), 1.46 – 1.31 (m, 2H), 0.75 (d, *J* = 6.3 Hz, 3H), 0.64 (d, *J* = 5.8 Hz, 3H). **¹³C-NMR** (101 MHz, D_2O) δ 177.6, 173.3, 169.5, 154.7, 130.4, 127.1, 115.6, 59.4, 56.6, 52.0, 46.5, 39.4, 35.7, 29.8, 23.6, 23.4, 22.3, 20.2. **HR-MS** (ESI) m/z: calc. for $C_{17}H_{31}N_4O_3$ 391.2340 $(M+H)^+$, found 391.2340.

H-D-Pro-CyLeu-L-Leu-NH₂ · TFA (UTS-64):

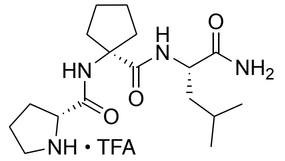

The peptide was synthesized according to the general procedure B for solid phase peptide synthesis.
¹H-NMR (400 MHz, D_2O) δ 4.39 (ddd, *J* = 15.2, 9.7, 5.0 Hz, 2H), 3.49 – 3.34 (m, 2H), 2.55 – 2.39 (m, 1H), 2.22 (dt, *J* = 14.4, 8.6 Hz, 1H), 2.17 – 2.00 (m, 4H), 1.93 (t, *J* = 15.0 Hz, 2H), 1.85 – 1.66 (m, 4H), 1.62 (td, *J* = 9.3, 4.3 Hz, 2H), 0.91 (dd, *J* = 22.6, 5.7 Hz, 6H). **¹³C-NMR** (101 MHz, D_2O) δ 177.8, 176.0, 169.5, 67.3, 59.6, 52.2, 46.6, 39.3, 36.4, 36.2, 29.7, 24.4, 23.8, 23.6, 23.6, 22.2, 20.3. **HR-MS** (ESI) m/z: calc. for $C_{17}H_{31}N_4O_3$ 339.2391 $(M+H)^+$, found 339.2390.

H-D-Pro-Abz-L-Leu-NH₂ · TFA (UTS-65):

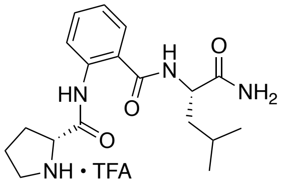

The peptide was synthesized according to the general procedure C for solid phase peptide synthesis.
¹H-NMR (400 MHz, D_2O) δ 7.63 (ddd, *J* = 7.6, 6.4, 1.7 Hz, 2H), 7.51 (dd, *J* = 8.4, 1.2 Hz, 1H), 7.44 (td, *J* = 7.6, 1.2 Hz, 1H), 4.60 (dd, *J* = 8.8, 6.3 Hz, 1H), 4.48 (dd, *J* = 10.4, 4.7 Hz, 1H), 3.55 – 3.37 (m, 2H), 2.63 – 2.46 (m, 1H), 2.24 (tt, *J* = 13.0, 6.1 Hz, 1H), 2.12 (qd, *J* = 13.6, 6.7 Hz, 2H), 1.91 – 1.63 (m, 3H), 1.11 – 0.80 (m, 6H). **¹³C-NMR** (101 MHz, D_2O) δ 177.6, 170.5, 168.5, 133.0, 132.2, 129.1, 128.5, 127.1, 125.4, 60.0, 52.4, 46.6, 39.8, 29.4, 24.6, 23.7, 22.3, 20.4. **HR-MS** (ESI) m/z: calc. for $C_{18}H_{27}N_4O_3$ 347.2078 $(M+H)^+$, found 347.2078.

H-D-Pro-D-Ind-L-Leu-NH₂ · TFA (UTS-66):

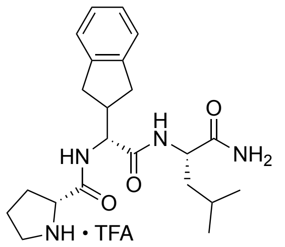

The peptide was synthesized according to the general procedure B for solid phase peptide synthesis.
^{1}H-NMR (400 MHz, D_2O) δ 7.27 (q, *J* = 4.2 Hz, 2H), 7.23 – 7.15 (m, 2H), 4.38 (dd, *J* = 8.6, 5.8 Hz, 1H), 4.33 (d, *J* = 8.2 Hz, 1H), 4.25 (dd, *J* = 10.7, 4.2 Hz, 1H), 3.37 (tq, *J* = 12.0, 6.0 Hz, 2H), 3.19 – 3.06 (m, 1H), 3.01 (dd, *J* = 14.6, 6.5 Hz, 1H), 2.83 (td, *J* = 15.5, 6.7 Hz, 3H), 2.43 (q, *J* = 6.0 Hz, 1H), 2.09 – 1.91 (m, 3H), 1.72 – 1.53 (m, 3H), 0.91 (dd, *J* = 16.8, 6.0 Hz, 6H). **^{13}C-NMR** (101 MHz, D_2O) δ 177.4, 173.3, 169.8, 141.9, 141.8, 126.7, 126.7, 124.6, 124.5, 59.4, 58.1, 52.2, 46.5, 40.5, 39.4, 35.5, 34.9, 29.8, 24.4, 23.6, 22.3, 20.0. **HR-MS** (ESI) m/z: calc. for $C_{22}H_{33}N_4O_3$ 401.2547 $(M+H)^+$, found 401.2545.

H-D-Pro-D-Leu-D-Leu-NH₂ · TFA (UTS-67):

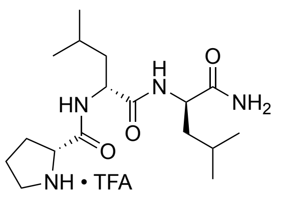

The peptide was synthesized according to the general procedure B for solid phase peptide synthesis.
^{1}H-NMR (400 MHz, D_2O) δ 4.45 – 4.32 (m, 3H), 3.41 (ddt, *J* = 18.6, 11.5, 6.0 Hz, 2H), 2.53 – 2.38 (m, 1H), 2.14 – 1.96 (m, 3H), 1.73 – 1.55 (m, 6H), 1.00 – 0.85 (m, 12H). **^{13}C-NMR** (101 MHz, D_2O) δ 177.1, 174.1, 169.5, 59.5, 52.8, 52.0, 46.5, 39.8, 39.4, 29.8, 24.3, 24.3, 23.7, 22.1, 21.9, 20.9, 20.6. **HR-MS** (ESI) m/z: calc. for $C_{17}H_{33}N_4O_3$ 341.2547 $(M+H)^+$, found 341.2547.

H-D-Pro-D-Leu-L-Gln-NH₂ · TFA (UTS-68):

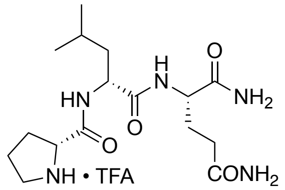

The peptide was synthesized according to the general procedure B for solid phase peptide synthesis.
^{1}H-NMR (400 MHz, D_2O) δ 4.39 (d, *J* = 5.6 Hz, 2H), 4.30 (dd, *J* = 9.6, 4.8 Hz, 1H), 3.40 (dt, *J* = 12.3, 6.3 Hz, 2H), 2.46 (d, *J* = 8.5 Hz, 1H), 2.38 (t, *J* = 7.6 Hz, 2H), 2.18 (dt, *J* = 12.8, 6.4 Hz, 1H), 2.12 – 1.91 (m, 4H), 1.65 (d, *J* = 18.1 Hz, 3H), 0.93 (dd, *J* = 16.2, 5.0 Hz, 6H). **^{13}C-NMR** (101 MHz, D_2O) δ 177.7, 175.9, 174.6, 169.7, 59.5, 53.1, 53.0, 46.5, 39.4, 31.2, 29.8, 26.6, 24.3, 23.7, 21.8, 21.0. **HR-MS** (ESI) m/z: calc. for $C_{16}H_{30}N_5O_4$ 356.2292 $(M+H)^+$, found 356.2294.

H-D-Pro-D-Leu-D-Gln-NH₂ · TFA (UTS-69):

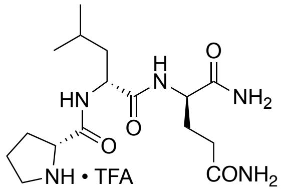

The peptide was synthesized according to the general procedure B for solid phase peptide synthesis.
^{1}H-NMR (400 MHz, D_2O) δ 4.38 (ddd, *J* = 12.6, 8.8, 5.8 Hz, 2H), 4.32 (dd, *J* = 9.0, 5.5 Hz, 1H), 3.49 – 3.32 (m, 2H), 2.53 – 2.43 (m, 1H), 2.38 (t, *J* = 7.5 Hz, 2H), 2.18 – 1.92 (m, 5H), 1.73 – 1.53 (m, 3H), 1.01 – 0.82 (m, 6H). **^{13}C-NMR** (101 MHz, D_2O) δ 177.9, 175.5, 174.3, 169.7, 59.5, 52.9, 52.74, 46.5, 39.5, 31.0, 29.8, 26.8, 24.3, 23.7, 22.0, 20.8. **HR-MS** (ESI) m/z: calc. for $C_{16}H_{30}N_5O_4$ 356.2292 $(M+H)^+$, found 356.2293.

H-D-Pro-D-Leu-L-Glu-NH₂ · TFA (UTS-70):

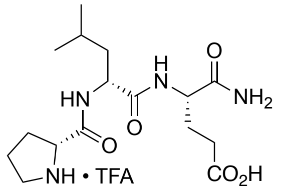

The peptide was synthesized according to the general procedure B for solid phase peptide synthesis.
^{1}H-NMR (400 MHz, D_2O) δ 4.45 – 4.31 (m, 3H), 3.50 – 3.34 (m, 2H), 2.55 – 2.40 (m, 3H), 2.22 (dtd, *J* = 12.9, 7.8, 4.8 Hz, 1H), 2.14 – 1.91 (m, 4H), 1.72 – 1.57 (m, 3H), 1.02 – 0.88 (m, 6H). **^{13}C-NMR** (101 MHz, D_2O) δ 177.3, 176.0, 174.6, 169.6, 59.4, 53.1, 52.8, 46.5, 39.5, 30.3, 29.8, 26.0, 24.3, 23.6, 21.7, 21.1. **HR-MS** (ESI) m/z: calc. for $C_{16}H_{29}N_4O_5$ 357.2132 $(M+H)^+$, found 357.2131.

H-D-Pro-D-Leu-D-Glu-NH_2 · TFA (UTS-71):

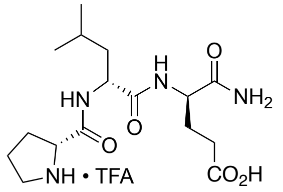

The peptide was synthesized according to the general procedure B for solid phase peptide synthesis.
^{1}H-NMR (500 MHz, D_2O) δ 4.40 (ddt, *J* = 13.4, 9.1, 4.9 Hz, 3H), 3.50 – 3.35 (m, 2H), 2.47 (td, *J* = 7.0, 2.9 Hz, 3H), 2.20 – 1.93 (m, 5H), 1.72 – 1.58 (m, 3H), 0.94 (dd, *J* = 19.9, 5.8 Hz, 6H). **^{13}C-NMR** (126 MHz, D_2O) δ 177.5, 175.6, 174.3, 169.5, 59.5, 52.9, 52.6, 46.5, 39.4, 30.2, 29.8, 26.3, 24.3, 23.7, 21.9, 20.8. **HR-MS** (ESI) m/z: calc. for $C_{16}H_{29}N_4O_5$ 357.2132 $(M+H)^+$, found 357.2131.

H-D-Pro-D-Leu-L-Tyr-NH_2 · TFA (UTS-72):

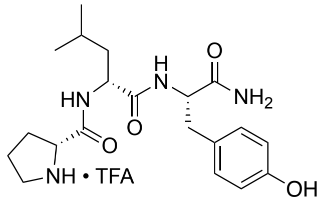

The peptide was synthesized according to the general procedure B for solid phase peptide synthesis.
^{1}H-NMR (500 MHz, D_2O) δ 7.21 – 7.13 (m, 2H), 6.90 – 6.81 (m, 2H), 4.70 (dd, *J* = 11.5, 4.6 Hz, 1H), 4.35 (dd, *J* = 8.6, 6.2 Hz, 1H), 4.16 (t, *J* = 7.7 Hz, 1H), 3.45 – 3.30 (m, 3H), 2.80 (dd, *J* = 14.4, 11.5 Hz, 1H), 2.40 (ddd, *J* = 13.1, 8.6, 6.3 Hz, 1H), 2.04 (dt, *J* = 13.7, 6.8 Hz, 1H), 2.00 – 1.88 (m, 2H), 1.28 (dt, *J* = 13.6, 7.8 Hz, 1H), 1.11 (dt, *J* = 13.8, 7.1 Hz, 1H), 1.00 (hept, *J* = 6.6 Hz, 1H), 0.74 (t, *J* = 6.4 Hz, 6H). **^{13}C-NMR** (126 MHz, D_2O) δ 176.0, 174.5, 169.6, 154.3, 130.2, 128.5, 115.5, 59.3, 54.2, 53.1, 46.5, 39.3, 35.8, 29.8, 23.7, 23.6, 21.6, 21.2. **HR-MS** (ESI) m/z: calc. for $C_{20}H_{31}N_4O_4$ 391.2340 $(M+H)^+$, found 391.2340.

H-D-Pro-D-Leu-D-Tyr-NH_2 · TFA (UTS-73):

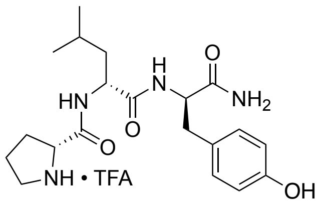

The peptide was synthesized according to the general procedure B for solid phase peptide synthesis.
^{1}H-NMR (400 MHz, D_2O) δ 7.12 (d, *J* = 8.4 Hz, 2H), 6.80 (dd, *J* = 8.9, 2.6 Hz, 2H), 4.57 (dd, *J* = 9.4, 5.8 Hz, 1H), 4.30 (dd, *J* = 8.6, 6.6 Hz, 1H), 4.25 (dd, *J* = 9.0, 5.5 Hz, 1H), 3.37 (tt, *J* = 7.1, 3.9 Hz, 2H), 3.08 (dd, *J* = 14.1, 5.9 Hz, 1H), 2.89 (dd, *J* = 14.0, 9.4 Hz, 1H), 2.41 – 2.28 (m, 1H), 1.97 (dtd, *J* = 26.9, 13.4, 6.8 Hz, 2H), 1.83 (dq, *J* = 14.1, 7.0 Hz, 1H), 1.54 – 1.35 (m, 3H), 0.84 (dd, *J* = 18.9, 5.8 Hz, 6H). **^{13}C-NMR** (101 MHz, D_2O) δ 175.4, 173.8, 169.2, 154.3, 130.5, 128.2, 115.3, 59.3, 54.3, 52.7, 46.5, 39.6, 36.1, 29.8, 24.1, 23.6, 21.7, 20.6. **HR-MS** (ESI) m/z: calc. for $C_{20}H_{31}N_4O_4$ 391.2340 $(M+H)^+$, found 391.2341.

H-D-Pro-D-Leu-CyLeu-NH_2 · TFA (UTS-74):

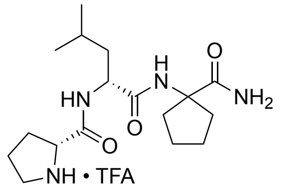

The peptide was synthesized according to the general procedure B for solid phase peptide synthesis.
^{1}H-NMR (500 MHz, D_2O) δ 4.41 (dd, *J* = 8.6, 6.0 Hz, 1H), 4.35 (t, *J* = 7.5 Hz, 1H), 3.48 – 3.42 (m, 1H), 3.39 (dt, *J* = 11.7, 7.0 Hz, 1H), 2.52 – 2.40 (m, 1H), 2.27 – 2.17 (m, 1H), 2.13 – 2.00 (m, 4H), 2.00 – 1.92 (m, 2H), 1.76 (ddt, *J* = 25.2, 10.4, 6.0 Hz, 4H), 1.67 – 1.55 (m, 3H), 0.99 – 0.90 (m, 6H). **^{13}C-NMR** (126 MHz, D_2O) δ 179.2, 174.0, 169.7, 66.9, 59.4, 52.8, 46.5, 39.3, 37.5, 35.3, 29.9, 24.3, 23.8, 23.8, 23.6, 21.7, 21.1. **HR-MS** (ESI) m/z: calc. for $C_{17}H_{31}N_4O_3$ 339.2391 $(M+H)^+$, found 339.2389.

H-D-Pro-D-Leu-Abz-NH$_2$ · TFA (UTS-75):

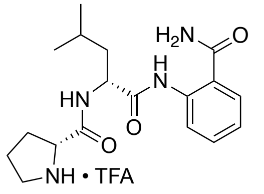

The peptide was synthesized according to the general procedure C for solid phase peptide synthesis.
^{1}H-NMR (500 MHz, D_2O) δ 7.71 (ddd, *J* = 19.4, 8.0, 1.2 Hz, 2H), 7.64 – 7.57 (m, 1H), 7.38 (td, *J* = 7.6, 1.2 Hz, 1H), 4.54 – 4.41 (m, 2H), 3.51 – 3.36 (m, 2H), 3.00 (s, 2H), 2.51 (dt, *J* = 12.5, 7.5 Hz, 1H), 2.09 (dddq, *J* = 41.3, 20.8, 14.1, 7.1 Hz, 3H), 1.75 (d, *J* = 6.3 Hz, 3H), 0.99 (ddd, *J* = 18.3, 14.8, 5.7 Hz, 7H). **^{13}C-NMR** (126 MHz, D_2O) δ 173.1, 172.9, 169.8, 134.2, 132.3, 128.5, 126.7, 126.2, 124.3, 59.6, 53.8, 46.5, 39.4, 29.8, 24.4, 23.7, 22.0, 20.8. **HR-MS** (ESI) m/z: calc. for $C_{18}H_{27}N_4O_3$ 347.2078 $(M+H)^+$, found 347.2077.

H-D-Pro-D-Leu-D-Ind-NH$_2$ · TFA (UTS-76):

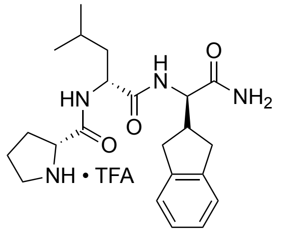

The peptide was synthesized according to the general procedure B for solid phase peptide synthesis.
^{1}H-NMR (400 MHz, D_2O) δ 7.26 (d, *J* = 5.1 Hz, 2H), 7.22 – 7.17 (m, 2H), 4.42 – 4.30 (m, 3H), 3.37 (tt, *J* = 7.0, 3.7 Hz, 2H), 3.06 (td, *J* = 15.6, 7.4 Hz, 2H), 2.86 (dt, *J* = 15.3, 7.6 Hz, 1H), 2.76 (ddd, *J* = 15.7, 11.7, 7.3 Hz, 2H), 2.41 (dt, *J* = 8.5, 6.0 Hz, 1H), 2.11 – 1.90 (m, 3H), 1.66 – 1.51 (m, 3H), 0.89 (dd, *J* = 16.3, 6.2 Hz, 6H). **^{13}C-NMR** (101 MHz, D_2O) δ 184.1, 175.3, 174.1, 142.2, 142.0, 126.7, 124.45, 59.4, 56.5, 52.8, 46.5, 41.0, 39.3, 35.2, 34.9, 29.8, 24.3, 23.7, 21.9, 20.8. **HR-MS** (ESI) m/z: calc. for $C_{22}H_{33}N_4O_3$ 401.2547 $(M+H)^+$, found 401.2548.

H-D-Pro-L-Gln-D-Leu-NH$_2$ · TFA (UTS-77):

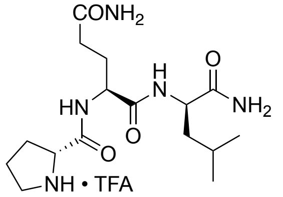

The peptide was synthesized according to the general procedure B for solid phase peptide synthesis.
^{1}H-NMR (500 MHz, D_2O) δ 4.47 – 4.38 (m, 2H), 4.31 (dd, *J* = 10.9, 3.8 Hz, 1H), 3.50 – 3.35 (m, 2H), 2.55 – 2.42 (m, 1H), 2.39 (t, *J* = 7.5 Hz, 2H), 2.18 – 1.98 (m, 5H), 1.74 – 1.58 (m, 3H), 1.00 – 0.86 (m, 6H). **^{13}C-NMR** (126 MHz, D_2O) δ 177.5, 177.4, 173.3, 59.7, 53.5, 52.3, 46.5, 39.6, 30.9, 29.7, 26.8, 24.4, 23.8, 22.2, 20.3. **HR-MS** (ESI) m/z: calc. for $C_{16}H_{30}N_5O_4$ 356.2292 $(M+H)^+$, found 356.2291.

H-D-Pro-D-Gln-D-Leu-NH$_2$ · TFA (UTS-78):

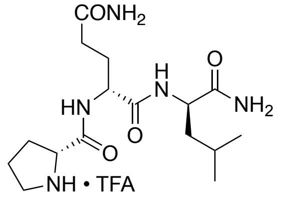

The peptide was synthesized according to the general procedure B for solid phase peptide synthesis.
^{1}H-NMR (500 MHz, D_2O) δ 4.46 – 4.39 (m, 2H), 4.36 (dd, *J* = 10.0, 4.7 Hz, 1H), 3.50 – 3.36 (m, 2H), 2.53 – 2.42 (m, 1H), 2.39 (t, *J* = 7.5 Hz, 2H), 2.17 – 1.98 (m, 5H), 1.73 – 1.55 (m, 3H), 0.92 (dd, *J* = 26.8, 6.0 Hz, 6H). **^{13}C-NMR** ^{13}C NMR (126 MHz, D_2O) δ 177.8, 177.2, 172.8, 169.5, 59.5, 53.1, 52.1, 46.5, 39.8, 30.9, 29.8, 26.7, 24.3, 23.7, 22.1, 20.5. **HR-MS** (ESI) m/z: calc. for $C_{16}H_{30}N_5O_4$ 356.2292 $(M+H)^+$, found 356.2291.

H-D-Pro-L-Glu-D-Leu-NH$_2$ · TFA (UTS-79)

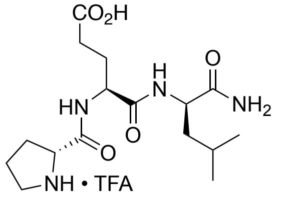

The peptide was synthesized according to the general procedure B for solid phase peptide synthesis.
^{1}H-NMR (400 MHz, D_2O) δ 4.51 – 4.39 (m, 2H), 4.35 – 4.26 (m, 1H), 3.42 (ddt, *J* = 18.4, 11.5, 5.6 Hz, 2H), 2.55 – 2.41 (m, 3H), 2.20 – 1.97 (m, 5H), 1.76 – 1.57 (m, 3H), 0.93 (dd, *J* = 21.8, 5.7 Hz, 6H). **^{13}C-NMR** (101 MHz, D_2O) δ 177.5, 176.9, 173.3, 169.7, 59.7, 53.4, 52.3, 46.5, 39.6, 30.0, 29.8, 26.3, 24.4, 23.8, 22.2, 20.2. **HR-MS** (ESI) m/z: calc. for $C_{16}H_{29}N_4O_5$ 357.2132 $(M+H)^+$, found 357.2132.

H-D-Pro-D-Glu-D-Leu-NH$_2$ · TFA (UTS-80):

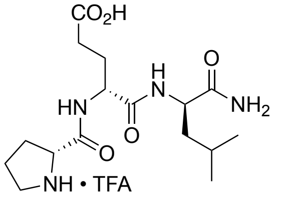

The peptide was synthesized according to the general procedure B for solid phase peptide synthesis.
^{1}H-NMR (400 MHz, D_2O) δ 4.44 (td, *J* = 8.6, 5.9 Hz, 2H), 4.35 (dd, *J* = 10.0, 4.6 Hz, 1H), 3.43 (tdd, *J* = 11.7, 6.4, 4.3 Hz, 2H), 2.57 – 2.40 (m, 3H), 2.20 – 1.95 (m, 5H), 1.76 – 1.53 (m, 3H), 0.92 (dd, *J* = 21.4, 5.9 Hz, 6H). **^{13}C-NMR** (101 MHz, D_2O) δ 177.2, 172.8, 169.5, 59.5, 53.1, 52.1, 46.5, 39.8, 30.0, 29.8, 26.1, 24.3, 23.7, 22.1, 20.5. **HR-MS** (ESI) m/z: calc. for $C_{16}H_{29}N_4O_5$ 357.2132 $(M+H)^+$, found 357.2133.

H-D-Pro-L-Tyr-D-Leu-NH$_2$ · TFA (UTS-81):

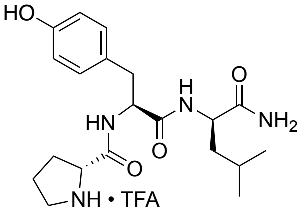

The peptide was synthesized according to the general procedure B for solid phase peptide synthesis.
^{1}H-NMR (400 MHz, D_2O) δ 7.10 (d, *J* = 8.3 Hz, 2H), 6.86 – 6.79 (m, 2H), 4.51 (dd, *J* = 10.2, 6.7 Hz, 1H), 4.34 (dd, *J* = 8.6, 6.3 Hz, 1H), 3.99 (t, *J* = 7.6 Hz, 1H), 3.43 – 3.28 (m, 2H), 3.05 (dd, *J* = 13.3, 6.6 Hz, 1H), 2.88 (dd, *J* = 13.3, 10.2 Hz, 1H), 2.38 (td, *J* = 14.3, 7.1 Hz, 1H), 2.09 – 1.85 (m, 3H), 1.39 (t, *J* = 6.8 Hz, 2H), 0.79 – 0.59 (m, 7H). **^{13}C-NMR** (101 MHz, D_2O) δ 177.6, 173.4, 154.6, 130.4, 127.2, 115.5, 59.5, 56.1, 52.0, 46.4, 39.4, 36.1, 29.7, 23.6, 23.5, 22.3, 20.2. **HR-MS** (ESI) m/z: calc. for $C_{20}H_{31}N_4O_4$ 391.2340 $(M+H)^+$, found 391.2340.

H-D-Pro-D-Tyr-D-Leu-NH$_2$ · TFA (UTS-82):

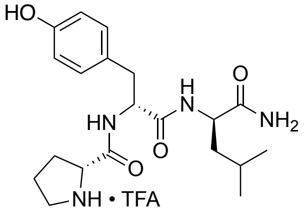

The peptide was synthesized according to the general procedure B for solid phase peptide synthesis.
^{1}H-NMR (400 MHz, D_2O) δ 7.20 – 7.15 (m, 2H), 6.90 – 6.84 (m, 2H), 4.61 (t, *J* = 8.0 Hz, 1H), 4.36 (dd, *J* = 8.7, 5.7 Hz, 1H), 4.27 (dd, *J* = 9.8, 5.0 Hz, 1H), 3.41 (dtd, *J* = 18.7, 11.6, 7.3 Hz, 2H), 3.08 – 2.97 (m, 2H), 2.50 – 2.37 (m, 1H), 2.13 – 1.94 (m, 3H), 1.63 – 1.46 (m, 3H), 0.96 – 0.78 (m, 6H). **^{13}C-NMR** (101 MHz, D_2O) δ 176.7, 172.6, 169.2, 154.6, 130.5, 127.7, 115.6, 59.5, 55.5, 51.9, 46.5, 39.6, 35.9, 29.8, 24.2, 23.6, 22.1, 20.5. **HR-MS** (ESI) m/z: calc. for $C_{20}H_{31}N_4O_4$ 391.2340 $(M+H)^+$, found 391.2338.

H-D-Pro-CyLeu-D-Leu-NH$_2$ · TFA (UTS-83):

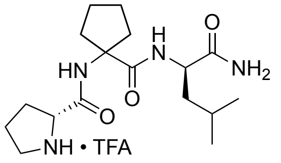

The peptide was synthesized according to the general procedure B for solid phase peptide synthesis.
^{1}H-NMR (400 MHz, D_2O) δ 4.44 – 4.32 (m, 2H), 3.50 – 3.34 (m, 2H), 2.53 – 2.40 (m, 1H), 2.25 – 2.01 (m, 5H), 1.95 (dd, *J* = 13.3, 5.5 Hz, 2H), 1.77 (q, *J* = 4.0 Hz, 4H), 1.70 (dd, *J* = 10.3, 8.4 Hz, 1H), 1.66 – 1.53 (m, 2H), 0.91 (dd, *J* = 18.7, 5.9 Hz, 6H). **^{13}C-NMR** (101 MHz, D_2O) δ 177.5, 175.9, 169.1, 67.3, 59.5, 52.1, 46.6, 39.4, 36.3, 35.8, 29.7, 24.4, 23.7, 23.6, 23.3, 22.2, 20.4. **HR-MS** (ESI) m/z: calc. for $C_{17}H_{33}N_4O_3$ 339.2391 $(M+H)^+$, found 339.2389.

H-D-Pro-Abz-D-Leu-NH$_2$ · TFA (UTS-84):

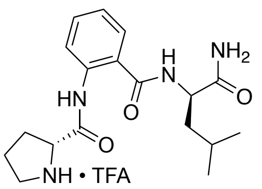

The peptide was synthesized according to the general procedure C for solid phase peptide synthesis.
^{1}H-NMR (400 MHz, D_2O) δ 7.72 – 7.48 (m, 2H), 7.43 – 7.19 (m, 1H), 4.46 (ddd, *J* = 14.3, 10.1, 4.5 Hz, 1H), 3.50 – 3.35 (m, 1H), 2.19 (dq, *J* = 13.3, 6.8 Hz, 1H), 2.14 – 2.00 (m, 1H), 1.85 – 1.71 (m, 2H), 1.66 (dt, *J* = 8.4, 6.6 Hz, 1H), 1.06 – 0.82 (m, 7H). **^{13}C-NMR** (101 MHz, D_2O) δ 177.5, 170.4, 168.4, 133.1, 132.1, 128.8,

128.4, 127.0, 125.5, 60.1, 52.5, 46.5, 39.8, 29.4, 24.4, 23.7, 22.2, 20.5. **HR-MS** (ESI) m/z: calc. for $C_{18}H_{27}N_4O_3$ 347.2078 $(M+H)^+$, found 347.2075.

H-D-Pro-D-Ind-D-Leu-NH$_2$ · TFA (UTS-85):

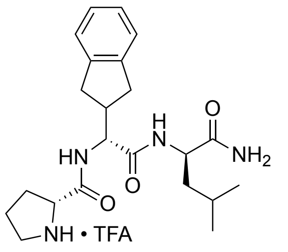

The peptide was synthesized according to the general procedure B for solid phase peptide synthesis.
^{1}H-NMR (400 MHz, D_2O) δ 7.31 – 7.23 (m, 2H), 7.20 (dd, *J* = 5.6, 3.3 Hz, 2H), 4.35 (td, *J* = 9.1, 4.4 Hz, 3H), 3.38 (qt, *J* = 11.7, 6.8 Hz, 2H), 3.10 (dd, *J* = 15.6, 6.9 Hz, 1H), 3.00 (dd, *J* = 14.4, 6.4 Hz, 1H), 2.82 (qd, *J* = 16.9, 7.2 Hz, 3H), 2.43 (q, *J* = 6.5 Hz, 1H), 2.00 (qd, *J* = 15.2, 6.5 Hz, 3H), 1.71 – 1.50 (m, 3H), 0.88 (dd, *J* = 19.9, 5.7 Hz, 6H). **^{13}C-NMR** (101 MHz, D_2O) δ 176.9, 172.7, 169.5, 142.1, 141.9, 126.7, 124.6, 124.5, 59.4, 57.5, 51.6, 46.5, 40.7, 39.7, 35.3, 35.0, 29.9, 24.3, 23.6, 22.0, 20.5. **HR-MS** (ESI) m/z: calc. for $C_{22}H_{33}N_4O_3$ 401.2547 $(M+H)^+$, found 401.2546.

H-D-Pro-L-Gln-L-Gln-NH$_2$ · TFA (UTS-86):

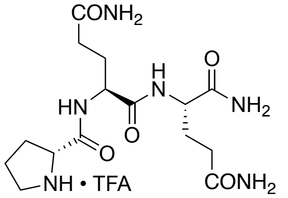

The peptide was synthesized according to the general procedure B for solid phase peptide synthesis.
^{1}H-NMR (400 MHz, D_2O) δ 4.38 (ddd, *J* = 14.1, 8.3, 5.7 Hz, 2H), 4.30 (dd, *J* = 9.0, 5.4 Hz, 1H), 3.48 – 3.32 (m, 2H), 2.51 – 2.40 (m, 1H), 2.36 (td, *J* = 7.4, 3.0 Hz, 4H), 2.18 – 1.91 (m, 7H). **^{13}C-NMR** (101 MHz, D_2O) δ 177.9, 177.6, 175.6, 173.0, 169.8, 59.7, 53.4, 53.0, 46.4, 31.0, 30.9, 29.7, 26.7, 23.7. **HR-MS** (ESI) m/z: calc. for $C_{15}H_{27}N_6O_5$ 371.2037 $(M+H)^+$, found 371.2039.

H-D-Pro-L-Gln-D-Gln-NH$_2$ · TFA (UTS-87):

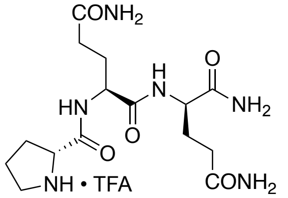

The peptide was synthesized according to the general procedure B for solid phase peptide synthesis.
^{1}H-NMR (400 MHz, D_2O) δ 4.43 (dt, *J* = 8.9, 5.7 Hz, 2H), 4.31 (dd, *J* = 9.2, 5.1 Hz, 1H), 3.43 (qt, *J* = 11.5, 7.1 Hz, 2H), 2.50 (dt, *J* = 8.5, 6.6 Hz, 1H), 2.40 (td, *J* = 7.5, 2.5 Hz, 4H), 2.23 – 1.94 (m, 7H). **^{13}C-NMR** (101 MHz, D_2O) δ 177.8, 177.6, 175.6, 173.2, 169.8, 59.8, 53.5, 53.2, 46.5, 31.2, 31.0, 29.7, 26.7, 26.6, 23.8. **HR-MS** (ESI) m/z: calc. for $C_{15}H_{27}N_6O_5$ 371.2037 $(M+H)^+$, found 371.2038.

H-D-Pro-L-Gln-L-Glu-NH$_2$ · TFA (UTS-88):

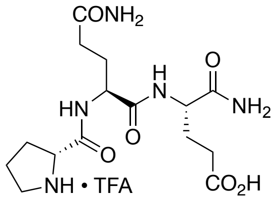

The peptide was synthesized according to the general procedure B for solid phase peptide synthesis.
^{1}H-NMR (400 MHz, D_2O) δ 4.43 – 4.30 (m, 3H), 3.47 – 3.31 (m, 2H), 2.51 – 2.40 (m, 3H), 2.36 (t, *J* = 7.4 Hz, 2H), 2.18 – 1.91 (m, 7H). **^{13}C-NMR** (101 MHz, D_2O) δ 177.6, 177.4, 175.7, 173.0, 169.7, 59.7, 53.4, 52.8, 46.4, 30.9, 30.2, 29.7, 26.6, 26.1, 23.7. **HR-MS** (ESI) m/z: calc. for $C_{15}H_{26}N_5O_6$ 372.1878 $(M+H)^+$, found 372.1877.

H-D-Pro-L-Gln-D-Glu-NH$_2$ · TFA (UTS-89):

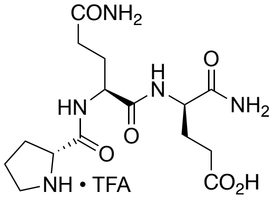

The peptide was synthesized according to the general procedure B for solid phase peptide synthesis.
^{1}H-NMR (400 MHz, D_2O) δ 4.39 (dt, *J* = 9.5, 6.8 Hz, 2H), 4.29 (dd, *J* = 9.5, 5.0 Hz, 1H), 3.39 (dtd, *J* = 18.3, 11.6, 7.0 Hz, 2H), 2.46 (t, *J* = 7.2 Hz, 3H), 2.36 (t, *J* = 7.4 Hz, 2H), 2.18 – 1.92 (m, 7H). **^{13}C-NMR** (101 MHz, D_2O) δ 177.5, 175.9,

173.2, 169.8, 59.7, 53.5, 53.1, 46.4, 30.9, 30.4, 29.6, 26.6, 26.0, 23.7. **HR-MS** (ESI) m/z: calc. for $C_{15}H_{26}N_5O_6$ 372.1878 $(M+H)^+$, found 372.1877.

H-D-Pro-L-Gln-L-Tyr-NH$_2$ · TFA (UTS-90):

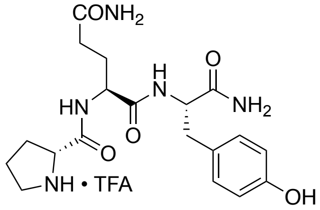

The peptide was synthesized according to the general procedure B for solid phase peptide synthesis.
^{1}H-NMR (500 MHz, D_2O) δ 7.22 – 7.12 (m, 2H), 6.90 – 6.81 (m, 2H), 4.60 (dd, *J* = 9.3, 6.2 Hz, 1H), 4.38 (dd, *J* = 8.5, 6.4 Hz, 1H), 4.28 (dd, *J* = 8.0, 6.3 Hz, 1H), 3.43 (qt, *J* = 11.6, 7.3 Hz, 2H), 3.14 (dd, *J* = 14.0, 6.2 Hz, 1H), 2.93 (dd, *J* = 14.0, 9.4 Hz, 1H), 2.52 – 2.43 (m, 1H), 2.24 – 2.14 (m, 2H), 2.12 – 2.01 (m, 3H), 1.97 – 1.88 (m, 2H). **^{13}C-NMR** (126 MHz, D_2O) δ 177.5, 175.6, 172.6, 169.7, 154.4, 130.5, 128.3, 115.4, 59.7, 54.7, 53.6, 46.5, 36.0, 30.7, 29.7, 26.7, 23.7. **HR-MS** (ESI) m/z: calc. for $C_{19}H_{28}N_5O_5$ 406.2085 $(M+H)^+$, found 406.2085.

H-D-Pro-L-Gln-D-Tyr-NH$_2$ · TFA (UTS-91):

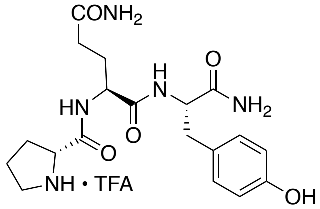

The peptide was synthesized according to the general procedure B for solid phase peptide synthesis.
^{1}H-NMR (400 MHz, D_2O) δ 7.24 – 7.10 (m, 2H), 6.91 – 6.76 (m, 2H), 4.67 (dd, *J* = 11.1, 4.9 Hz, 2H), 4.37 (dd, *J* = 8.3, 6.3 Hz, 1H), 4.27 (t, *J* = 6.8 Hz, 1H), 3.41 (qt, *J* = 11.7, 7.3 Hz, 2H), 3.28 (dd, *J* = 14.3, 4.9 Hz, 1H), 2.83 (dd, *J* = 14.3, 11.1 Hz, 1H), 2.53 – 2.35 (m, 1H), 2.14 – 1.97 (m, 3H), 1.93 – 1.82 (m, 1H), 1.82 – 1.65 (m, 3H). **^{13}C-NMR** (101 MHz, D_2O) δ 177.3, 175.9, 172.9, 169.6, 154.5, 130.4, 128.4, 115.5, 59.6, 54.6, 53.6, 46.5, 36.0, 30.4, 29.7, 26.8, 23.7. **HR-MS** (ESI) m/z: calc. for $C_{19}H_{28}N_5O_5$ 406.2085 $(M+H)^+$, found 406.2084.

H-D-Pro-L-Gln-CyLeu-NH$_2$ · TFA (UTS-92):

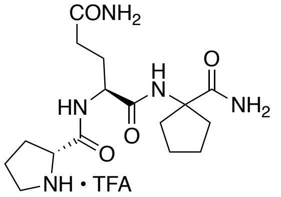

The peptide was synthesized according to the general procedure B for solid phase peptide synthesis.
^{1}H-NMR (400 MHz, D_2O) δ 4.46 – 4.39 (m, 1H), 4.36 (dd, *J* = 8.7, 5.7 Hz, 1H), 3.43 (qt, *J* = 11.6, 7.2 Hz, 2H), 2.56 – 2.44 (m, 1H), 2.39 (t, *J* = 7.5 Hz, 2H), 2.22 (dd, *J* = 14.4, 7.1 Hz, 1H), 2.16 – 2.02 (m, 6H), 2.02 – 1.87 (m, 3H), 1.85 – 1.66 (m, 4H). **^{13}C-NMR** (101 MHz, D_2O) δ 179.2, 177.5, 172.8, 169.8, 67.0, 59.7, 53.4, 46.5, 37.2, 35.8, 30.9, 29.7, 26.6, 23.9, 23.8, 23.8. **HR-MS** (ESI) m/z: calc. for $C_{16}H_{28}N_5O_4$ 354.2136 $(M+H)^+$, found 354.2137.

H-D-Pro-L-Gln-Abz-NH$_2$ · TFA (UTS-93):

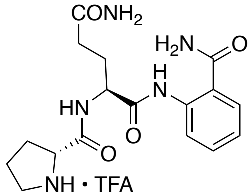

The peptide was synthesized according to the general procedure C for solid phase peptide synthesis.
^{1}H-NMR (400 MHz, D_2O) δ 7.72 (dd, *J* = 8.1, 1.2 Hz, 1H), 7.66 (dd, *J* = 7.8, 1.5 Hz, 1H), 7.57 (td, *J* = 7.9, 1.6 Hz, 1H), 7.34 (td, *J* = 7.6, 1.2 Hz, 1H), 4.52 – 4.41 (m, 2H), 3.49 – 3.32 (m, 2H), 2.55 – 2.38 (m, 3H), 2.26 (dq, *J* = 13.5, 7.2 Hz, 1H), 2.15 – 2.00 (m, 4H). **^{13}C-NMR** (101 MHz, D_2O) δ 177.6, 171.6, 134.3, 132.3, 128.5, 126.4, 126.2, 124.1, 59.8, 54.2, 46.4, 31.1, 29.6, 26.4, 23.8. **HR-MS** (ESI) m/z: calc. for $C_{17}H_{24}N_5O_4$ 362.1823 $(M+H)^+$, found 362.1825.

H-D-Pro-L-Gln-D-Ind-NH_2 · TFA (UTS-94):

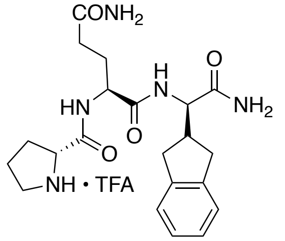

The peptide was synthesized according to the general procedure B for solid phase peptide synthesis.
^{1}H-NMR (400 MHz, D_2O) δ 7.34 – 7.26 (m, 2H), 7.26 – 7.18 (m, 2H), 4.47 (d, *J* = 7.3 Hz, 1H), 4.40 (ddd, *J* = 7.4, 5.9, 4.7 Hz, 2H), 3.50 – 3.34 (m, 2H), 3.16 (dd, *J* = 16.0, 8.1 Hz, 2H), 2.98 (h, *J* = 7.3 Hz, 1H), 2.82 (dt, *J* = 16.0, 6.0 Hz, 2H), 2.54 – 2.41 (m, 1H), 2.28 (dd, *J* = 8.3, 6.9 Hz, 2H), 2.15 – 2.01 (m, 3H), 1.92 – 1.81 (m, 2H). **^{13}C-NMR** (101 MHz, D_2O) δ 177.5, 175.7, 173.1, 169.6, 142.4, 142.0, 126.8, 126.7, 124.5, 124.5, 59.7, 57.3, 53.4, 46.5, 40.2, 35.6, 34.9, 31.0, 29.7, 26.9, 23.8. **HR-MS** (ESI) m/z: calc. for $C_{21}H_{30}N_5O_4$ 416.2292 $(M+H)^+$, found 416.2292

H-D-Pro-D-Gln-L-Gln-NH_2 · TFA (UTS-95):

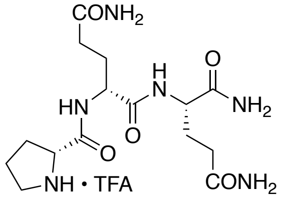

The peptide was synthesized according to the general procedure B for solid phase peptide synthesis.
^{1}H-NMR (400 MHz, D_2O) δ 4.42 (ddd, *J* = 12.2, 8.5, 6.0 Hz, 2H), 4.31 (dd, *J* = 9.2, 5.1 Hz, 1H), 3.42 (qt, *J* = 11.5, 7.1 Hz, 2H), 2.54 – 2.43 (m, 1H), 2.40 (t, *J* = 7.5 Hz, 4H), 2.23 – 1.94 (m, 7H). **^{13}C-NMR** (101 MHz, D_2O) δ 177.7, 177.6, 175.8, 173.1, 169.7, 59.6, 53.6, 53.1, 46.5, 31.2, 30.9, 29.7, 26.7, 26.6, 23.7. **HR-MS** (ESI) m/z: calc. for $C_{15}H_{27}N_6O_5$ 371.2037 $(M+H)^+$, found 371.2037.

H-D-Pro-L-Glu-L-Gln-NH_2 · TFA (UTS-96):

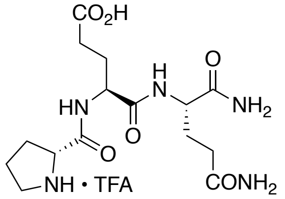

The peptide was synthesized according to the general procedure B for solid phase peptide synthesis.
^{1}H-NMR (400 MHz, D_2O) δ 4.40 (dd, *J* = 8.9, 5.3 Hz, 2H), 4.29 (dd, *J* = 9.0, 5.3 Hz, 1H), 3.39 (dtd, *J* = 18.2, 11.6, 7.1 Hz, 2H), 2.46 (t, *J* = 7.3 Hz, 3H), 2.37 (t, *J* = 7.4 Hz, 2H), 2.20 – 1.90 (m, 7H). **^{13}C-NMR** (101 MHz, D_2O) δ 177.9, 177.0, 175.6, 173.1, 169.9, 59.7, 53.2, 53.0, 46.4, 31.0, 30.0, 29.7, 26.7, 26.1, 23.7. **HR-MS** (ESI) m/z: calc. for $C_{15}H_{26}N_5O_6$ 372.1878 $(M+H)^+$, found 372.1878.

H-D-Pro-D-Glu-L-Gln-NH_2 · TFA (UTS-97):

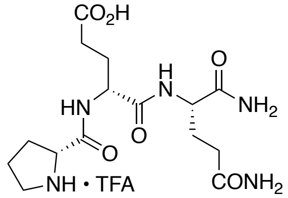

The peptide was synthesized according to the general procedure B for solid phase peptide synthesis.
^{1}H-NMR (400 MHz, D_2O) δ 4.39 (ddd, *J* = 8.7, 6.1, 3.1 Hz, 2H), 4.26 (dd, *J* = 9.4, 5.0 Hz, 1H), 3.39 (qd, *J* = 11.5, 5.9 Hz, 2H), 2.45 (q, *J* = 7.1 Hz, 3H), 2.36 (t, *J* = 7.5 Hz, 2H), 2.17 – 1.91 (m, 7H). **^{13}C-NMR** (101 MHz, D_2O) δ 177.7, 177.1, 175.8, 173.2, 169.7, 59.5, 53.5, 53.0, 46.4, 31.1, 30.1, 29.7, 26.6, 26.0, 23.6. **HR-MS** (ESI) m/z: calc. for $C_{15}H_{26}N_5O_6$ 372.1878 $(M+H)^+$, found 372.1877.

H-D-Pro-L-Tyr-L-Gln-NH_2 · TFA (UTS-98):

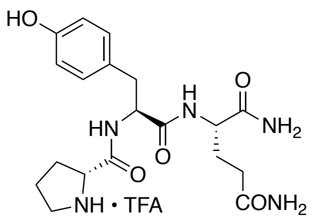

The peptide was synthesized according to the general procedure B for solid phase peptide synthesis.
^{1}H-NMR (500 MHz, D_2O) δ 7.23 – 7.11 (m, 2H), 6.92 – 6.81 (m, 2H), 4.72 (dd, *J* = 9.4, 6.5 Hz, 1H), 4.39 – 4.32 (m, 1H), 4.32 – 4.25 (m, 1H), 3.41 – 3.28 (m, 2H), 3.17 (dd, *J* = 14.0, 6.4 Hz, 1H), 2.91 (dd, *J* = 13.9, 9.6 Hz, 1H), 2.38 – 2.31 (m, 2H), 2.28 (dt, *J* = 13.7, 7.7 Hz, 1H), 2.16 – 2.05 (m, 1H), 2.03 – 1.91 (m, 2H), 1.81 (dp, *J* = 14.1, 7.2 Hz, 1H), 1.65 (dq, *J* = 13.5, 6.8 Hz, 1H). **^{13}C-NMR** (126 MHz, D_2O) δ 177.9, 175.4, 172.9, 169.5, 154.5, 130.5, 127.9, 115.5, 59.6, 55.0, 52.9, 46.4, 36.4, 31.0, 29.8, 26.7, 23.5. **HR-MS** (ESI) m/z: calc. for $C_{19}H_{28}N_5O_5$ 406.2085 $(M+H)^+$, found 406.2086.

H-D-Pro-D-Tyr-L-Gln-NH$_2$ · TFA (UTS-99):

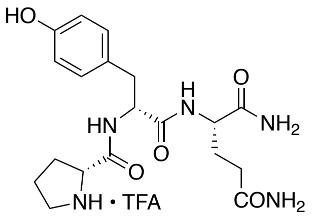

The peptide was synthesized according to the general procedure B for solid phase peptide synthesis.
^{1}H-NMR (400 MHz, D_2O) δ 7.13 (d, *J* = 8.4 Hz, 2H), 6.83 (d, *J* = 8.5 Hz, 2H), 4.46 (dd, *J* = 10.6, 6.1 Hz, 1H), 4.37 (dd, *J* = 8.7, 5.9 Hz, 1H), 4.04 (dd, *J* = 10.1, 4.2 Hz, 1H), 3.44 – 3.29 (m, 2H), 3.09 (dd, *J* = 13.2, 6.1 Hz, 1H), 2.86 (dd, *J* = 13.3, 10.7 Hz, 1H), 2.42 (dq, *J* = 9.7, 5.1 Hz, 1H), 2.09 – 1.95 (m, 3H), 1.90 (ddd, *J* = 19.4, 10.1, 5.1 Hz, 1H), 1.84 – 1.72 (m, 1H), 1.63 (ddd, *J* = 13.7, 9.8, 4.8 Hz, 1H), 1.53 (ddt, *J* = 15.2, 10.3, 5.6 Hz, 1H). **^{13}C-NMR** (101 MHz, D_2O) δ 177.7, 175.8, 173.2, 169.5, 154.7, 130.6, 127.3, 115.6, 59.4, 56.4, 52.8, 46.4, 35.7, 30.8, 29.7, 26.3, 23.6. **HR-MS** (ESI) m/z: calc. for $C_{19}H_{28}N_5O_5$ 406.2085 $(M+H)^+$, found 406.2085.

H-D-Pro-CyLeu-L-Gln-NH$_2$ · TFA (UTS-100):

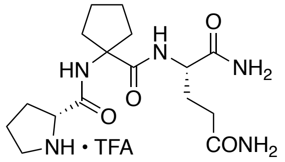

The peptide was synthesized according to the general procedure B for solid phase peptide synthesis.
^{1}H-NMR (500 MHz, D_2O) δ 4.43 (dd, *J* = 8.3, 5.6 Hz, 1H), 4.33 (dd, *J* = 9.7, 4.7 Hz, 1H), 3.48 – 3.35 (m, 2H), 2.54 – 2.42 (m, 1H), 2.39 (t, *J* = 7.6 Hz, 2H), 2.26 (dt, *J* = 13.7, 8.0 Hz, 1H), 2.21 – 2.12 (m, 1H), 2.12 – 1.95 (m, 6H), 1.91 (ddd, *J* = 13.9, 8.4, 4.0 Hz, 1H), 1.86 – 1.65 (m, 4H). **^{13}C-NMR** (126 MHz, D_2O) δ 178.0, 176.1, 176.0, 169.6, 67.4, 59.7, 53.3, 46.6, 36.8, 35.8, 31.3, 29.7, 26.2, 23.8, 23.7, 23.6. **HR-MS** (ESI) m/z: calc. for $C_{16}H_{28}N_5O_4$ 354.2136 $(M+H)^+$, found 354.2135

H-D-Pro-Abz-L-Gln-NH$_2$ · TFA (UTS-101):

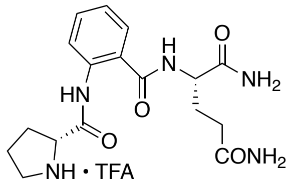

The peptide was synthesized according to the general procedure C for solid phase peptide synthesis.
^{1}H-NMR (400 MHz, D_2O) δ 7.73 – 7.35 (m, 4H), 7.27 – 7.14 (m, 1H), 4.61 – 4.40 (m, 2H), 3.49 – 3.34 (m, 1H), 2.45 (t, *J* = 7.3 Hz, 3H), 2.28 – 2.17 (m, 2H), 2.17 – 1.97 (m, 3H). **^{13}C-NMR** (101 MHz, D_2O) δ 177.9, 176.2, 168.4, 133.2, 132.2, 128.7, 128.5, 127.0, 125.3, 60.0, 53.4, 46.5, 31.3, 29.3, 26.7, 23.7. **HR-MS** (ESI) m/z: calc. for $C_{17}H_{24}N_5O_4$ 362.1823 $(M+H)^+$, found 362.1822.

H-D-Pro-Ind-L-Gln-NH$_2$ · TFA (UTS-102):

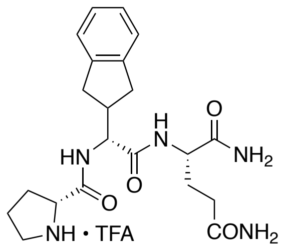

The peptide was synthesized according to the general procedure B for solid phase peptide synthesis.
^{1}H-NMR (400 MHz, D_2O) δ 7.31 (q, *J* = 3.8 Hz, 2H), 7.27 – 7.20 (m, 2H), 4.49 – 4.34 (m, 2H), 4.26 (dd, *J* = 9.2, 5.2 Hz, 1H), 3.42 (tt, *J* = 11.6, 5.7 Hz, 2H), 3.18 (dd, *J* = 14.7, 6.5 Hz, 1H), 3.07 (dd, *J* = 14.6, 6.3 Hz, 1H), 2.98 – 2.76 (m, 3H), 2.54 – 2.34 (m, 3H), 2.22 – 1.88 (m, 5H). **^{13}C-NMR** (101 MHz, D_2O) δ 177.7, 175.8, 173.1, 169.8, 142.0, 141.9, 126.8, 124.6, 124.5, 59.4, 58.0, 53.1, 46.5, 40.6, 35.5, 35.0, 31.2, 29.9, 26.6, 23.7. **HR-MS** (ESI) m/z: calc. for $C_{21}H_{30}N_5O_4$ 416.2292 $(M+H)^+$, found 416.2293.

H-D-Pro-D-Gln-D-Gln-NH$_2$ · TFA (UTS-103):

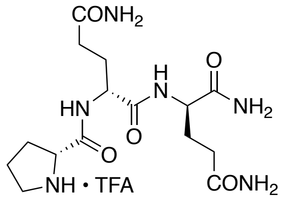

The peptide was synthesized according to the general procedure B for solid phase peptide synthesis.
^{1}H-NMR (400 MHz, D_2O) δ 4.51 – 4.27 (m, 3H), 3.43 (dtd, *J* = 18.3, 11.7, 7.1 Hz, 2H), 2.49 (dt, *J* = 8.4, 7.0 Hz, 1H), 2.40 (td, *J* = 7.5, 1.5 Hz, 4H), 2.21 – 1.93 (m, 7H). **^{13}C-NMR** (101 MHz, D_2O) δ 177.9, 177.7, 175.6, 172.9, 169.6, 59.6,

53.4, 52.9, 46.5, 31.0, 30.9, 29.7, 26.9, 26.7, 23.7. **HR-MS** (ESI) m/z: calc. for $C_{15}H_{27}N_6O_5$ 371.2037 $(M+H)^+$, found 371.2035.

H-D-Pro-D-Gln-L-Glu-NH$_2$ · TFA UTS-104):

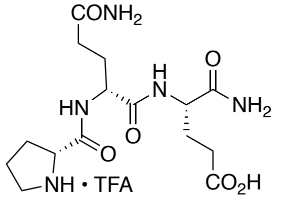

The peptide was synthesized according to the general procedure B for solid phase peptide synthesis.
^{1}H-NMR (400 MHz, D_2O) δ 4.42 (ddd, *J* = 14.3, 8.5, 6.2 Hz, 2H), 4.34 (dd, *J* = 9.4, 5.1 Hz, 1H), 3.50 – 3.35 (m, 2H), 2.49 (td, *J* = 7.1, 1.6 Hz, 3H), 2.40 (t, *J* = 7.5 Hz, 2H), 2.25 – 1.93 (m, 7H). **^{13}C-NMR** (101 MHz, D_2O) δ 177.6, 177.4, 175.9, 173.1, 169.7, 59.6, 53.6, 52.9, 46.5, 30.9, 30.5, 29.7, 26.6, 26.17, 23.7. **HR-MS** (ESI) m/z: calc. for $C_{15}H_{26}N_5O_6$ 372.1878 $(M+H)^+$, found 372.1878.

H-D-Pro-D-Gln-D-Glu-NH$_2$ · TFA (UTS-105):

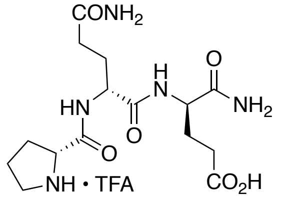

The peptide was synthesized according to the general procedure B for solid phase peptide synthesis.
^{1}H-NMR (400 MHz, D_2O) δ 4.48 – 4.32 (m, 3H), 3.51 – 3.34 (m, 2H), 2.48 (ddd, *J* = 10.3, 6.5, 2.3 Hz, 3H), 2.40 (t, *J* = 7.5 Hz, 2H), 2.20 – 1.92 (m, 7H). **^{13}C-NMR** (101 MHz, D_2O) δ 177.7, 177.5, 175.7, 172.9, 169.6, 59.5, 53.4, 52.7, 46.5, 30.9, 30.2, 29.7, 26.6, 26.4, 23.7. **HR-MS** (ESI) m/z: calc. for $C_{15}H_{26}N_5O_6$ 372.1878 $(M+H)^+$, found 372.1877.

H-D-Pro-D-Gln-L-Tyr-NH$_2$ · TFA (UTS-106):

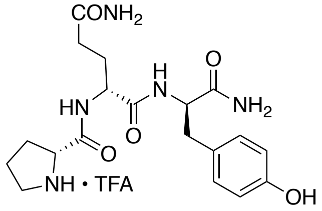

The peptide was synthesized according to the general procedure B for solid phase peptide synthesis.
^{1}H-NMR (400 MHz, D_2O) δ 7.14 (d, *J* = 8.5 Hz, 2H), 6.84 – 6.77 (m, 2H), 4.65 (dd, *J* = 11.1, 4.7 Hz, 1H), 4.33 (dd, *J* = 8.7, 6.4 Hz, 1H), 4.20 (t, *J* = 6.8 Hz, 1H), 3.41 – 3.31 (m, 2H), 3.31 – 3.20 (m, 1H), 2.79 (dd, *J* = 14.4, 11.1 Hz, 1H), 2.42 – 2.30 (m, 1H), 2.07 – 1.79 (m, 4H), 1.79 – 1.63 (m, 3H). **^{13}C-NMR** (101 MHz, D_2O) δ 177.4, 175.8, 172.8, 154.4, 130.3, 128.3, 115.4, 59.4, 54.3, 53.6, 46.4, 35.9, 30.4, 29.7, 26.6, 23.6. **HR-MS** (ESI) m/z: calc. for $C_{19}H_{27}N_5O_5Na$ 428.1904 $(M+Na)^+$, found 428.1904.

H-D-Pro-D-Gln-D-Tyr-NH$_2$ · TFA (UTS-107):

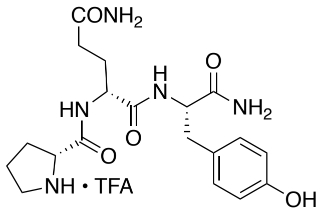

The peptide was synthesized according to the general procedure B for solid phase peptide synthesis.
^{1}H-NMR (400 MHz, D_2O) δ 7.13 (d, *J* = 8.4 Hz, 2H), 6.83 – 6.75 (m, 2H), 4.57 (dd, *J* = 9.5, 5.8 Hz, 1H), 4.31 (dd, *J* = 8.6, 6.8 Hz, 1H), 4.26 (dd, *J* = 8.1, 6.6 Hz, 1H), 3.37 (td, *J* = 7.1, 2.1 Hz, 2H), 3.09 (dd, *J* = 14.0, 5.8 Hz, 1H), 2.88 (dd, *J* = 14.0, 9.5 Hz, 1H), 2.40 – 2.28 (m, 1H), 2.20 (dtd, *J* = 23.7, 15.2, 6.9 Hz, 2H), 2.07 – 1.87 (m, 4H), 1.87 – 1.75 (m, 1H). **^{13}C-NMR** (101 MHz, D_2O) δ 177.6, 175.5, 172.4, 169.3, 154.3, 130.5, 128.1, 115.3, 59.4, 54.4, 53.2, 46.5, 36.2, 30.7, 29.7, 26.8, 23.7. **HR-MS** (ESI) m/z: calc. for $C_{19}H_{28}N_5O_5$ 406.2085 $(M+H)^+$, found 406.2083.

H-D-Pro-D-Gln-CyLeu-NH2 · TFA (UTS-108):

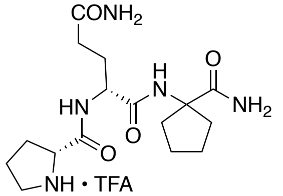

The peptide was synthesized according to the general procedure B for solid phase peptide synthesis.
^{1}H-NMR (500 MHz, D_2O) δ 4.46 – 4.39 (m, 1H), 4.35 (dd, *J* = 8.4, 6.1 Hz, 1H), 3.49 – 3.35 (m, 2H), 2.52 – 2.43 (m, 1H), 2.39 (dd, *J* = 8.1, 7.0 Hz, 2H), 2.25 – 2.16 (m, 1H), 2.15 – 1.99 (m, 6H), 1.99 – 1.90 (m, 2H), 1.84 – 1.67 (m, 4H). **^{13}C-NMR** (126 MHz, D_2O) δ 179.1, 177.6, 172.5, 169.7, 66.9, 59.5, 53.4, 46.5, 37.2, 35.8, 30.8, 29.8, 26.5, 23.9, 23.8, 23.7. **HR-MS** (ESI) m/z: calc. for $C_{16}H_{28}N_5O_4$ 354.2136 $(M+H)^+$, found 354.2135.

H-D-Pro-D-Gln-Abz-NH2 · TFA (UTS-109):

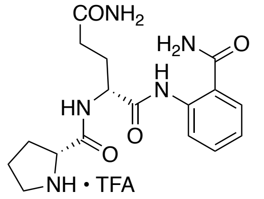

The peptide was synthesized according to the general procedure C for solid phase peptide synthesis.
^{1}H-NMR (500 MHz, D_2O) δ 7.82 – 7.56 (m, 4H), 7.42 – 7.31 (m, 2H), 4.48 (td, *J* = 8.6, 6.3 Hz, 2H), 3.51 – 3.36 (m, 2H), 2.56 – 2.43 (m, 3H), 2.25 (dq, *J* = 14.0, 7.2 Hz, 1H), 2.21 – 2.00 (m, 4H). **^{13}C-NMR** (126 MHz, D_2O) δ 177.7, 172.9, 171.6, 169.9, 132.3, 128.5, 126.2, 124.3, 59.7, 54.4, 46.5, 31.0, 29.6, 26.5, 23.7. **HR-MS** (ESI) m/z: calc. for $C_{17}H_{24}N_5O_4$ 362.1823 $(M+H)^+$, found 362.1821.

H-D-Pro-D-Gln-D-Ind-NH2 · TFA (UTS-110):

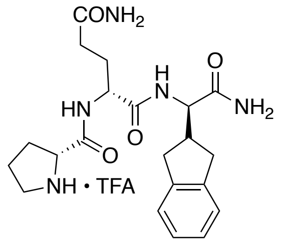

The peptide was synthesized according to the general procedure B for solid phase peptide synthesis.
^{1}H-NMR (500 MHz, D_2O) δ 7.32 – 7.27 (m, 2H), 7.26 – 7.21 (m, 2H), 4.46 – 4.34 (m, 3H), 3.46 – 3.34 (m, 2H), 3.10 (td, *J* = 16.1, 7.6 Hz, 2H), 2.98 – 2.86 (m, 1H), 2.81 (td, *J* = 14.9, 7.6 Hz, 2H), 2.49 – 2.40 (m, 1H), 2.37 (dd, *J* = 8.0, 7.1 Hz, 2H), 2.13 – 1.93 (m, 5H). **^{13}C-NMR** (126 MHz, D_2O) δ 177.7, 175.4, 172.7, 142.3, 142.0, 126.7, 124.5, 124.5, 59.5, 56.6, 53.3, 46.5, 41.1, 35.2, 34.9, 30.9, 29.8, 26.6, 23.7. **HR-MS** (ESI) m/z: calc. for $C_{21}H_{30}N_5O_4$ 416.2292 $(M+H)^+$, found 416.2289

H-D-Pro-L-Glu-D-Gln-NH2 · TFA (UTS-111):

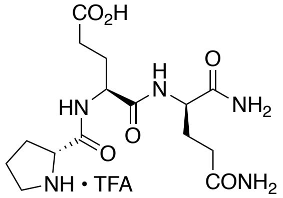

The peptide was synthesized according to the general procedure B for solid phase peptide synthesis.
^{1}H-NMR (400 MHz, D_2O) δ 4.45 – 4.36 (m, 2H), 4.26 (dd, *J* = 9.3, 5.0 Hz, 1H), 3.39 (dtd, *J* = 18.3, 11.6, 7.1 Hz, 2H), 2.49 – 2.41 (m, 3H), 2.37 (t, *J* = 7.4 Hz, 2H), 2.20 – 1.90 (m, 7H). **^{13}C-NMR** (101 MHz, D_2O) δ 177.8, 177.0, 175.9, 173.3, 169.8, 59.7, 53.3, 53.2, 46.4, 31.1, 30.1, 29.7, 26.5, 26.1, 23.7. **HR-MS** (ESI) m/z: calc. for $C_{15}H_{26}N_5O_6$ 372.1878 $(M+H)^+$, found 372.1878.

H-D-Pro-D-Glu-D-Gln-NH2 · TFA (UTS-112):

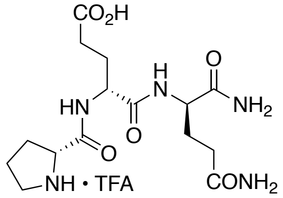

The peptide was synthesized according to the general procedure B for solid phase peptide synthesis.
^{1}H-NMR (400 MHz, D_2O) δ 4.43 (dt, *J* = 9.0, 6.5 Hz, 2H), 4.34 (dd, *J* = 8.9, 5.4 Hz, 1H), 3.50 – 3.36 (m, 2H), 2.49 (ddd, *J* = 10.7, 8.9, 6.5 Hz, 3H), 2.40 (t, *J* = 7.5 Hz, 2H), 2.21 – 1.95 (m, 7H). **^{13}C-NMR** (101 MHz, D_2O) δ 177.9, 177.8, 175.5, 173.0, 169.7, 59.5, 53.3, 52.9, 46.5, 31.0, 30.1, 29.7, 26.8, 26.1, 23.7. **HR-MS** (ESI) m/z: calc. for $C_{15}H_{26}N_5O_6$ 372.1878 $(M+H)^+$, found 372.1876.

H-D-Pro-L-Tyr-D-Gln-NH$_2$ · TFA (UTS-113):

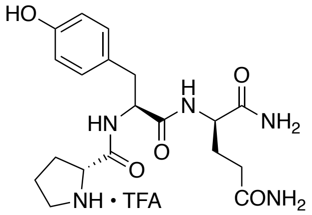

The peptide was synthesized according to the general procedure B for solid phase peptide synthesis.
^{1}H-NMR (400 MHz, D$_2$O) δ 7.20 – 7.13 (m, 2H), 6.90 – 6.84 (m, 2H), 4.61 (t, *J* = 8.2 Hz, 1H), 4.37 (dd, *J* = 8.6, 6.5 Hz, 1H), 4.16 (dd, *J* = 9.7, 4.0 Hz, 1H), 3.44 – 3.30 (m, 2H), 3.03 (d, *J* = 8.3 Hz, 2H), 2.45 – 2.30 (m, 1H), 2.09 – 1.96 (m, 3H), 1.95 – 1.88 (m, 1H), 1.88 – 1.73 (m, 3H). **^{13}C-NMR** (101 MHz, D$_2$O) δ 177.8, 175.9, 173.3, 169.6, 154.7, 130.6, 127.6, 115.6, 59.6, 55.7, 53.1, 46.4, 36.2, 31.0, 29.8, 26.4, 23.6. **HR-MS** (ESI) m/z: calc. for $C_{19}H_{28}N_5O_5$ 406.2085 $(M+H)^+$, found 406.2086.

H-D-Pro-D-Tyr-D-Gln-NH$_2$ · TFA (UTS-114):

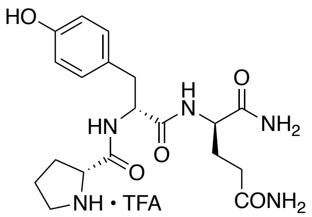

The peptide was synthesized according to the general procedure B for solid phase peptide synthesis.
^{1}H-NMR (400 MHz, D$_2$O) δ 7.23 – 7.09 (m, 2H), 6.92 – 6.80 (m, 2H), 4.57 (t, *J* = 7.9 Hz, 1H), 4.36 (ddd, *J* = 8.6, 6.0, 4.7 Hz, 1H), 4.25 (dd, *J* = 9.1, 5.3 Hz, 1H), 3.41 (ddt, *J* = 14.8, 11.6, 6.1 Hz, 2H), 3.12 – 2.94 (m, 2H), 2.54 – 2.36 (m, 2H), 2.29 (t, *J* = 7.8 Hz, 2H), 2.19 – 1.96 (m, 5H), 1.96 – 1.82 (m, 1H). **^{13}C-NMR** (101 MHz, D$_2$O) δ 177.9, 175.0, 172.7, 169.4, 154.6, 130.5, 127.6, 115.6, 59.5, 55.8, 52.6, 46.5, 36.0, 30.9, 29.7, 26.8, 23.7. **HR-MS** (ESI) m/z: calc. for $C_{19}H_{28}N_5O_5$ 406.2085 $(M+H)^+$, found 406.2092.

H-D-Pro-CyLeu-D-Gln-NH$_2$ · TFA (UTS-115):

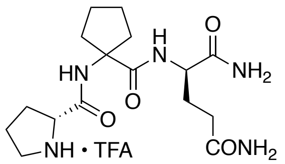

The peptide was synthesized according to the general procedure B for solid phase peptide synthesis.
^{1}H-NMR (400 MHz, D$_2$O) δ 4.41 (dd, *J* = 8.7, 6.0 Hz, 1H), 4.33 (dd, *J* = 9.4, 4.8 Hz, 1H), 3.49 – 3.34 (m, 2H), 2.54 – 2.43 (m, 1H), 2.38 (td, *J* = 7.2, 1.3 Hz, 2H), 2.23 – 1.90 (m, 9H), 1.78 (q, *J* = 5.1 Hz, 4H). **^{13}C-NMR** (101 MHz, D$_2$O) δ 178.1, 176.0, 175.9, 169.3, 67.3, 59.7, 53.3, 46.6, 36.5, 35.9, 31.2, 29.7, 26.4, 23.7, 23.7, 23.5. **HR-MS** (ESI) m/z: calc. for $C_{16}H_{27}N_5O_5Na$ 376.1955 $(M+Na)^+$, found 376.1954.

H-D-Pro-Abz-D-Gln-NH$_2$ · TFA (UTS-116):

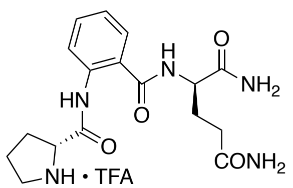

The peptide was synthesized according to the general procedure C for solid phase peptide synthesis.
^{1}H-NMR (500 MHz, D$_2$O) δ 7.74 (ddd, *J* = 7.9, 1.5, 0.4 Hz, 1H), 7.71 – 7.67 (m, 1H), 7.66 – 7.60 (m, 1H), 7.59 – 7.54 (m, 2H), 7.47 – 7.41 (m, 1H), 7.29 – 7.22 (m, 2H), 4.58 (dd, *J* = 8.7, 6.8 Hz, 1H), 4.50 (ddd, *J* = 15.5, 9.1, 5.2 Hz, 2H), 3.54 – 3.40 (m, 2H), 2.59 – 2.52 (m, 1H), 2.48 (q, *J* = 7.3 Hz, 4H), 2.29 – 2.19 (m, 3H), 2.19 – 2.05 (m, 4H). **^{13}C-NMR** (126 MHz, D$_2$O) δ 178.0, 176.2, 170.3, 168.4, 133.2, 132.2, 128.8, 127.0, 125.4, 121.5, 60.2, 53.4, 46.5, 31.4, 29.3, 26.7, 23.8. **HR-MS** (ESI) m/z: calc. for $C_{17}H_{24}N_5O_4$ 362.1823 $(M+H)^+$, found 362.1823.

H-D-Pro-D-Ind-D-Gln-NH_2 · TFA (UTS-117):

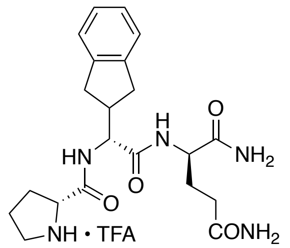

The peptide was synthesized according to the general procedure B for solid phase peptide synthesis.
^{1}H-NMR (400 MHz, D_2O) δ 7.30 (h, *J* = 3.7 Hz, 2H), 7.24 (dd, *J* = 5.6, 3.2 Hz, 2H), 4.41 (dt, *J* = 8.7, 3.3 Hz, 2H), 4.32 (dd, *J* = 8.8, 5.6 Hz, 1H), 3.50 – 3.34 (m, 2H), 3.15 (dd, *J* = 14.8, 6.5 Hz, 1H), 3.06 (q, *J* = 10.3 Hz, 1H), 2.95 – 2.77 (m, 3H), 2.55 – 2.42 (m, 1H), 2.38 (t, *J* = 7.6 Hz, 2H), 2.05 (dddd, *J* = 27.9, 14.6, 10.2, 6.4 Hz, 5H). **^{13}C-NMR** (101 MHz, D_2O) δ 177.9, 175.3, 172.8, 169.7, 142.1, 141.9, 126.7, 124.6, 124.5, 59.5, 57.7, 52.7, 46.6, 40.8, 35.4, 34.9, 31.0, 29.8, 26.9, 23.7. **HR-MS** (ESI) m/z: calc. for $C_{21}H_{30}N_5O_4$ 416.2292 $(M+H)^+$, found 416.2290.

H-D-Pro-L-Glu-L-Glu-NH_2 · TFA (UTS-118):

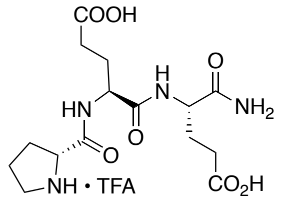

The peptide was synthesized according to the general procedure B for solid phase peptide synthesis.
^{1}H-NMR (400 MHz, D_2O) δ 4.44 (ddd, *J* = 9.1, 5.7, 2.7 Hz, 2H), 4.37 (dd, *J* = 9.2, 5.3 Hz, 1H), 3.44 (qt, *J* = 11.5, 7.0 Hz, 2H), 2.56 – 2.41 (m, 5H), 2.24 – 1.94 (m, 7H). **^{13}C-NMR** (101 MHz, D_2O) δ 177.6, 177.3, 175.7, 173.1, 169.9, 59.8, 53.3, 52.9, 46.5, 30.3, 30.3, 29.7, 26.2, 23.8. **HR-MS** (ESI) m/z: calc. for $C_{15}H_{25}N_4O_7$ 373.1718 $(M+H)^+$, found 373.1717.

H-D-Pro-L-Glu-D-Glu-NH_2 · TFA (UTS-119):

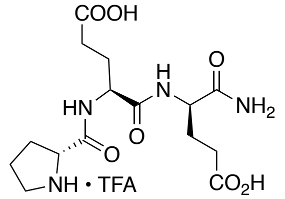

The peptide was synthesized according to the general procedure B for solid phase peptide synthesis.
^{1}H-NMR (400 MHz, D_2O) δ 4.40 (dd, *J* = 8.9, 5.8 Hz, 2H), 4.29 (dd, *J* = 9.5, 5.0 Hz, 1H), 3.39 (qt, *J* = 11.5, 7.1 Hz, 2H), 2.45 (q, *J* = 7.3 Hz, 5H), 2.19 – 1.91 (m, 7H). **^{13}C-NMR** (101 MHz, D_2O) δ 177.5, 177.2, 175.9, 173.3, 169.8, 59.7, 53.4, 53.1, 46.4, 30.4, 30.2, 29.7, 26.2, 26.0, 23.7. **HR-MS** (ESI) m/z: calc. for $C_{15}H_{25}N_4O_7$ 373.1718 $(M+H)^+$, found 373.1717.

H-D-Pro-L-Glu-L-Tyr-NH_2 · TFA (UTS-120):

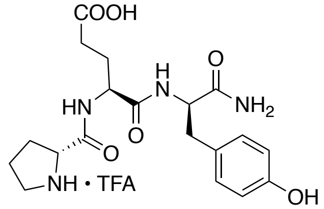

The peptide was synthesized according to the general procedure B for solid phase peptide synthesis.
^{1}H-NMR (400 MHz, D_2O) δ 7.20 – 7.14 (m, 2H), 6.88 – 6.82 (m, 2H), 4.60 (dd, *J* = 9.3, 6.2 Hz, 1H), 4.39 (dd, *J* = 8.2, 6.1 Hz, 1H), 4.30 (dd, *J* = 8.4, 6.2 Hz, 1H), 3.50 – 3.36 (m, 2H), 3.14 (dd, *J* = 14.0, 6.2 Hz, 1H), 2.98 – 2.89 (m, 1H), 2.53 – 2.41 (m, 1H), 2.37 – 2.19 (m, 2H), 2.13 – 2.01 (m, 3H), 2.00 – 1.84 (m, 2H). **^{13}C-NMR** (101 MHz, D_2O) δ 177.3, 175.6, 172.7, 169.8, 154.4, 130.5, 128.3, 115.4, 59.8, 54.7, 53.5, 46.5, 36.1, 30.2, 29.7, 26.3, 23.7. **HR-MS** (ESI) m/z: calc. for $C_{19}H_{27}N_4O_6$ 407.1925 $(M+H)^+$, found 407.1925.

H-D-Pro-L-Glu-D-Tyr-NH_2 · TFA (UTS-121):

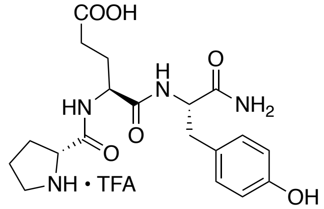

The peptide was synthesized according to the general procedure B for solid phase peptide synthesis.
^{1}H-NMR (500 MHz, D_2O) δ 7.21 – 7.15 (m, 2H), 6.87 – 6.82 (m, 2H), 4.67 (dd, *J* = 10.9, 5.0 Hz, 1H), 4.38 (dd, *J* = 8.2, 6.3 Hz, 1H), 4.32 (dd, *J* = 7.6, 6.2 Hz, 1H), 3.48 – 3.33 (m, 2H), 3.27 (dd, *J* = 14.3, 5.0 Hz, 1H), 2.84 (dd, *J* = 14.2, 10.9 Hz, 1H), 2.44 (dq, *J* = 10.5, 5.4 Hz, 1H), 2.12 – 2.01 (m, 3H), 2.01 – 1.90 (m, 2H), 1.75 (ddt, *J* = 22.4, 14.3, 6.9 Hz, 2H). **^{13}C-NMR** (126 MHz,

D_2O) δ 175.9, 172.9, 169.5, 154.5, 130.3, 128.4, 115.5, 59.6, 54.6, 53.3, 46.5, 36.0, 29.7, 23.7. **HR-MS** (ESI) m/z: calc. for $C_{19}H_{27}N_4O_6$ 407.1925 $(M+H)^+$, found 407.1924.

H-D-Pro-L-Glu-CyLeu-NH₂ · TFA (UTS-122):

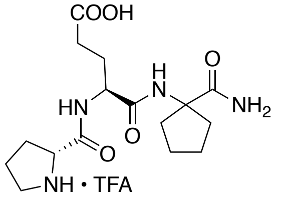

The peptide was synthesized according to the general procedure B for solid phase peptide synthesis.
^{1}H-NMR (500 MHz, D_2O) δ 4.40 (ddd, *J* = 14.8, 8.6, 6.1 Hz, 2H), 3.51 – 3.35 (m, 2H), 2.53 – 2.42 (m, 3H), 2.21 (dt, *J* = 13.4, 7.3 Hz, 1H), 2.15 – 2.05 (m, 5H), 2.05 – 1.90 (m, 3H), 1.83 – 1.69 (m, 4H). **^{13}C-NMR** (126 MHz, D_2O) δ 179.2, 172.8, 169.8, 67.0, 59.7, 53.3, 46.5, 37.2, 35.7, 29.8, 23.9, 23.8, 23.7. **HR-MS** (ESI) m/z: calc. for $C_{16}H_{27}N_4O_5$ 355.1976 $(M+H)^+$, found 355.1975.

H-D-Pro-L-Glu-Abz-NH₂ · TFA (UTS-123):

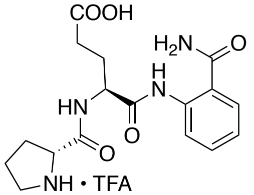

The peptide was synthesized according to the general procedure C for solid phase peptide synthesis.
^{1}H-NMR (400 MHz, D_2O) δ 7.74 – 7.69 (m, 1H), 7.68 – 7.62 (m, 1H), 7.60 – 7.53 (m, 2H), 7.41 – 7.31 (m, 1H), 4.54 – 4.44 (m, 2H), 3.48 – 3.34 (m, 2H), 2.53 (dt, *J* = 24.7, 7.3 Hz, 4H), 2.34 – 2.17 (m, 2H), 2.08 (tt, *J* = 15.8, 8.4 Hz, 4H). **^{13}C-NMR** (101 MHz, D_2O) δ 177.6, 171.7, 169.95, 132.3, 128.5, 126.1, 124.1, 59.8, 54.1, 46.5, 30.6, 29.7, 26.1, 23.8. **HR-MS** (ESI) m/z: calc. for $C_{17}H_{23}N_4O_5$ 363.1663 $(M+H)^+$, found 363.1662.

H-D-Pro-L-Glu-L-Ind-NH₂ · TFA (UTS-124):

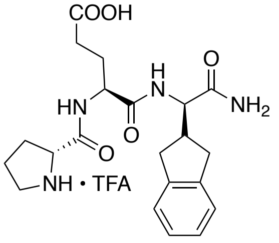

The peptide was synthesized according to the general procedure B for solid phase peptide synthesis.
^{1}H-NMR (400 MHz, D_2O) δ 7.29 (t, *J* = 4.9 Hz, 2H), 7.26 – 7.19 (m, 2H), 4.48 – 4.36 (m, 3H), 3.51 – 3.35 (m, 2H), 3.15 (dt, *J* = 15.7, 7.8 Hz, 2H), 3.02 – 2.90 (m, 1H), 2.82 (dt, *J* = 15.8, 7.9 Hz, 2H), 2.53 – 2.43 (m, 1H), 2.39 (t, *J* = 7.3 Hz, 2H), 2.14 – 2.01 (m, 3H), 1.96 – 1.85 (m, 2H). **^{13}C-NMR** (101 MHz, D_2O) δ 177.2, 175.8, 173.3, 169.7, 142.4, 142.1, 126.8, 126.8, 124.6, 124.6, 59.8, 57.4, 53.3, 46.6, 40.4, 35.6, 35.1, 30.3, 29.8, 26.5, 23.8. **HR-MS** (ESI) m/z: calc. for $C_{21}H_{29}N_4O_5$ 417.2132 $(M+H)^+$, found 417.2130.

H-D-Pro-D-Glu-L-Glu-NH₂· TFA (UTS-125):

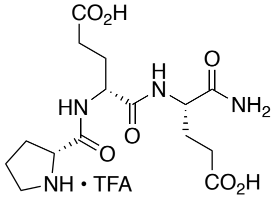

The peptide was synthesized according to the general procedure B for solid phase peptide synthesis.
^{1}H-NMR (400 MHz, D_2O) δ 4.48 – 4.38 (m, 2H), 4.34 (dd, *J* = 9.5, 5.0 Hz, 1H), 3.42 (qt, *J* = 11.6, 7.1 Hz, 2H), 2.55 – 2.41 (m, 5H), 2.24 – 1.94 (m, 7H). **^{13}C-NMR** (101 MHz, D_2O) δ 177.4, 177.3, 175.9, 173.2, 169.7, 59.5, 53.9, 52.9, 46.5, 30.4, 30.3, 29.7, 26.2, 26.1, 23.7. **HR-MS** (ESI) m/z: calc. for $C_{15}H_{25}N_4O_7$ 373.1718 $(M+H)^+$, found 373.1717.

H-D-Pro-L-Tyr-L-Glu-NH₂ · TFA (UTS-126):

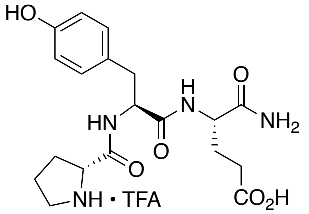

The peptide was synthesized according to the general procedure B for solid phase peptide synthesis.
^{1}H-NMR (400 MHz, D_2O) δ 7.20 – 7.14 (m, 2H), 6.89 – 6.83 (m, 2H), 4.71 (dd, *J* = 9.3, 6.5 Hz, 1H), 4.34 (td, *J* = 8.9, 5.8 Hz, 2H), 3.41 – 3.28 (m, 2H), 3.16 (dd, *J* = 13.9, 6.6 Hz, 1H), 2.98 – 2.85 (m, 1H), 2.43 (t, *J* = 7.3 Hz, 2H), 2.34 – 2.23 (m, 1H), 2.12 (dtd, *J* = 13.0, 7.6, 5.1 Hz, 1H), 1.96 (ddt, *J* = 14.1, 9.4, 7.0 Hz, 2H), 1.81 (dp, *J* = 14.2, 7.2 Hz, 1H), 1.66 (dq, *J* = 13.7, 6.8 Hz,

1H). **^{13}C-NMR** (101 MHz, D_2O) δ 177.8, 175.5, 172.9, 169.5, 154.5, 130.5, 127.9, 115.5, 59.6, 55.0, 52.8, 46.4, 36.3, 30.4, 29.8, 26.7, 23.5. **HR-MS** (ESI) m/z: calc. for $C_{19}H_{27}N_4O_6$ 407.1925 $(M+H)^+$, found 407.1925.

H-D-Pro-D-Tyr-L-Glu-NH₂ · TFA (UTS-127):

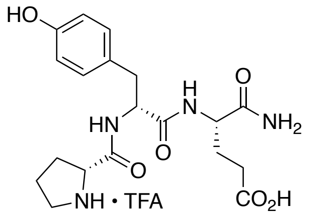

The peptide was synthesized according to the general procedure B for solid phase peptide synthesis.
^{1}H-NMR (400 MHz, D_2O) δ 7.19 – 7.13 (m, 2H), 6.90 – 6.84 (m, 2H), 4.51 (dd, *J* = 10.4, 6.2 Hz, 1H), 4.47 – 4.37 (m, 2H), 4.14 (dd, *J* = 10.0, 4.1 Hz, 1H), 3.51 – 3.33 (m, 3H), 3.12 (dd, *J* = 13.3, 6.2 Hz, 1H), 2.91 (dd, *J* = 13.3, 10.4 Hz, 1H), 2.54 – 2.39 (m, 3H), 2.23 – 1.77 (m, 10H), 1.73 – 1.59 (m, 1H). **^{13}C-NMR** (101 MHz, D_2O) δ 177.4, 175.9, 173.2, 169.5, 154.8, 130.5, 127.3, 115.6, 59.5, 56.4, 52.7, 46.5, 35.8, 29.7, 29.7, 25.9, 23.6. **HR-MS** (ESI) m/z: calc. for $C_{19}H_{27}N_4O_6$ 407.1925 $(M+H)^+$, found 407.1931.

H-D-Pro-CyLeu-L-Glu-NH₂ · TFA (UTS-128):

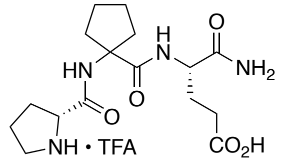

The peptide was synthesized according to the general procedure B for solid phase peptide synthesis.
^{1}H-NMR (400 MHz, D_2O) δ 4.47 – 4.33 (m, 2H), 3.49 – 3.35 (m, 2H), 2.48 (t, *J* = 7.2 Hz, 3H), 2.31 – 2.15 (m, 2H), 2.15 – 1.86 (m, 7H), 1.86 – 1.66 (m, 4H). **^{13}C-NMR** (101 MHz, D_2O) δ 177.3, 176.1, 176.0, 169.6, 67.4, 59.7, 53.0, 46.6, 36.8, 35.8, 30.2, 29.7, 25.7, 23.8, 23.7, 23.6. **HR-MS** (ESI) m/z: calc. for $C_{16}H_{27}N_4O_5$ 355.1976 $(M+H)^+$, found 355.1974.

H-D-Pro-Abz-L-Glu-NH₂ · TFA (UTS-129):

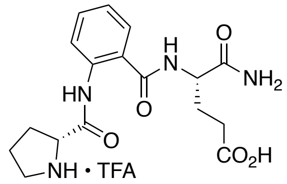

The peptide was synthesized according to the general procedure C for solid phase peptide synthesis.
^{1}H-NMR (400 MHz, D_2O) δ 7.73 – 7.59 (m, 3H), 7.56 – 7.50 (m, 1H), 7.50 – 7.40 (m, 1H), 7.20 – 7.12 (m, 1H), 4.63 – 4.57 (m, 1H), 4.54 (td, *J* = 9.2, 5.2 Hz, 1H), 3.54 – 3.40 (m, 2H), 2.63 – 2.49 (m, 4H), 2.33 – 2.19 (m, 2H), 2.19 – 2.03 (m, 3H). **^{13}C-NMR** (101 MHz, D_2O) δ 177.4, 176.0, 170.4, 168.5, 133.2, 132.2, 128.5, 127.1, 125.3, 120.5, 60.1, 53.1, 46.6, 30.5, 29.4, 26.3, 23.8. **HR-MS** (ESI) m/z: calc. for $C_{17}H_{23}N_4O_5$ 363.1663 $(M+H)^+$, found 363.1663.

H-D-Pro-D-Ind-L-Glu-NH₂ · TFA (UTS-130):

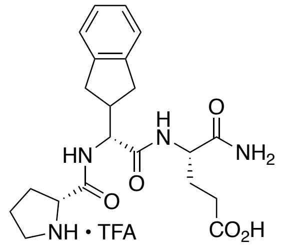

The peptide was synthesized according to the general procedure B for solid phase peptide synthesis.
^{1}H-NMR (400 MHz, D_2O) δ 7.31 (dd, *J* = 5.5, 3.3 Hz, 2H), 7.28 – 7.21 (m, 2H), 4.50 – 4.35 (m, 2H), 4.30 (dd, *J* = 9.6, 5.0 Hz, 1H), 3.42 (pd, *J* = 11.8, 7.1 Hz, 2H), 3.18 (dd, *J* = 14.4, 6.2 Hz, 1H), 3.12 – 3.00 (m, 1H), 2.95 – 2.80 (m, 3H), 2.58 – 2.40 (m, 3H), 2.18 (td, *J* = 13.4, 7.7 Hz, 1H), 2.13 – 1.89 (m, 4H). **^{13}C-NMR** (101 MHz, D_2O) δ 177.1, 175.9, 173.2, 169.8, 142.0, 141.9, 126.8, 124.6, 124.5, 59.4, 58.0, 52.9, 46.5, 40.6, 35.5, 35.0, 30.3, 29.8, 26.0, 23.6. **HR-MS** (ESI) m/z: calc. for $C_{21}H_{29}N_4O_5$ 417.2132 $(M+H)^+$, found 417.2130.

H-D-Pro-D-Glu-D-Glu-NH₂ · TFA (UTS-131):

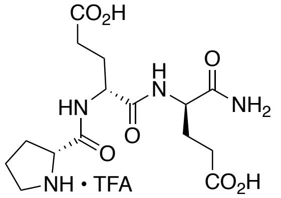

The peptide was synthesized according to the general procedure B for solid phase peptide synthesis.
¹H-NMR (400 MHz, D_2O) δ 4.43 – 4.31 (m, 3H), 3.38 (qt, *J* = 11.3, 7.0 Hz, 2H), 2.51 – 2.37 (m, 5H), 2.17 – 1.90 (m, 7H). **¹³C-NMR** (101 MHz, D_2O) δ 177.4, 175.6, 173.0, 169.6, 59.5, 53.3, 52.7, 46.4, 30.2, 30.1, 29.7, 26.3, 26.1, 23.6. **HR-MS** (ESI) m/z: calc. for $C_{15}H_{25}N_4O_7$ 373.1718 $(M+H)^+$, found 373.1716.

H-D-Pro-D-Glu-L-Tyr-NH₂ · TFA (UTS-132):

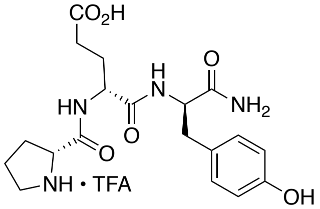

The peptide was synthesized according to the general procedure B for solid phase peptide synthesis.
¹H-NMR (400 MHz, D_2O) δ 7.16 – 7.10 (m, 2H), 6.82 – 6.77 (m, 2H), 4.64 (dd, *J* = 10.9, 4.7 Hz, 1H), 4.33 (dd, *J* = 8.6, 6.3 Hz, 1H), 4.25 (t, *J* = 6.9 Hz, 1H), 3.41 – 3.30 (m, 2H), 3.24 (dd, *J* = 14.3, 4.8 Hz, 1H), 2.79 (dd, *J* = 14.3, 10.9 Hz, 1H), 2.44 – 2.30 (m, 1H), 2.03 (d, *J* = 0.7 Hz, 1H), 2.01 – 1.84 (m, 5H), 1.80 – 1.62 (m, 2H). **¹³C-NMR** (101 MHz, D_2O) δ 177.0, 175.8, 172.8, 169.5, 154.4, 130.3, 128.2, 115.4, 59.4, 54.3, 53.4, 46.4, 36.0, 29.7, 29.5, 26.0, 23.6. **HR-MS** (ESI) m/z: calc. for $C_{19}H_{27}N_4O_6$ 407.1925 $(M+H)^+$, found 407.1925.

H-D-Pro-D-Glu-D-Tyr-NH₂ · TFA (UTS-133):

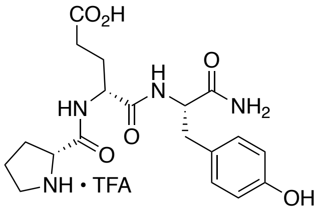

The peptide was synthesized according to the general procedure B for solid phase peptide synthesis.
¹H-NMR (500 MHz, D_2O) δ 7.21 – 7.15 (m, 2H), 6.89 – 6.81 (m, 2H), 4.58 (ddd, *J* = 31.1, 9.0, 6.4 Hz, 1H), 4.37 (dddd, *J* = 31.4, 19.8, 8.4, 6.2 Hz, 3H), 3.49 – 3.33 (m, 3H), 3.11 (ddd, *J* = 25.7, 14.0, 6.4 Hz, 1H), 3.03 – 2.86 (m, 1H), 2.55 – 2.43 (m, 1H), 2.43 – 2.33 (m, 2H), 2.33 – 2.22 (m, 1H), 2.19 – 1.82 (m, 7H). **¹³C-NMR** (126 MHz, D_2O) δ 175.5, 172.5, 169.4, 154.4, 130.5, 128.2, 115.4, 115.3, 59.6, 59.4, 54.4, 53.3, 46.5, 46.5, 36.2, 29.7, 29.7, 26.3, 23.7, 23.7. **HR-MS** (ESI) m/z: calc. for $C_{19}H_{27}N_4O_6$ 407.1925 $(M+H)^+$, found 407.1923.

H-D-Pro-D-Glu-CyLeu-NH₂ · TFA (UTS-134):

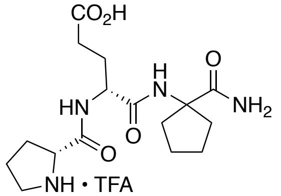

The peptide was synthesized according to the general procedure B for solid phase peptide synthesis.
¹H-NMR (500 MHz, D_2O) δ 4.45 – 4.39 (m, 1H), 4.37 (dd, *J* = 8.4, 6.2 Hz, 1H), 3.48 – 3.35 (m, 2H), 2.53 – 2.43 (m, 3H), 2.26 – 2.16 (m, 1H), 2.15 – 2.00 (m, 6H), 2.00 – 1.91 (m, 2H), 1.84 – 1.68 (m, 4H). **¹³C-NMR** (126 MHz, D_2O) δ 179.2, 177.0, 172.6, 169.7, 67.0, 59.5, 53.3, 46.5, 37.3, 35.7, 30.0, 29.8, 25.9, 23.9, 23.8, 23.6. **HR-MS** (ESI) m/z: calc. for $C_{16}H_{27}N_4O_5$ 355.1976 $(M+H)^+$, found 355.1976.

H-D-Pro-D-Glu-Abz-NH₂ · TFA (UTS-135):

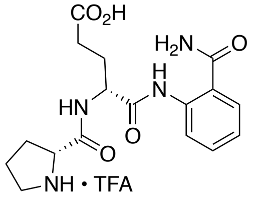

The peptide was synthesized according to the general procedure C for solid phase peptide synthesis.
¹H-NMR (500 MHz, D_2O) δ 7.75 – 7.71 (m, 1H), 7.71 – 7.66 (m, 1H), 7.62 – 7.57 (m, 1H), 7.40 – 7.35 (m, 1H), 4.49 (ddd, *J* = 23.0, 8.7, 6.2 Hz, 2H), 3.50 – 3.36 (m, 2H), 2.58 – 2.44 (m, 3H), 2.26 (dq, *J* = 13.7, 7.4 Hz, 1H), 2.20 – 1.98 (m, 4H). **¹³C-NMR** (126 MHz, D_2O) δ 177.5, 172.9, 171.7, 169.9, 132.3, 128.5, 126.2, 124.3, 59.6, 54.3, 46.5, 30.5, 29.6, 26.1, 23.7. **HR-MS** (ESI) m/z: calc. for $C_{17}H_{23}N_4O_5$ 363.1663 $(M+H)^+$, found 363.1661.

H-D-Pro-D-Glu-D-Ind-NH₂ · TFA (UTS-136):

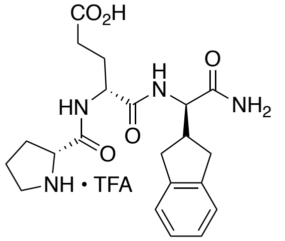

The peptide was synthesized according to the general procedure B for solid phase peptide synthesis.
^{1}H-NMR (400 MHz, D_2O) δ 7.34 – 7.27 (m, 2H), 7.27 – 7.19 (m, 2H), 4.42 (td, *J* = 8.0, 5.6 Hz, 3H), 3.49 – 3.33 (m, 2H), 3.19 – 3.03 (m, 2H), 2.97 – 2.73 (m, 3H), 2.55 – 2.37 (m, 3H), 2.17 – 1.91 (m, 5H). **^{13}C-NMR** (101 MHz, D_2O) δ 177.2, 175.4, 172.8, 169.5, 142.3, 142.0, 126.7, 124.5, 124.5, 59.5, 56.6, 53.2, 46.5, 41.1, 35.2, 34.9, 30.1, 29.8, 26.0, 23.7. **HR-MS** (ESI) m/z: calc. for $C_{21}H_{29}N_4O_5$ 417.2132 $(M+H)^+$, found 417.2132.

H-D-Pro-L-Tyr-D-Glu-NH₂ · TFA (UTS-137):

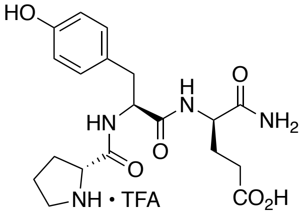

The peptide was synthesized according to the general procedure B for solid phase peptide synthesis.
^{1}H-NMR (400 MHz, D_2O) δ 7.21 – 7.11 (m, 2H), 6.90 – 6.80 (m, 2H), 4.61 (t, *J* = 8.2 Hz, 1H), 4.37 (dd, *J* = 8.7, 6.6 Hz, 1H), 4.25 – 4.17 (m, 1H), 3.45 – 3.29 (m, 2H), 3.07 – 2.98 (m, 2H), 2.42 – 2.29 (m, 1H), 2.12 – 1.96 (m, 4H), 1.92 (dt, *J* = 13.6, 7.0 Hz, 1H), 1.88 – 1.71 (m, 3H). **^{13}C-NMR** (101 MHz, D_2O) δ 177.4, 175.9, 173.2, 169.5, 154.7, 130.5, 127.6, 115.6, 59.6, 55.7, 52.9, 46.4, 36.3, 30.0, 29.8, 25.9, 23.6. **HR-MS** (ESI) m/z: calc. for $C_{19}H_{27}N_4O_6$ 407.1925 $(M+H)^+$, found 407.1924.

H-D-Pro-D-Tyr-D-Glu-NH₂ · TFA (UTS-138):

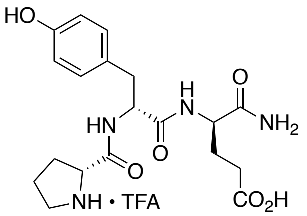

The peptide was synthesized according to the general procedure B for solid phase peptide synthesis.
^{1}H-NMR (400 MHz, D_2O) δ 7.20 – 7.14 (m, 2H), 6.90 – 6.84 (m, 2H), 4.61 – 4.52 (m, 1H), 4.38 (dd, *J* = 8.3, 5.7 Hz, 1H), 4.30 (dd, *J* = 9.5, 5.0 Hz, 1H), 3.41 (qt, *J* = 11.8, 5.5 Hz, 2H), 3.03 (qd, *J* = 13.7, 8.1 Hz, 2H), 2.53 – 2.42 (m, 1H), 2.38 (t, *J* = 7.3 Hz, 2H), 2.15 – 1.96 (m, 4H), 1.87 (ddd, *J* = 14.2, 9.6, 7.0 Hz, 1H). **^{13}C-NMR** (101 MHz, D_2O) δ 177.4, 175.1, 172.7, 169.3, 154.6, 130.5, 127.7, 115.6, 59.5, 55.8, 52.4, 46.5, 35.9, 29.9, 29.7, 26.2, 23,7. **HR-MS** (ESI) m/z: calc. for $C_{19}H_{27}N_4O_6$ 407.1925 $(M+H)^+$, found 407.1925.

H-D-Pro-CyLeu-D-Glu-NH₂ · TFA (UTS-139):

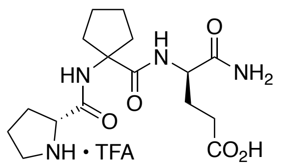

The peptide was synthesized according to the general procedure B for solid phase peptide synthesis.
^{1}H-NMR (400 MHz, D_2O) δ 4.39 (ddd, *J* = 18.3, 9.2, 5.5 Hz, 2H), 3.50 – 3.35 (m, 2H), 2.48 (t, *J* = 7.0 Hz, 3H), 2.26 – 1.88 (m, 9H), 1.78 (s, 4H). **^{13}C-NMR** (101 MHz, D_2O) δ 176.0, 176.0, 169.3, 67.4, 59.7, 53.2, 46.6, 36.5, 35.8, 30.4, 29.7, 25.8, 23.7, 23.7, 23.5. **HR-MS** (ESI) m/z: calc. for $C_{16}H_{27}N_4O_5$ 355.1976 $(M+H)^+$, found 355.1974.

H-D-Pro-Abz-D-Glu-NH₂ · TFA (UTS-140):

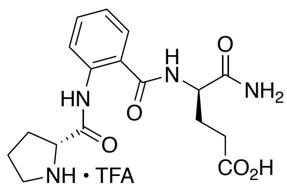

The peptide was synthesized according to the general procedure C for solid phase peptide synthesis.
^{1}H-NMR (400 MHz, D_2O) δ 7.74 – 7.58 (m, 3H), 7.58 – 7.48 (m, 1H), 7.48 – 7.39 (m, 1H), 7.20 – 7.12 (m, 1H), 4.64 – 4.46 (m, 3H), 3.56 – 3.38 (m, 2H), 2.58 (tt, *J* = 9.6, 5.1 Hz, 4H), 2.34 – 2.02 (m, 6H). **^{13}C-NMR** (101 MHz, D_2O) δ 177.4, 177.4, 175.9, 170.4, 168.4, 133.2, 132.2, 128.7, 127.0, 125.41, 120.5, 60.2, 53.1, 46.5, 30.5, 29.4, 26.3, 23.8. **HR-MS** (ESI) m/z: calc. for $C_{17}H_{23}N_4O_5$ 363.1663 $(M+H)^+$, found 363.1661.

H-D-Pro-D-Ind-D-Glu-NH2 · TFA (UTS-141):

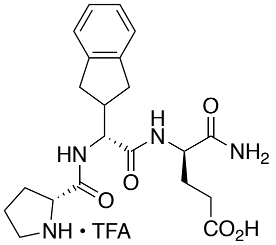

The peptide was synthesized according to the general procedure B for solid phase peptide synthesis.
^{1}H-NMR (400 MHz, D_2O) δ 7.29 (p, *J* = 4.0 Hz, 2H), 7.24 (dt, *J* = 5.6, 3.3 Hz, 2H), 4.47 – 4.34 (m, 3H), 3.51 – 3.35 (m, 2H), 3.15 (dd, *J* = 15.2, 6.5 Hz, 1H), 3.04 (p, *J* = 5.9 Hz, 1H), 2.98 – 2.77 (m, 3H), 2.55 – 2.39 (m, 3H), 2.19 – 1.90 (m, 5H). **^{13}C-NMR** (101 MHz, D_2O) δ 177.1, 175.4, 172.8, 169.6, 142.1, 141.9, 126.7, 126.7, 124.6, 124.5, 59.4, 57.7, 52.5, 46.6, 40.7, 35.4, 34.9, 29.8, 26.3, 23.7. **HR-MS** (ESI) m/z: calc. for $C_{21}H_{29}N_4O_5$ 417.2132 $(M+H)^+$, found 417.2131.

H-D-Pro-L-Tyr-L-Tyr-NH2 · TFA (UTS-142):

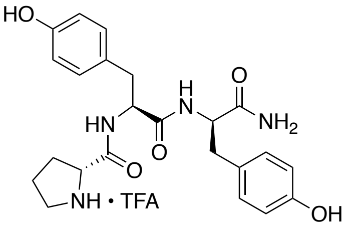

The peptide was synthesized according to the general procedure B for solid phase peptide synthesis.
^{1}H-NMR (400 MHz, D_2O) δ 7.16 (d, *J* = 8.4 Hz, 2H), 7.12 – 7.04 (m, 2H), 6.90 – 6.78 (m, 4H), 4.62 (dd, *J* = 9.6, 6.2 Hz, 1H), 4.55 (dd, *J* = 8.9, 6.2 Hz, 1H), 3.33 (tt, *J* = 7.3, 3.5 Hz, 2H), 3.07 (ddd, *J* = 25.3, 14.0, 6.2 Hz, 2H), 2.90 (dd, *J* = 14.0, 9.0 Hz, 1H), 2.78 (dd, *J* = 14.0, 9.7 Hz, 1H), 2.24 (dq, *J* = 13.8, 7.6 Hz, 1H), 1.96 (dp, *J* = 13.8, 7.0 Hz, 1H), 1.75 (dp, *J* = 14.3, 7.2 Hz, 1H), 1.58 (dq, *J* = 13.3, 6.6 Hz, 1H). **^{13}C-NMR** (101 MHz, D_2O) δ 175.3, 172.4, 169.2, 154.5, 154.4, 130.6, 130.5, 128.3, 128.0, 115.4, 115.4, 59.6, 54.9, 54.7, 46.5, 36.5, 36.2, 29.9, 23.4. **HR-MS** (ESI) m/z: calc. for $C_{23}H_{29}N_4O_5$ 441.2132 $(M+H)^+$, found 441.2132.

H-D-Pro-L-Tyr-D-Tyr-NH2 · TFA (UTS-143):

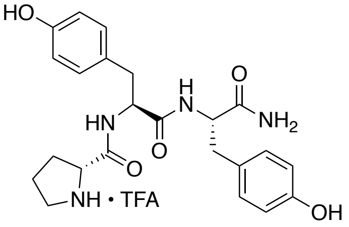

The peptide was synthesized according to the general procedure B for solid phase peptide synthesis.
^{1}H-NMR (400 MHz, D_2O) δ 7.11 – 7.02 (m, 2H), 6.94 – 6.87 (m, 2H), 6.85 – 6.71 (m, 4H), 4.65 – 4.55 (m, 1H), 4.55 – 4.45 (m, 1H), 4.26 (dd, *J* = 8.6, 6.5 Hz, 1H), 3.28 (tt, *J* = 11.6, 5.9 Hz, 2H), 3.05 (dd, *J* = 14.1, 5.6 Hz, 1H), 2.85 – 2.71 (m, 2H), 2.61 (dd, *J* = 14.0, 8.8 Hz, 1H), 2.28 – 2.14 (m, 1H), 1.93 (dp, *J* = 14.1, 7.1 Hz, 1H), 1.76 (dp, *J* = 14.2, 7.2 Hz, 1H), 1.60 (dq, *J* = 13.7, 6.8 Hz, 1H). **^{13}C-NMR** (101 MHz, D_2O) δ 175.8, 172.7, 169.2, 154.4, 154.4, 130.4, 130.4, 128.2, 127.6, 115.4, 115.3, 59.5, 54.7, 46.3, 36.5, 36.1, 29.8, 23.4. **HR-MS** (ESI) m/z: calc. for $C_{23}H_{29}N_4O_5$ 441.2132 $(M+H)^+$, found 441.2132.

H-D-Pro-L-Tyr-CyLeu-NH2 · TFA (UTS-144):

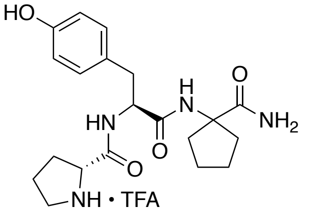

The peptide was synthesized according to the general procedure B for solid phase peptide synthesis.
^{1}H-NMR (500 MHz, D_2O) δ 7.20 – 7.14 (m, 2H), 6.90 – 6.85 (m, 2H), 4.56 (dd, *J* = 8.9, 7.7 Hz, 1H), 4.37 (dd, *J* = 8.6, 6.6 Hz, 1H), 3.47 – 3.30 (m, 2H), 3.09 – 2.91 (m, 2H), 2.37 (dq, *J* = 14.6, 7.3 Hz, 1H), 2.23 – 2.10 (m, 1H), 2.03 (dq, *J* = 13.1, 6.6 Hz, 1H), 1.98 – 1.75 (m, 4H), 1.73 – 1.53 (m, 3H), 1.39 (d, *J* = 12.0 Hz, 2H). **^{13}C-NMR** (126 MHz, D_2O) δ 179.3, 172.8, 169.5, 154.6, 130.6, 127.4, 115.5, 66.8, 59.5, 55.4, 46.4, 38.2, 36.2, 35.1, 29.8, 24.1, 23.6. **HR-MS** (ESI) m/z: calc. for $C_{20}H_{28}N_4O_4Na$ 411.2003 $(M+Na)^+$, found 411.2002.

H-D-Pro-L-Tyr-Abz-NH$_2$ · TFA (UTS-145):

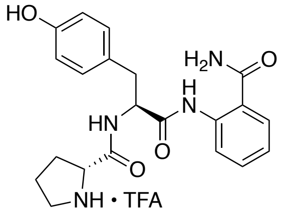

The peptide was synthesized according to the general procedure C for solid phase peptide synthesis.
^{1}H-NMR (500 MHz, D_2O) δ 7.74 (dd, J = 8.2, 1.2 Hz, 1H), 7.69 (dd, J = 7.8, 1.5 Hz, 1H), 7.60 (ddd, J = 8.2, 7.5, 1.6 Hz, 1H), 7.36 (td, J = 7.6, 1.2 Hz, 1H), 7.23 – 7.16 (m, 2H), 6.88 – 6.83 (m, 2H), 4.41 (dd, J = 8.6, 6.4 Hz, 1H), 3.40 – 3.32 (m, 2H), 3.28 (dd, J = 14.0, 6.2 Hz, 1H), 2.98 (dd, J = 14.0, 9.7 Hz, 1H), 2.30 (ddt, J = 13.6, 8.6, 7.1 Hz, 1H), 2.04 – 1.94 (m, 1H), 1.80 (dq, J = 14.3, 7.2 Hz, 1H), 1.65 (dq, J = 13.7, 6.8 Hz, 1H). **^{13}C-NMR** (126 MHz, D_2O) δ 172.8, 171.5, 169.7, 154.5, 134.5, 132.4, 130.5, 128.6, 128.1, 126.0, 125.8, 123.8, 115.5, 59.7, 56.0, 46.4, 36.2, 29.8, 23.5. **HR-MS** (ESI) m/z: calc. for $C_{21}H_{25}N_4O_4$ 397.1870 $(M+H)^+$, found 397.1875.

H-D-Pro-L-Tyr-D-Ind-NH$_2$ · TFA (UTS-146):

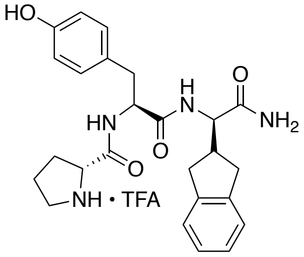

The peptide was synthesized according to the general procedure B for solid phase peptide synthesis.
^{1}H-NMR (400 MHz, D_2O) δ 7.19 (dp, J = 13.4, 4.6 Hz, 4H), 7.09 (d, J = 8.4 Hz, 2H), 6.80 (d, J = 8.4 Hz, 2H), 4.59 (dd, J = 9.3, 7.3 Hz, 1H), 4.32 (dd, J = 8.7, 6.4 Hz, 1H), 4.13 (d, J = 7.4 Hz, 1H), 3.43 – 3.25 (m, 2H), 2.98 – 2.88 (m, 2H), 2.89 – 2.76 (m, 2H), 2.76 – 2.63 (m, 2H), 2.47 (dd, J = 15.7, 7.3 Hz, 1H), 2.40 – 2.19 (m, 2H), 1.99 (td, J = 14.1, 7.5 Hz, 1H), 1.93 – 1.78 (m, 2H). **^{13}C-NMR** (101 MHz, D_2O) δ 175.8, 173.1, 169.2, 154.6, 142.3, 141.9, 130.5, 127.4, 126.6, 126.6, 124.5, 124.4, 115.5, 59.5, 56.9, 55.5, 46.4, 40.2, 36.4, 35.0, 34.6, 29.8, 23.6. **HR-MS** (ESI) m/z: calc. for $C_{25}H_{32}N_4O_4$ 451.2340 $(M+H)^+$, found 451.2337.

H-D-Pro-D-Tyr-L-Tyr-NH$_2$ · TFA (UTS-147):

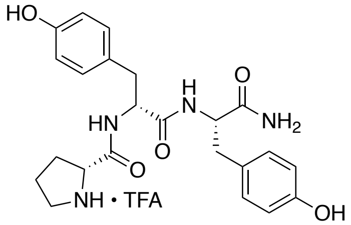

The peptide was synthesized according to the general procedure B for solid phase peptide synthesis.
^{1}H-NMR (400 MHz, D_2O) δ 7.03 (d, J = 8.5 Hz, 2H), 7.00 – 6.91 (m, 2H), 6.90 – 6.75 (m, 4H), 4.53 (t, J = 7.5 Hz, 1H), 4.47 (dd, J = 8.7, 5.7 Hz, 1H), 4.32 (dd, J = 8.6, 6.5 Hz, 1H), 3.46 – 3.29 (m, 2H), 2.99 (dd, J = 14.4, 5.9 Hz, 1H), 2.90 – 2.74 (m, 3H), 2.47 – 2.31 (m, 1H), 2.09 – 1.85 (m, 3H). **^{13}C-NMR** (101 MHz, D_2O) δ 175.7, 172.7, 169.3, 154.6, 154.4, 130.5, 130.4, 128.0, 127.5, 115.4, 59.4, 55.5, 54.6, 46.5, 36.5, 36.0, 29.7, 23.7. **HR-MS** (ESI) m/z: calc. for $C_{23}H_{29}N_4O_5$ 441.2132 $(M+H)^+$, found 441.2133.

H-D-Pro-CyLeu-L-Tyr-NH$_2$ · TFA (UTS-148):

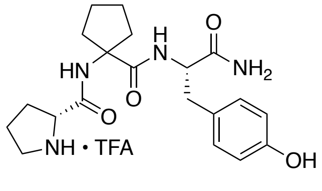

The peptide was synthesized according to the general procedure B for solid phase peptide synthesis.
^{1}H-NMR (400 MHz, D_2O) δ 7.21 – 7.09 (m, 2H), 6.92 – 6.77 (m, 2H), 4.67 (dd, J = 10.3, 5.1 Hz, 1H), 4.33 (dd, J = 8.6, 7.3 Hz, 1H), 3.44 – 3.29 (m, 2H), 3.22 (dd, J = 14.3, 5.2 Hz, 1H), 2.87 (dd, J = 14.4, 10.5 Hz, 1H), 2.44 – 2.30 (m, 1H), 2.00 (tp, J = 19.9, 6.4 Hz, 4H), 1.85 – 1.71 (m, 4H), 1.66 (t, J = 4.6 Hz, 4H). **^{13}C-NMR** (101 MHz, D_2O) δ 176.2, 175.7, 169.3, 154.3, 130.3, 128.5, 115.4, 67.2, 59.6, 54.3, 46.6, 36.4, 36.0, 35.6, 29.6, 23.8, 23.6, 23.6. **HR-MS** (ESI) m/z: calc. for $C_{20}H_{29}N_4O_4$ 389.2183 $(M+H)^+$, found 389.2181.

H-D-Pro-Abz-L-Tyr-NH$_2$ · TFA (UTS-149):

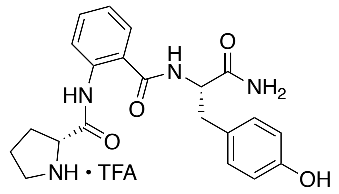

The peptide was synthesized according to the general procedure C for solid phase peptide synthesis.
^{1}H-NMR (400 MHz, D_2O) δ 7.67 – 7.50 (m, 1H), 7.41 (t, *J* = 8.0 Hz, 1H), 7.37 – 7.27 (m, 1H), 7.24 – 7.15 (m, 3H), 6.84 (dd, *J* = 8.2, 5.9 Hz, 2H), 4.70 (dd, *J* = 10.2, 5.4 Hz, 1H), 4.59 – 4.44 (m, 1H), 3.45 – 3.35 (m, 2H), 3.24 (ddd, *J* = 20.3, 14.0, 5.6 Hz, 1H), 2.95 (ddd, *J* = 29.0, 14.1, 9.8 Hz, 1H), 2.57 – 2.37 (m, 1H), 2.11 (ddtt, *J* = 33.2, 26.6, 13.3, 6.9 Hz, 3H). **^{13}C-NMR** (101 MHz, D_2O) δ 175.9, 170.2, 168.3, 154.4, 132.9, 132.1, 130.5, 128.6, 128.5, 128.3, 126.9, 125.0, 115.4, 60.0, 54.9, 46.5, 36.2, 29.3, 23.7. **HR-MS** (ESI) m/z: calc. for $C_{21}H_{25}N_4O_4$ 397.1870 $(M+H)^+$, found 397.1868

H-D-Pro-D-Ind-L-Tyr-NH$_2$ · TFA (UTS-150):

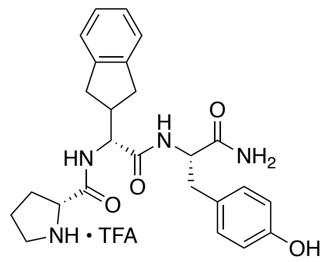

The peptide was synthesized according to the general procedure B for solid phase peptide synthesis.
^{1}H-NMR (400 MHz, D_2O) δ 7.27 – 7.19 (m, 5H), 7.18 (q, *J* = 4.4 Hz, 1H), 6.90 – 6.82 (m, 2H), 4.70 (dd, *J* = 11.9, 4.3 Hz, 1H), 4.39 (dd, *J* = 8.6, 6.0 Hz, 1H), 4.26 (d, *J* = 8.4 Hz, 1H), 3.47 – 3.28 (m, 3H), 2.86 – 2.71 (m, 2H), 2.62 – 2.48 (m, 2H), 2.48 – 2.37 (m, 1H), 2.33 (dd, *J* = 16.0, 7.4 Hz, 1H), 2.19 (dd, *J* = 16.0, 8.7 Hz, 1H), 2.11 – 1.92 (m, 3H). **^{13}C-NMR** (101 MHz, D_2O) δ 176.0, 173.2, 169.7, 154.5, 142.0, 141.9, 130.3, 128.6, 126.7, 126.6, 124.6, 124.3, 115.5, 59.4, 57.9, 54.6, 46.5, 40.7, 35.8, 35.1, 34.3, 29.8, 23.6. **HR-MS** (ESI) m/z: calc. for $C_{25}H_{31}N_4O_4$ 451.2340 $(M+H)^+$, found 451.2336.

H-D-Pro-D-Tyr-D-Tyr-NH$_2$ · TFA (UTS-151):

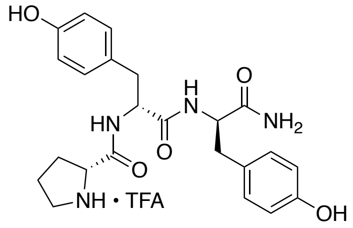

The peptide was synthesized according to the general procedure B for solid phase peptide synthesis.
^{1}H-NMR (400 MHz, D_2O) δ 7.18 – 7.00 (m, 2H), 6.86 – 6.70 (m, 2H), 4.53 – 4.43 (m, 1H), 4.32 – 4.19 (m, 1H), 3.35 (t, *J* = 7.2 Hz, 1H), 3.08 – 2.97 (m, 1H), 2.95 – 2.83 (m, 1H), 2.34 (ddt, *J* = 28.3, 15.0, 7.2 Hz, 1H), 1.95 (dh, *J* = 27.1, 7.0 Hz, 1H). **^{13}C-NMR** (101 MHz, D_2O) δ 175.0, 172.1, 168.8, 154.5, 130.5, 128.1, 115.2, 59.3, 55.4, 54.3, 46.5, 36.2, 29.8, 23.7. **HR-MS** (ESI) m/z: calc. for $C_{23}H_{29}N_4O_5$ 441.2132 $(M+H)^+$, found 441.2132.

H-D-Pro-D-Tyr-CyLeu-NH$_2$ · TFA (UTS-152):

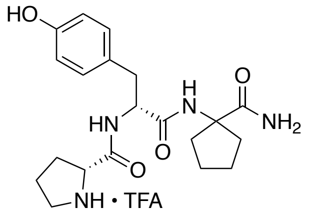

The peptide was synthesized according to the general procedure B for solid phase peptide synthesis.
^{1}H-NMR (400 MHz, D_2O) δ 7.13 (d, *J* = 8.5 Hz, 2H), 6.88 – 6.80 (m, 2H), 4.43 (dd, *J* = 10.6, 6.3 Hz, 1H), 4.37 (dd, *J* = 8.7, 5.9 Hz, 1H), 3.44 – 3.29 (m, 2H), 3.15 – 3.03 (m, 1H), 2.83 (dd, *J* = 13.3, 10.7 Hz, 1H), 2.42 (ddd, *J* = 10.4, 8.3, 5.1 Hz, 1H), 2.14 – 1.90 (m, 4H), 1.74 (t, *J* = 8.1 Hz, 2H), 1.54 (dtt, *J* = 22.2, 11.6, 6.1 Hz, 3H), 1.29 – 1.11 (m, 2H). **^{13}C-NMR** (101 MHz, D_2O) δ 179.3, 172.6, 169.6, 154.5, 130.6, 127.2, 115.5, 66.7, 59.4, 55.9, 46.4, 38.6, 35.7, 34.7, 29.8, 24.1, 24.1, 23.6. **HR-MS** (ESI) m/z: calc. for $C_{20}H_{29}N_4O_4$ 389.2183 $(M+H)^+$, found 389.2182.

H-D-Pro-D-Tyr-Abz-NH$_2$ · TFA (UTS-153):

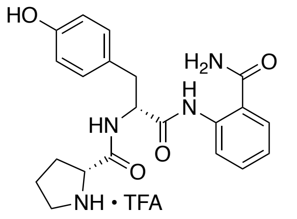

The peptide was synthesized according to the general procedure C for solid phase peptide synthesis.
^{1}H-NMR (400 MHz, D$_2$O) δ 7.75 – 7.67 (m, 1H), 7.67 – 7.59 (m, 1H), 7.56 (dddd, *J* = 8.2, 7.4, 1.6, 0.7 Hz, 1H), 7.36 – 7.27 (m, 1H), 7.21 – 7.12 (m, 2H), 6.87 – 6.78 (m, 2H), 4.61 (t, *J* = 7.7 Hz, 1H), 4.41 (dd, *J* = 8.7, 6.1 Hz, 1H), 3.43 (dtd, *J* = 23.7, 12.3, 6.2 Hz, 2H), 3.16 – 3.04 (m, 2H), 2.60 – 2.42 (m, 1H), 2.31 – 1.96 (m, 3H). **^{13}C-NMR** (101 MHz, D$_2$O) δ 172.5, 171.4, 169.6, 154.6, 134.5, 132.4, 130.5, 128.5, 127.6, 125.7, 125.1, 123.4, 115.6, 59.6, 57.1, 46.5, 35.9, 29.7, 23.7. **HR-MS** (ESI) m/z: calc. for $C_{21}H_{24}N_4O_4Na$ 419.1690 (M+Na)$^+$, found 419.1687.

H-D-Pro-D-Tyr-D-Ind-NH$_2$ · TFA (UTS-154):

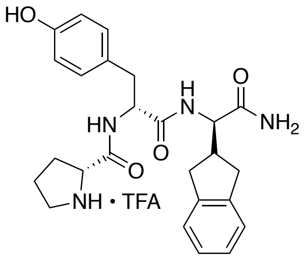

The peptide was synthesized according to the general procedure B for solid phase peptide synthesis.
^{1}H-NMR (400 MHz, D$_2$O) δ 7.32 – 7.18 (m, 4H), 7.18 – 7.11 (m, 2H), 6.90 – 6.81 (m, 2H), 4.58 (t, *J* = 7.9 Hz, 1H), 4.35 (dd, *J* = 13.8, 8.4 Hz, 2H), 3.39 (dp, *J* = 9.7, 4.5 Hz, 2H), 3.08 – 2.94 (m, 4H), 2.82 (h, *J* = 7.5 Hz, 1H), 2.76 – 2.63 (m, 2H), 2.44 (dt, *J* = 9.2, 6.3 Hz, 1H), 2.13 – 1.92 (m, 3H). **^{13}C-NMR** (101 MHz, D$_2$O) δ 174.8, 172.5, 169.2, 154.6, 142.3, 142.1, 130.5, 127.6, 126.7, 124.5, 115.5, 59.5, 56.3, 55.7, 46.5, 41.1, 35.9, 35.2, 34.7, 29.8, 23.7. **HR-MS** (ESI) m/z: calc. for $C_{25}H_{31}N_4O_4$ 451.2340 (M+H)$^+$, found 451.2338.

H-D-Pro-CyLeu-D-Tyr-NH$_2$ · TFA (UTS-155):

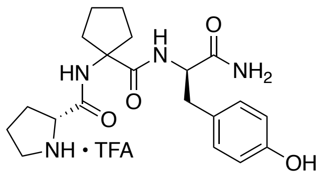

The peptide was synthesized according to the general procedure B for solid phase peptide synthesis.
^{1}H-NMR (400 MHz, D$_2$O) δ 7.21 – 7.10 (m, 2H), 6.89 – 6.80 (m, 2H), 4.62 (dd, *J* = 8.7, 5.9 Hz, 1H), 4.31 (dd, *J* = 8.7, 6.5 Hz, 1H), 3.43 – 3.34 (m, 2H), 3.14 (dd, *J* = 14.1, 5.9 Hz, 1H), 2.97 (dd, *J* = 14.1, 8.7 Hz, 1H), 2.46 – 2.32 (m, 1H), 2.11 – 1.85 (m, 7H), 1.85 – 1.73 (m, 2H), 1.67 (q, *J* = 4.5 Hz, 4H). **^{13}C-NMR** (101 MHz, D$_2$O) δ 175.8, 175.4, 169.2, 154.4, 130.6, 128.3, 115.4, 67.3, 59.5, 54.2, 46.6, 36.2, 35.8, 35.6, 29.7, 23.7, 23.5, 23.3. **HR-MS** (ESI) m/z: calc. for $C_{20}H_{28}N_4O_4Na$ 411.2003 (M+Na)$^+$, found 411.2002.

H-D-Pro-Abz-D-Tyr-NH$_2$ · TFA (UTS-156):

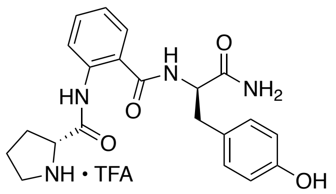

The peptide was synthesized according to the general procedure C for solid phase peptide synthesis.
^{1}H-NMR (500 MHz, D$_2$O) δ 7.72 – 7.50 (m, 2H), 7.45 – 7.34 (m, 2H), 7.31 – 7.18 (m, 2H), 6.95 – 6.82 (m, 2H), 4.71 (dd, *J* = 9.6, 5.6 Hz, 1H), 4.62 – 4.44 (m, 1H), 3.54 – 3.38 (m, 2H), 3.23 (dd, *J* = 14.0, 5.5 Hz, 1H), 2.99 (dd, *J* = 14.0, 9.7 Hz, 1H), 2.61 – 2.42 (m, 1H), 2.30 – 2.00 (m, 3H). **^{13}C-NMR** (126 MHz, D$_2$O) δ 175.8, 154.4, 132.1, 130.6, 130.5, 128.8, 128.3, 126.9, 125.4, 115.4, 60.1, 55.2, 46.4, 36.3, 29.3, 23.8. **HR-MS** (ESI) m/z: calc. for $C_{21}H_{25}N_4O_4$ 397.1870 (M+H)$^+$, found 397.1868.

H-D-Pro-D-Ind-D-Tyr-NH$_2$ · TFA (UTS-157):

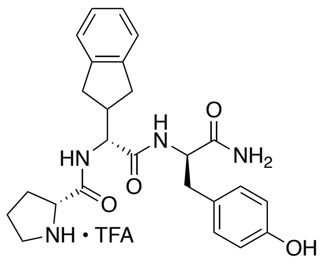

The peptide was synthesized according to the general procedure B for solid phase peptide synthesis.
^{1}H-NMR (400 MHz, D_2O) δ 7.33 – 7.20 (m, 4H), 7.20 – 7.11 (m, 2H), 6.87 – 6.74 (m, 2H), 4.63 (dd, *J* = 9.9, 5.6 Hz, 1H), 4.35 (dd, *J* = 8.6, 6.7 Hz, 1H), 4.29 (d, *J* = 7.7 Hz, 1H), 3.41 (td, *J* = 7.2, 2.6 Hz, 2H), 3.18 – 2.98 (m, 2H), 2.90 (dd, *J* = 14.1, 9.9 Hz, 1H), 2.81 – 2.60 (m, 4H), 2.39 (ddd, *J* = 15.7, 13.3, 7.2 Hz, 1H), 2.12 – 1.93 (m, 2H), 1.86 (dq, *J* = 14.0, 7.0 Hz, 1H). **^{13}C-NMR** (101 MHz, D_2O) δ 175.4, 172.4, 154.4, 142.2, 141.9, 130.5, 126.7, 124.5, 115.3, 59.4, 57.9, 54.3, 46.6, 41.1, 36.2, 35.4, 34.8, 29.9, 23.7. **HR-MS** (ESI) m/z: calc. for $C_{25}H_{31}N_4O_4$ 451.2340 $(M+H)^+$, found 451.2340.

H-D-Pro-CyLeu-CyLeu-NH$_2$ · TFA (UTS-158):

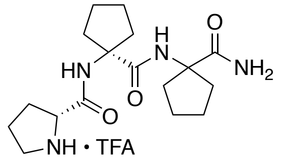

The peptide was synthesized according to the general procedure B for solid phase peptide synthesis.
^{1}H-NMR (400 MHz, D_2O) δ 4.40 (dd, *J* = 8.2, 5.9 Hz, 1H), 3.52 – 3.30 (m, 2H), 2.56 – 2.39 (m, 1H), 2.26 – 2.01 (m, 7H), 1.89 (t, *J* = 14.6 Hz, 4H), 1.84 – 1.61 (m, 8H). **^{13}C-NMR** (101 MHz, D_2O) δ 179.8, 175.4, 169.4, 67.2, 66.9, 59.6, 46.6, 37.0, 36.4, 36.0, 35.7, 29.7, 29.7, 24.2, 23.7, 23.6, 23.4. **HR-MS** (ESI) m/z: calc. for $C_{17}H_{28}N_4O_3Na$ 359.2054 $(M+Na)^+$, found 359.2053.

H-D-Pro-CyLeu-D-Ind-NH$_2$ · TFA (UTS-159):

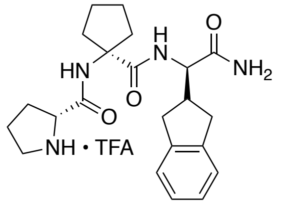

The peptide was synthesized according to the general procedure B for solid phase peptide synthesis.
^{1}H-NMR (400 MHz, D_2O) δ 7.30 (dt, *J* = 7.5, 3.7 Hz, 2H), 7.28 – 7.16 (m, 2H), 4.46 (d, *J* = 8.0 Hz, 1H), 4.34 (dd, *J* = 8.5, 5.9 Hz, 1H), 3.41 (q, *J* = 6.6 Hz, 2H), 3.11 (ddd, *J* = 16.0, 11.5, 7.9 Hz, 2H), 3.00 – 2.88 (m, 1H), 2.78 (ddd, *J* = 22.0, 15.9, 6.5 Hz, 2H), 2.45 (dt, *J* = 8.6, 6.3 Hz, 1H), 2.20 – 1.94 (m, 5H), 1.94 – 1.79 (m, 2H), 1.72 (d, *J* = 7.5 Hz, 4H). **^{13}C-NMR** (101 MHz, D_2O) δ 175.7, 175.6, 169.1, 142.5, 142.0, 126.8, 126.7, 124.6, 67.3, 59.5, 57.1, 46.6, 40.6, 36.1, 36.0, 35.6, 34.9, 29.8, 23.7, 23.6, 23.5. **HR-MS** (ESI) m/z: calc. for $C_{22}H_{31}N_4O_3$ 399.2391 $(M+H)^+$, found 399.2388.

H-D-Pro-D-Ind-CyLeu-NH$_2$ · TFA (UTS-160):

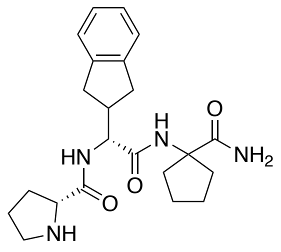

The peptide was synthesized according to the general procedure B for solid phase peptide synthesis.
^{1}H-NMR (500 MHz, D_2O) δ 7.30 (dq, *J* = 8.3, 3.9 Hz, 2H), 7.27 – 7.21 (m, 2H), 4.42 (dd, *J* = 8.6, 6.0 Hz, 1H), 4.34 (d, *J* = 7.8 Hz, 1H), 3.49 – 3.36 (m, 2H), 3.18 (td, *J* = 9.9, 4.4 Hz, 1H), 3.10 – 3.00 (m, 1H), 2.92 – 2.80 (m, 3H), 2.52 – 2.41 (m, 1H), 2.28 – 2.18 (m, 1H), 2.13 – 1.90 (m, 5H), 1.87 – 1.70 (m, 5H). **^{13}C-NMR** (126 MHz, D_2O) δ 179.2, 172.5, 169.8, 142.0, 141.9, 126.8, 126.8, 124.6, 124.5, 67.0, 59.4, 57.9, 46.5, 40.5, 37.9, 35.6, 35.1, 34.8, 29.9, 24.1, 24.0, 23.6. **HR-MS** (ESI) m/z: calc. for $C_{22}H_{31}N_4O_3$ 399.2391 $(M+H)^+$, found 399.2389.

H-D-Pro-D-Ind-D-Ind-NH$_2$ · TFA (UTS-161):

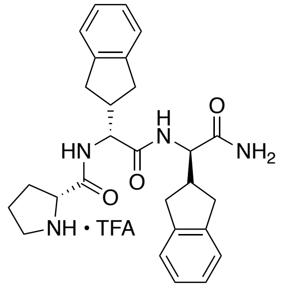

The peptide was synthesized according to the general procedure B for solid phase peptide synthesis.
^{1}H-NMR (500 MHz, D_2O) δ 7.29 (s, 2H), 7.23 (td, *J* = 5.4, 3.1 Hz, 2H), 4.46 – 4.35 (m, 1H), 3.41 (h, *J* = 4.8 Hz, 1H), 3.18 – 2.98 (m, 2H), 2.94 – 2.73 (m, 3H), 2.12 – 1.95 (m, 2H). **^{13}C-NMR** (126 MHz, D_2O) δ 175.2, 172.7, 169.5, 142.3, 142.1, 142.0, 141.9, 126.7, 124.5, 59.4, 57.7, 56.5, 46.6, 41.1, 40.7, 35.4, 35.2, 35.0, 34.9, 29.9, 23.7. **HR-MS** (ESI) m/z: calc. for $C_{27}H_{33}N_4O_3$ 461.2547 $(M+H)^+$, found 461.2546.

Analytical Data of Predicted Peptides for the Annulation Reaction

H-D-Pro-D-Pyr-4-MePhe-NH_2 · TFA (P(1)-1):

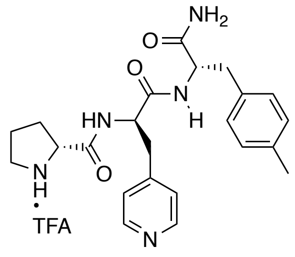

The peptide was synthesized according to the general procedure B for solid phase peptide synthesis.
^{1}H-NMR (500 MHz, D_2O) δ 8.46 (s, 2H), 7.34 (d, *J* = 5.5 Hz, 2H), 7.21 – 7.15 (m, 2H), 7.10 (d, *J* = 7.9 Hz, 2H), 4.76 – 4.69 (m, 1H), 4.56 (dd, *J* = 10.2, 4.9 Hz, 1H), 4.33 (dd, *J* = 8.7, 6.4 Hz, 1H), 3.44 – 3.33 (m, 2H), 3.16 (dd, *J* = 14.4, 4.9 Hz, 1H), 3.01 (d, *J* = 7.4 Hz, 2H), 2.82 (dd, *J* = 14.3, 10.2 Hz, 1H), 2.46 – 2.35 (m, 1H), 2.31 (s, 3H), 2.04 (dt, *J* = 13.6, 6.7 Hz, 1H), 2.00 – 1.89 (m, 2H). **^{13}C-NMR** (126 MHz, D_2O) δ 175.7, 171.6, 169.4, 145.2, 137.1, 133.3, 129.4, 128.8, 126.0, 59.4, 54.6, 53.8, 46.5, 36.4, 29.7, 23.6, 20.1. **HR-MS** (ESI) m/z: calc. for $C_{23}H_{30}N_5O_3$ 424.2343 $(M+H)^+$, found 424.2339.

H-D-Pro-L-Aze-L-Naph-NH_2 · TFA (P(1)-2):

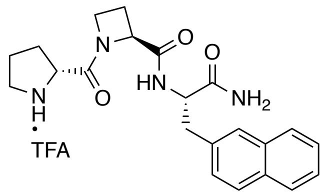

The peptide was synthesized according to the general procedure B for solid phase peptide synthesis.
^{1}H-NMR (400 MHz, D_2O) δ 8.22 (dd, *J* = 14.5, 8.5 Hz, 1H), 8.00 (d, *J* = 8.1 Hz, 1H), 7.91 (d, *J* = 7.8 Hz, 1H), 7.73 – 7.57 (m, 2H), 7.57 – 7.43 (m, 2H), 4.99 – 4.84 (m, 2H), 4.35 (dd, *J* = 8.8, 7.1 Hz, 1H), 4.20 – 4.02 (m, 1H), 4.02 – 3.76 (m, 1H), 3.71 (dd, *J* = 14.1, 6.9 Hz, 1H), 3.59 – 3.47 (m, 1H), 3.47 – 3.36 (m, 2H), 3.25 (ddt, *J* = 60.0, 11.5, 7.4 Hz, 1H), 2.76 – 2.36 (m, 2H), 2.14 – 1.89 (m, 2H), 1.84 – 1.58 (m, 1H). **^{13}C-NMR** (101 MHz, D_2O) δ 175.3, 171.6, 169.1, 133.5, 132.5, 131.6, 128.9, 128.0, 127.9, 126.6, 126.1, 125.7, 123.4, 61.1, 57.3, 53.7, 48.9, 46.6, 34.0, 28.0, 24.0, 20.4. **HR-MS** (ESI) m/z: calc. for $C_{22}H_{27}N_4O_3$ 395.2078 $(M+H)^+$, found 395.2073.

H-D-Pro-D-cisHyd-D-Pyr-NH_2 · TFA (P(1)-3):

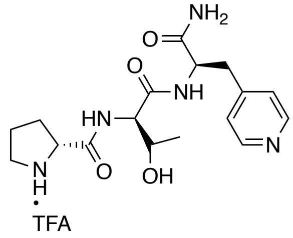

The peptide was synthesized according to the general procedure B for solid phase peptide synthesis.
^{1}H-NMR (400 MHz, D_2O) δ 8.58 (d, *J* = 5.8 Hz, 2H), 7.74 (d, *J* = 5.7 Hz, 2H), 4.41 (dd, *J* = 8.6, 6.3 Hz, 1H), 4.23 (d, *J* = 5.4 Hz, 1H), 4.08 (p, *J* = 6.2 Hz, 1H), 3.46 – 3.31 (m, 3H), 3.21 (dd, *J* = 14.0, 9.2 Hz, 1H), 2.45 – 2.33 (m, 1H), 2.04 (dt, *J* = 13.6, 6.8 Hz, 1H), 2.00 – 1.83 (m, 2H), 1.13 (d, *J* = 6.4 Hz, 3H). **^{13}C-NMR** (101 MHz, D_2O) δ 173.9, 171.1, 169.8, 161.1, 143.2, 133.9, 127.0, 66.9, 59.4, 59.4, 53.0, 46.5, 36.9, 29.8, 23.6, 18.7. **HR-MS** (ESI) m/z: calc. for $C_{17}H_{26}N_5O_4$ 364.1979 $(M+H)^+$, found 364.1979.

H-D-Pro-L-αMePro-L-HomoGlu-NH_2 · TFA (P(1)-4):

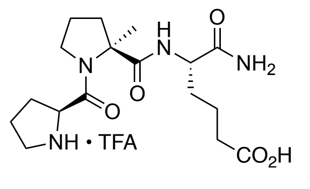

The peptide was synthesized according to the general procedure B for solid phase peptide synthesis.
^{1}H-NMR (500 MHz, D_2O) δ 4.56 (dd, *J* = 8.7, 6.2 Hz, 1H), 4.30 (dd, *J* = 9.0, 5.3 Hz, 1H), 3.82 – 3.73 (m, 1H), 3.67 (q, *J* = 8.5 Hz, 1H), 3.38 (qt, *J* = 11.6, 7.1 Hz, 2H), 2.53 (dq, *J* = 14.2, 5.4 Hz, 1H), 2.44 – 2.35 (m, 2H), 2.18 – 2.09 (m, 2H), 2.09 – 1.98 (m, 5H), 1.89 – 1.73 (m, 2H), 1.71 – 1.59 (m, 2H), 1.57 (s, 3H). **^{13}C-NMR** (126 MHz, D_2O) δ 178.5, 176.4, 175.8, 167.4, 68.2, 59.5, 53.5, 48.3, 46.6, 38.5, 33.3, 30.1, 28.0, 23.9, 23.4, 20.8, 19.7. **HR-MS** (ESI) m/z: calc. for $C_{17}H_{29}N_4O_5$ 369.2132 $(M+H)^+$, found 369.2133.

H-D-Pro-D-Pro-L-Styr-NH_2 · TFA (P(1)-5):

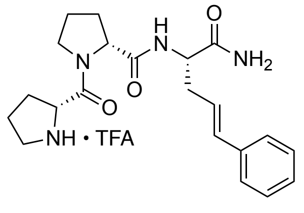

The peptide was synthesized according to the general procedure B for solid phase peptide synthesis.
^{1}H-NMR (400 MHz, D_2O) δ 7.50 – 7.43 (m, 2H), 7.40 (dd, *J* = 8.3, 6.9 Hz, 2H), 7.36 – 7.28 (m, 1H), 6.56 (d, *J* = 15.7 Hz, 1H), 6.22 (ddd, *J* = 16.2, 8.3, 6.1 Hz, 1H), 4.58 (td, *J* = 10.2, 5.4 Hz, 2H), 4.49 (dd, *J* = 8.4, 5.9 Hz, 1H), 3.66 (dt, *J* = 10.5, 6.3 Hz, 1H), 3.53 (dt, *J* = 10.3, 7.1 Hz, 1H), 3.40 – 3.24 (m, 2H), 2.86 (dddd, *J* = 14.5, 6.1, 4.4, 1.5 Hz, 1H), 2.64 – 2.47 (m, 2H), 2.22 (ddd, *J* = 14.9, 12.2, 6.5 Hz, 1H), 2.02 (dddd, *J* = 18.9, 16.8, 8.6, 5.5 Hz, 3H), 1.93 – 1.74 (m, 3H). **^{13}C-NMR** (101 MHz, D_2O) δ 175.8, 173.6, 168.1, 136.8, 133.4, 128.9, 127.8, 126.2, 124.8, 60.7, 59.0, 52.7, 47.6, 46.6, 35.0, 29.6, 28.4, 24.4, 23.9. **HR-MS** (ESI) m/z: calc. for $C_{21}H_{29}N_4O_3$ 385.2234 $(M+H)^+$, found 385.2235.

H-D-Pro-HomoPip-D-Arg-NH_2 · TFA (P(1)-6):

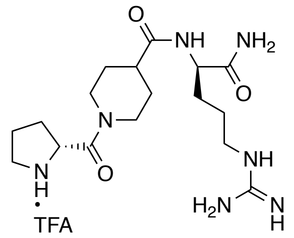

The peptide was synthesized according to the general procedure B for solid phase peptide synthesis.
^{1}H-NMR (400 MHz, D_2O) δ 4.76 – 4.69 (m, 1H), 4.40 (d, *J* = 13.6 Hz, 1H), 4.28 (dt, *J* = 8.7, 5.5 Hz, 1H), 3.90 (d, *J* = 13.7 Hz, 1H), 3.54 – 3.34 (m, 2H), 3.34 – 3.26 (m, 1H), 3.23 (t, *J* = 6.8 Hz, 2H), 2.96 – 2.84 (m, 1H), 2.72 (ddt, *J* = 11.6, 7.8, 3.7 Hz, 1H), 2.66 – 2.46 (m, 1H), 2.10 (h, *J* = 5.8 Hz, 2H), 2.05 – 1.81 (m, 5H), 1.81 – 1.45 (m, 5H). **^{13}C-NMR** (101 MHz, D_2O) δ 177.6, 177.6, 176.6, 167.5, 156.8, 58.5, 53.2, 53.1, 46.5, 46.5, 44.9, 44.7, 42.3, 41.6, 40.4, 29.3, 28.7, 28.2, 28.1, 28.1, 27.9, 27.7, 27.4, 24.4, 24.0, 23.9. **HR-MS** (ESI) m/z: calc. for $C_{17}H_{32}N_7O_3$ 382.2561 $(M+H)^+$, found 382.2561.

H-D-Pro-L-(4*S*)-Azp-L-His-NH_2 · TFA (P(1)-7):

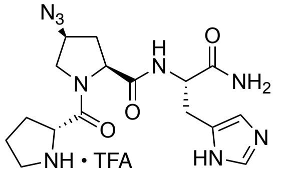

The peptide was synthesized according to the general procedure B for solid phase peptide synthesis.
^{1}H-NMR (400 MHz, D_2O) δ 8.64 – 8.55 (m, 1H), 7.35 (s, 1H), 4.70 – 4.58 (m, 2H), 4.53 (ddt, *J* = 7.2, 4.9, 2.0 Hz, 1H), 3.86 (dd, *J* = 11.5, 5.1 Hz, 1H), 3.75 (dt, *J* = 11.5, 1.7 Hz, 1H), 3.53 – 3.40 (m, 2H), 3.40 – 3.26 (m, 1H), 3.17 (ddd, *J* = 24.4, 15.4, 9.2 Hz, 1H), 2.66 – 2.49 (m, 2H), 2.19 (dq, *J* = 14.4, 2.0 Hz, 1H), 2.11 (dq, *J* = 13.5, 7.0 Hz, 2H), 2.05 – 1.94 (m, 2H). **^{13}C-NMR** (101 MHz, D_2O) δ 174.1, 172.6, 169.1, 133.6, 128.8, 117.8, 117.1, 59.8, 59.5, 59.4, 52.3, 46.7, 34.2, 27.8, 26.2, 24.0. **HR-MS** (ESI) m/z: calc. for $C_{16}H_{24}N_9O_3$ 390.1997 $(M+H)^+$, found 390.1998.

H-D-Pro-L-Oic-L-Glu-NH_2 · TFA (P(2)-2):

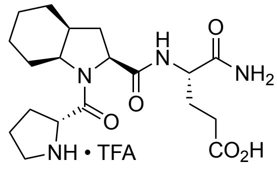

The peptide was synthesized according to the general procedure B for solid phase peptide synthesis.
^{1}H-NMR (400 MHz, D_2O) δ 4.56 (dd, *J* = 8.7, 6.9 Hz, 1H), 4.47 (dd, *J* = 10.2, 8.2 Hz, 1H), 4.38 (dd, *J* = 9.3, 5.2 Hz, 1H), 3.95 (dt, *J* = 11.9, 6.1 Hz, 1H), 3.49 (dt, *J* = 11.5, 7.0 Hz, 1H), 3.40 (dt, *J* = 11.5, 7.1 Hz, 1H), 2.65 – 2.45 (m, 4H), 2.27 (ddd, *J* = 12.5, 8.2, 6.6 Hz, 1H), 2.23 – 1.87 (m, 8H), 1.85 – 1.61 (m, 3H), 1.61 – 1.43 (m, 2H), 1.39 – 1.13 (m, 2H). **^{13}C-NMR** (101 MHz, D_2O) δ 177.3, 175.9, 173.9, 167.9, 60.8, 58.8, 58.5, 52.9, 46.8, 36.9, 30.3, 30.1, 29.0, 27.3, 26.1, 24.7, 24.0, 23.1, 19.3. **HR-MS** (ESI) m/z: calc. for $C_{19}H_{31}N_4O_5$ 395.2289 $(M+H)^+$, found 395.2287.

H-D-Pro-L-(4*S*)-Hyp-L-DiGlu-NH$_2$ · TFA (P(2)-3):

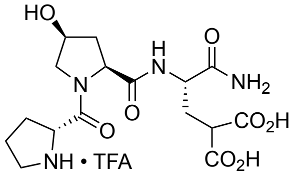

The peptide was synthesized according to the general procedure D for solid phase peptide synthesis. ^{1}**H-NMR** (400 MHz, D_2O) δ 4.74 – 4.66 (m, 1H), 4.63 – 4.54 (m, 2H), 4.42 (dd, *J* = 10.3, 4.3 Hz, 1H), 3.83 (dd, *J* = 11.3, 4.3 Hz, 1H), 3.75 (d, *J* = 11.3 Hz, 1H), 3.45 (qt, *J* = 11.5, 7.1 Hz, 2H), 2.60 – 2.49 (m, 2H), 2.49 – 2.40 (m, 1H), 2.28 – 1.95 (m, 5H). ^{13}**C-NMR** (101 MHz, D_2O) δ 175.5, 174.0, 173.7, 173.6, 169.5, 69.6, 60.3, 59.5, 54.9, 51.7, 46.6, 36.8, 29.9, 27.8, 24.0. **HR-MS** (ESI) m/z: calc. for $C_{16}H_{25}N_4O_8$ 401.1667 $(M+H)^+$, found 401.1659.

H-D-Pro-L-(4*S*)-Hyp-L-Glu-NH$_2$ · TFA (P(2)-4):

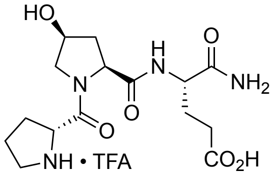

The peptide was synthesized according to the general procedure D for solid phase peptide synthesis. ^{1}**H-NMR** (400 MHz, D_2O) δ 4.67 (dd, *J* = 8.8, 7.2 Hz, 1H), 4.61 – 4.51 (m, 2H), 4.34 (dd, *J* = 9.8, 4.6 Hz, 1H), 3.79 (dd, *J* = 11.3, 4.3 Hz, 1H), 3.70 (d, *J* = 11.2 Hz, 1H), 3.41 (qt, *J* = 11.6, 7.0 Hz, 2H), 2.57 – 2.37 (m, 4H), 2.20 – 1.87 (m, 7H). ^{13}**C-NMR** (101 MHz, D_2O) δ 177.8, 175.9, 173.6, 169.4, 69.5, 60.2, 59.4, 54.9, 52.8, 46.6, 36.9, 30.2, 27.8, 25.9, 24.0. **HR-MS** (ESI) m/z: calc. for $C_{15}H_{25}N_4O_6$ 357.1769 $(M+H)^+$, found 357.1765.

H-D-Pro-L-(4*S*)-Hyp-L-Lys-NH$_2$ · TFA (P(2)-5):

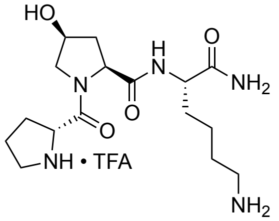

The peptide was synthesized according to the general procedure D for solid phase peptide synthesis. ^{1}**H-NMR** (400 MHz, D_2O) δ 4.65 (t, *J* = 8.0 Hz, 1H), 4.60 – 4.51 (m, 2H), 4.30 (dd, *J* = 9.6, 5.1 Hz, 1H), 3.79 (dd, *J* = 11.3, 4.4 Hz, 1H), 3.69 (d, *J* = 11.3 Hz, 1H), 3.40 (ddt, *J* = 18.4, 11.7, 5.8 Hz, 2H), 2.95 (t, *J* = 7.6 Hz, 2H), 2.50 (tt, *J* = 10.4, 4.5 Hz, 2H), 2.14 – 2.03 (m, 3H), 1.97 (dq, *J* = 14.6, 7.0 Hz, 1H), 1.86 (td, *J* = 14.3, 6.1 Hz, 1H), 1.74 (tq, *J* = 9.0, 4.8 Hz, 1H), 1.64 (h, *J* = 8.5 Hz, 2H), 1.42 (dh, *J* = 22.5, 8.2 Hz, 2H). ^{13}**C-NMR** (101 MHz, D_2O) δ 176.5, 173.6, 169.3, 69.5, 60.1, 59.4, 54.8, 53.1, 46.6, 39.1, 36.9, 30.0, 27.8, 26.1, 23.9, 22.0. **HR-MS** (ESI) m/z: calc. for $C_{16}H_{29}N_5O_4$ 356.2298 $(M+H)^+$, found 356.2292.

H-D-Pro-L-Oic-L-HomoGlu-NH$_2$ · TFA (P(2)-6):

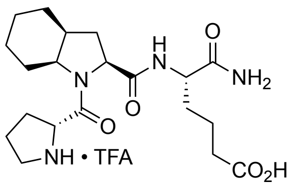

The peptide was synthesized according to the general procedure B for solid phase peptide synthesis. ^{1}**H-NMR** (500 MHz, D_2O) δ 4.56 (dd, *J* = 8.7, 7.0 Hz, 1H), 4.48 (dd, *J* = 10.2, 8.2 Hz, 1H), 4.32 (dd, *J* = 9.0, 5.3 Hz, 1H), 3.95 (dt, *J* = 12.0, 6.1 Hz, 1H), 3.49 (dt, *J* = 11.6, 7.0 Hz, 1H), 3.40 (dt, *J* = 11.5, 7.1 Hz, 1H), 2.58 (ddt, *J* = 13.2, 8.8, 6.7 Hz, 1H), 2.54 – 2.46 (m, 1H), 2.46 – 2.39 (m, 2H), 2.27 (ddd, *J* = 12.5, 8.2, 6.6 Hz, 1H), 2.14 – 2.05 (m, 2H), 2.05 – 1.98 (m, 2H), 1.95 (dd, *J* = 18.2, 10.9 Hz, 1H), 1.91 – 1.83 (m, 1H), 1.83 – 1.61 (m, 6H), 1.56 – 1.44 (m, 2H), 1.37 – 1.14 (m, 2H).
^{13}**C-NMR** (126 MHz, D_2O) δ 178.4, 176.5, 173.9, 167.9, 60.7, 58.8, 58.5, 53.4, 46.8, 36.9, 33.1, 30.4, 30.1, 29.0, 27.3, 24.6, 24.0, 23.1, 20.7, 19.3. **HR-MS** (ESI) m/z: calc. for $C_{20}H_{33}N_4O_5$ 409.2445 $(M+H)^+$, found 409.2444.

H-D-Pro-L-(4*S*)-Hyp-D-Asp-NH$_2$ · TFA (P(2)-7):

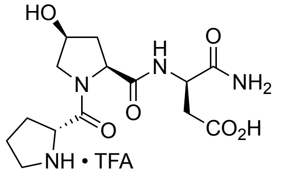

The peptide was synthesized according to the general procedure D for solid phase peptide synthesis.
^{1}H-NMR (500 MHz, D$_2$O) δ 4.67 (dd, *J* = 8.8, 7.2 Hz, 1H), 4.64 – 4.58 (m, 2H), 3.84 (dd, *J* = 11.2, 4.5 Hz, 1H), 3.75 (dt, *J* = 11.4, 1.8 Hz, 1H), 3.48 (dt, *J* = 11.5, 7.1 Hz, 1H), 3.42 (dt, *J* = 11.5, 7.0 Hz, 1H), 2.95 (dd, *J* = 16.8, 5.0 Hz, 1H), 2.83 (dd, *J* = 16.8, 8.0 Hz, 1H), 2.61 – 2.47 (m, 2H), 2.18 – 2.06 (m, 3H), 2.06 – 1.97 (m, 1H). **^{13}C-NMR** (126 MHz, D$_2$O) δ 175.1, 174.7, 173.4, 168.8, 69.6, 60.2, 59.3, 54.8, 49.9, 46.7, 36.9, 35.6, 27.9, 23.9. **HR-MS** (ESI) m/z: calc. for $C_{14}H_{23}N_4O_6$ 343.1612 $(M+H)^+$, found 343.1615.

H-D-Pro-L-αMePro-L-Glu-NH$_2$ · TFA (P(2)-8):

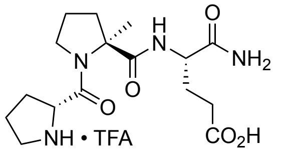

The peptide is identical to peptide **P5**, see above.

H-D-Pro-L-(4*S*)-Azp-L-Gln-NH$_2$ · TFA (P(3)-1):

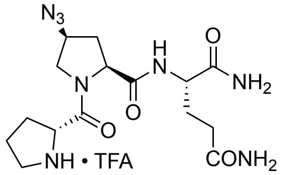

The peptide was synthesized according to the general procedure B for solid phase peptide synthesis.
^{1}H-NMR (400 MHz, D$_2$O) δ 4.73 – 4.62 (m, 2H), 4.57 (dp, *J* = 5.0, 2.2 Hz, 1H), 4.36 (dq, *J* = 9.4, 4.5 Hz, 1H), 3.89 (dd, *J* = 11.5, 5.1 Hz, 1H), 3.80 (dt, *J* = 11.5, 1.7 Hz, 1H), 3.45 (qt, *J* = 11.5, 7.0 Hz, 2H), 2.69 – 2.50 (m, 2H), 2.44 (t, *J* = 7.4 Hz, 2H), 2.36 – 2.27 (m, 1H), 2.22 – 1.94 (m, 6H). **^{13}C-NMR** (101 MHz, D$_2$O) δ 178.0, 175.8, 172.7, 169.1, 59.8, 59.5, 59.4, 53.1, 52.3, 46.7, 34.2, 31.0, 27.8, 26.5, 24.0. **HR-MS** (ESI) m/z: calc. for $C_{15}H_{25}N_8O_4$ 381.1993 $(M+H)^+$, found 381.1993.

H-D-Pro-L-(4S)-Flp-L-Arg-NH$_2$ · TFA (P(3)-2):

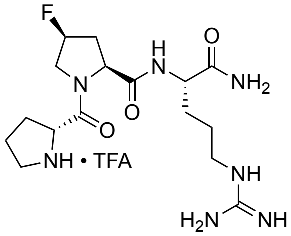

The peptide was synthesized according to the general procedure B for solid phase peptide synthesis.
^{1}H-NMR (500 MHz, D$_2$O) δ 5.48 (dt, *J* = 51.4, 3.2 Hz, 1H), 4.75 – 4.68 (m, 2H), 4.36 (dd, *J* = 9.4, 5.2 Hz, 1H), 4.12 (ddd, *J* = 23.1, 12.8, 1.9 Hz, 1H), 3.98 – 3.83 (m, 1H), 3.52 – 3.39 (m, 2H), 3.22 (t, *J* = 6.9 Hz, 2H), 2.67 (ddd, *J* = 14.6, 10.5, 3.6 Hz, 1H), 2.62 – 2.52 (m, 2H), 2.49 (d, *J* = 15.7 Hz, 1H), 2.16 – 2.07 (m, 2H), 2.07 – 1.98 (m, 1H), 1.98 – 1.84 (m, 1H), 1.82 – 1.73 (m, 1H), 1.73 – 1.57 (m, 2H). **^{13}C-NMR** (126 MHz, D$_2$O) δ 176.2, 172.9, 169.1, 156.7, 92.8 (d, *J* = 175.2 Hz), 59.8, 59.4, 53.7 (d, *J* = 23.0 Hz), 53.0, 46.6, 40.4, 36.0 (d, *J* = 20.9 Hz), 27.9, 27.7, 24.3, 23.9.**HR-MS** (ESI) m/z: calc. for $C_{16}H_{29}N_7O_3F$ 386.2310 $(M+H)^+$, found 386.2308.

H-D-Pro-L-Pro-L-DiGlu-NH$_2$ · TFA (P(3)-3):

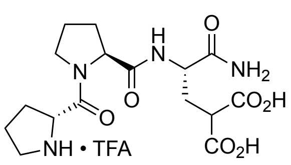

The peptide was synthesized according to the general procedure B for solid phase peptide synthesis.
^{1}H-NMR (400 MHz, D$_2$O) δ 4.68 (dd, *J* = 8.9, 6.9 Hz, 1H), 4.48 (dd, *J* = 8.5, 3.6 Hz, 1H), 4.41 (dd, *J* = 10.1, 4.6 Hz, 1H), 3.77 (dt, *J* = 9.9, 6.3 Hz, 1H), 3.63 (dt, *J* = 10.2, 7.0 Hz, 1H), 3.54 – 3.36 (m, 2H), 2.57 (ddd, *J* = 12.7, 8.8, 6.5 Hz, 1H), 2.43 (dd, *J* = 14.4, 4.6 Hz, 1H), 2.38 – 2.29 (m, 1H), 2.24 (dd, *J* = 14.5, 10.2 Hz, 1H), 2.08 (dddd, *J* = 16.2, 14.9, 12.2, 6.8 Hz, 7H), 1.50 – 1.46 (m, 1H). **^{13}C-NMR** (101 MHz, D$_2$O) δ 175.4, 174.0, 168.5, 61.0, 59.3, 51.8, 47.6, 46.6, 30.2, 29.3, 28.0, 24.2, 23.9. **HR-MS** (ESI) m/z: calc. for $C_{16}H_{25}N_4O_7$ 385.1718 $(M+H)^+$, found 385.1713.

H-D-Pro-L-(4*S*)-Azp-L-Glu-NH2 · TFA (P(3)-4):

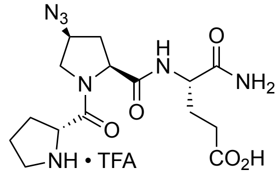

The peptide was synthesized according to the general procedure B for solid phase peptide synthesis.
^{1}H-NMR (400 MHz, D_2O) δ 4.72 – 4.60 (m, 2H), 4.57 (dt, *J* = 5.2, 2.9 Hz, 1H), 4.41 (dt, *J* = 9.6, 5.5 Hz, 1H), 3.89 (dd, *J* = 11.5, 5.1 Hz, 1H), 3.79 (d, *J* = 11.4 Hz, 1H), 3.55 – 3.36 (m, 2H), 2.68 – 2.45 (m, 4H), 2.32 (d, *J* = 14.1 Hz, 1H), 2.25 – 1.94 (m, 6H). **^{13}C-NMR** (101 MHz, D_2O) δ 177.5, 175.8, 172.7, 169.1, 59.9, 59.6, 59.4, 53.0, 52.3, 46.7, 34.2, 30.0, 27.8, 25.9, 24.0. **HR-MS** (ESI) m/z: calc. for $C_{15}H_{24}N_7O_5$ 382.1833 $(M+H)^+$, found 382.1832.

H-D-Pro-L-(4*S*)-Flp-L-Asp-NH2 · TFA (P(3)-5):

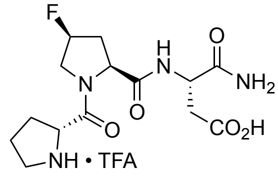

The peptide was synthesized according to the general procedure B for solid phase peptide synthesis.
^{1}H-NMR (400 MHz, D_2O) δ 5.46 (dt, *J* = 51.2, 3.1 Hz, 1H), 4.74 – 4.67 (m, 2H), 4.11 (ddd, *J* = 22.9, 12.7, 1.9 Hz, 1H), 4.00 – 3.79 (m, 1H), 3.45 (qt, *J* = 11.5, 7.1 Hz, 2H), 2.97 (ddt, *J* = 17.0, 5.4, 1.3 Hz, 1H), 2.89 – 2.75 (m, 1H), 2.73 – 2.42 (m, 3H), 2.10 (dq, *J* = 12.5, 6.6 Hz, 2H), 2.05 – 1.93 (m, 1H). **^{13}C-NMR** (101 MHz, D_2O) δ 174.8, 174.3, 172.7, 169.2, 92.8 (d, *J* = 175.1 Hz), 59.9, 59.4, 53.7 (d, *J* = 23.2 Hz), 49.9, 46.6, 35.8 (d, *J* = 21.0 Hz), 35.5, 27.7, 24.0. **HR-MS** (ESI) m/z: calc. for $C_{14}H_{22}N_4O_5F$ 345.1569 $(M+H)^+$, found 345.1572.

H-D-Pro-L-(4*S*)-Azp-L-Cit-NH2 · TFA (P(3)-6):

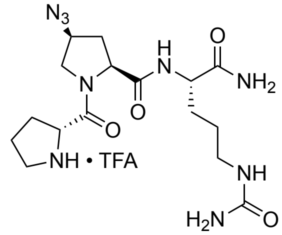

The peptide was synthesized according to the general procedure B for solid phase peptide synthesis.
^{1}H-NMR (400 MHz, D_2O) δ 4.71 – 4.60 (m, 2H), 4.57 (dt, *J* = 5.4, 2.8 Hz, 1H), 4.35 (dd, *J* = 9.3, 5.2 Hz, 1H), 3.88 (dd, *J* = 11.4, 5.2 Hz, 1H), 3.78 (d, *J* = 11.4 Hz, 1H), 3.45 (qt, *J* = 11.4, 7.0 Hz, 2H), 3.13 (t, *J* = 6.8 Hz, 2H), 2.71 – 2.59 (m, 1H), 2.59 – 2.42 (m, 1H), 2.31 (dd, *J* = 14.7, 3.2 Hz, 1H), 2.11 (p, *J* = 7.0 Hz, 2H), 2.02 (ddd, *J* = 14.8, 12.9, 7.1 Hz, 1H), 1.95 – 1.82 (m, 1H), 1.76 (dtd, *J* = 14.0, 9.7, 4.8 Hz, 1H), 1.70 – 1.48 (m, 2H). **^{13}C-NMR** (101 MHz, D_2O) δ 176.4, 172.7, 169.1, 161.5, 59.8, 59.5, 59.4, 53.4, 52.3, 46.7, 34.2, 28.1, 27.8, 25.7, 24.0. **HR-MS** (ESI) m/z: calc. for $C_{16}H_{28}N_9O_4$ 410.2259 $(M+H)^+$, found 410.2257.

H-D-Pro-L-(4*S*)-Azp-L-"F"Phen-NH2 · TFA (P(3)-7):

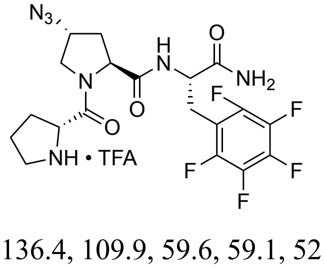

The peptide was synthesized according to the general procedure B for solid phase peptide synthesis.
^{1}H-NMR (500 MHz, D_2O) δ 4.66 – 4.52 (m, 3H), 4.52 – 4.45 (m, 1H), 3.85 (dd, *J* = 11.6, 4.8 Hz, 1H), 3.72 (d, *J* = 11.6 Hz, 1H), 3.51 (dt, *J* = 11.5, 7.0 Hz, 1H), 3.42 (dt, *J* = 11.5, 7.2 Hz, 1H), 3.30 (dd, *J* = 14.3, 6.8 Hz, 1H), 3.23 (dd, *J* = 14.3, 9.0 Hz, 1H), 2.66 – 2.56 (m, 1H), 2.55 – 2.44 (m, 1H), 2.17 – 2.08 (m, 3H), 2.08 – 1.97 (m, 1H). **^{13}C-NMR** (126 MHz, D_2O) δ 174.2, 172.7, 168.5, 136.4, 109.9, 59.6, 59.1, 52.4, 52.2, 46.8, 34.4, 28.3, 24.3, 23.8. **HR-MS** (ESI) m/z: calc. for $C_{19}H_{21}N_7O_3F_5$ 490.1621 $(M+H)^+$, found 490.1619.

H-D-Pro-D-His-L-(4*S*)-Flp-NH_2 · TFA (P(4)-1):

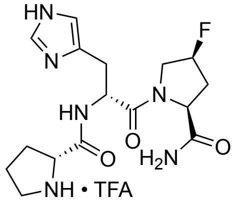

The peptide was synthesized according to the general procedure B for solid phase peptide synthesis.
^{1}H-NMR (400 MHz, D_2O) δ 8.68 – 8.58 (m, 1H), 7.43 – 7.30 (m, 1H), 5.55 – 5.31 (m, 1H), 5.10 – 4.92 (m, 1H), 4.69 – 4.62 (m, 1H), 4.44 – 4.32 (m, 1H), 4.26 – 4.11 (m, 1H), 4.05 – 3.70 (m, 1H), 3.50 – 3.33 (m, 2H), 3.32 – 3.23 (m, 1H), 3.24 – 3.13 (m, 1H), 2.78 – 2.51 (m, 1H), 2.51 – 2.39 (m, 2H), 2.15 – 1.96 (m, 3H). **^{13}C-NMR** (101 MHz, D_2O) δ 175.5, 170.1, 169.5, 133.8, 128.0, 117.8, 117.4, 117.3, 93.8, 92.1, 59.5, 59.2, 53.9 (d, *J* = 23.7 Hz), 51.2, 46.4, 35.8 (d, *J* = 20.9 Hz), 29.7, 25.4, 23.6. **HR-MS** (ESI) m/z: calc. for $C_{15}H_{25}N_8O_4$ 381.1993 $(M+H)^+$, found 381.1993.

H-L-Pro-L-His-D-Pro-NH_2 · TFA (P(4)-2):

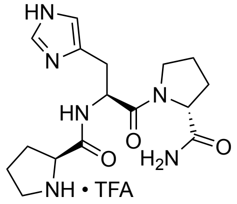

The peptide was synthesized according to the general procedure B for solid phase peptide synthesis.
^{1}H-NMR (400 MHz, D_2O) δ 8.67 – 8.58 (m, 1H), 7.39 – 7.28 (m, 1H), 5.07 (ddd, *J* = 28.4, 8.0, 6.2 Hz, 1H), 4.50 – 4.32 (m, 2H), 3.89 – 3.70 (m, 1H), 3.67 – 3.48 (m, 1H), 3.49 – 3.32 (m, 2H), 3.32 – 3.22 (m, 1H), 3.23 – 3.06 (m, 1H), 2.52 – 2.34 (m, 1H), 2.34 – 2.20 (m, 1H), 2.12 – 2.01 (m, 4H), 2.01 – 1.88 (m, 2H). **^{13}C-NMR** (101 MHz, D_2O) δ 176.6, 169.5, 169.2, 133.7, 128.2, 117.3, 60.5, 59.6, 51.1, 48.0, 46.4, 29.7, 29.5, 25.7, 24.3, 23.7, 23.6, 23.6. **HR-MS** (ESI) m/z: calc. for $C_{16}H_{29}N_7O_3F$ 386.2310 $(M+H)^+$, found 386.2308.

H-D-Pro-D-His-L-(4*S*)-Hyp-NH_2 · TFA (P(4)-3):

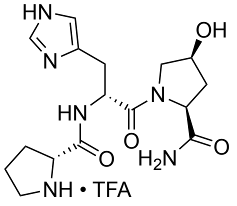

The peptide was synthesized according to the general procedure D for solid phase peptide synthesis.
^{1}H-NMR (400 MHz, D_2O) δ 8.56 (d, *J* = 18.6 Hz, 1H), 7.32 (s, 1H), 4.95 (t, *J* = 7.2 Hz, 1H), 4.55 – 4.45 (m, 2H), 4.38 (ddd, *J* = 20.6, 8.4, 6.1 Hz, 1H), 3.80 – 3.69 (m, 1H), 3.66 (dd, *J* = 11.2, 4.9 Hz, 1H), 3.50 – 3.33 (m, 3H), 3.21 (dtt, *J* = 23.0, 15.5, 8.3 Hz, 3H), 2.63 – 2.37 (m, 3H), 2.17 – 1.93 (m, 5H). **^{13}C-NMR** (101 MHz, D_2O) δ 176.3, 170.4, 169.5, 69.5, 59.5, 59.4, 54.9, 51.7, 46.4, 36.8, 29.7, 25.7, 23.6. **HR-MS** (ESI) m/z: calc. for $C_{16}H_{25}N_4O_7$ 385.1718 $(M+H)^+$, found 385.1713.

H-D-Pro-D-*iso*Leu-L-(4*S*)-Hyp-NH_2 · TFA (P(4)-4):

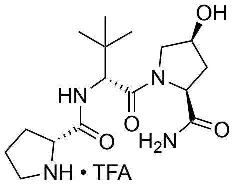

The peptide was synthesized according to the general procedure D for solid phase peptide synthesis.
^{1}H-NMR (400 MHz, D_2O) δ 4.57 (tt, *J* = 4.8, 2.5 Hz, 1H), 4.54 – 4.44 (m, 3H), 4.04 – 3.90 (m, 2H), 3.52 – 3.33 (m, 2H), 2.57 – 2.37 (m, 2H), 2.14 – 1.92 (m, 4H), 1.05 (s, 9H). **^{13}C-NMR** (101 MHz, D_2O) δ 176.8, 171.6, 170.1, 69.7, 59.5, 59.5, 59.4, 56.1, 46.8, 37.0, 33.9, 30.0, 25.5, 23.6. **HR-MS** (ESI) m/z: calc. for $C_{15}H_{24}N_7O_5$ 382.1833 $(M+H)^+$, found 382.1832.

H-D-Pro-D-Gln-L-(4*S*)-Hyp-NH_2 · TFA (P(4)-5):

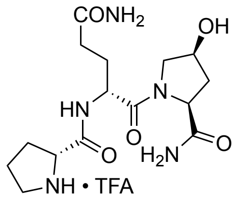

The peptide was synthesized according to the general procedure D for solid phase peptide synthesis.
^{1}H-NMR (400 MHz, D_2O) δ 4.67 (dd, *J* = 9.0, 4.9 Hz, 1H), 4.59 (tt, *J* = 4.9, 2.6 Hz, 1H), 4.50 (dd, *J* = 9.7, 3.1 Hz, 1H), 4.48 – 4.41 (m, 1H), 3.92 (dd, *J* = 11.4, 4.7 Hz, 1H), 3.88 – 3.80 (m, 1H), 3.51 – 3.32 (m, 2H), 2.56 – 2.45 (m, 2H), 2.42 (ddd, *J* = 10.0, 6.0, 2.5 Hz, 2H), 2.19 – 1.92 (m, 7H). **^{13}C-NMR** (101 MHz, D_2O) δ 177.6,

176.5, 171.7, 169.8, 69.7, 59.5, 59.5, 55.0, 51.5, 46.5, 36.9, 30.6, 29.7, 25.4, 23.7. **HR-MS** (ESI) m/z: calc. for $C_{14}H_{22}N_4O_5F$ 345.1569 $(M+H)^+$, found 345.1572.

H-D-Pro-D-*iso*Leu-L-Pro-NH₂ · TFA (P(4)-6):

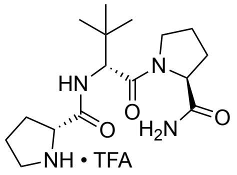

The peptide was synthesized according to the general procedure B for solid phase peptide synthesis.
^{1}H-NMR (400 MHz, D_2O) δ 4.63 (s, 1H), 4.52 – 4.45 (m, 1H), 4.41 (dd, *J* = 8.9, 3.7 Hz, 1H), 3.92 (dt, *J* = 10.1, 6.3 Hz, 1H), 3.83 (dt, *J* = 10.5, 7.2 Hz, 1H), 3.53 – 3.34 (m, 2H), 2.53 – 2.39 (m, 1H), 2.37 – 2.25 (m, 1H), 2.13 – 1.94 (m, 6H), 1.04 (s, 9H). **^{13}C-NMR** (101 MHz, D_2O) δ 176.9, 170.9, 169.7, 60.3, 59.5, 59.0, 48.8, 46.6, 34.3, 30.0, 29.6, 25.6, 24.1, 23.6. **HR-MS** (ESI) m/z: calc. for $C_{16}H_{28}N_9O_4$ 410.2259 $(M+H)^+$, found 410.2257.

H-D-Pro-D-Gln-L-*iso*Leu-NH₂ · TFA (P(4)-7):

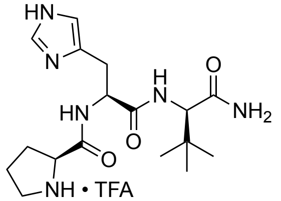

The peptide was synthesized according to the general procedure B for solid phase peptide synthesis.
^{1}H-NMR (400 MHz, D_2O) δ 8.64 (dd, *J* = 4.1, 1.4 Hz, 1H), 7.38 – 7.30 (m, 1H), 4.88 – 4.82 (m, 1H), 4.40 (dt, *J* = 9.9, 6.7 Hz, 1H), 4.11 (d, *J* = 50.2 Hz, 1H), 3.50 – 3.34 (m, 2H), 3.34 – 3.15 (m, 2H), 2.54 – 2.39 (m, 1H), 2.15 – 1.98 (m, 3H), 0.96 (d, *J* = 34.1 Hz, 9H). **^{13}C-NMR** (101 MHz, D_2O) δ 174.6, 171.3, 169.5, 133.8, 128.2, 117.3, 61.9, 59.6, 53.1, 46.5, 32.9, 29.8, 26.4, 25.7, 23.6. **HR-MS** (ESI) m/z: calc. for $C_{19}H_{21}N_7O_3F_5$ 490.1621 $(M+H)^+$, found 490.1619.

H-L-Pro-L-His-D-*iso*Leu-NH₂ · TFA (P(4)-8):

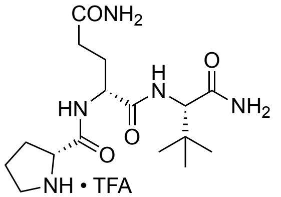

The peptide was synthesized according to the general procedure B for solid phase peptide synthesis.
^{1}H-NMR (400 MHz, D_2O) δ 4.53 (dd, *J* = 8.8, 5.6 Hz, 1H), 4.44 (dd, *J* = 8.5, 6.2 Hz, 1H), 4.14 (s, 1H), 3.50 – 3.34 (m, 2H), 2.54 – 2.44 (m, 1H), 2.41 (t, *J* = 7.5 Hz, 2H), 2.21 – 1.96 (m, 5H), 1.03 (s, 9H). **^{13}C-NMR** (101 MHz, D_2O) δ 177.7, 174.8, 172.9, 169.6, 61.8, 59.6, 53.4, 46.5, 33.1, 31.0, 29.8, 27.0, 25.9, 23.7. **HR-MS** (ESI) m/z: calc. for $C_{16}H_{29}N_7O_3F$ 386.2310 $(M+H)^+$, found 386.2308.

H-D-Pro-D-His-L-4-MePhe-NH₂ · TFA (P(4)-9):

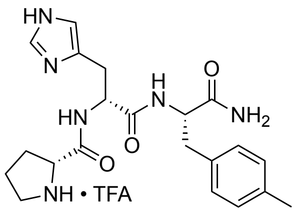

The peptide was synthesized according to the general procedure B for solid phase peptide synthesis.
^{1}H-NMR (400 MHz, D_2O) δ 8.46 (d, *J* = 1.4 Hz, 1H), 7.24 – 7.16 (m, 3H), 7.14 (d, *J* = 8.2 Hz, 2H), 6.94 (d, *J* = 1.3 Hz, 1H), 4.65 (t, *J* = 7.1 Hz, 1H), 4.58 (dd, *J* = 10.4, 4.7 Hz, 1H), 4.40 – 4.33 (m, 1H), 3.46 – 3.33 (m, 2H), 3.24 – 3.16 (m, 1H), 3.07 – 3.00 (m, 2H), 2.85 (dd, *J* = 14.3, 10.5 Hz, 1H), 2.47 – 2.35 (m, 1H), 2.32 (s, 3H), 2.09 – 1.88 (m, 3H). **^{13}C-NMR** (101 MHz, D_2O) δ 175.7, 171.3, 169.5, 137.1, 133.4, 129.3, 129.2, 128.9, 127.6, 116.9, 59.5, 54.7, 52.9, 46.5, 36.4, 29.7, 26.1, 23.6, 20.0. **HR-MS** (ESI) m/z: calc. for $C_{16}H_{25}N_4O_7$ 385.1718 $(M+H)^+$, found 385.1713.

Analytical Data of Additional Training Set Peptides

H-D-Pro-D-Gln-L-Ile-NH$_2$ · TFA

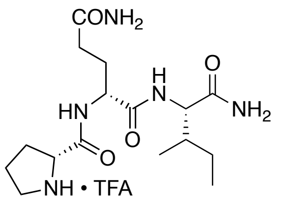

The peptide was synthesized according to the general procedure B for solid phase peptide synthesis.
^{1}H-NMR ^{1}H NMR (500 MHz, D$_2$O) δ 4.44 (td, *J* = 8.9, 6.0 Hz, 2H), 4.17 (dd, *J* = 7.2, 1.0 Hz, 1H), 3.42 (qt, *J* = 12.2, 6.9 Hz, 2H), 2.48 (q, *J* = 6.4 Hz, 1H), 2.39 (t, *J* = 7.5 Hz, 2H), 2.07 (dddd, *J* = 29.7, 15.3, 11.3, 7.2 Hz, 5H), 1.97 – 1.84 (m, 1H), 1.48 (ddd, *J* = 13.5, 7.4, 3.6 Hz, 1H), 1.30 – 1.14 (m, 1H), 0.96 (dd, *J* = 6.8, 1.0 Hz, 3H), 0.94 – 0.80 (m, 3H). **^{13}C-NMR** (126 MHz, D$_2$O) δ 177.6, 176.1, 173.2, 169.6, 59.5, 58.3, 53.5, 46.5, 35.7, 30.9, 29.7, 26.9, 24.5, 23.7, 14.9, 10.2. **HR-MS** (ESI) m/z: calc. for $C_{16}H_{30}N_5O_4^+$ 356.2292 (M+H)$^+$, found 356.2287.

H-D-Pro-D-Gln-L-Val-NH$_2$ · TFA

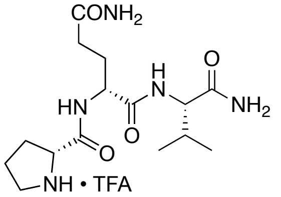

The peptide was synthesized according to the general procedure B for solid phase peptide synthesis.
^{1}H-NMR ^{1}H NMR (400 MHz, D$_2$O) δ 4.45 (ddd, *J* = 14.5, 8.6, 6.1 Hz, 2H), 4.14 (d, *J* = 6.7 Hz, 1H), 3.51 – 3.34 (m, 2H), 2.96 (s, 1H), 2.47 (dd, *J* = 8.7, 6.0 Hz, 1H), 2.40 (t, *J* = 7.5 Hz, 2H), 2.21 – 1.97 (m, 7H), 0.97 (dd, *J* = 6.8, 5.5 Hz, 6H). **^{13}C-NMR** (101 MHz, D$_2$O) δ 177.6, 176.0, 173.2, 169.6, 59.5, 59.3, 53.5, 46.5, 31.0, 29.7, 29.6, 26.9, 23.7, 18.4, 17.3. **HR-MS** (ESI) m/z: calc. for $C_{15}H_{28}N_5O_4^+$ 342.2136 (M+H)$^+$, found 342.2134.

H-D-Pro-D-Gln-L-Pro-NH$_2$

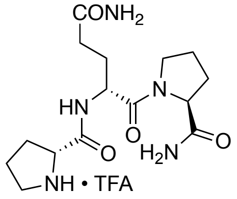

The peptide was synthesized according to the general procedure B for solid phase peptide synthesis.
^{1}H-NMR (400 MHz, D$_2$O) δ 4.73 (dd, *J* = 9.2, 4.7 Hz, 1H), 4.42 (ddd, *J* = 15.9, 8.0, 4.5 Hz, 2H), 3.85 (dt, *J* = 10.0, 6.2 Hz, 1H), 3.71 (dt, *J* = 10.4, 7.0 Hz, 1H), 3.50 – 3.32 (m, 2H), 2.53 – 2.37 (m, 3H), 2.36 – 2.25 (m, 1H), 2.16 – 1.90 (m, 9H). **^{13}C-NMR** (101 MHz, D$_2$O) δ 177.7, 176.8, 171.1, 169.5, 60.6, 59.6, 51.3, 47.8, 46.5, 30.6, 29.7, 29.6, 25.8, 24.2, 23.7. **HR-MS** (ESI) m/z: calc. for $C_{15}H_{26}N_5O_4^+$ 340.1979 (M+H)$^+$, found 340.1975.

H-D-Pro-D-Gln-L-Phe-NH$_2$ · TFA

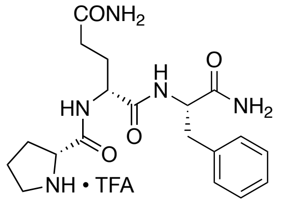

The peptide was synthesized according to the general procedure B for solid phase peptide synthesis.
^{1}H-NMR (400 MHz, D$_2$O) δ 7.32 – 7.25 (m, 2H), 7.25 – 7.17 (m, 3H), 4.66 (dd, *J* = 11.0, 4.8 Hz, 2H), 4.28 (dd, *J* = 8.6, 6.1 Hz, 1H), 4.15 (t, *J* = 7.0 Hz, 1H), 3.38 – 3.23 (m, 3H), 2.87 (d, *J* = 0.6 Hz, 1H), 2.82 (dd, *J* = 14.3, 11.0 Hz, 1H), 2.38 – 2.26 (m, 1H), 2.03 – 1.82 (m, 3H), 1.82 – 1.64 (m, 2H), 1.59 (ddd, *J* = 9.2, 7.4, 4.4 Hz, 2H). **^{13}C-NMR** (101 MHz, D$_2$O) δ 177.5, 175.8, 172.8, 169.5, 136.6, 129.0, 128.8, 127.3, 59.4, 54.3, 53.6, 46.5, 38.8, 36.8, 30.5, 29.7, 26.6, 23.7. **HR-MS** (ESI) m/z: calc. for $C_{19}H_{28}N_5O_4^+$ 390.2136 (M+H)$^+$, found 390.2131.

H-D-Pro-D-Gln-L-Asn-NH₂ · TFA

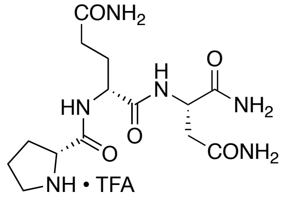

The peptide was synthesized according to the general procedure B for solid phase peptide synthesis.
^{1}H-NMR (400 MHz, D_2O) δ 4.75 (dd, *J* = 9.2, 4.7 Hz, 1H), 4.44 (dd, *J* = 8.0, 5.4 Hz, 1H), 4.37 (dd, *J* = 7.9, 6.5 Hz, 1H), 3.42 (qt, *J* = 11.5, 7.0 Hz, 2H), 2.89 (dd, *J* = 15.7, 4.8 Hz, 1H), 2.74 (dd, *J* = 15.7, 9.2 Hz, 1H), 2.49 (tq, *J* = 8.8, 3.2 Hz, 1H), 2.37 (t, *J* = 7.5 Hz, 2H), 2.16 – 1.96 (m, 5H). **^{13}C-NMR** (101 MHz, D_2O) δ 177.6, 175.0, 174.4, 172.9, 169.7, 59.5, 53.7, 50.2, 46.5, 36.3, 30.8, 29.7, 26.6, 23.7. **HR-MS** (ESI) m/z: calc. for $C_{14}H_{25}N_6O_5^+$ 357.1881 $(M+H)^+$, found 357.1875.

H-D-Pro-D-Asn-L-Leu-NH₂

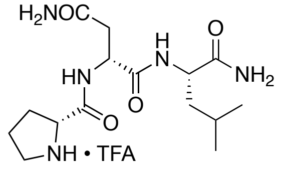

The peptide was synthesized according to the general procedure B for solid phase peptide synthesis.
^{1}H-NMR (500 MHz, D_2O) δ 4.73 (dd, *J* = 7.6, 6.9 Hz, 1H), 4.46 – 4.39 (m, 1H), 4.31 (dd, *J* = 10.5, 3.8 Hz, 1H), 3.50 – 3.35 (m, 2H), 2.86 – 2.75 (m, 2H), 2.51 – 2.41 (m, 1H), 2.13 – 1.98 (m, 3H), 1.74 – 1.57 (m, 3H), 0.94 (d, *J* = 5.9 Hz, 3H), 0.88 (d, *J* = 5.8 Hz, 3H). **^{13}C-NMR** (126 MHz, D_2O) δ 177.4, 173.74, 172.3, 169.6, 59.5, 52.2, 51.1, 46.5, 39.6, 36.2, 29.7, 24.3, 23.7, 22.3, 20.2. **HR-MS** (ESI) m/z: calc. for $C_{15}H_{28}N_5O_4^+$ 342.2136 $(M+H)^+$, found 342.2130.

H-D-Pro-D-His-L-Leu-NH₂ · TFA

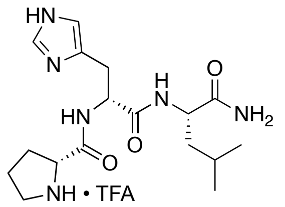

The peptide was synthesized according to the general procedure B for solid phase peptide synthesis.
^{1}H-NMR ^{1}H NMR (500 MHz, D_2O) δ 8.70 (d, *J* = 1.4 Hz, 1H), 7.37 (d, *J* = 1.4 Hz, 1H), 4.66 (dd, *J* = 8.9, 7.0 Hz, 1H), 4.48 – 4.40 (m, 1H), 4.20 (dd, *J* = 10.8, 4.6 Hz, 1H), 3.47 – 3.34 (m, 2H), 3.30 – 3.20 (m, 2H), 2.52 – 2.40 (m, 1H), 2.13 – 1.96 (m, 3H), 1.63 – 1.48 (m, 2H), 1.09 – 0.97 (m, 1H), 0.86 (d, *J* = 6.7 Hz, 3H), 0.77 (d, *J* = 6.5 Hz, 3H). **^{13}C-NMR** (126 MHz, D_2O) δ 177.2, 171.7, 169.6, 133.9, 127.9, 117.5, 59.5, 53.8, 52.0, 46.5, 39.3, 29.7, 26.0, 24.1, 23.6, 22.2, 19.9. **HR-MS** (ESI) m/z: calc. for $C_{17}H_{29}N_6O_3^+$ 365.2296 $(M+H)^+$, found 365.2293.

2.2 Catalysis Screenings

Screening of Conjugate Addition Reactions

General Procedure: Two stock solutions were prepared: The first one of the peptide catalyst (10 mM, 15 mmol, *for details see below*) in 15 mL $CHCl_3$/*i*PrOH 9:1, the second one of nitrostyrene derivative (1.0 M, 10 mmol, *for details see below*), aldehyde (1.5 M, 15 mmol, *for details see below*), and *N*-methyl morpholine (10 mM, 0.1 mmol, 11 µL) in 10 mL $CHCl_3$/*i*PrOH 9:1. 200 µL of both stock solutions were combined and the reaction mixture was stirred in a sealed vial for 24 h. Conversion and diastereomeric ratio were determined by ^{1}H NMR spectroscopy (50 µL crude mixture in 600 µL $CDCl_3$). The enantiomeric excess was determined by chiral stationary phase HPLC.

Overview of peptide amounts used in first stock solution:

		M (g/mol)	m (mg)			M (g/mol)	m (mg)
pPE	**P1**	454.4	6.8	p"MePro"N	**P26**	453.4	6.8
ppE	**P2**	454.4	6.8	pPQ	**P27**	453.4	6.8
PpE	**P3**	454.4	6.8	pPR	**P28**	481.5	7.2
PPE	**P4**	454.4	6.8	ppR	**P29**	481.5	7.2
p"MePro"E	**P5**	468.4	7.0	PpR	**P30**	481.5	7.2
P"MePro"e	**P6**	468.4	7.0	PPR	**P31**	481.5	7.2
P"D-MePro"E	**P7**	468.4	7.0	pPH	**P32**	462.4	6.9
P"MePro"E	**P8**	468.4	7.0	ppH	**P33**	462.4	6.9
p4E	**P9**	440.4	6.6	PpH	**P34**	462.4	6.9
p6E	**P10**	468.4	7.0	PPH	**P35**	462.4	6.9
p"Tic"E	**P11**	516.5	7.7	pPF	**P36**	472.5	7.1
p"4R-Hyp"E	**P12**	470.4	7.1	p"MePro"F	**P37**	486.5	7.3
p"4R-Flp"E	**P13**	472.4	7.1	P"MePro"F	**P38**	486.5	7.3
p"4S-Flp"E	**P14**	472.4	7.1	pP"p-NO2-F"	**P39**	517.5	7.8
p"4R-Azp"E	**P15**	495.4	7.4	pPY	**P40**	488.5	7.3
p"4S-Azp"E	**P16**	495.4	7.4	pPW	**P41**	511.5	7.7
pGE	**P17**	414.3	6.2	pPS	**P42**	412.4	6.2
pAE	**P18**	428.4	6.4	pPT	**P43**	426.4	6.4
PPD	**P19**	440.4	6.6	PGG	**P44**	342.3	5.1
pPD	**P20**	440.4	6.6	PWQ	**P45**	542.5	8.1
P6D	**P21**	454.4	6.8	PSQ	**P46**	443.4	6.7
P"MePro"D	**P22**	454.4	6.8	PGL	**P47**	398.4	6.0
pPN	**P23**	439.4	6.6	PQQ	**P48**	484.4	7.3
p4N	**P24**	425.4	6.4	PWG	**P49**	471.4	7.1
p6N	**P25**	453.4	6.8	PLS	**P50**	428.4	6.4

Overview of substrate amounts used in second stock solution:

Reaction (I): 1.492 g nitrostyrene, 1.35 mL butanal.

Reaction (II): 1.492 g nitrostyrene, 1.08 mL propanal.

Reaction (III): 1.792 g 4-methoxy nitrostyrene, 1.35 mL butanal.

Reaction (IV): 2.280 g 2-bromo nitrostyrene, 1.35 mL butanal.

Examples for sample analyses:

Reaction (**I**) in the presence of **P1**:

The enantiomeric excess was determined by chiral stationary phase HPLC: Chiralcel IC, hexane/iPrOH 9:1, 1.0 mL/min, 25 °C, 210 nm, 32.3 min (*syn*, major), 36.2 min (*syn*, minor).

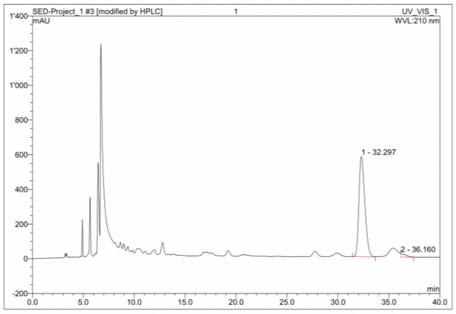

No.	Ret.Time min	Peak Name	Height mAU	Area mAU*min	Rel.Area %	Amount	Type
1	32.30	n.a.	578.209	388.735	97.37	n.a.	BMB*
2	36.16	n.a.	18.804	10.499	2.63	n.a.	MB*
Total:			597.013	399.234	100.00	0.000	

The diastereomeric ratio was determined by comparing the signals corresponding to the aldehyde C<u>H</u>O of the *syn* (9.74 ppm) and *anti*-diastereomer (9.51 ppm). The conversion was determined by comparing the signals corresponding to the aldehyde C<u>H</u>O of the products (9.74 ppm, 9.51 ppm) and the olefinic signal of nitrostyrene Ph-CH-C<u>H</u>-NO_2 (8.04 ppm).

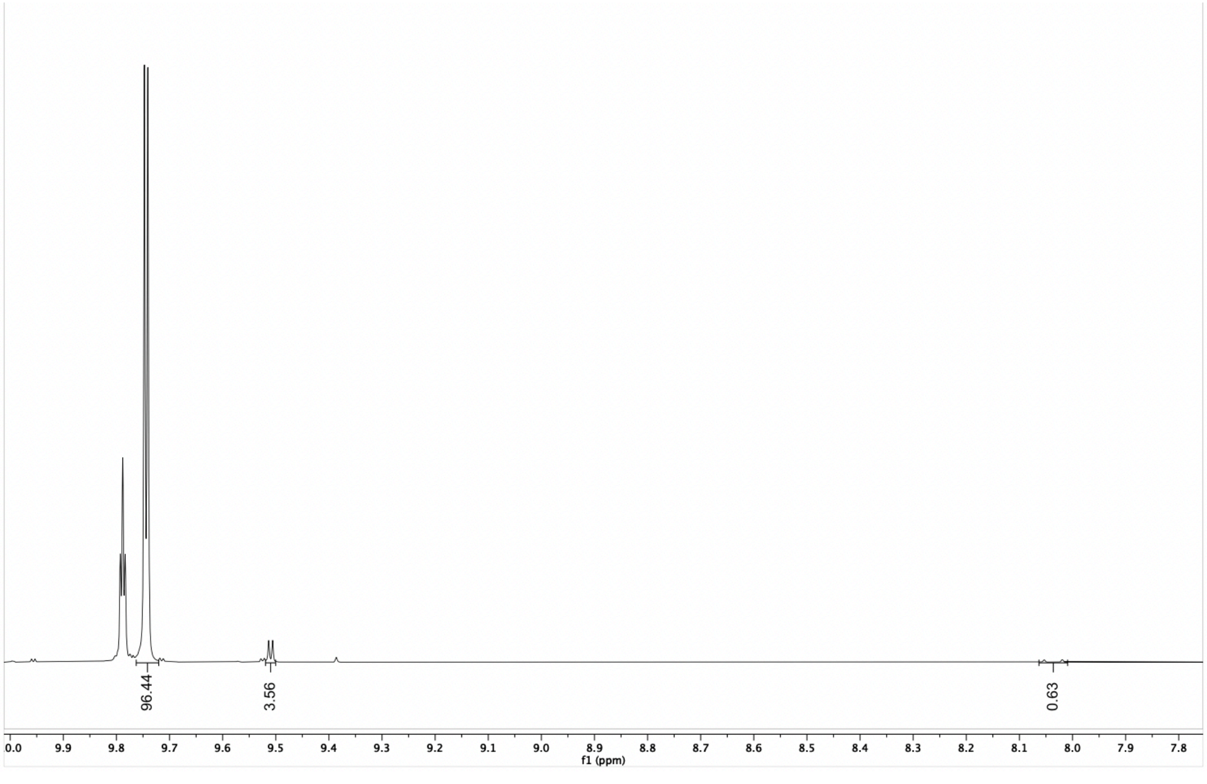

Reaction (**II**) in the presence of **P1**:

The enantiomeric excess was determined by chiral stationary phase HPLC: Chiralcel IC, hexane/iPrOH 9:1, 1.0 mL/min, 25 °C, 210 nm, 36.6 min (*syn*, major), 43.9 min (*syn*, minor).

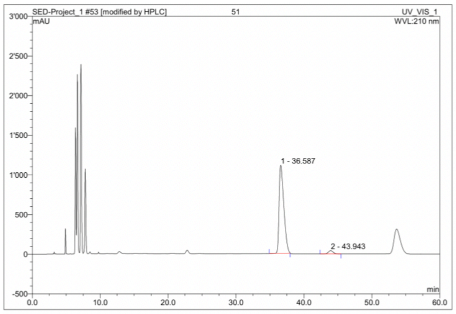

No.	Ret.Time min	Peak Name	Height mAU	Area mAU*min	Rel.Area %	Amount	Type
1	36.59	n.a.	1109.460	890.271	96.05	n.a.	BMB*
2	43.94	n.a.	41.887	36.614	3.95	n.a.	BMB*
Total:			1151.347	926.885	100.00	0.000	

The diastereomeric ratio was determined by comparing the signals corresponding to the aldehyde C<u>H</u>O of the *syn* (9.74 ppm) and *anti*-diastereomer (9.56 ppm). The conversion was determined by comparing the signals corresponding to the aldehyde C<u>H</u>O of the products (9.74 ppm, 9.56 ppm) versus the olefinic signal of nitrostyrene Ph-CH-C<u>H</u>-NO_2 (8.04 ppm).

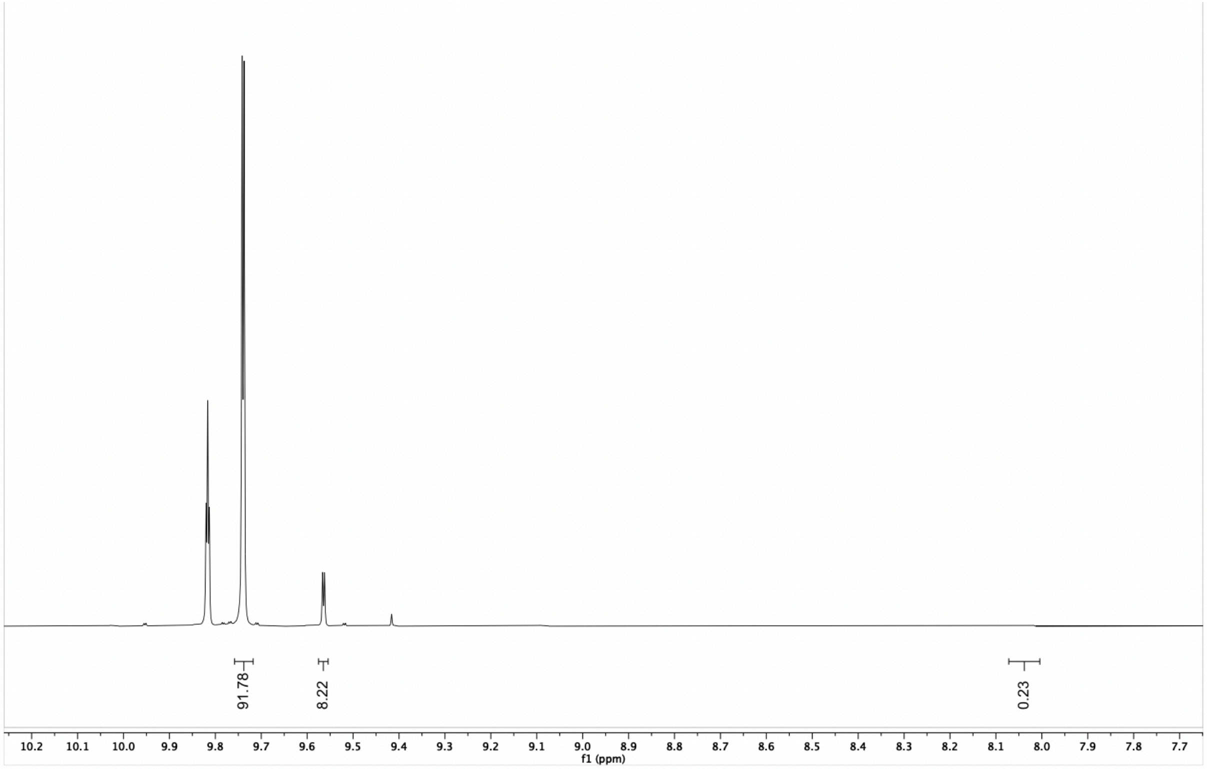

Reaction (**III**) in the presence of **P1**:

The enantiomeric excess was determined by chiral stationary phase HPLC: Chiralcel IC, hexane/iPrOH 9:1, 1.0 mL/min, 25 °C, 210 nm, 25.0 min (*syn*, major), 27.6 min (*syn*, minor).

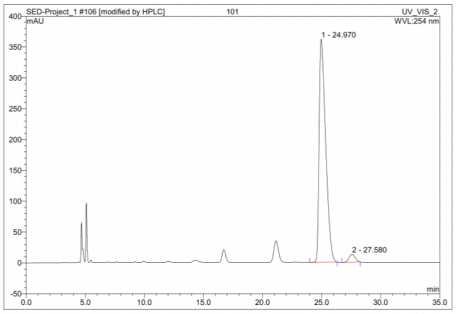

No.	Ret.Time min	Peak Name	Height mAU	Area mAU*min	Rel.Area %	Amount	Type
1	24.97	n.a.	361.691	232.014	96.82	n.a.	BMB*
2	27.58	n.a.	12.887	7.613	3.18	n.a.	BMB*
Total:			374.577	239.626	100.00	0.000	

The diastereomeric ratio was determined by comparing the signals corresponding to the aldehyde C<u>H</u>O of the *syn* (9.73 ppm) and *anti*-diastereomer (9.49 ppm). The conversion was determined by comparing the signals corresponding to the aldehyde C<u>H</u>O of the products (9.73 ppm, 9.49 ppm) versus the olefinic signal of nitrostyrene derivative Ar-CH-C<u>H</u>-NO_2 (8.00 ppm).

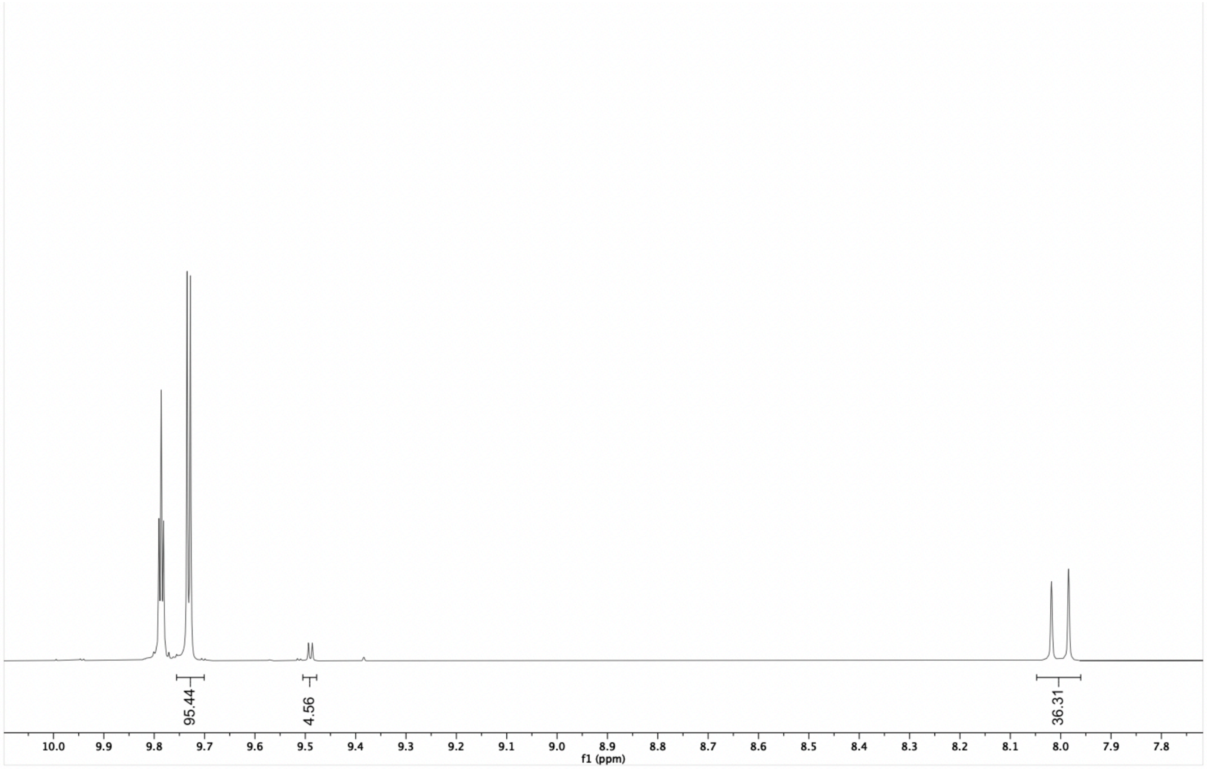

Reaction (**IV**) in the presence of **P1**:

The enantiomeric excess was determined by chiral stationary phase HPLC: Chiralcel AD-H, hexane/iPrOH 98.5:1.5, 0.9 mL/min, 25 °C, 210 nm, 27.6 min (*syn*, minor), 30.5 min (*syn*, major).

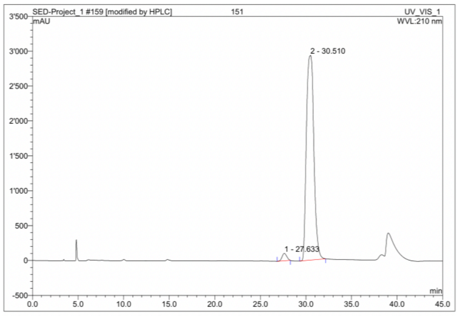

No.	Ret.Time min	Peak Name	Height mAU	Area mAU*min	Rel.Area %	Amount	Type
1	27.63	n.a.	105.877	62.685	2.20	n.a.	BMB*
2	30.51	n.a.	2936.353	2784.303	97.80	n.a.	BMB*
Total:			3042.229	2846.989	100.00	0.000	

The diastereomeric ratio was determined by comparing the signals corresponding to the aldehyde C<u>H</u>O of the *syn* (9.76 ppm) and *anti*-diastereomer (9.62 ppm). The conversion was determined by comparing the signals corresponding to the aldehyde C<u>H</u>O of the products (9.76 ppm, 9.62 ppm) versus the olefinic signal of nitrostyrene derivative Ar-CH-C<u>H</u>-NO_2 (7.63 ppm).

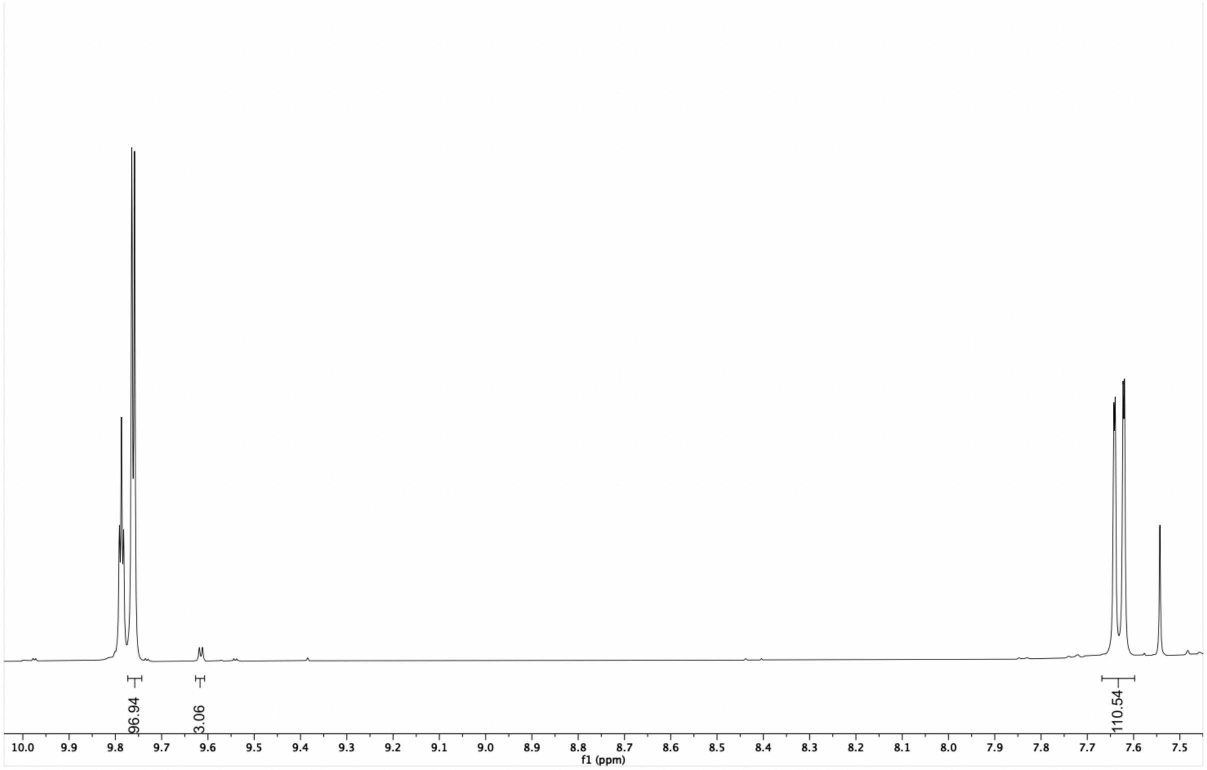

Screening results:

	Reaction (**I**)			Reaction (**II**)			Reaction (**III**)			Reaction (**IV**)		
	conv. (%)	d.r. (x:1)	ee^{syn} (%)	conv. (%)	d.r. (x:1)	ee^{syn} (%)	conv. (%)	d.r. (x:1)	ee^{syn} (%)	conv. (%)	d.r. (x:1)	ee^{syn} (%)
**P1**	100	27	97	100	11	92	73	21	94	100	32	96
**P2**	44	18	68	49	10	57	36	15	64	93	23	84
**P3**	73	23	-86	85	12	-82	34	16	-83	88	32	-89
**P4**	77	33	-73	83	18	-82	58	30	-81	100	28	-94
**P5**	100	28	98	100	11	94	87	21	98	100	35	97
**P6**	31	9	-60	33	3	-38	32	7	-58	58	15	-72
**P7**	97	23	-88	100	9	-84	73	18	-86	94	33	-91
**P8**	100	13	-83	100	8	-79	95	11	-79	100	17	-88
**P9**	100	23	78	100	8	90	93	18	63	100	22	-65
**P10**	100	26	99	100	10	98	96	23	97	100	24	-
**P11**	100	30	97	100	11	99	84	22	95	100	29	95
**P12**	9	25	94	10	14	81	22	20	90	64	35	94
**P13**	100	25	92	100	9	89	82	17	88	100	29	94
**P14**	100	23	97	100	13	94	89	16	93	100	27	96
**P15**	100	26	94	100	9	90	81	18	90	100	30	95
**P16**	100	26	96	100	14	94	79	17	92	100	34	96
**P17**	2	24	40	8	14	81	1	18	-	4,5	34	-
**P18**	60	40	92	68	20	92	23	26	90	49	44	92
**P19**	97	14	-83	94	8	-81	80	13	-80	100	16	-86
**P20**	85	18	90	70	9	86	70	12	89	100	17	94
**P21**	100	23	-83	100	6	-84	89	19	-80	100	22	-86
**P22**	74	14	76	93	9	77	67	10	75	97	20	78
**P23**	38	10	80	25	2,5	58	26	7	83	63	14	92
**P24**	20	11	68	20	3,3	65	19	7	56	7	15	55
**P25**	34	11	79	28	2,3	58	40	8	76	97	15	84
**P26**	50	12	88	79	6	78	34	8	86	80	13	92
**P27**	46	18	90	33	4,5	64	46	10	89	100	20	93
**P28**	6	6	85	5	1,8	81	3	5	55	8	13	90
**P29**	6	8	60	4	2,1	28	3	7	52	7	14	76
**P30**	3	6	-69	2	1,7	-56	1	6	-43	5	13	-78
**P31**	5	10	-78	2	1,4	-57	2	8	-67	6	13	-81
**P32**	11	11	60	64	4,3	80	37	8	70	16	14	87
**P33**	7	12	57	6	3,3	14	8	8	50	23	16	80
**P34**	13	9	-66	32	4,3	-60	17	7	-65	71	11	-80
**P35**	3	11	-68	1	2,6	-19	4	9	-49	28	16	-85
**P36**	43	12	77	46	4,6	64	39	7	69	64	17	87
**P37**	31	12	87	39	6	79	27	9	84	40	16	91
**P38**	16	6	-50	17	3,3	-33	15	4	-50	22	12	-67
**P39**	47	11	-78	63	6	-77	38	7	-70	64	16	-85
**P40**	76	16	84	30	4,4	60	61	10	77	100	20	90
**P41**	78	21	87	29	5,2	67	57	14	82	100	26	93
**P42**	55	15	85	64	15	84	30	9	80	61	16	90
**P43**	47	12	82	79	6	79	33	8	78	69	18	90
**P44**	1	12	-39	1	3,8	-45	0,5	12	23	-	-	-59
**P45**	4	10	-50	3	3	-44	3	7	-28	13	13	-60
**P46**	1	-	-27	0,2	7	-	-	-	32	-	-	-
**P47**	3	15	-51	6	7	-48	1	12	26	28	28	-69
**P48**	3	7	-39	2	2	-35	2	6	-17	16	16	-59
**P49**	3	12	-46	3	3,5	-38	2	8	-30	3	18	-59
**P50**	2	9	-10	2	3,4	-25	2	8	45	24	24	-38

Screening of the Annulation Reaction

General Procedure and Results of the Reaction Optimization:

In 0.1 ml of ***solvent***, 5 mol% of the ***tripeptide*** catalyst (2.08 µmol, 0.05 equiv.) and 5 mol% NMM (0.23µl, 2.08 µmol, 0.05 equiv.) were dissolved. A stock solution of 3-methyl crotonaldehyde (**xx** equiv.) and 2,6-dimethylbenzoquinone (**yy** equiv.) in 0.15 ml of ***solvent*** was added. The reaction mixture was stirred at r.t. for **zz** h. A sample (50 µl) was taken from the reaction mixture to estimate the conversion by ^{1}H-NMR spectroscopy. The crude mixture was purified by preparative TLC (silica, *n*-hexane/EtOAc 9:1) and the enantiomeric excess was determined by SFC on a chiral stationary phase.

^{1}H-NMR (400 MHz, $CDCl_3$) δ 7.07 (dt, *J* = 5.7, 0.8 Hz, 1H), 6.77 (q, *J* = 1.5 Hz, 1H), 6.00 (ddq, *J* = 5.8, 3.0, 1.5 Hz, 1H), 2.56 (dt, *J* = 18.4, 2.2 Hz, 1H), 2.43 (d, *J* = 18.4 Hz, 1H), 2.06 (d, *J* = 1.5 Hz, 3H), 1.97 (t, *J* = 1.7 Hz, 3H), 1.19 (s, 3H). **SFC** IC-3 column, CO_2/MeOH 95:5, 2 ml/min, T = 40°C, λ = 214 nm, t_R = 1.66 min ((*R*)-enantiomer) and 1.82 min ((*S*)-enantiomer).

Table 1 Peptide and solvent screening for the annulation reaction.

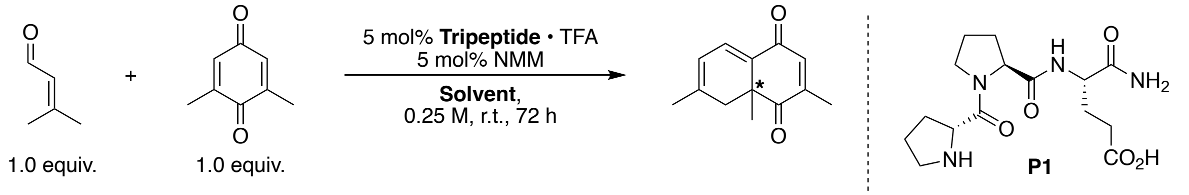

Entry	Catalyst	Solvent	Conv.[a] [%]	*ee*[b] [%]
1	**P1**	MeOH	quant.	57
2	H-DPro-DLeu-DGln-NH_2	MeOH	23	24
3	H-DPro-DTyr-Gln-NH_2	MeOH	11	26
4	H-DPro-Glu-(4*S*)Flp[c]-NH_2	MeOH	45	53
5	H-DPro-Pro-Asn-NH_2	MeOH	quant.	54
6	**P1**	EtOH	94	64
7	**P1**	^{i}PrOH	77	64
8	**P1**	H_2O	41	29
9	**P1**	Brine	25	49
10	**P1**	DMSO	39	18
11	**P1**	DMF	39	15
12	**P1**	NMP	17	15
13	**P1**	THF	55	31
14	**P1**	1,4-Dioxane	50	11
15	**P1**	AcOEt	78	14
16	**P1**	MeCN	72	35
17	**P1**	$CHCl_3$	65	80
18	**P1**	CH_2Cl_2	14	64
19	**P1**	Toluene	7[d]	44

[a] Conversions determined by ^{1}H-NMR spectroscopy from the crude reaction mixture; [b] Enantiomeric excess determined by SFC on a chiral stationary phase. [c] (4*S*)-Flp: (2*S*,4*S*)-fluoroproline. [d] Low conv. due to low solubility of the peptide in toluene.

Table 2 Stoichiometry and solvent mixture screening for the annulation reaction.

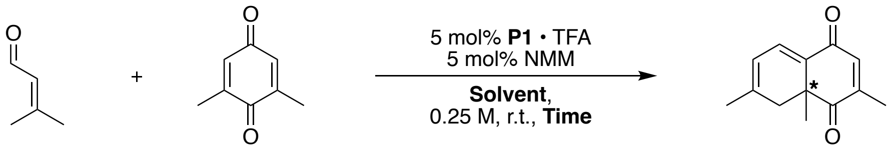

Entry	**Ald.** (equiv.)	**Quin.** (equiv.)	Solvent	Time [h]	Conv.[a] [%]	*ee*[b] [%]
1	3.0	1.0	MeOH	24	quant.	56
2	2.0	1.0	MeOH	24	quant.	56
3	1.5	1.0	MeOH	24	quant.	56
4	1.0	1.0	MeOH	24	45	55
5	1.0	1.5	MeOH	24	89	56
6	1.0	2.0	MeOH	24	88	56
7	1.0	3.0	MeOH	24	91	56
8	1.5	1.0	$CHCl_3$/MeOH 9:1	22	84	86
9	1.5	1.0	$CHCl_3$/EtOH 9:1	22	83	86
10	1.5	1.0	$CHCl_3$/^{i}PrOH 9:1	22	86	86
11	1.5	1.0	$CHCl_3$/MeOH 9:2	22	92	83
12	1.5	1.0	$CHCl_3$/EtOH 9:2	22	92	83
13	1.5	1.0	$CHCl_3$/^{i}PrOH 9:2	22	91	83

[a] Conversions determined by ^{1}H-NMR spectroscopy from the crude reaction mixture; [b] Enantiomeric excess determined by SFC on a chiral stationary phase.

General Screening Conditions for Annulation Reactions with UTS-Peptides and Predicted Peptides:

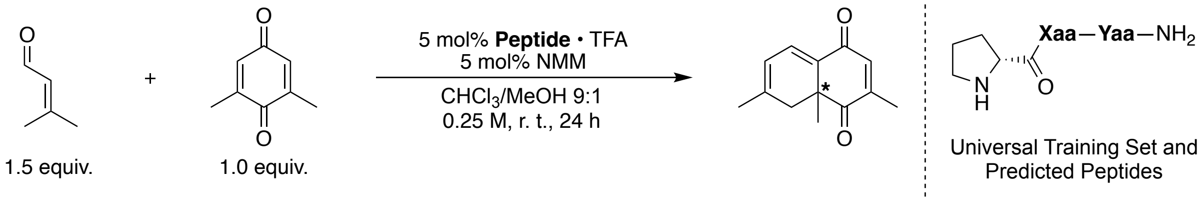

In 0.1 ml of a $CHCl_3$/MeOH 9:1, 5 mol% of tripeptide catalyst (2.08 µmol, 0.05 equiv.) and 5 mol% of NMM (0.23µl, 2.08 µmol, 0.05 equiv.) were dissolved. A stock solution of 3-methyl crotonaldehyde (**11**) (5.8 µl, 62.5 µmol, 1.5 equiv.) and 2,6-dimethylbenzoquinone (**12**) (5.7 mg, 41.7 µmol, 1 equiv.) in 0.15 ml of $CHCl_3$/MeOH 9:1 was added. The reaction mixture was stirred at r.t. for 24 h. A sample (50 µl) was taken from the reaction mixture to estimate the conversion by ^{1}H-NMR spectroscopy. The crude mixture was purified by preparative TLC (silica, *n*-hexane/EtOAc 9:1) and the enantiomeric excess was determined by SFC on a chiral stationary phase.

Screening Data of UTS-Peptide Screening:

Note: Enantiomeric excess with and excess of the (*S*)-enantiomer are defined as positive "+" ee, with and excess of the (*R*)-enantiomer as negative "–". The analytical data is in agreement with the literature.[10]

Table 3 Screening Results of Annulation Reaction with UTS-Peptides.

Peptide	*Sequence*	*Conv. (%) in 24h[a]*	*ee (SFC) in %[b]*
**UTS-1**	H-D-Pro-D-Pro-D-Pro-NH_2	19	52
**UTS-2**	H-D-Pro-L-(4*S*)-Flp-D-Pro-NH_2	17	62
**UTS-3**	H-D-Pro-L-Leu-D-Pro-NH_2	8	64
**UTS-4**	H-D-Pro-D-Leu-D-Pro-NH_2	5	-35
**UTS-5**	H-D-Pro-L-Gln-D-Pro-NH_2	6	58
**UTS-6**	H-D-Pro-D-Gln-D-Pro-NH_2	10	-47
**UTS-7**	H-D-Pro-L-Glu-D-Pro-NH_2	9	61
**UTS-8**	H-D-Pro-D-Glu-D-Pro-NH_2	14	-34
**UTS-9**	H-D-Pro-L-Tyr-D-Pro-NH_2	8	57
**UTS-10**	H-D-Pro-D-Tyr-D-Pro-NH_2	6	-21
**UTS-11**	H-D-Pro-CyLeu-D-Pro-NH_2	6	9
**UTS-12**	H-D-Pro-D-Ind-D-Pro-NH_2	6	-26
**UTS-13**	H-D-Pro-D-Pro-L-(4*S*)Flp-NH_2	18	50
**UTS-14**	H-D-Pro-D-Pro-L-Leu-NH_2	24	58
**UTS-15**	H-D-Pro-D-Pro-D-Leu-NH_2	15	57
**UTS-16**	H-D-Pro-D-Pro-L-Gln-NH_2	21	56
**UTS-17**	H-D-Pro-D-Pro-D-Gln-NH_2	24	60
**UTS-18**	H-D-Pro-D-Pro-L-Glu-NH_2	62	58
**UTS-19**	H-D-Pro-D-Pro-D-Glu-NH_2	91	71
**UTS-20**	H-D-Pro-D-Pro-L-Tyr-NH_2	29	56
**UTS-21**	H-D-Pro-D-Pro-D-Tyr-NH_2	20	59
**UTS-22**	H-D-Pro-D-Pro-CyLeu-NH_2	21	51
**UTS-23**	H-D-Pro-D-Pro-Abz-NH_2	19	49
**UTS-24**	H-D-Pro-D-Pro-Ind-NH_2	87	55
**UTS-25**	H-D-Pro-L-(4*S*)-Flp-L-(4*S*)-Flp-NH_2	20	59
**UTS-26**	H-D-Pro-L-(4*S*)-Flp-L-Leu-NH_2	49	75
**UTS-27**	H-D-Pro-L-(4*S*)-Flp-D-Leu-NH_2	25	69
**UTS-28**	H-D-Pro-L-(4*S*)-Flp-L-Gln-NH_2	64	90
**UTS-29**	H-D-Pro-L-(4*S*)-Flp-D-Gln-NH_2	51	84
**UTS-30**	H-D-Pro-L-(4*S*)-Flp-L-Glu-NH_2	99	91
**UTS-31**	H-D-Pro-L-(4*S*)-Flp-D-Glu-NH_2	87	88
**UTS-32**	H-D-Pro-L-(4*S*)-Flp-L-Tyr-NH_2	35	76
**UTS-33**	H-D-Pro-L-(4*S*)-Flp-D-Tyr-NH_2	22	66
**UTS-34**	H-D-Pro-L-(4*S*)-Flp-CyLeu-NH_2	26	57
**UTS-35**	H-D-Pro-L-(4*S*)-Flp-D-Ind-NH_2	24	70

**UTS-36**	H-D-Pro-L-Leu-L-(4*S*)-Flp-NH_2	5	67
**UTS-37**	H-D-Pro-D-Leu-L-(4*S*)-Flp-NH_2	5	-60
**UTS-38**	H-D-Pro-L-Gln-L-(4*S*)-Flp-NH_2	7	58
**UTS-39**	H-D-Pro-D-Gln-L-(4*S*)-Flp-NH_2	5	-78
**UTS-40**	H-D-Pro-L-Glu-L-(4*S*)-Flp-NH_2	10	59
**UTS-41**	H-D-Pro-D-Glu-L-(4*S*)-Flp-NH_2	5	-62
**UTS-42**	H-D-Pro-L-Tyr-L-(4*S*)-Flp-NH_2	4	51
**UTS-43**	H-D-Pro-D-Tyr-L-(4*S*)-Flp-NH_2	4	-59
**UTS-44**	H-D-Pro-CyLeu-L-(4*S*)-Flp-NH_2	1	62
**UTS-45**	H-D-Pro-D-Ind-L-(4*S*)-Flp-NH_2	3	-48
**UTS-46**	H-D-Pro-L-Leu-L-Leu-NH_2	5	23
**UTS-47**	H-D-Pro-L-Leu-D-Leu-NH_2	7	48
**UTS-48**	H-D-Pro-L-Leu-L-Gln-NH_2	5	64
**UTS-49**	H-D-Pro-L-Leu-D-Gln-NH_2	6	43
**UTS-50**	H-D-Pro-L-Leu-L-Glu-NH_2	77	27
**UTS-51**	H-D-Pro-L-Leu-D-Glu-NH_2	75	32
**UTS-52**	H-D-Pro-L-Leu-L-Tyr-NH_2	4	35
**UTS-53**	H-D-Pro-L-Leu-D-Tyr-NH_2	4	4
**UTS-54**	H-D-Pro-L-Leu-CyLeu-NH_2	8	51
**UTS-55**	H-D-Pro-L-Leu-Abz-NH_2	5	56
**UTS-56**	H-D-Pro-L-Leu-D-Ind-NH2	5	54
**UTS-57**	H-D-Pro-D-Leu-L-Leu-NH_2	7	-59
**UTS-58**	H-D-Pro-L-Gln-L-Leu-NH_2	6	32
**UTS-59**	H-D-Pro-D-Gln-L-Leu-NH_2	8	-77
**UTS-60**	H-D-Pro-L-Glu-L-Leu-NH_2	16	25
**UTS-61**	H-D-Pro-D-Glu-L-Leu-NH_2	11	-53
**UTS-62**	H-D-Pro-L-Tyr-L-Leu-NH_2	7	28
**UTS-63**	H-D-Pro-D-Tyr-L-Leu-NH_2	5	-49
**UTS-64**	H-D-Pro-CyLeu-L-Leu-NH_2	10	-22
**UTS-65**	H-D-Pro-Abz-L-Leu-NH_2	5	8
**UTS-66**	H-D-Pro-D-Ind-L-Leu-NH_2	7	-59
**UTS-67**	H-D-Pro-D-Leu-D-Leu-NH_2	3	13
**UTS-68**	H-D-Pro-D-Leu-L-Gln-NH_2	3	-32
**UTS-69**	H-D-Pro-D-Leu-D-Gln-NH_2	3	-19
**UTS-70**	H-D-Pro-D-Leu-L-Glu-NH_2	58	-61
**UTS-71**	H-D-Pro-D-Leu-D-Glu-NH_2	24	-44
**UTS-72**	H-D-Pro-D-Leu-L-Tyr-NH_2	5	-46
**UTS-73**	H-D-Pro-D-Leu-D-Tyr-NH2	4	3
**UTS-74**	H-D-Pro-D-Leu-CyLeu-NH_2	6	-39
**UTS-75**	H-D-Pro-D-Leu-Abz-NH_2	3	-40
**UTS-76**	H-D-Pro-D-Leu-D-Ind-NH_2	2	15

**UTS-77**	H-D-Pro-L-Gln-D-Leu-NH_2	4	30
**UTS-78**	H-D-Pro-D-Gln-D-Leu-NH_2	6	-35
**UTS-79**	H-D-Pro-L-Glu-D-Leu-NH_2	11	33
**UTS-80**	H-D-Pro-D-Glu-D-Leu-NH_2	13	-9
**UTS-81**	H-D-Pro-L-Tyr-D-Leu-NH_2	8	40
**UTS-82**	H-D-Pro-D-Tyr-D-Leu-NH_2	3	19
**UTS-83**	H-D-Pro-CyLeu-D-Leu-NH_2	3	22
**UTS-84**	H-D-Pro-Abz-D-Leu-NH_2	4	37
**UTS-85**	H-D-Pro-D-Ind-D-Leu-NH_2	3	7
**UTS-86**	H-D-Pro-L-Gln-L-Gln-NH_2	8	51
**UTS-87**	H-D-Pro-L-Gln-D-Gln-NH_2	6	31
**UTS-88**	H-D-Pro-L-Gln-L-Glu-NH_2	51	19
**UTS-89**	H-D-Pro-L-Gln-D-Glu-NH_2	66	27
**UTS-90**	H-D-Pro-L-Gln-L-Tyr-NH_2	6	21
**UTS-91**	H-D-Pro-L-Gln-D-Tyr-NH_2	5	32
**UTS-92**	H-D-Pro-L-Gln-CyLeu-NH_2	5	37
**UTS-93**	H-D-Pro-L-Gln-Abz-NH_2	6	32
**UTS-94**	H-D-Pro-L-Gln-D-Ind-NH_2	4	25
**UTS-95**	H-D-Pro-D-Gln-L-Gln-NH_2	5	-54
**UTS-96**	H-D-Pro-L-Glu-L-Gln-NH_2	15	48
**UTS-97**	H-D-Pro-D-Glu-L-Gln-NH_2	11	-38
**UTS-98**	H-D-Pro-L-Tyr-L-Gln-NH_2	9	57
**UTS-99**	H-D-Pro-D-Tyr-L-Gln-NH_2	4	-37
**UTS-100**	H-D-Pro-CyLeu-L-Gln-NH_2	5	38
**UTS-101**	H-D-Pro-Abz-L-Gln-NH_2	3	14
**UTS-102**	H-D-Pro-D-Ind-L-Gln-NH_2	3	-40
**UTS-103**	H-D-Pro-D-Gln-D-Gln-NH_2	3	-39
**UTS-104**	H-D-Pro-D-Gln-L-Glu-NH_2	56	-72
**UTS-105**	H-D-Pro-D-Gln-D-Glu-NH_2	16	-39
**UTS-106**	H-D-Pro-D-Gln-L-Tyr-NH_2	8	-65
**UTS-107**	H-D-Pro-D-Gln-D-Tyr-NH_2	6	-29
**UTS-108**	H-D-Pro-D-Gln-CyLeu-NH_2	8	-78
**UTS-109**	H-D-Pro-D-Gln-Abz-NH_2	4	-35
**UTS-110**	H-D-Pro-D-Gln-D-Ind-NH_2	6	-40
**UTS-111**	H-D-Pro-L-Glu-D-Gln-NH_2	11	30
**UTS-112**	H-D-Pro-D-Glu-D-Gln-NH_2	10	-35
**UTS-113**	H-D-Pro-L-Tyr-D-Gln-NH_2	8	34
**UTS-114**	H-D-Pro-D-Tyr-D-Gln-NH_2	5	-19
**UTS-115**	H-D-Pro-CyLeu-D-Gln-NH_2	6	-45
**UTS-116**	H-D-Pro-Abz-D-Gln-NH_2	4	33
**UTS-117**	H-D-Pro-D-Ind-D-Gln-NH_2	4	-14

**UTS-118**	H-D-Pro-L-Glu-L-Glu-NH_2	59	27
**UTS-119**	H-D-Pro-L-Glu-D-Glu-NH_2	72	30
**UTS-120**	H-D-Pro-L-Glu-L-Tyr-NH_2	15	19
**UTS-121**	H-D-Pro-L-Glu-D-Tyr-NH_2	11	33
**UTS-122**	H-D-Pro-L-Glu-CyLeu-NH_2	7	36
**UTS-123**	H-D-Pro-L-Glu-Abz-NH_2	10	38
**UTS-124**	H-D-Pro-L-Glu-D-Ind-NH_2	10	26
**UTS-125**	H-D-Pro-D-Glu-L-Glu-NH_2	51	-52
**UTS-126**	H-D-Pro-L-Tyr-L-Glu-NH_2	69	30
**UTS-127**	H-D-Pro-D-Tyr-L-Glu-NH_2	43	-45
**UTS-128**	H-D-Pro-CyLeu-L-Glu-NH_2	15	-12
**UTS-129**	H-D-Pro-Abz-L-Glu-NH_2	9	25
**UTS-130**	H-D-Pro-D-Ind-L-Glu-NH_2	42	-63
**UTS-131**	H-D-Pro-D-Glu-D-Glu-NH_2	22	-23
**UTS-132**	H-D-Pro-D-Glu-L-Tyr-NH_2	10	-44
**UTS-133**	H-D-Pro-D-Glu-D-Tyr-NH_2	10	-13
**UTS-134**	H-D-Pro-D-Glu-CyLeu-NH_2	8	-52
**UTS-135**	H-D-Pro-D-Glu-Abz-NH_2	10	-43
**UTS-136**	H-D-Pro-D-Glu-D-Ind-NH_2	12	-12
**UTS-137**	H-D-Pro-L-Tyr-D-Glu-NH_2	71	31
**UTS-138**	H-D-Pro-D-Tyr-D-Glu-NH_2	31	-32
**UTS-139**	H-D-Pro-CyLeu-D-Glu-NH_2	73	-57
**UTS-140**	H-D-Pro-Abz-D-Glu-NH_2	6	39
**UTS-141**	H-D-Pro-D-Ind-D-Glu-NH_2	13	-29
**UTS-142**	H-D-Pro-L-Tyr-L-Tyr-NH_2	6	37
**UTS-143**	H-D-Pro-L-Tyr-D-Tyr-NH_2	6	45
**UTS-144**	H-D-Pro-L-Tyr-CyLeu-NH_2	9	38
**UTS-145**	H-D-Pro-L-Tyr-Abz-NH_2	7	46
**UTS-146**	H-D-Pro-L-Tyr-D-Ind-NH_2	8	43
**UTS-147**	H-D-Pro-D-Tyr-L-Tyr-NH_2	5	-6
**UTS-148**	H-D-Pro-CyLeu-L-Tyr-NH_2	6	-25
**UTS-149**	H-D-Pro-Abz-L-Tyr-NH_2	6	18
**UTS-150**	H-D-Pro-D-Ind-L-Tyr-NH_2	5	-57
**UTS-151**	H-D-Pro-D-Tyr-D-Tyr-NH_2	3	11
**UTS-152**	H-D-Pro-D-Tyr-CyLeu-NH_2	8	-50
**UTS-153**	H-D-Pro-D-Tyr-Abz-NH_2	4	12
**UTS-154**	H-D-Pro-D-Tyr-D-Ind-NH_2	2	27
**UTS-155**	H-D-Pro-CyLeu-D-Tyr-NH_2	4	18
**UTS-156**	H-D-Pro-Abz-D-Tyr-NH_2	5	45
**UTS-157**	H-D-Pro-D-Ind-D-Tyr-NH_2	4	0
**UTS-158**	H-D-Pro-CyLeu-CyLeu-NH_2	11	-18

**UTS-159**	H-D-Pro-CyLeu-D-Ind-NH_2	3	36
**UTS-160**	H-D-Pro-D-Ind-CyLeu-NH_2	5	-51
**UTS-161**	H-D-Pro-D-Ind-D-Ind-NH_2	3	24

[a] Conversions determined by ^{1}H-NMR spectroscopy from the crude reaction mixture; [b] Enantiomeric excess determined by SFC on a chiral stationary phase.

3. Computational Methods

All data, modeling results, and example code are available on our Zenodo repository at DOI 10.5281/zenodo.10008997.

Combinatorial, in silico Library Generation.

A database of tripeptides was constructed combinatorically from a set of 174 amino acids. The amino acids were selected on the basis of commercial availability. The structure files for the fragments used in library constructure are provided in mol2 format in the Xaa folder provided in the supplementary folders. The first residue of each catalyst was held constant as L-proline. Next, a preliminary set of dipeptides was constructed by appending the 174 amino acids in the amino acid database to the first residue (analogous to running the 174 peptide couplings *in silico*). Finally, the dipeptide set was converted to a tripeptide set through coupling each of the 174 amino acids with each of the 174 dipeptides, resulting in 30,276 possible tripeptide catalysts. At the outset, each of the amino acid structures were hand drawn, with structures modified to contain a label for the proper attachment point. This process was accomplished using ccheminfolib's in silico library construction features.[11] The resulting tripeptides were then loaded into Schrodinger suit and minimized with MacroModel using the OPLS3 force field, selecting chloroform as solvent.[12] After minimization, a conformer library of each catalyst was obtained with Macromodel with the same force field and solvent model, setting the energy window to 7 kcal / mol, setting minimum RMSE difference to 2 Å, and the maximum number of conformers to 350. All other parameters were set as defaults. Upon completion of the conformer search, all compounds were aligned with Maestro to the stereocenter of the L-proline on the first residue. After alignment, a common grid was calculated with ccheminfolib, and ASO descriptors were calculated (example code available via GitLab).[13] To calculate "fragment based" descriptors, the individual amino acids were subjected to the same workflow, aligning the amino acids to the nitrogen, carboxylate carbon, and the carbon α to the carboxylate carbon.

Descriptor Calculations.

Calculation of Average Steric Occupancy (ASO) descriptors is identical to what has been previously reported using ccheminfolib. Additionally, Average Electronic Indicator Field (AEIF) descriptors were calculated as have previously been reported,[14] using Gasteiger charges calculated with OpenBabel.[15] This workflow was performed for both the tripeptides and the individual amino acids. In addition to these descriptors, a conformer averaged hydrogen bonding field (AHBF) was calculated. This field is calculated in a conceptually similar way to the ASO descriptors, which operate by iterating through all conformers of a molecule. For the AHBF descriptors, if a grid point falls withing the Van der Waals radius of a hydrogen atom bound to an electronegative heteroatom, that grid point receives a value of 1. If not, that grid point receives a value of 0. This process is repeated for every conformer and averaged. As such, in the end each grid point will have a value ranging from 0 (never overlaps with a hydrogen atom capable of being a hydrogen bond donor) or 1 (always overlaps with a hydrogen atom capable of being a hydrogen bond donor). The code available for completing this calculation is available in ccheminfolib.[12] The descriptors used for the amino acid based clustering analysis can be found in the supplemental folder Amino_Acid_Descriptors. The ASO, AEIF, and AHBF descriptors can be found in the ASO, AEIF, and HBond supplemental folders, respectively. Truncated ASO and AEIF descriptors using radius away from the active center (to be discussed in the round 3 modeling section) can be found in the Radius_Descriptors supplemental folder.

Modeling the Enantioselective 1,4-Addition to Nitroolefins.

In this case study, the data was randomly divided using an 85:15 train / test partition (164 and 29 reactions, respectively). Notably, the selectivities measured experimentally for catalysts with D-proline as the first residue were "corrected" such that it reflects the value the catalyst which would have been obtained had its enantiomer been used to be consistent with the in silico library composition (i.e. the sign of % ee was flipped for catalysts with D-proline as the first residue). For each catalyst, the AEIF and ASO profiles were flattened and concatenated. Next, dimension with zero variance were dropped using a variance threshold in SKlearn.[15] The descriptor set was scaled using StandardScaler in SKlearn. Next, f-regression was used to select the top 500 features (also implemented in SKlearn). Using this reduced feature set, a 3-component PLS model was constructed and evaluated internally (5-fold cross validation of the training data) and externally with the test set. This process was repeated with 5 different random train/test partitions for both enantioselectivity and diastereoselectivity prediction, and the summary of the results are given in Figure 1 of the main text.

Universal Training Set Selection

With the descriptor set validated, we next set out selecting a universal training set for tripeptide catalysts. For cost considerations, we reasoned the most effective way to generate a large, diverse set of peptide catalysts would be to select the most representative set of amino acids and combinatorically generate a training set of possible tripeptides from those selections. To do this, we used the "fragment based" descriptors, i.e. the ASO and AEIF descriptors for the individual amino acid residues. These descriptors were first scaled and zero variance descriptors removed. Principal component analysis was performed, returning the first 20 principal components. Finally, KMeans clustering was performed in SKlearn using 2-50 clusters, plotting the average distortion vs number of clusters. Using this analysis, an optimal number of 10 clusters was identified. The amino acid nearest to the centroid of each cluster was identified as a representative. In addition to these 10 amino acids, three additional selections were made. Two of these selections were based on our own knowledge of the how the ASO and AEIF descriptors perform in unsupervised selection protocols. In the selection process, both D and L glutamine were selected as representative amino acids. In the unsupervised analysis, both of these amino acids are nearest neighbors to their glutamic acid analogs. Despite the similarity of these molecules, both clearly have significantly different chemical behavior. Thus, in order to allow our supervised models to learn the significance of the dimensions distinguishing these molecules, both D and L glutamic acid were added to the set of selected amino acids. In addition, one of the next selections if more clusters were taken were L-proline or its analogs. Because a D-proline analog was selected and we recognized a pattern in the unsupervised selection process in which both enantiomers of amino acids were selected, we made the decision to add L-proline to the set. In addition to these considerations, these residues were also demonstrated to be associated with highly selective catalysts in previous studies.

Supervised Learning Campaign
Round 1 Modeling

In the first round of modeling, our efforts focused primarily on the use of neural networks with Keras.[16] However, prior to construction of the neural network, the dimensionality of the input space was dramatically reduced. First, the data was partitioned into train, validation, and test sets (100, 31, and 30 datapoints, respectively). Next, using the training data, the data was passed through

a variance threshold, removing all dimensions which had zero variance in the training data. Next, the best 3000 dimensions were selected using f_regression). The dimensionality was further reduced via recursive feature elimination using a ridge model. This reduced the input space to 1500 dimensions. Next, a lasso model was constructed to further reduce the dimensionality of the input space. At this point, all modeling steps were performed using SKLearn. The number of input dimensions were then treated as a hyperparameter, along with the parameters of the neural network. The input layer (and number of dimensions output from the Lasso model) was allowed to be any number ranging from 10 to 999. The neural network employed consisted of an input layer, two hidden layers, and one output layer. The activation functions for each layer were tunable, with activation functions relu, elu, selu, softplus, linear, and sigmoid tested. The number of nodes for each hidden layer was set to a number between 2 and the maximum of the number of input dimensions divided by 1.25 (the hypothesis being a smaller number of nodes in the hidden layers would reduce the degrees of freedom of the model). Additionally, the number of epochs was tuned as a hyperparameter from 5 to 25. This number was intentionally small, as we also attempted to use undertraining as a strategy to reduce overfitting. Dropouts in hidden layers were also used to reduce overfitting, assigning values ranging from 0 to 0.3 for each hidden layer. All these parameters were tuned randomly, randomly generating 1000 models. The best model was then selected on the basis of the validation MAE. The best model had linear, softplus, and selu activation functions (respectively), 110 nodes in the first hidden layer, 53 nodes in the second hidden layer, dropouts of 0.18 and 0.01, respectively, was trained for 10 epochs, and had 342 dimensions as input. In addition to the enantioselectivity models discussed in the main text, models for yield were also constructed. The predicted and observed values for both enantioselectivity and yield, as well as the summary statistics and parity plots for these models, are available in the file Round_1.xlsx.

Round 2 Modeling

The selections for Round 1 were made primarily on the basis of predicted yield. As such, these predictions were viewed as “high-risk, high-reward” selections. Because of the lackluster performance of the first round, in our second rounds we elected to make the following changes: (1) we would use an ensembling approach to make many models, allowing us to use the standard deviation in predicted outcome for a given catalyst as a certainty metric and (2) we would explore more simple models, as neural networks are likely too complicated to generalize well with the limited training data available to us. In this endeavor, we also set out to use only the training data from the original survey in model construction. The catalysts selected in round 1 would then serve as an additional test set. With this outline, we performed three separate modeling endeavors. The first was to use only Projection to Latent Structure (PLS) models. The next was to use an ensemble of different types of regression models. The final was to use an ensemble of neural networks. In these cases, the data partitioning scheme was also changed to a “leave-two-amino-acids-out” approach. The logic for this decision was to use data partitioning as a method to overcome the combinatorial nature of the original data set. In this scheme, two amino acids were selected as “left out” amino acids, meaning no tripepetide which contained one of those amino acids was allowed in the training set. Tripeptides containing one of these amino acids comprised the validation set, and tripeptides containing the other comprised the test set. If an amino acid contained both, it was placed in the test set. This process was constructed until every amino acid had been left out. The aggregate test set scores were then used to compare the three different modeling strategies. Additionally, for each partition, a different model was trained. Each model was used to make predictions for the entire *in silico* library – as such, each library member is assigned many predictions. The average of the predictions was used as the predicted value for each catalyst, and

the standard deviation in predictions used as a certainty metric. In this case, lower standard deviation in predictions is viewed as a more certain prediction because there is less disagreement between the models. As a final comparison between models, the seven predictions from round 1 were also compared as a final test using the averaged predicted value.

Prior to modeling in any case studies, additional dimensionality reduction procedures were used. In this case, we decided to use chemical information to inform which grid points were selected. Specifically, the role of the proline at the first residue is well established as the catalytically active center. As such, the most important regions of chemical space are likely those around this center. As such, the full grid for ASO and AEIF descriptors were truncated at increasing radii from the nitrogen atom of the first residue. Five through fifteen angstrom cutoffs were used to return a smaller, more focused grid. Next, for the ASO and AEIF descriptors separately, a lasso model was used to reduce the dimensionality of the input space, followed by the construction of 3-component PLS using the same partitioning scheme described above. The test set scores were then used to decide an optimal radius for the grid, which was found to be 13 angstroms for the AEIF and 14 angstroms for the ASO. These feature vectors were concatenated for all tripeptides prior to being fed into the modeling workflow described above for constructing the three ensembles (workflow represented in figure SX).

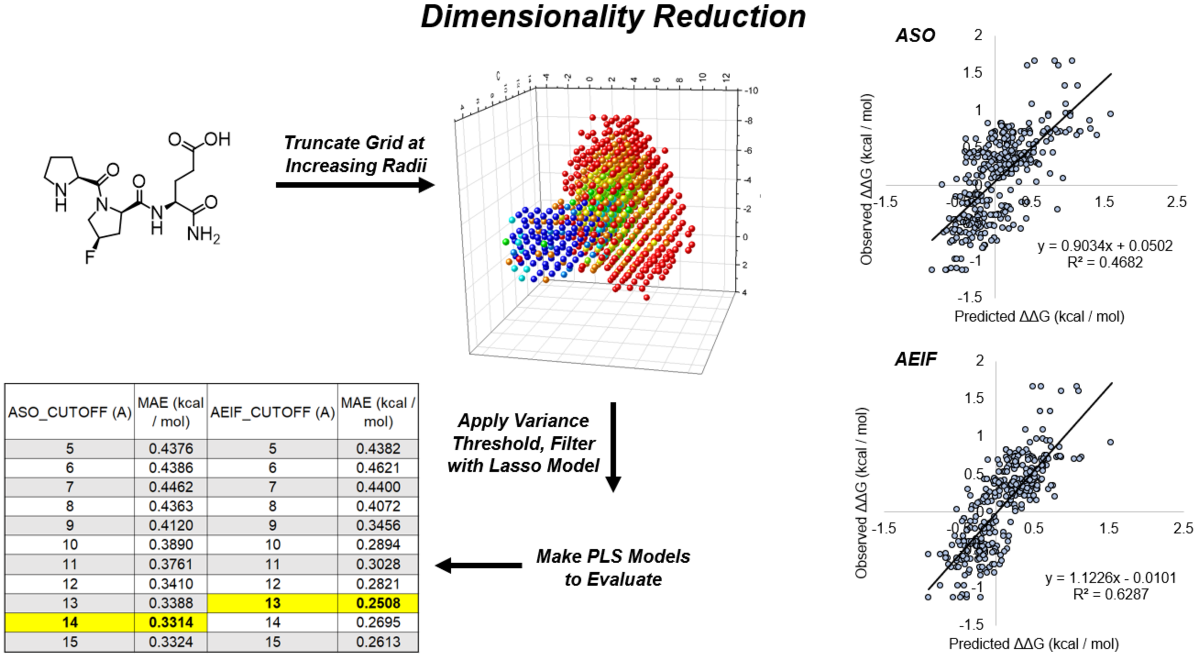

ASO_CUTOFF (A)	MAE (kcal / mol)	AEIF_CUTOFF (A)	MAE (kcal / mol)
5	0.4376	5	0.4382
6	0.4386	6	0.4621
7	0.4462	7	0.4400
8	0.4363	8	0.4072
9	0.4120	9	0.3456
10	0.3890	10	0.2894
11	0.3761	11	0.3028
12	0.3410	12	0.2821
13	0.3388	**13**	**0.2508**
**14**	**0.3314**	14	0.2695
15	0.3324	15	0.2613

Figure SX. Radius-based dimensionality reduction workflow (left) and parity plots for the test sets using ASO and AEIF descriptors alone (right).

The ensemble of PLS models performed the best when predicting the outcome for the peptides previously selected in Round 1 (MAE = 0.33 kcal / mol) , followed by the mixed ensemble of regressors (MAE = 0.41 kcal / mol), followed by the neural network ensemble (MAE = 0.60 kcal / mol), supporting our hypothesis that using simpler models would result in more general predictions. A summary of the model performance, combined test set predictions, redone round 1 predictions, and parity plots for each modeling approach is provided in the supplementary file Round_2.xlsx.

Round 3 Modeling

Given the relative success of PLS models with respect to our other attempts, the final round of modeling only used the ensemble of 3-component PLS models. In this case, a leave-one-amino-acid out approach was used, in which all tripeptides containing one amino acid were left out as a test set and all others were use to train the model. In addition, all catalysts from round 1 were included in the training data, but all round 2 catalysts were held out as an additional test set. All other modeling, prediction, and uncertainty estimation protocols were identical to the PLS ensemble in round 2. The results from this round are provided in the Round_3.xlsx supplementary file.

Screening Data of First Round Predicted Peptides:

Table 4 Synthesized tripeptides from the first round of machine learning predictions. The table shows the predicted enantioselectivities and the experimentally determined results. Peptides **P1** and **UTS-30** serve as references.

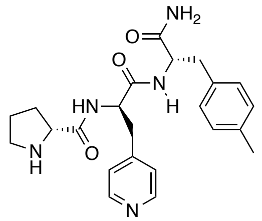

P(1)-1

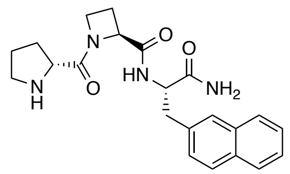

P(1)-2

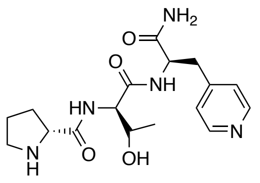

P(1)-3

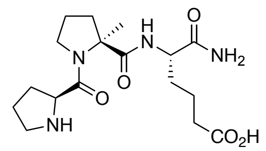

P(1)-4

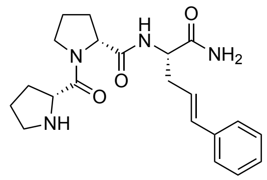

P(1)-5

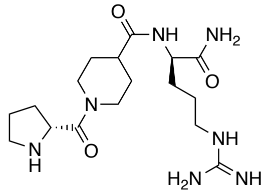

P(1)-6

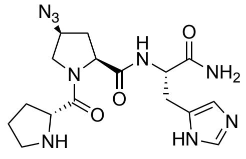

P(1)-7

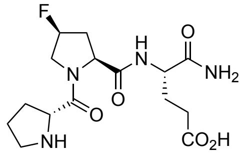

UTS-30

			Initial Experiments		Validation Experiments	
Entry	Tripeptide	Pred.[a] *ee* [%]	Conv.[b] [%]	*ee*[c] [%]	Conv.[b] [%]	*ee*[c] [%]
1	**P(1)-1**	86	7	- 69	7	- 69
2	**P(1)-2**	83	22	56	18	55
3	**P(1)-3**	81	6	- 59	6	- 59
4	**P(1)-4**[d]	80	25	- 29	19	- 29
5	**P(1)-5**	80	27	56	24	57
6	**P(1)-6**	80	4	49	5	49
7	**P(1)-7**	78	60	60	48	62
8	**UTS-30**	77	-	-	quant.	91
9	**P1**	-	-	-	92	86

[a] Predicted *ee* by machine learning [b] Conversions determined by ^{1}H-NMR spectroscopy from the crude reaction mixture; [c] Enantiomeric excess determined by SFC on a chiral stationary phase. [d] The opposite enantiomer was synthesized due to a better availability of amino acid building blocks.

Screening Data of Second Round Predicted Peptides:

Table 5 Synthesized tripeptides from the second round of machine learning predictions. The table shows the predicted enantioselectivities and the experimentally measured results from catalysis of the model reaction.

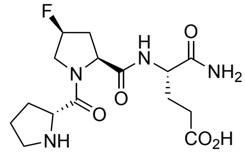

P(2)-1
(UTS-30)

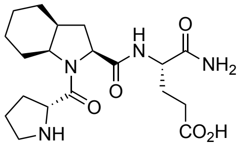

P(2)-2

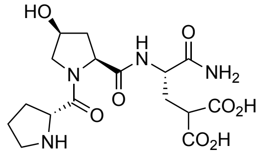

P(2)-3

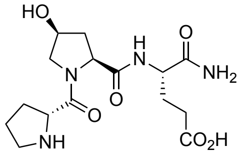

P(2)-4

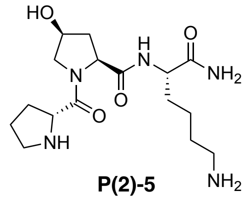

P(2)-5

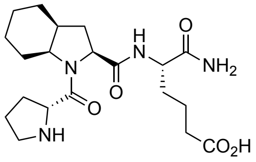

P(2)-6

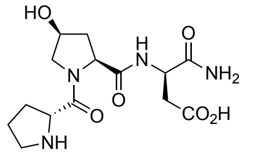

P(2)-7

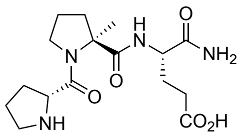

P(2)-8

Entry	Tripeptide	PLS[a] Pred. *ee* [%]	NNet[b] Pred. *ee* [%]	MR[c] Pred. *ee* [%]	Conv.[d] [%]	*ee*[e] [%]
1	**P(2)-1**	89	85	89	quant.	90
2	**P(2)-2**	89	83	85	99	71
3	**P(2)-3**	87	82	80	4[f]	63
4	**P(2)-4**	89	75	90	98	88
5	**P(2)-5**	75	77	89	33	62
6	**P(2)-6**	69	74	88	91	31
7	**P(2)-7**	46	74	81	44	52
8	**P(2)-8**	87	69	82	71	90

Predicted *ee* by machine learning model: [a] Partial Least Square Regression [b] Neuronal Network [c] Mixed Regressors; [d] Conversions determined by ^{1}H-NMR spectroscopy from the crude reaction mixture; [e] Enantiomeric excess determined by SFC on a chiral stationary phase. [f] Low conversion due to low solubility of tripeptide catalyst **P(2)-3**.

Screening Data of Third Round Predicted Peptides:

Table 6 Synthesized tripeptides from the third round of machine learning predictions. The table shows the predicted enantioselectivities and the experimentally measured results from application on the model reaction.

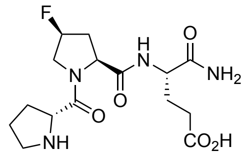
P(3)-1
(UTS-30)

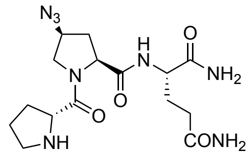
P(3)-2

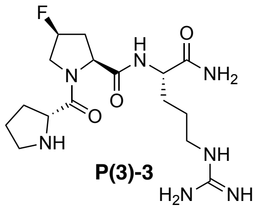
P(3)-3

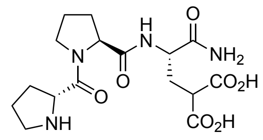
P(3)-4

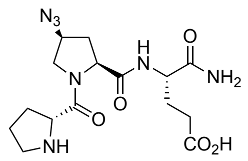
P(3)-5

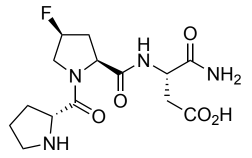
P(3)-6

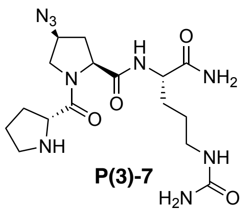
P(3)-7

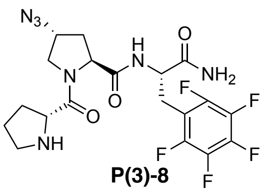
P(3)-8

Entry	Tripeptide	Pred.[a] *ee* [%]	Conv.[b] [%]	*ee*[c] [%]
1	**P(3)-1**	86	quant.	91
2	**P(3)-2**	85	67	91
3	**P(3)-3**	82	69	76
4	**P(3)-4**	81	14[d]	74
5	**P(3)-5**	78	99	92
6	**P(3)-6**	77	98	87
7	**P(3)-7**	75	63	77
9	**P(3)-8**	75	23	42

[a] Predicted *ee* by machine learning [b] Conversions determined by ^{1}H-NMR spectroscopy from the crude reaction mixture; [c] Enantiomeric excess determined by SFC on a chiral stationary phase. [d] Low conversion due to low solubility of tripeptide catalyst **P(3)-4**.

ScreeningData of Additional Training Set Peptides:

Table 7 Best performing UTS-peptides with opposite enantioselectivity and variations with alternative amino acids in the Xaa and Yaa position, respectively.

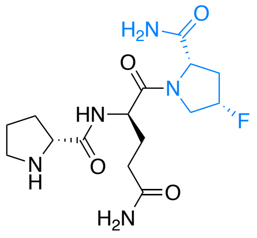

UTS-39

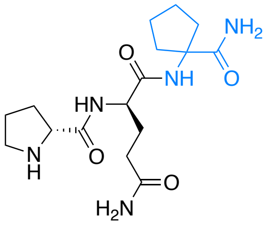

UTS-108

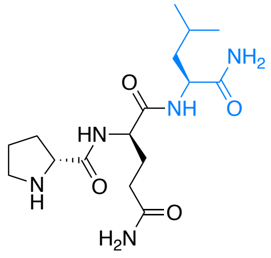

UTS-59

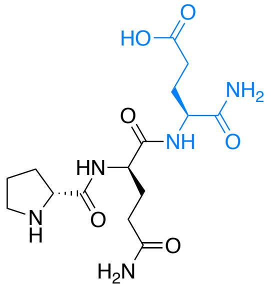

UTS-104

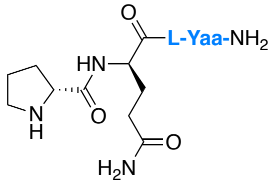

L-Ile
L-Val
L-Pro
L-Phe
L-Asn

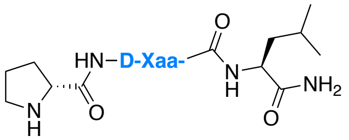

D-Asn
D-His

Entry	Tripeptide	Conv.[a] [%]	*ee*[b] [%]
1	**UTS-39**	5	-78
2	**UTS-108**	8	-78
3	**UTS-59**	8	-77
4	**UTS-104**	56	-72
5	H-DPro-DGln-Ile-NH_2	12	-76
6	H-DPro-DGln-Val-NH_2	12	-76
7	H-DPro-DGln-Pro-NH_2	12	-81
8	H-DPro-DGln-Phe-NH_2	13	-72
9	H-DPro-DGln-Asn-NH_2	12	-61
10	H-DPro-DAsn-Leu-NH_2	6	-71
11	H-DPro-DHis-Leu-NH_2	13	-80

[a] Conversions determined by ^{1}H-NMR spectroscopy from the crude reaction mixture; [b] Enantiomeric excess determined by SFC on a chiral stationary phase.

4. NMR-Spectra of Peptides

^{1}H and ^{13}C-NMR of *P6:*

^{1}H NMR (600 MHz, D_2O + H_2O), H_2O signal supressed

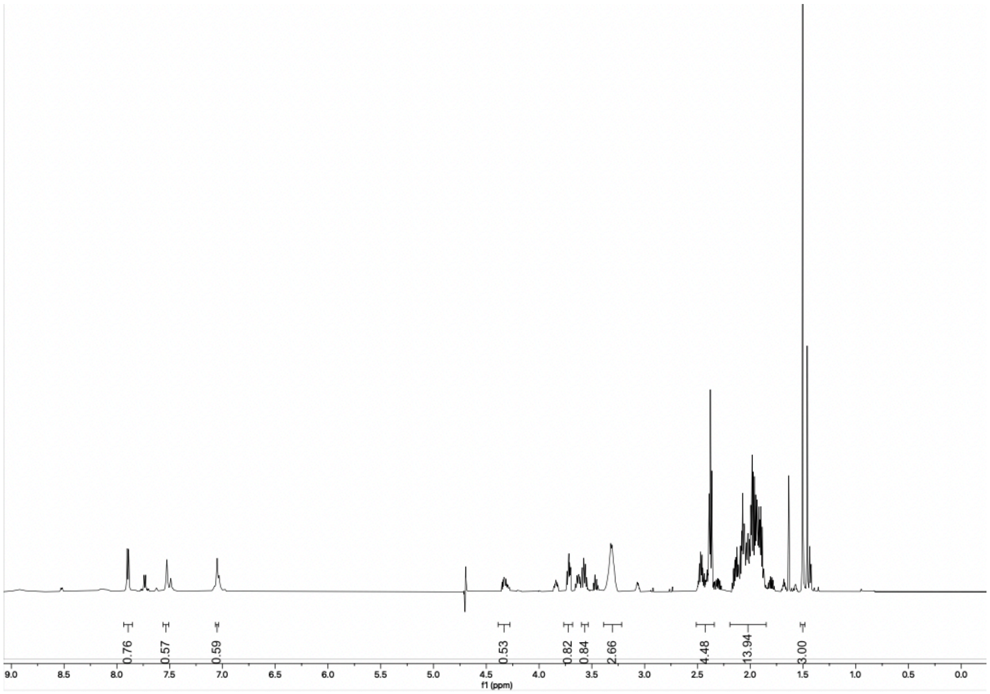

^{13}C NMR (151 MHz, D_2O + H_2O) H_2O signal supressed

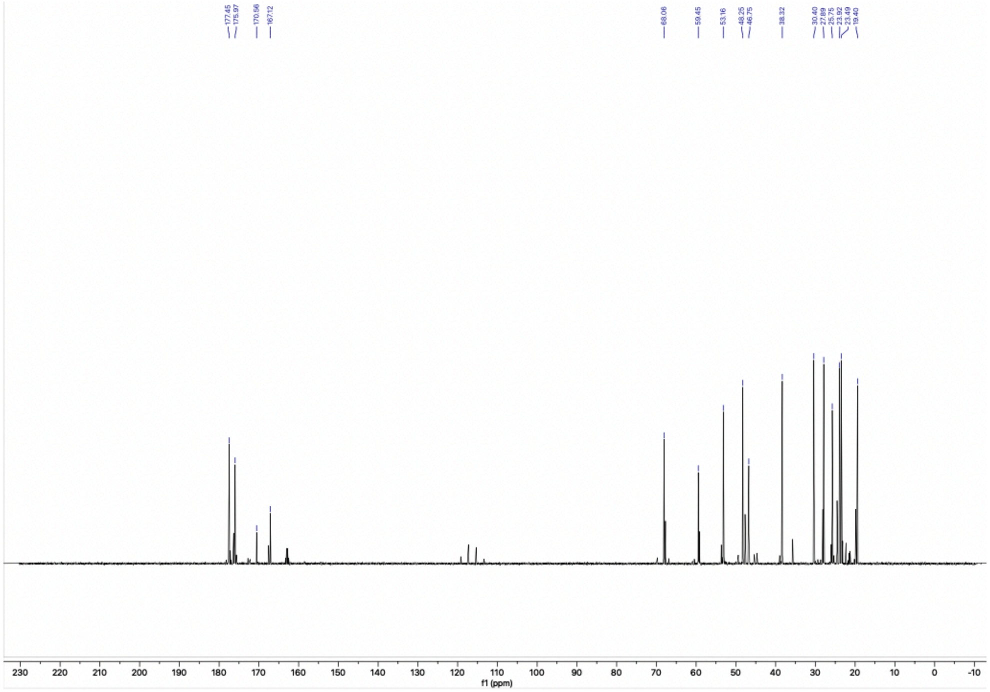

^{1}H and ^{13}C-NMR of *P7:*

^{1}H NMR (600 MHz, D_2O + H_2O), H_2O signal supressed

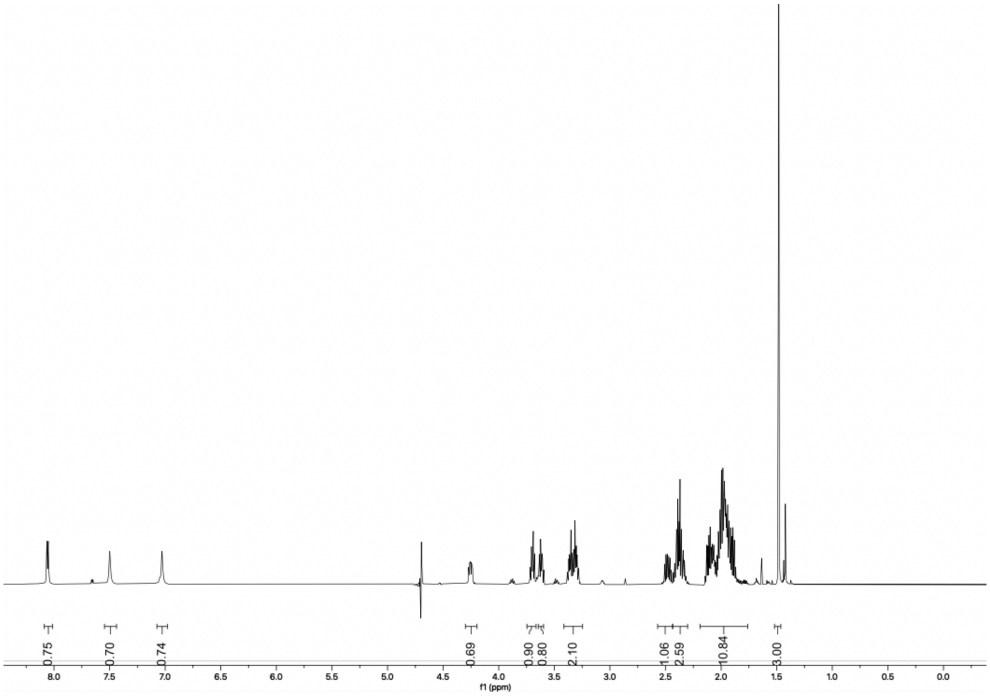

^{13}C NMR (151 MHz, D_2O + H_2O) H_2O signal supressed

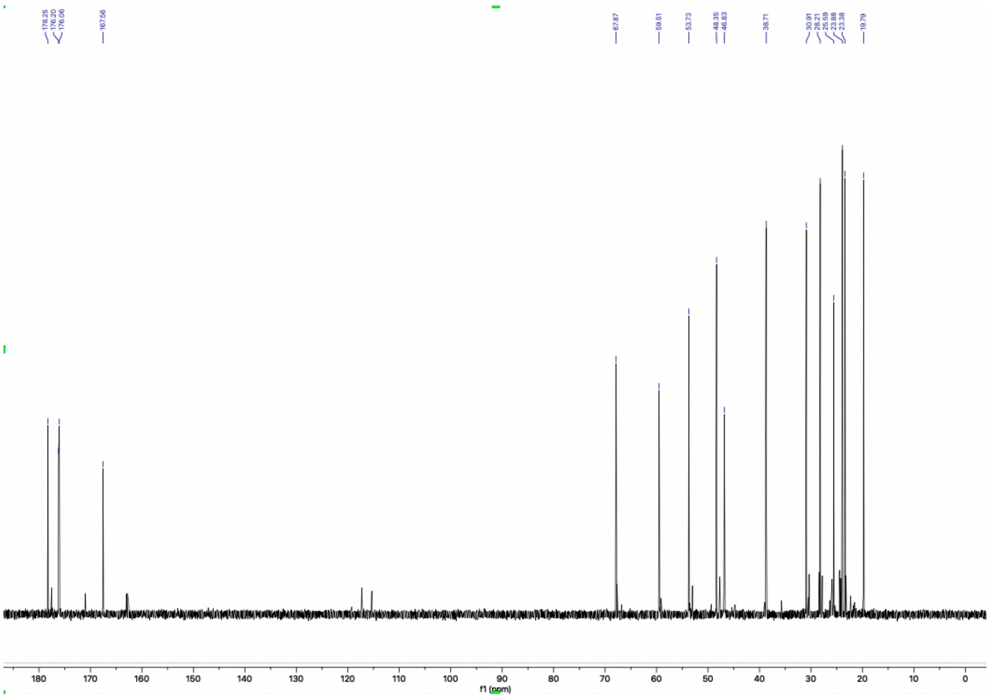

^{1}H and ^{13}C-NMR of *P8:*

^{1}H NMR (600 MHz, D_2O + H_2O), H_2O signal supressed

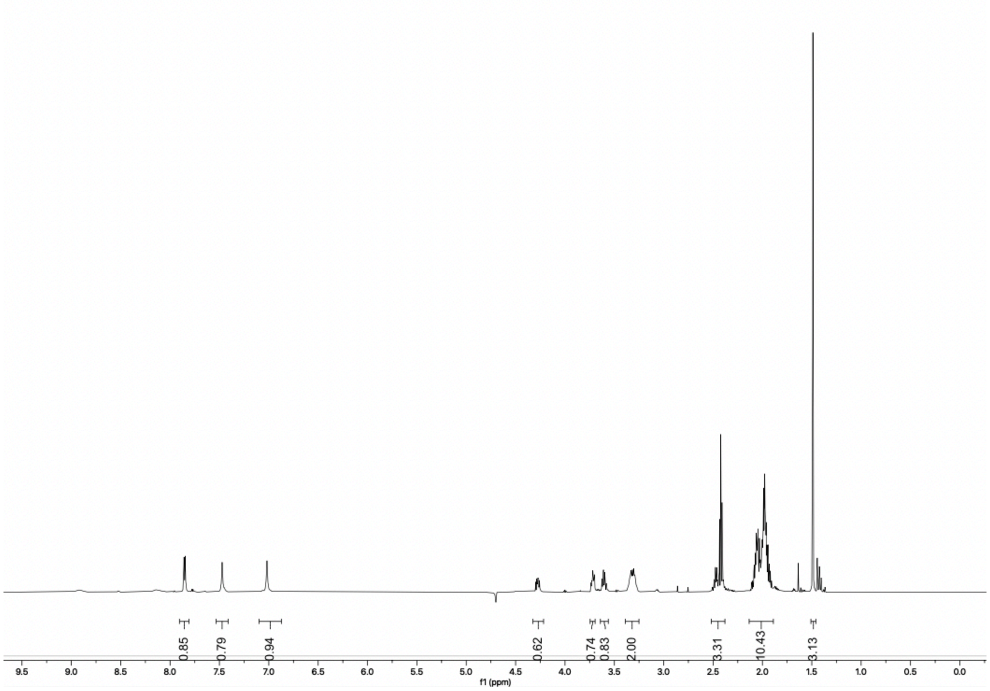

^{13}C NMR (151 MHz, D_2O + H_2O) H_2O signal supressed

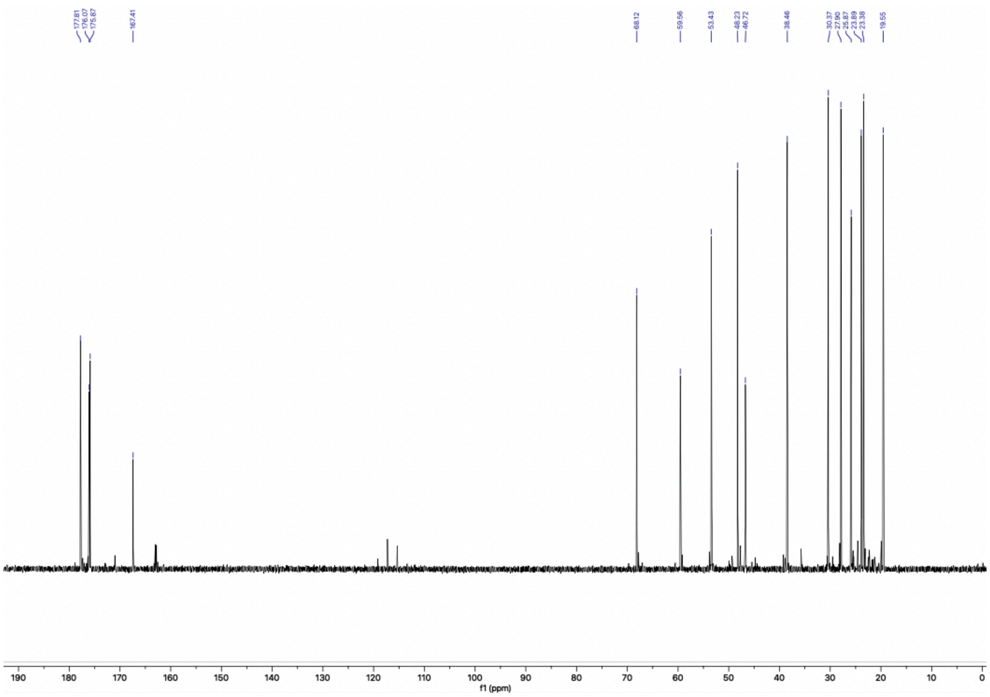

^{1}H and ^{13}C-NMR of *P11:*

^{1}H NMR (400 MHz, D_2O)

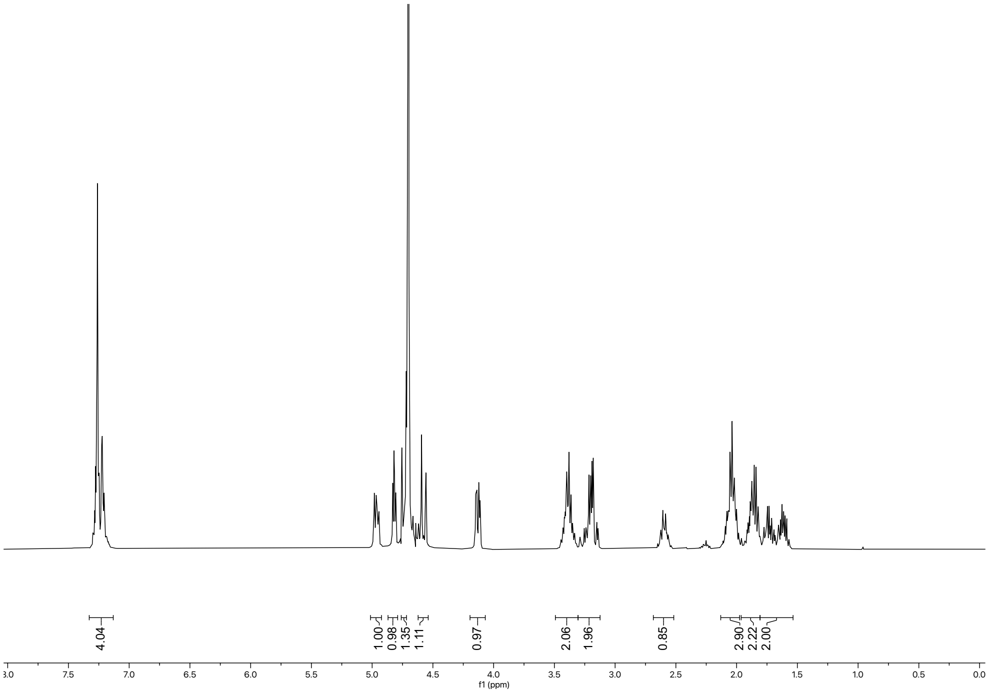

^{13}C NMR (101 MHz, D_2O)

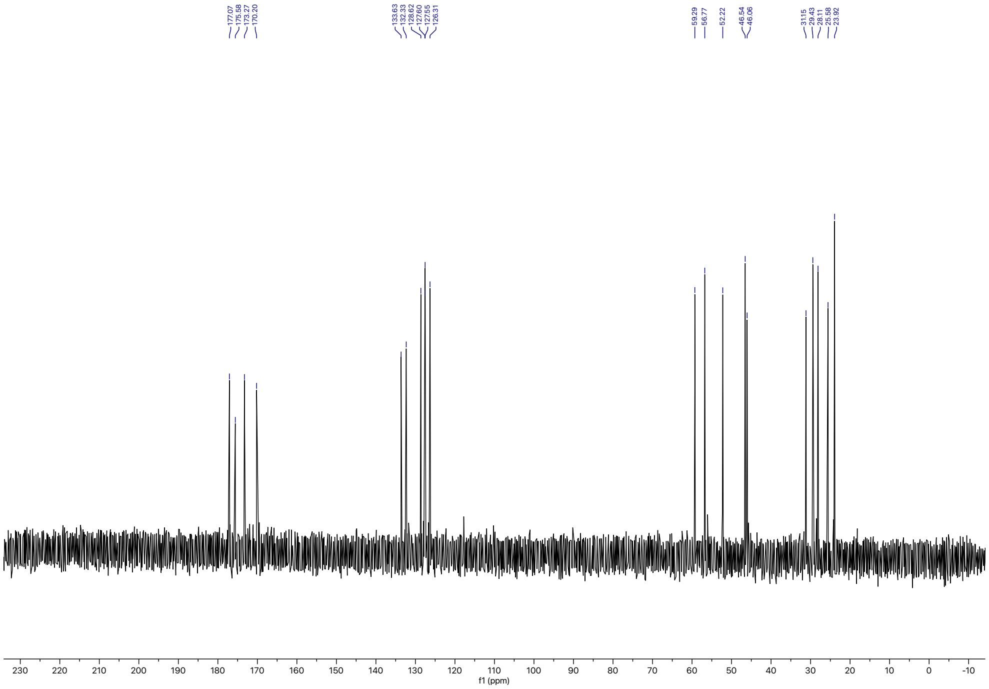

^{1}H and ^{13}C-NMR of *P17:*

^{1}H NMR (400 MHz, D_2O)

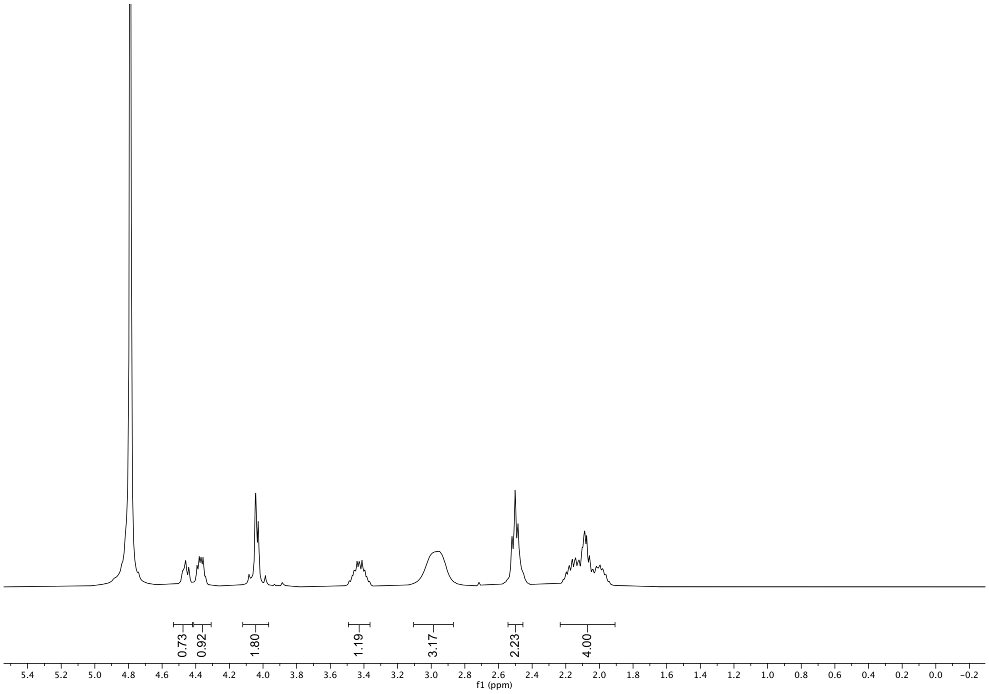

^{13}C NMR (101 MHz, D_2O)

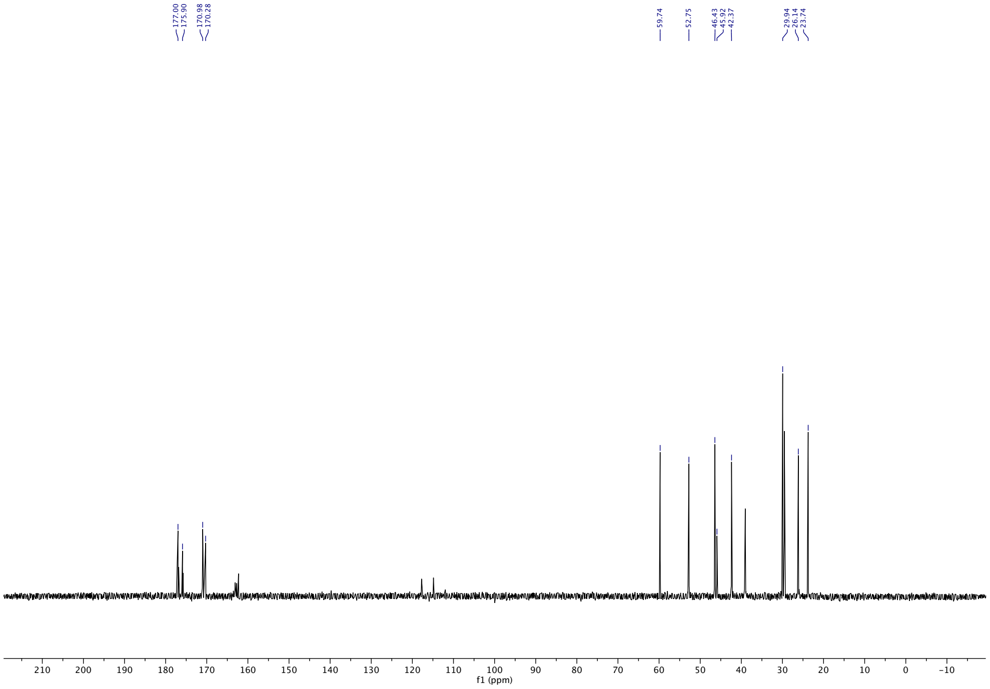

^{1}H and ^{13}C-NMR of *P21:*

^{1}H NMR (400 MHz, D_2O)

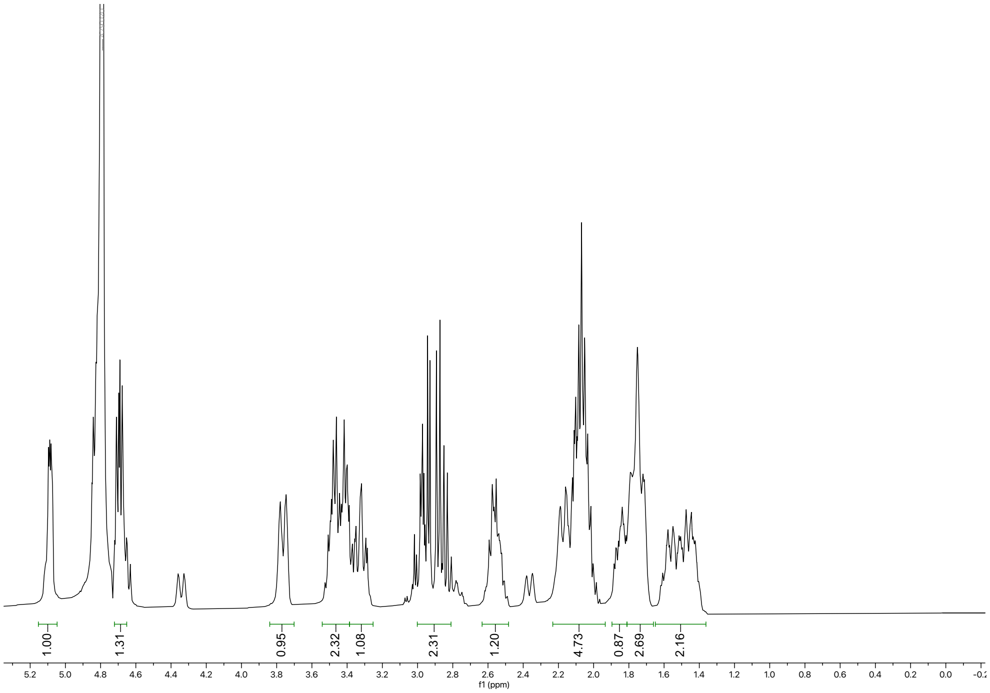

^{13}C NMR (101 MHz, D_2O)

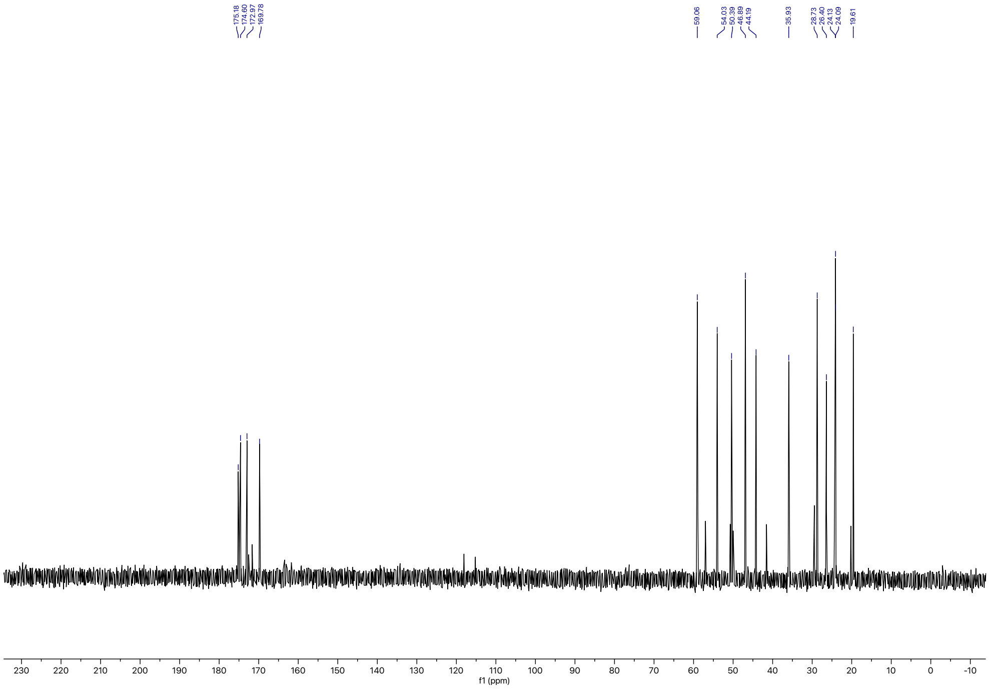

^{1}H and ^{13}C-NMR of *P22:*

^{1}H NMR (400 MHz, D_2O)

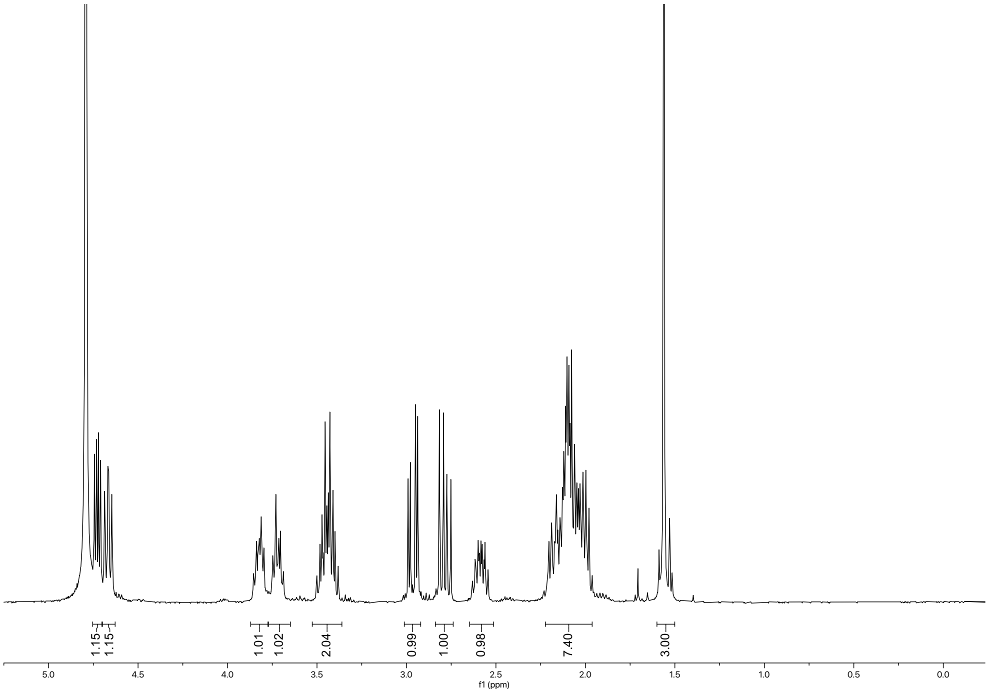

^{13}C NMR (101 MHz, D_2O)

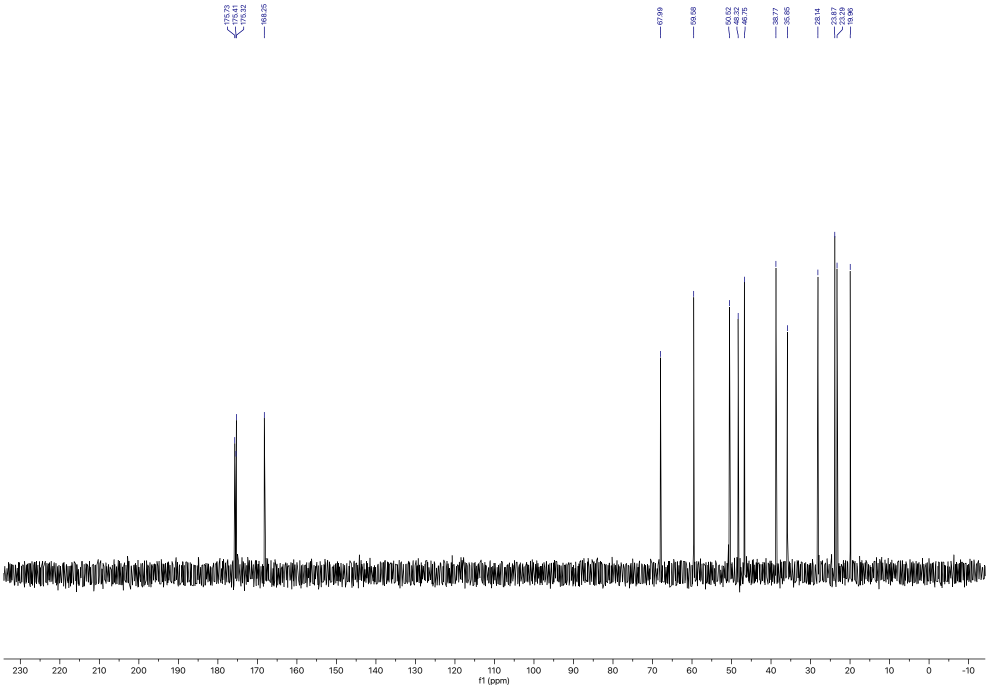

^{1}H and ^{13}C-NMR of *P24:*

^{1}H NMR (400 MHz, D_2O)

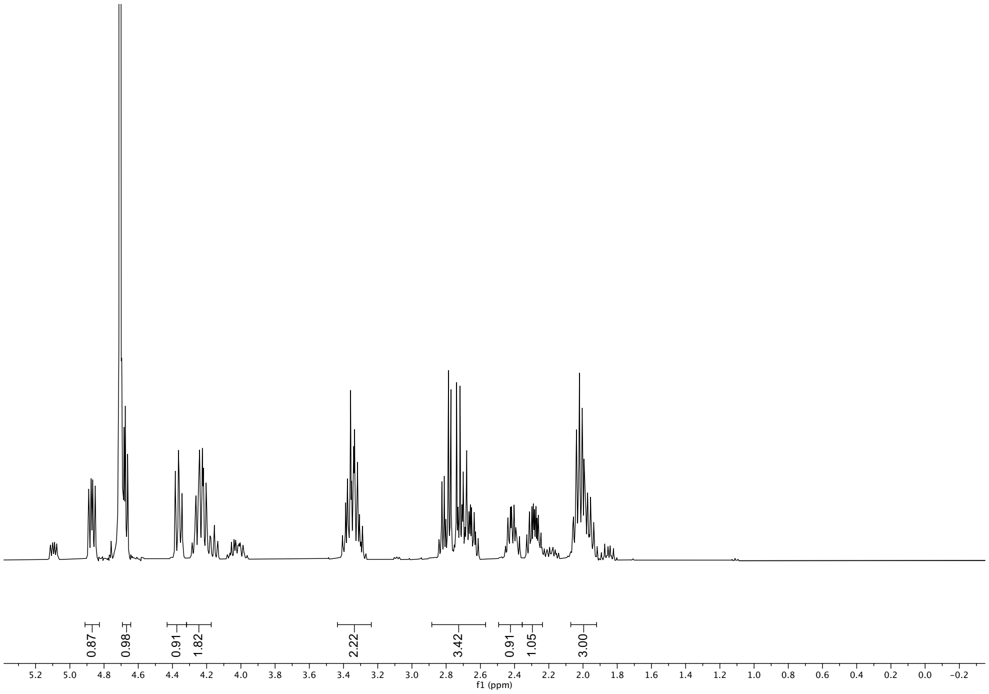

^{13}C NMR (101 MHz, D_2O)

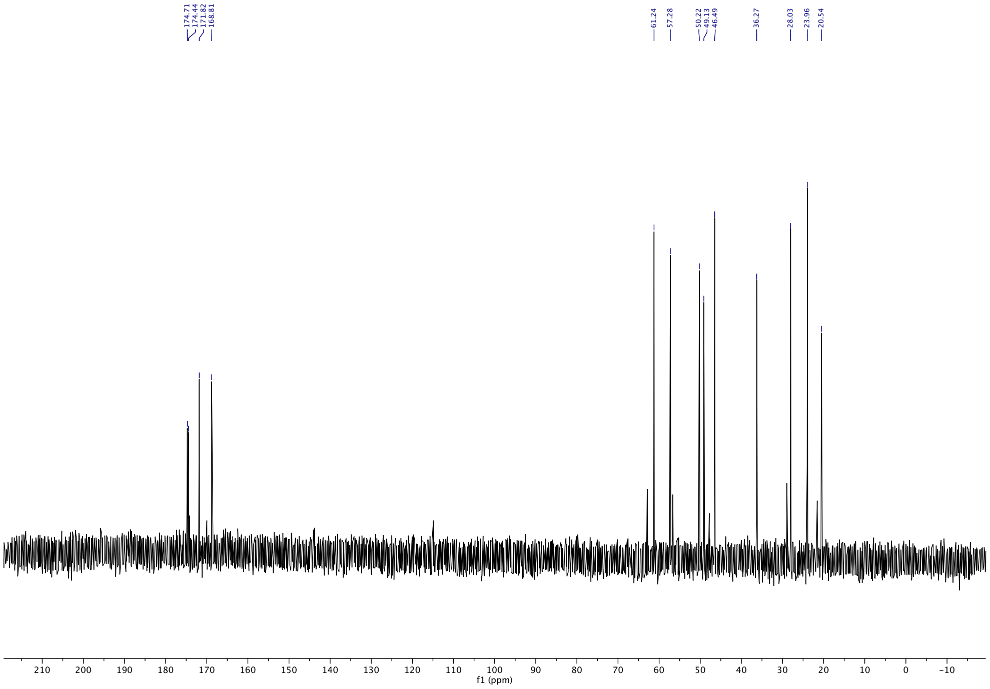

^{1}H and ^{13}C-NMR of *P26:*

^{1}H NMR (400 MHz, D_2O)

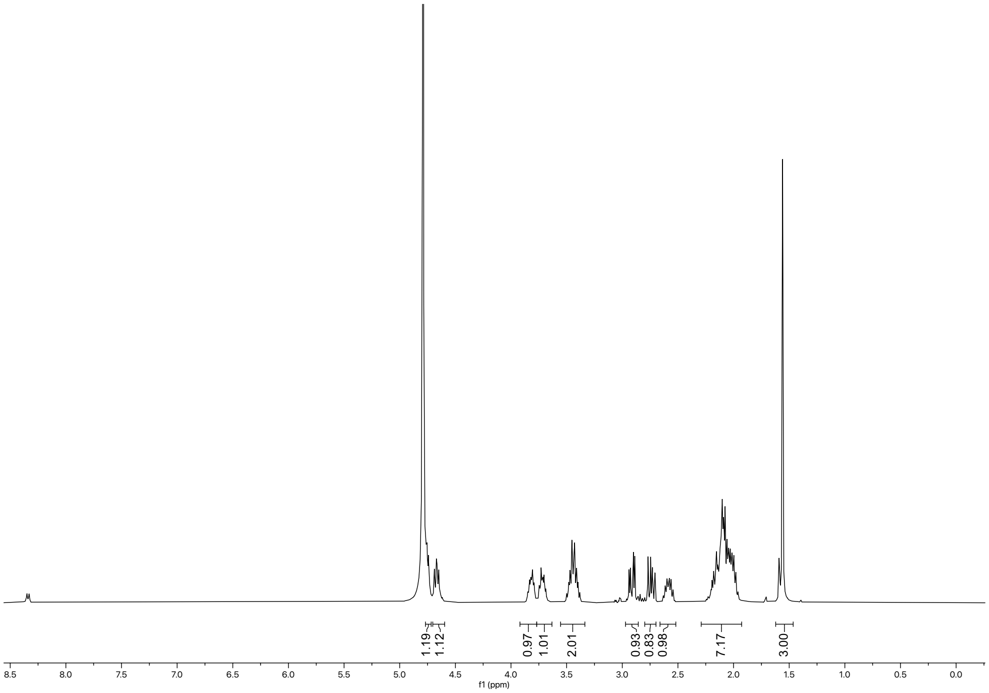

^{13}C NMR (101 MHz, D_2O)

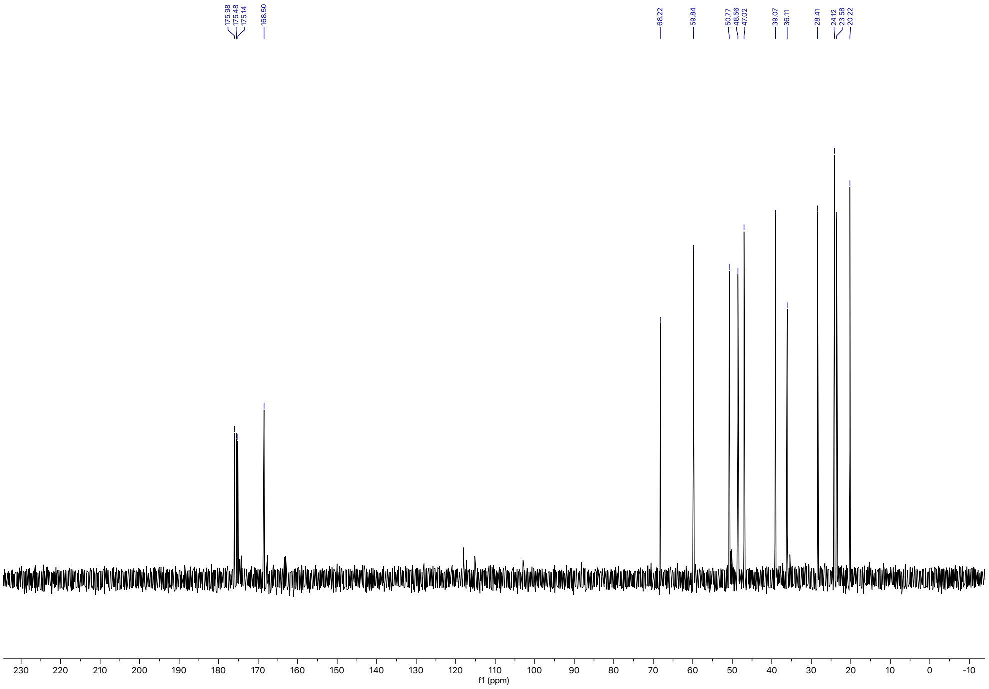

^{1}H and ^{13}C-NMR of *P29:*

^{1}H NMR (400 MHz, D_2O)

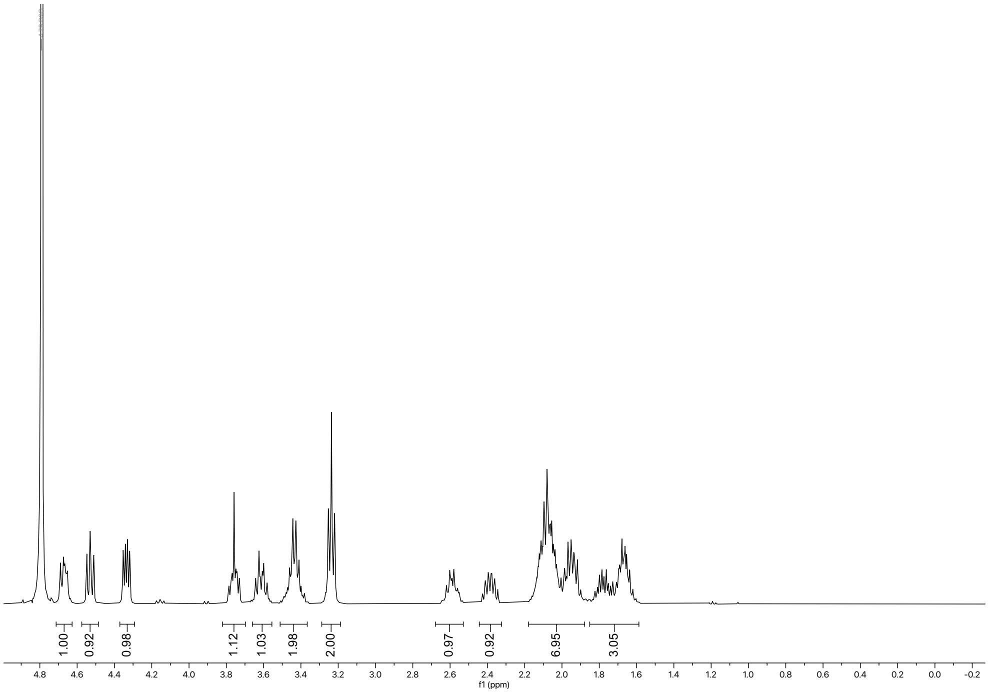

^{13}C NMR (101 MHz, D_2O)

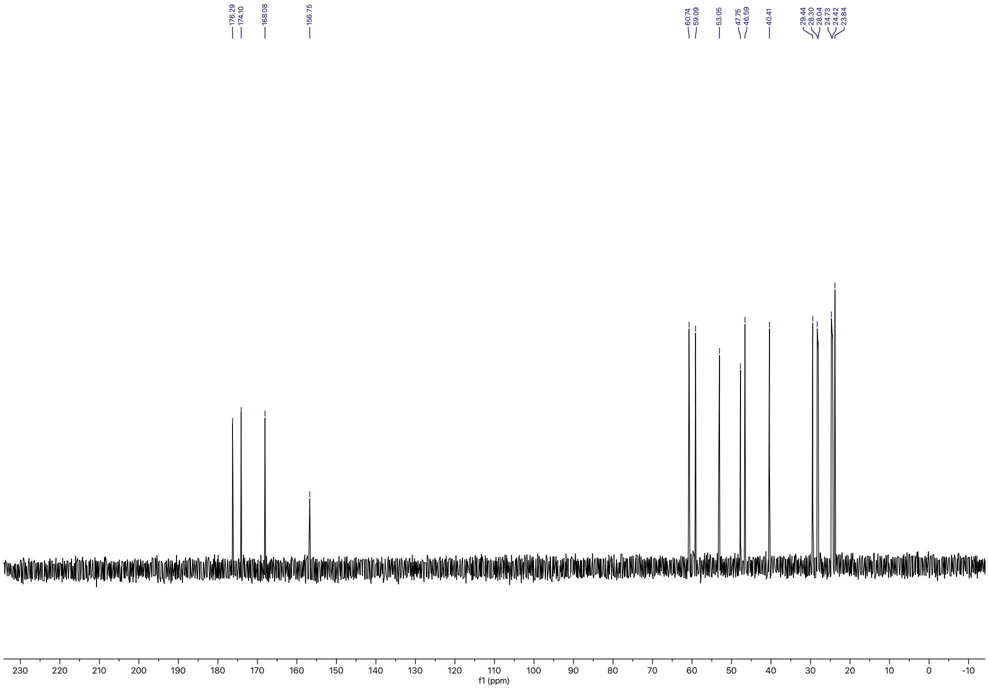

^{1}H and ^{13}C-NMR of *P30:*

^{1}H NMR (400 MHz, D_2O)

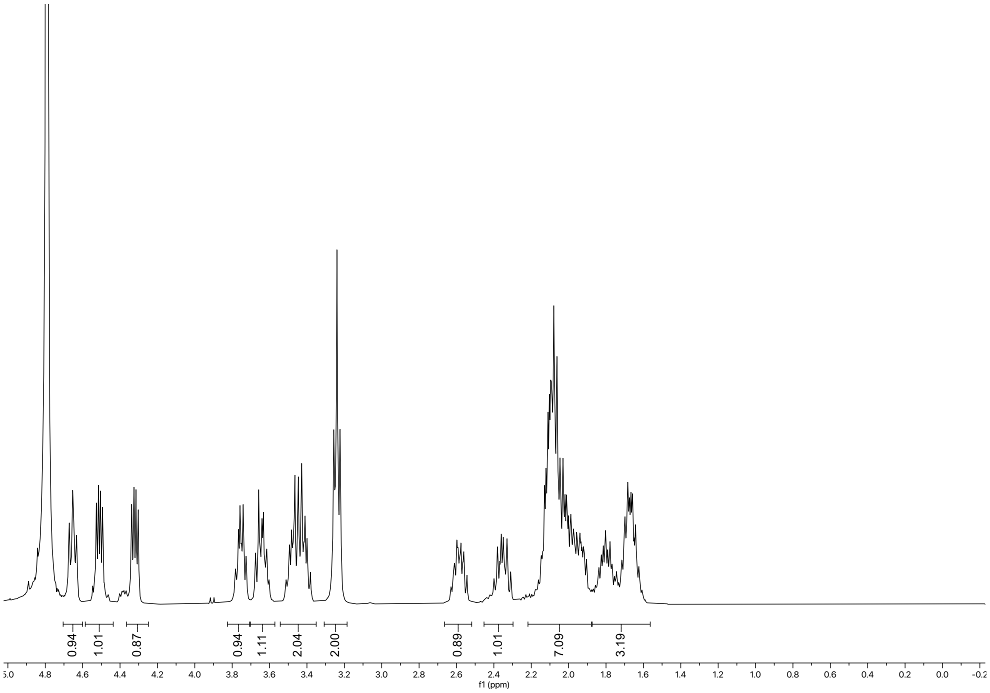

^{13}C NMR (101 MHz, D_2O)

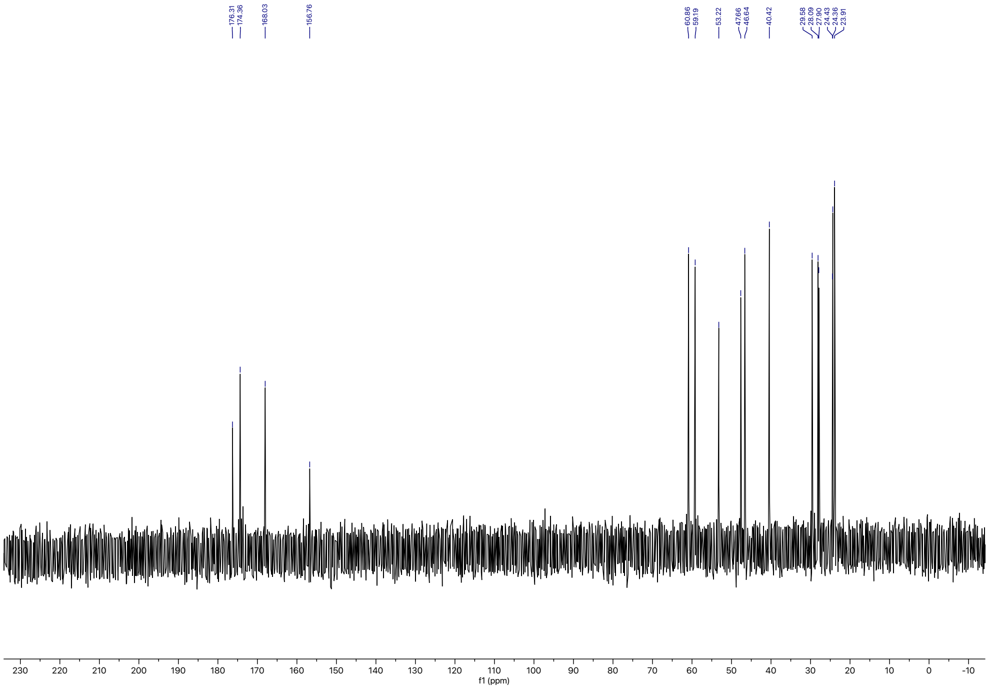

^{1}H and ^{13}C-NMR of *P31:*

^{1}H NMR (400 MHz, D_2O)

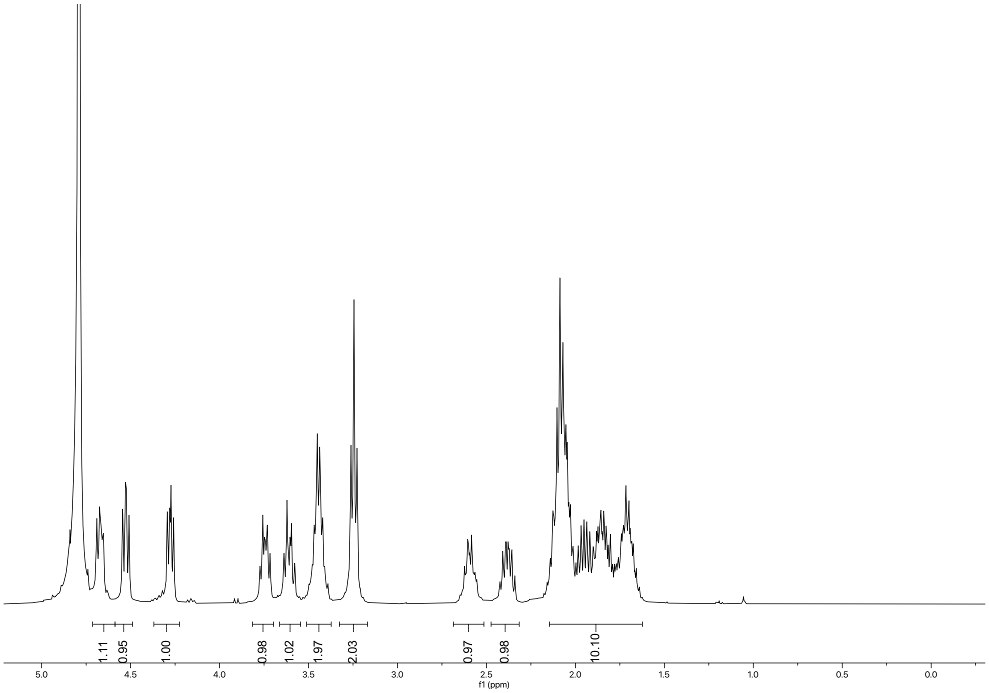

^{13}C NMR (101 MHz, D_2O)

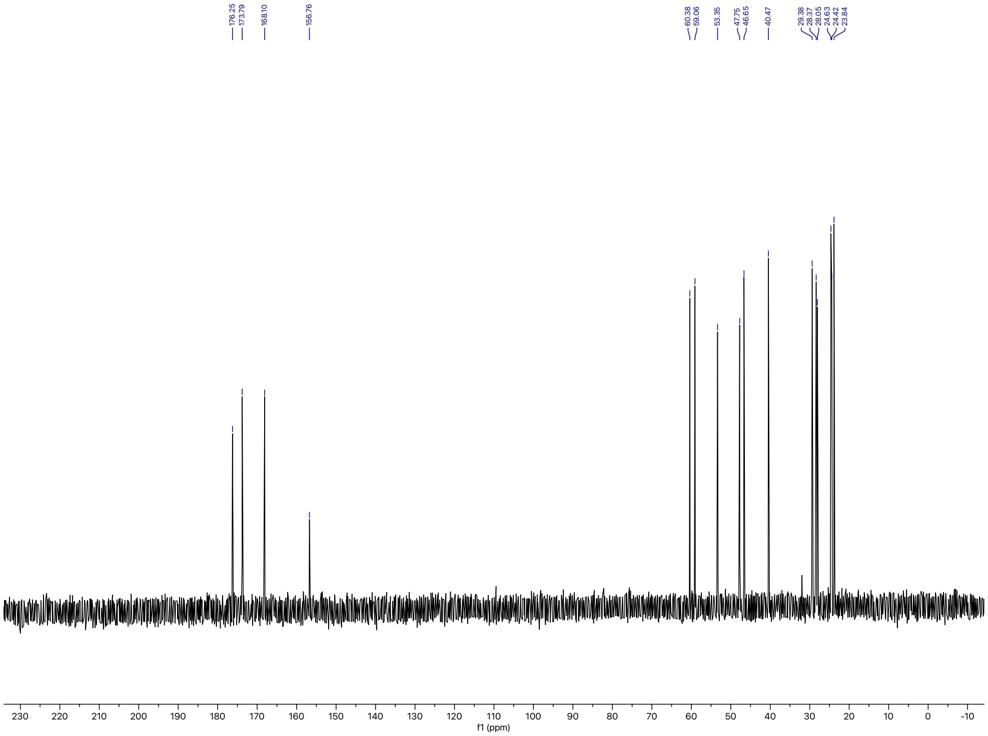

^{1}H and ^{13}C-NMR of *P33*:

^{1}H NMR (400 MHz, D_2O)

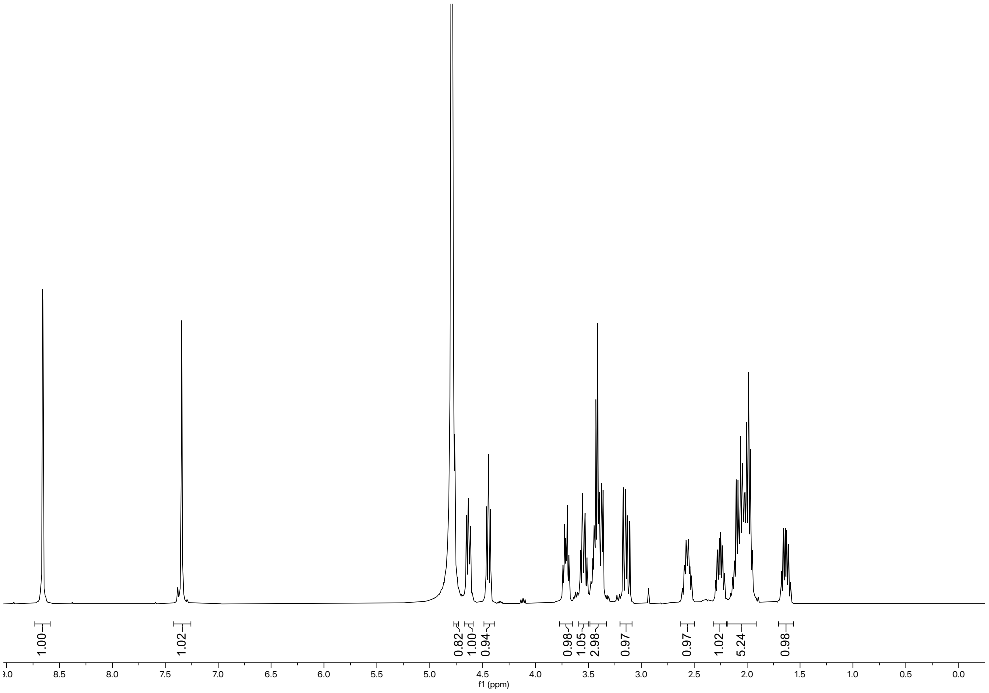

^{13}C NMR (101 MHz, D_2O)

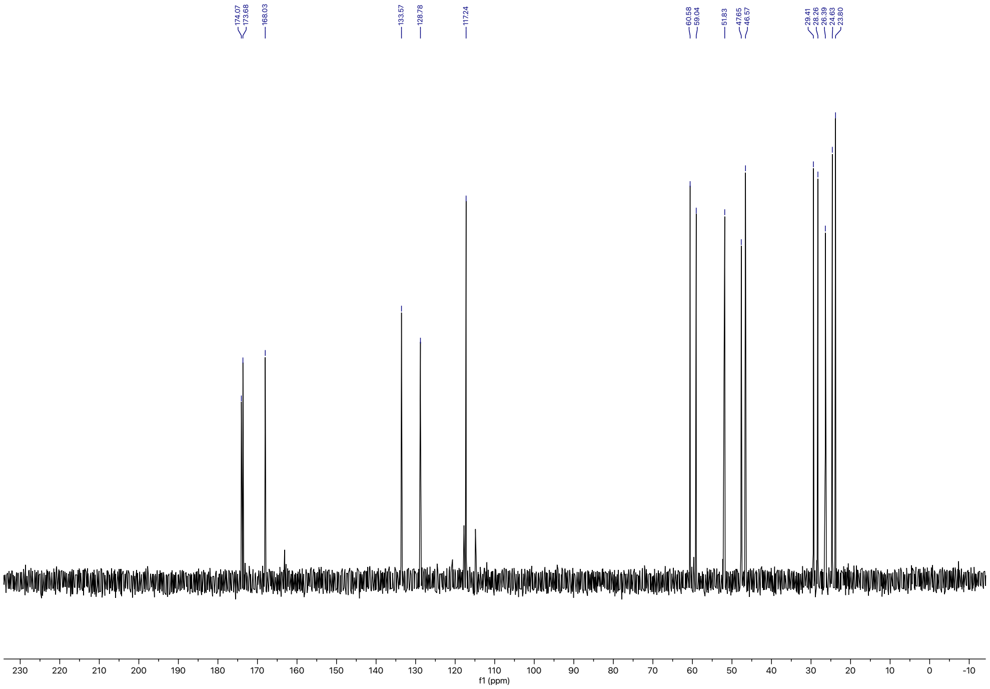

^{1}H and ^{13}C-NMR of *P34:*

^{1}H NMR (400 MHz, D_2O)

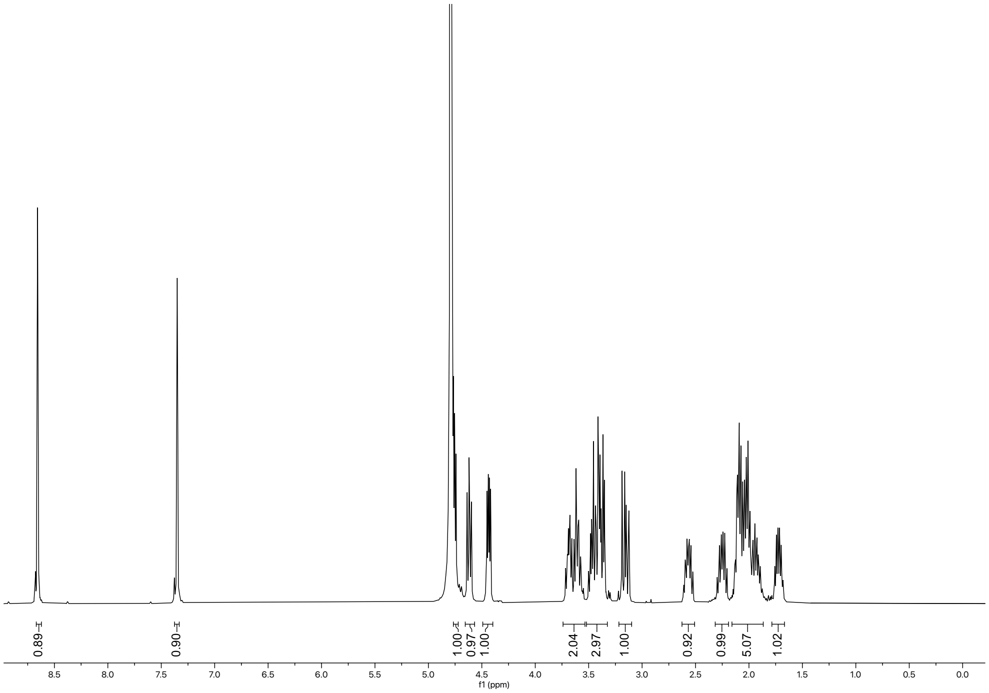

^{13}C NMR (101 MHz, D_2O)

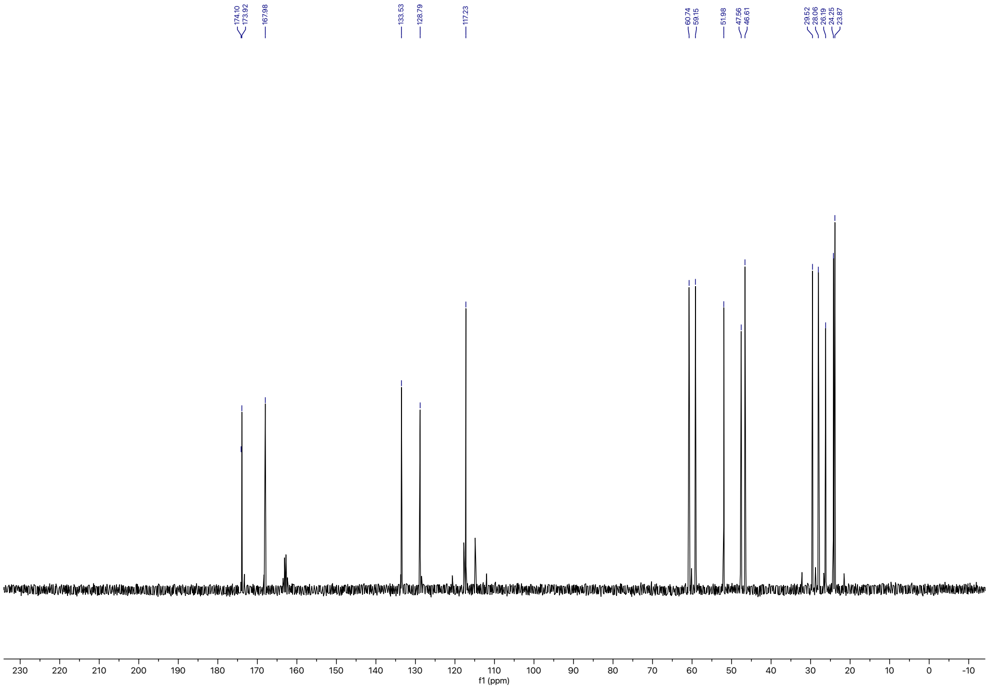

^{1}H and ^{13}C-NMR of *P35:*

^{1}H NMR (400 MHz, D_2O)

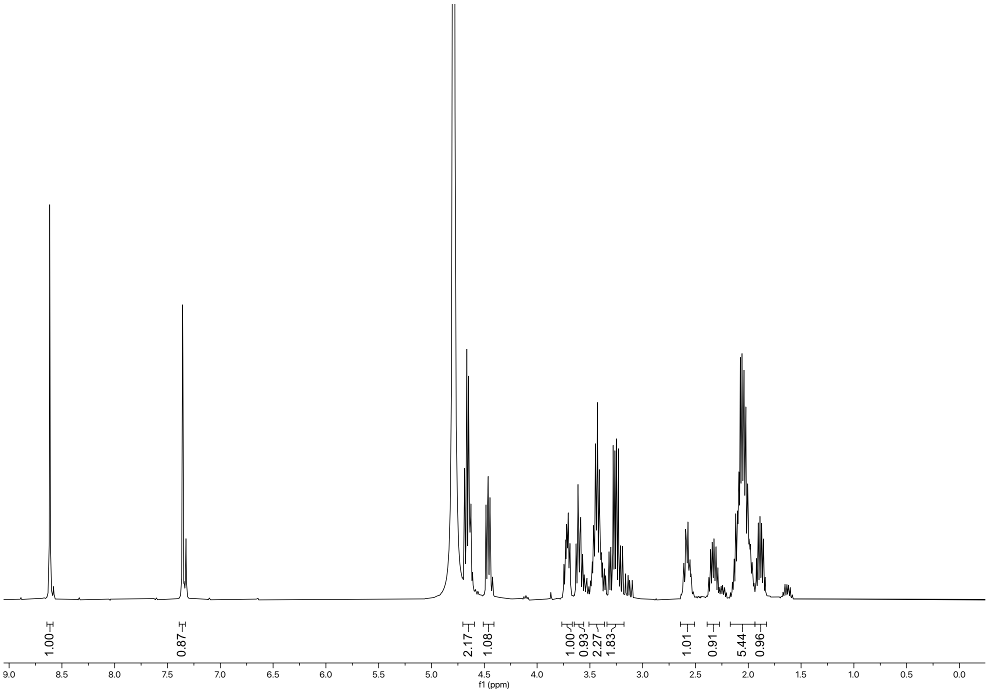

^{13}C NMR (101 MHz, D_2O)

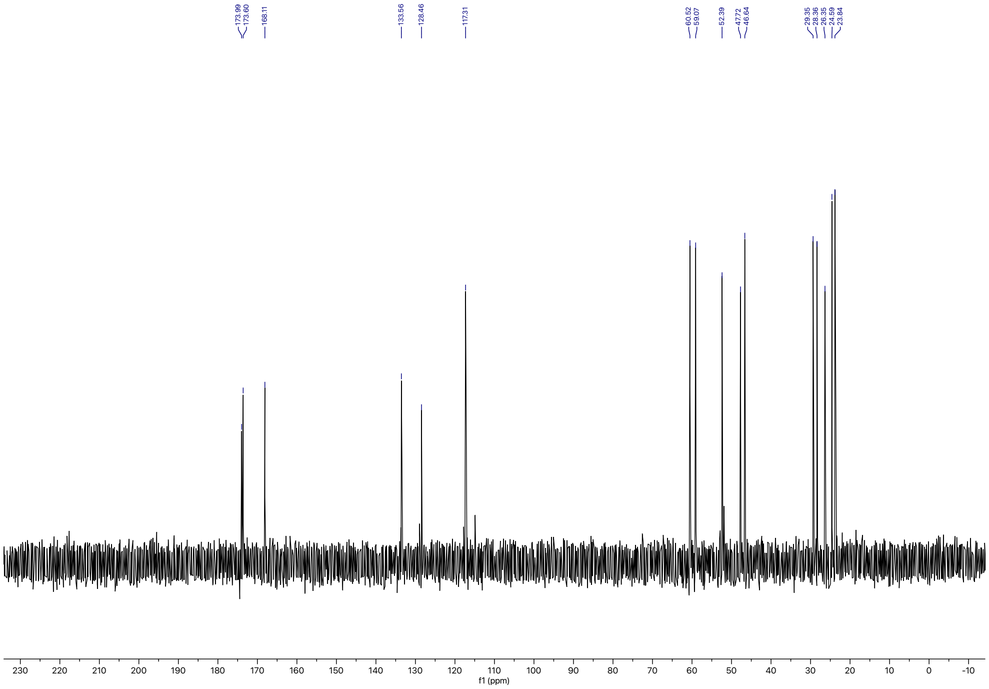

^{1}H and ^{13}C-NMR of *P37:*

^{1}H NMR (600 MHz, $D_2O + H_2O$) , signal of H_2O supressed

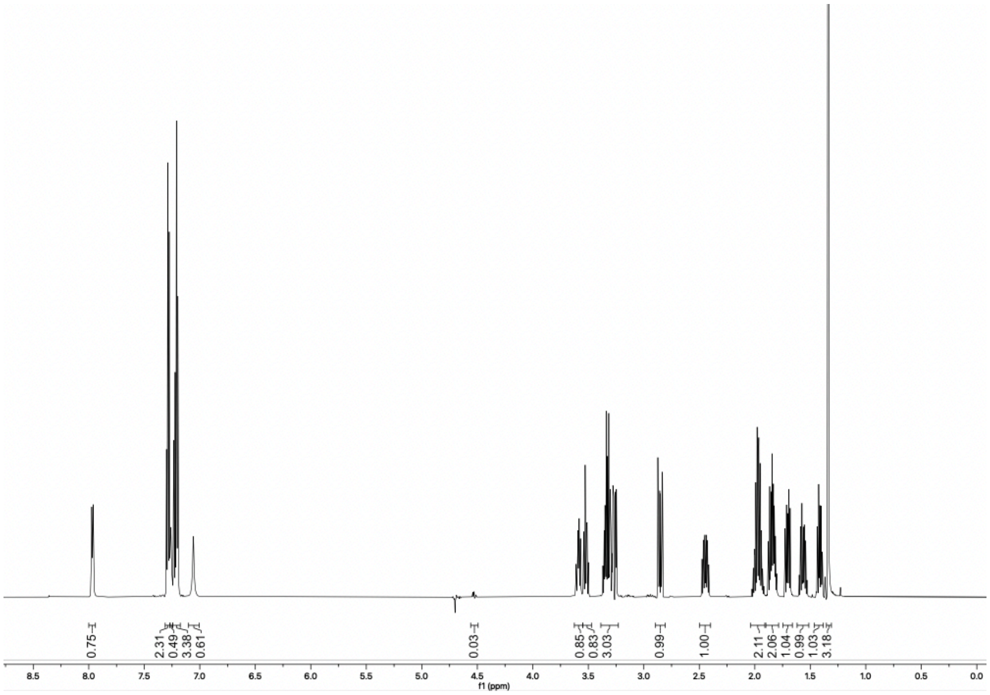

^{13}C NMR (150 MHz, $D_2O + H_2O$)

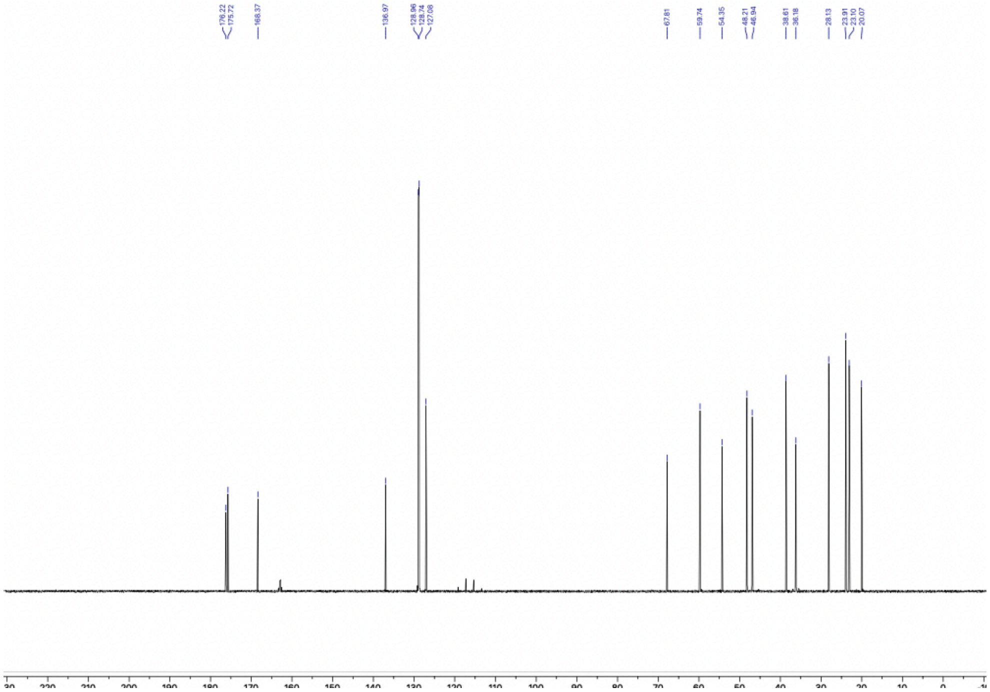

^{1}H and ^{13}C-NMR of *P38:*

^{1}H NMR (600 MHz, D_2O + H_2O), signal of H_2O supressed

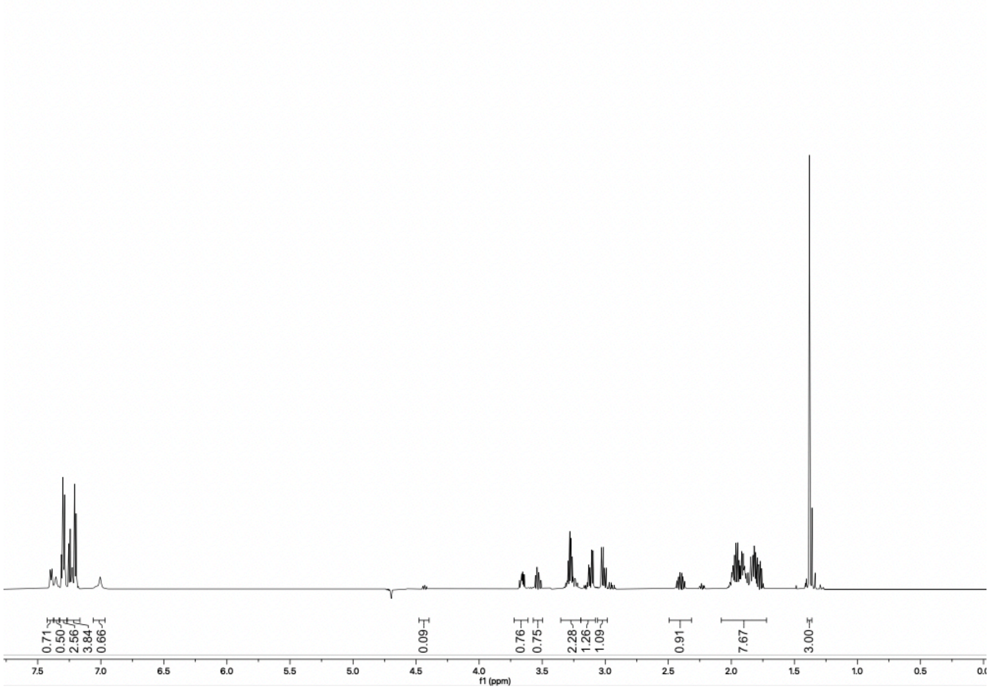

^{13}C NMR (150 MHz, D_2O + H_2O)

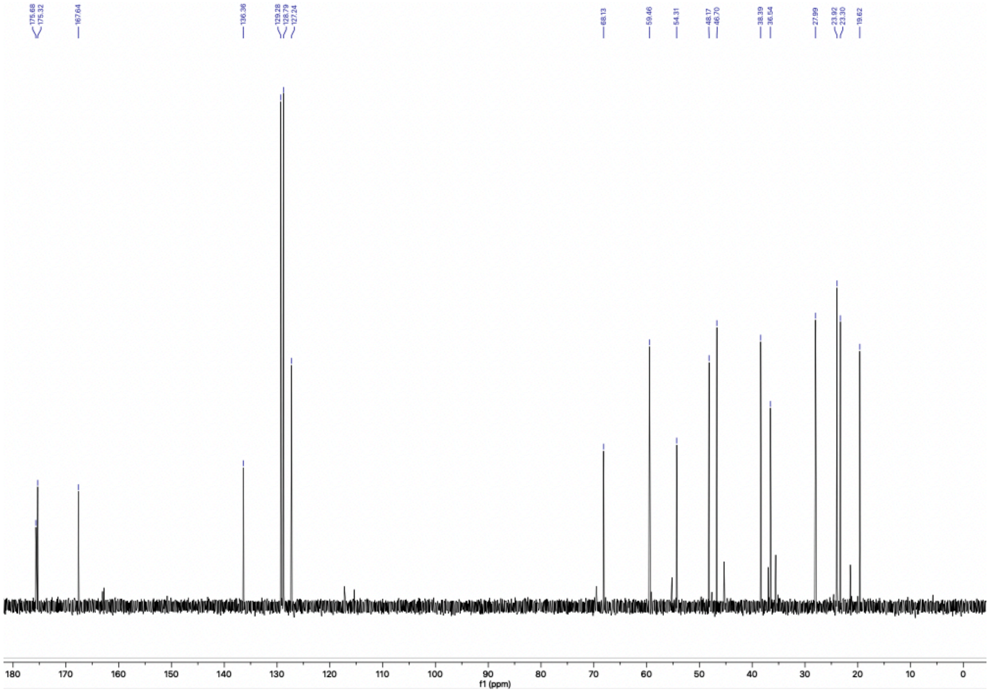

^{1}H and ^{13}C-NMR of *P44:*

^{1}H NMR (400 MHz, D_2O)

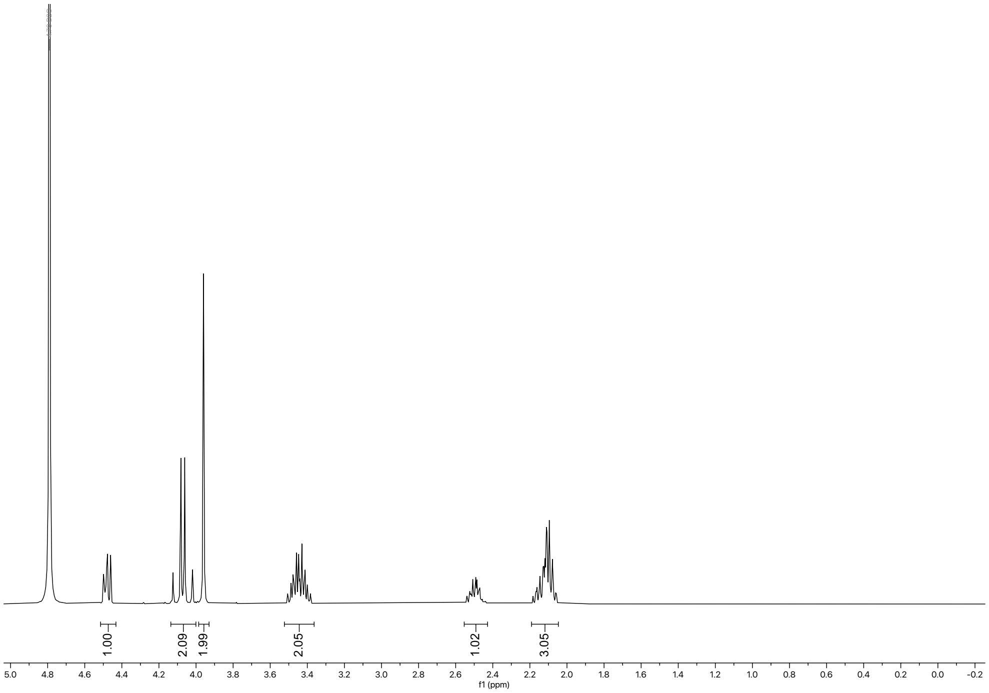

^{13}C NMR (101 MHz, D_2O)

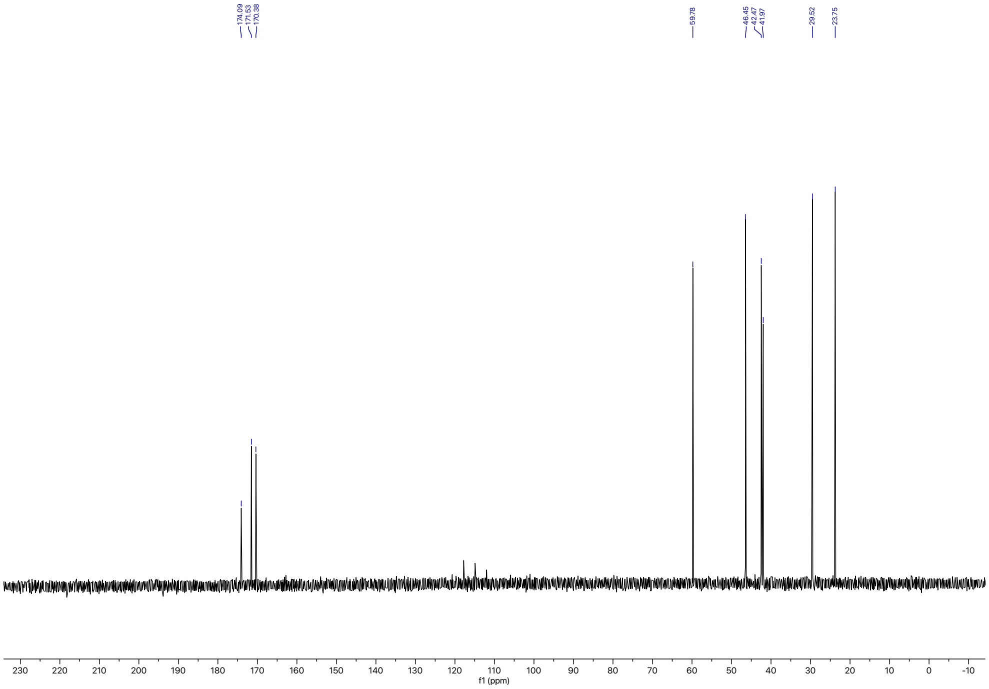

^{1}H and ^{13}C-NMR of *P45:*

^{1}H NMR (400 MHz, D_2O)

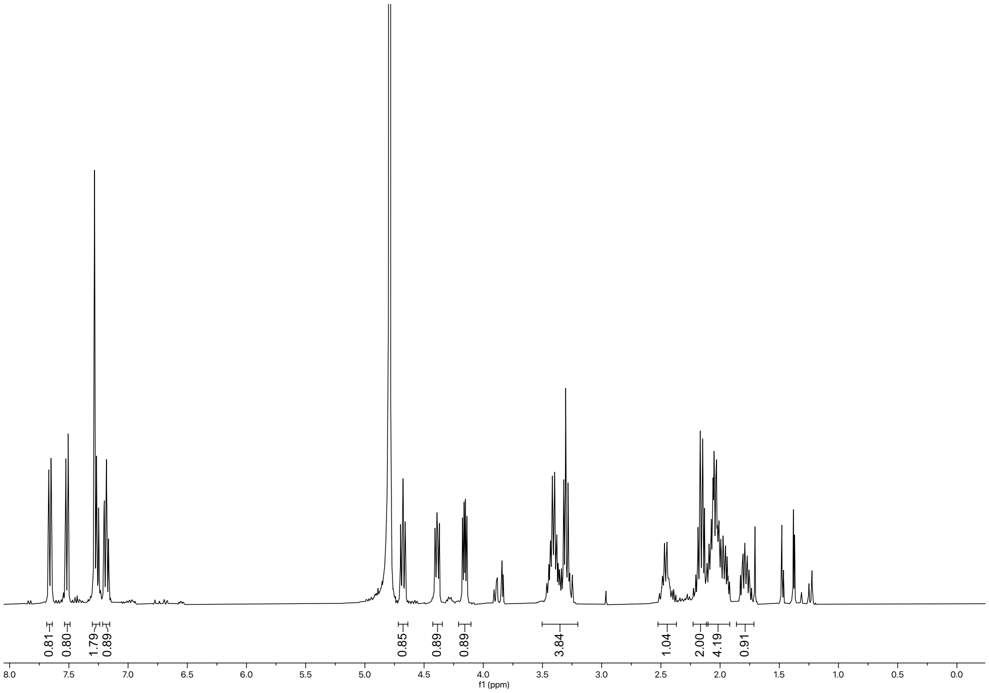

^{13}C NMR (101 MHz, D_2O)

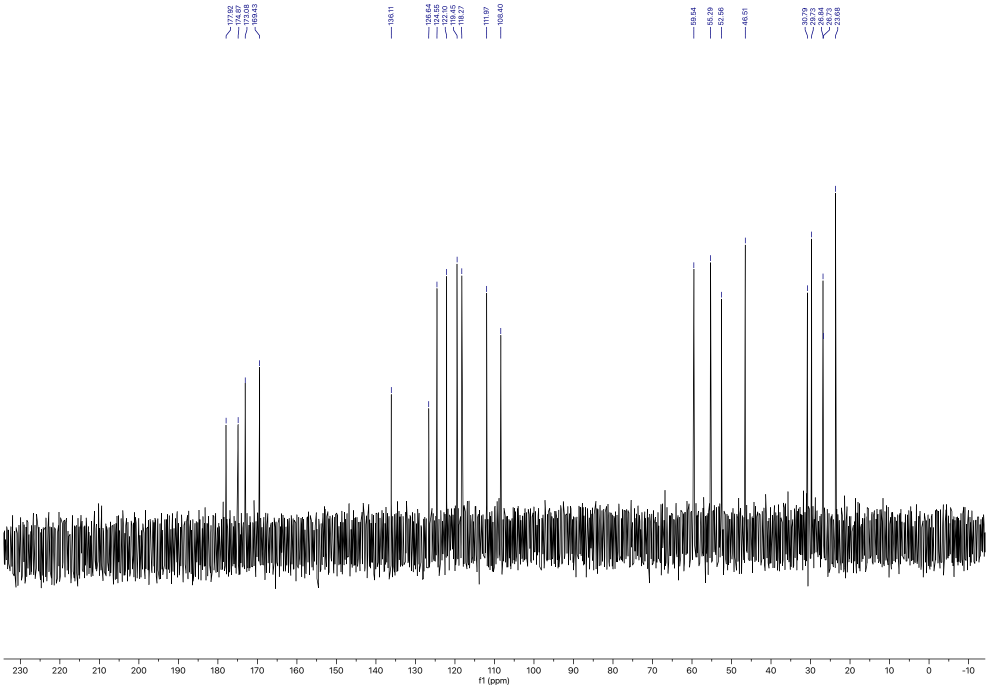

^{1}H and ^{13}C-NMR of *P46:*

^{1}H NMR (400 MHz, D_2O)

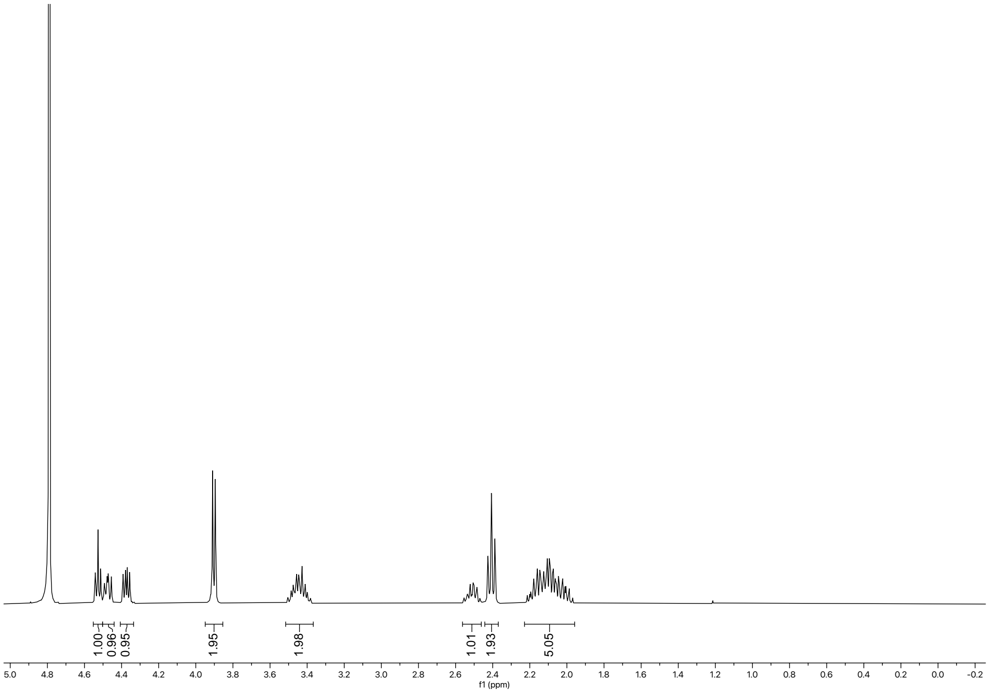

^{13}C NMR (101 MHz, D_2O)

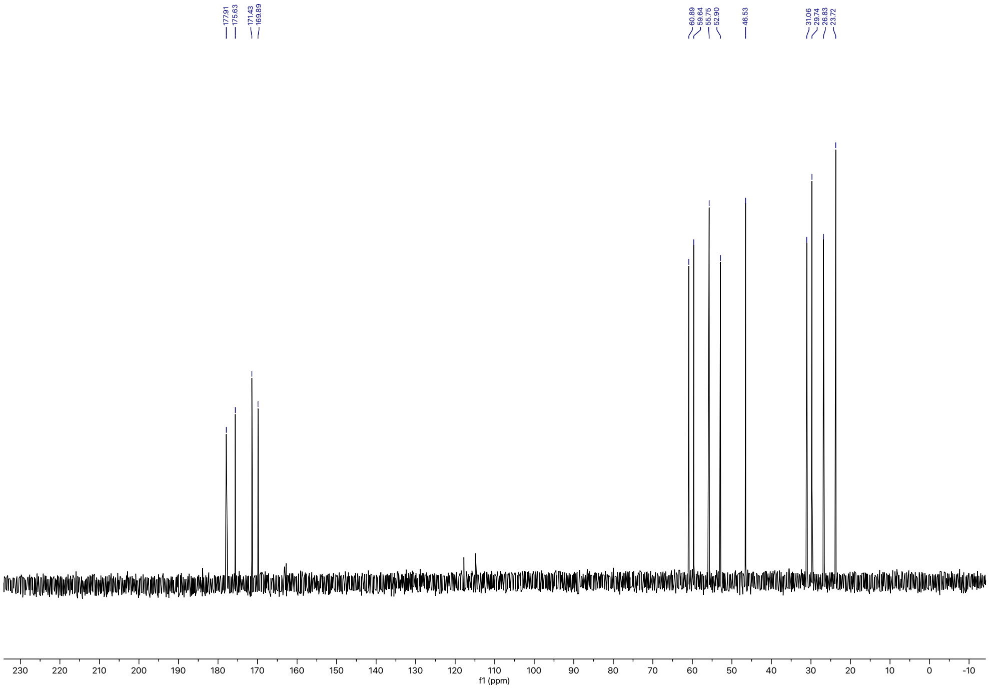

^{1}H and ^{13}C-NMR of *P47:*

^{1}H NMR (400 MHz, D_2O)

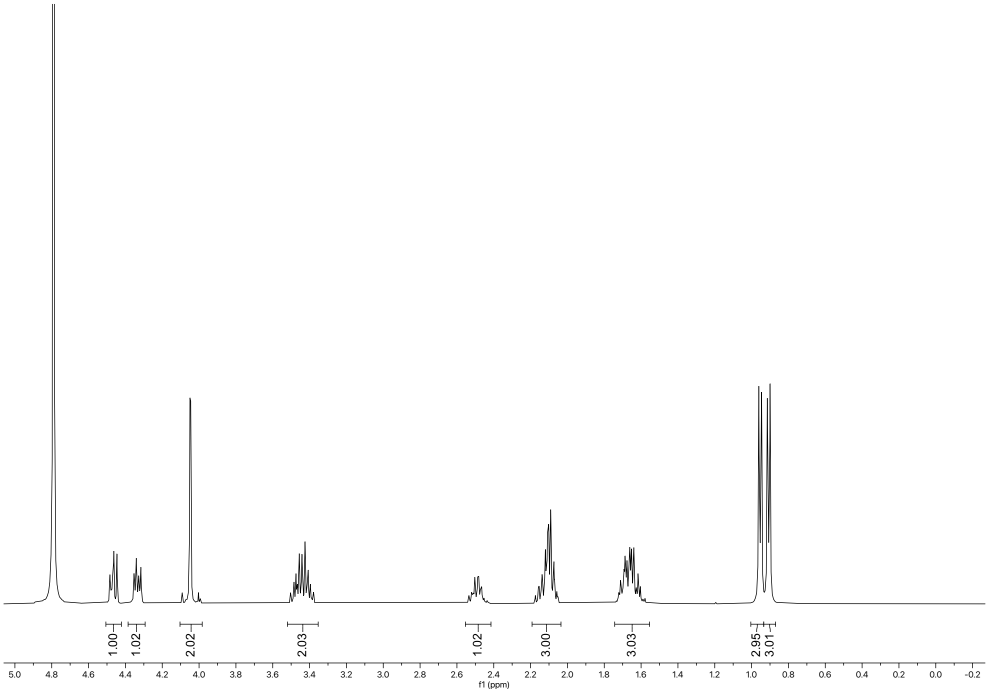

^{13}C NMR (101 MHz, D_2O)

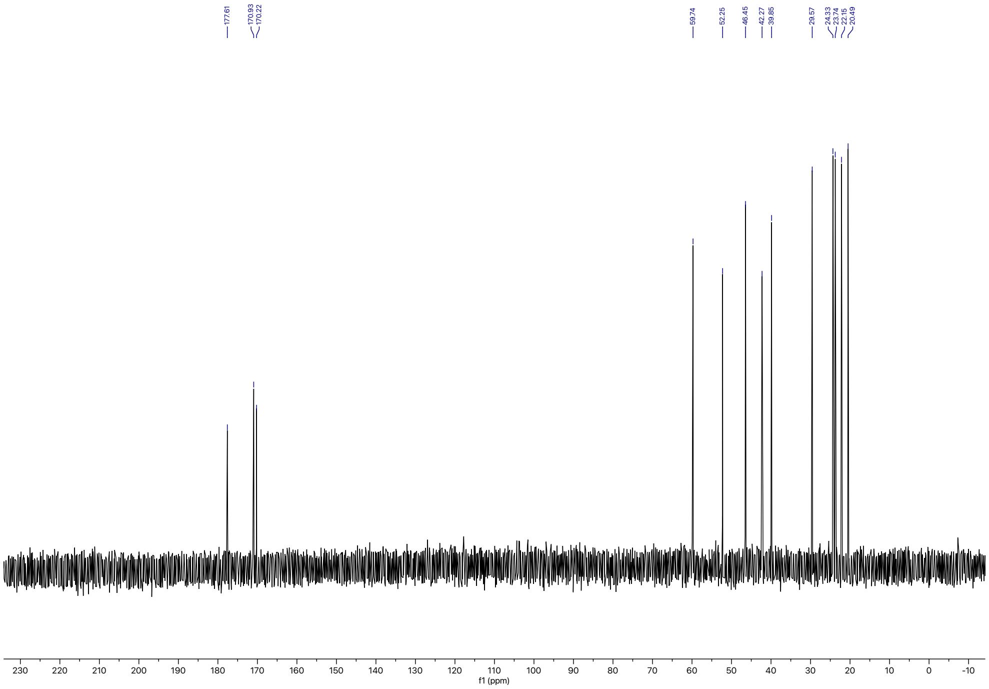

^{1}H and ^{13}C-NMR of *P48:*

^{1}H NMR (400 MHz, D_2O)

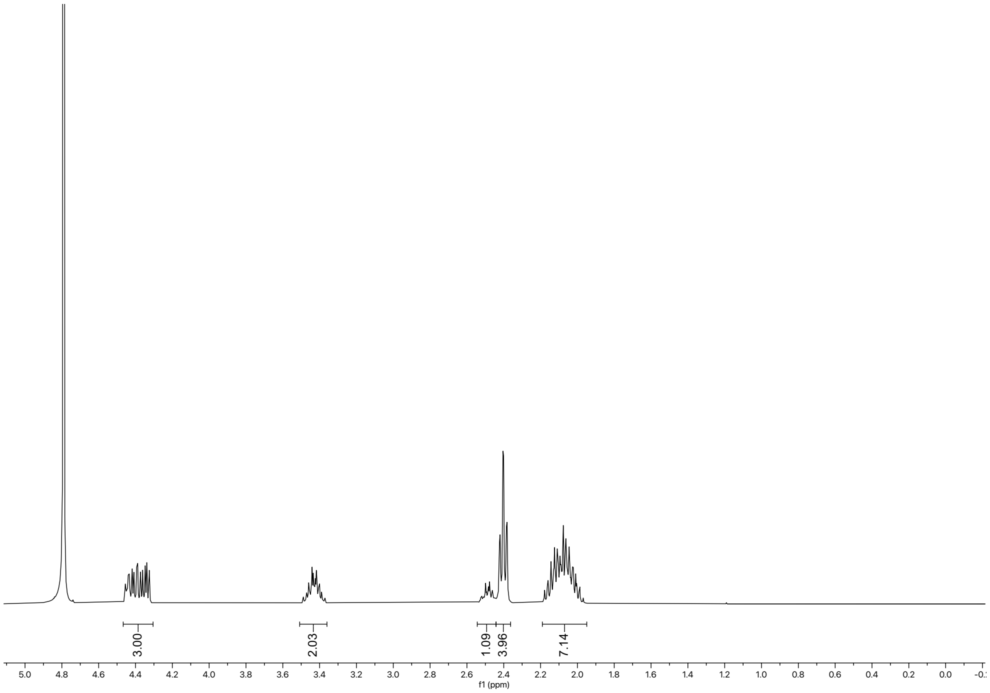

^{13}C NMR (101 MHz, D_2O)

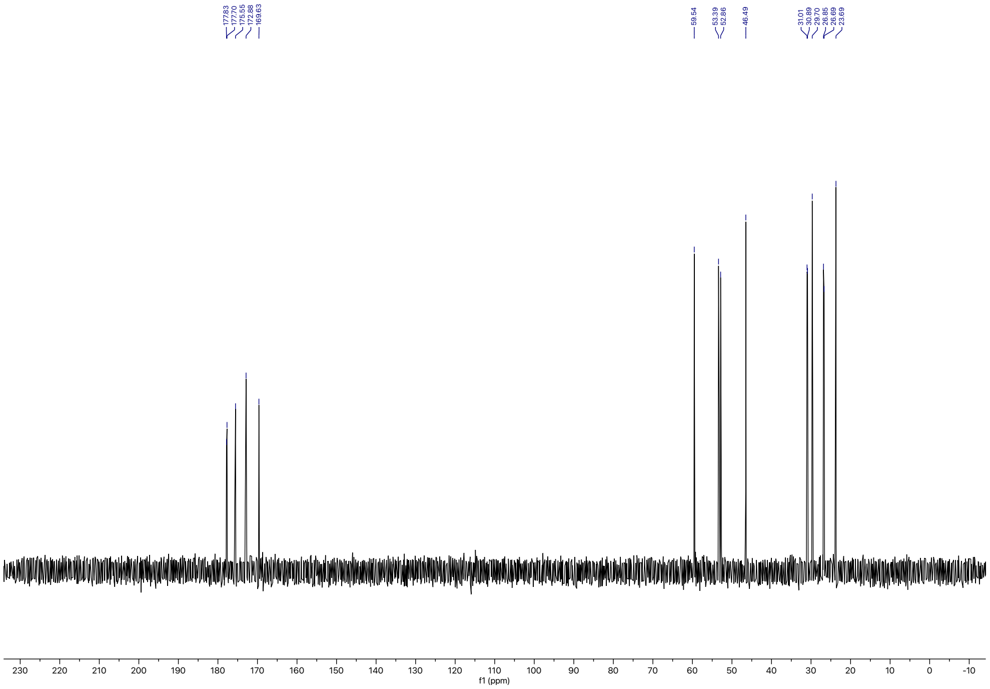

^{1}H and ^{13}C-NMR of *P49:*

^{1}H NMR (400 MHz, D_2O)

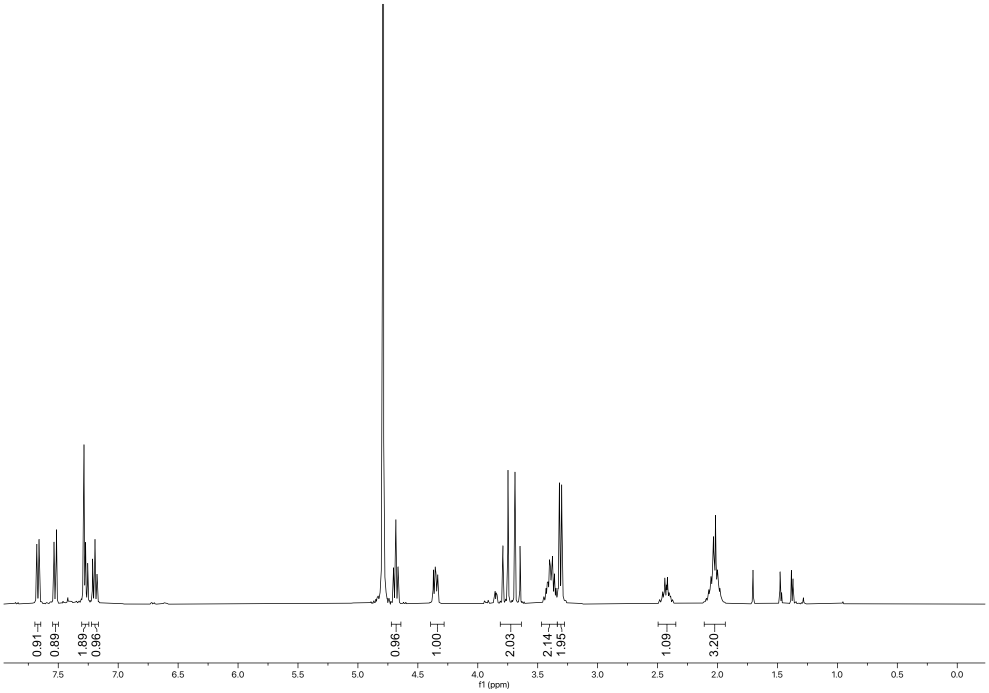

^{13}C NMR (101 MHz, D_2O)

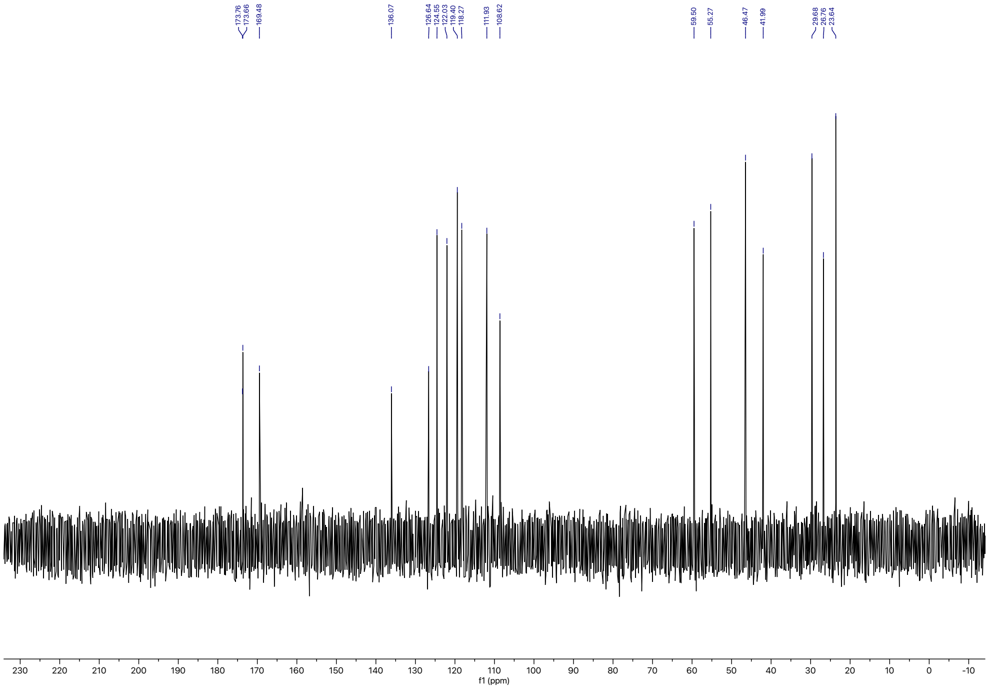

^{1}H and ^{13}C-NMR of *P50:*

^{1}H NMR (400 MHz, D_2O)

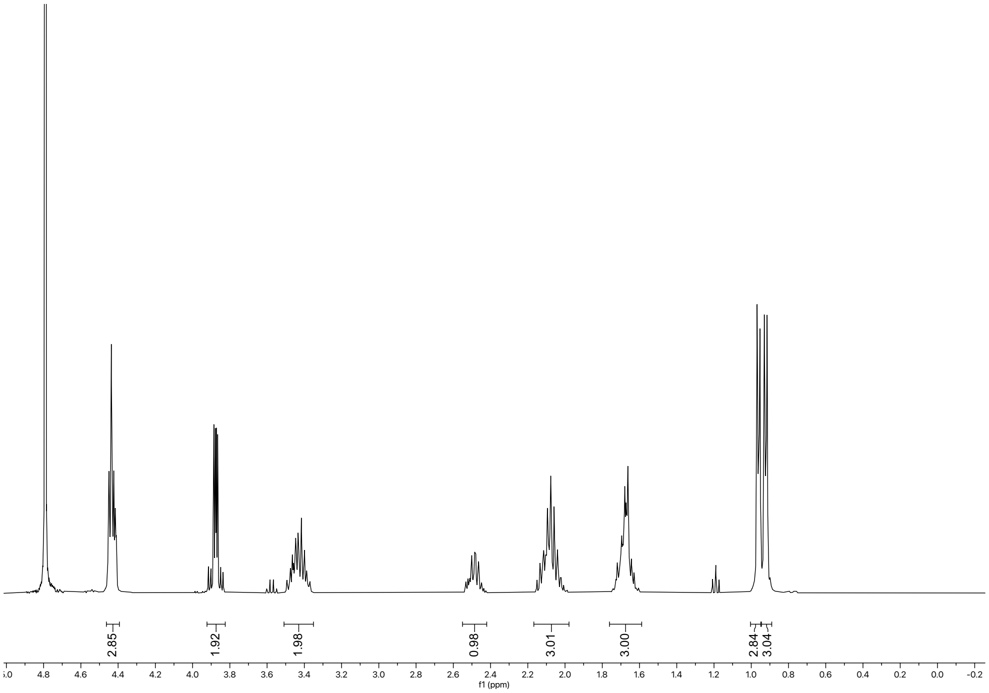

^{13}C NMR (101 MHz, D_2O)

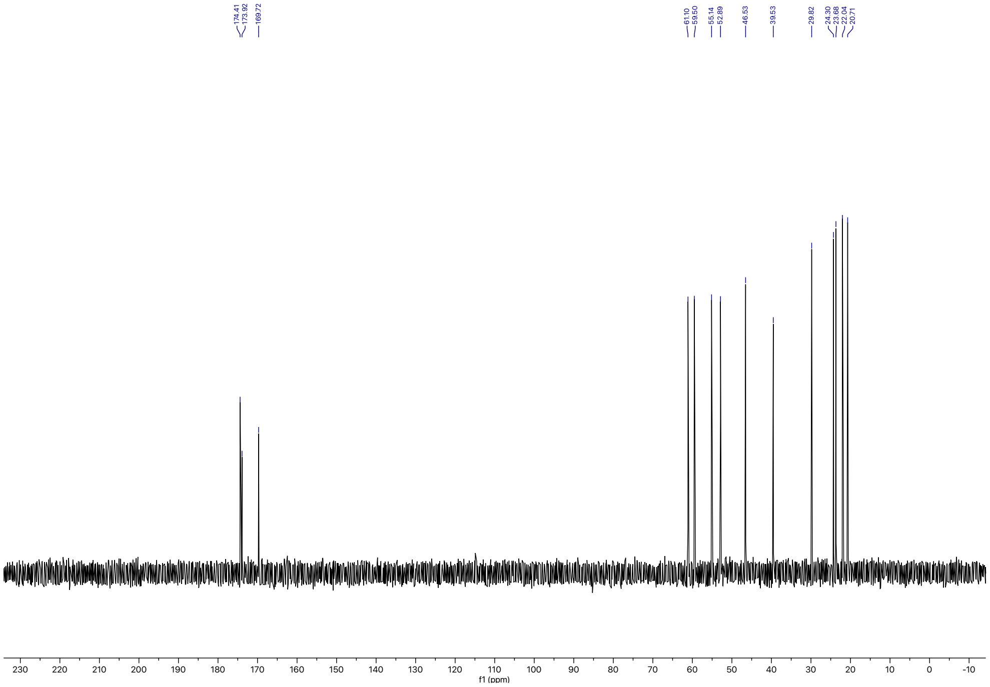

^{1}H and ^{13}C-NMR of H-D-Pro-D-Pro-D-Pro-NH_2 · TFA (UTS-1)

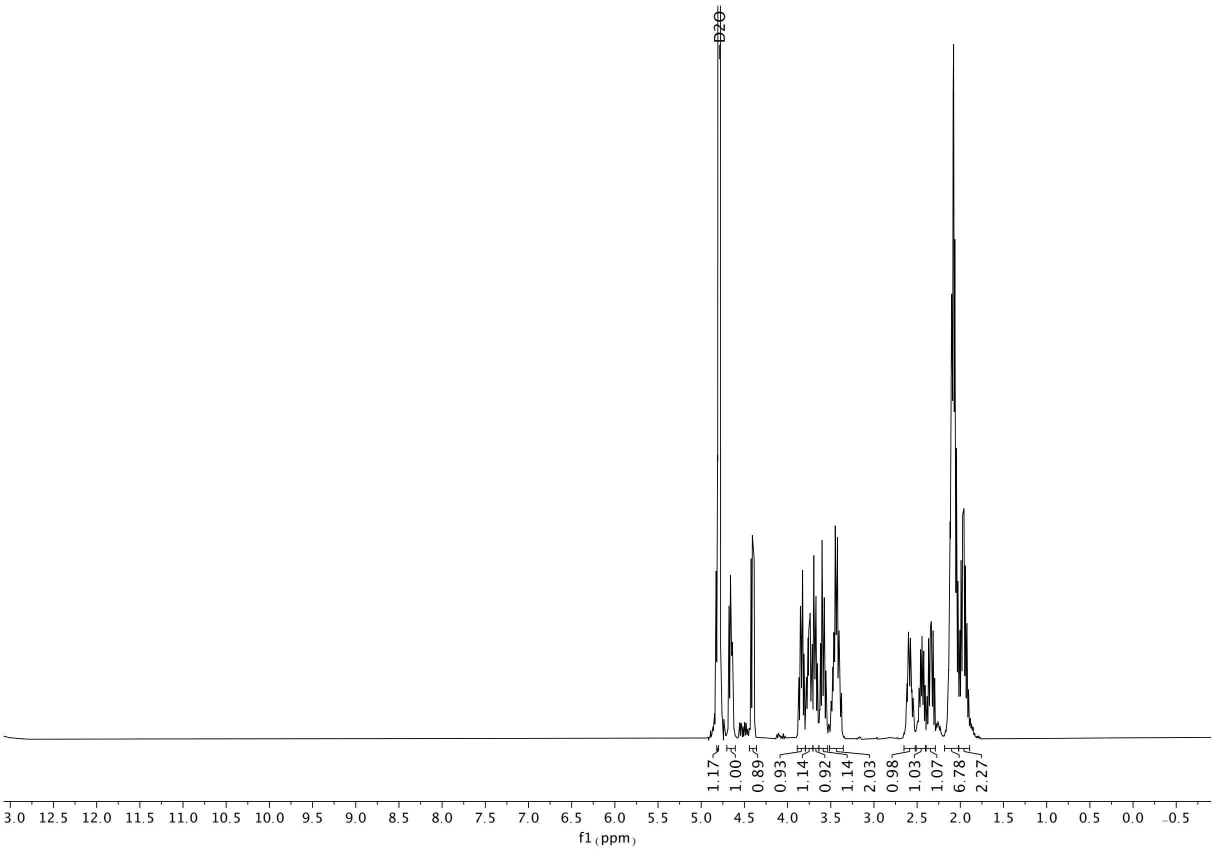

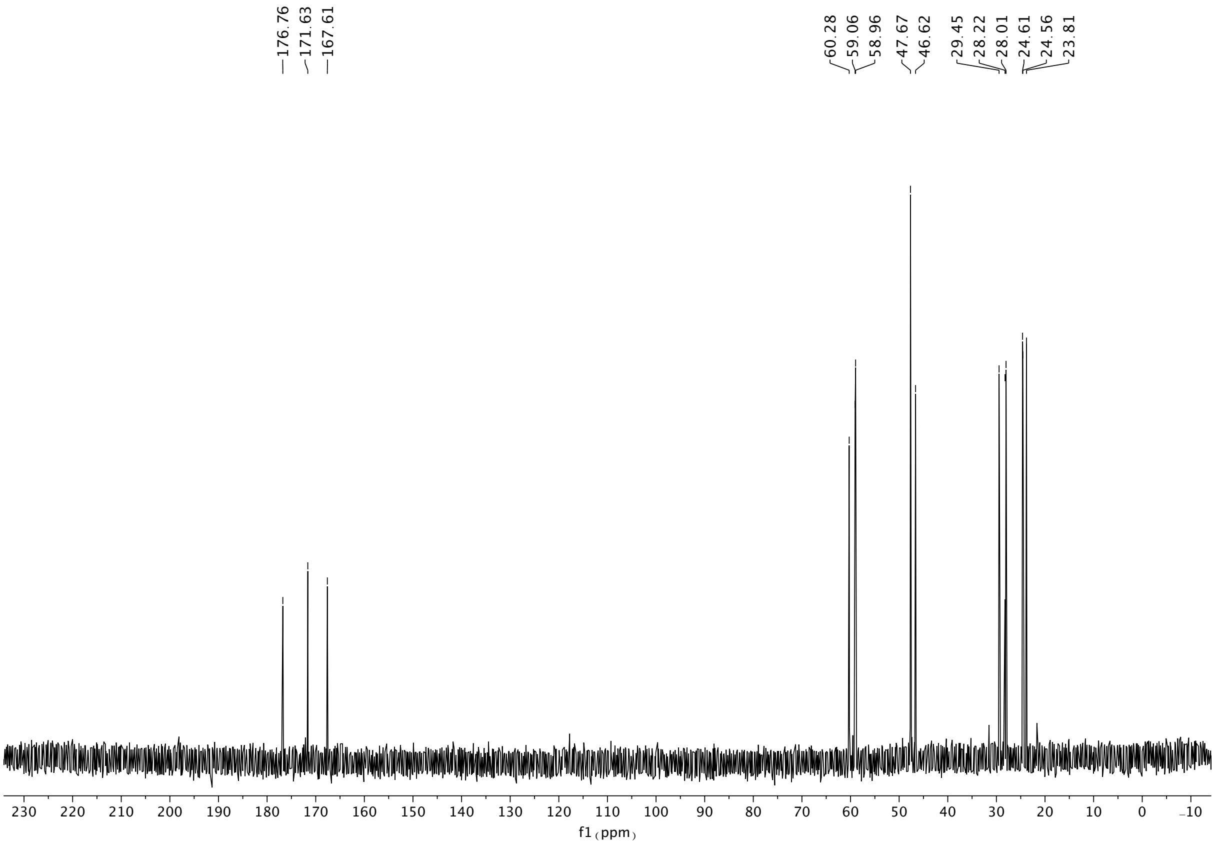

^{1}H and ^{13}C-NMR of H-D-Pro-L-(4*S*)-Flp-D-Pro-NH_2 · TFA (UTS-2)

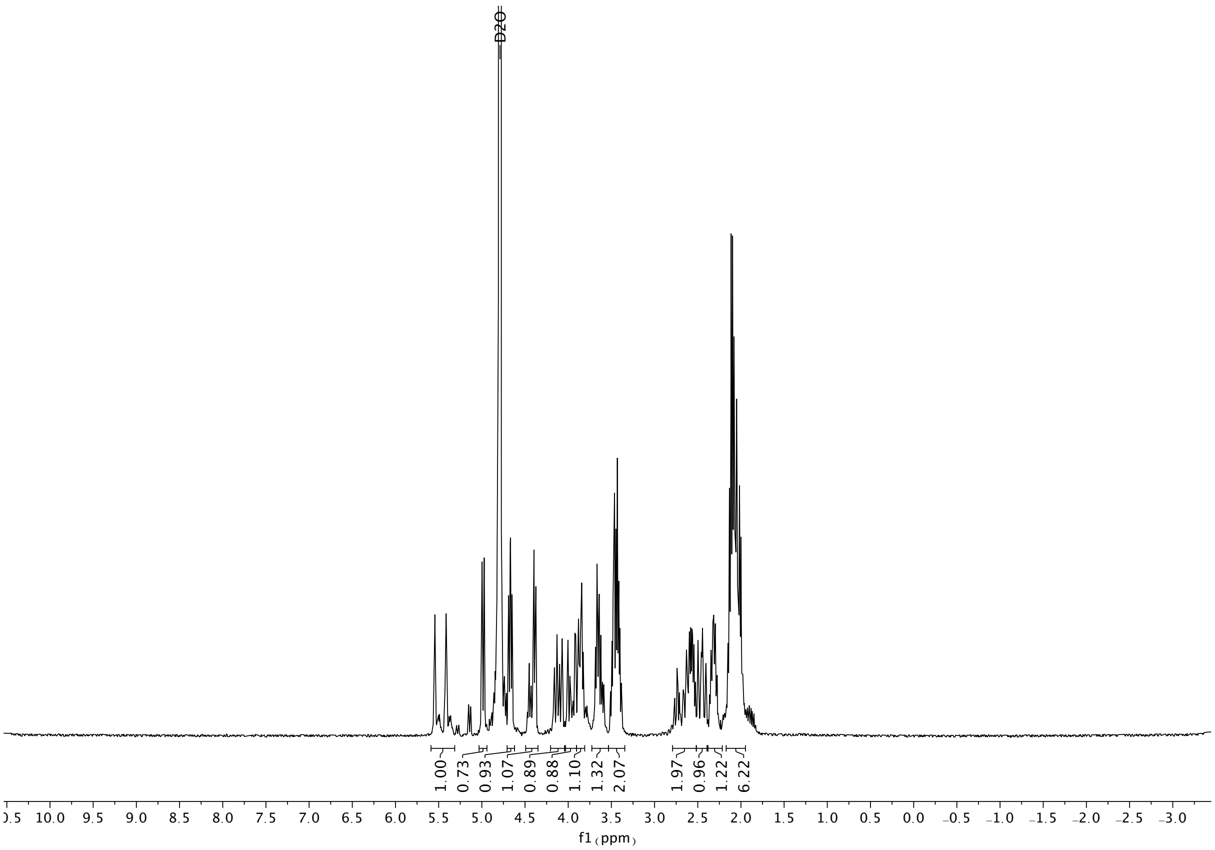

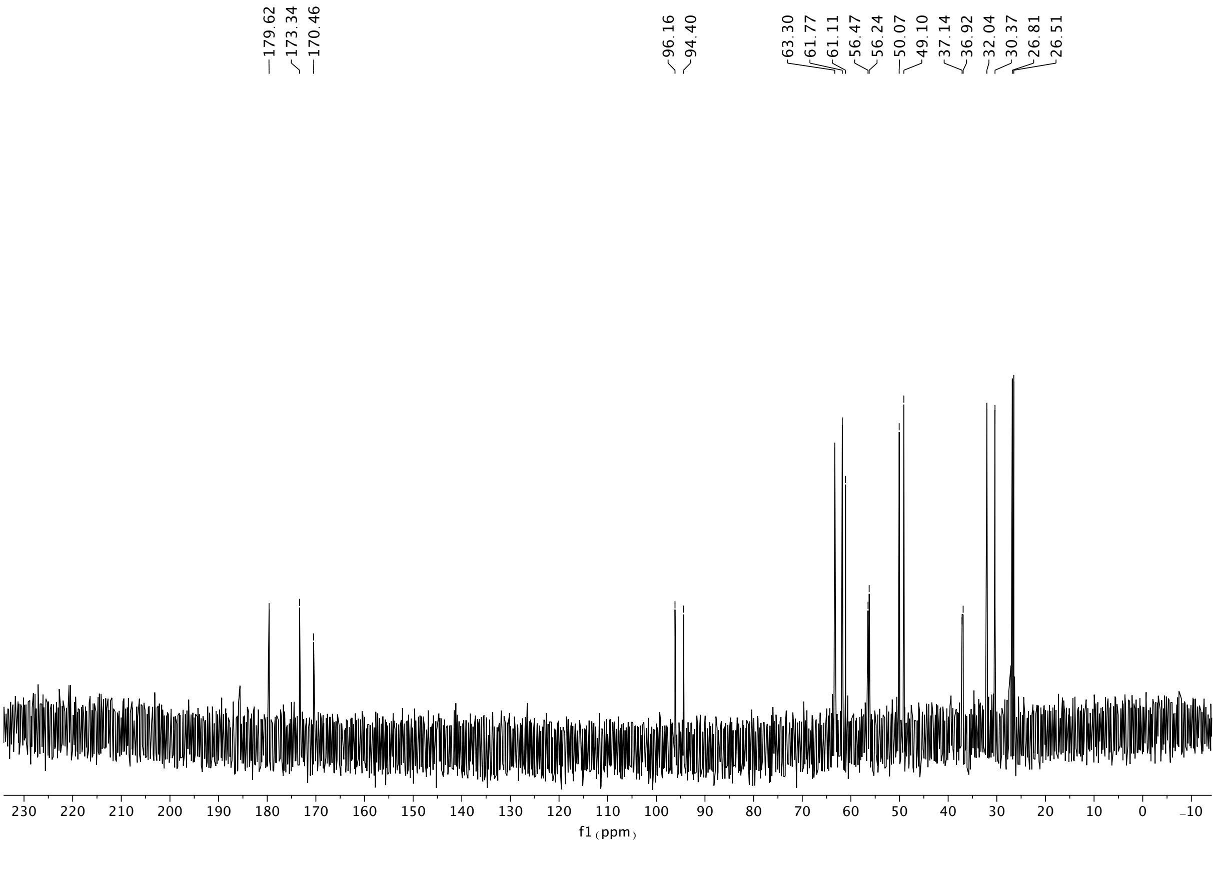

^{1}H and ^{13}C-NMR of H-D-Pro-L-Leu-D-Pro-NH$_2$ · TFA (UTS-3)

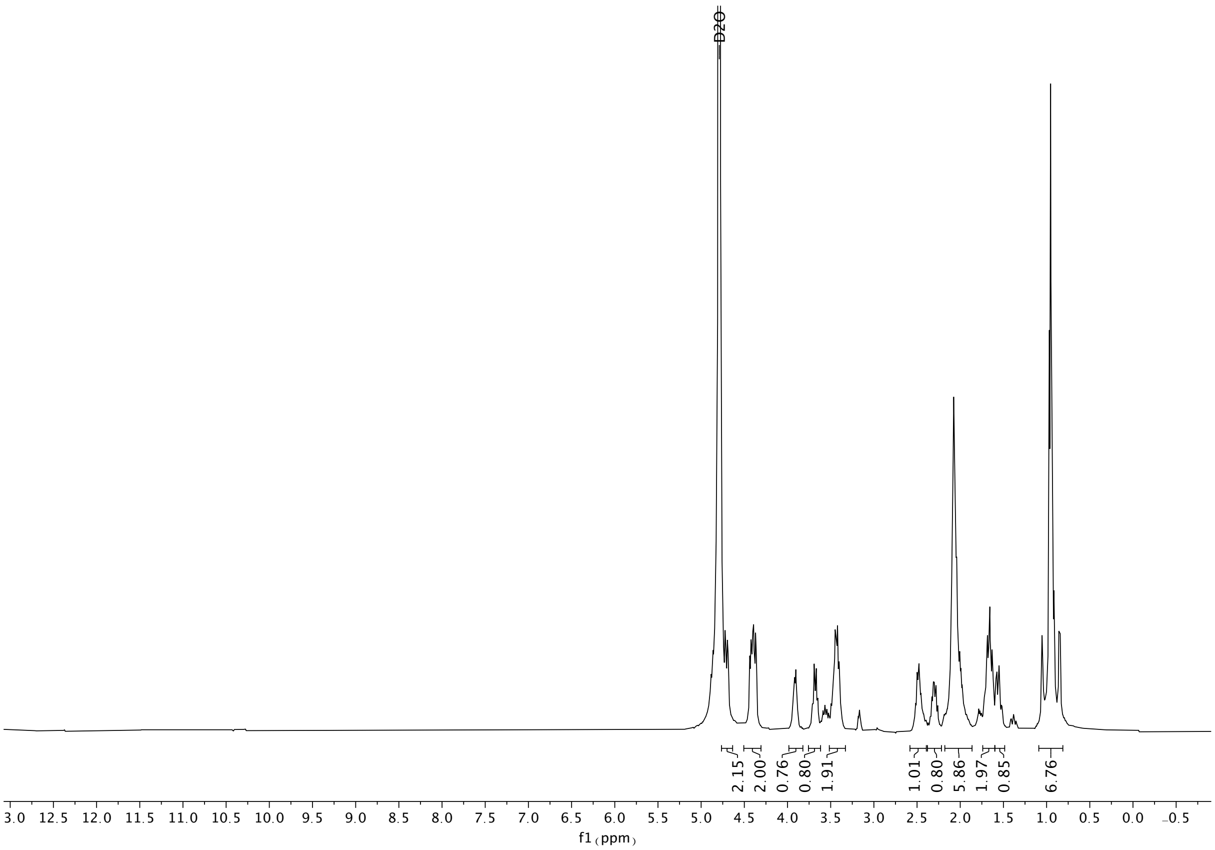

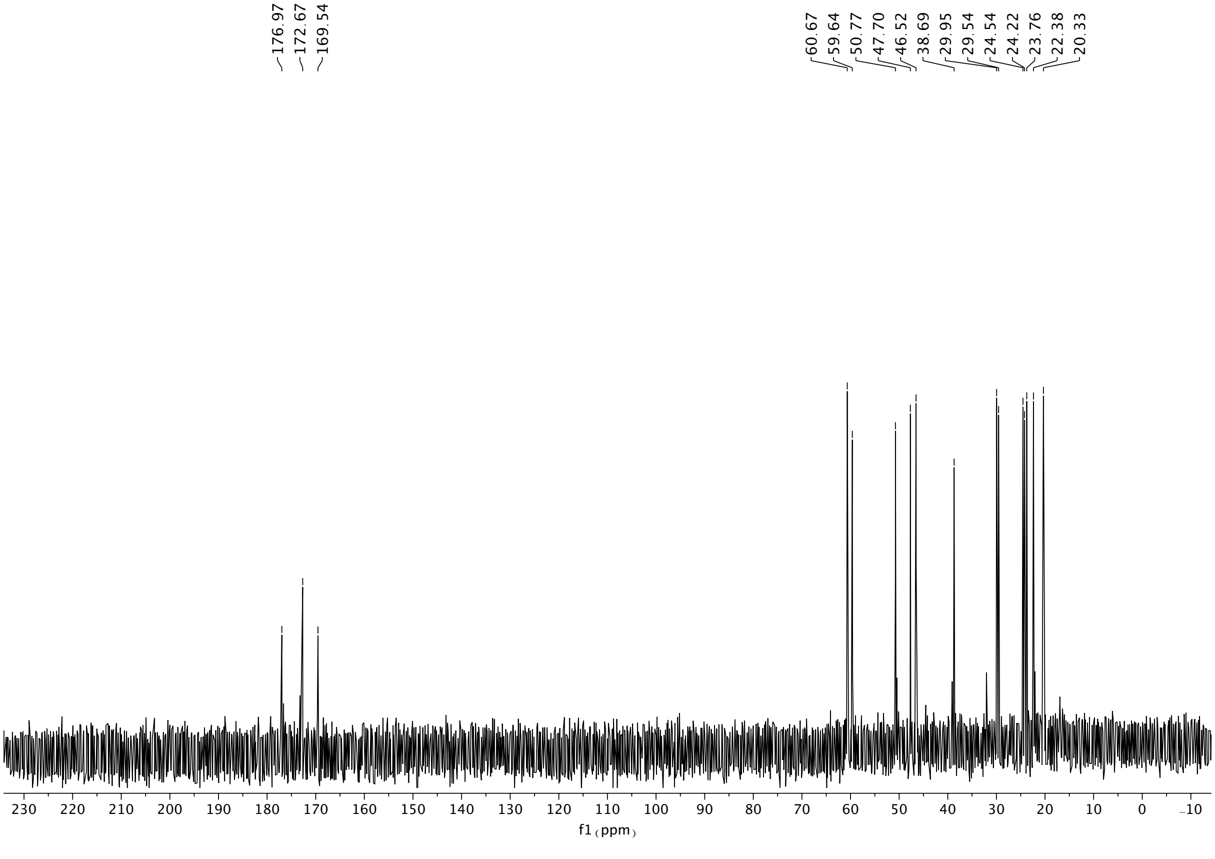

^{1}H and ^{13}C-NMR of H-D-Pro-D-Leu-D-Pro-NH$_2$ · TFA (UTS-4)

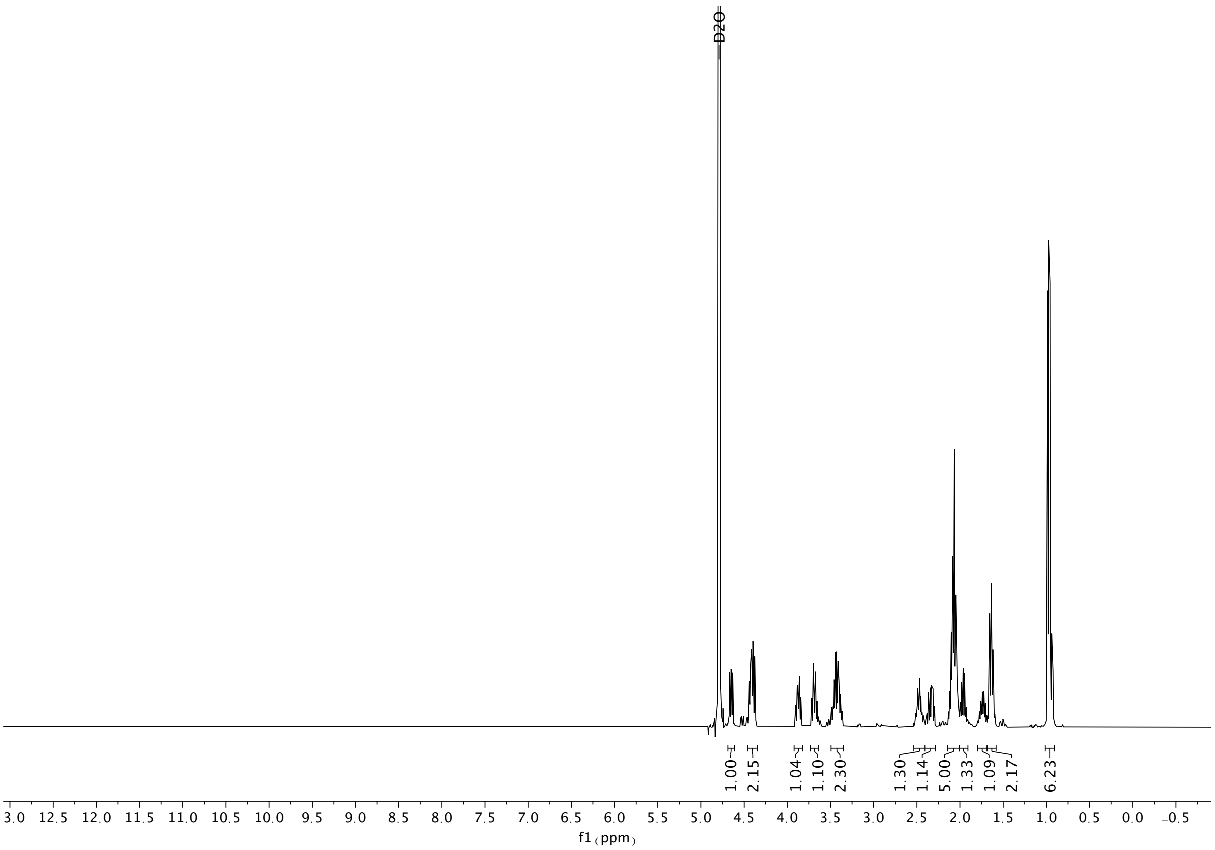

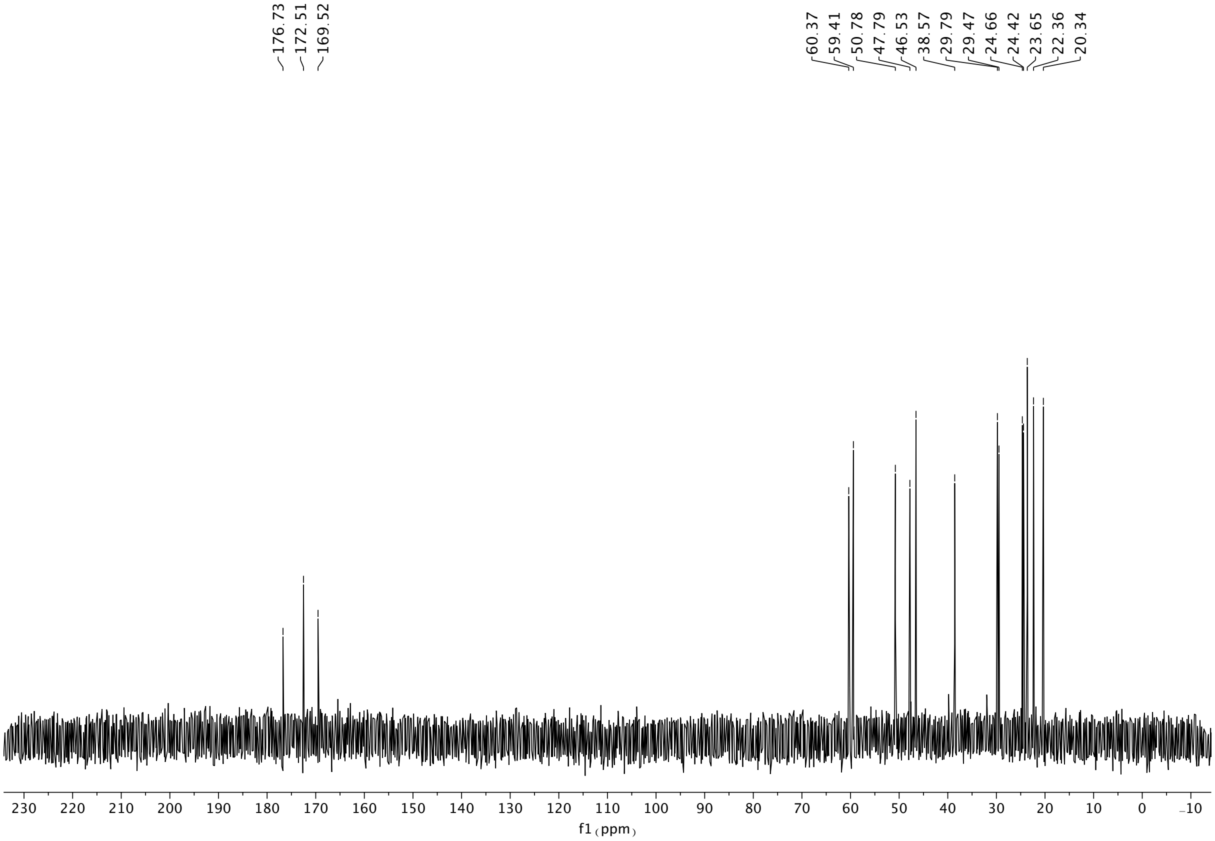

^{1}H and ^{13}C-NMR of H-D-Pro-L-Gln-D-Pro-NH$_2$ · TFA (UTS-5)

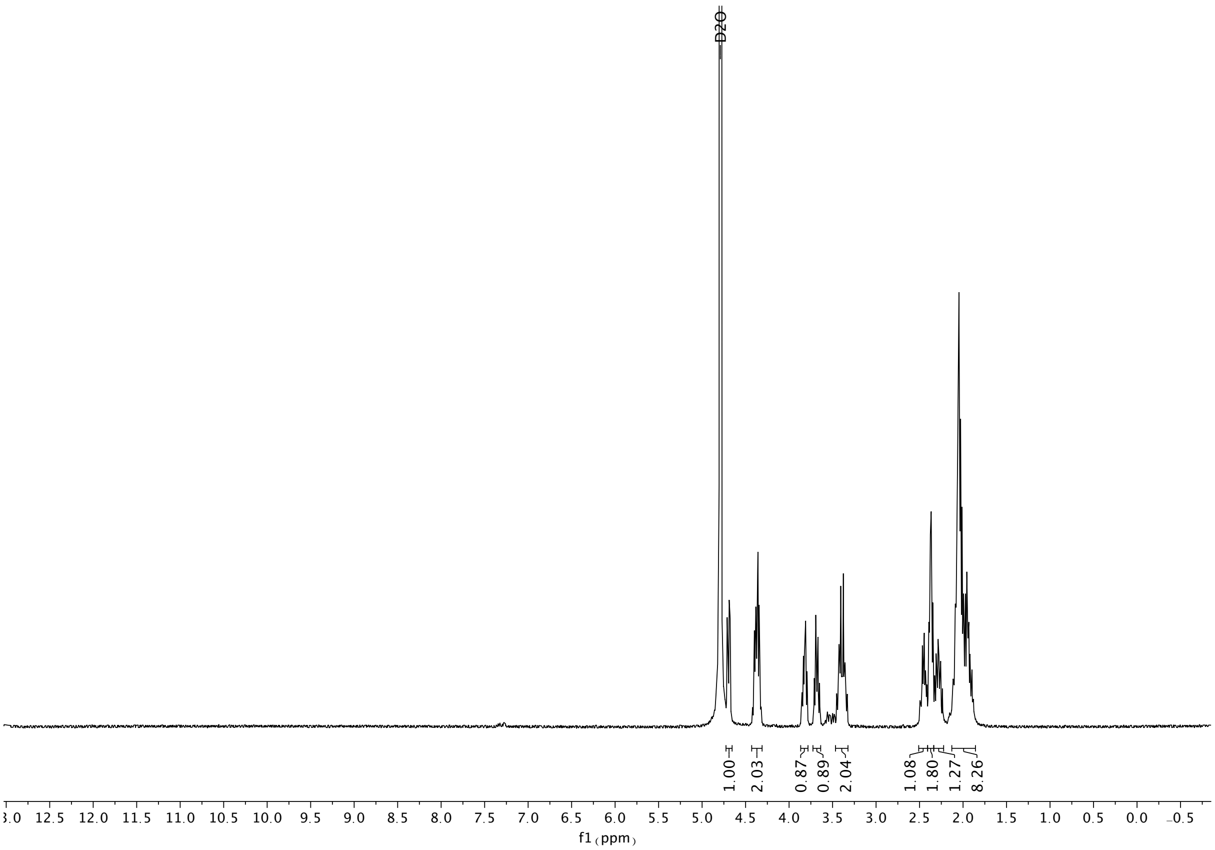

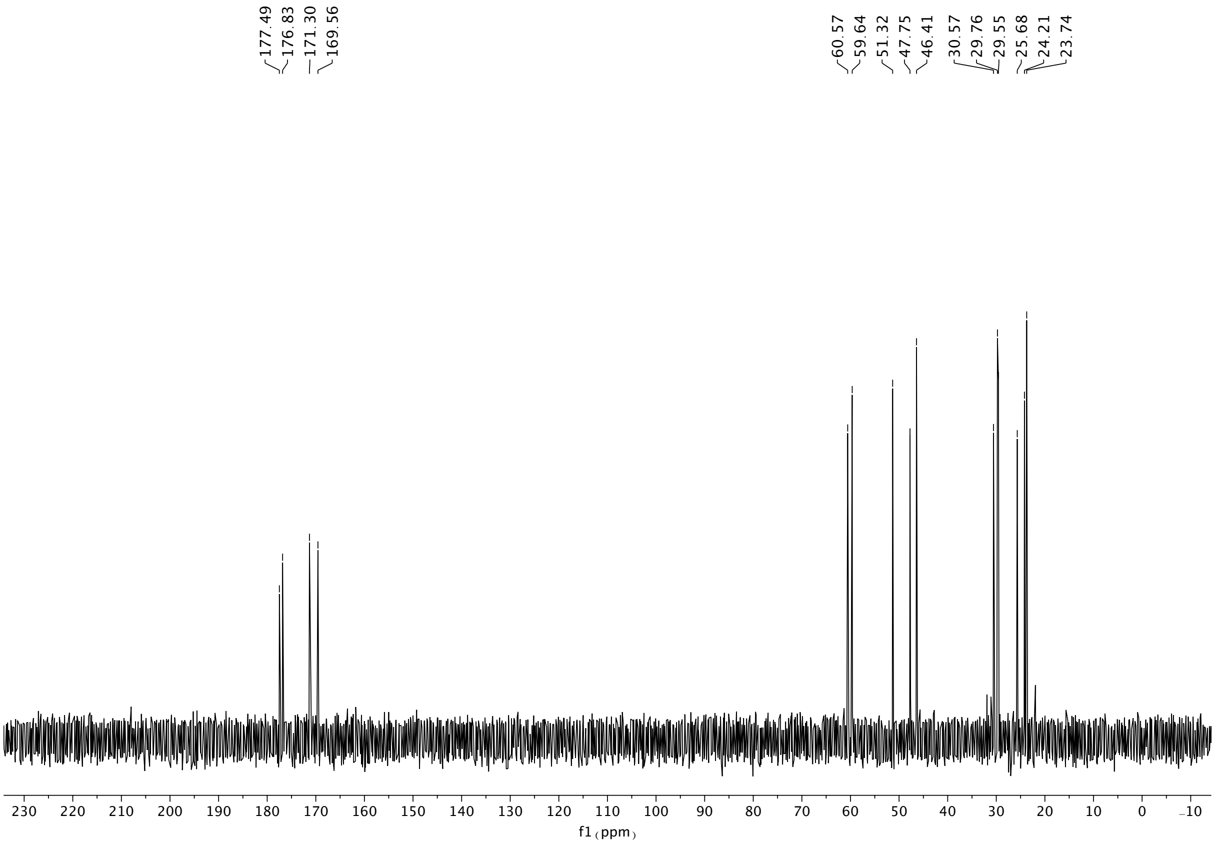

^{1}H and ^{13}C-NMR of H-D-Pro-D-Gln-D-Pro-NH2 · TFA (UTS-6)

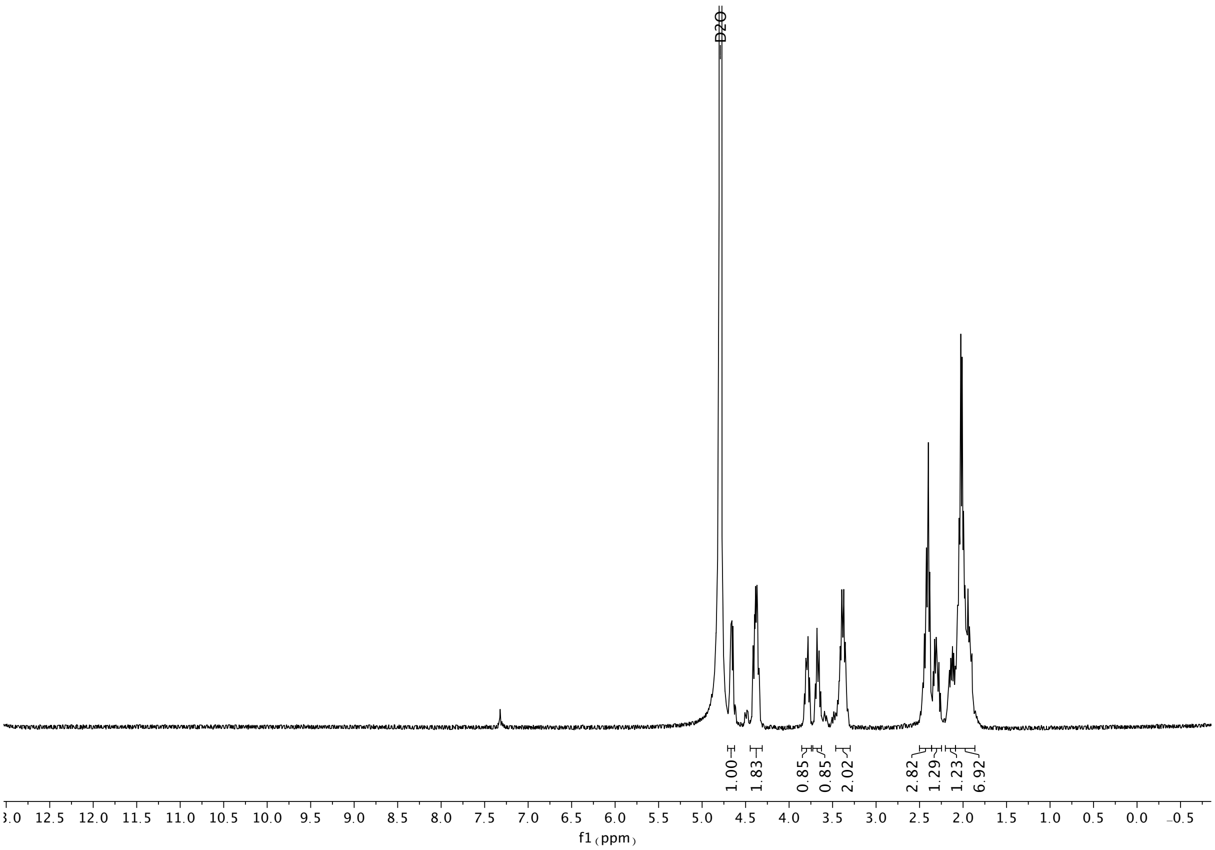

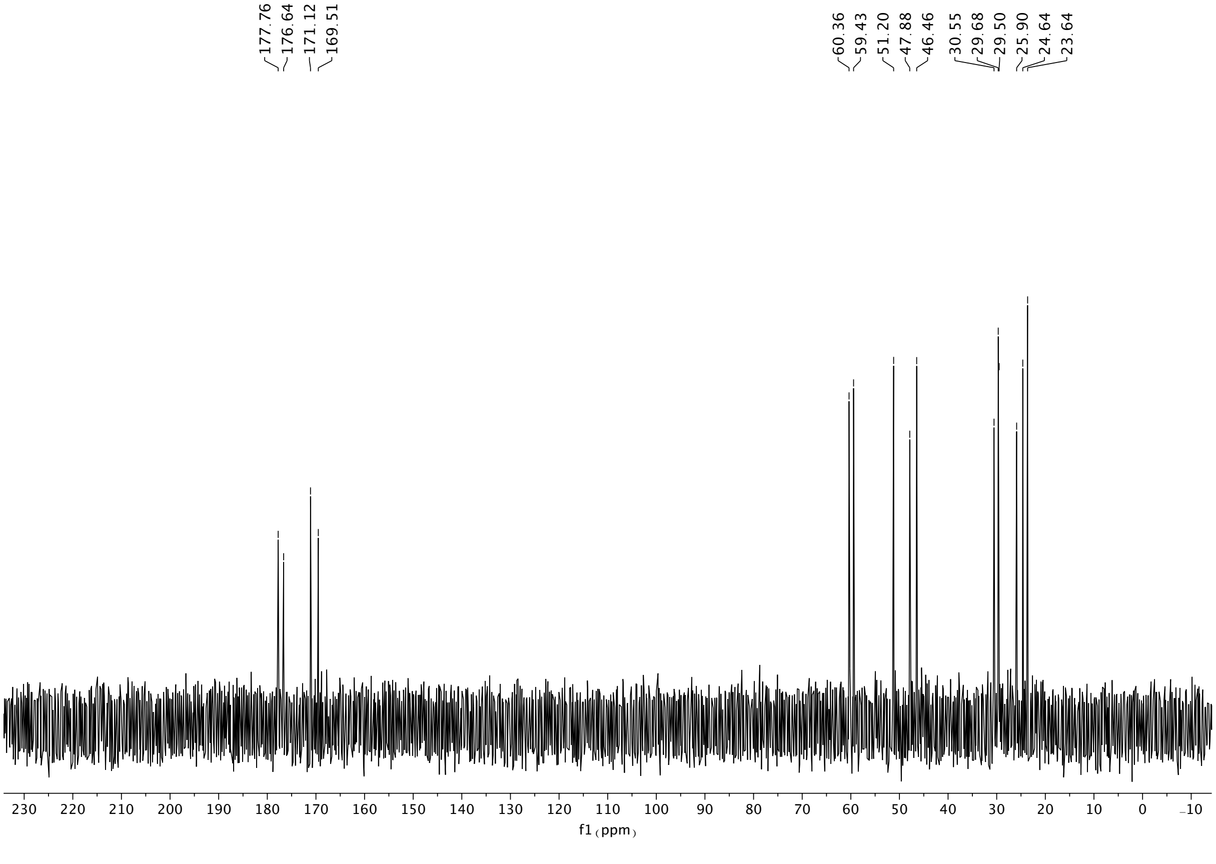

^{1}H and ^{13}C-NMR of H-D-Pro-L-Glu-D-Pro-NH$_2$ · TFA (UTS-7)

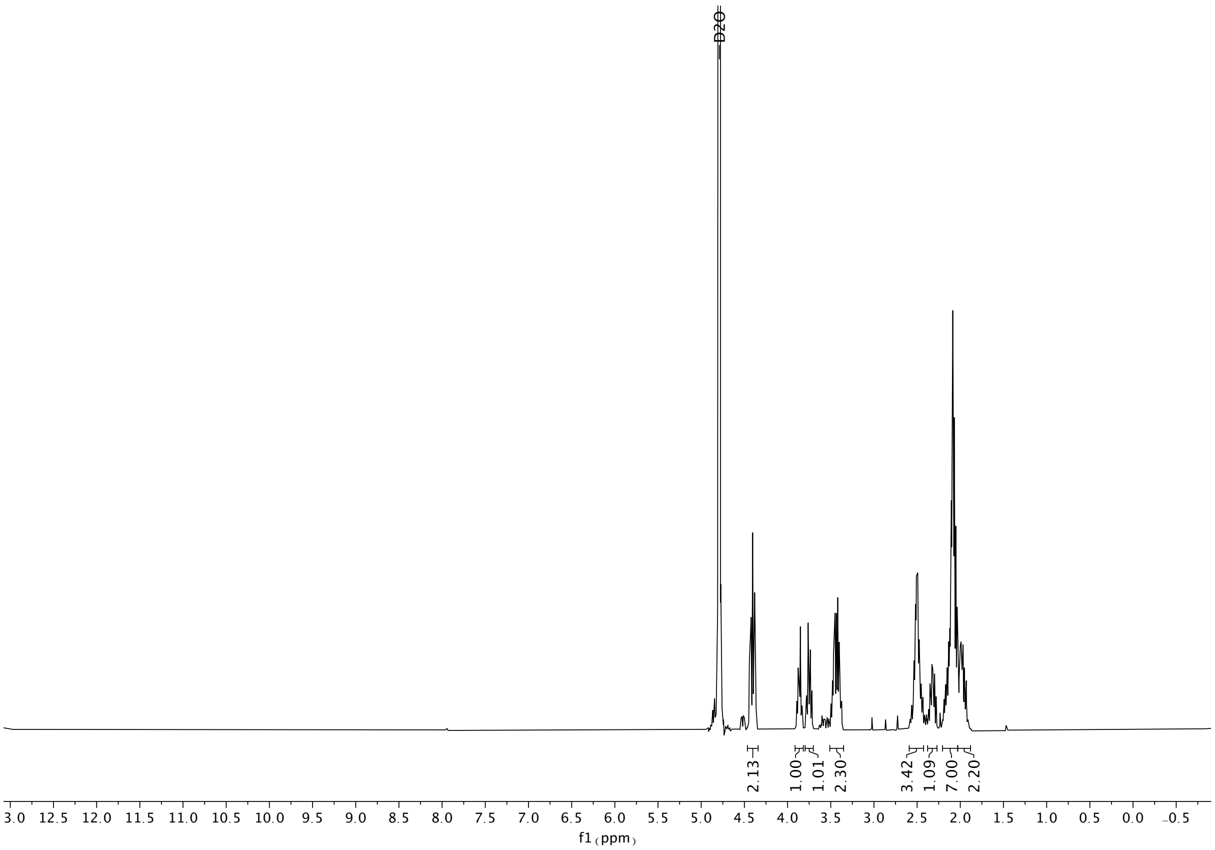

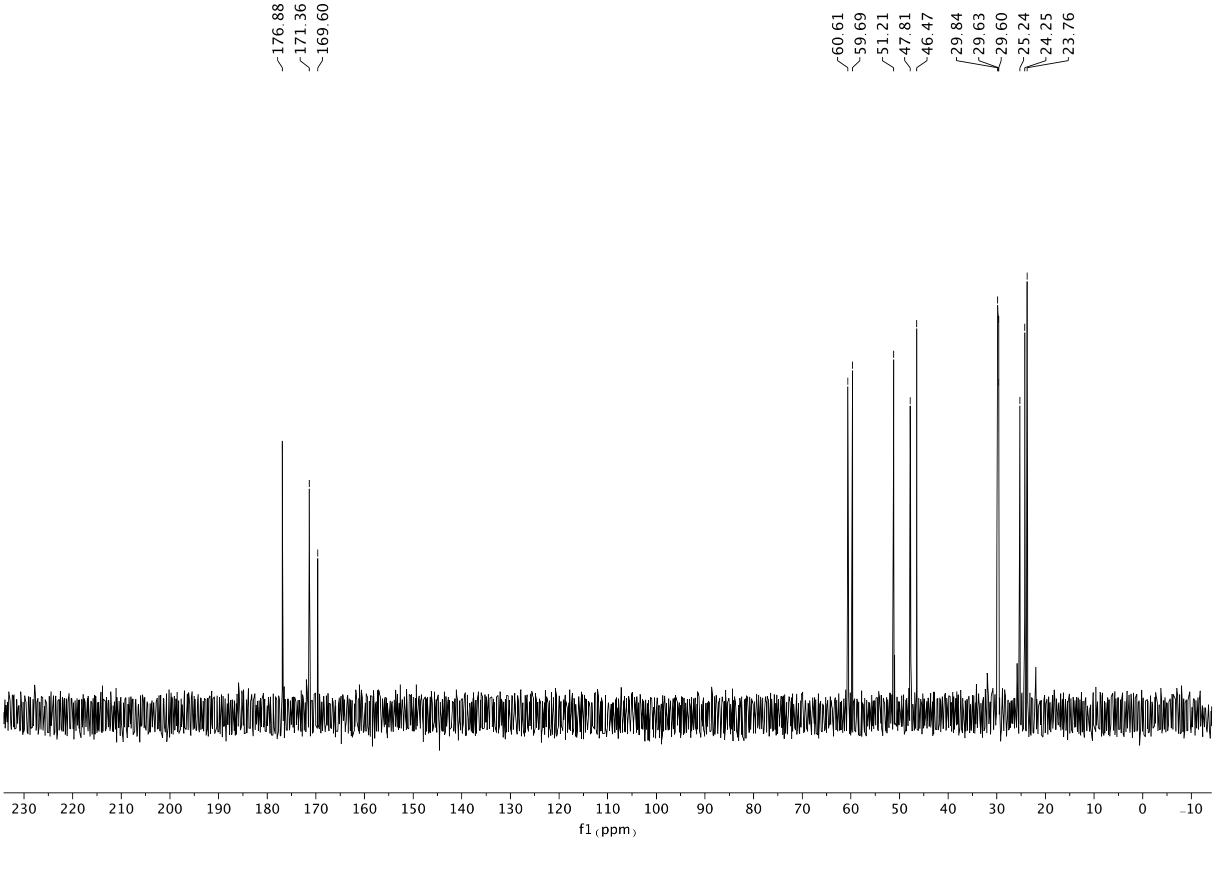

^{1}H and ^{13}C-NMR of H-D-Pro-D-Glu-D-Pro-NH_2 · TFA (UTS-8)

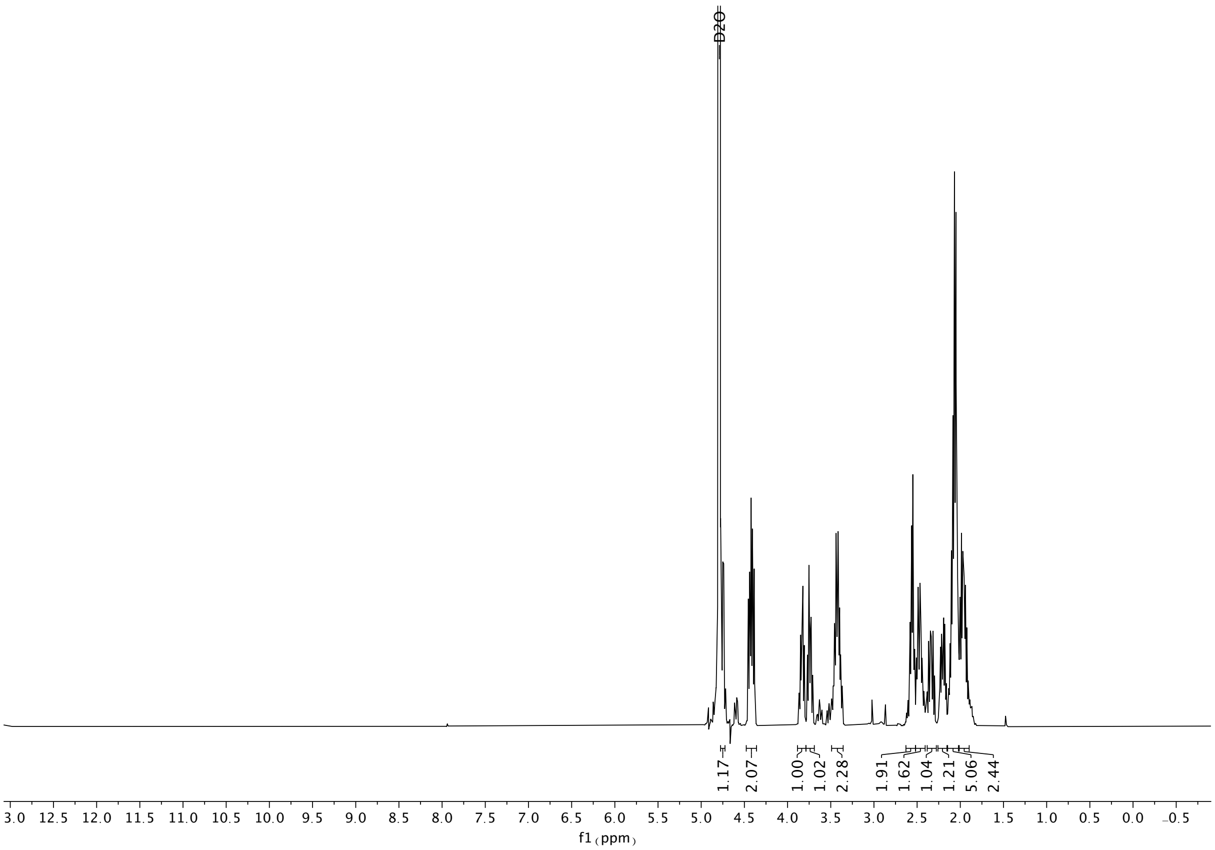

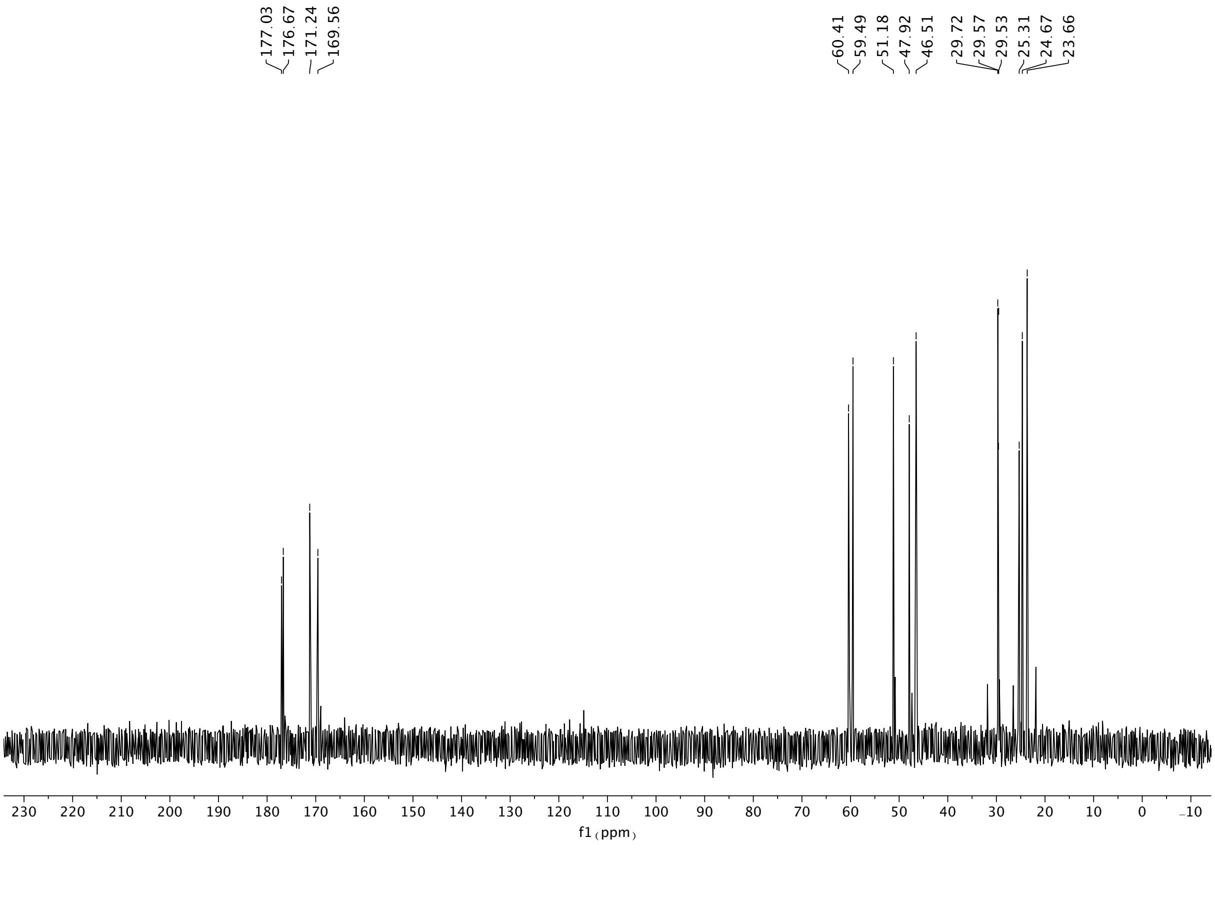

^{1}H and ^{13}C-NMR of H-D-Pro-L-Tyr-D-Pro-NH_2 · TFA (UTS-9)

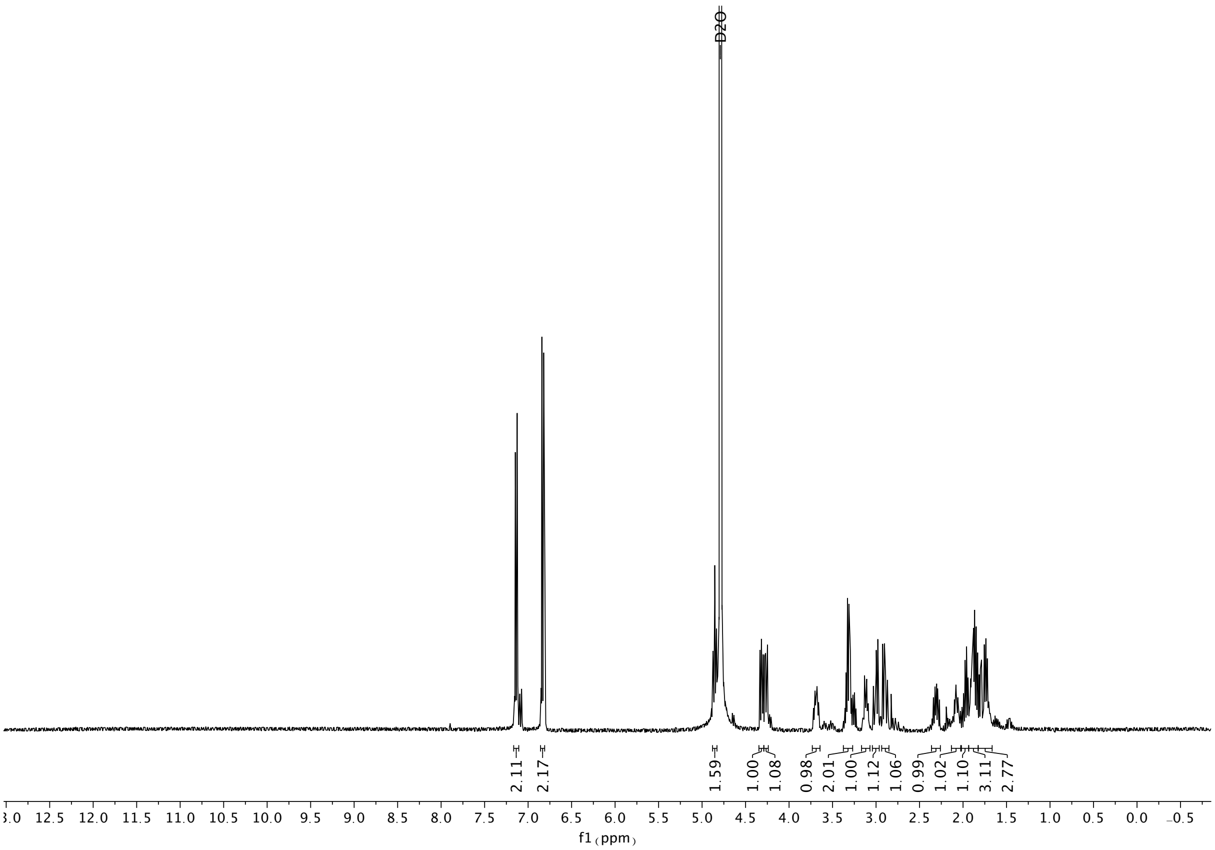

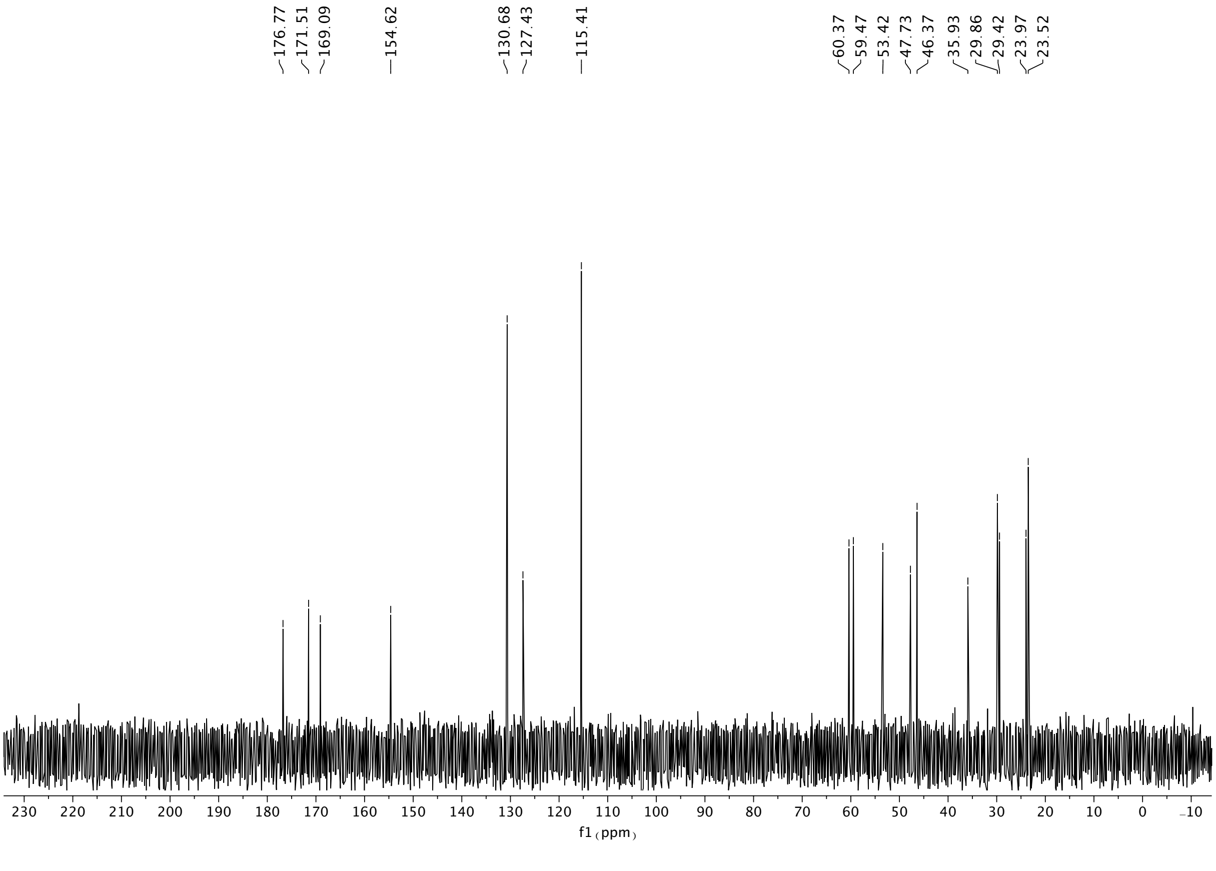

^{1}H and ^{13}C-NMR of H-D-Pro-D-Tyr-D-Pro-NH_2 · TFA (UTS-10)

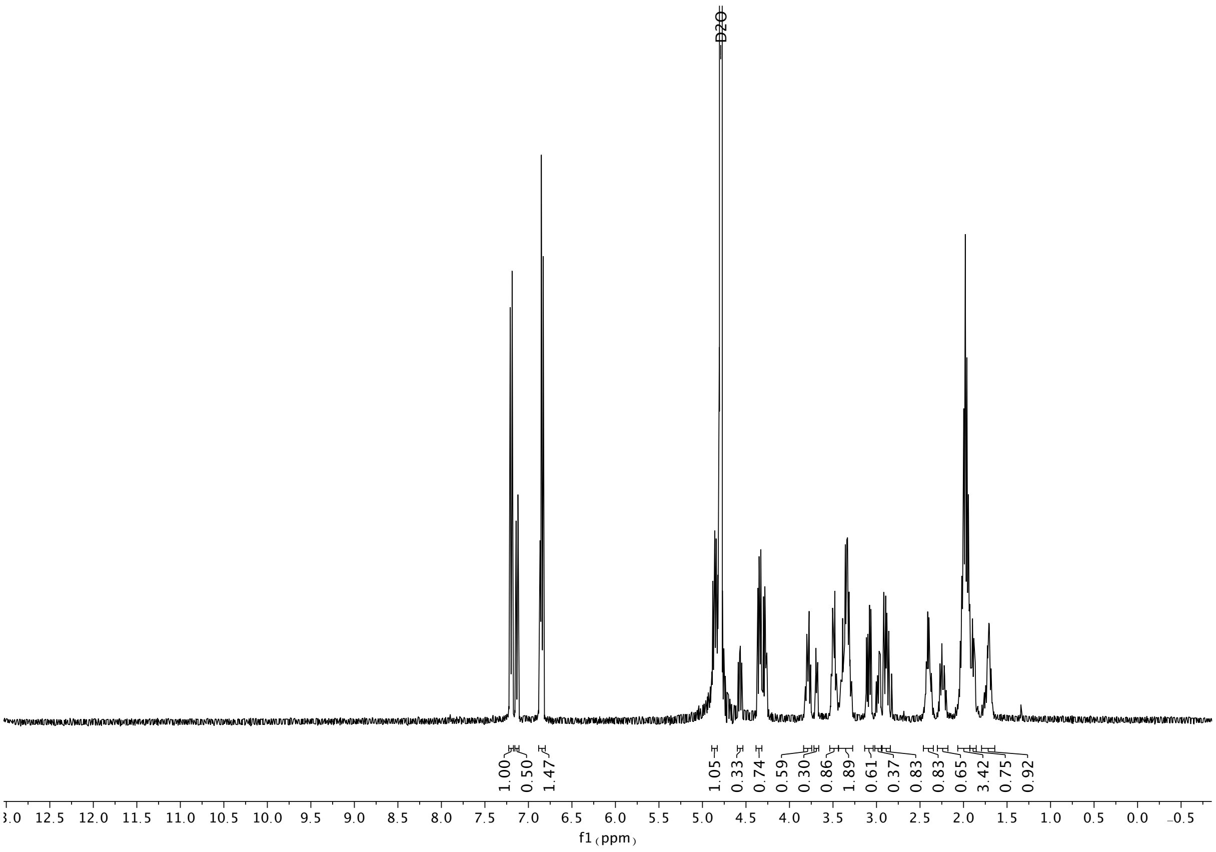

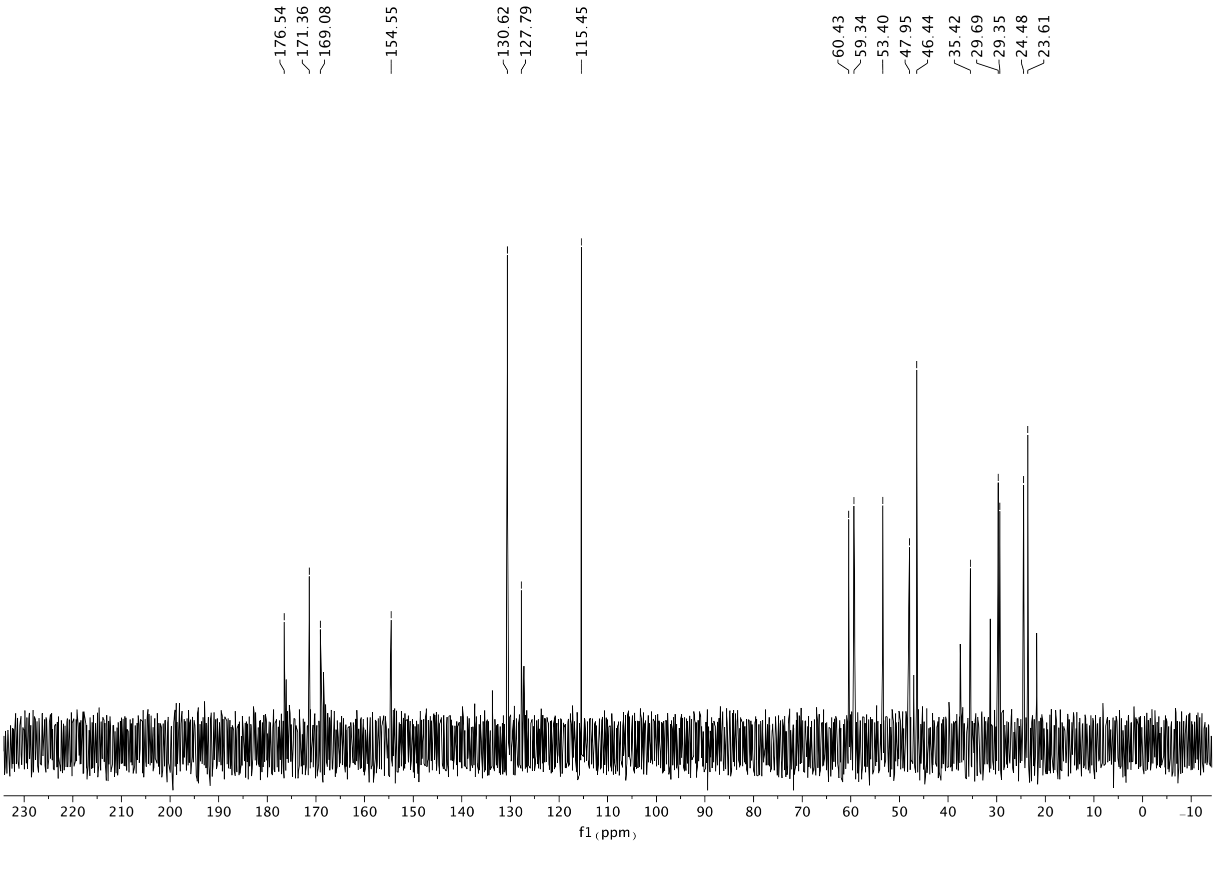

^{1}H and ^{13}C-NMR of H-D-Pro-CyLeu-D-Pro-NH_2 · TFA (UTS-11)

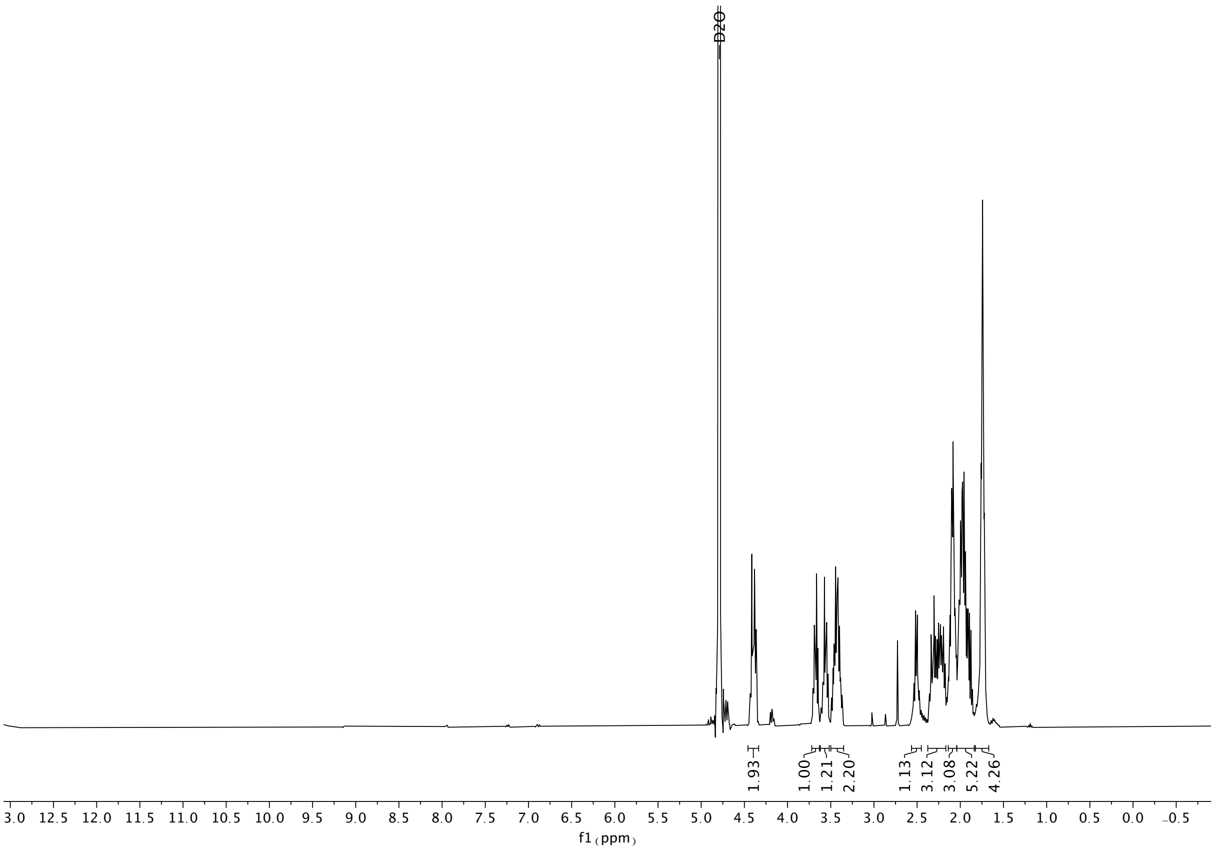

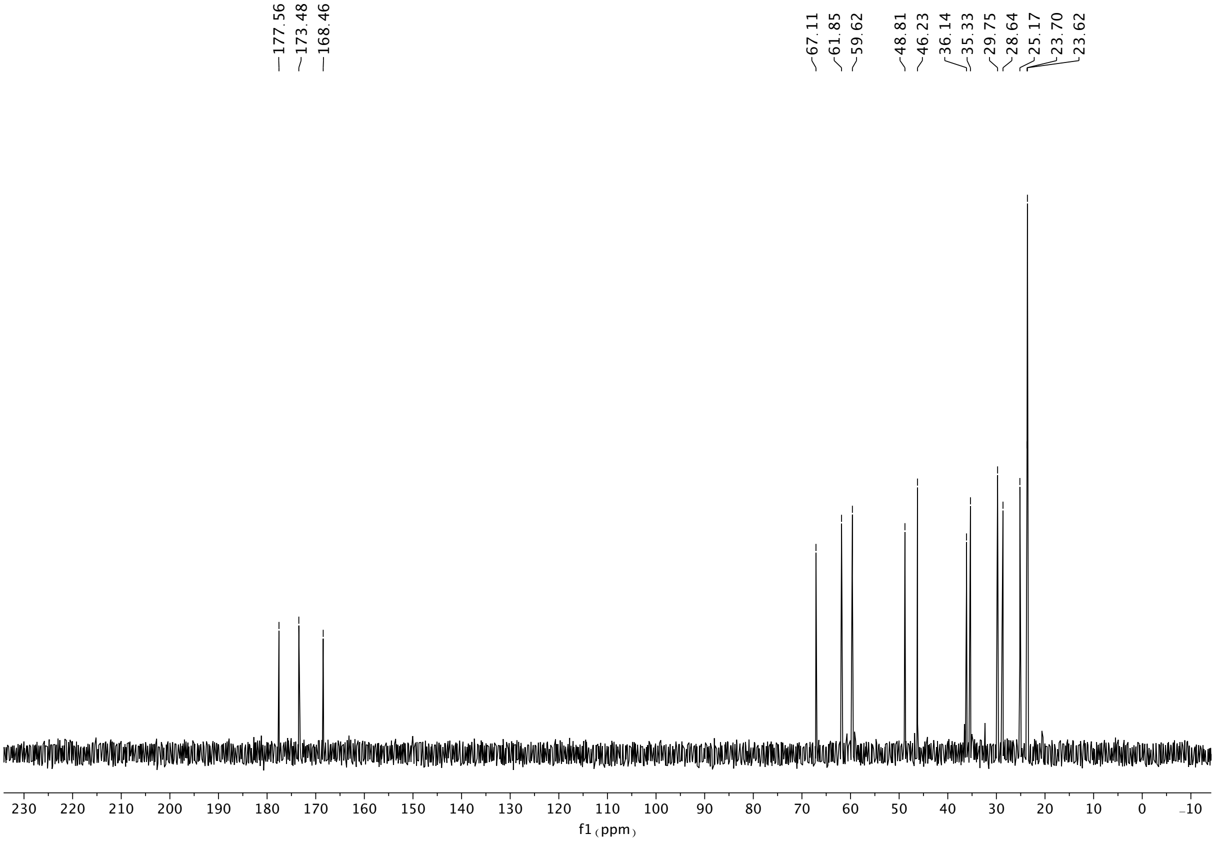

^{1}H and ^{13}C-NMR of H-D-Pro-D-Ind-D-Pro-NH₂ · TFA (UTS-12)

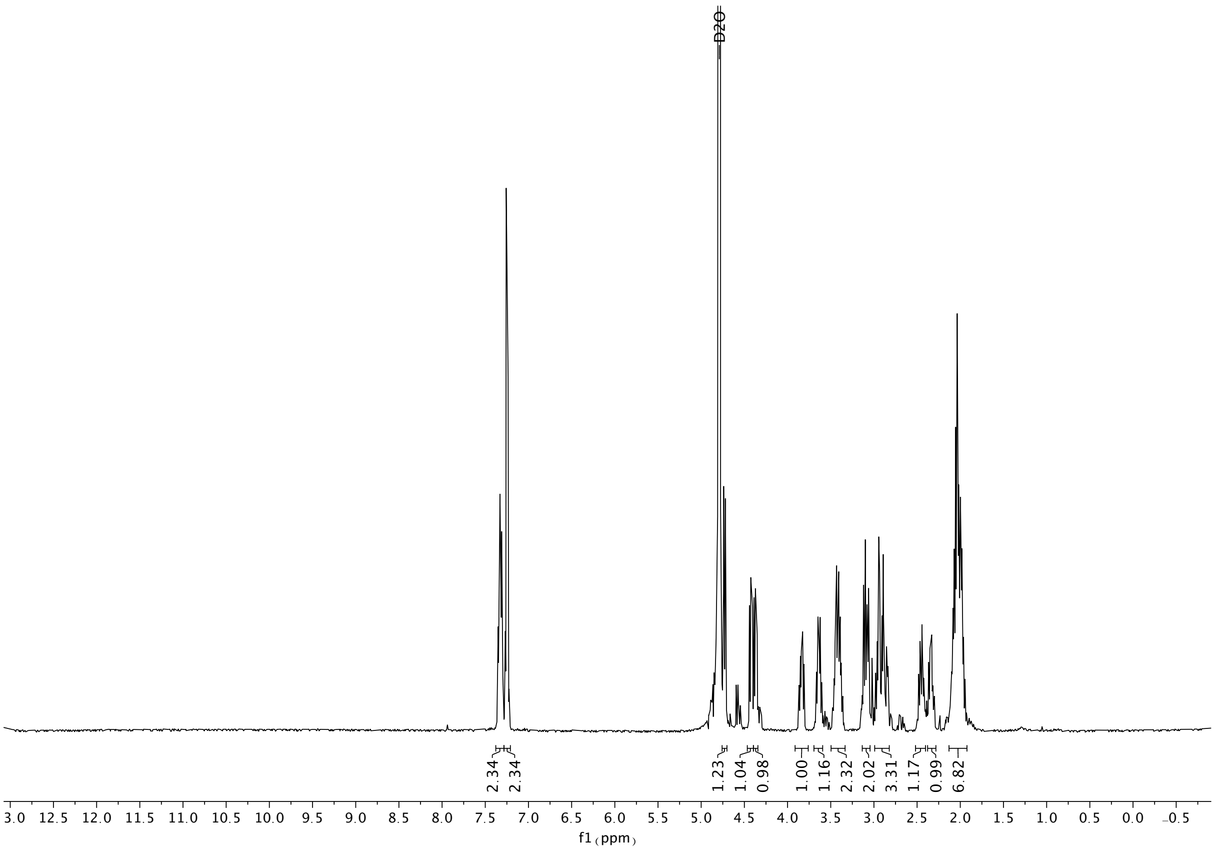

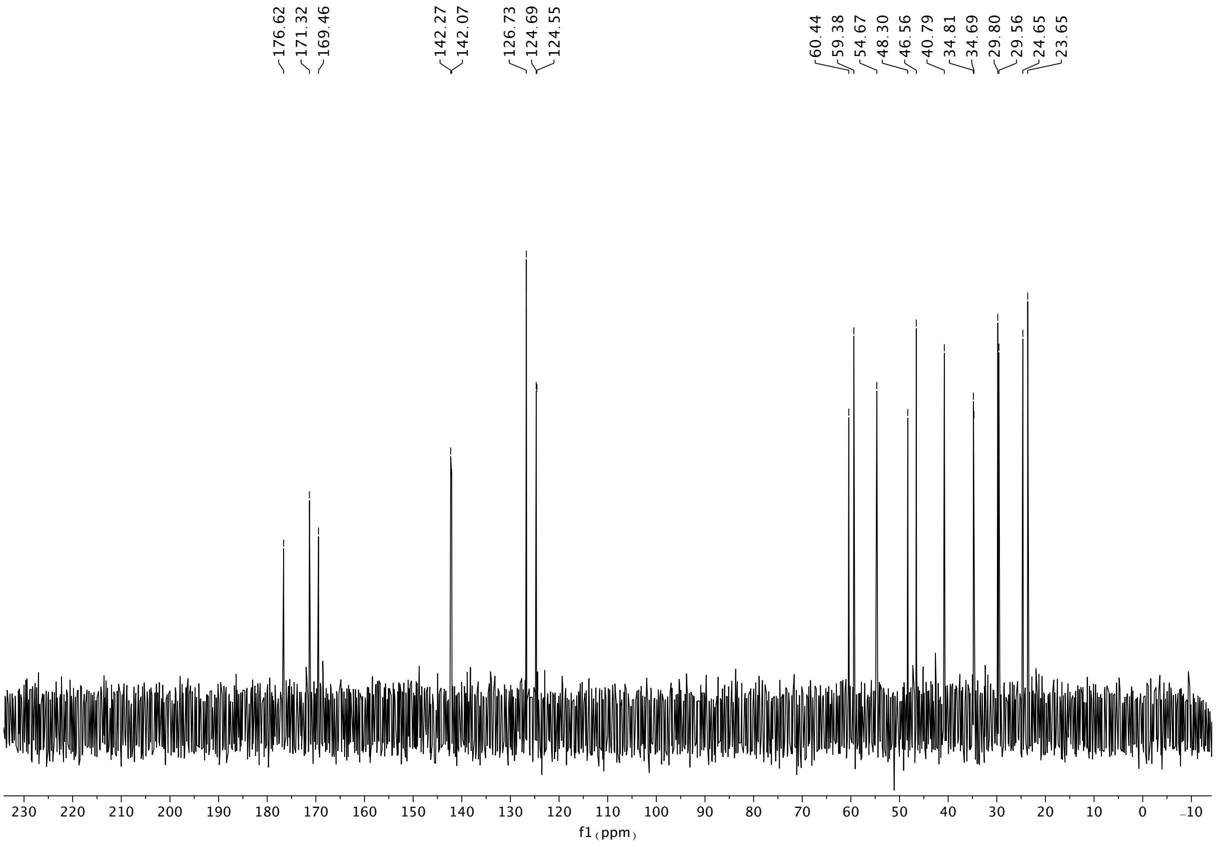

^{1}H and ^{13}C-NMR of H-D-Pro-D-Pro-L-Flp-NH2 · TFA (UTS-13)

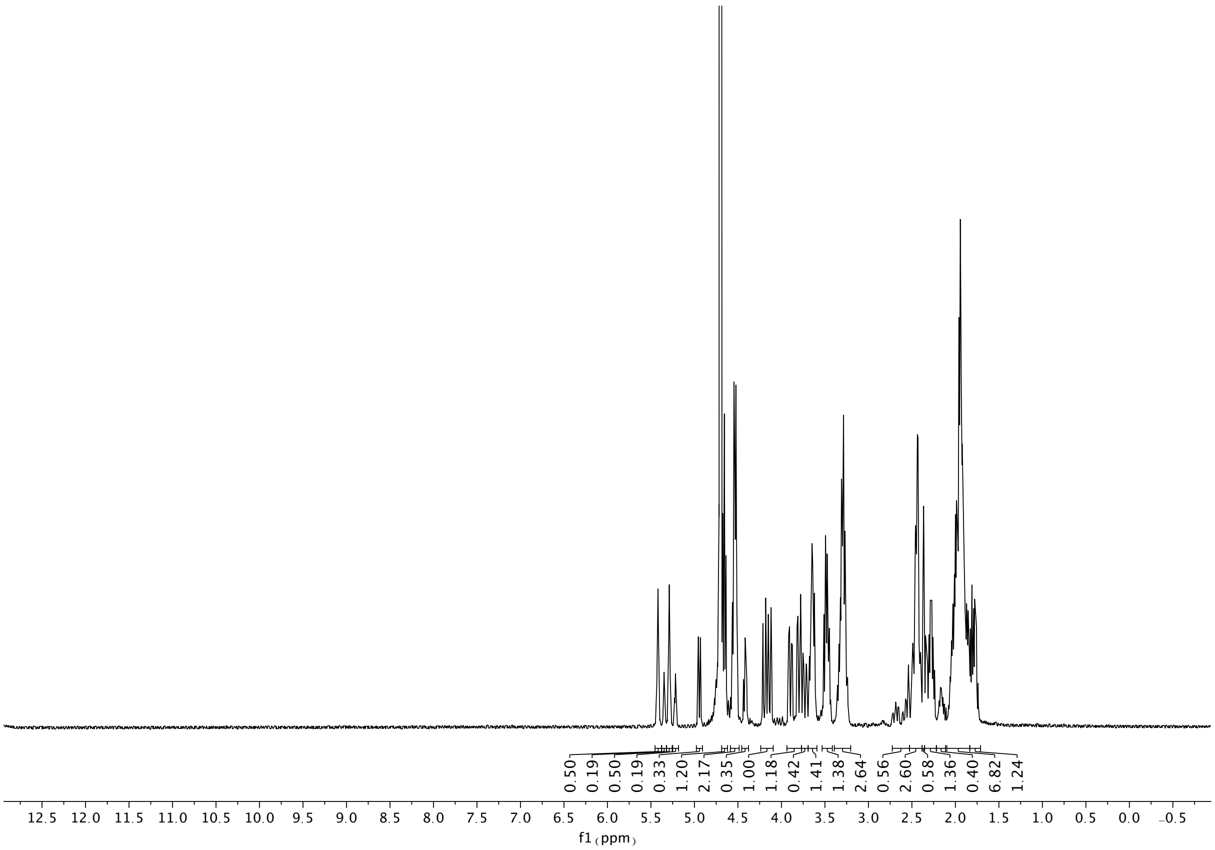

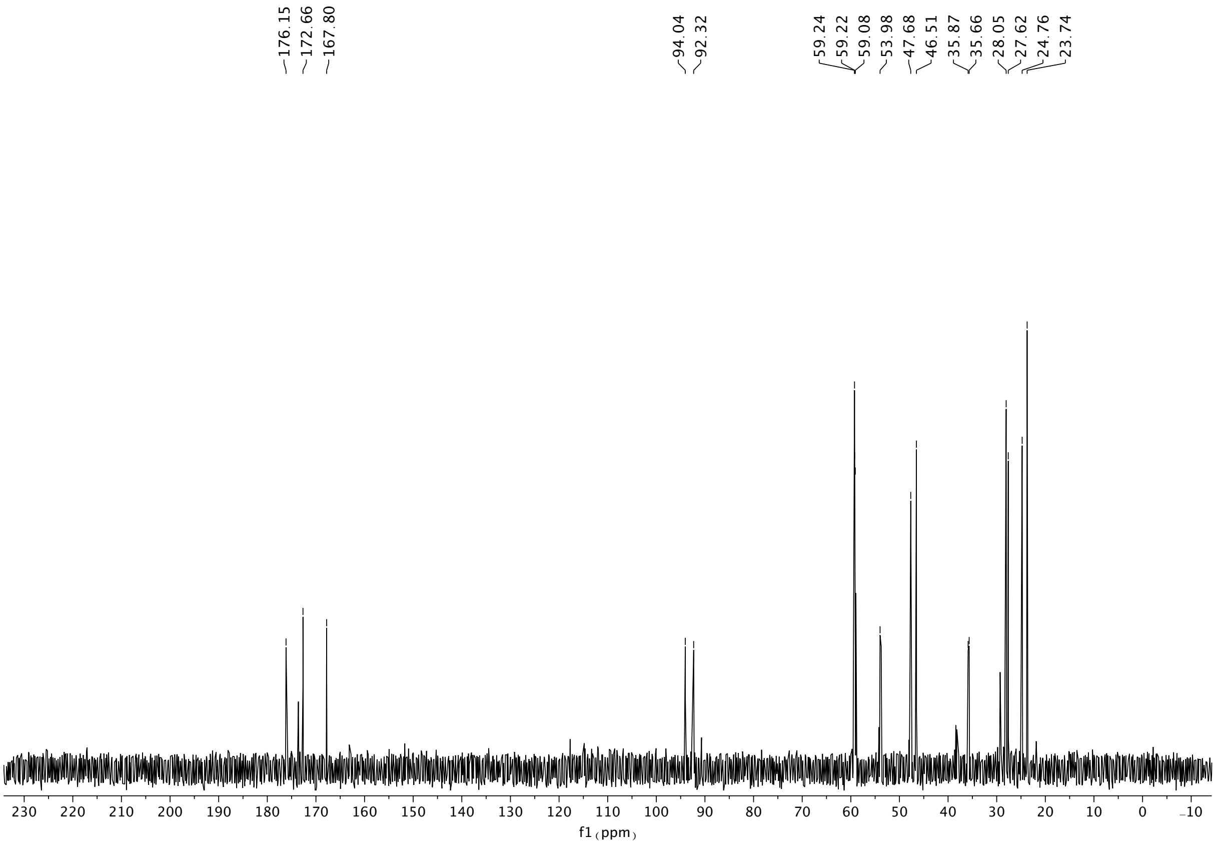

^{1}H and ^{13}C-NMR of H-D-Pro-D-Pro-L-Leu-NH_2 · TFA (UTS-14):

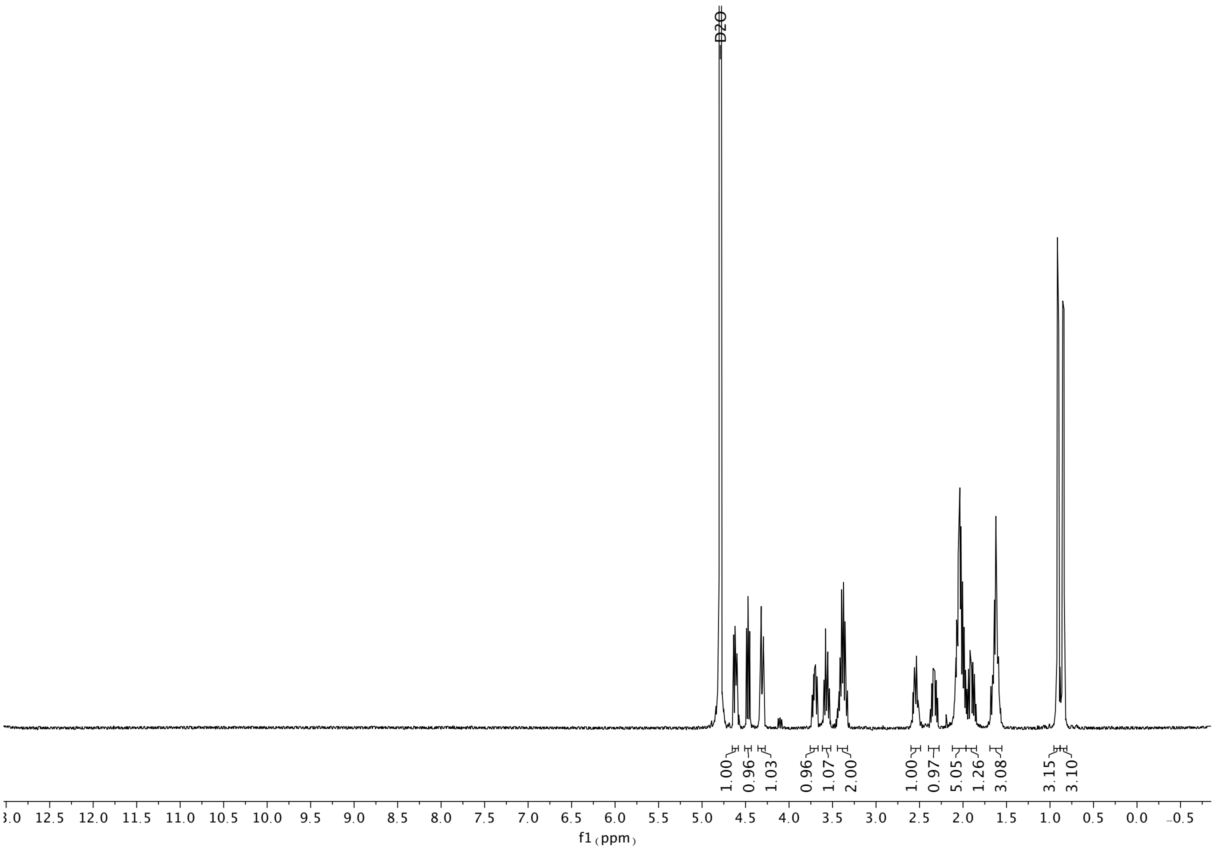

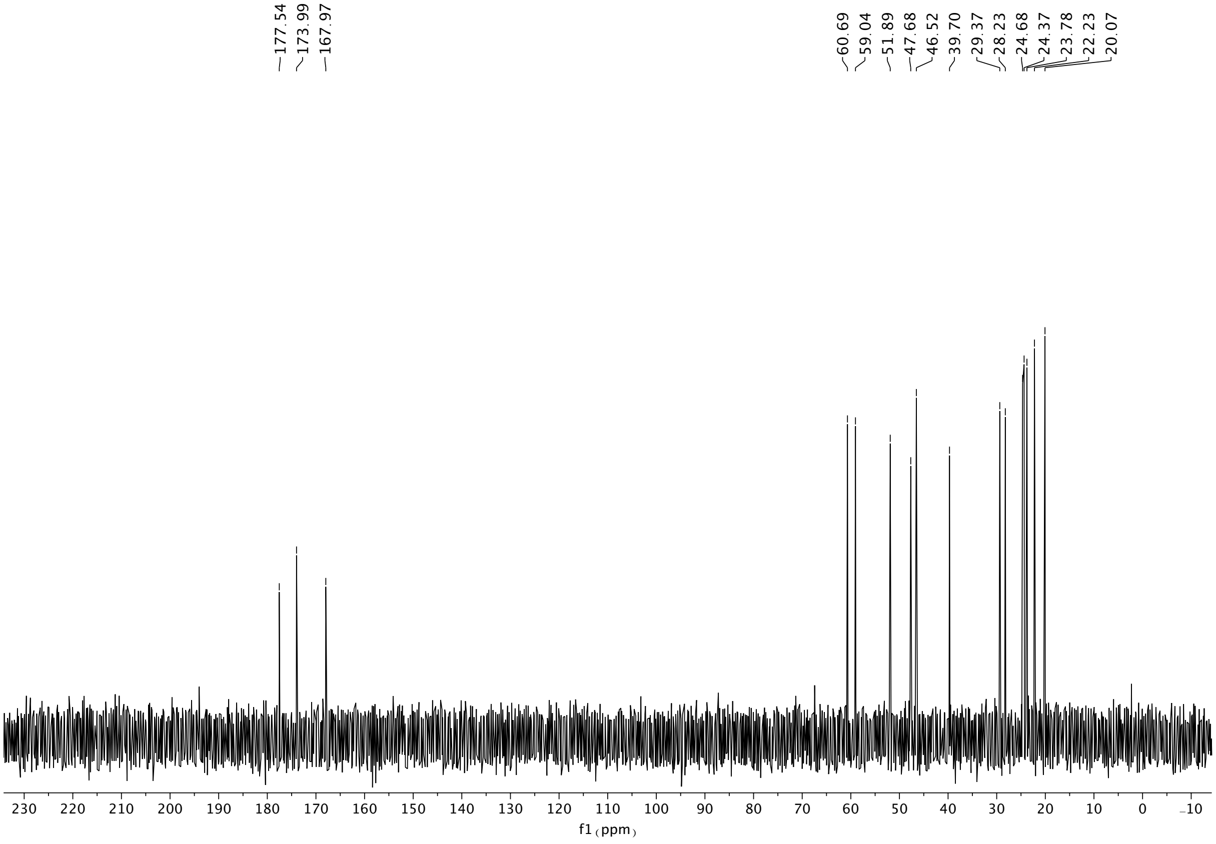

^{1}H and ^{13}C-NMR of H-D-Pro-D-Pro-D-Leu-NH_2 · TFA (UTS-15)

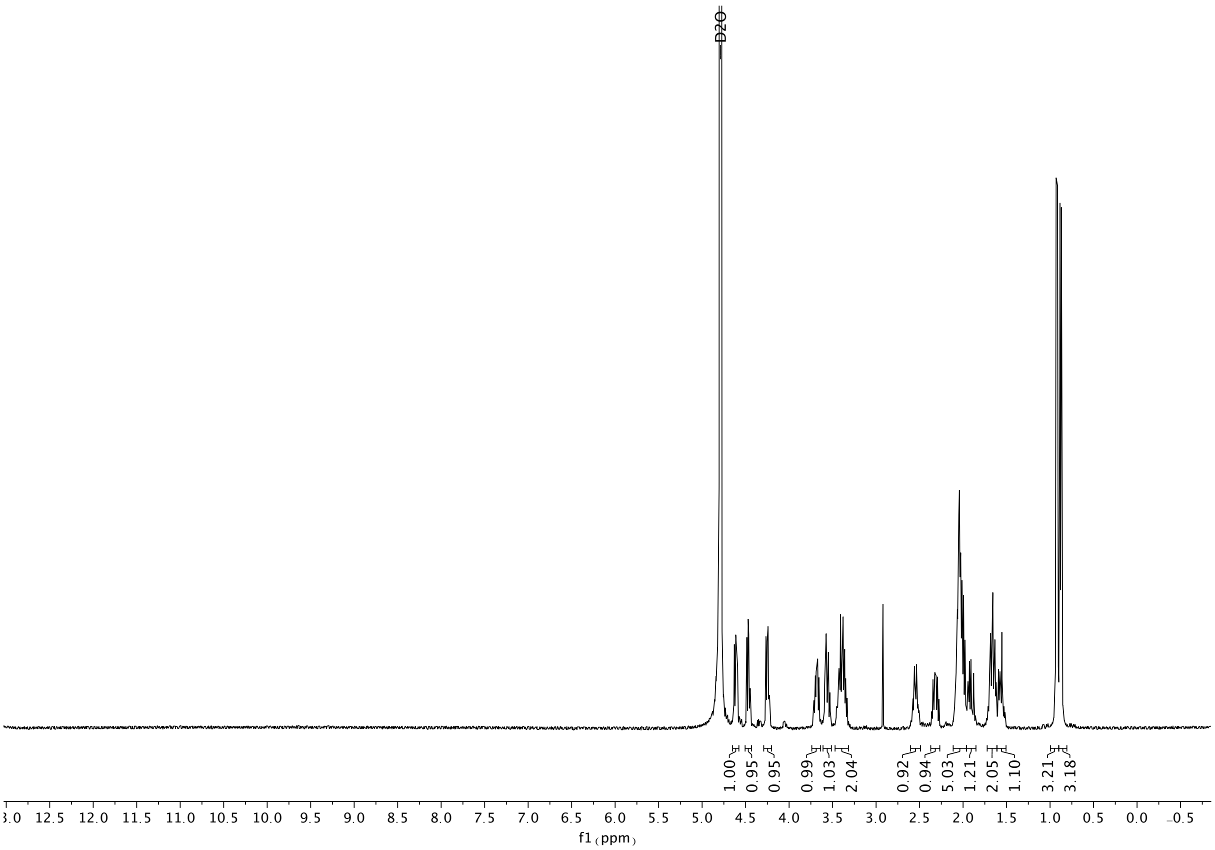

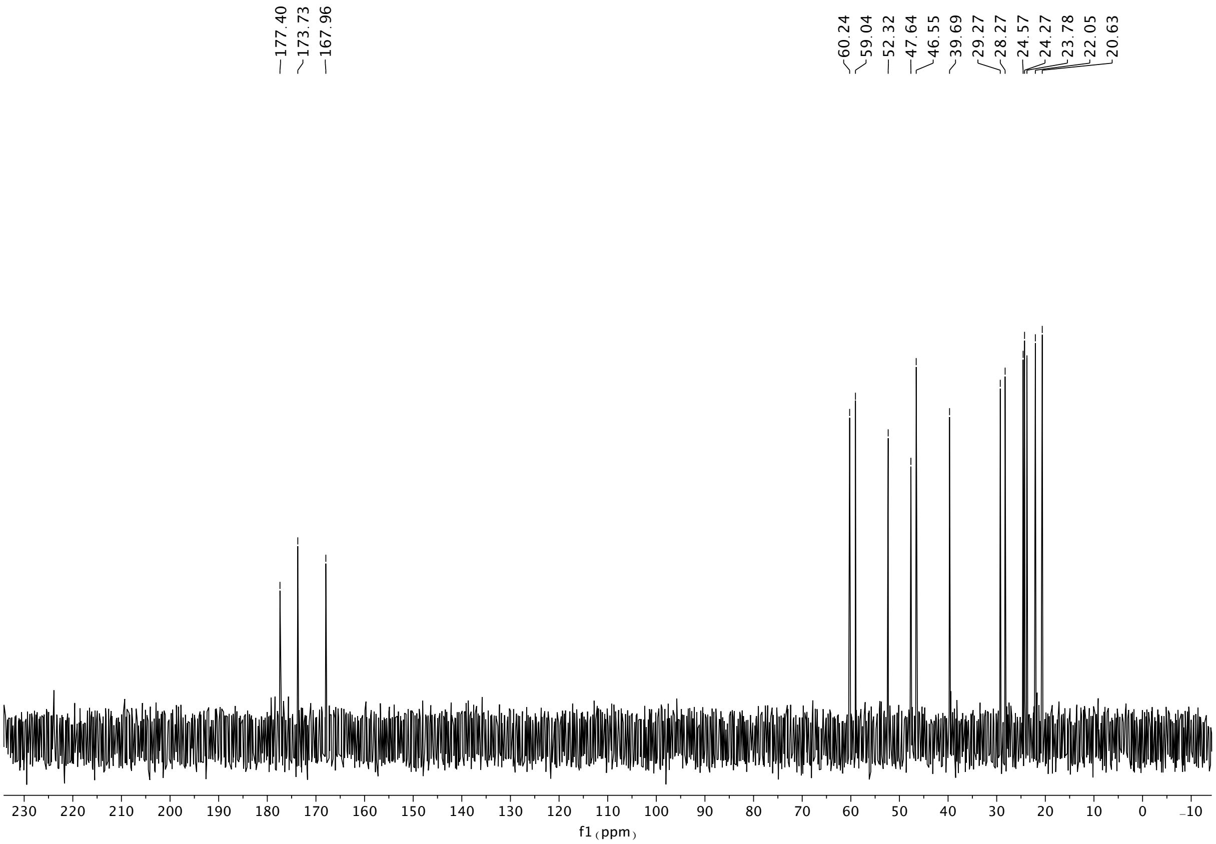

^{1}H and ^{13}C-NMR of H-D-Pro-D-Pro-L-Gln-NH₂ · TFA (UTS-16)

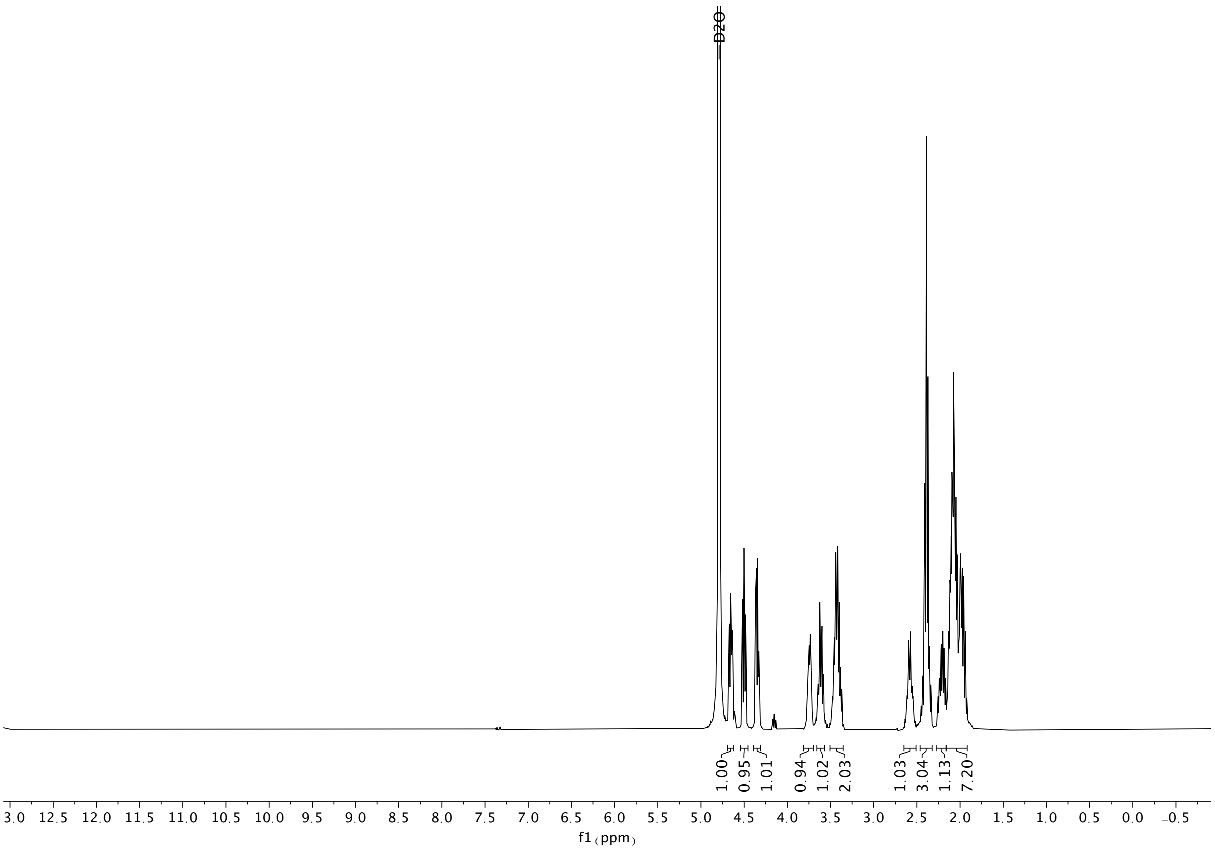

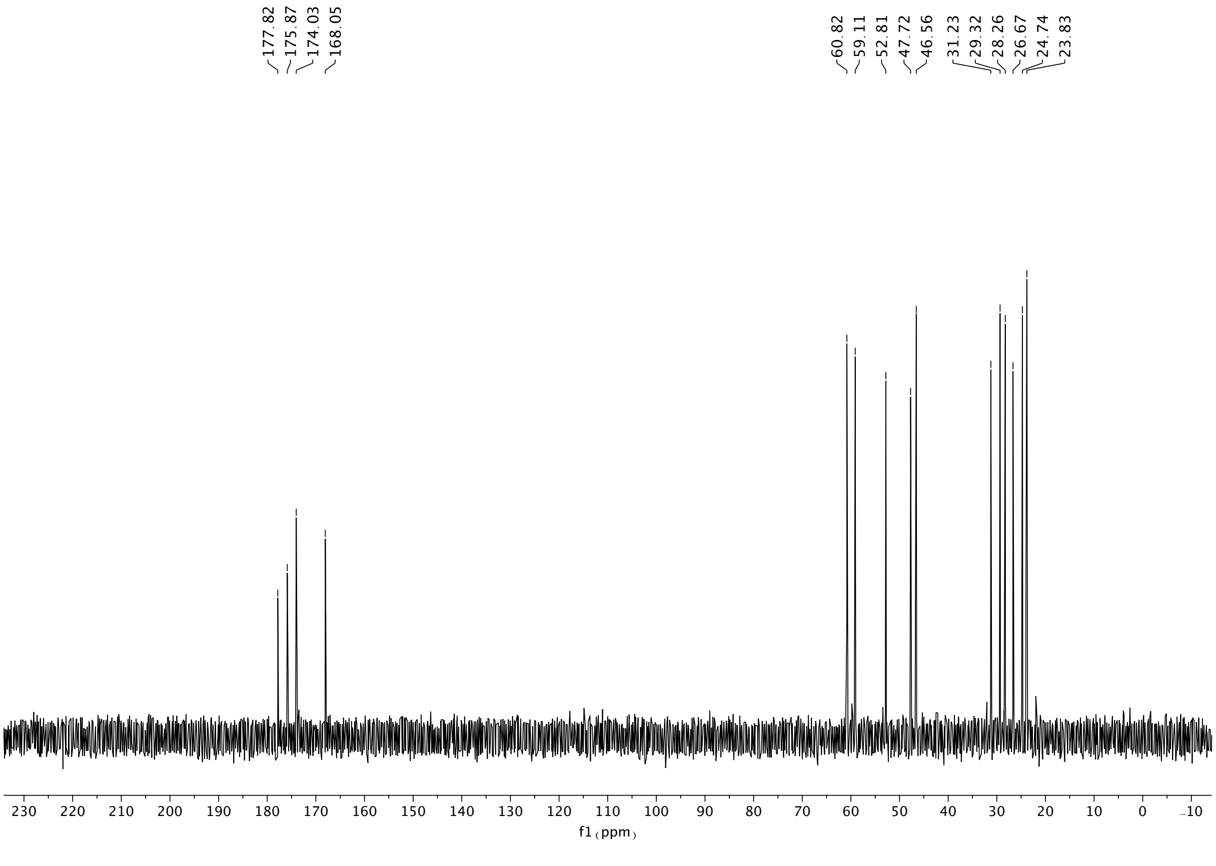

^{1}H and ^{13}C-NMR of H-D-Pro-D-Pro-D-Gln-NH$_2$ · TFA (UTS-17)

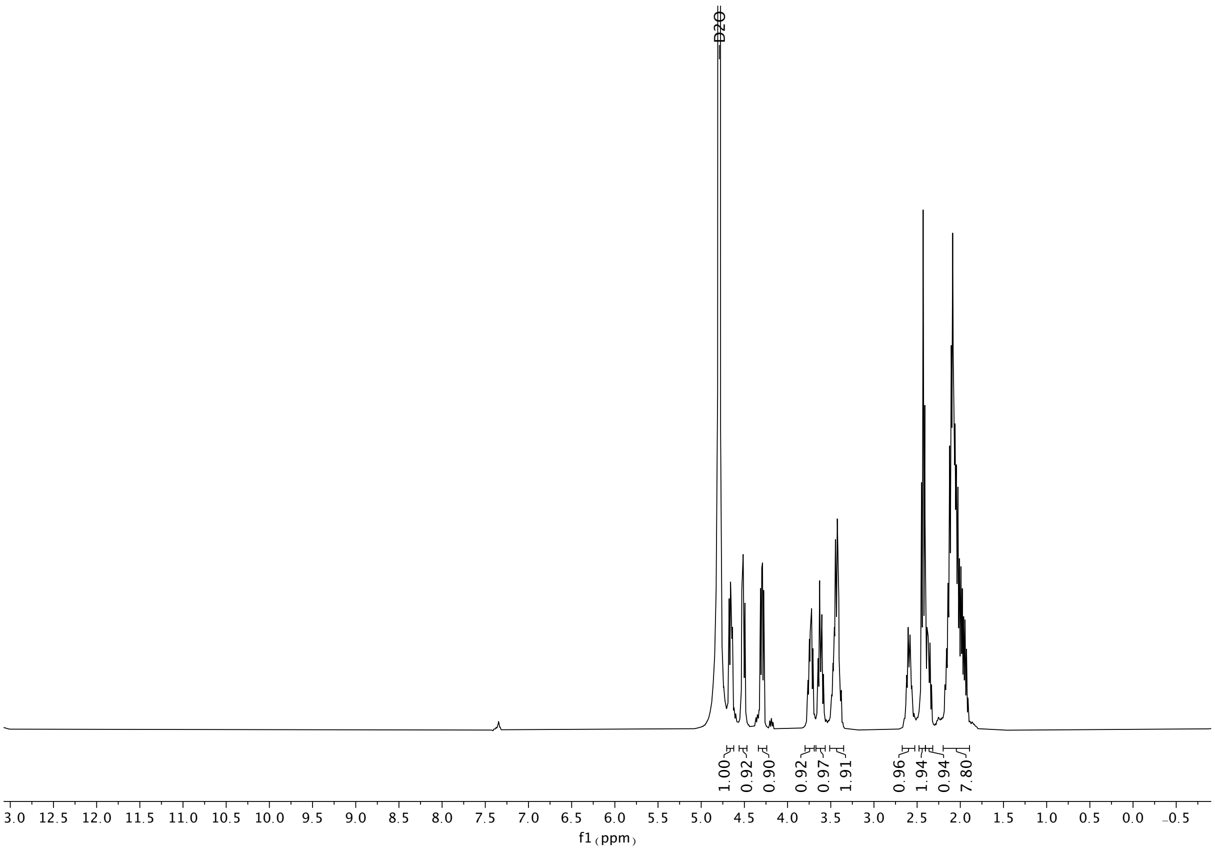

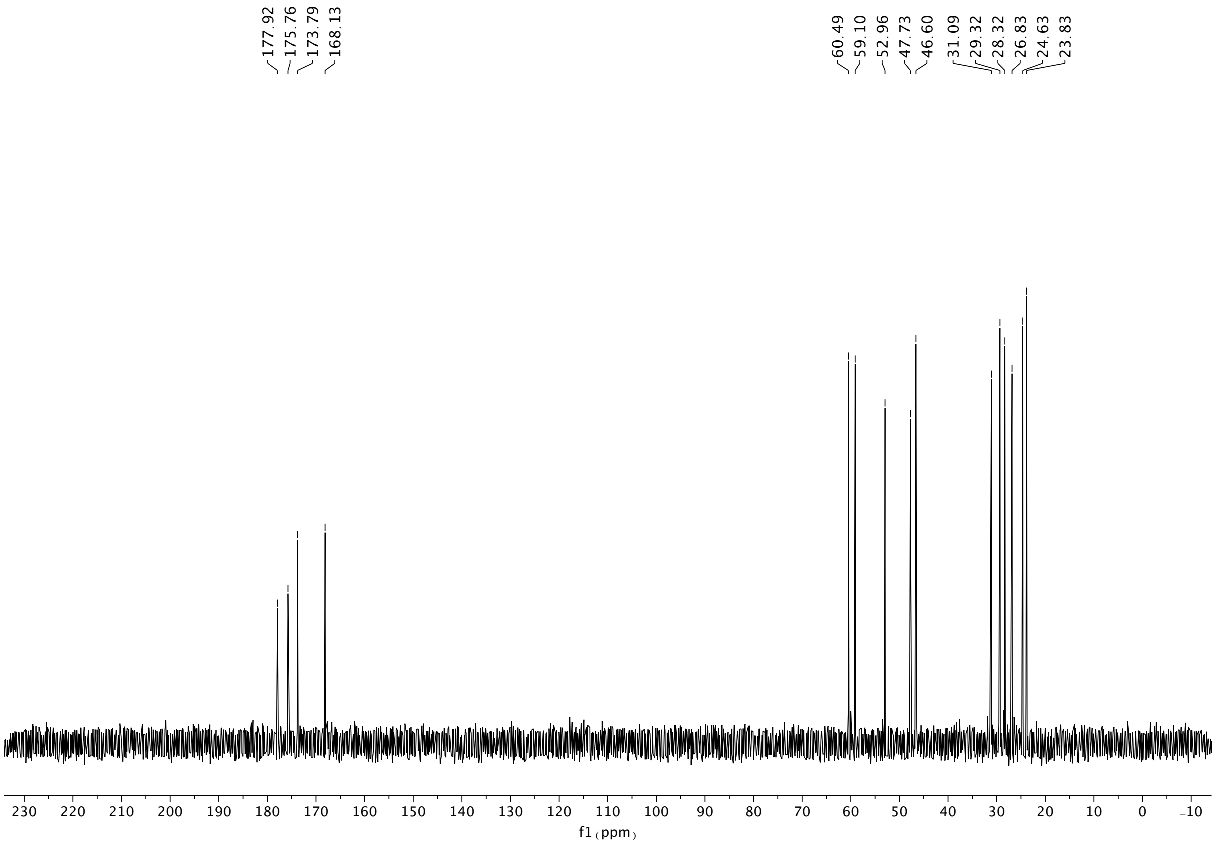

^{1}H and ^{13}C-NMR of H-D-Pro-D-Pro-L-Glu-NH$_2$ · TFA (UTS-18)

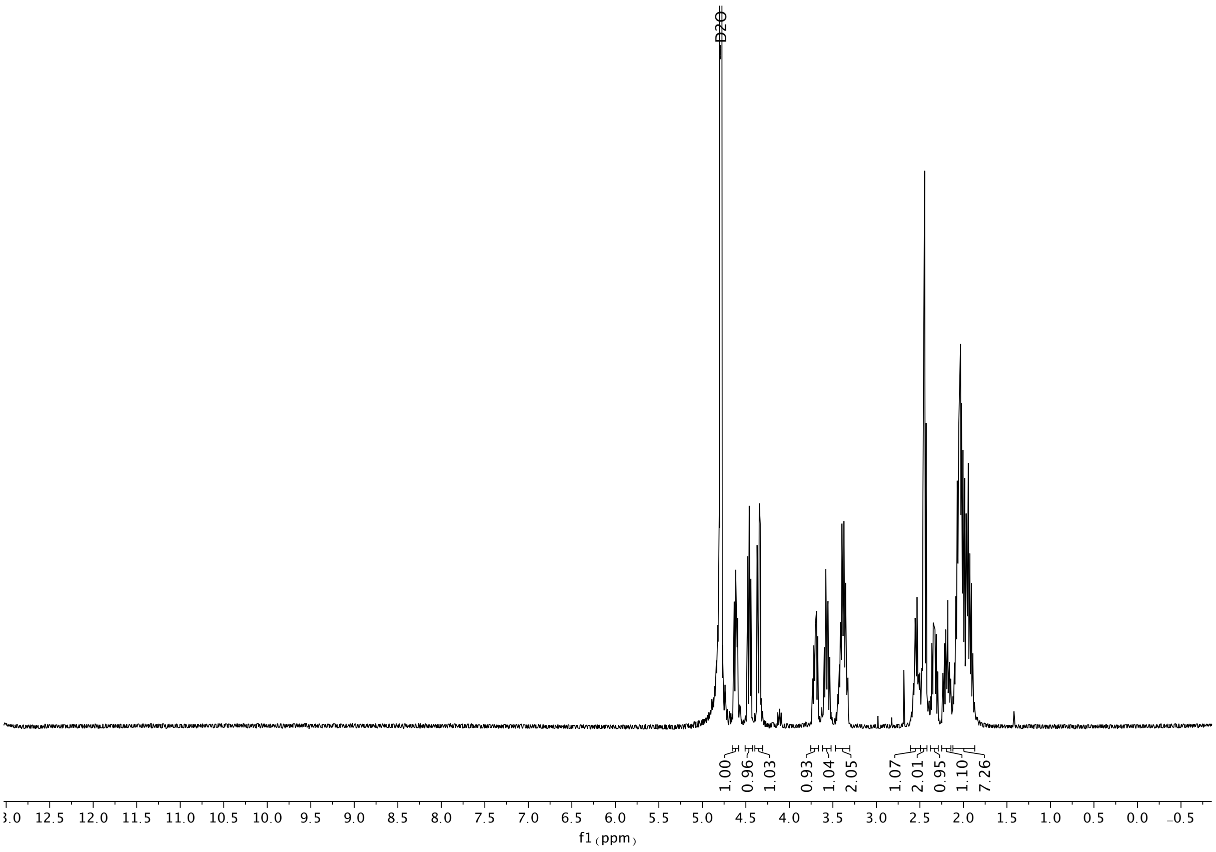

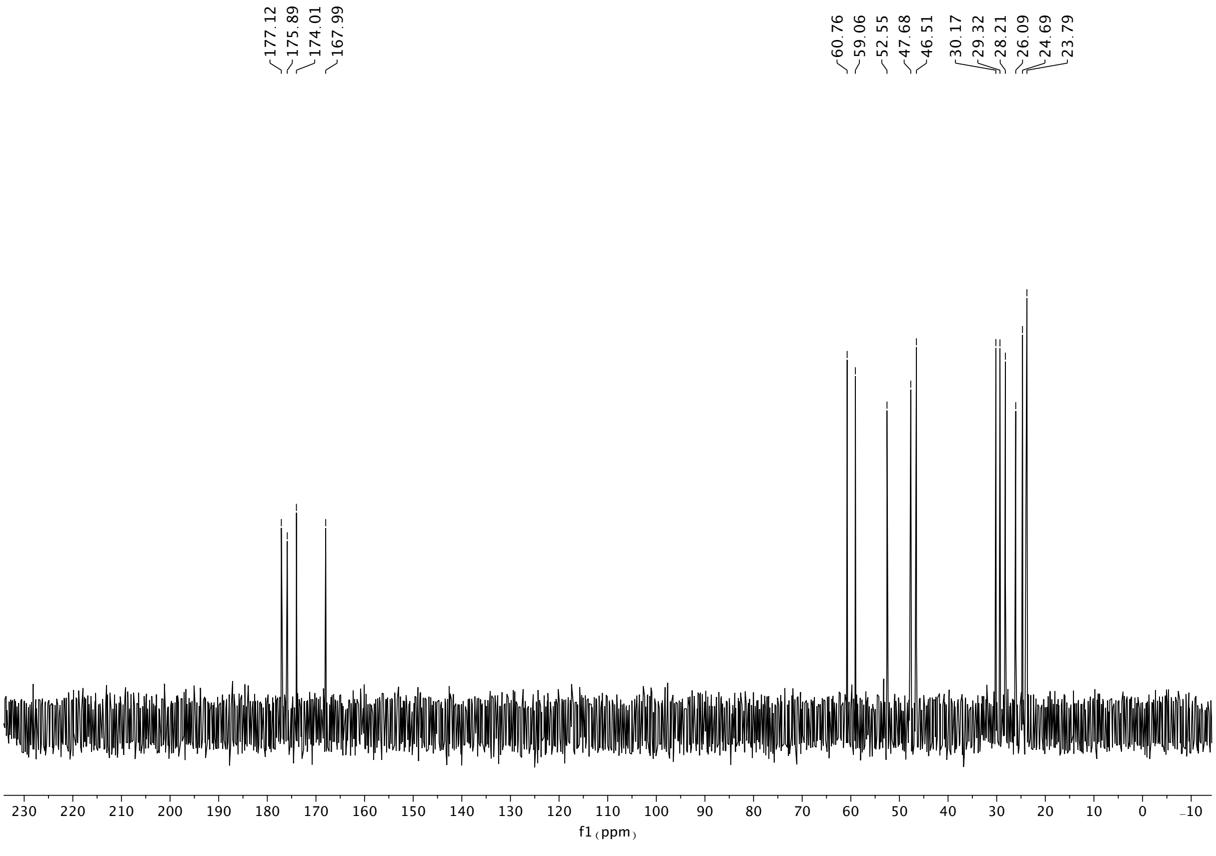

^{1}H and ^{13}C-NMR of H-D-Pro-D-Pro-D-Glu-NH$_2$ · TFA (UTS-19):

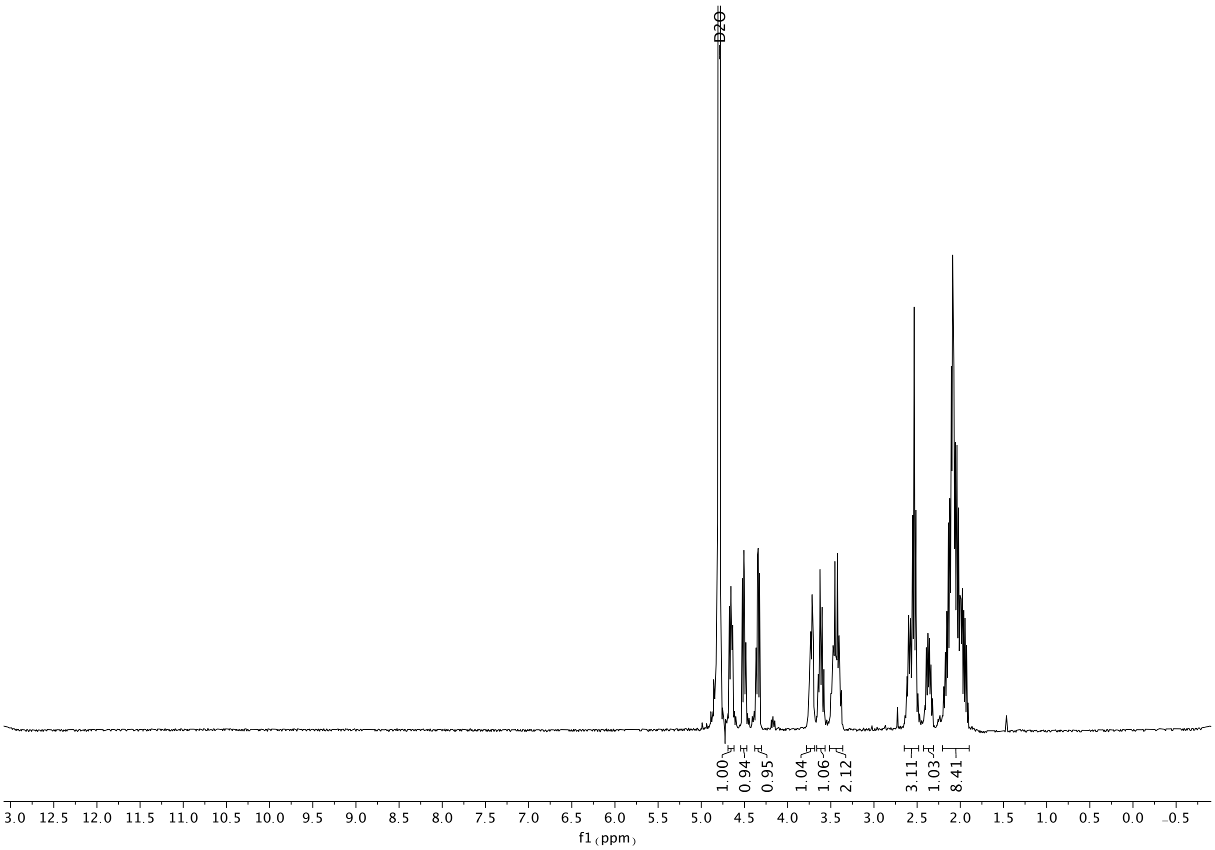

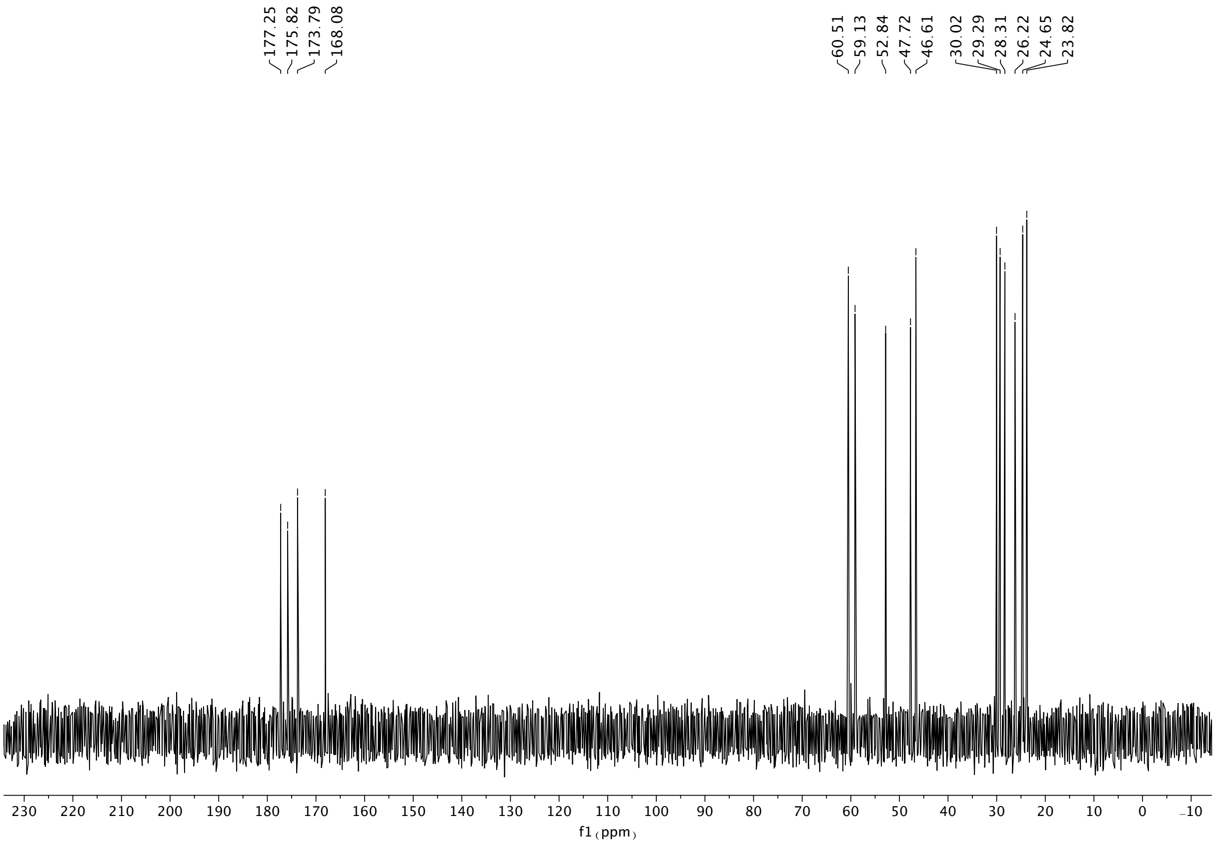

^{1}H and ^{13}C-NMR of H-D-Pro-D-Pro-L-Tyr-NH$_2$ · TFA (UTS-20):

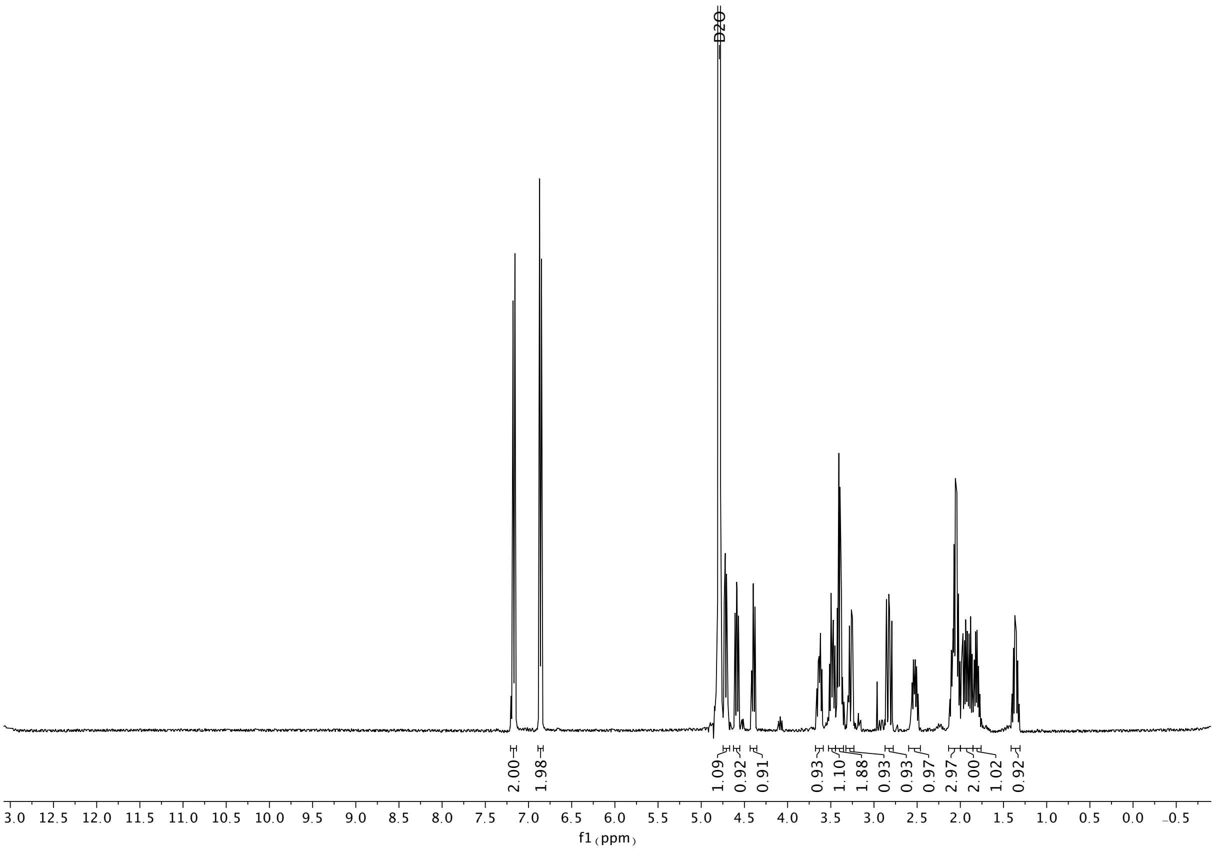

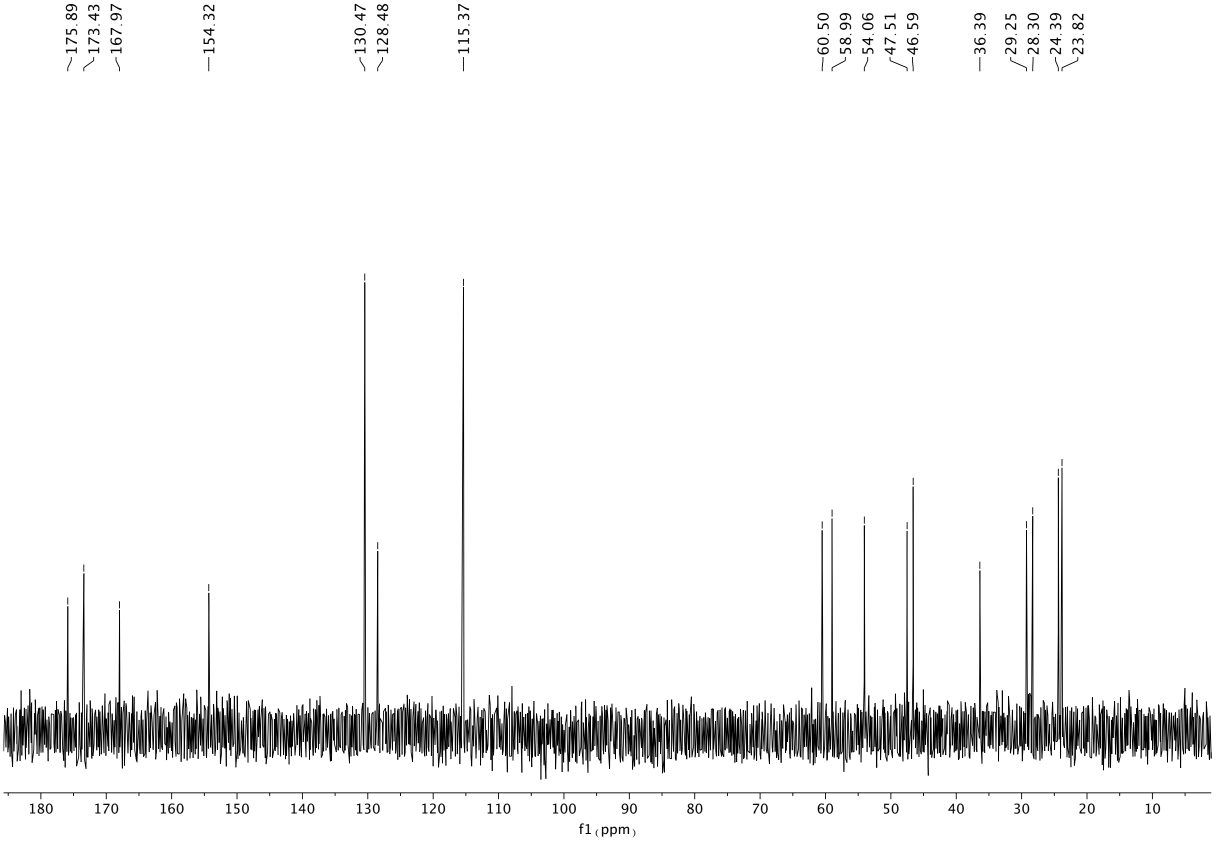

^{1}H and ^{13}C-NMR of H-D-Pro-D-Pro-D-Tyr-NH$_2$ · TFA (UTS-21):

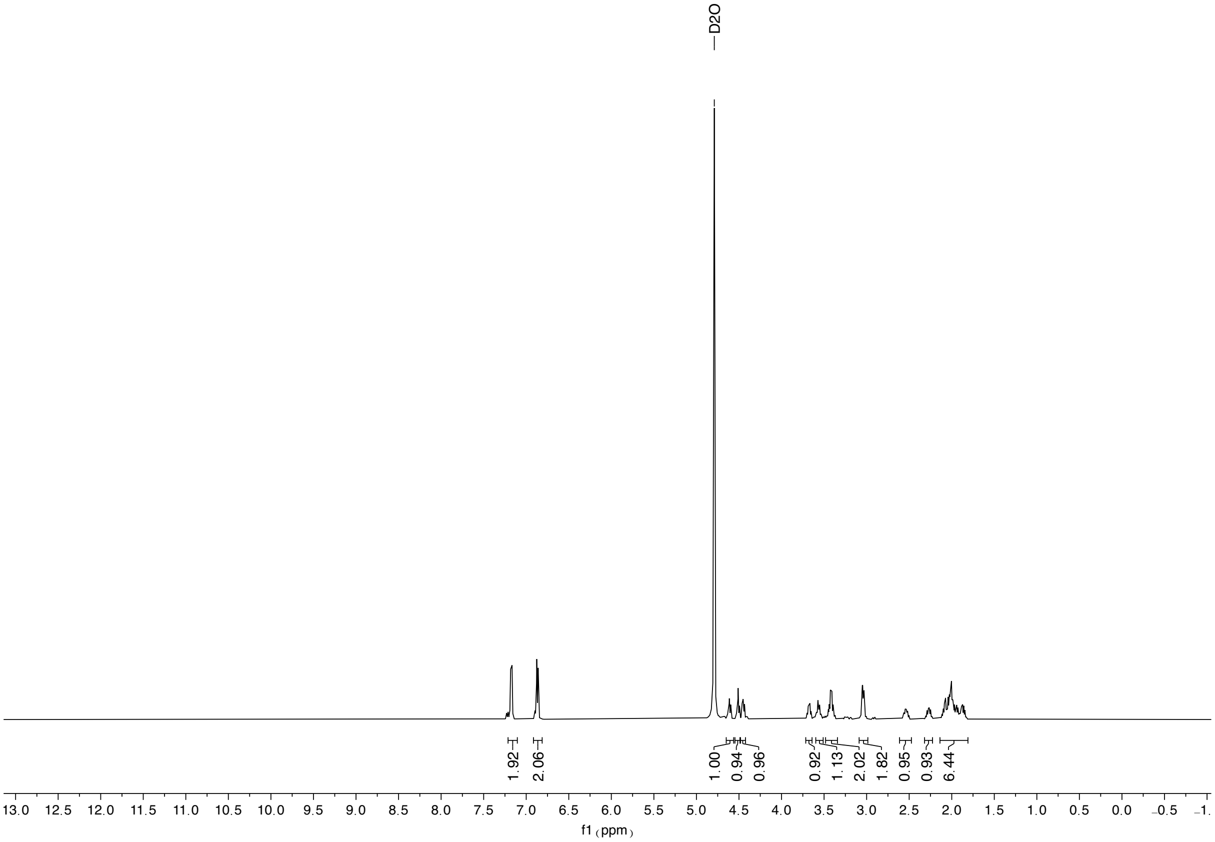

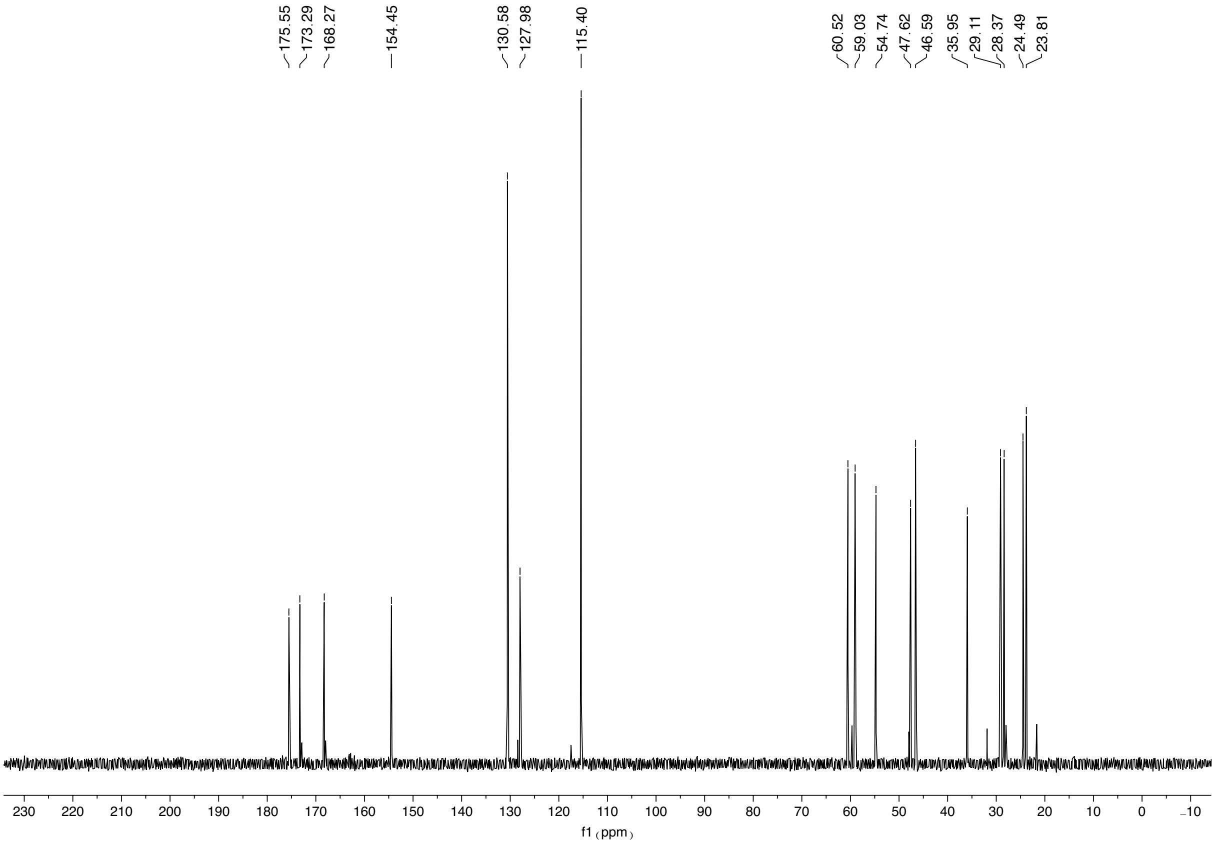

^{1}H and ^{13}C-NMR of H-D-Pro-D-Pro-CyLeu-NH₂ · TFA (UTS-22):

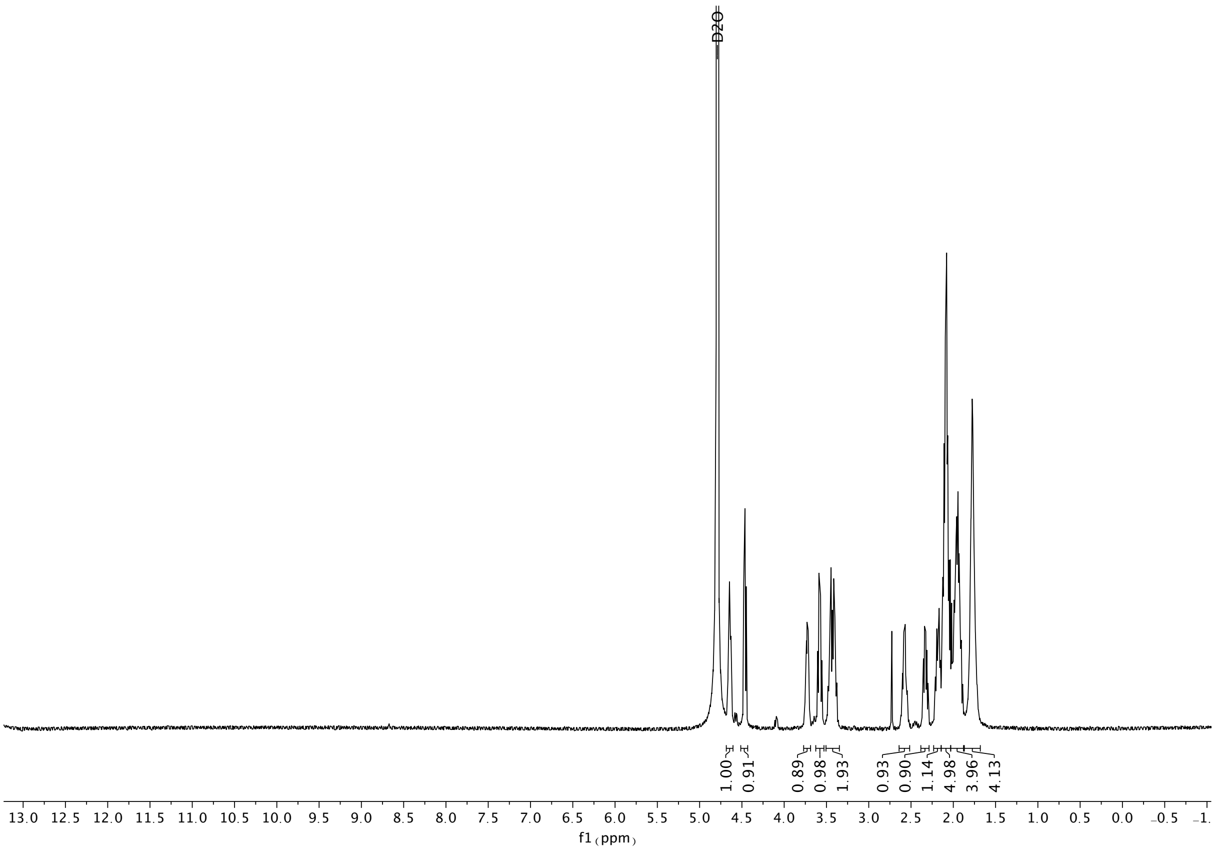

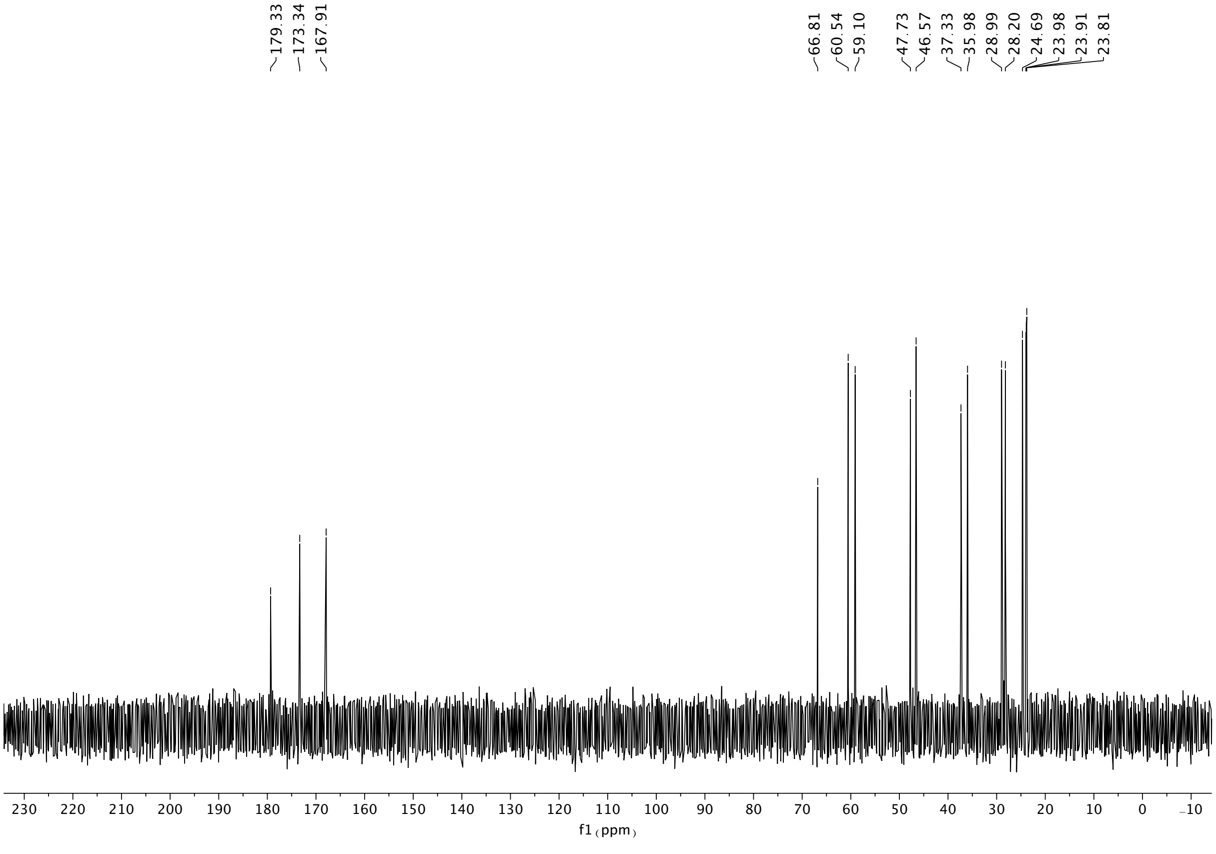

^{1}H and ^{13}C-NMR of H-D-Pro-D-Pro-Abz-NH₂ · TFA (UTS-23):

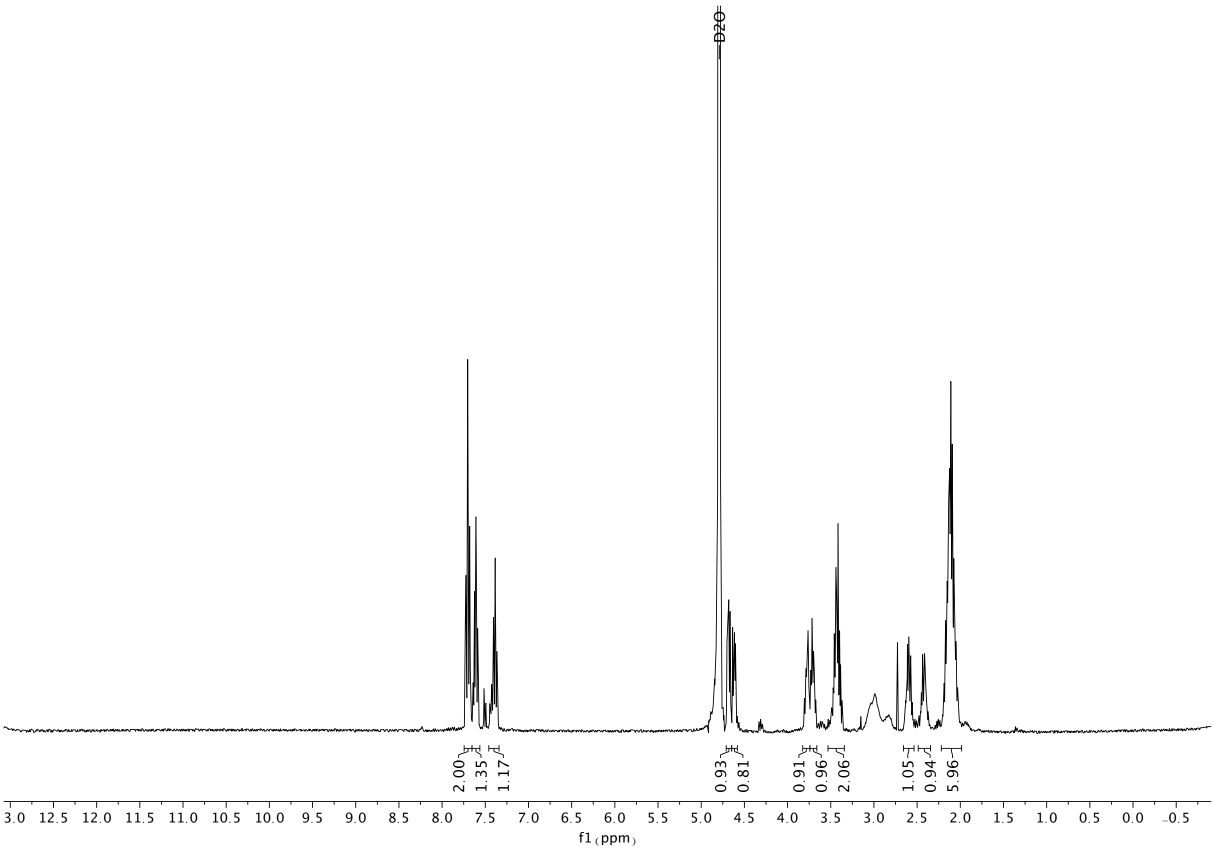

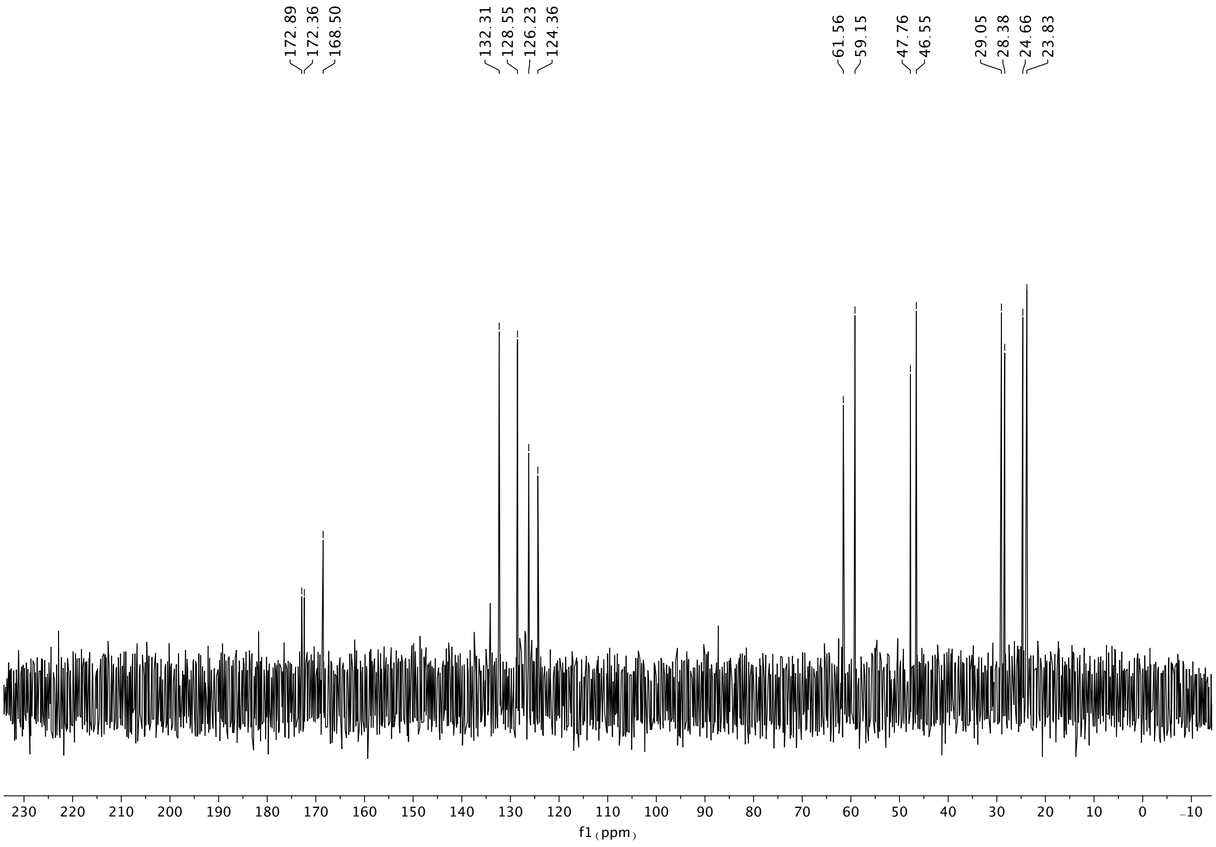

^{1}H and ^{13}C-NMR of H-D-Pro-D-Pro-D-Ind-NH_2 · TFA (UTS-24):

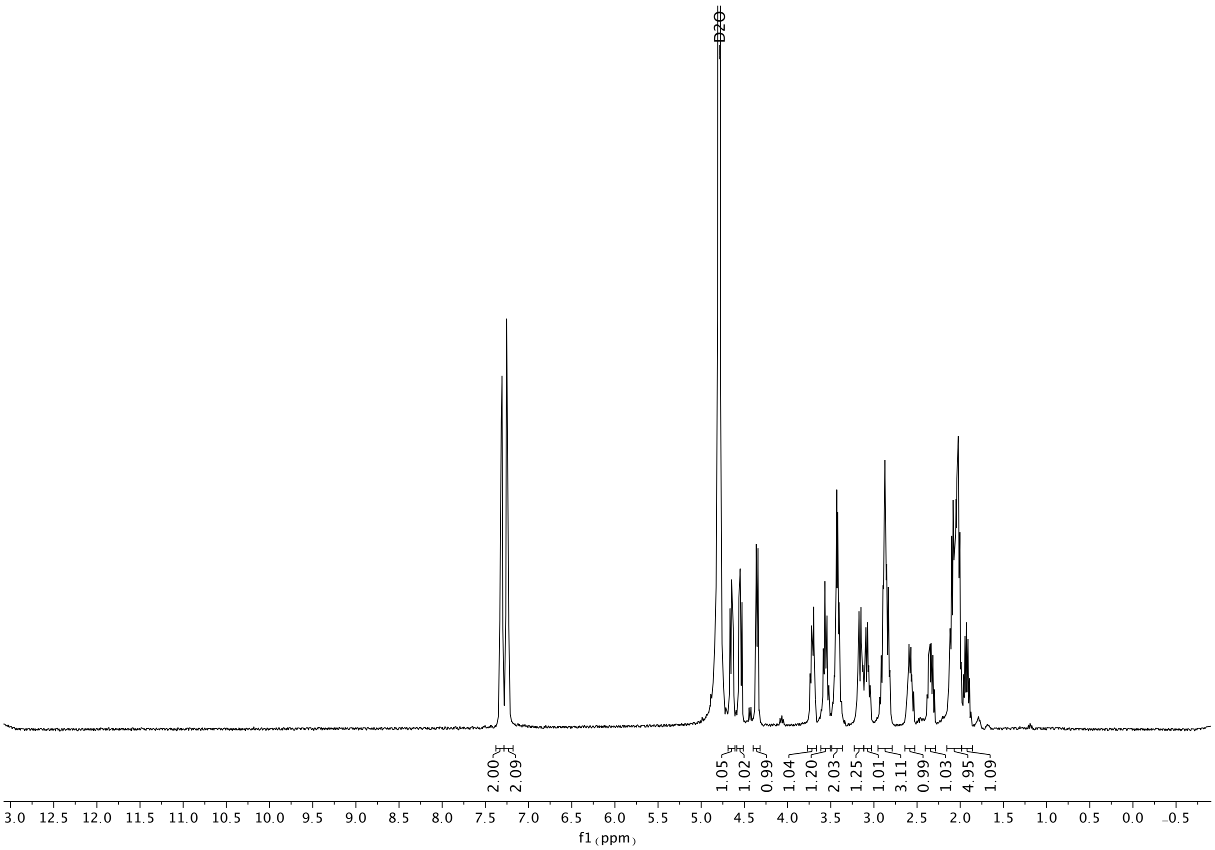

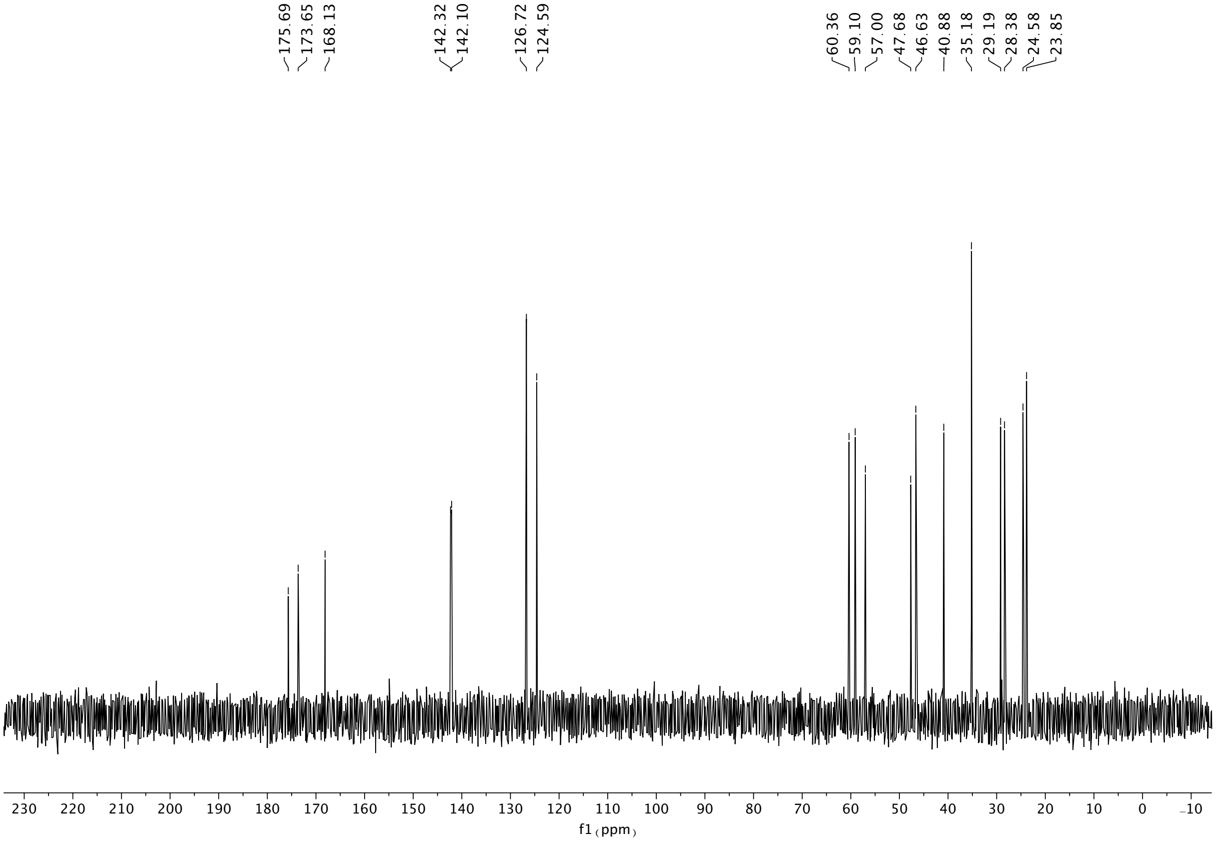

^{1}H and ^{13}C-NMR of H-D-Pro-L-(4*S*)-Flp-L-Flp-NH₂ · TFA (UTS-25):

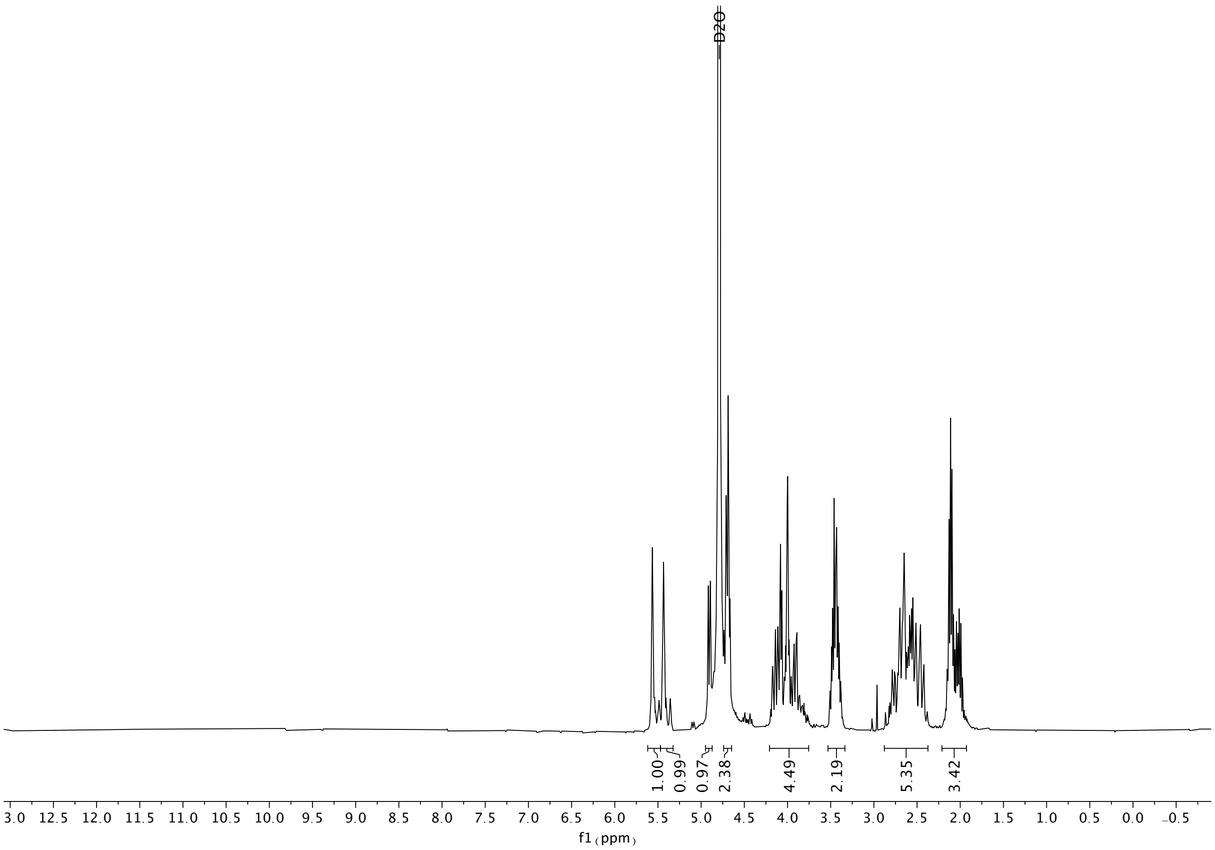

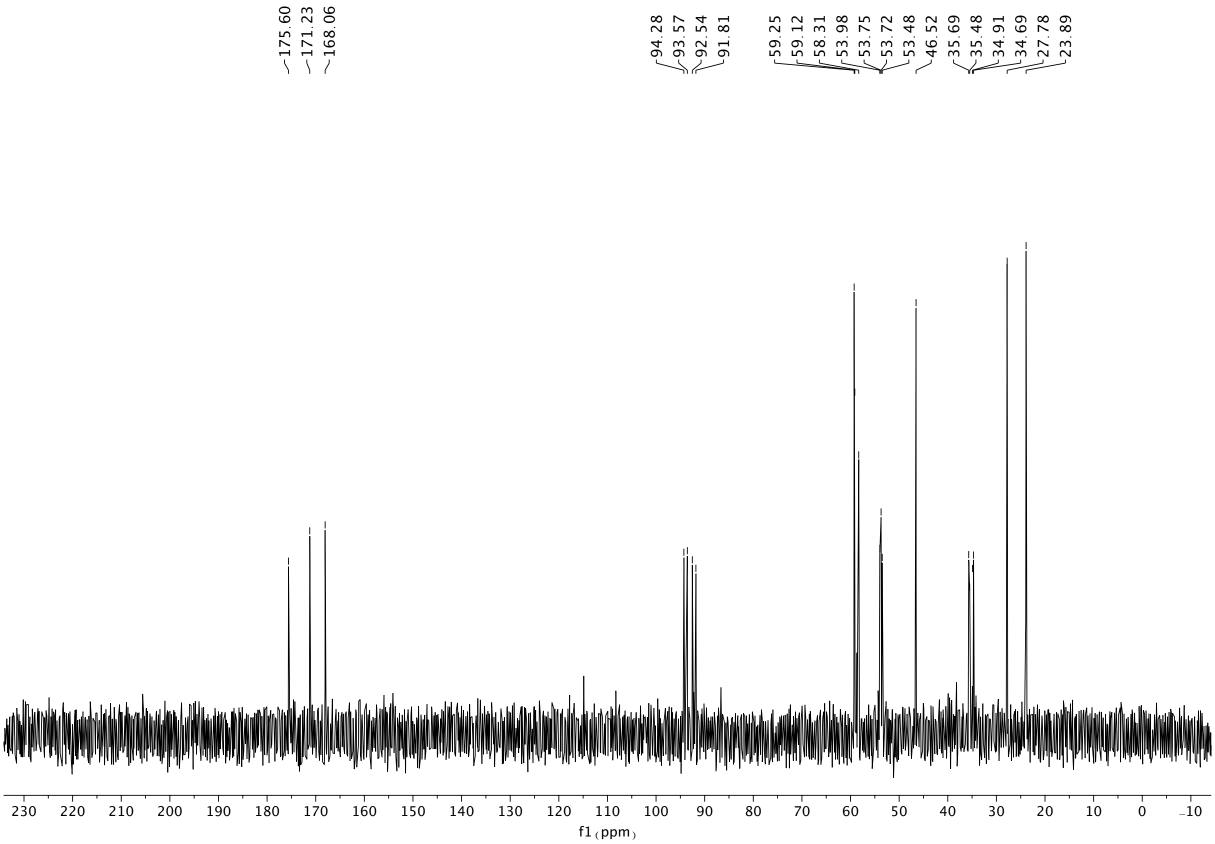

^{1}H and ^{13}C-NMR of H-D-Pro-L-(4*S*)-Flp-L-Leu-NH_2 · TFA (UTS-26):

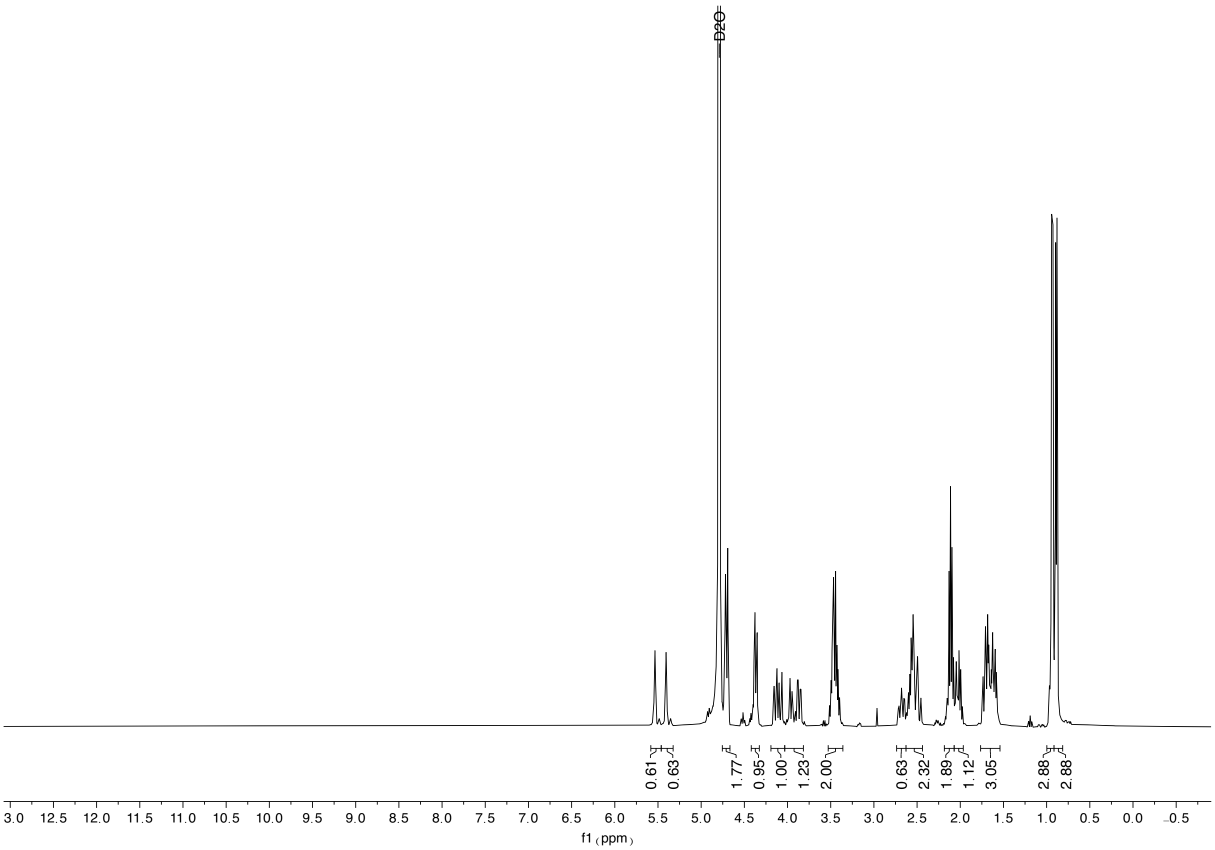

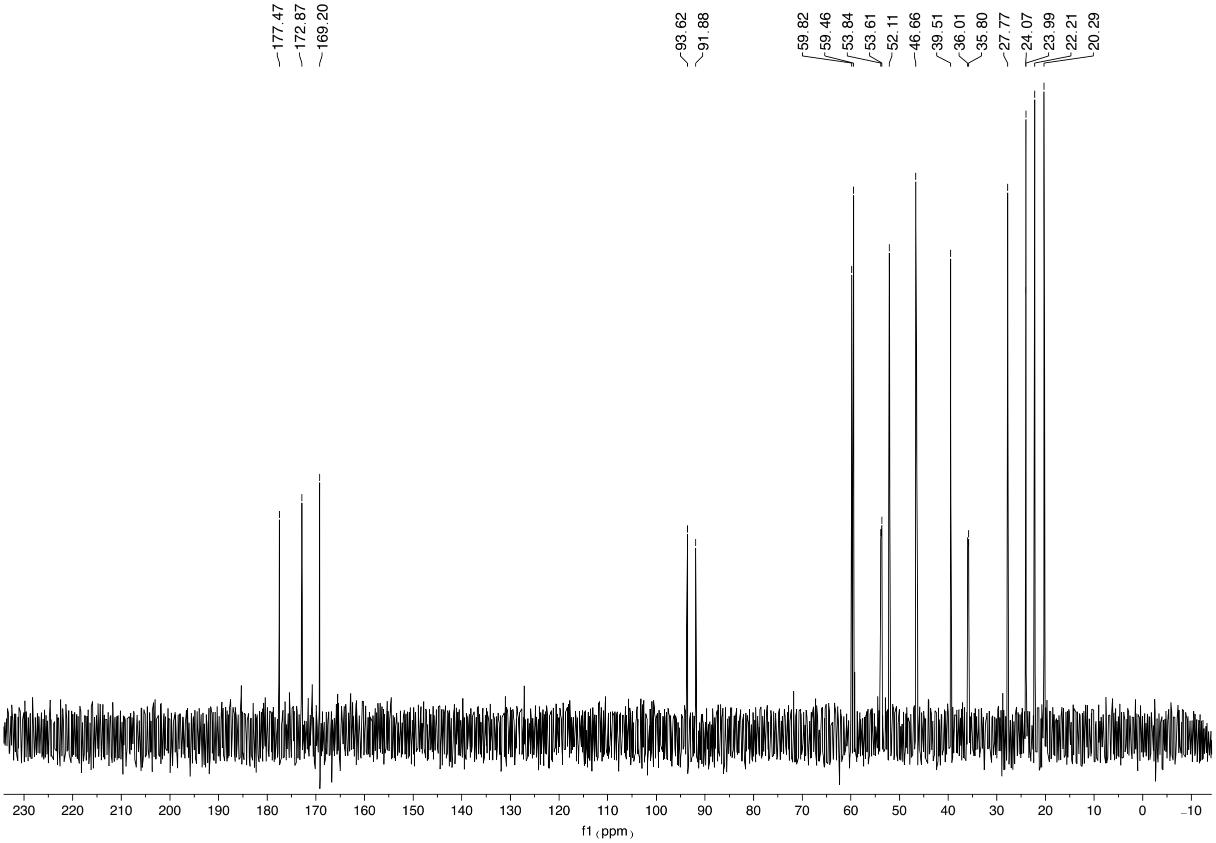

^{1}H and ^{13}C-NMR of H-D-Pro-L-(4*S*)-Flp-D-Leu-NH₂ · TFA (UTS-27):

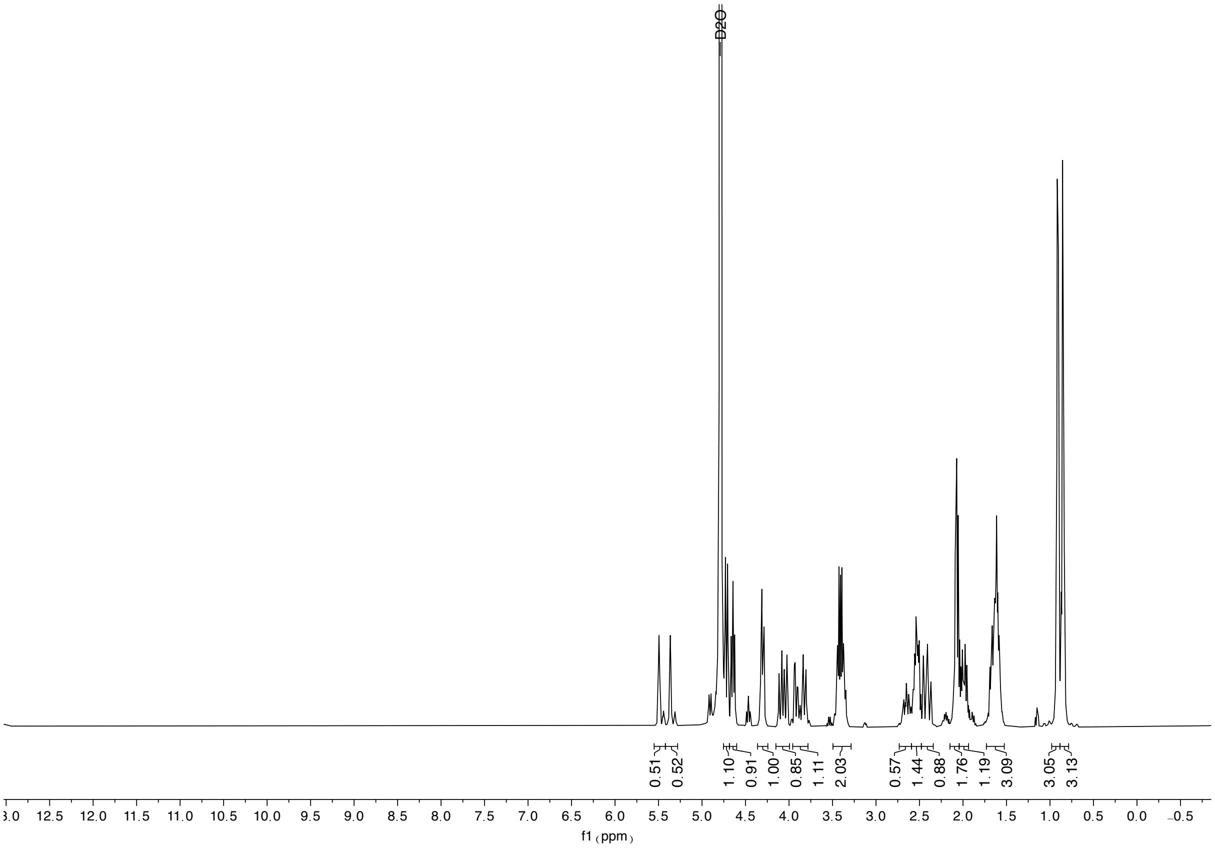

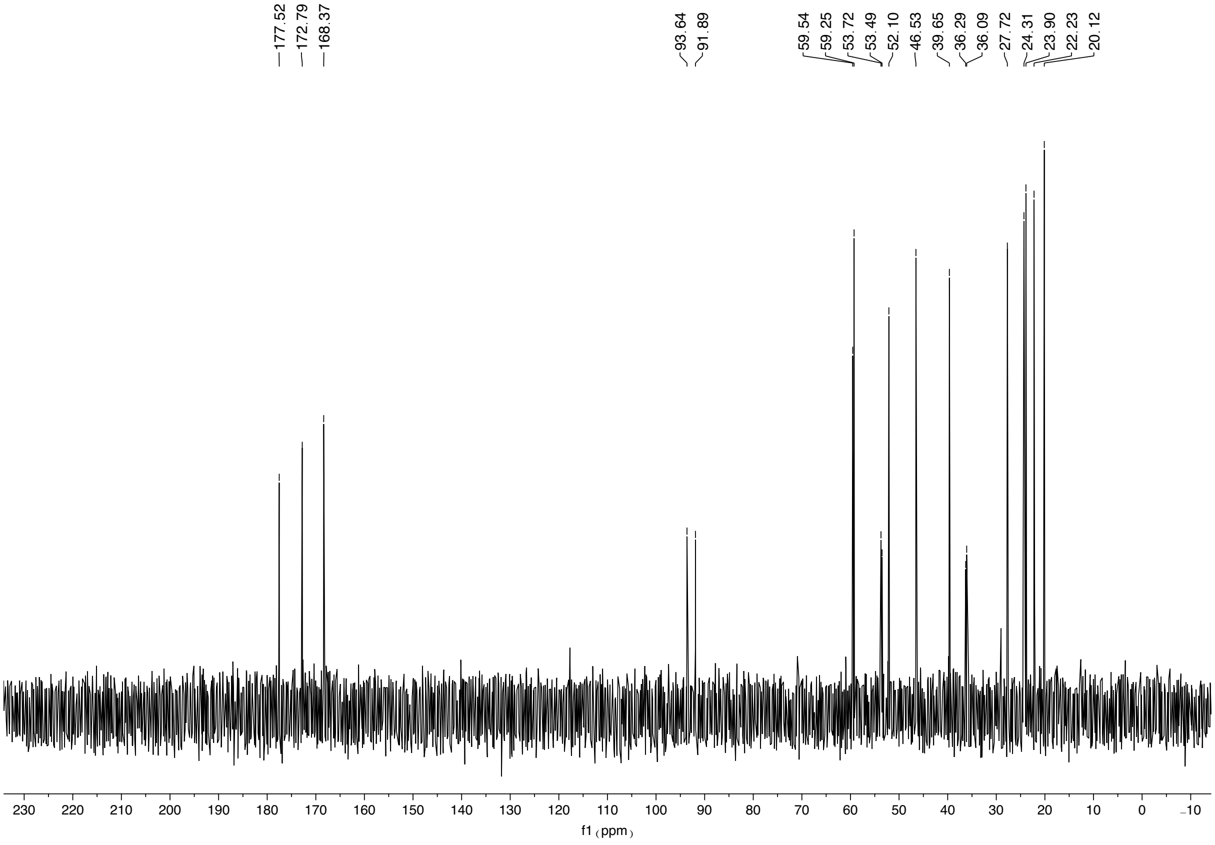

^{1}H and ^{13}C-NMR of H-D-Pro-L-(4*S*)-Flp-L-Gln-NH_2 · TFA (UTS-28):

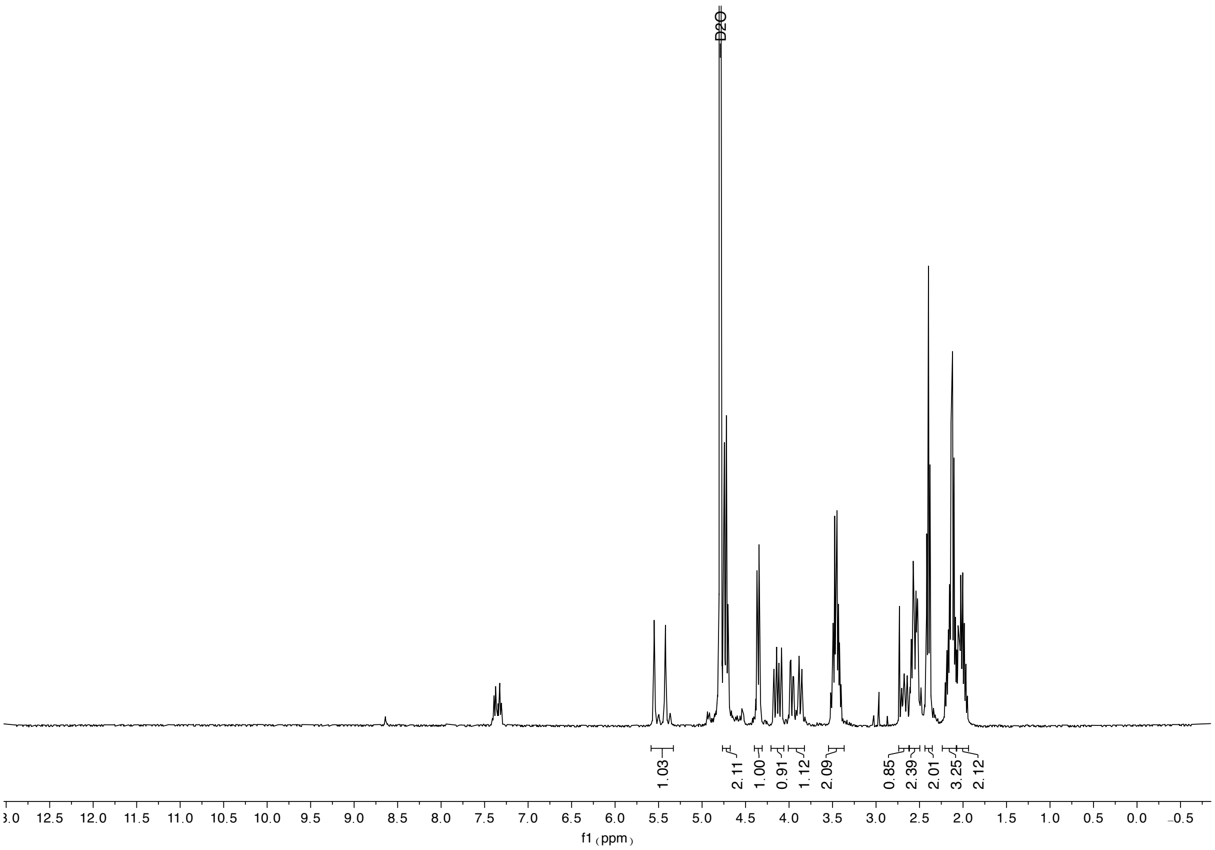

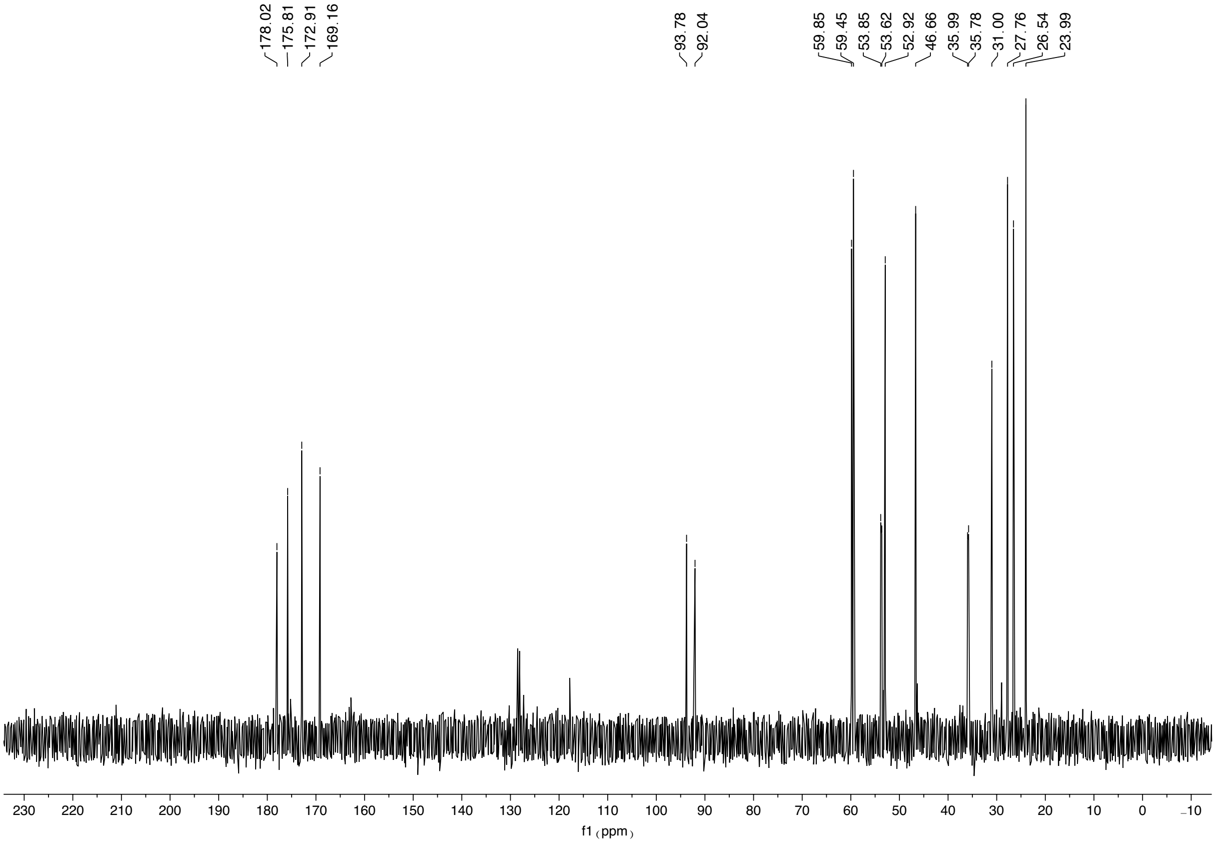

^{1}H and ^{13}C-NMR of H-D-Pro-L-(4*S*)-Flp-D-Gln-NH₂ · TFA (UTS-29):

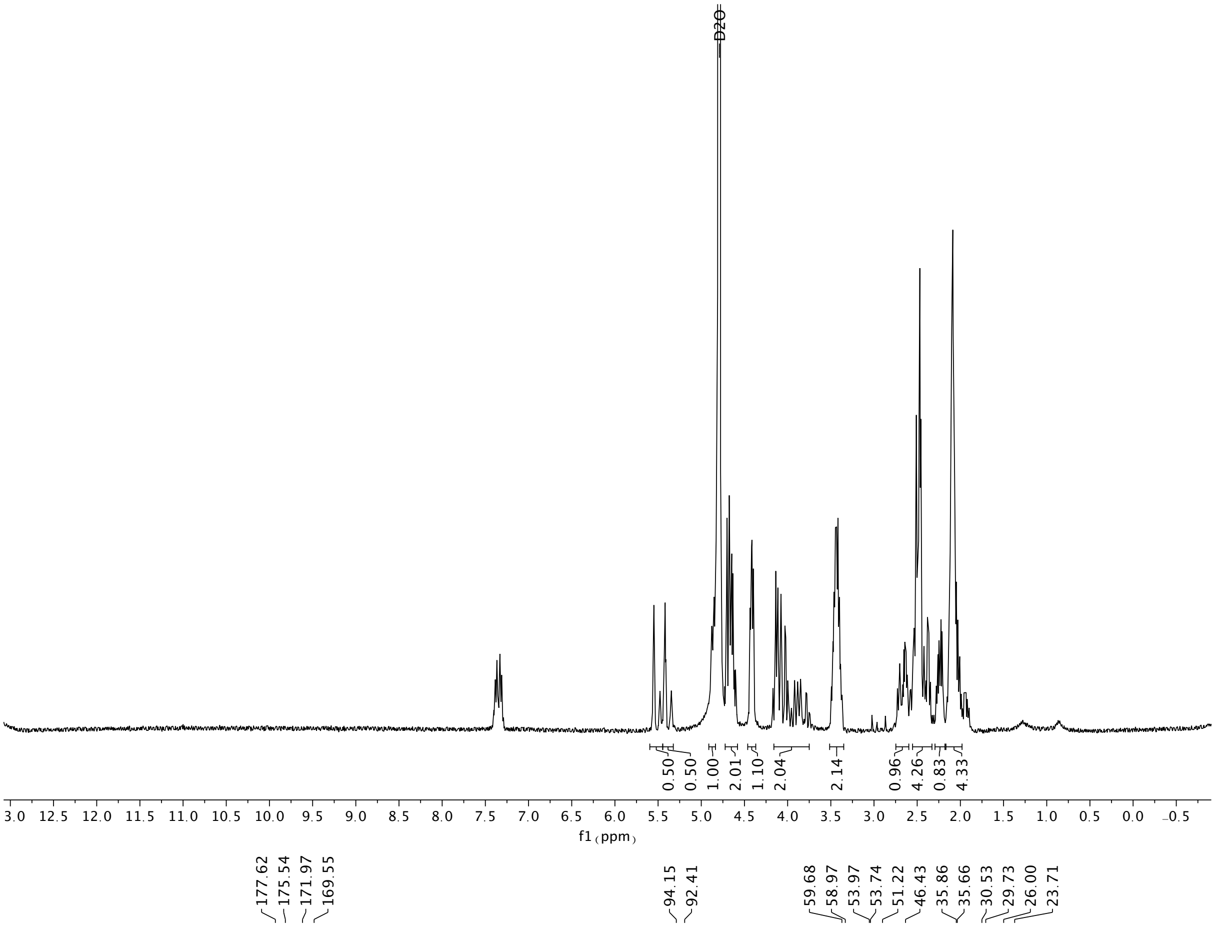

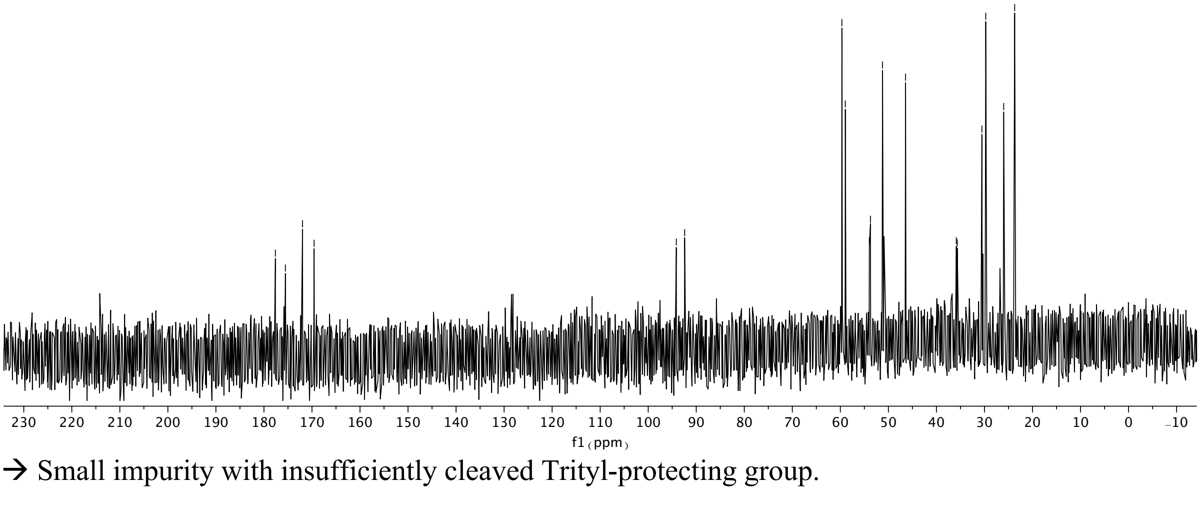

→ Small impurity with insufficiently cleaved Trityl-protecting group.

^{1}H and ^{13}C-NMR of H-D-Pro-L-(4*S*)-Flp-L-Glu-NH_2 · TFA (UTS-30):

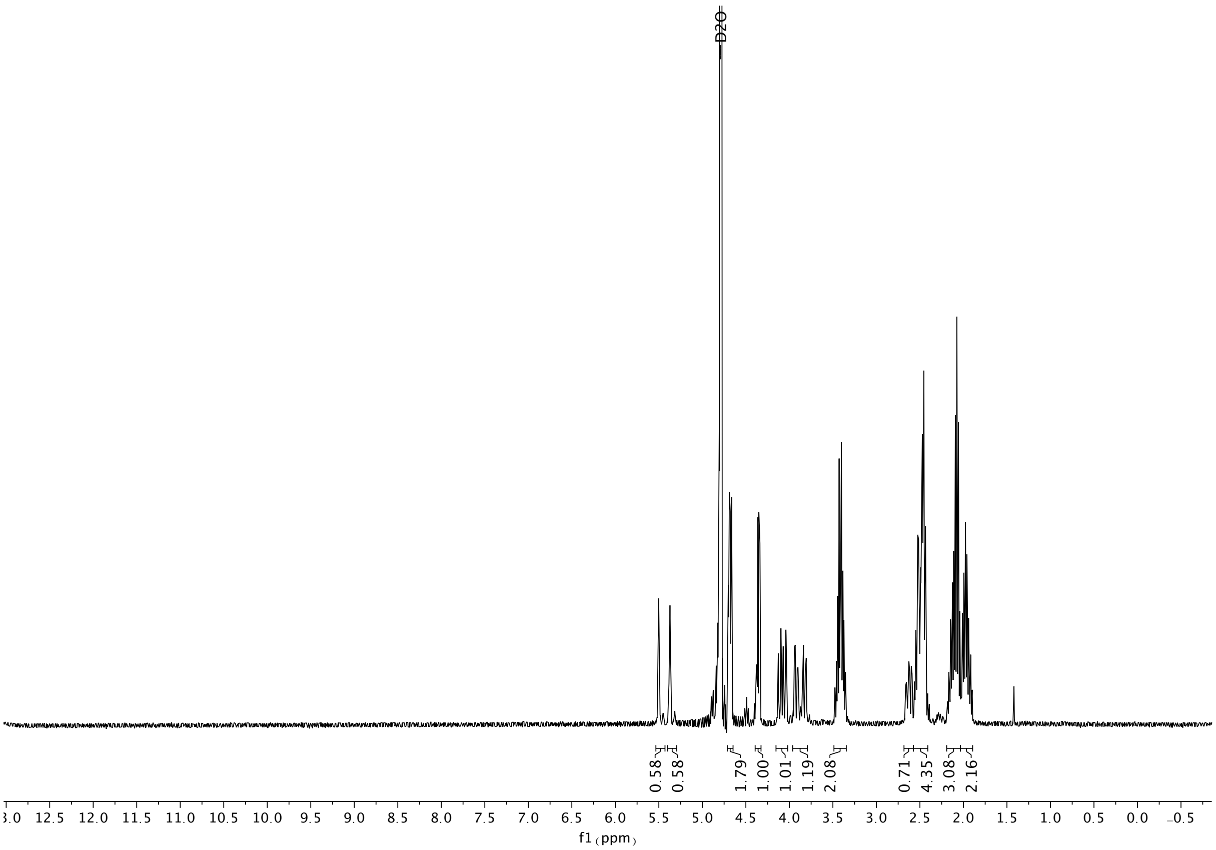

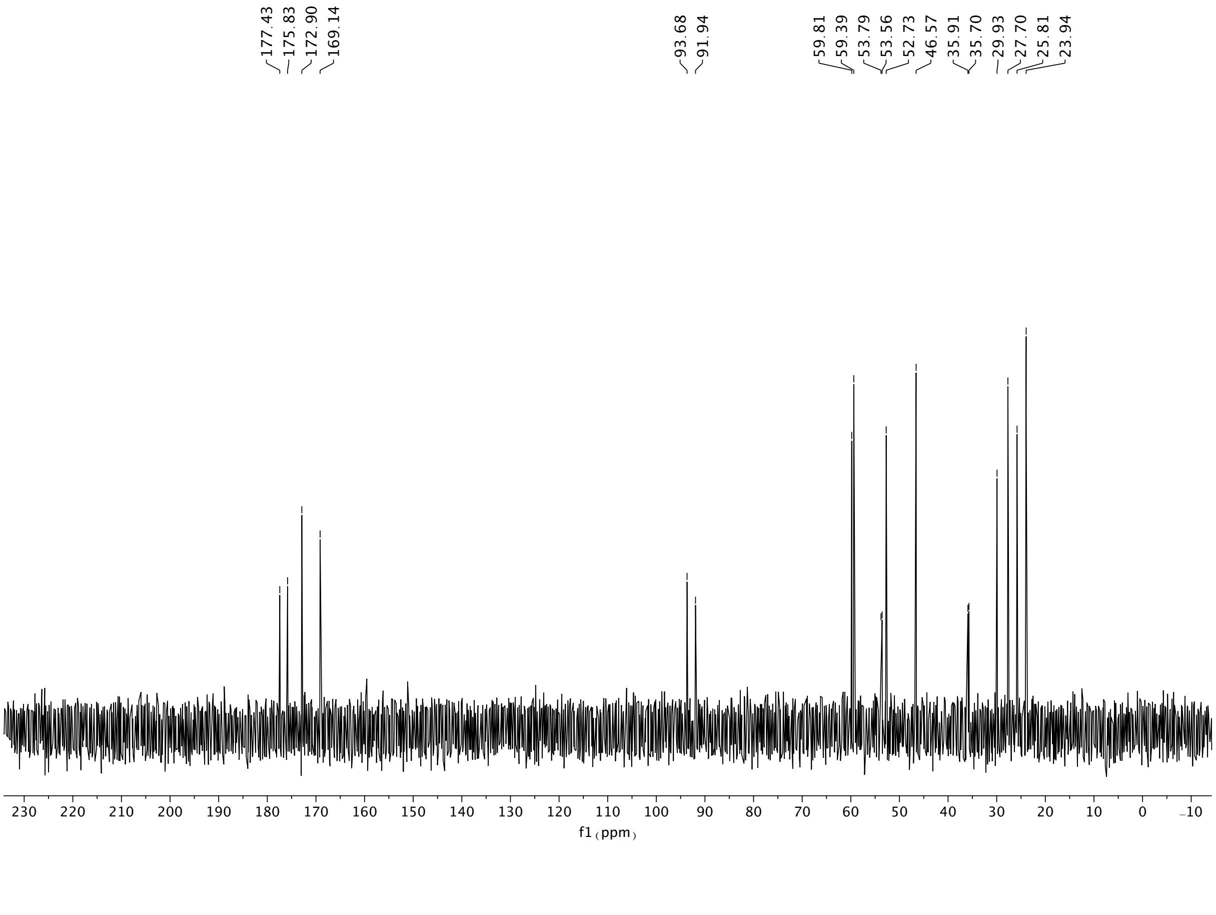

^{1}H and ^{13}C-NMR of H-D-Pro-L-(4*S*)-Flp-D-Glu-NH$_2$ · TFA (UTS-31):

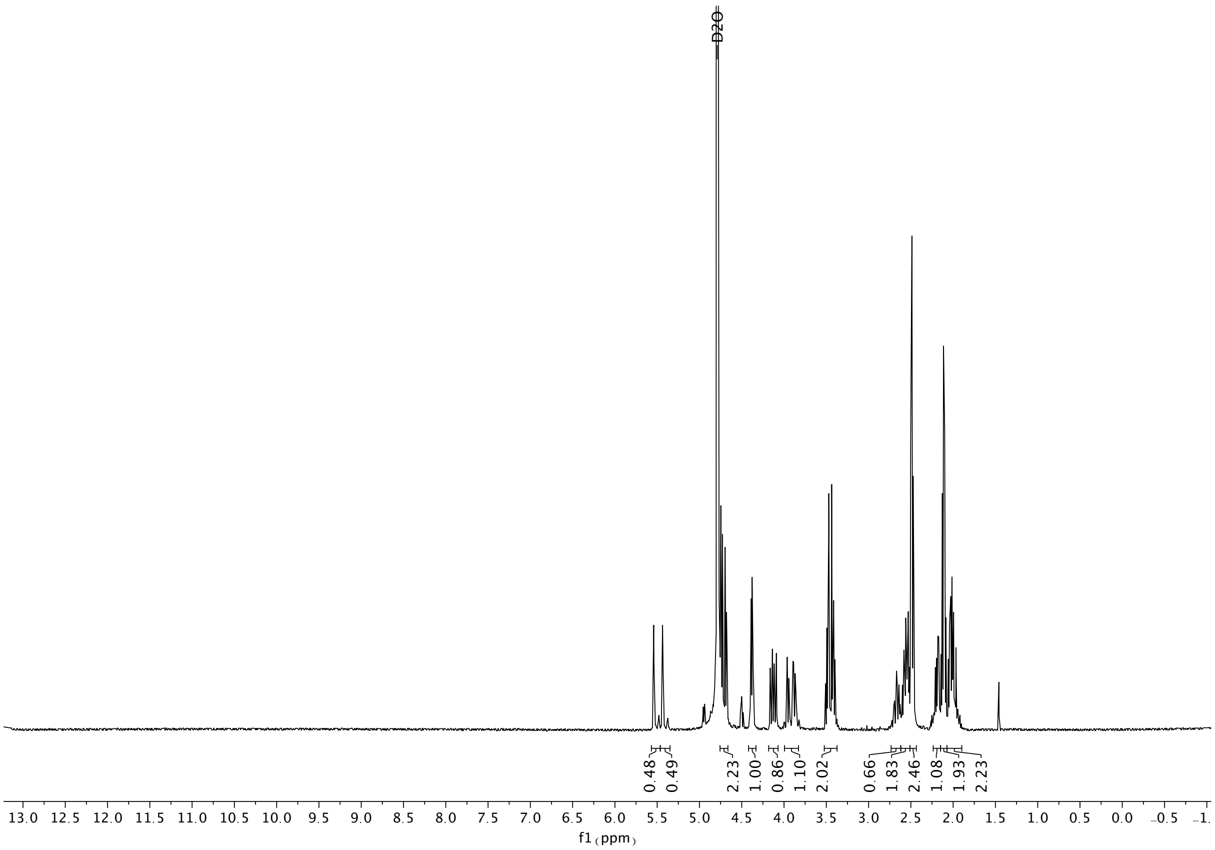

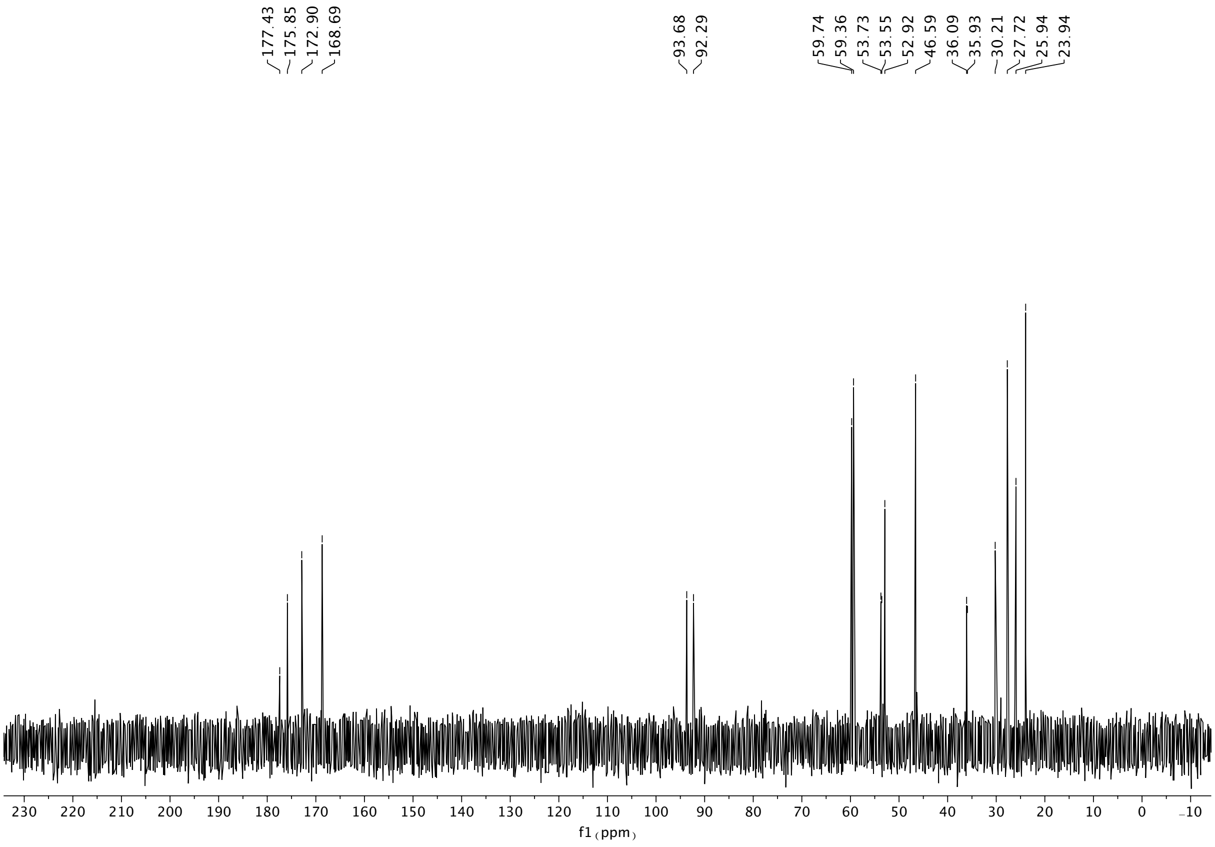

^{1}H and ^{13}C-NMR of H-D-Pro-L-(4*S*)-Flp-L-Tyr-NH₂ · TFA (UTS-32):

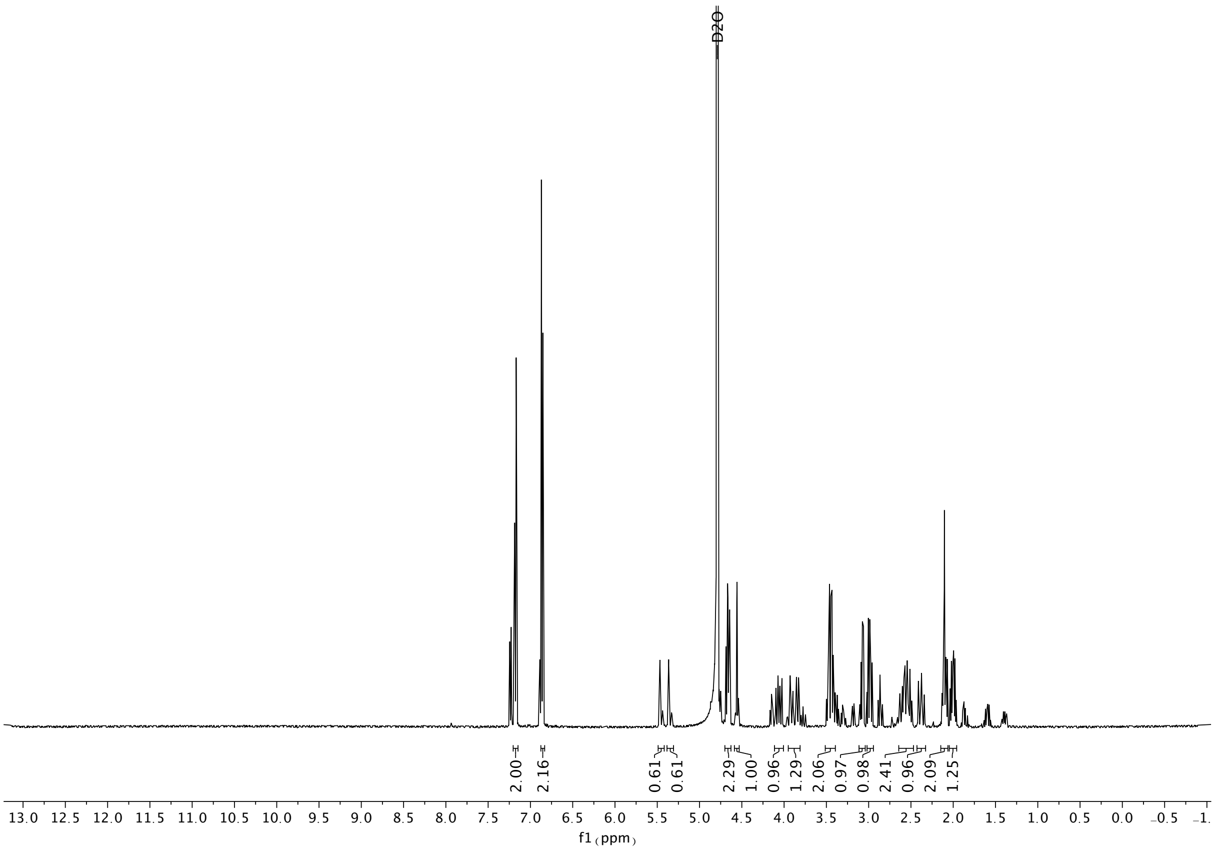

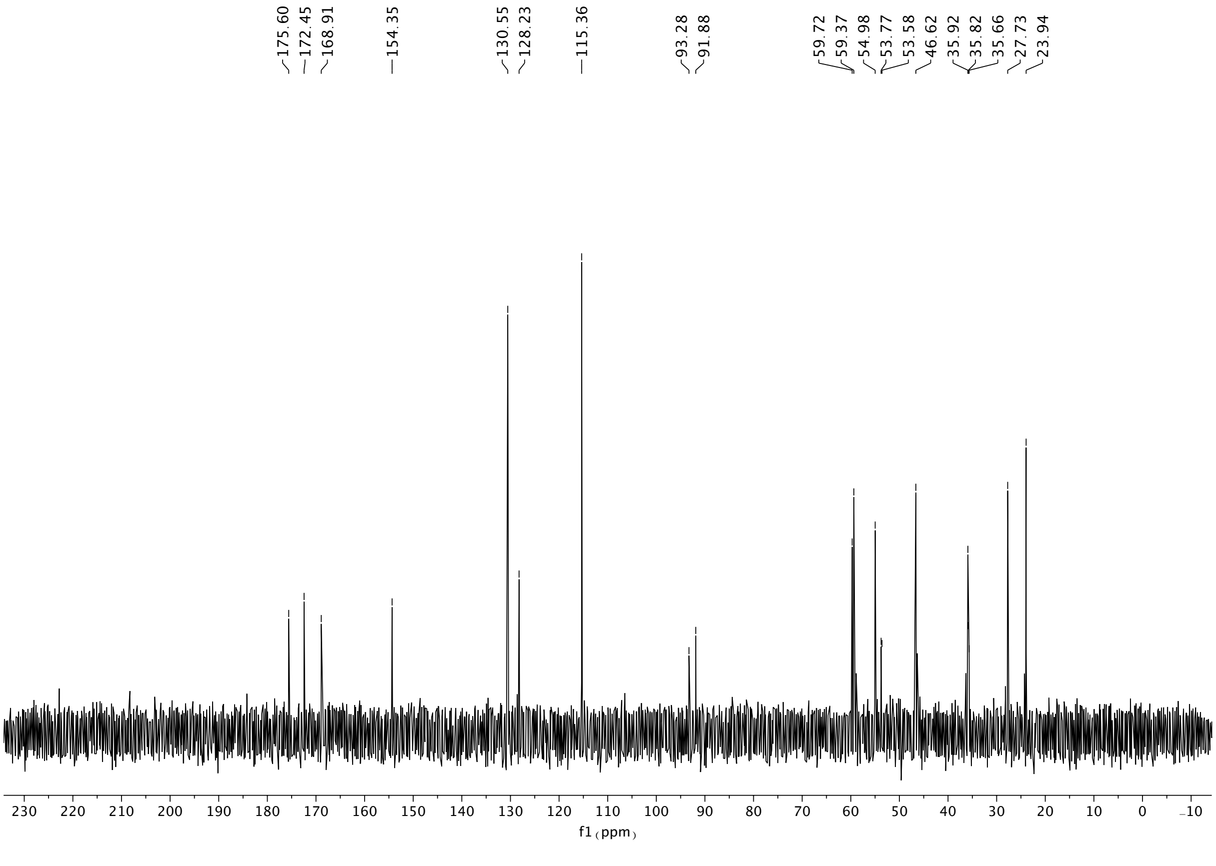

^{1}H and ^{13}C-NMR of H-D-Pro-L-(4*S*)-Flp-D-Tyr-NH2 · TFA (UTS-33):

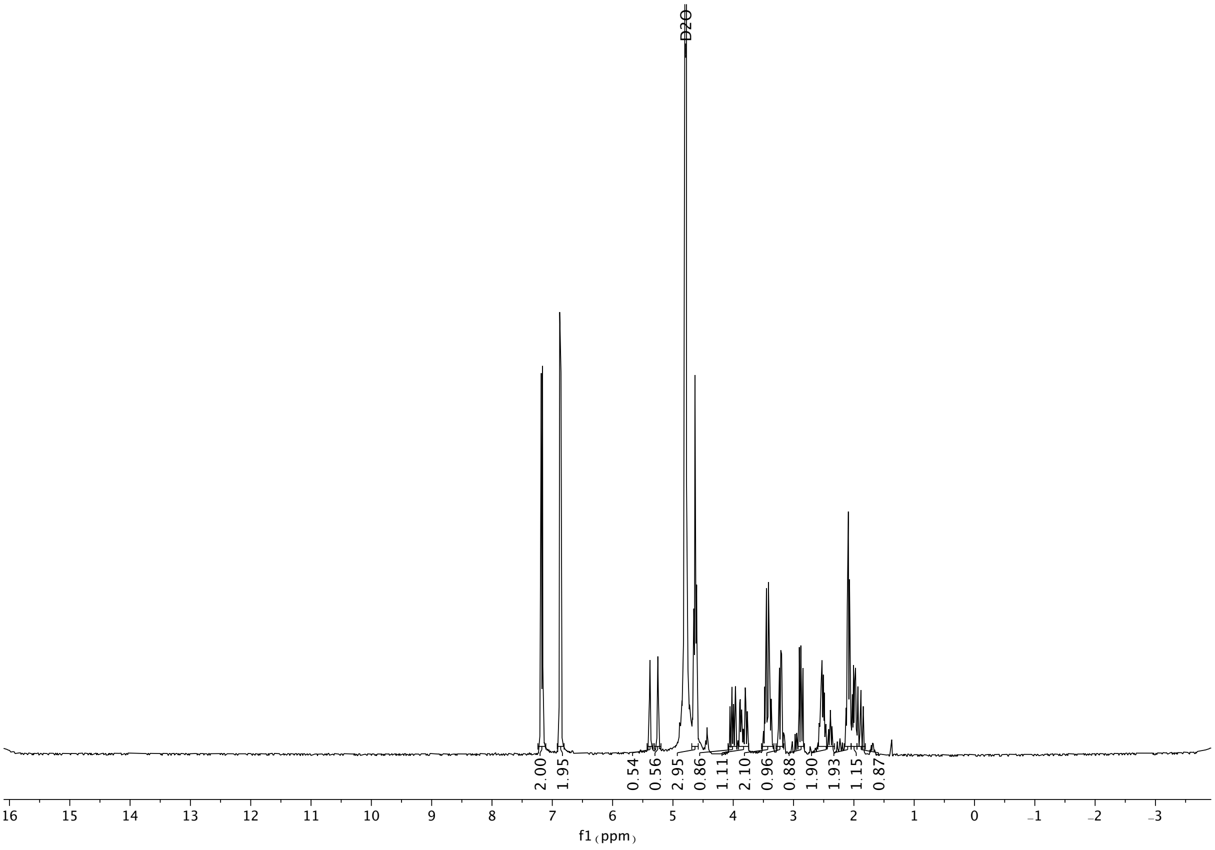

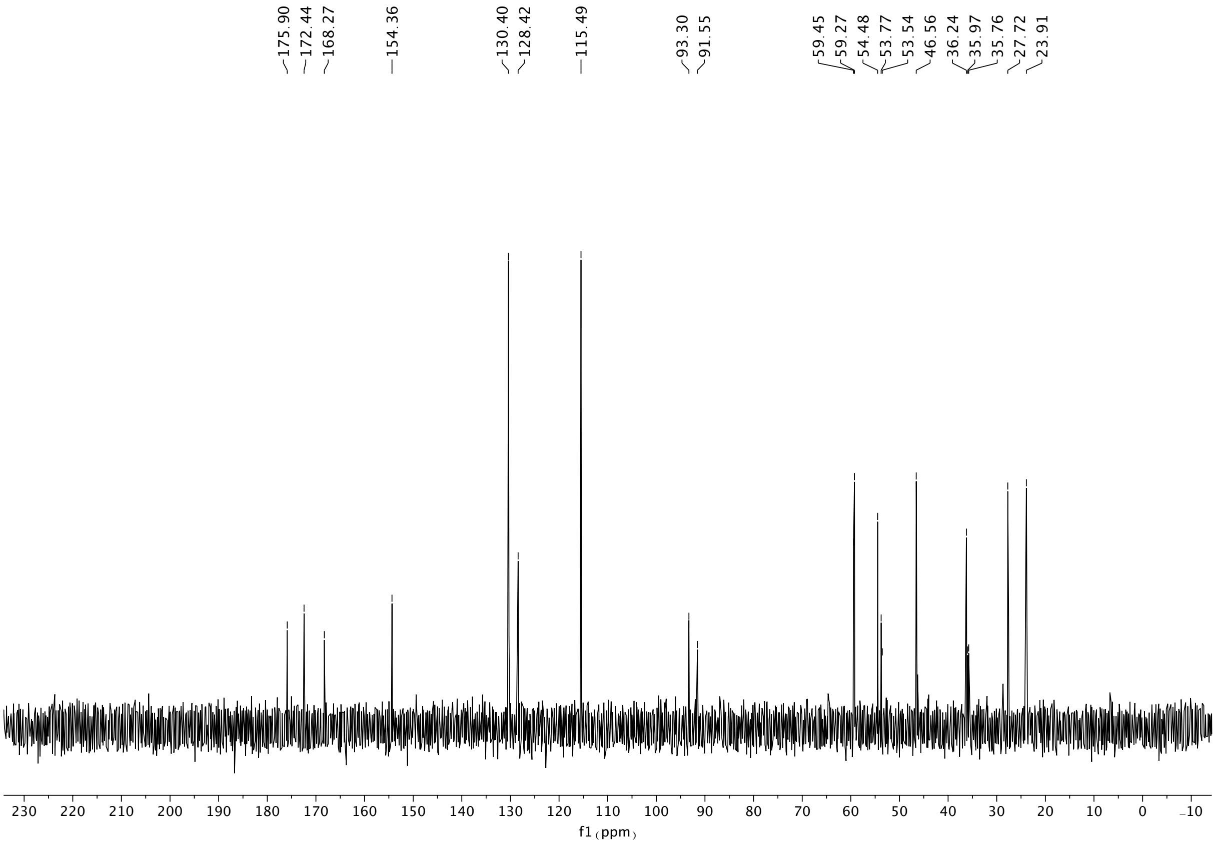

^{1}H and ^{13}C-NMR of H-D-Pro-L-(4*S*)-Flp-CyLeu-NH$_2$ · TFA (UTS-34):

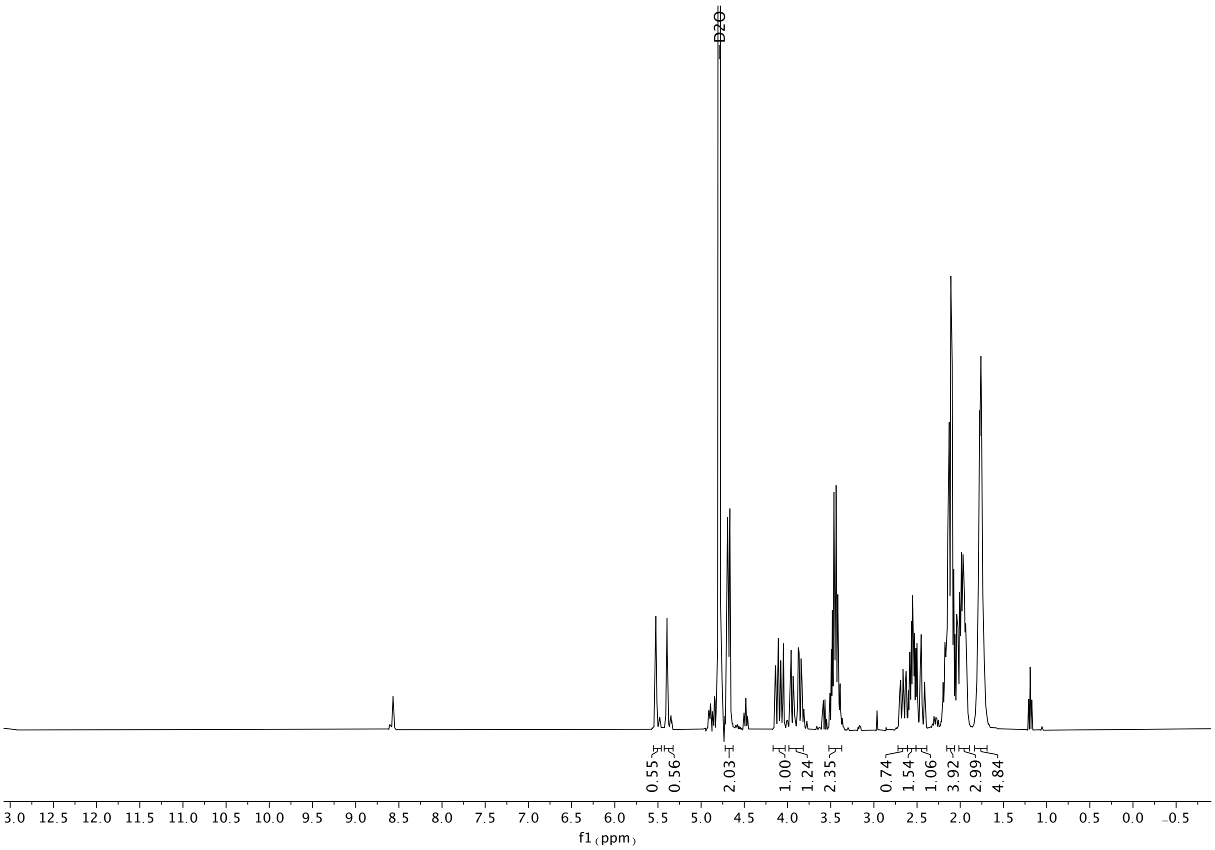

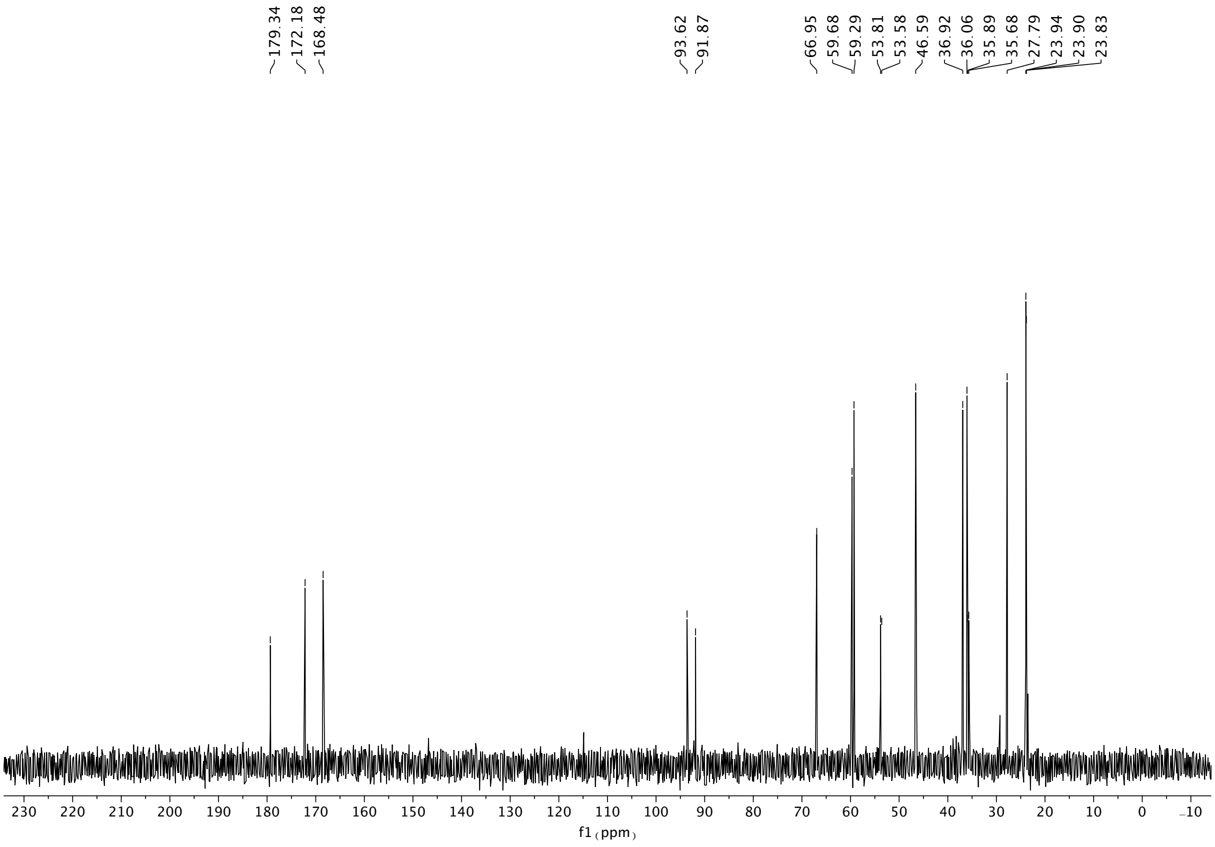

^{1}H and ^{13}C-NMR of H-D-Pro-L-(4*S*)-Flp-D-Ind-NH$_2$ · TFA (UTS-35):

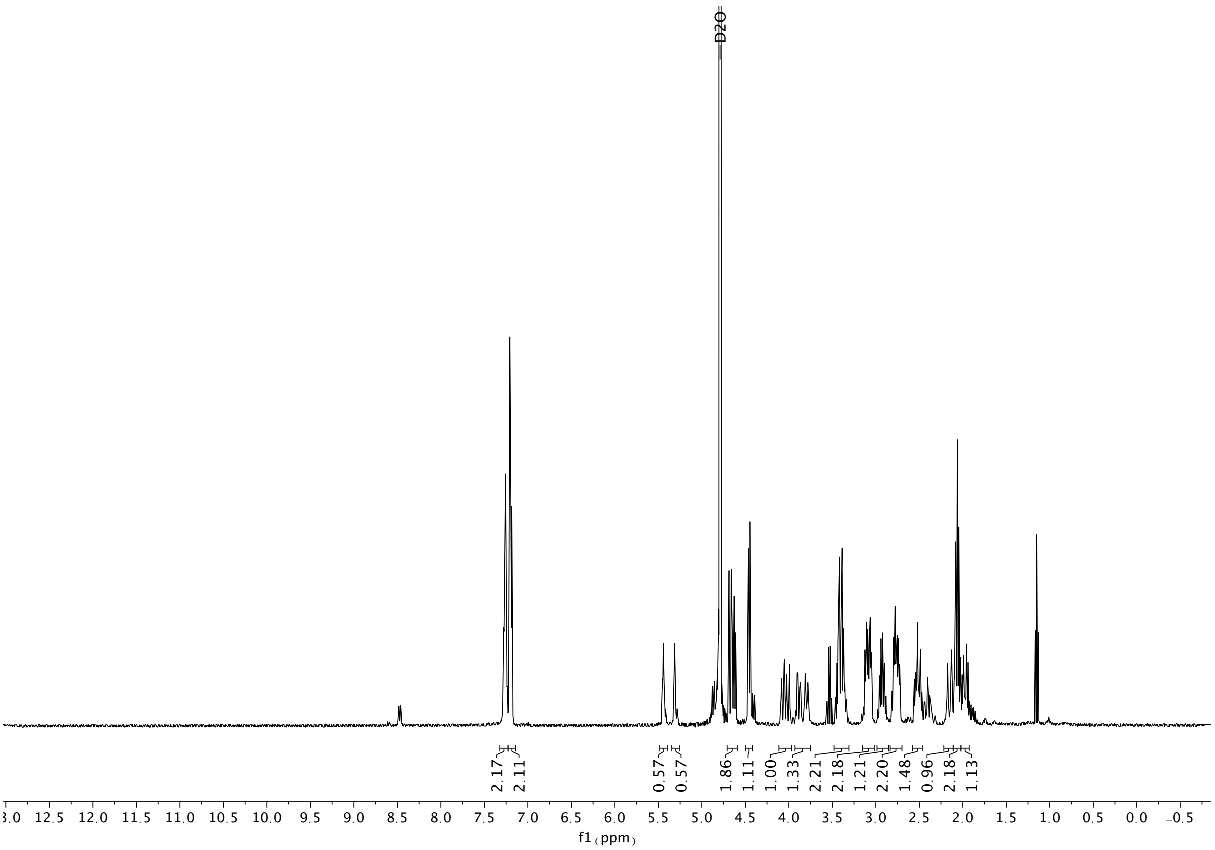

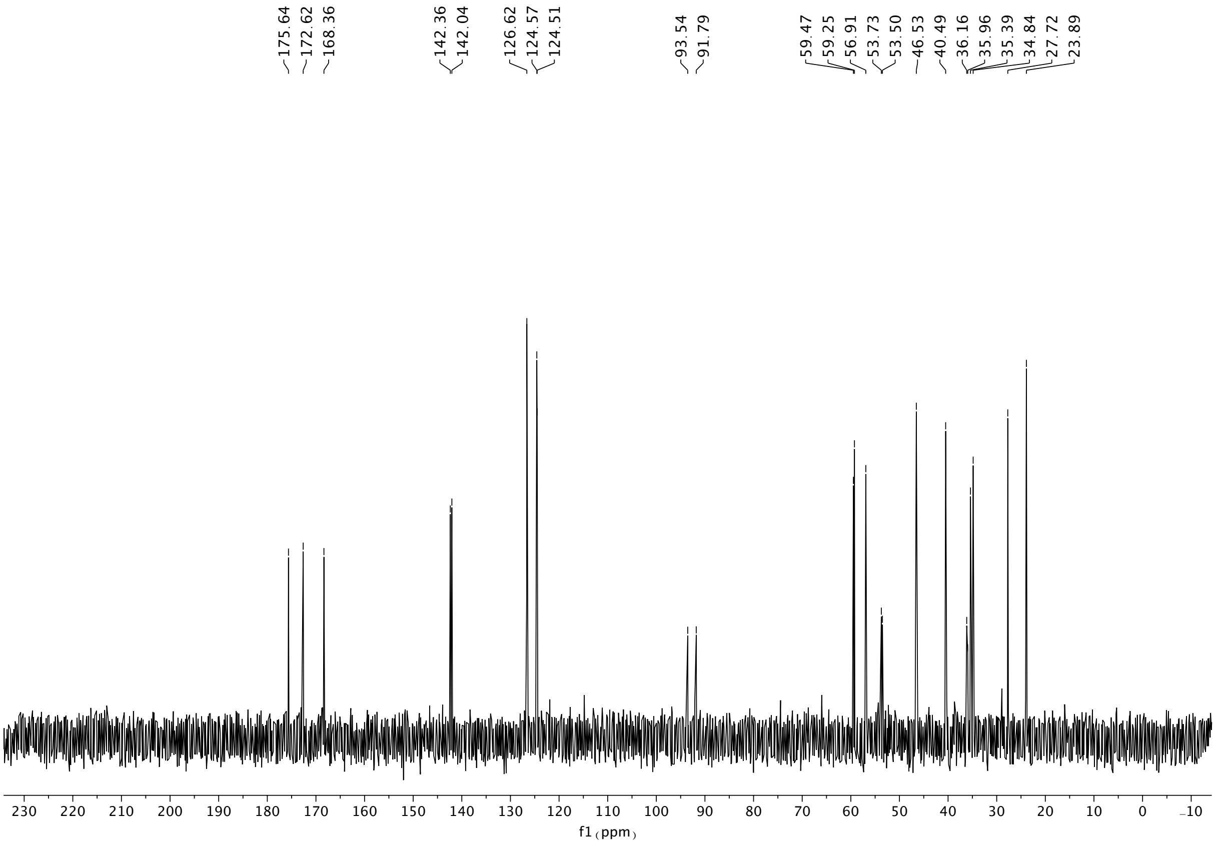

^{1}H and ^{13}C-NMR of H-D-Pro-L-Leu-L-(4*S*)-Flp-NH_2 · TFA (UTS-36):

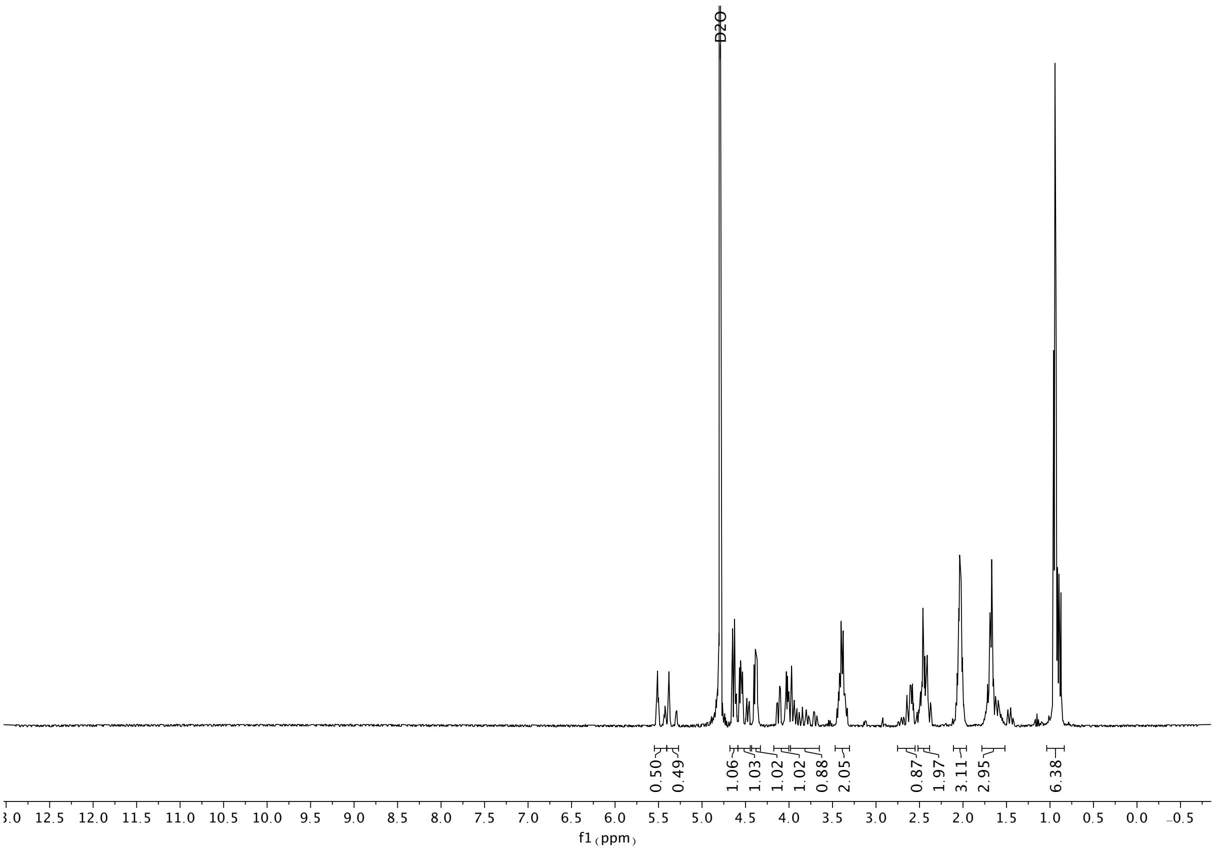

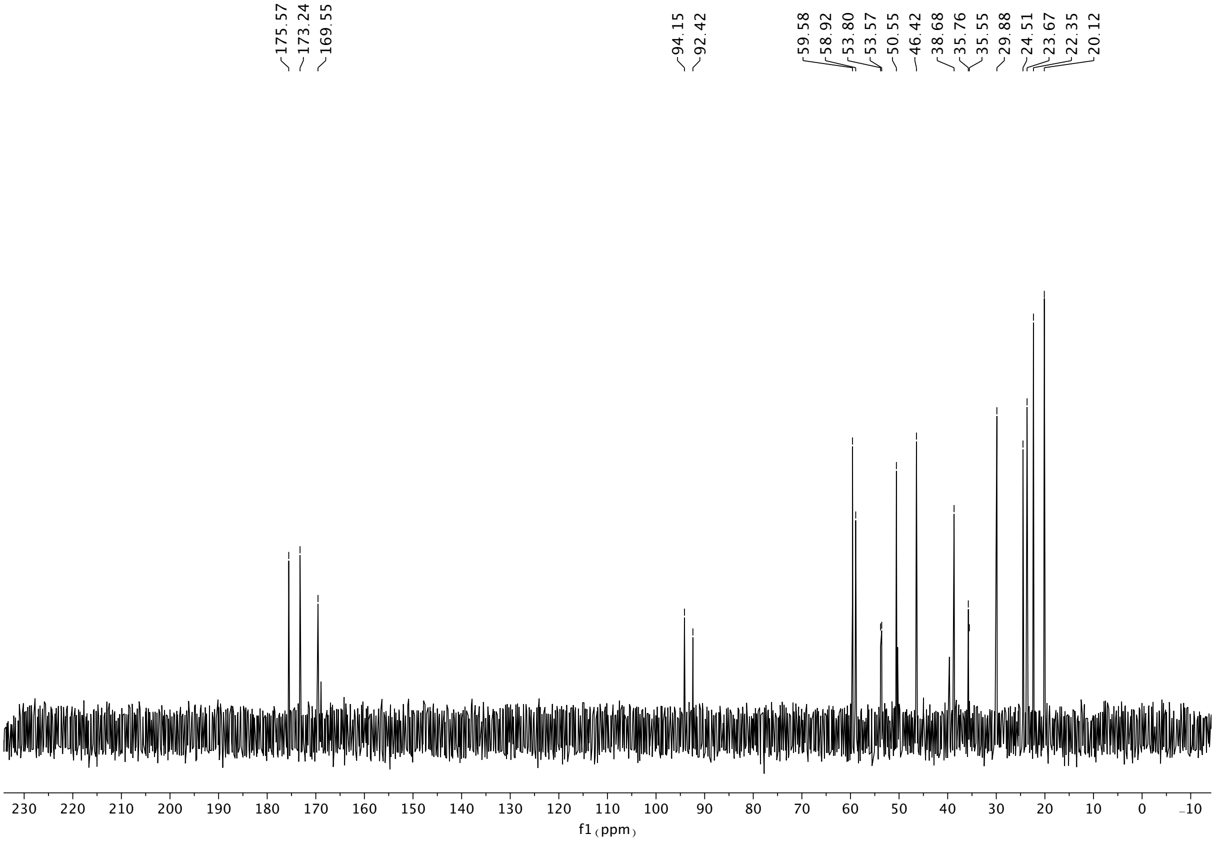

^{1}H and ^{13}C-NMR of H-D-Pro-D-Leu-L-(4*S*)-Flp-NH$_2$ · TFA (UTS-37):

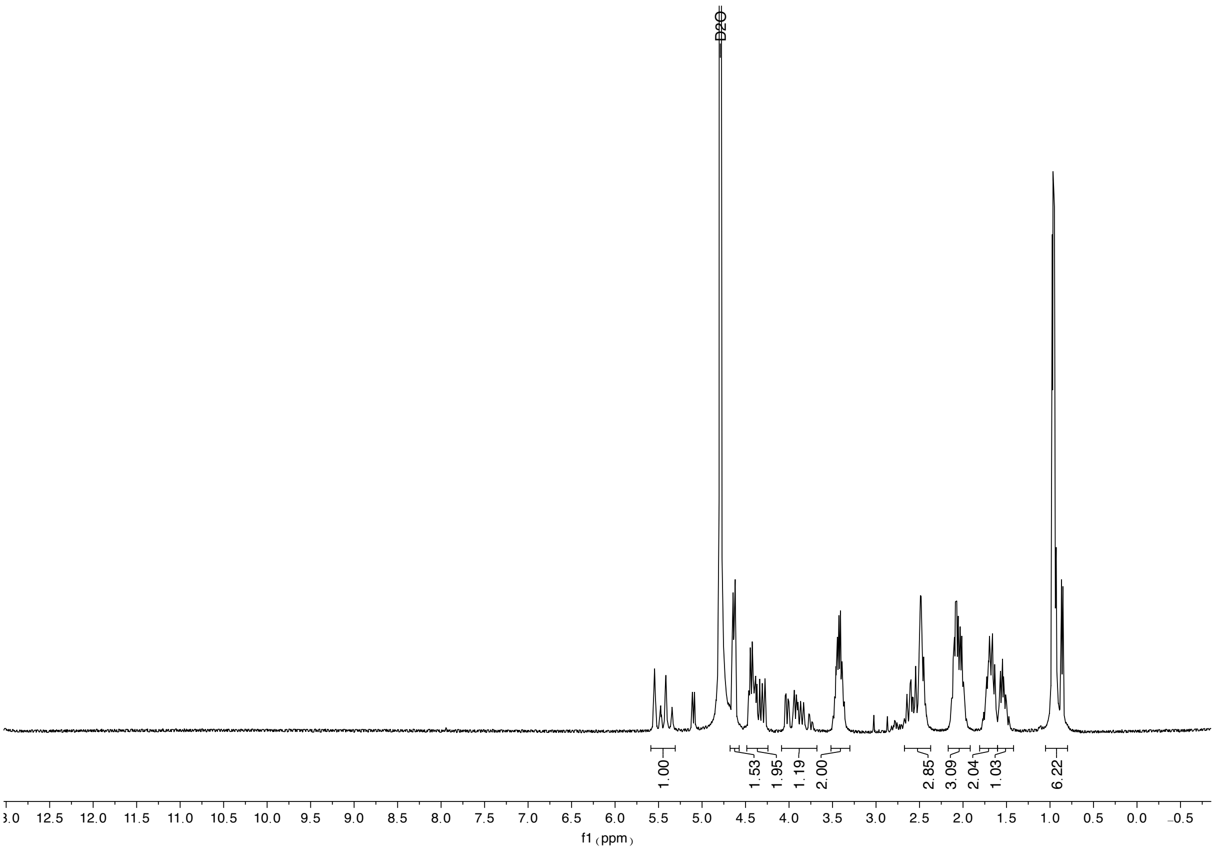

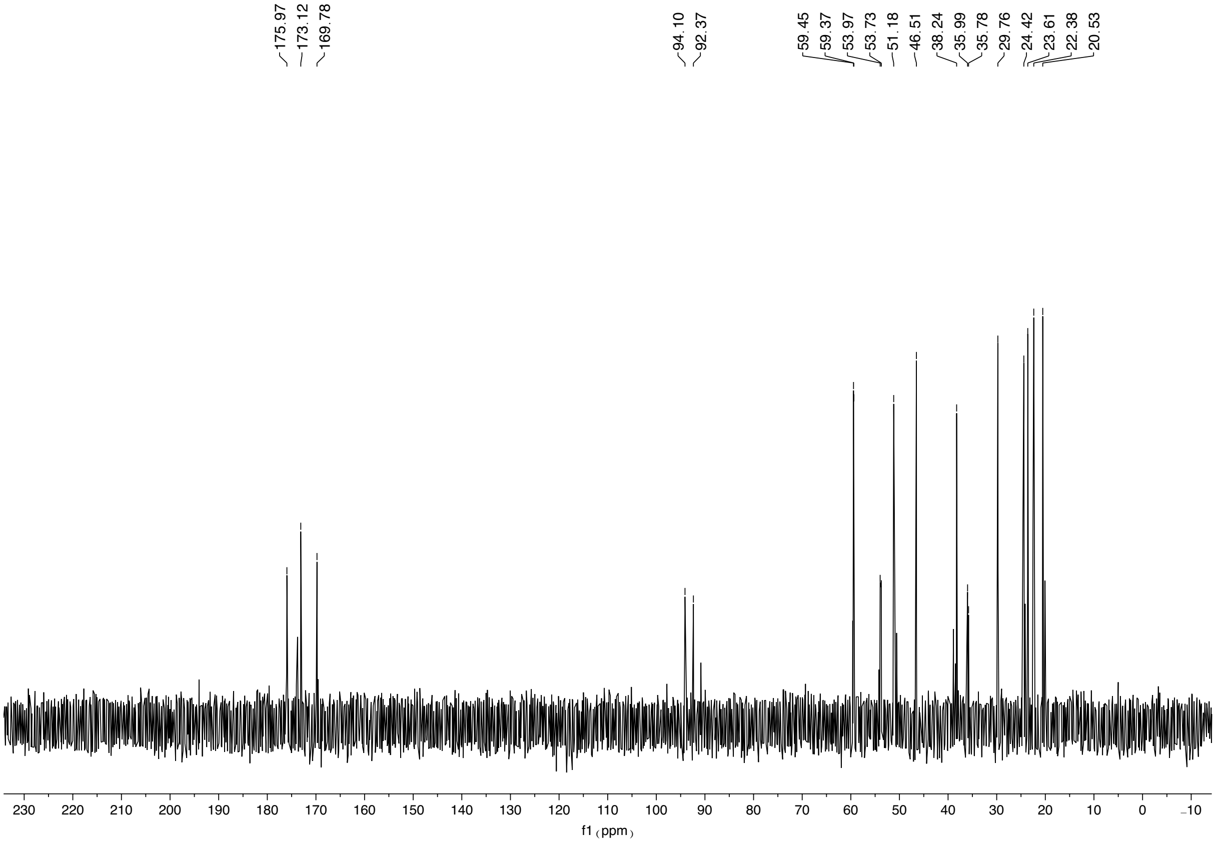

^{1}H and ^{13}C-NMR of H-D-Pro-L-Gln-L-(4*S*)-Flp-NH$_2$ · TFA (UTS-38):

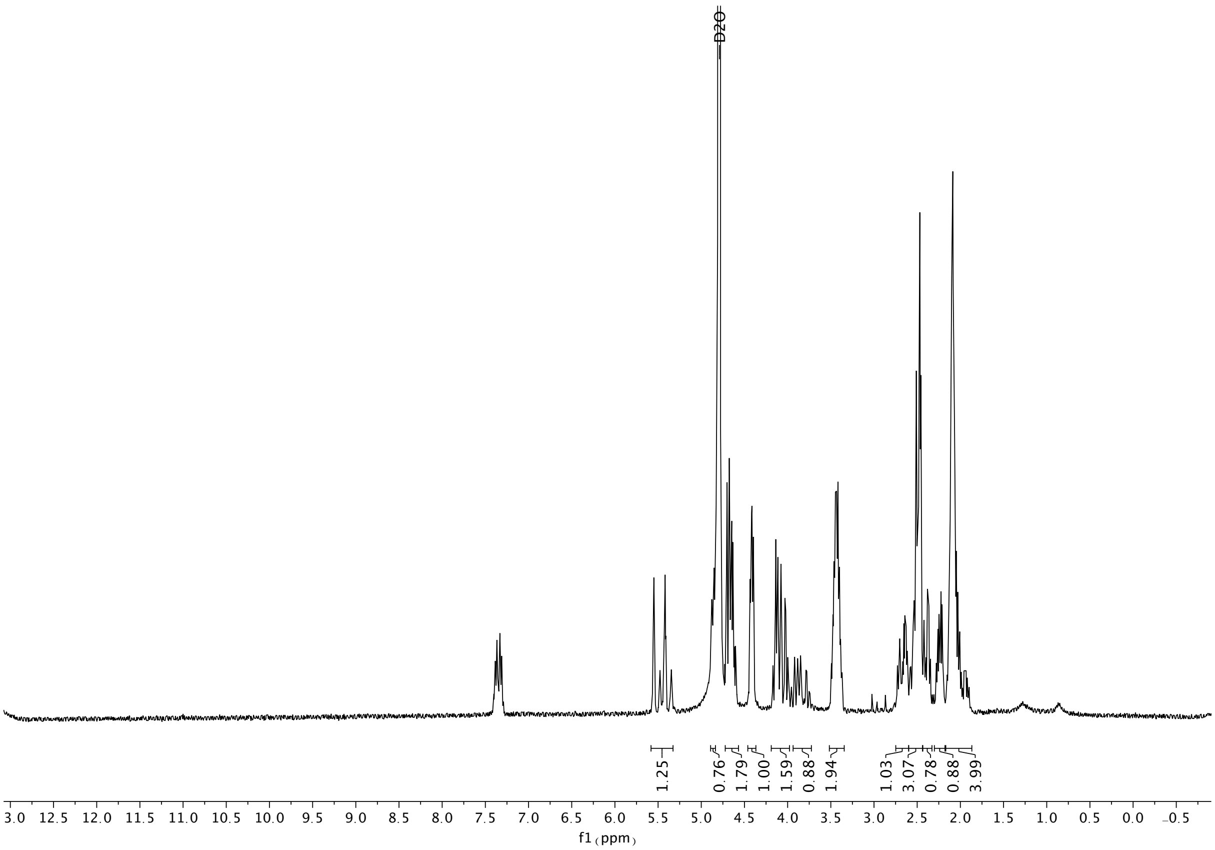

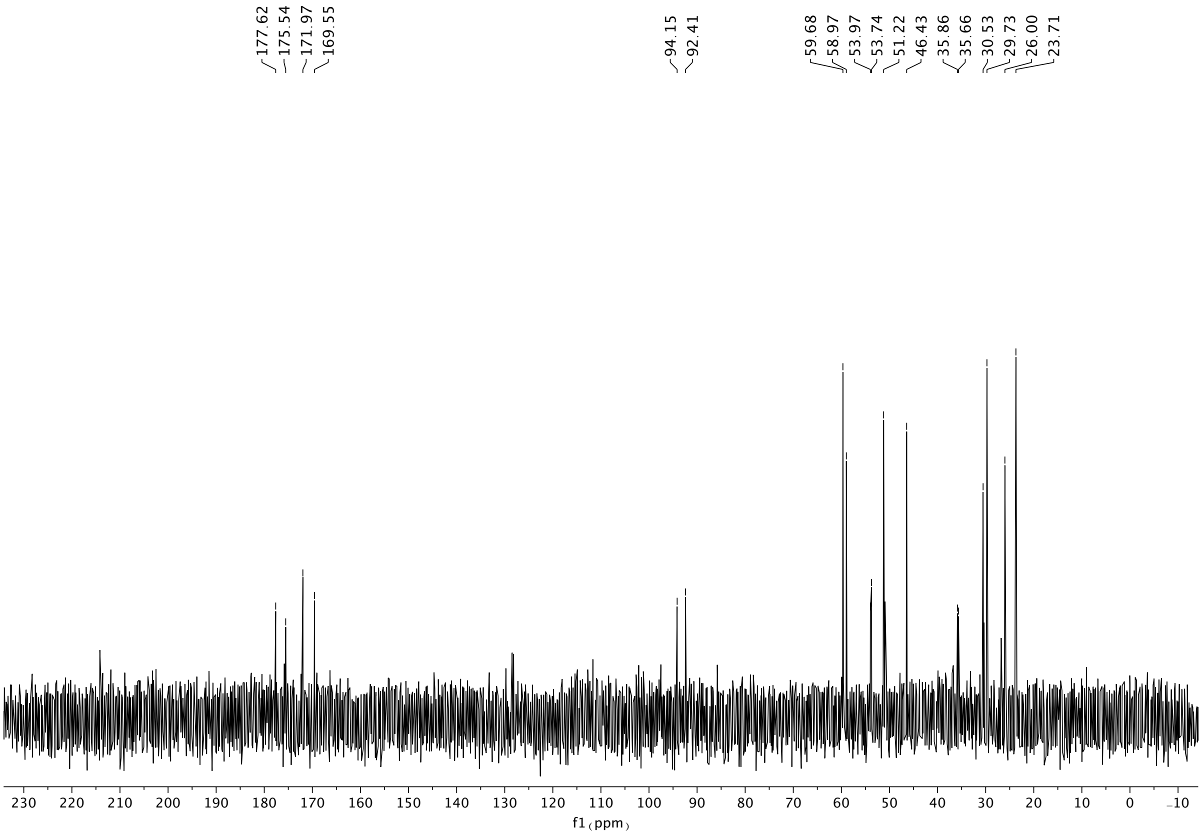

→ Small impurity of insufficiently cleaved Trityl-protecting group.

^{1}H and ^{13}C-NMR of H-D-Pro-D-Gln-L-(4*S*)-Flp-NH$_2$ · TFA (UTS-39):

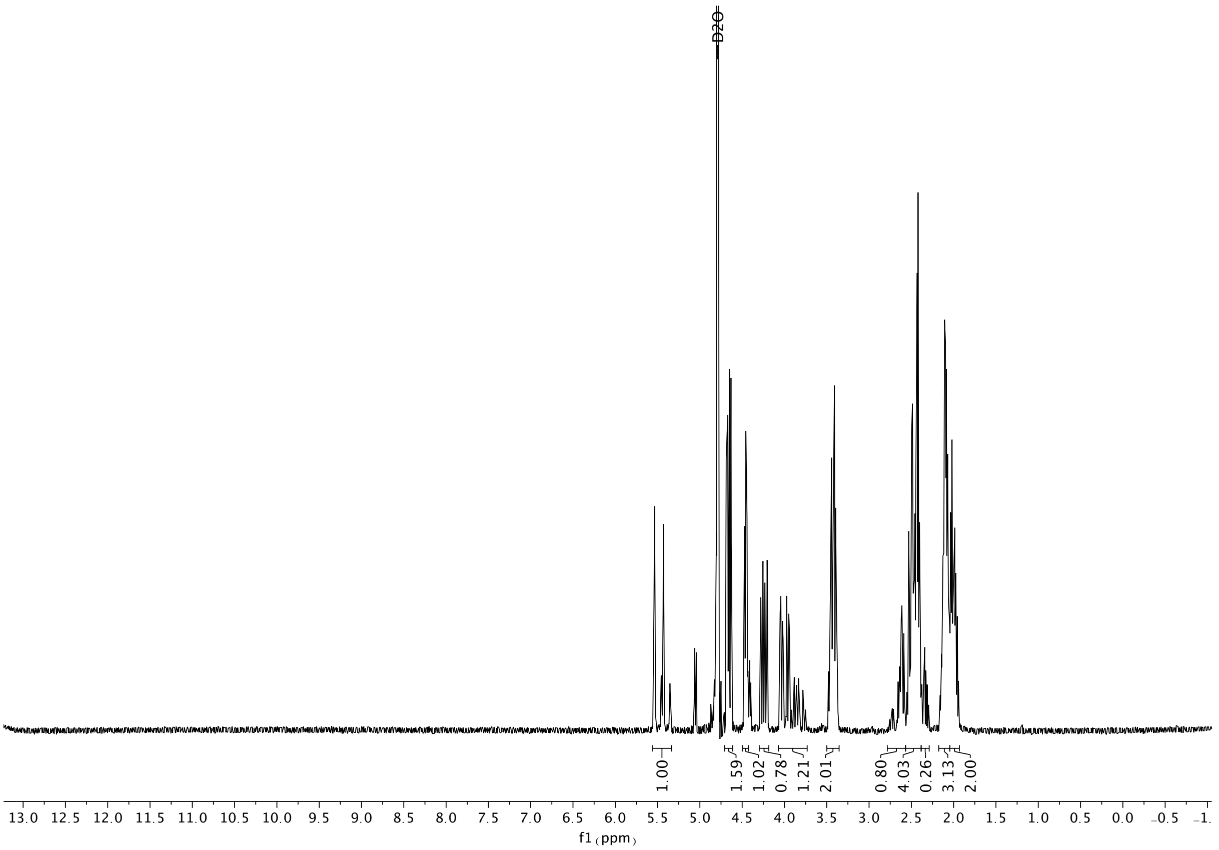

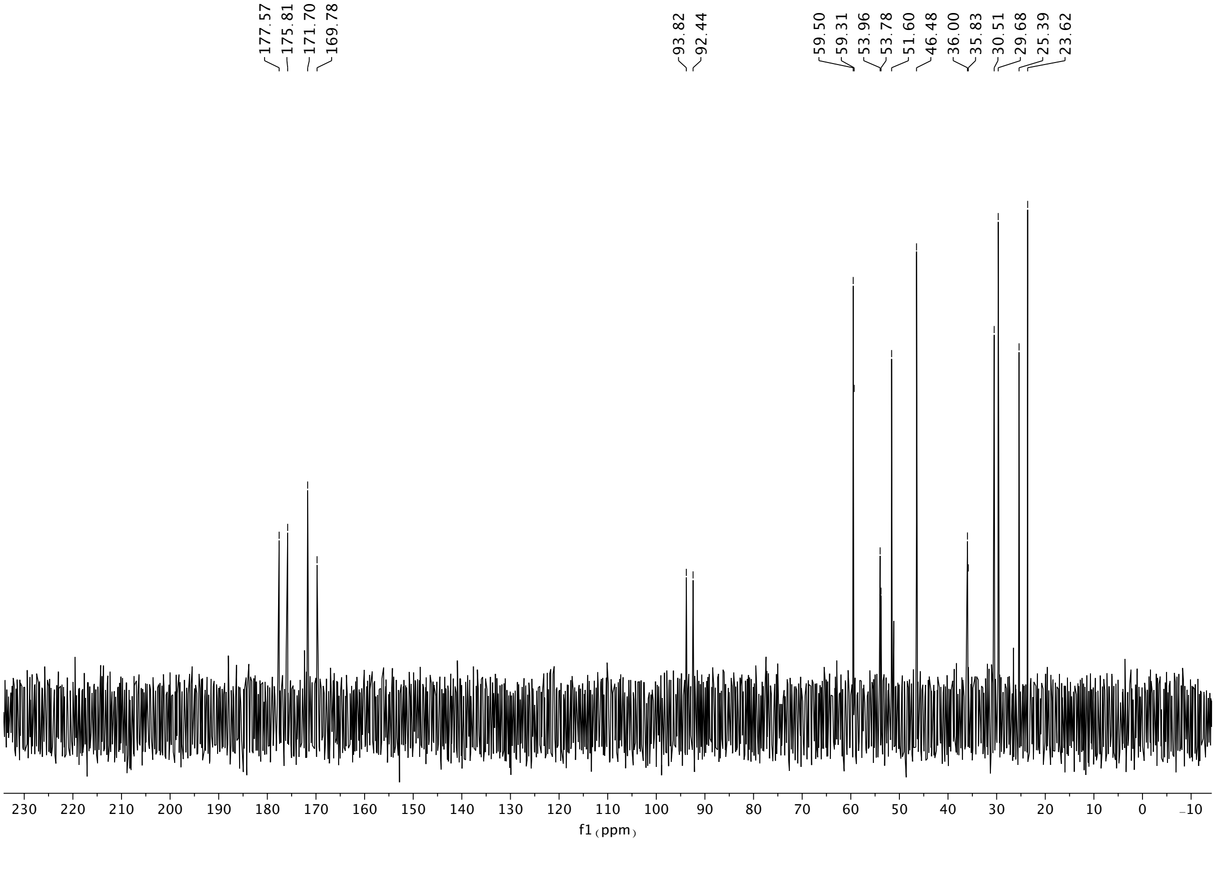

^{1}H and ^{13}C-NMR of H-D-Pro-L-Glu-L-(4*S*)-Flp-NH_2 · TFA (UTS-40):

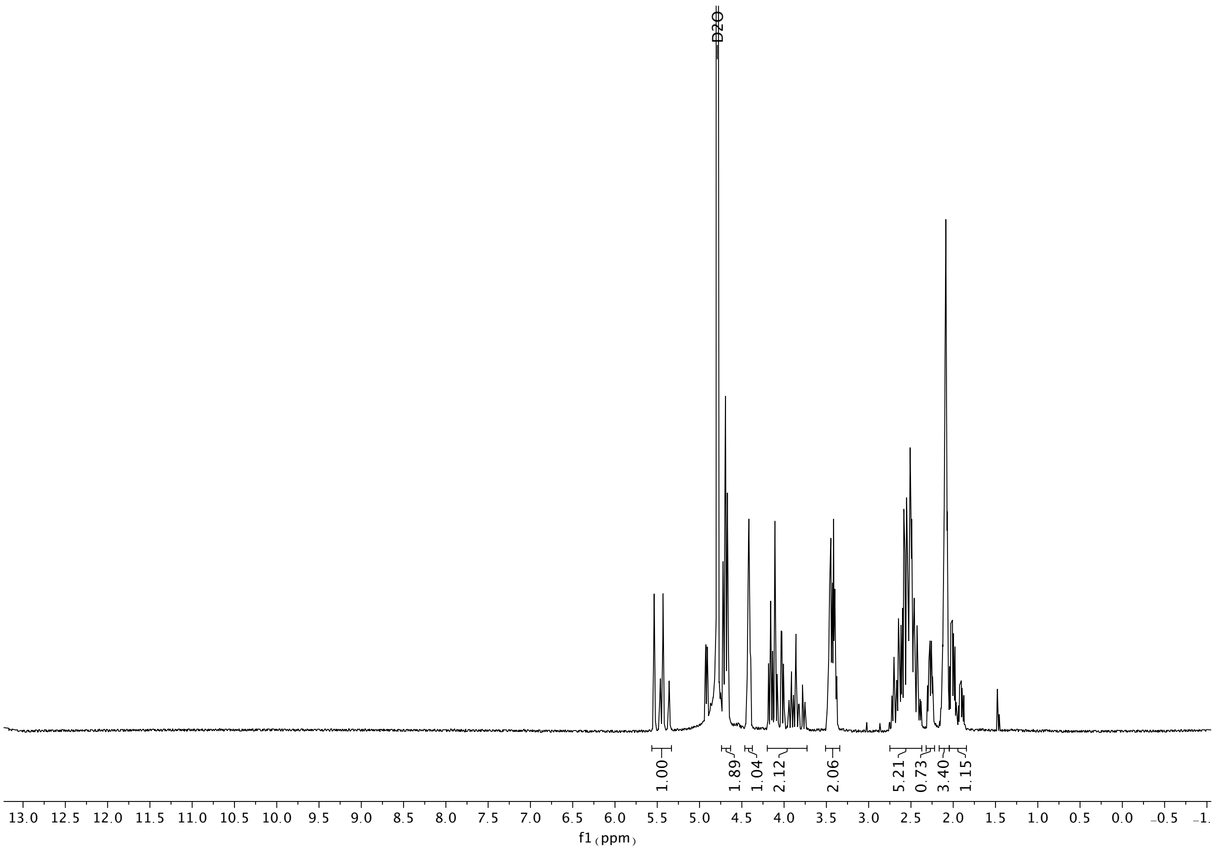

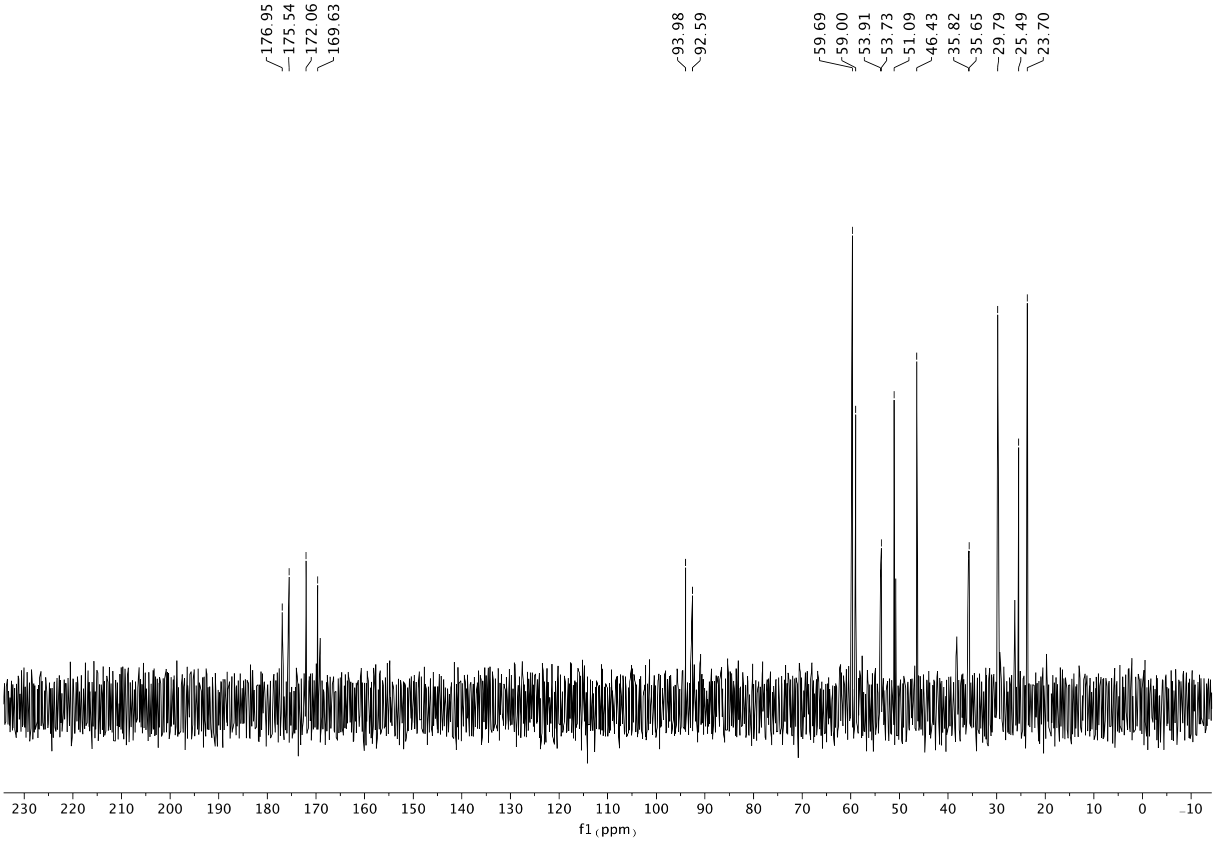

^{1}H and ^{13}C-NMR of H-D-Pro-D-Glu-L-(4*S*)-Flp-NH$_2$ · TFA (UTS-41):

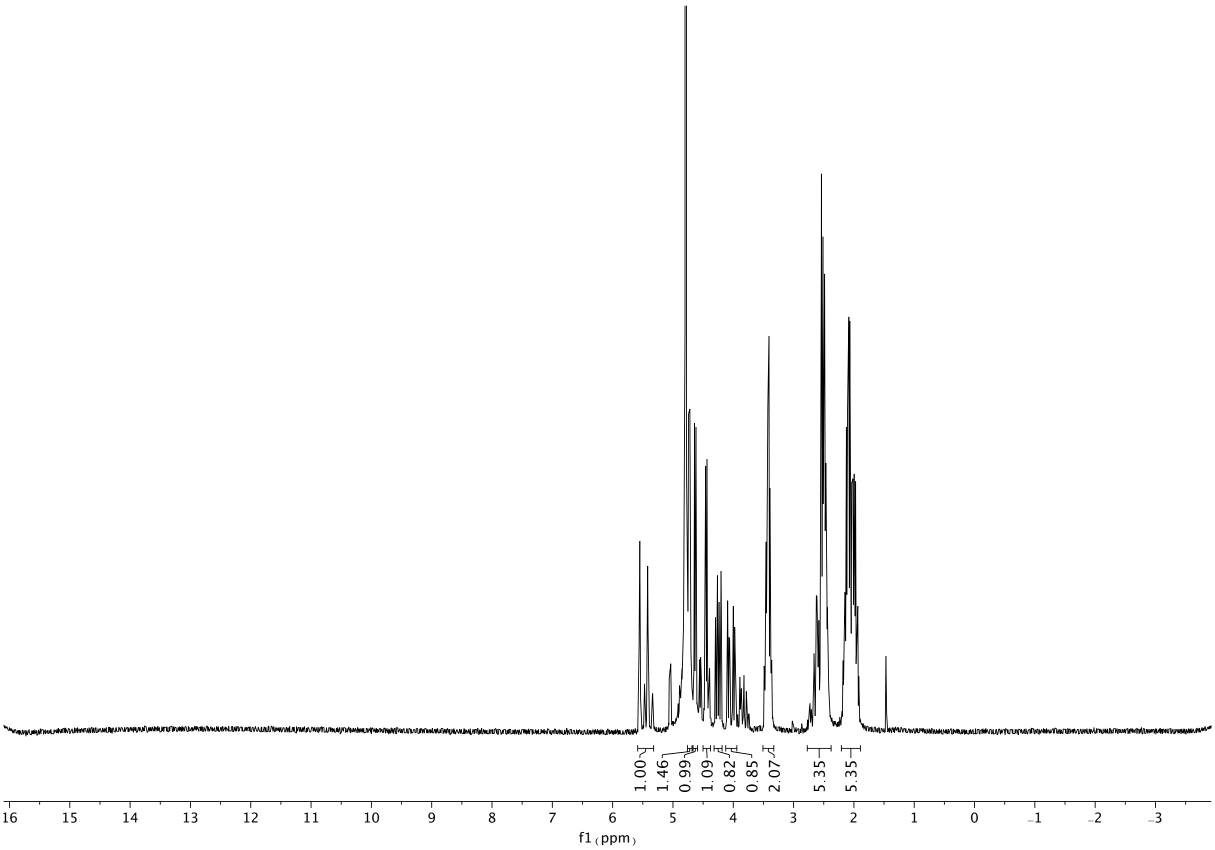

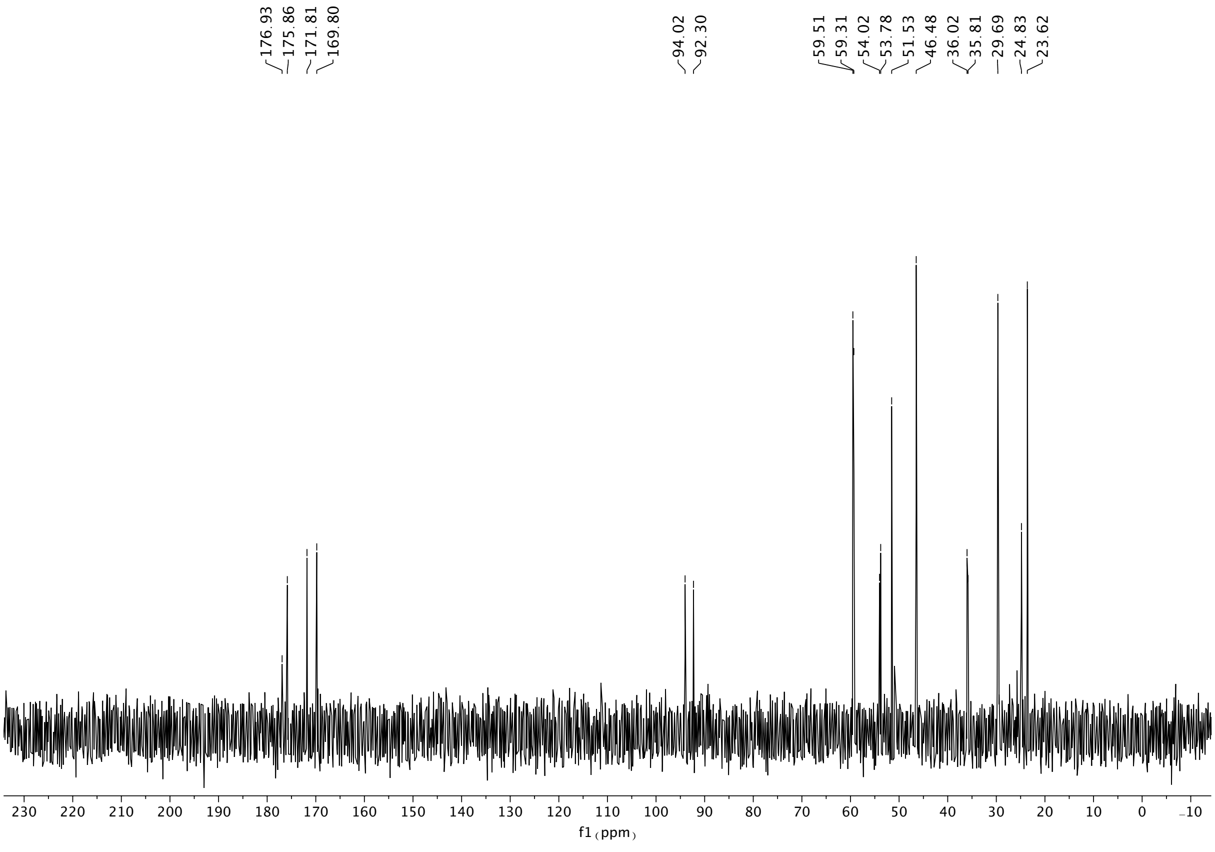

^{1}H and ^{13}C-NMR of H-D-Pro-L-Tyr-L-(4*S*)-Flp-NH$_2$ · TFA (UTS-42):

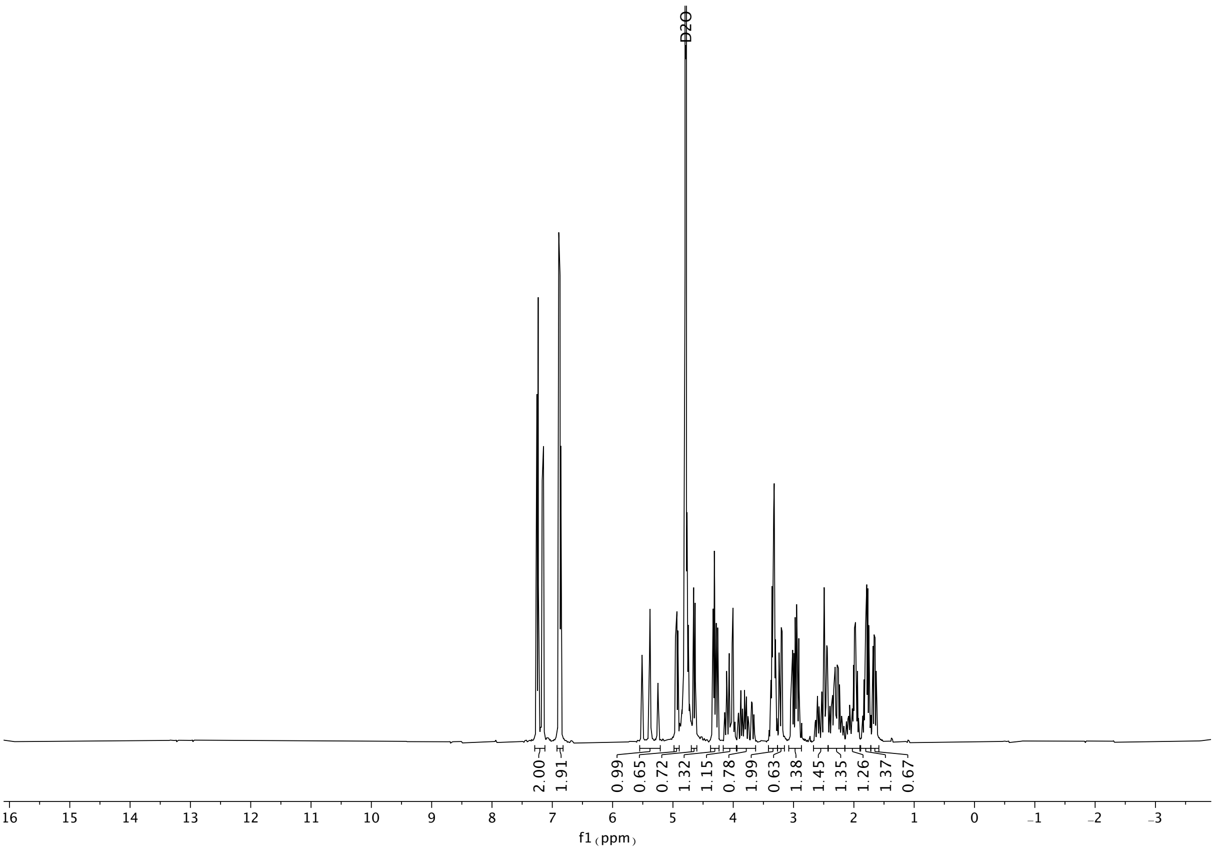

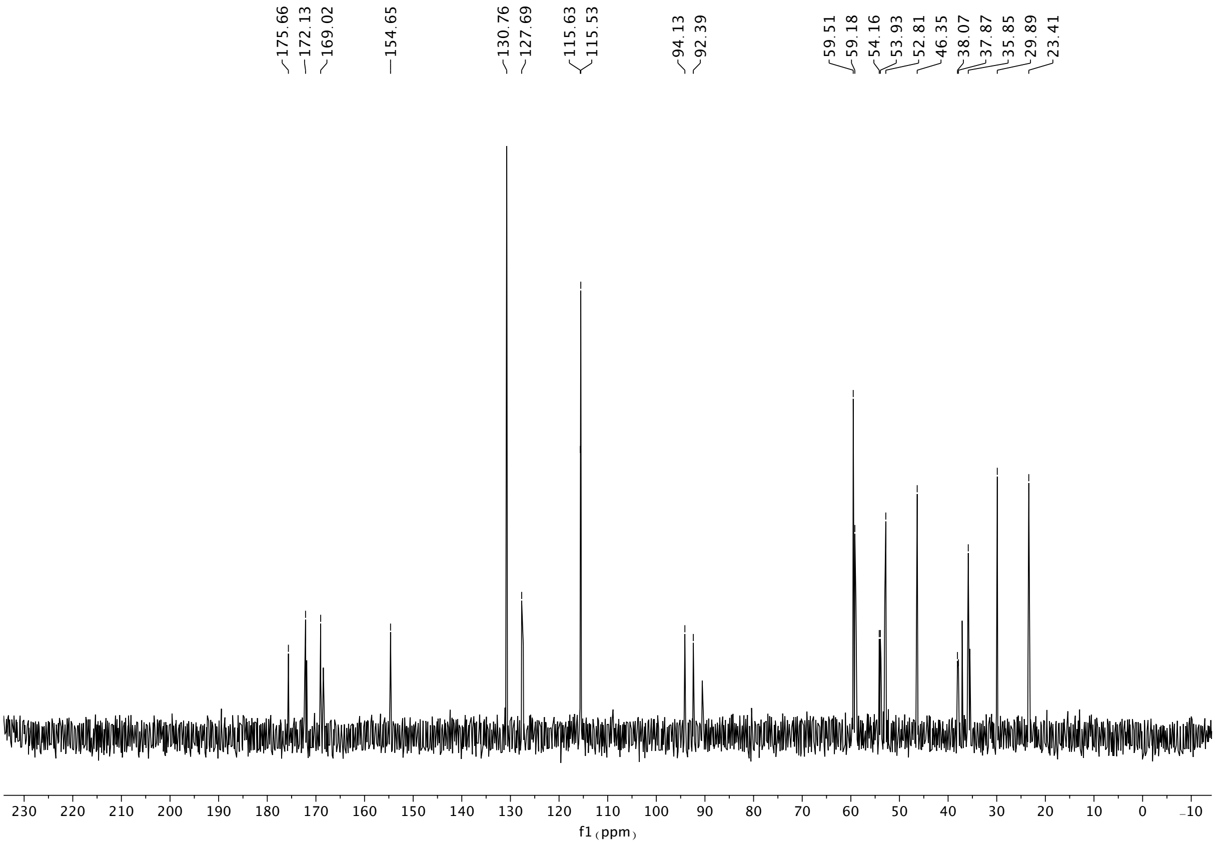

^{1}H and ^{13}C-NMR of H-D-Pro-D-Tyr-L-(4*S*)-Flp-NH$_2$ · TFA (UTS-43):

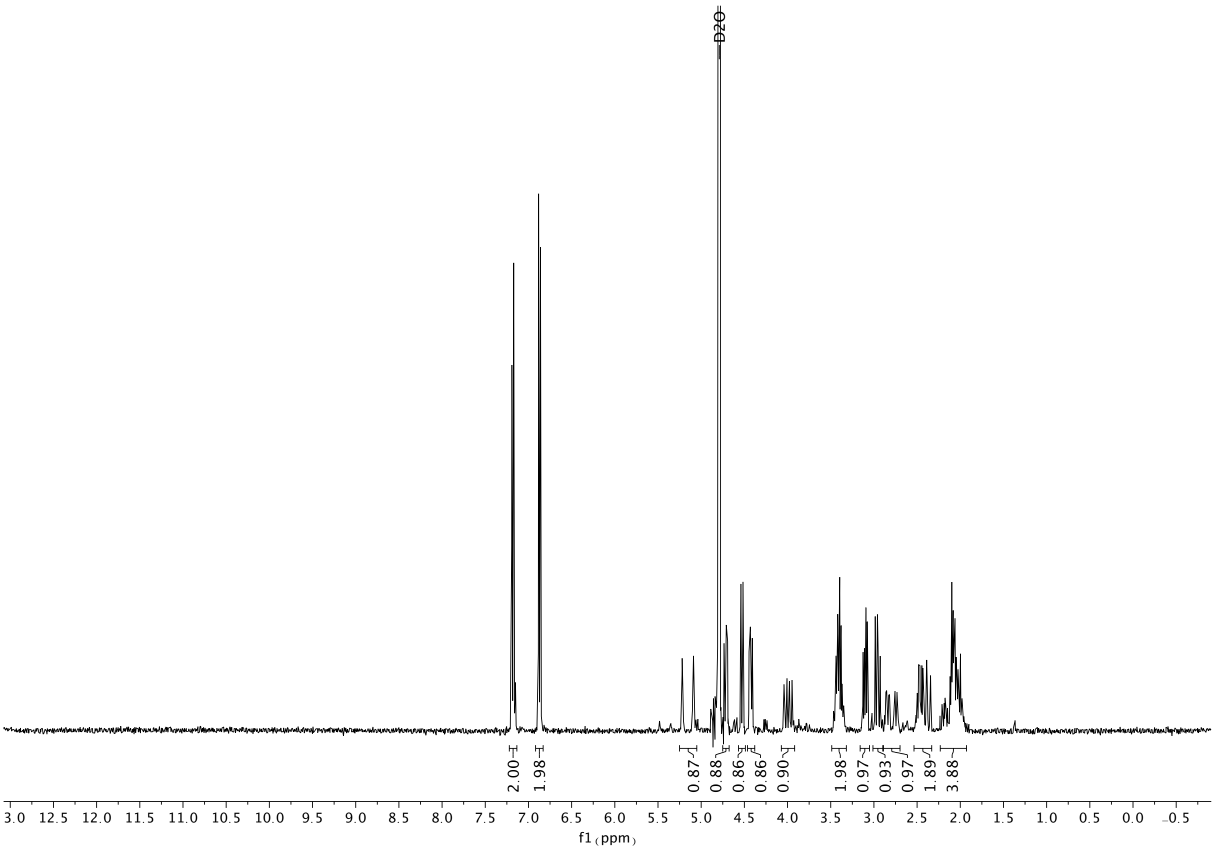

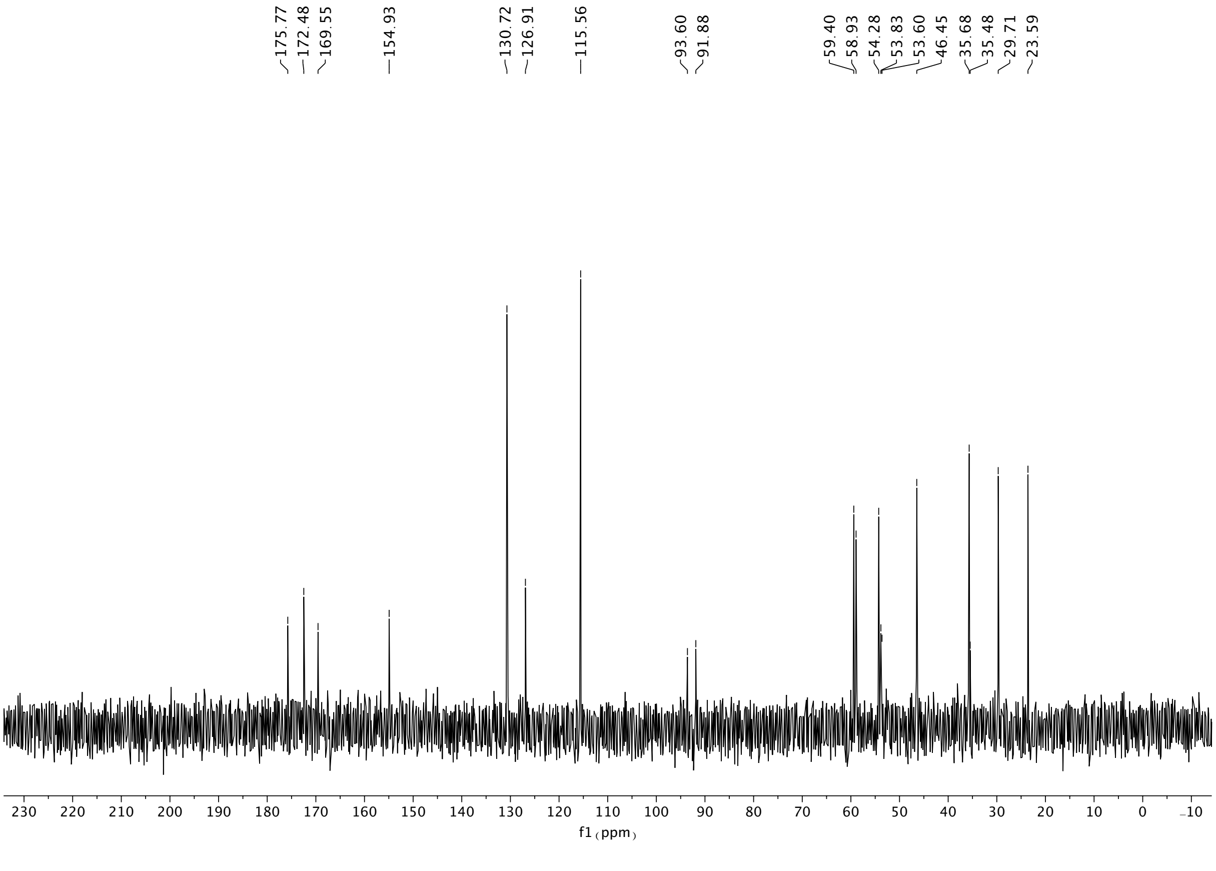

^{1}H and ^{13}C-NMR of H-D-Pro-CyLeu-L-(4*S*)-Flp-NH₂ · TFA (UTS-44):

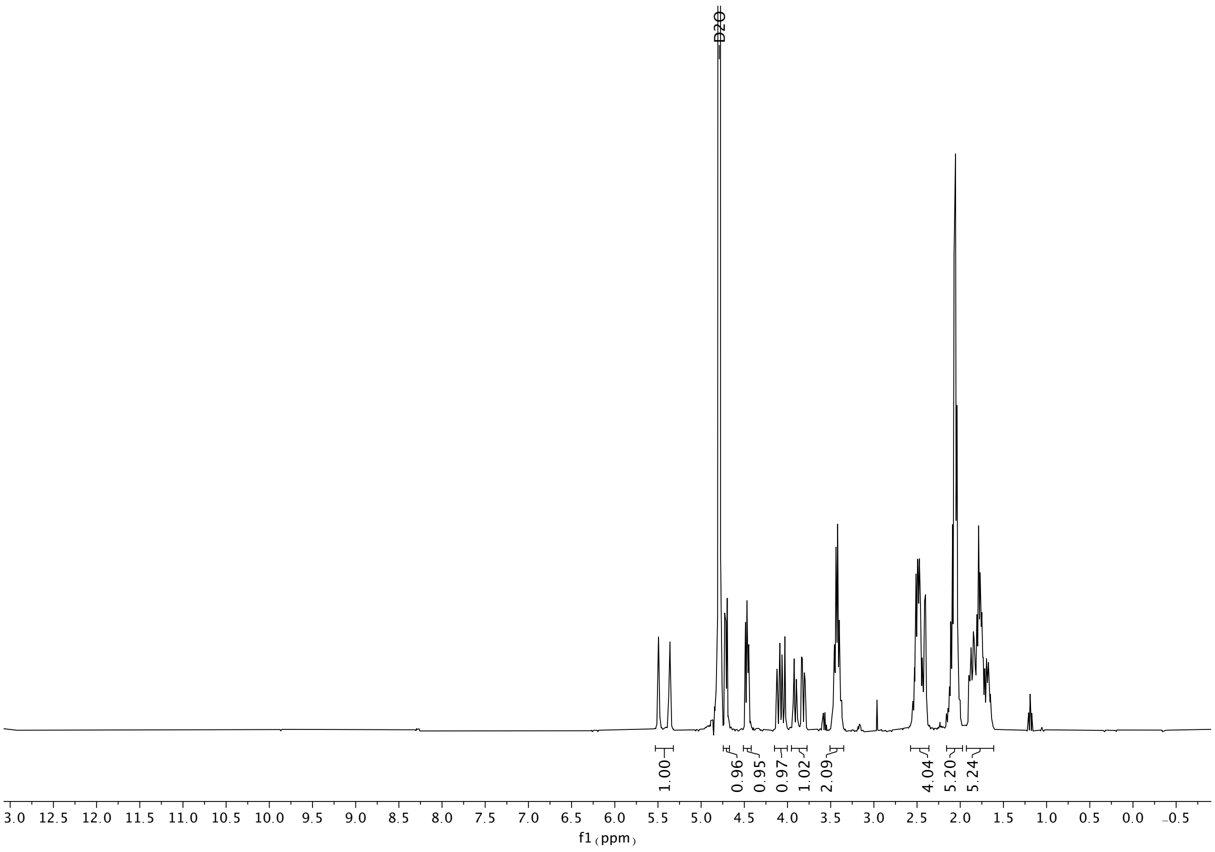

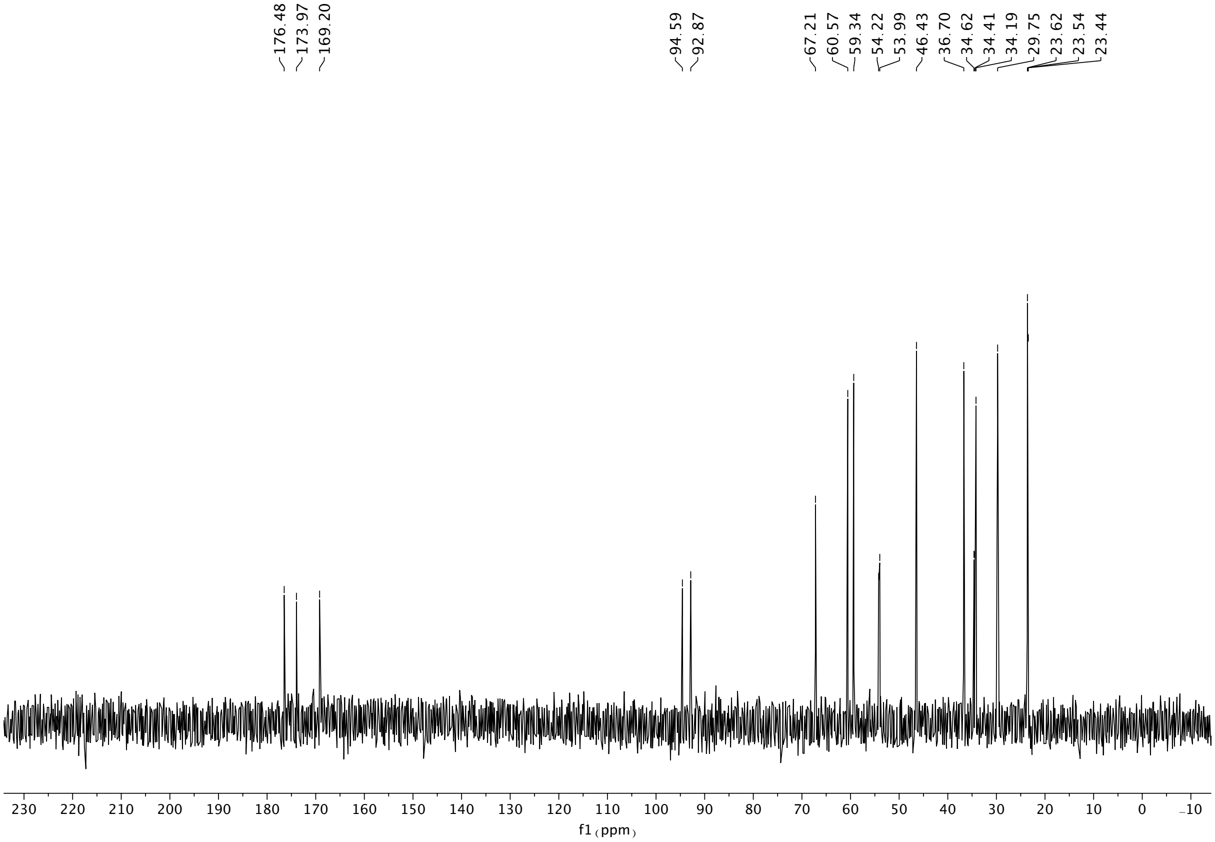

^{1}H and ^{13}C-NMR of H-D-Pro-D-Ind-L-(4*S*)-Flp-NH$_2$ · TFA (UTS-45):

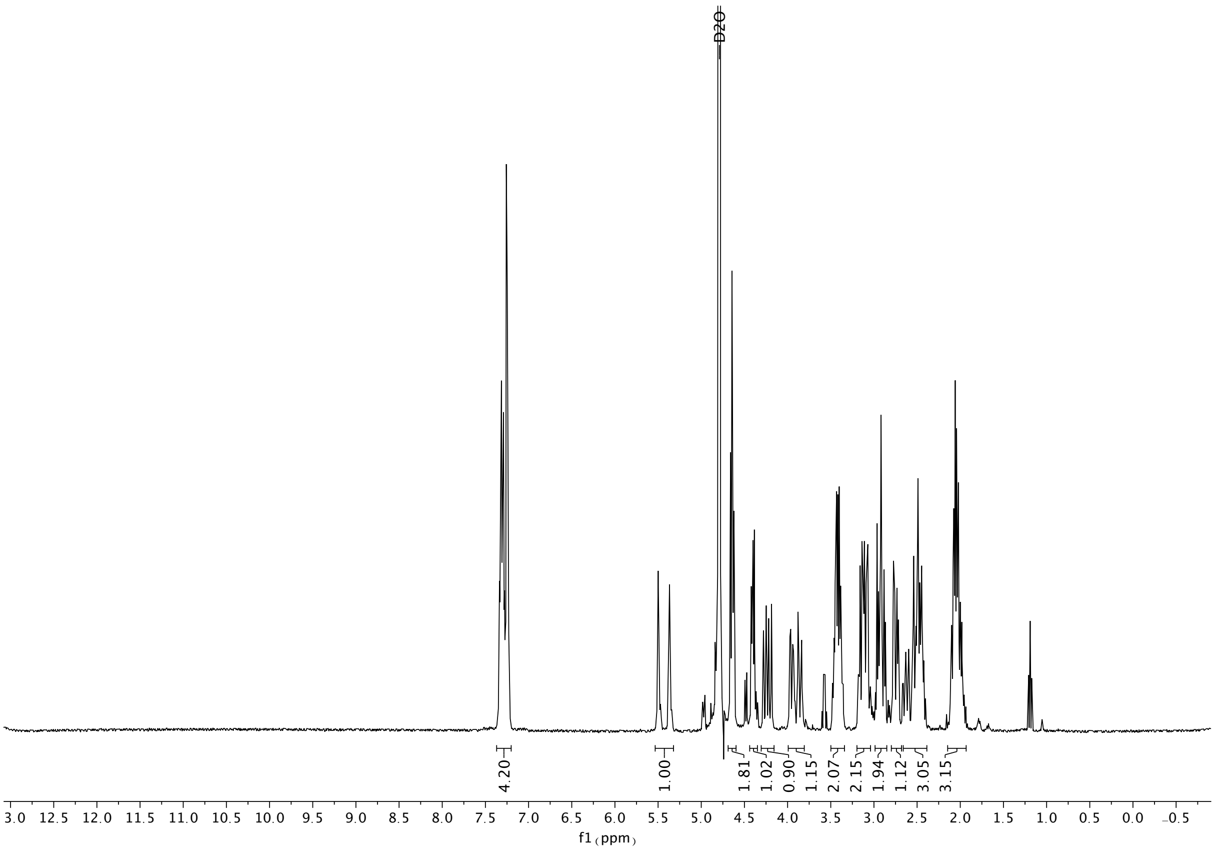

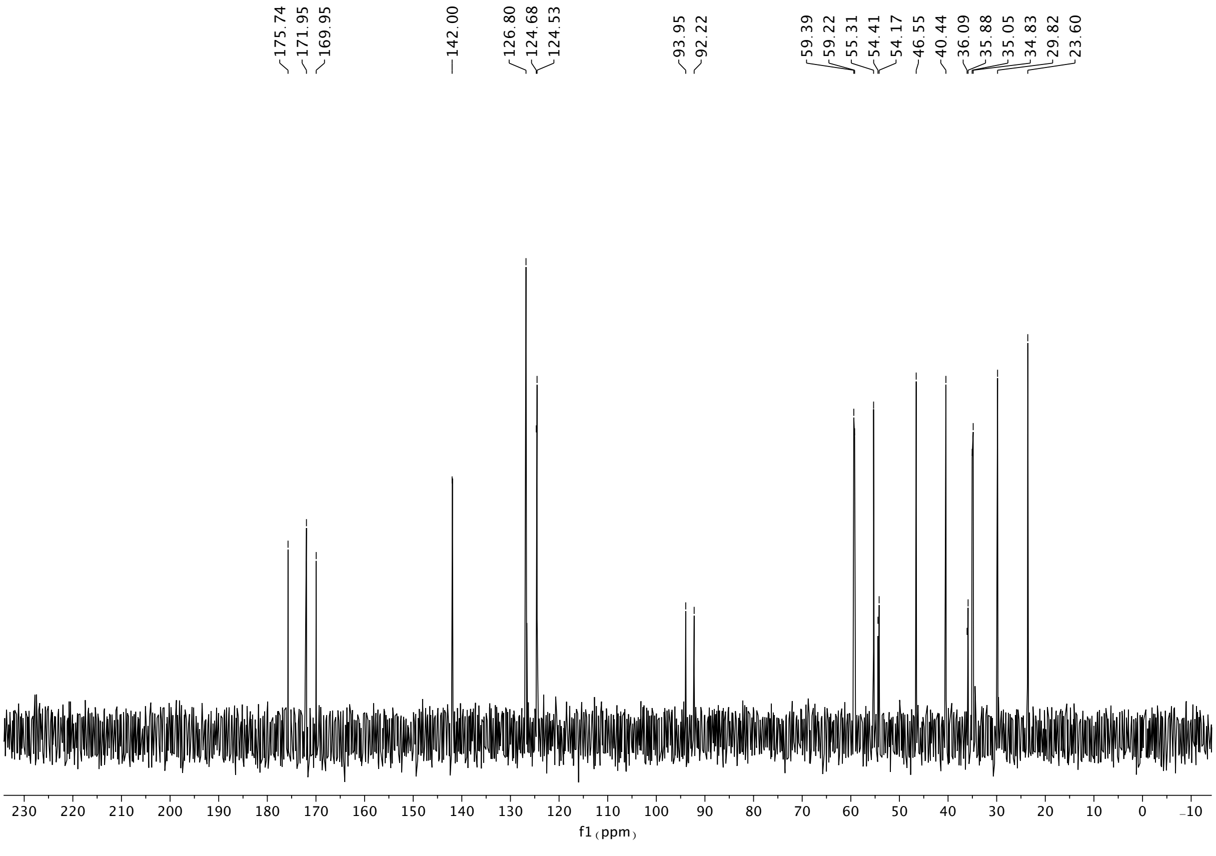

^{1}H and ^{13}C-NMR of H-D-Pro-L-Leu-L-Leu-NH_2 · TFA (UTS-46):

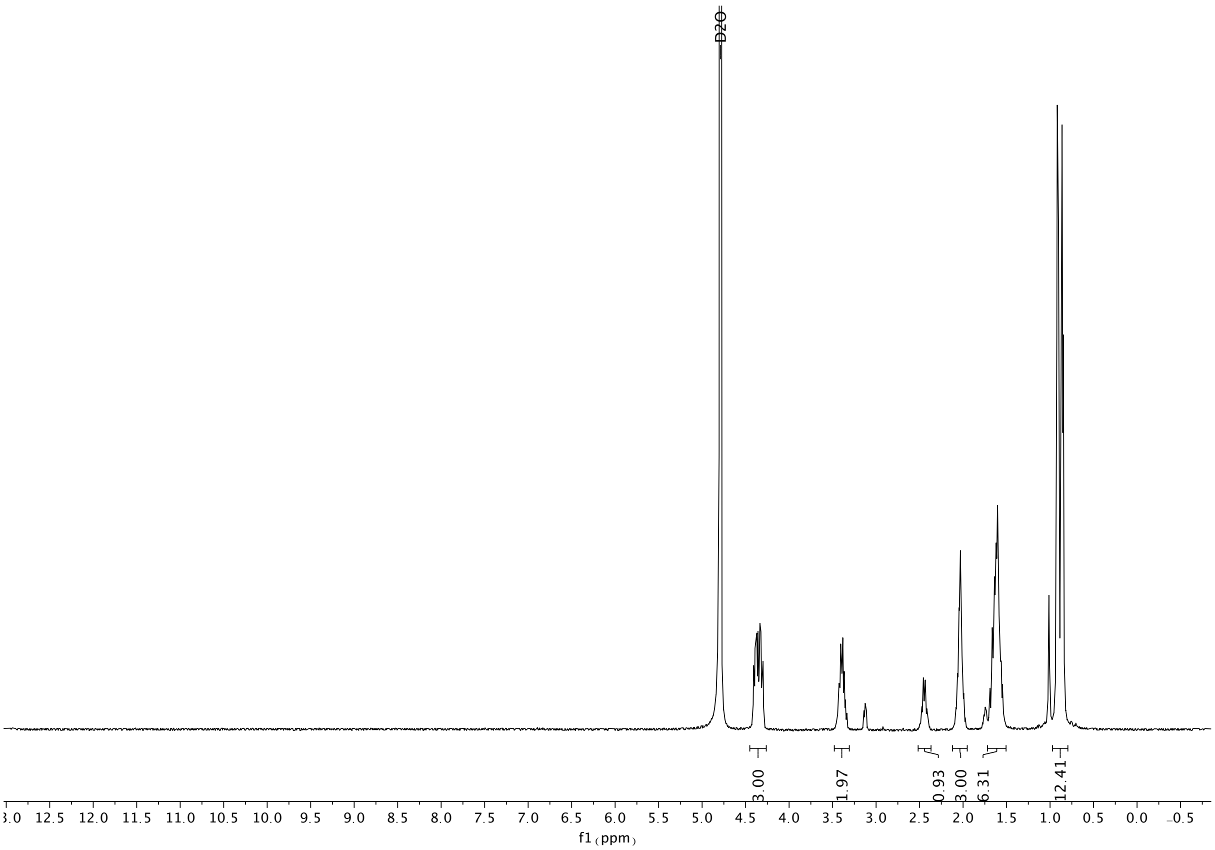

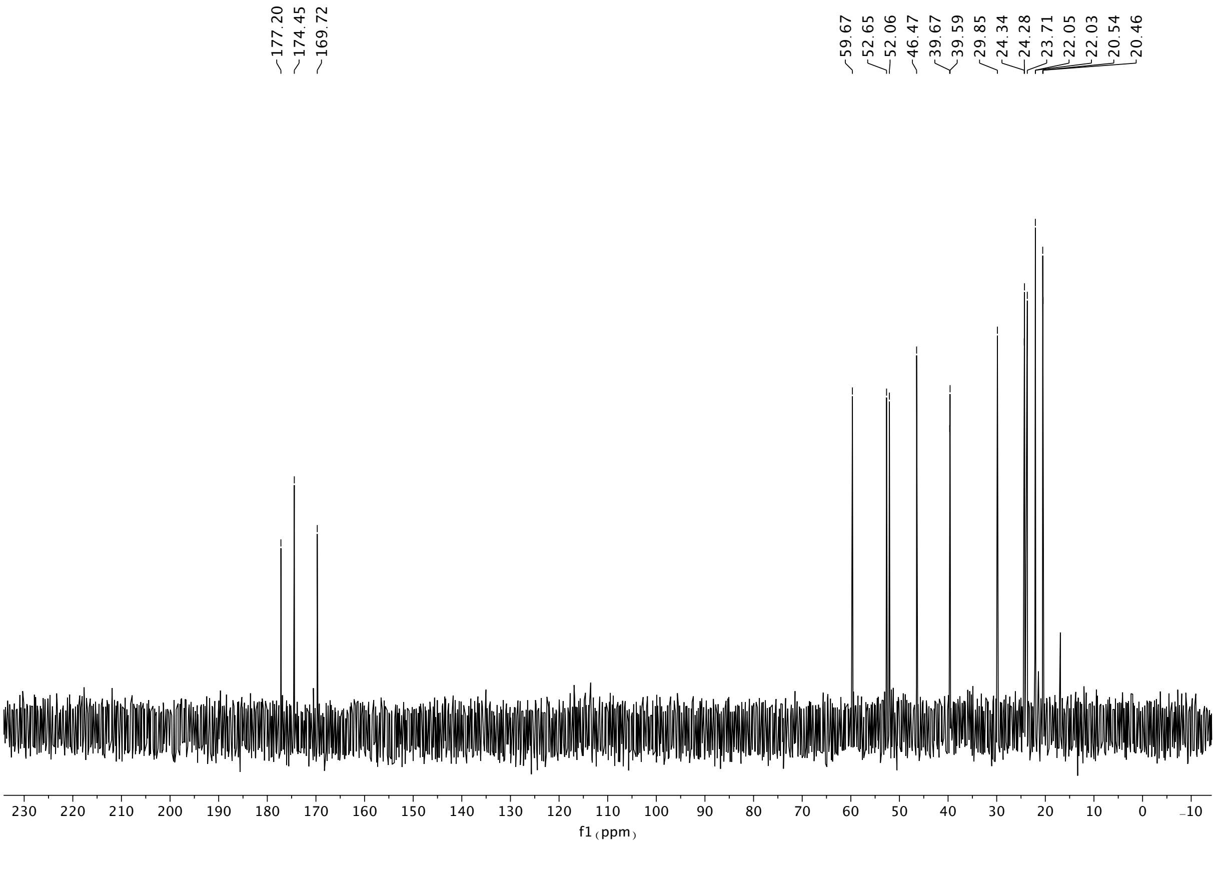

^{1}H and ^{13}C-NMR of H-D-Pro-L-Leu-D-Leu-NH₂ · TFA (UTS-47):

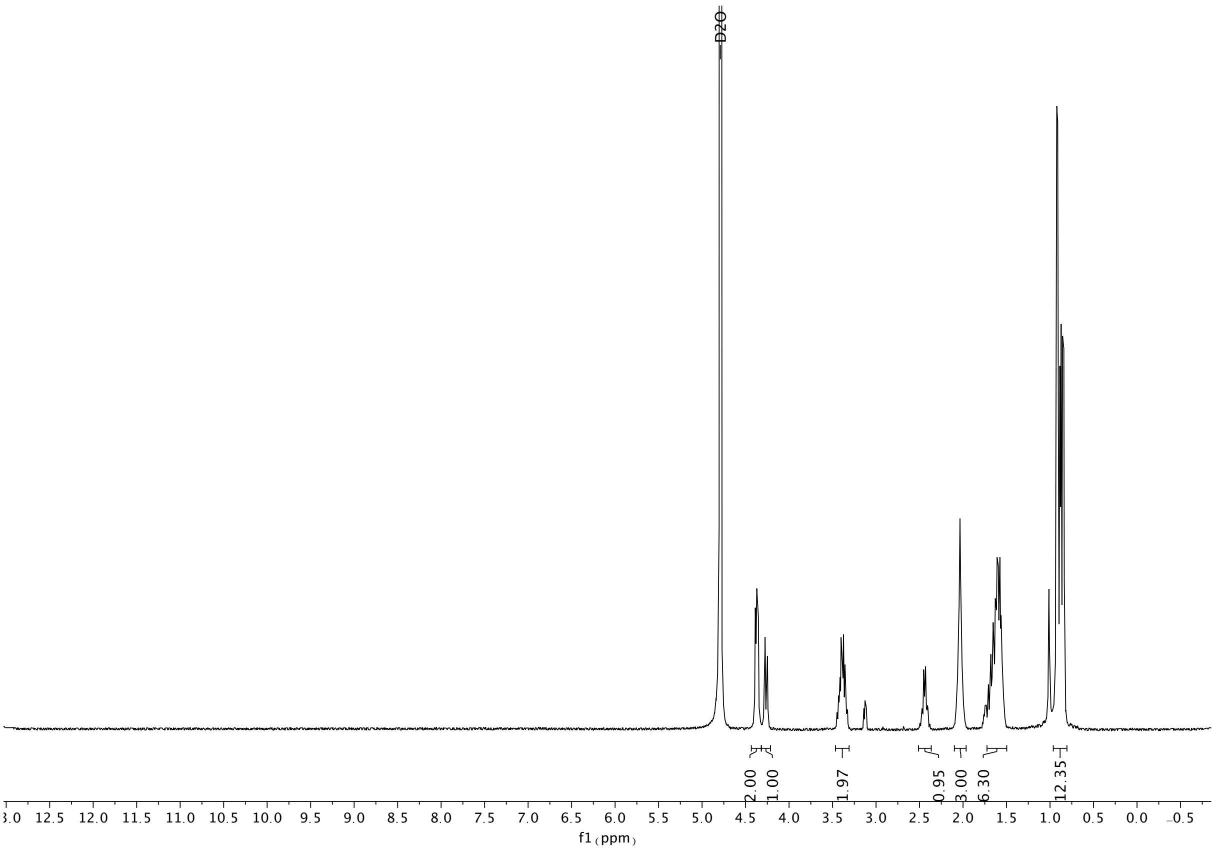

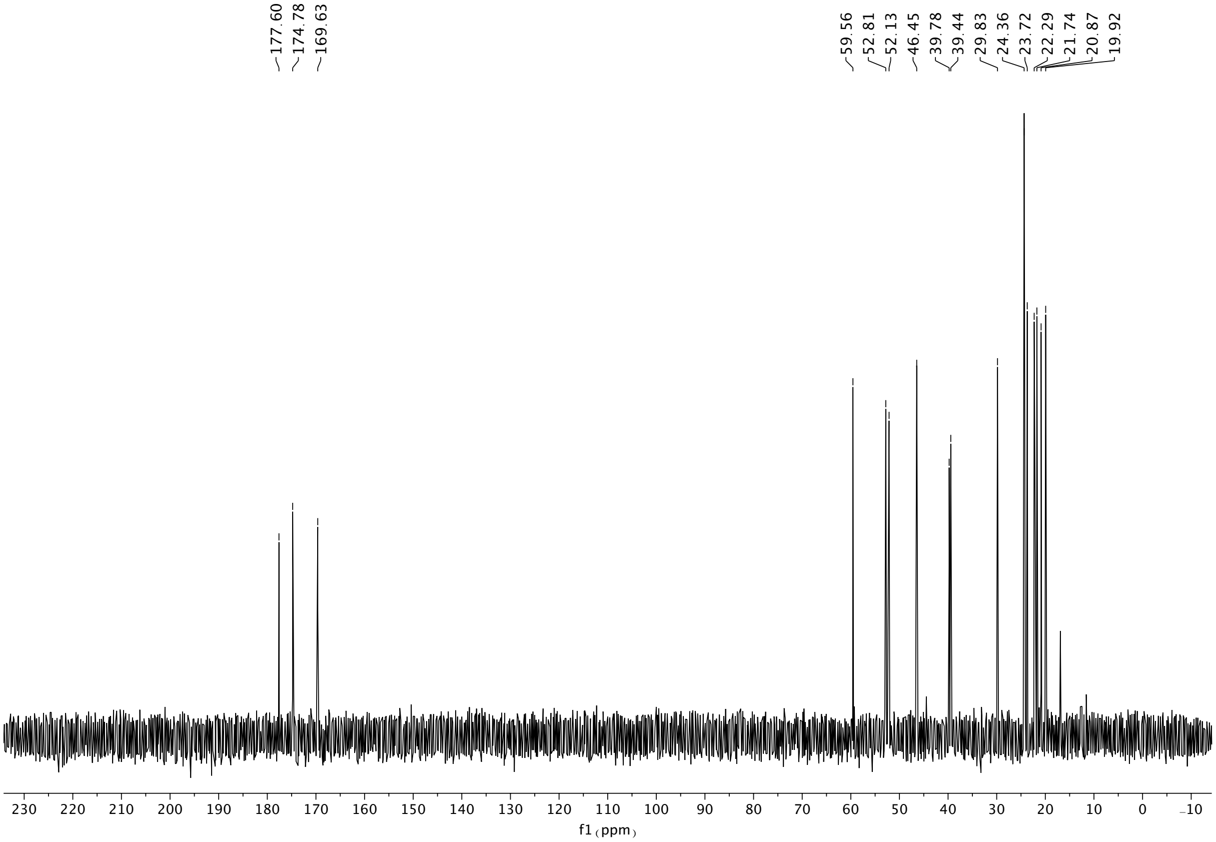

^{1}H and ^{13}C-NMR of H-D-Pro-L-Leu-L-Gln-NH_2 · TFA (UTS-48):

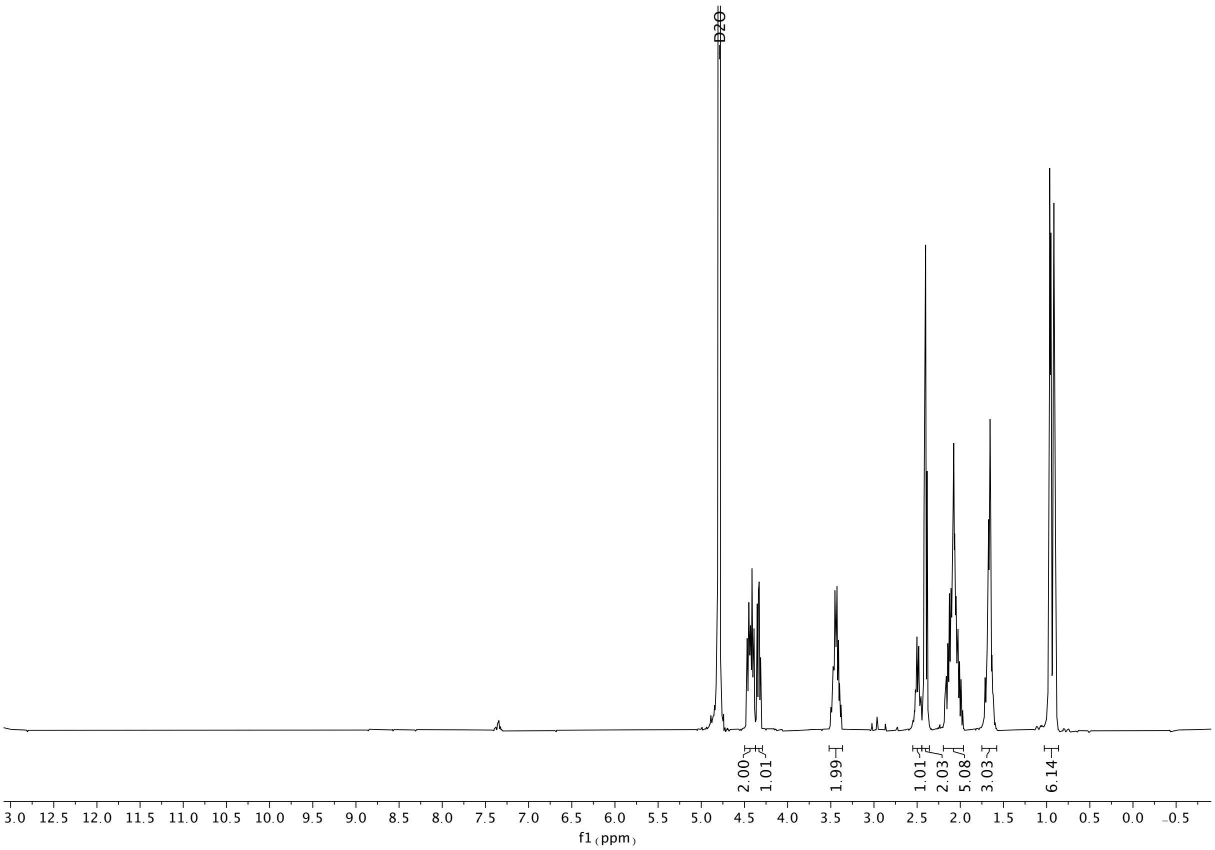

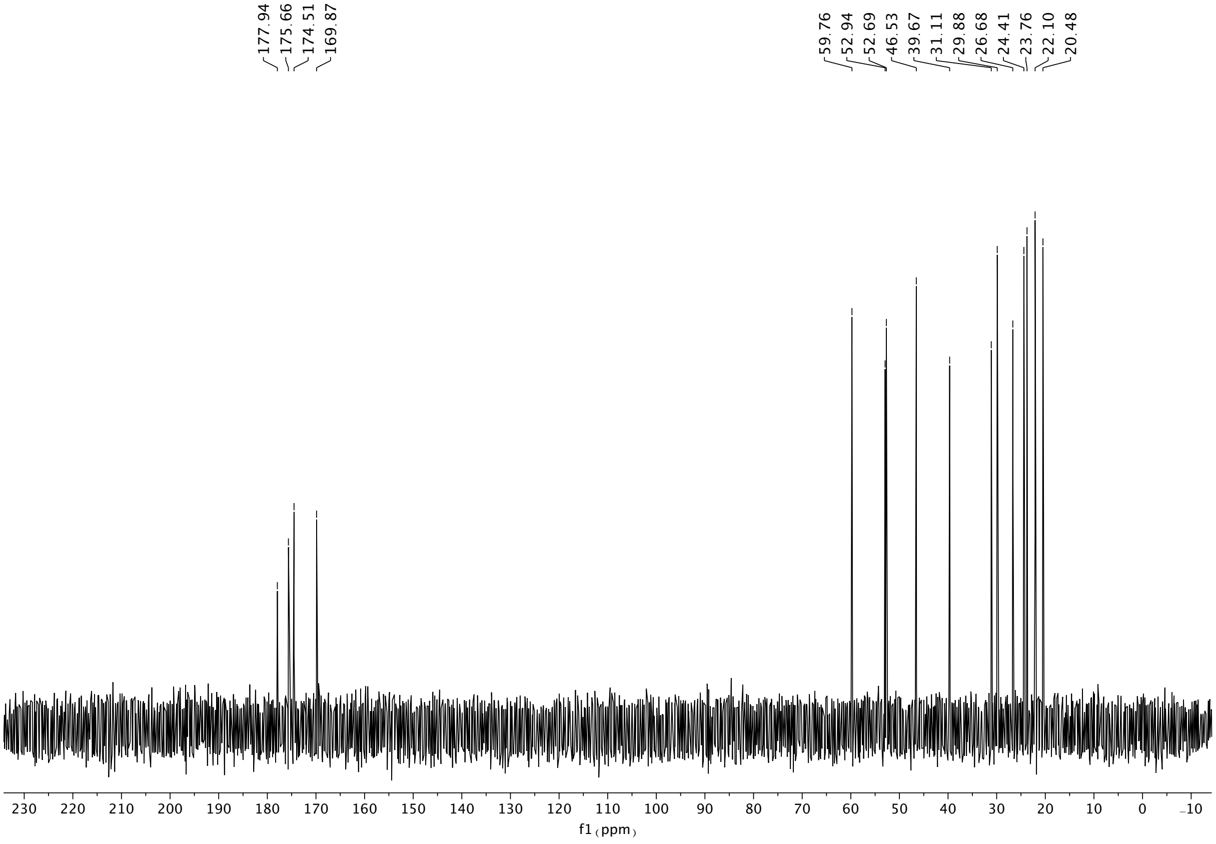

^{1}H and ^{13}C-NMR of H-D-Pro-L-Leu-D-Gln-NH_2 · TFA (UTS-49):

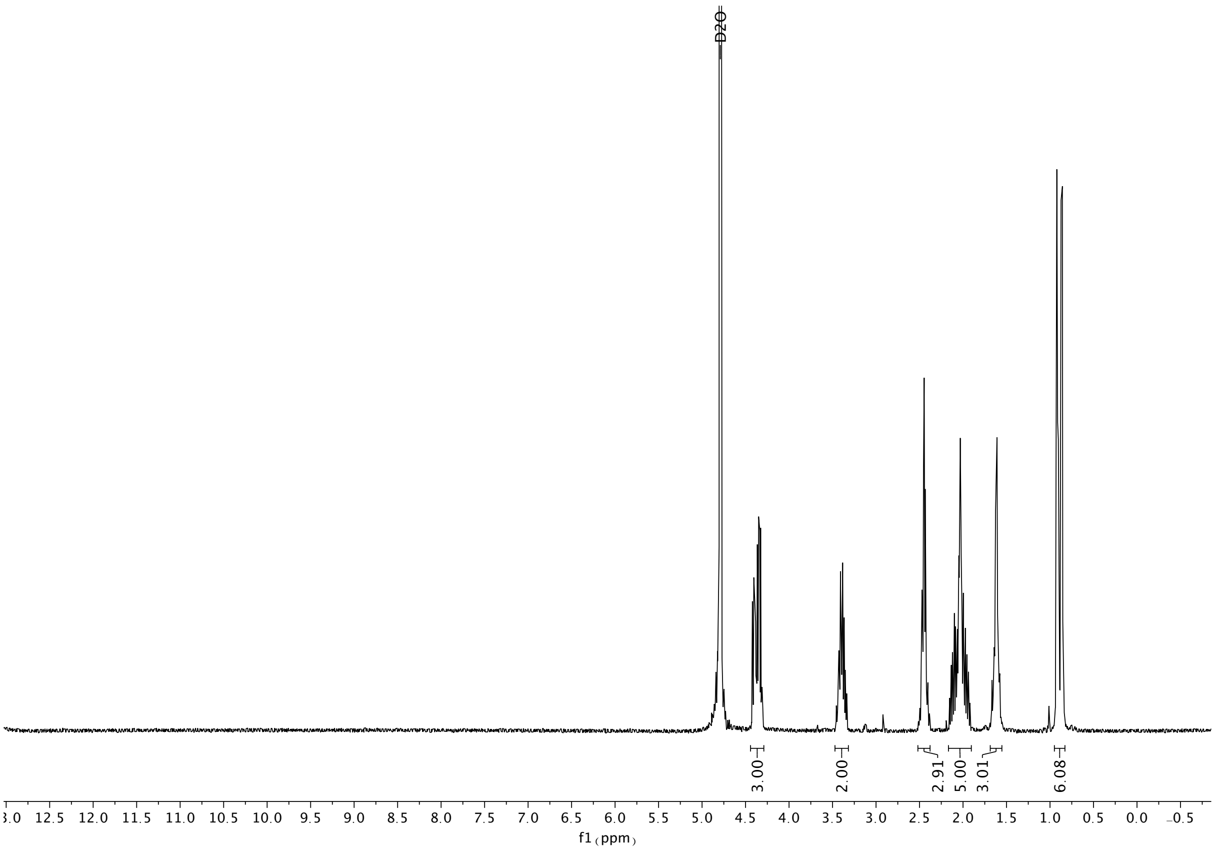

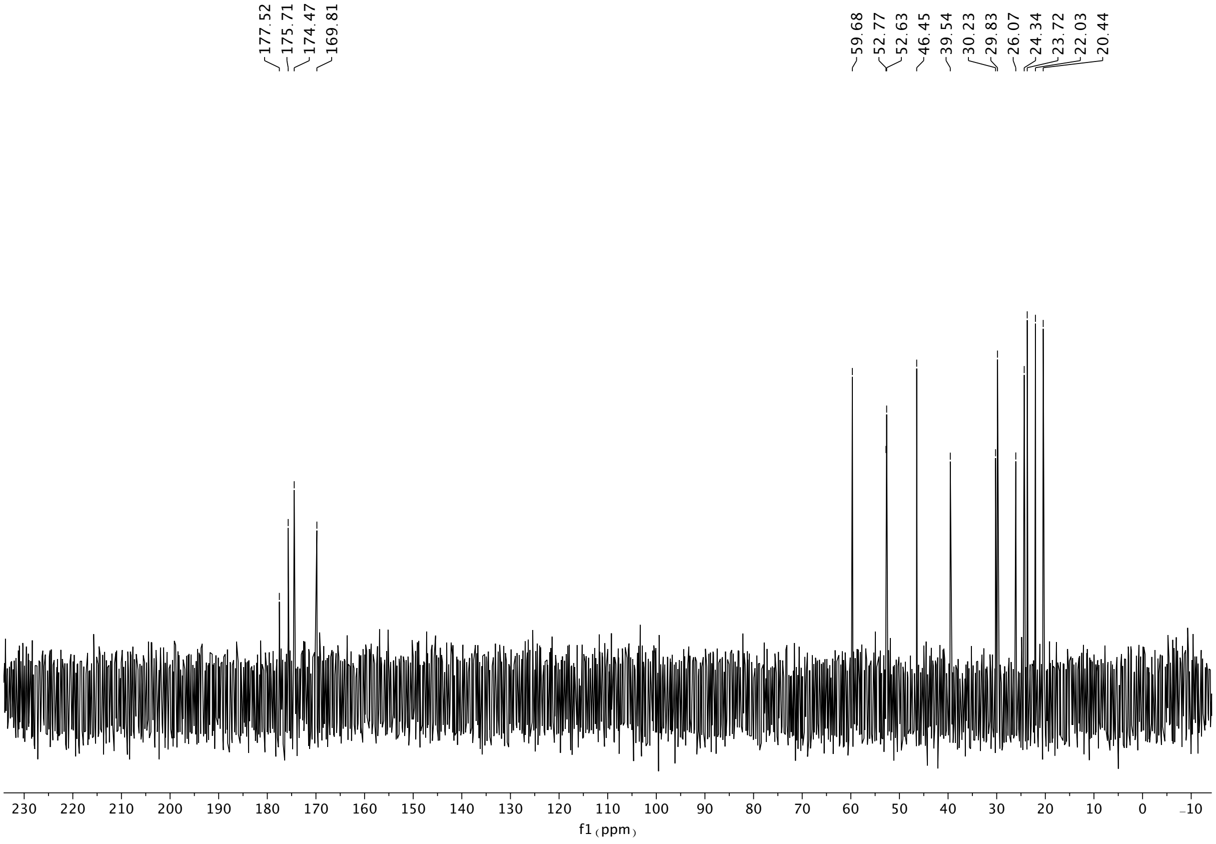

^{1}H and ^{13}C-NMR of H-D-Pro-L-Leu-L-Glu-NH_2 · TFA (UTS-50):

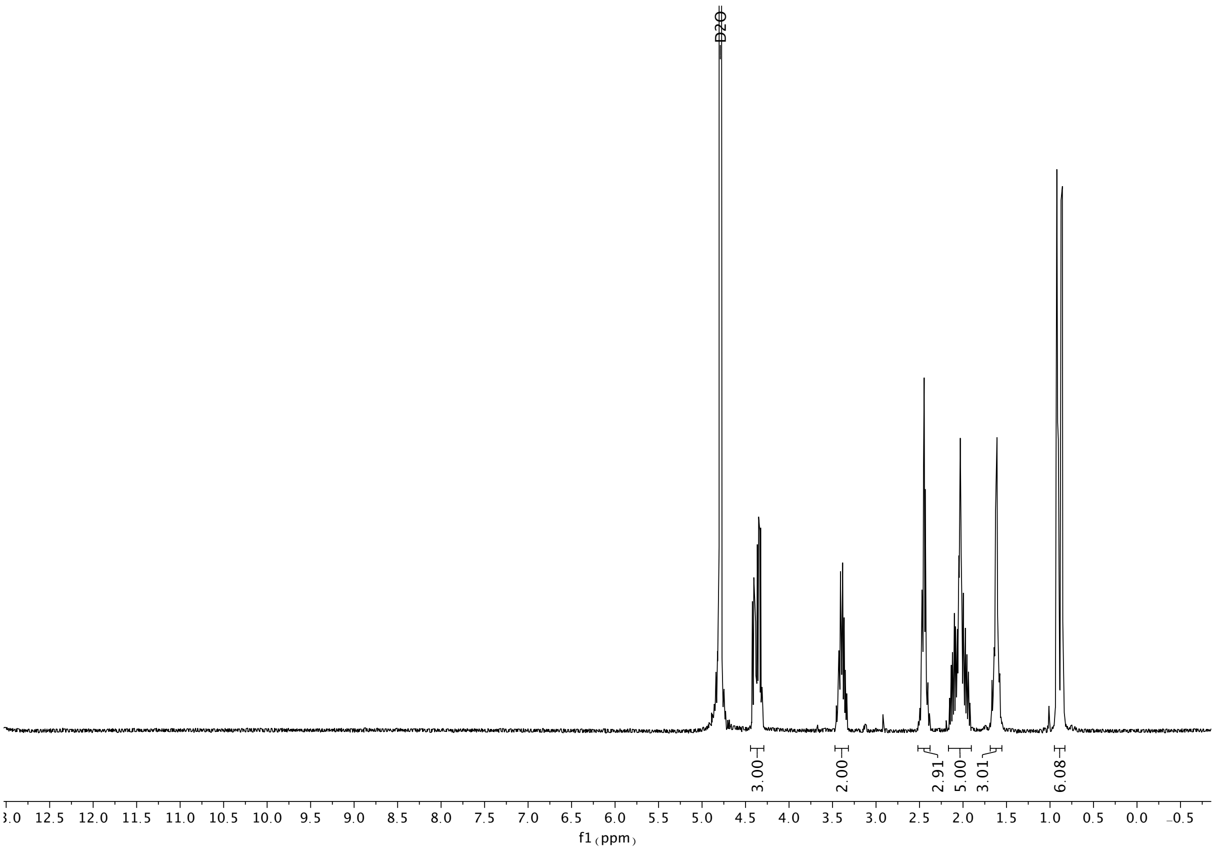

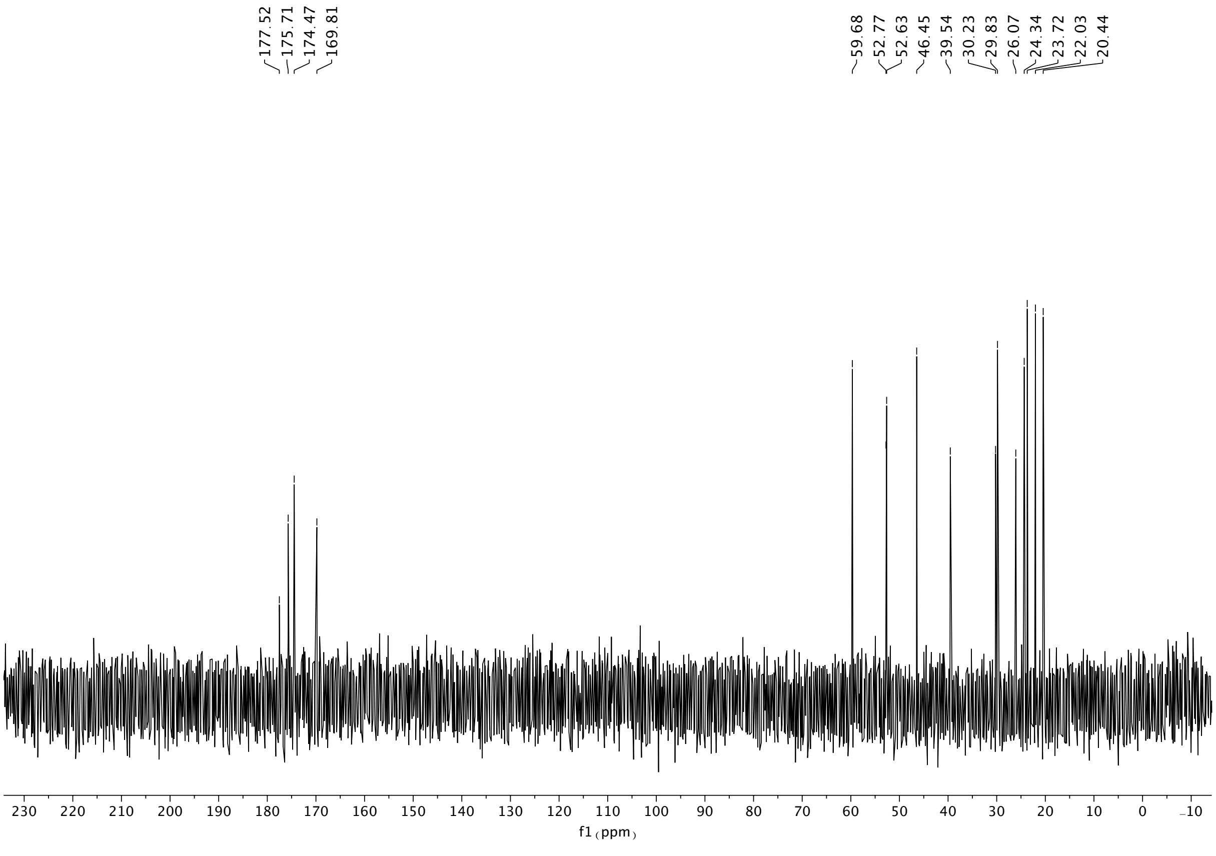

^{1}H and ^{13}C-NMR of H-D-Pro-L-Leu-D-Glu-NH₂ · TFA (UTS-51):

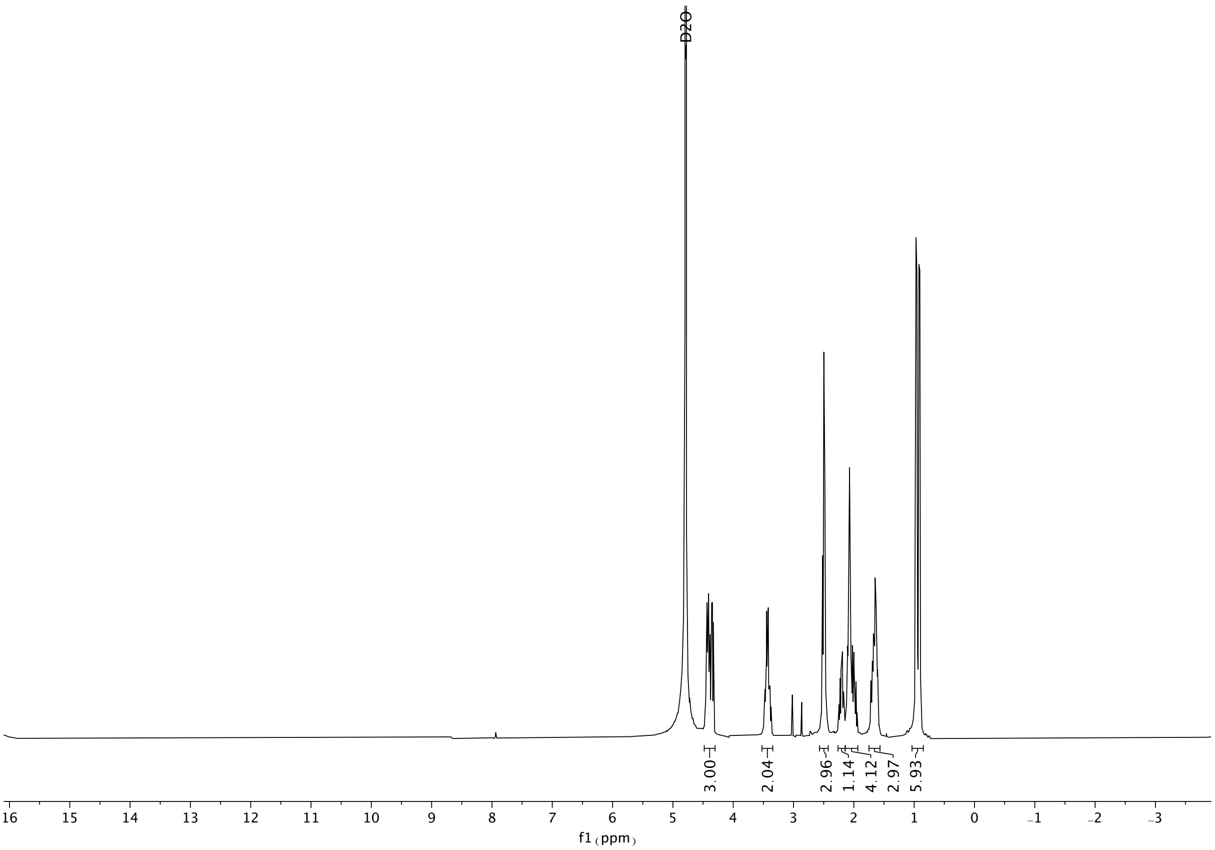

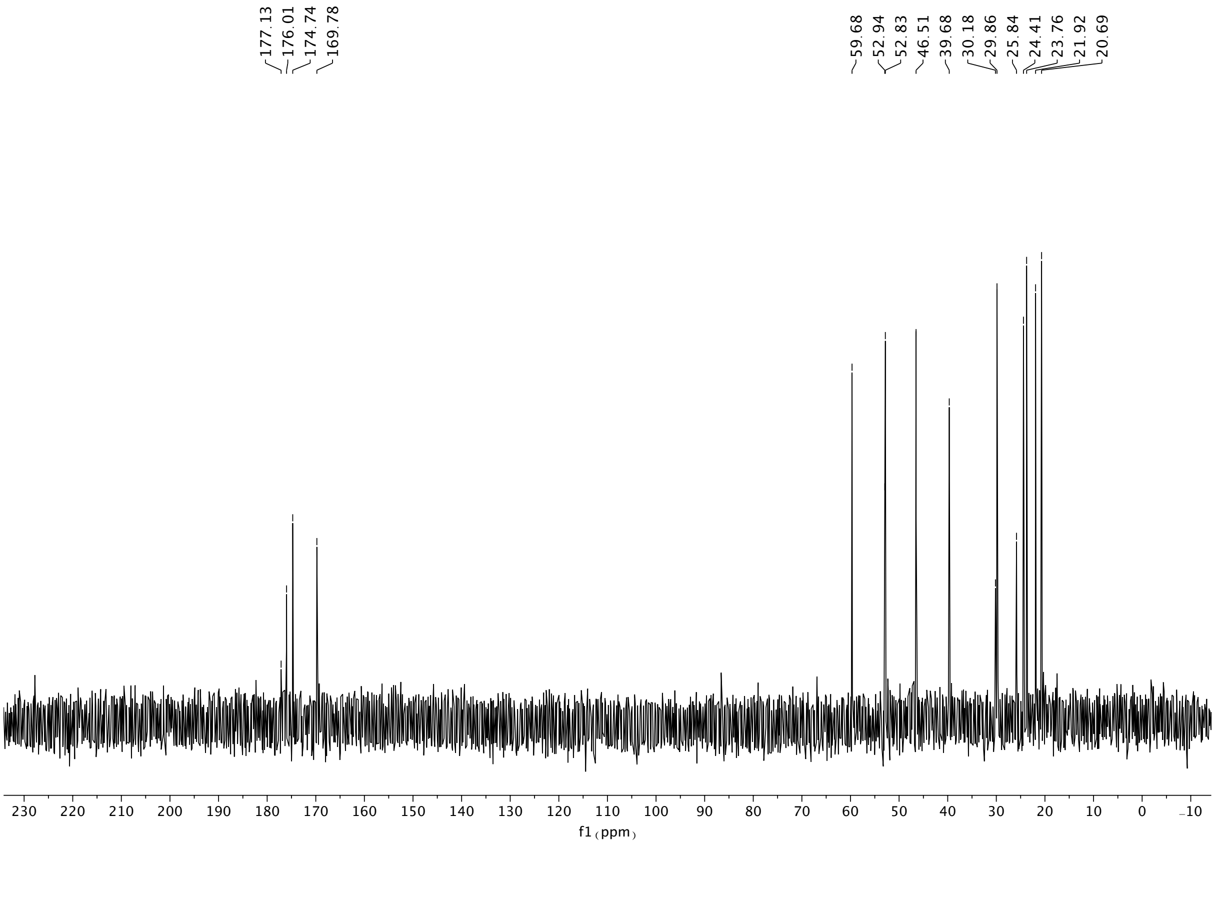

^{1}H and ^{13}C-NMR of H-D-Pro-L-Leu-L-Tyr-NH2 · TFA (UTS-52):

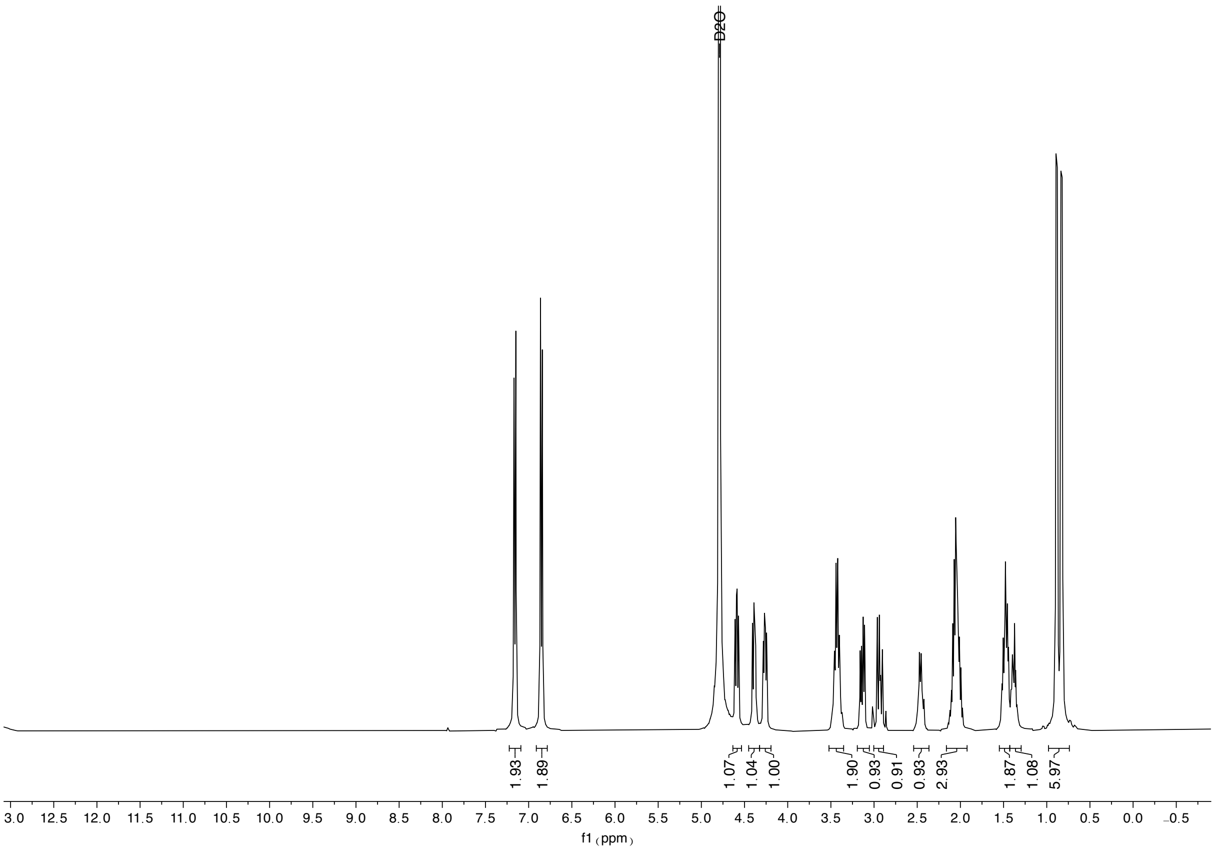

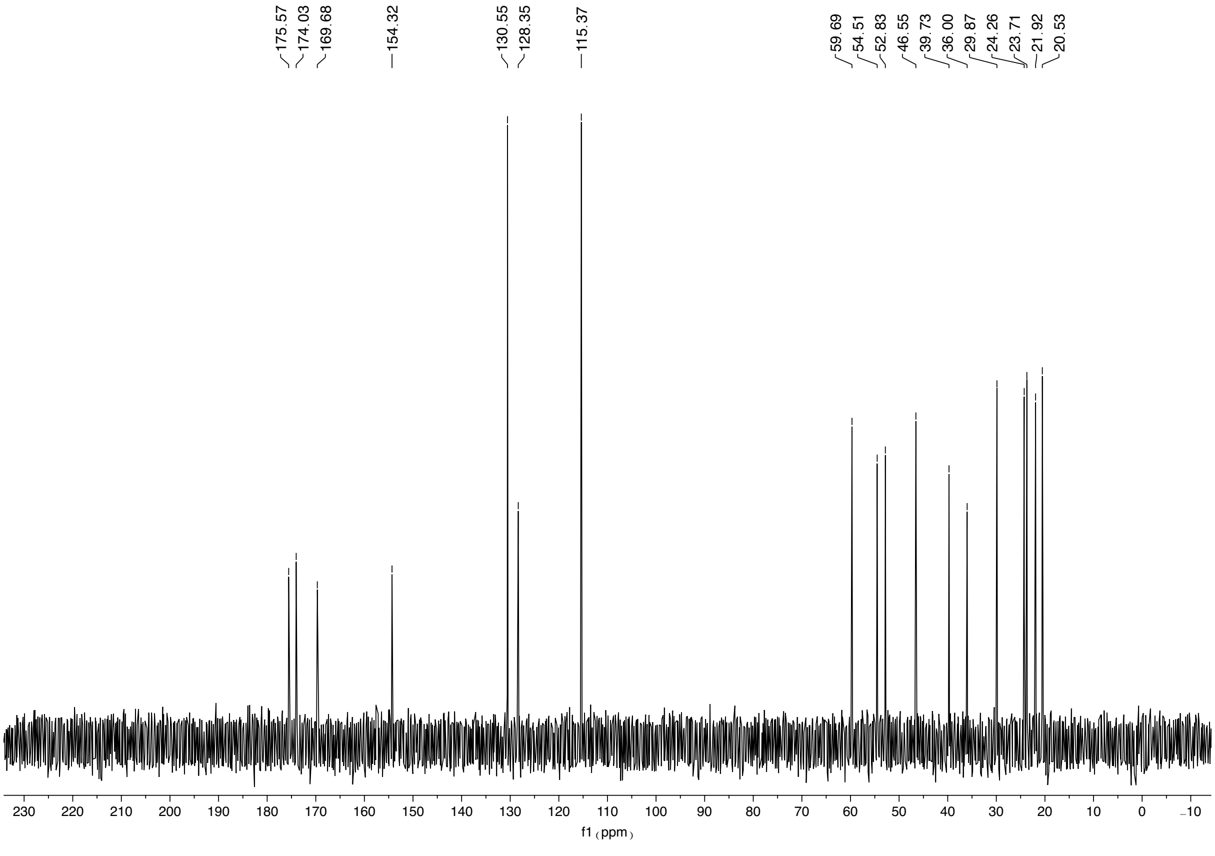

^{1}H and ^{13}C-NMR of H-D-Pro-L-Leu-D-Tyr-NH_2 · TFA (UTS-53):

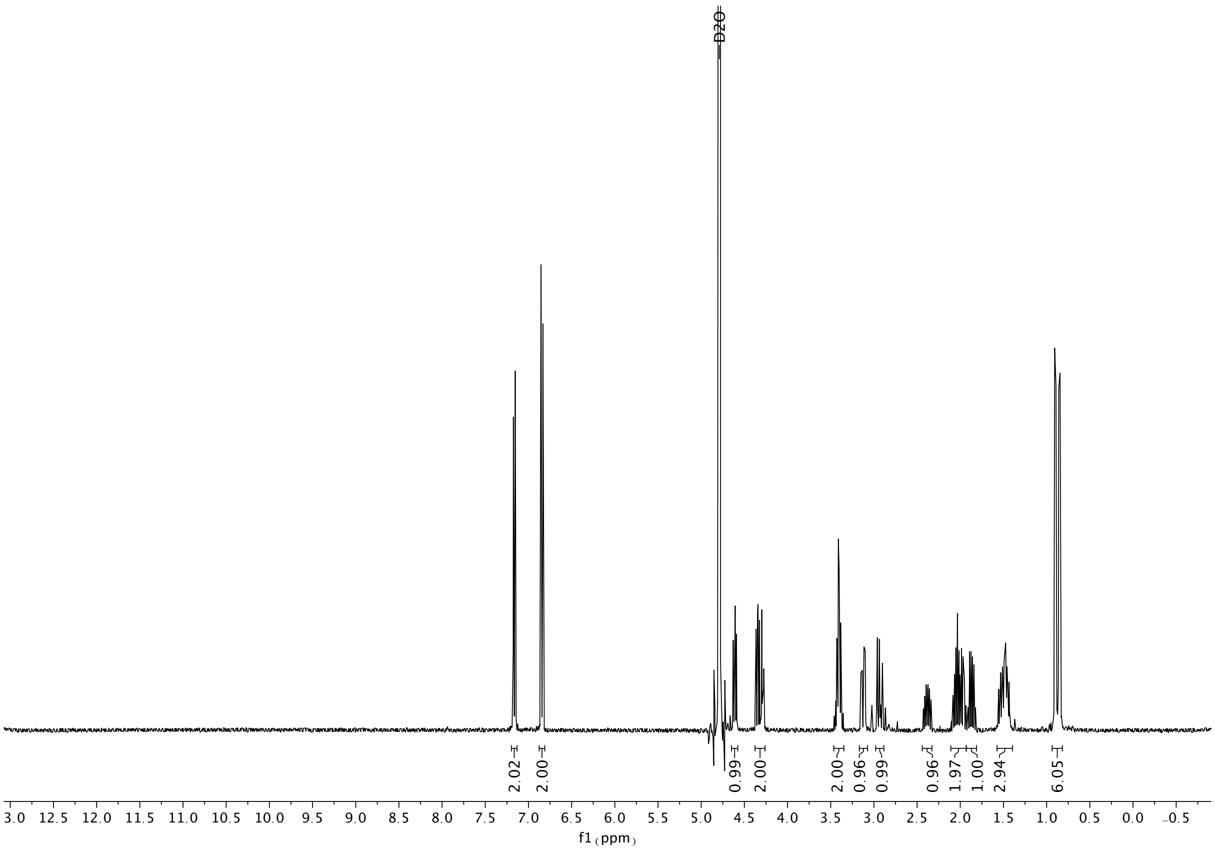

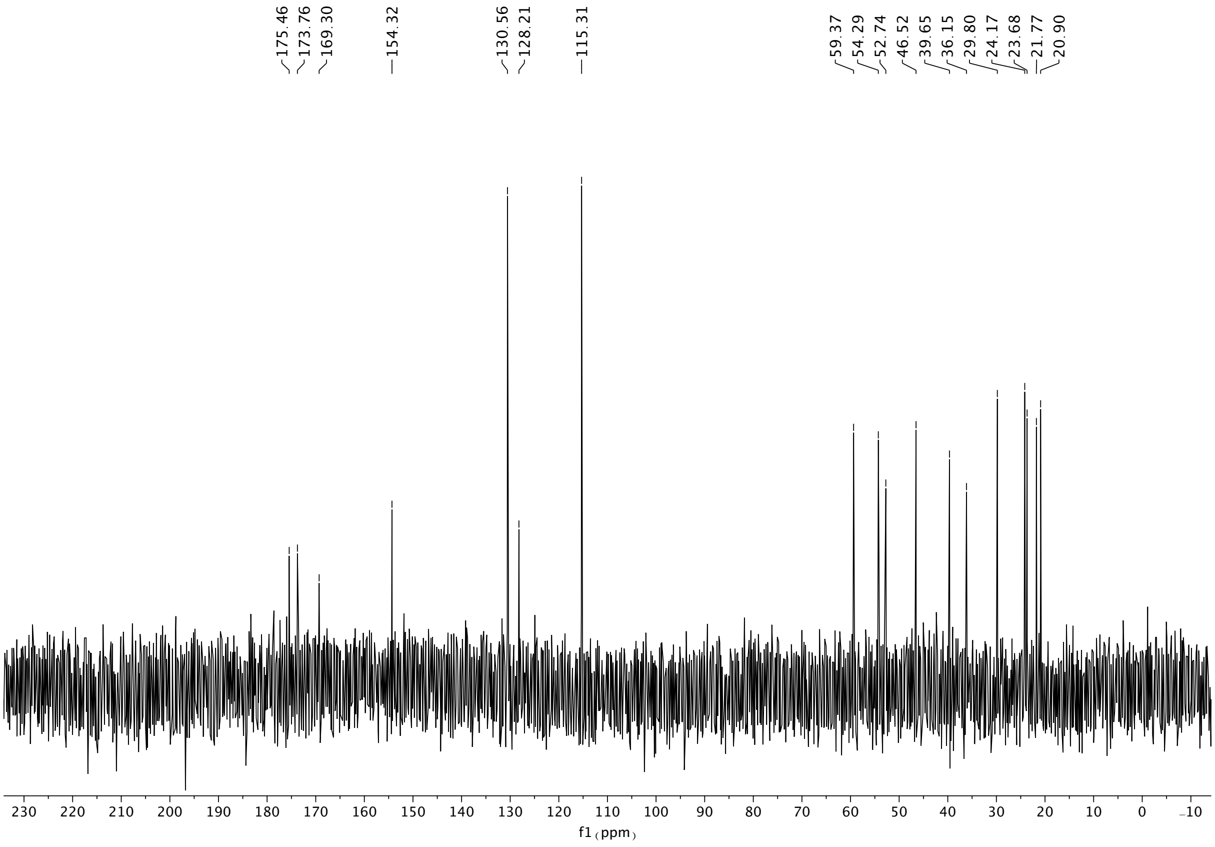

^{1}H and ^{13}C-NMR of H-D-Pro-L-Leu-CyLeu-NH$_2$ · TFA (UTS-54):

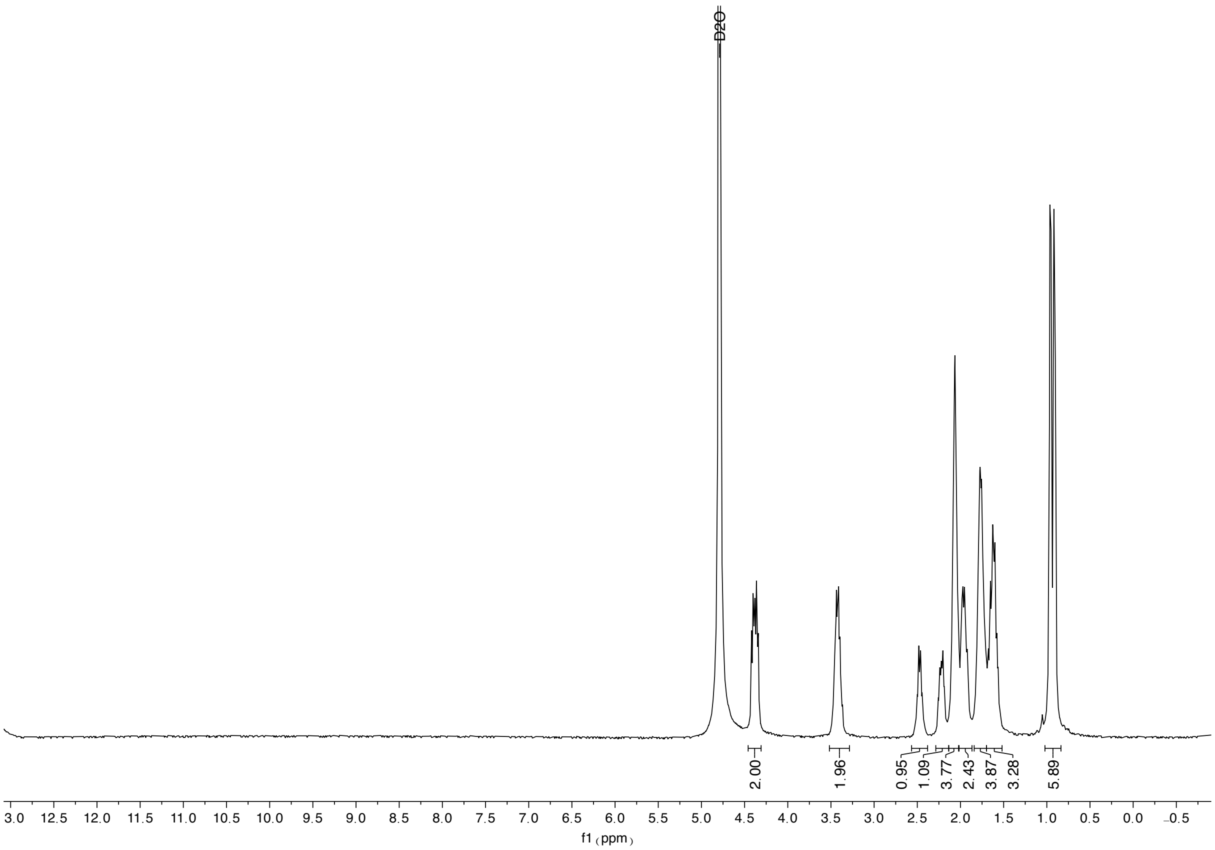

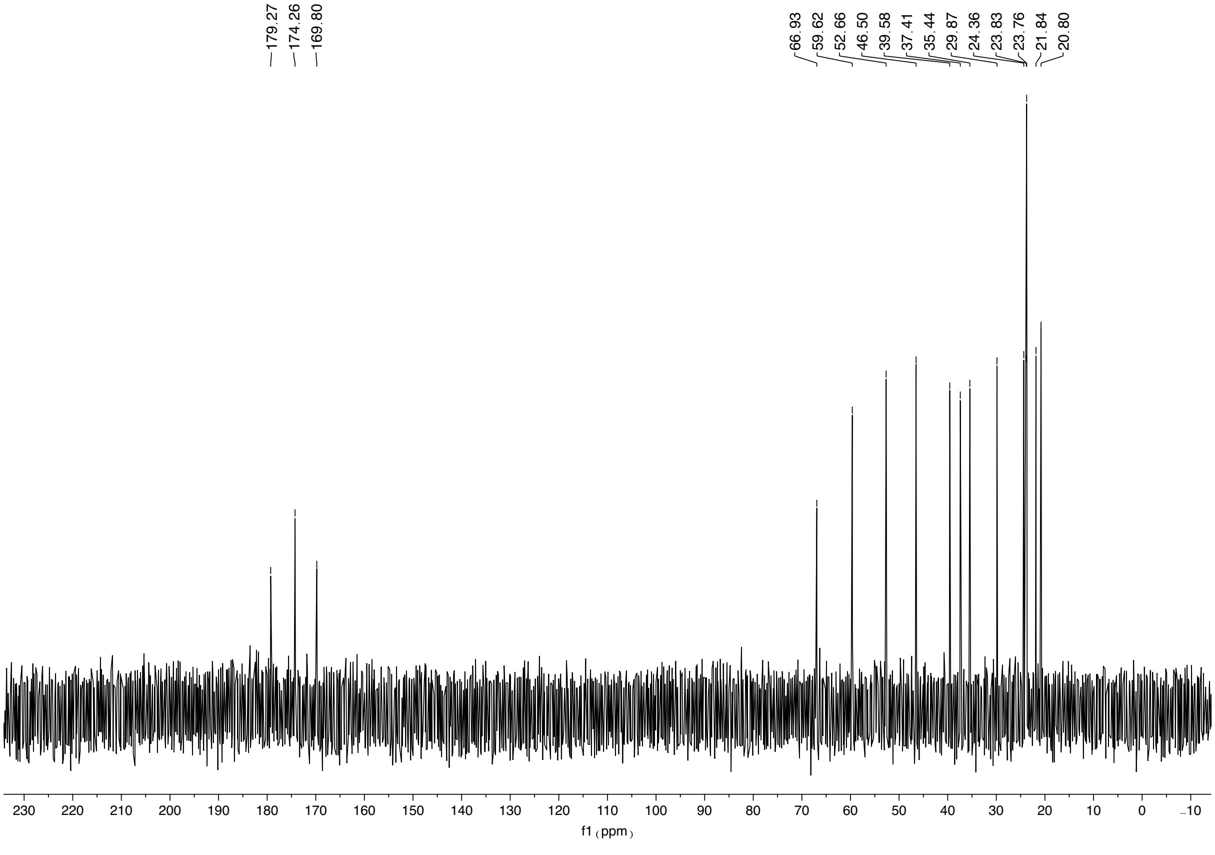

^{1}H and ^{13}C-NMR of H-D-Pro-L-Leu-Abz-NH$_2$ · TFA (UTS-55):

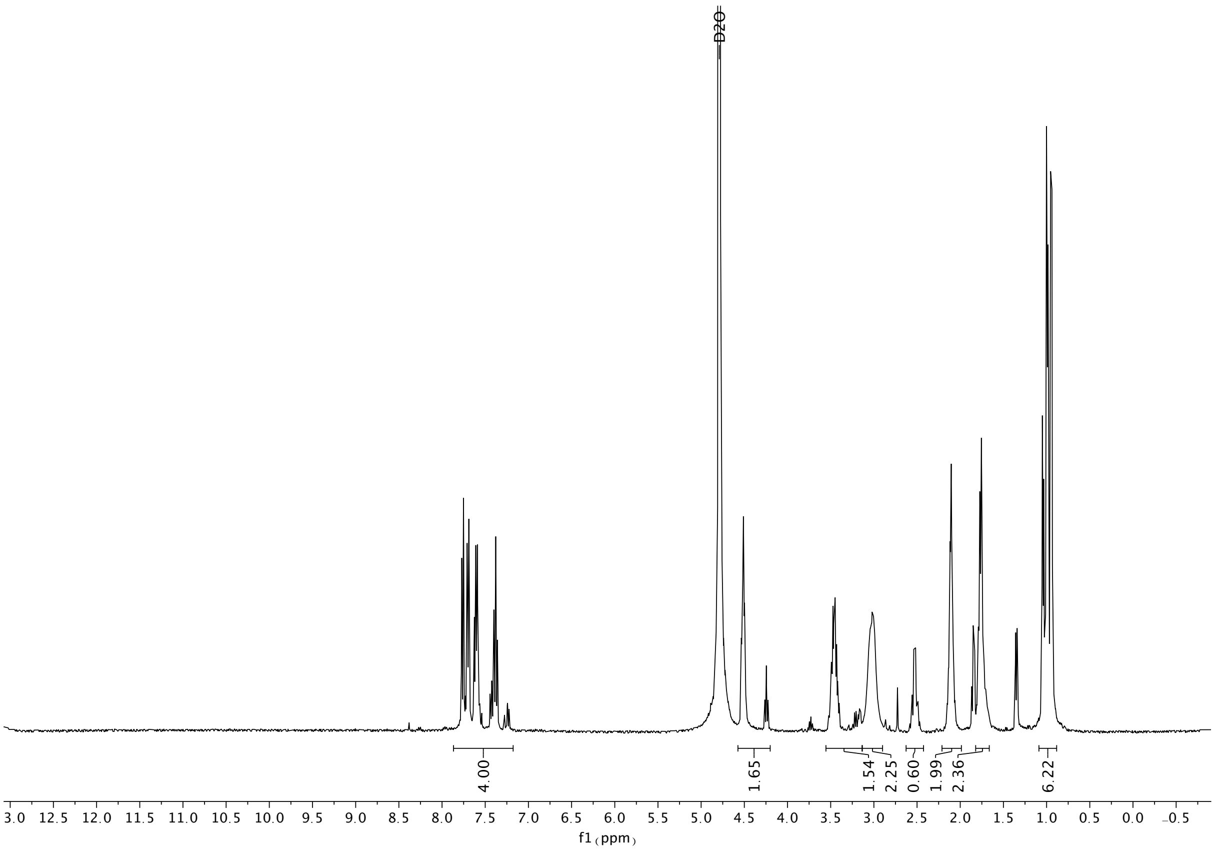

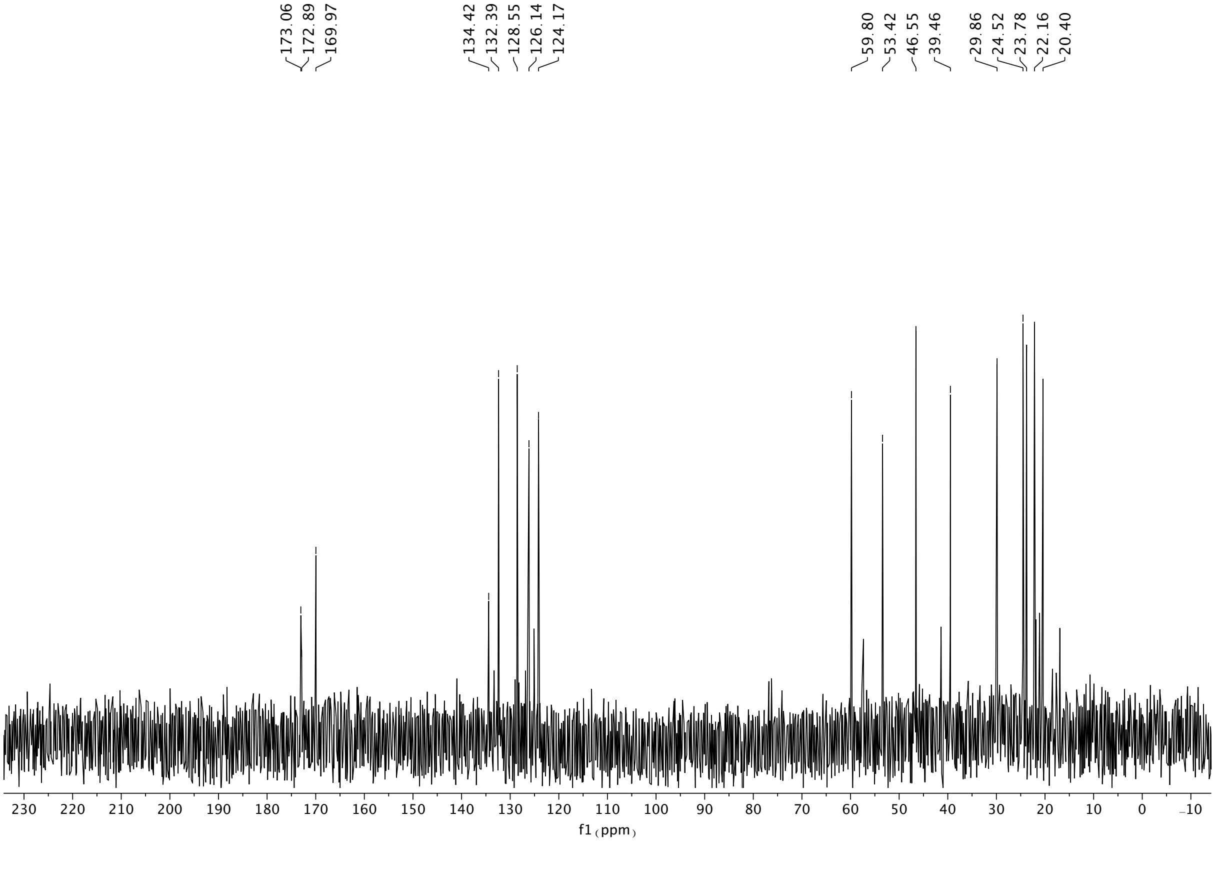

^{1}H and ^{13}C-NMR of H-D-Pro-L-Leu-D-Ind-NH_2 · TFA (UTS-56):

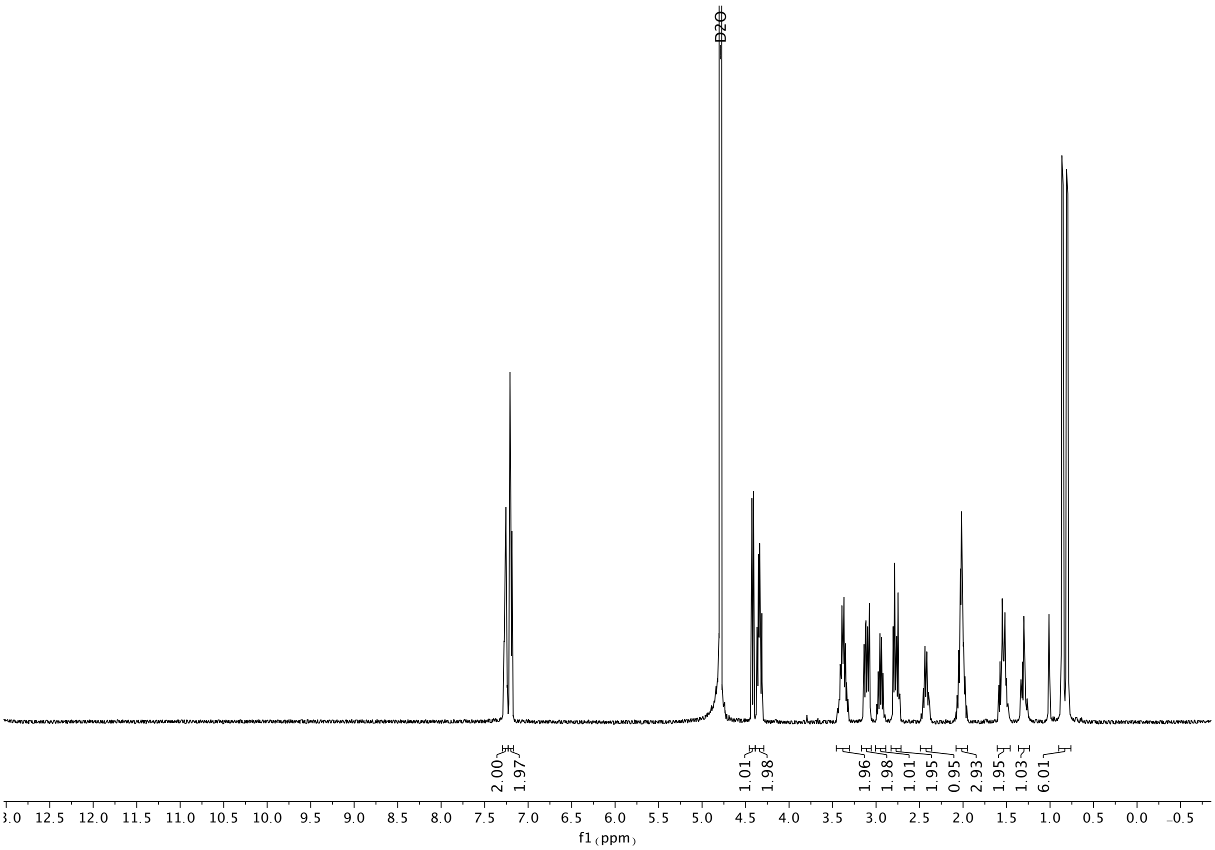

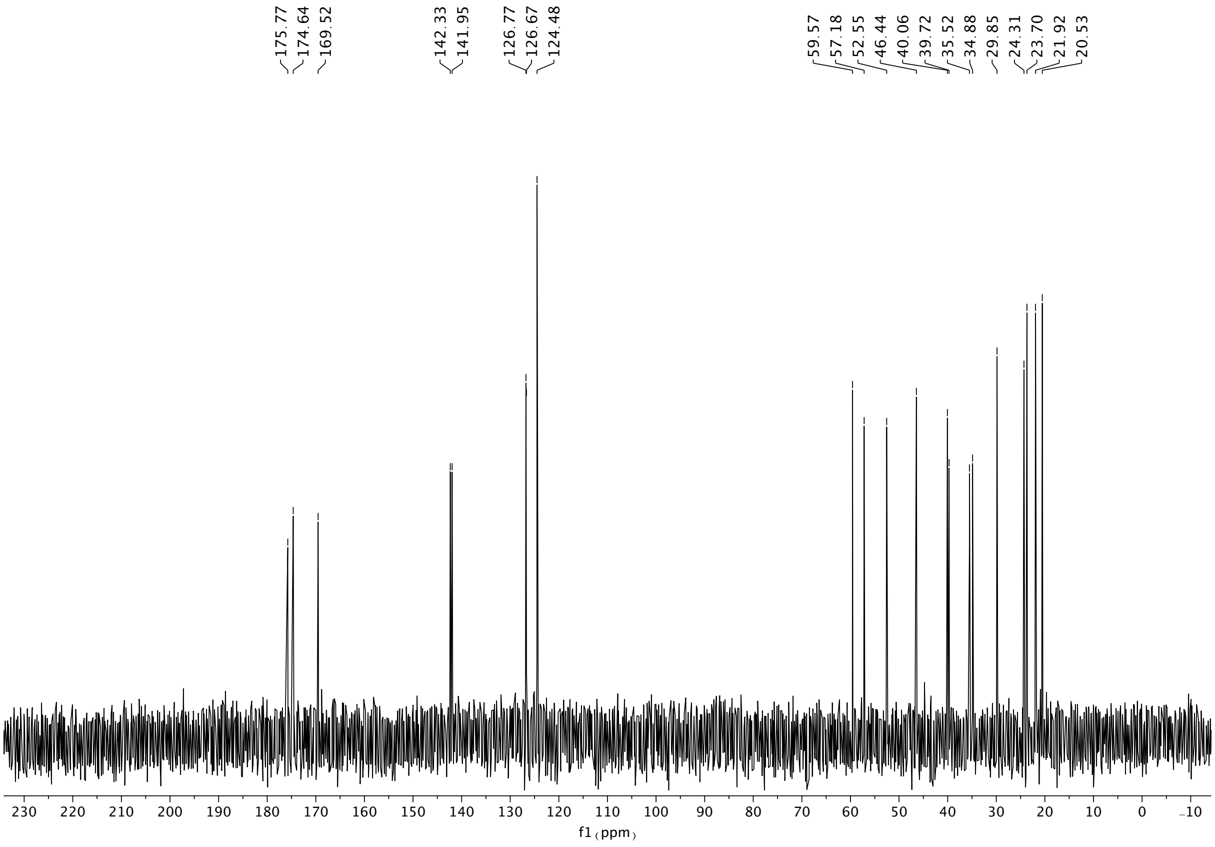

^{1}H and ^{13}C-NMR of H-D-Pro-D-Leu-L-Leu-NH_2 · TFA (UTS-57):

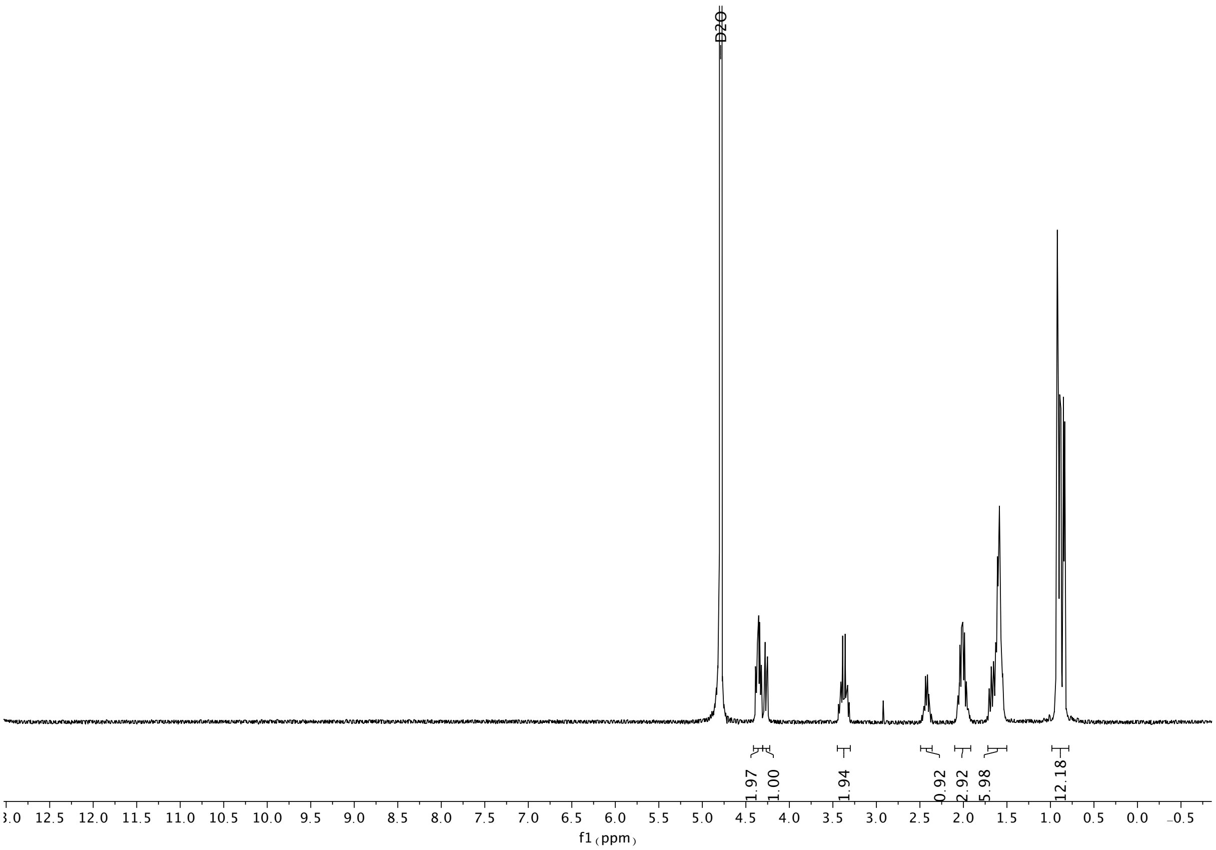

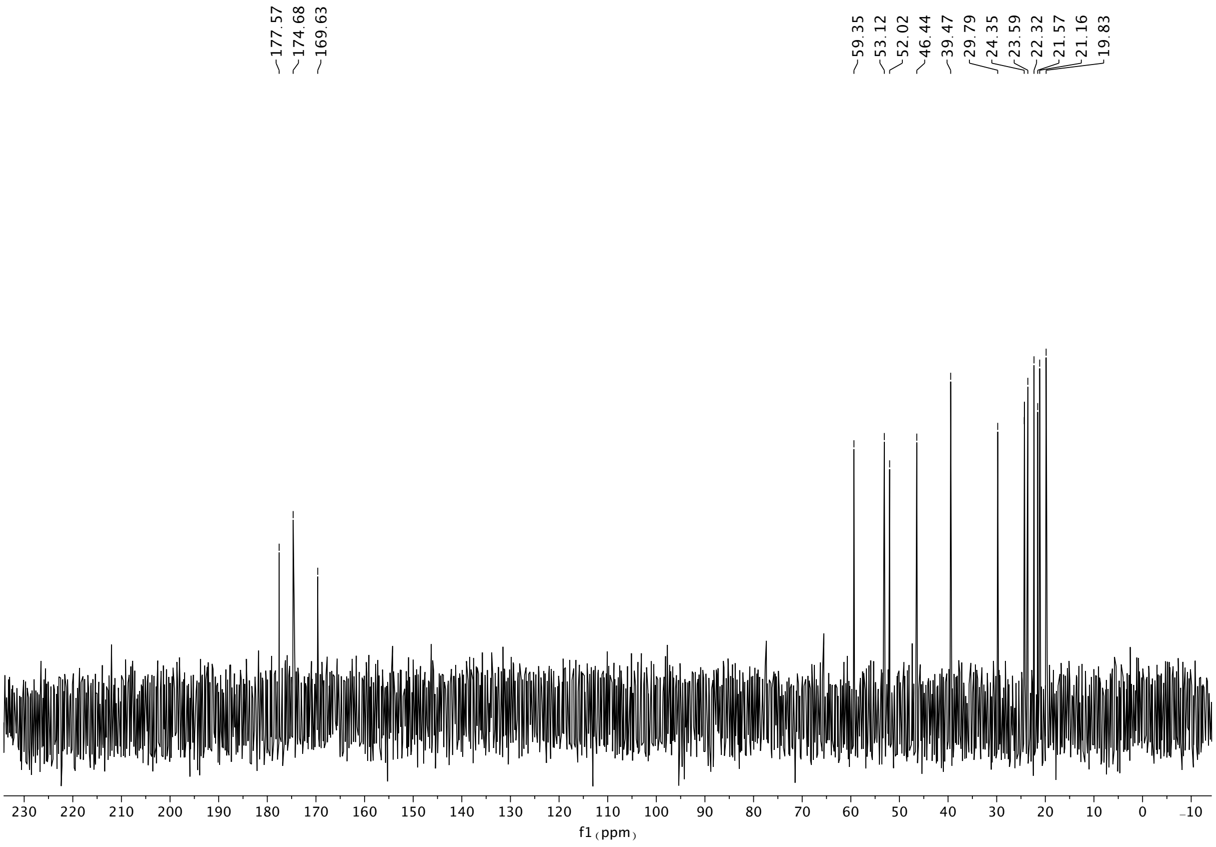

^{1}H and ^{13}C-NMR of H-D-Pro-L-Gln-L-Leu-NH_2 · TFA (UTS-58):

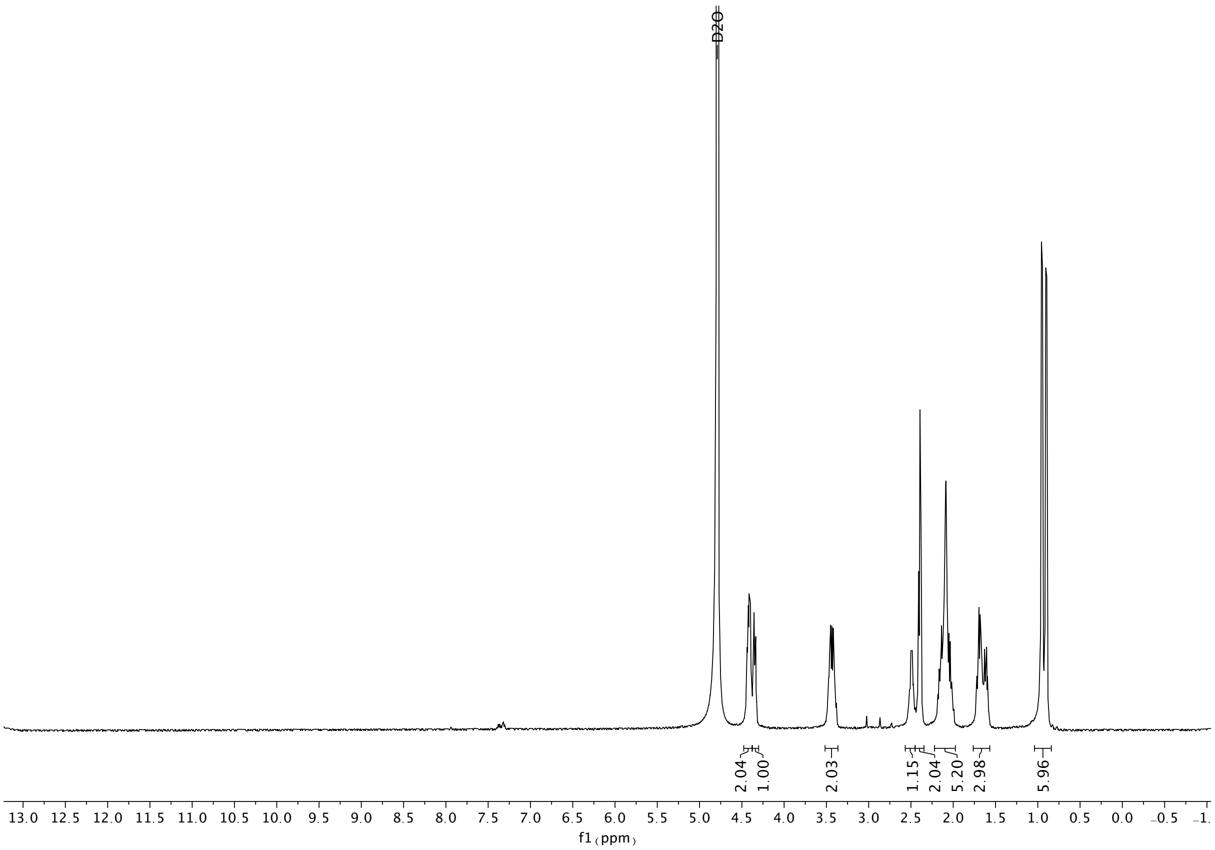

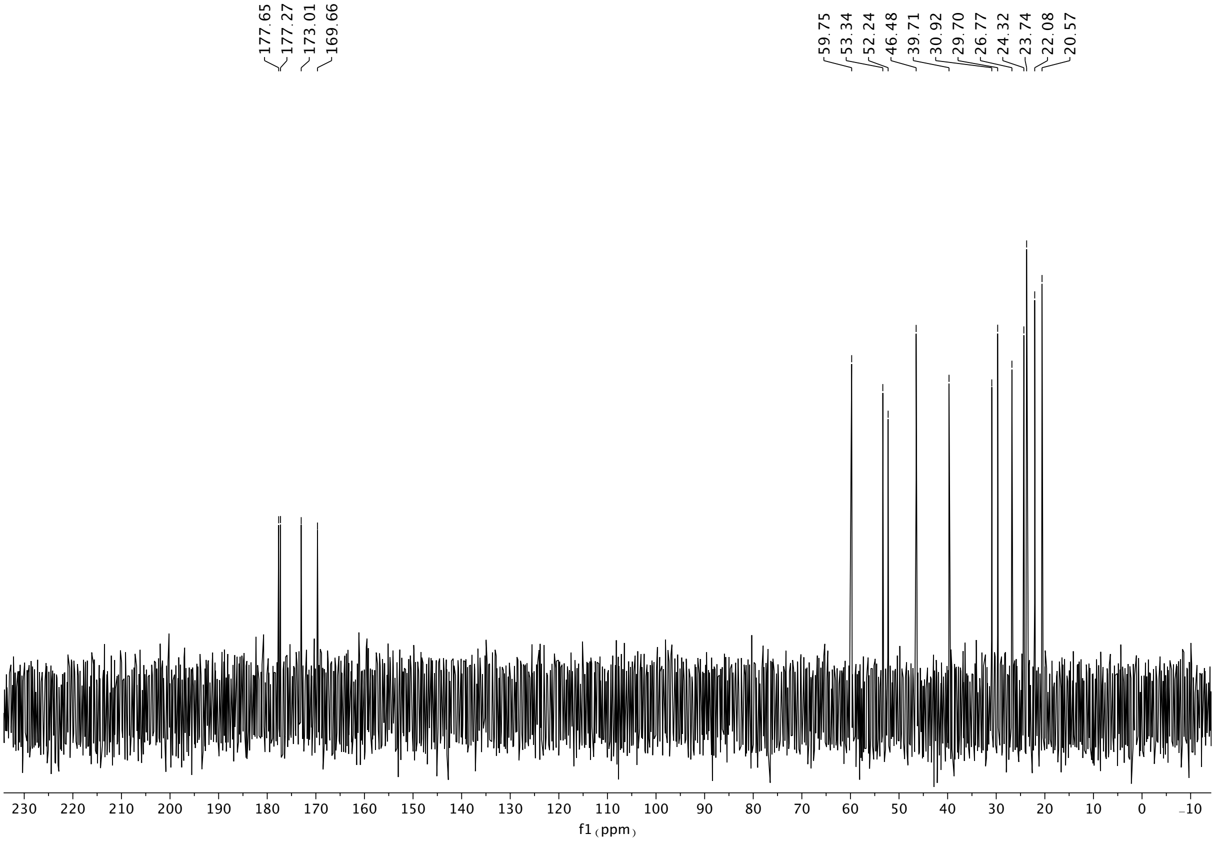

^{1}H and ^{13}C-NMR of H-D-Pro-D-Gln-L-Leu-NH_2 · TFA (UTS-59):

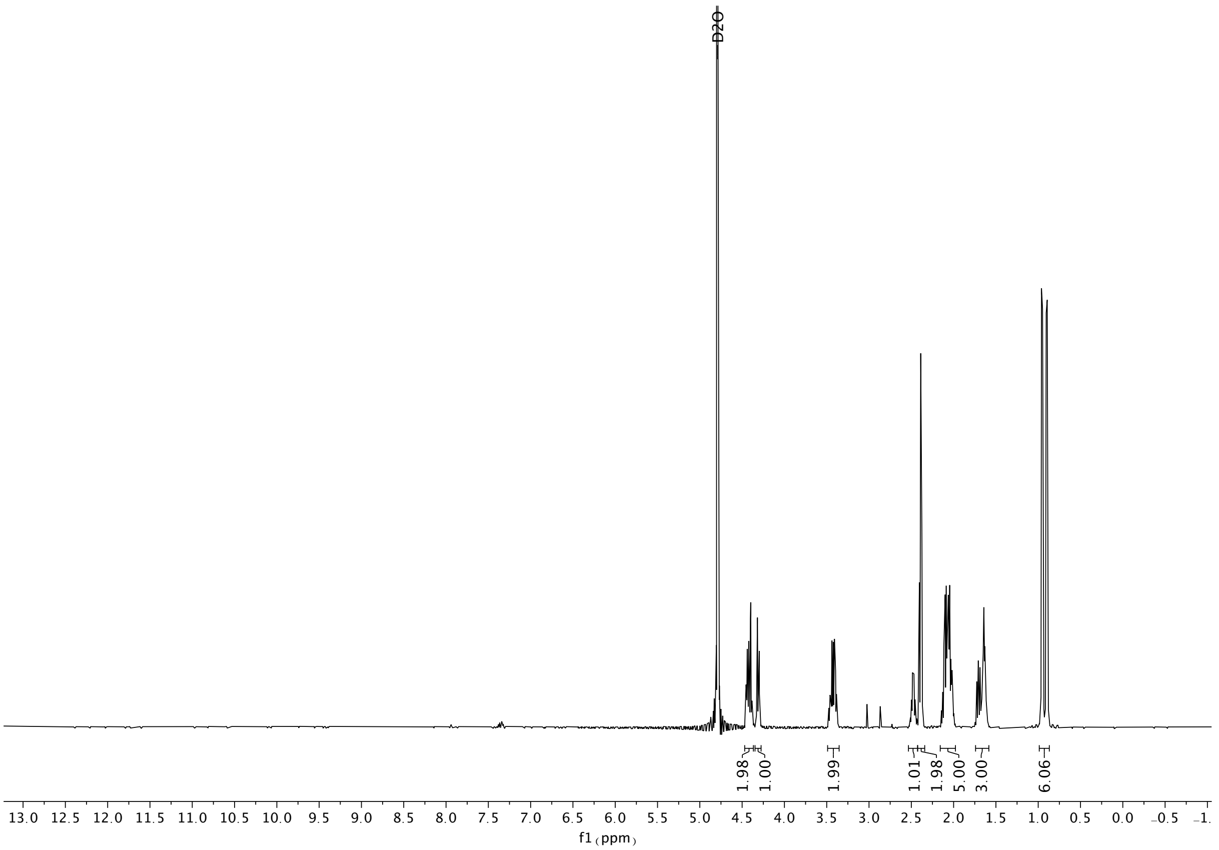

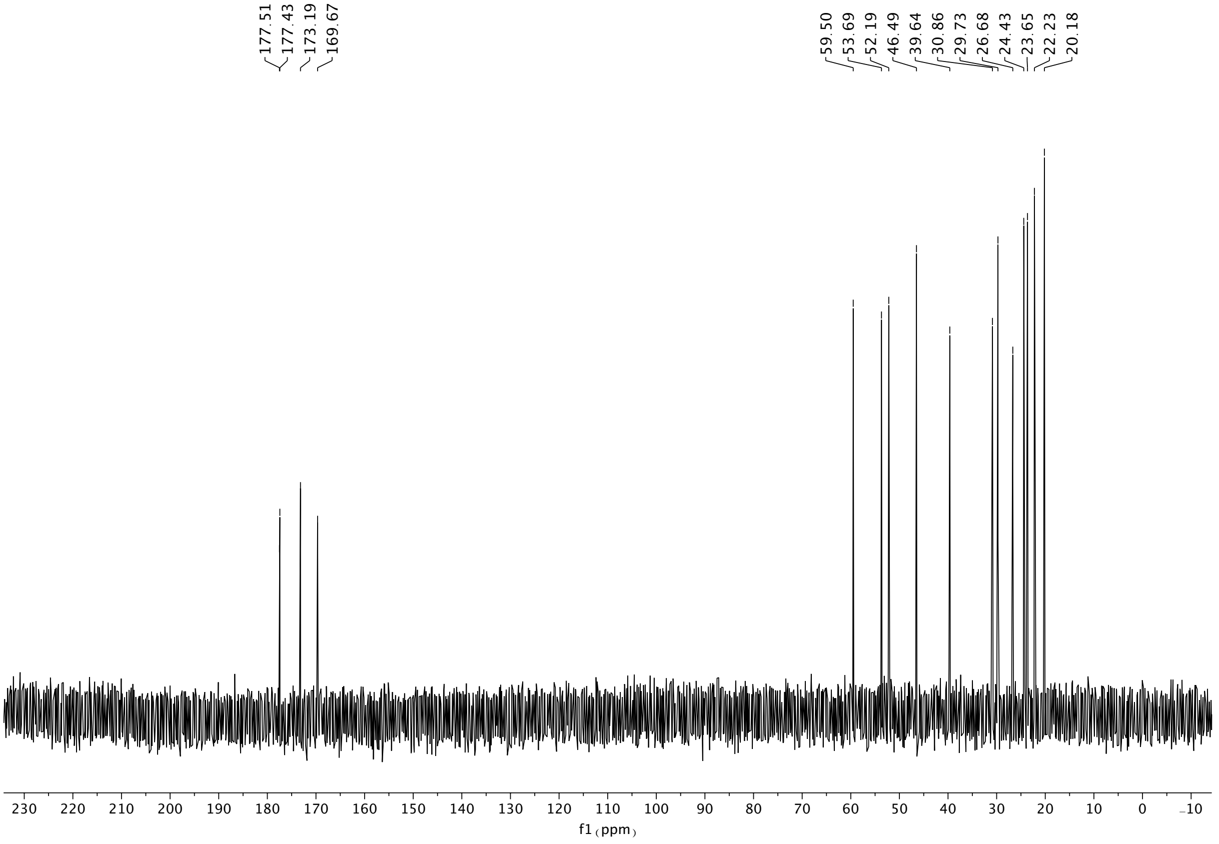

^{1}H and ^{13}C-NMR of H-D-Pro-L-Glu-L-Leu-NH₂ · TFA (UTS-60):

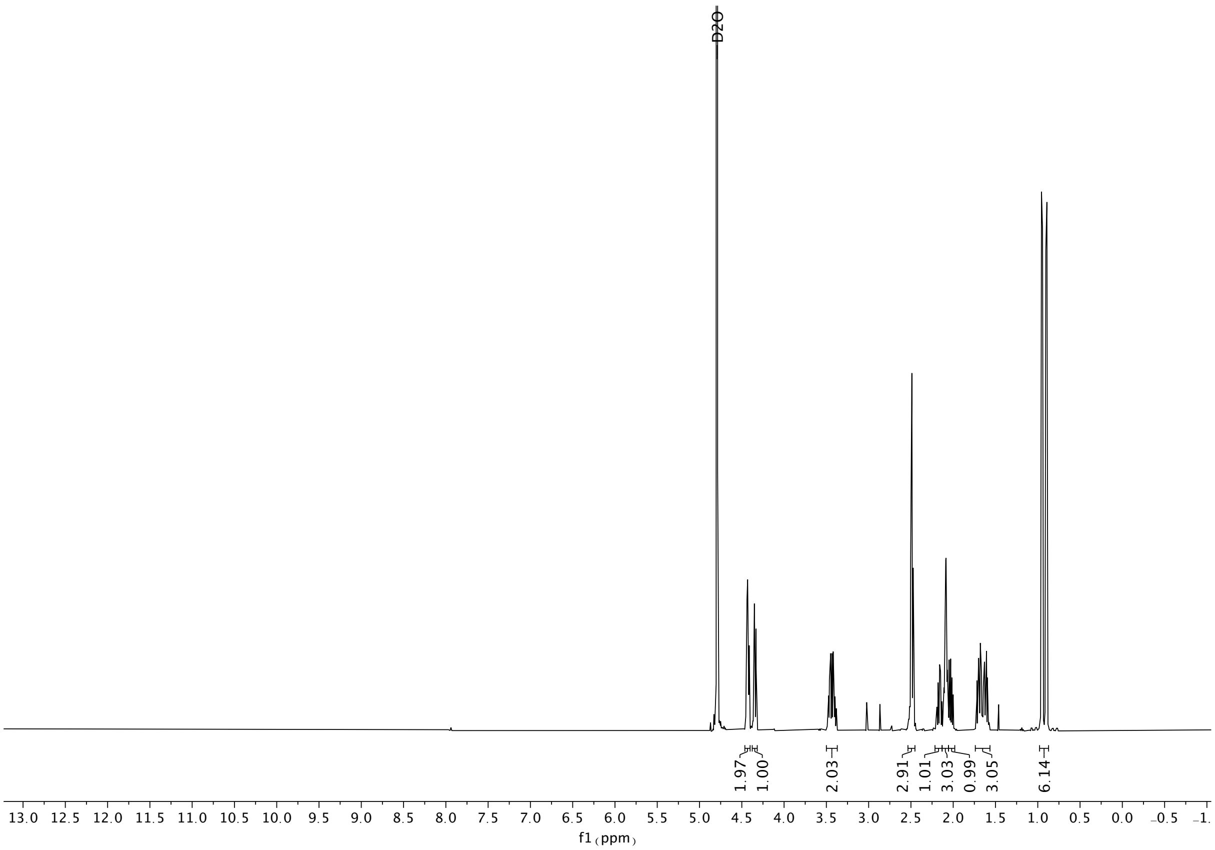

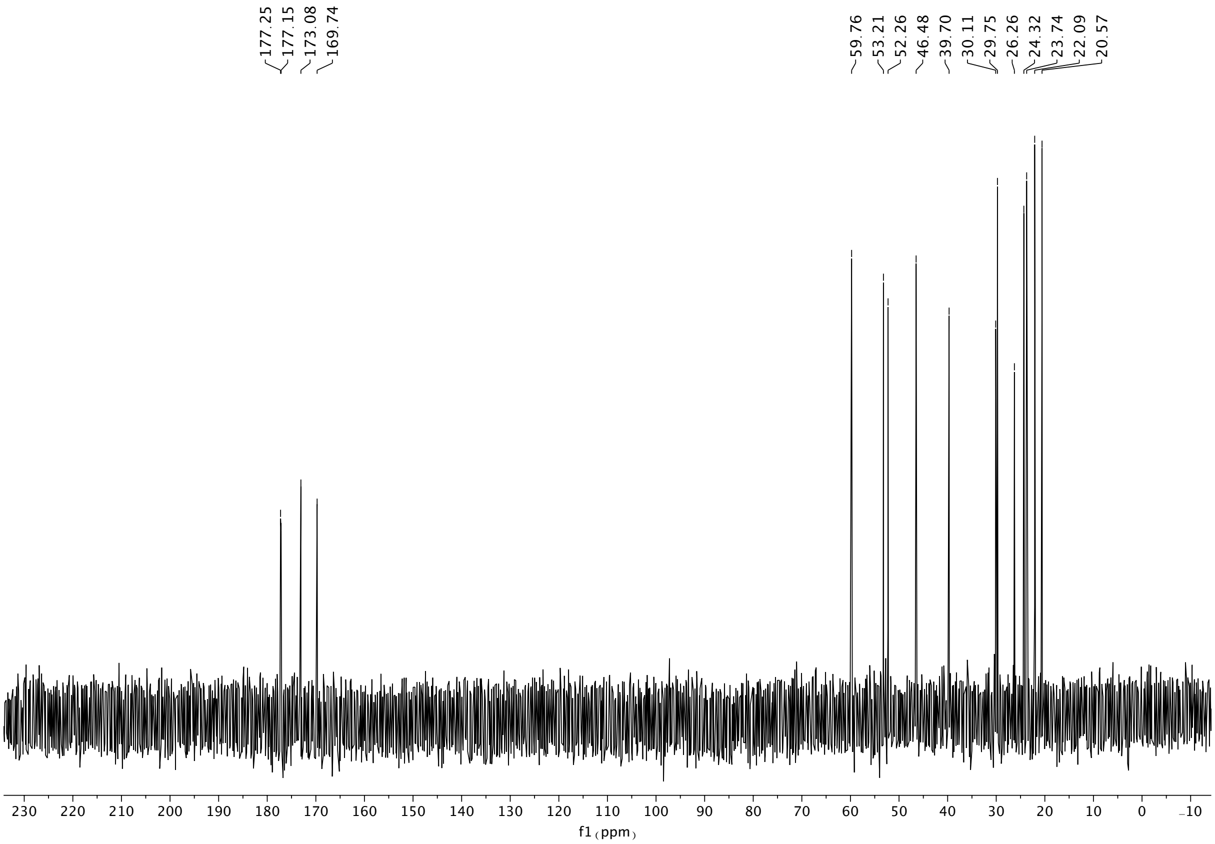

^{1}H and ^{13}C-NMR of H-D-Pro-D-Glu-L-Leu-NH$_2$ · TFA (UTS-61):

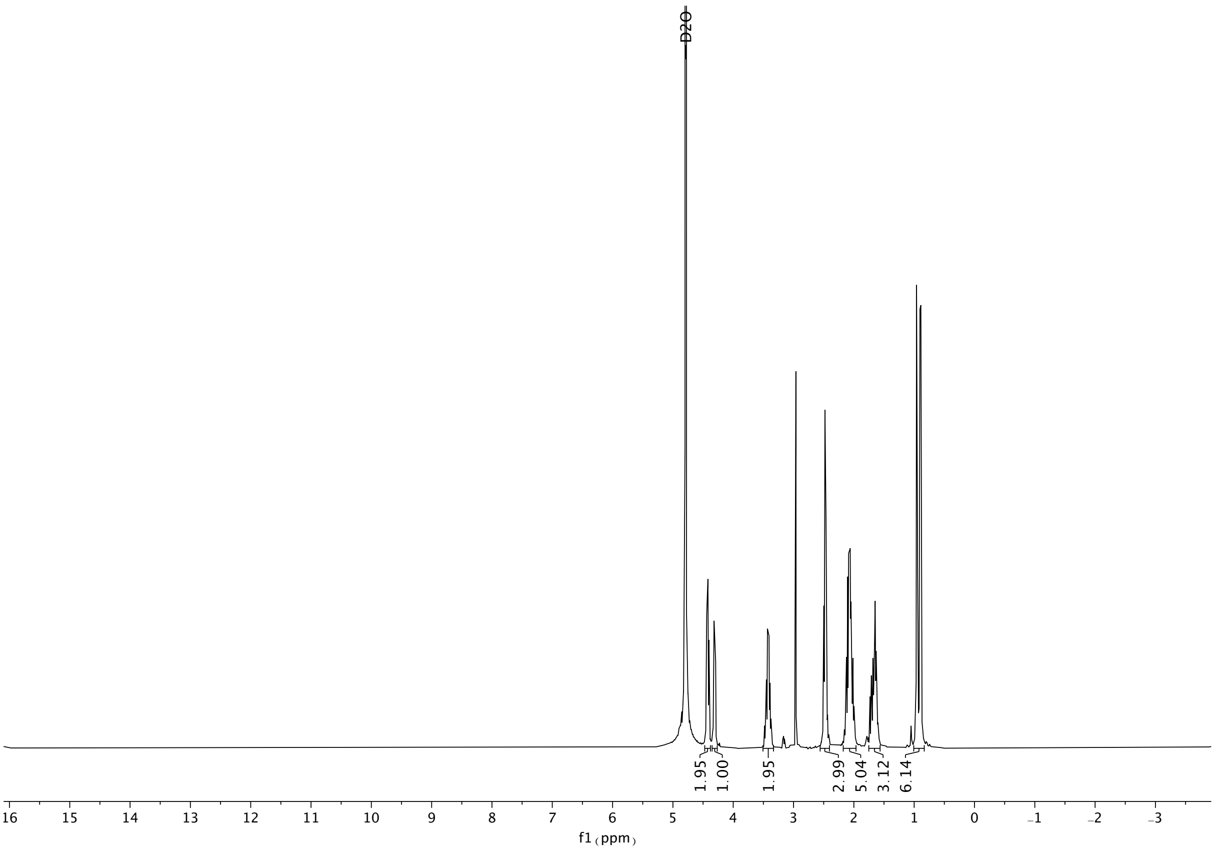

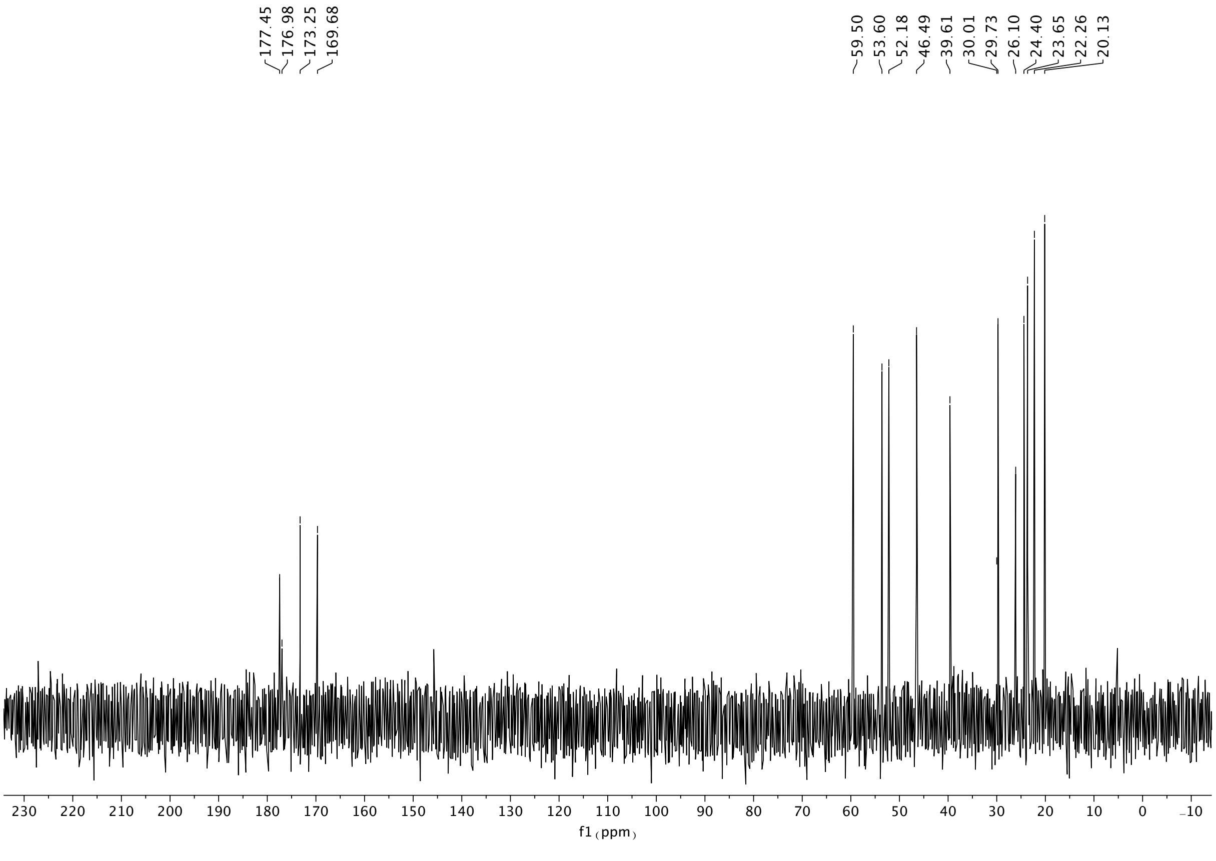

^{1}H and ^{13}C-NMR of H-D-Pro-L-Tyr-L-Leu-NH₂ · TFA (UTS-62):

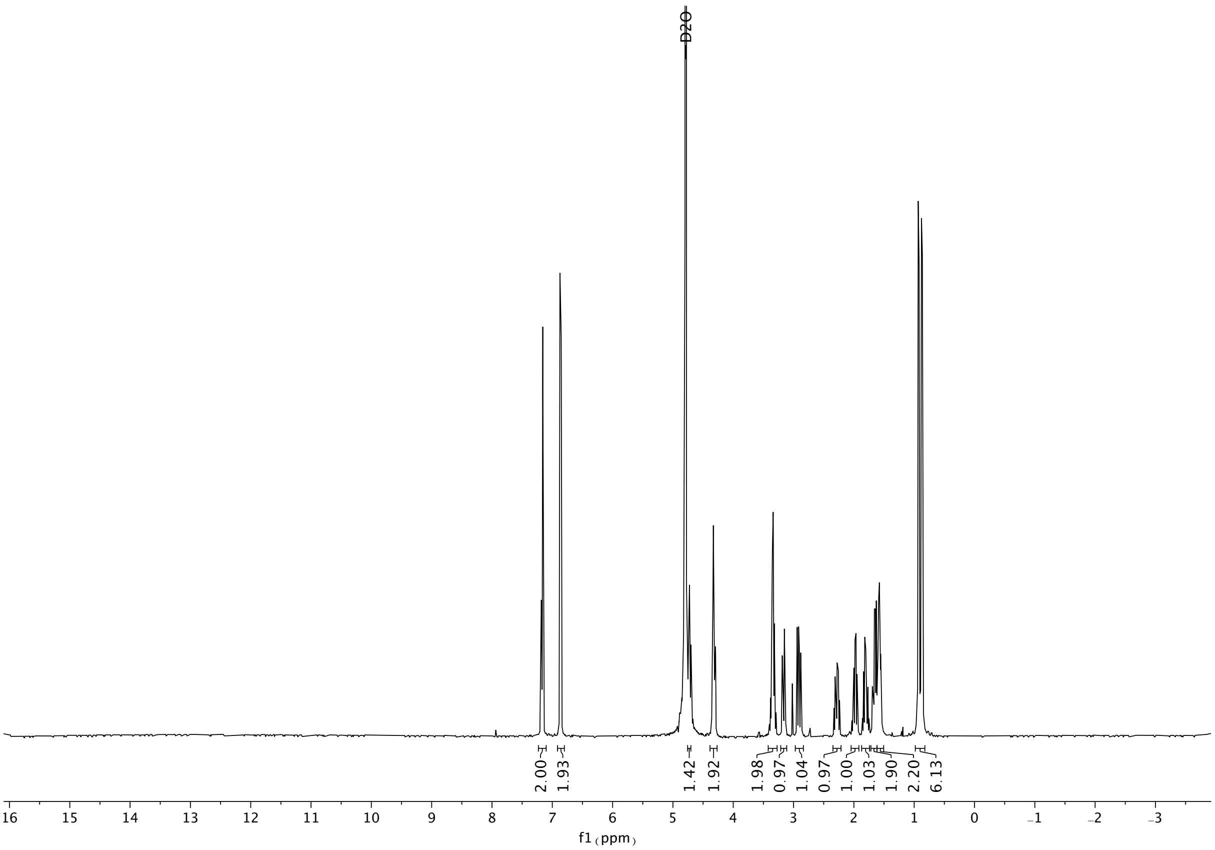

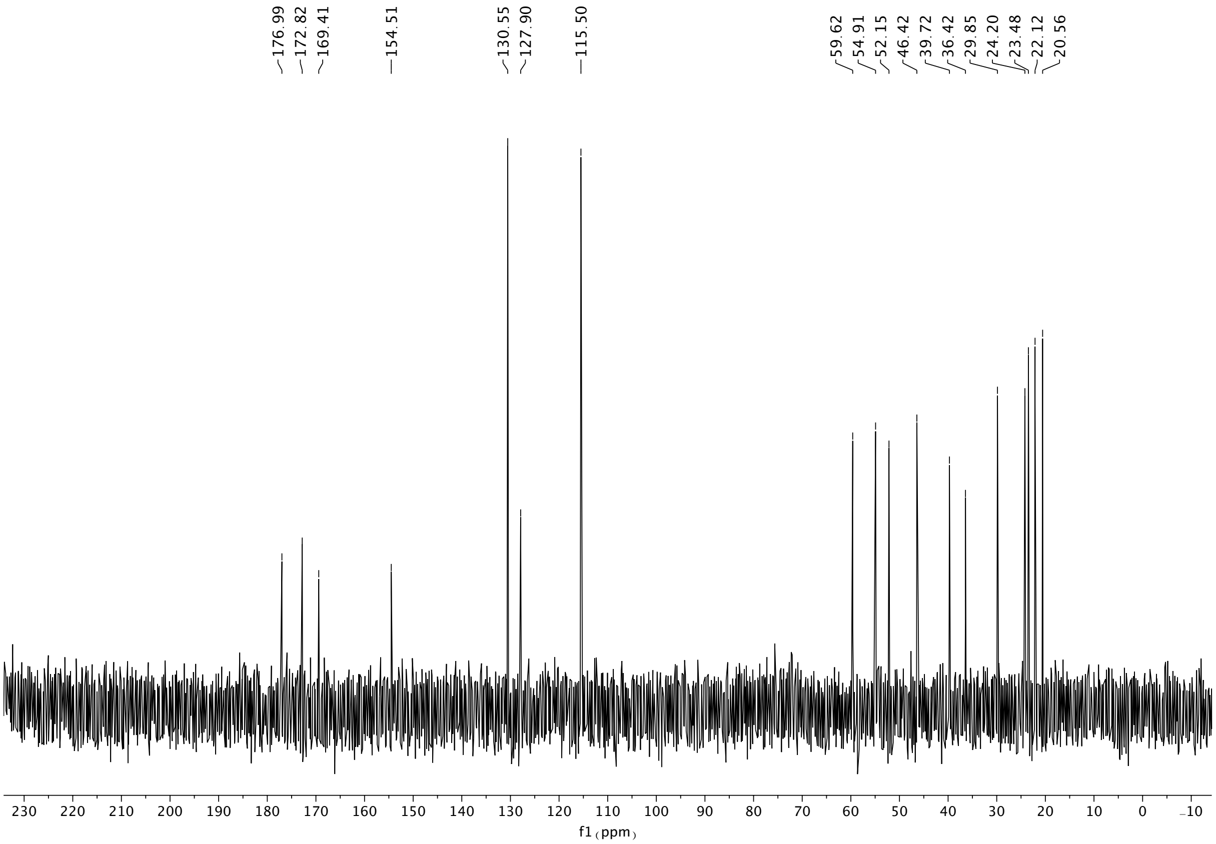

^{1}H and ^{13}C-NMR of H-D-Pro-D-Tyr-L-Leu-NH_2 · TFA (UTS-63):

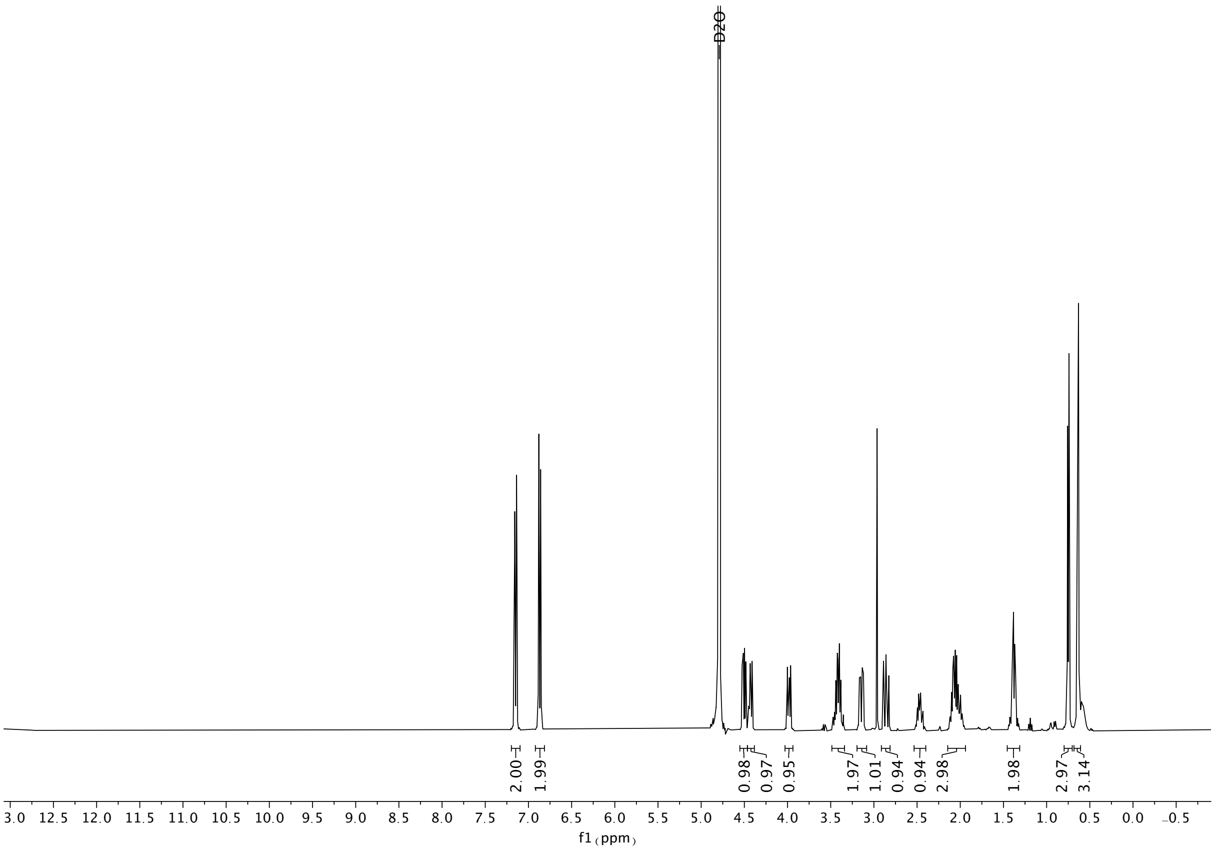

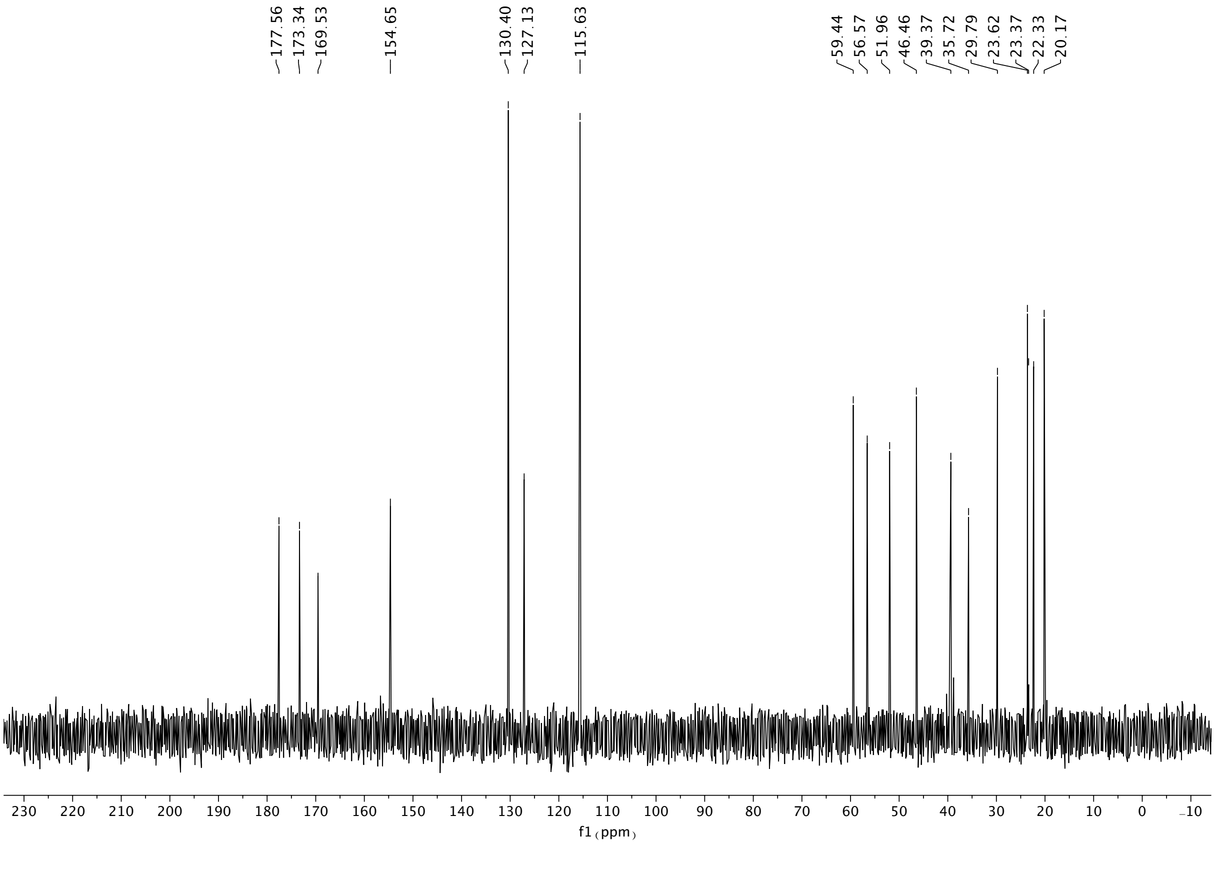

^{1}H and ^{13}C-NMR of H-D-Pro-CyLeu-L-Leu-NH₂ · TFA (UTS-64):

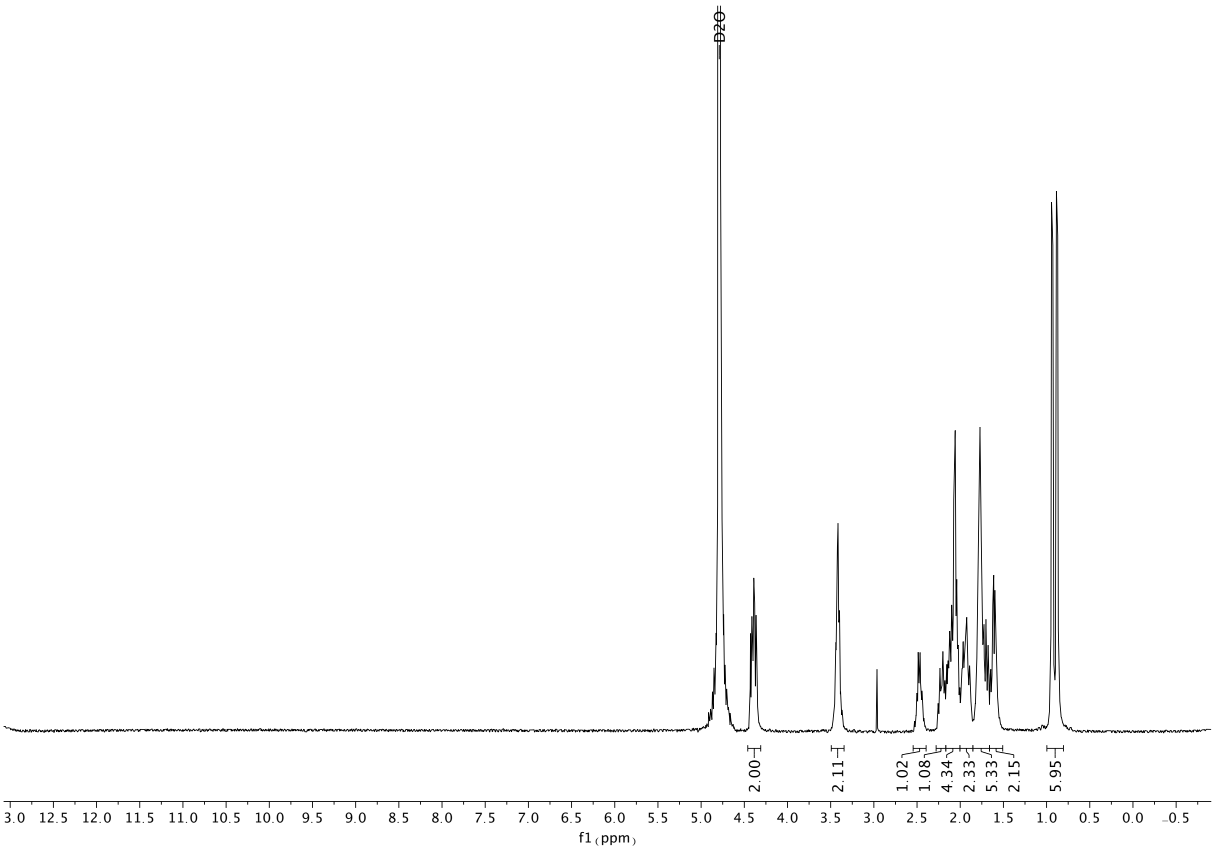

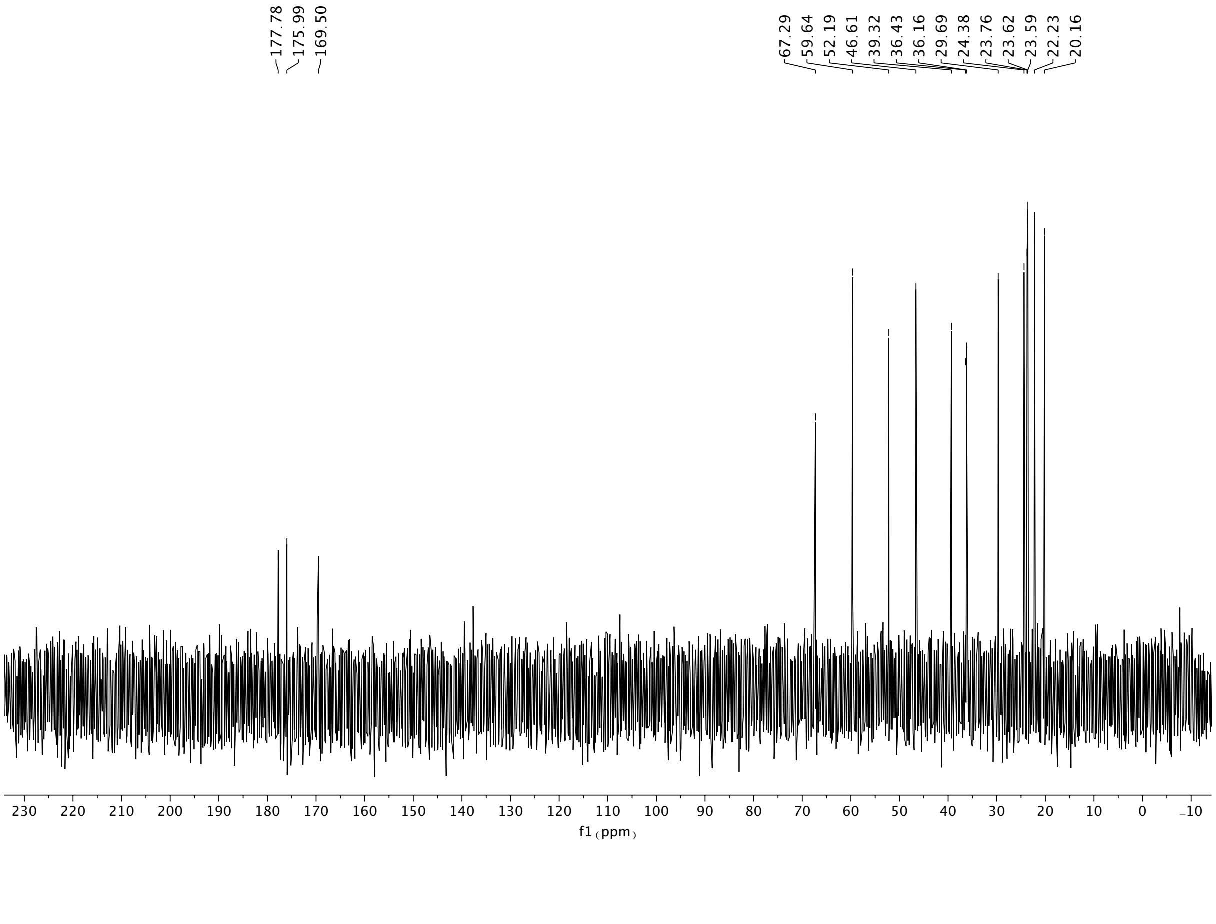

^{1}H and ^{13}C-NMR of H-D-Pro-Abz-L-Leu-NH_2 · TFA (UTS-65):

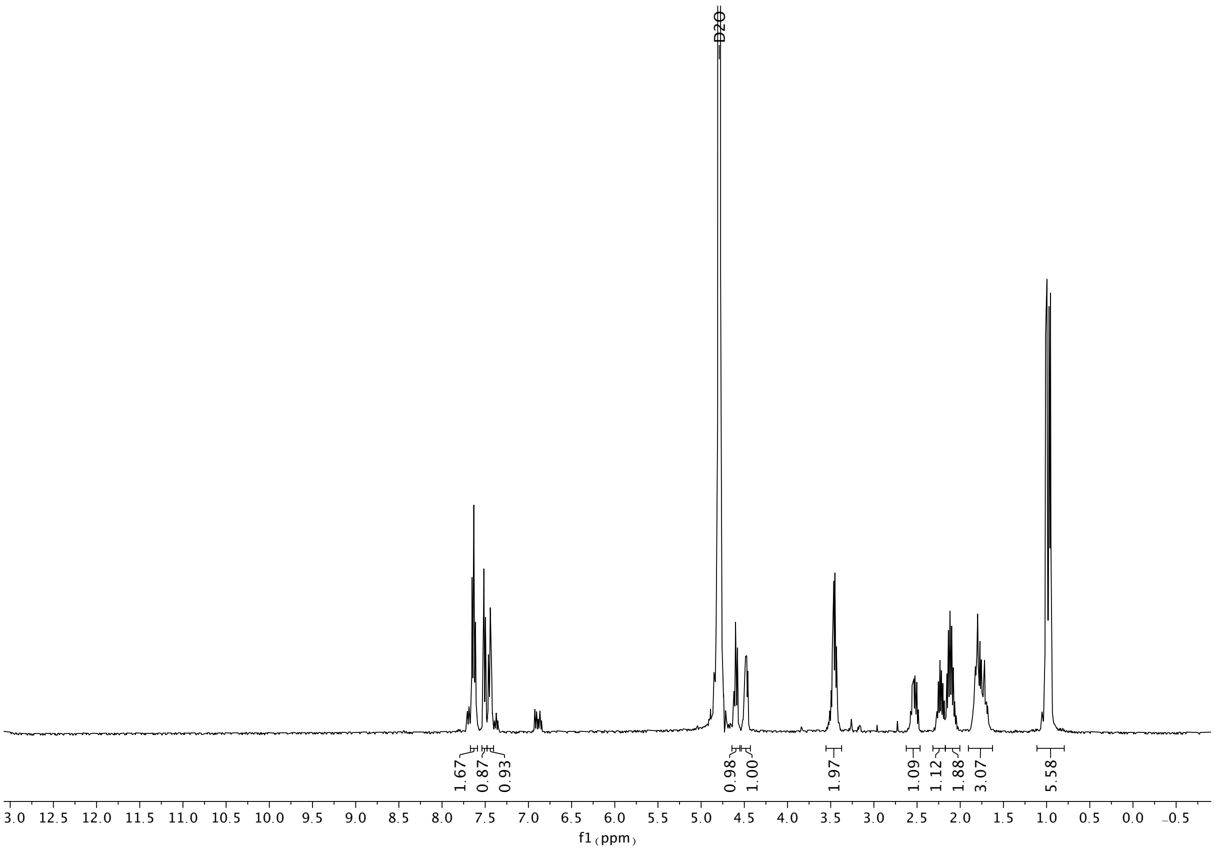

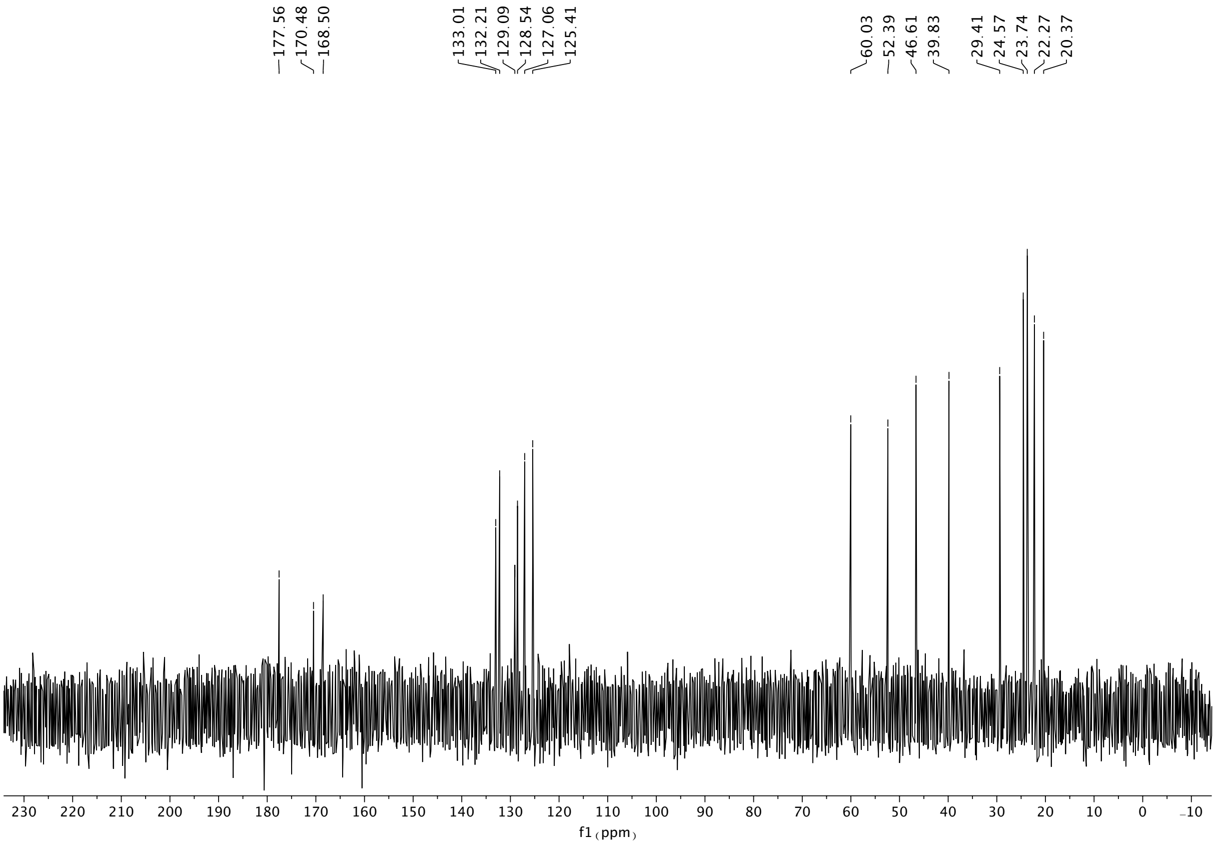

^{1}H and ^{13}C-NMR of H-D-Pro-D-Ind-L-Leu-NH_2 · TFA (UTS-66):

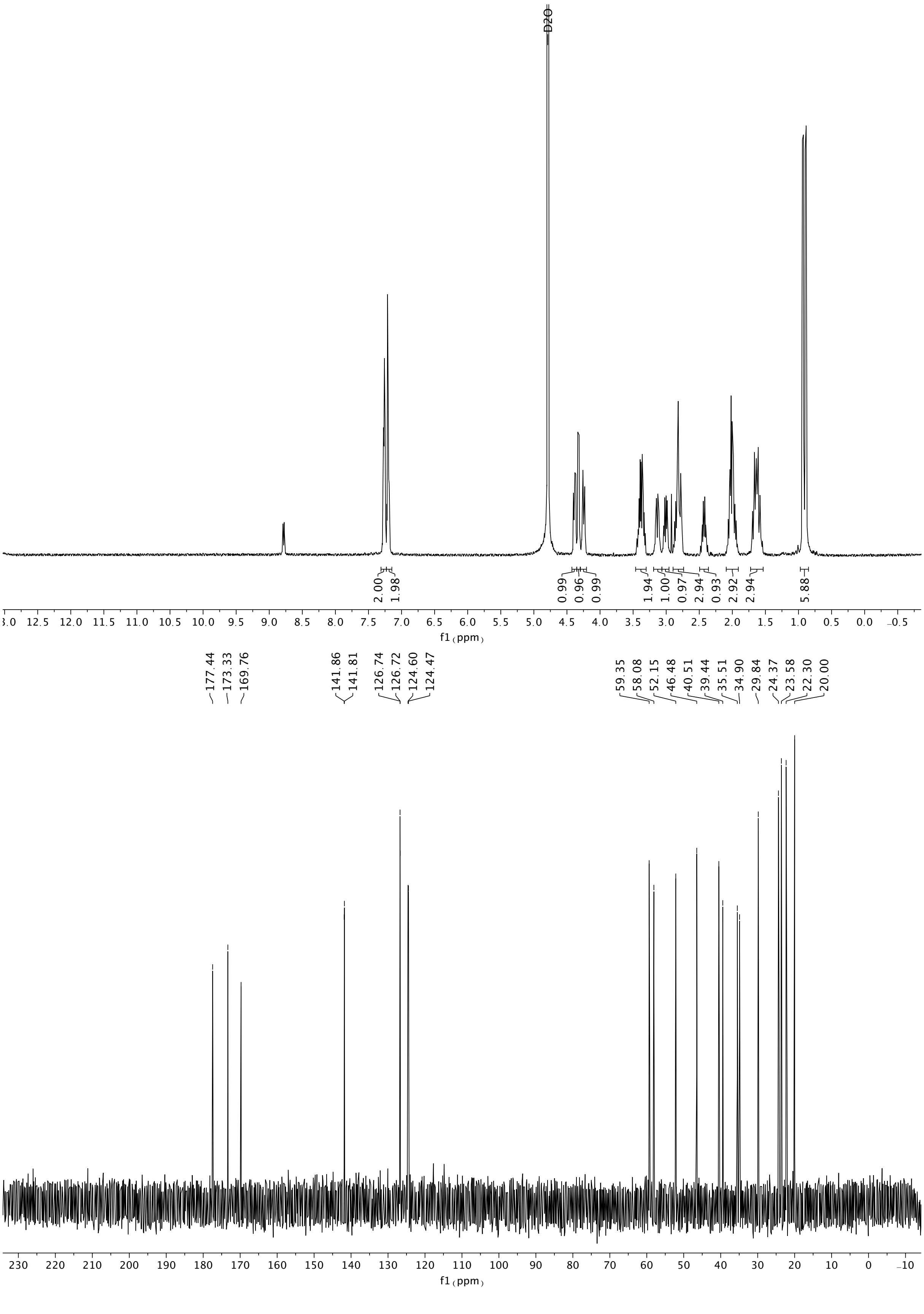

^{1}H and ^{13}C-NMR of H-D-Pro-D-Leu-D-Leu-NH_2 · TFA (UTS-67):

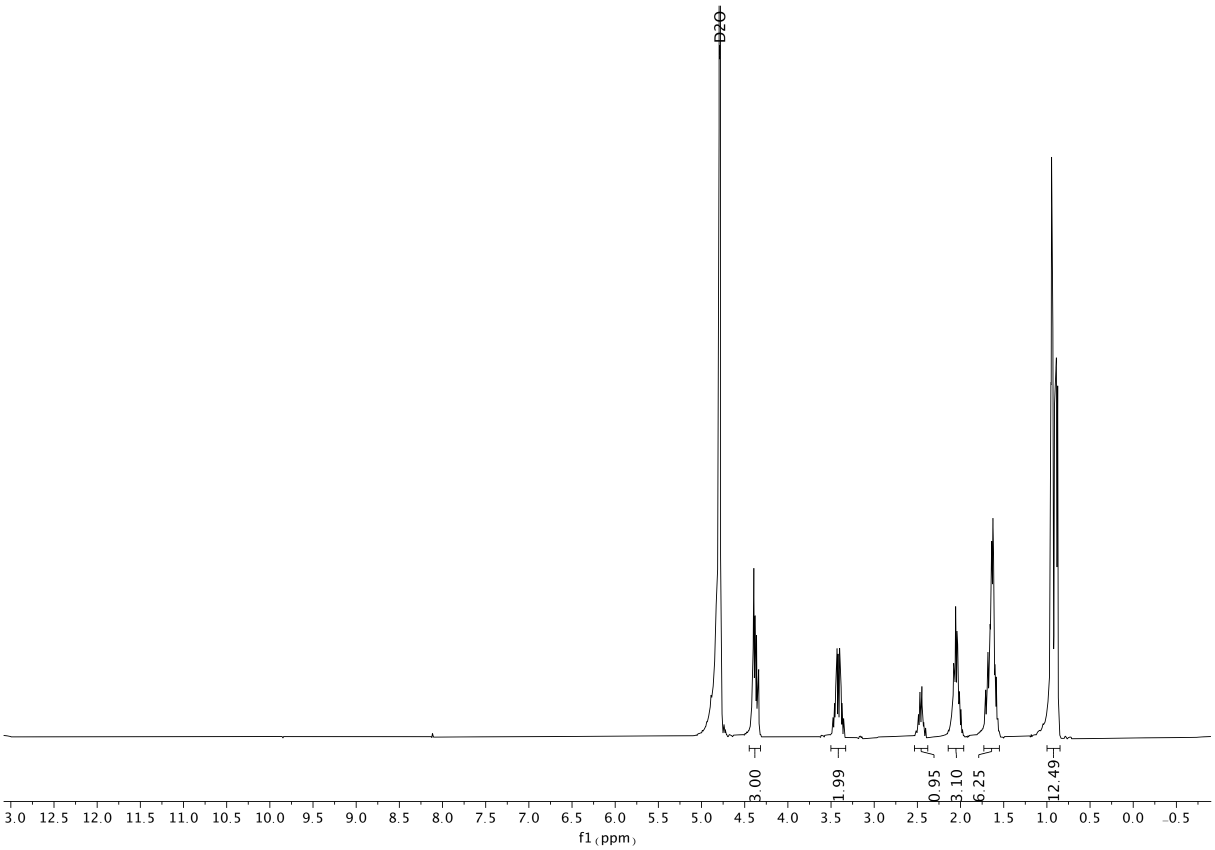

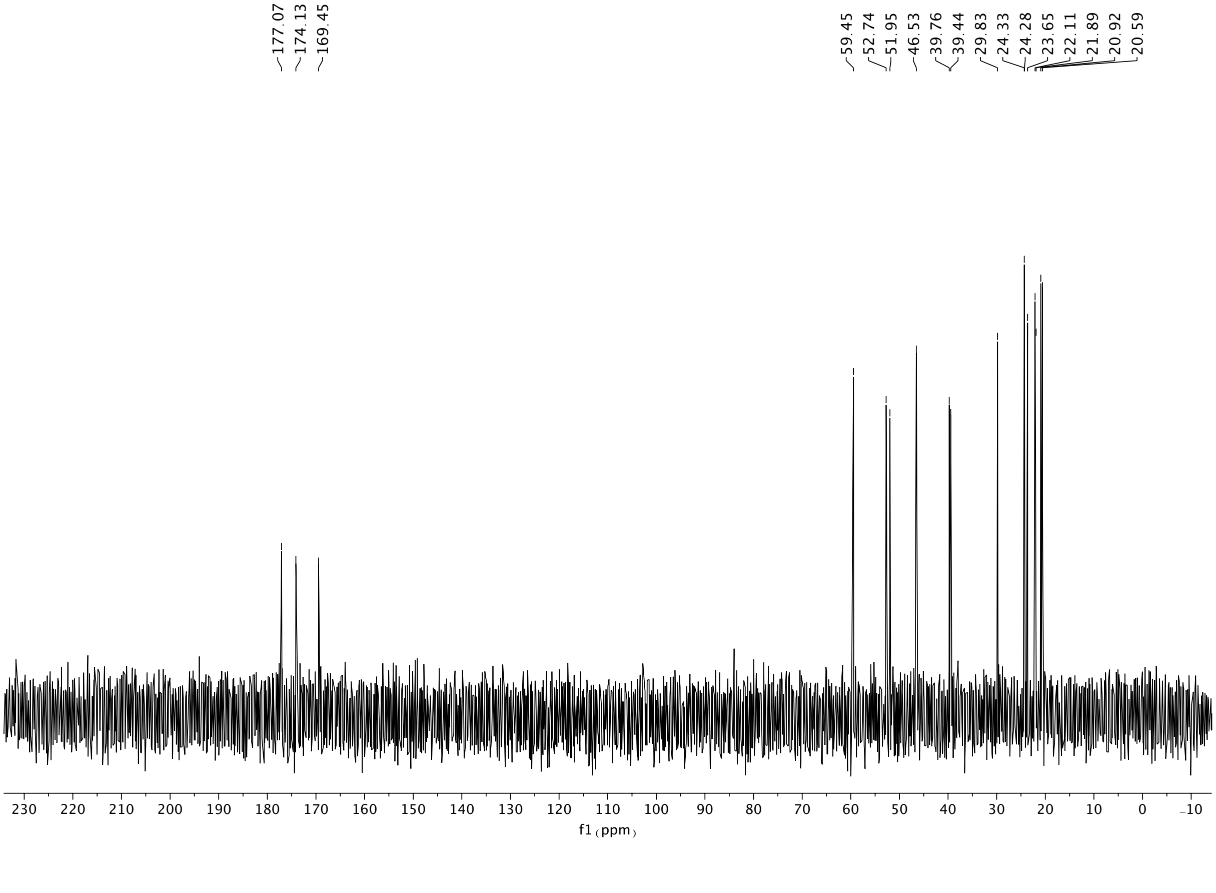

^{1}H and ^{13}C-NMR of H-D-Pro-D-Leu-L-Gln-NH_2 · TFA (UTS-68):

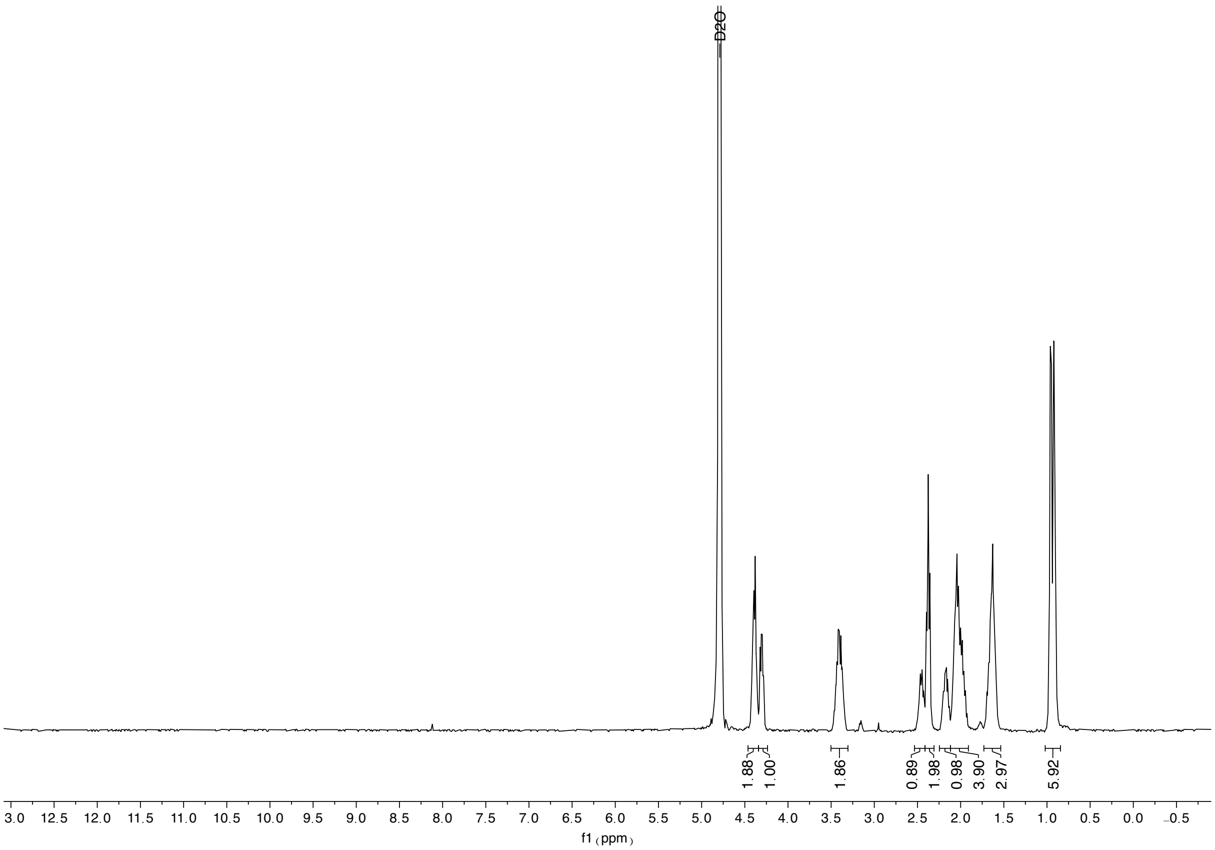

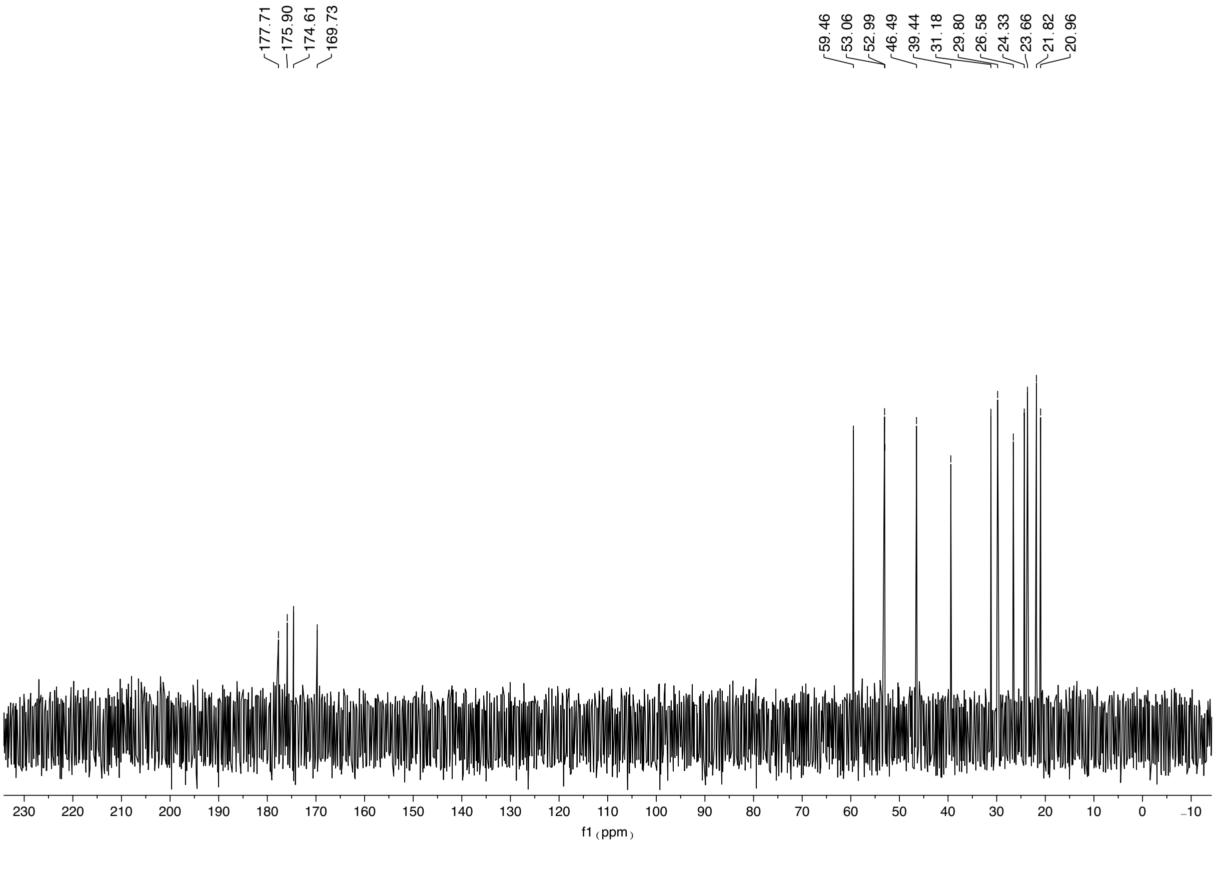

^{1}H and ^{13}C-NMR of H-D-Pro-D-Leu-D-Gln-NH_2 · TFA (UTS-69):

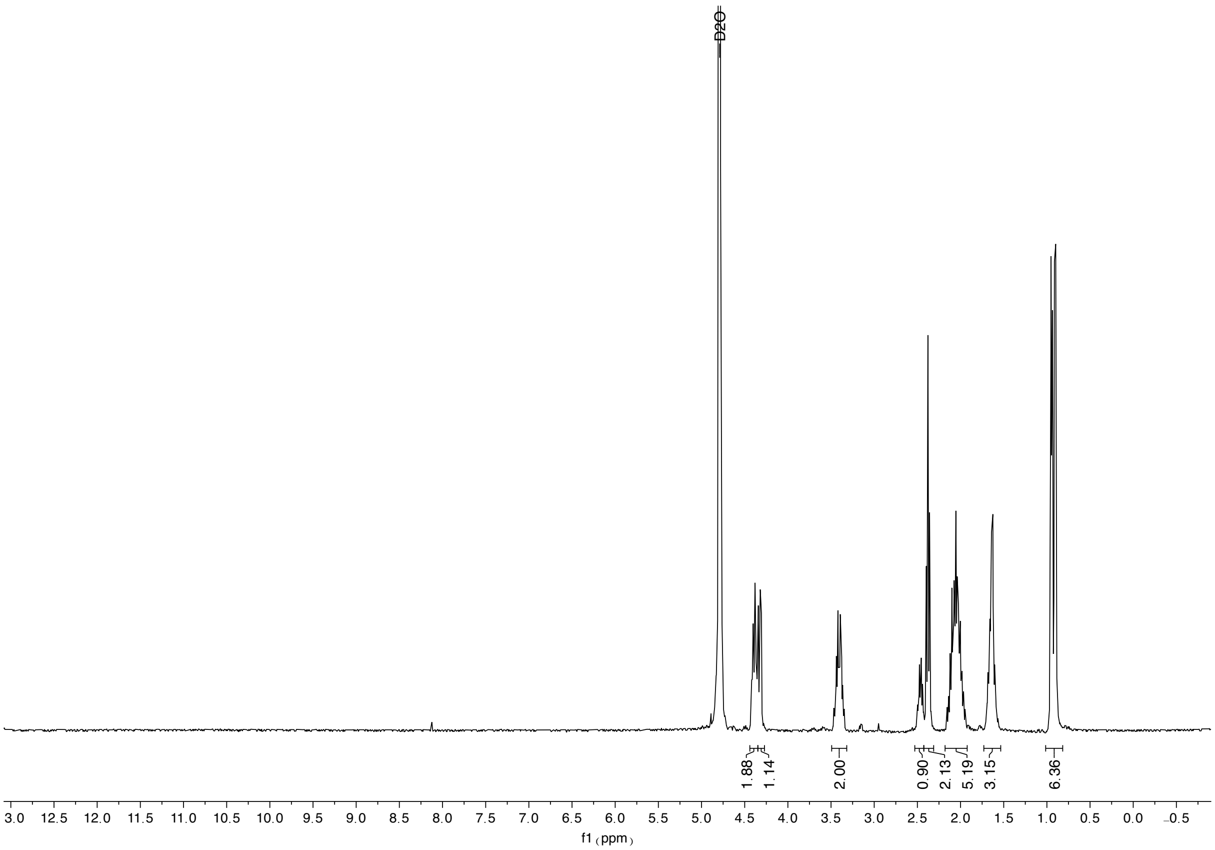

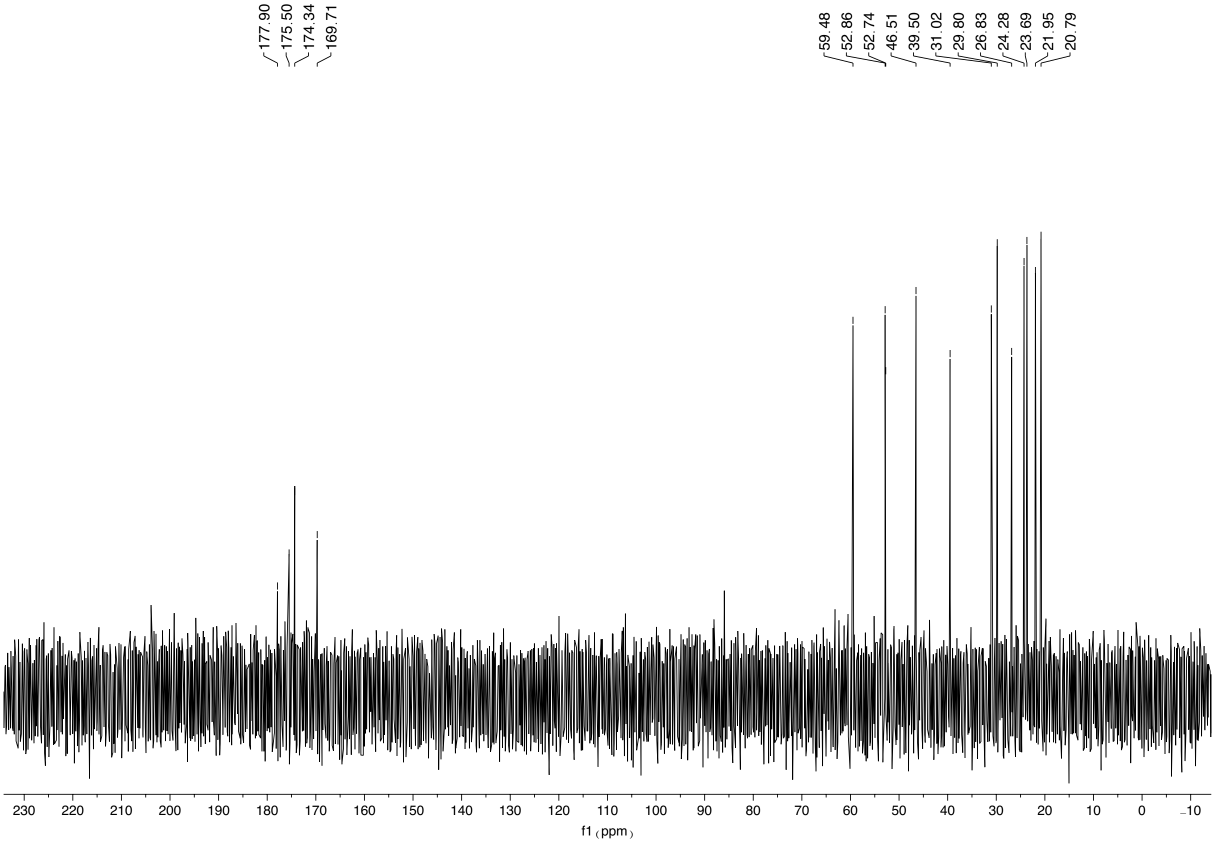

^{1}H and ^{13}C-NMR of H-D-Pro-D-Leu-L-Glu-NH$_2$ · TFA (UTS-70):

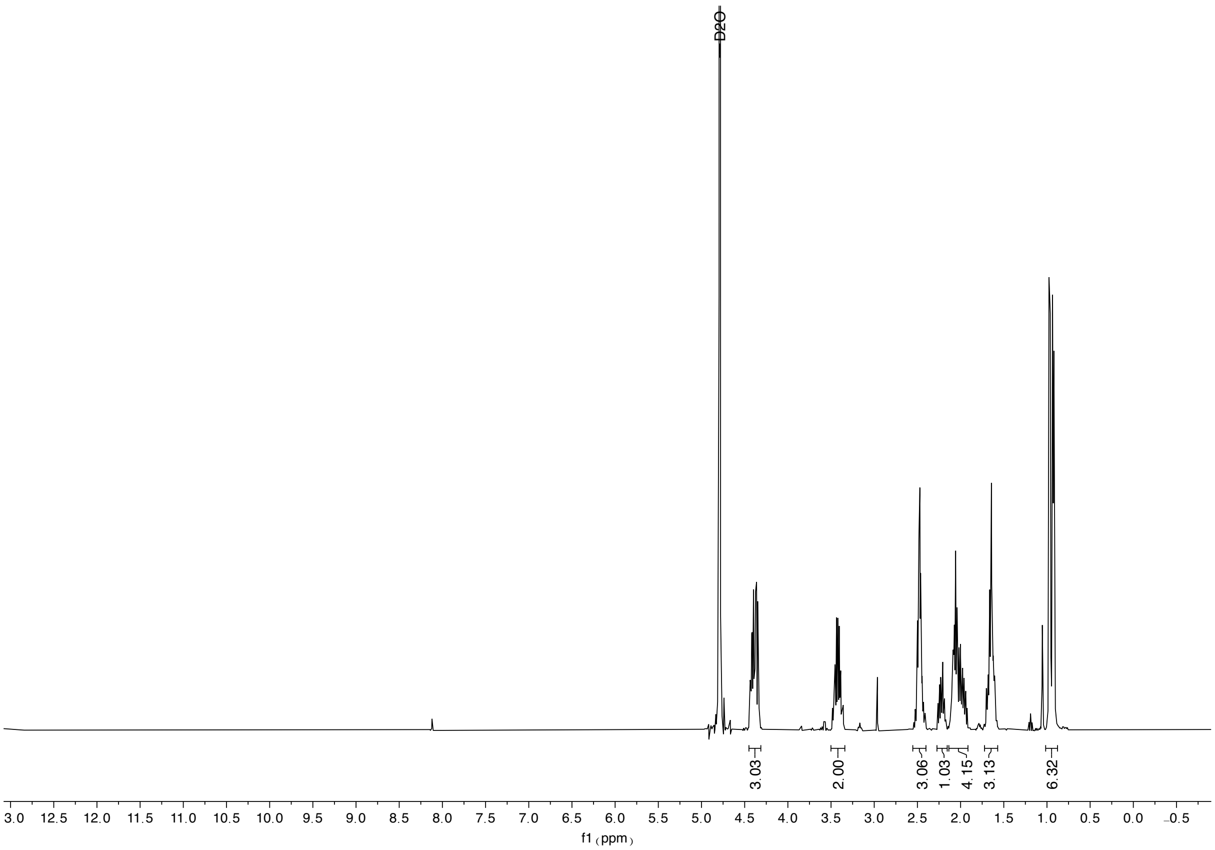

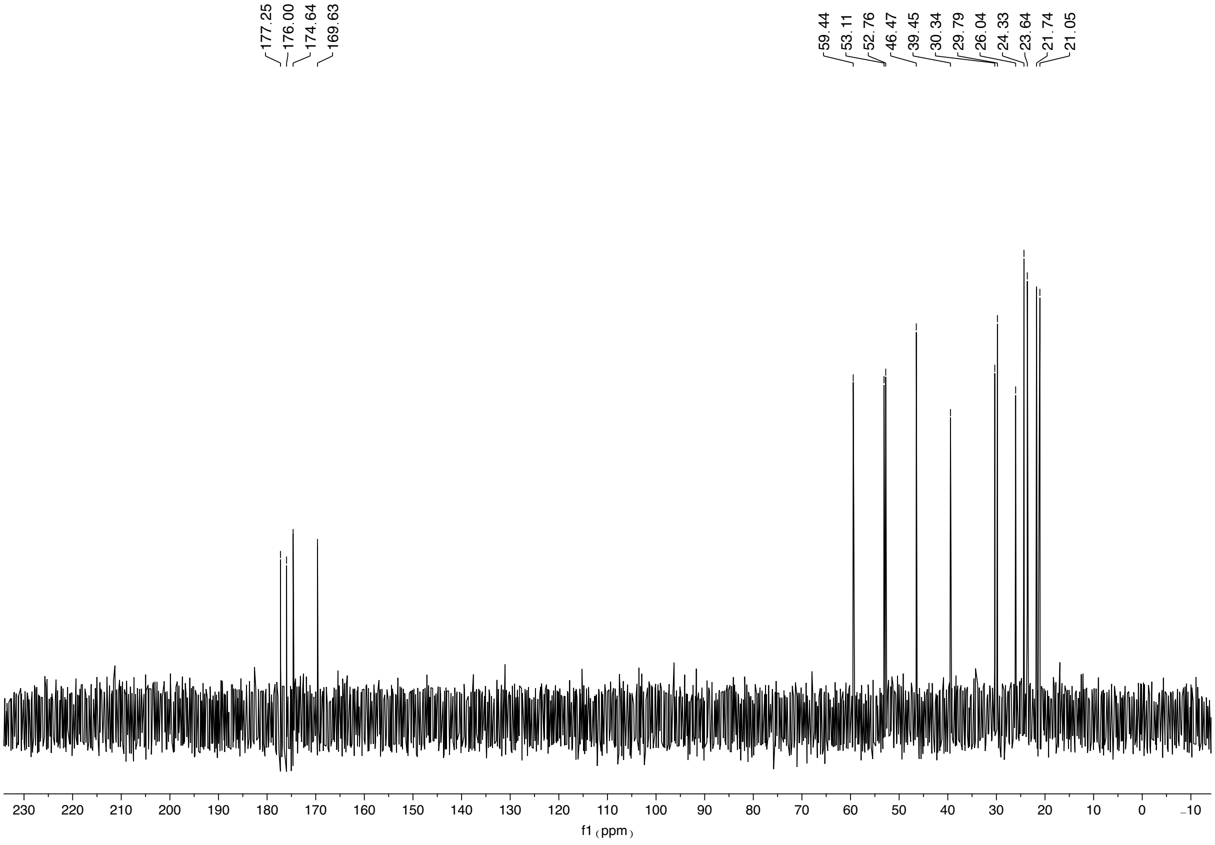

^{1}H and ^{13}C-NMR of H-D-Pro-D-Leu-D-Glu-NH$_2$ · TFA (UTS-71):

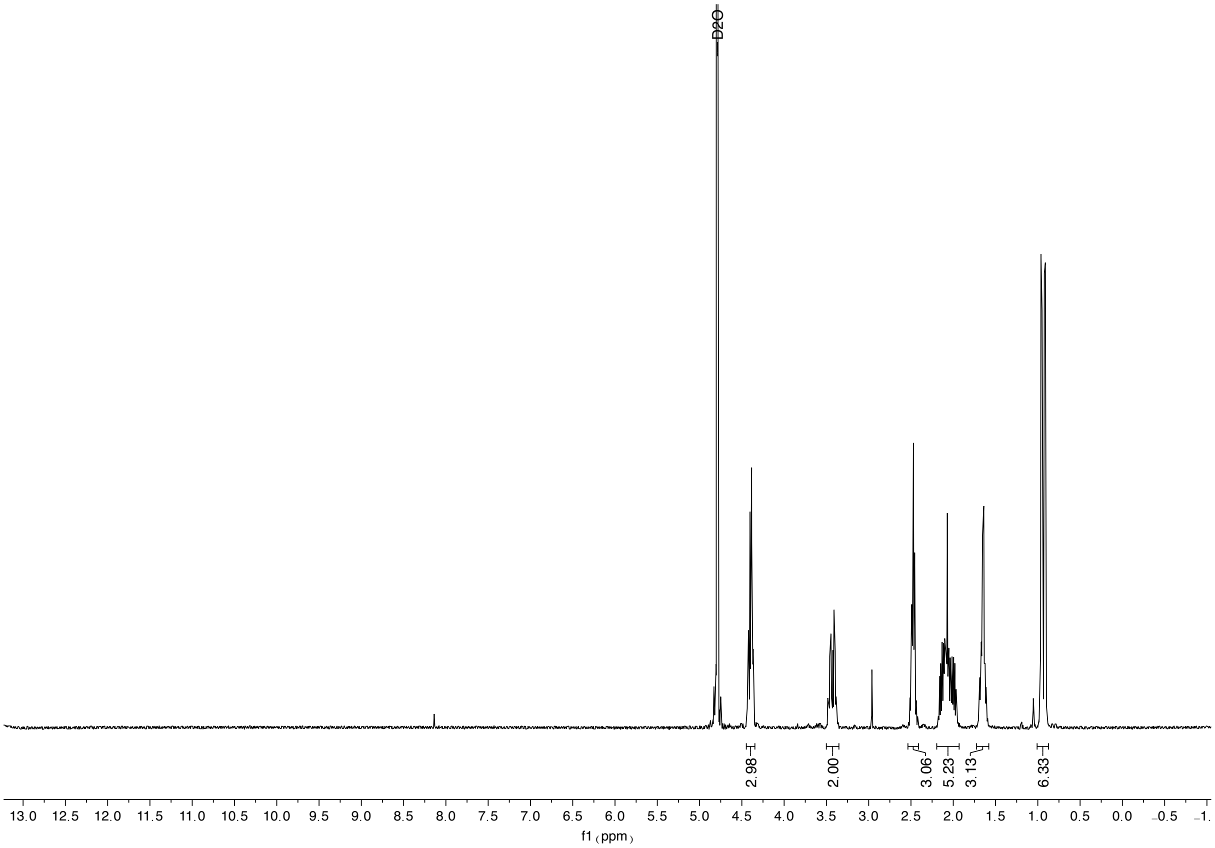

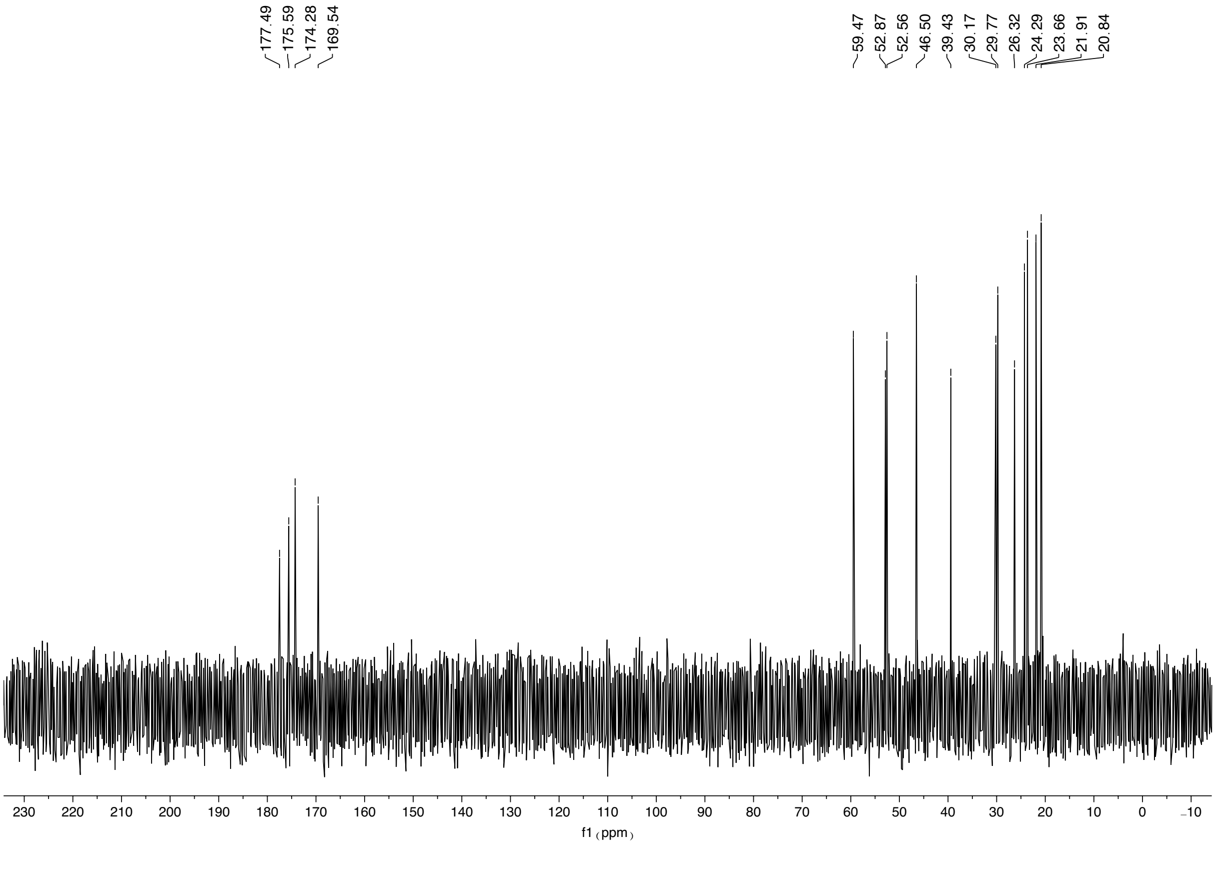

^{1}H and ^{13}C-NMR of H-D-Pro-D-Leu-L-Tyr-NH_2 · TFA (UTS-72):

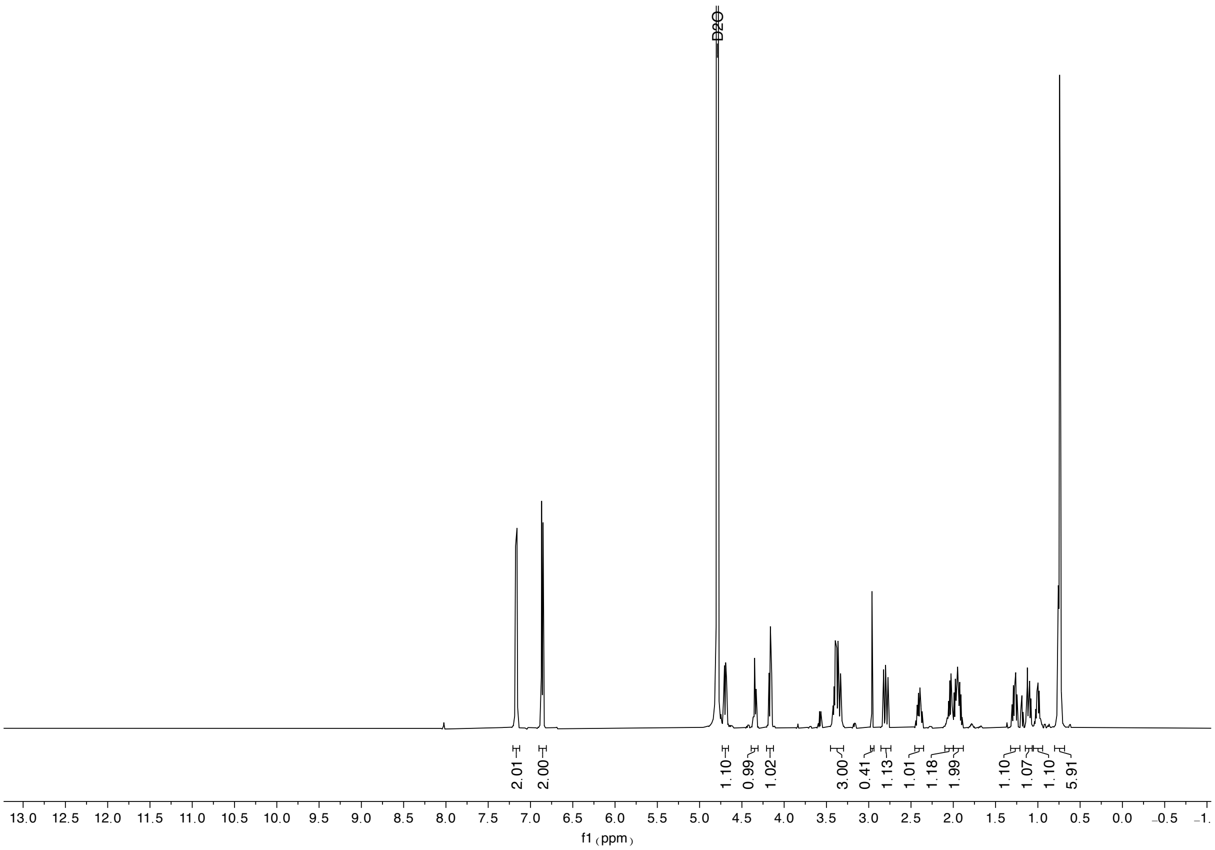

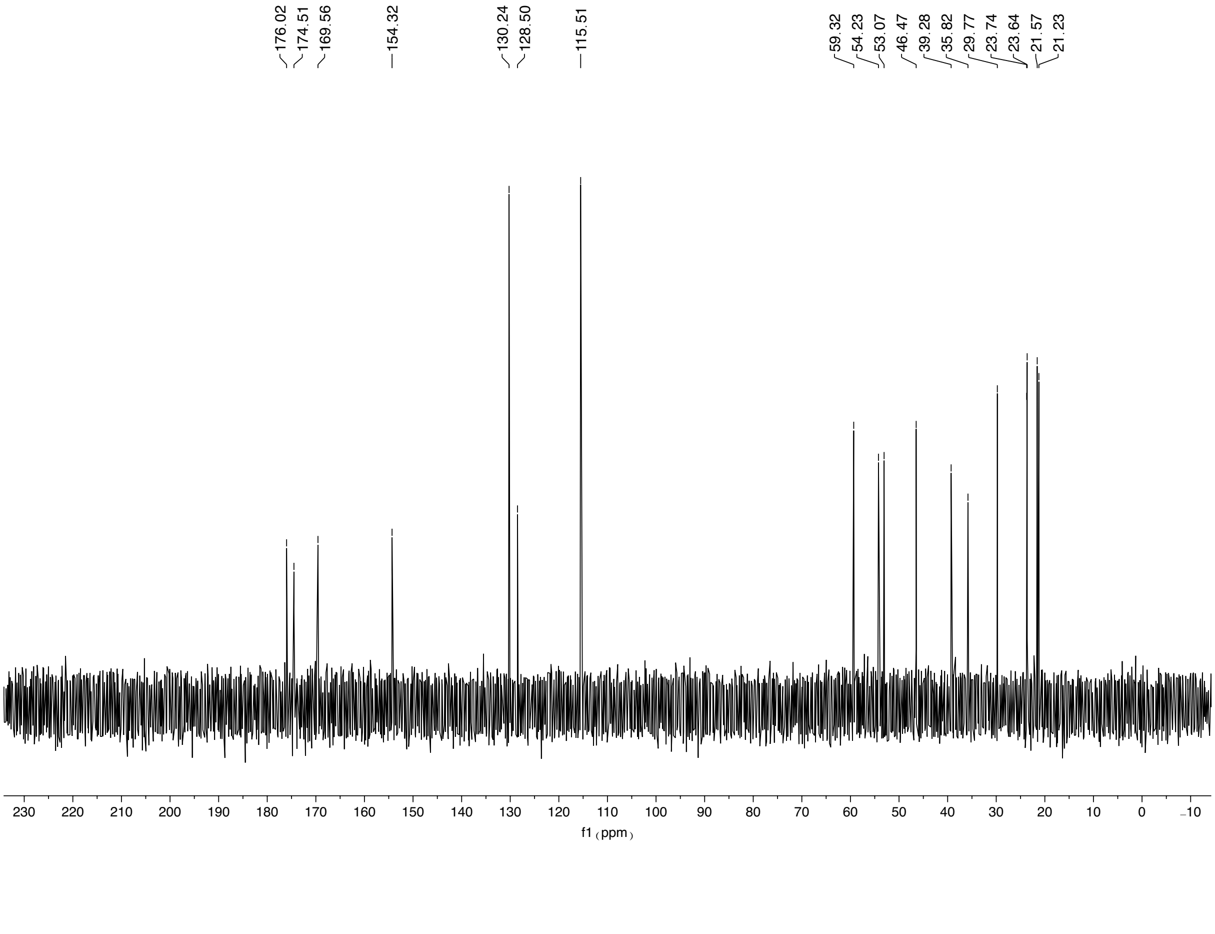

^{1}H and ^{13}C-NMR of H-D-Pro-D-Leu-D-Tyr-NH$_2$ · TFA (UTS-73):

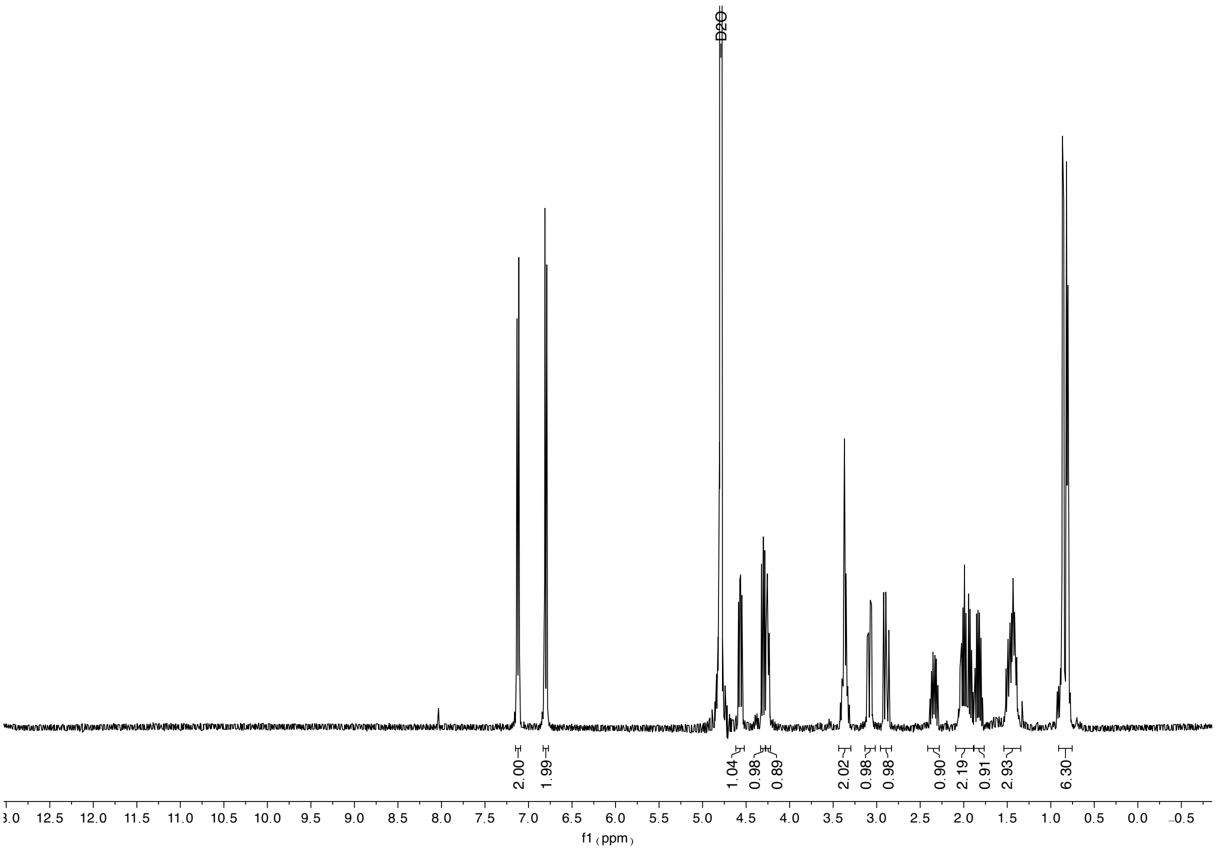

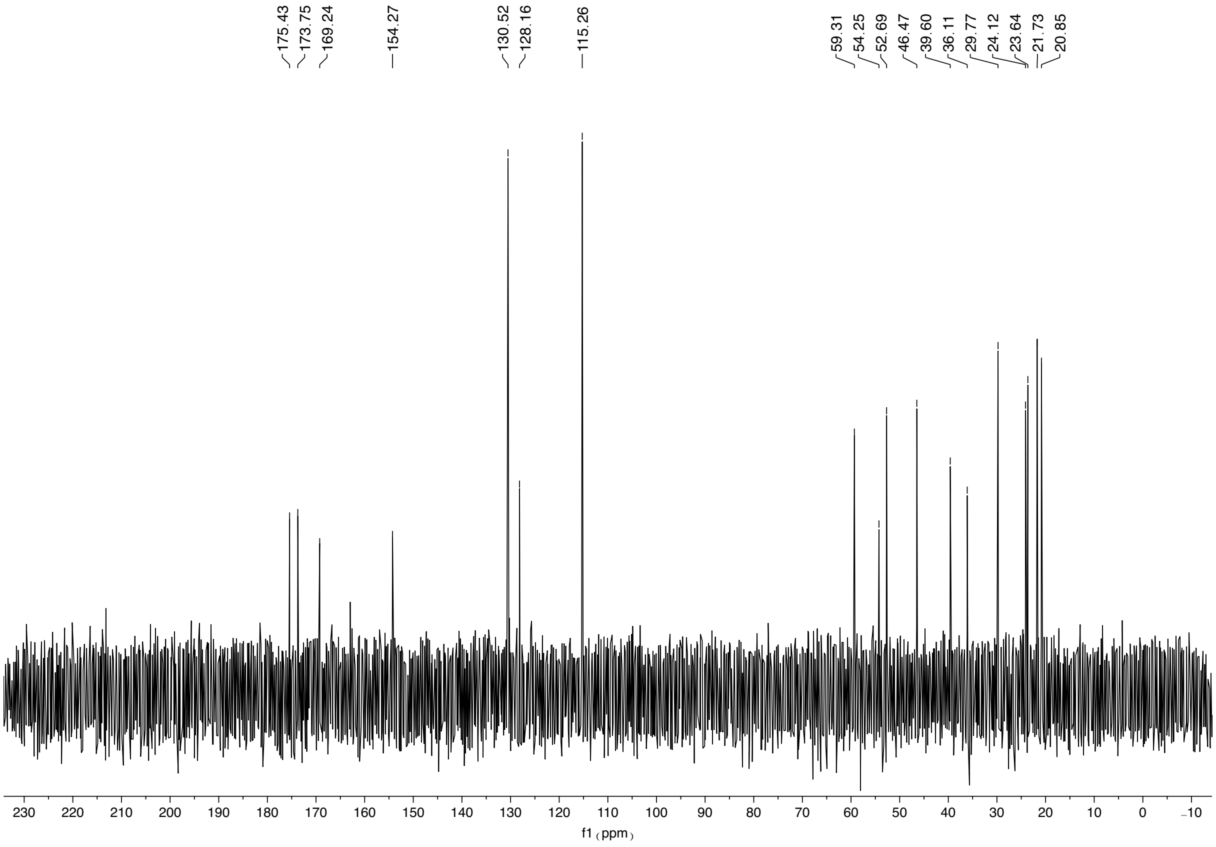

^{1}H and ^{13}C-NMR of H-D-Pro-D-Leu-CyLeu-NH$_2$ · TFA (UTS-74):

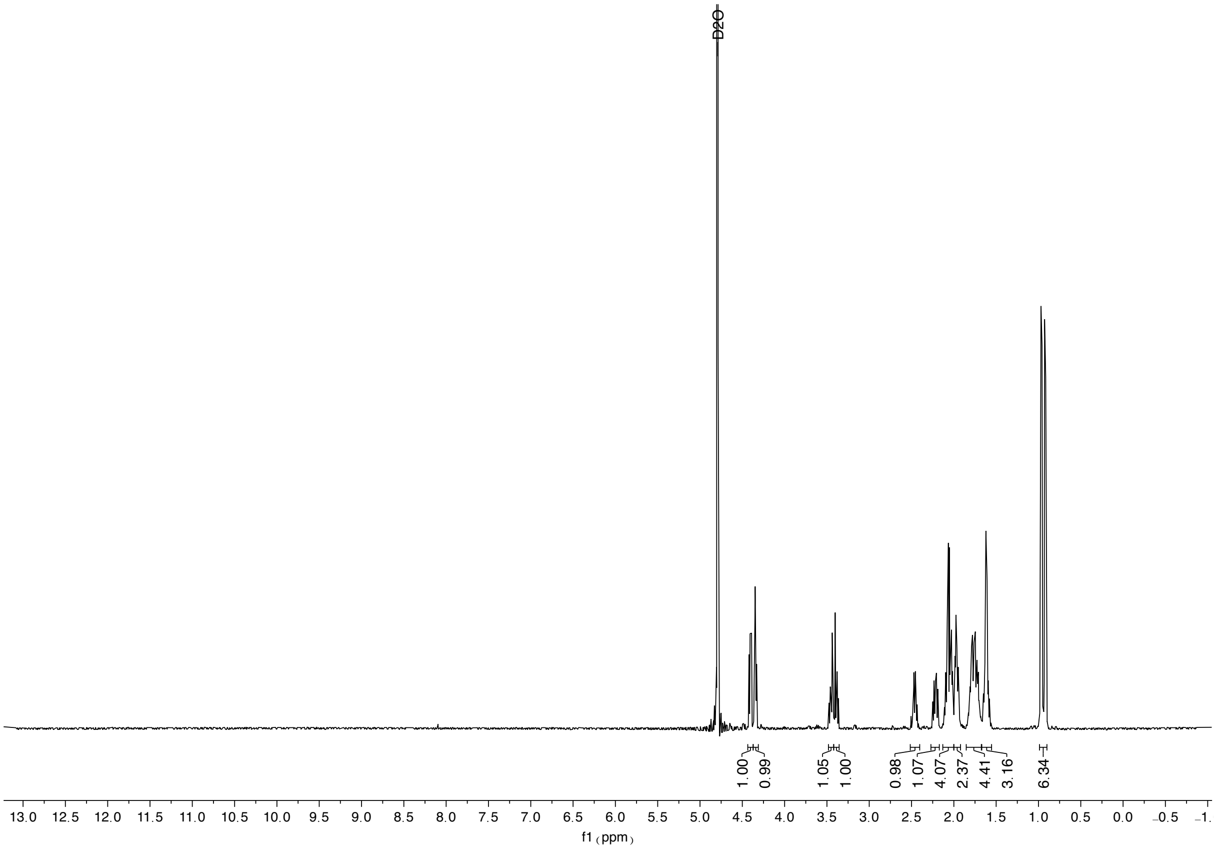

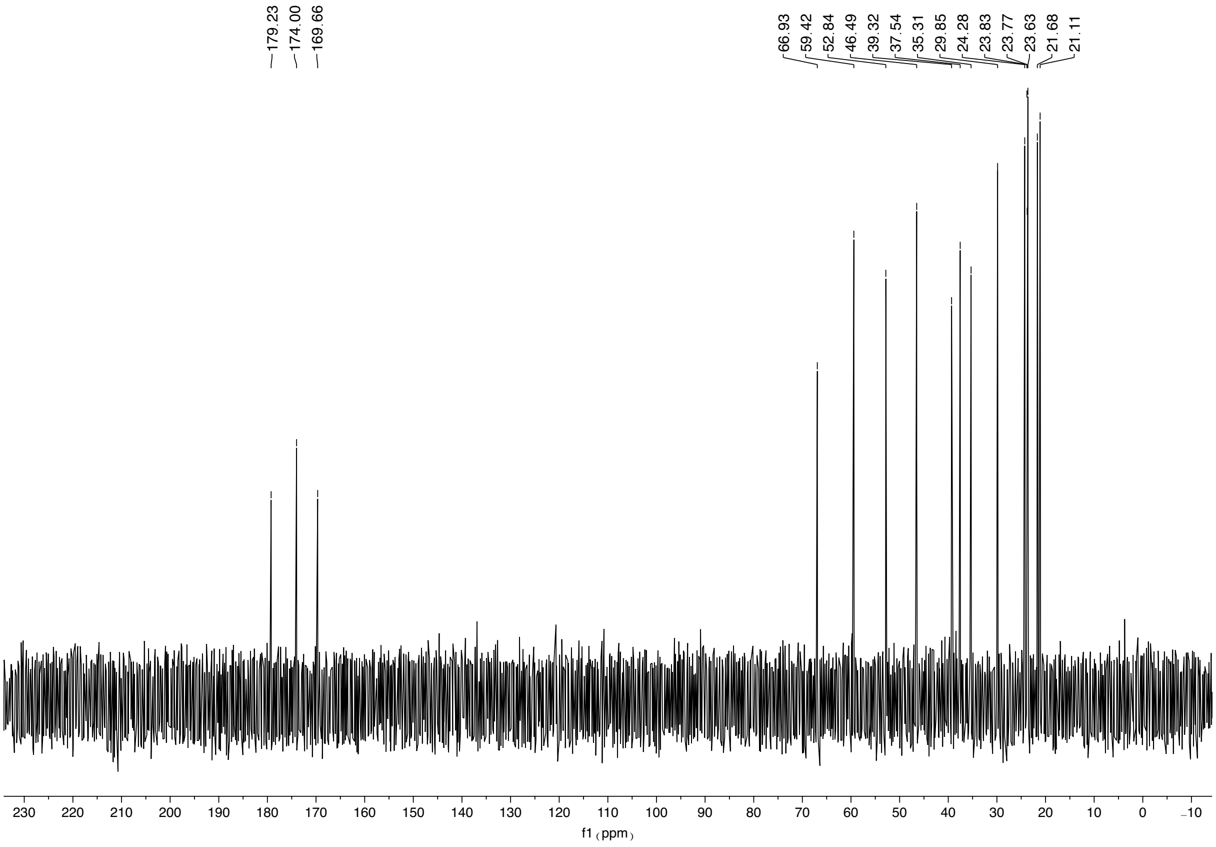

^{1}H and ^{13}C-NMR of H-D-Pro-D-Leu-Abz-NH_2 · TFA (UTS-75):

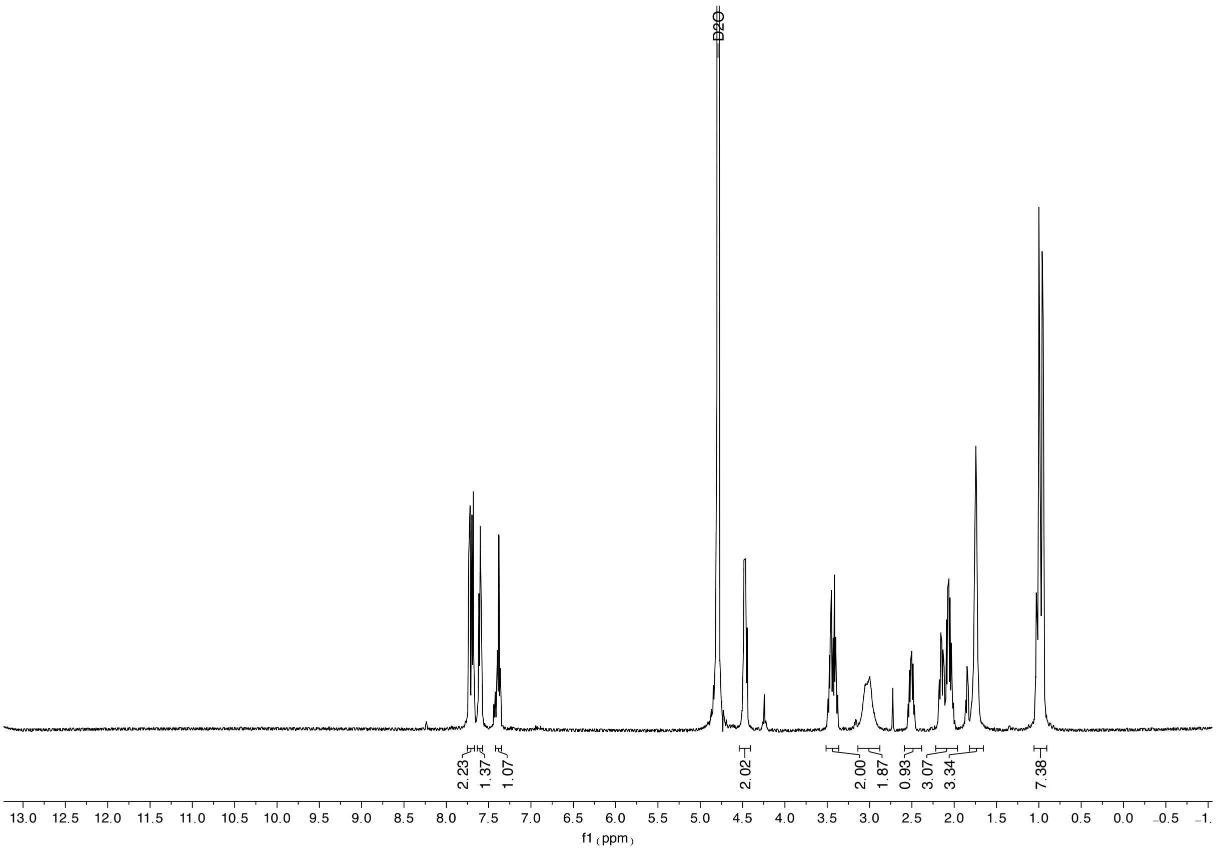

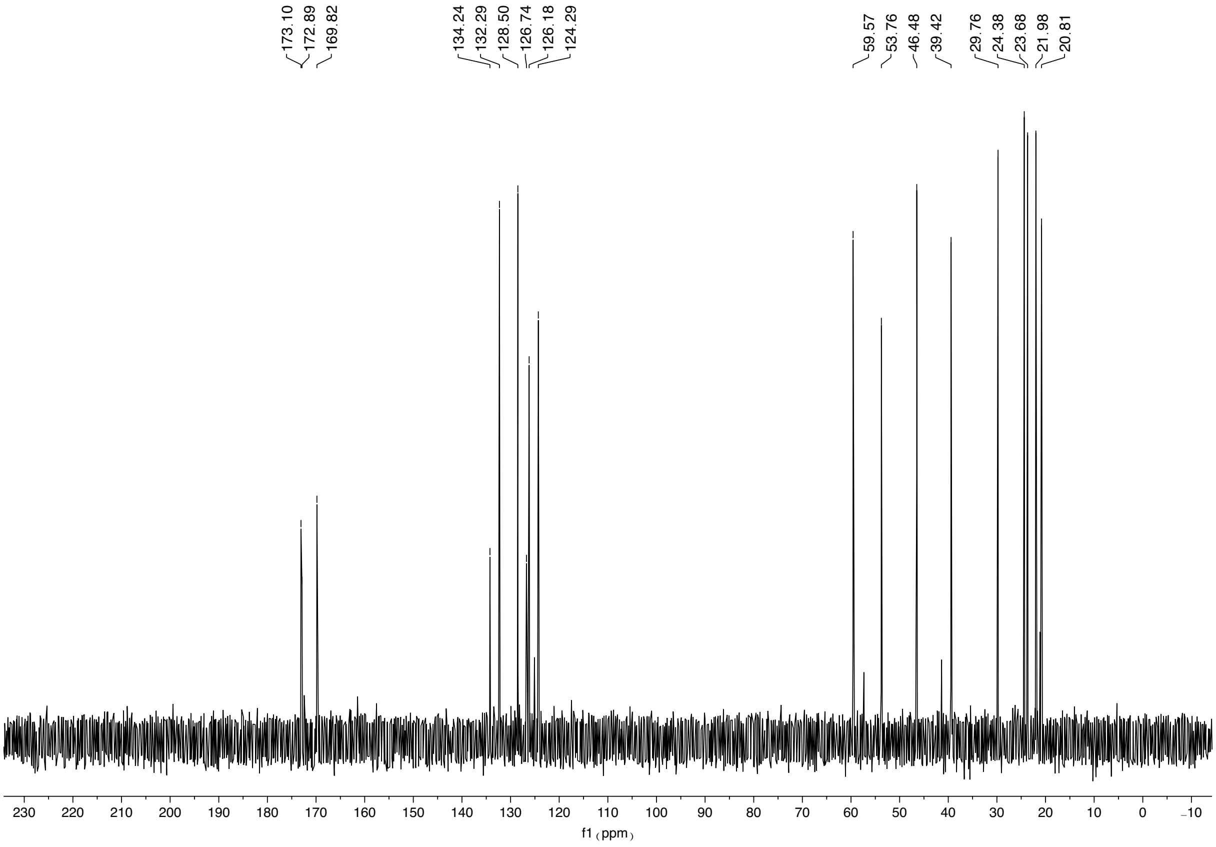

^{1}H and ^{13}C-NMR of H-D-Pro-D-Leu-D-Ind-NH_2 · TFA (UTS-76):

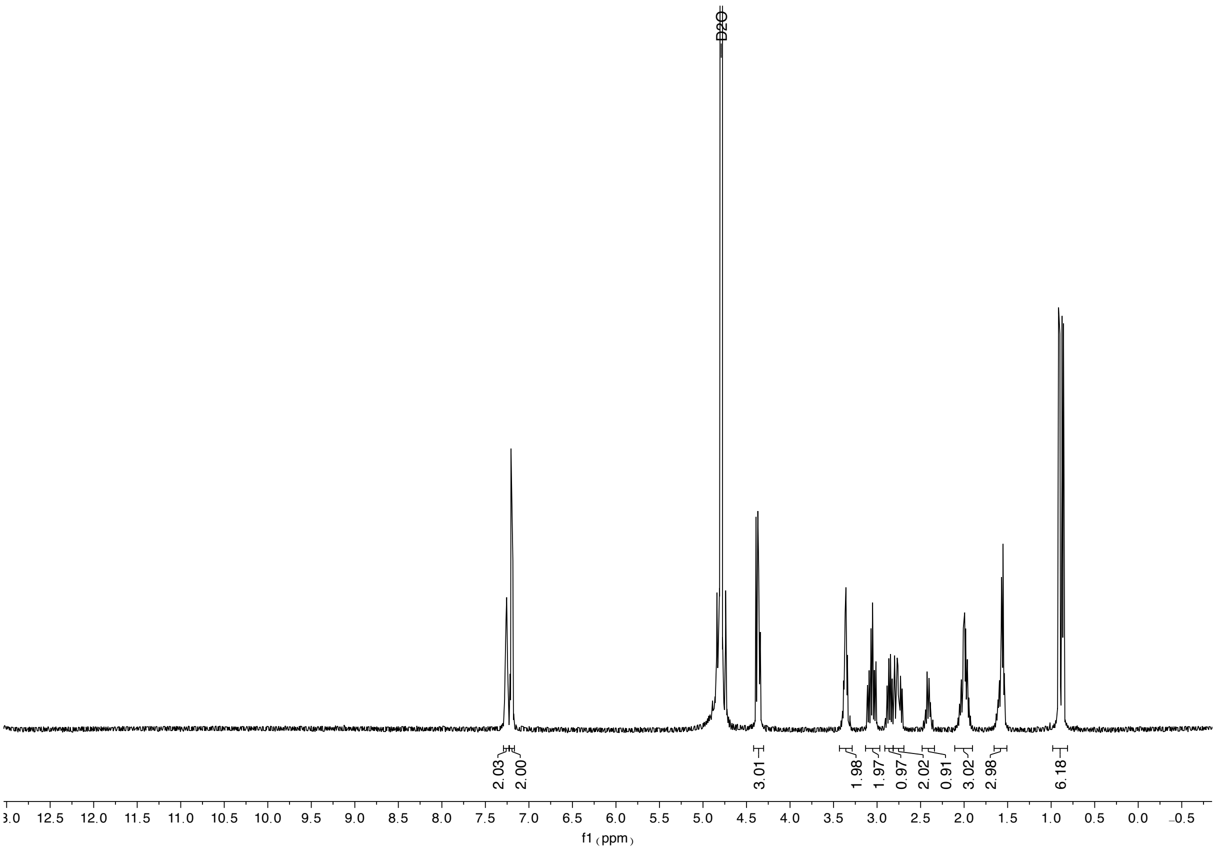

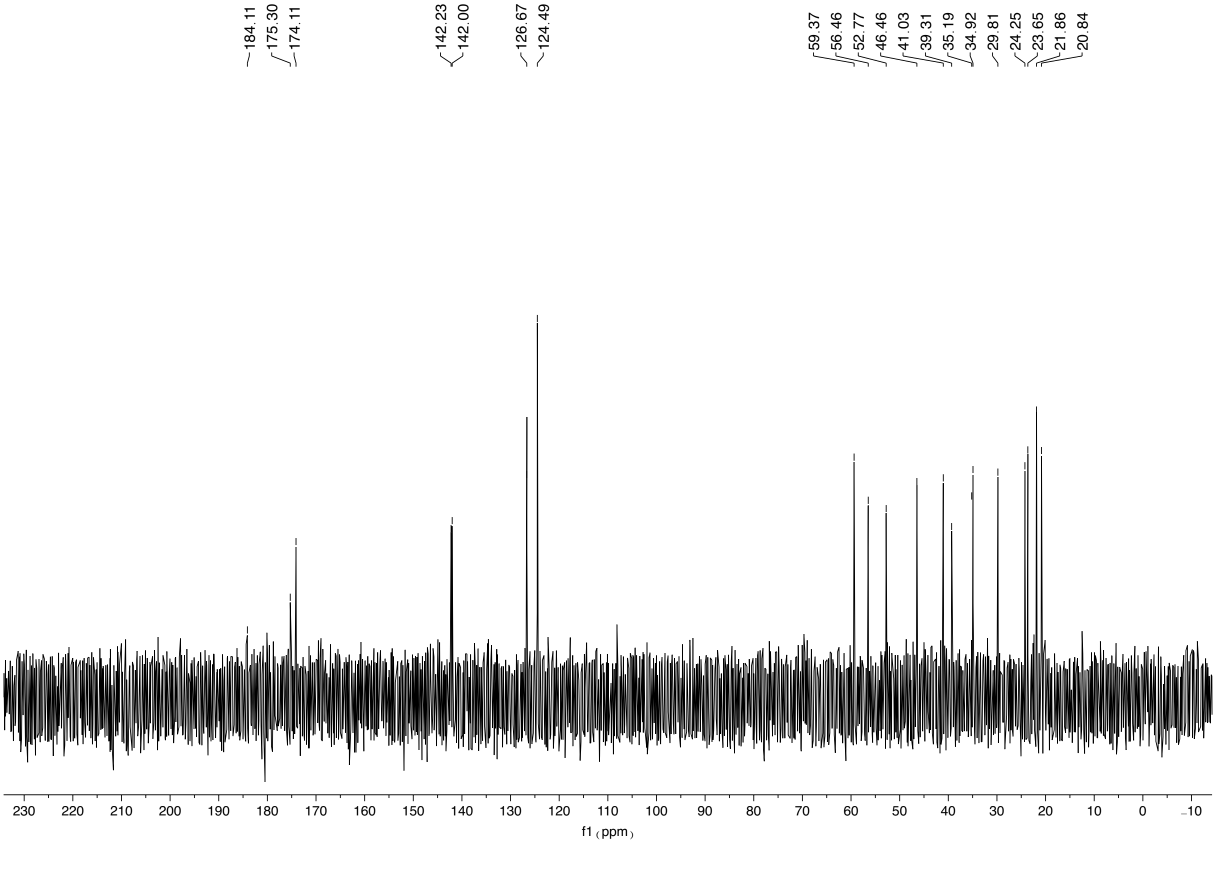

^{1}H and ^{13}C-NMR of H-D-Pro-L-Gln-D-Leu-NH$_2$ · TFA (UTS-77):

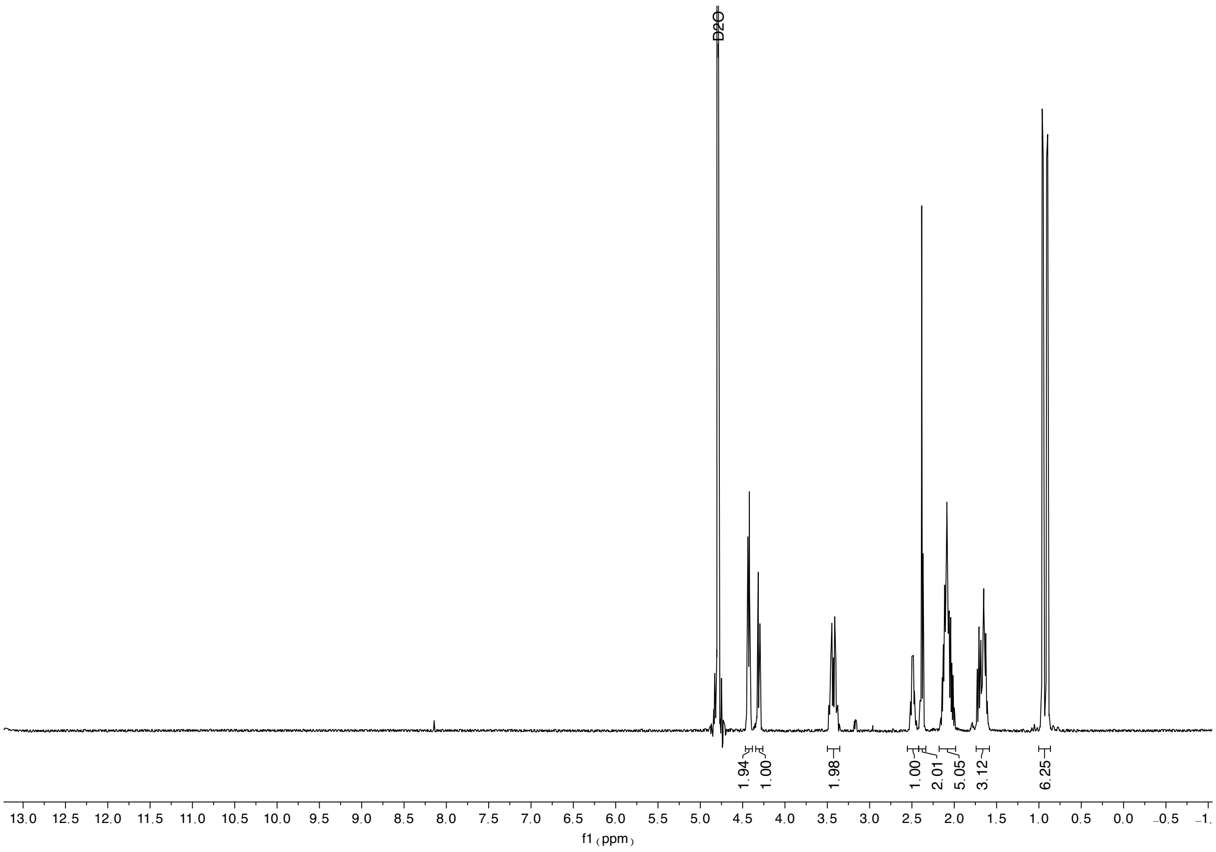

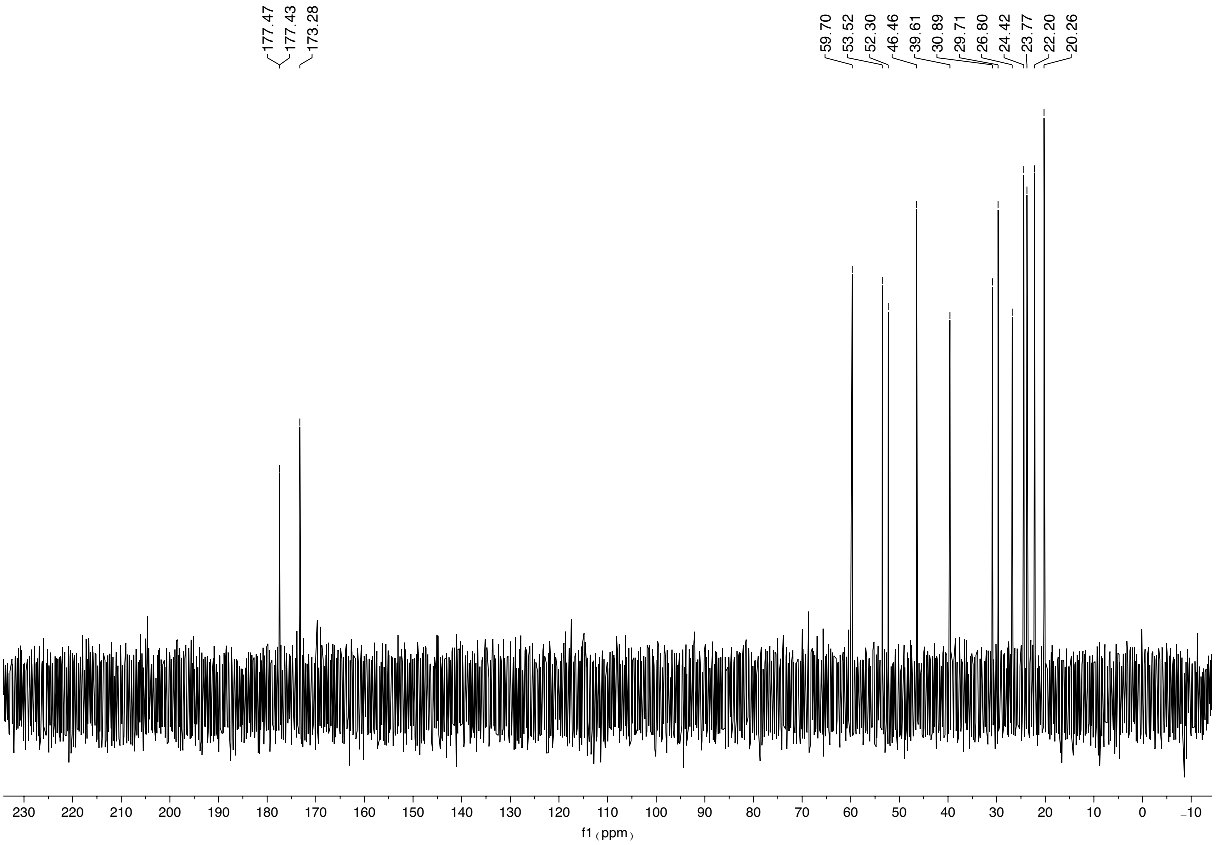

^{1}H and ^{13}C-NMR of H-D-Pro-D-Gln-D-Leu-NH$_2$ · TFA (UTS-78):

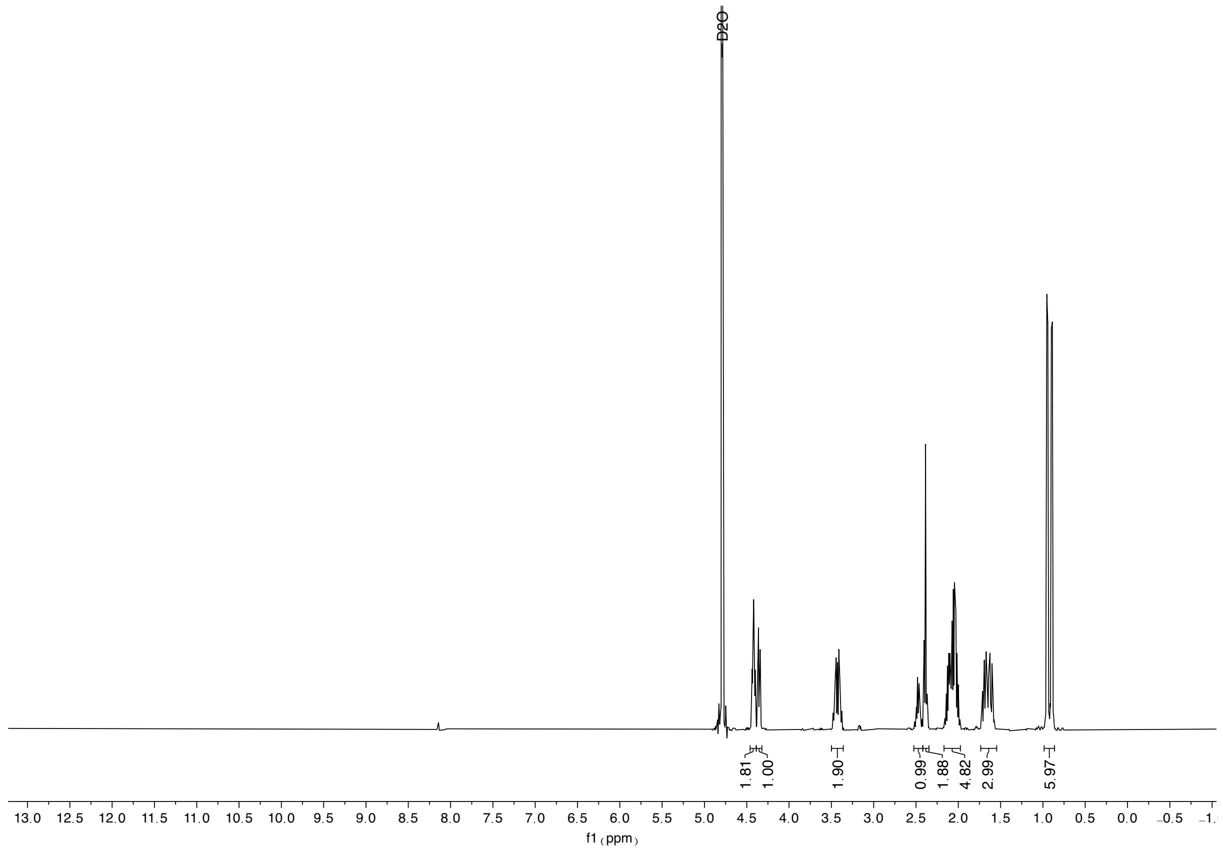

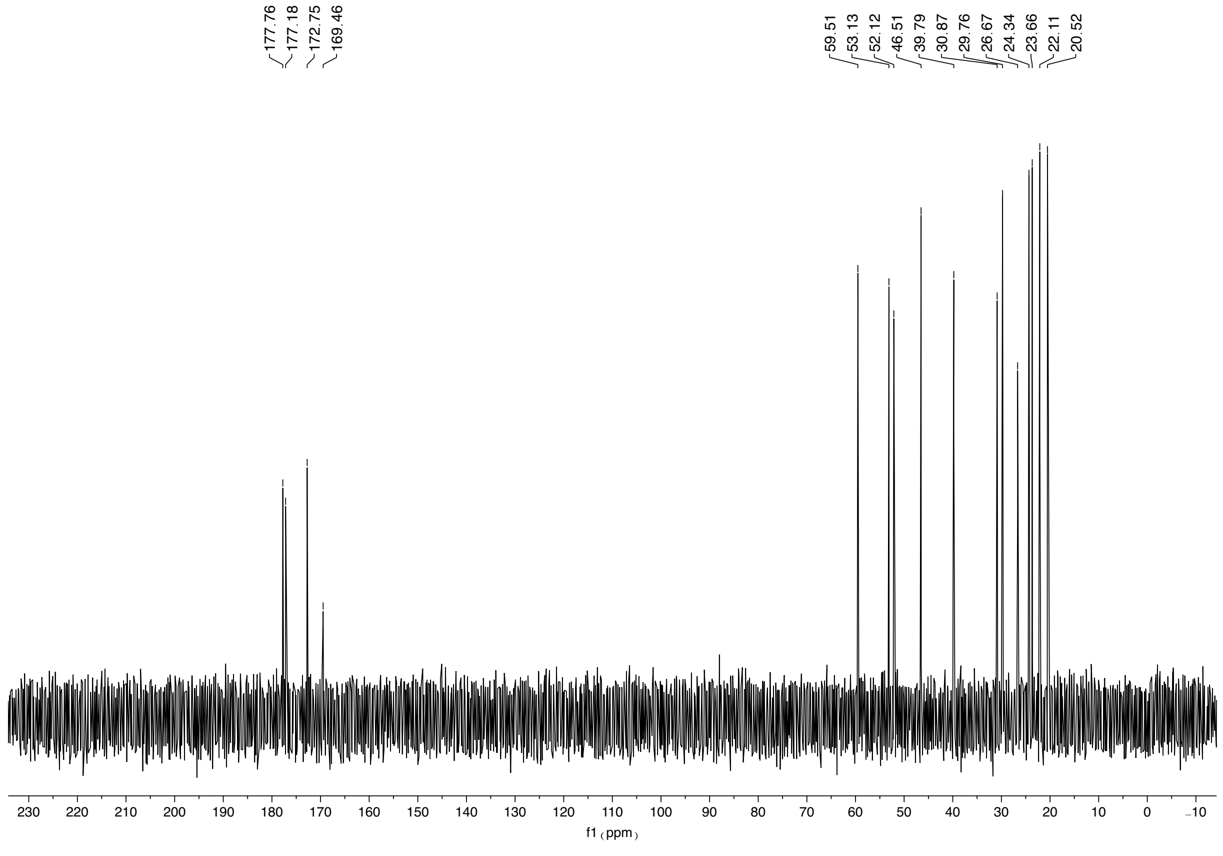

^{1}H and ^{13}C-NMR of H-D-Pro-L-Glu-D-Leu-NH$_2$ · TFA (UTS-79)

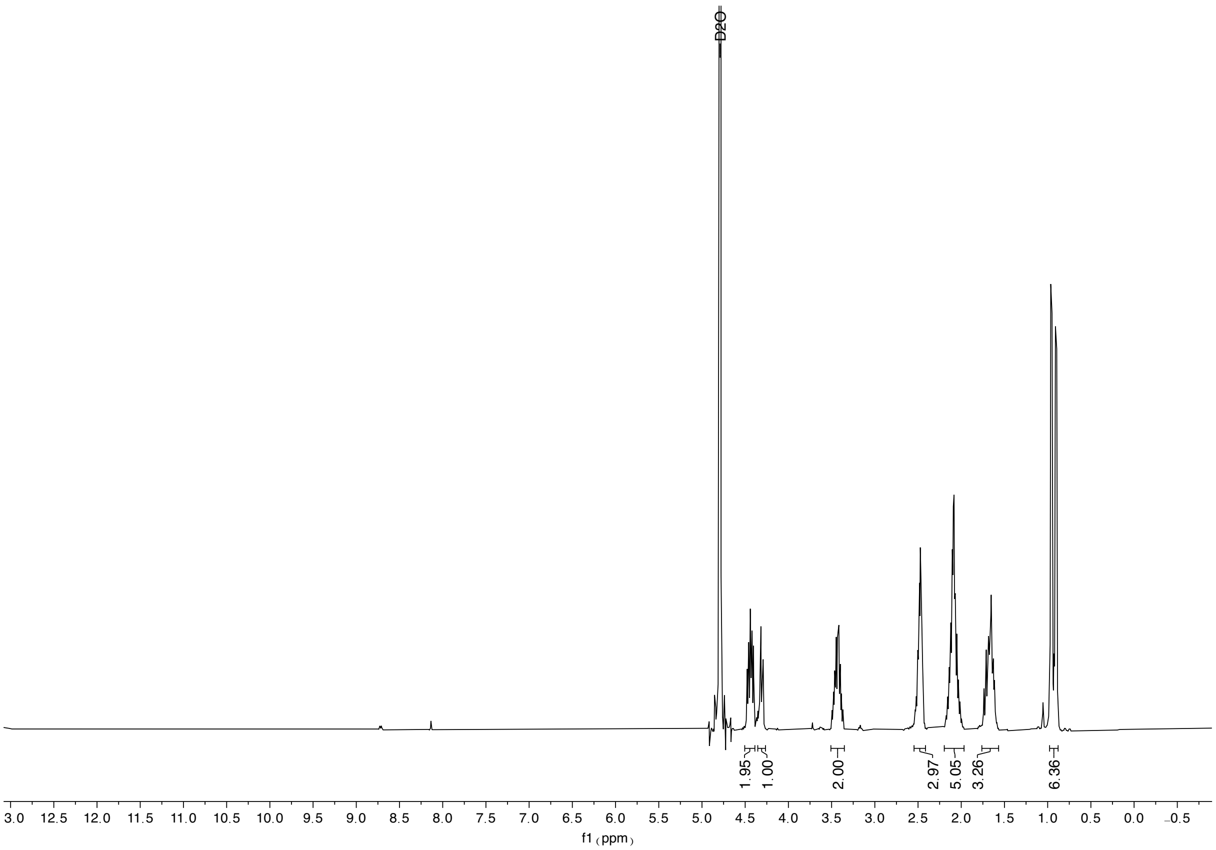

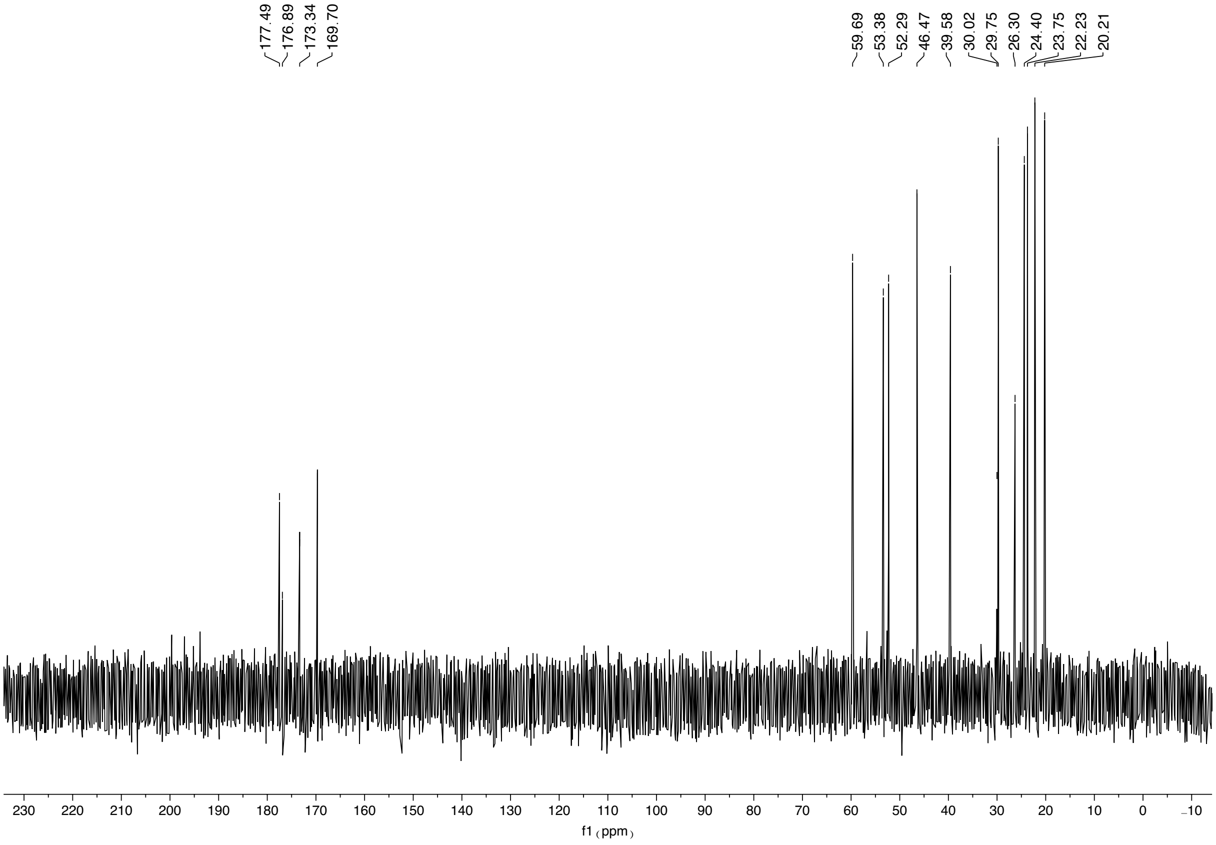

^{1}H and ^{13}C-NMR of H-D-Pro-D-Glu-D-Leu-NH_2 · TFA (UTS-80):

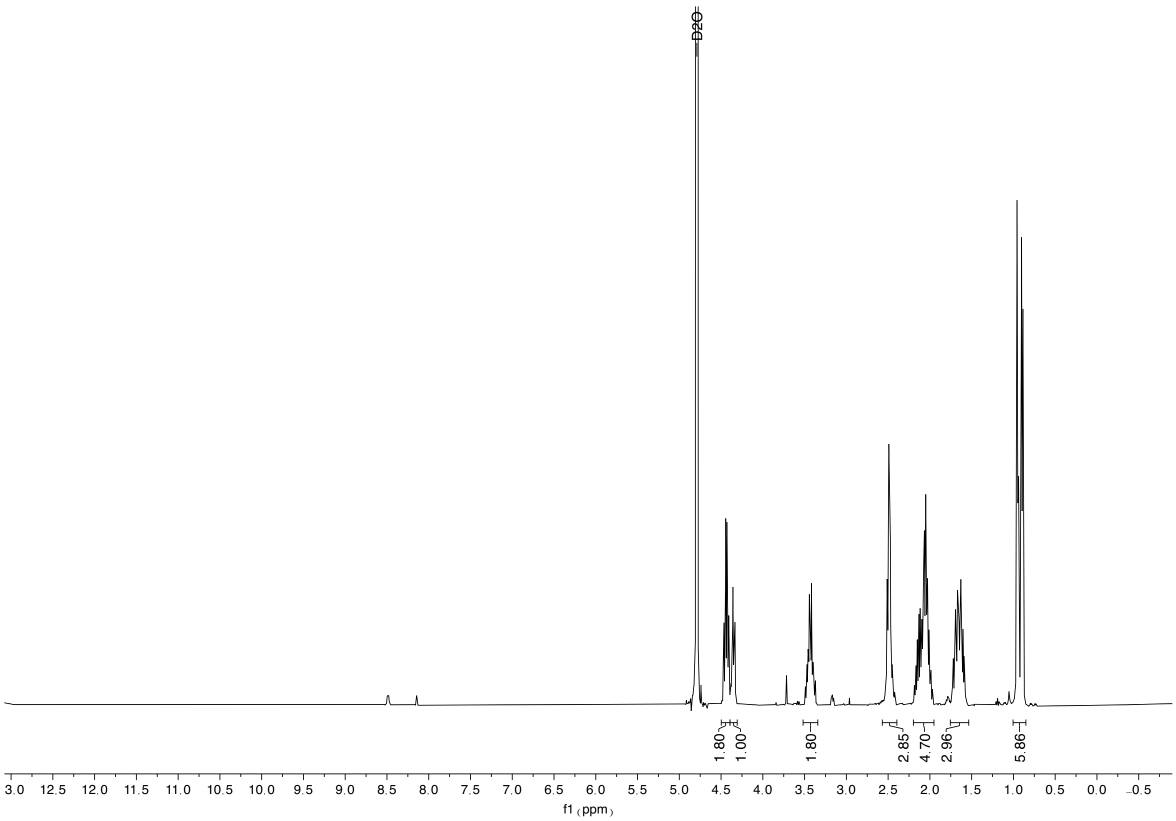

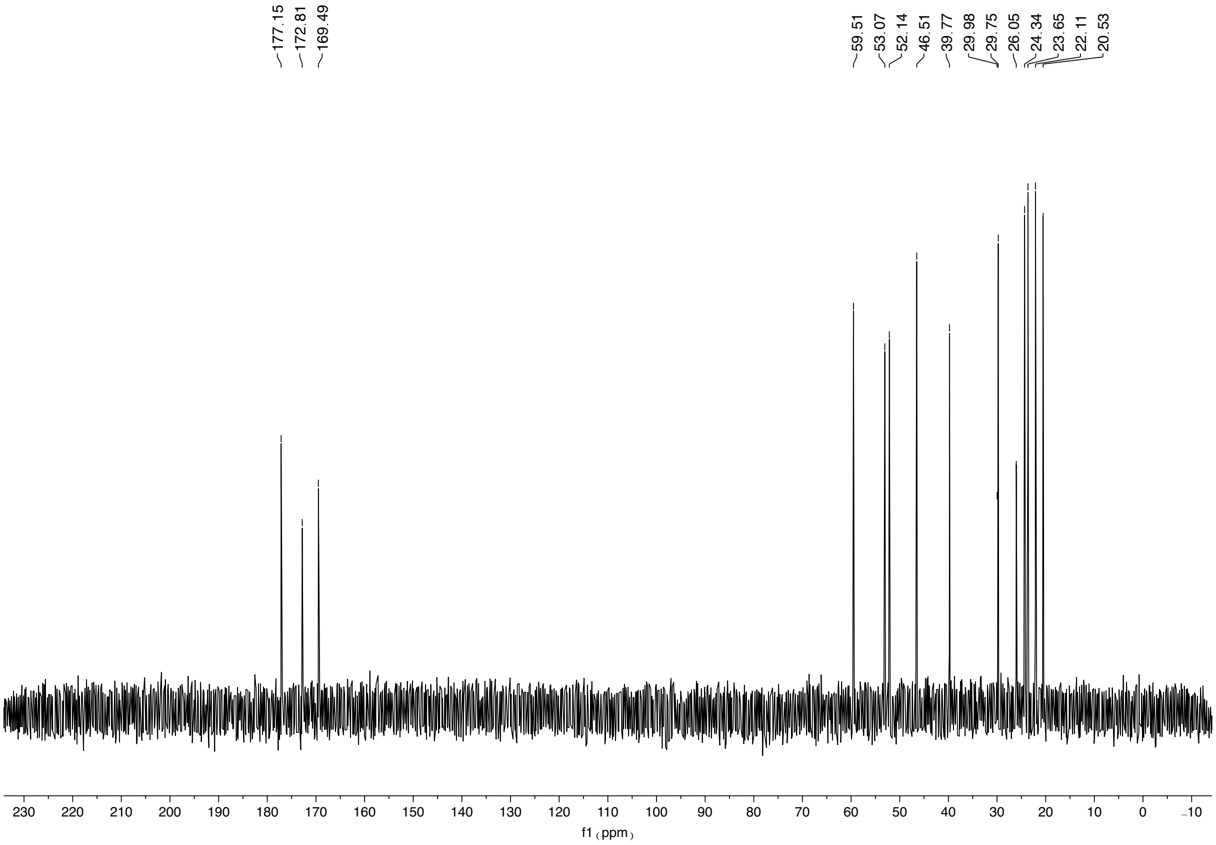

^{1}H and ^{13}C-NMR of H-D-Pro-L-Tyr-D-Leu-NH₂ · TFA (UTS-81):

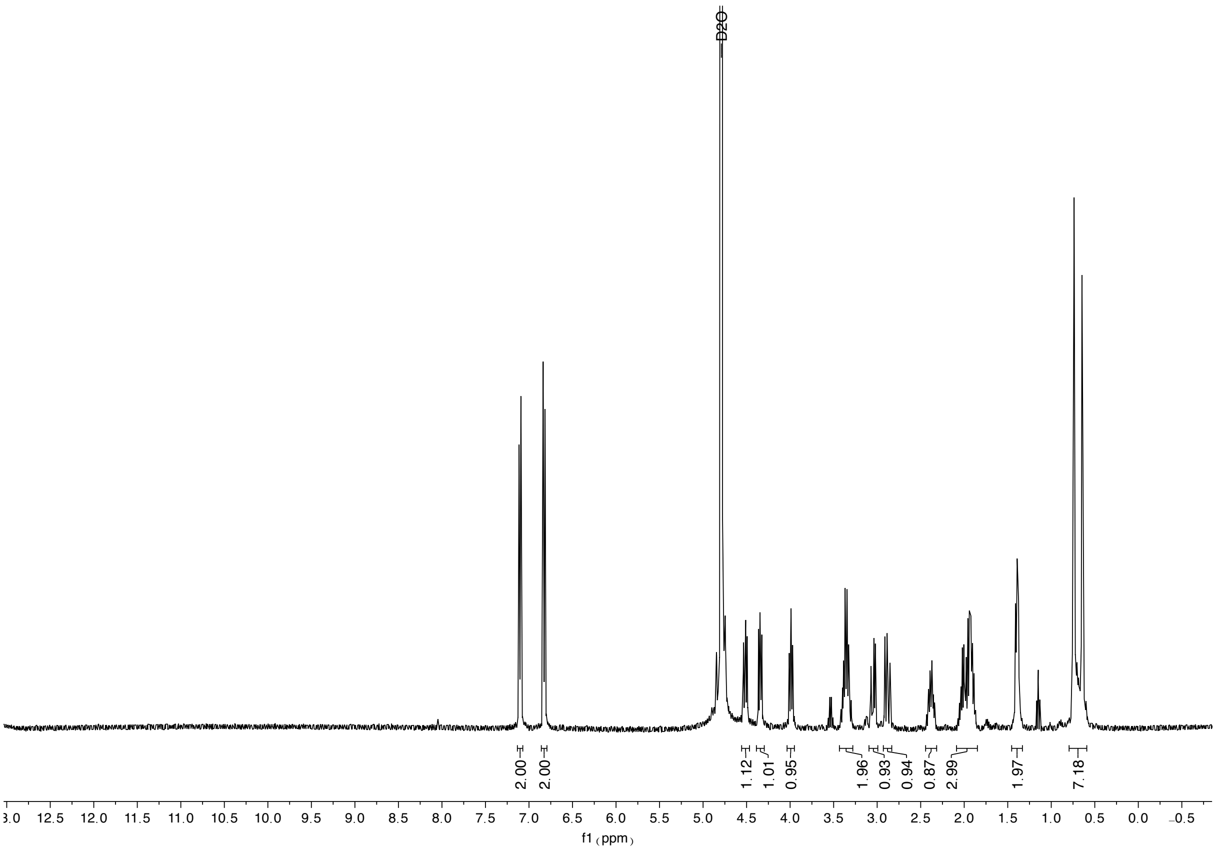

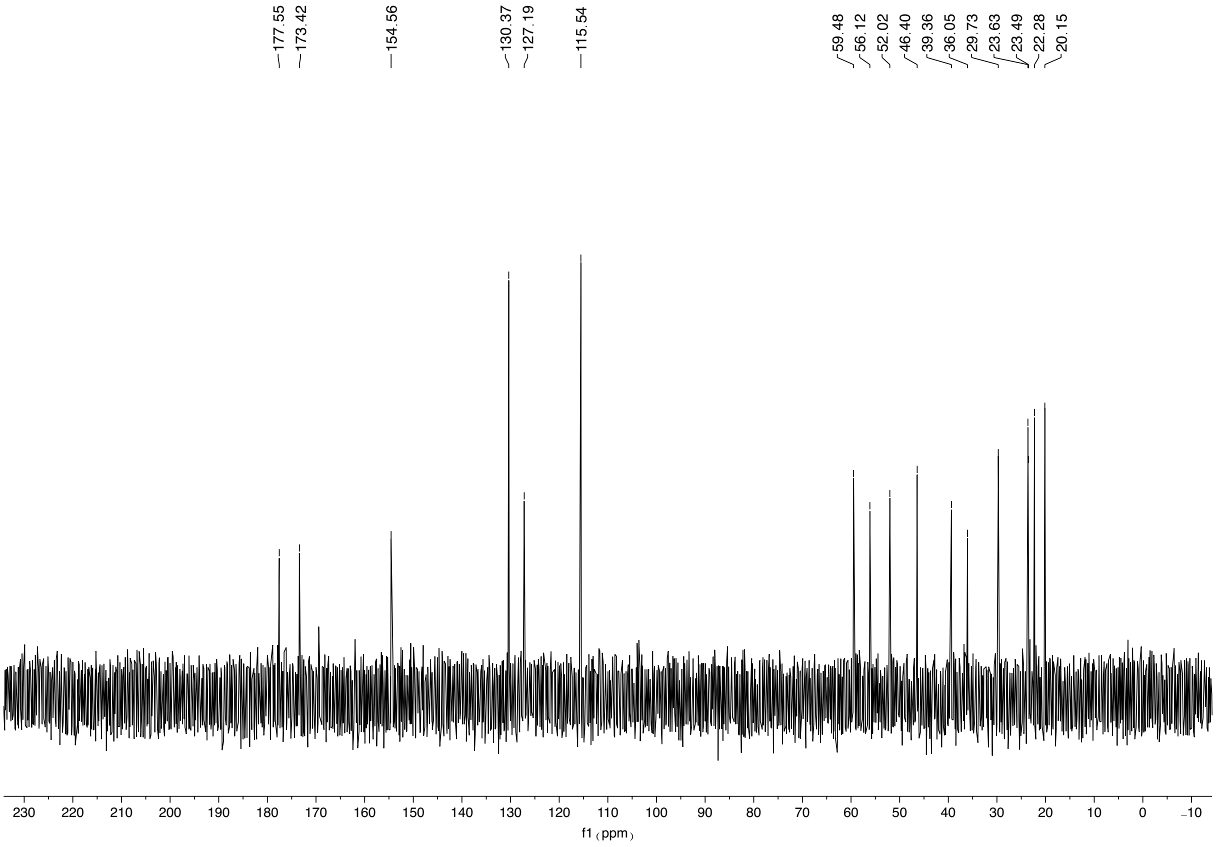

^{1}H and ^{13}C-NMR of H-D-Pro-D-Tyr-D-Leu-NH_2 · TFA (UTS-82):

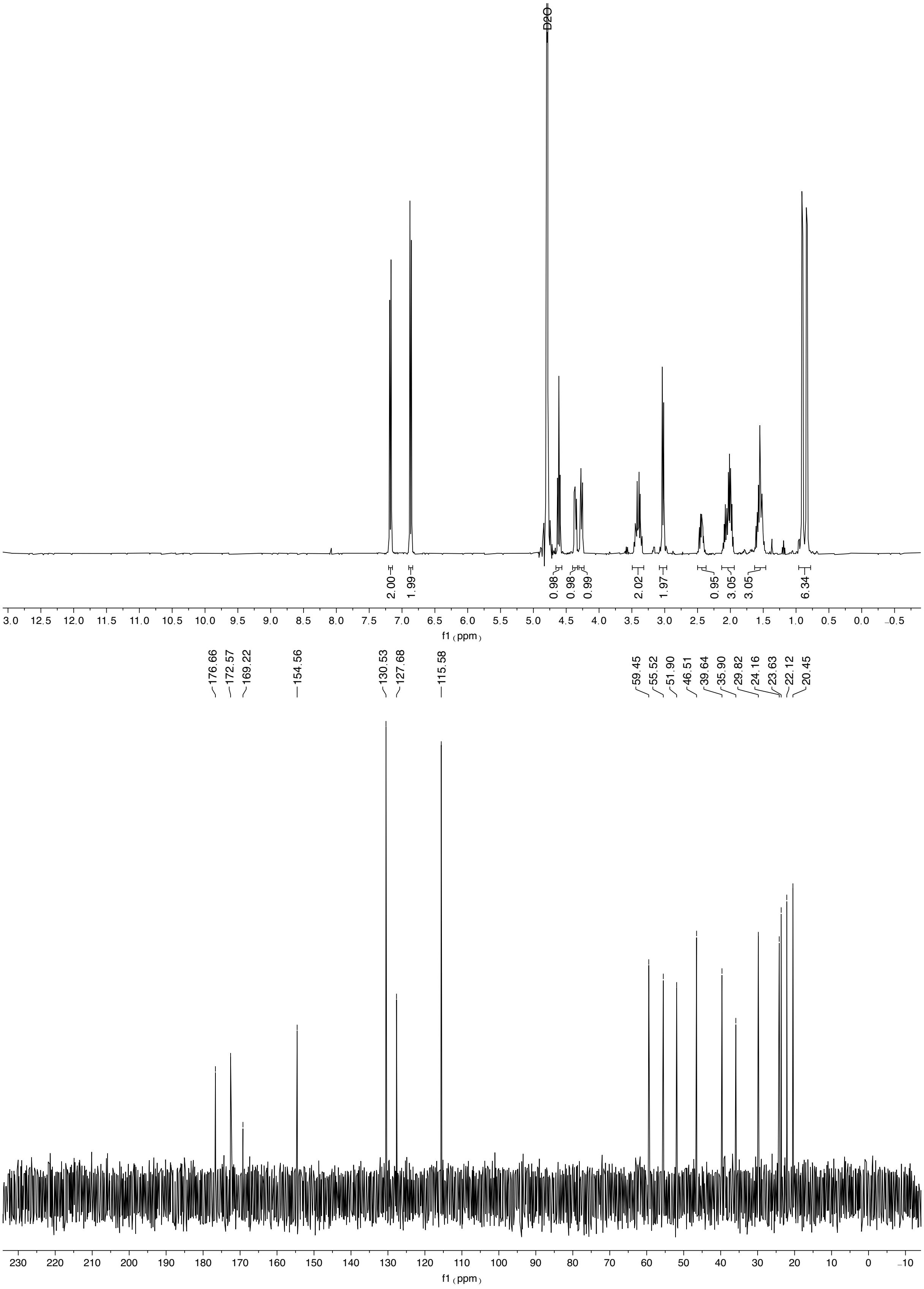

^{1}H and ^{13}C-NMR of H-D-Pro-CyLeu-D-Leu-NH$_2$ · TFA (UTS-83):

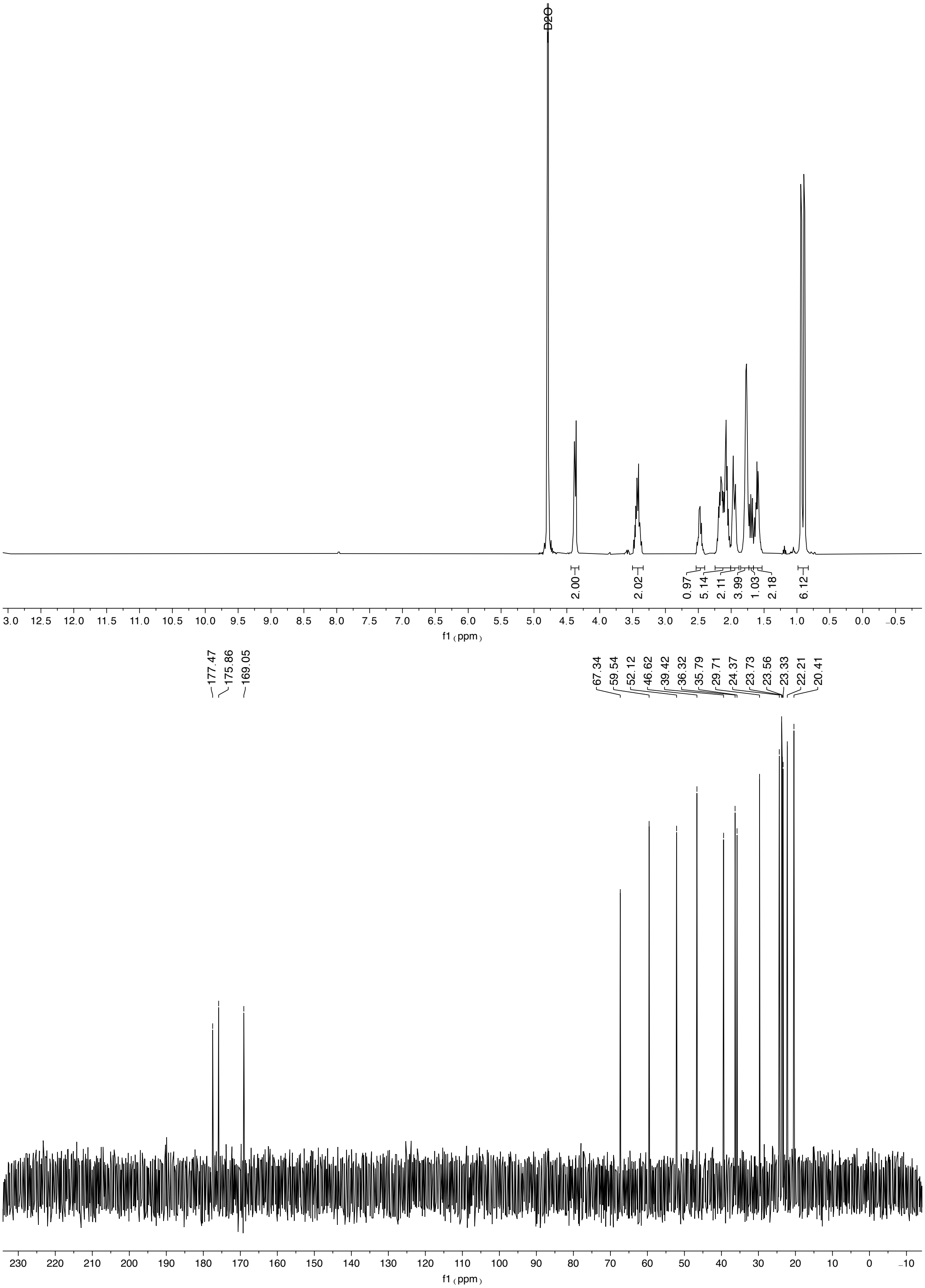

^{1}H and ^{13}C-NMR of H-D-Pro-Abz-D-Leu-NH_2 · TFA (UTS-84):

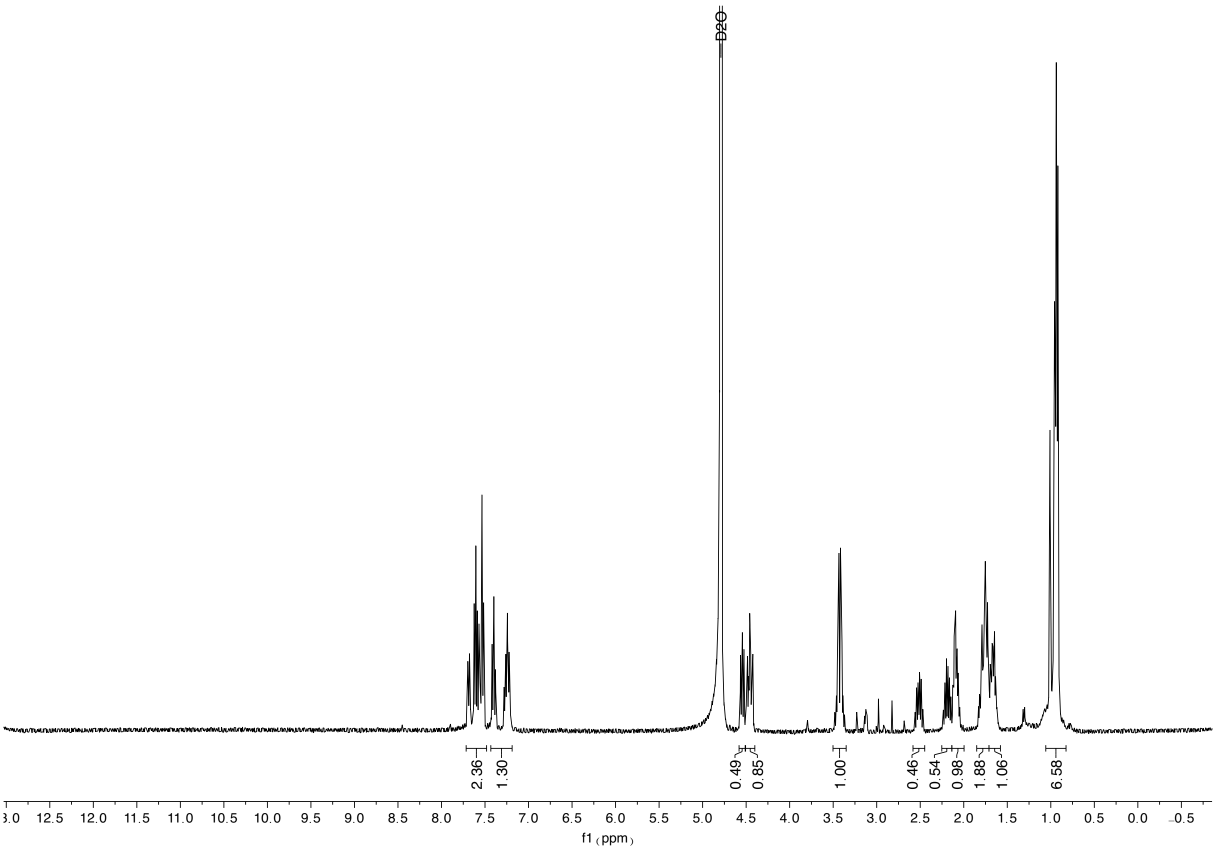

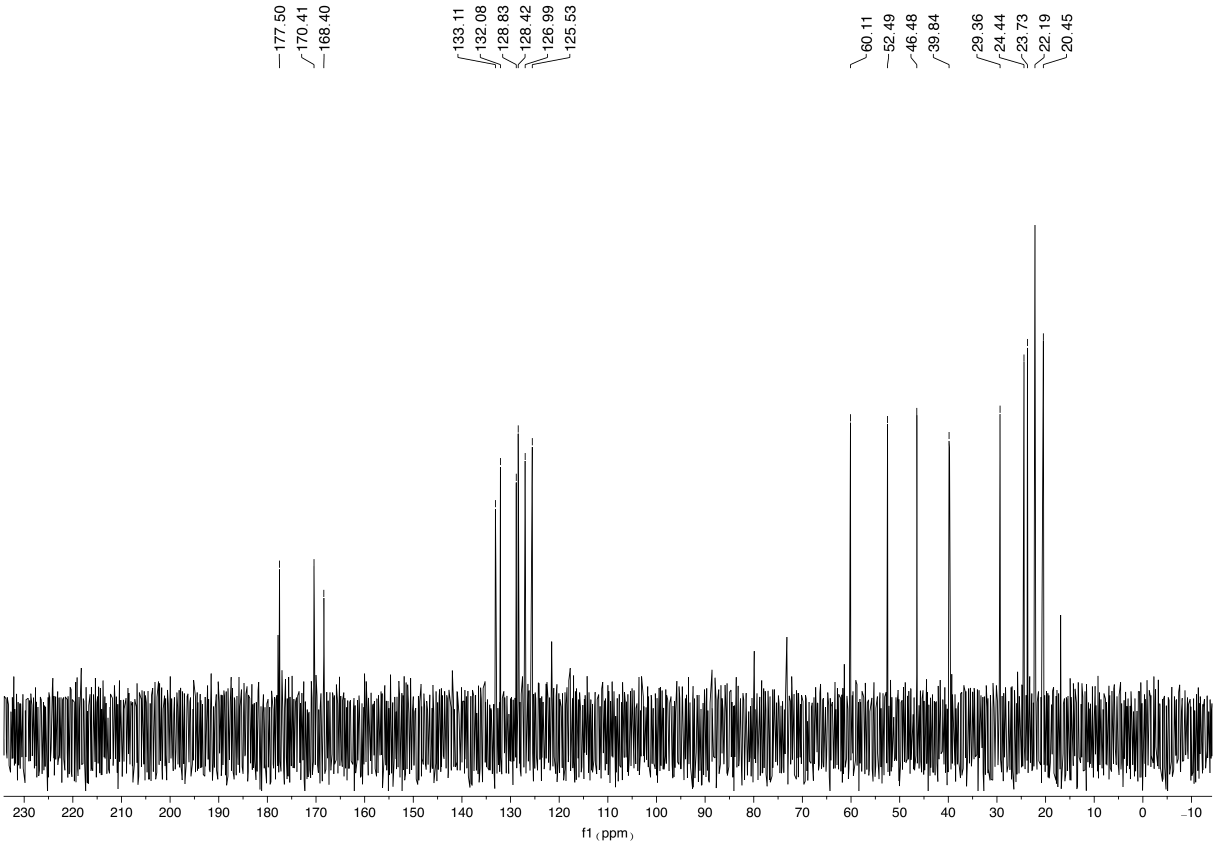

^{1}H and ^{13}C-NMR of H-D-Pro-D-Ind-D-Leu-NH_2 · TFA (UTS-85):

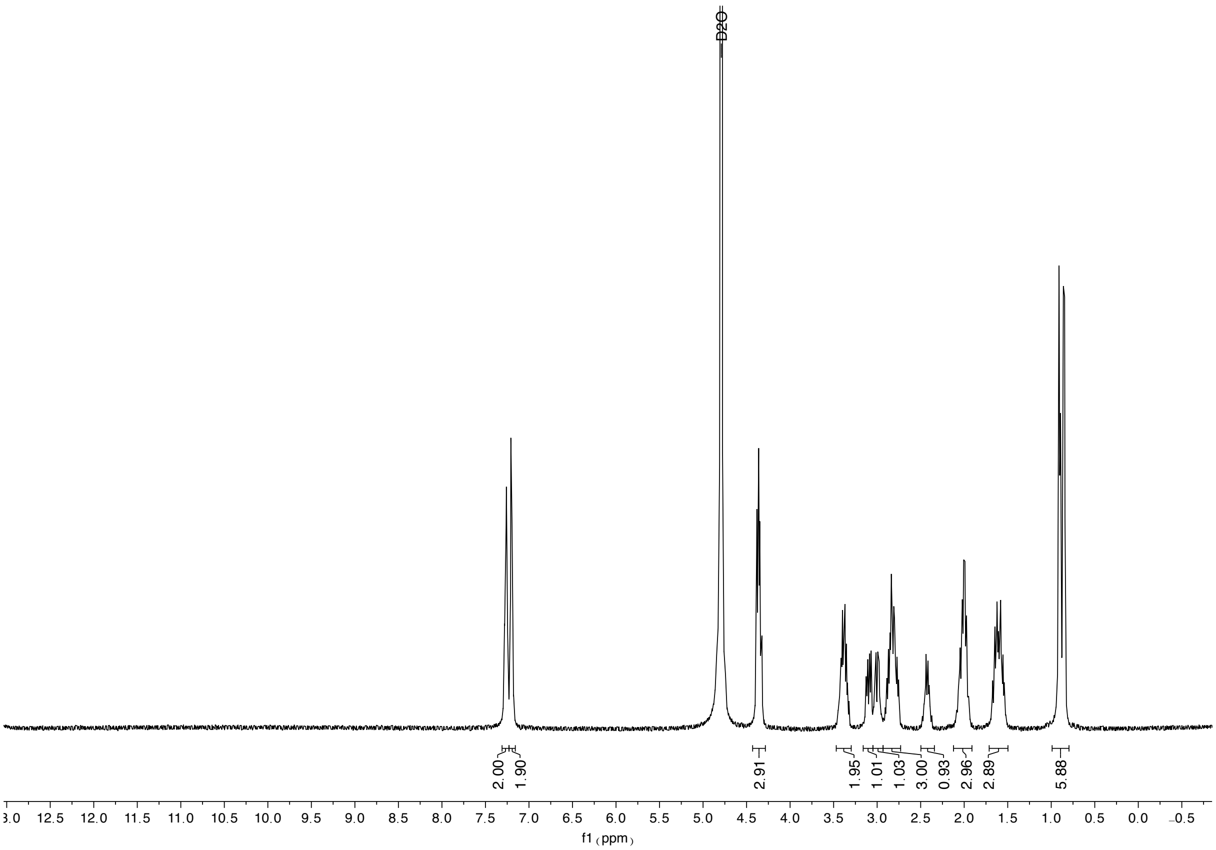

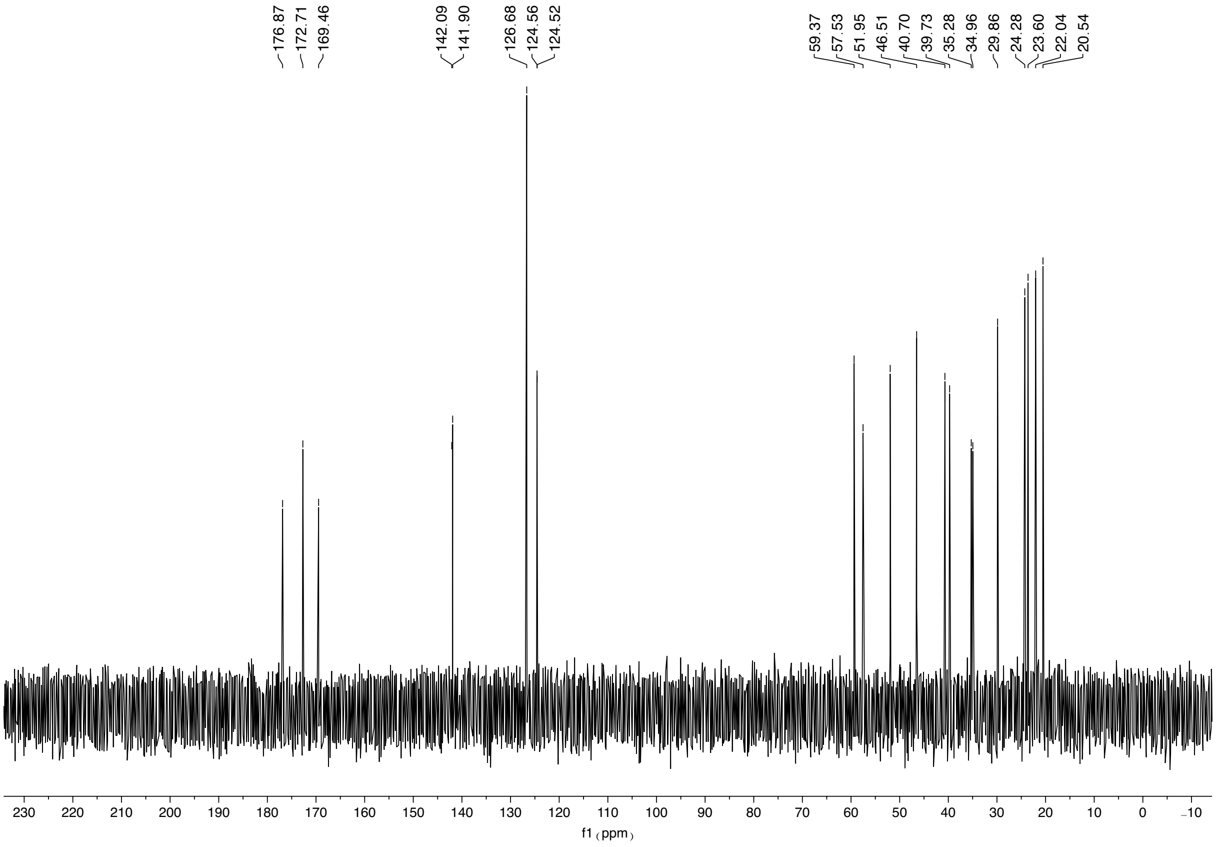

^{1}H and ^{13}C-NMR of H-D-Pro-L-Gln-L-Gln-NH_2 · TFA (UTS-86):

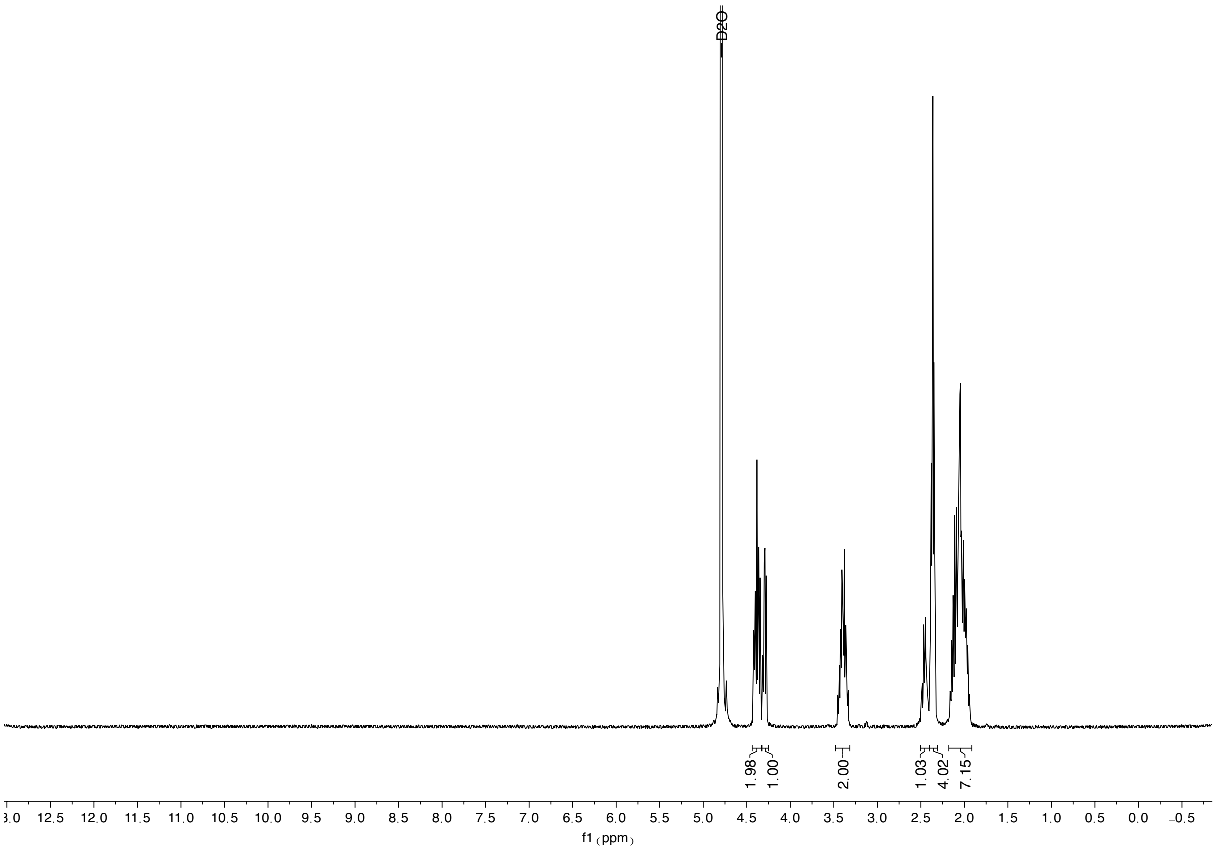

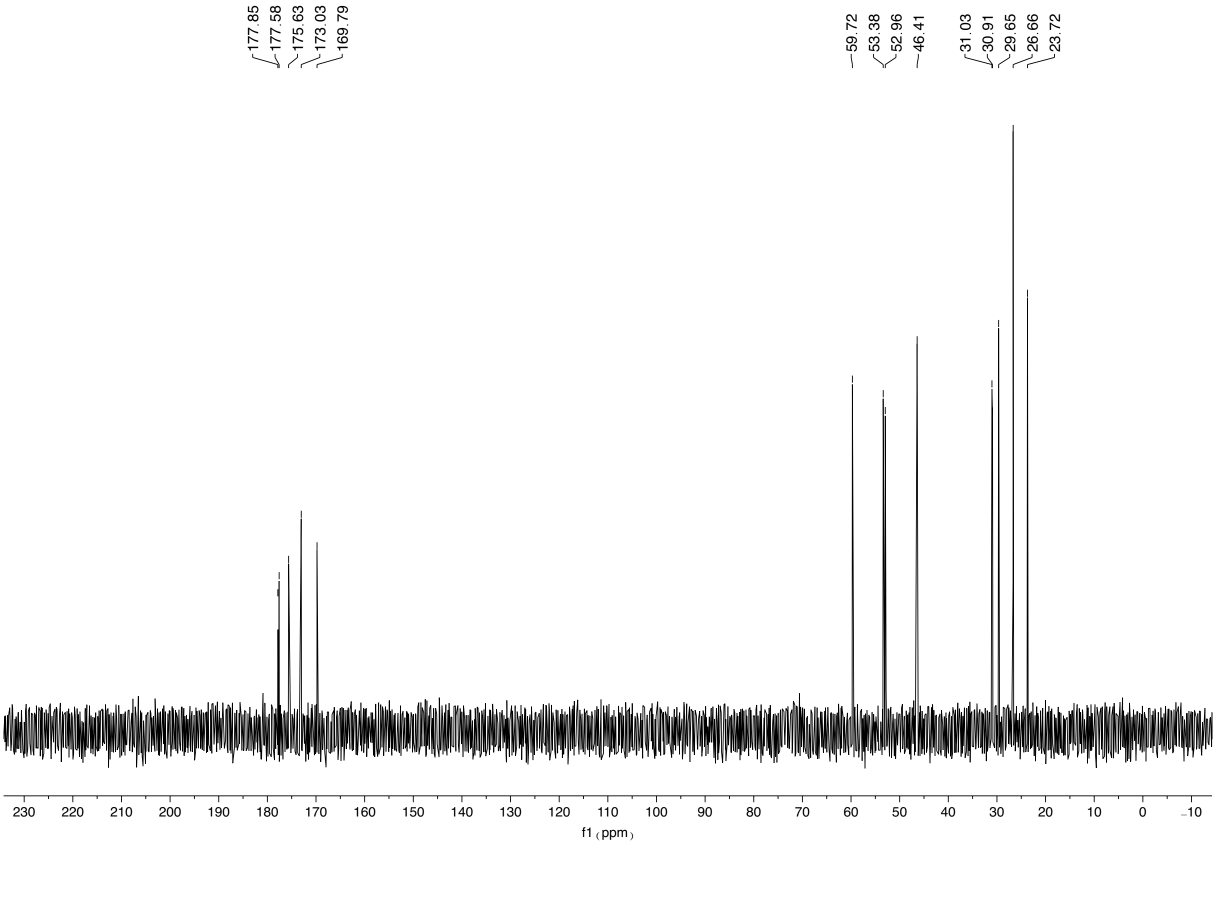

^{1}H and ^{13}C-NMR of H-D-Pro-L-Gln-D-Gln-NH₂ · TFA (UTS-87):

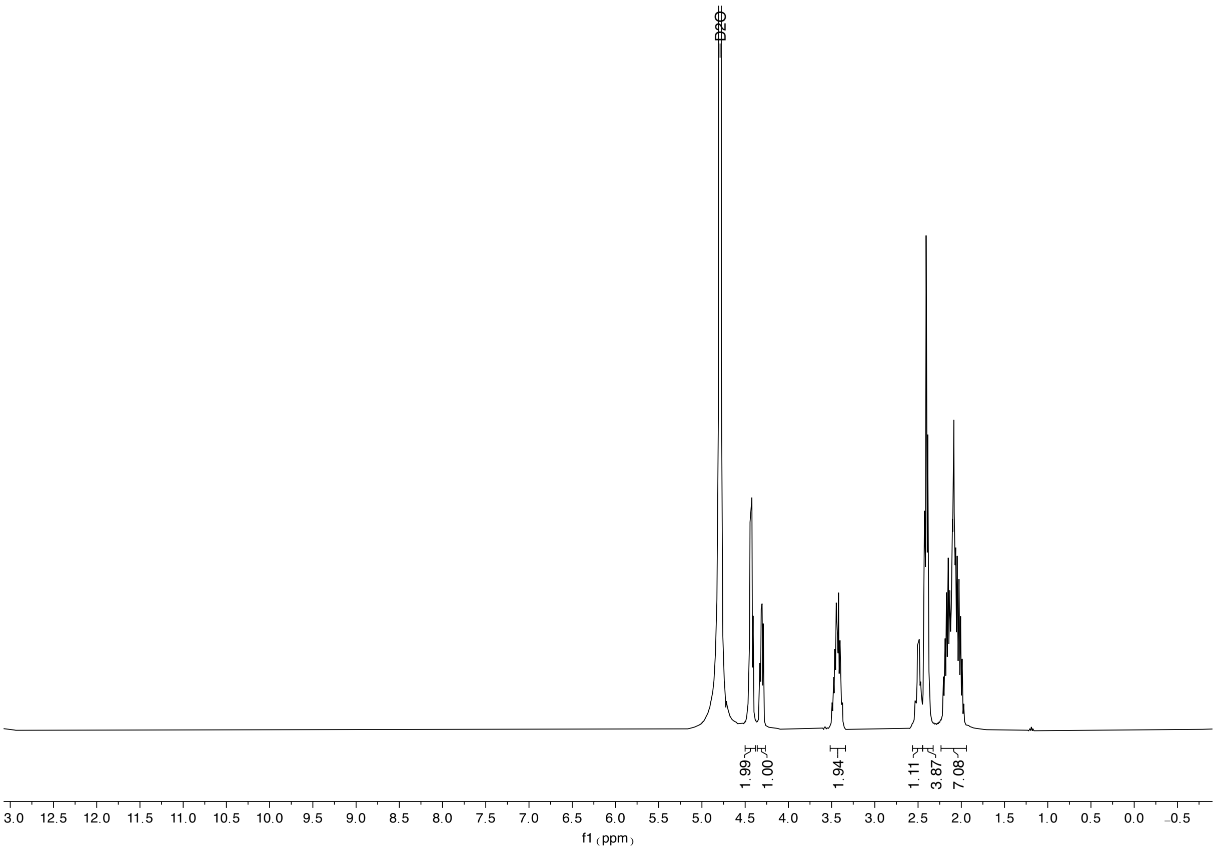

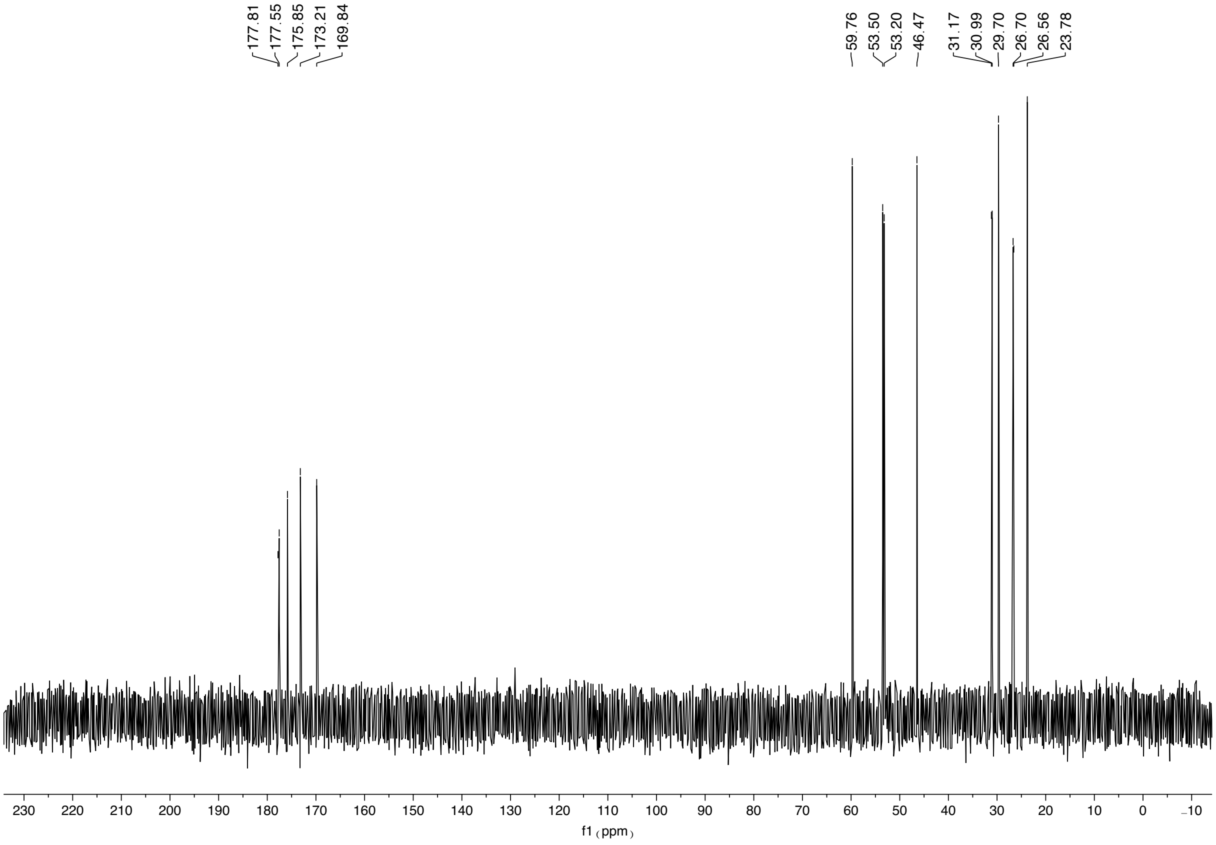

^{1}H and ^{13}C-NMR of H-D-Pro-L-Gln-L-Glu-NH_2 · TFA (UTS-88):

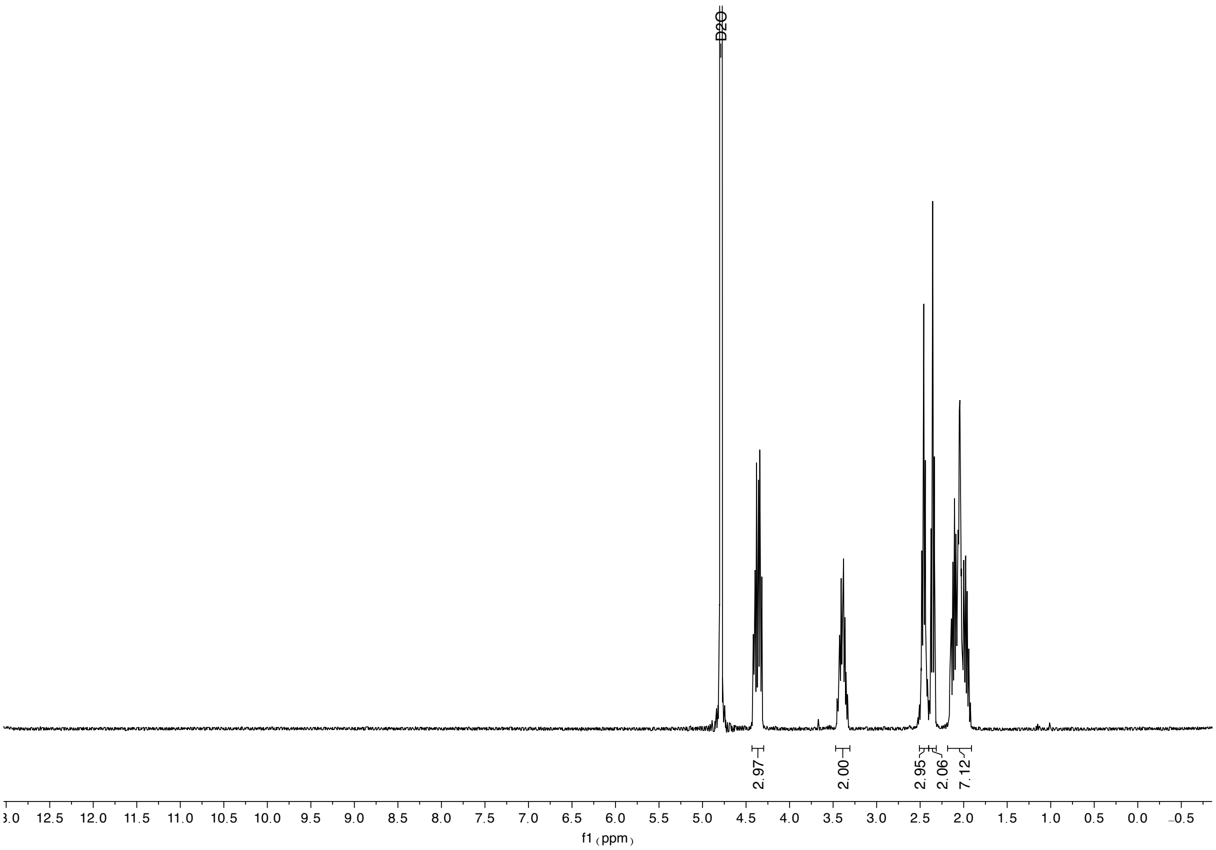

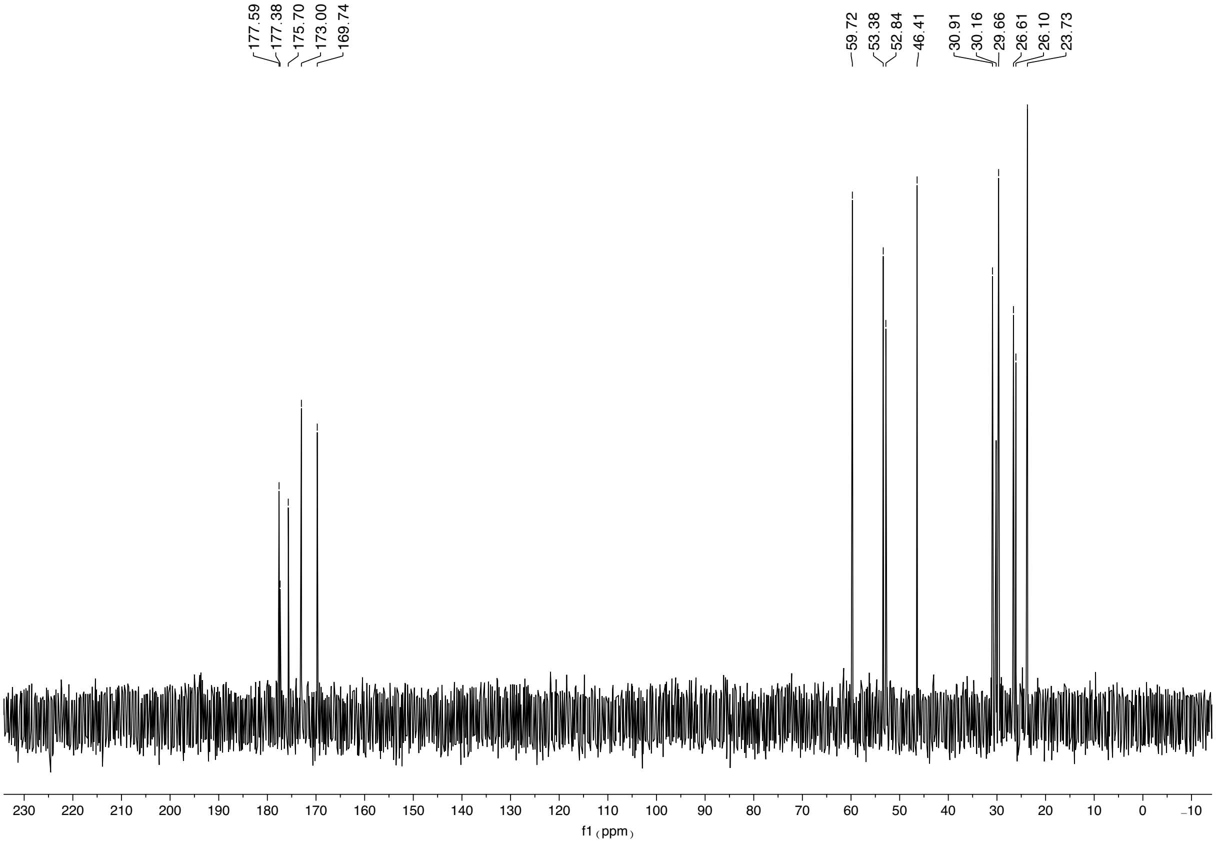

^{1}H and ^{13}C-NMR of H-D-Pro-L-Gln-D-Glu-NH$_2$ · TFA (UTS-89):

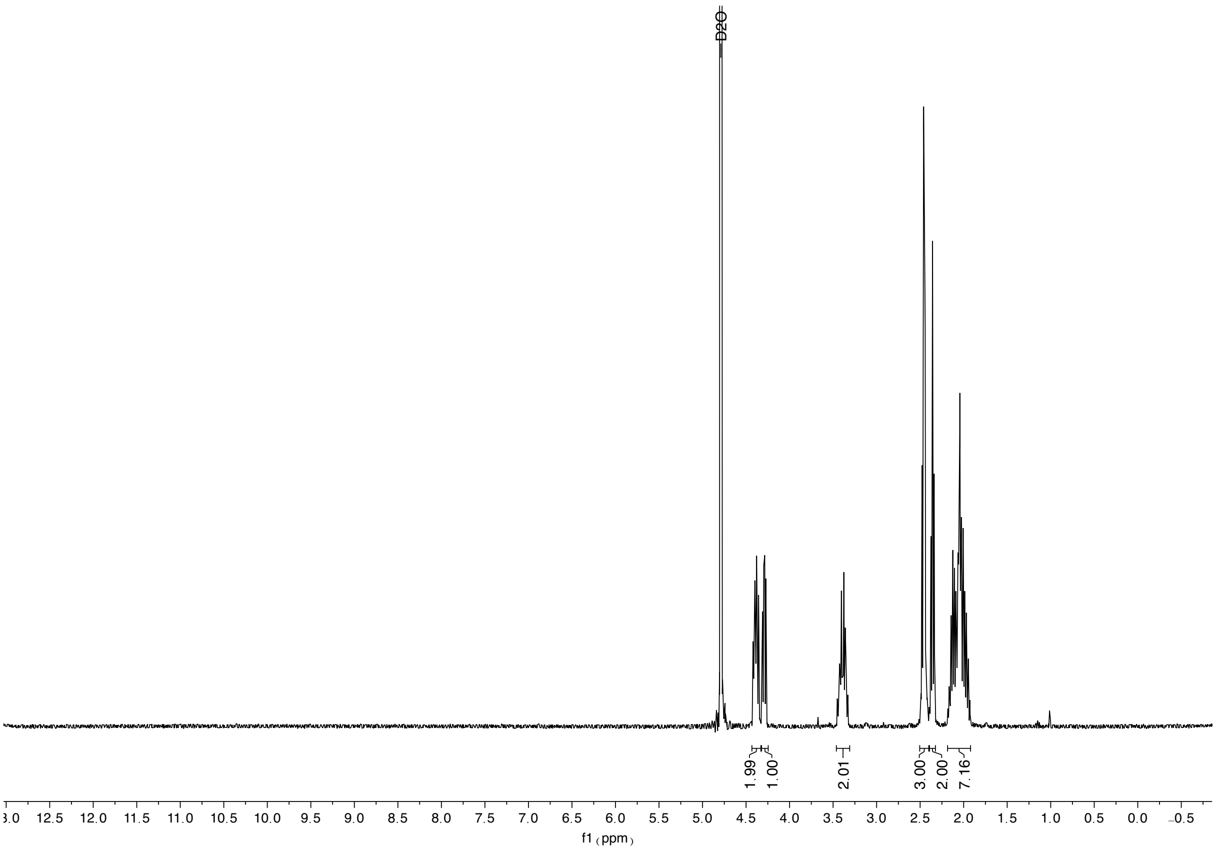

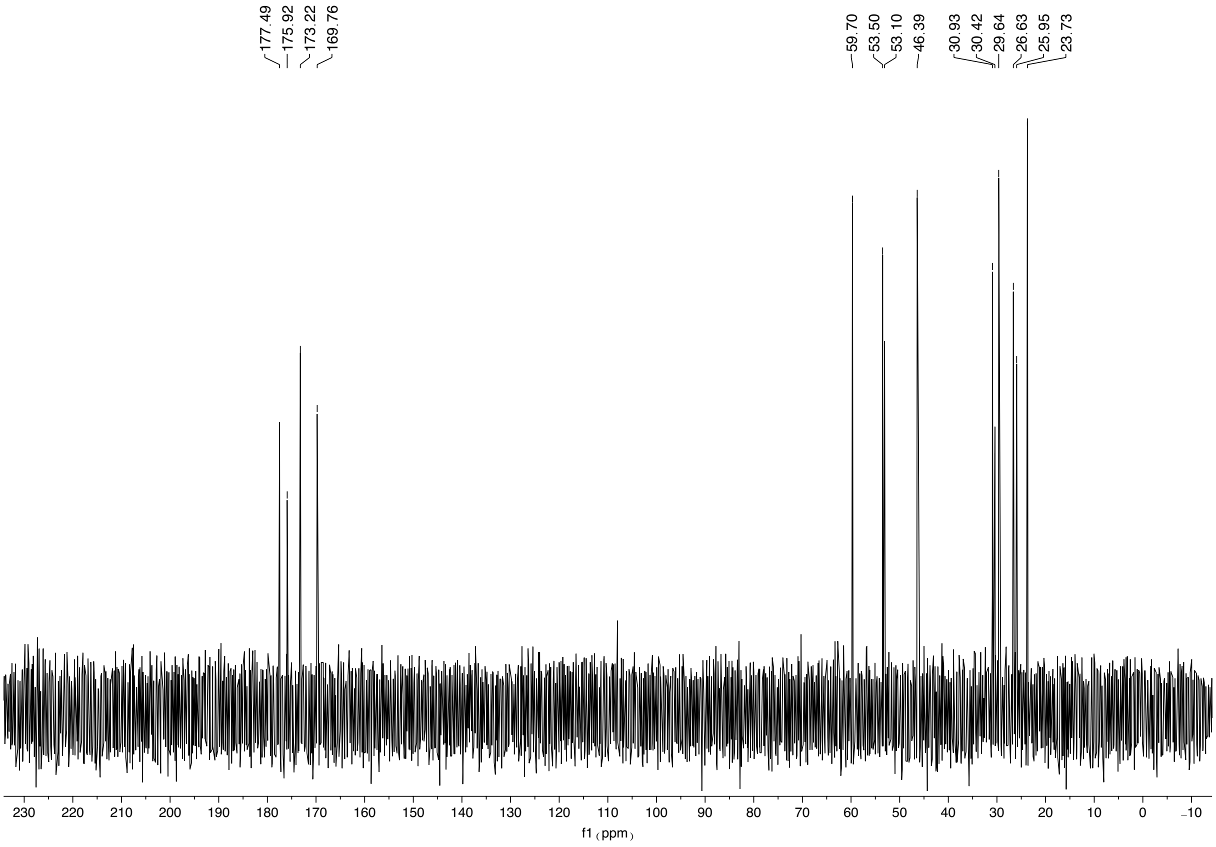

^{1}H and ^{13}C-NMR of H-D-Pro-L-Gln-L-Tyr-NH₂ · TFA (UTS-90):

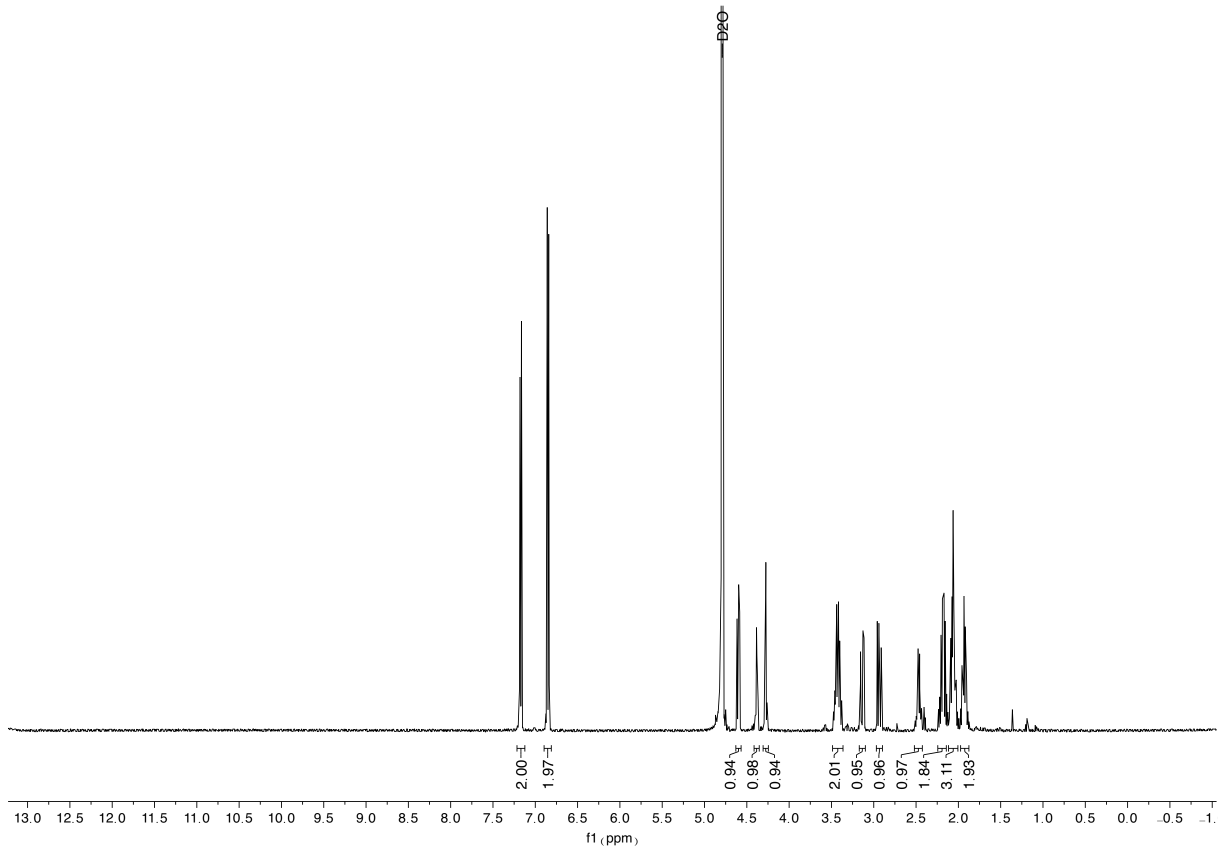

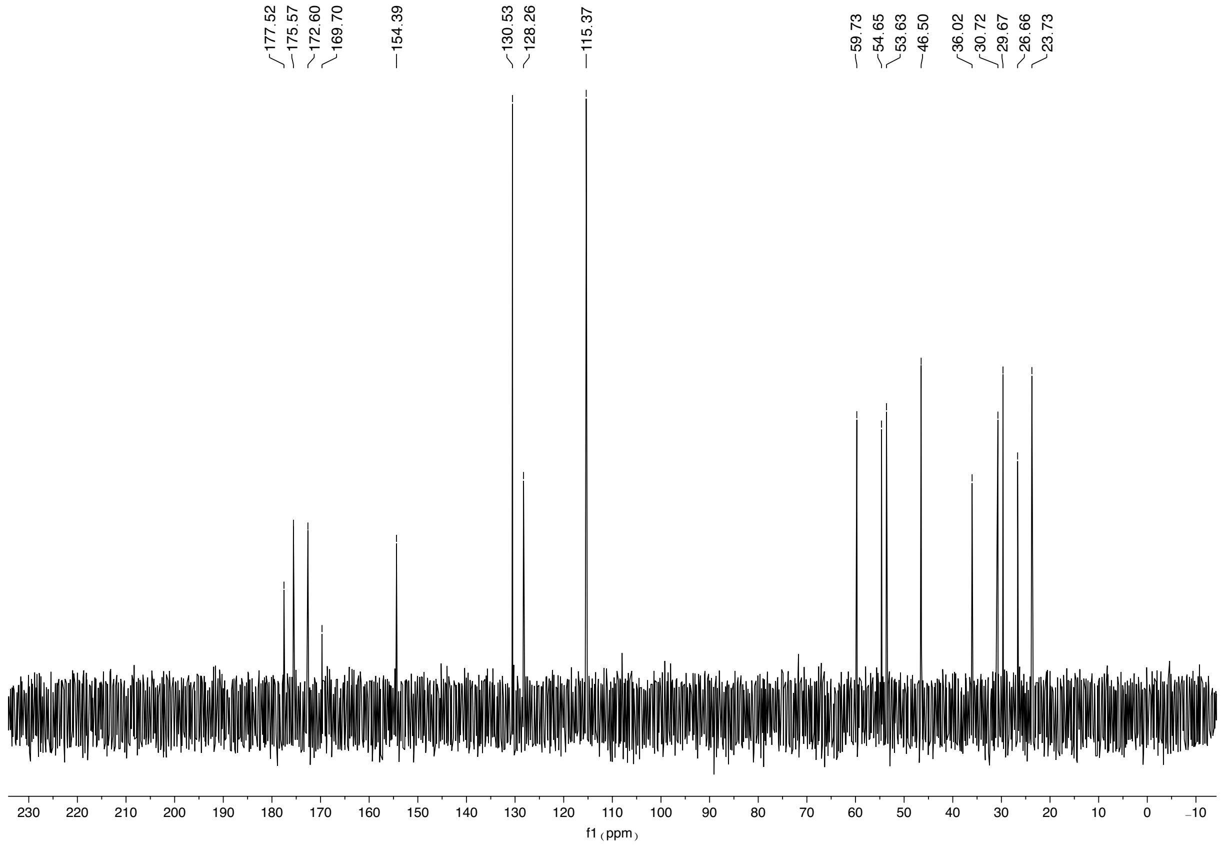

^{1}H and ^{13}C-NMR of H-D-Pro-L-Gln-D-Tyr-NH₂ · TFA (UTS-91):

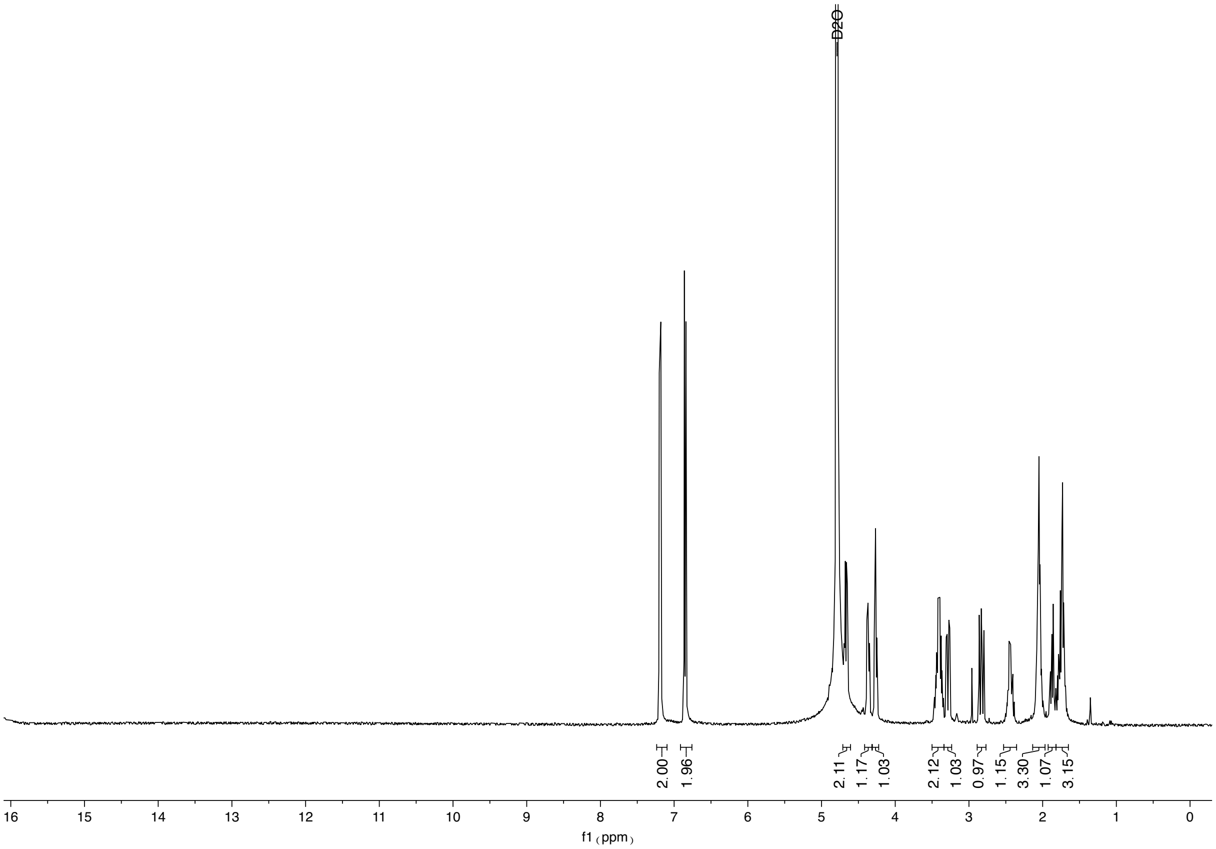

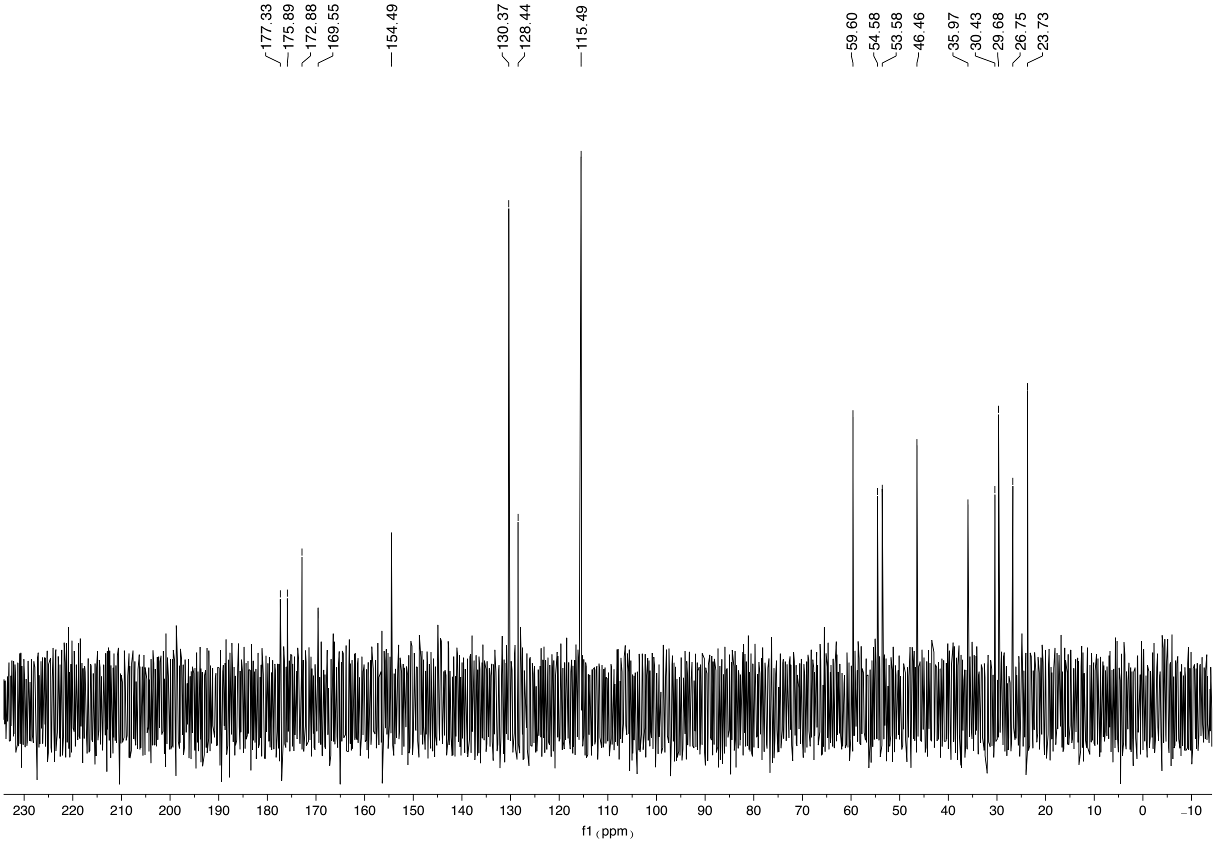

^{1}H and ^{13}C-NMR of H-D-Pro-L-Gln-CyLeu-NH$_2$ · TFA (UTS-92):

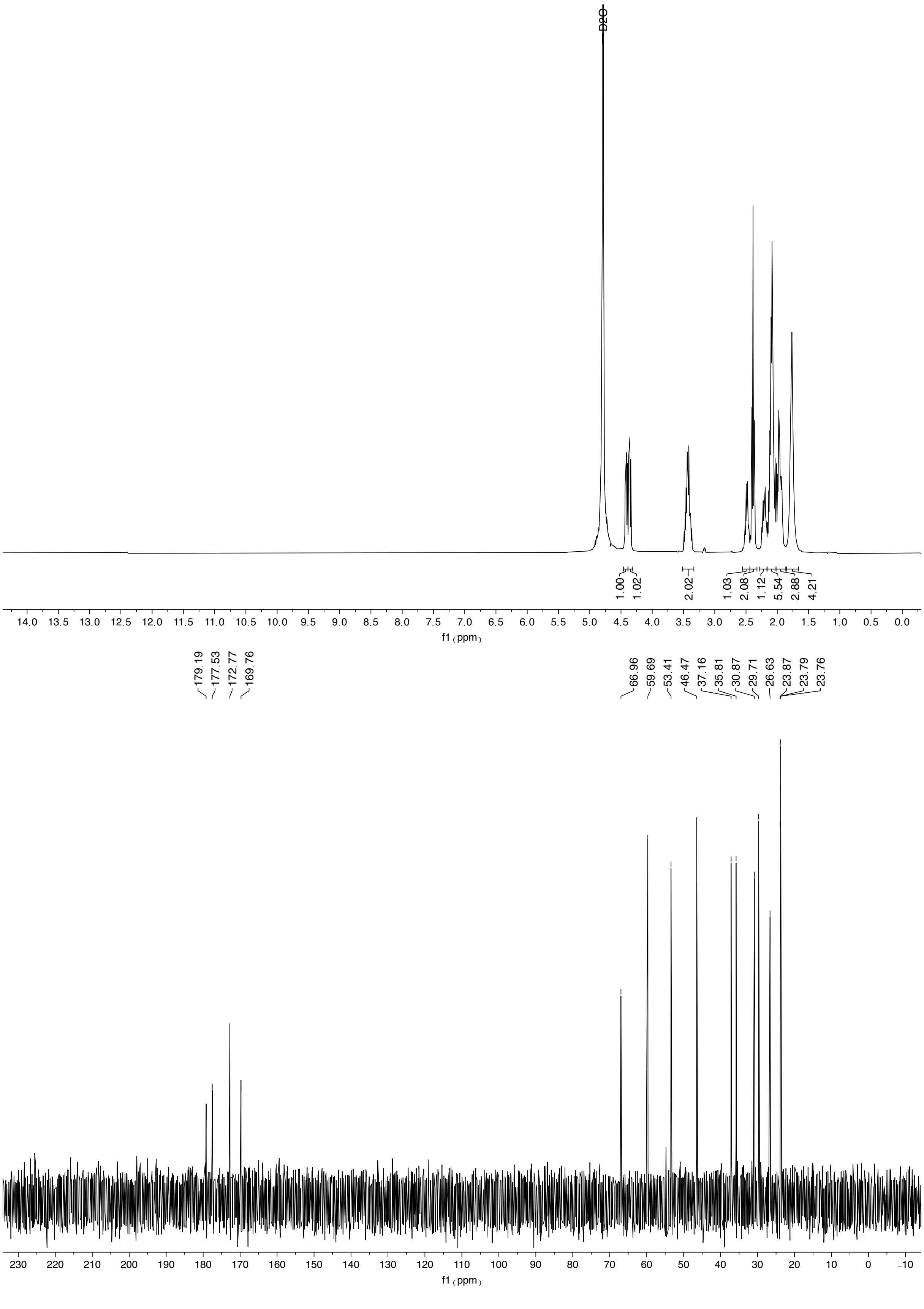

^{1}H and ^{13}C-NMR of H-D-Pro-L-Gln-Abz-NH_2 · TFA (UTS-93):

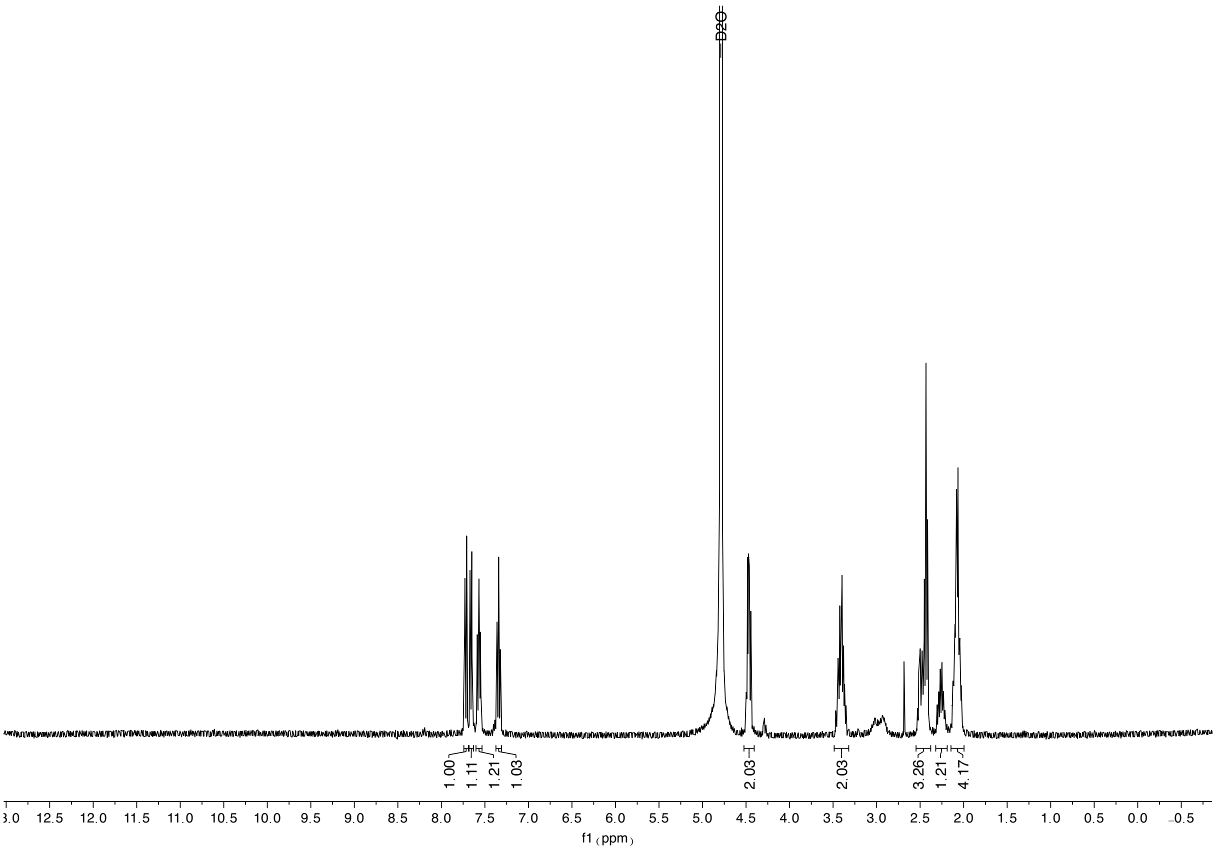

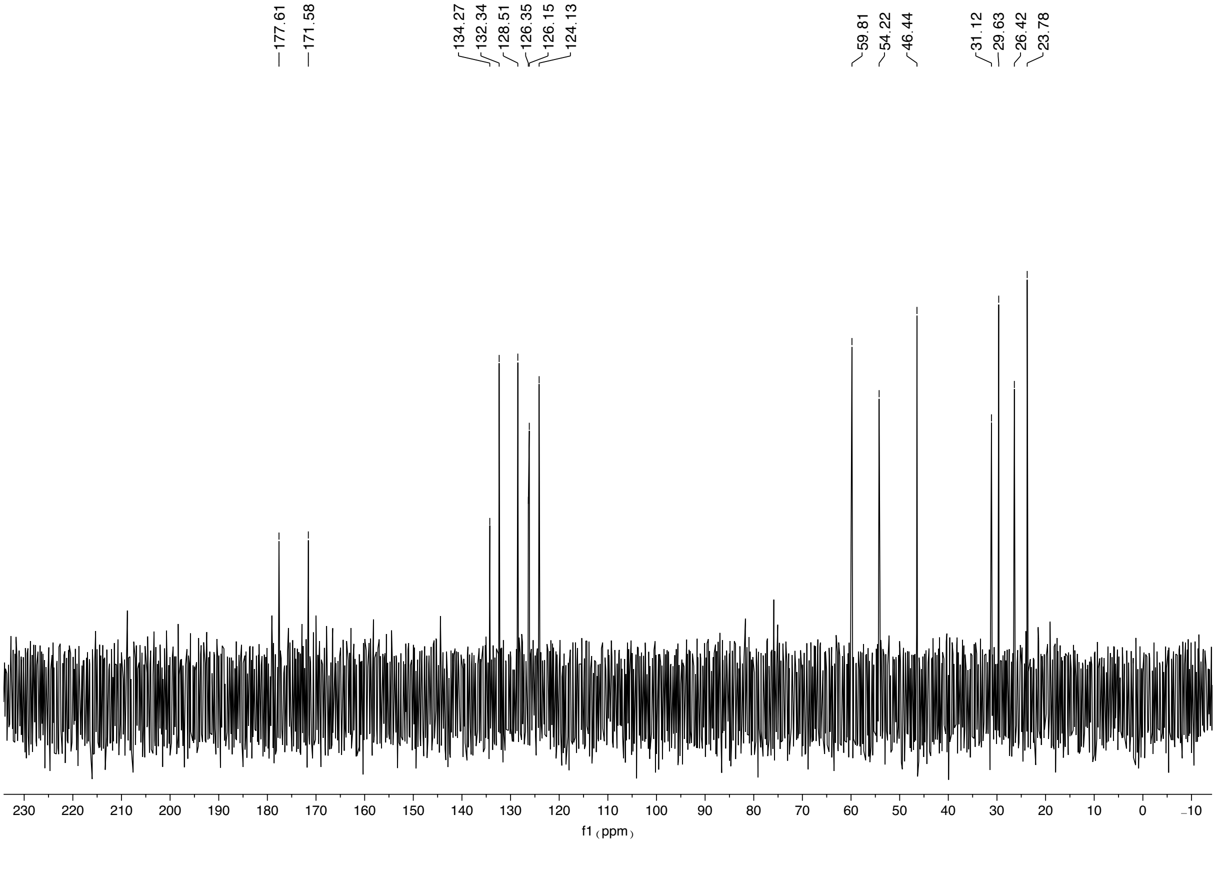

^{1}H and ^{13}C-NMR of H-D-Pro-L-Gln-D-Ind-NH$_2$ · TFA (UTS-94):

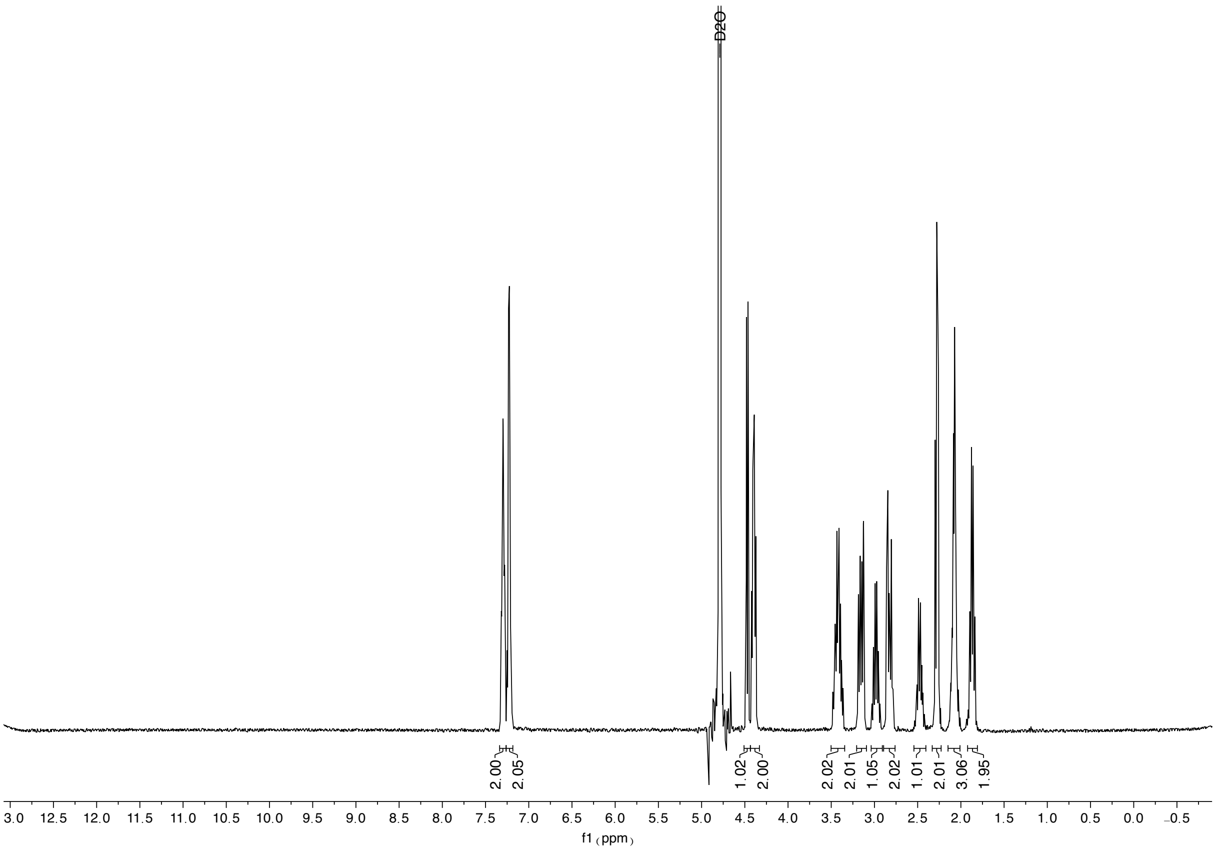

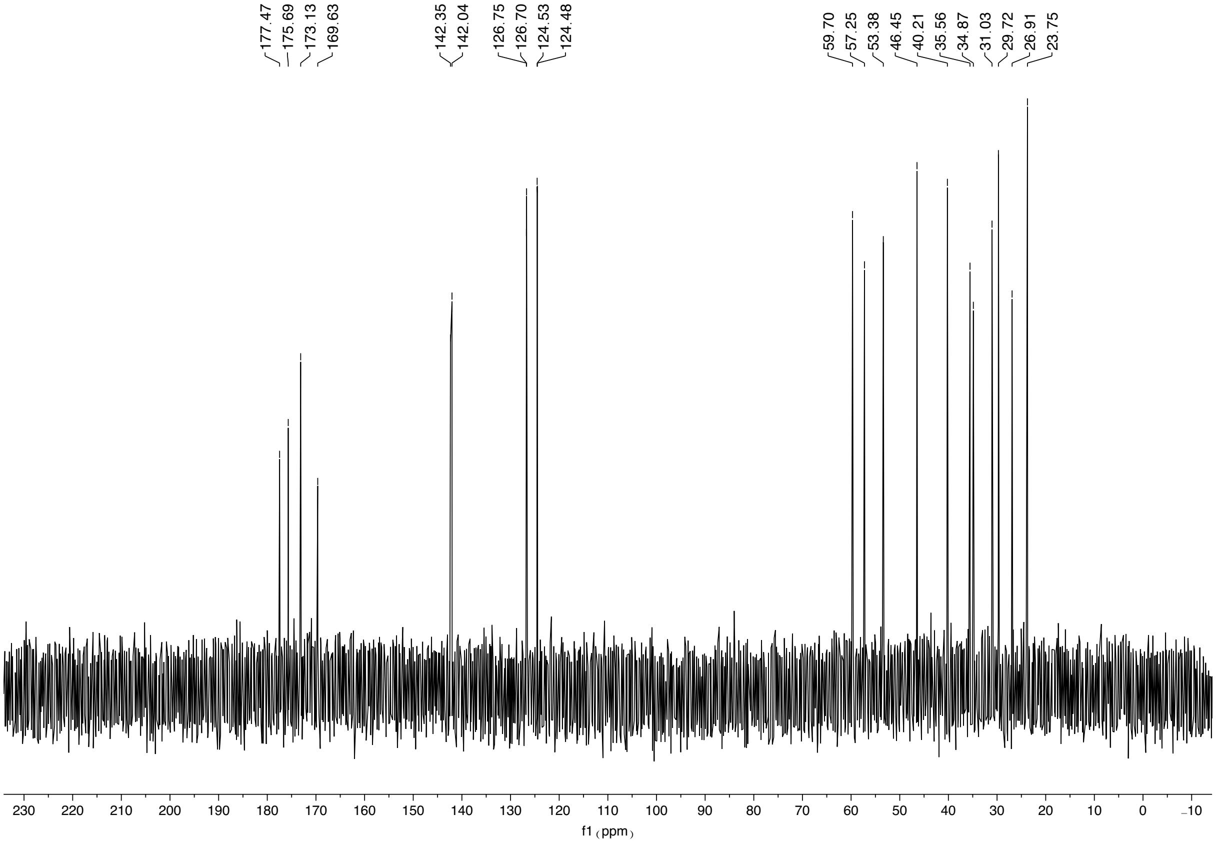

^{1}H and ^{13}C-NMR of H-D-Pro-D-Gln-L-Gln-NH_2 · TFA (UTS-95):

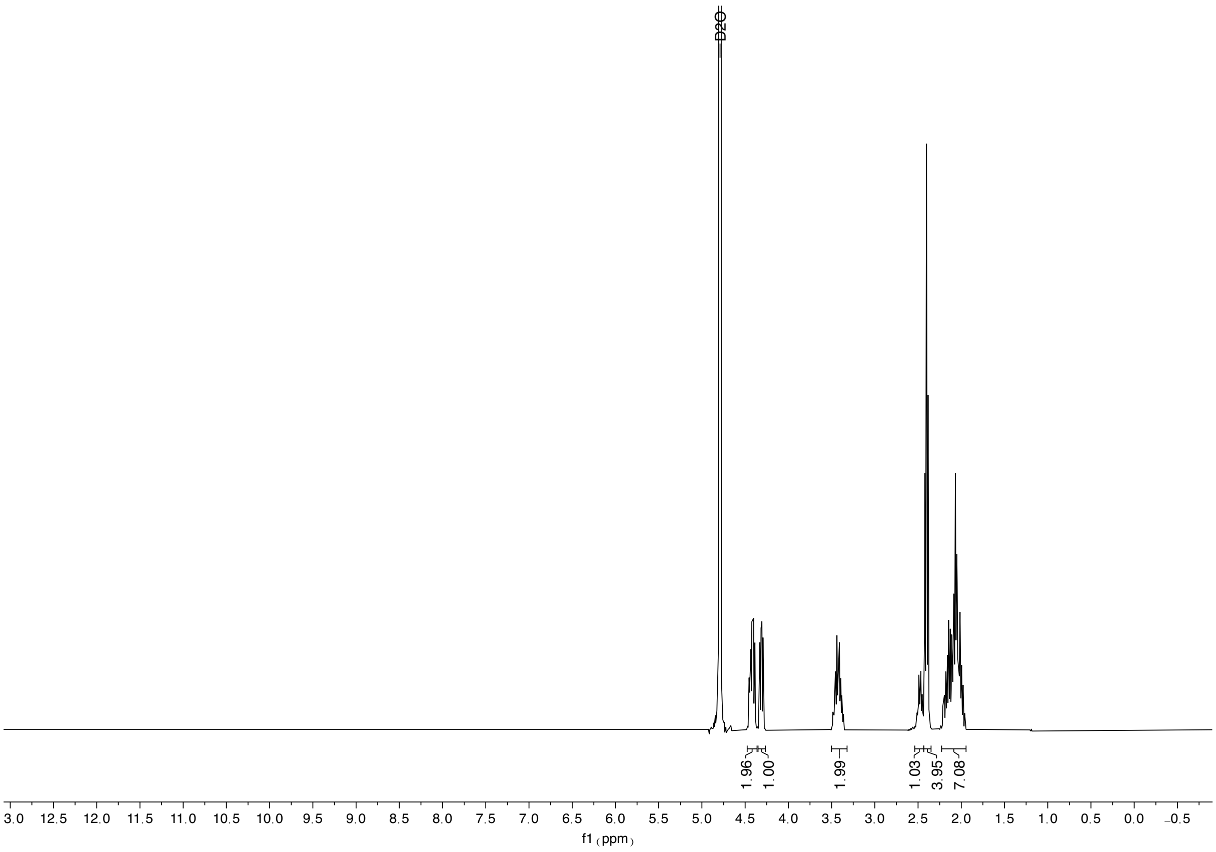

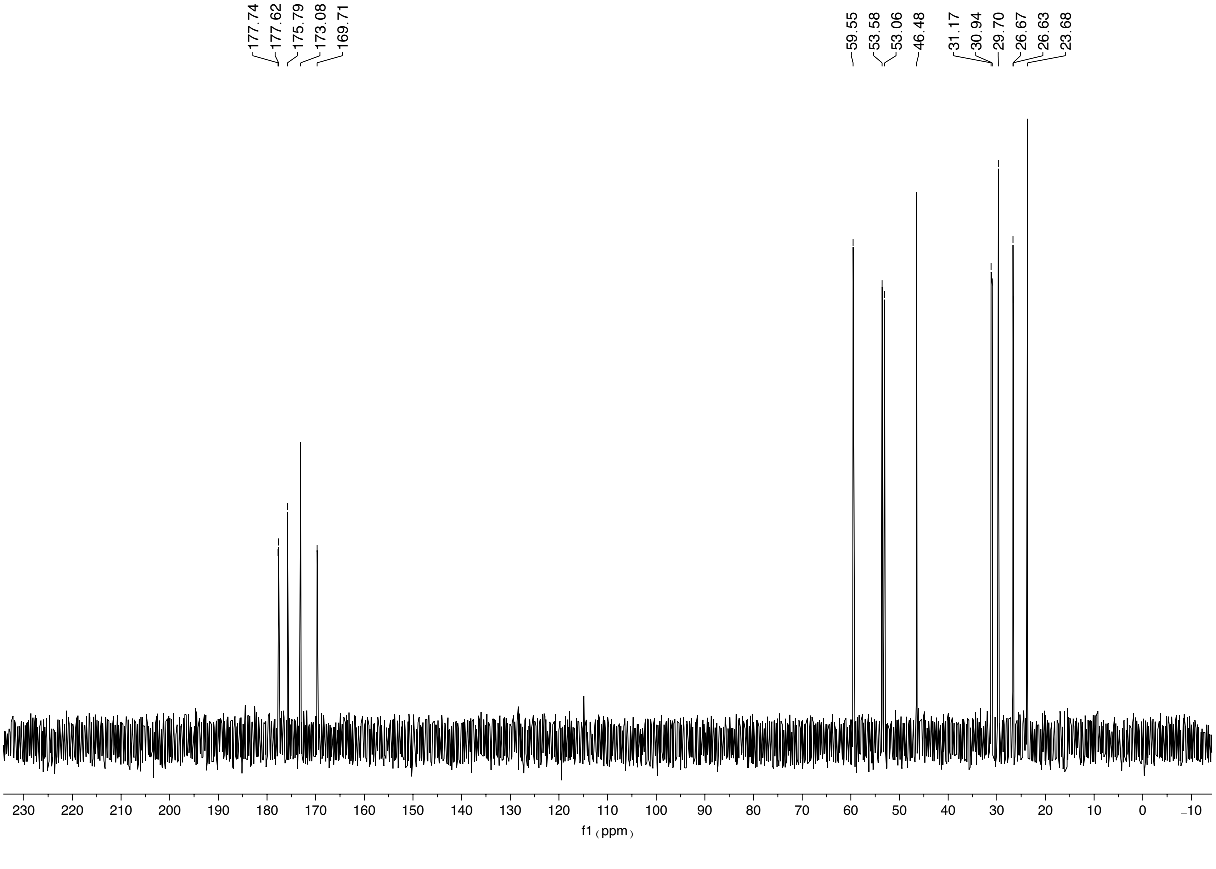

^{1}H and ^{13}C-NMR of H-D-Pro-L-Glu-L-Gln-NH_2 · TFA (UTS-96):

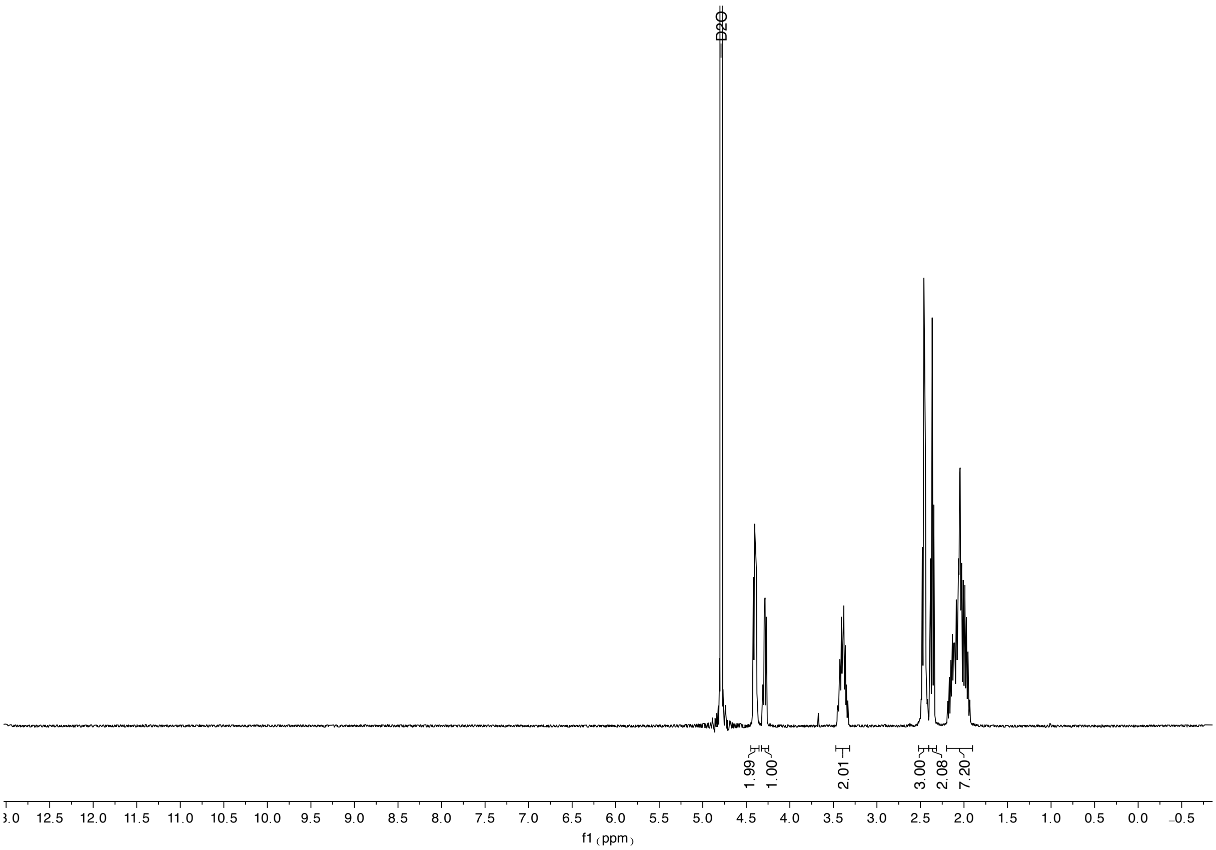

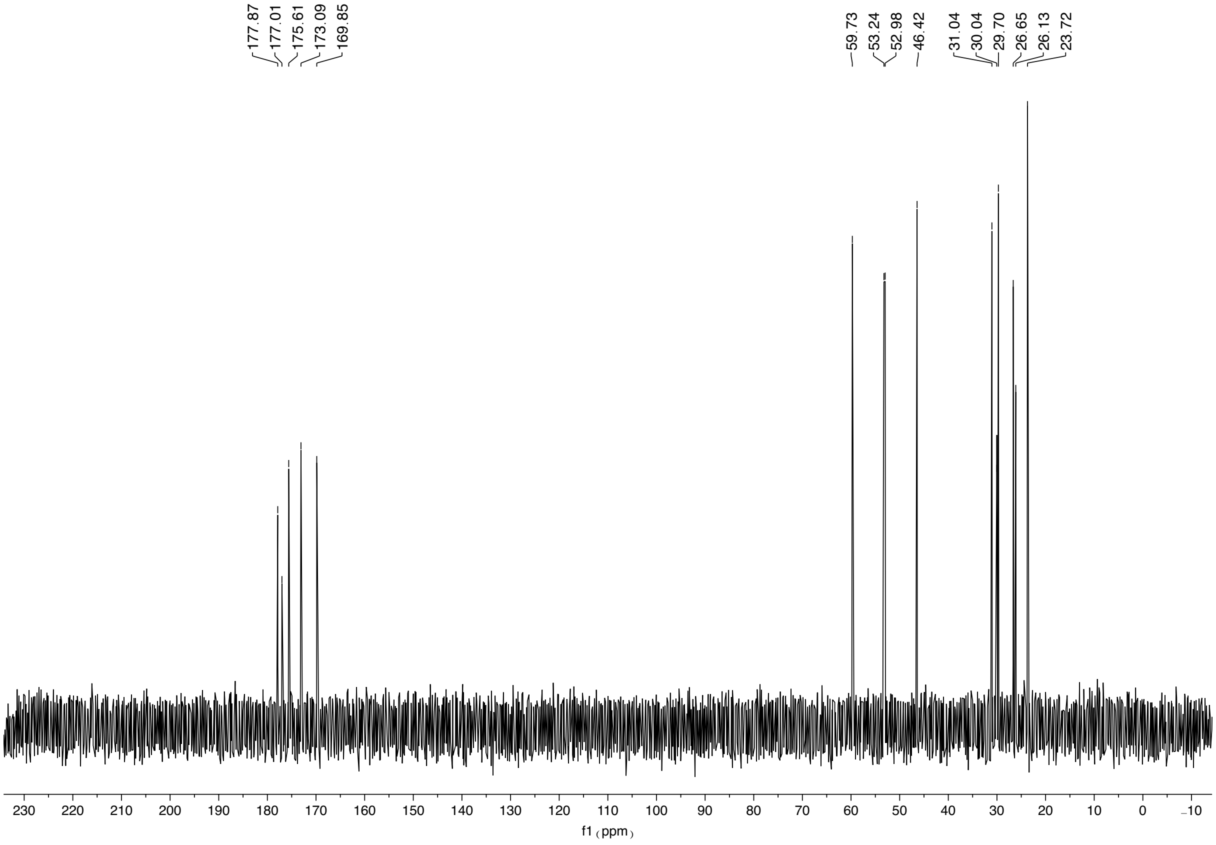

^{1}H and ^{13}C-NMR of H-D-Pro-D-Glu-L-Gln-NH$_2$ · TFA (UTS-97):

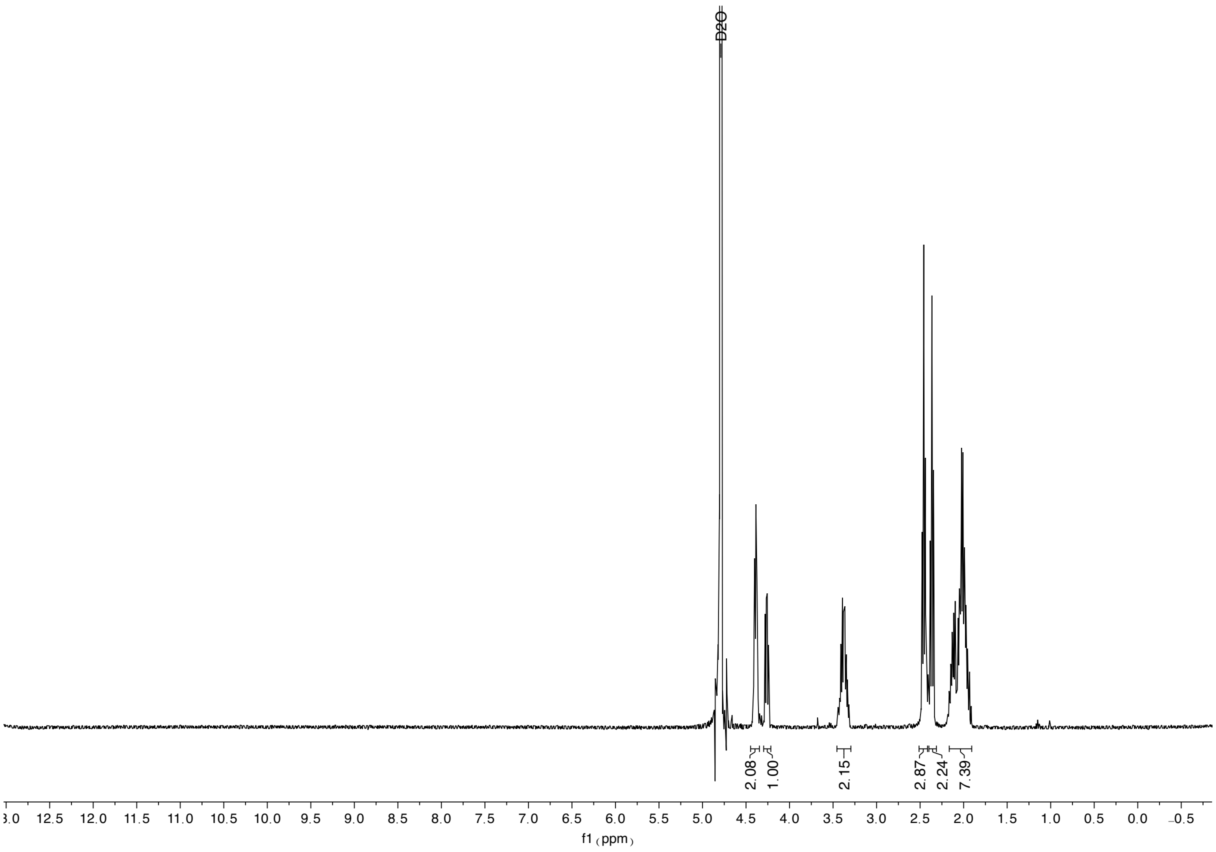

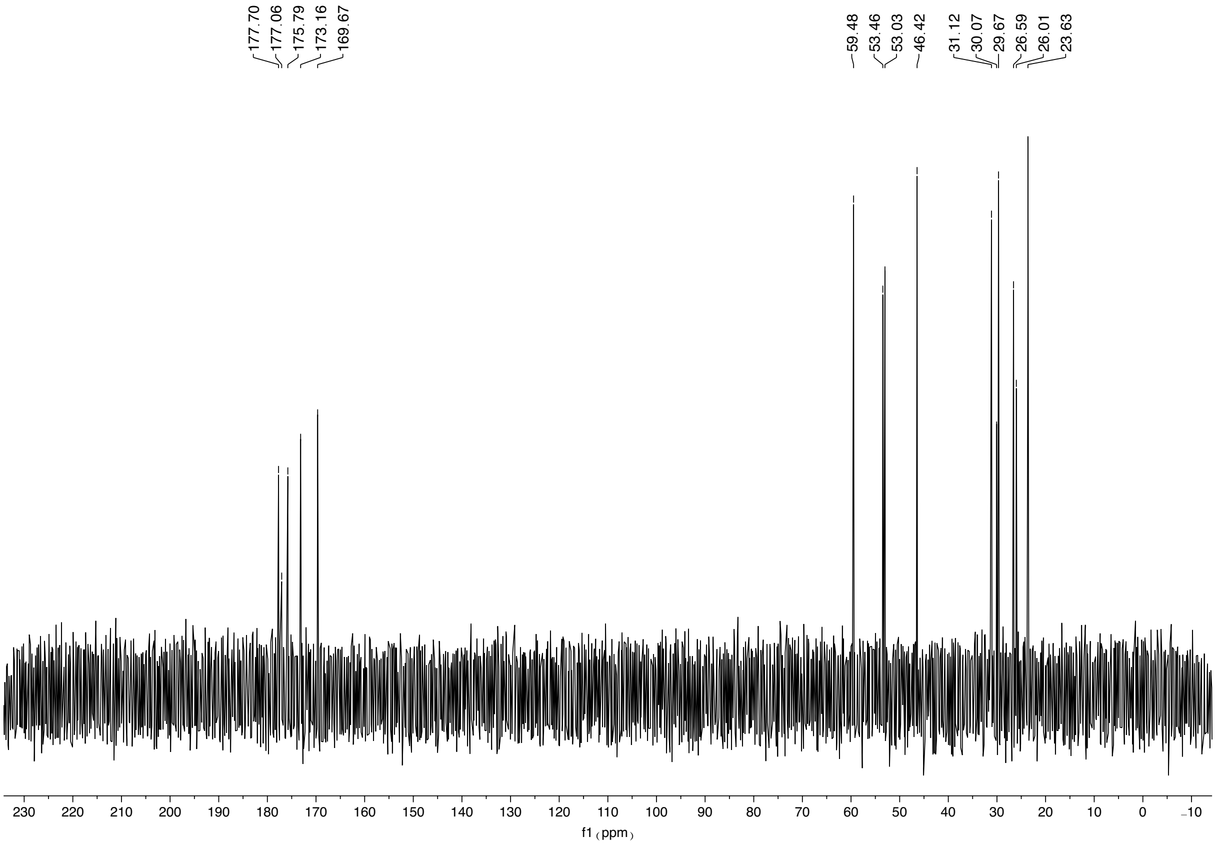

^{1}H and ^{13}C-NMR of H-D-Pro-L-Tyr-L-Gln-NH_2 · TFA (UTS-98):

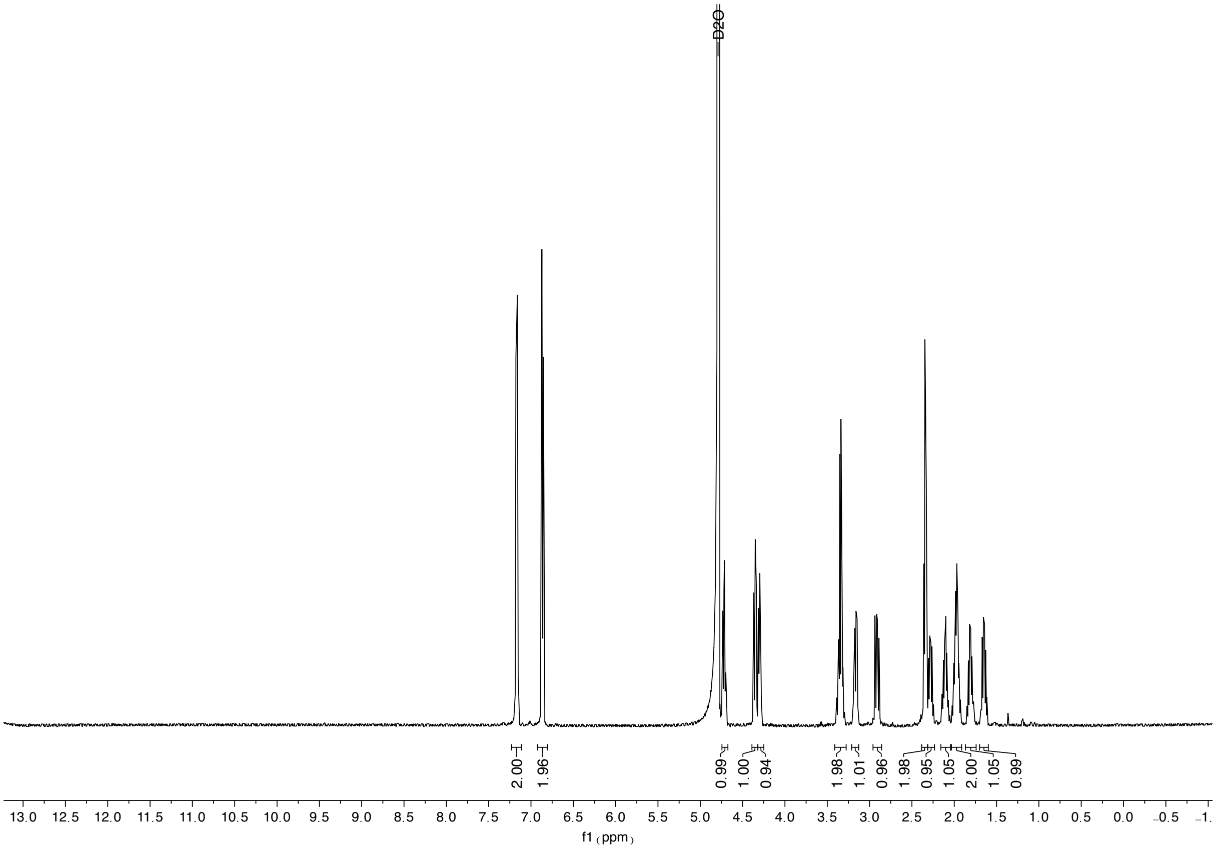

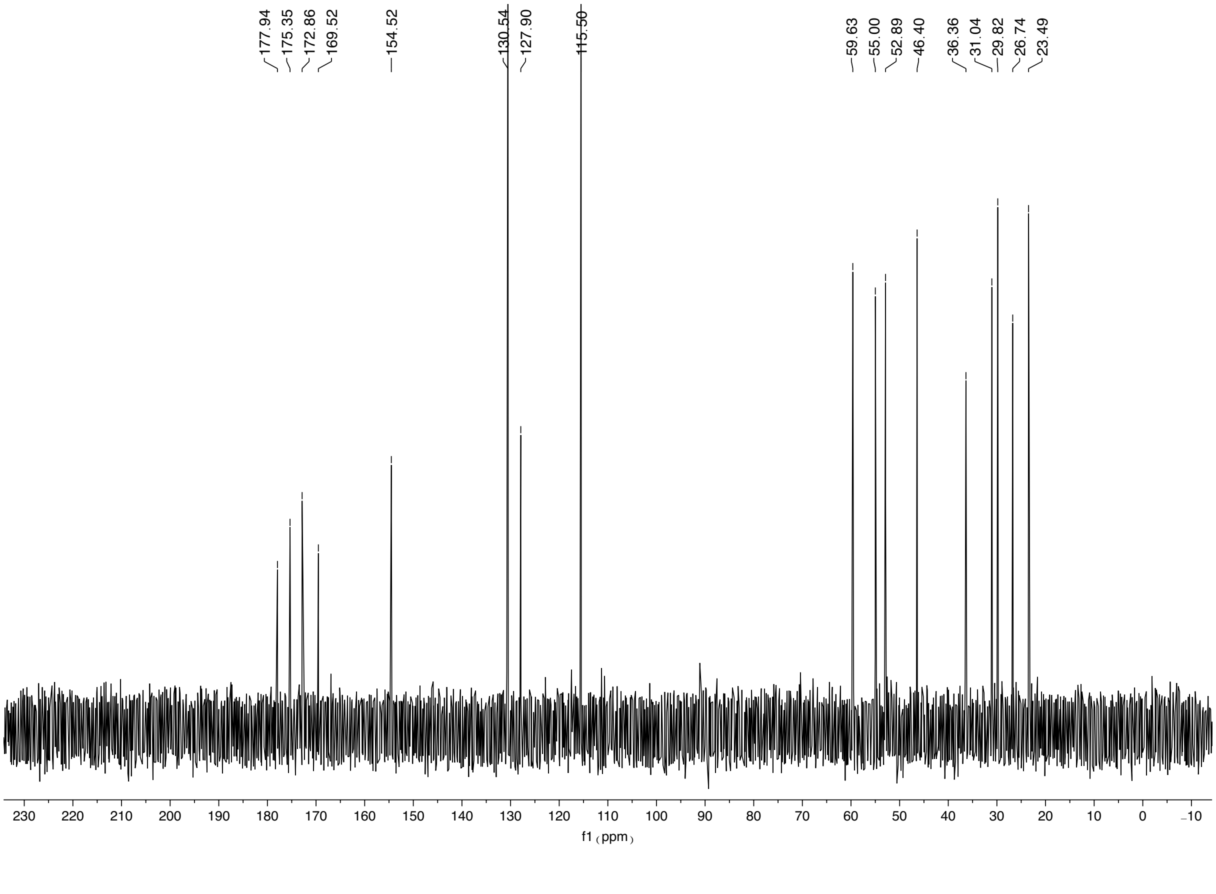

^{1}H and ^{13}C-NMR of H-D-Pro-D-Tyr-L-Gln-NH₂ · TFA (UTS-99):

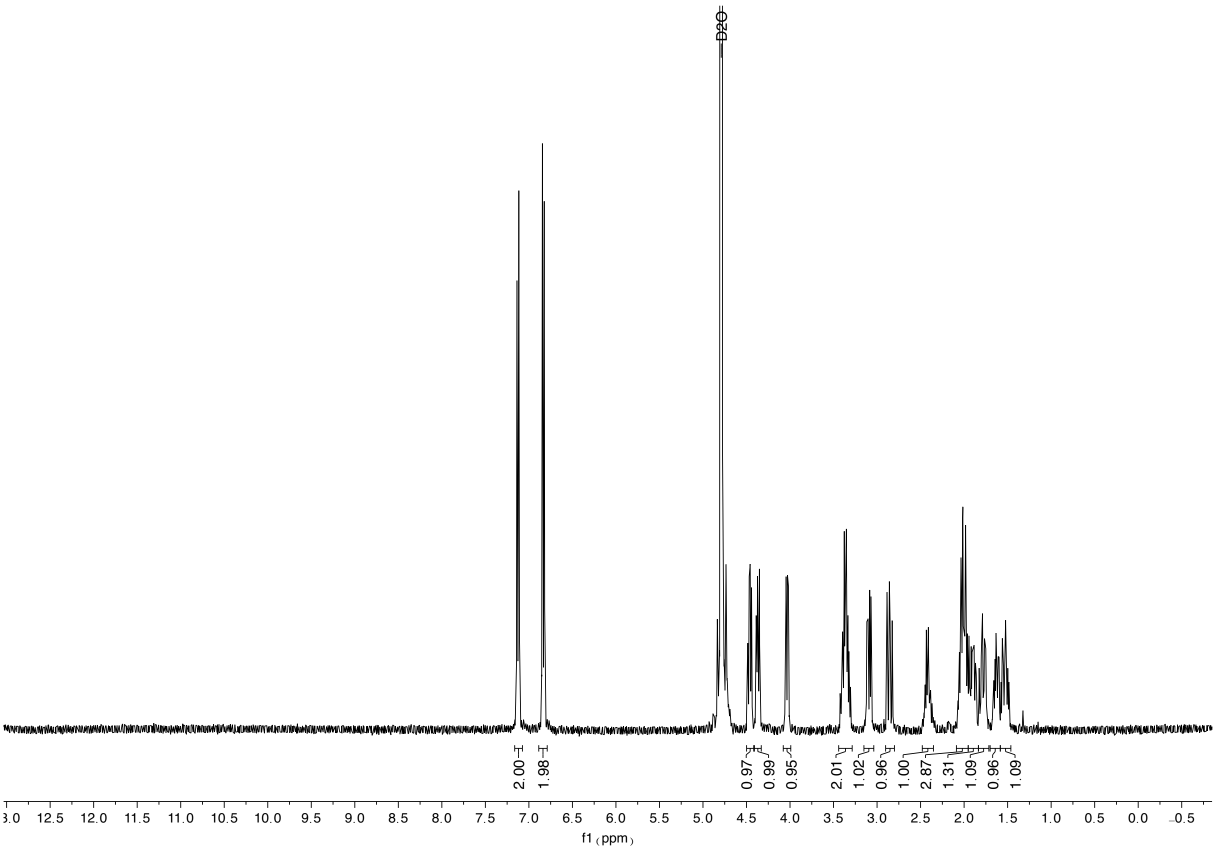

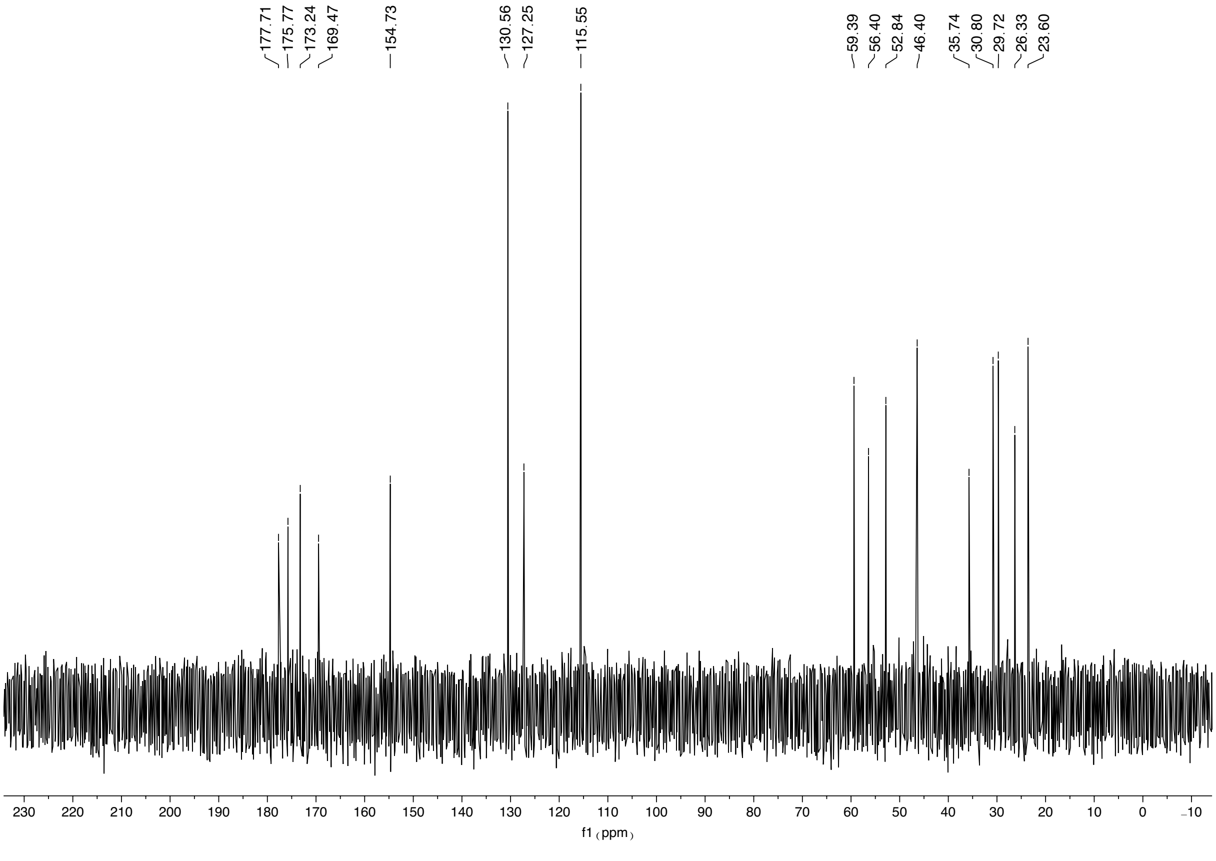

^{1}H and ^{13}C-NMR of H-D-Pro-CyLeu-L-Gln-NH$_2$ · TFA (UTS-100):

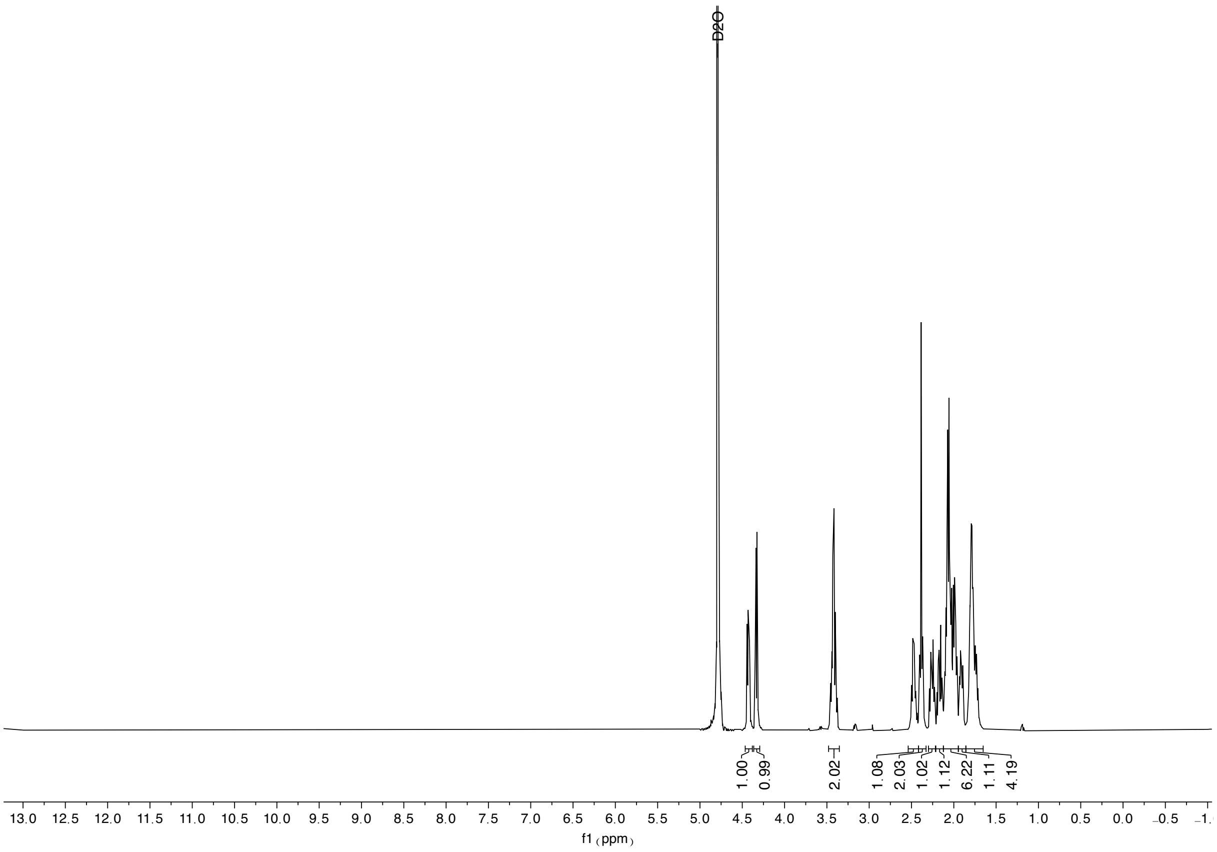

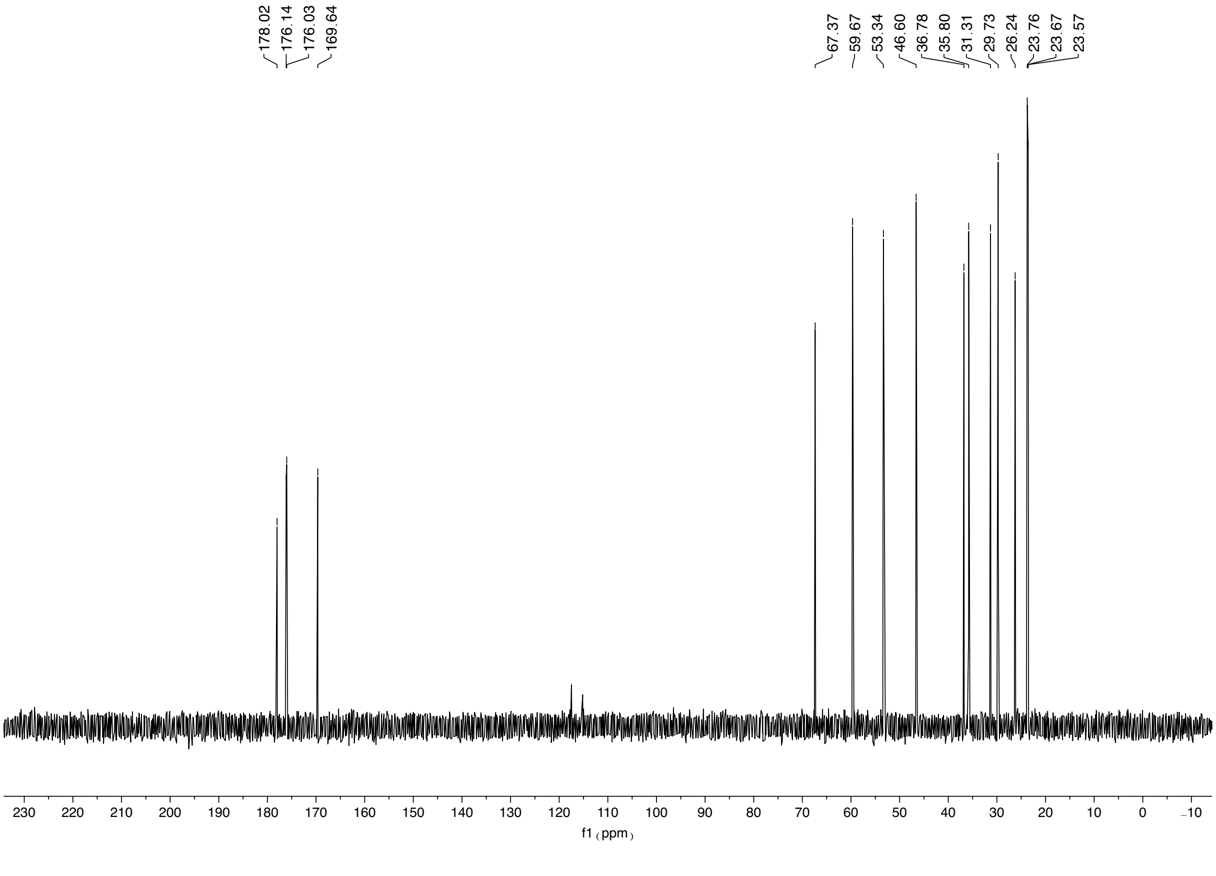

^{1}H and ^{13}C-NMR of H-D-Pro-Abz-L-Gln-NH$_2$ · TFA (UTS-101):

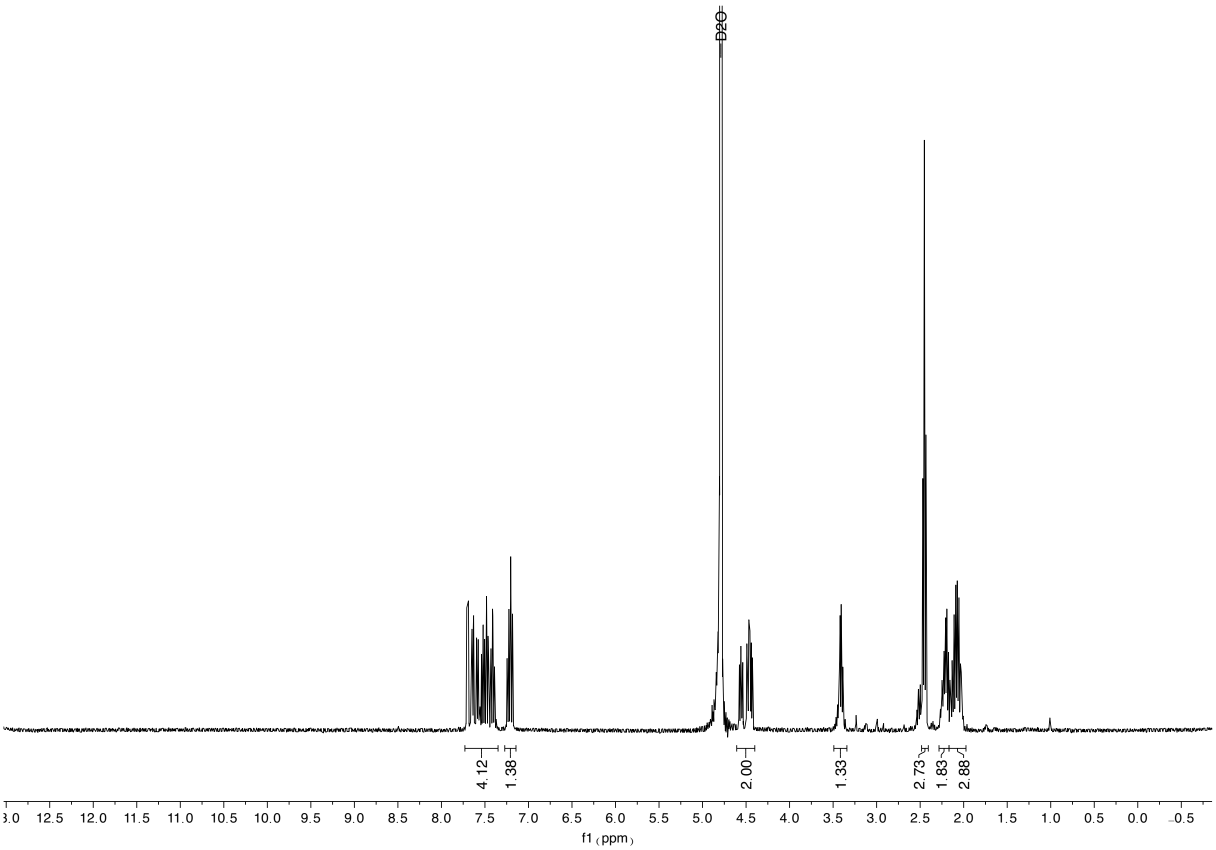

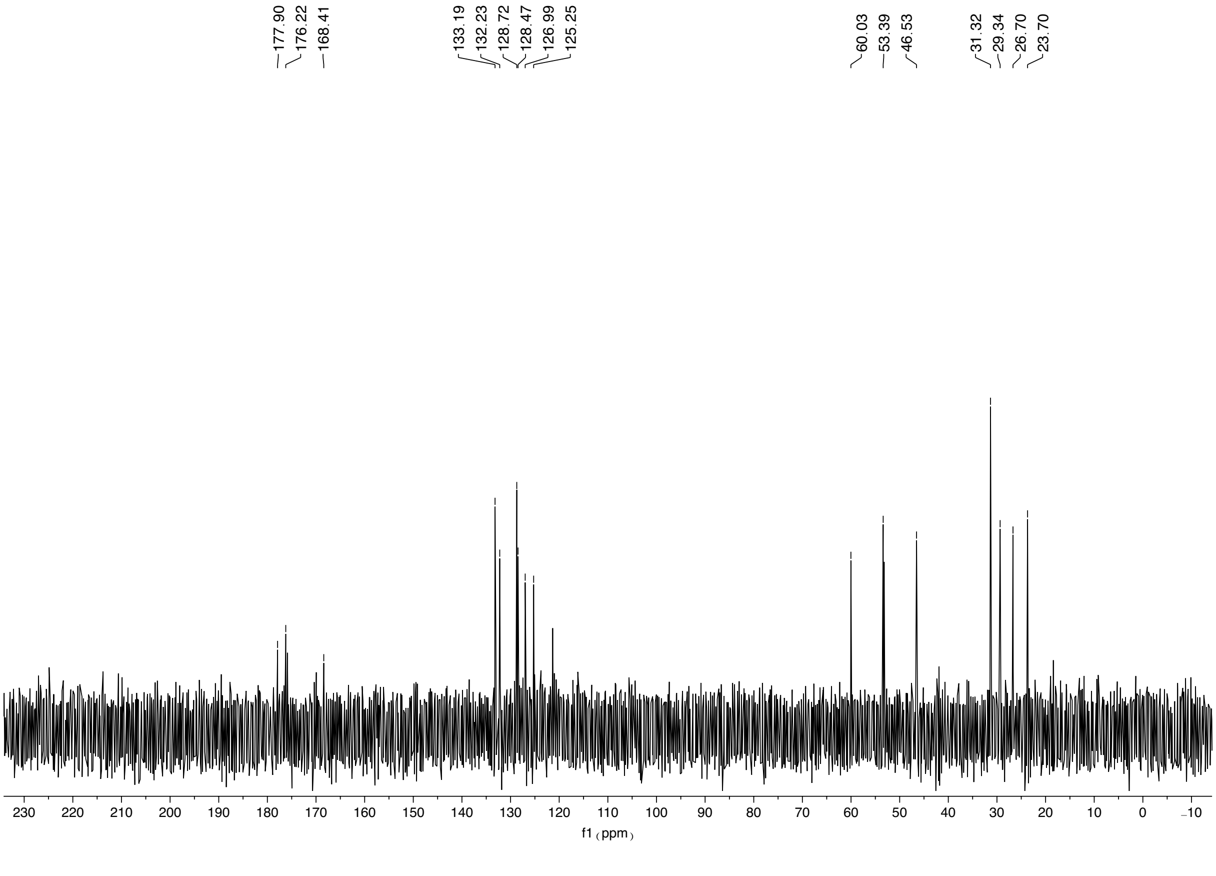

^{1}H and ^{13}C-NMR of H-D-Pro-Ind-L-Gln-NH₂ · TFA (UTS-102):

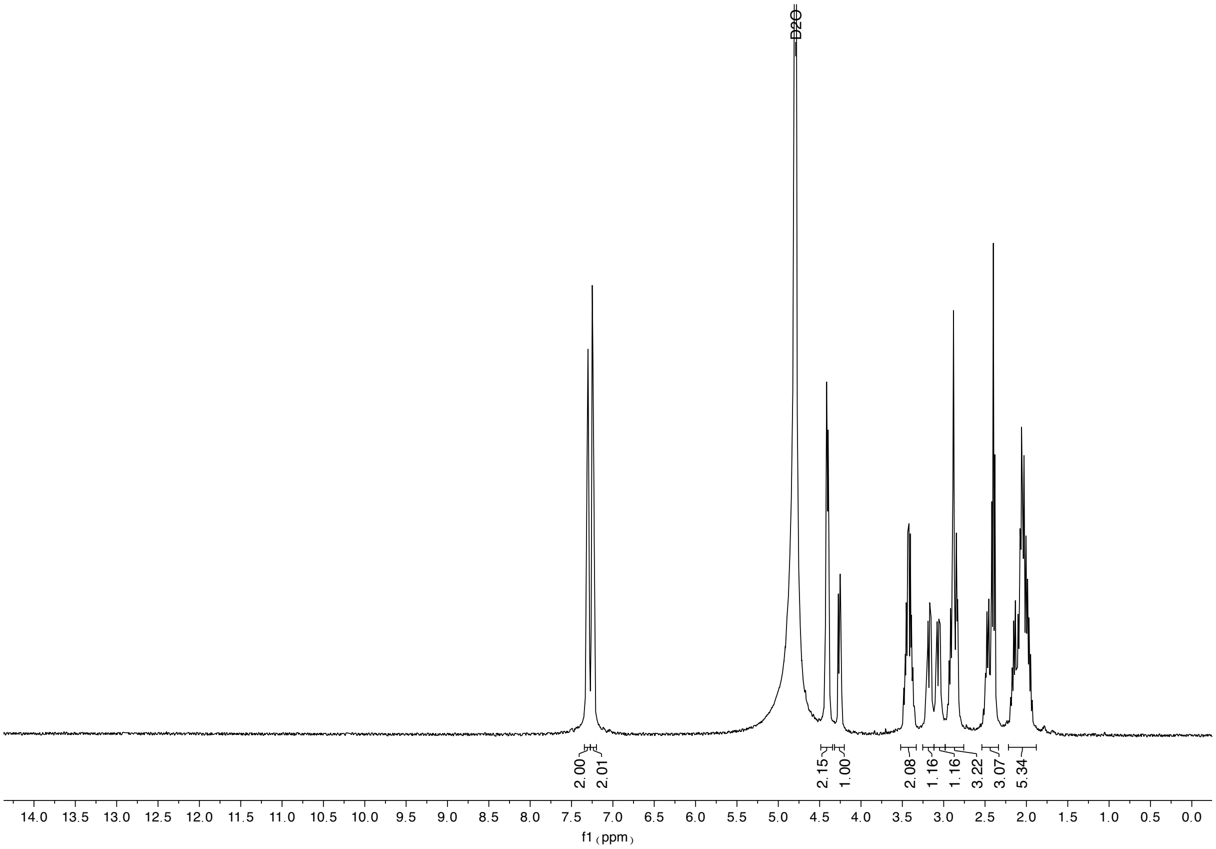

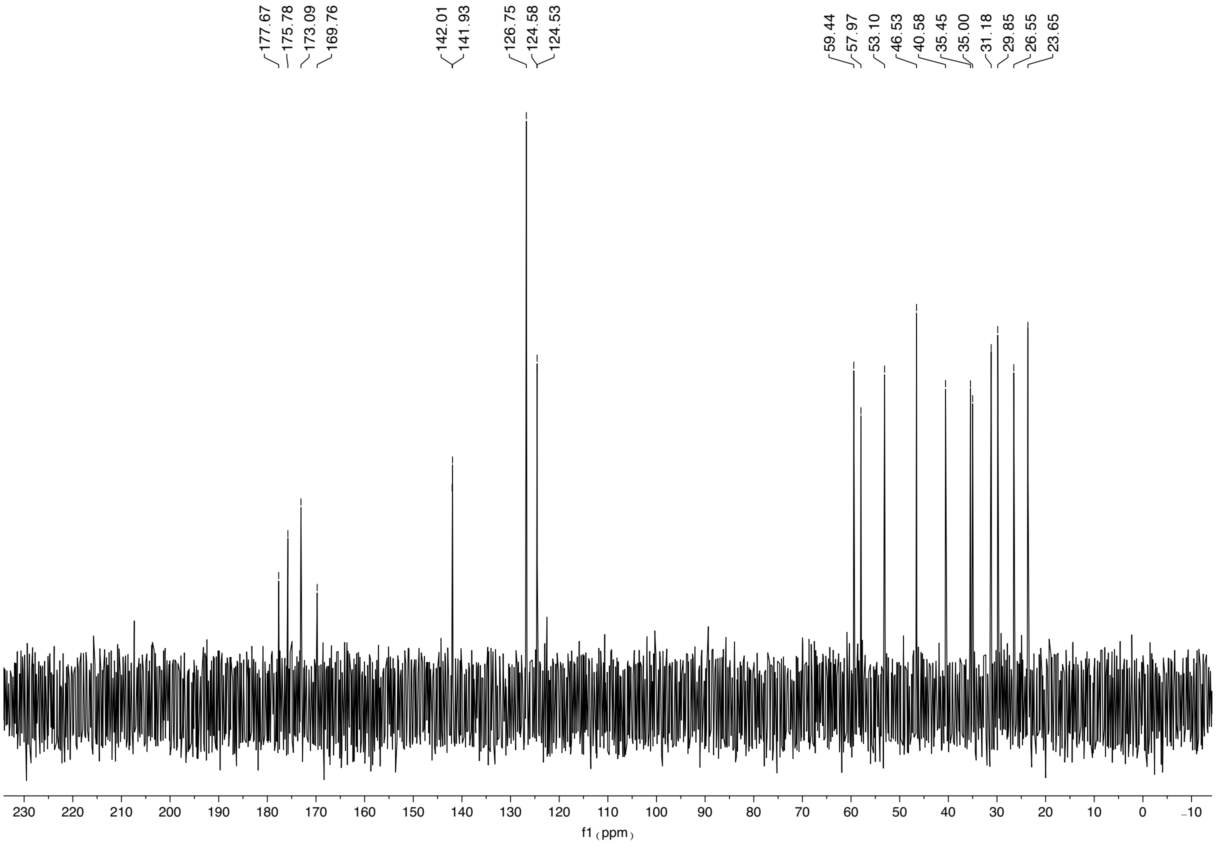

^{1}H and ^{13}C-NMR of H-D-Pro-D-Gln-D-Gln-NH$_2$ · TFA (UTS-103):

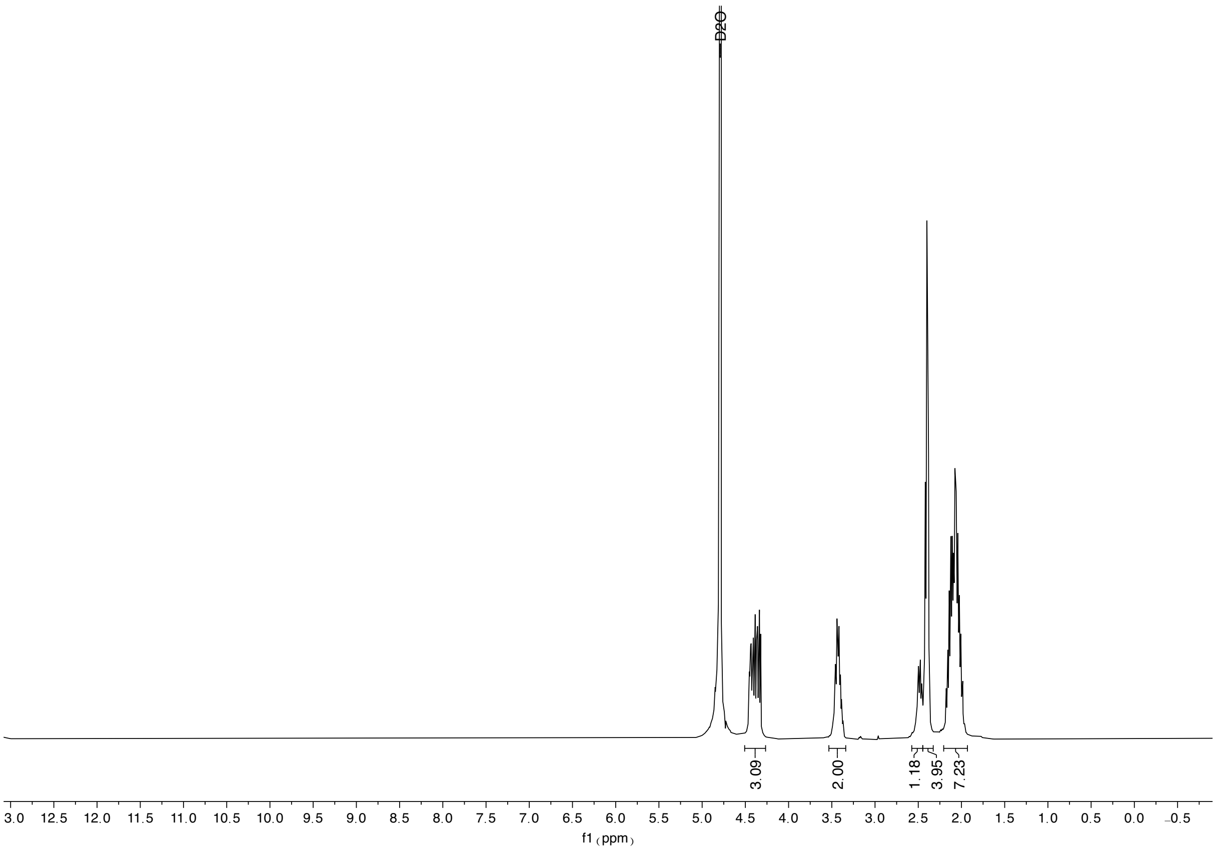

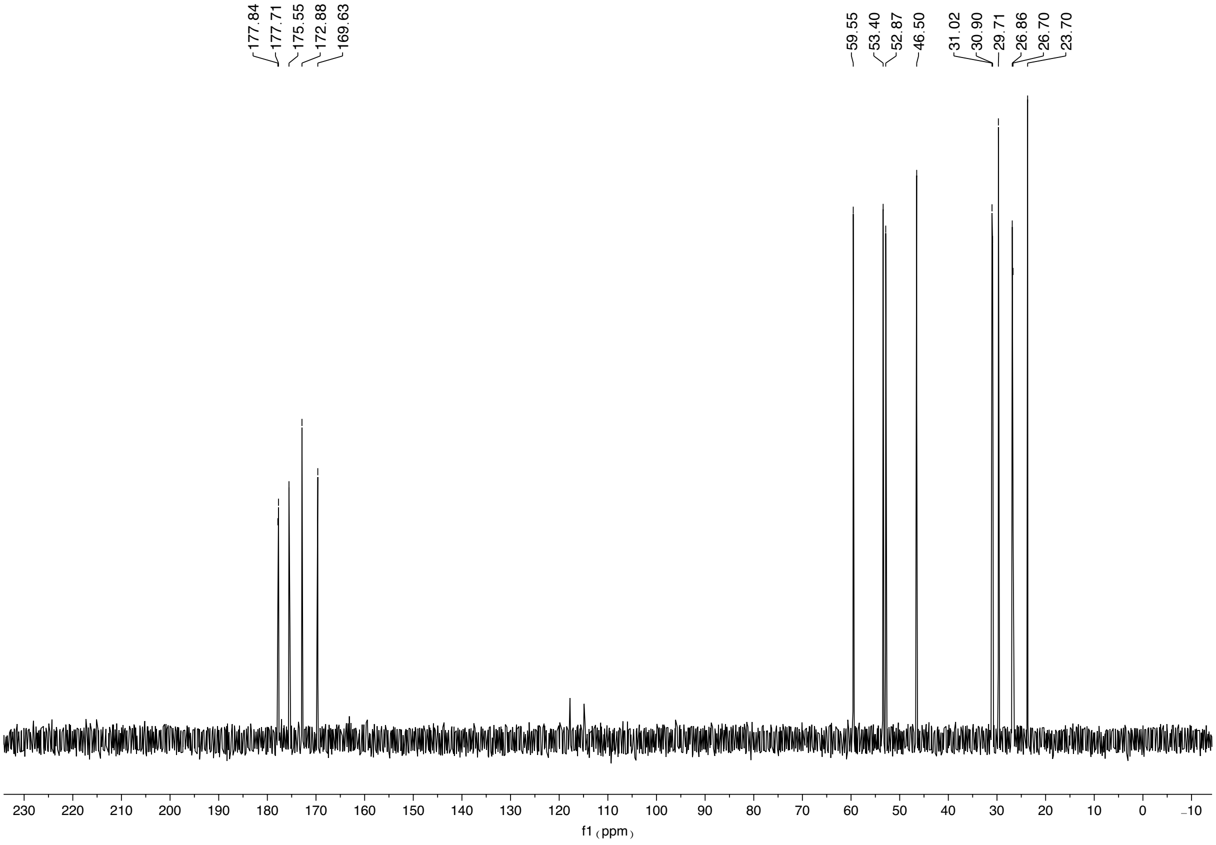

^{1}H and ^{13}C-NMR of H-D-Pro-D-Gln-L-Glu-NH$_2$ · TFA UTS-104):

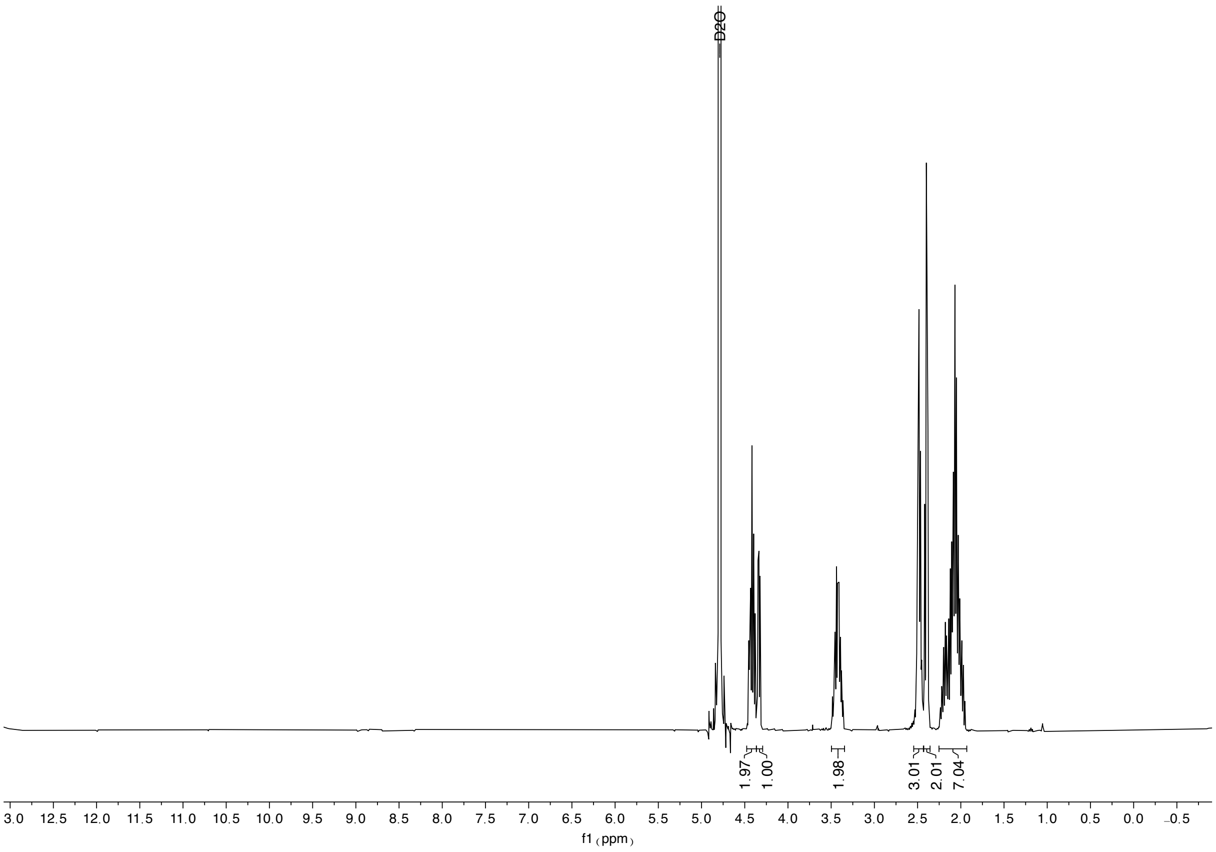

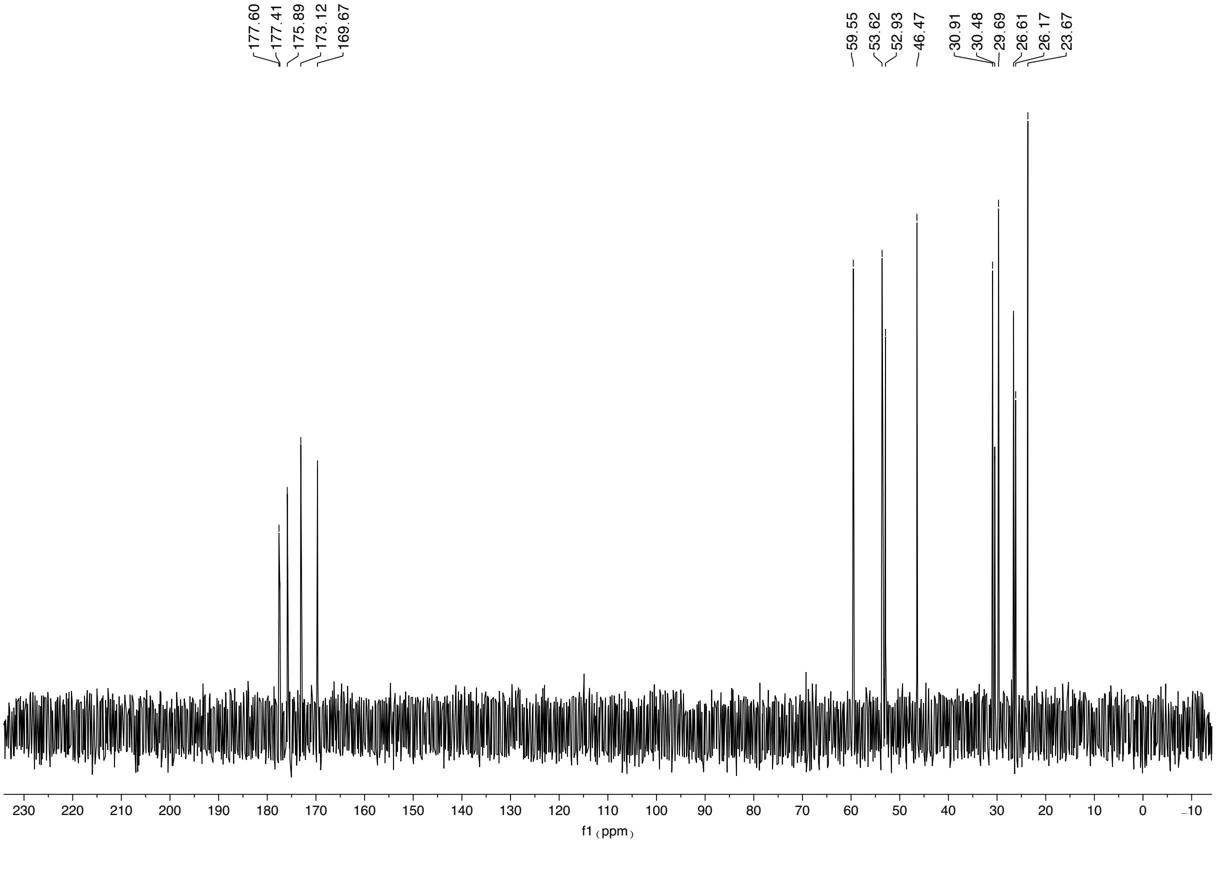

^{1}H and ^{13}C-NMR of H-D-Pro-D-Gln-D-Glu-NH$_2$ · TFA (UTS-105):

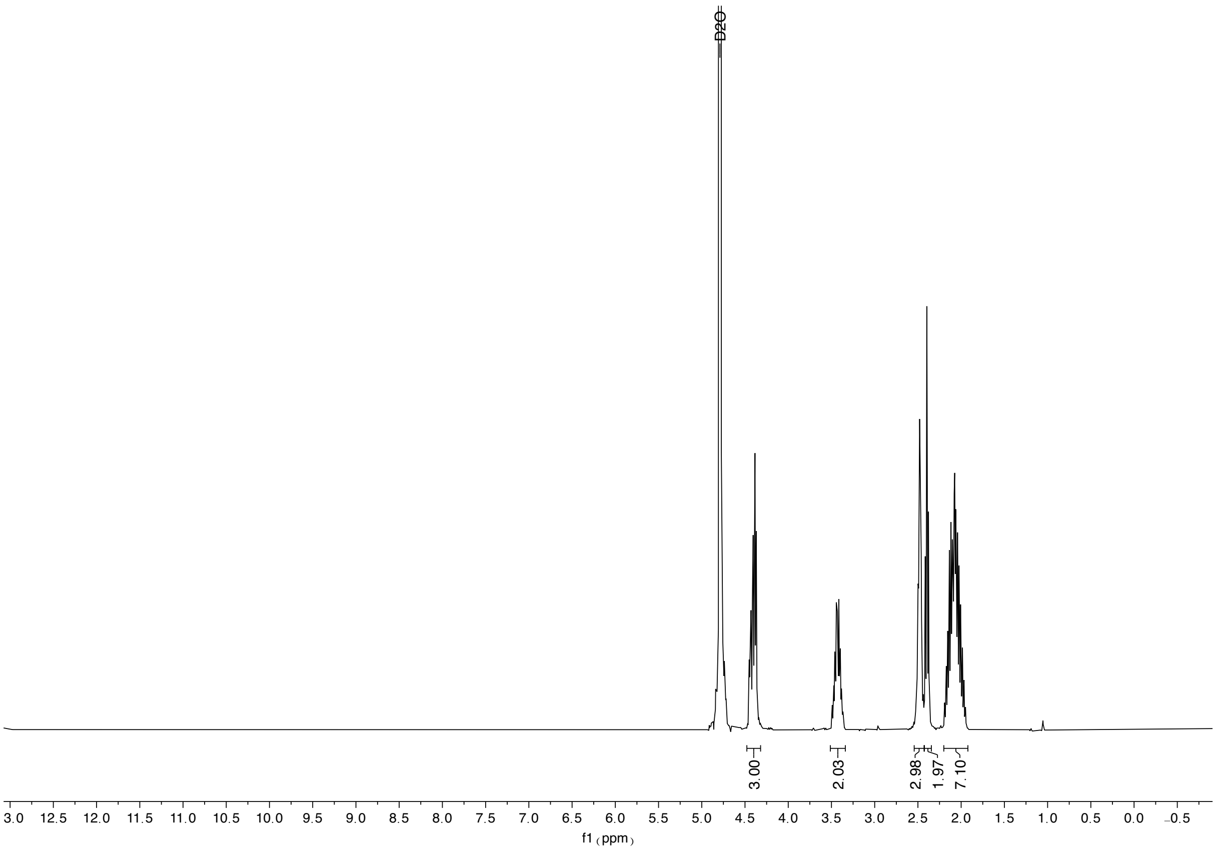

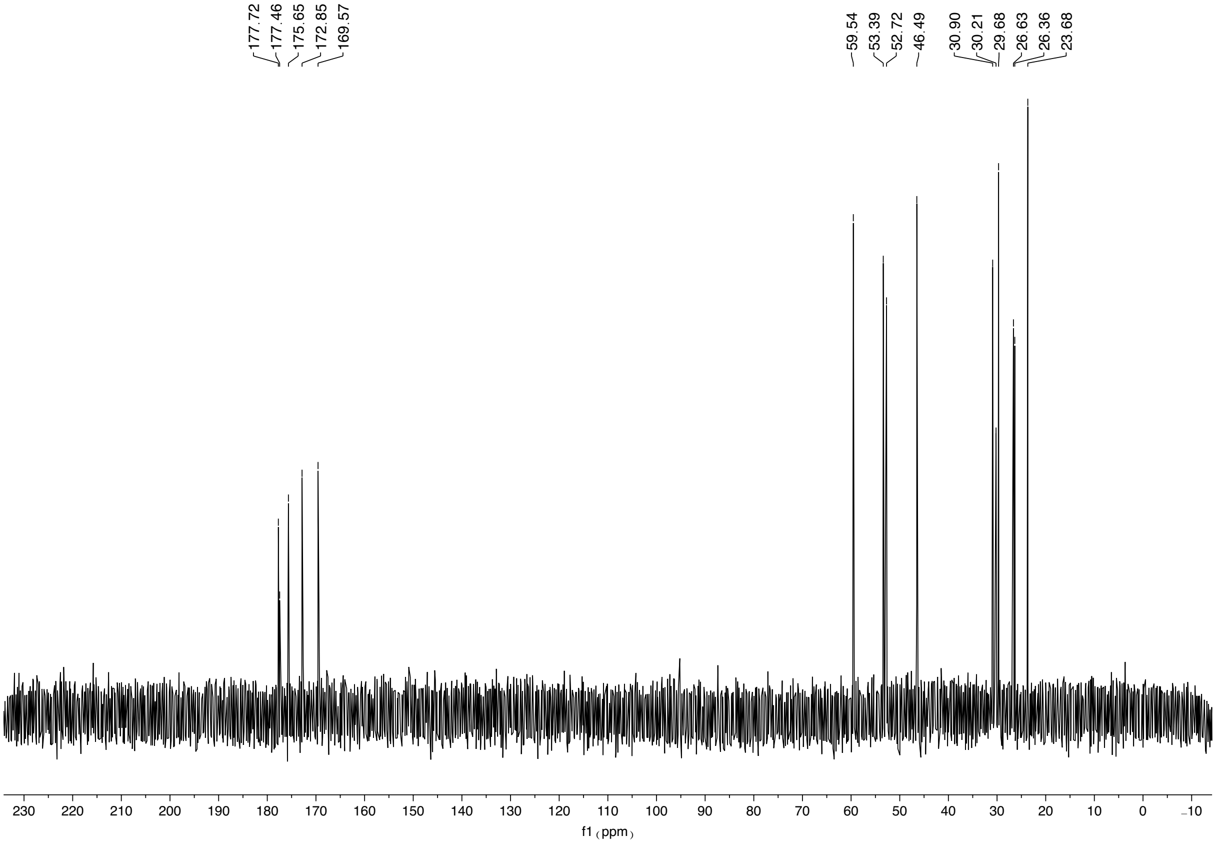

^{1}H and ^{13}C-NMR of H-D-Pro-D-Gln-L-Tyr-NH_2 · TFA (UTS-106):

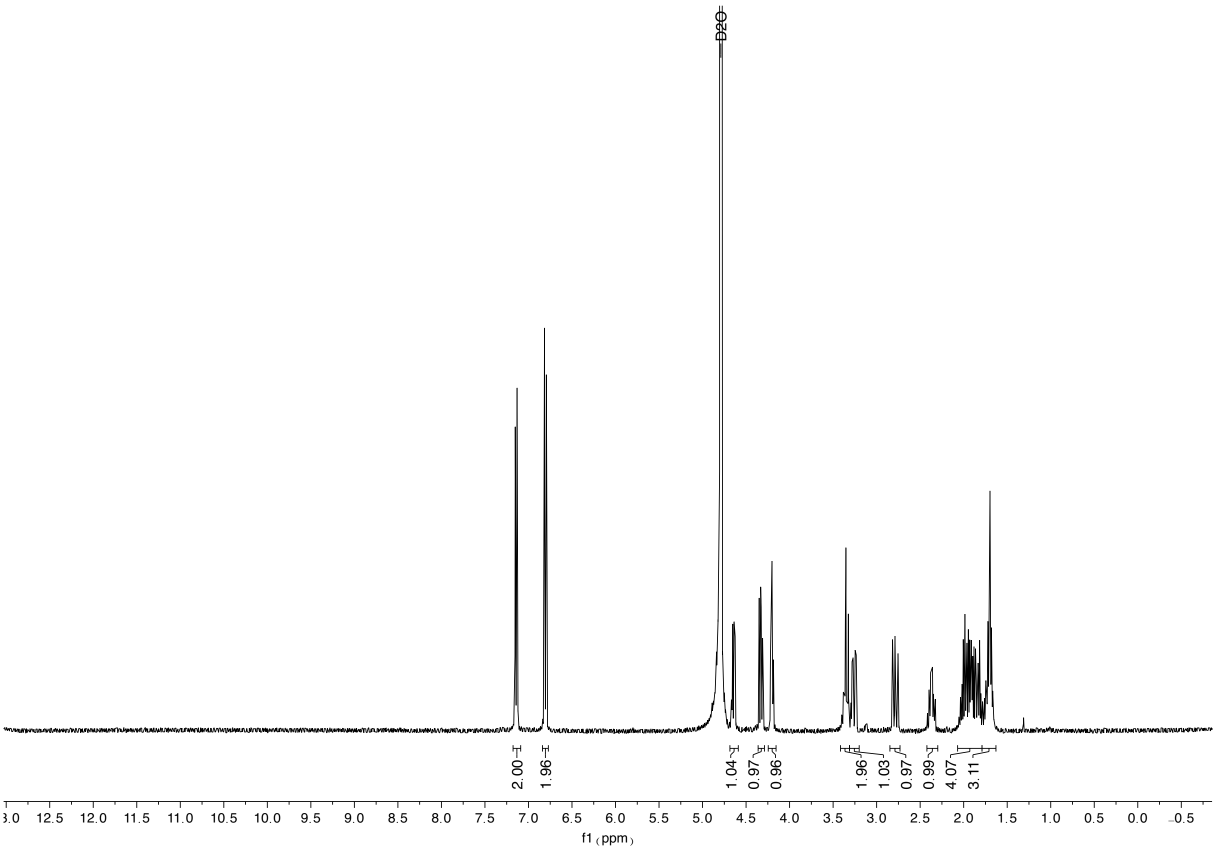

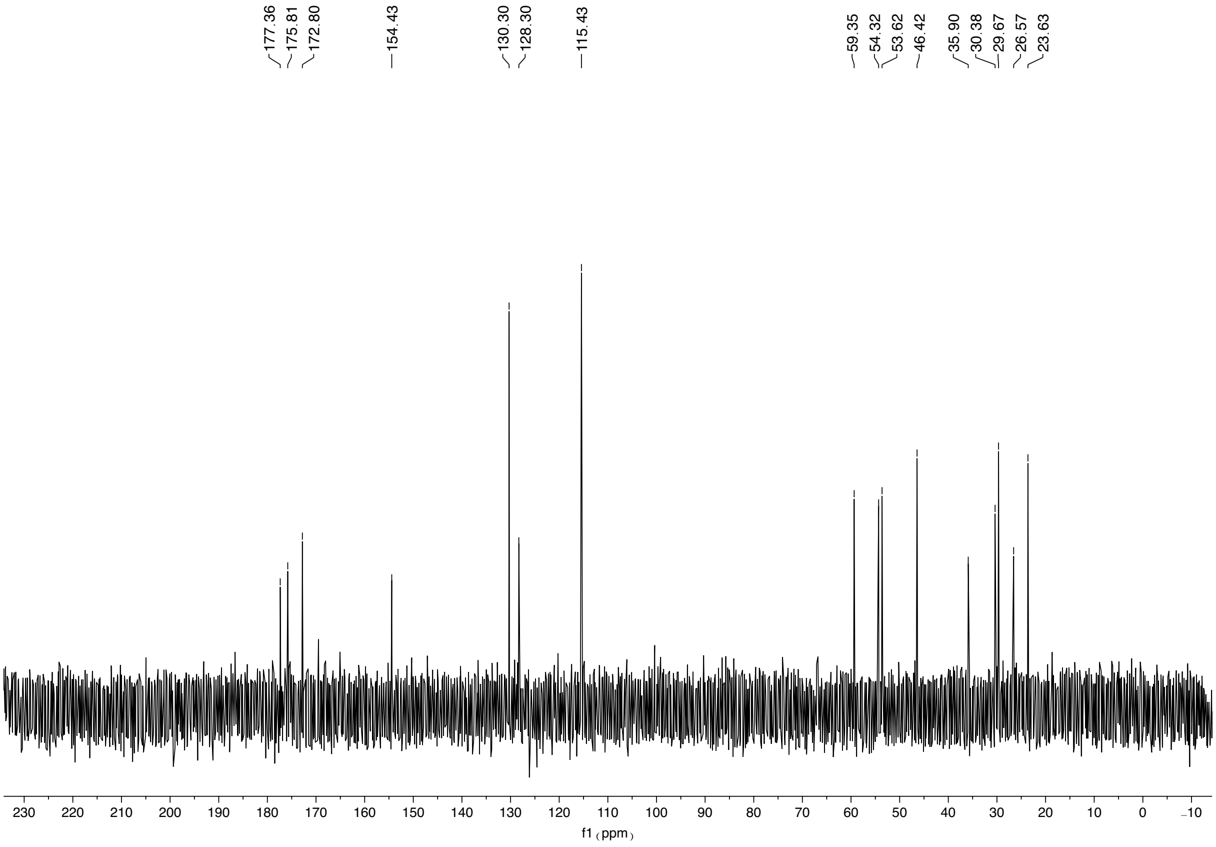

^{1}H and ^{13}C-NMR of H-D-Pro-D-Gln-D-Tyr-NH$_2$ · TFA (UTS-107):

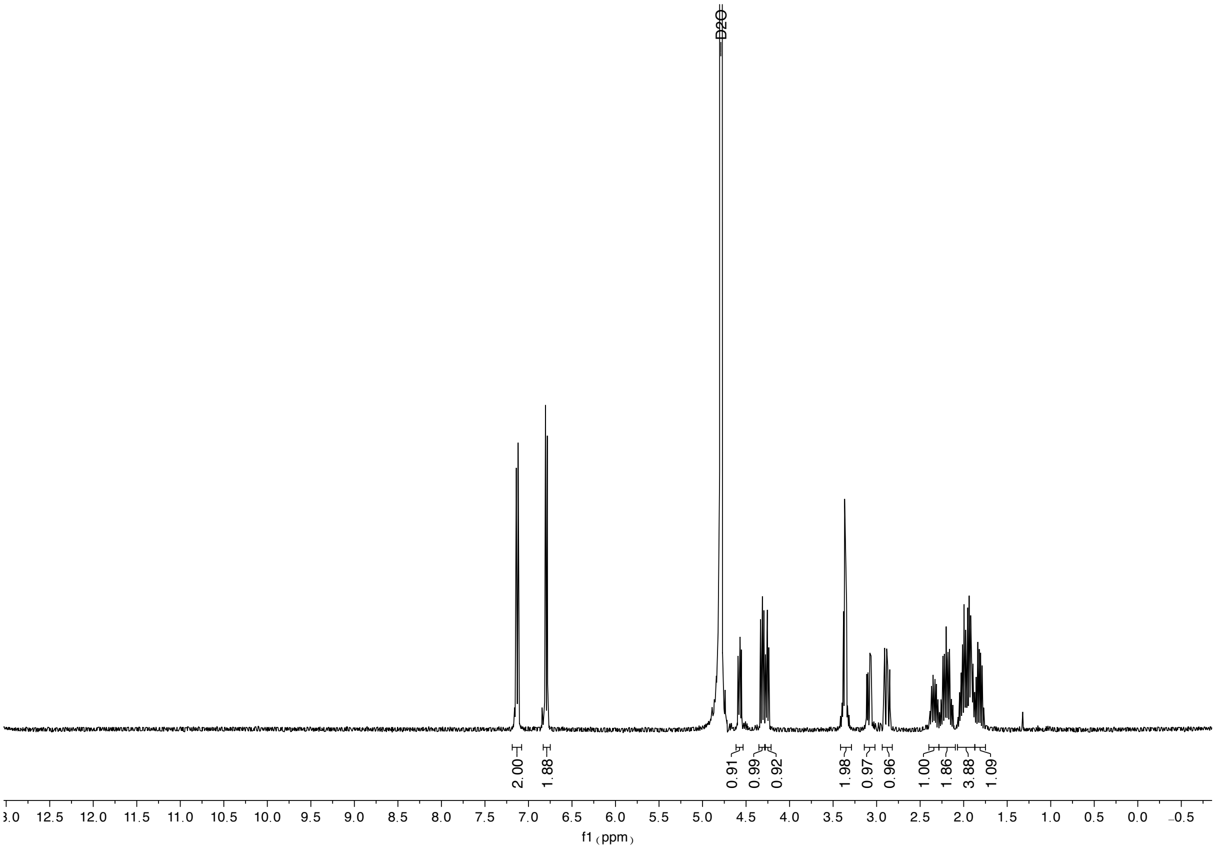

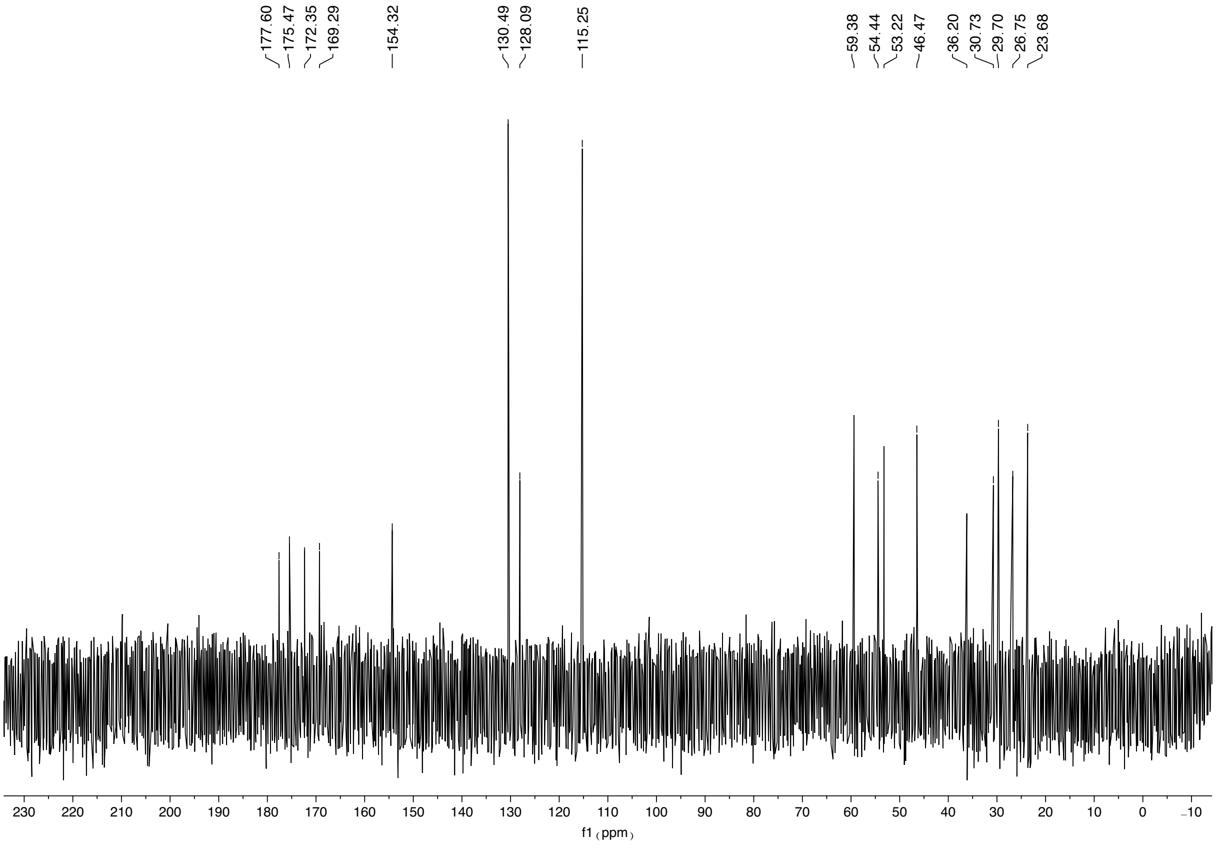

^{1}H and ^{13}C-NMR of H-D-Pro-D-Gln-CyLeu-NH$_2$ · TFA (UTS-108):

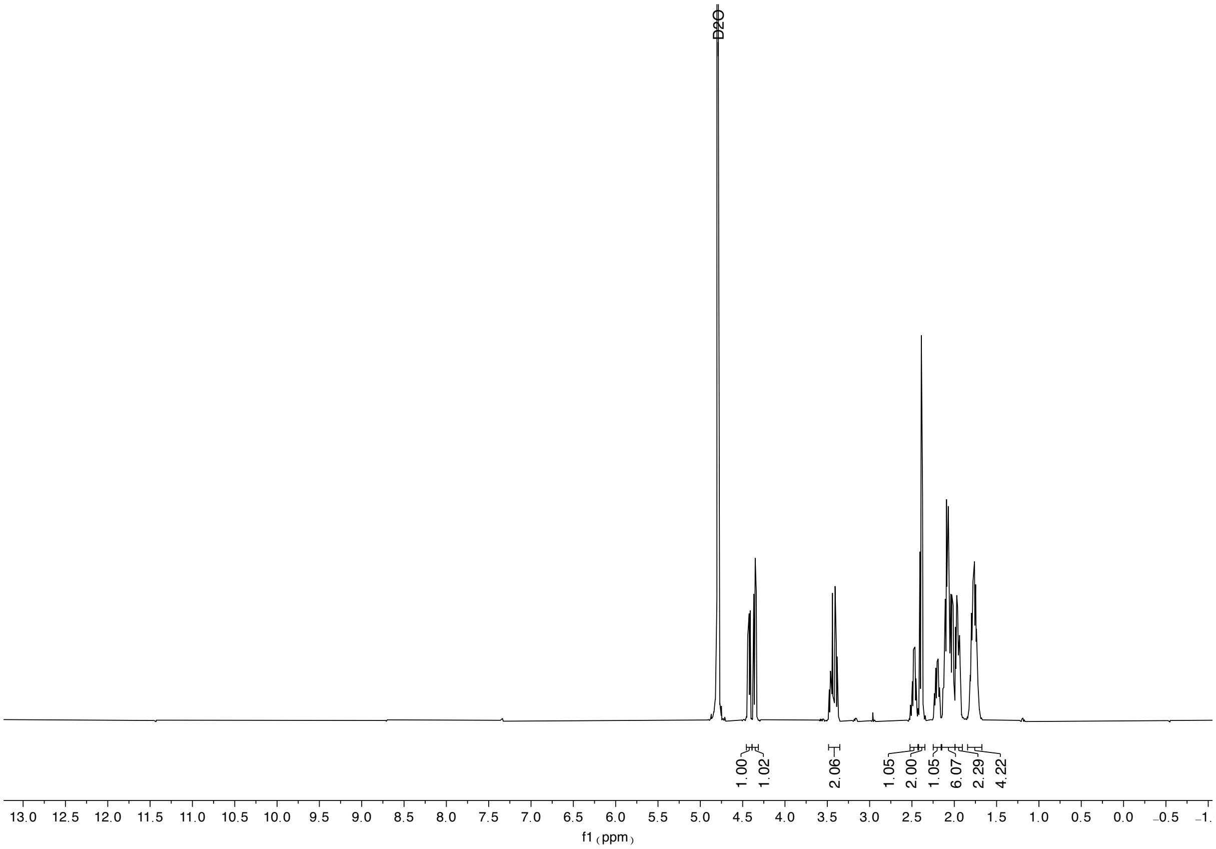

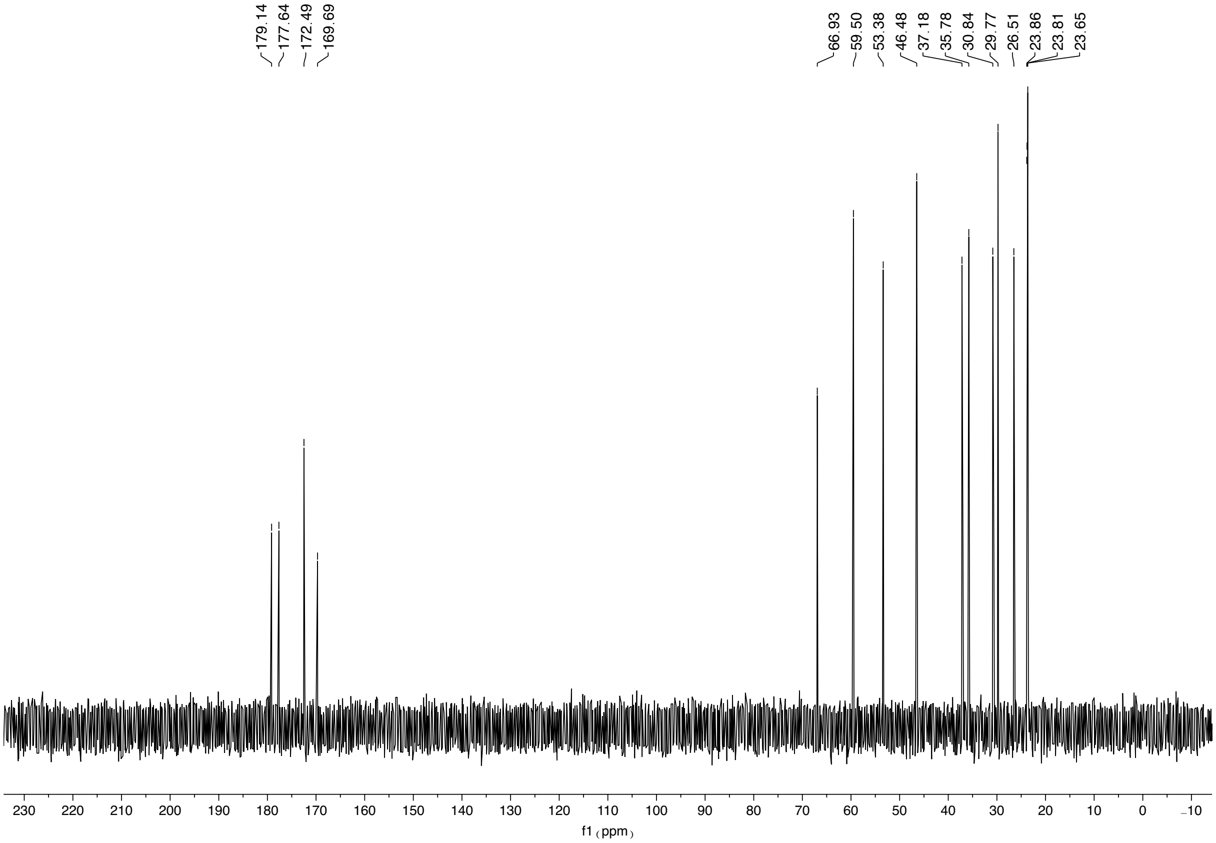

^{1}H and ^{13}C-NMR of H-D-Pro-D-Gln-Abz-NH$_2$ · TFA (UTS-109):

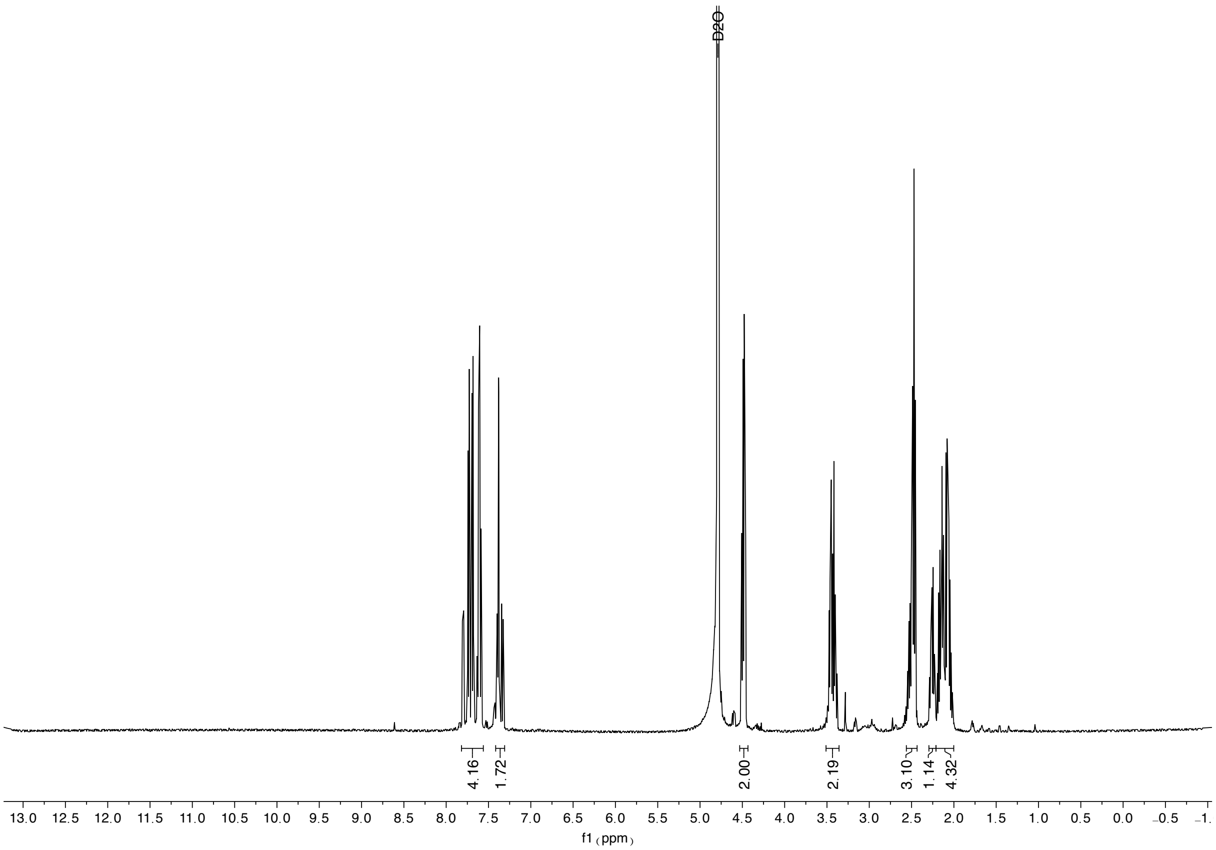

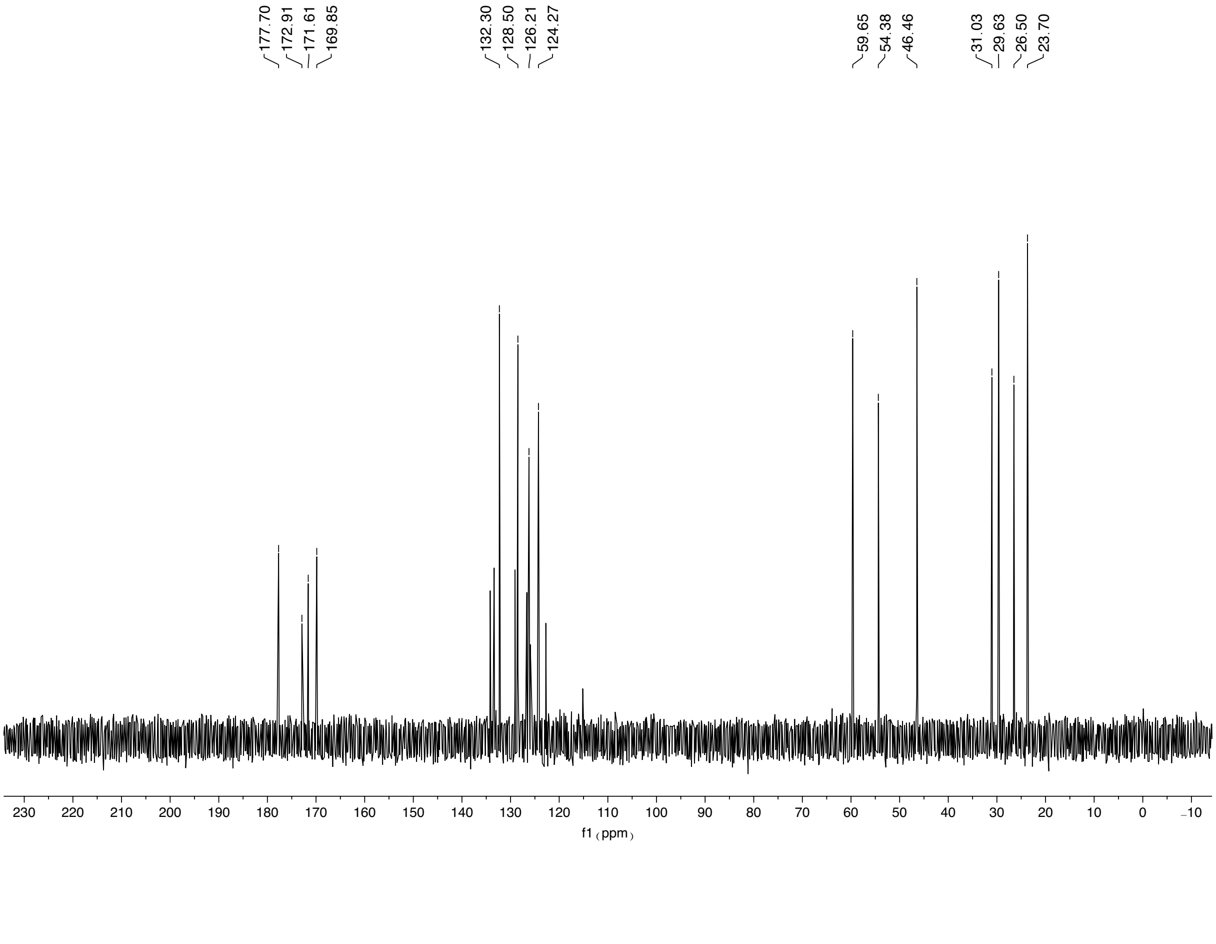

^{1}H and ^{13}C-NMR of H-D-Pro-D-Gln-D-Ind-NH$_2$ · TFA (UTS-110):

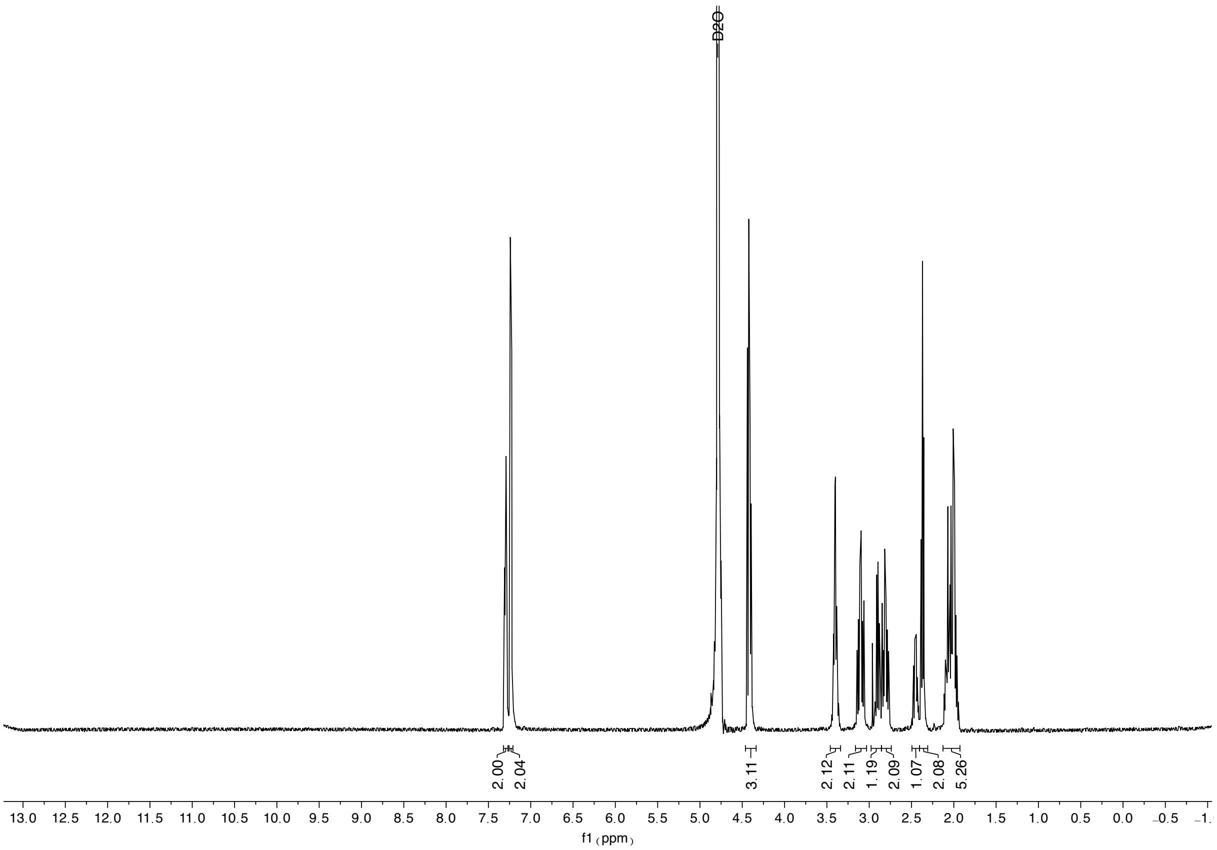

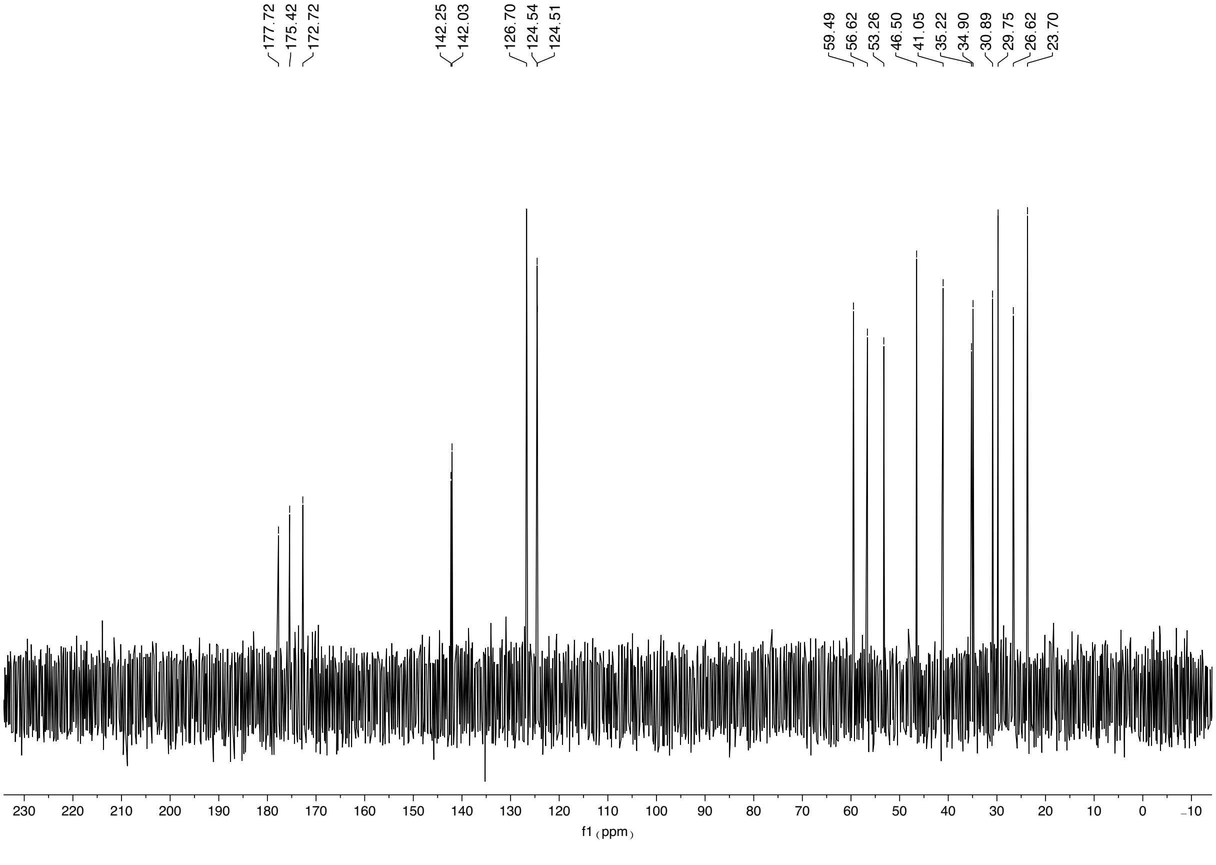

^{1}H and ^{13}C-NMR of H-D-Pro-L-Glu-D-Gln-NH$_2$ · TFA (UTS-111):

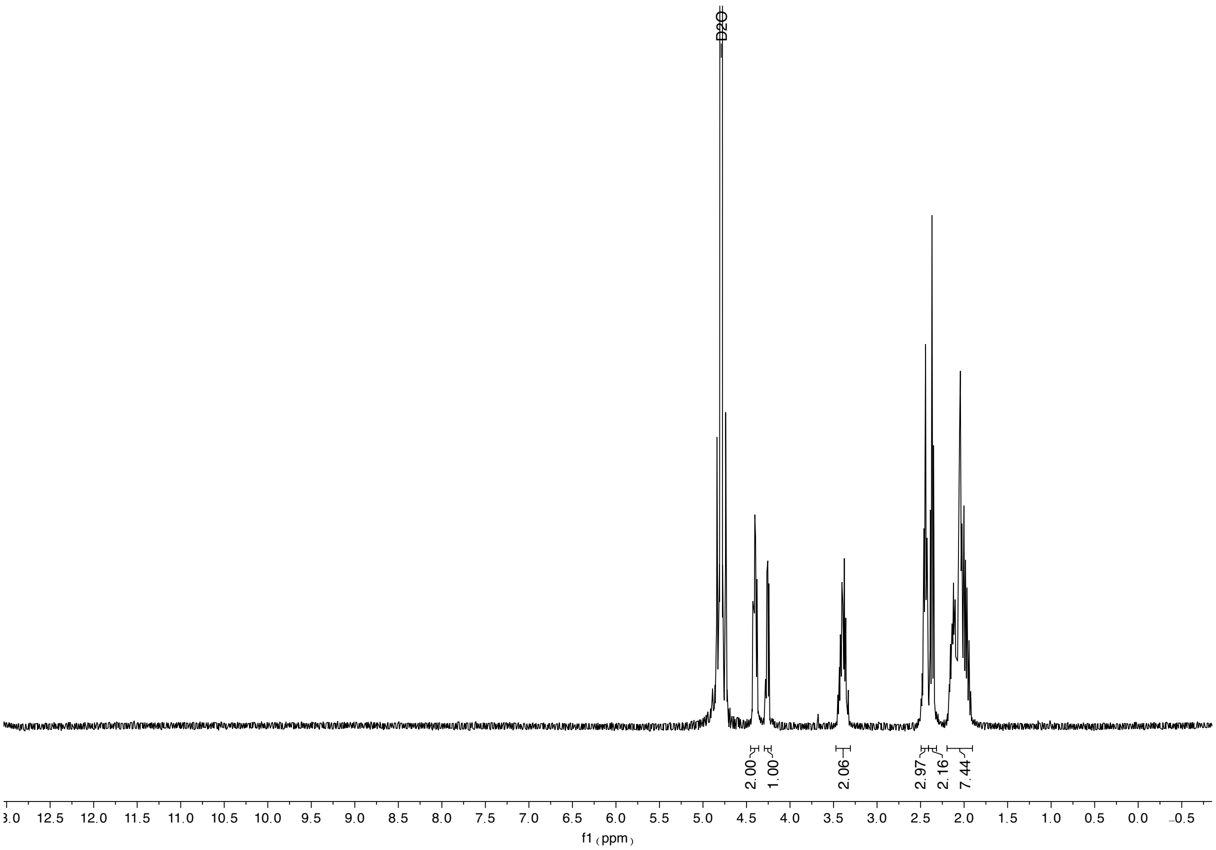

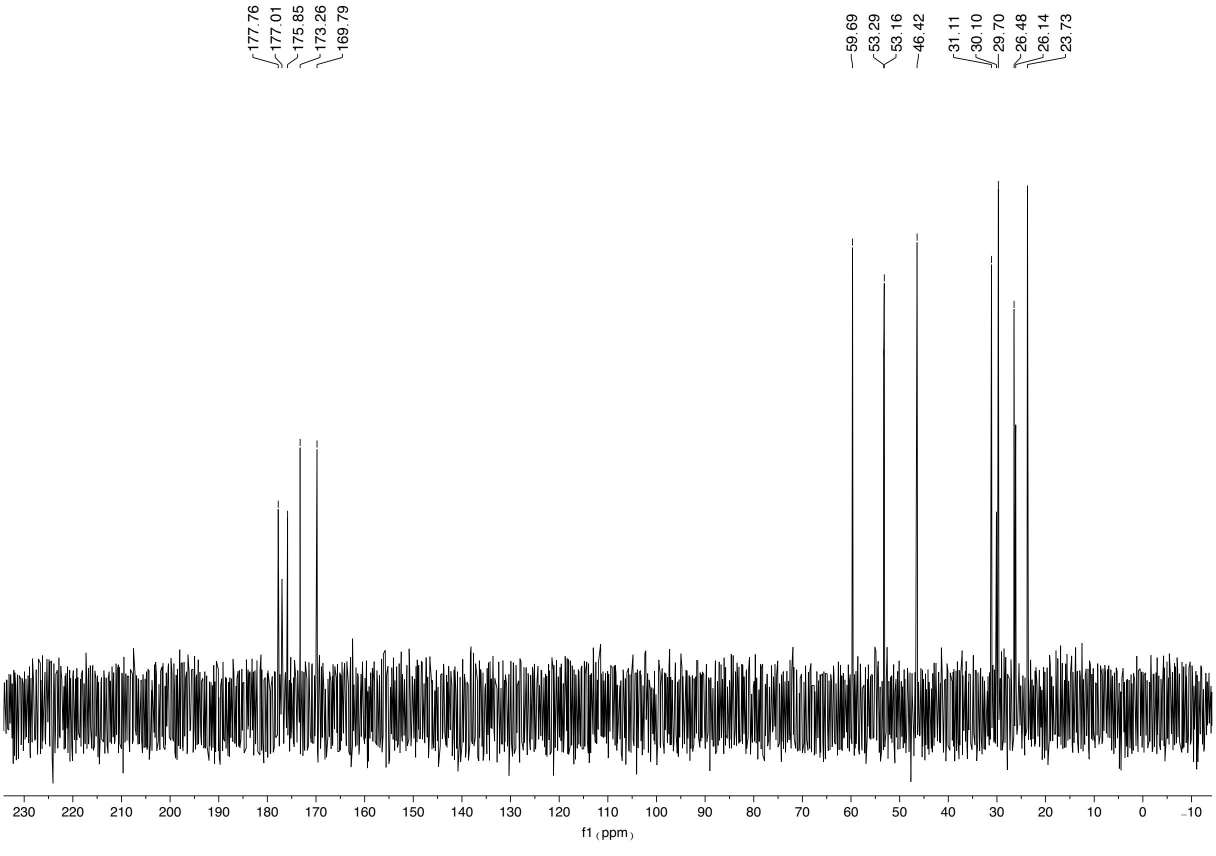

^{1}H and ^{13}C-NMR of H-D-Pro-D-Glu-D-Gln-NH_2 · TFA (UTS-112):

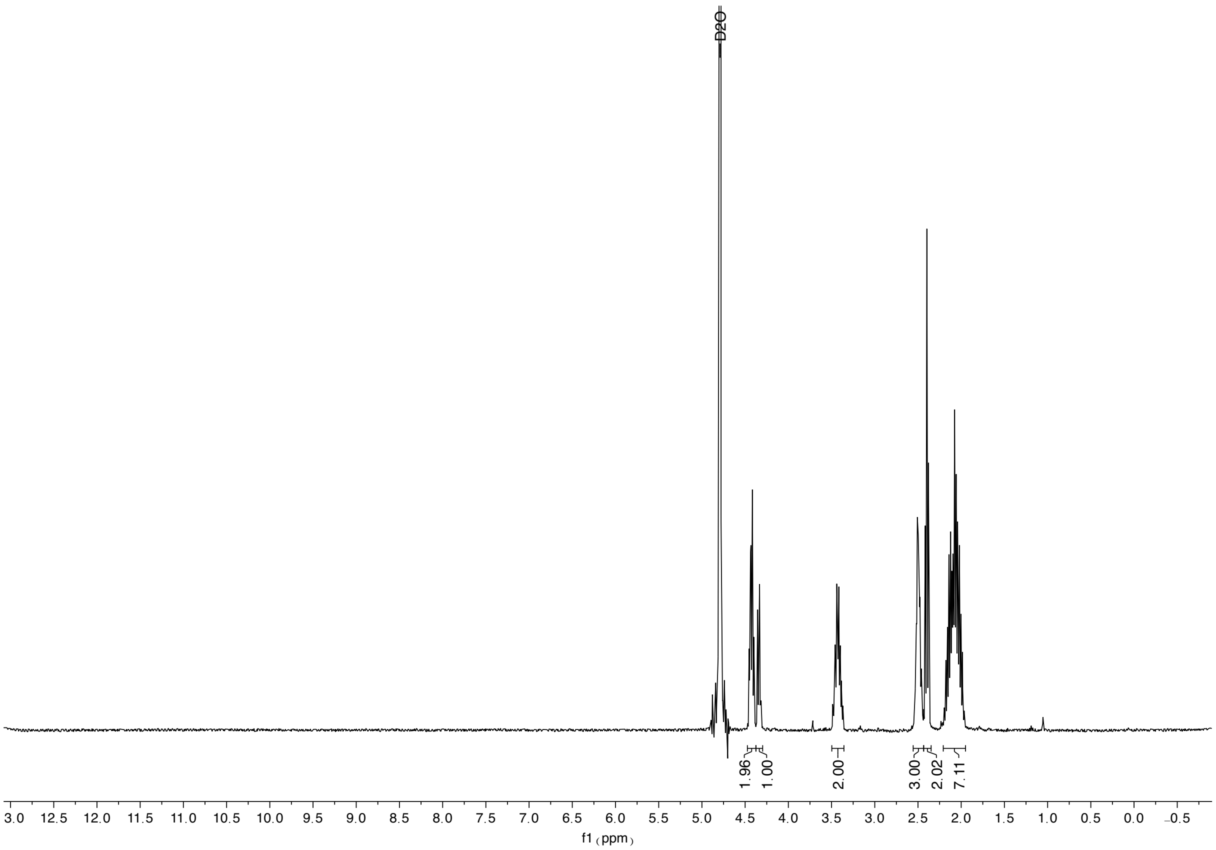

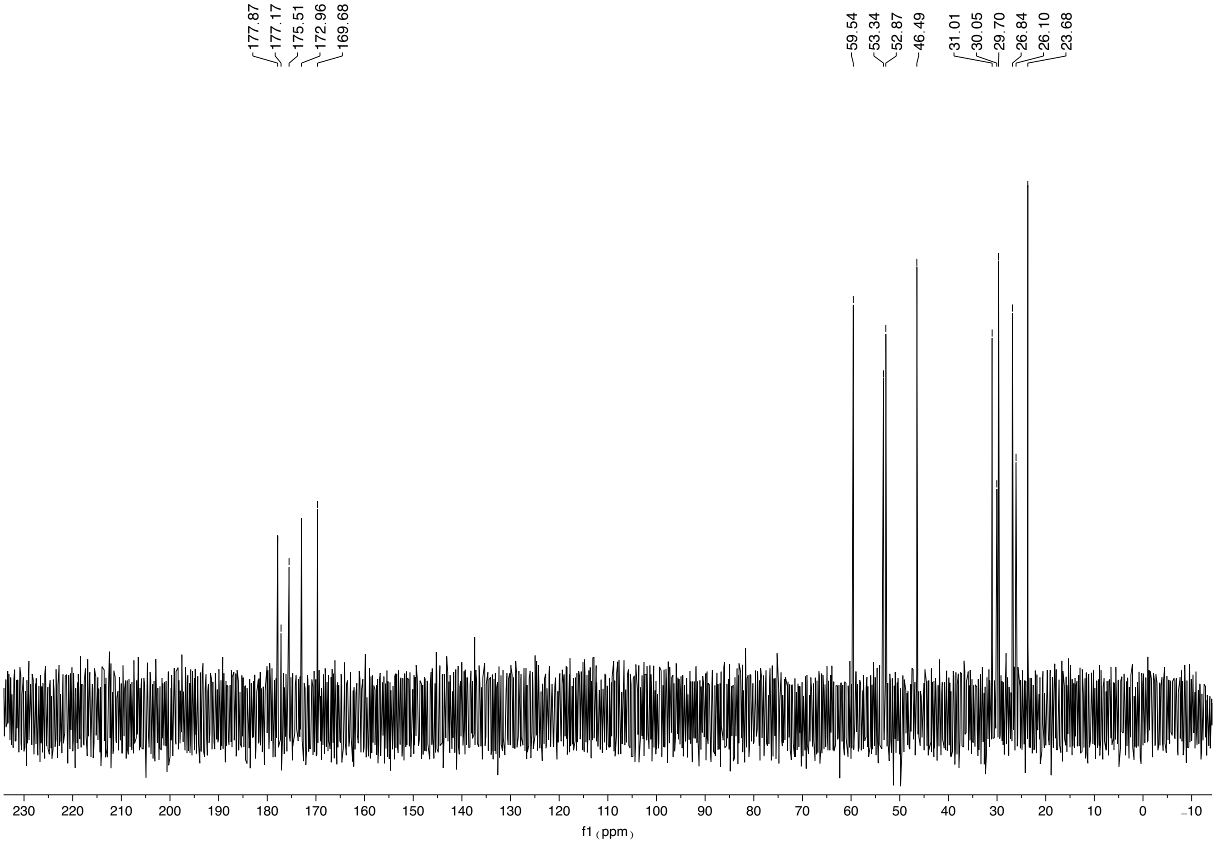

^{1}H and ^{13}C-NMR of H-D-Pro-L-Tyr-D-Gln-NH$_2$ · TFA (UTS-113):

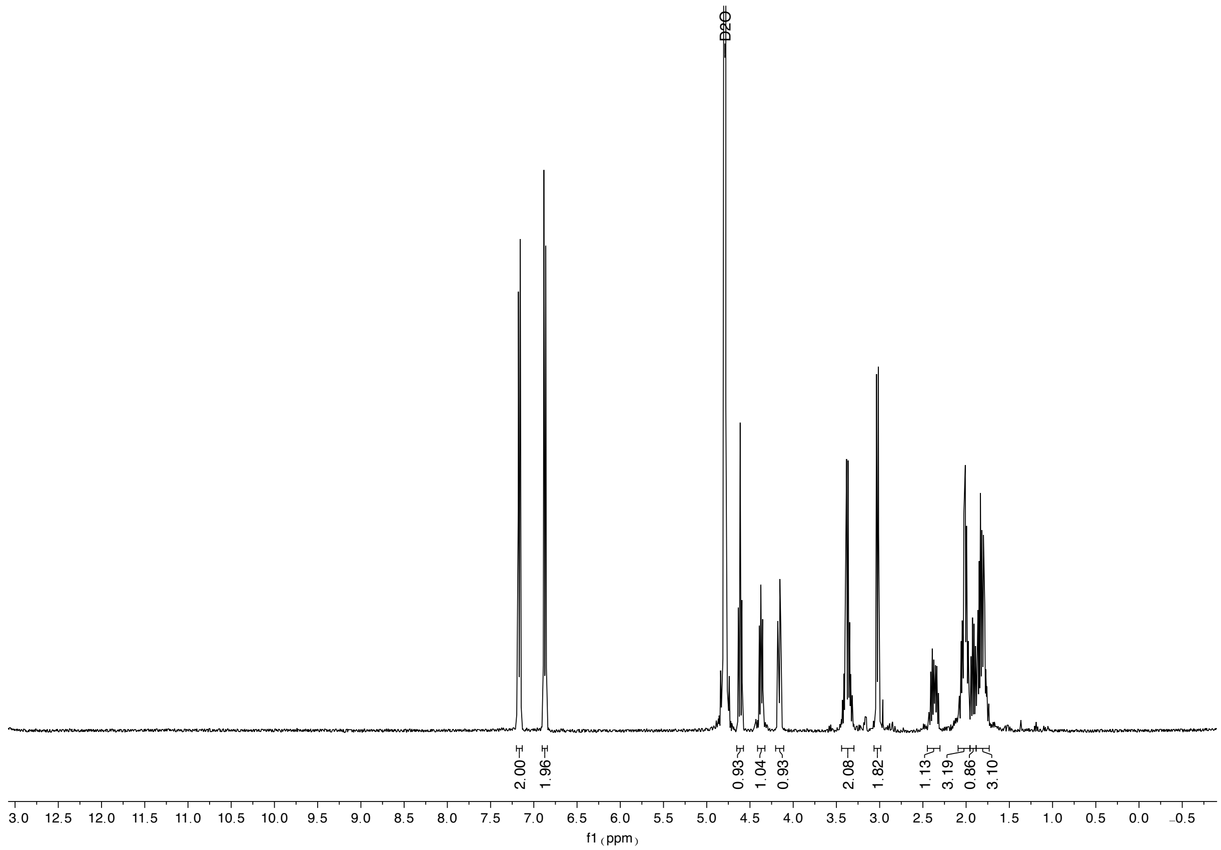

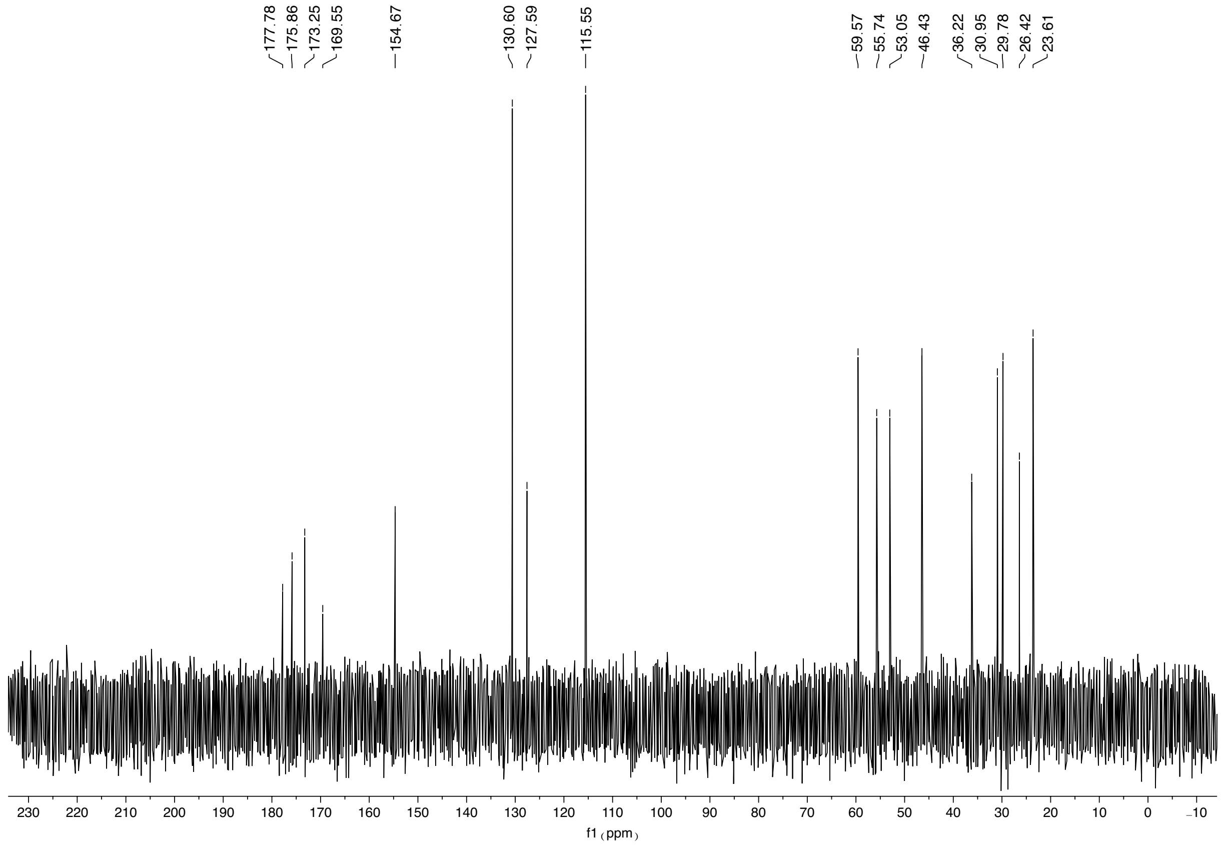

^{1}H and ^{13}C-NMR of H-D-Pro-D-Tyr-D-Gln-NH$_2$ · TFA (UTS-114):

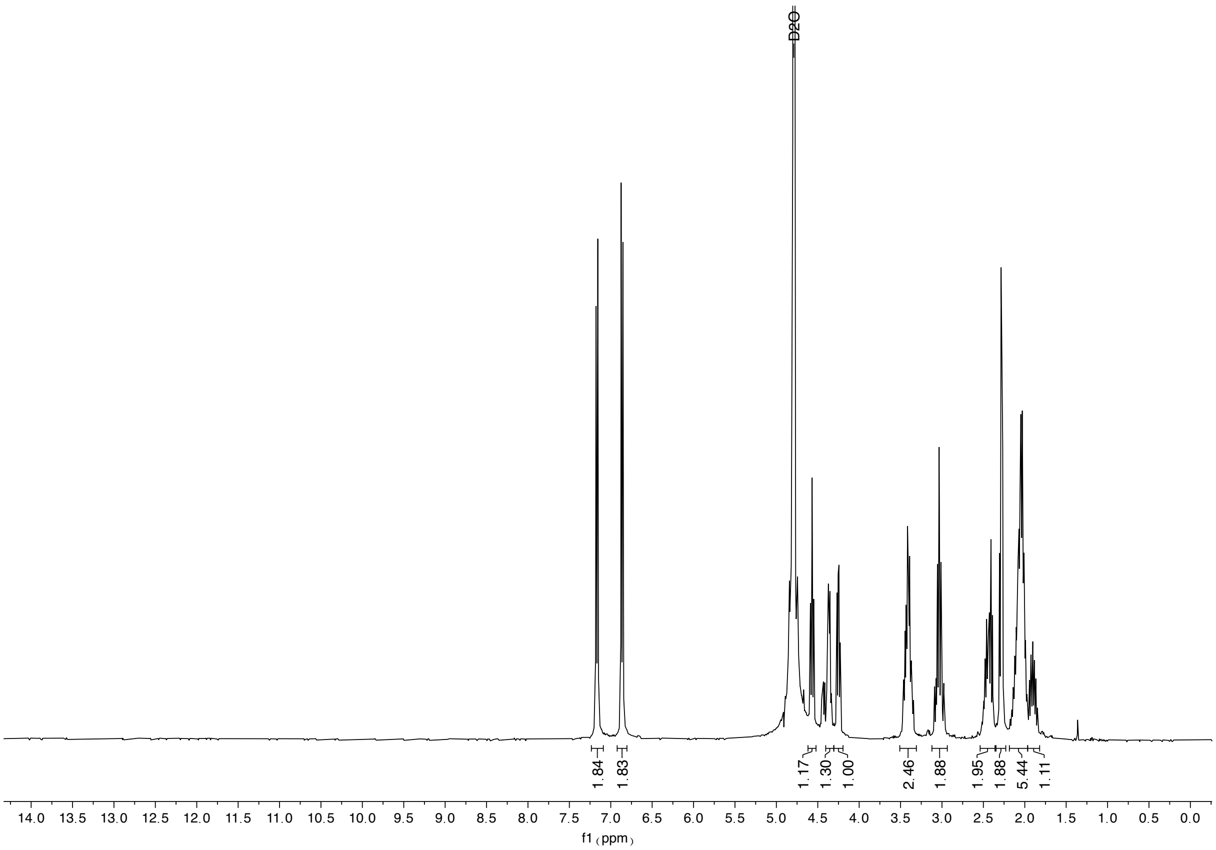

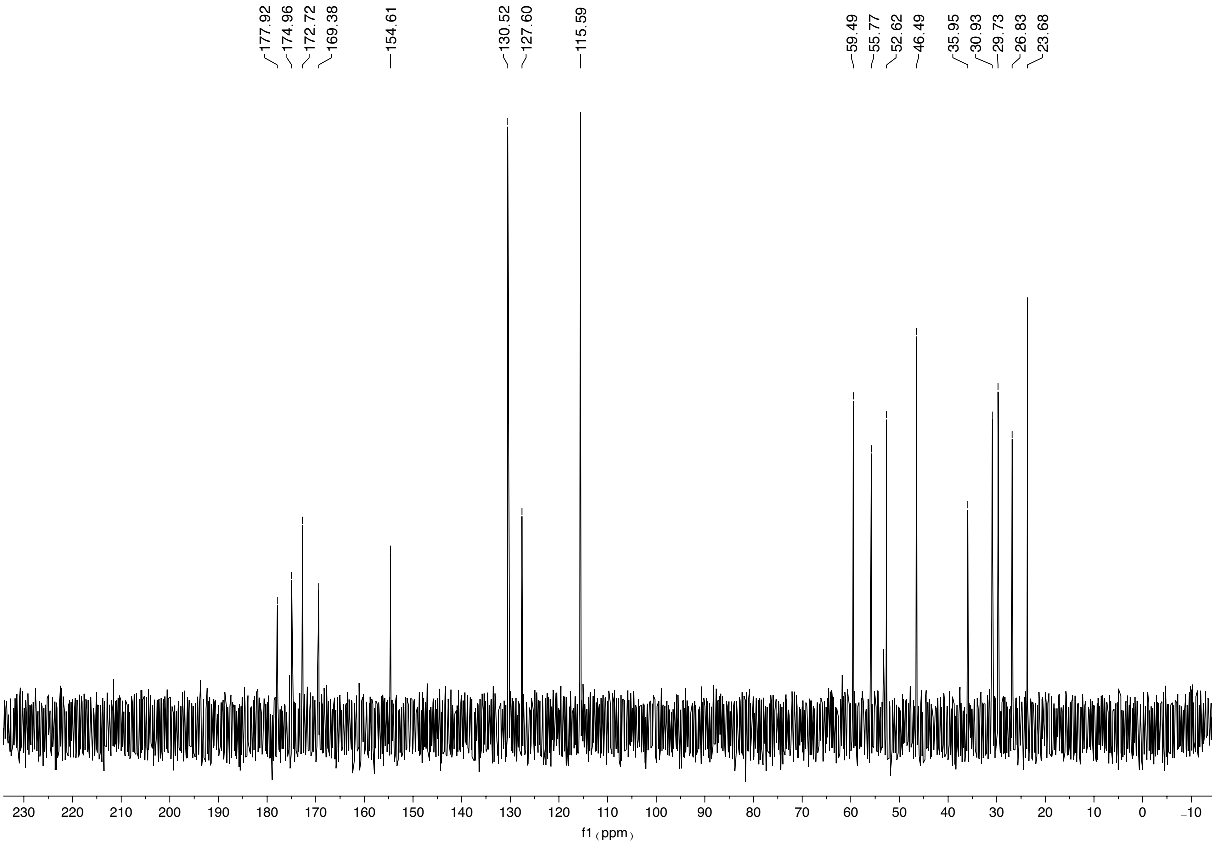

^{1}H and ^{13}C-NMR of H-D-Pro-CyLeu-D-Gln-NH$_2$ · TFA (UTS-115):

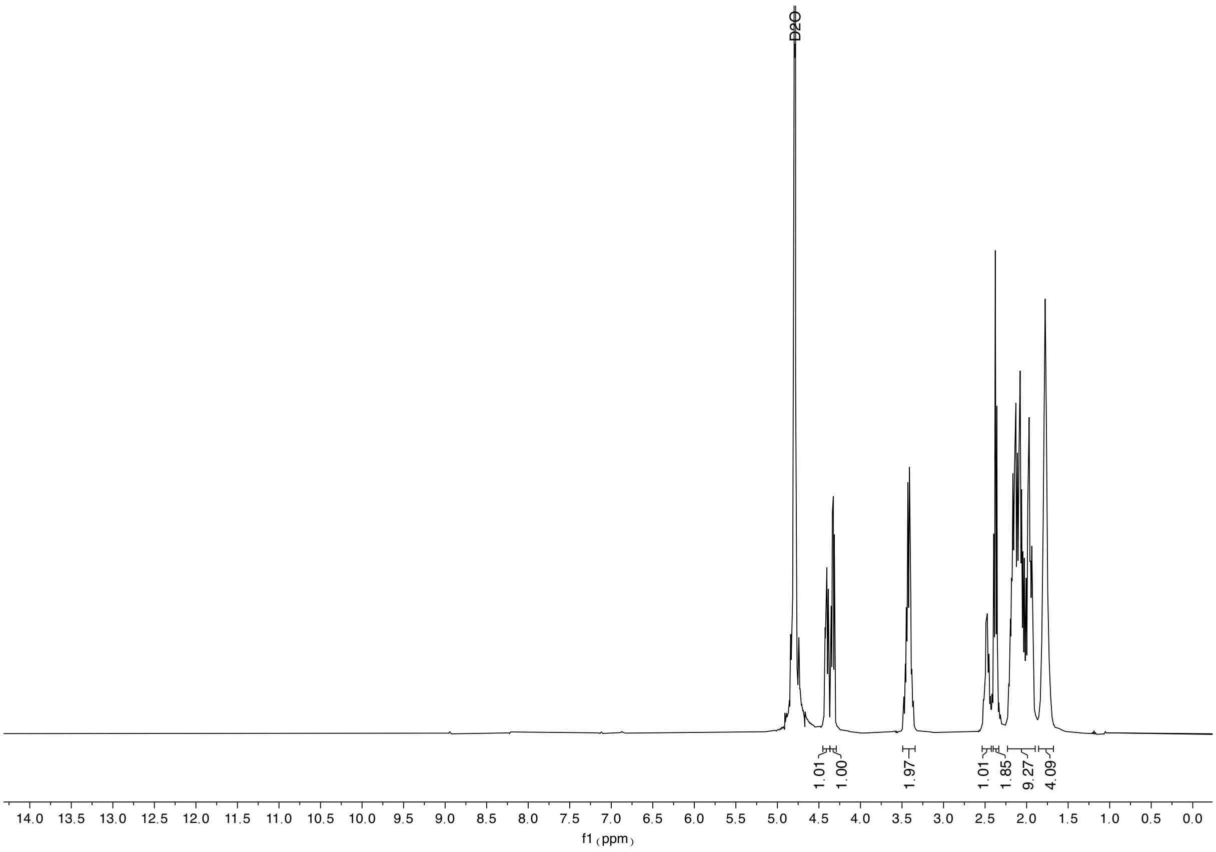

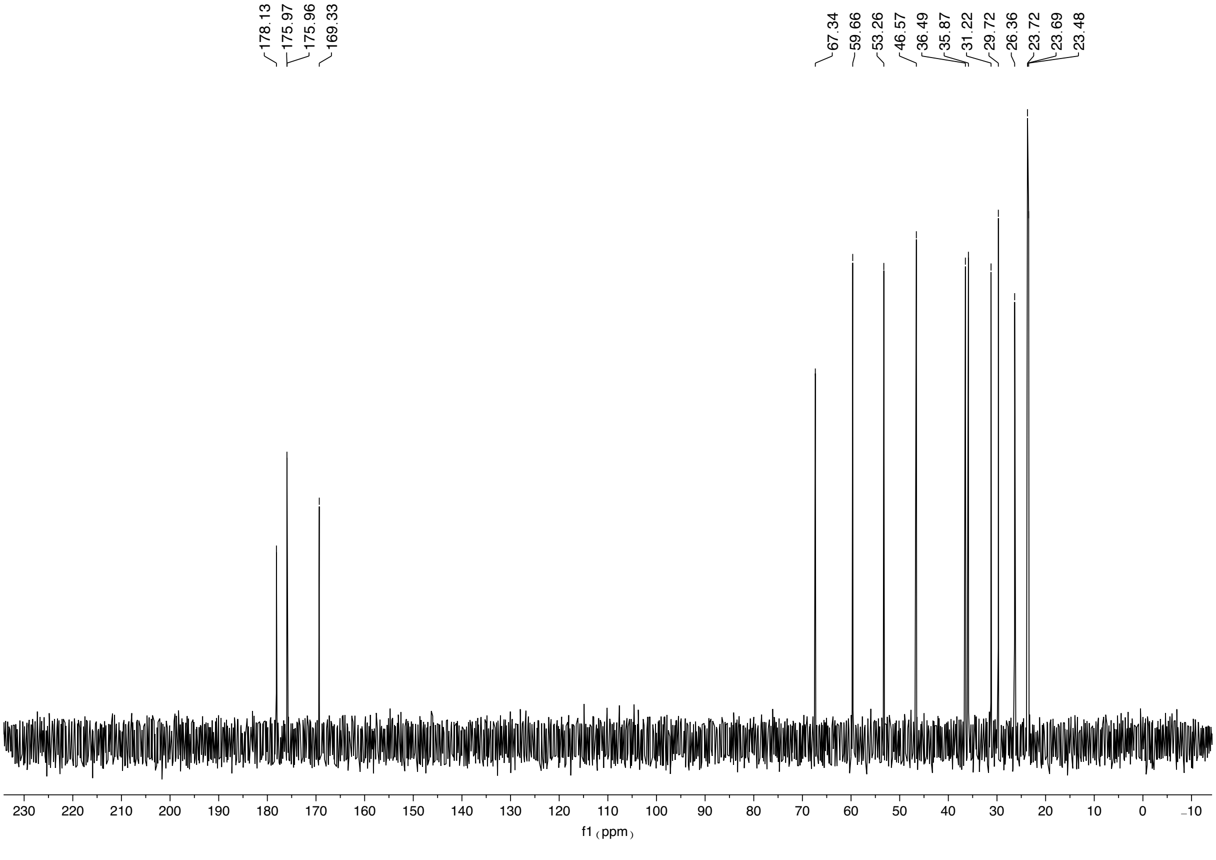

^{1}H and ^{13}C-NMR of H-D-Pro-Abz-D-Gln-NH₂ · TFA (UTS-116):

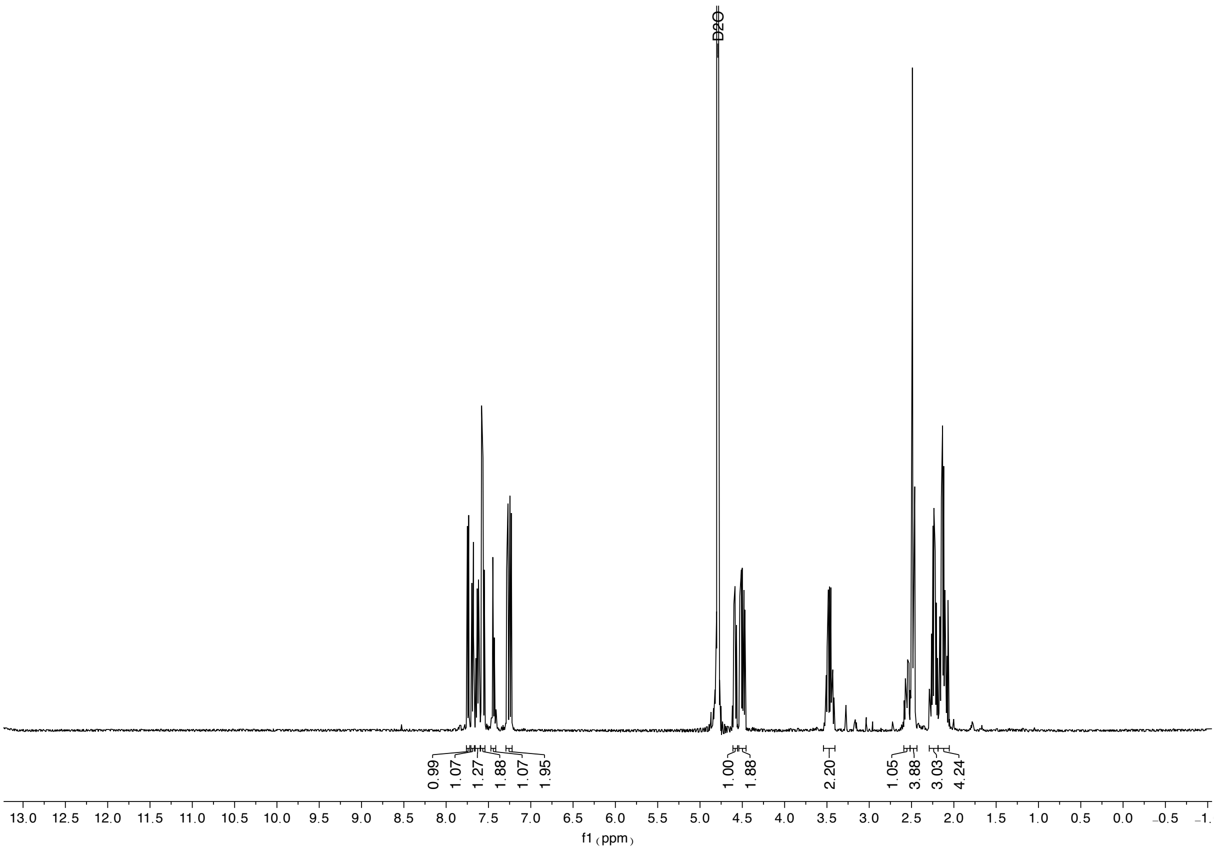

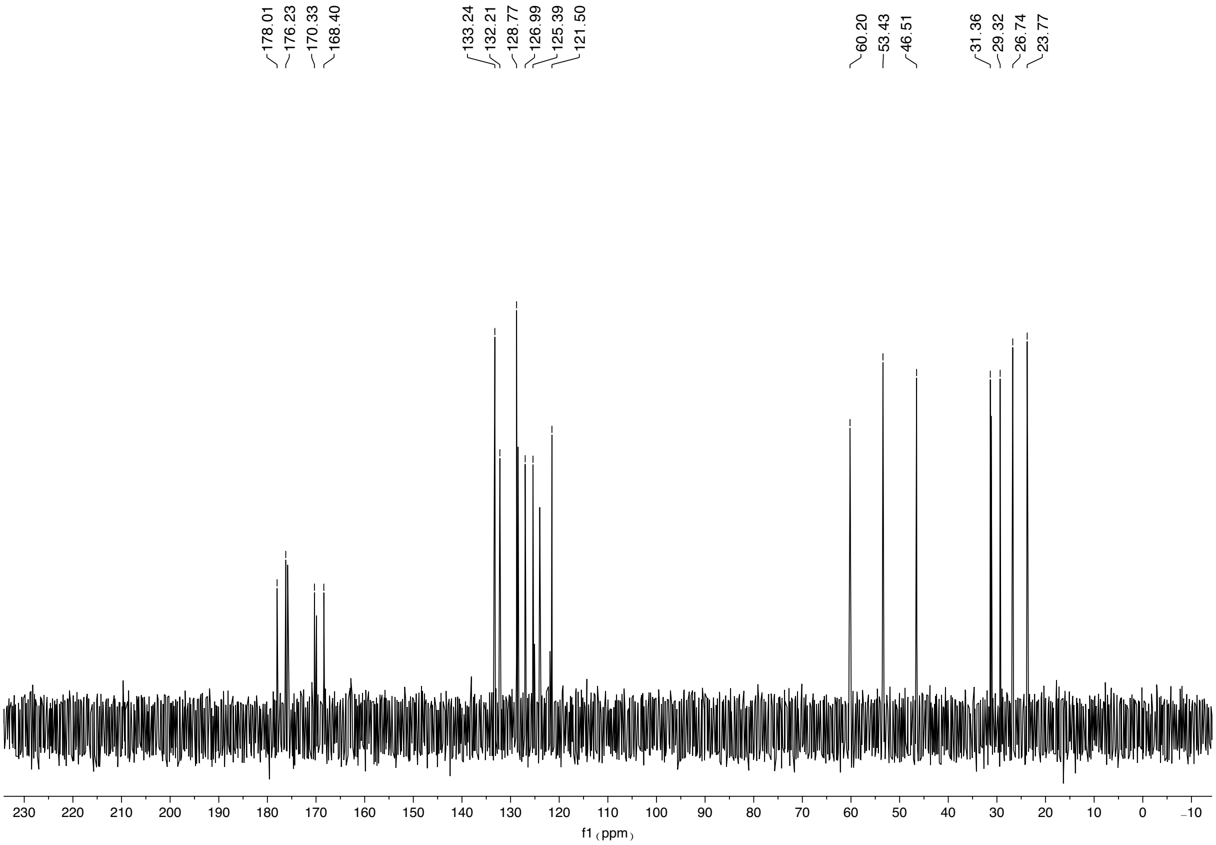

^{1}H and ^{13}C-NMR of H-D-Pro-D-Ind-D-Gln-NH$_2$ · TFA (UTS-117):

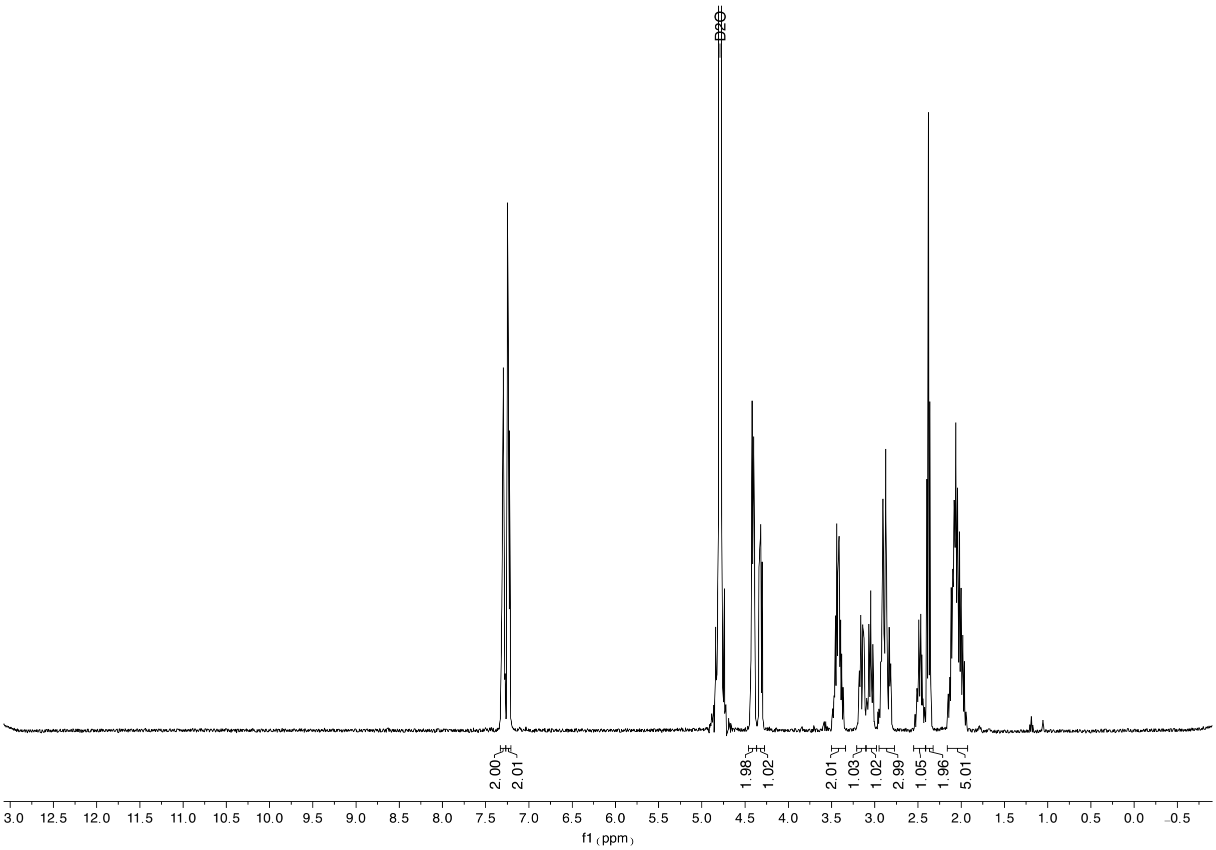

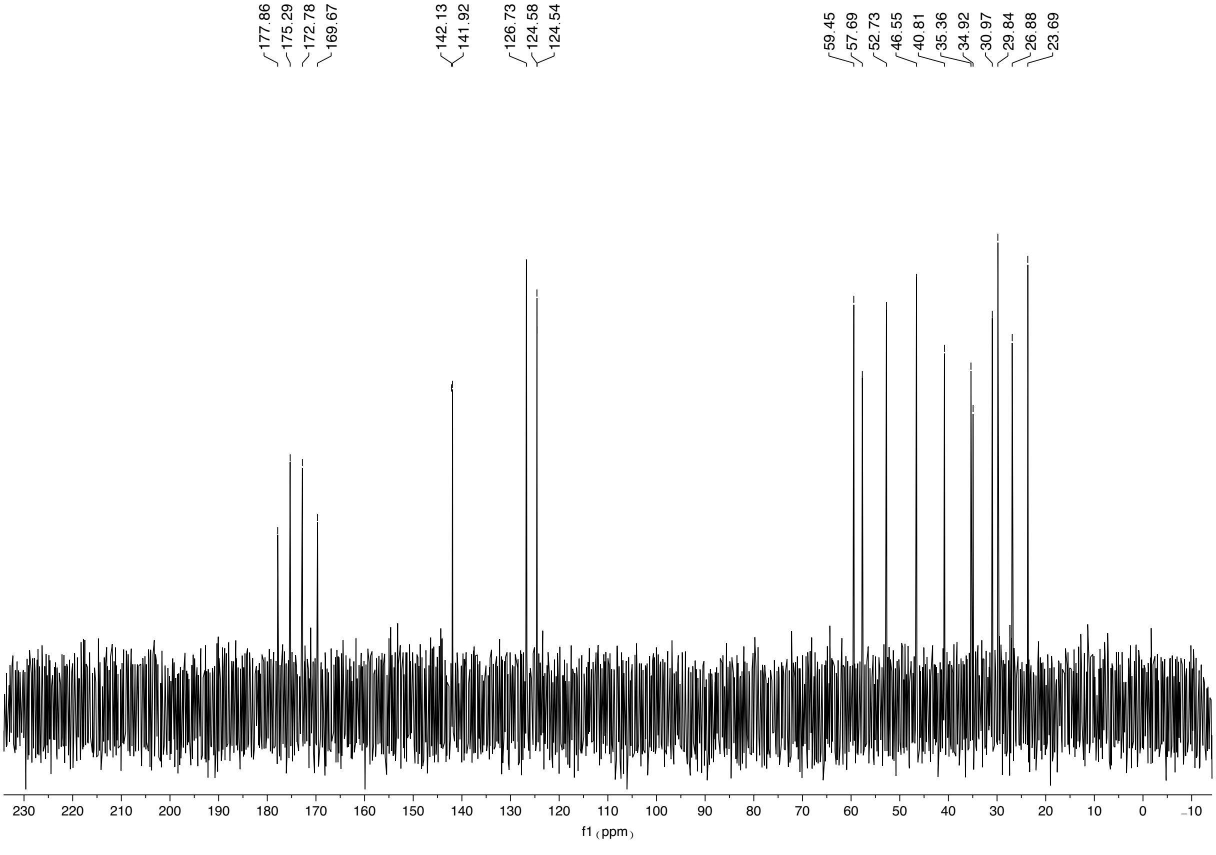

^{1}H and ^{13}C-NMR of H-D-Pro-L-Glu-L-Glu-NH$_2$ · TFA (UTS-118):

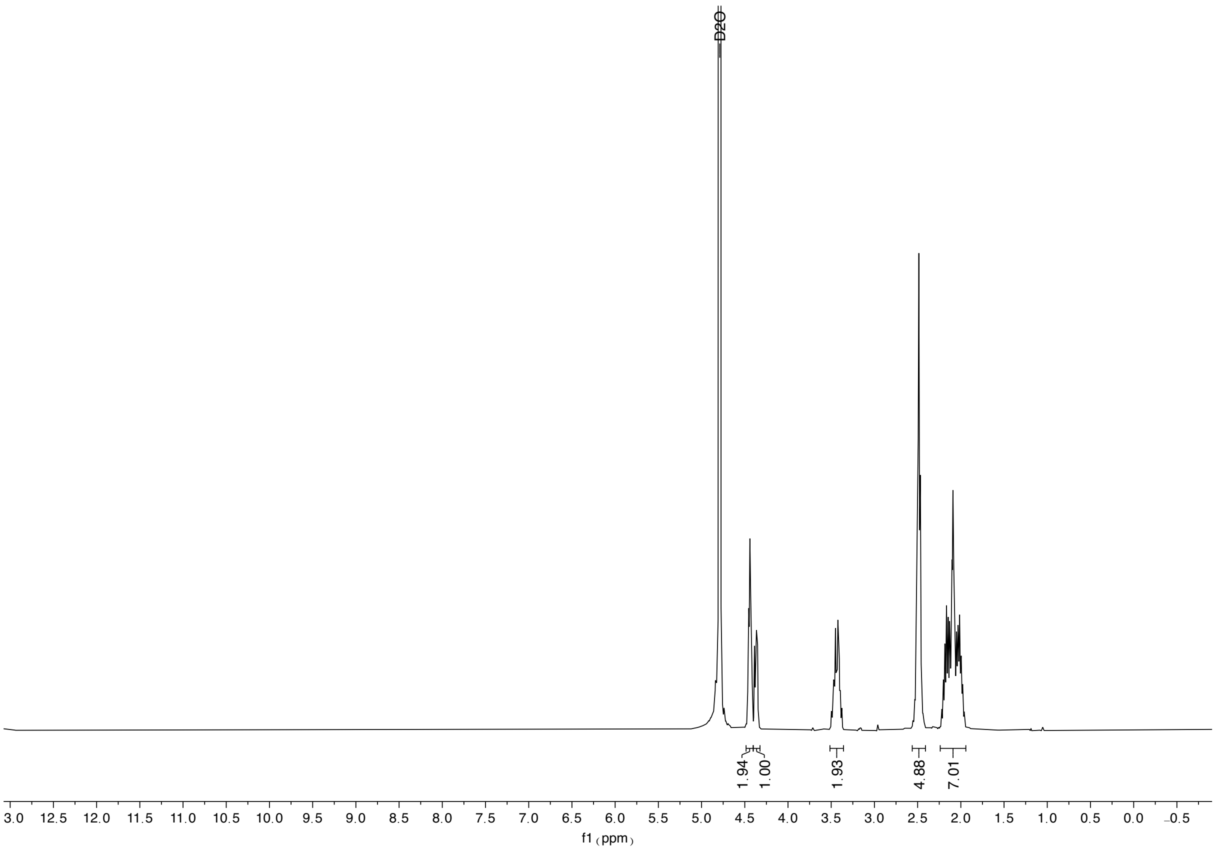

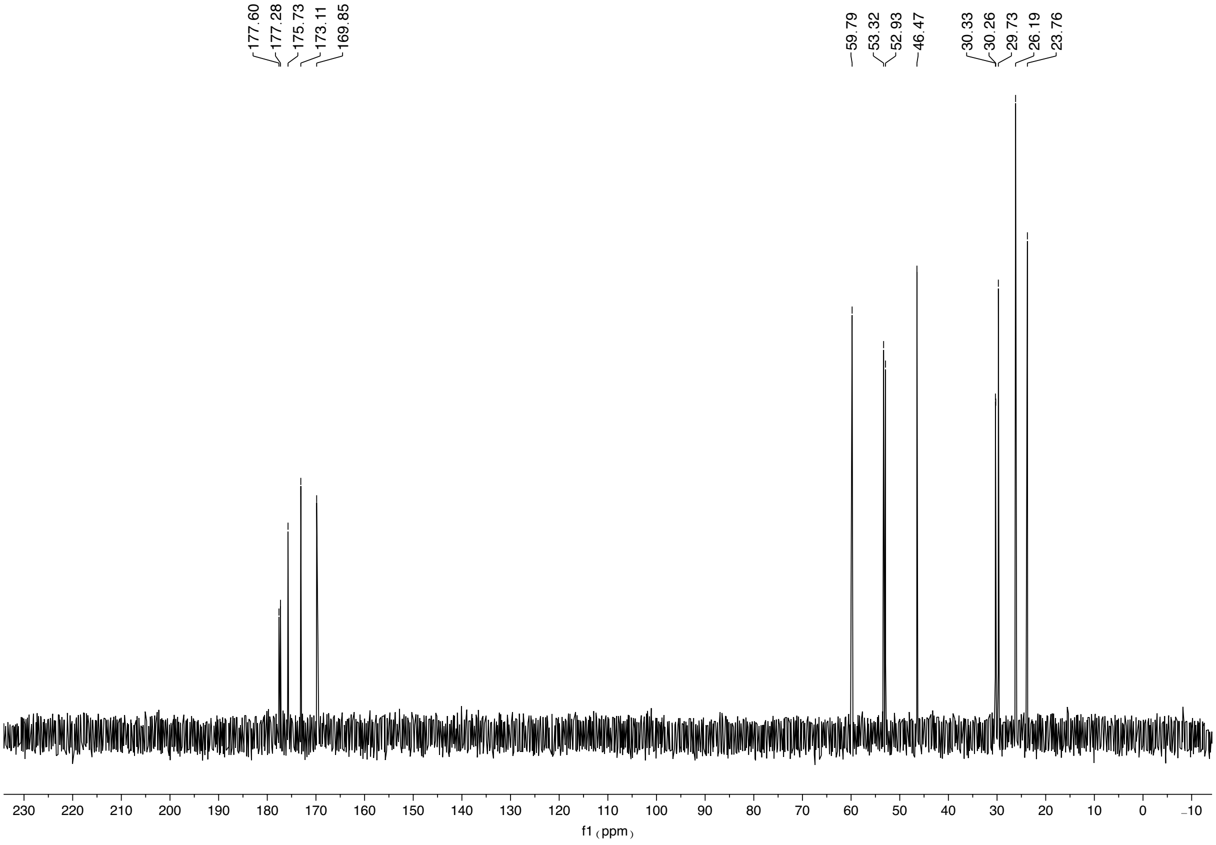

^{1}H and ^{13}C-NMR of H-D-Pro-L-Glu-D-Glu-NH$_2$ · TFA (UTS-119):

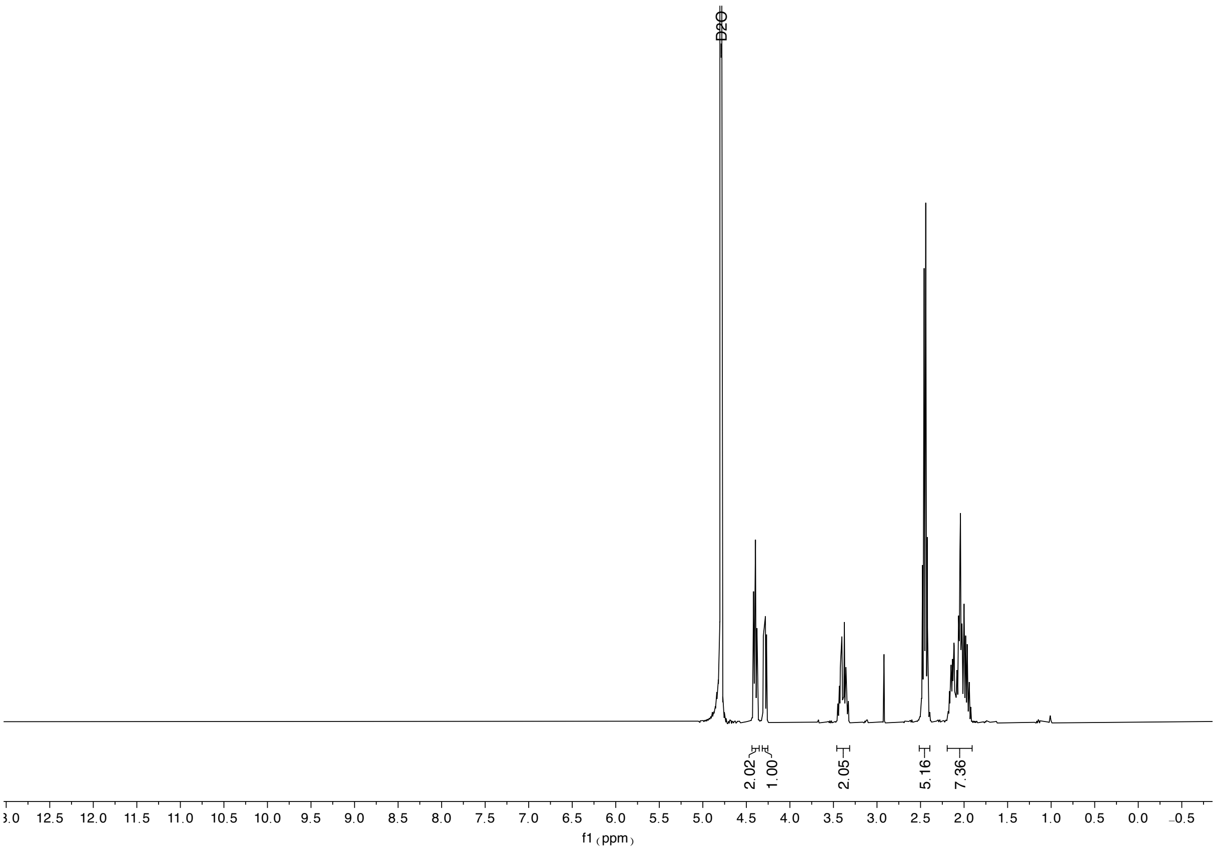

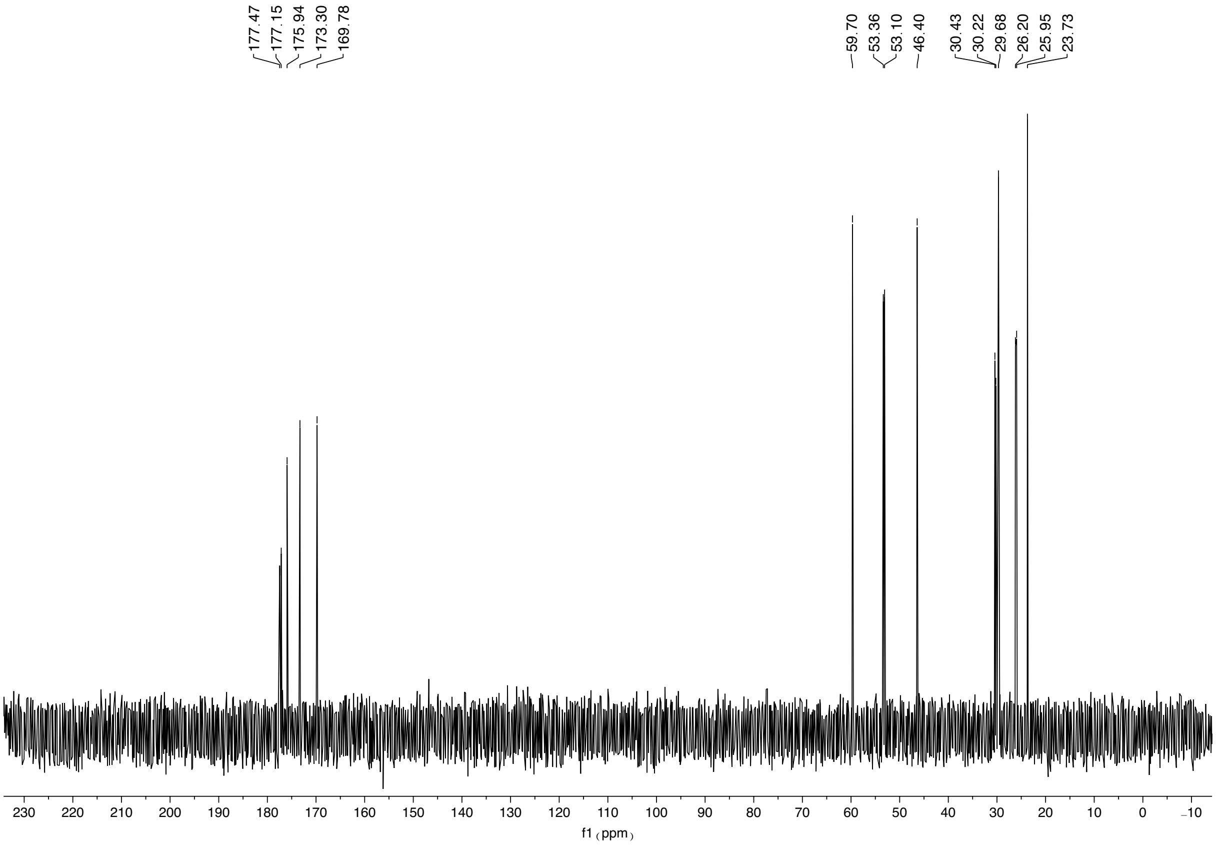

^{1}H and ^{13}C-NMR of H-D-Pro-L-Glu-L-Tyr-NH_2 · TFA (UTS-120):

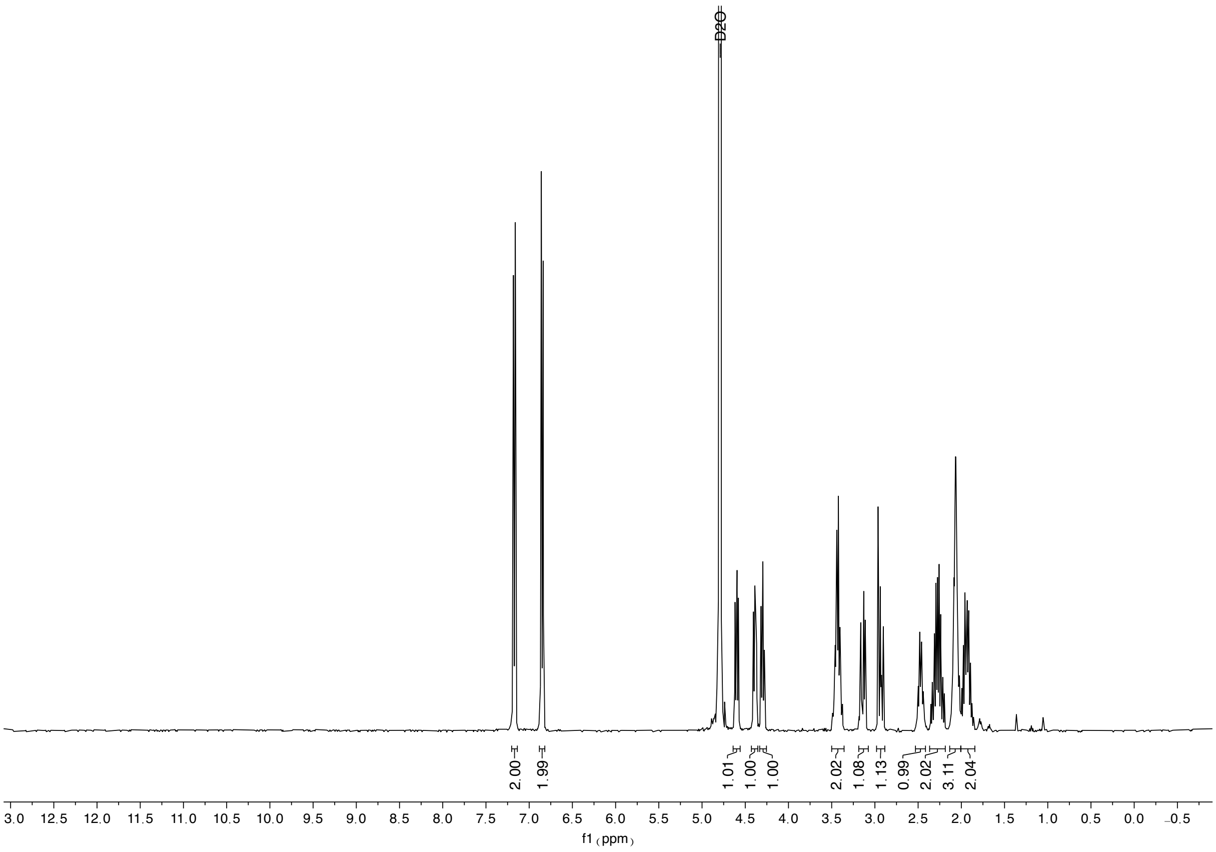

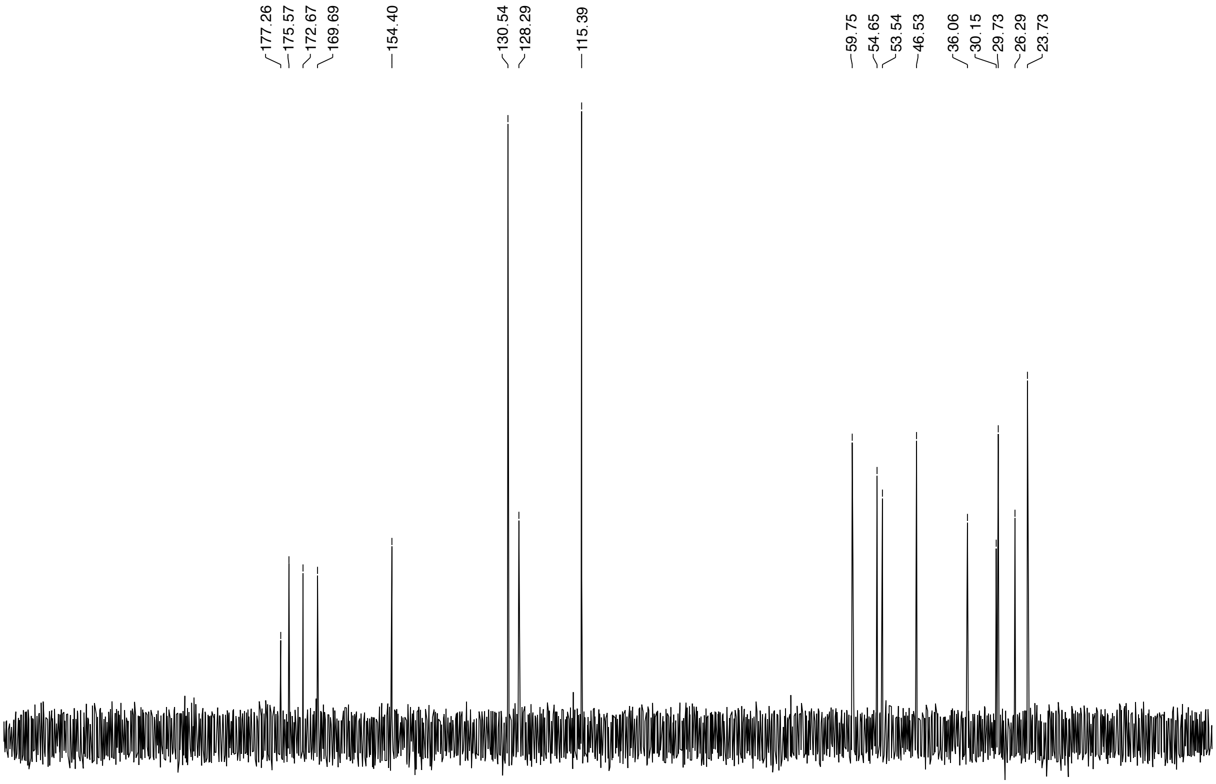

^{1}H and ^{13}C-NMR of H-D-Pro-L-Glu-D-Tyr-NH_2 · TFA (UTS-121):

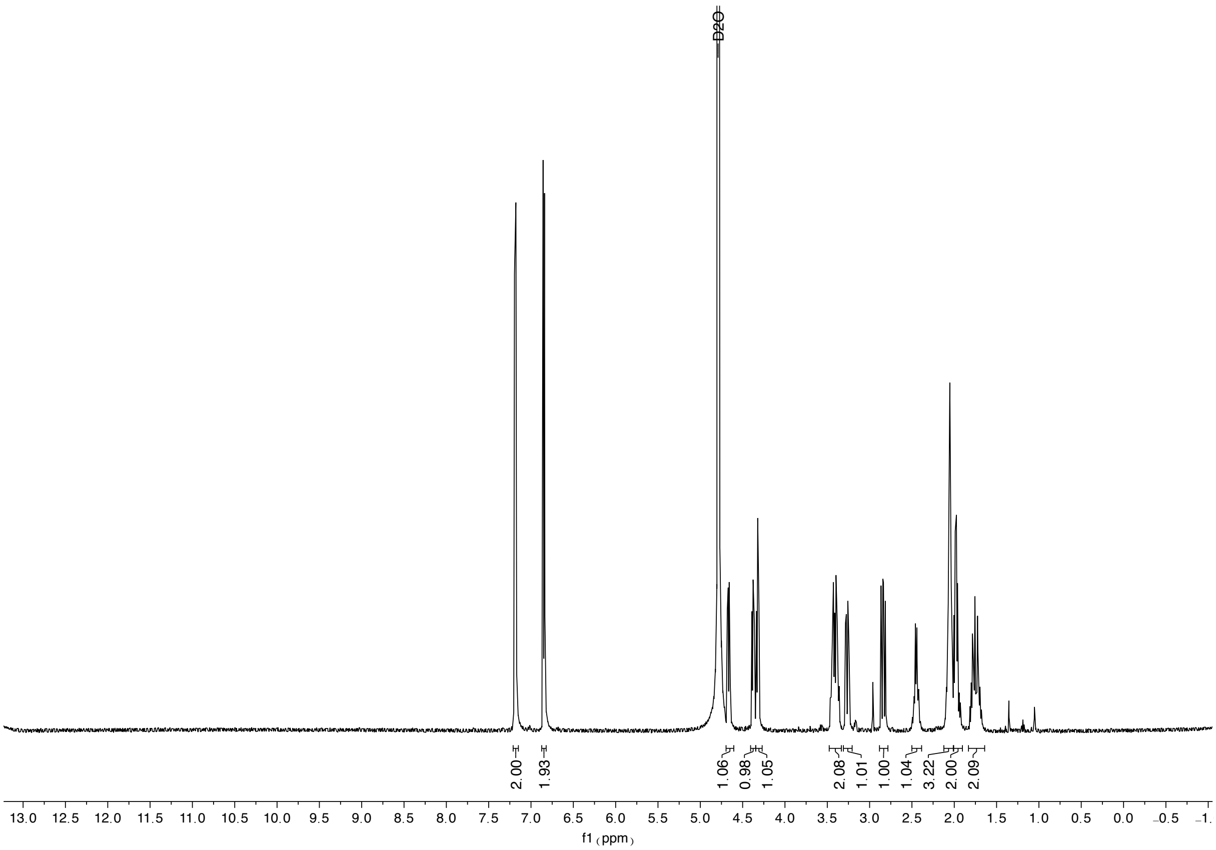

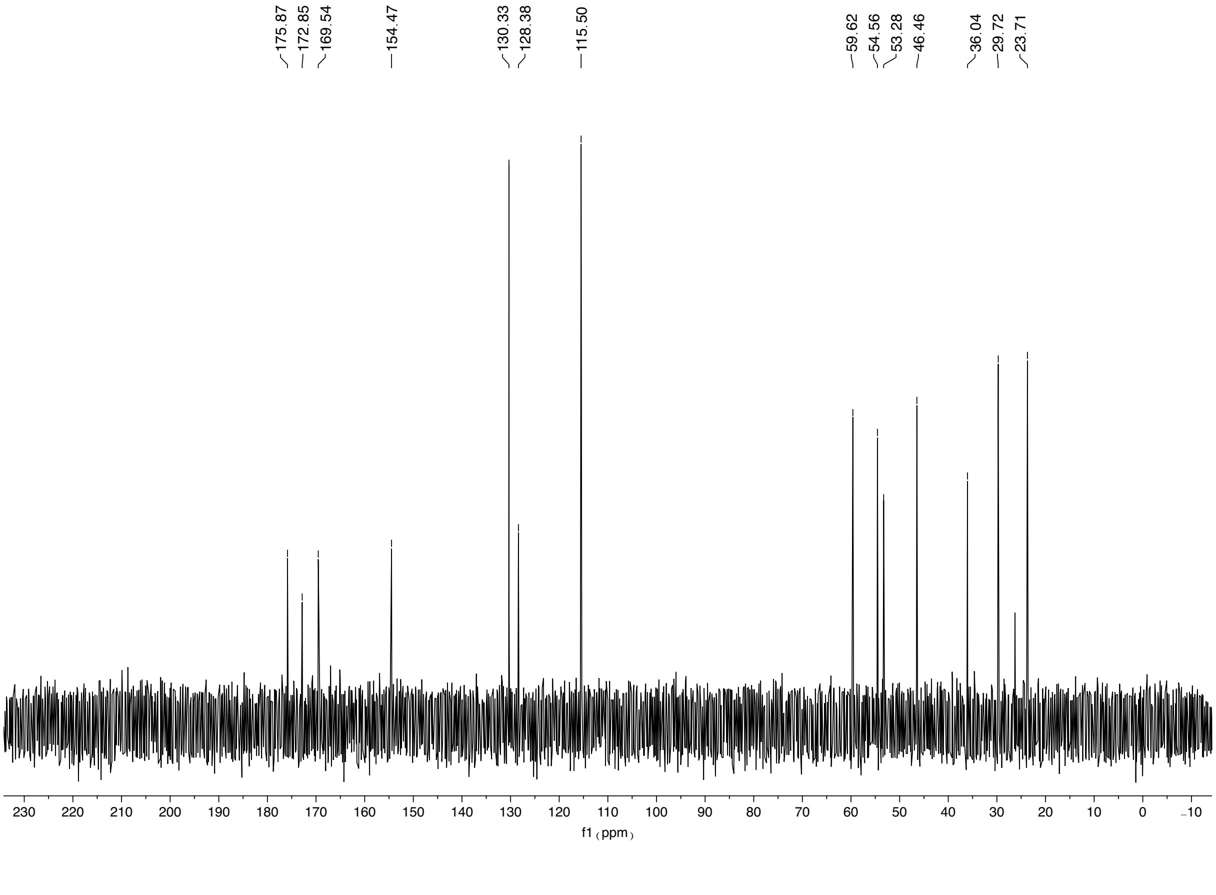

^{1}H and ^{13}C-NMR of H-D-Pro-L-Glu-CyLeu-NH$_2$ · TFA (UTS-122):

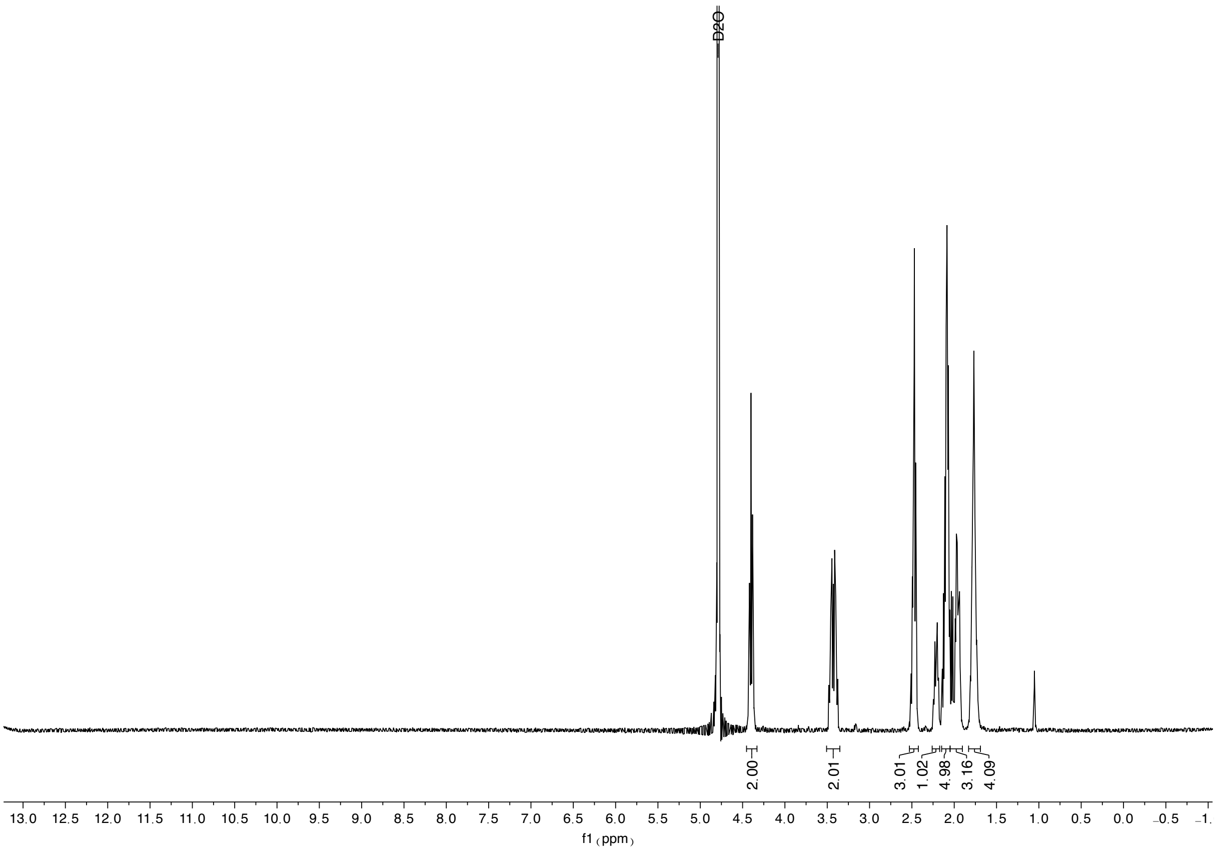

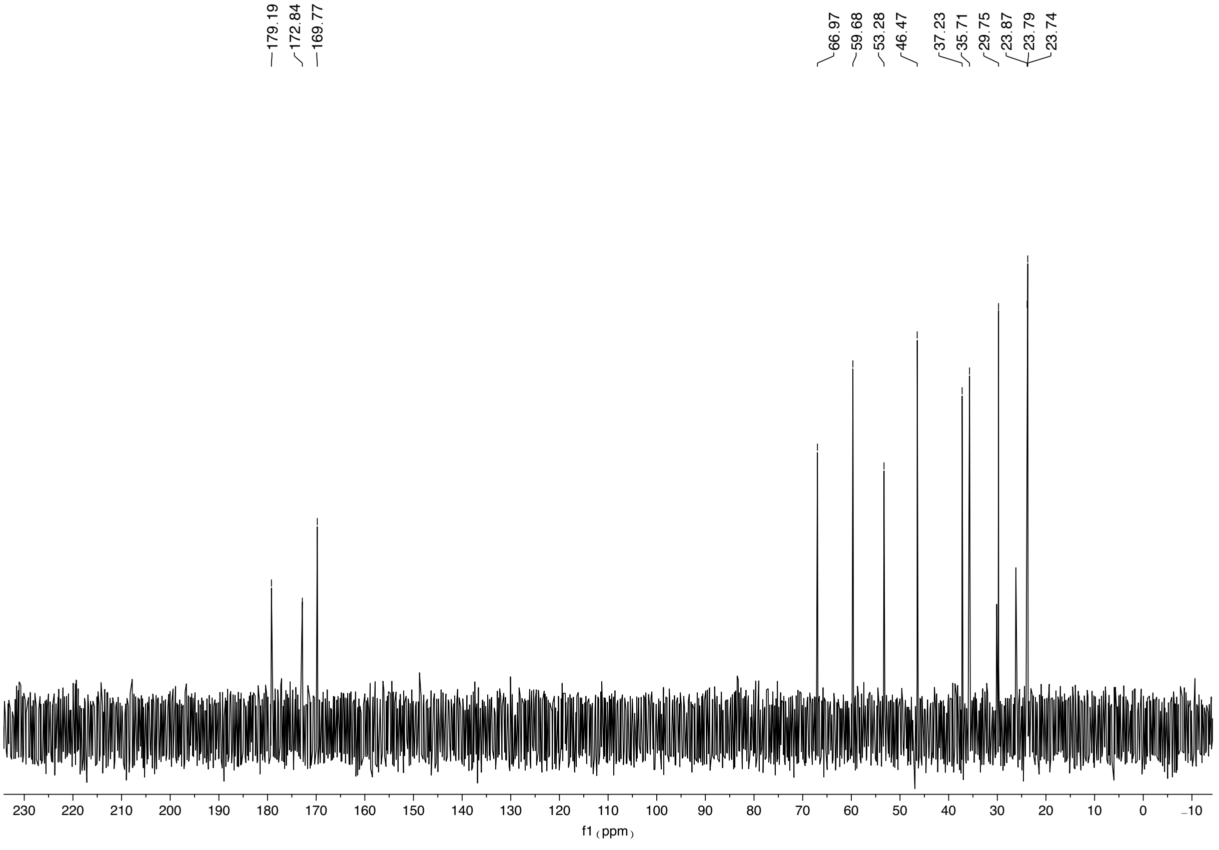

^{1}H and ^{13}C-NMR of H-D-Pro-L-Glu-Abz-NH$_2$ · TFA (UTS-123):

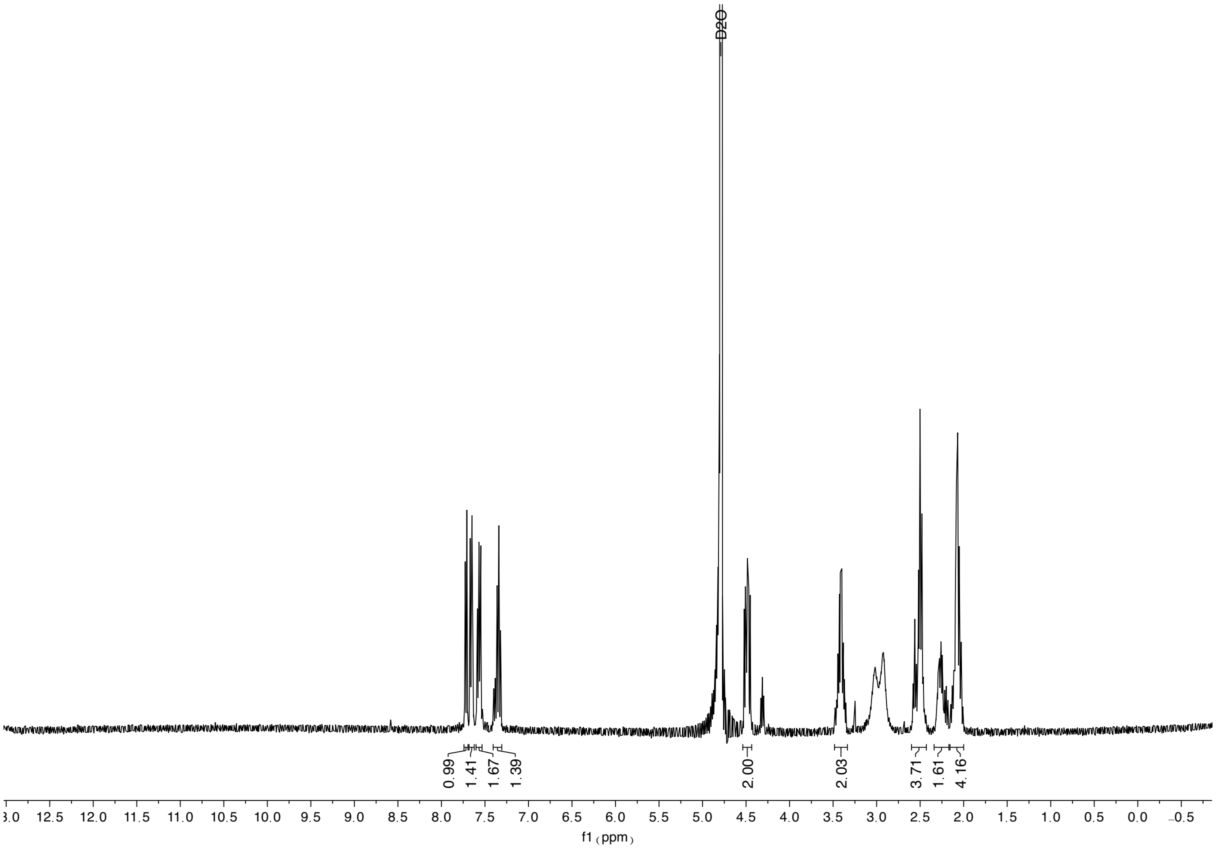

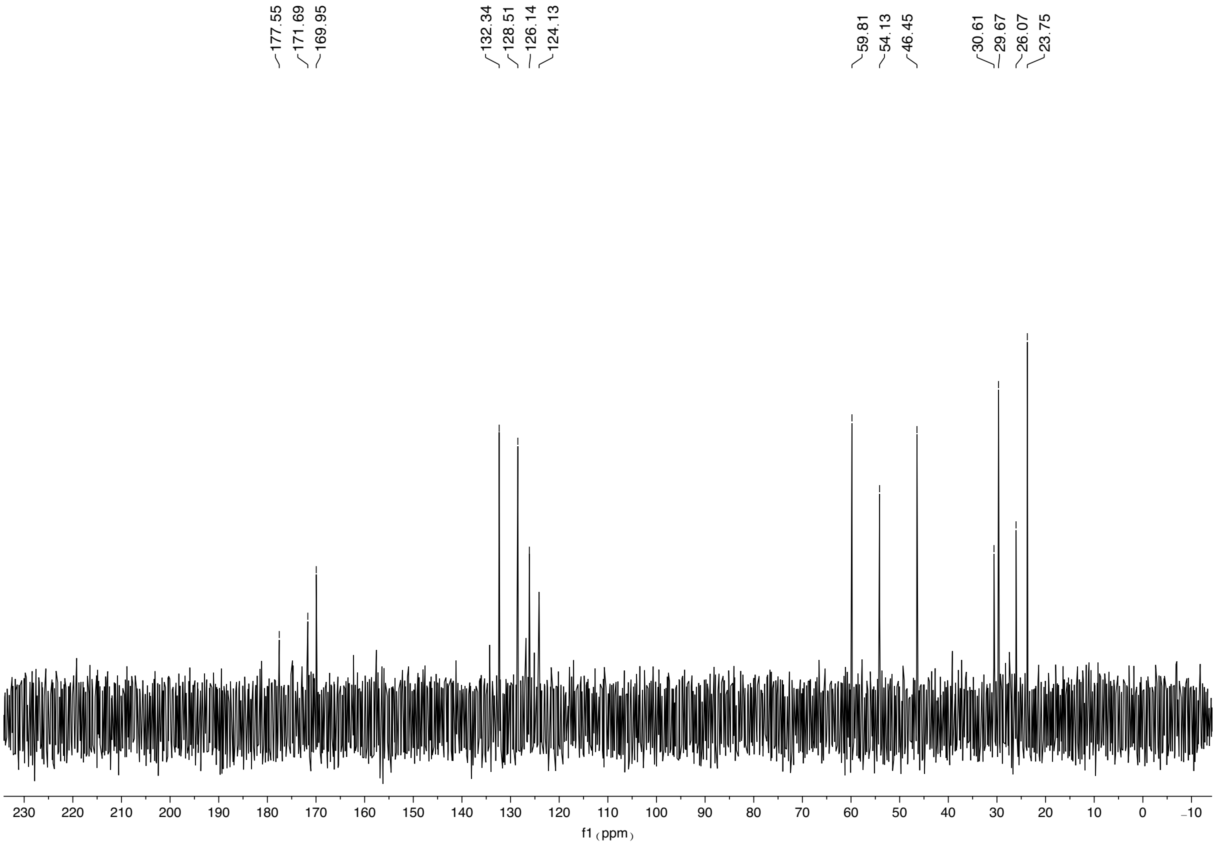

^{1}H and ^{13}C-NMR of H-D-Pro-L-Glu-L-Ind-NH_2 · TFA (UTS-124):

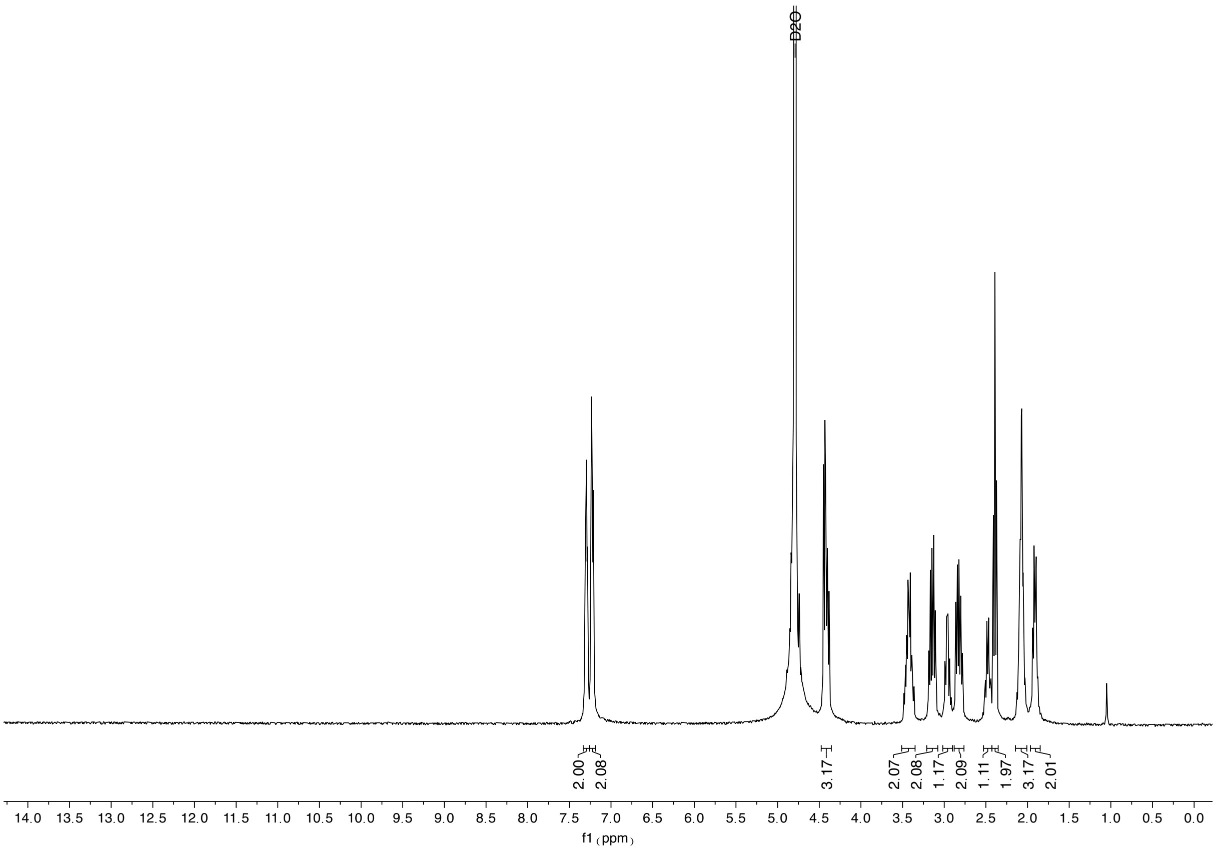

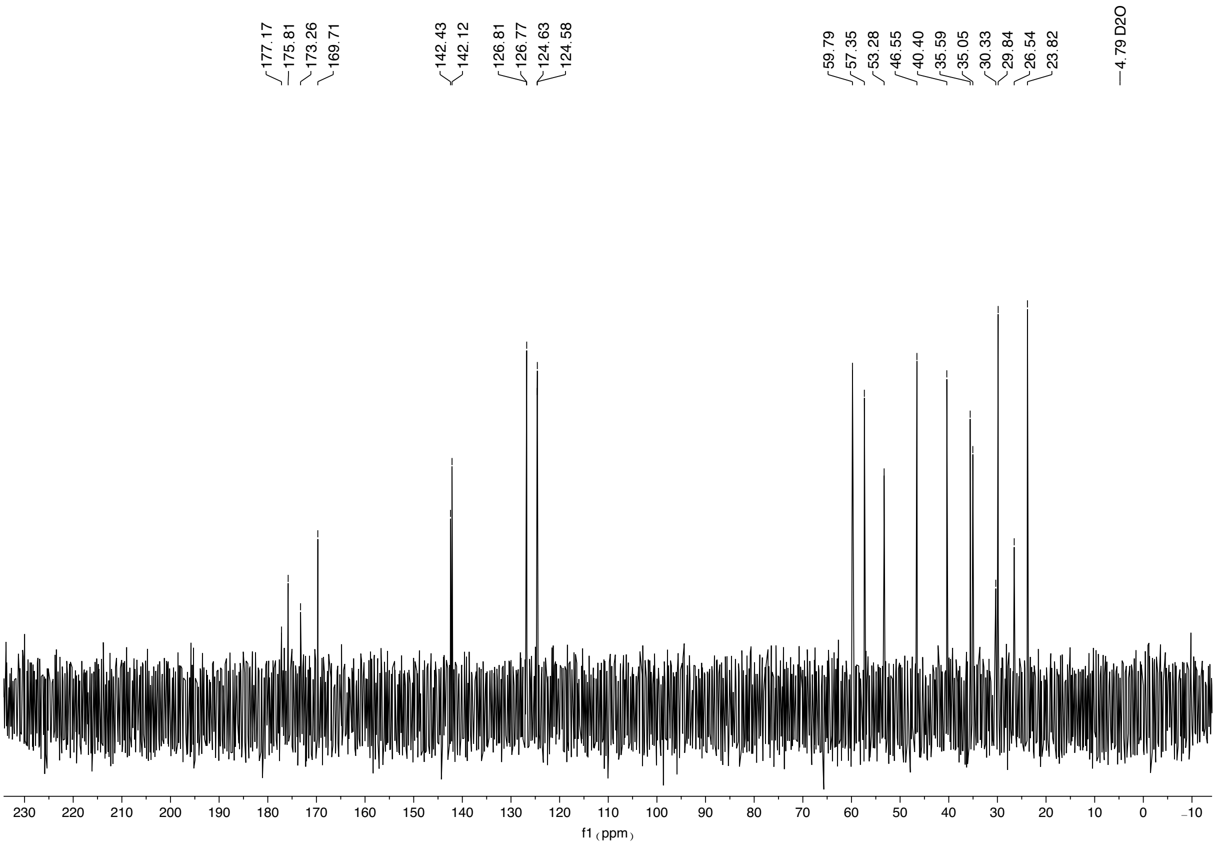

^{1}H and ^{13}C-NMR of H-D-Pro-L-Glu-L-Ind-NH_2 · TFA (UTS-124):

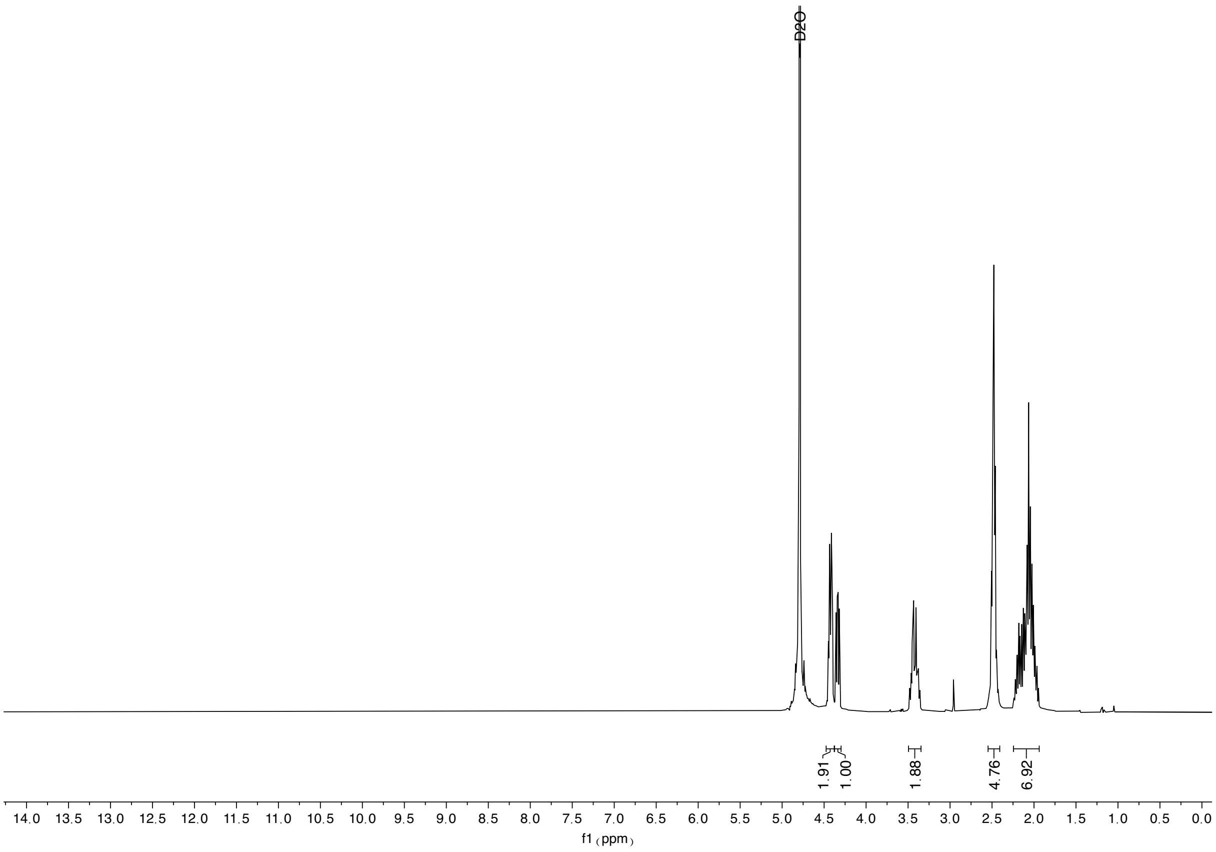

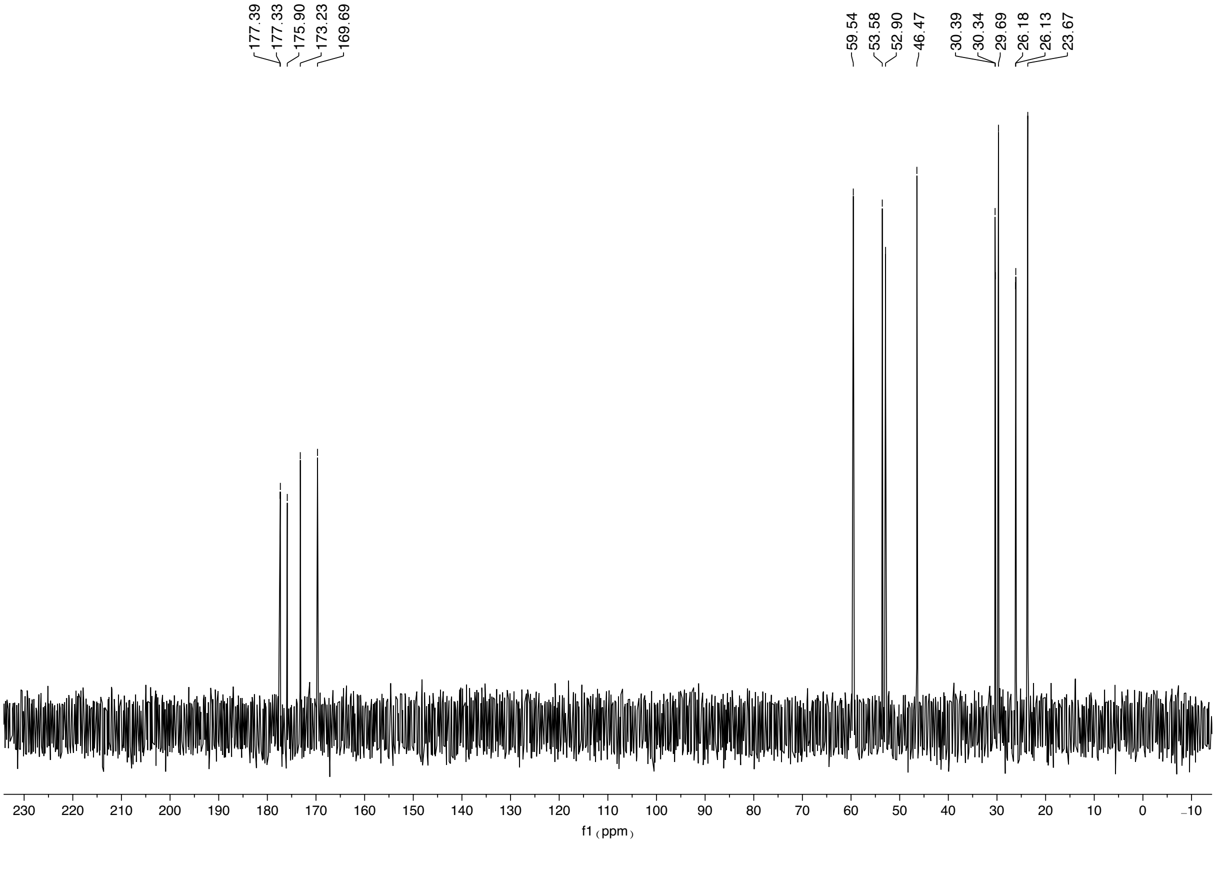

^{1}H and ^{13}C-NMR of H-D-Pro-L-Tyr-L-Glu-NH$_2$ · TFA (UTS-126):

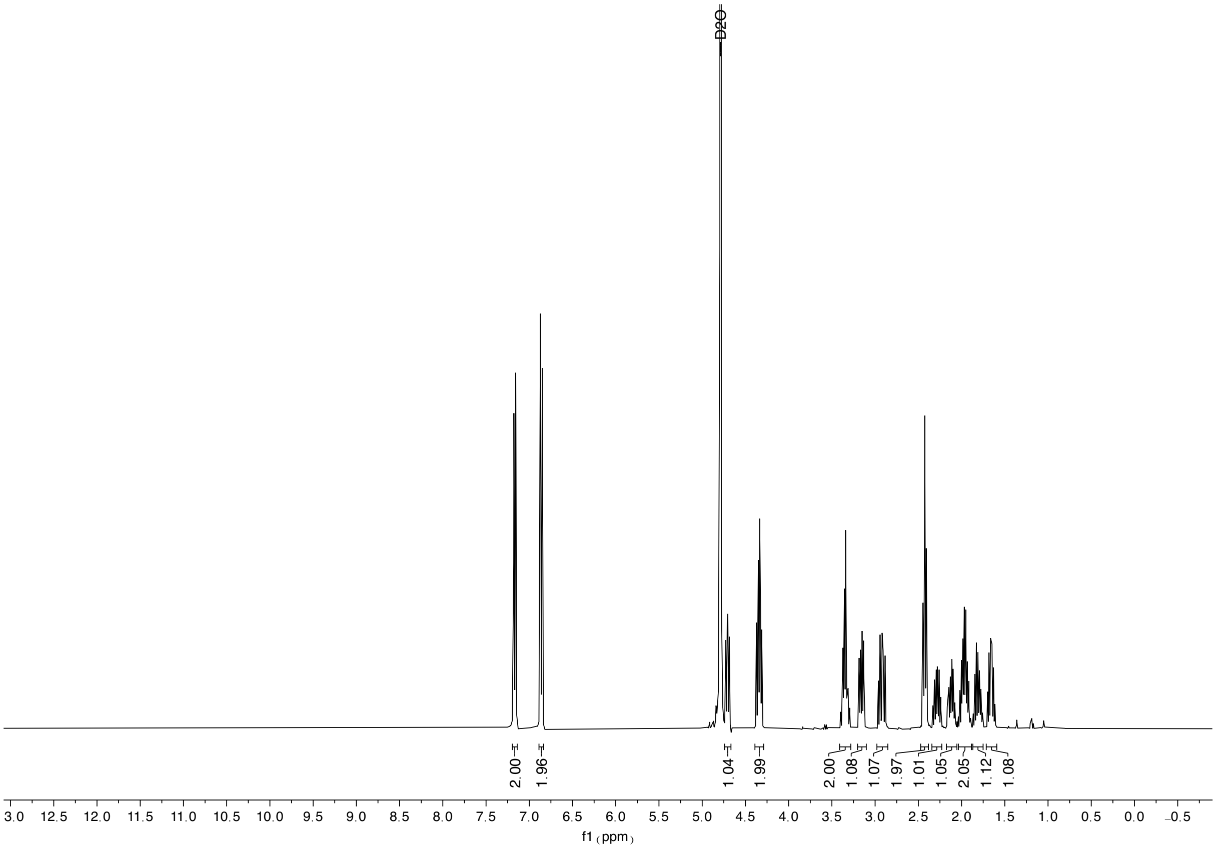

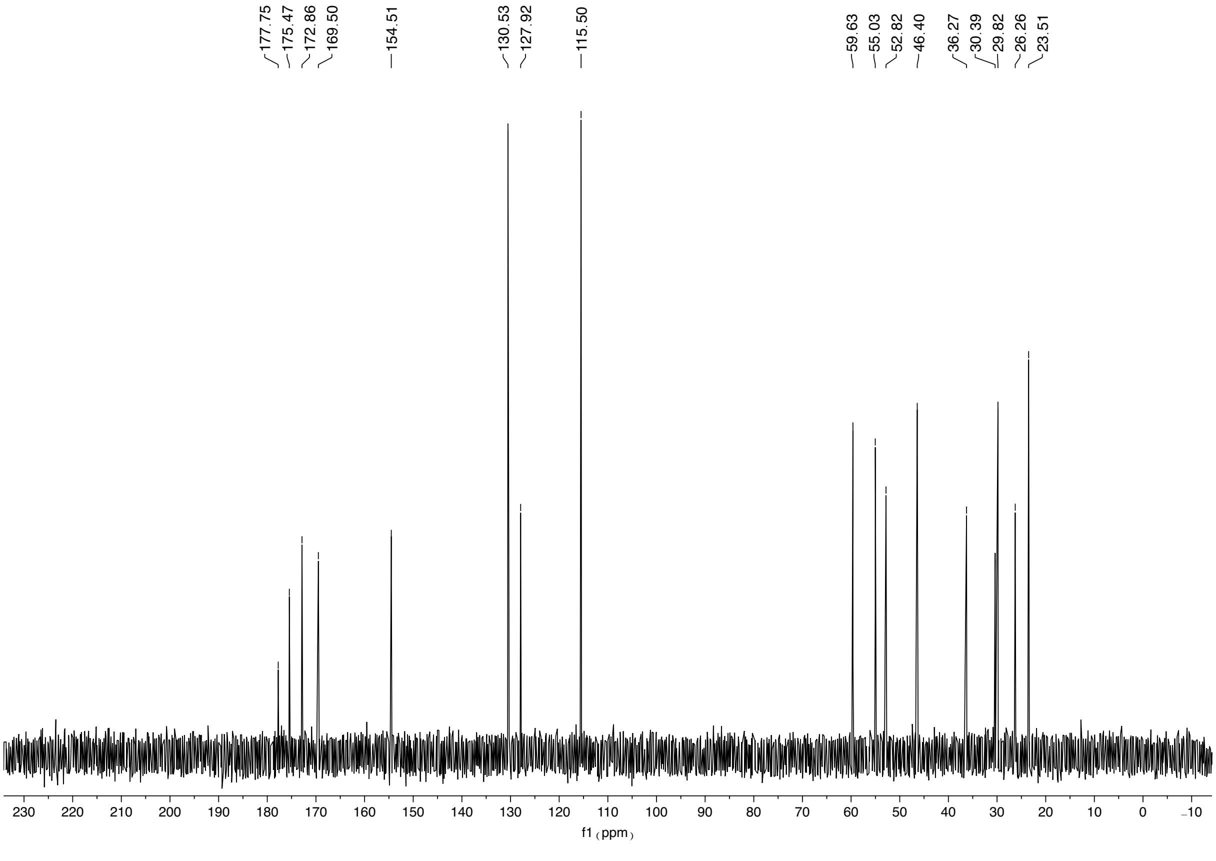

^{1}H and ^{13}C-NMR of H-D-Pro-D-Tyr-L-Glu-NH$_2$ · TFA (UTS-127):

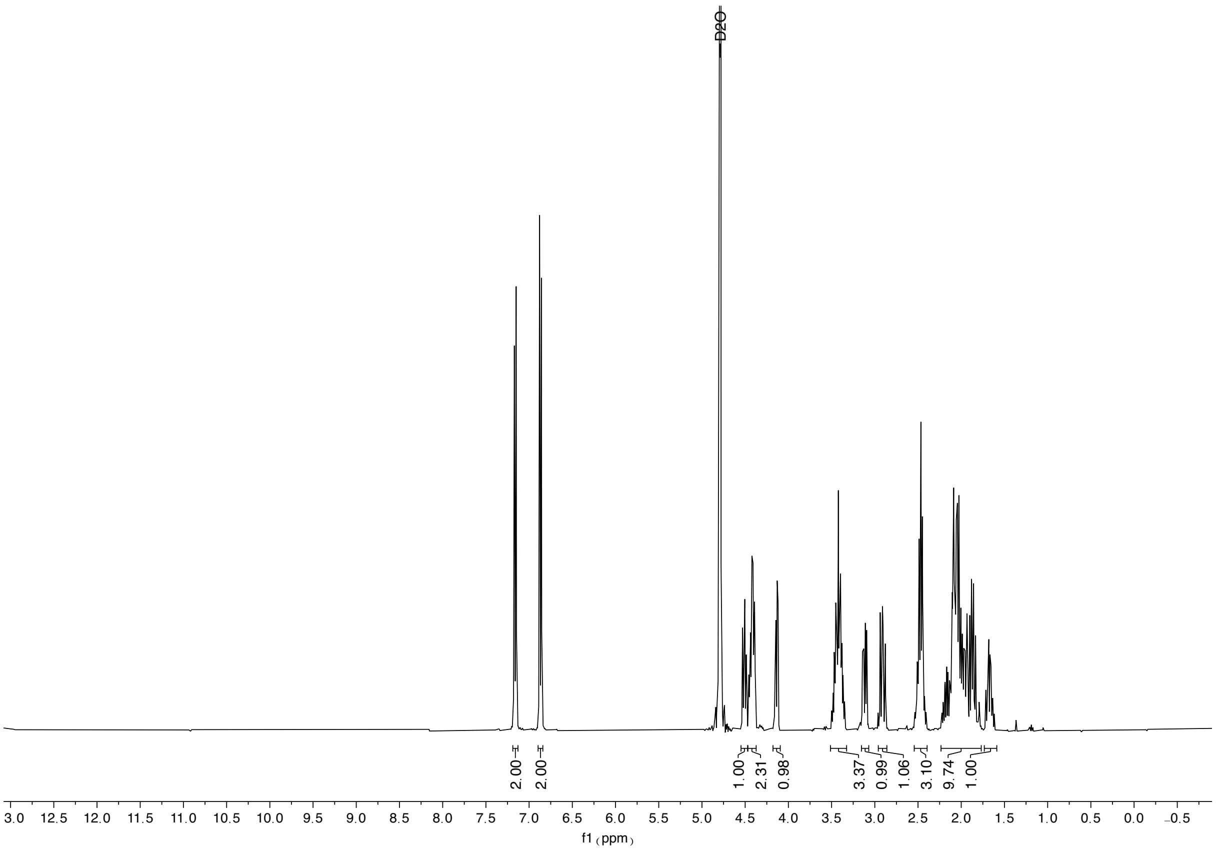

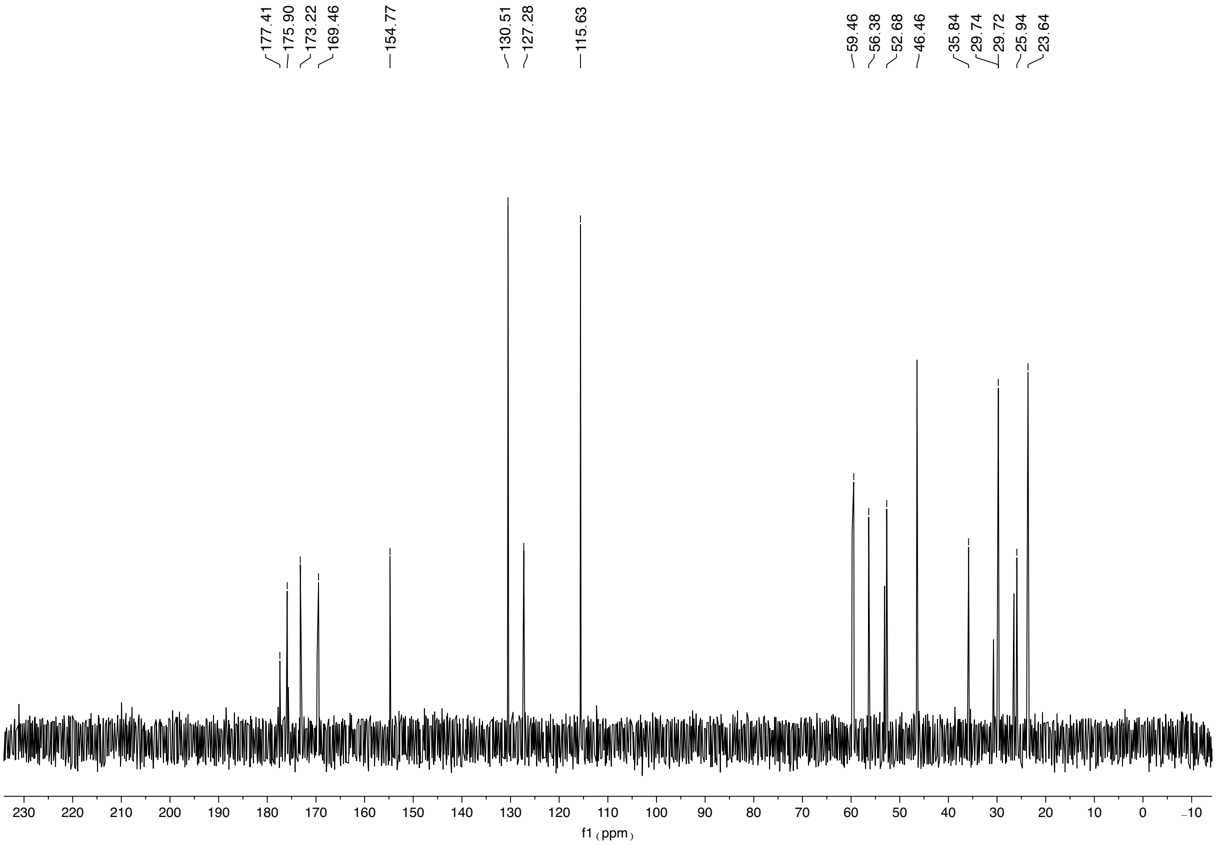

^{1}H and ^{13}C-NMR of H-D-Pro-CyLeu-L-Glu-NH$_2$ · TFA (UTS-128):

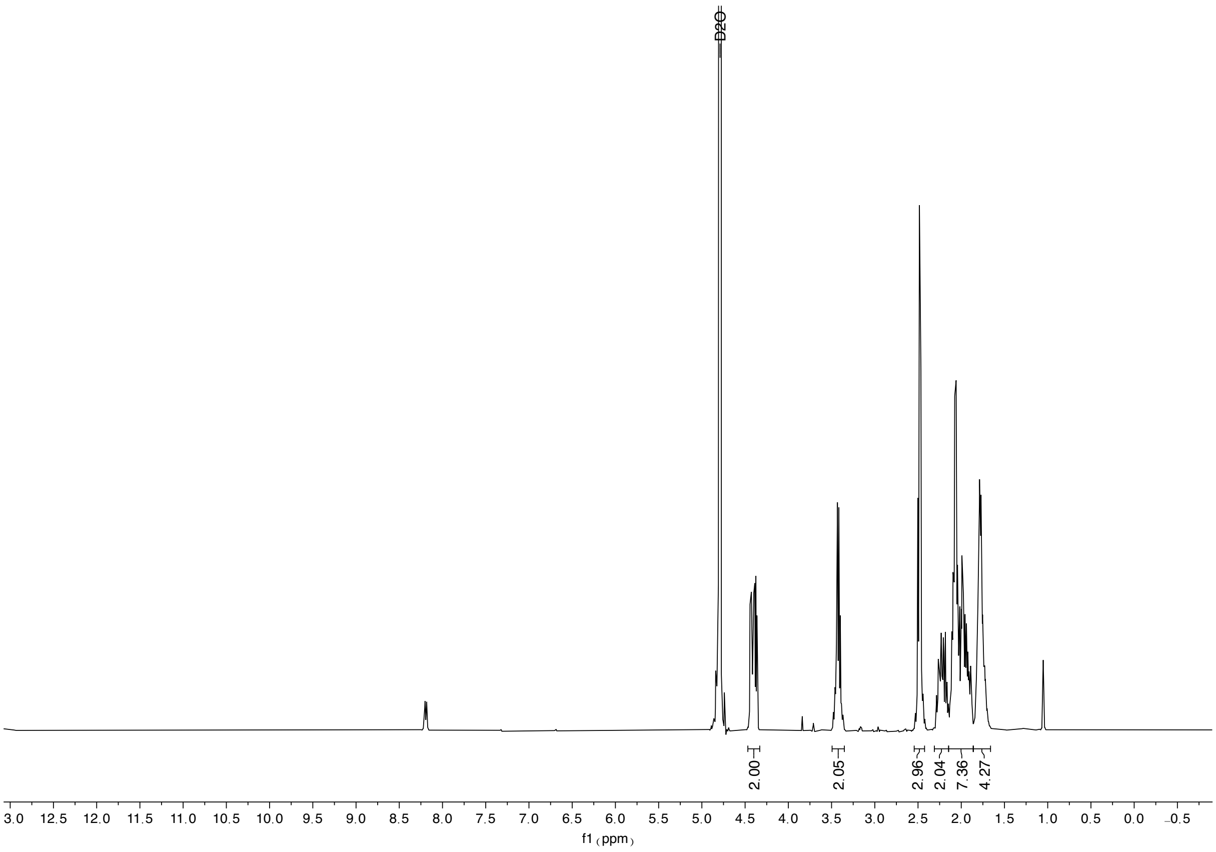

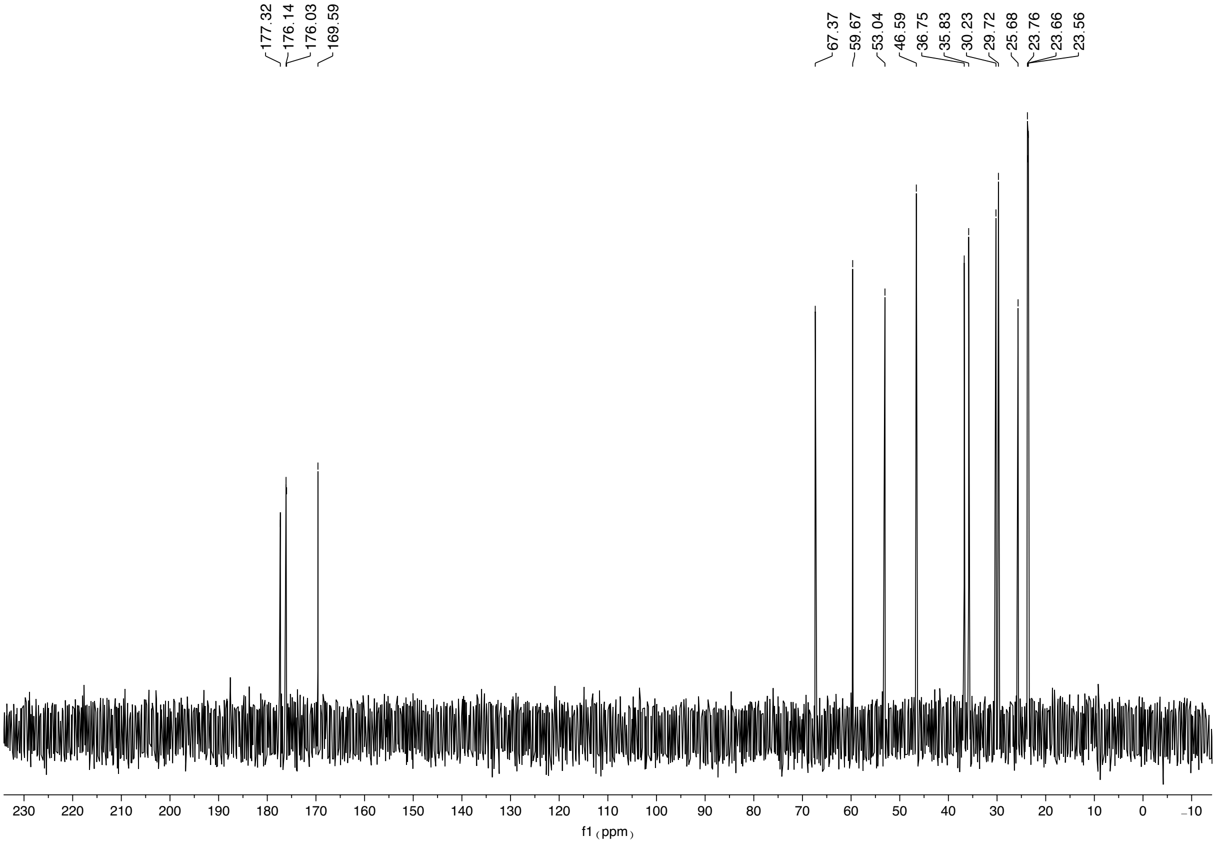

^{1}H and ^{13}C-NMR of H-D-Pro-Abz-L-Glu-NH_2 · TFA (UTS-129):

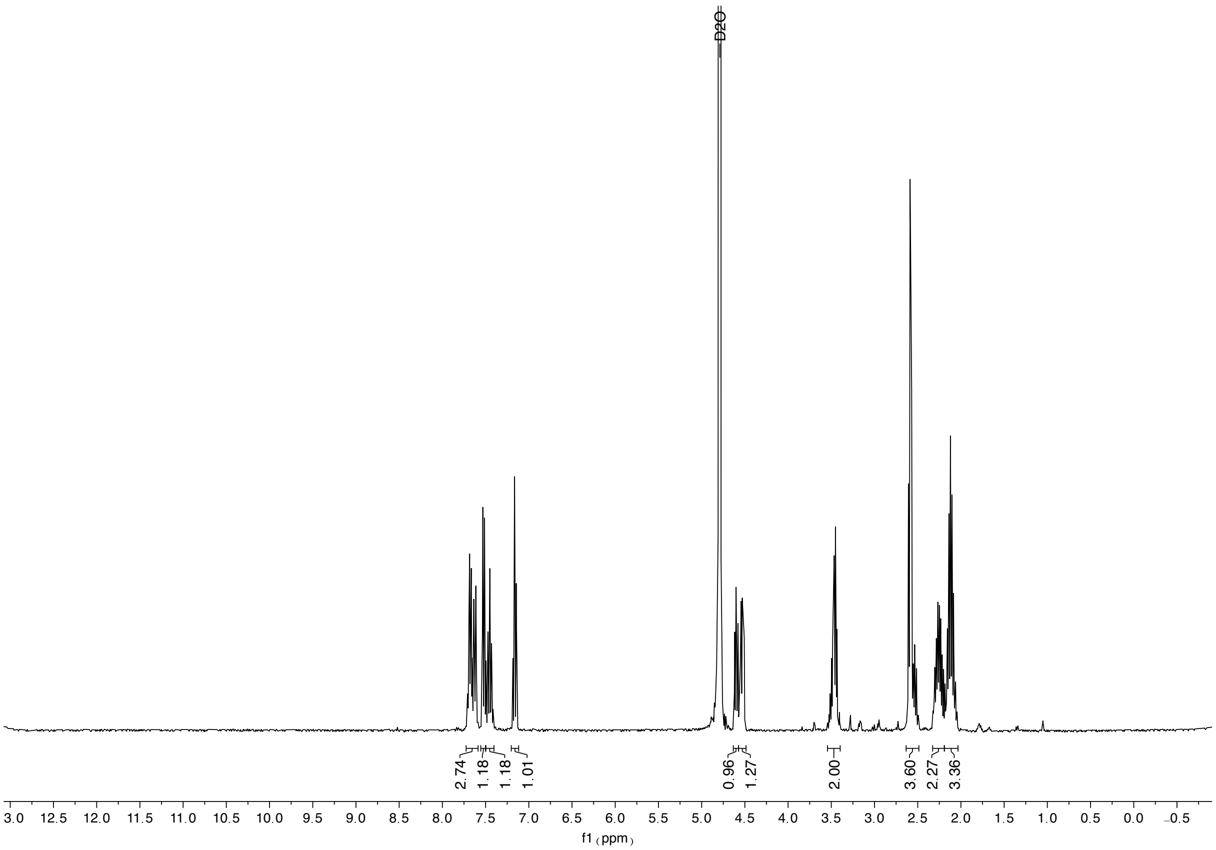

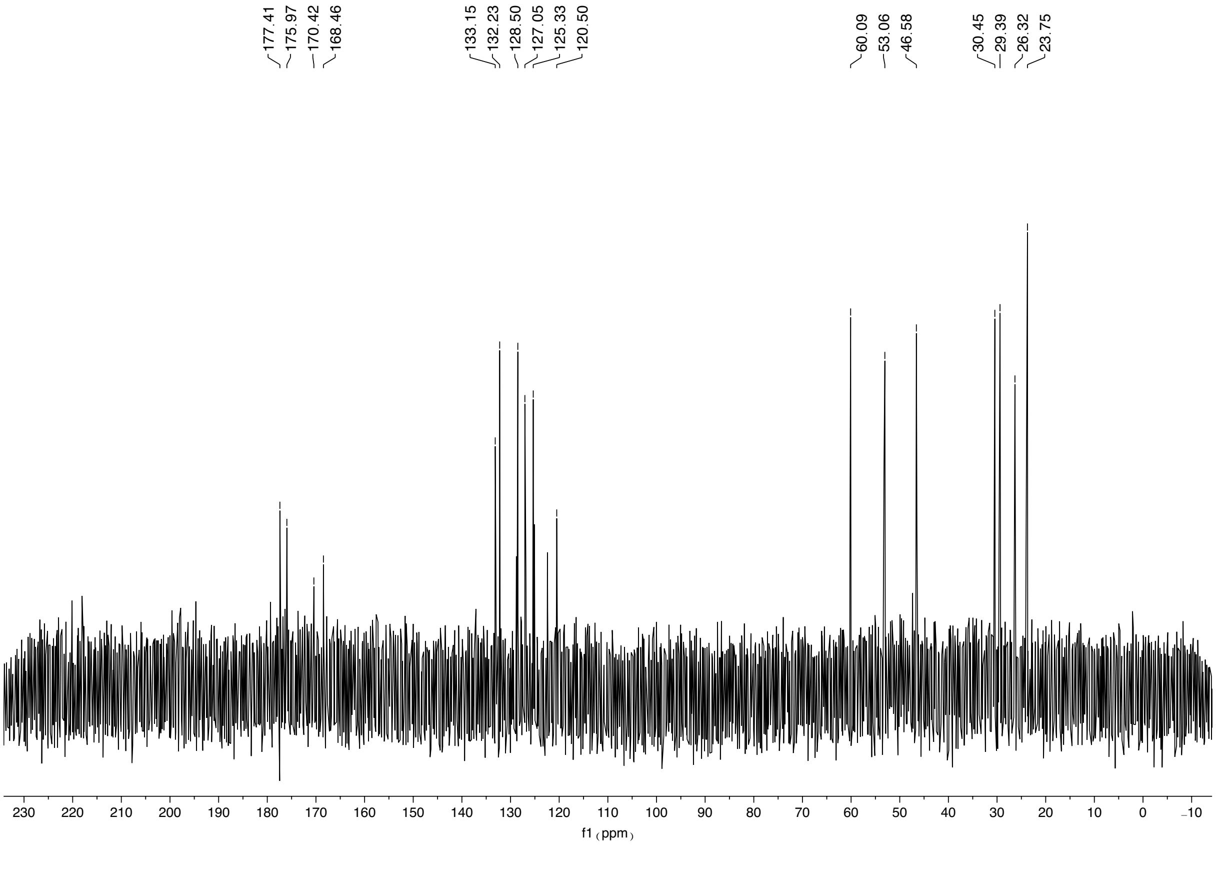

^{1}H and ^{13}C-NMR of H-D-Pro-D-Ind-L-Glu-NH$_2$ · TFA (UTS-130):

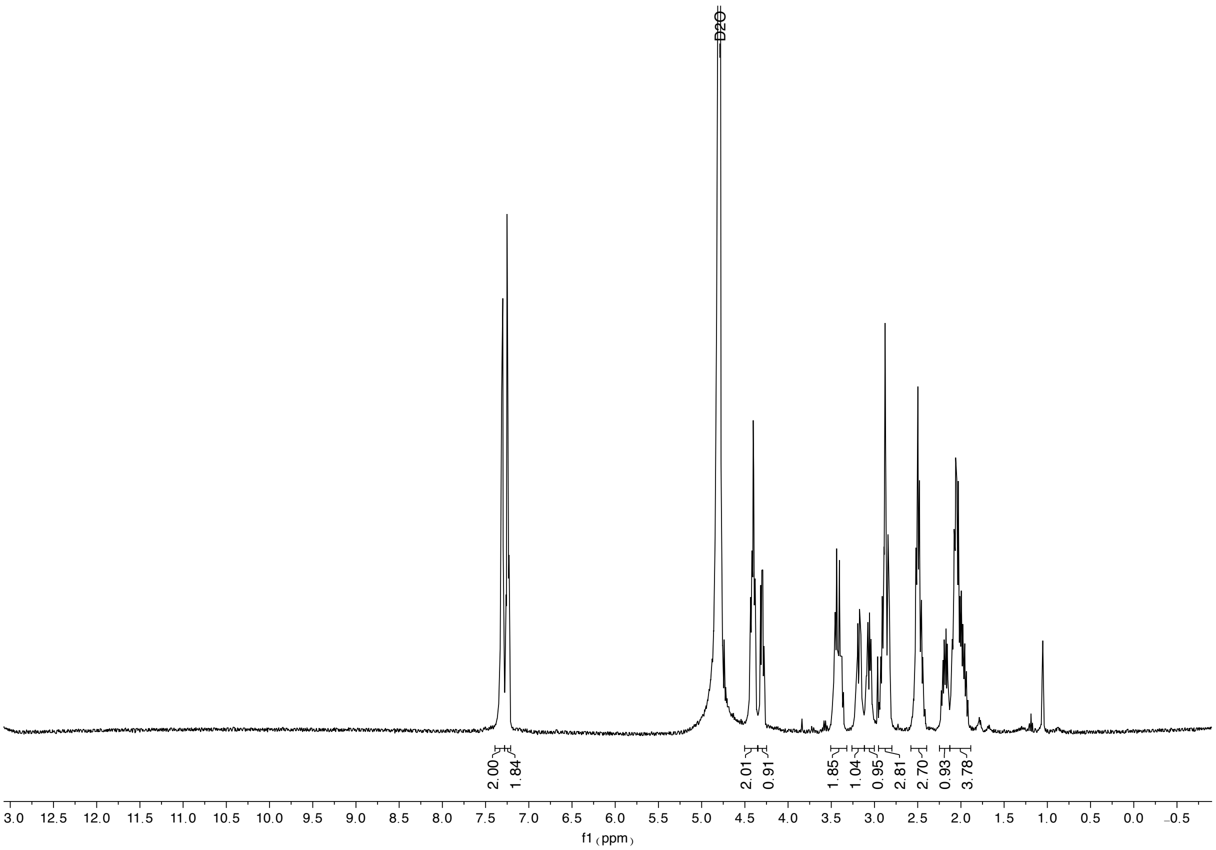

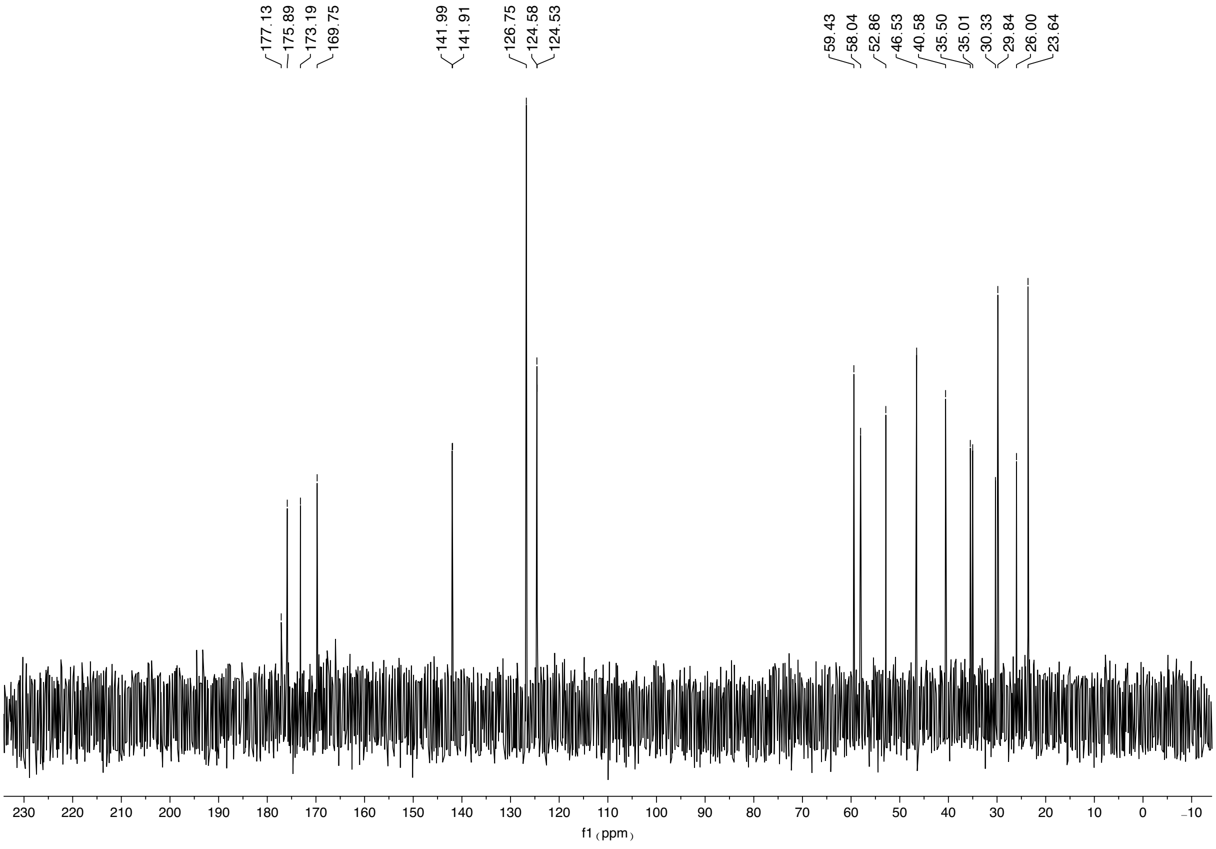

^{1}H and ^{13}C-NMR of H-D-Pro-D-Glu-D-Glu-NH$_2$ · TFA (UTS-131):

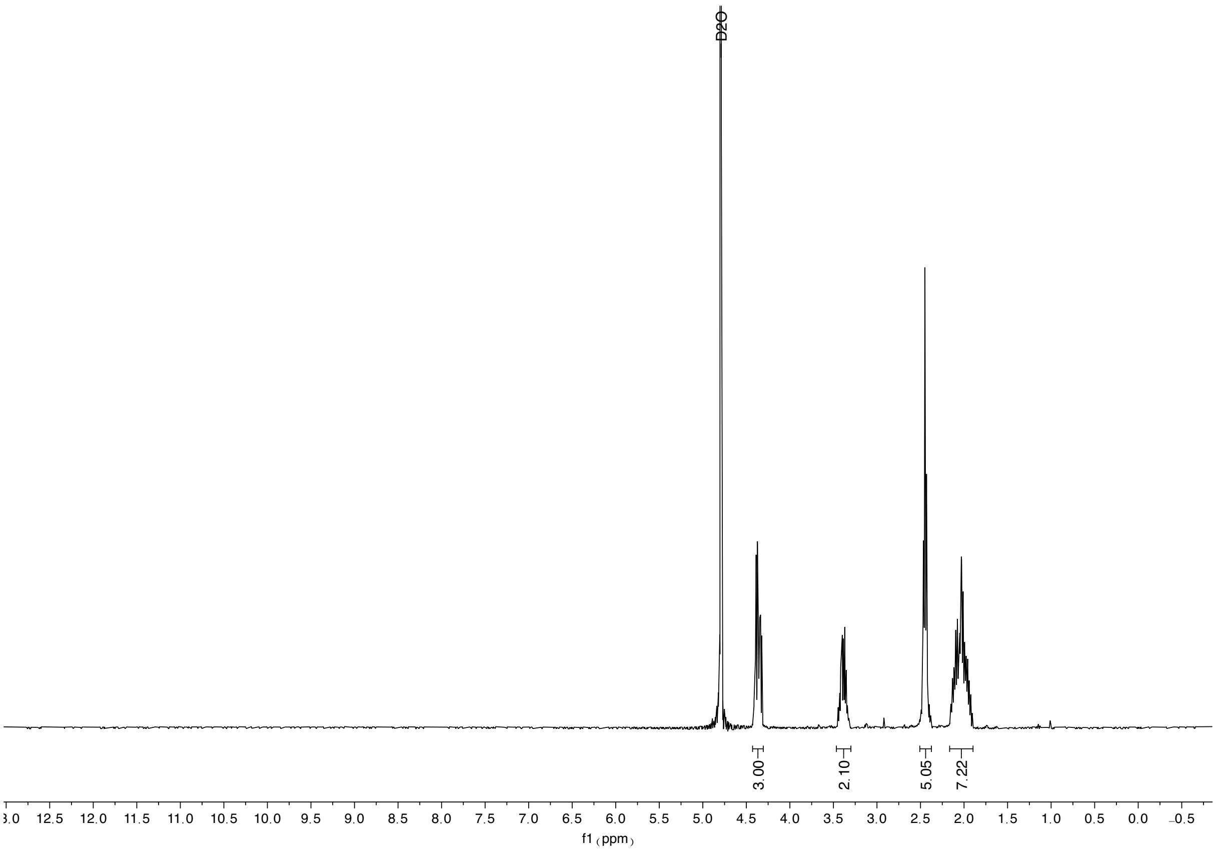

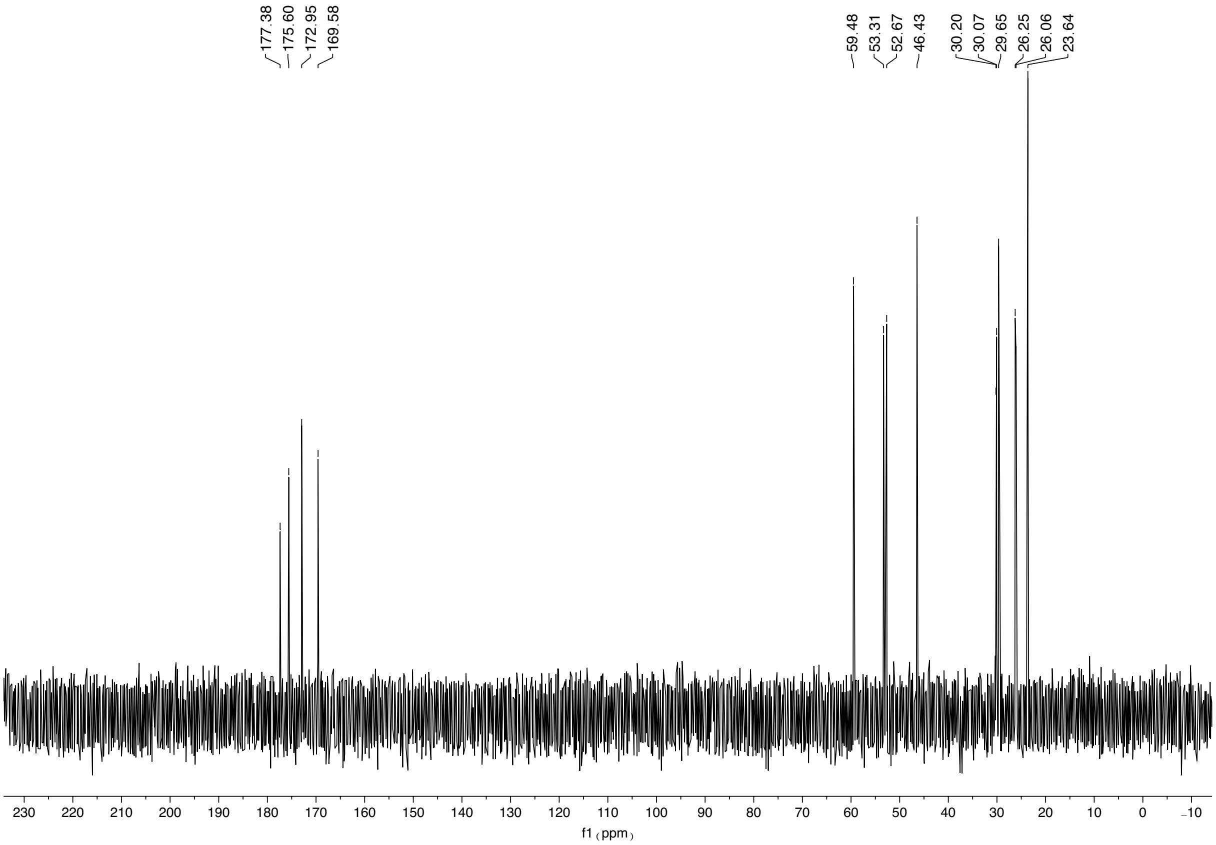

^{1}H and ^{13}C-NMR of H-D-Pro-D-Glu-L-Tyr-NH$_2$ · TFA (UTS-132):

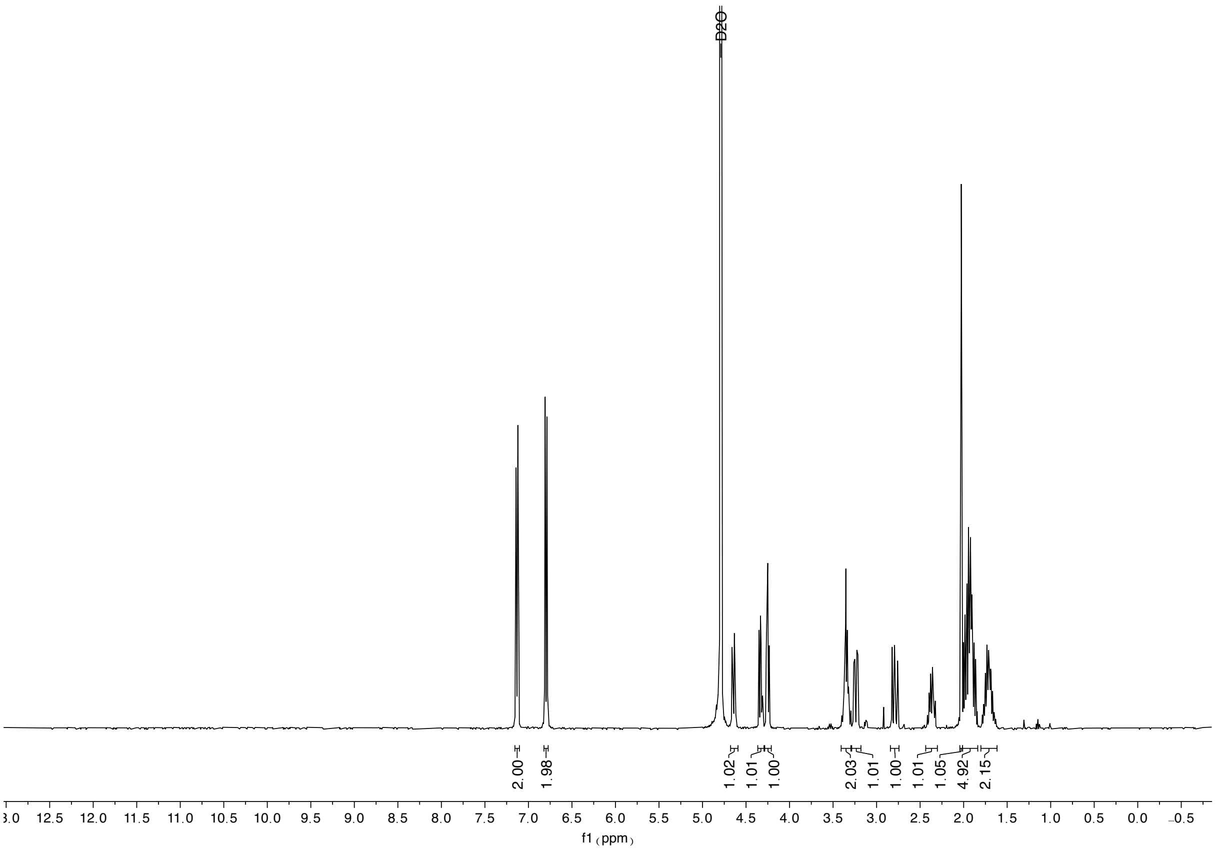

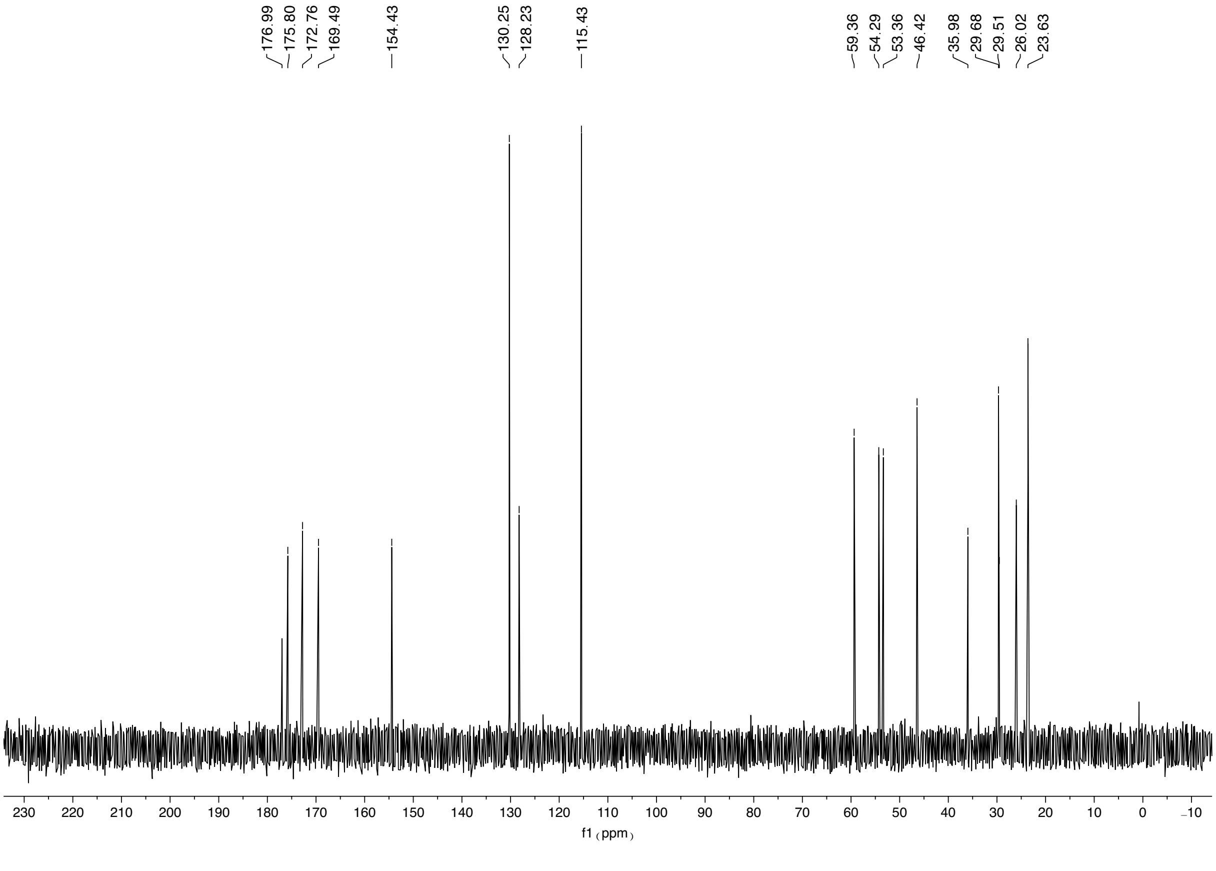

^{1}H and ^{13}C-NMR of H-D-Pro-D-Glu-D-Tyr-NH_2 · TFA (UTS-133):

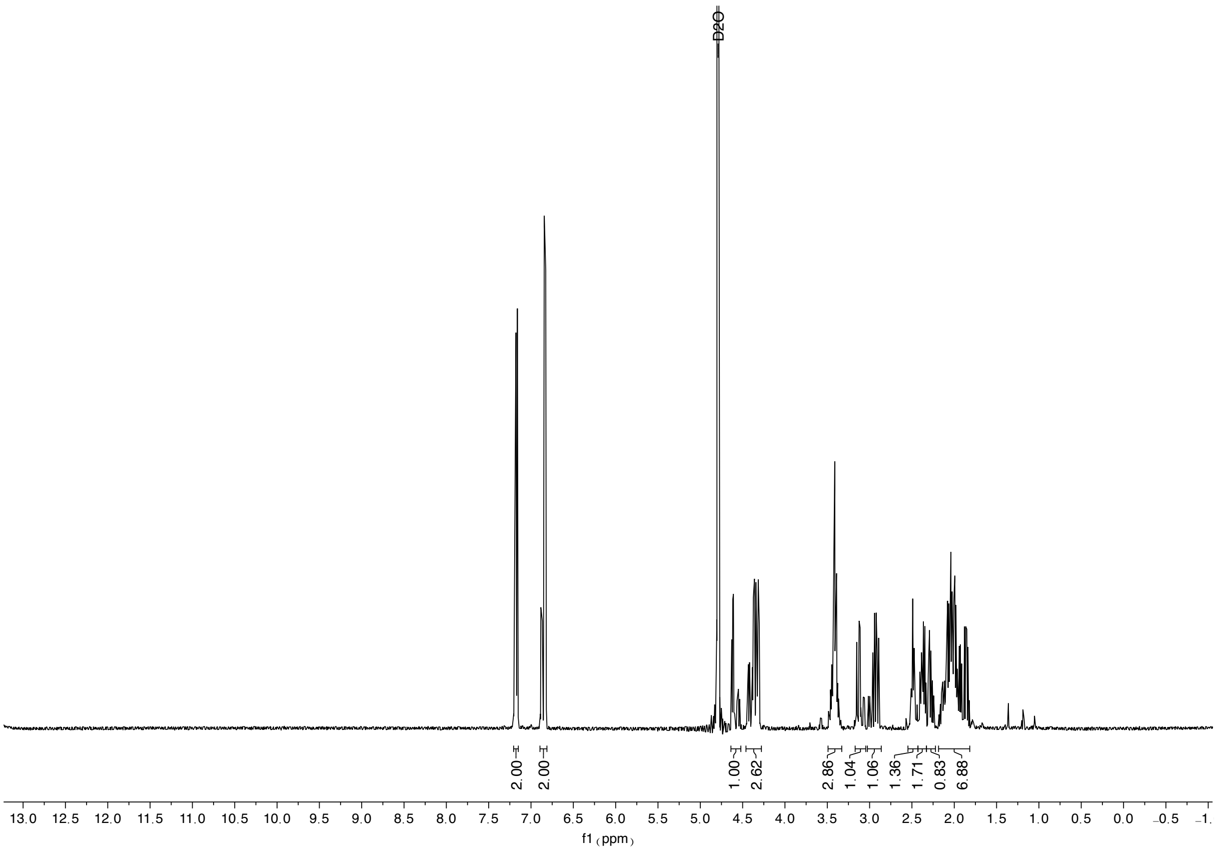

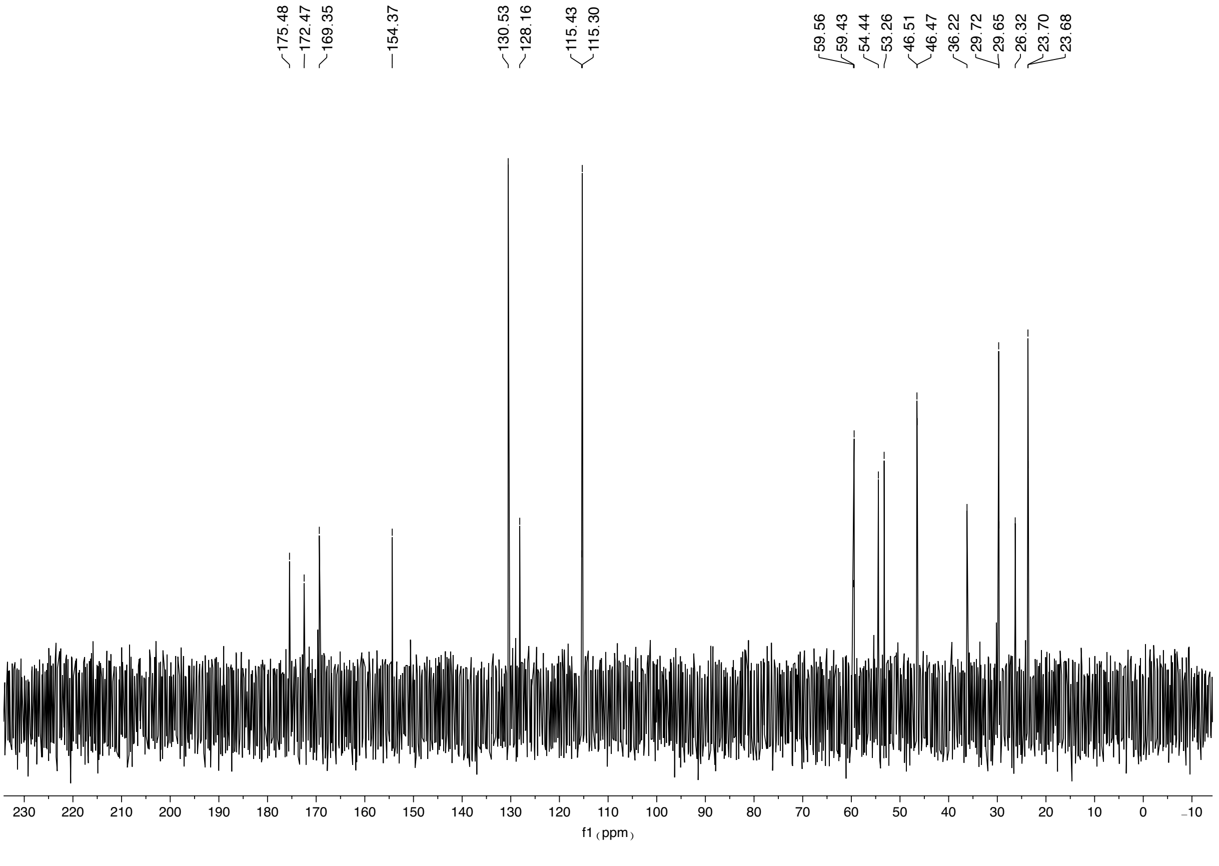

^{1}H and ^{13}C-NMR of H-D-Pro-D-Glu-CyLeu-NH$_2$ · TFA (UTS-134):

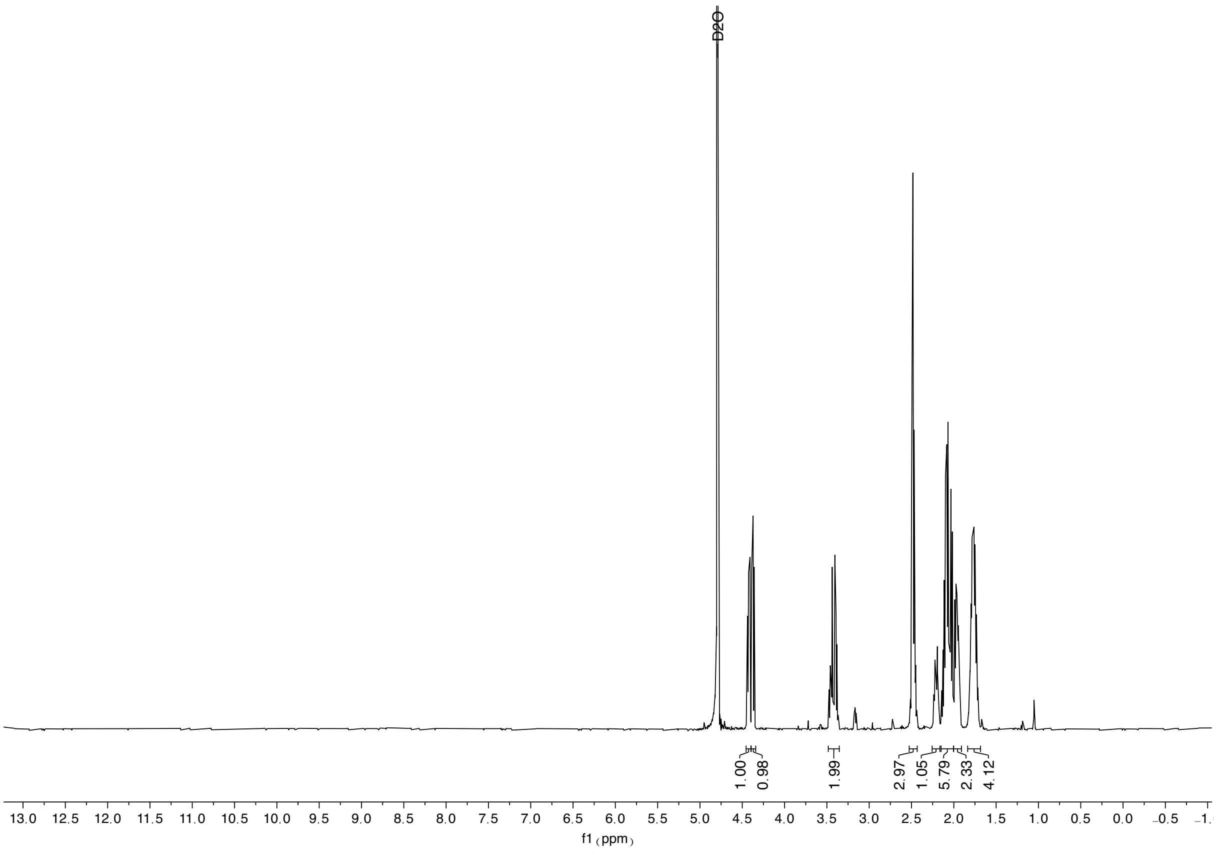

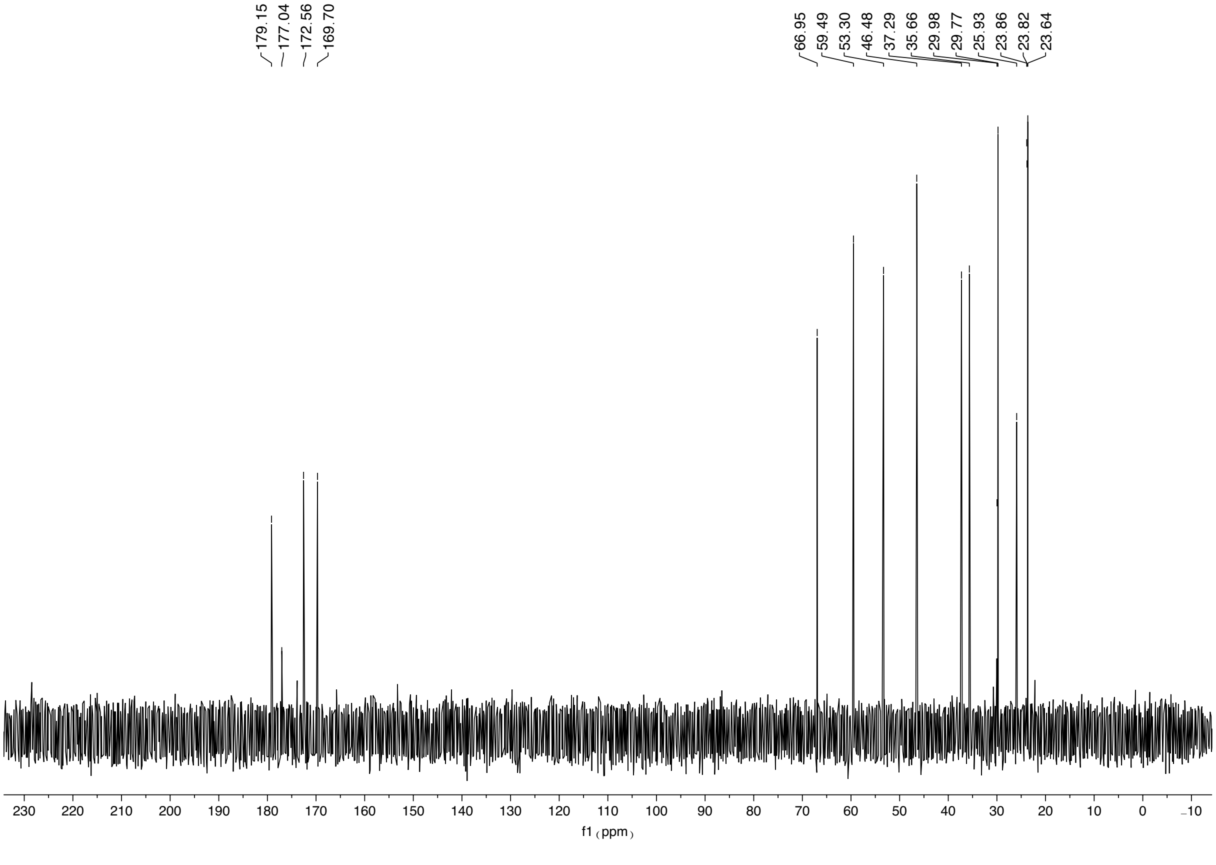

^{1}H and ^{13}C-NMR of H-D-Pro-D-Glu-Abz-NH_2 · TFA (UTS-135):

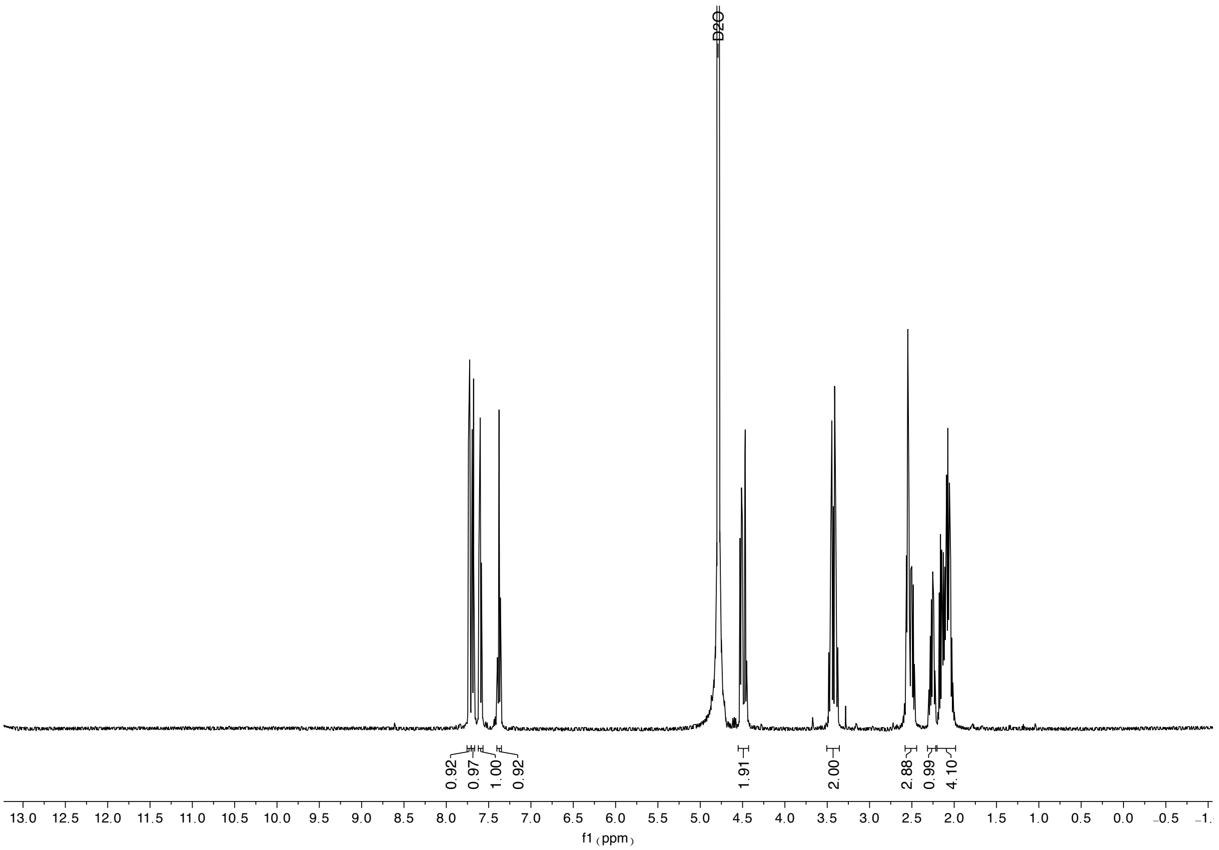

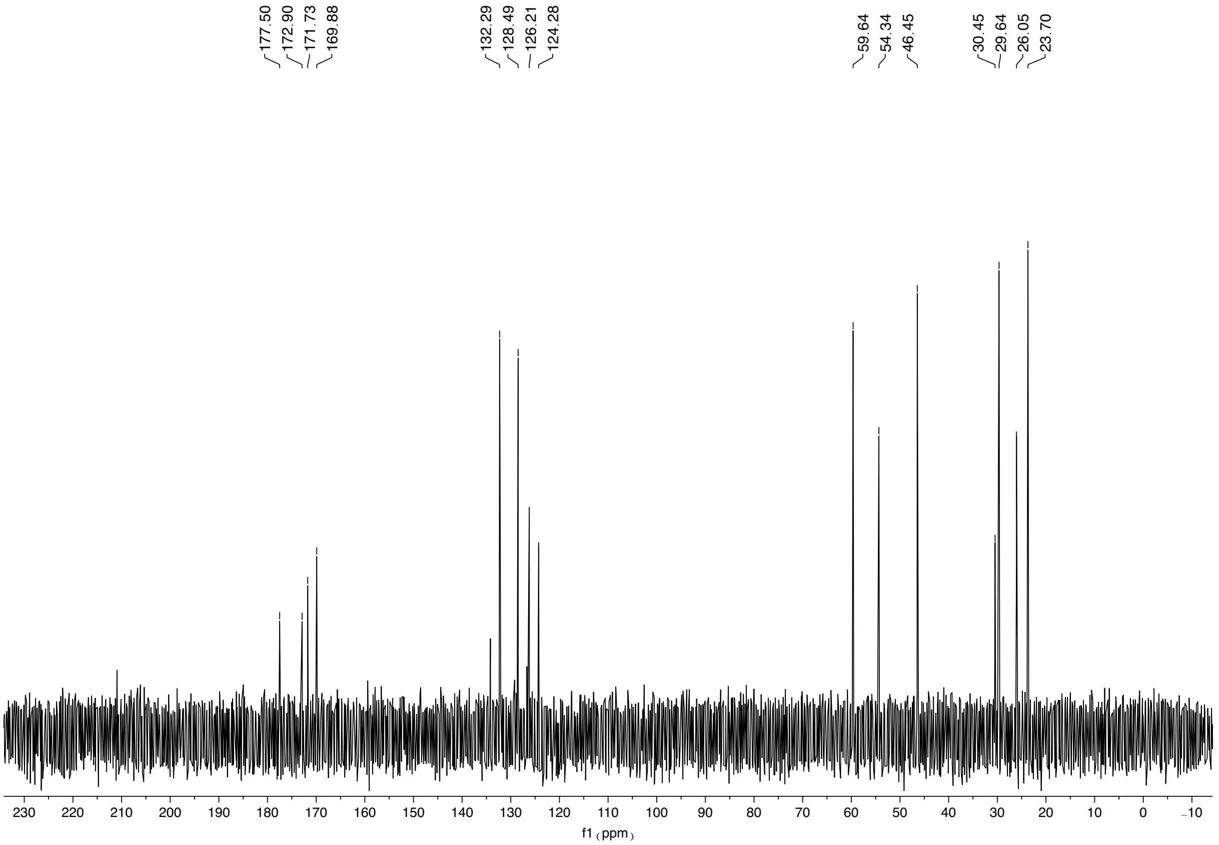

^{1}H and ^{13}C-NMR of H-D-Pro-D-Glu-D-Ind-NH$_2$ · TFA (UTS-136):

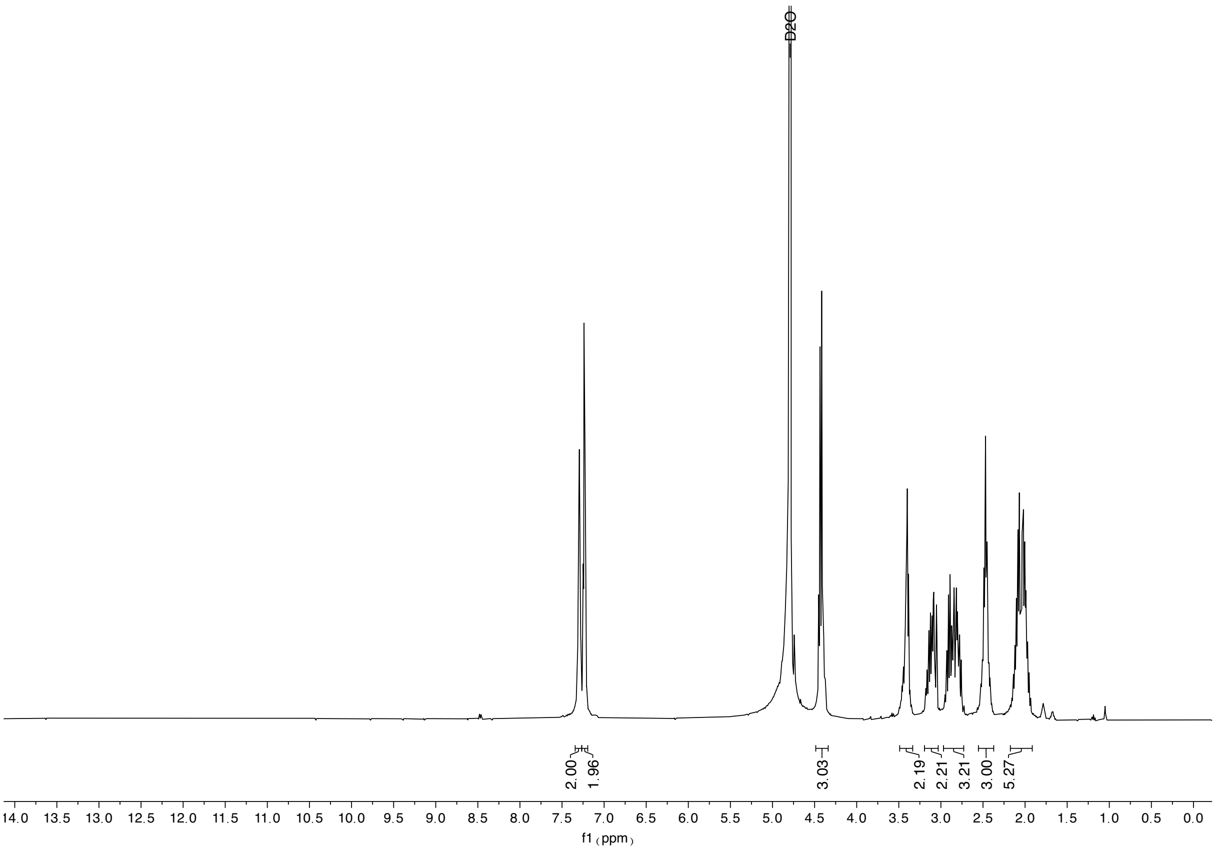

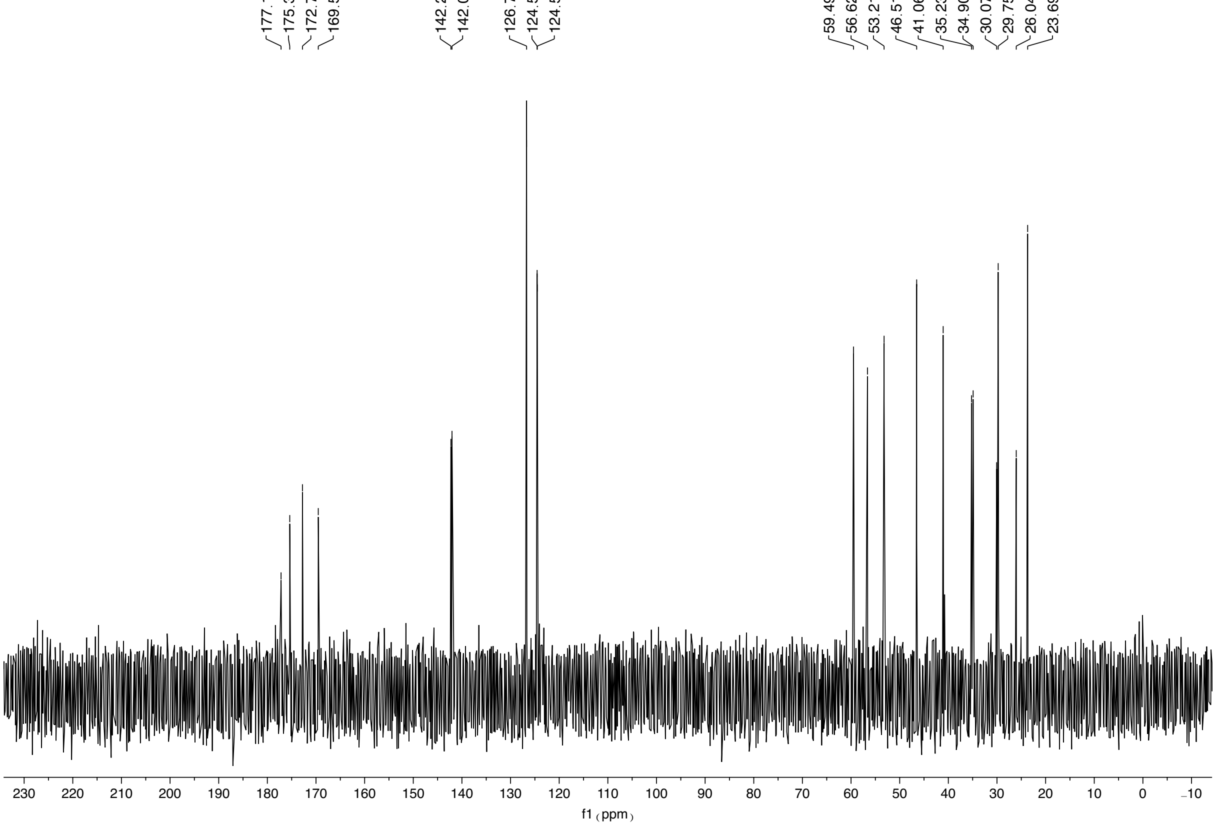

^{1}H and ^{13}C-NMR of H-D-Pro-L-Tyr-D-Glu-NH_2 · TFA (UTS-137):

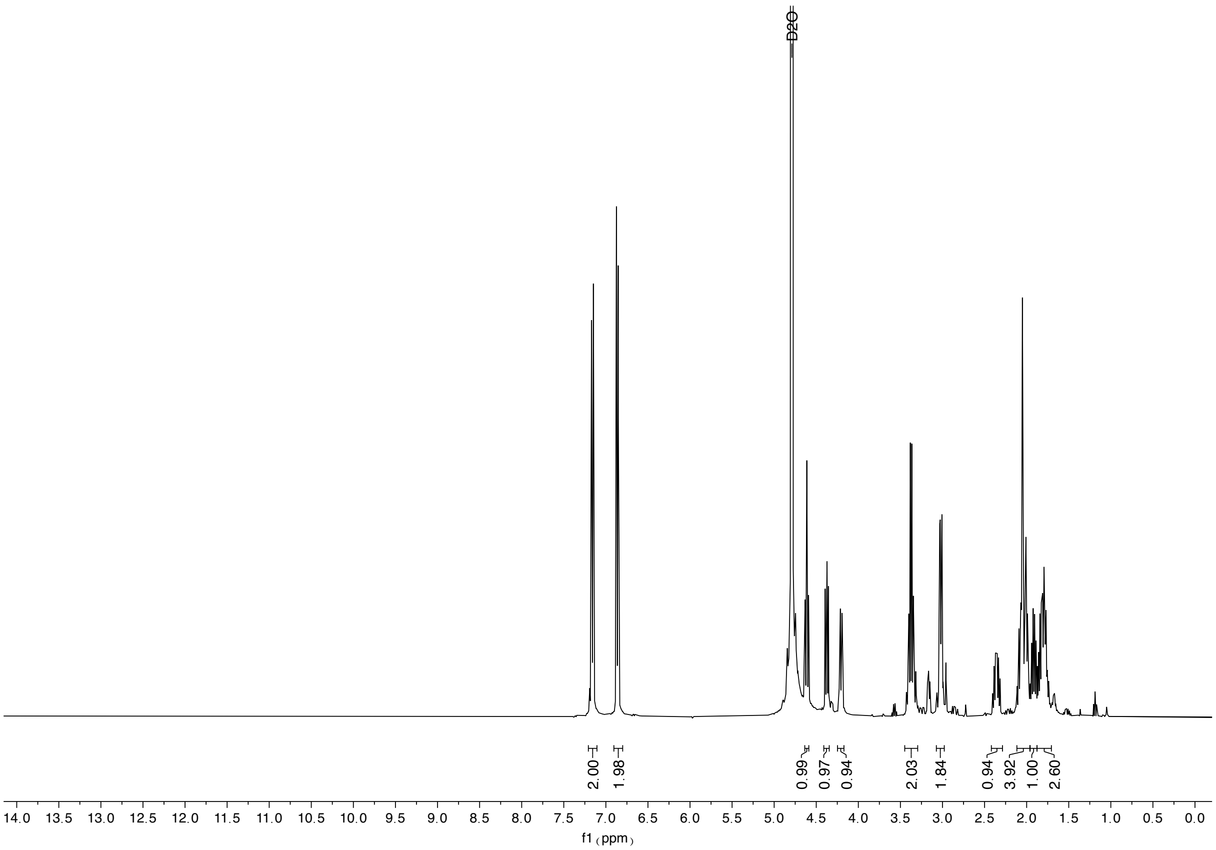

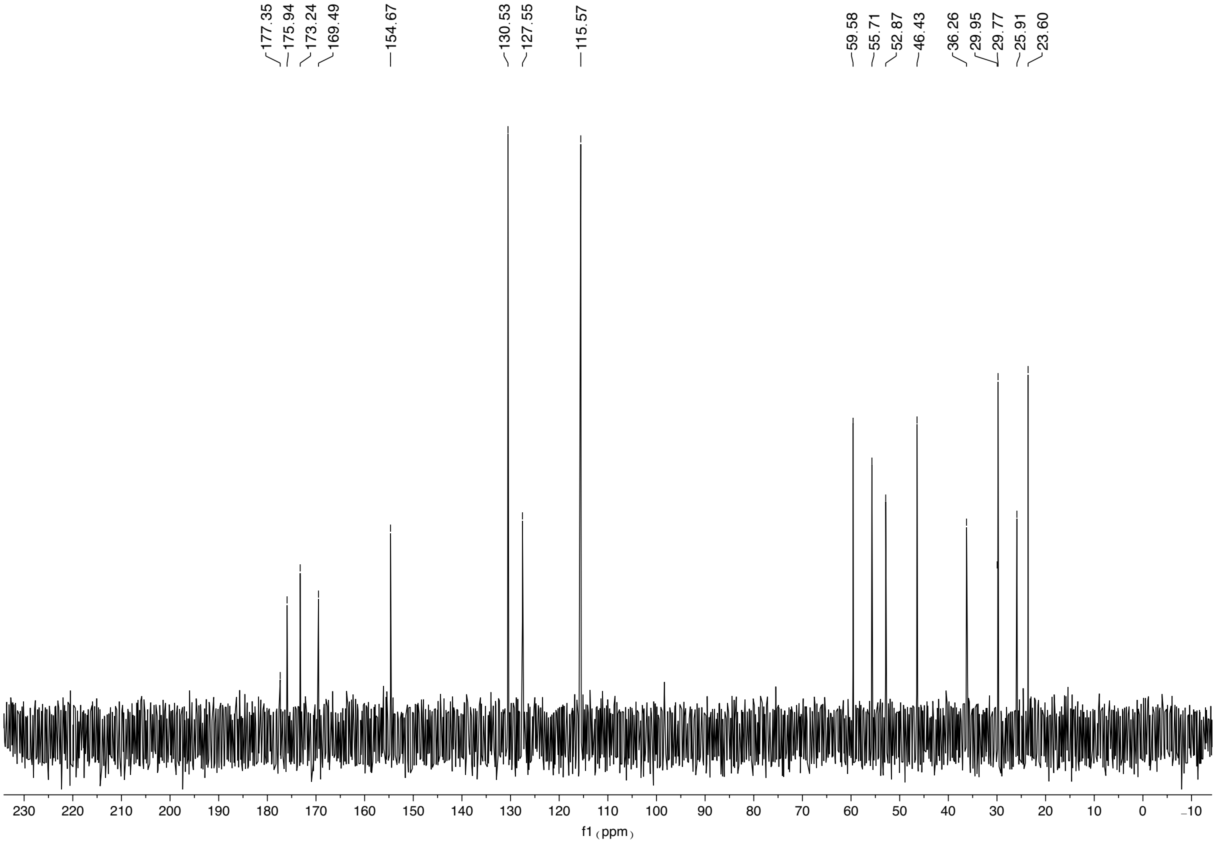

^{1}H and ^{13}C-NMR of H-D-Pro-D-Tyr-D-Glu-NH_2 · TFA (UTS-138):

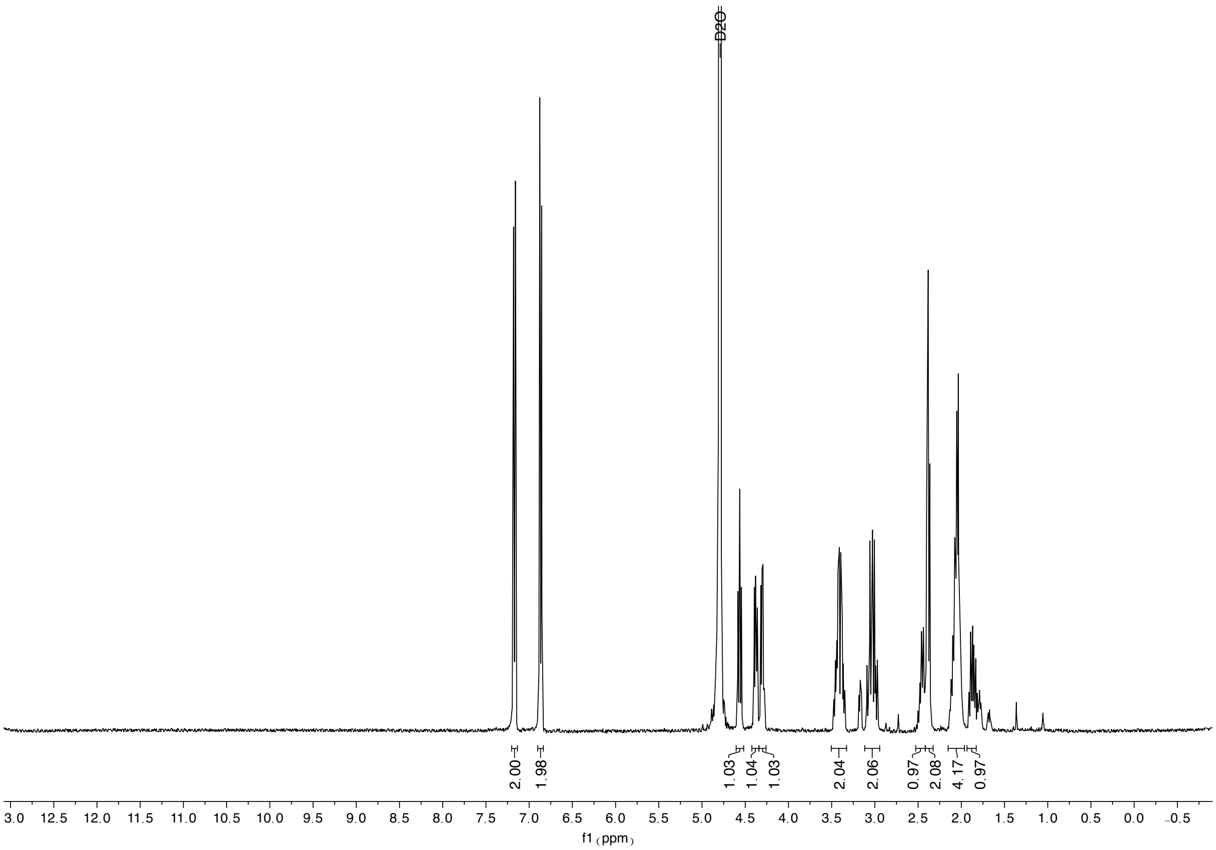

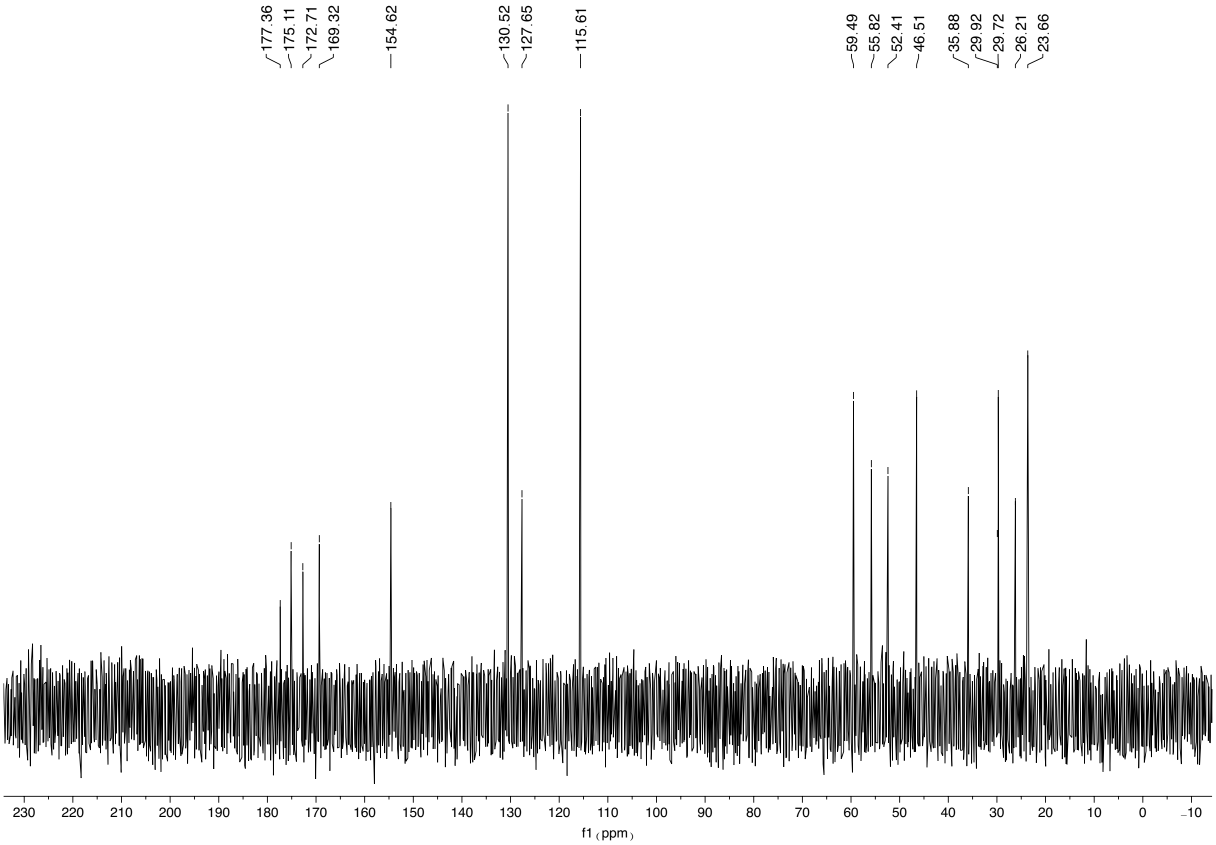

^{1}H and ^{13}C-NMR of H-D-Pro-CyLeu-D-Glu-NH$_2$ · TFA (UTS-139):

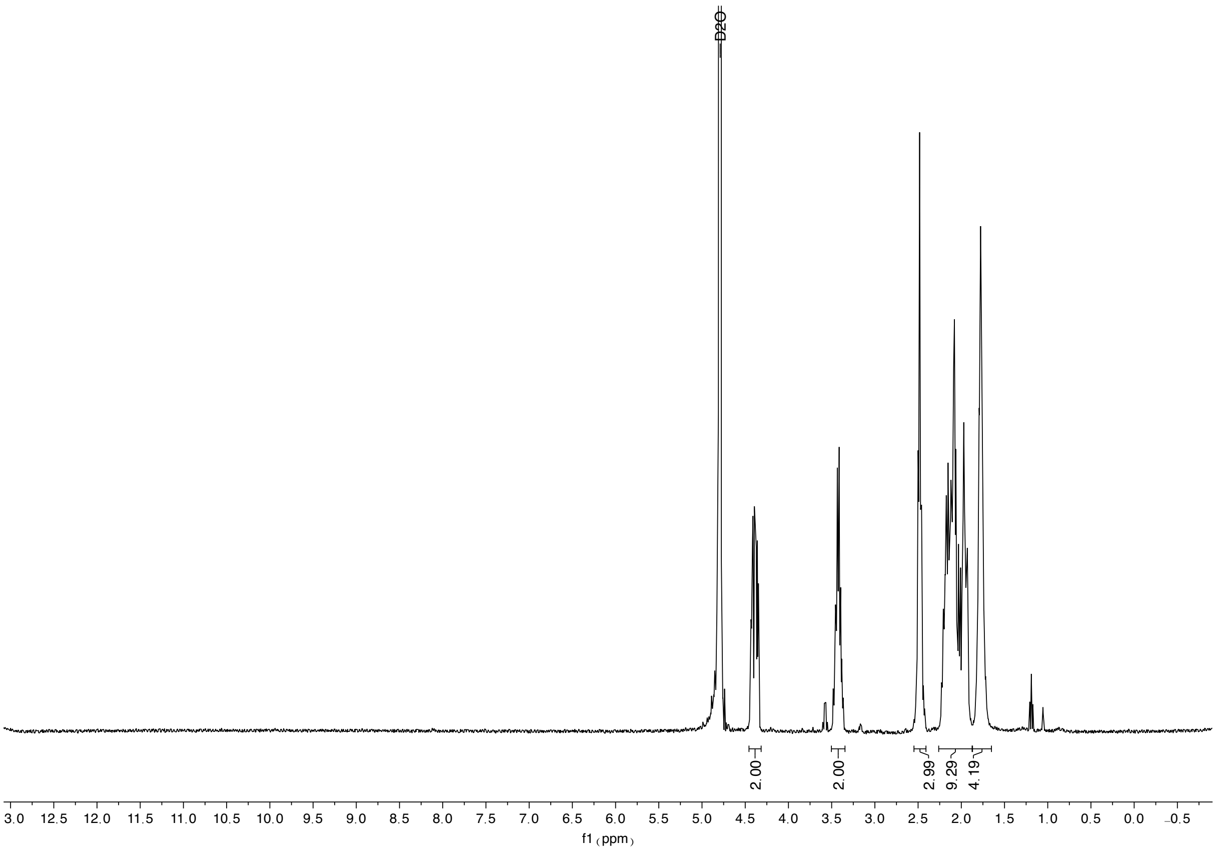

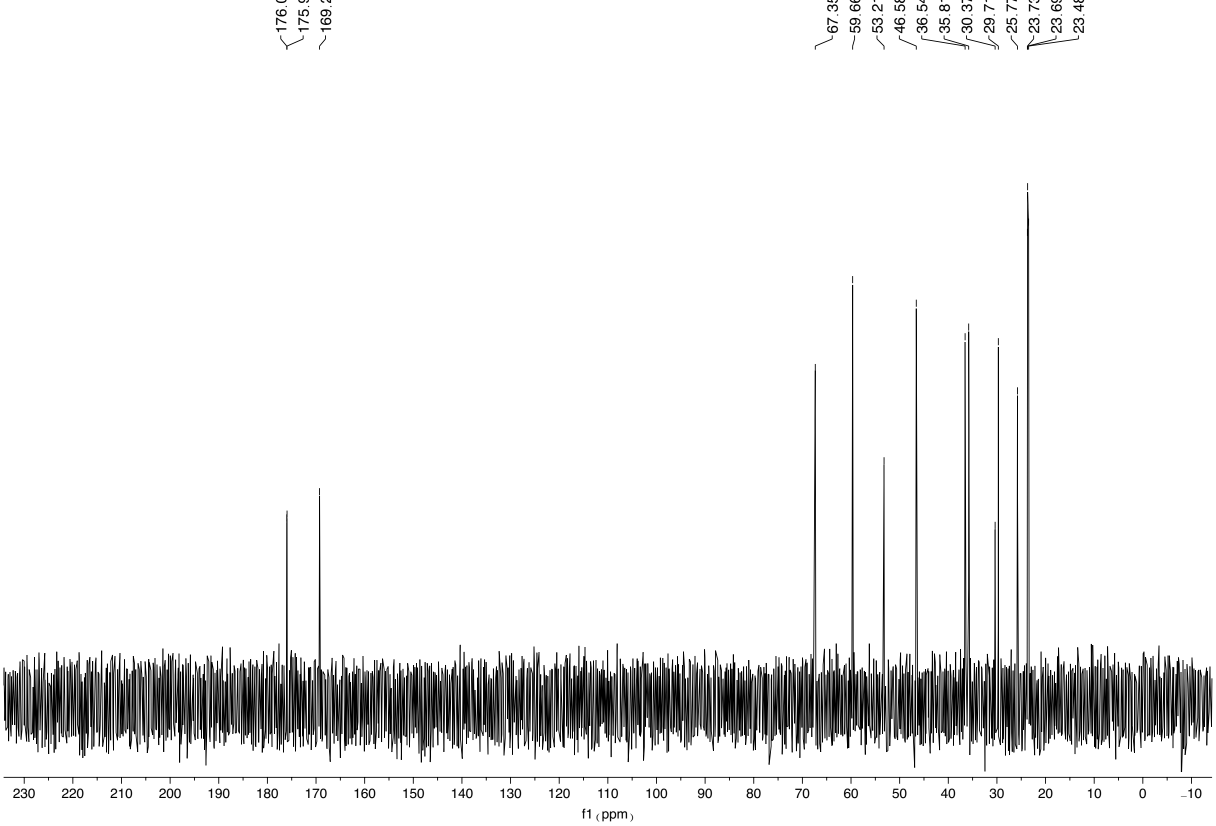

^{1}H and ^{13}C-NMR of H-D-Pro-Abz-D-Glu-NH$_2$ · TFA (UTS-140):

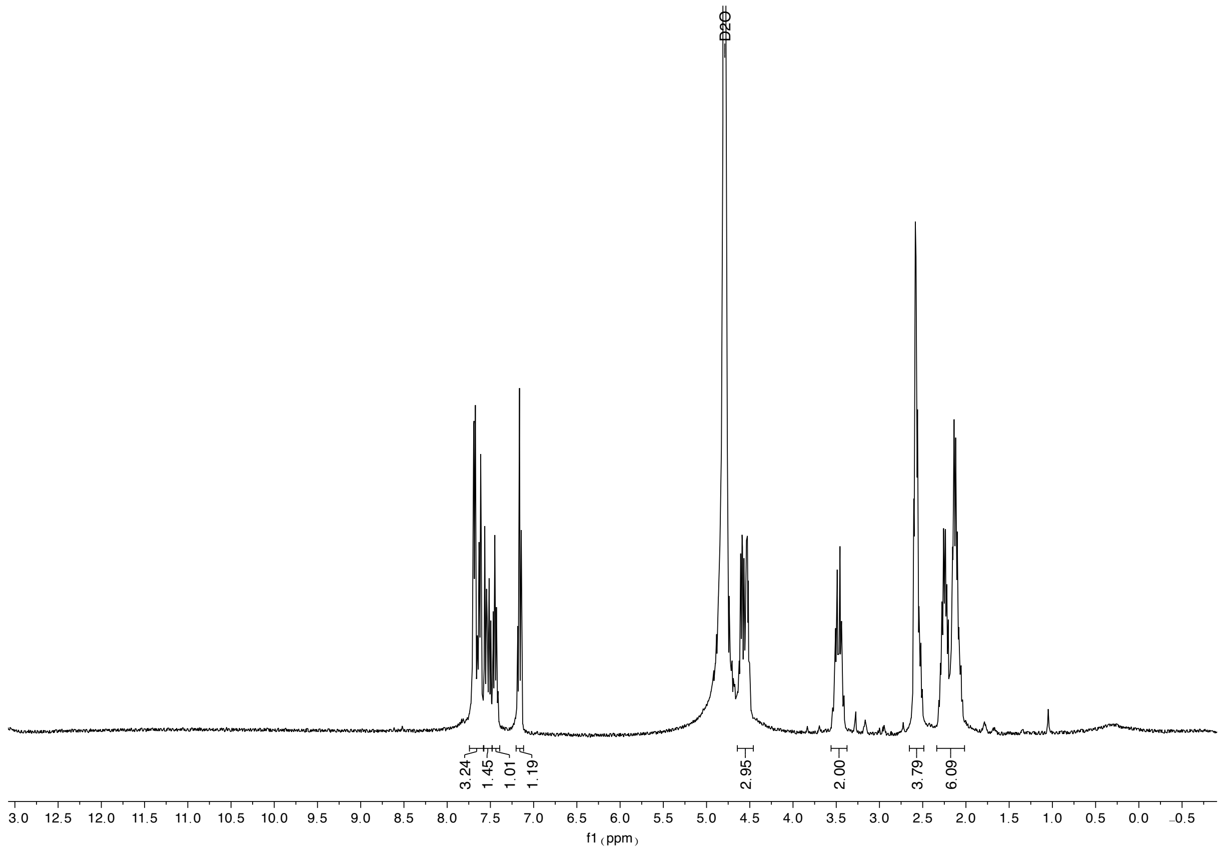

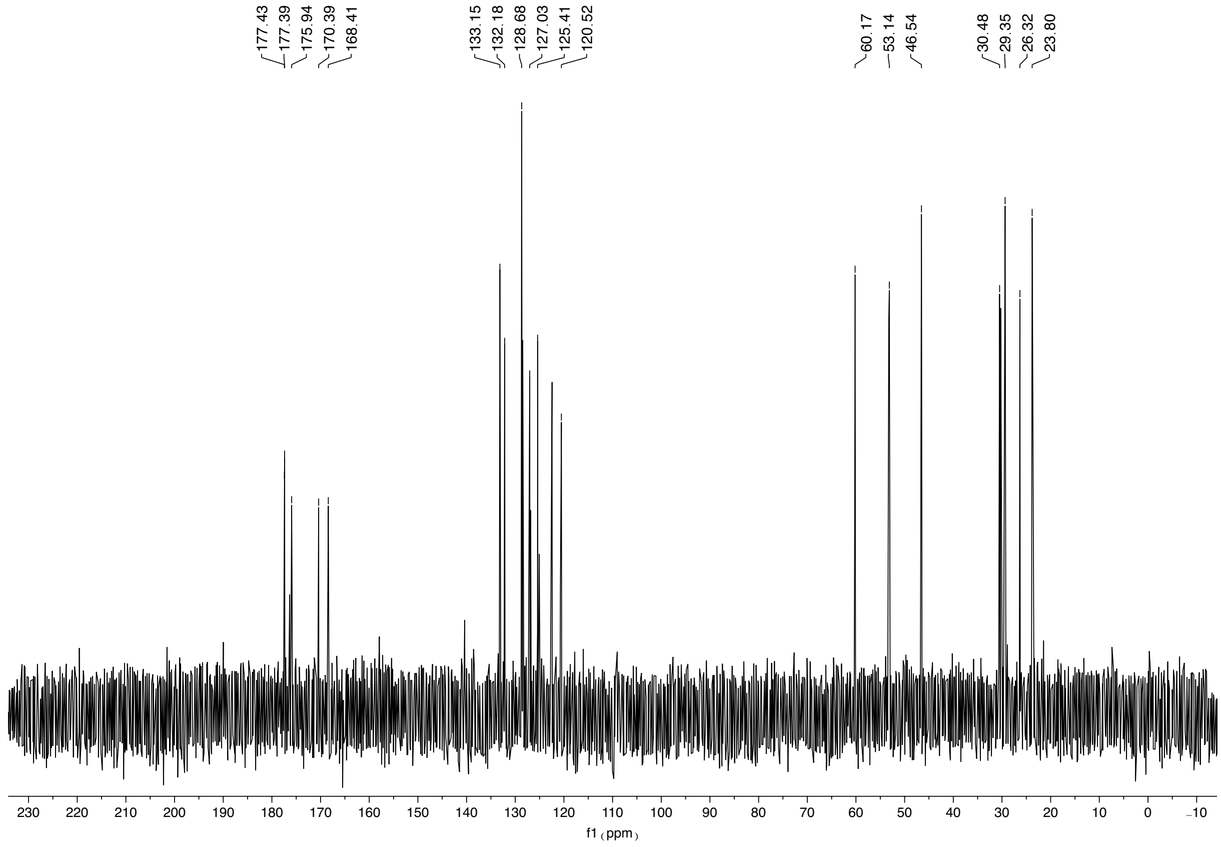

^{1}H and ^{13}C-NMR of H-D-Pro-Abz-D-Glu-NH$_2$ · TFA (UTS-140):

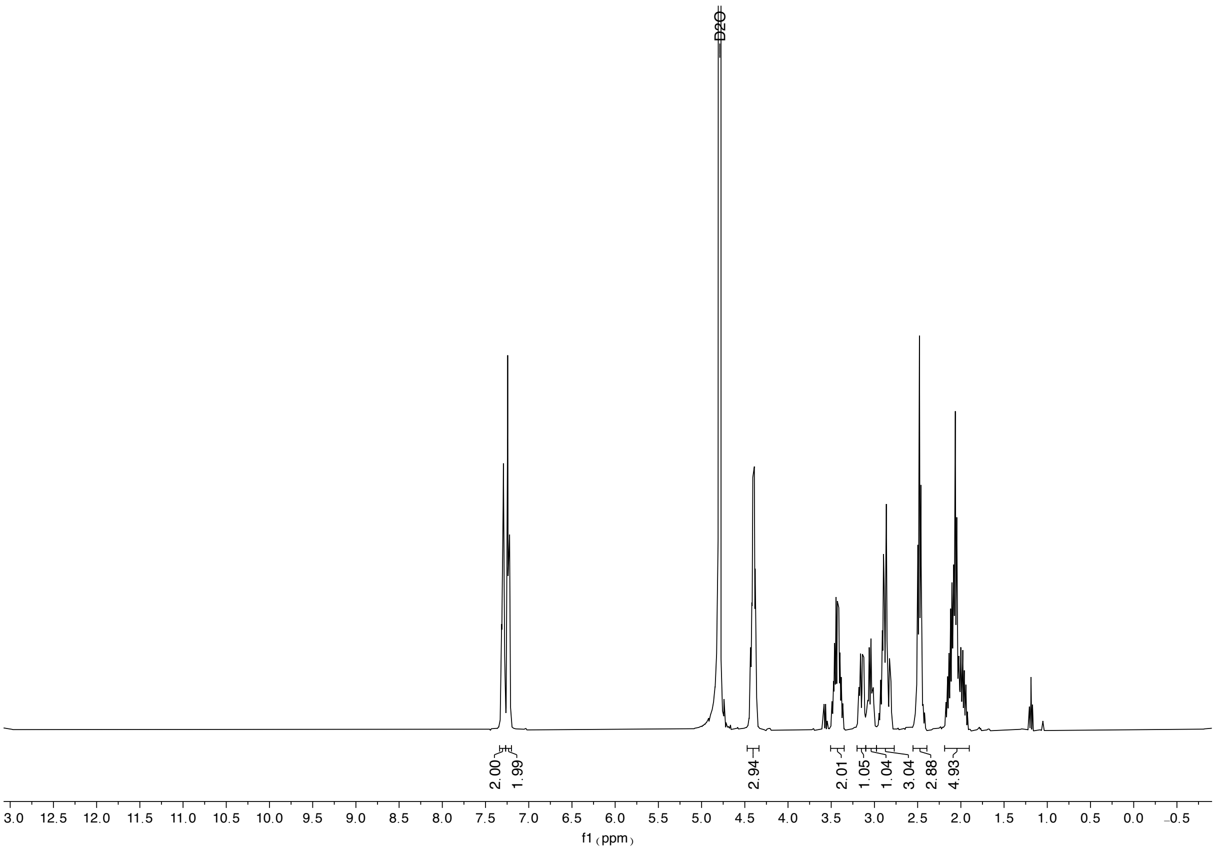

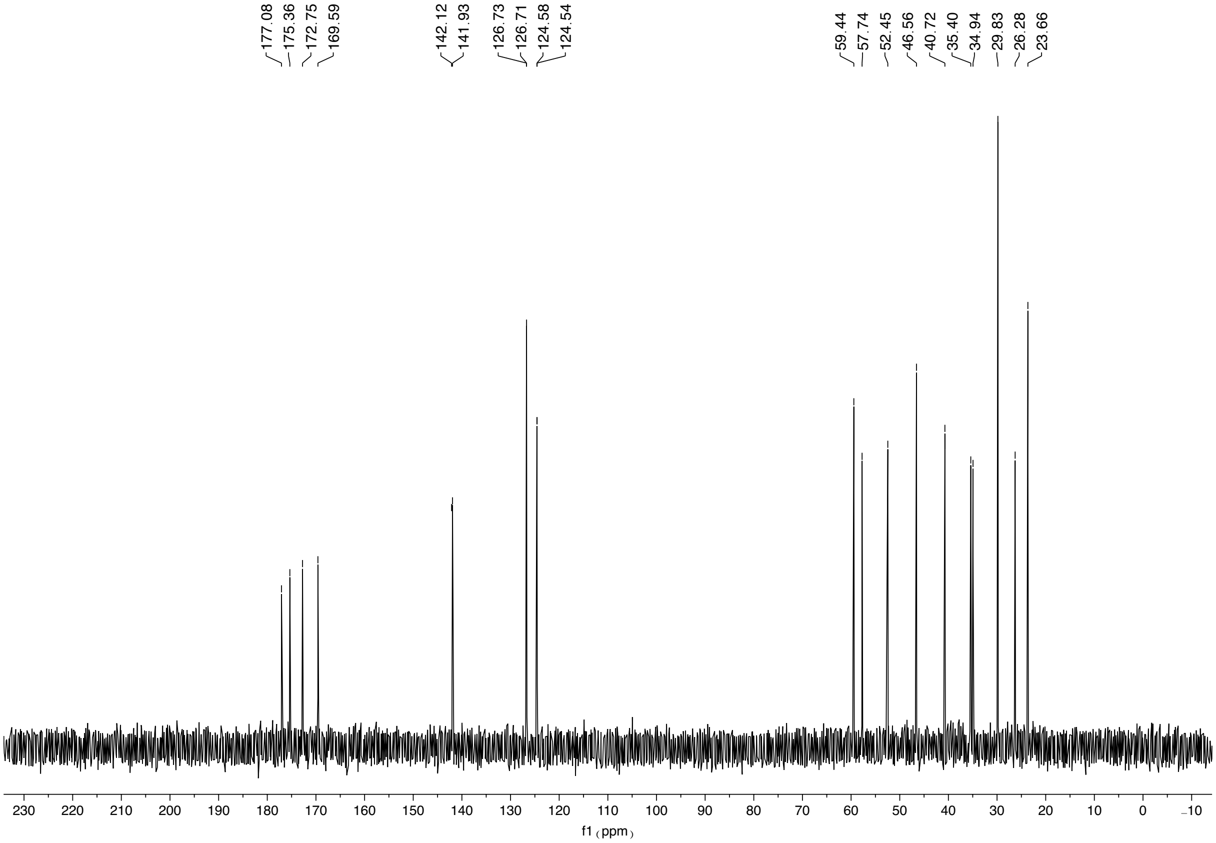

^{1}H and ^{13}C-NMR of H-D-Pro-L-Tyr-L-Tyr-NH_2 · TFA (UTS-142):

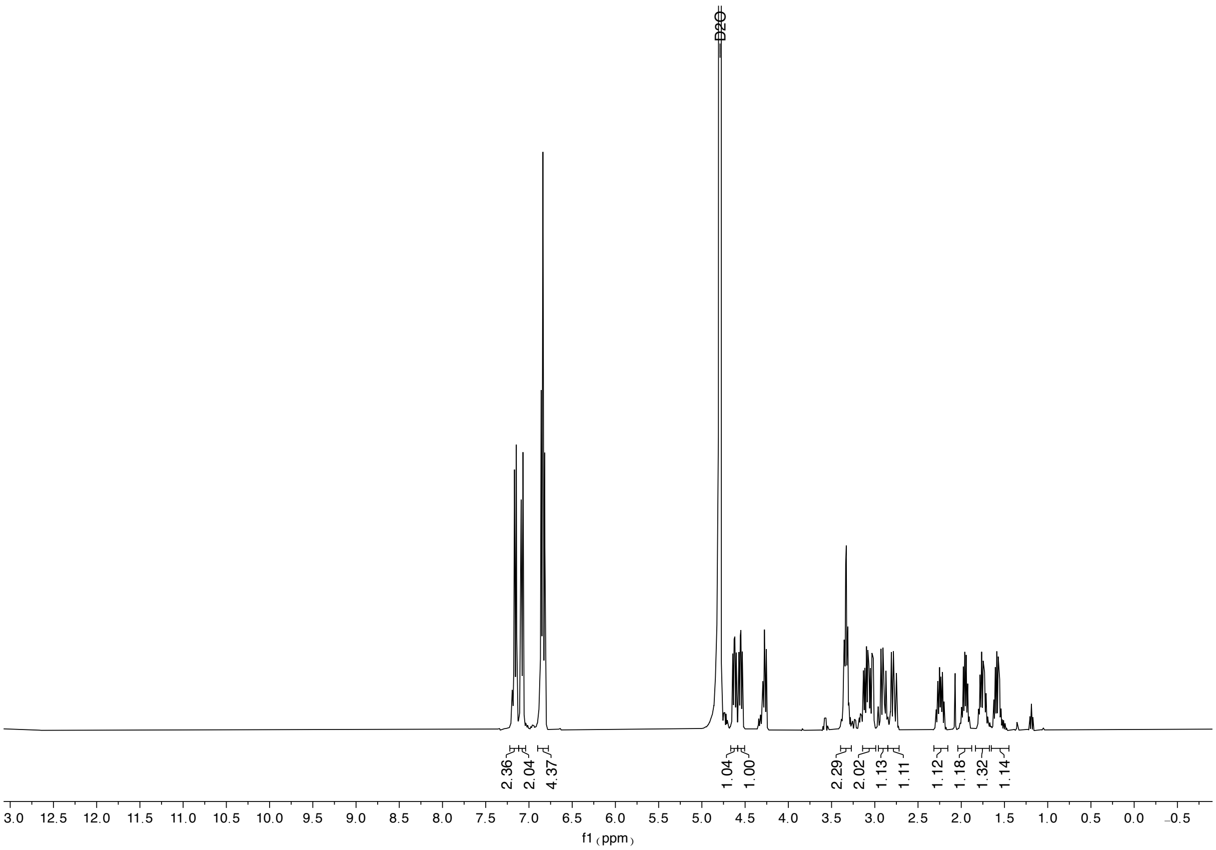

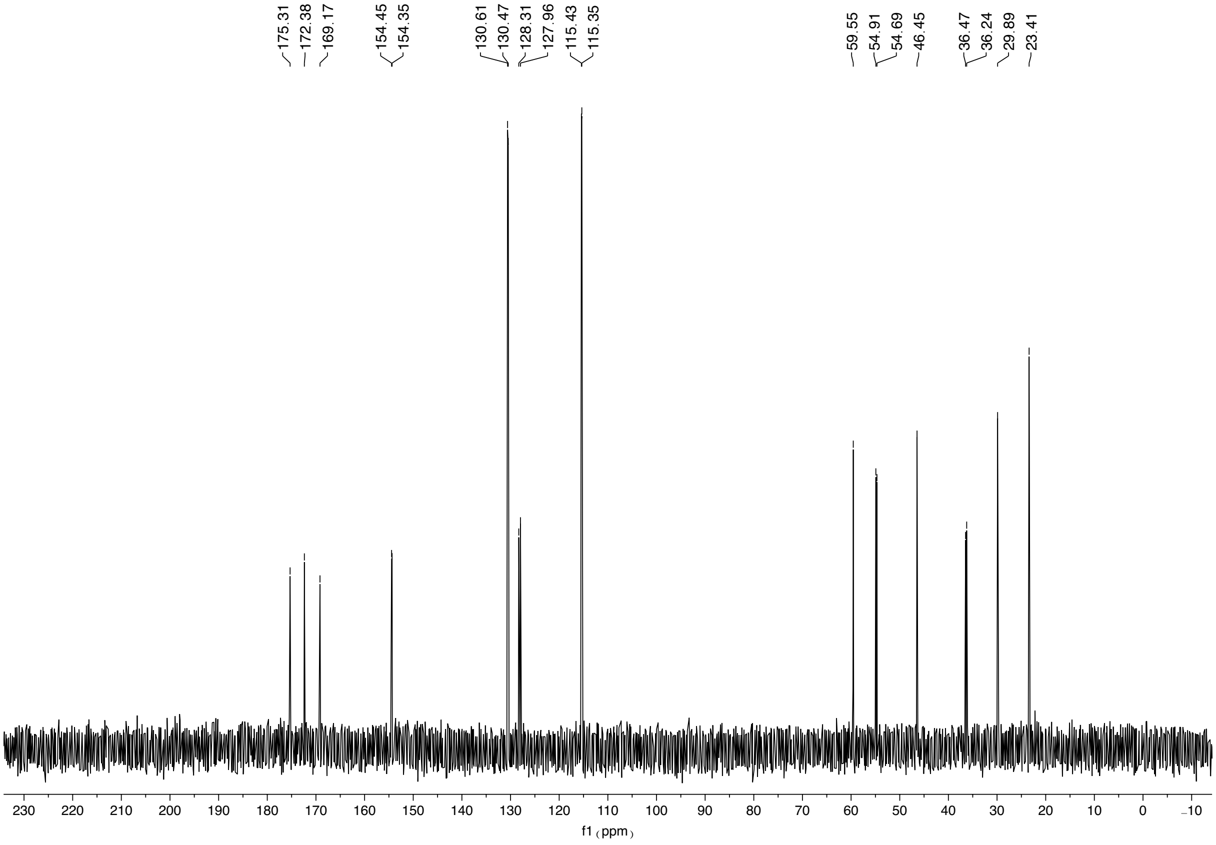

^{1}H and ^{13}C-NMR of H-D-Pro-L-Tyr-D-Tyr-NH$_2$ · TFA (UTS-143):

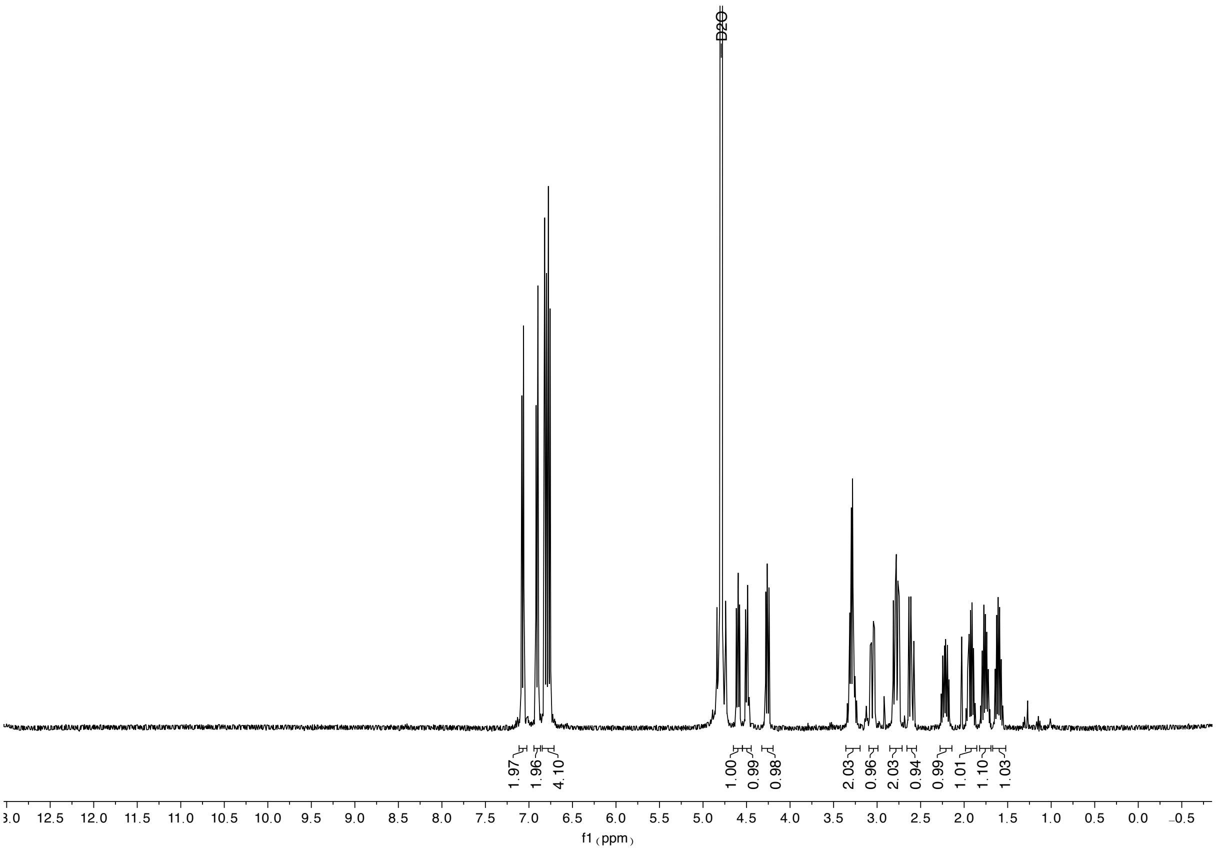

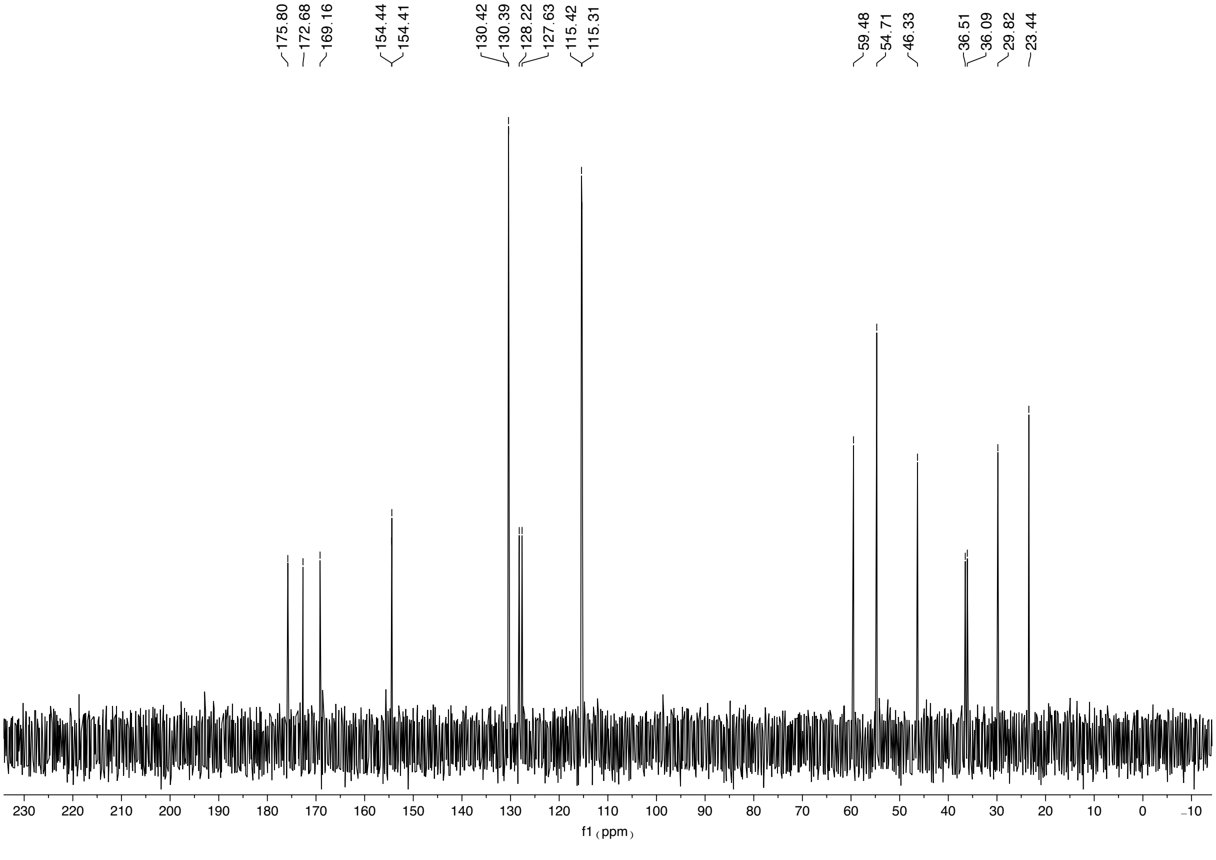

^{1}H and ^{13}C-NMR of H-D-Pro-L-Tyr-CyLeu-NH_2 · TFA (UTS-144):

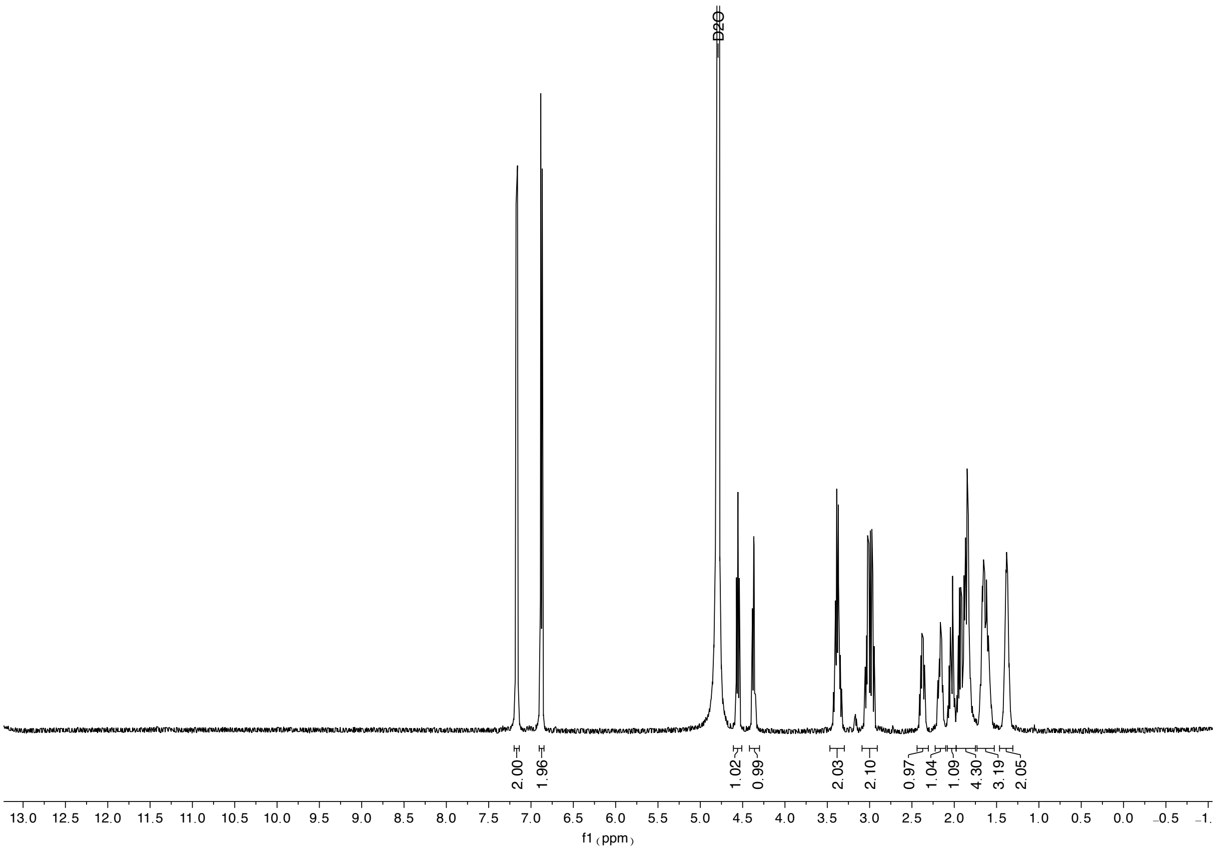

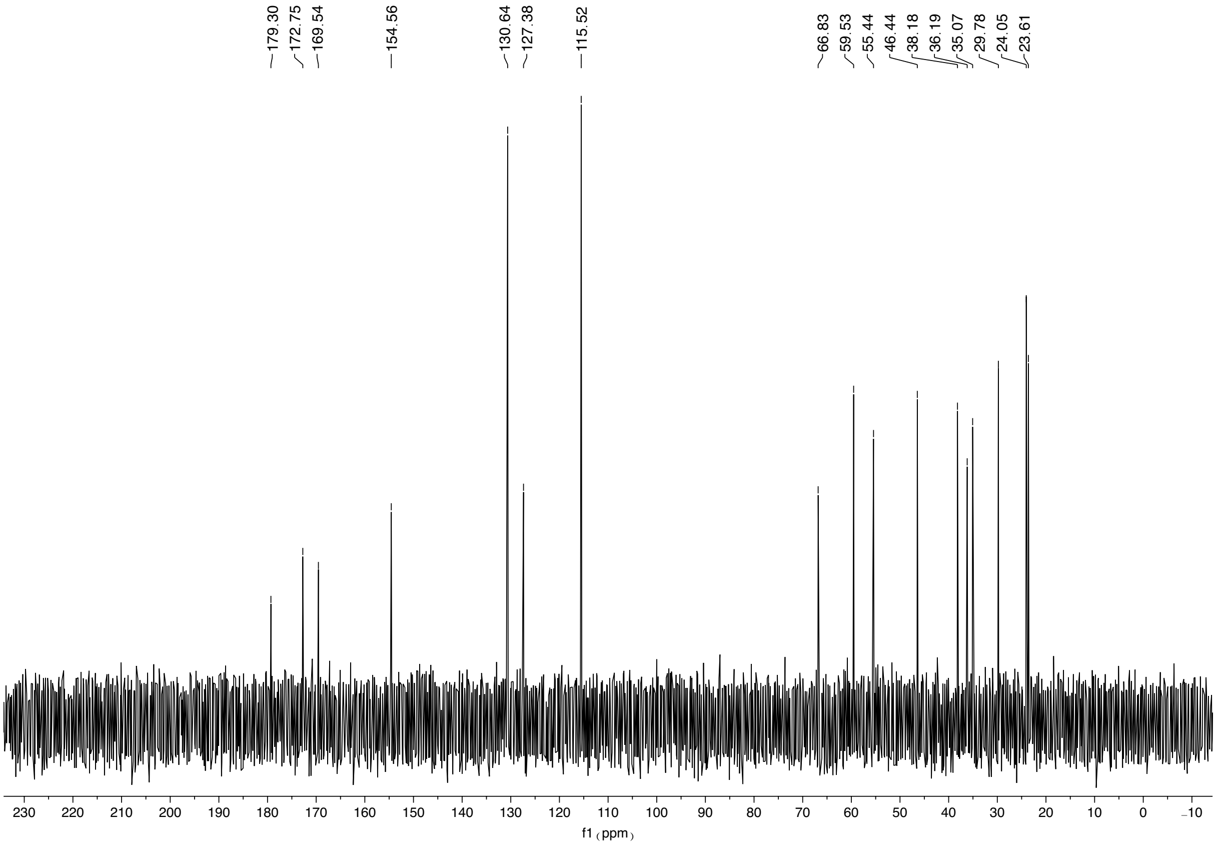

^{1}H and ^{13}C-NMR of H-D-Pro-L-Tyr-Abz-NH₂ · TFA (UTS-145):

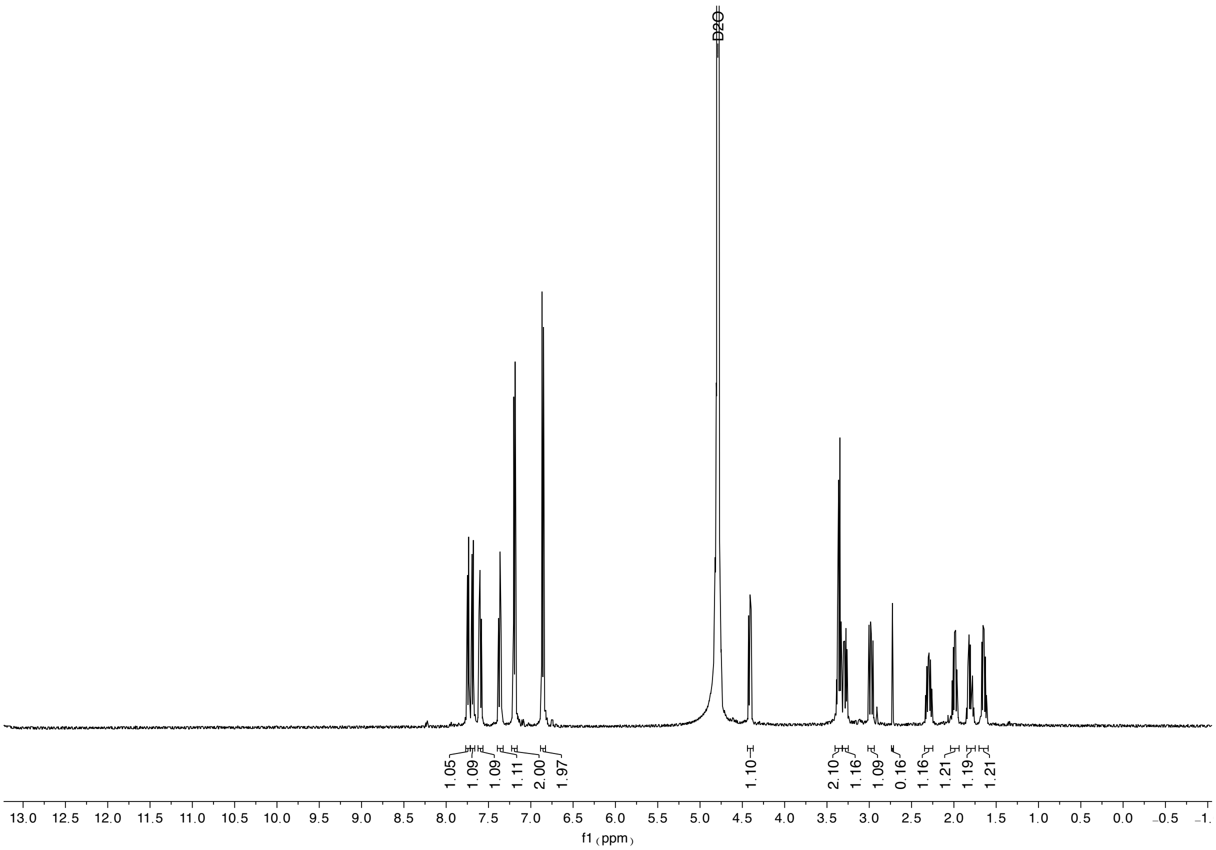

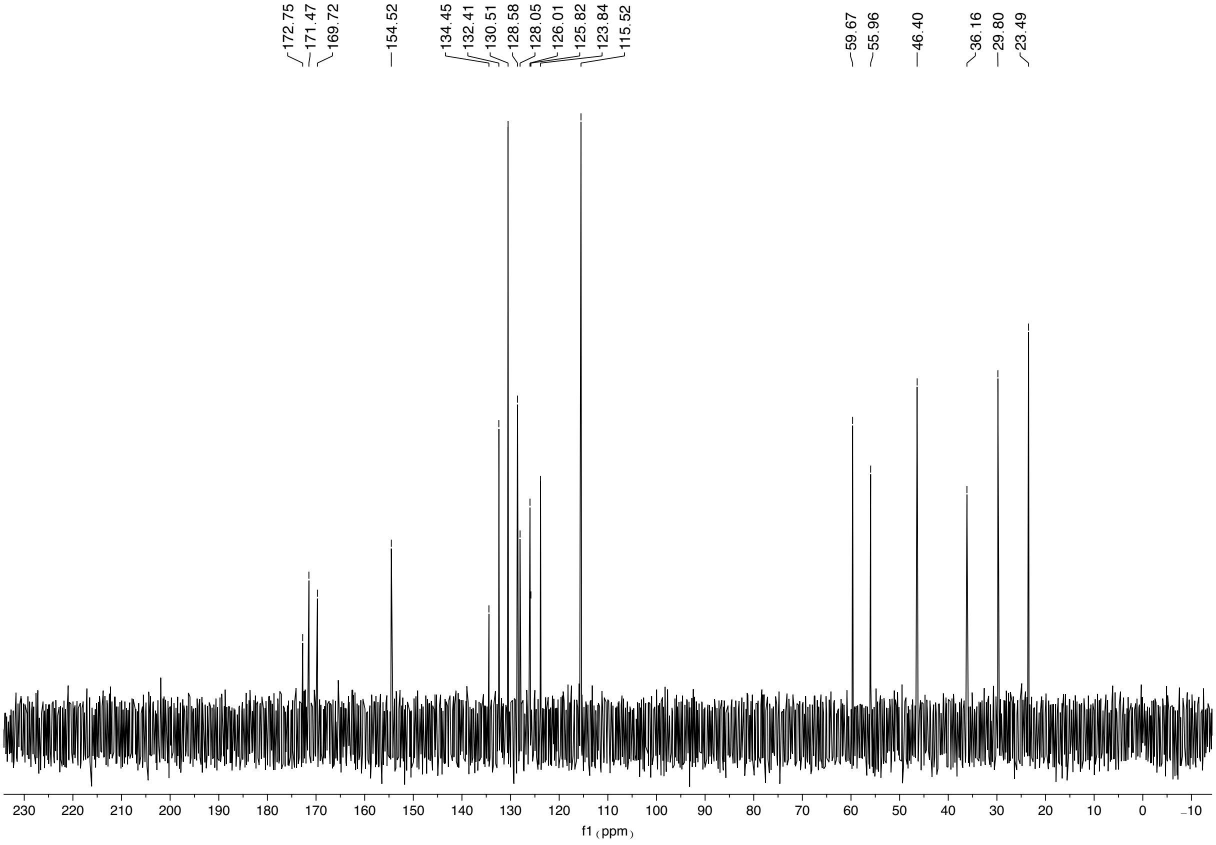

^{1}H and ^{13}C-NMR of H-D-Pro-L-Tyr-D-Ind-NH$_2$ · TFA (UTS-146):

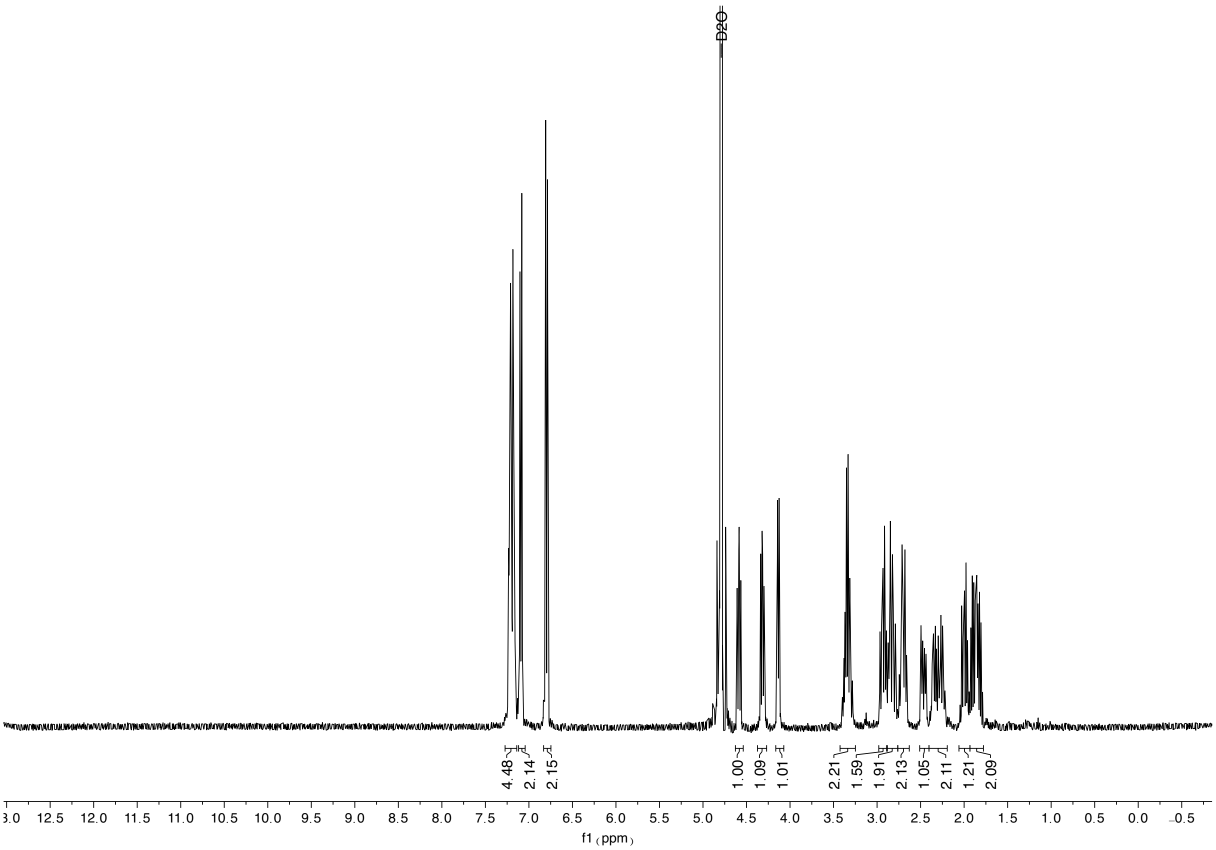

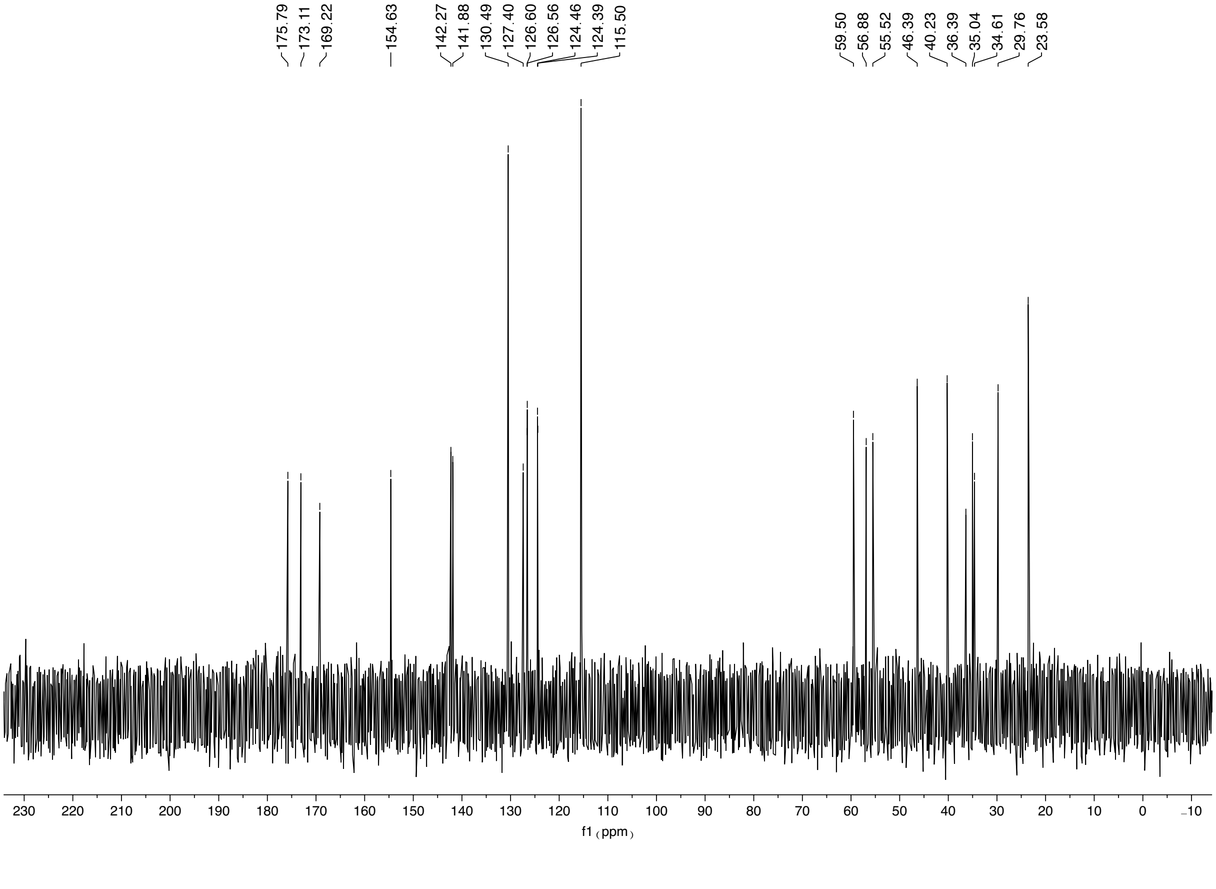

^{1}H and ^{13}C-NMR of H-D-Pro-D-Tyr-L-Tyr-NH$_2$ · TFA (UTS-147):

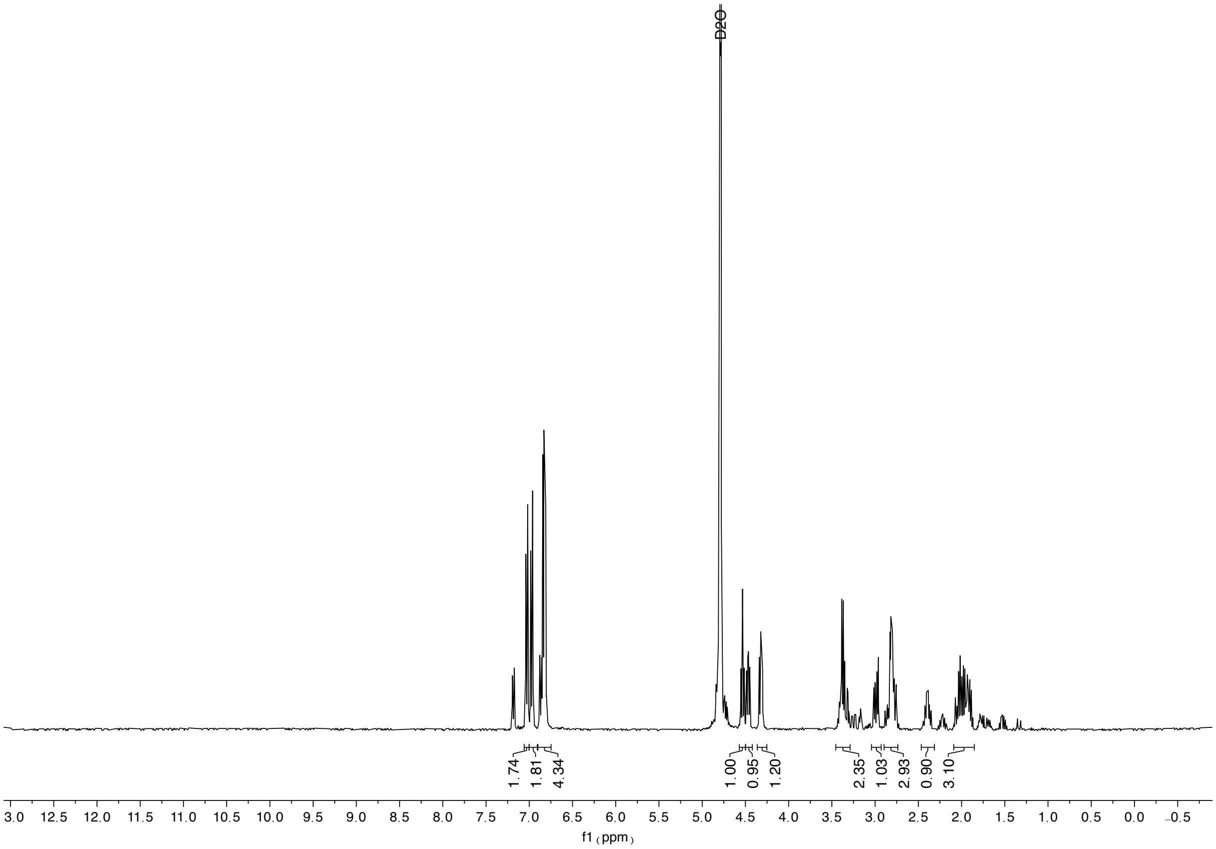

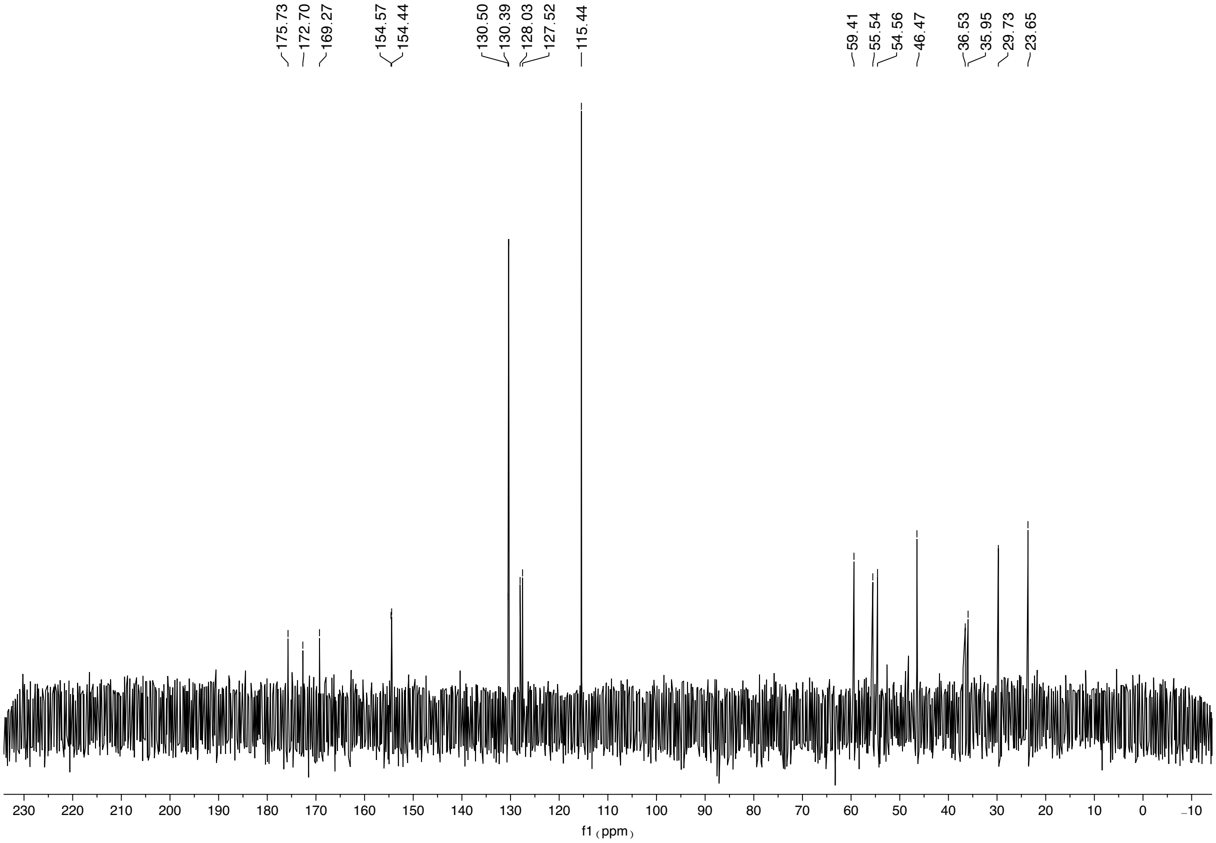

^{1}H and ^{13}C-NMR of H-D-Pro-CyLeu-L-Tyr-NH_2 · TFA (UTS-148):

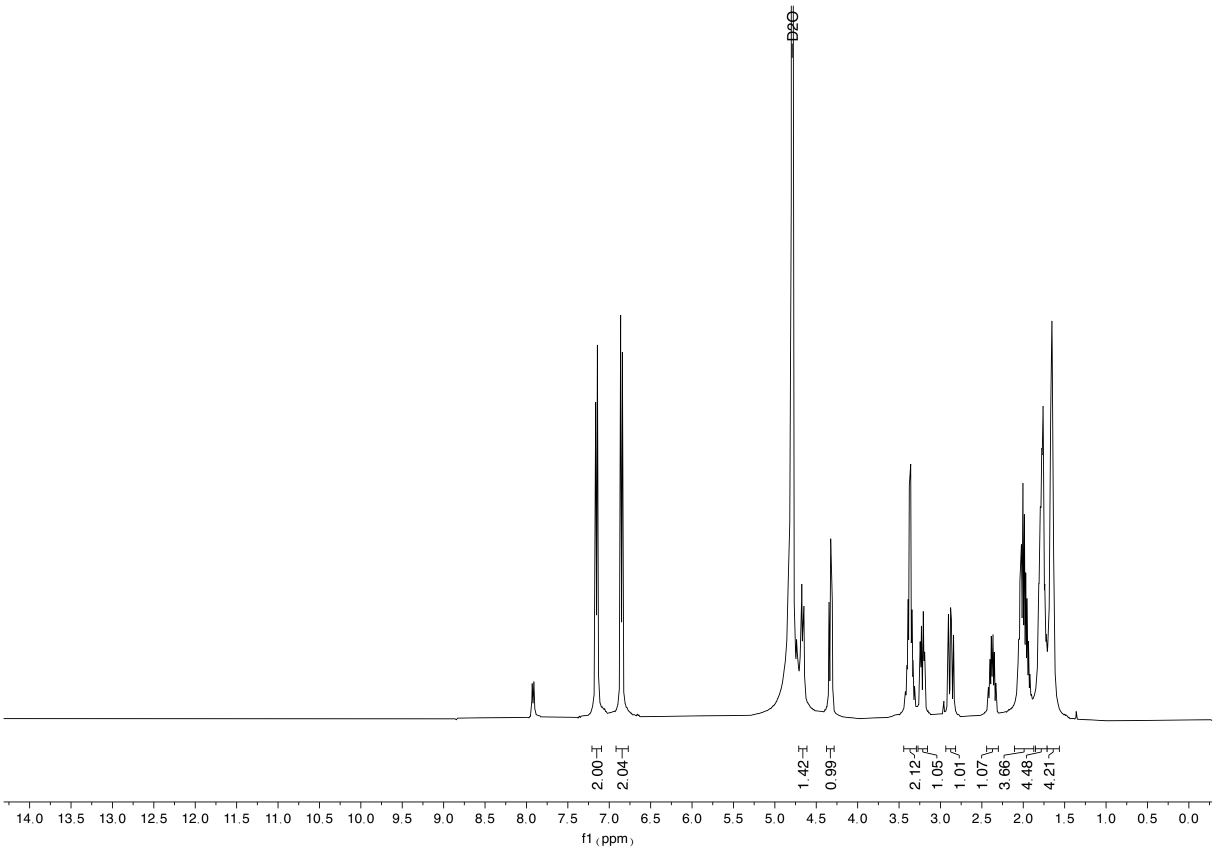

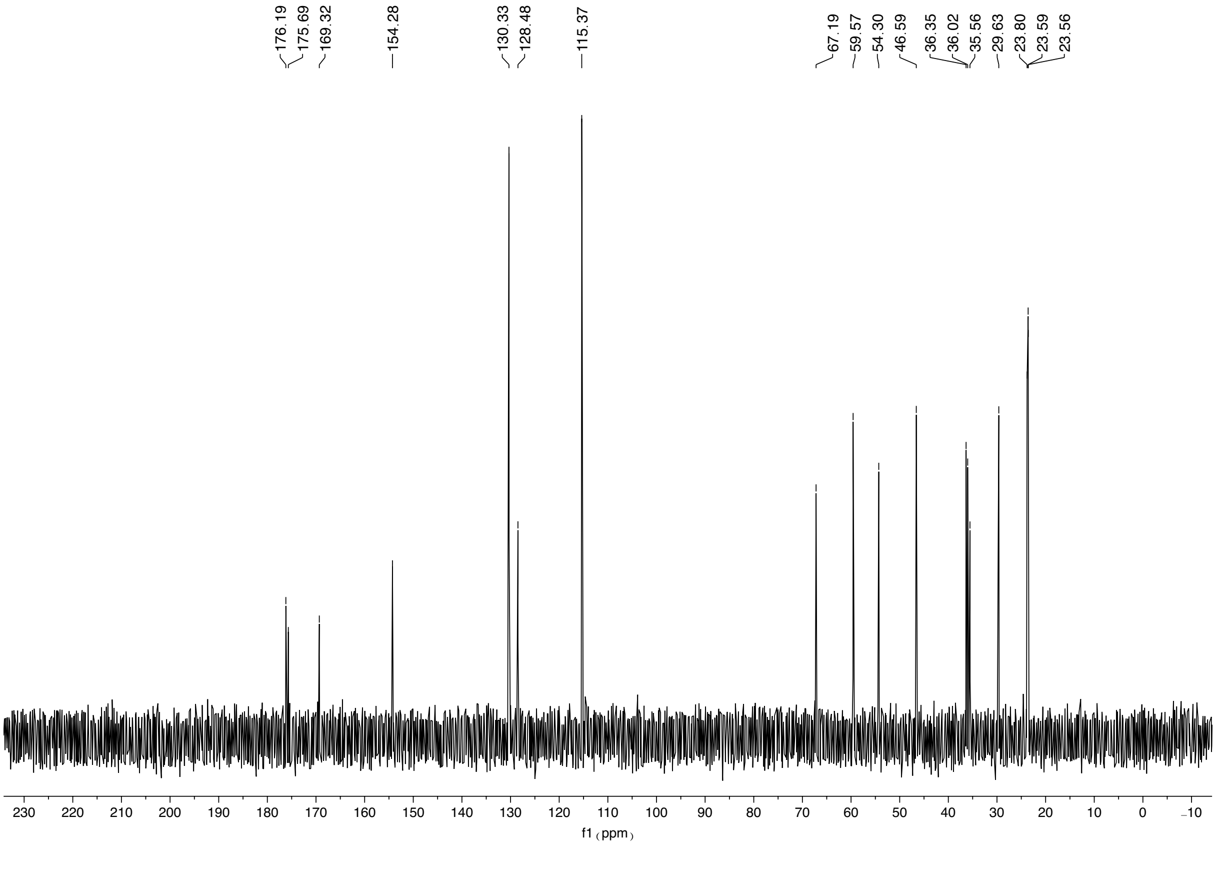

^{1}H and ^{13}C-NMR of H-D-Pro-Abz-L-Tyr-NH_2 · TFA (UTS-149):

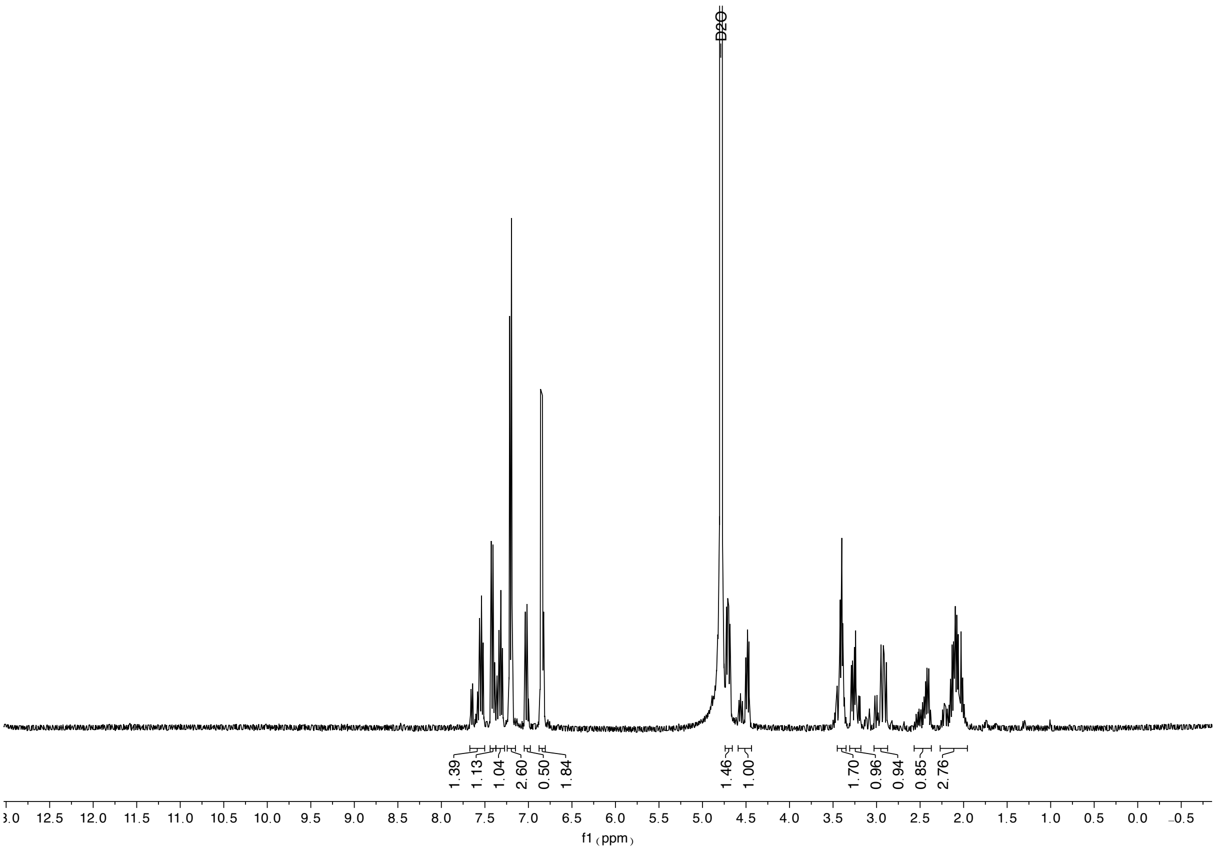

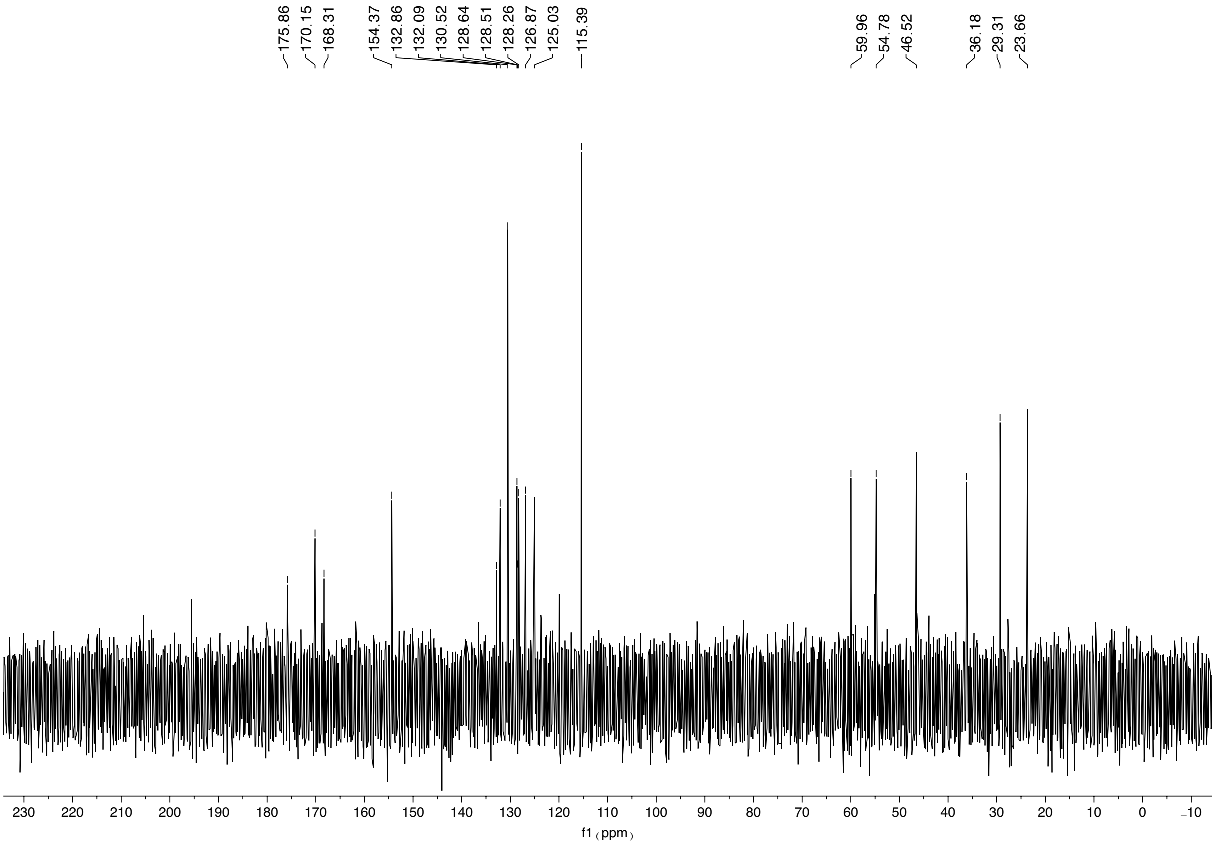

^{1}H and ^{13}C-NMR of H-D-Pro-D-Ind-L-Tyr-NH$_2$ · TFA (UTS-150):

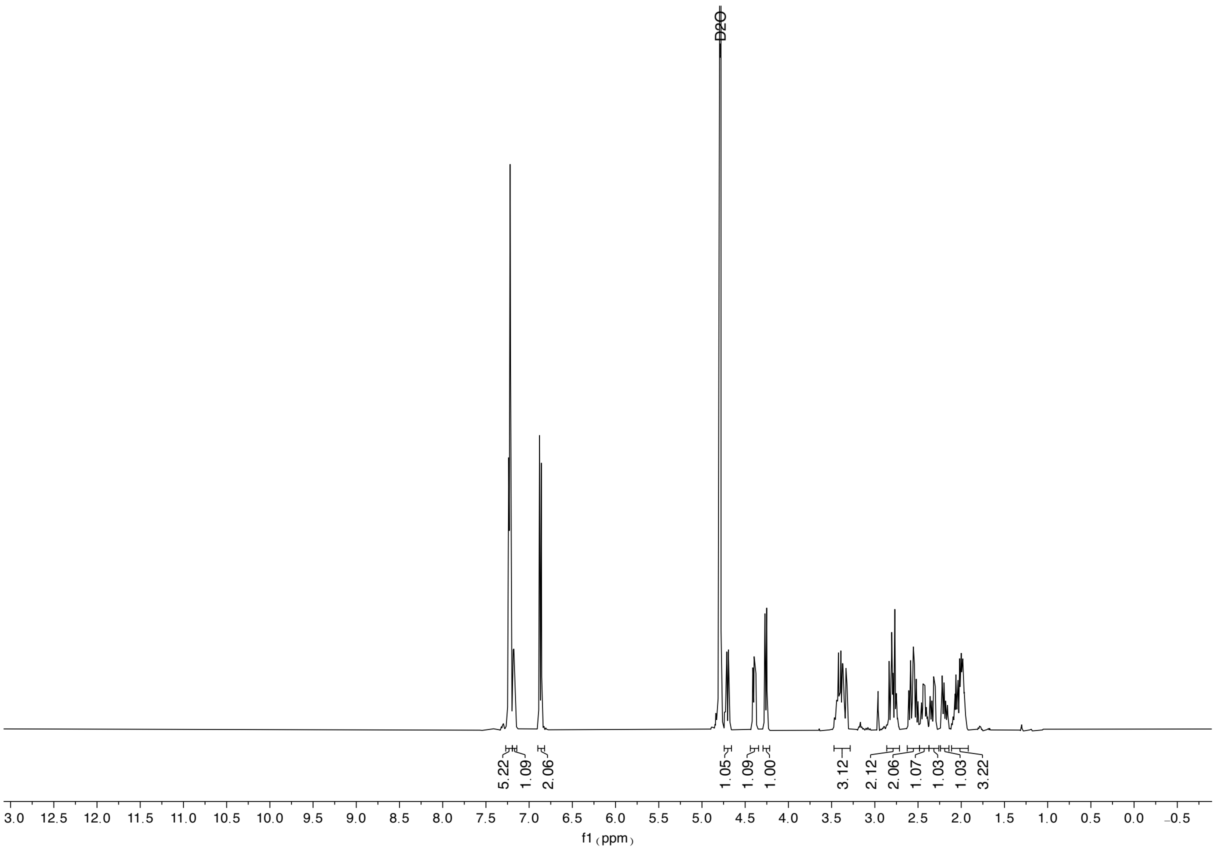

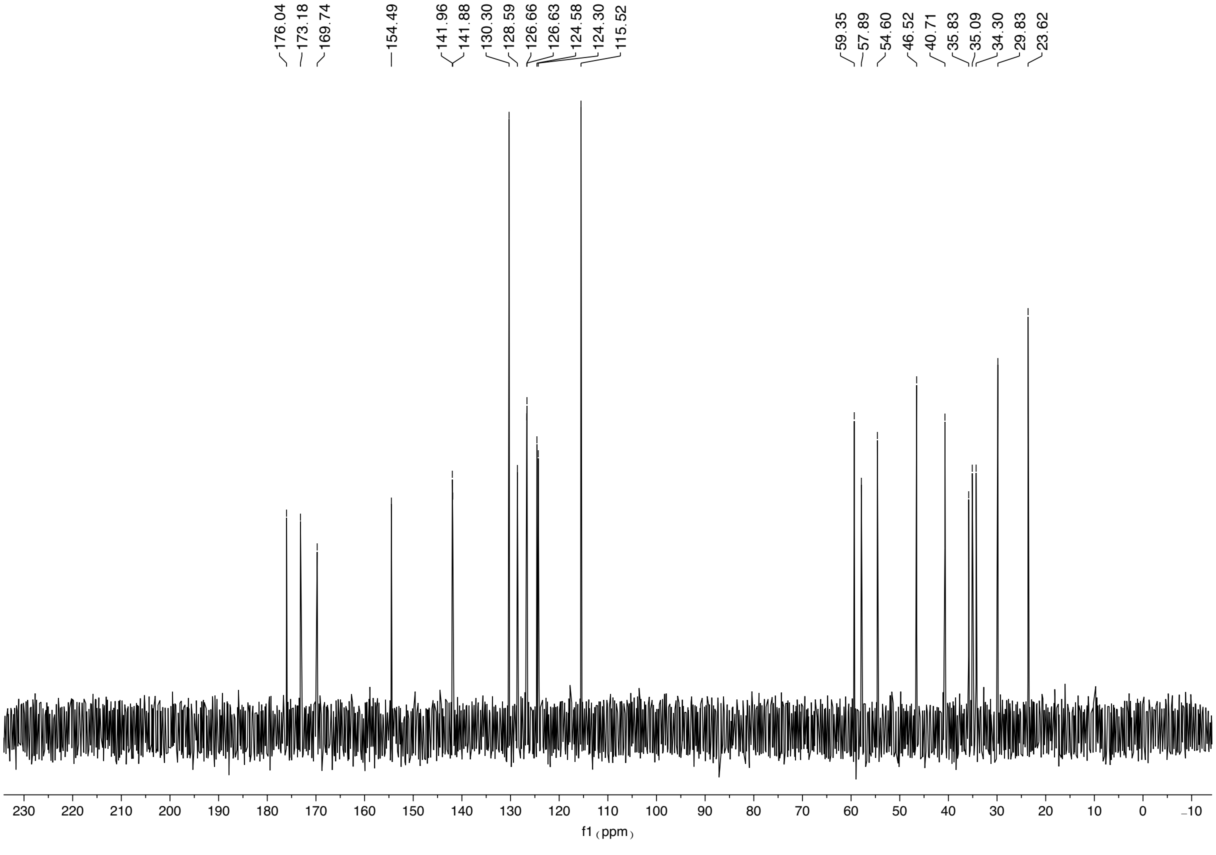

^{1}H and ^{13}C-NMR of H-D-Pro-D-Tyr-D-Tyr-NH_2 · TFA (UTS-151):

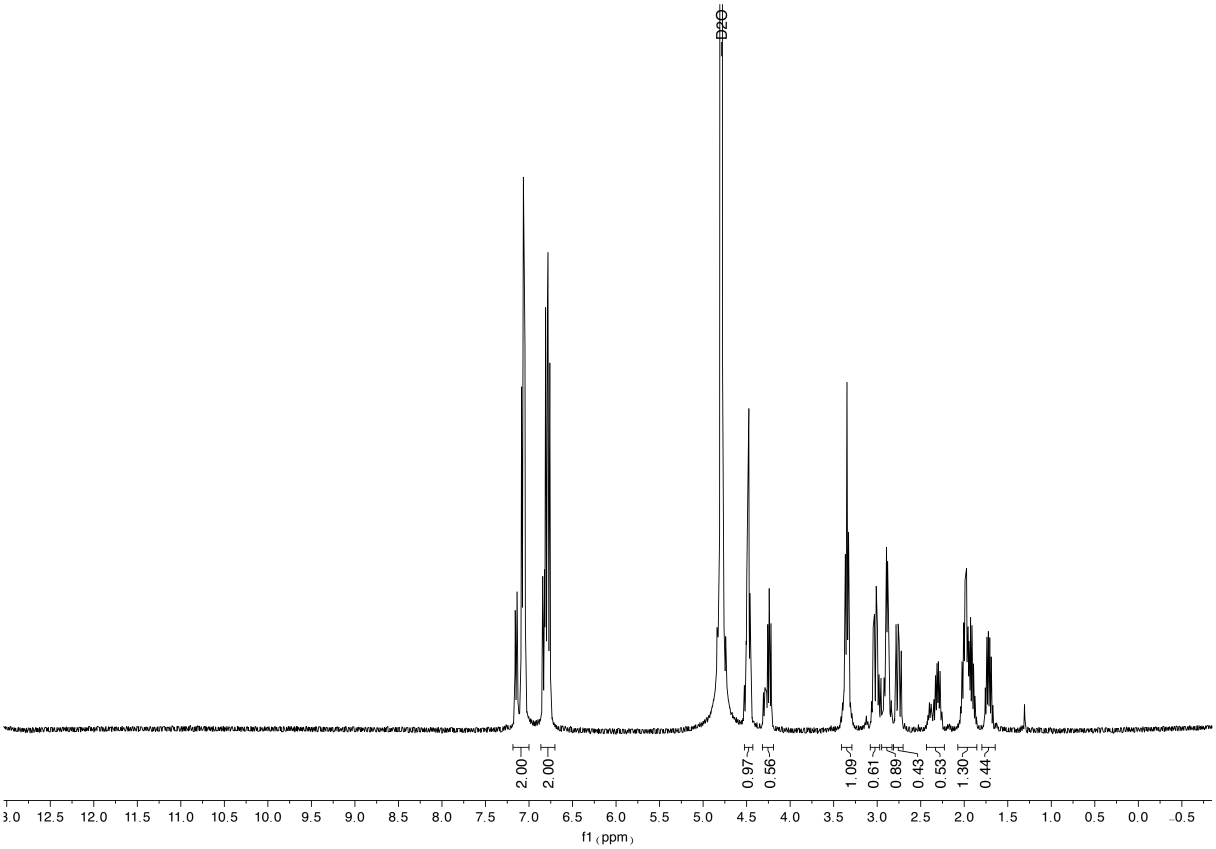

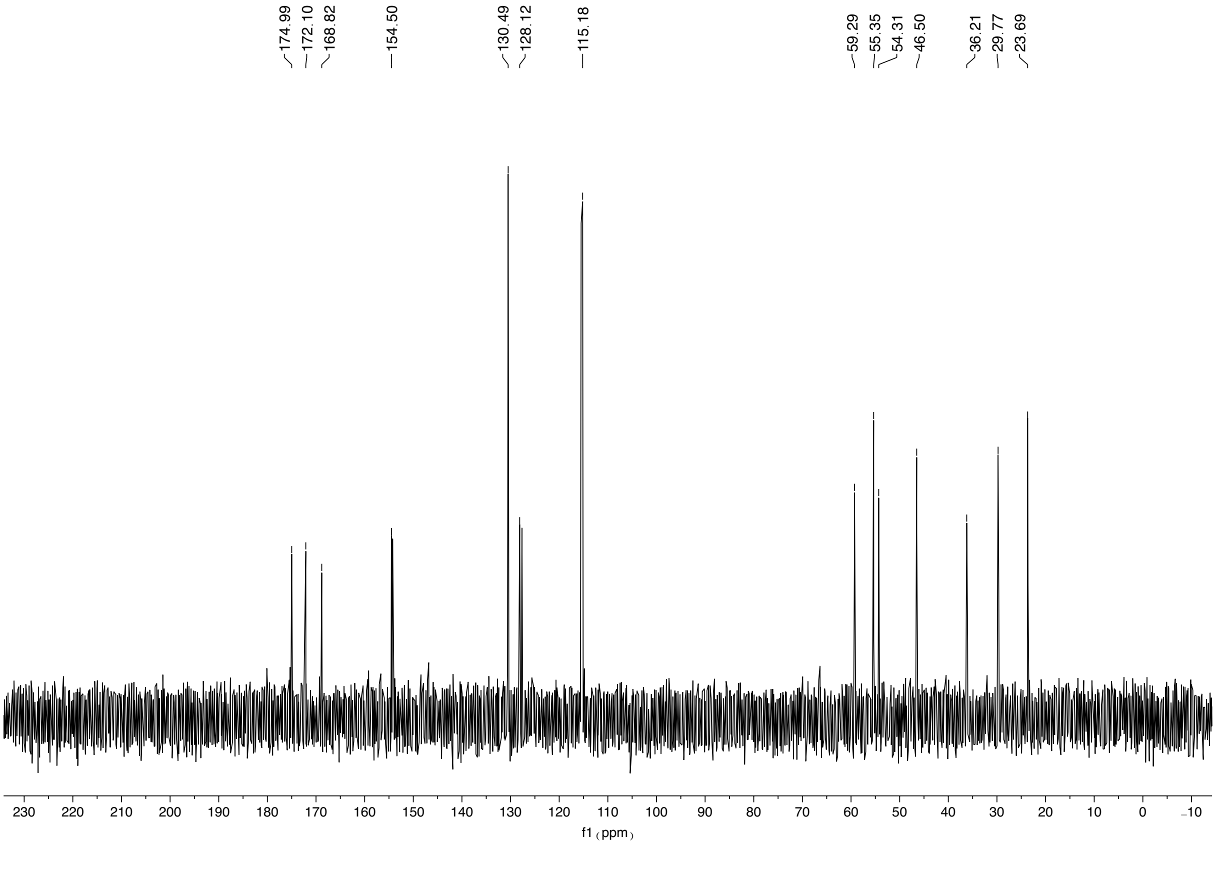

^{1}H and ^{13}C-NMR of H-D-Pro-D-Tyr-CyLeu-NH$_2$ · TFA (UTS-152):

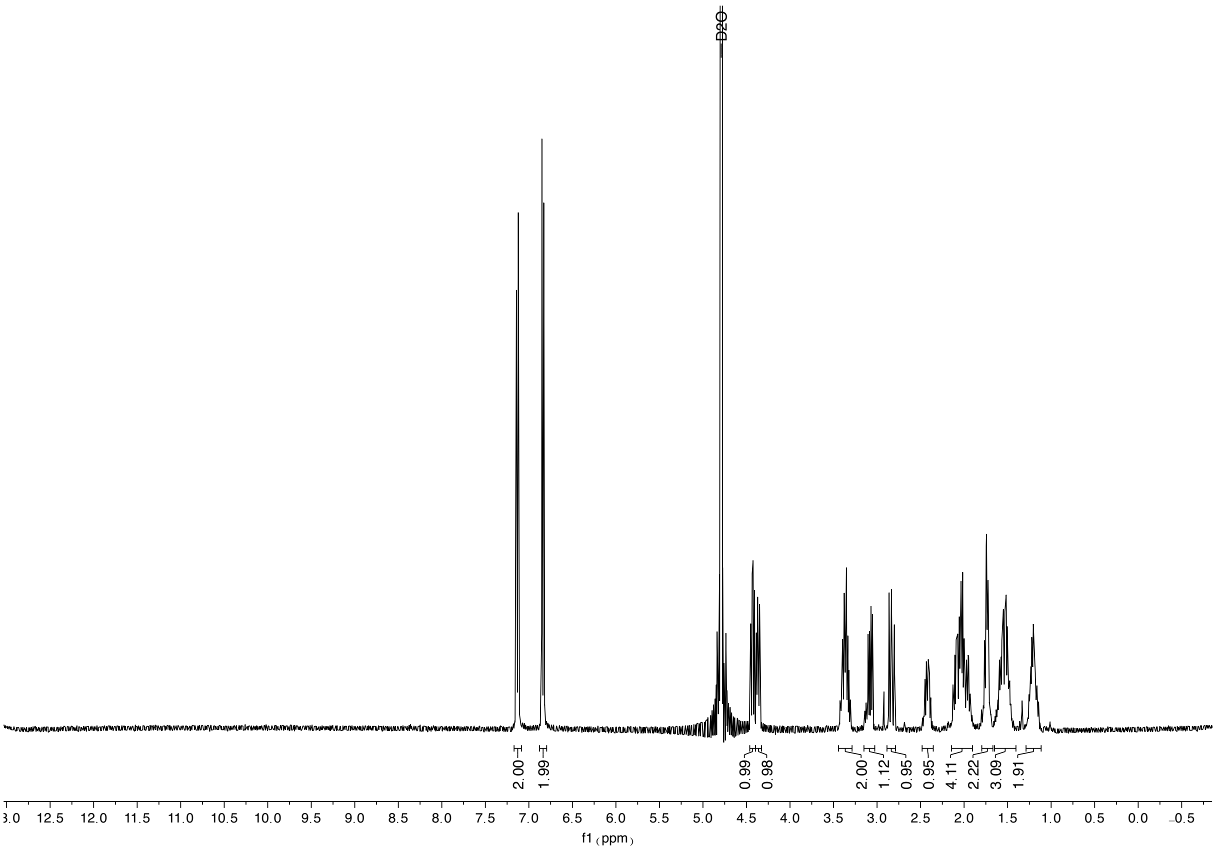

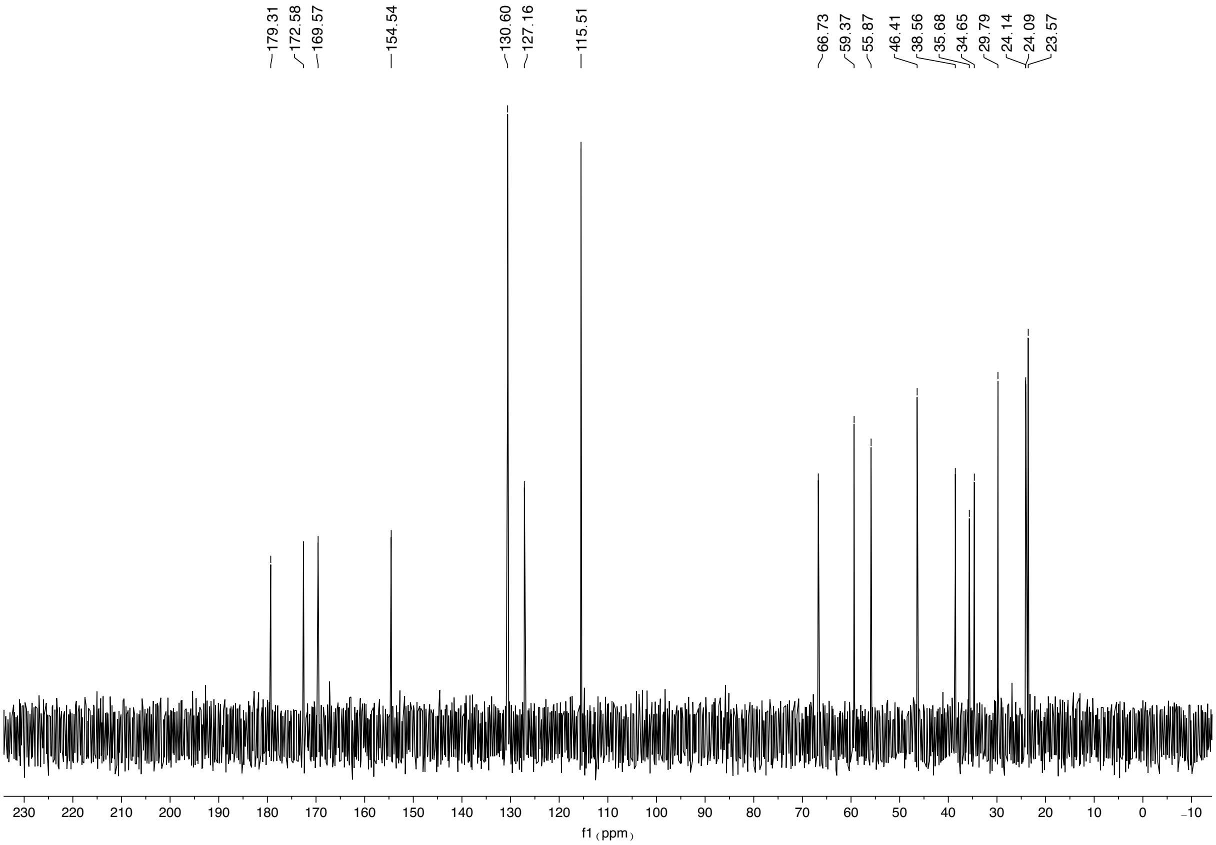

^{1}H and ^{13}C-NMR of H-D-Pro-D-Tyr-Abz-NH_2 · TFA (UTS-153):

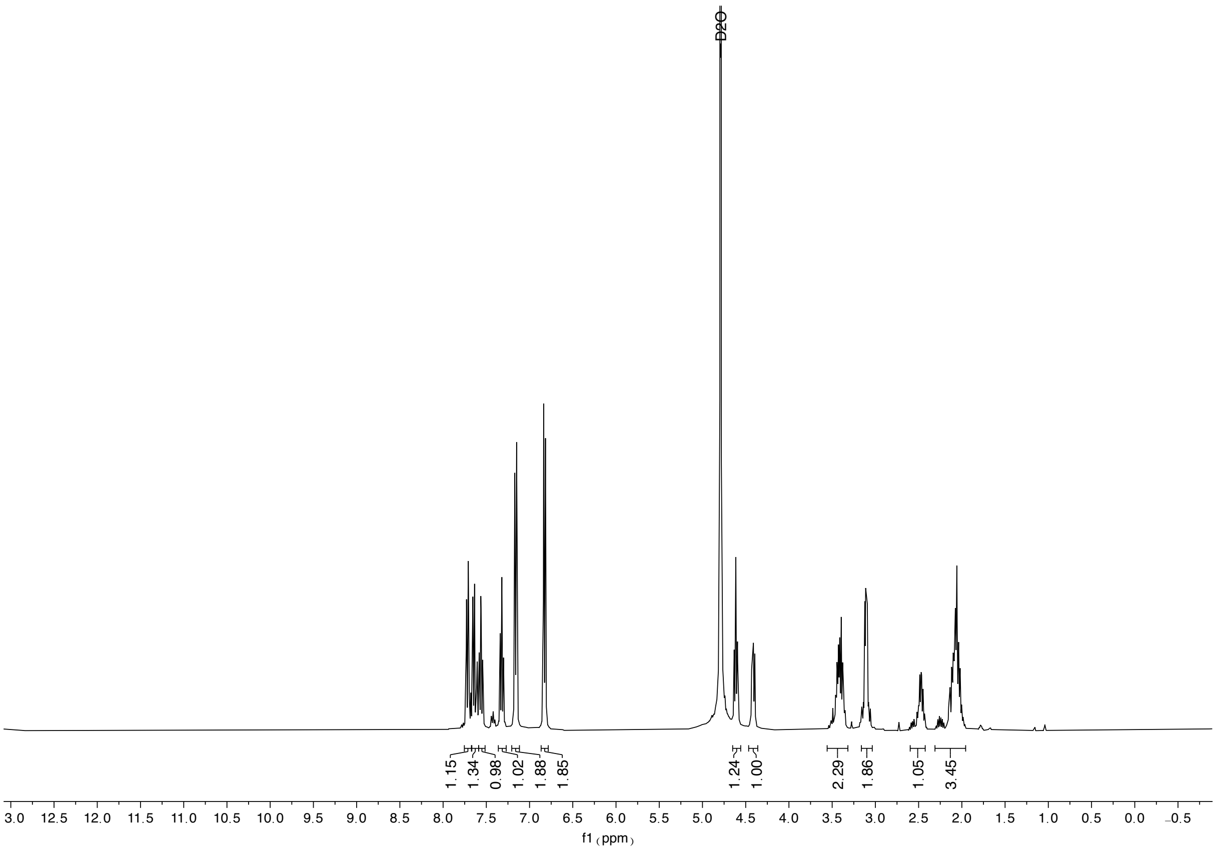

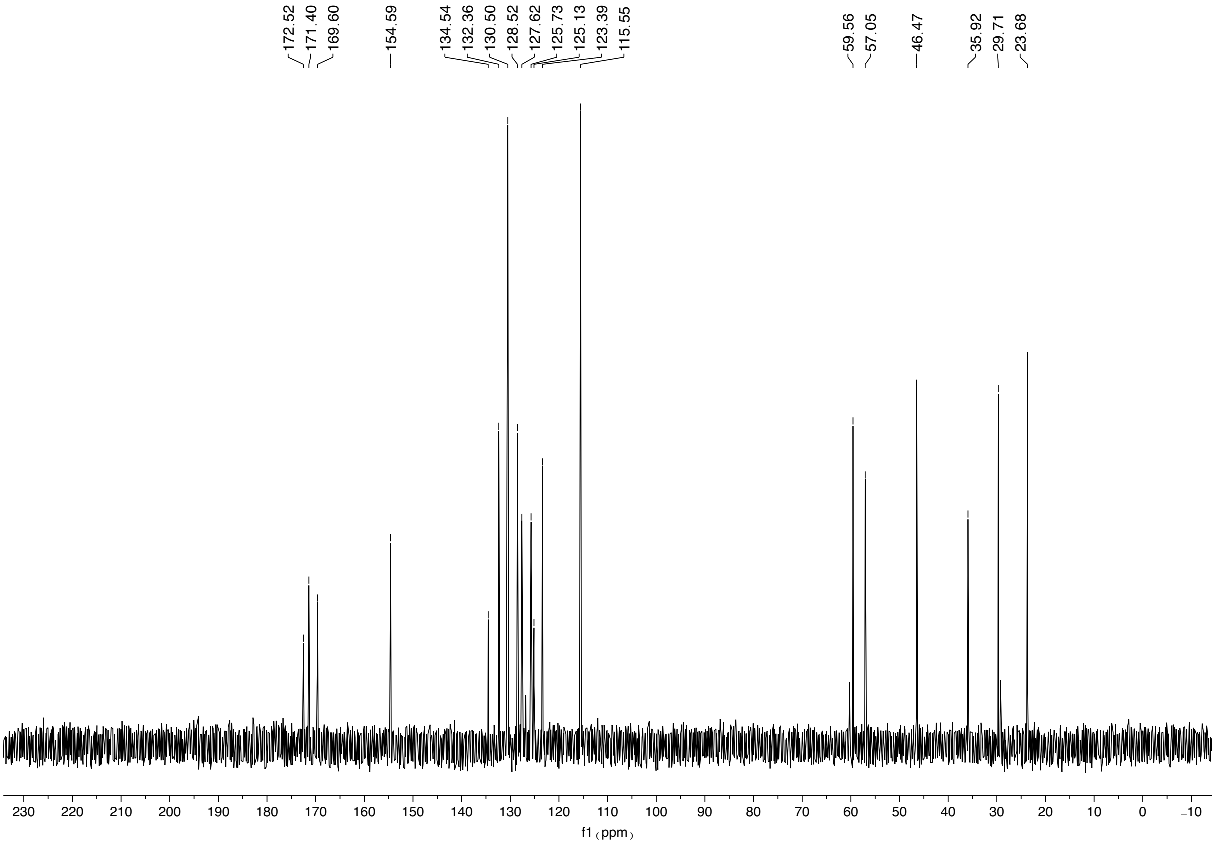

^{1}H and ^{13}C-NMR of H-D-Pro-D-Tyr-D-Ind-NH_2 · TFA (UTS-154):

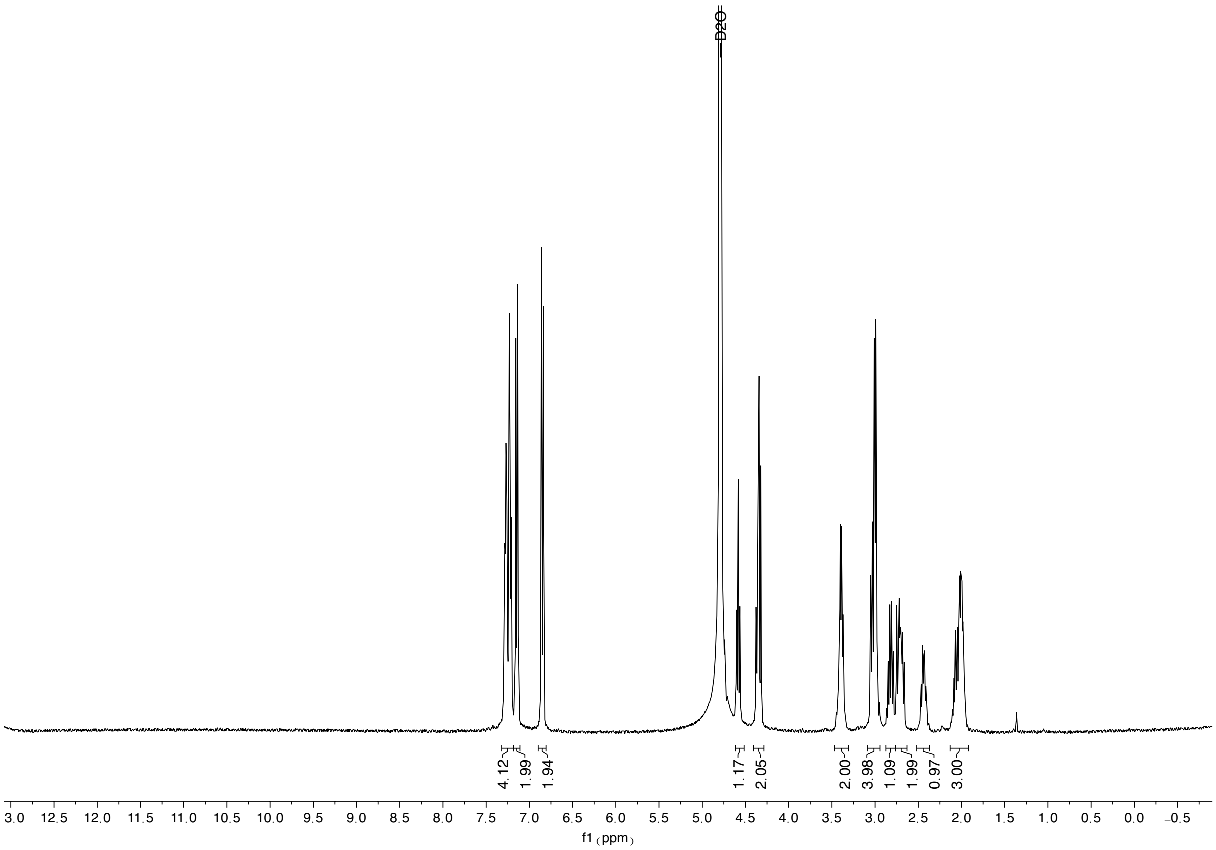

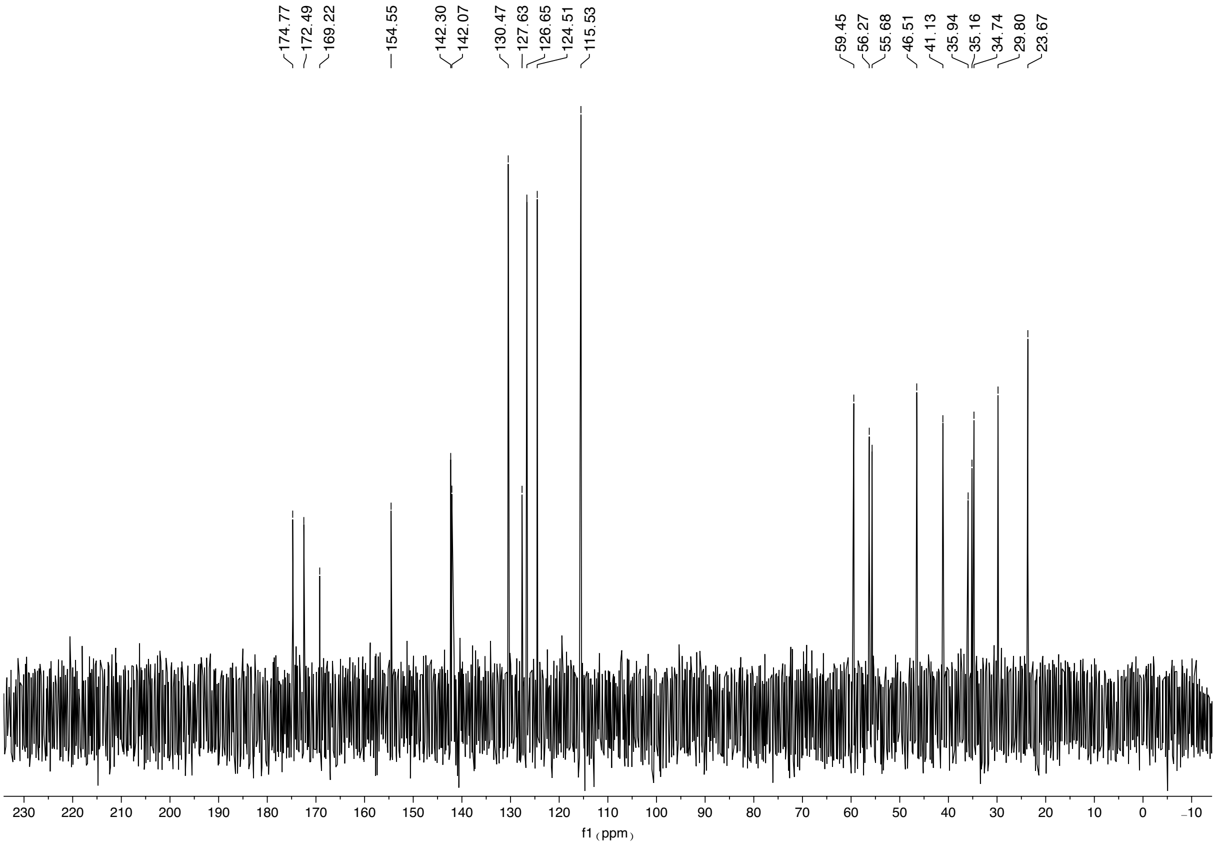

^{1}H and ^{13}C-NMR of H-D-Pro-CyLeu-D-Tyr-NH$_2$ · TFA (UTS-155):

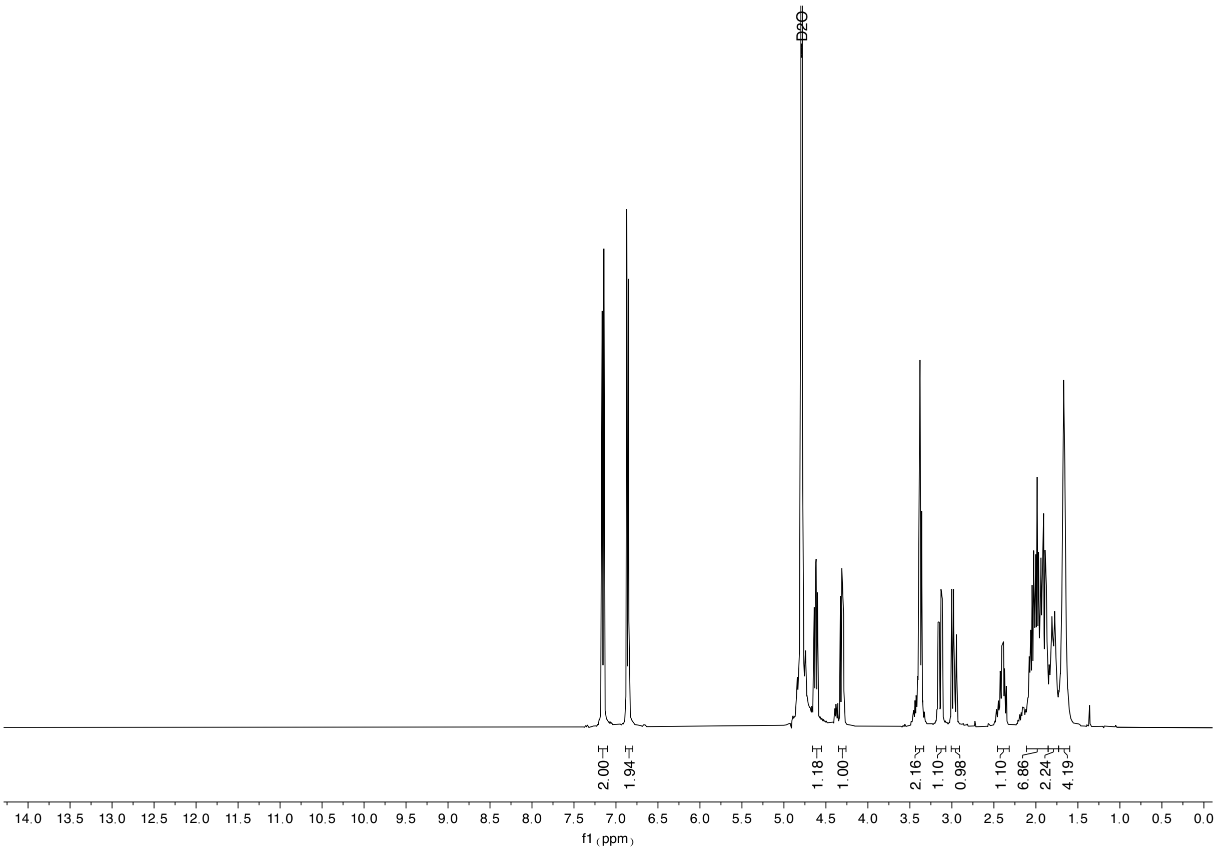

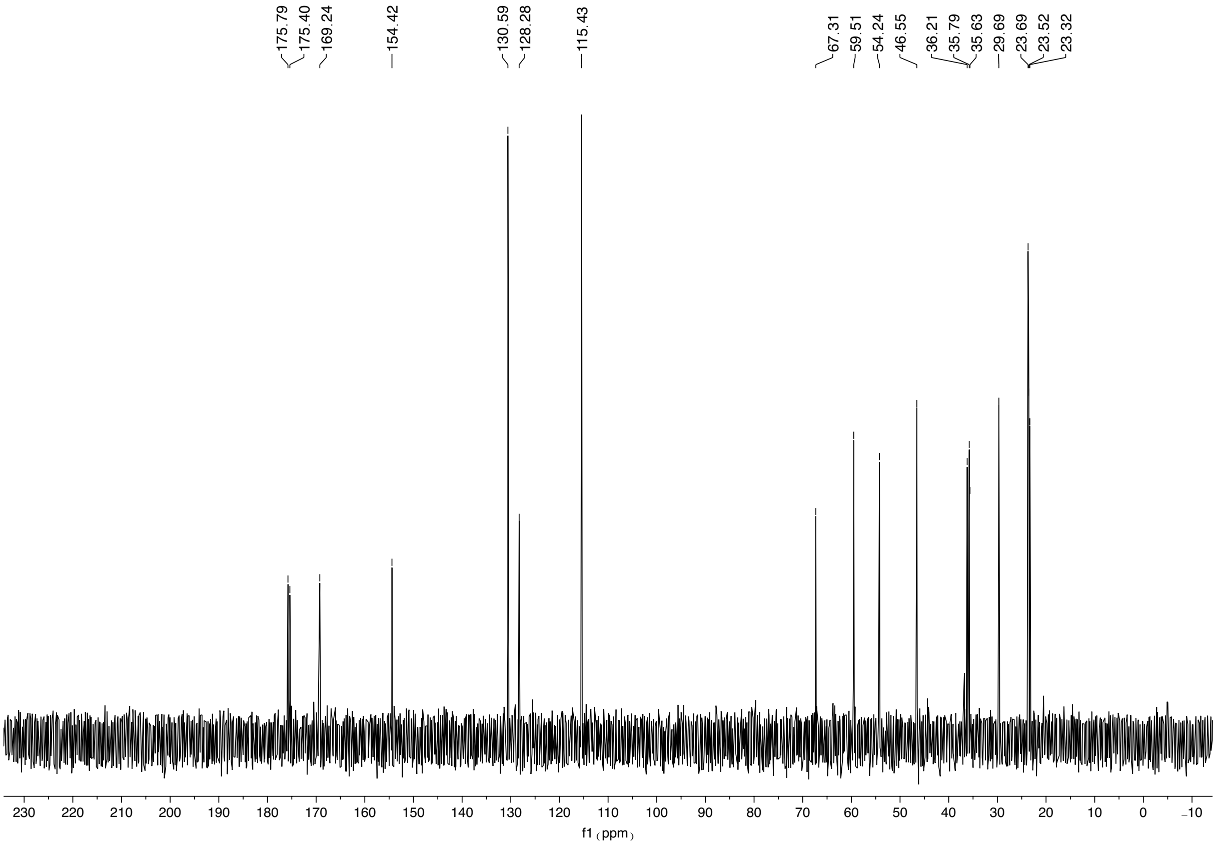

^{1}H and ^{13}C-NMR of H-D-Pro-Abz-D-Tyr-NH$_2$ · TFA (UTS-156):

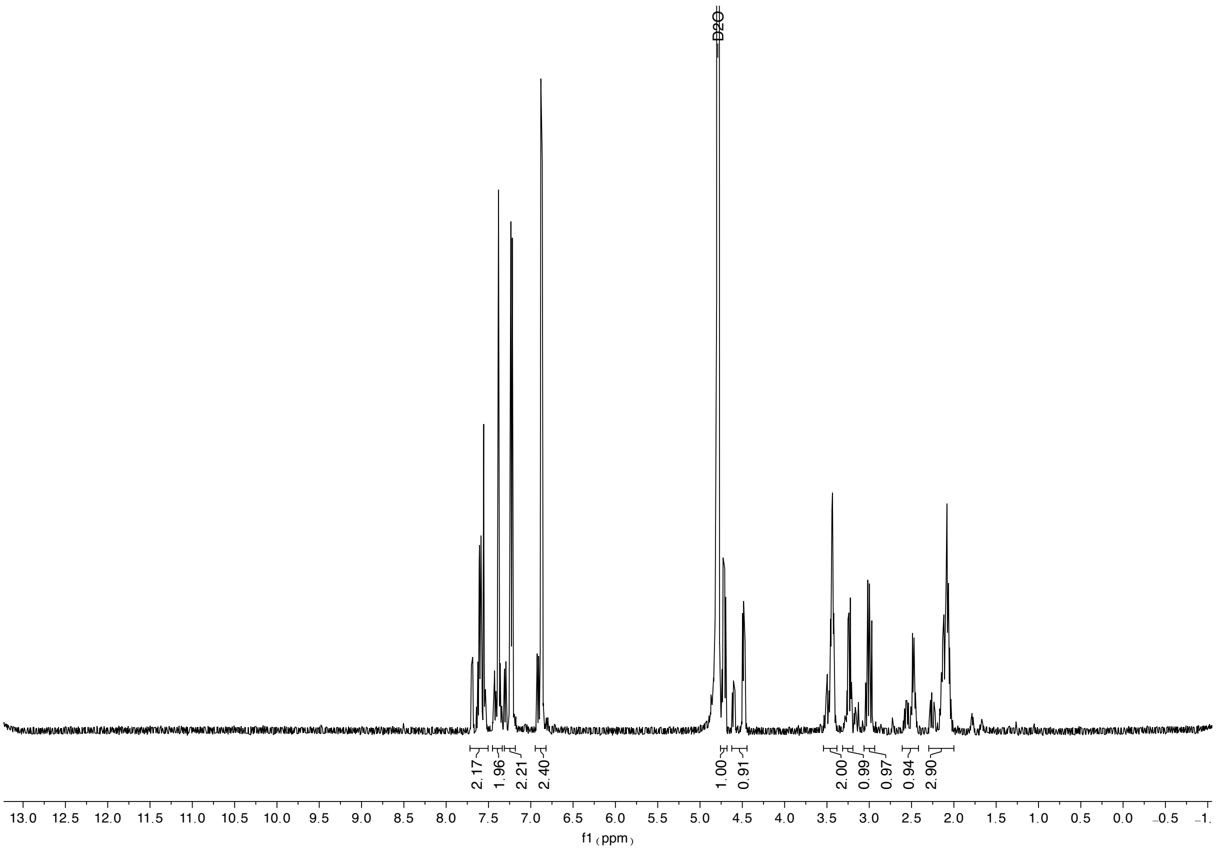

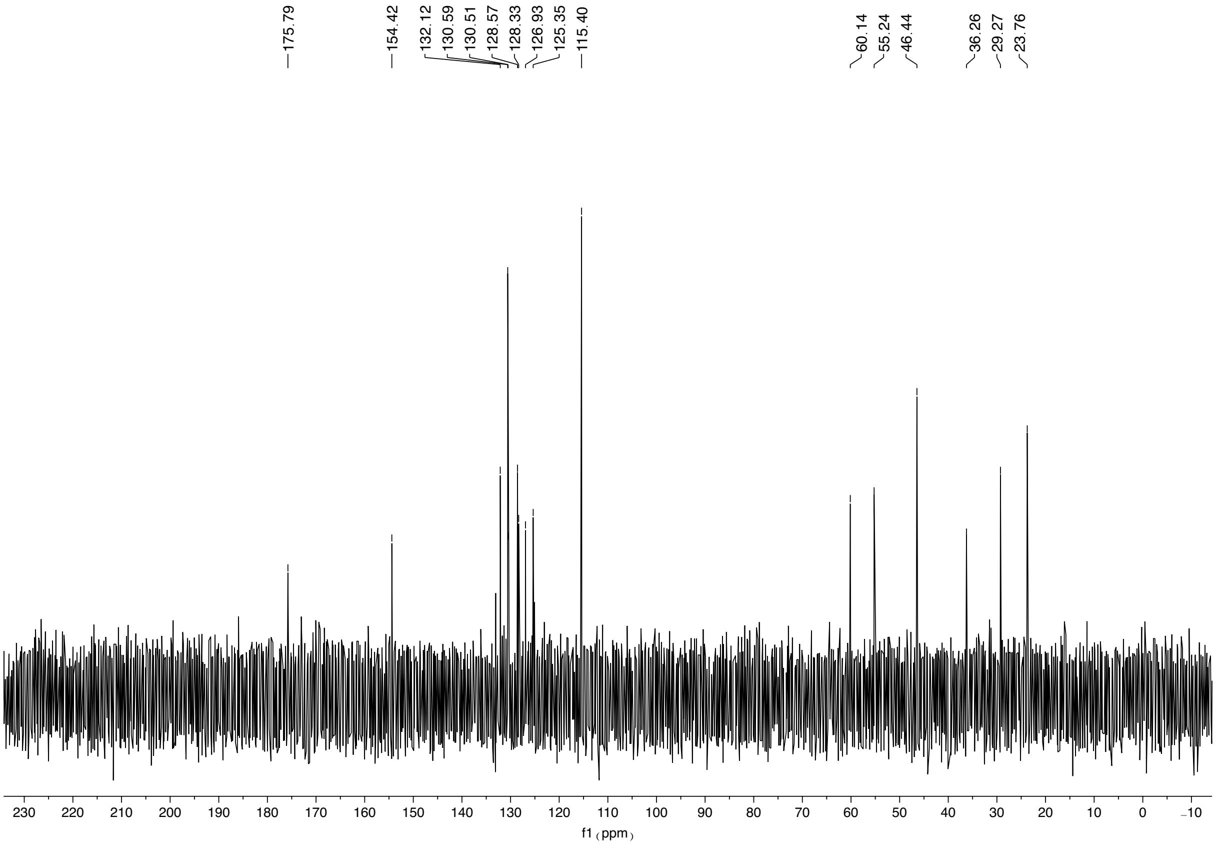

^{1}H and ^{13}C-NMR of H-D-Pro-D-Ind-D-Tyr-NH_2 · TFA (UTS-157):

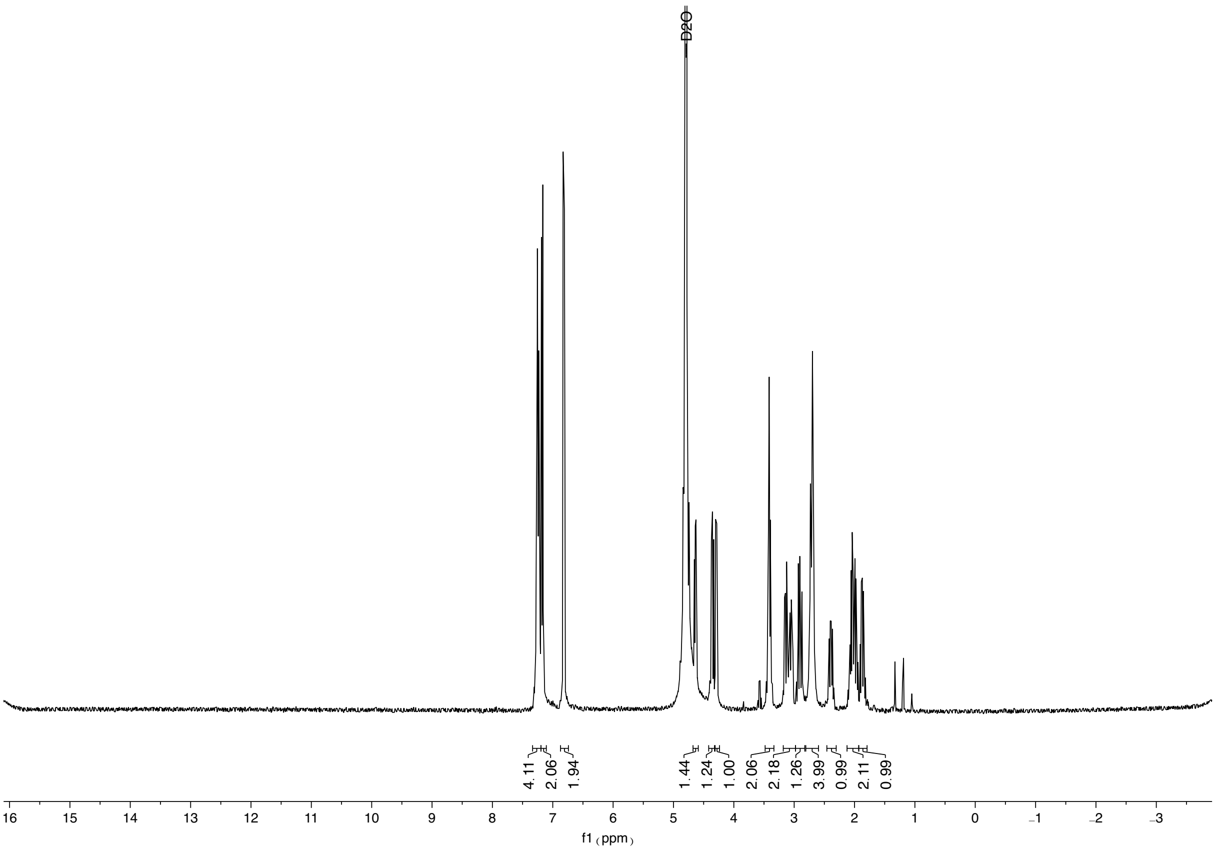

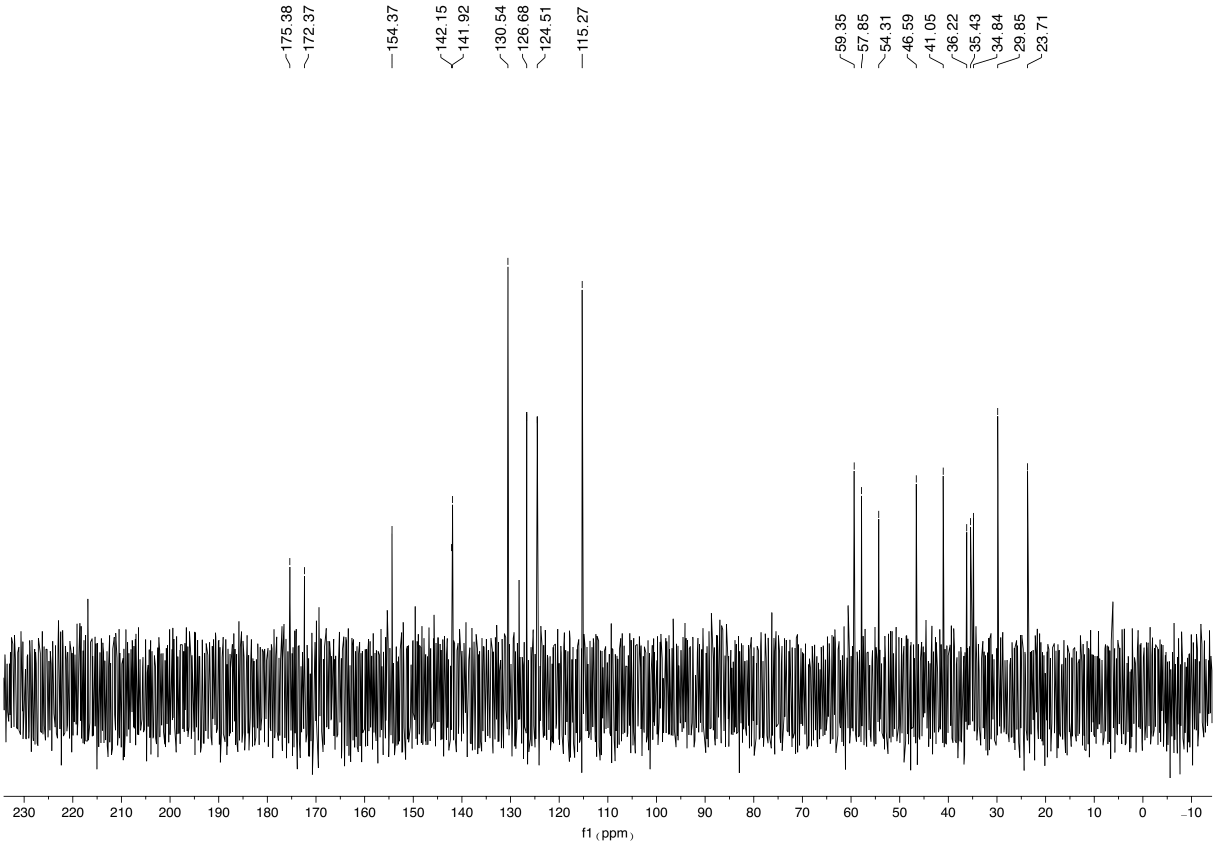

^{1}H and ^{13}C-NMR of H-D-Pro-CyLeu-CyLeu-NH_2 · TFA (UTS-158):

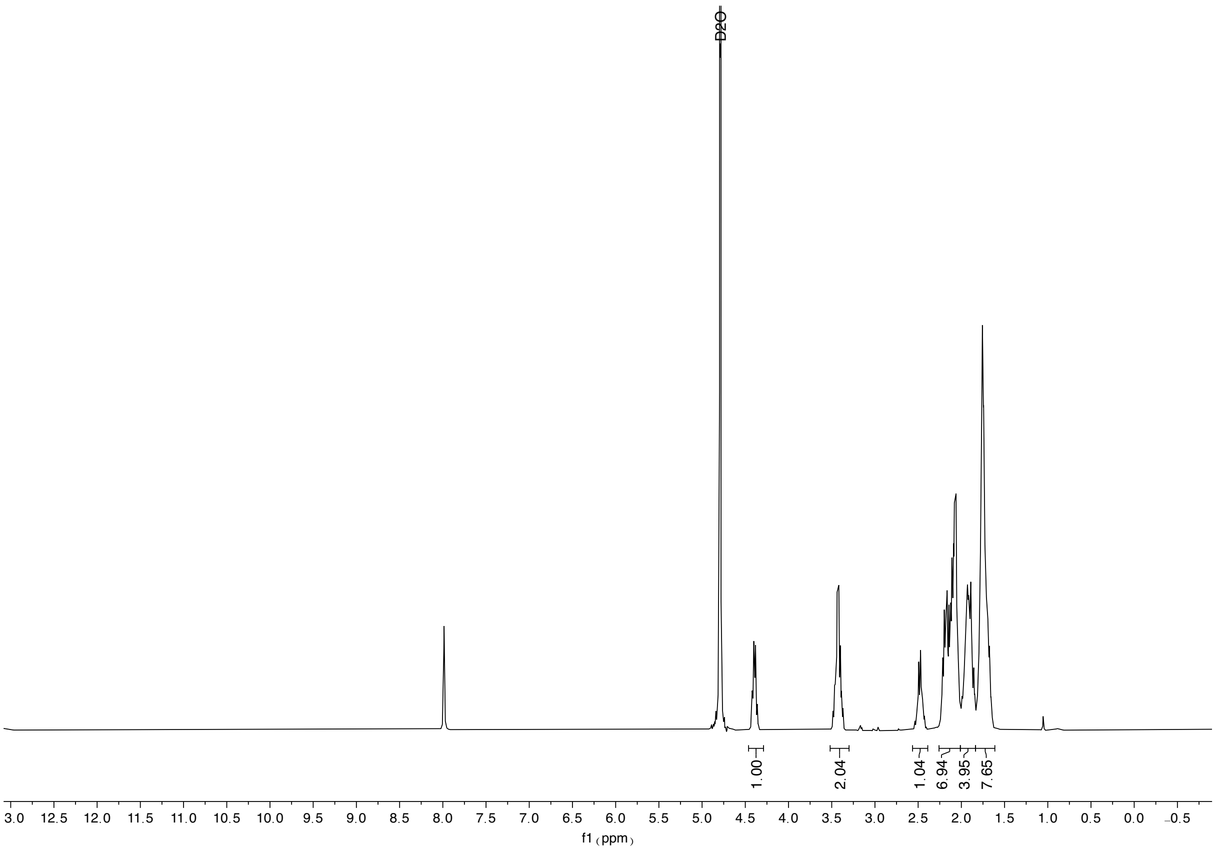

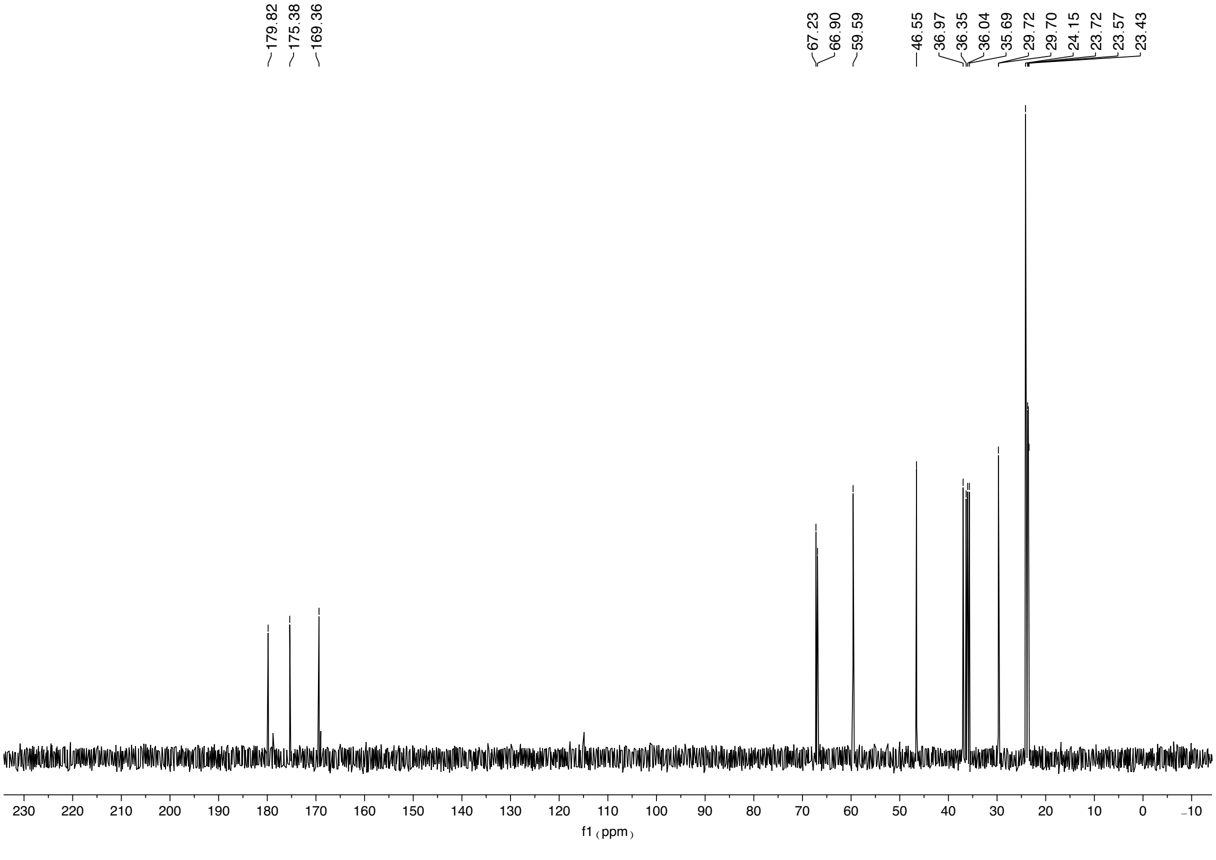

^{1}H and ^{13}C-NMR of H-D-Pro-CyLeu-D-Ind-NH₂ · TFA (UTS-159):

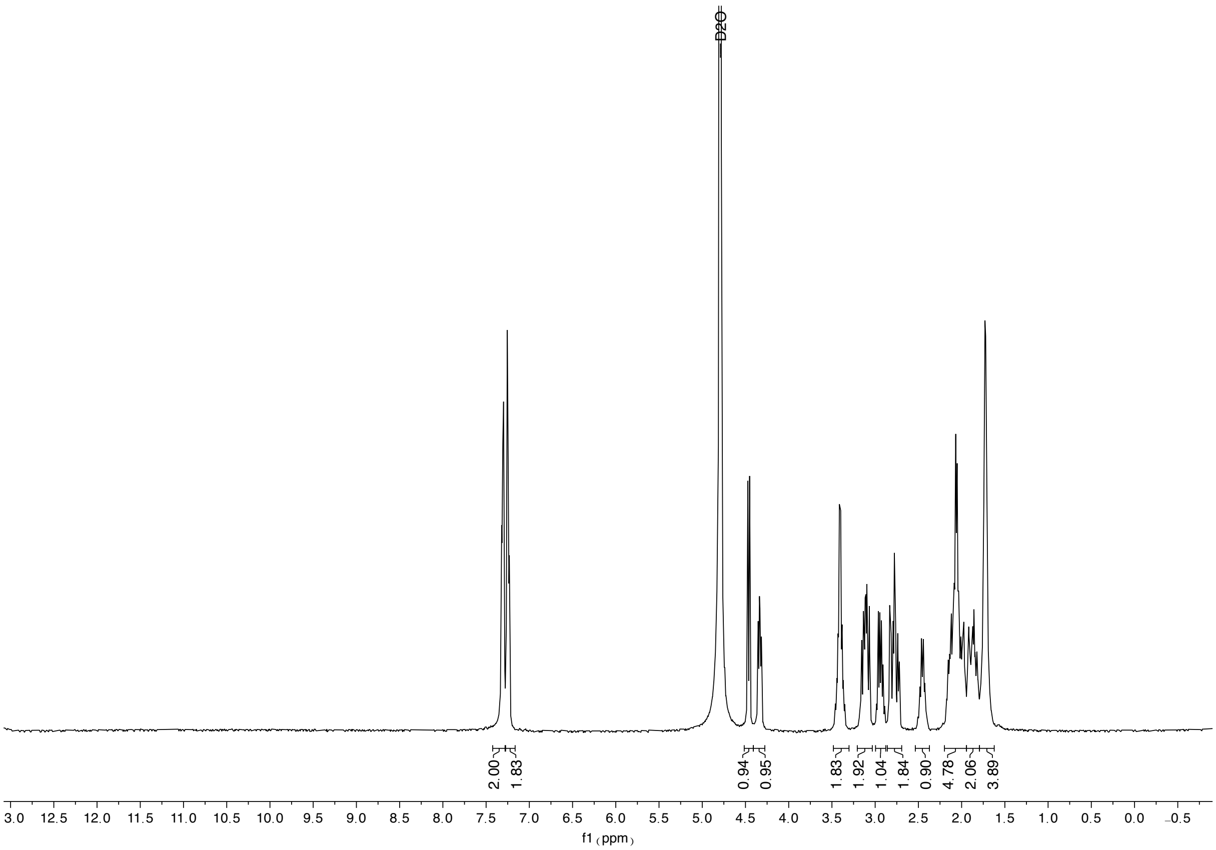

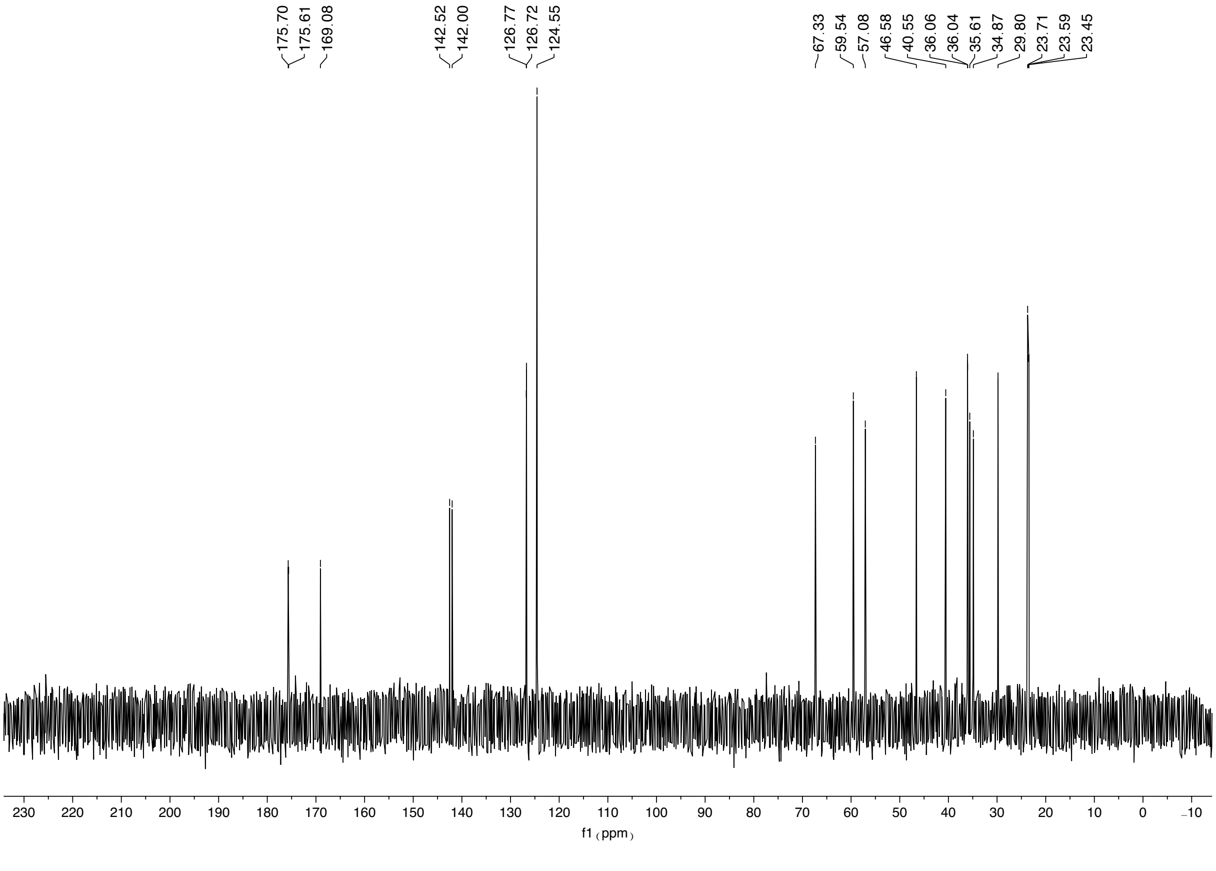

^{1}H and ^{13}C-NMR of H-D-Pro-D-Ind-CyLeu-NH$_2$ · TFA (UTS-160):

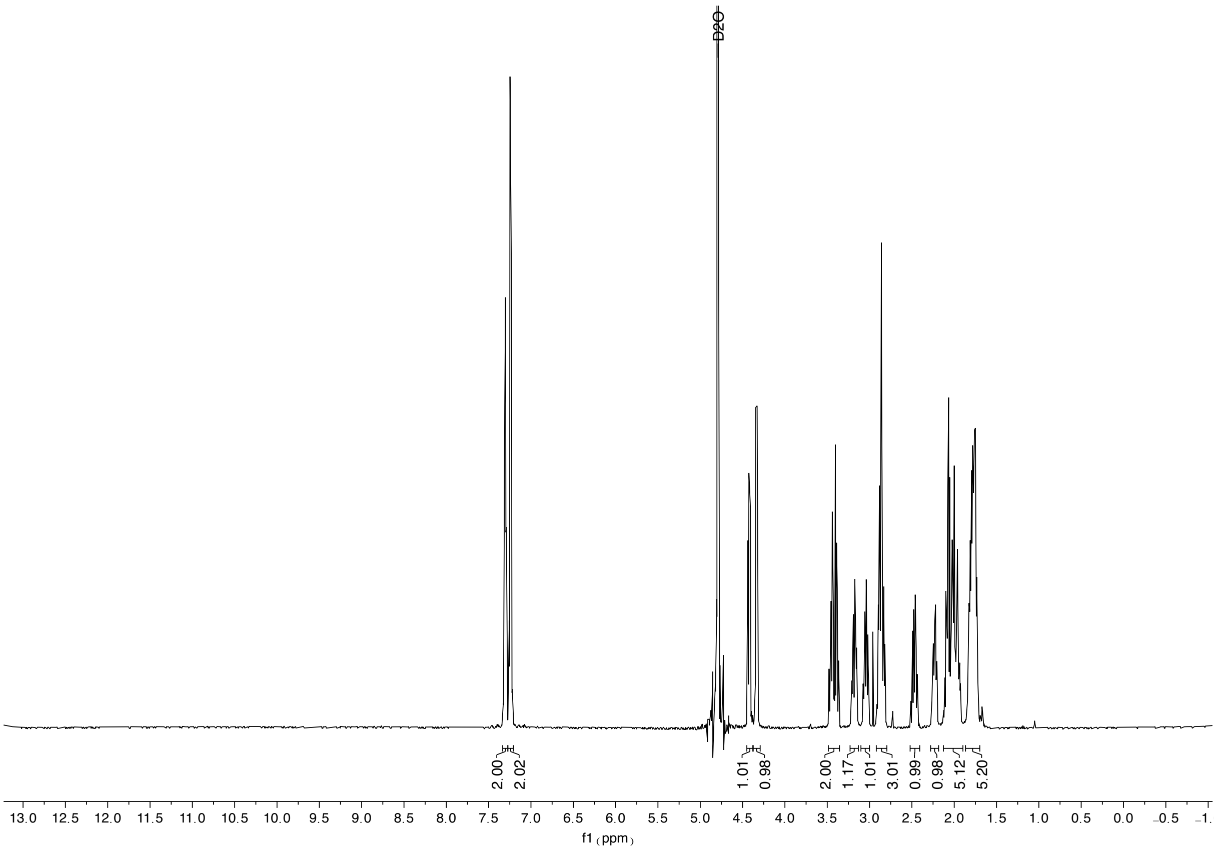

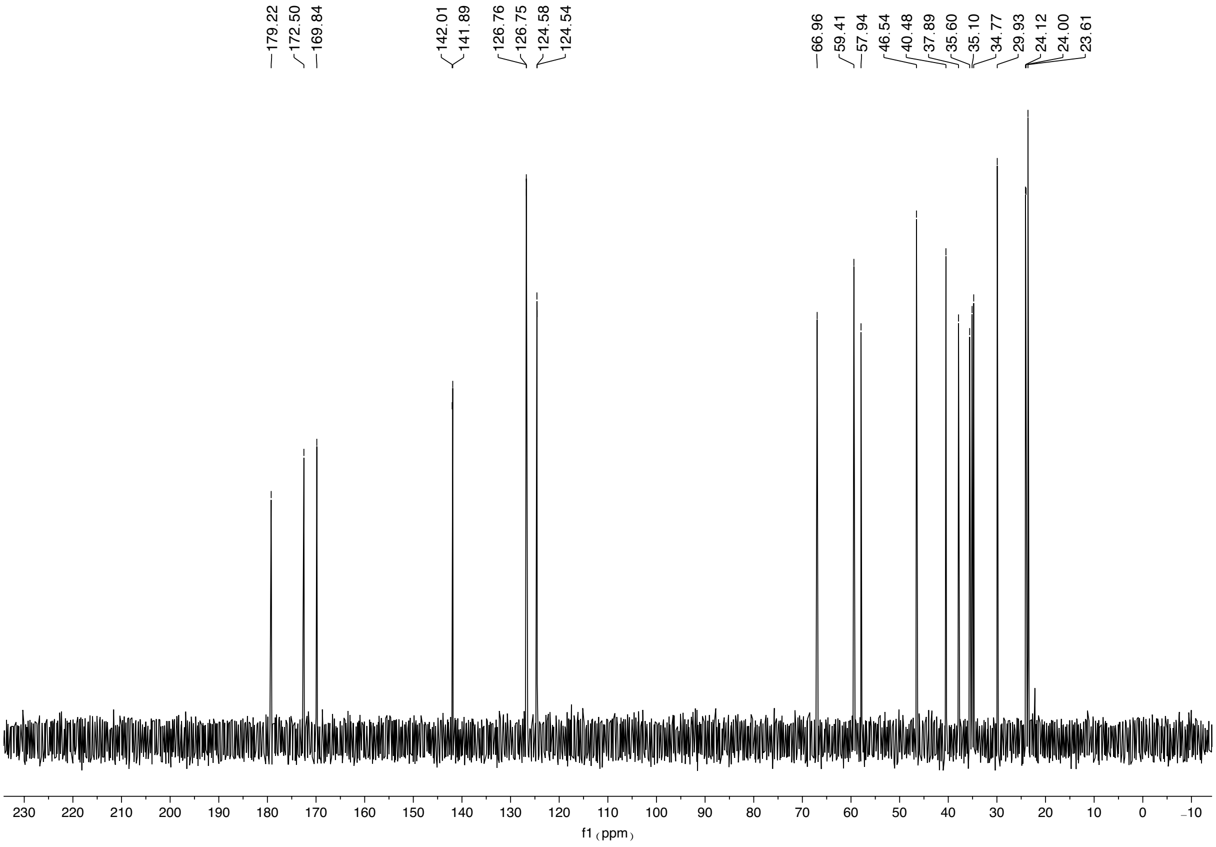

^{1}H and ^{13}C-NMR of H-D-Pro-D-Ind-D-Ind-NH_2 · TFA (UTS-161):

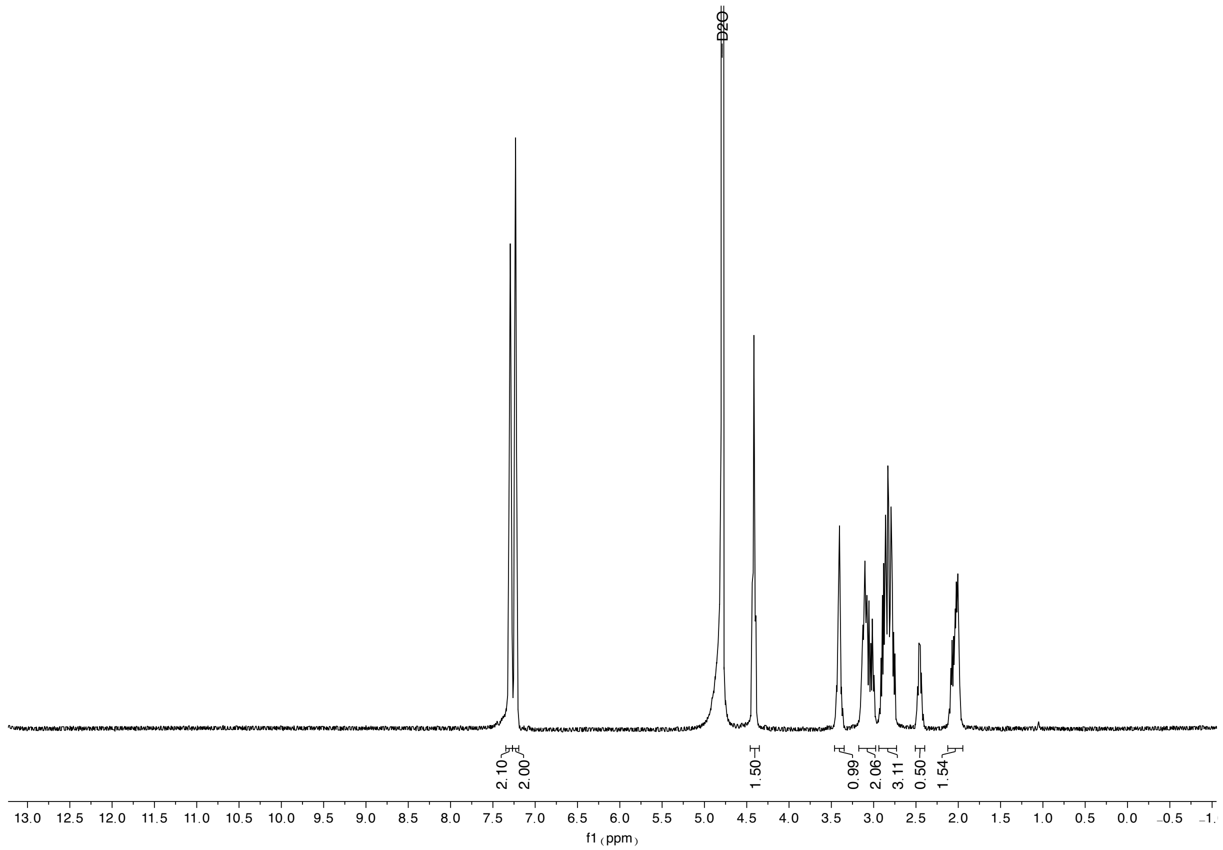

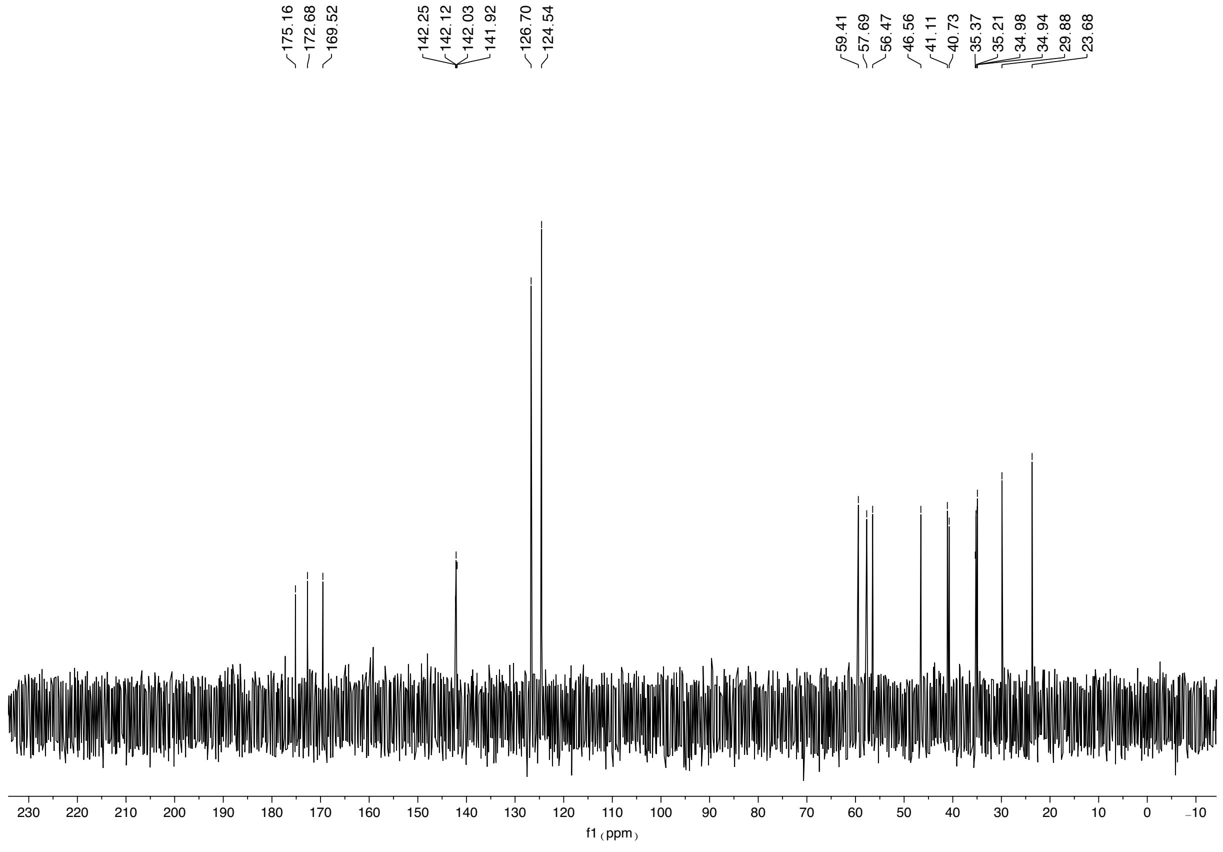

^{1}H and ^{13}C-NMR of H-D-Pro-D-Pyr-4-MePhe-NH₂ · TFA (P(1)-1):

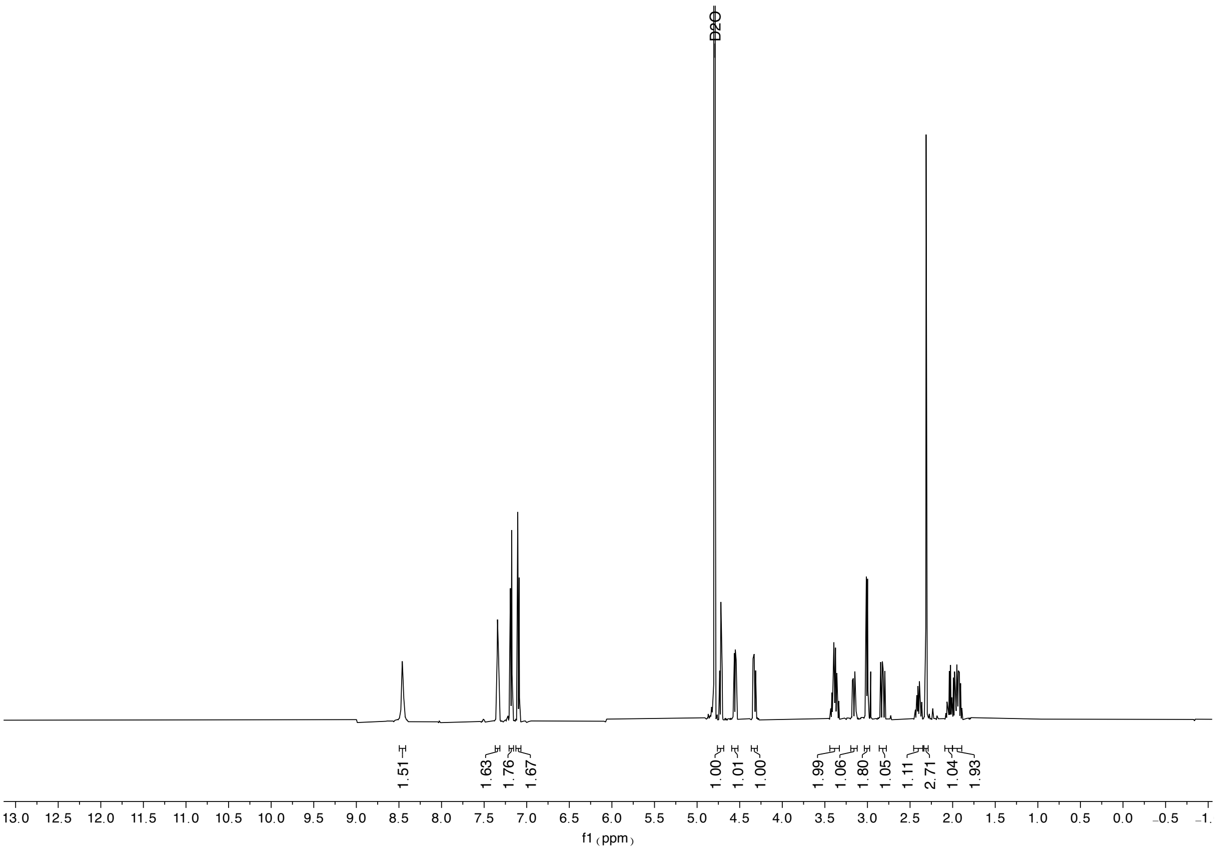

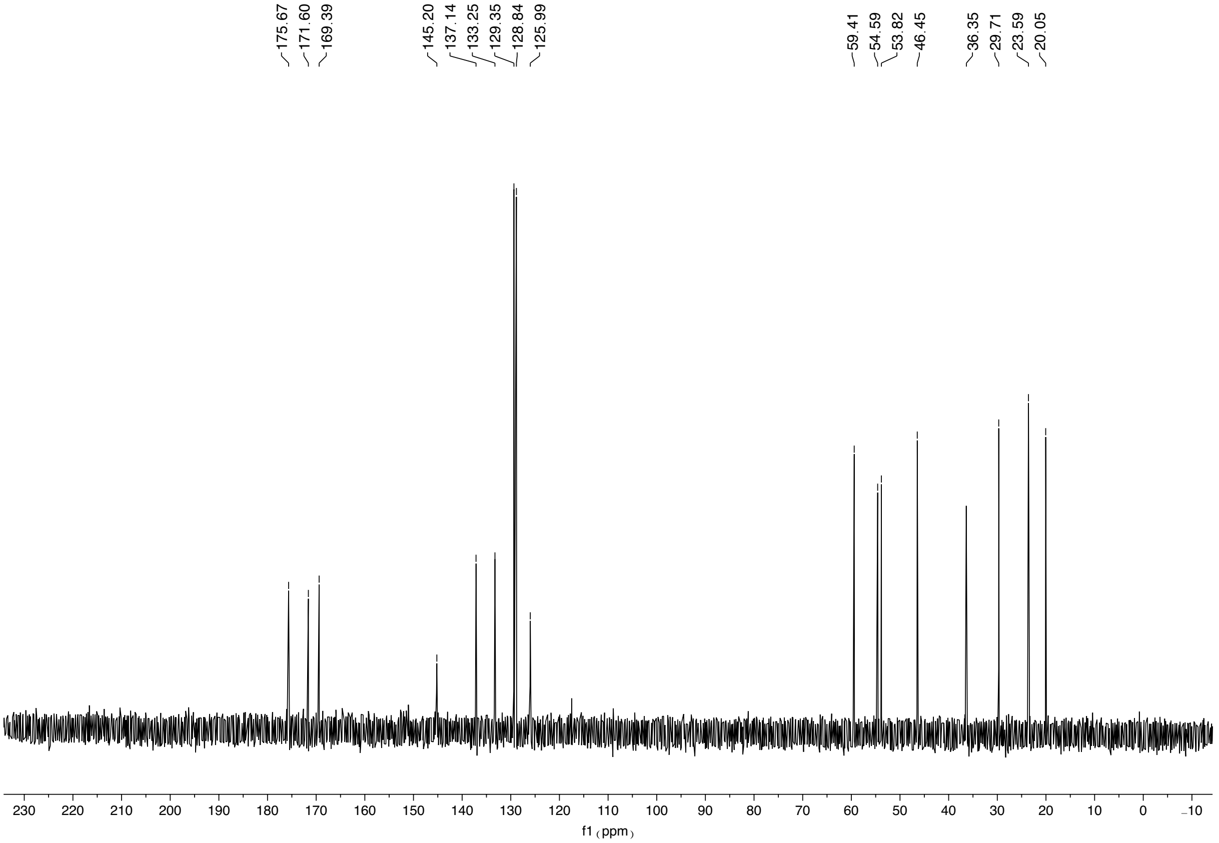

^{1}H and ^{13}C-NMR of H-D-Pro-L-Aze-L-Naph-NH$_2$ · TFA (P(1)-2):

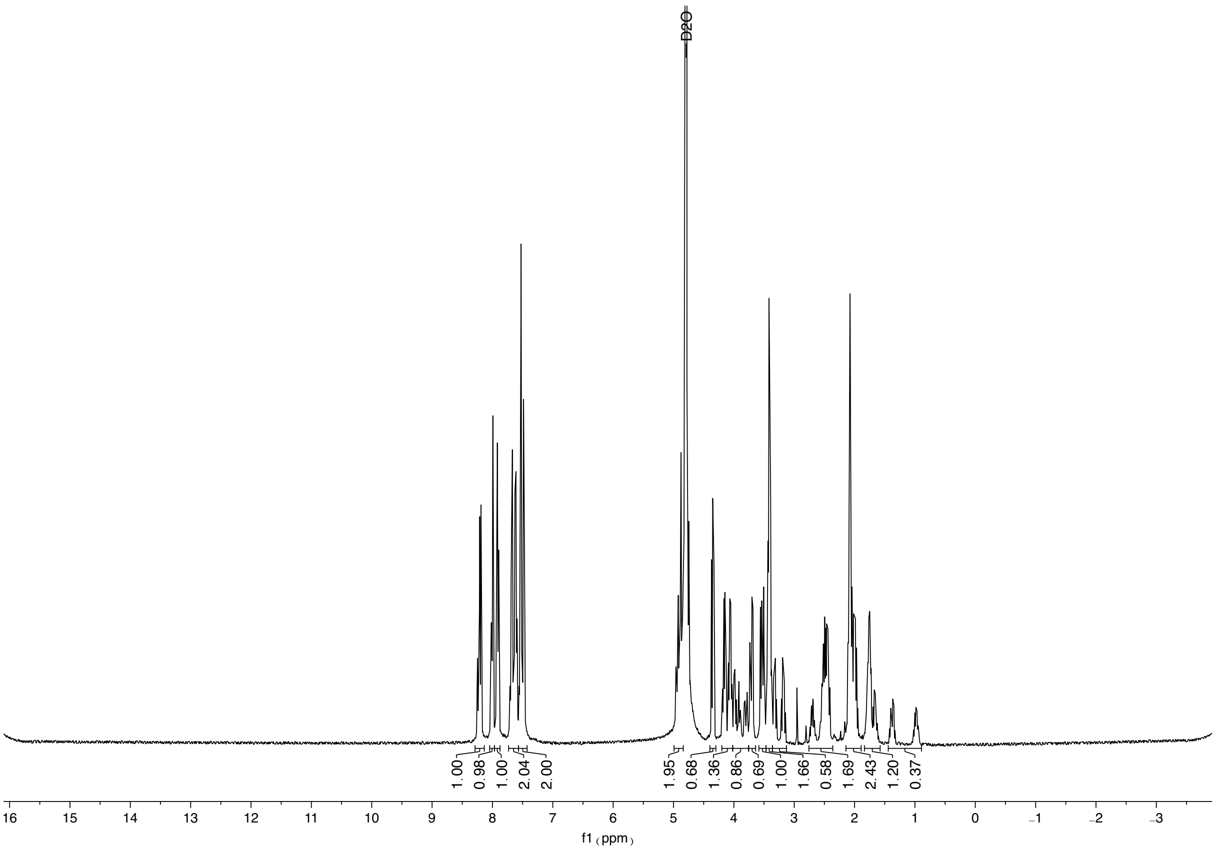

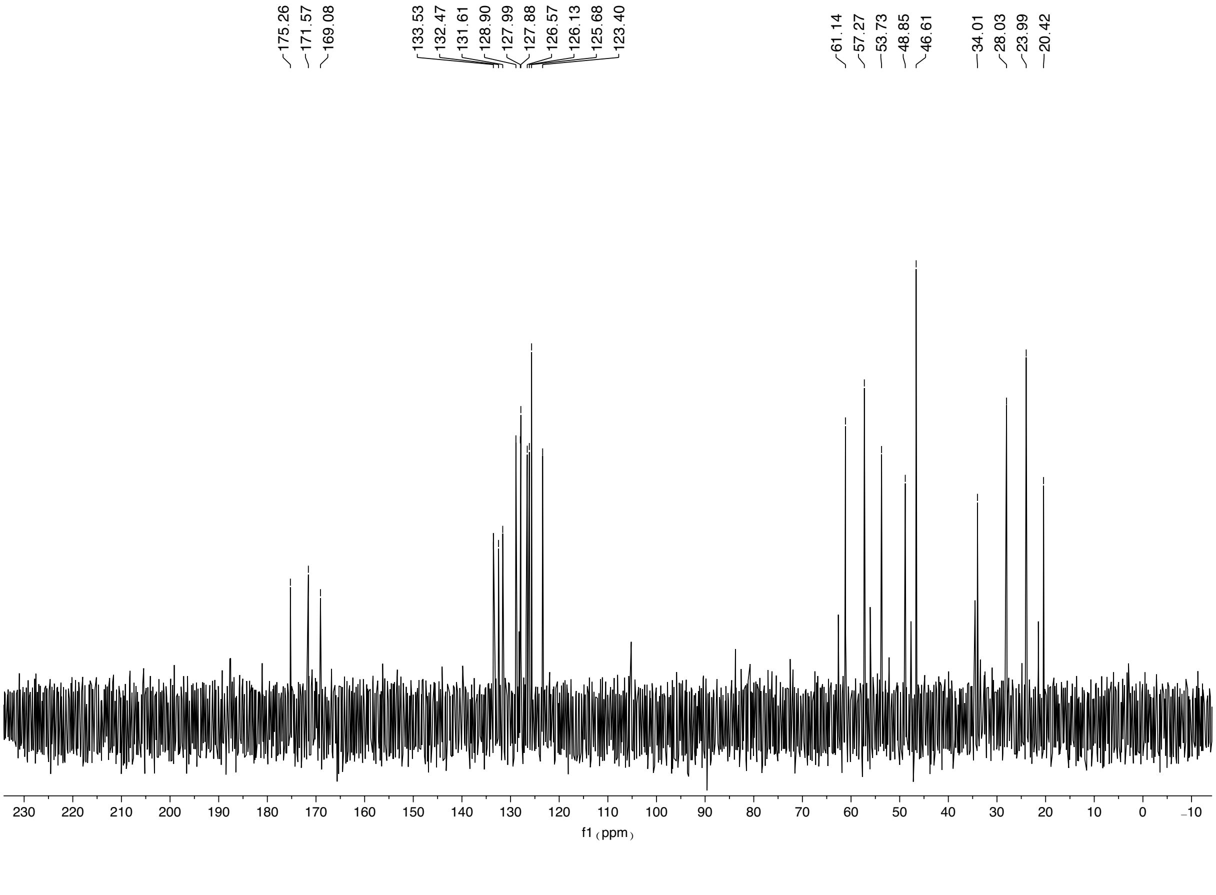

^{1}H and ^{13}C-NMR of H-D-Pro-D-cisHyd-D-Pyr-NH2 · TFA (P(1)-3):

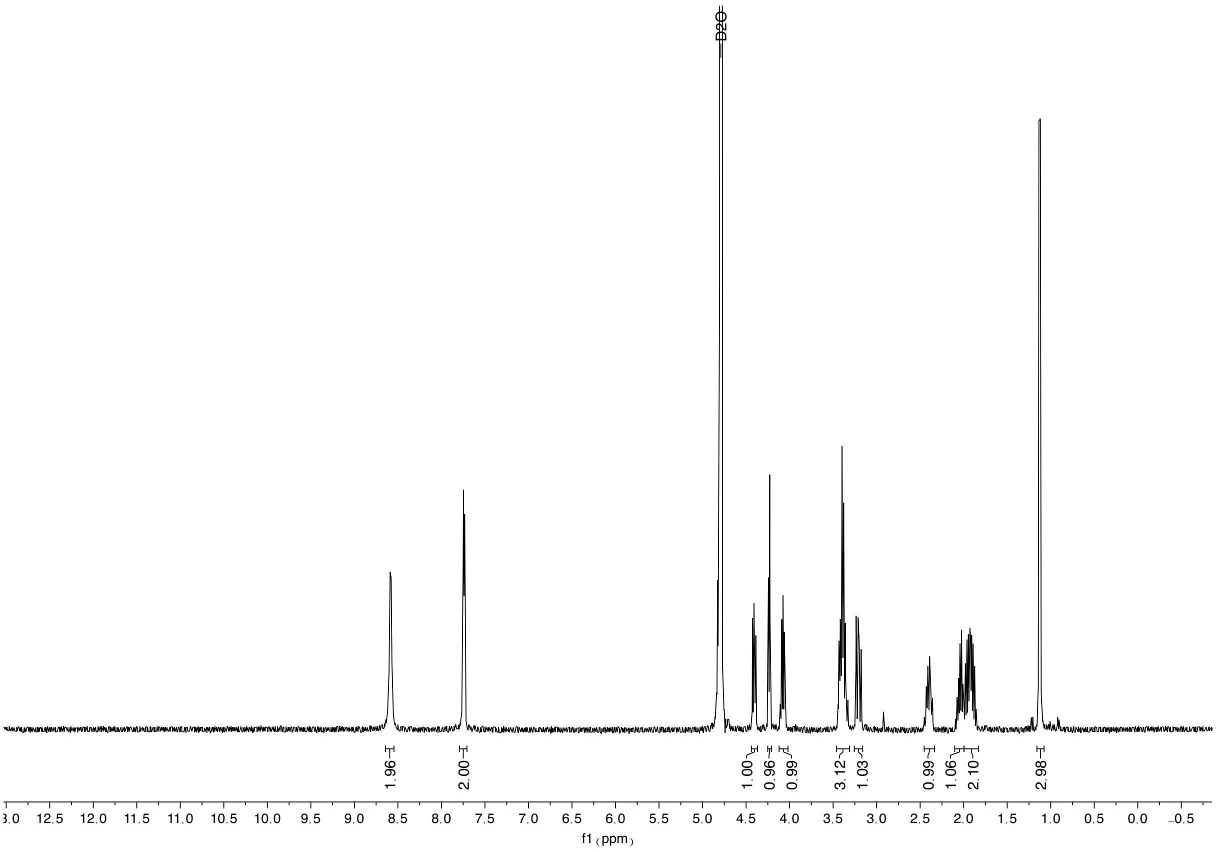

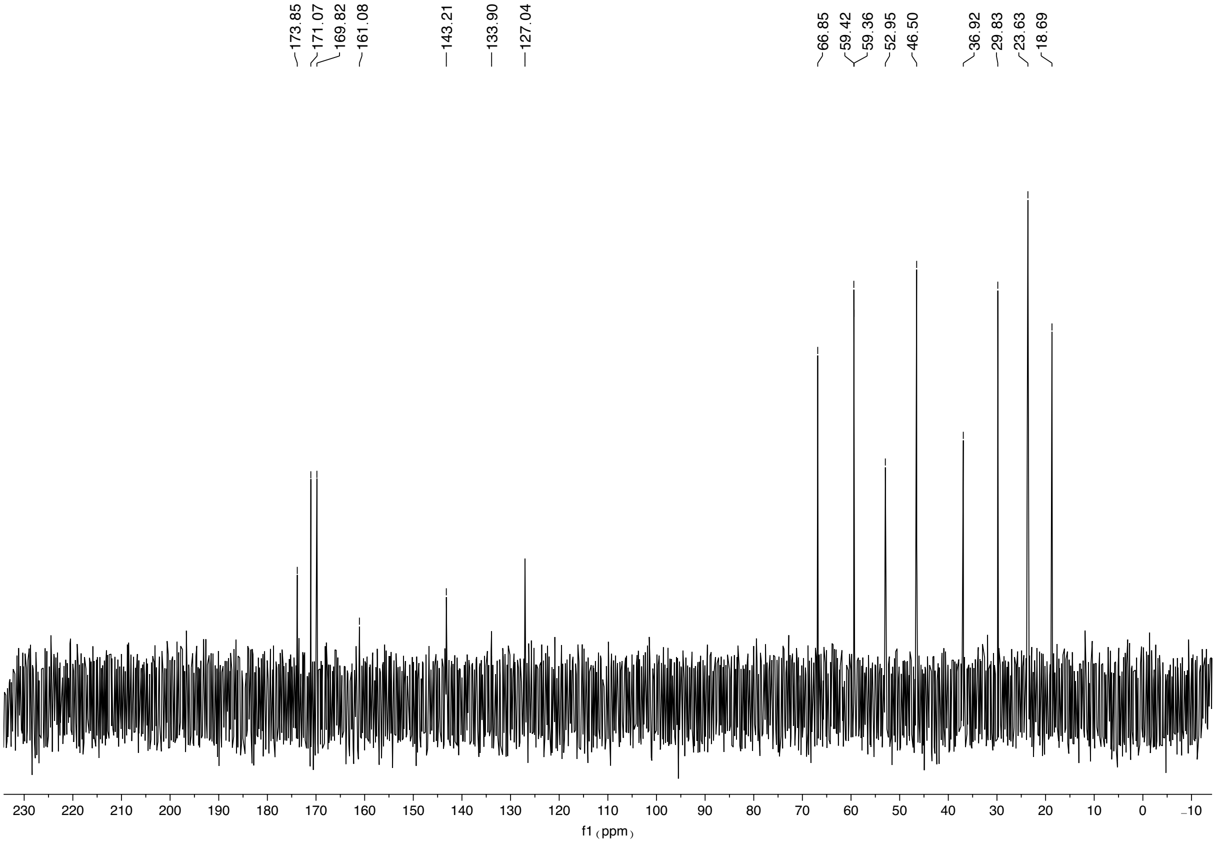

^{1}H and ^{13}C-NMR of H-D-Pro-L-αMePro-L-HomoGlu-NH_2 · TFA (P(1)-4):

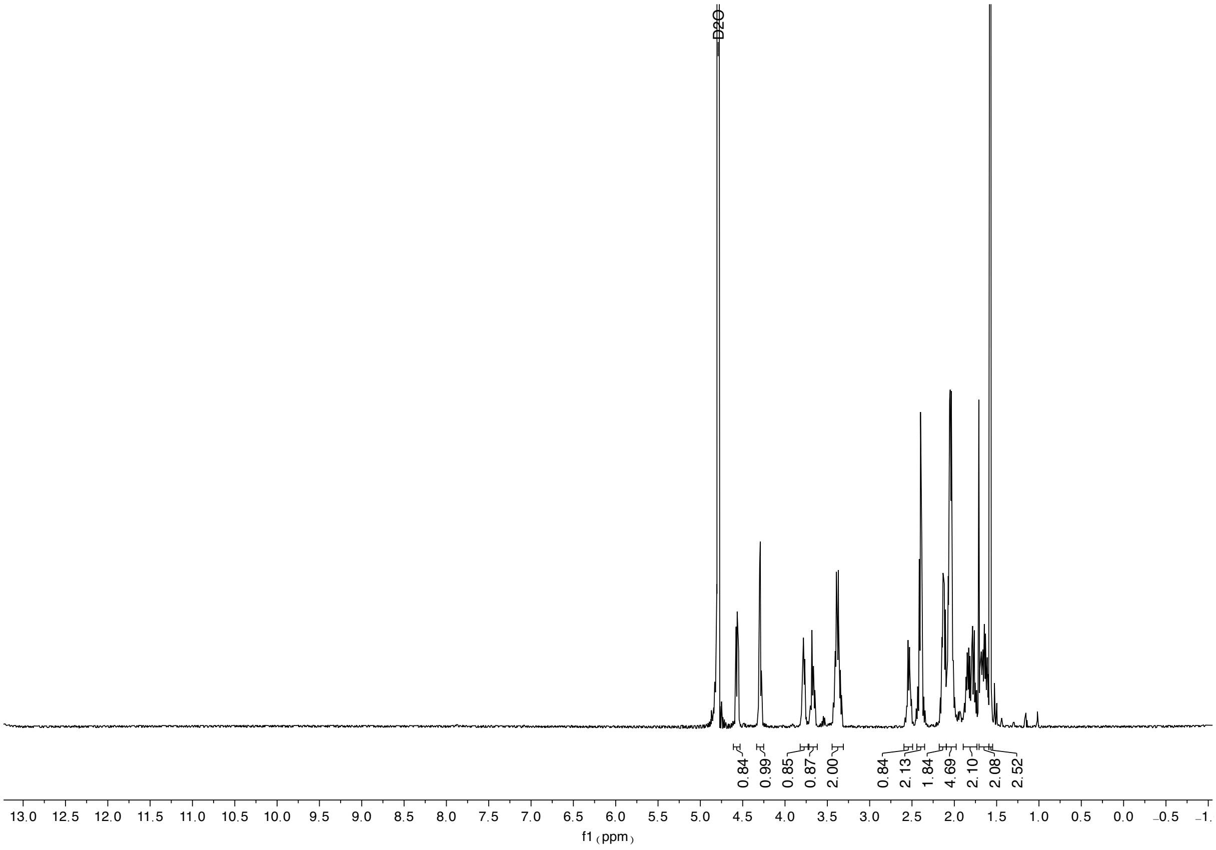

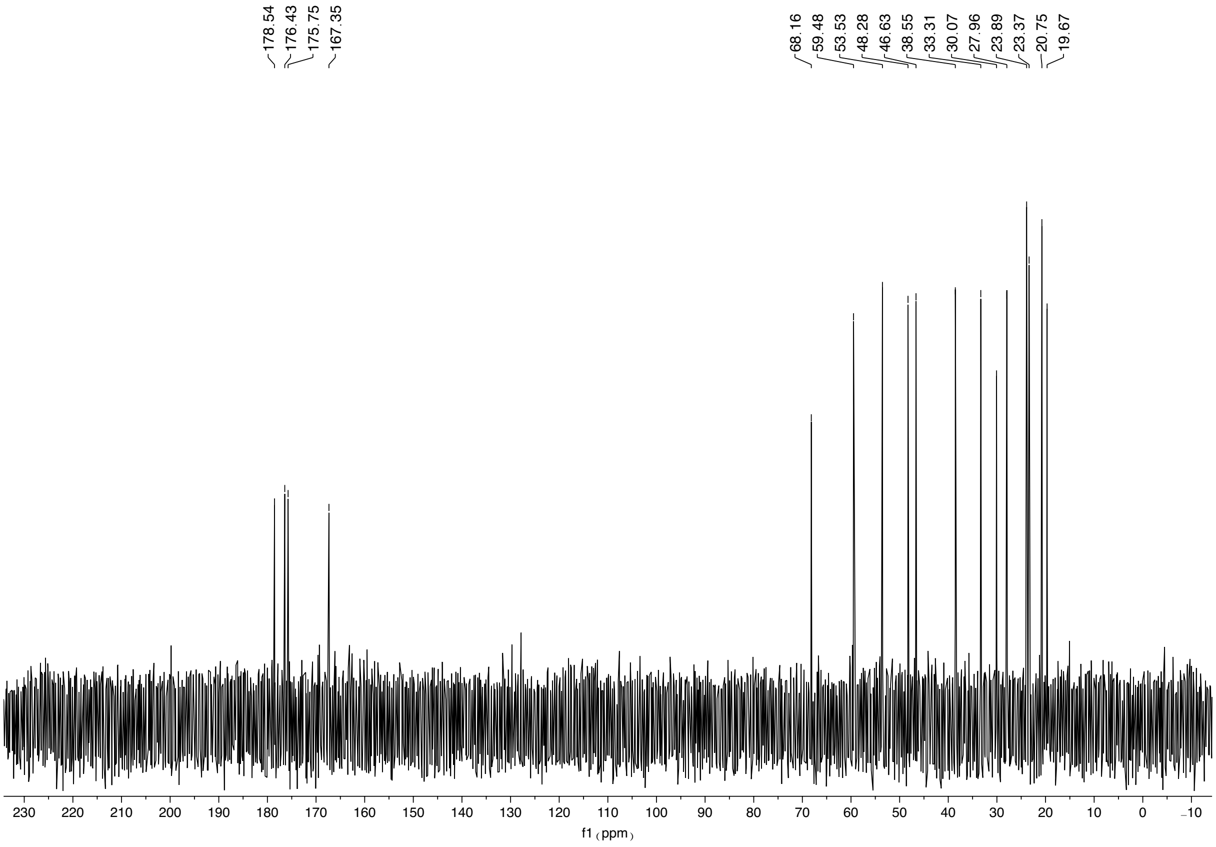

^{1}H and ^{13}C-NMR of H-D-Pro-D-Pro-L-Styr-NH$_2$ · TFA (P(1)-5):

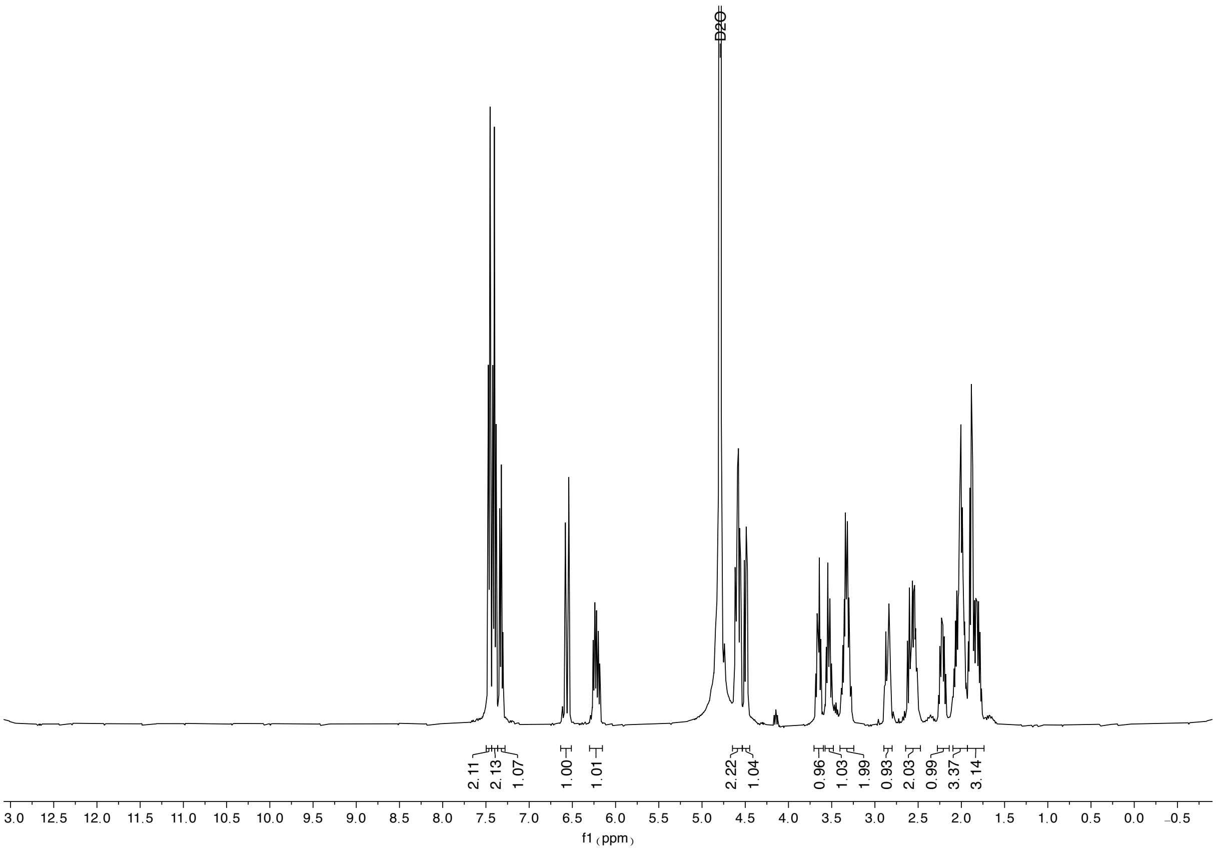

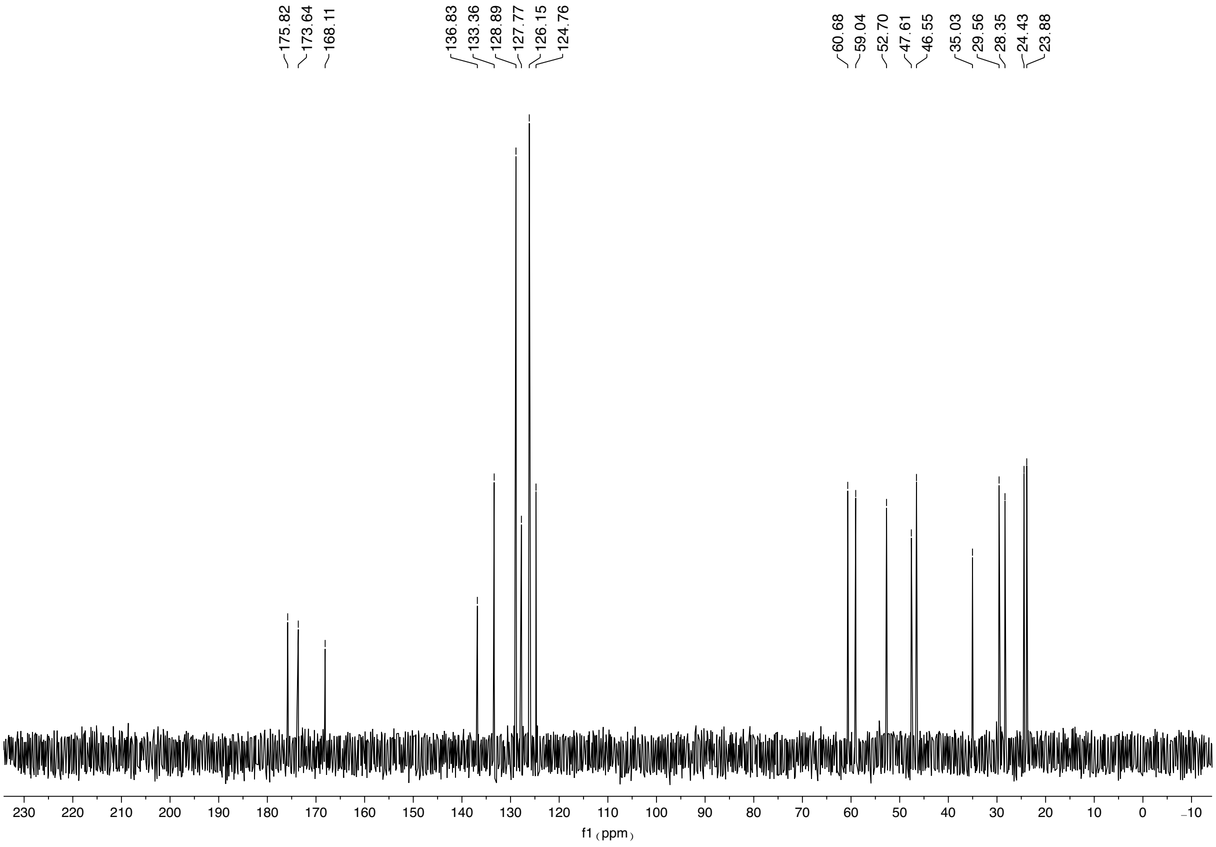

^{1}H and ^{13}C-NMR of H-D-Pro-HomoPip-D-Arg-NH2 · TFA (P(1)-6):

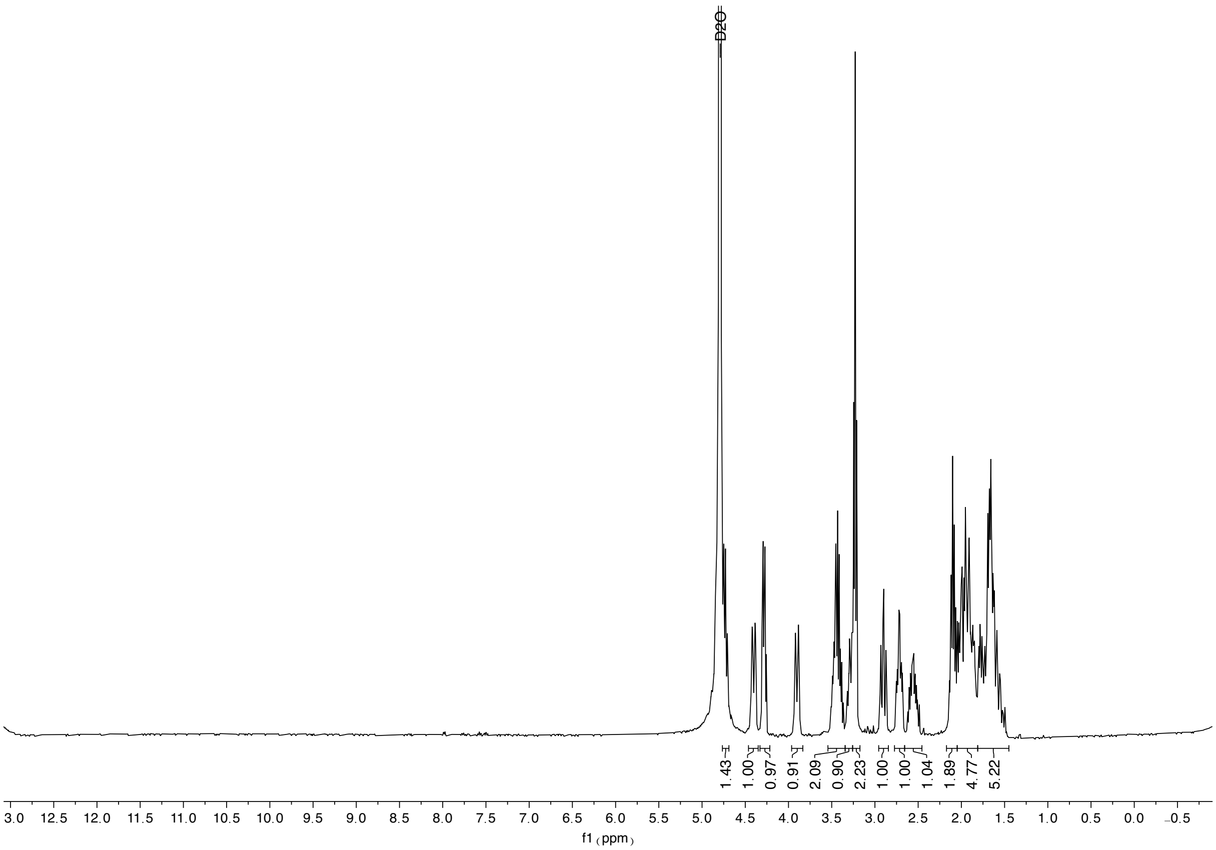

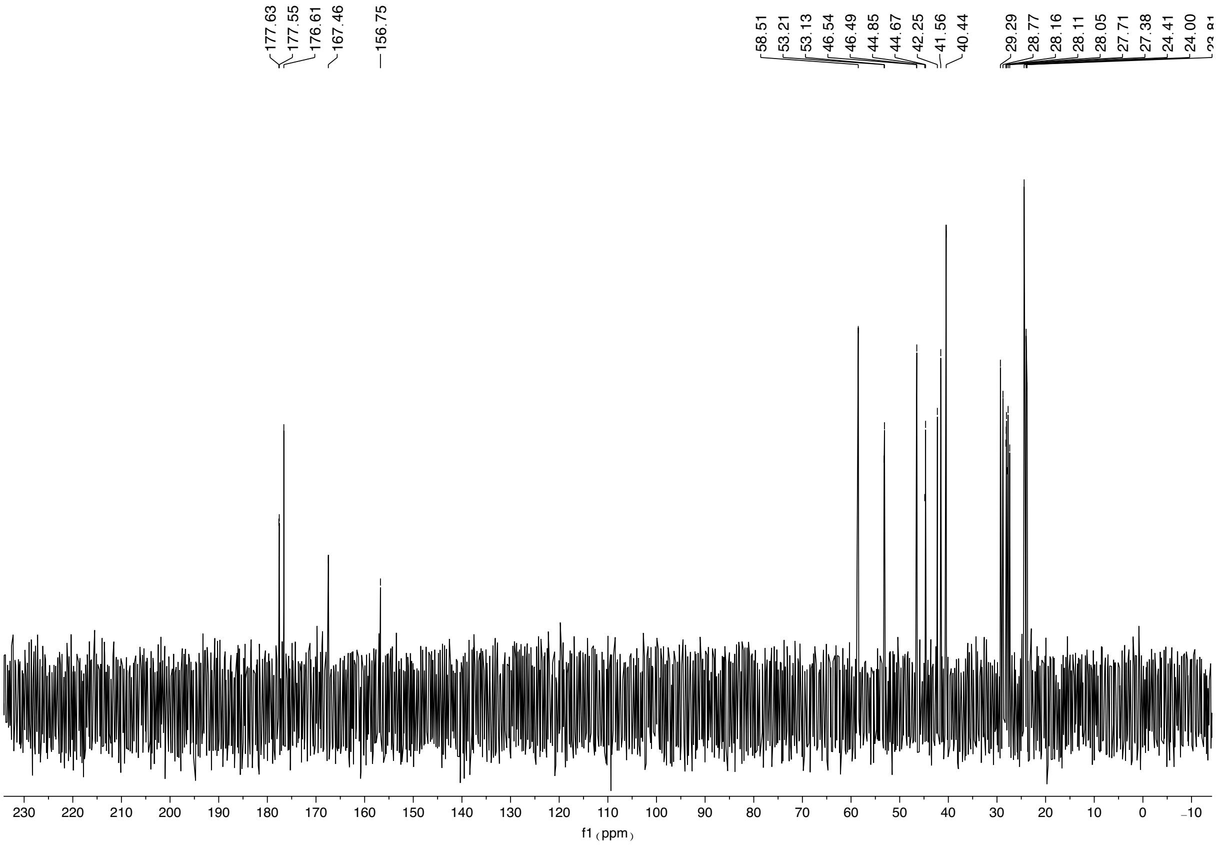

^{1}H and ^{13}C-NMR of H-D-Pro-L-(4*S*)-Azp-L-His-NH$_2$ · TFA (P(1)-7):

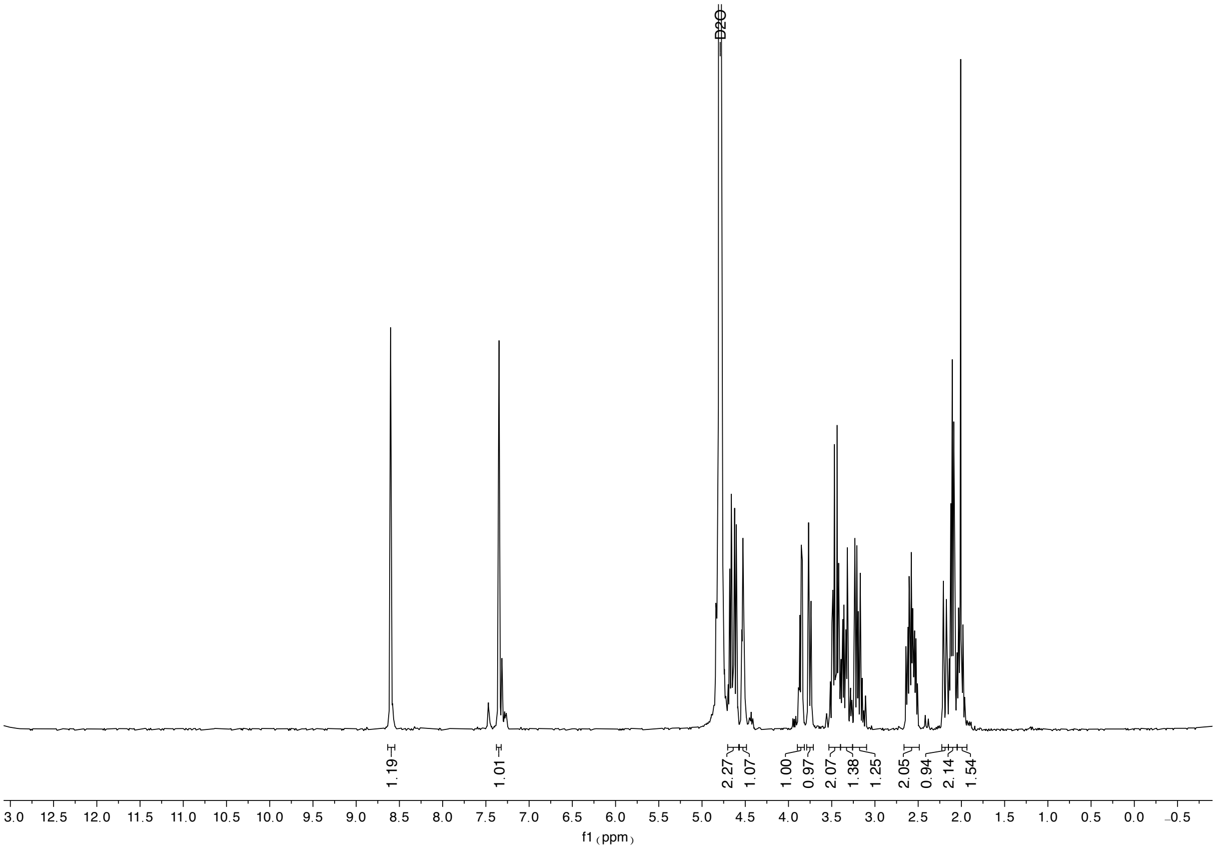

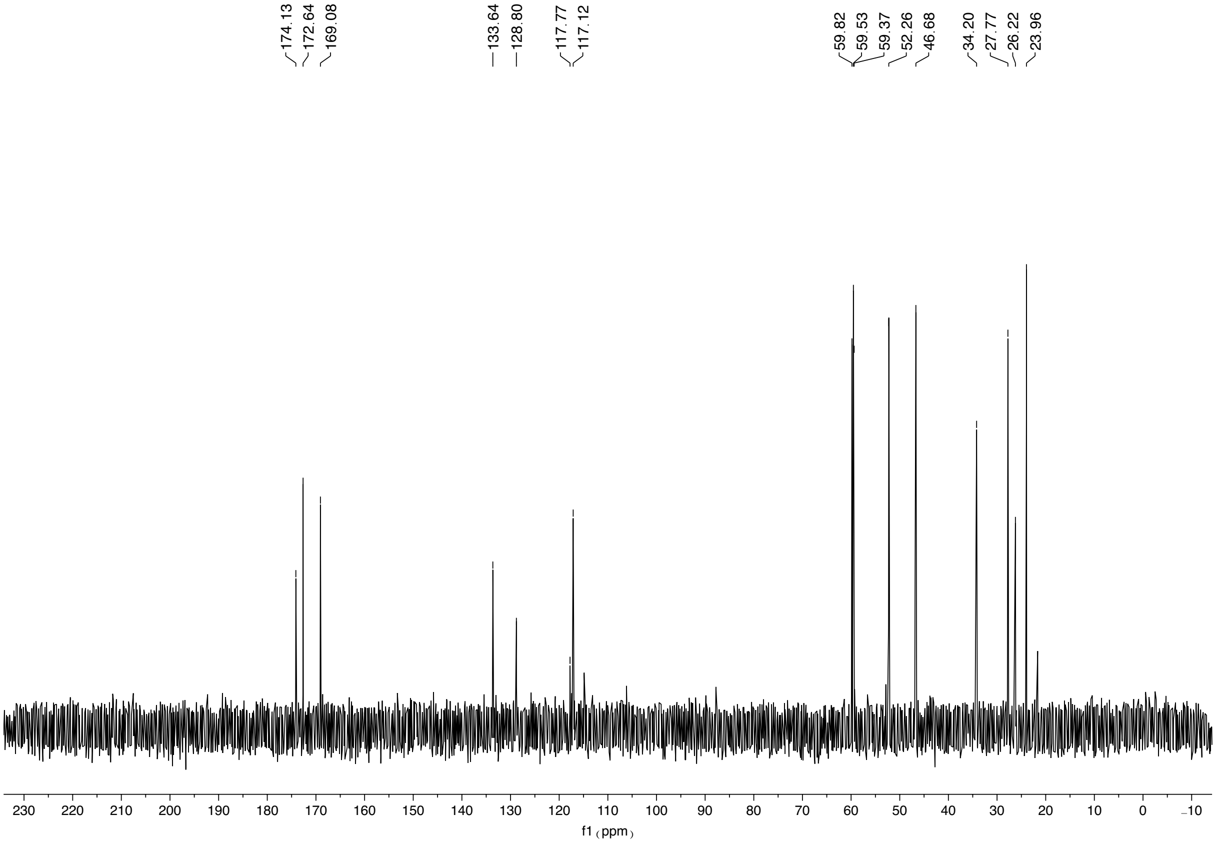

^{1}H and ^{13}C-NMR of H-D-Pro-L-Oic-L-Glu-NH$_2$ · TFA (P(2)-2):

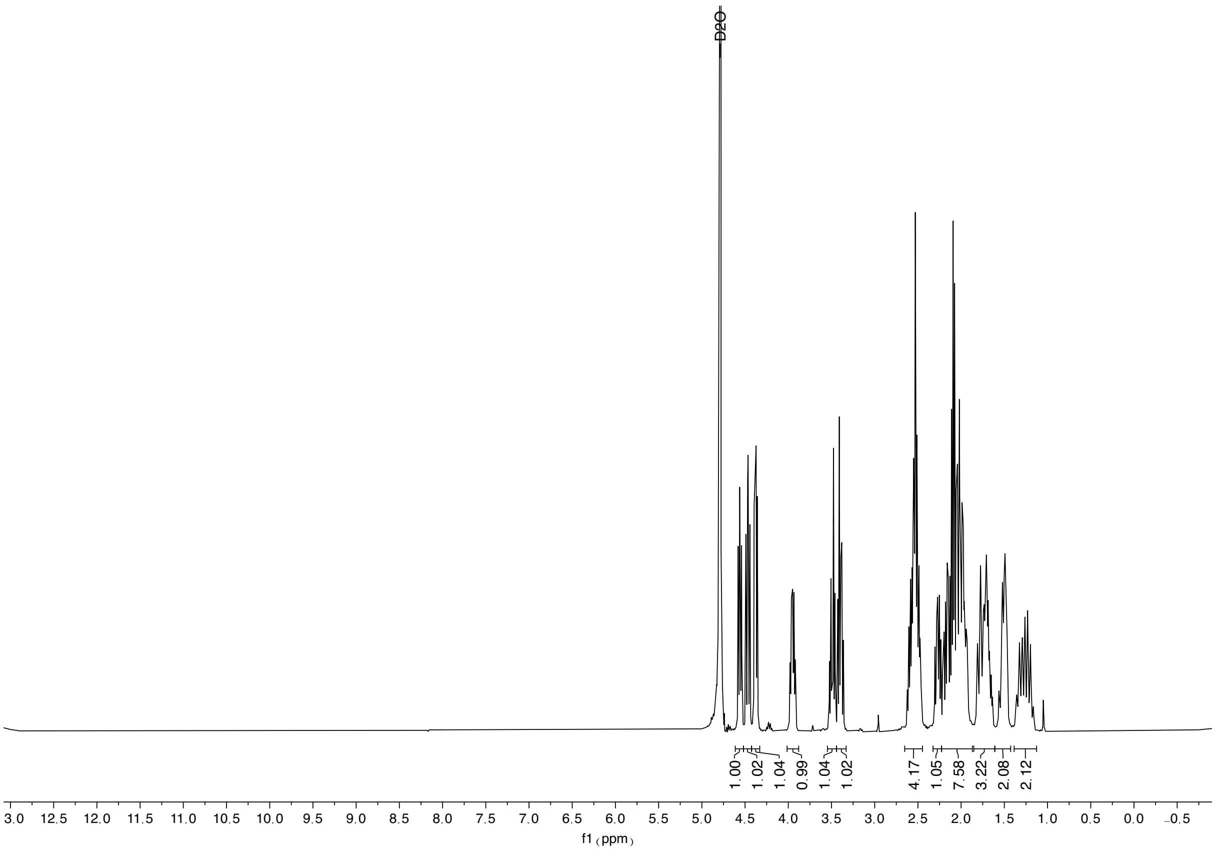

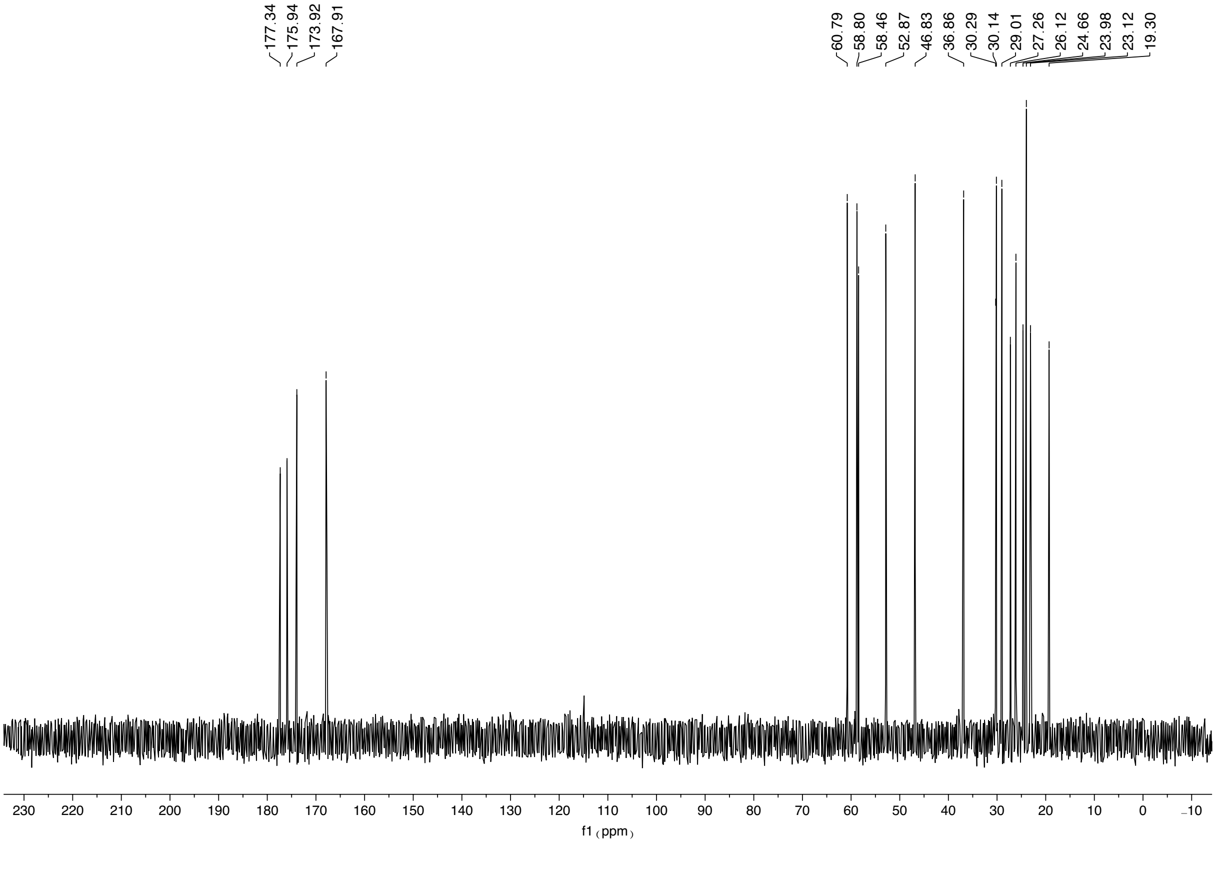

^{1}H and ^{13}C-NMR of H-D-Pro-L-(4*S*)-Hyp-L-DiGlu-NH₂ · TFA (P(2)-3):

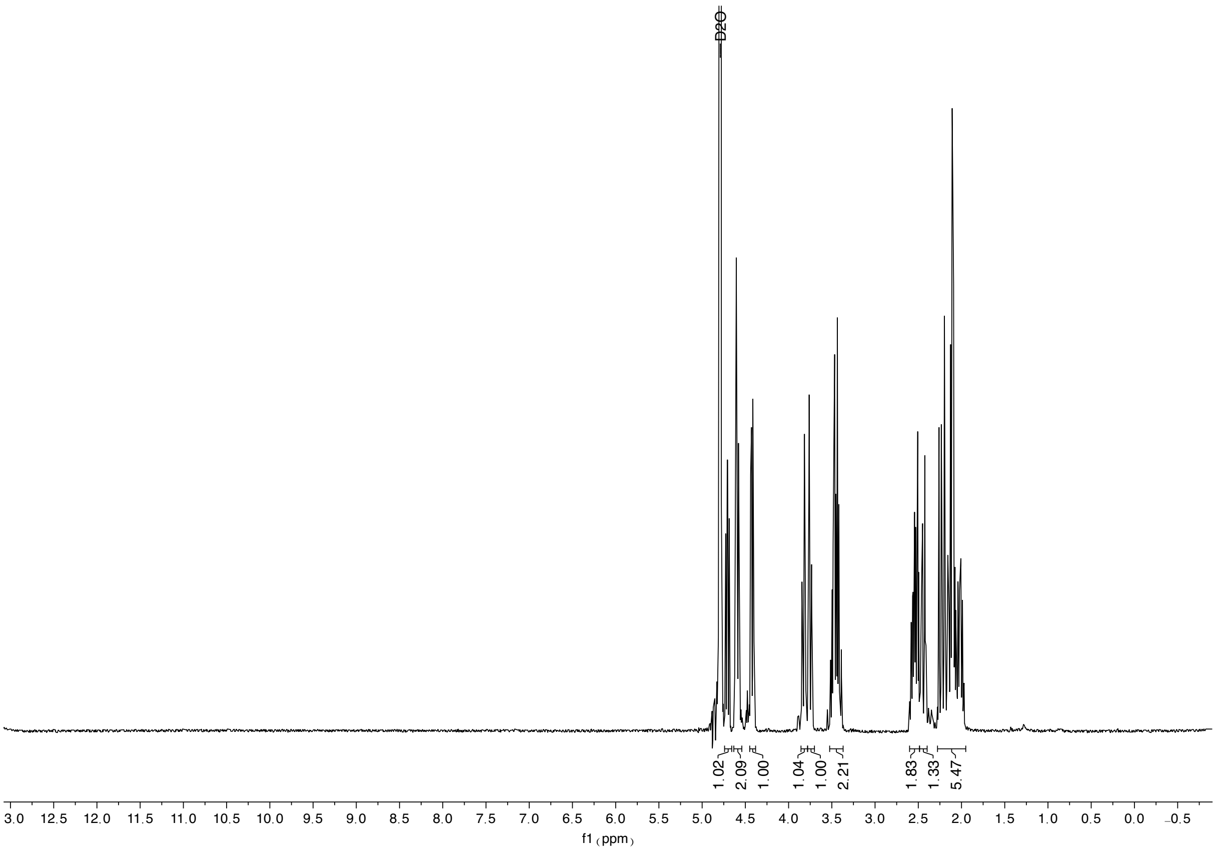

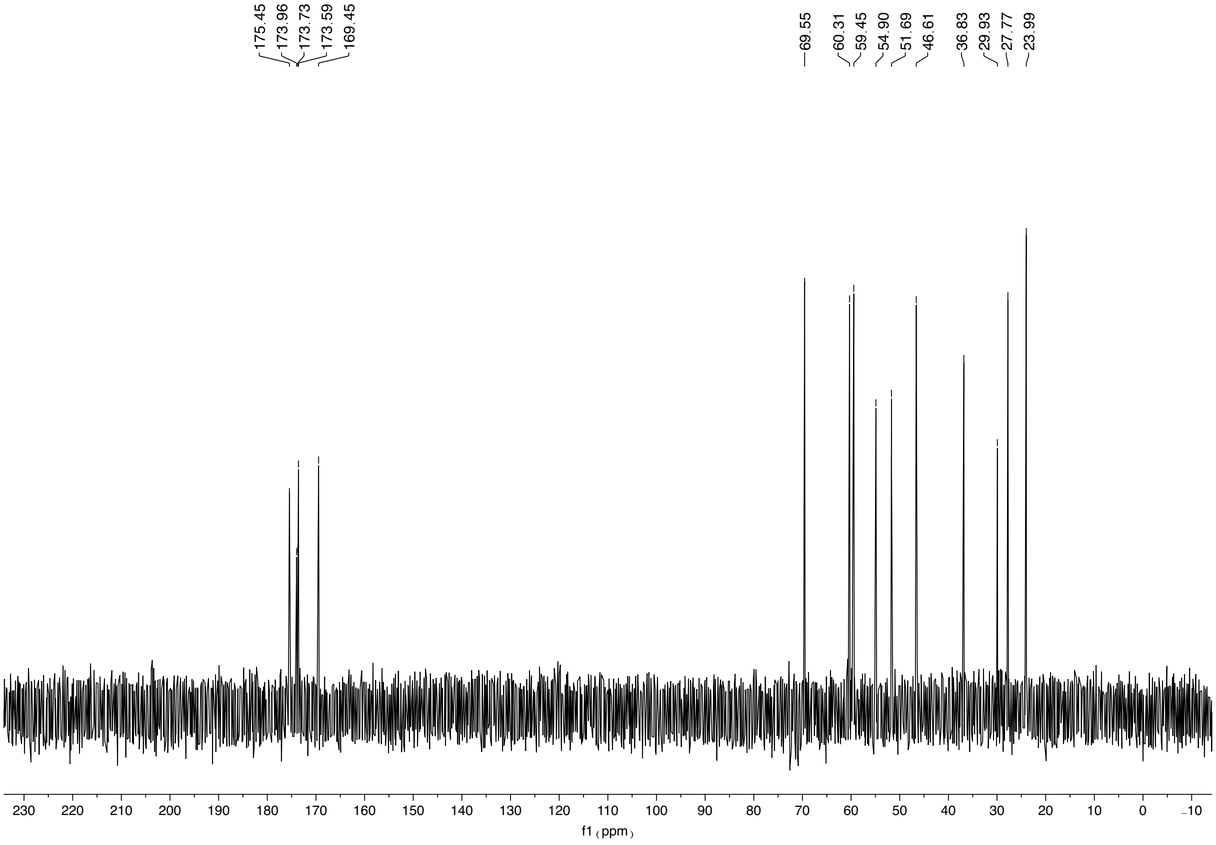

^{1}H and ^{13}C-NMR of H-D-Pro-L-(4*S*)-Hyp-L-Glu-NH₂ · TFA (P(2)-4):

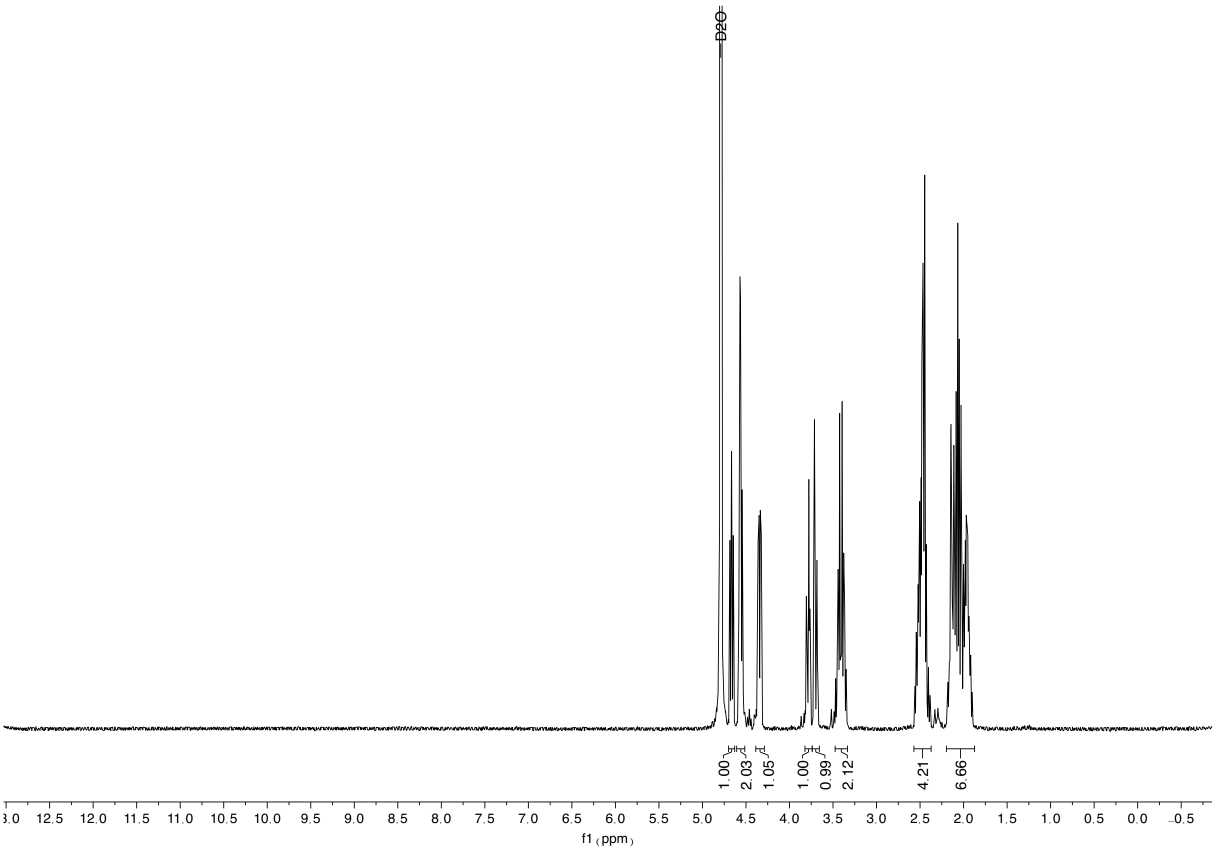

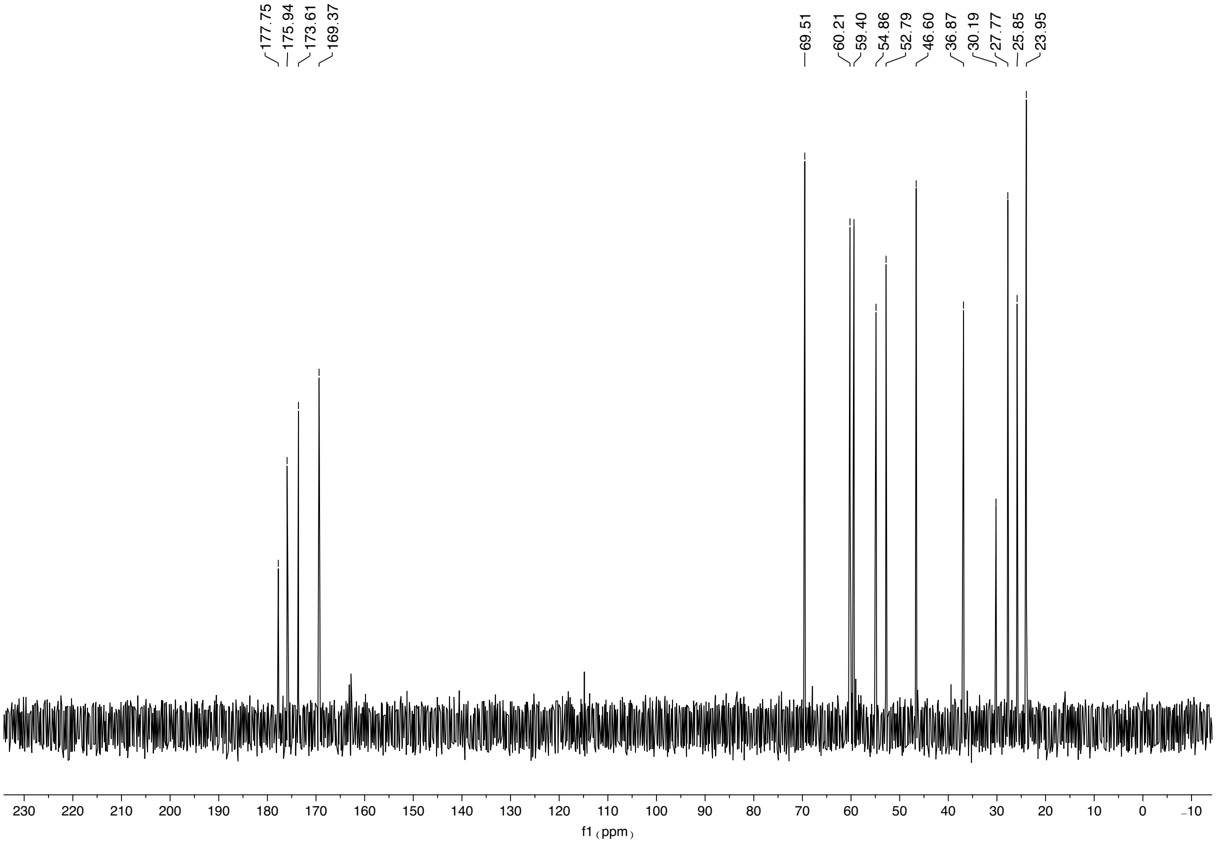

^{1}H and ^{13}C-NMR of H-D-Pro-L-(4*S*)-Hyp-L-Lys-NH_2 · TFA (P(2)-5):

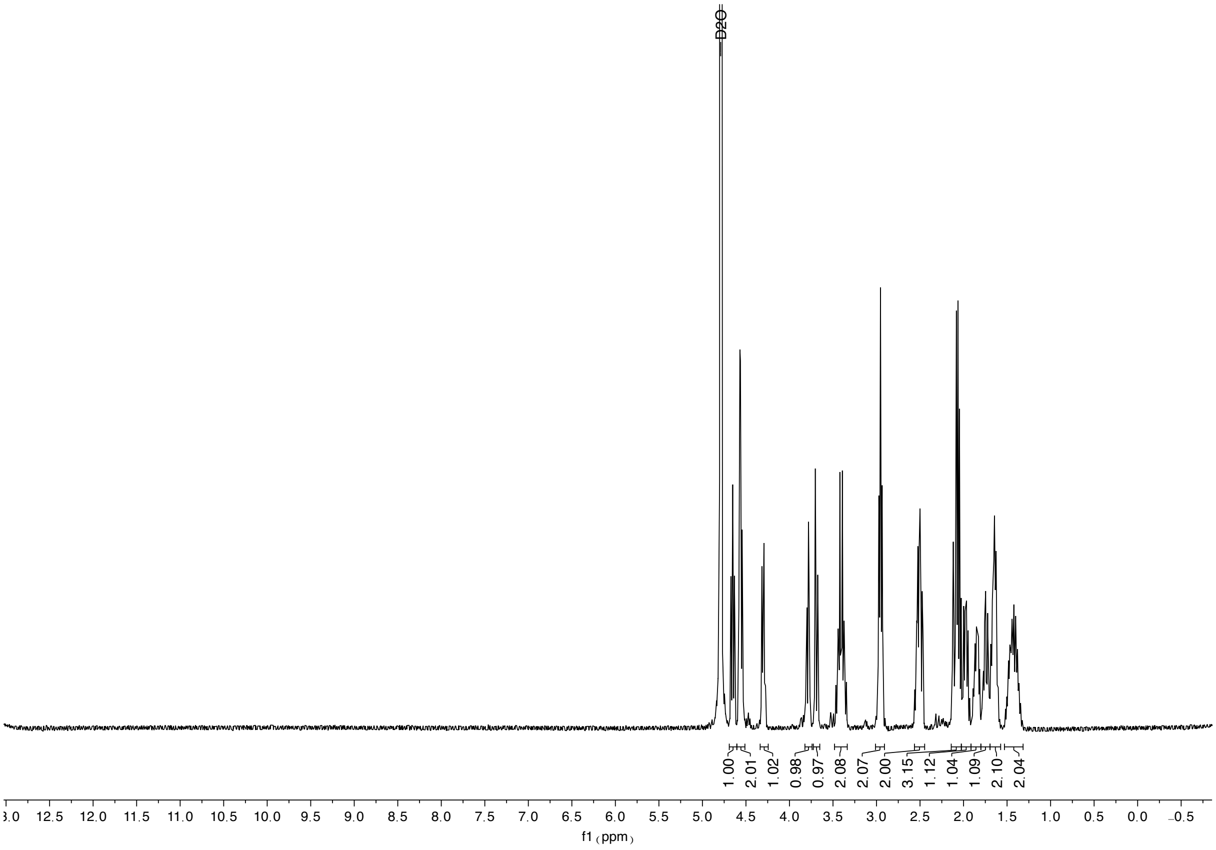

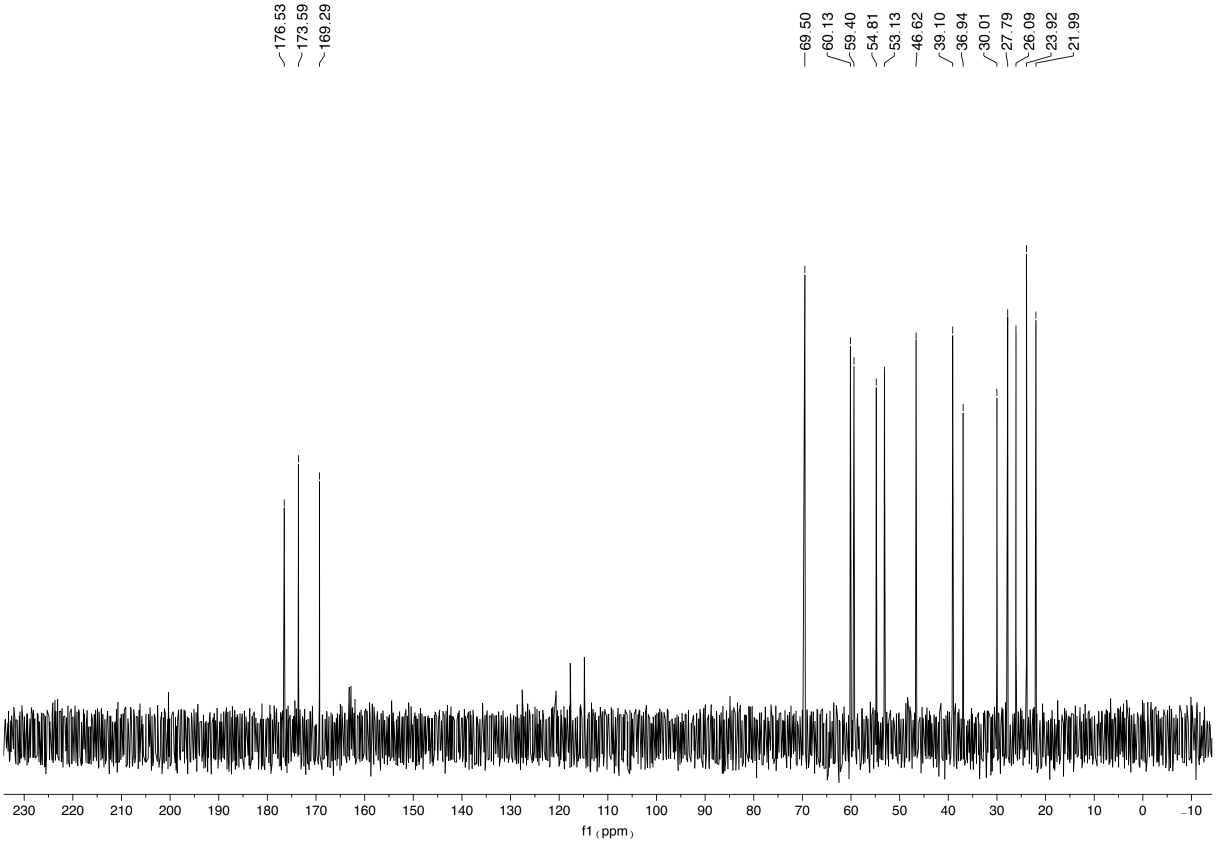

^{1}H and ^{13}C-NMR of H-D-Pro-L-Oic-L-HomoGlu-NH$_2$ · TFA (P(2)-6):

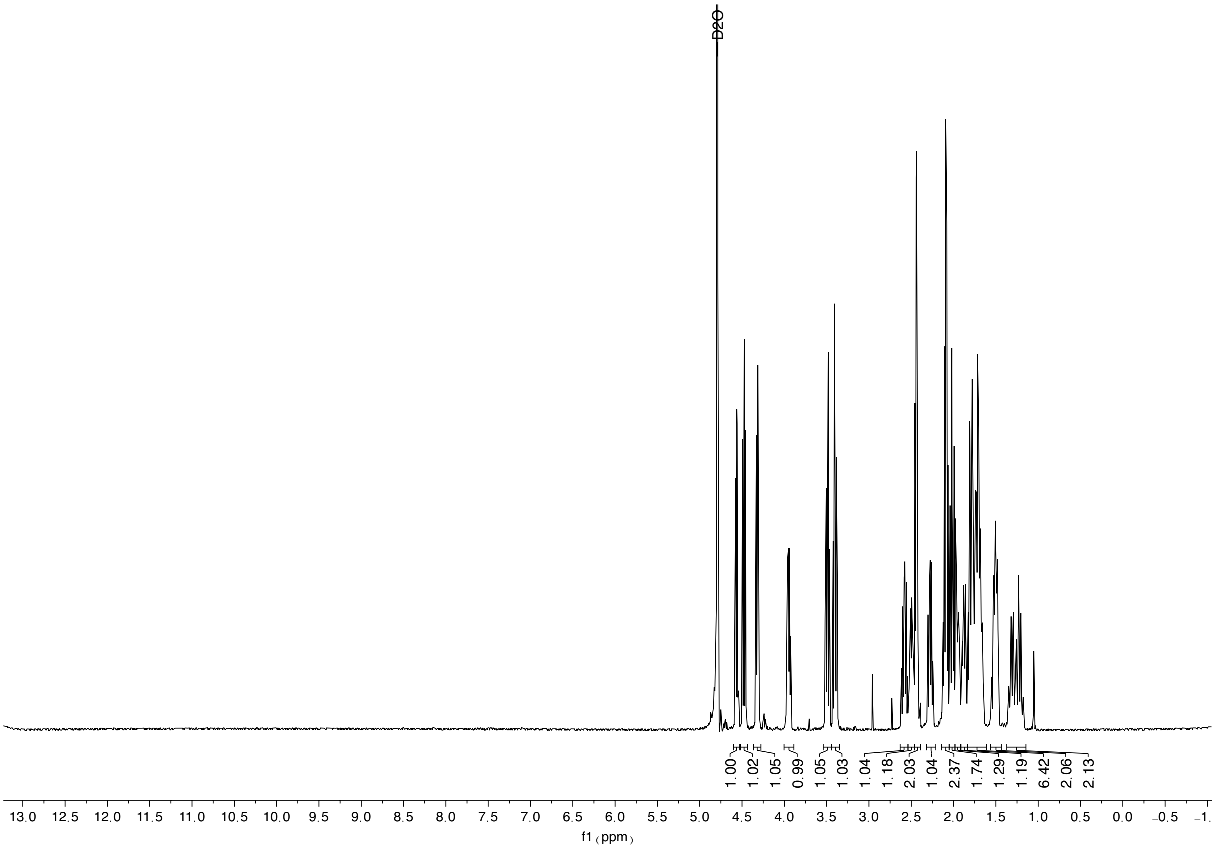

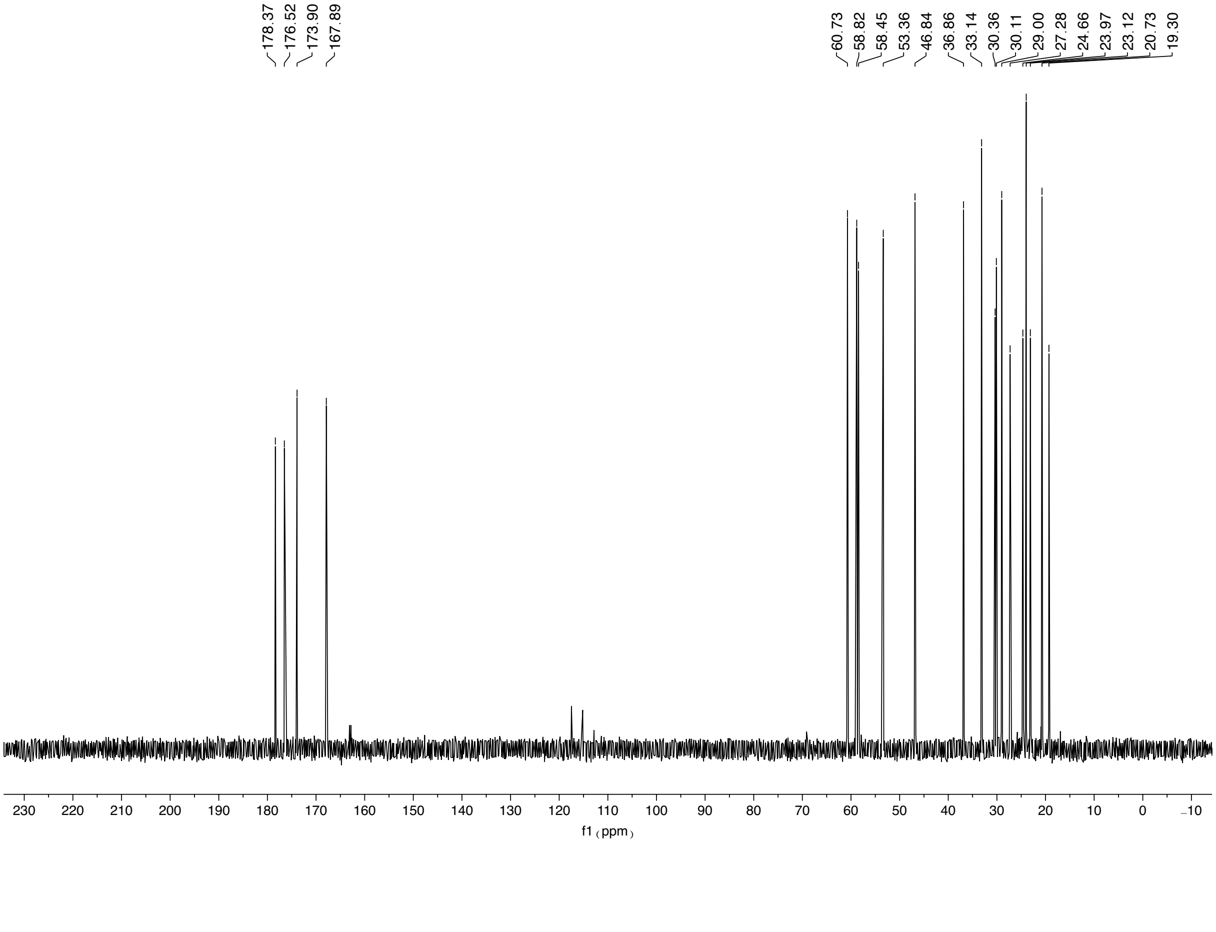

^{1}H and ^{13}C-NMR of H-D-Pro-L-(4*S*)-Hyp-D-Asp-NH$_2$ · TFA (P(2)-7):

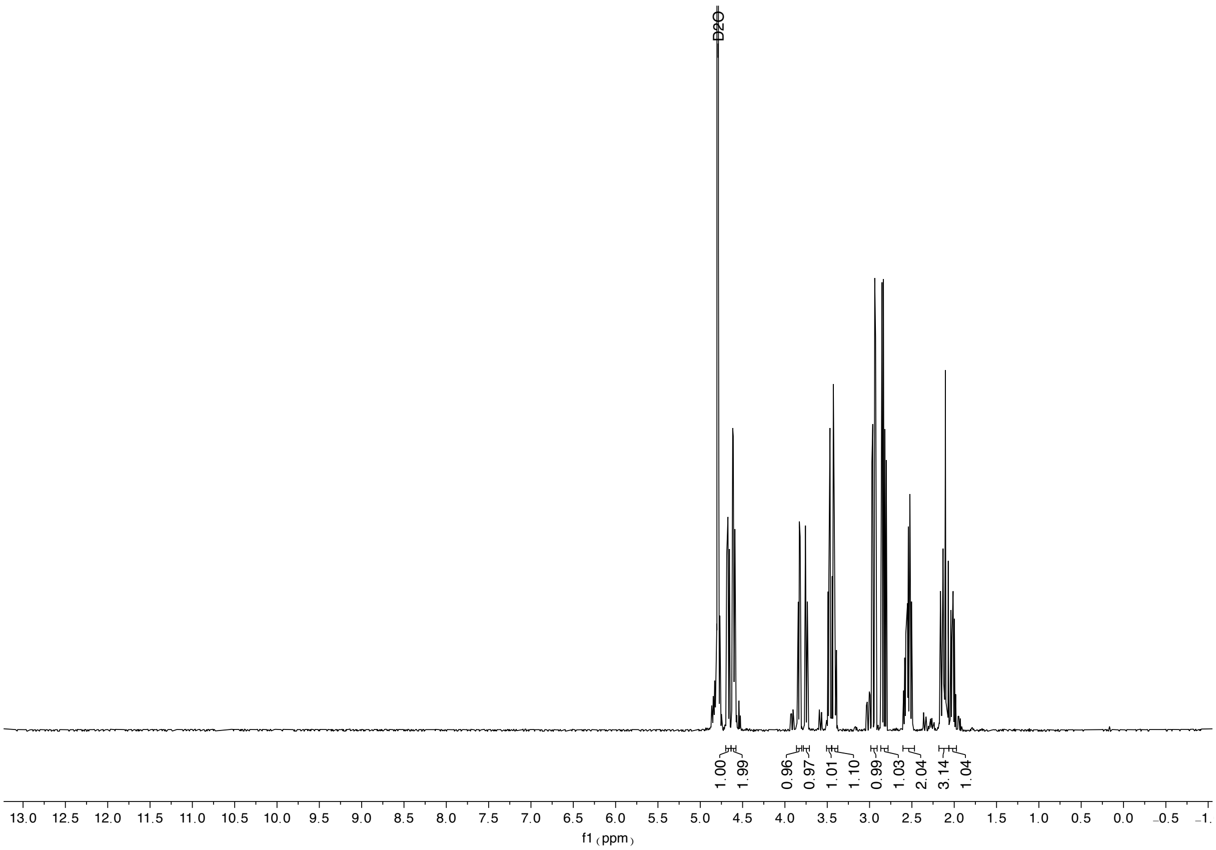

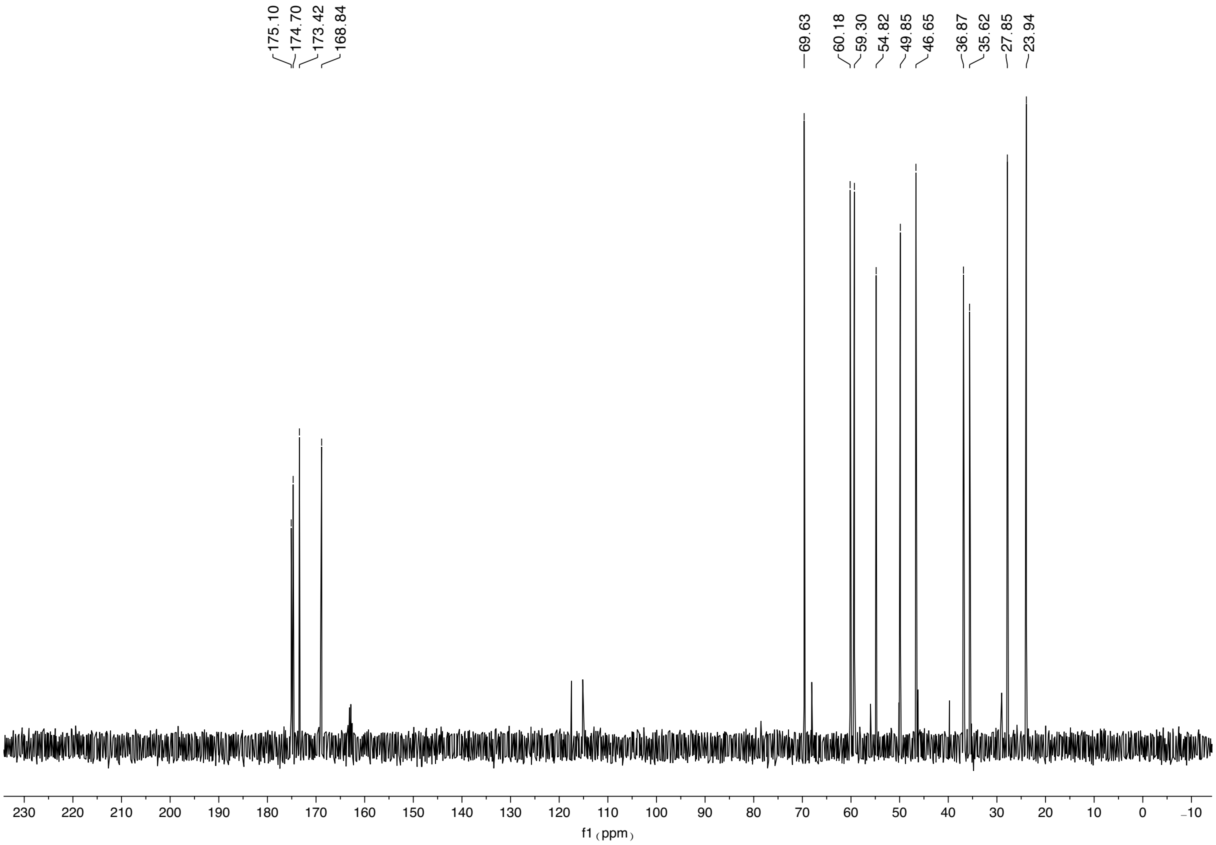

^{1}H and ^{13}C-NMR of H-D-Pro-L-(4*S*)-Azp-L-Gln-NH_2 · TFA (P(3)-1):

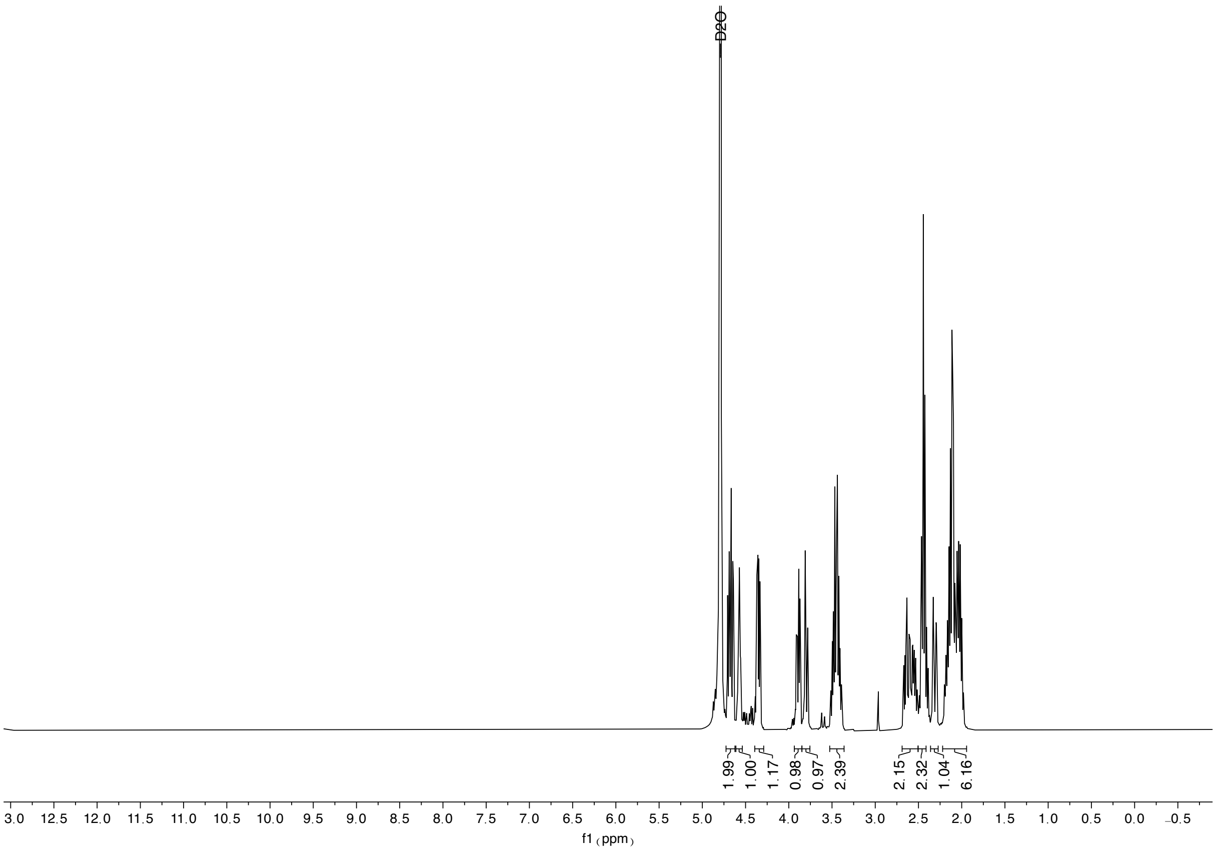

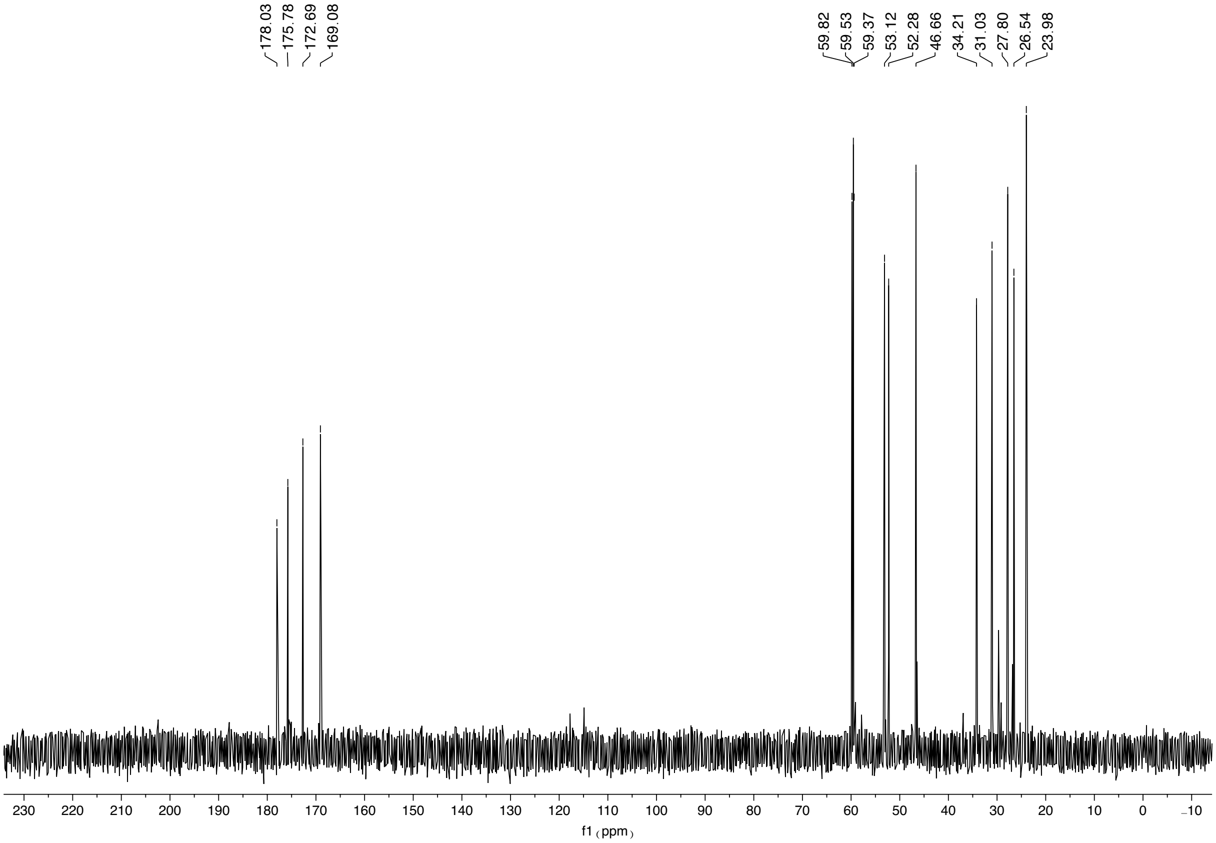

^{1}H and ^{13}C-NMR of H-D-Pro-L-(4S)-Flp-L-Arg-NH$_2$ · TFA (P(3)-2):

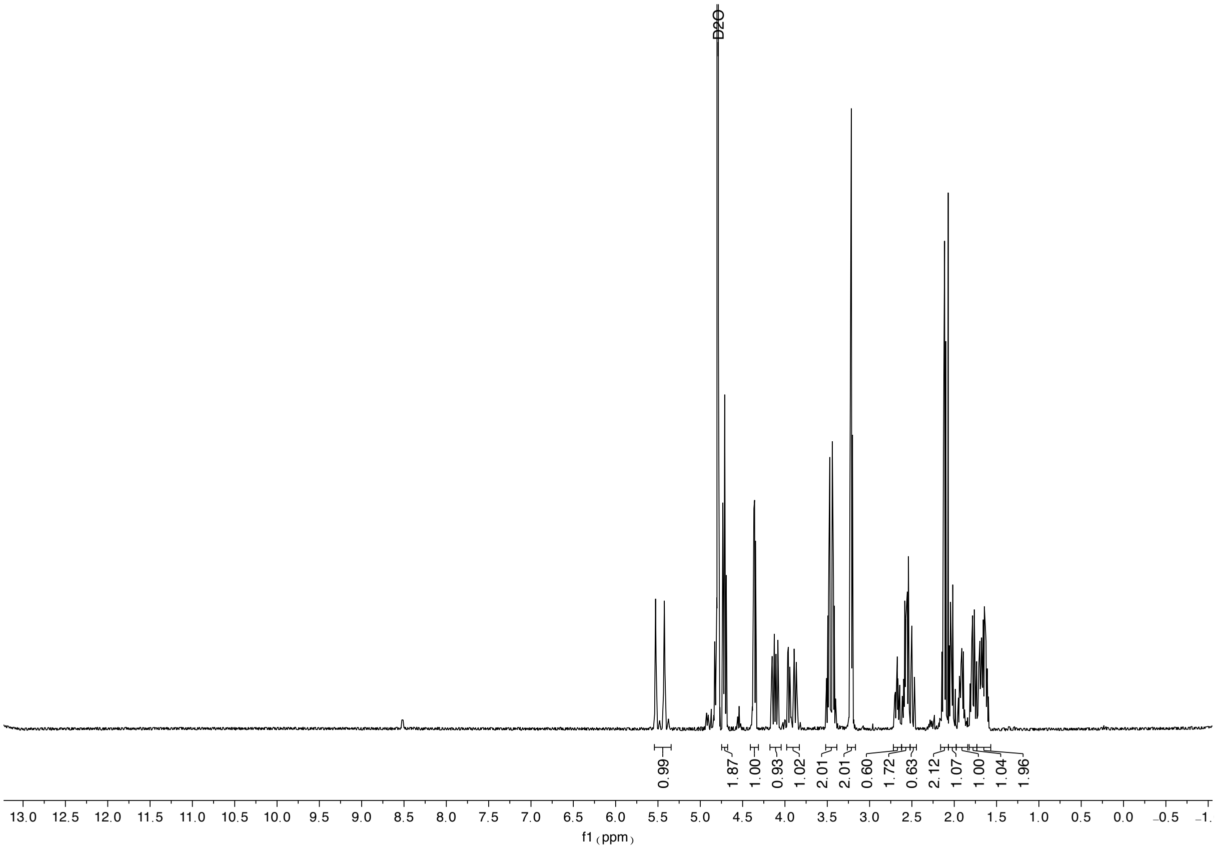

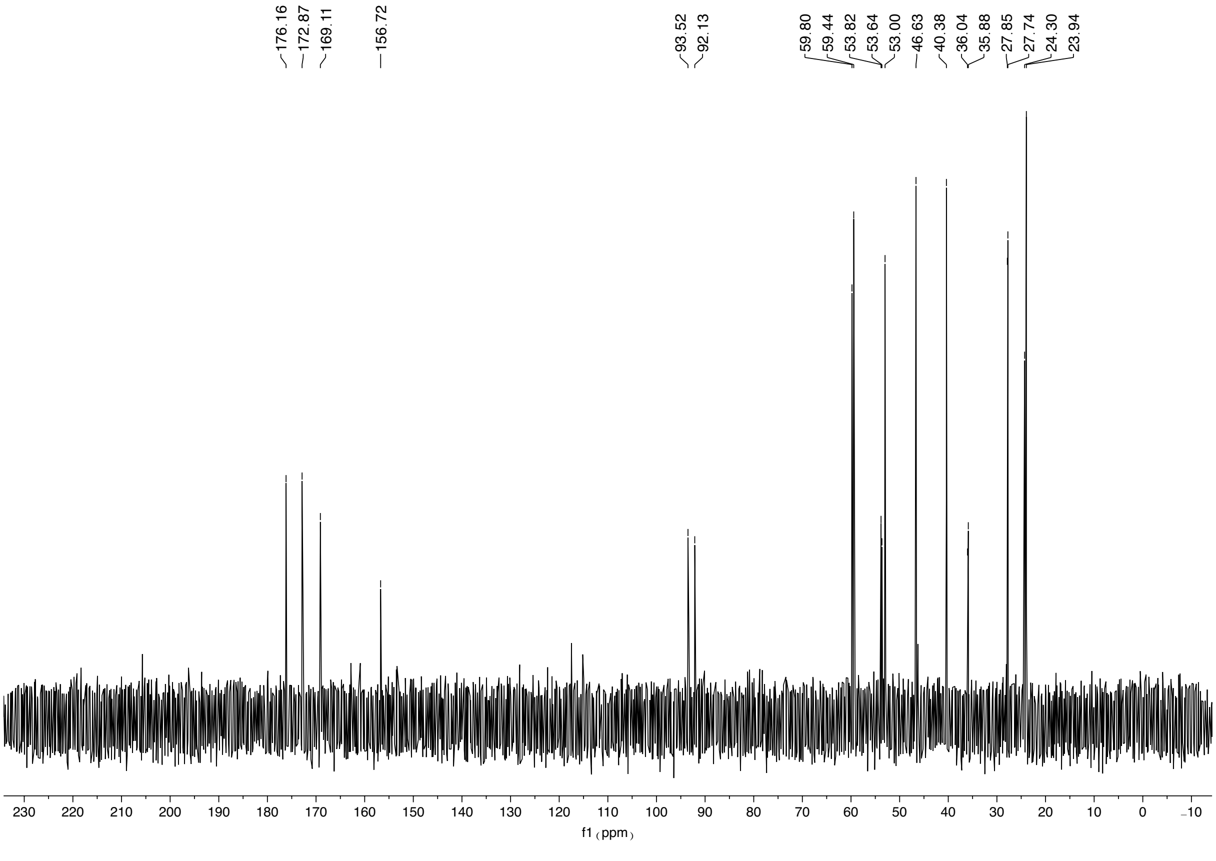

^{1}H and ^{13}C-NMR of H-D-Pro-L-Pro-L-DiGlu-NH$_2$ · TFA (P(3)-3):

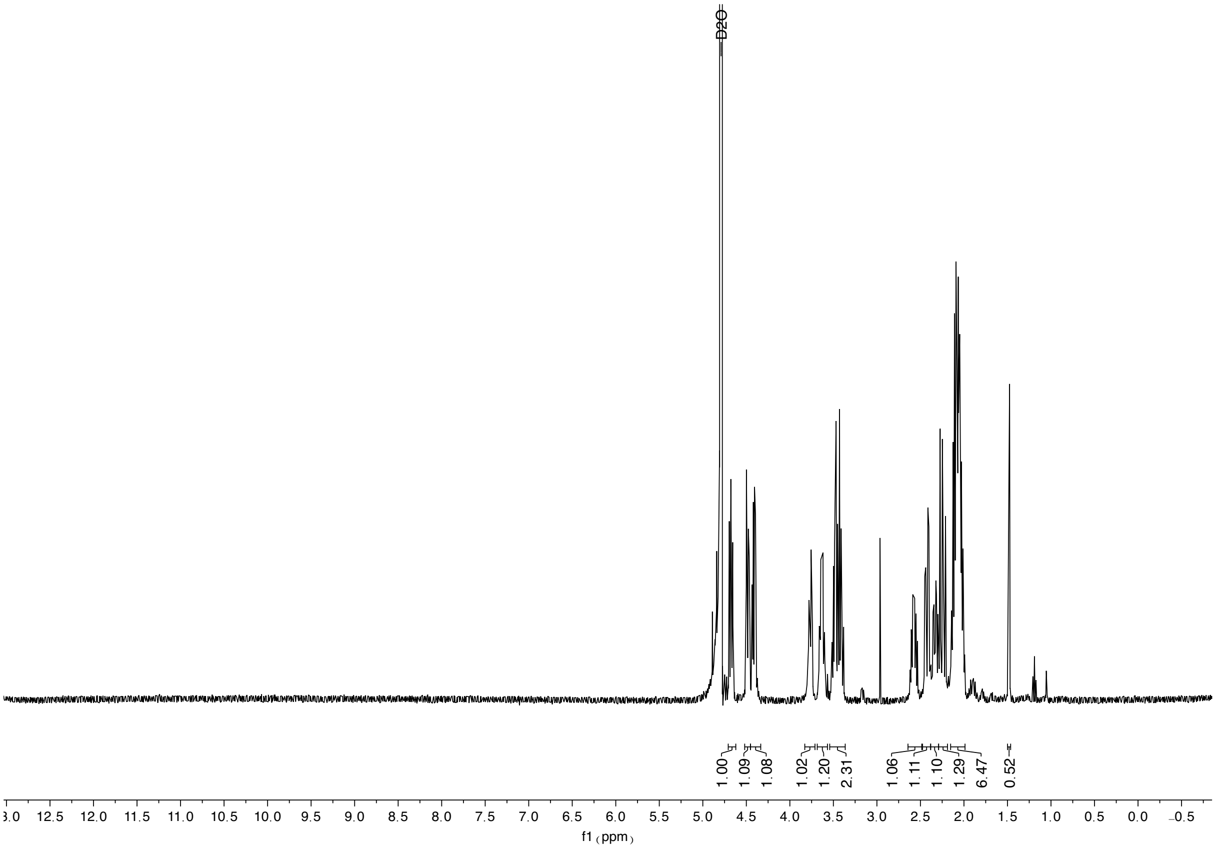

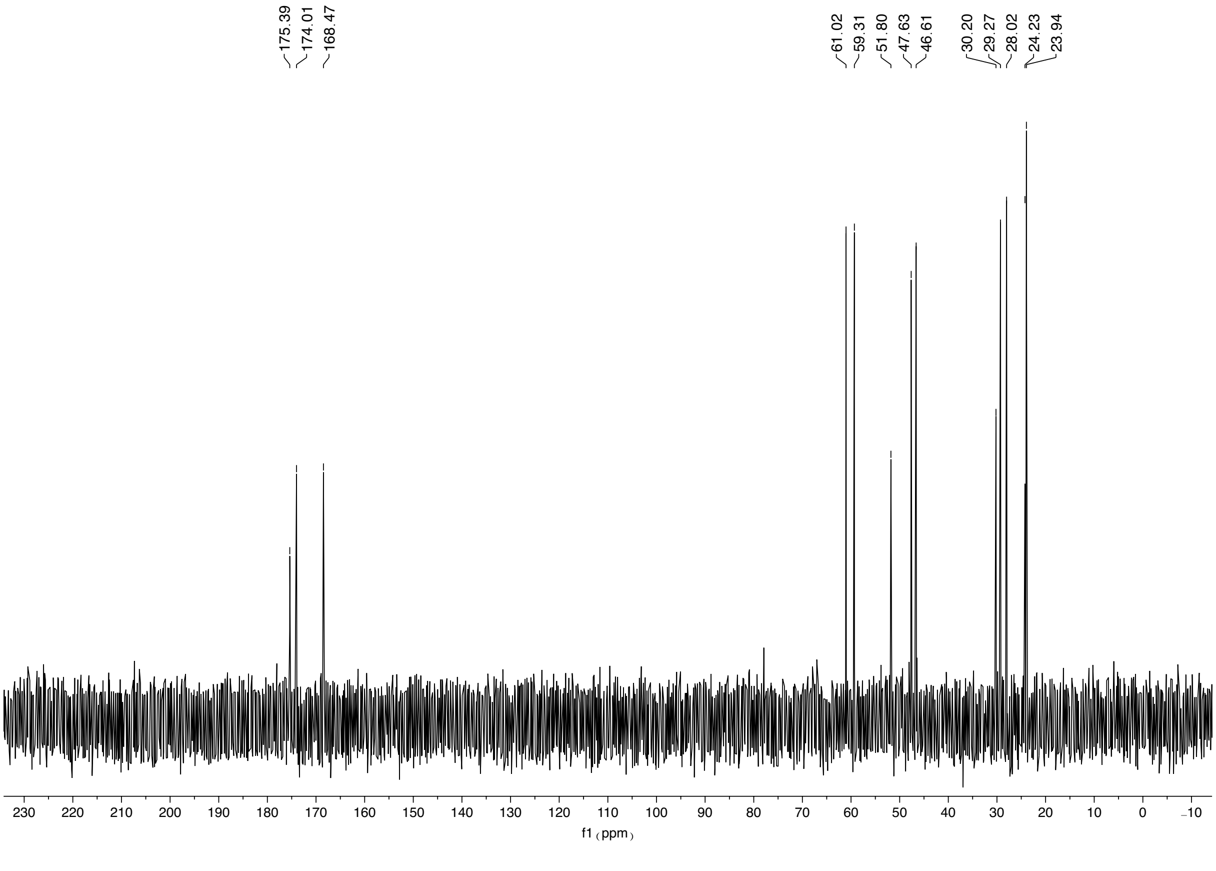

^{1}H and ^{13}C-NMR of H-D-Pro-L-(4*S*)-Azp-L-Glu-NH$_2$ · TFA (P(3)-4):

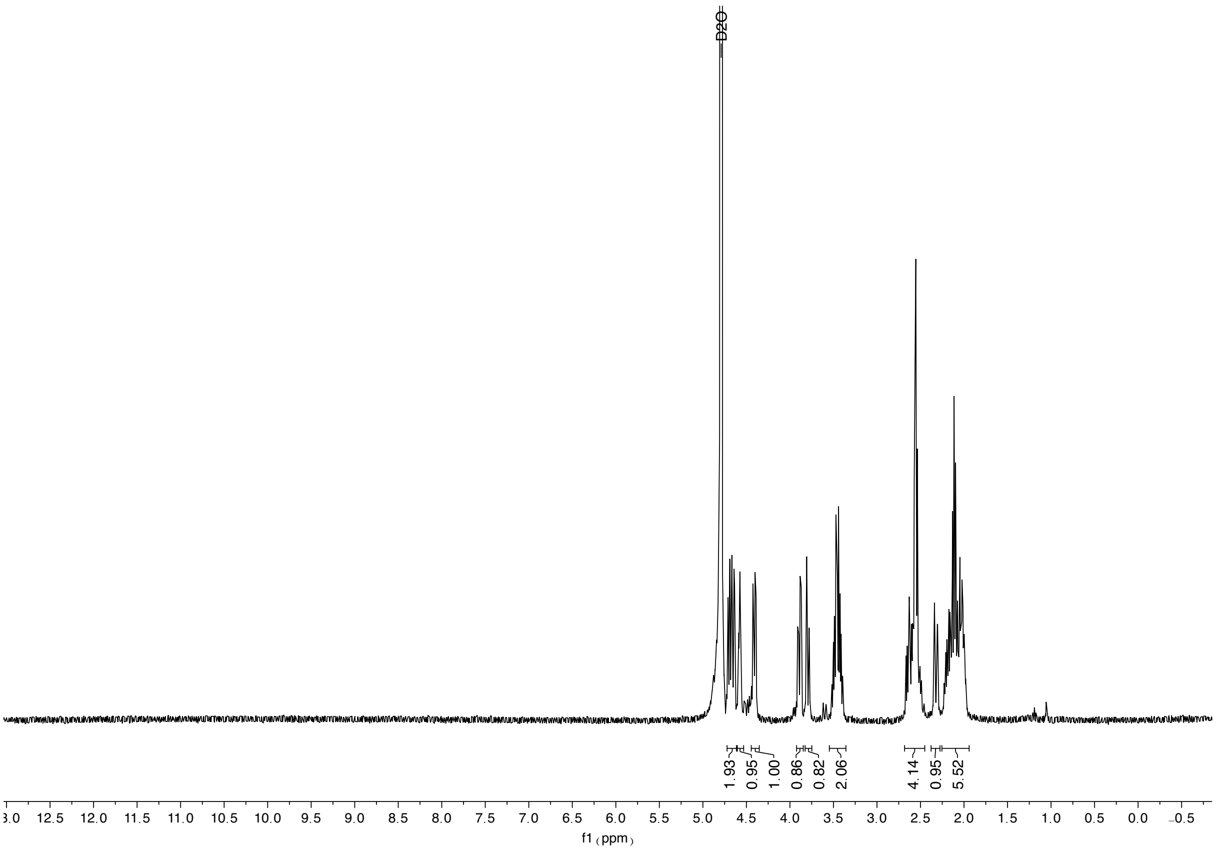

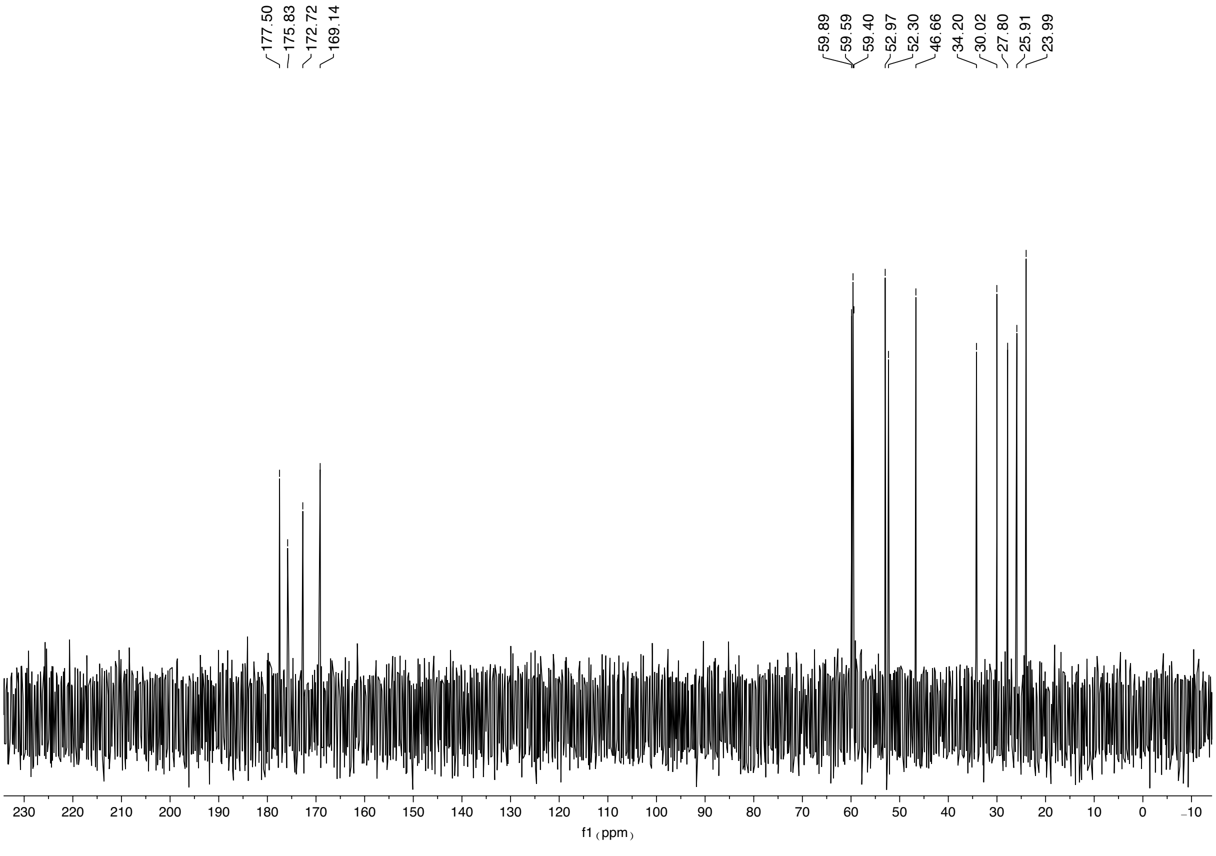

^{1}H and ^{13}C-NMR of H-D-Pro-L-(4*S*)-Flp-L-Asp-NH$_2$ · TFA (P(3)-5):

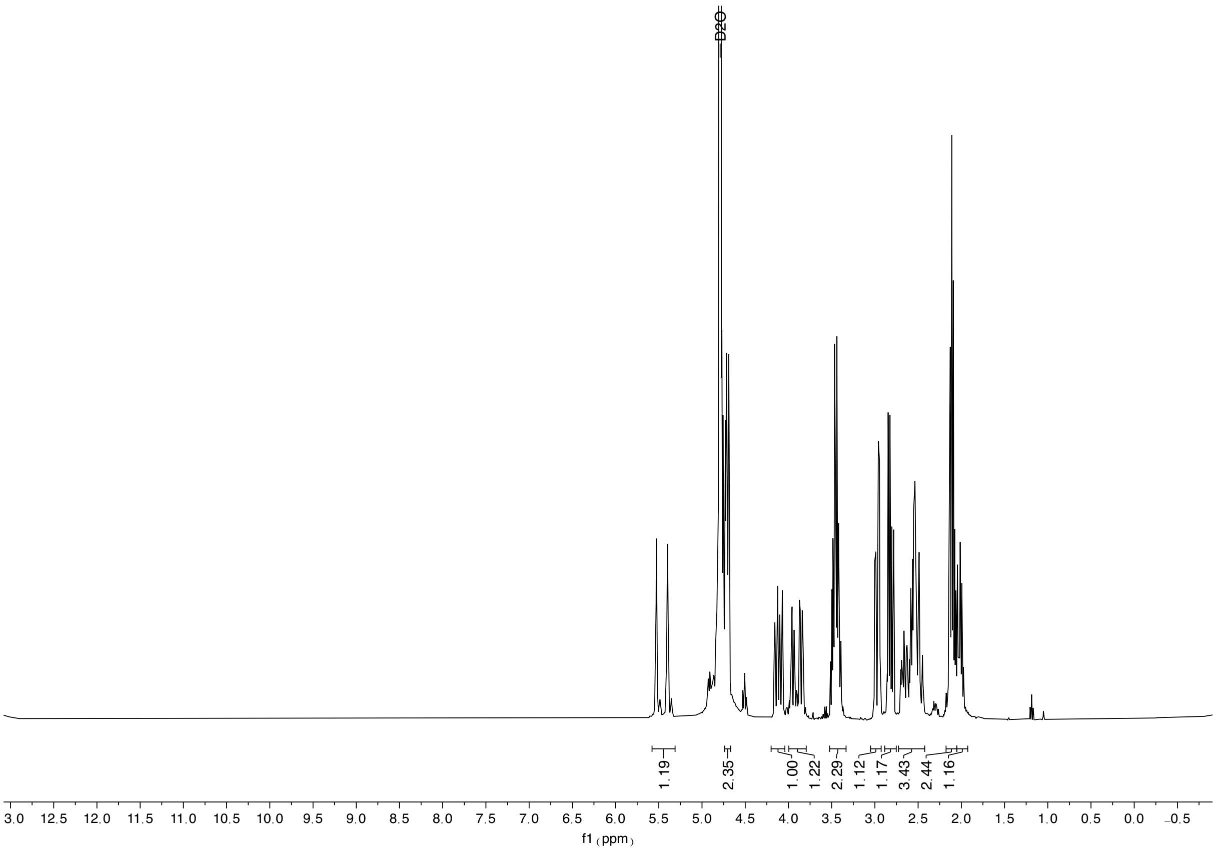

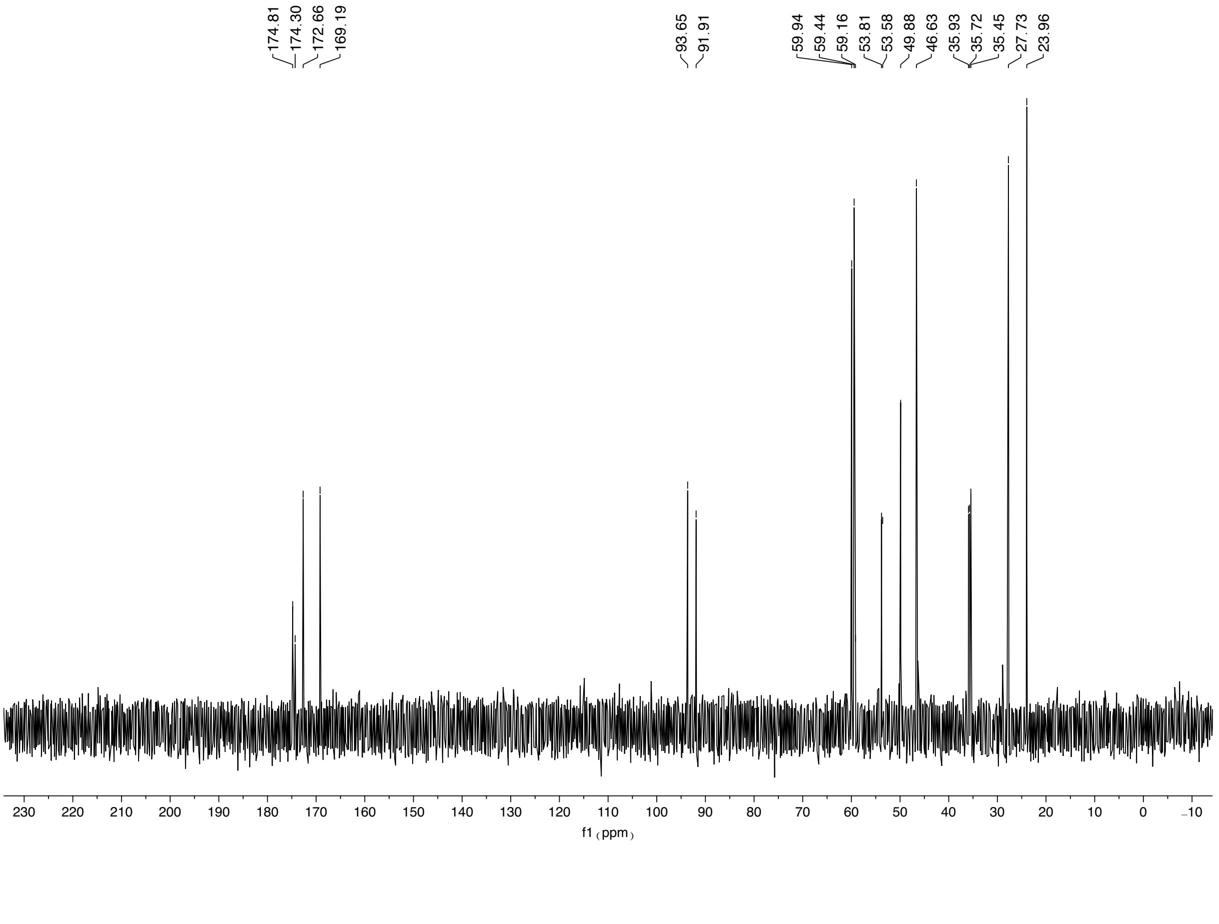

^{1}H and ^{13}C-NMR of H-D-Pro-L-(4*S*)-Azp-L-Cit-NH_2 · TFA (P(3)-6):

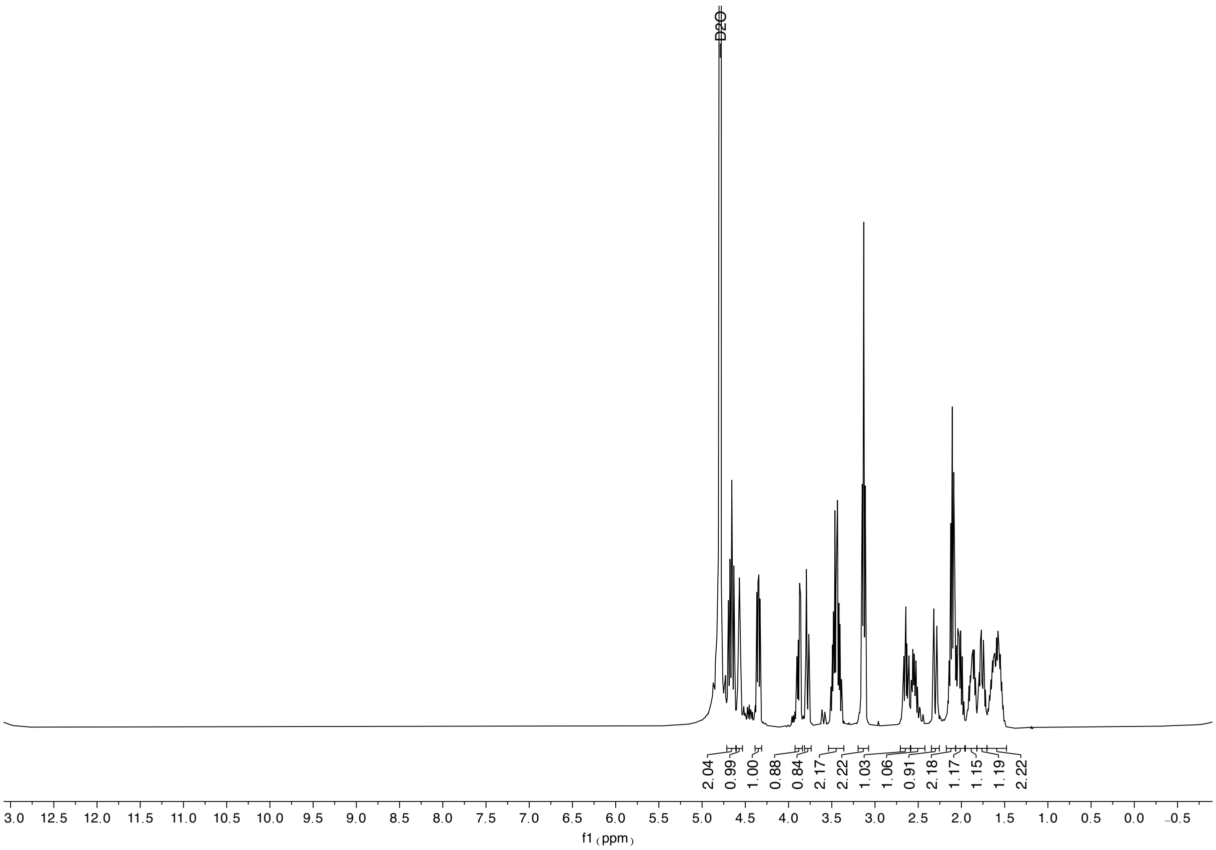

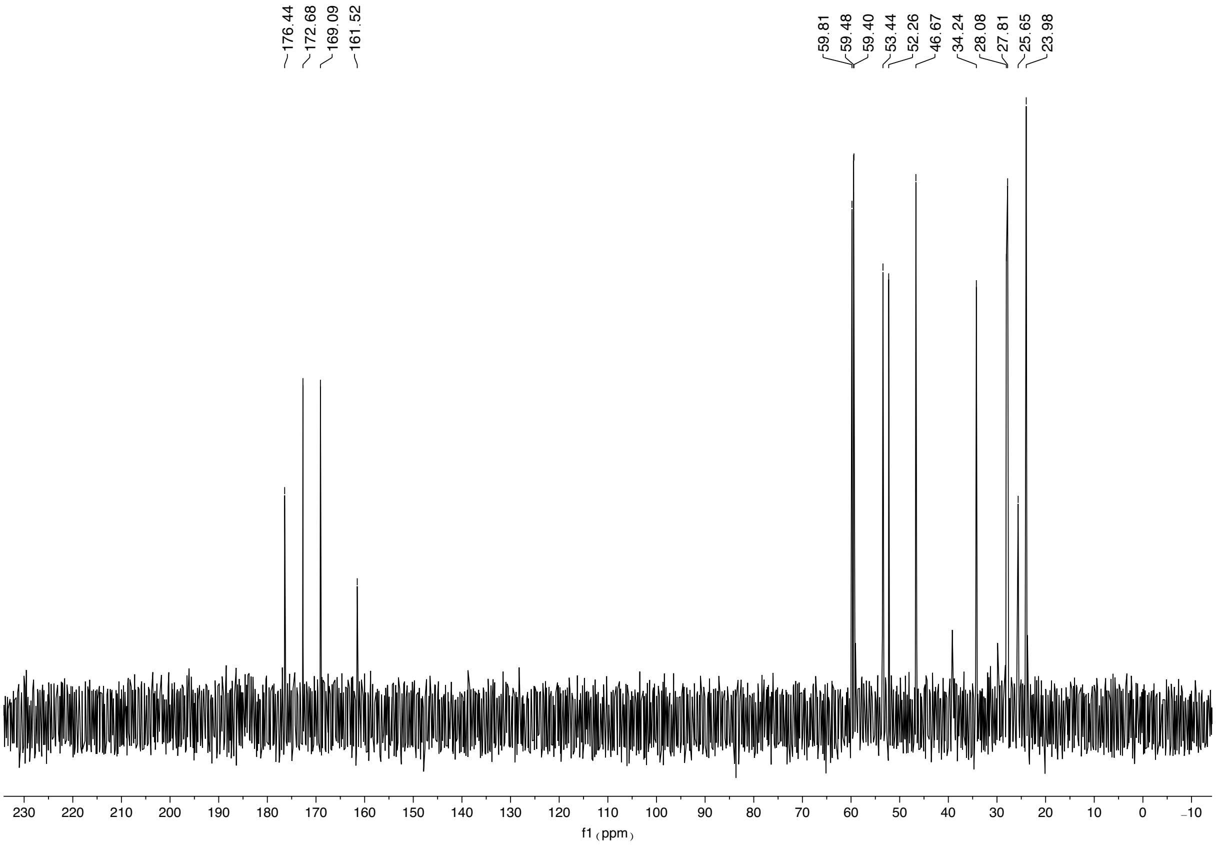

^{1}H and ^{13}C-NMR of H-D-Pro-L-(4*S*)-Azp-L-"F"Phen-NH_2 · TFA (P(3)-7):

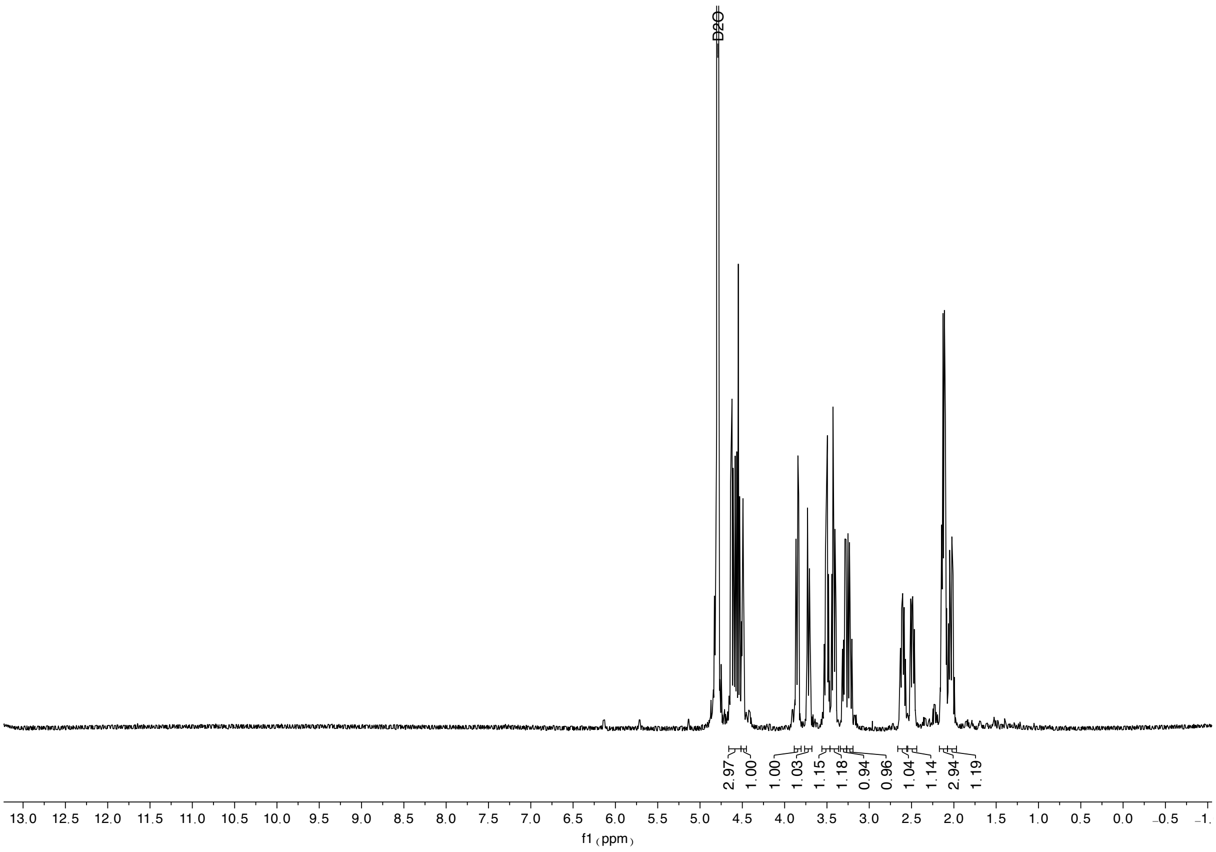

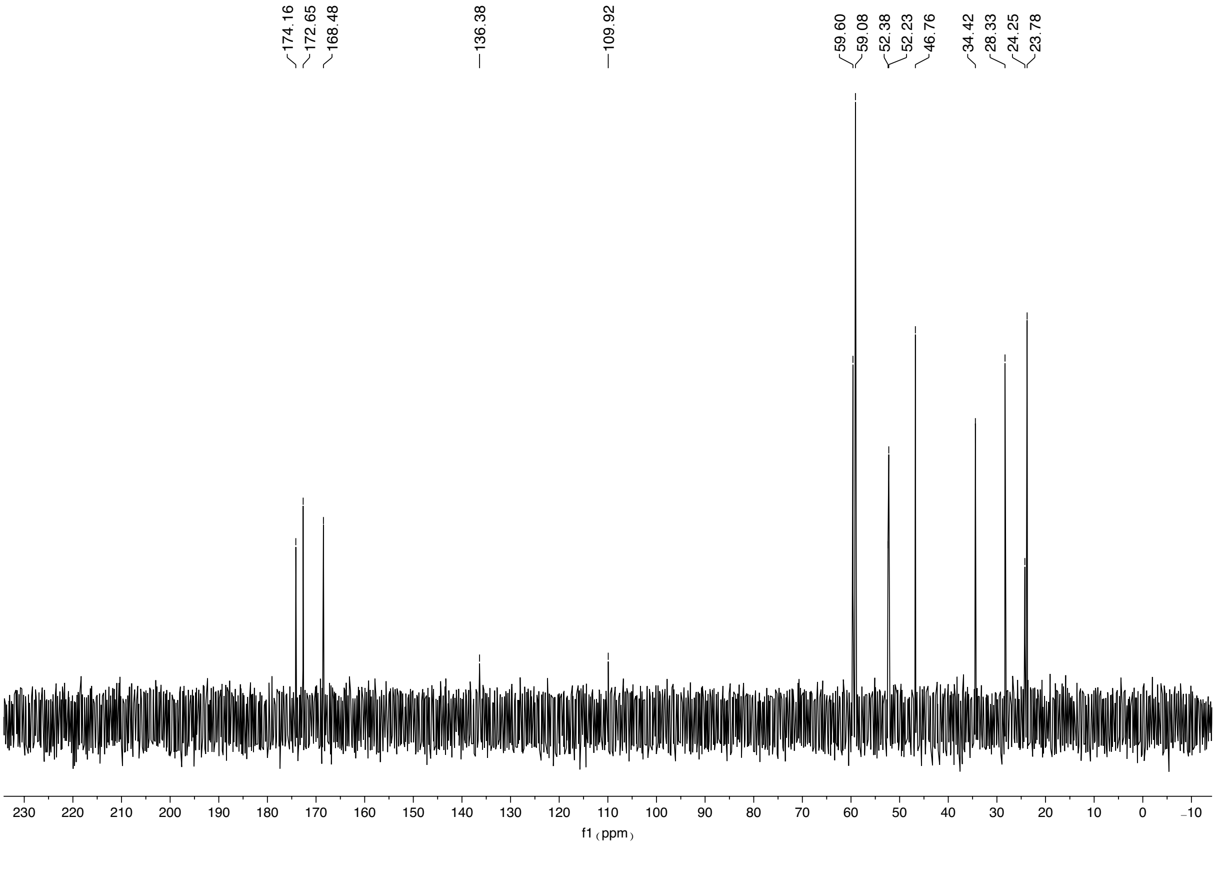

^{1}H and ^{13}C-NMR of H-D-Pro-D-His-L-(4*S*)-Flp-NH_2 · TFA (P(4)-1):

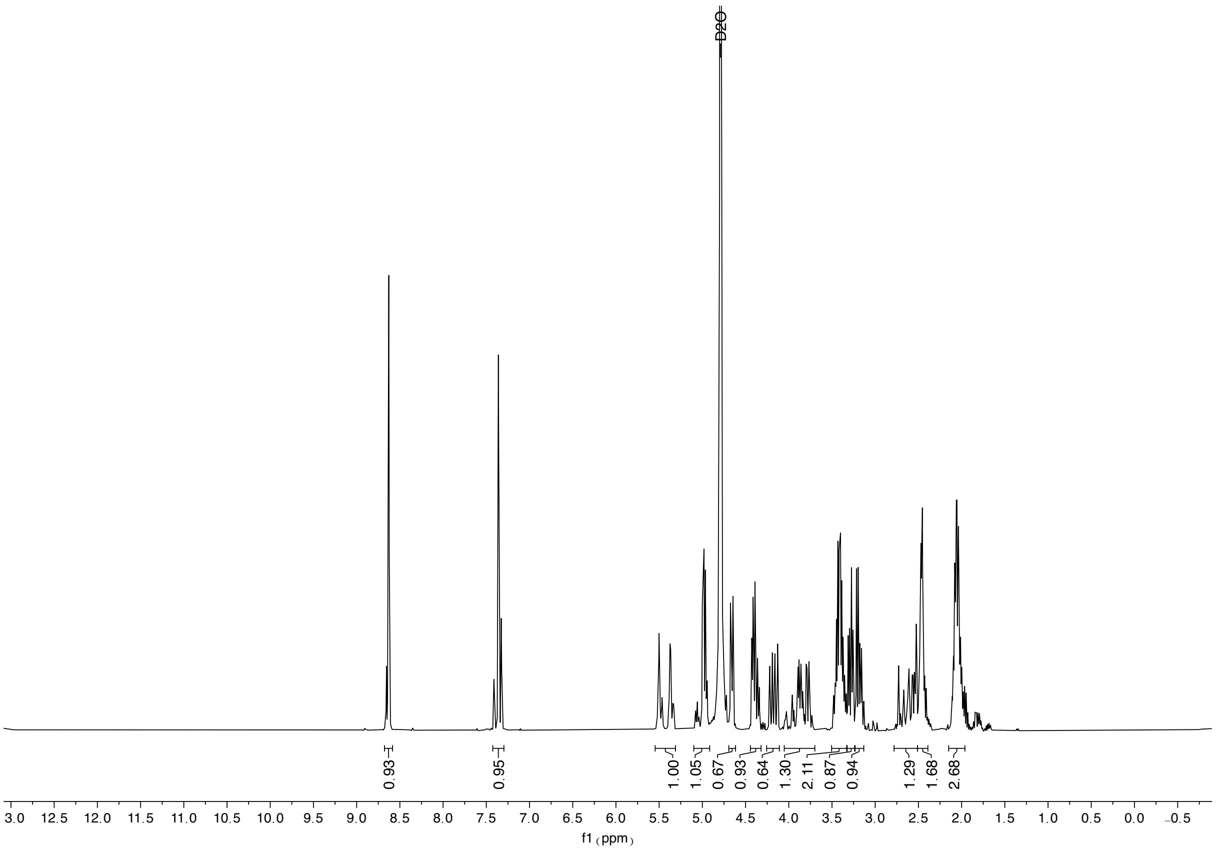

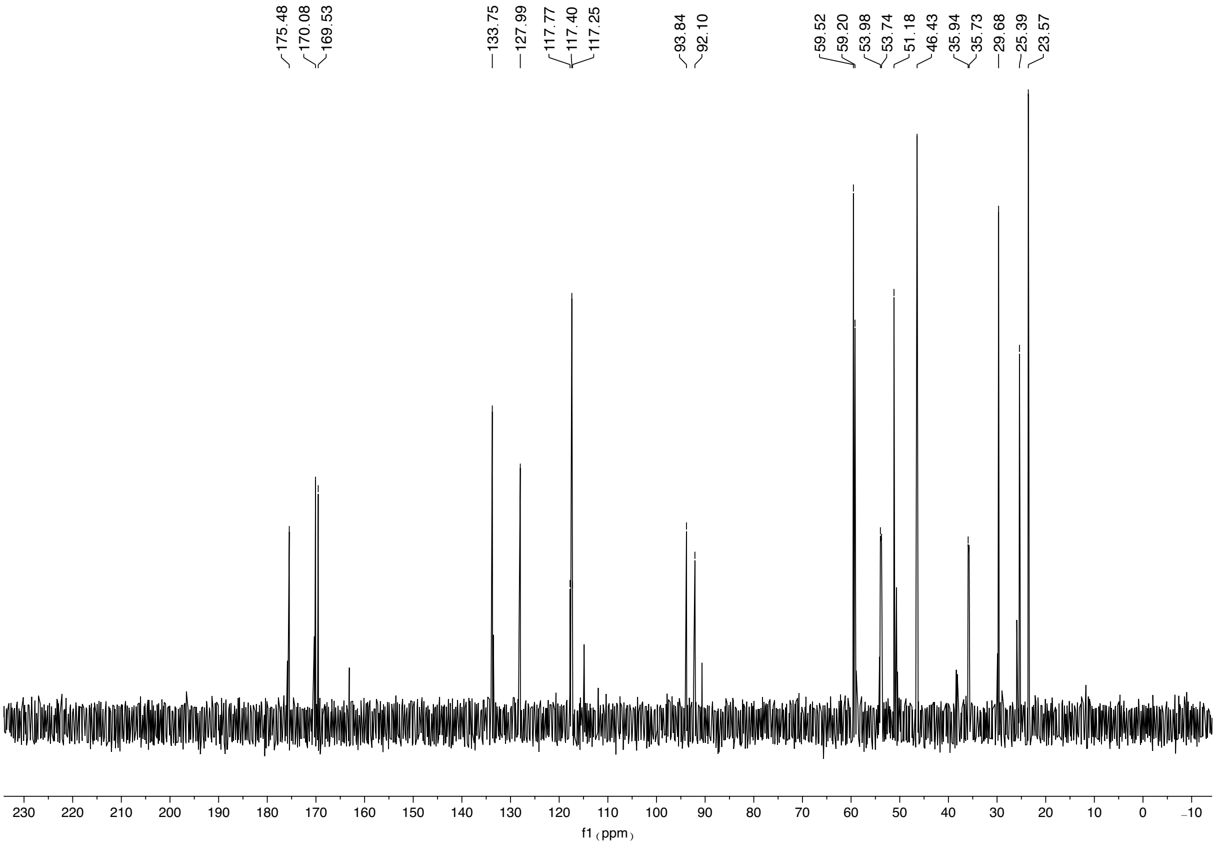

^{1}H and ^{13}C-NMR of H-L-Pro-L-His-D-Pro-NH$_2$ · TFA (P(4)-2):

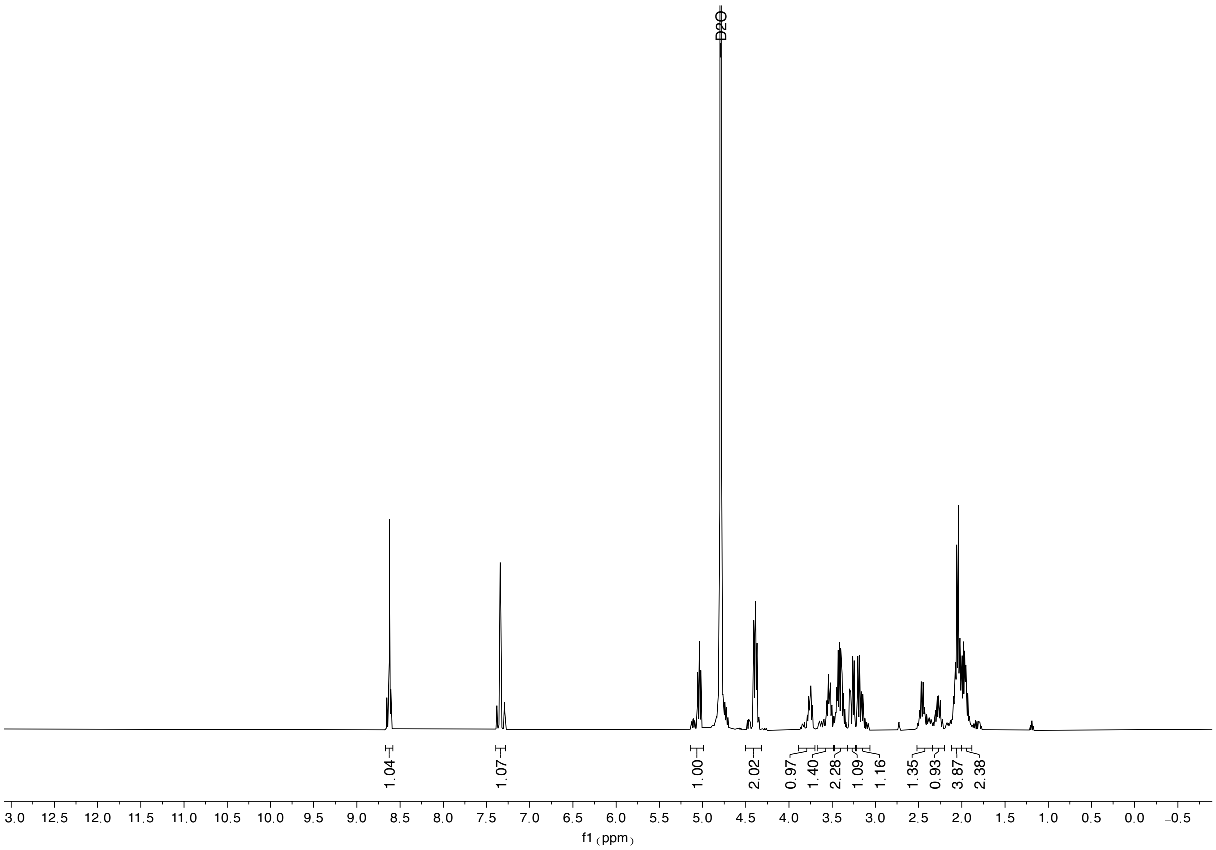

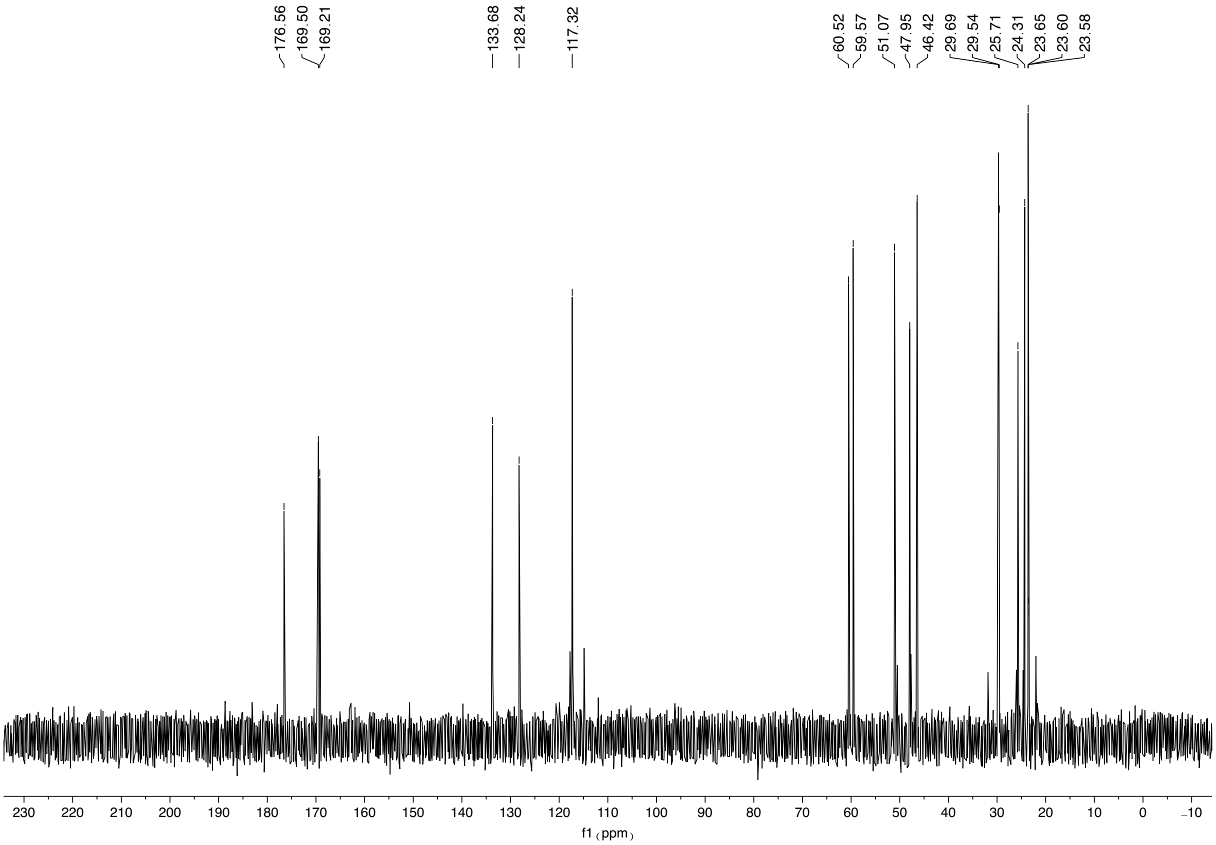

^{1}H and ^{13}C-NMR of H-D-Pro-D-His-L-(4*S*)-Hyp-NH$_2$ · TFA (P(4)-3):

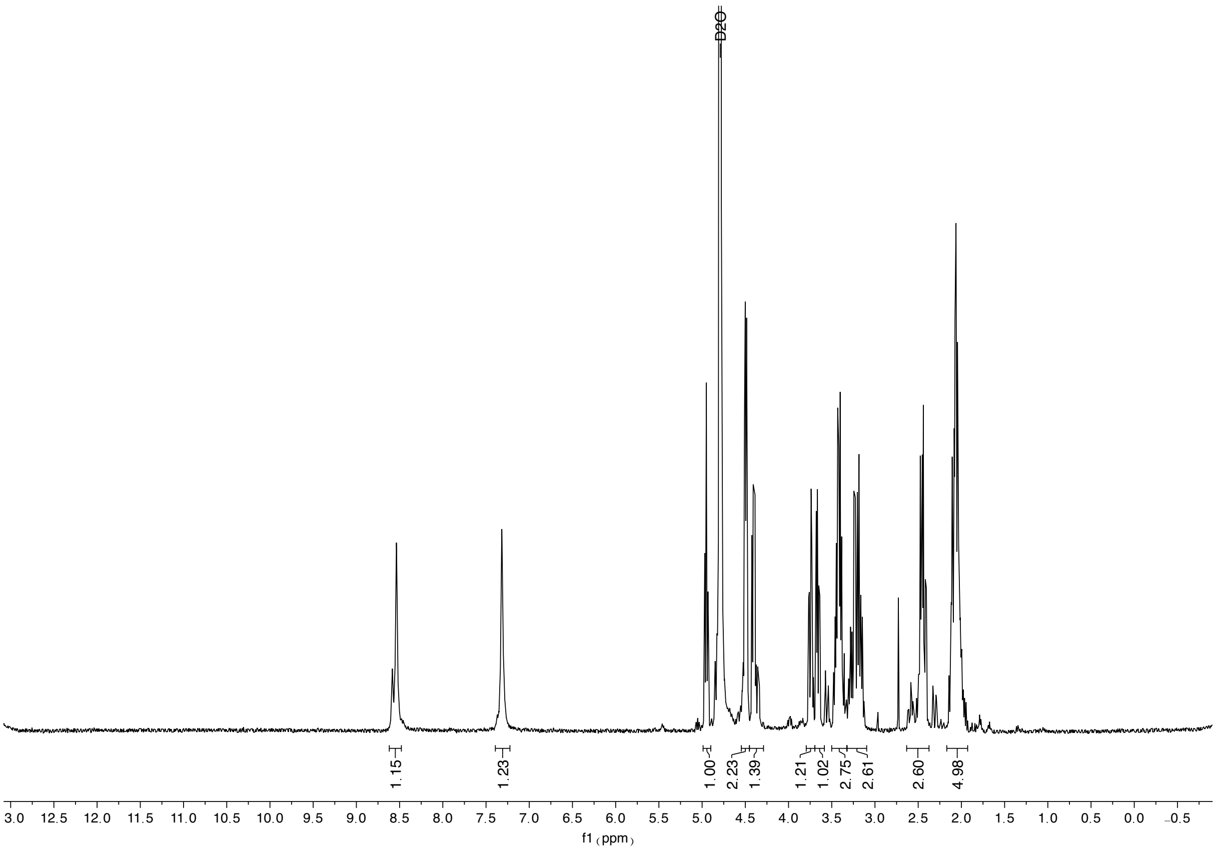

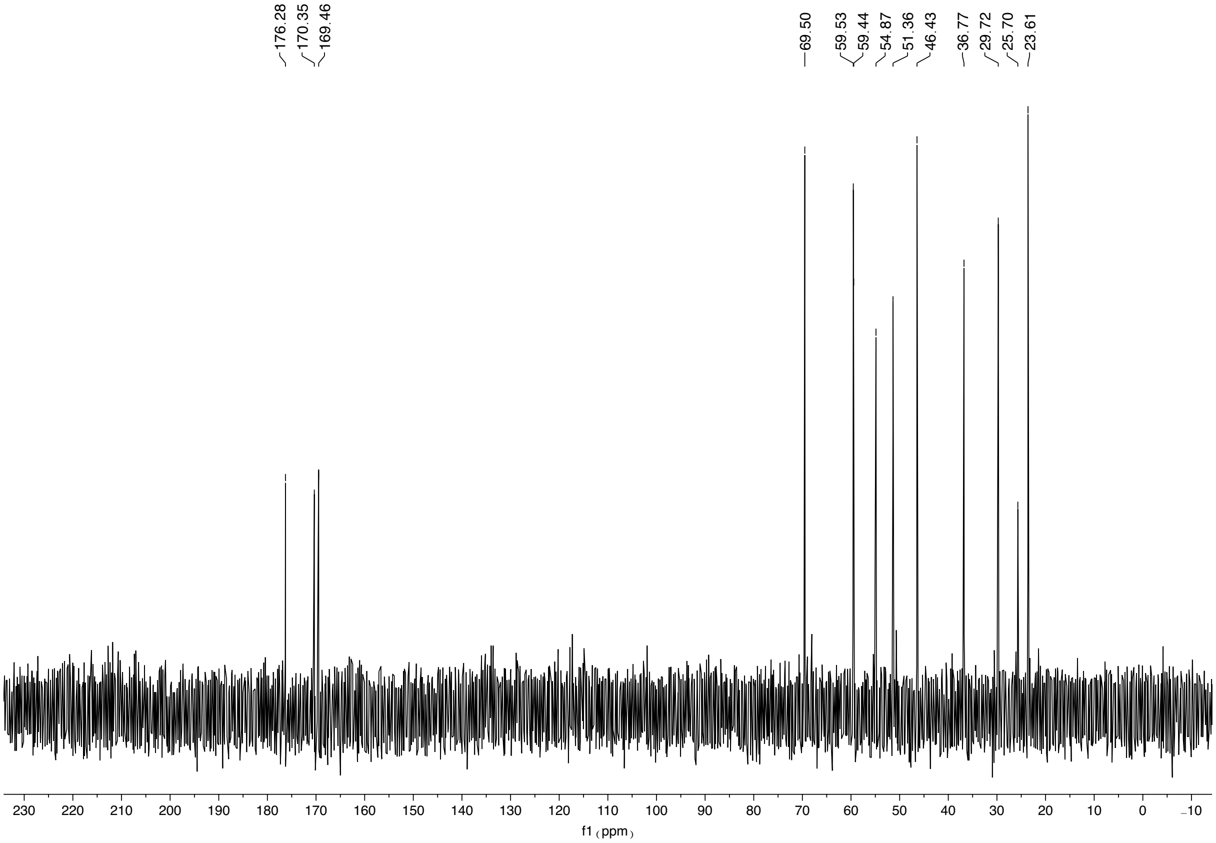

^{1}H and ^{13}C-NMR of H-D-Pro-D-*iso*Leu-L-(4*S*)-Hyp-NH₂ · TFA (P(4)-4):

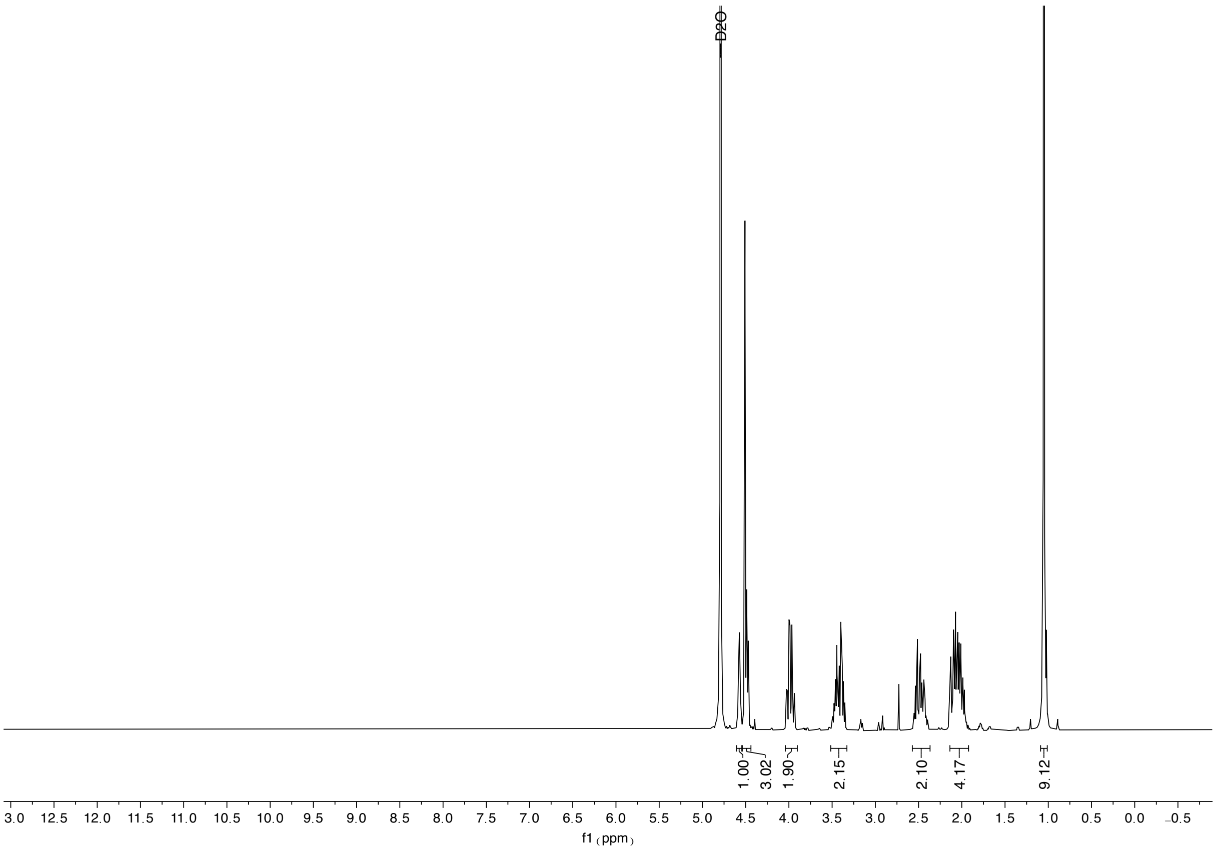

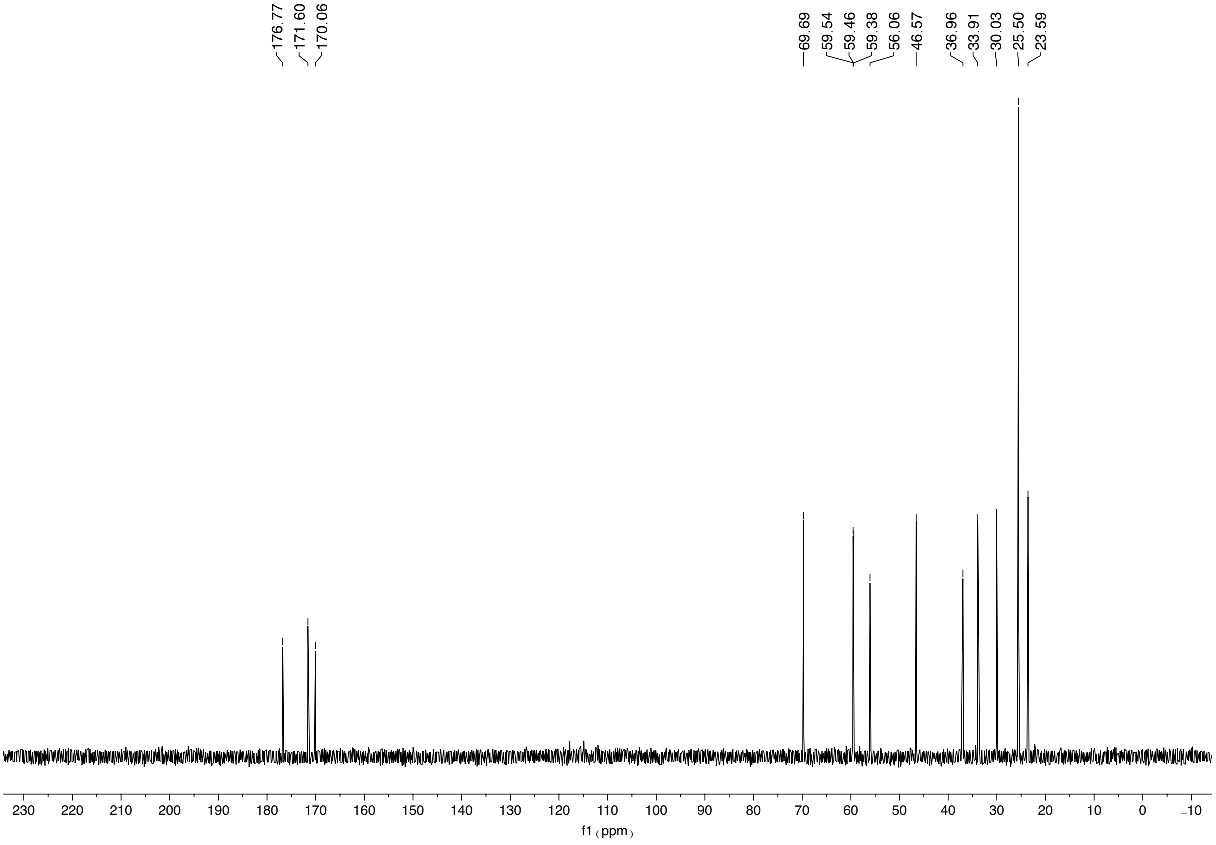

^{1}H and ^{13}C-NMR of H-D-Pro-D-Gln-L-(4*S*)-Hyp-NH_2 · TFA (P(4)-5):

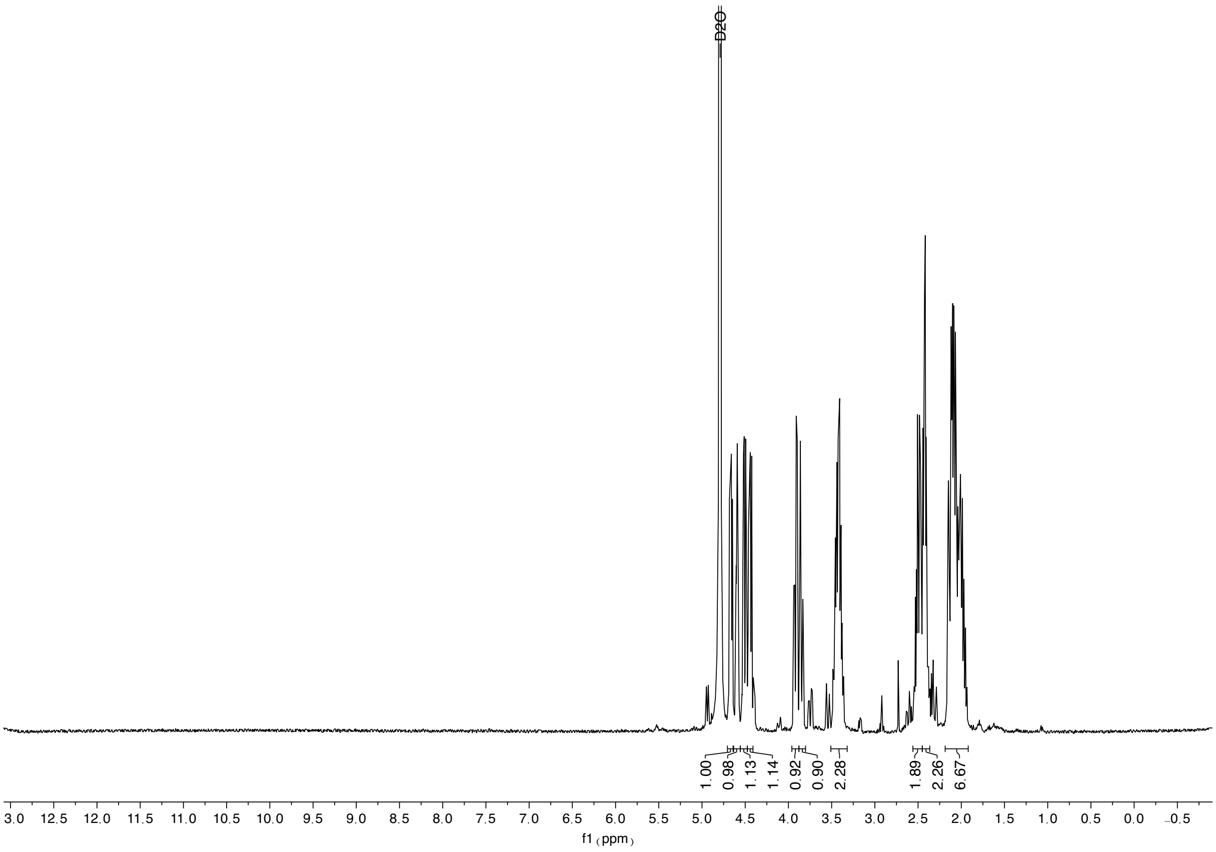

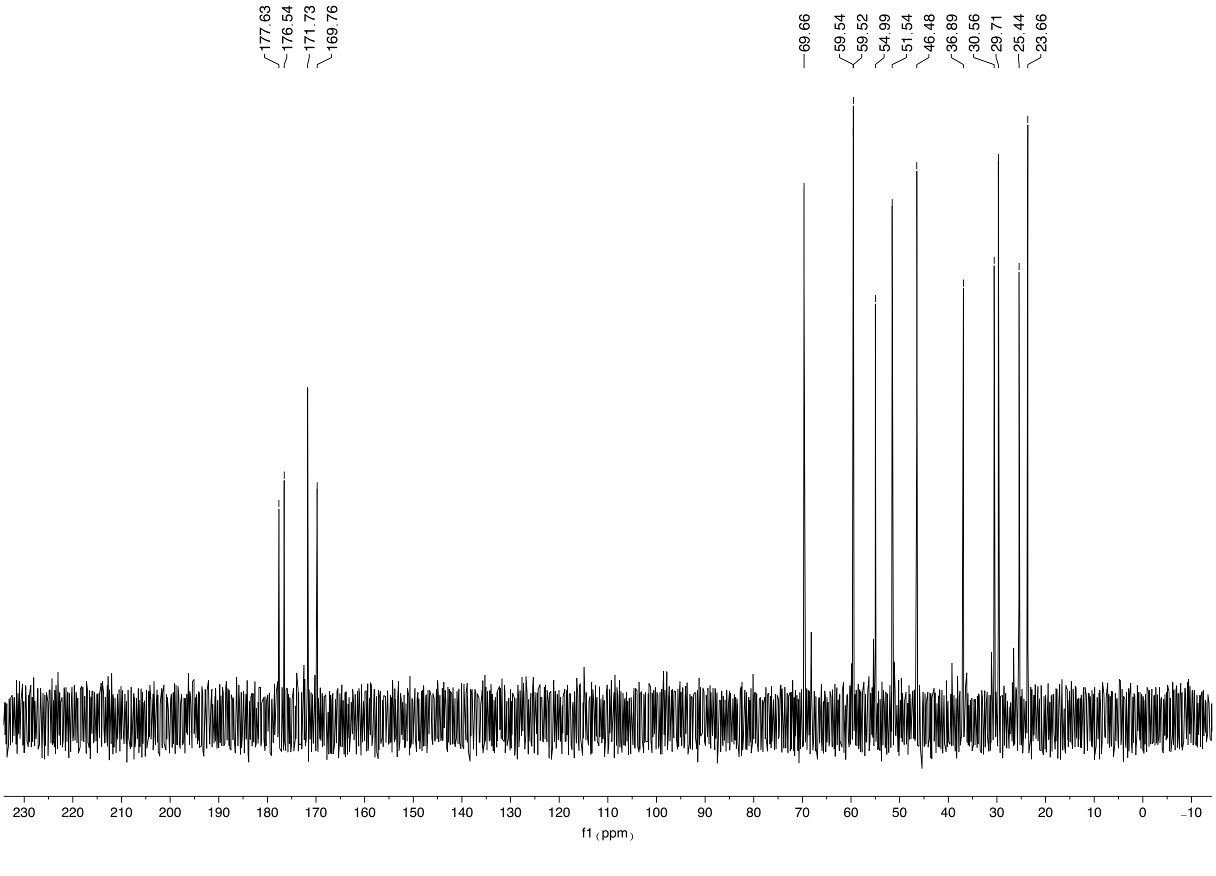

^{1}H and ^{13}C-NMR of H-D-Pro-D-*iso*Leu-L-Pro-NH$_2$ · TFA (P(4)-6):

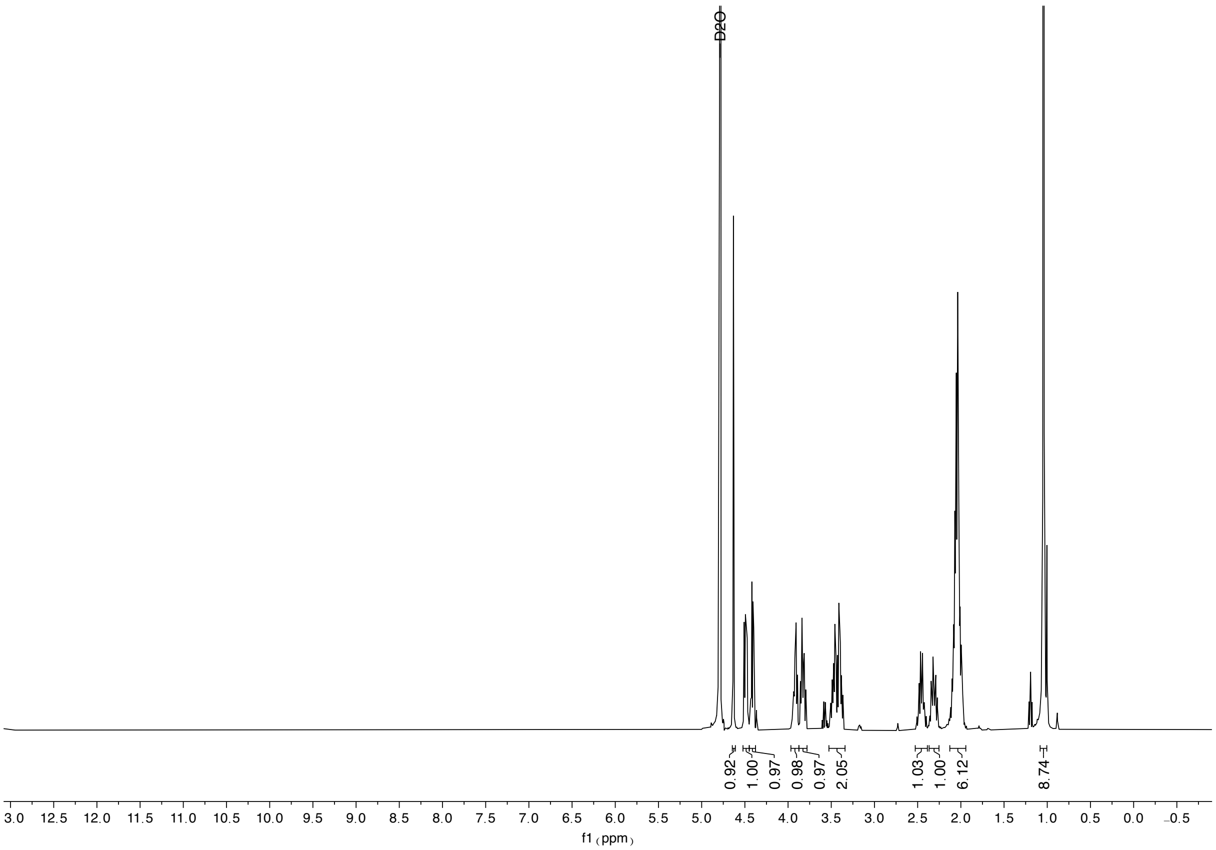

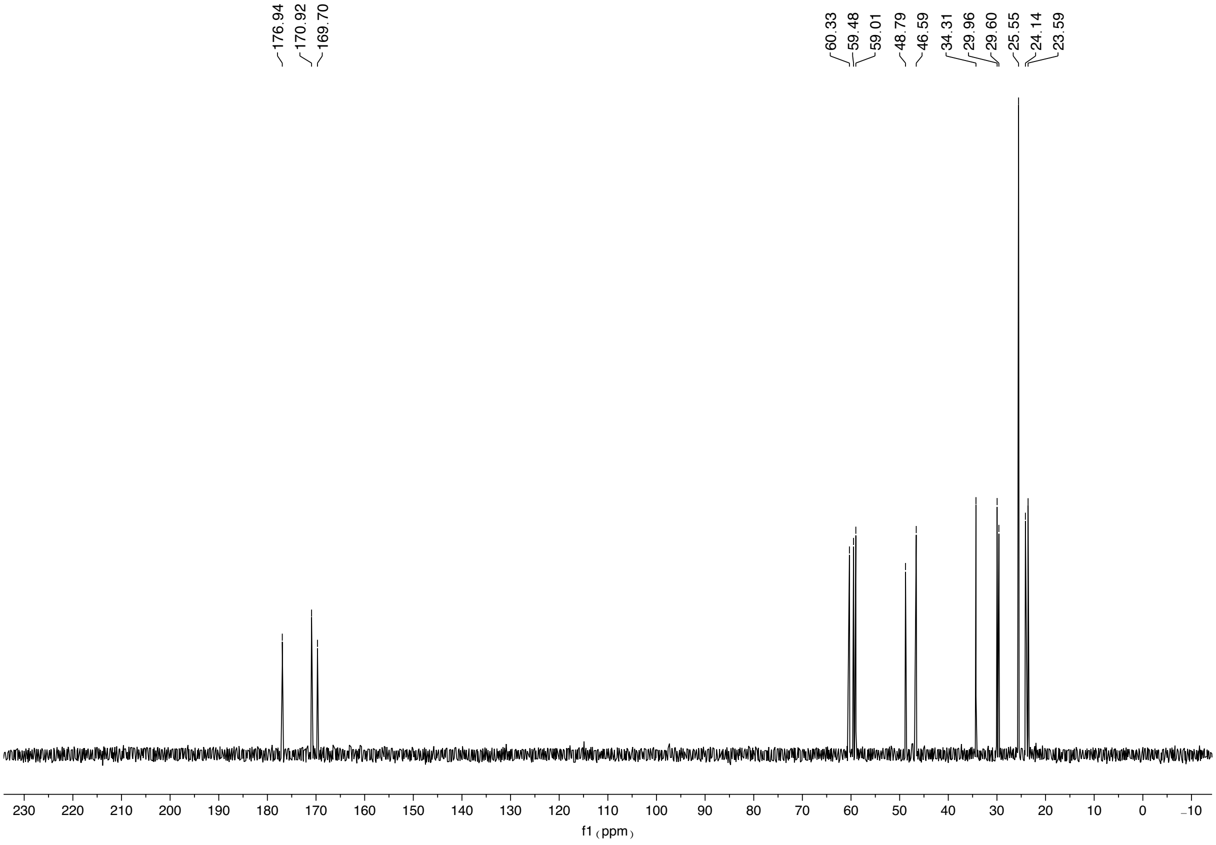

^{1}H and ^{13}C-NMR of H-D-Pro-D-Gln-L-*iso*Leu-NH$_2$ · TFA (P(4)-7):

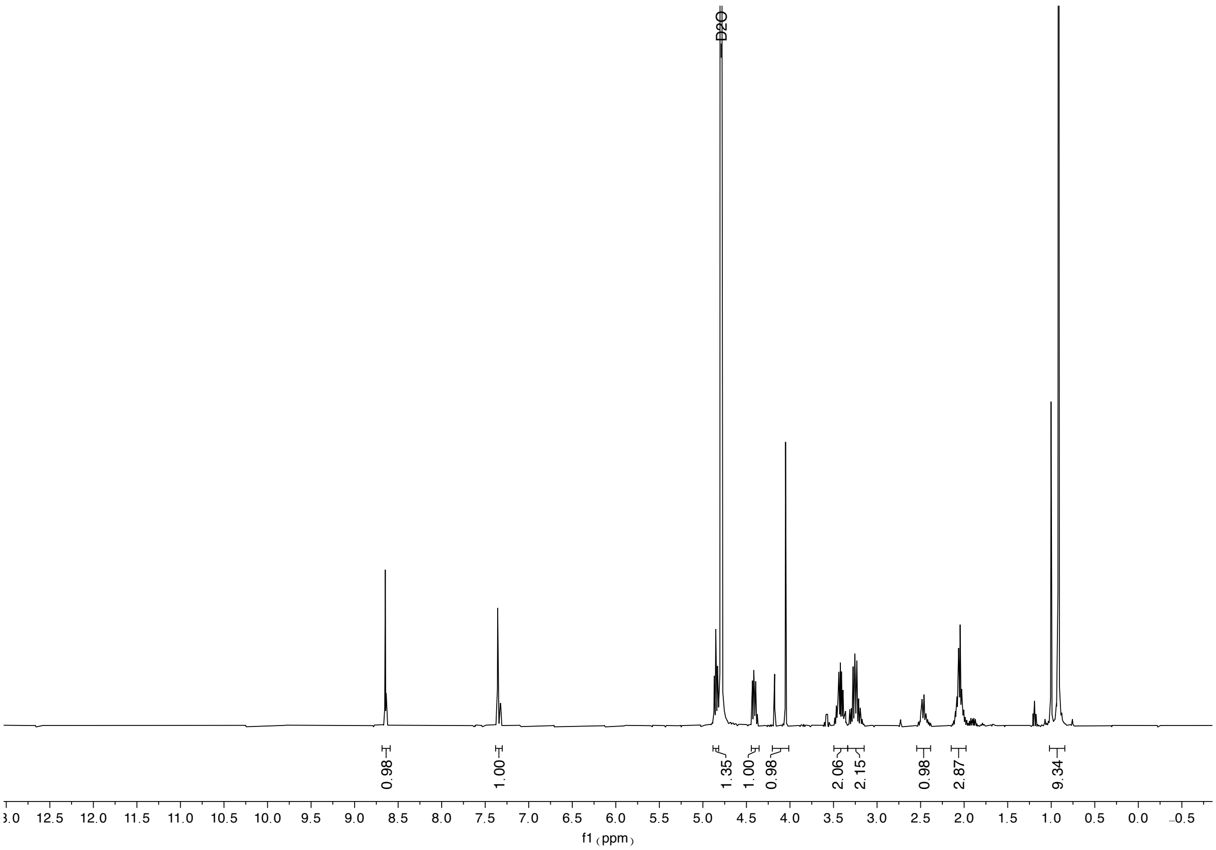

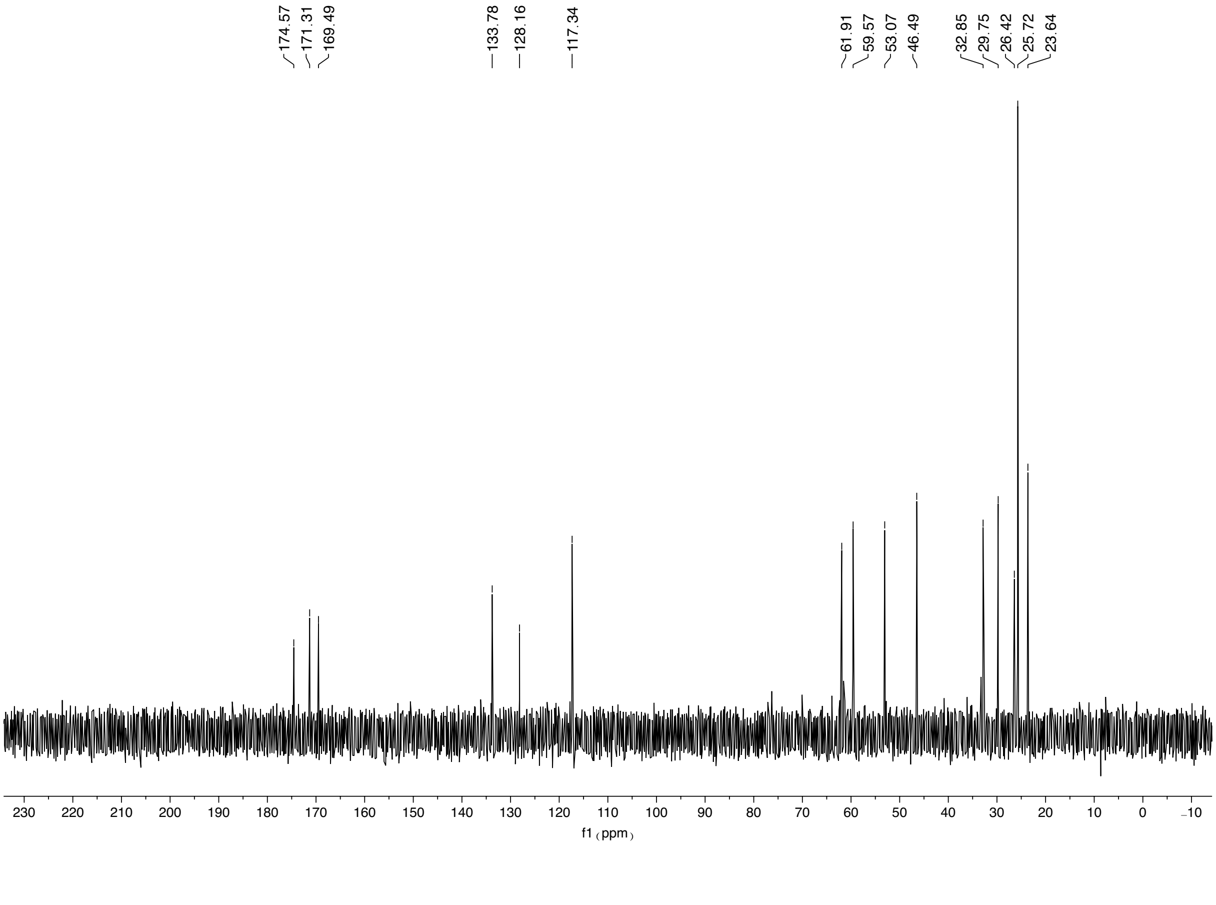

^{1}H and ^{13}C-NMR of H-L-Pro-L-His-D-*iso*Leu-NH_2 · TFA (P(4)-8):

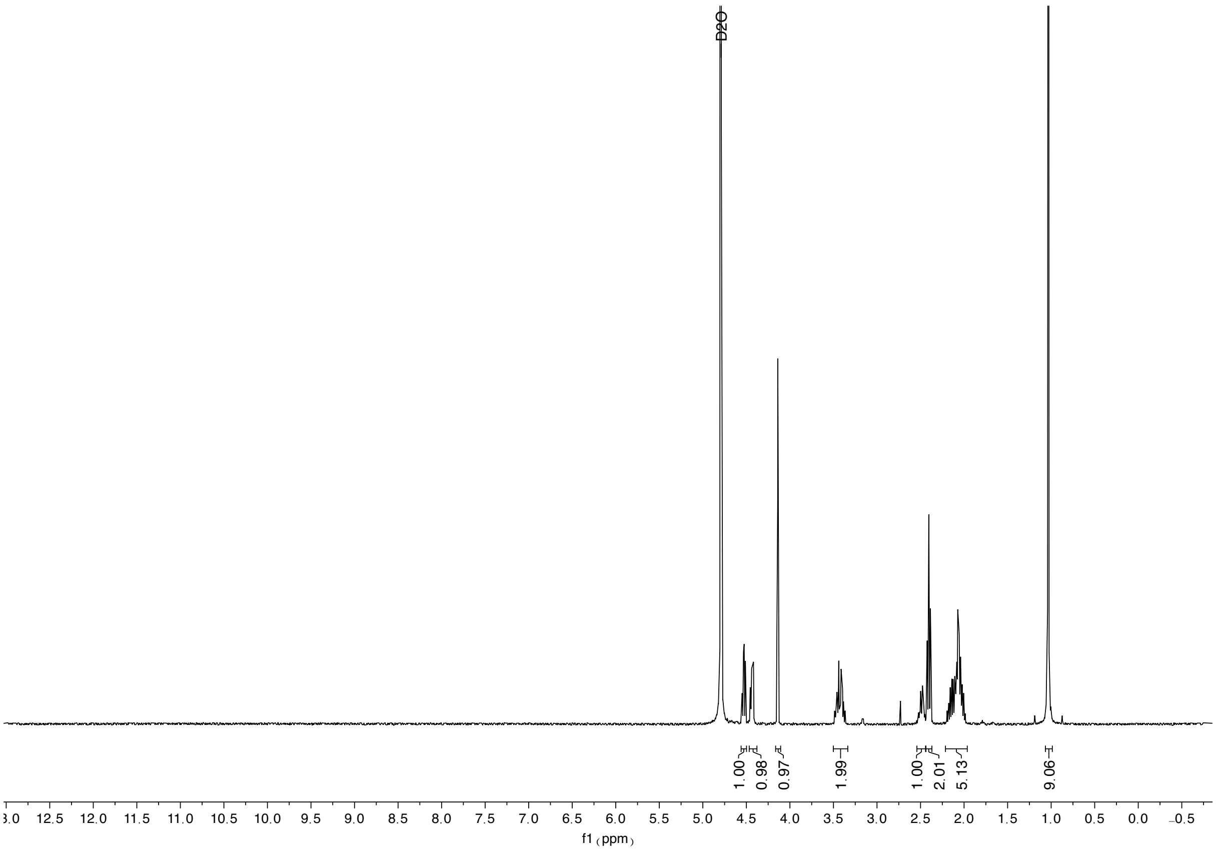

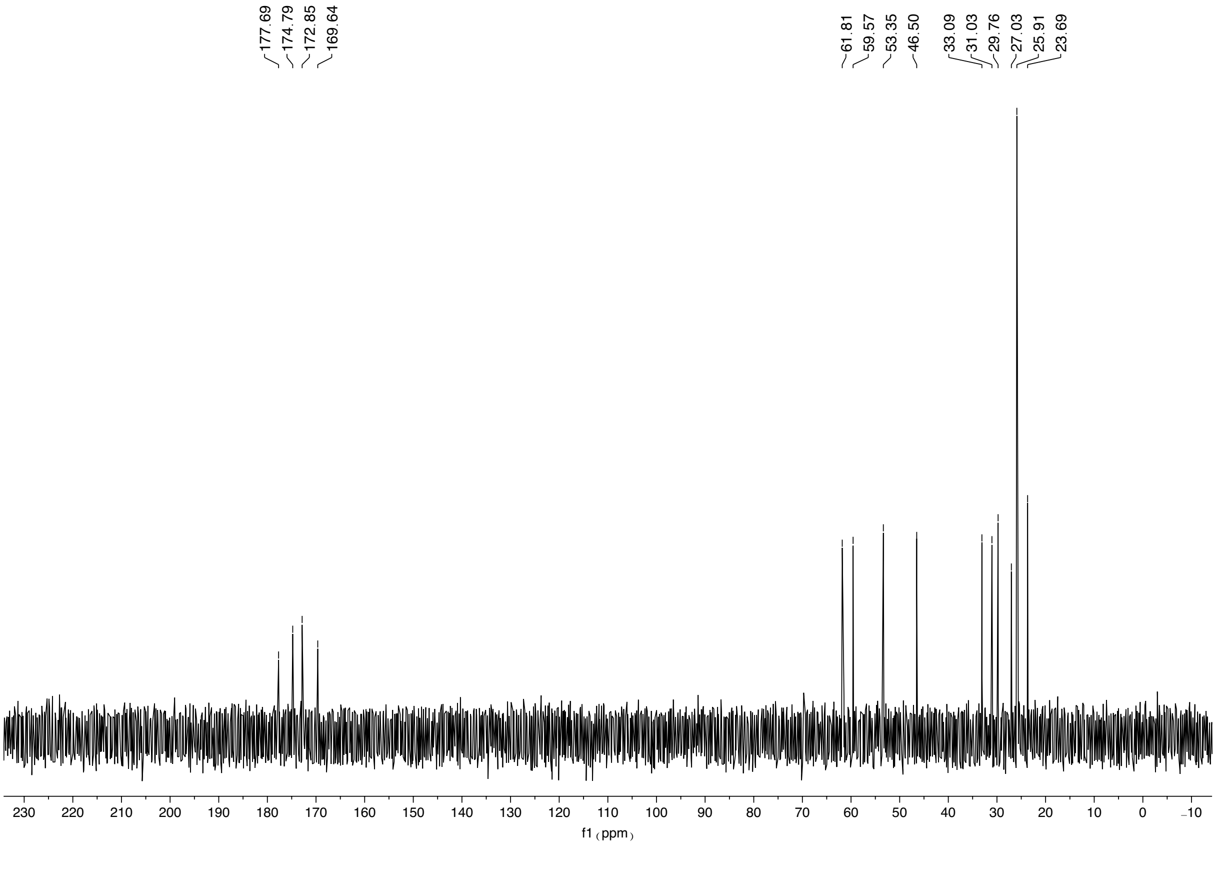

^{1}H and ^{13}C-NMR of H-D-Pro-D-His-L-4-MePhe-NH_2 · TFA (P(4)-9):

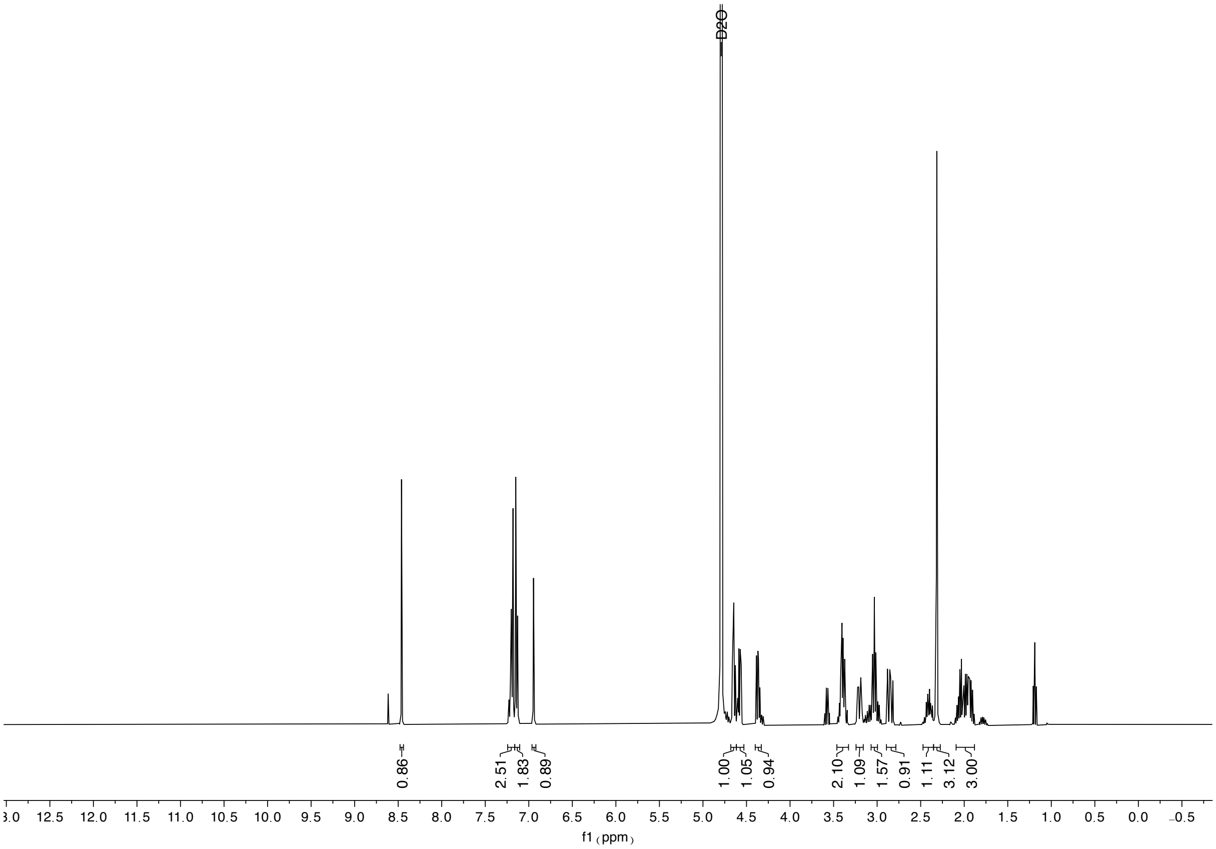

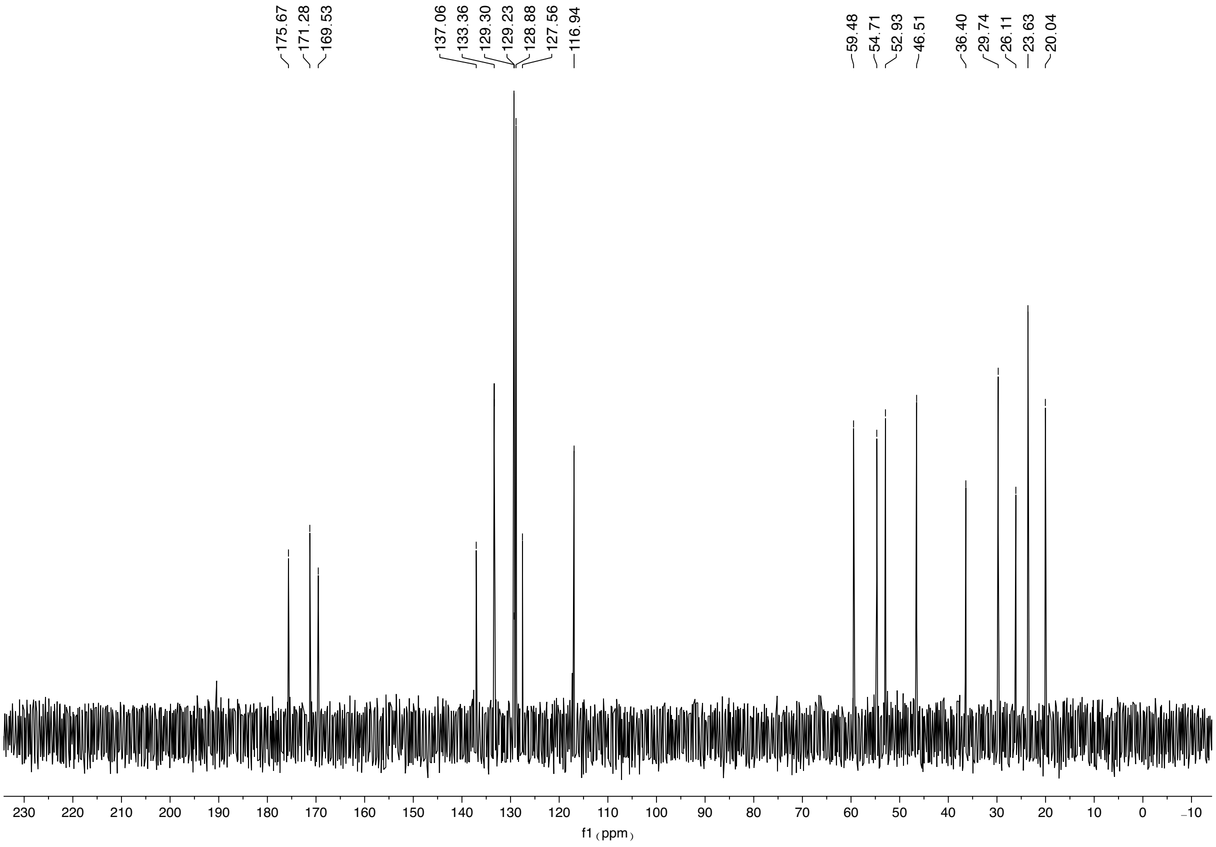

^{1}H and ^{13}C-NMR of H-D-Pro-D-Gln-L-Ill-NH$_2$ · TFA

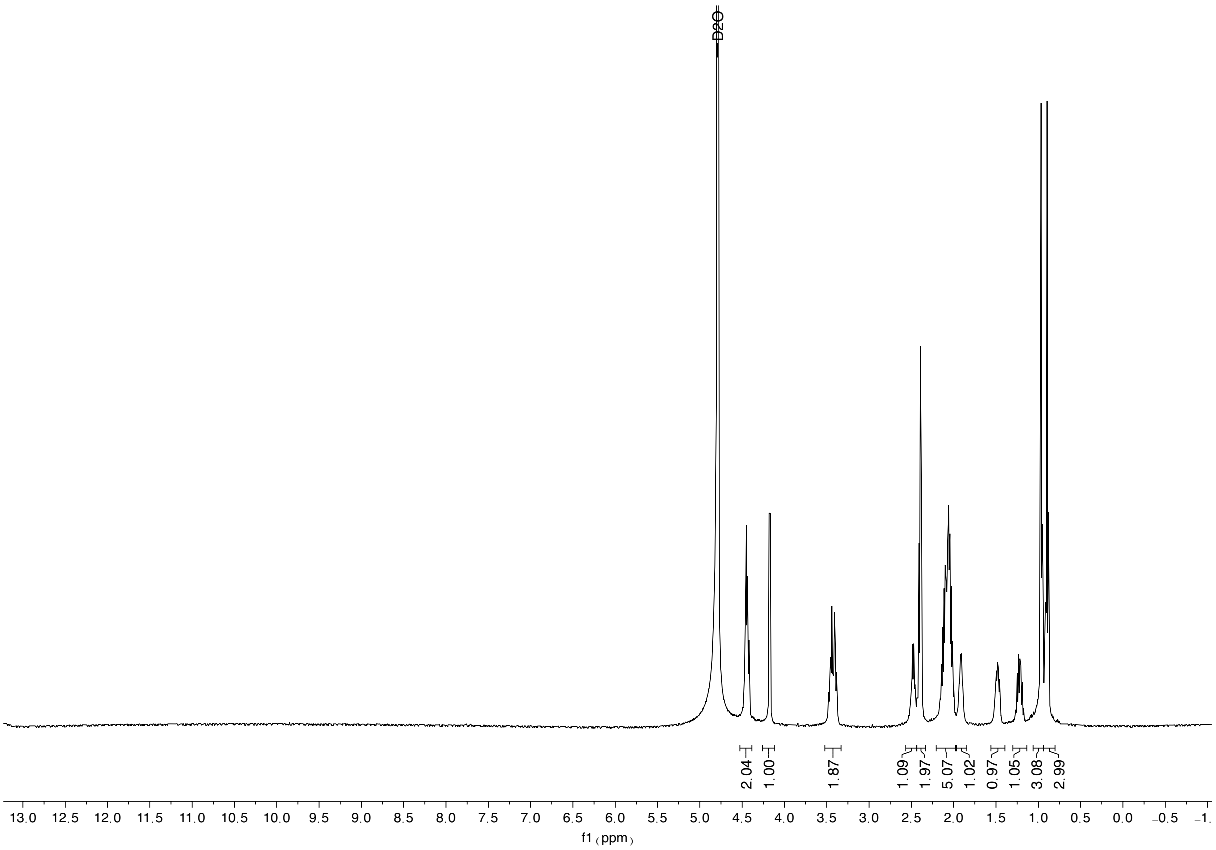

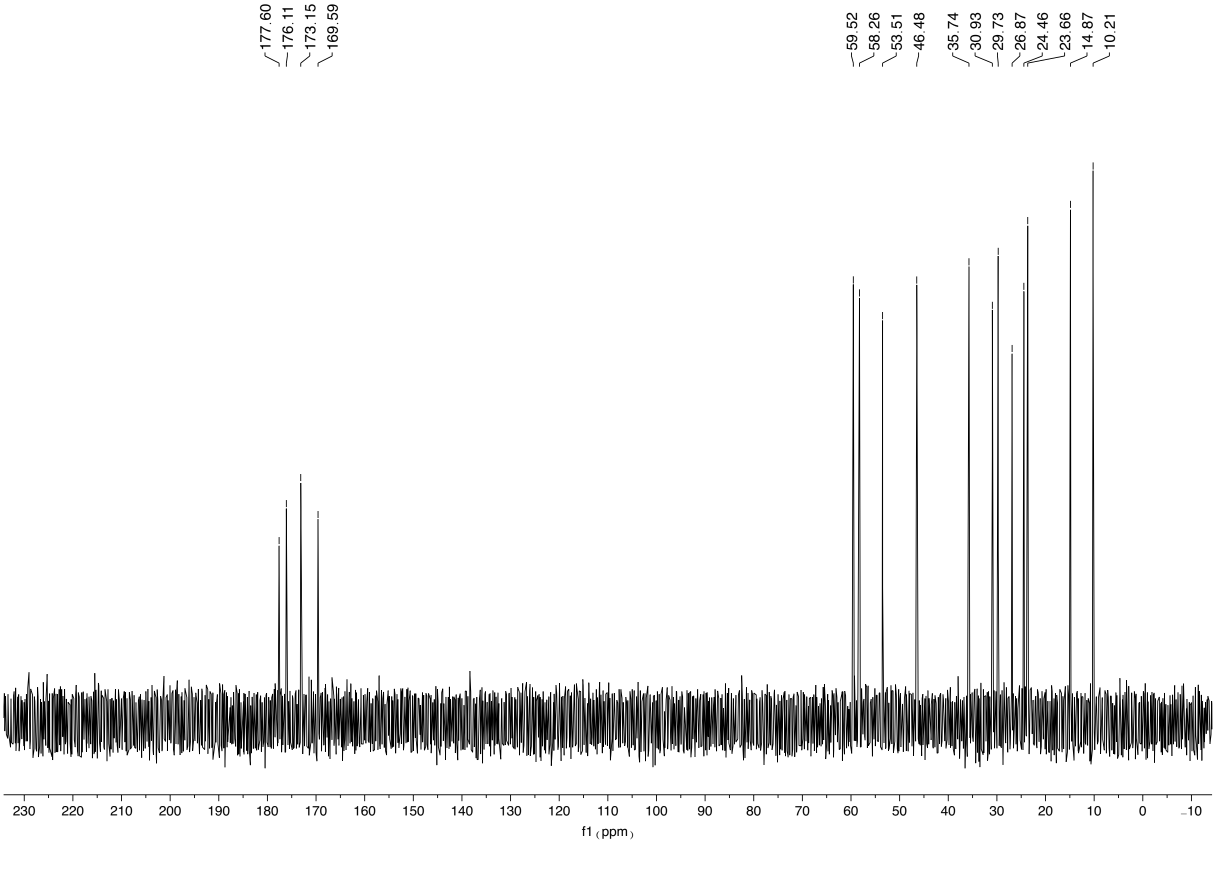

^{1}H and ^{13}C-NMR of H-D-Pro-D-Gln-L-Val-NH$_2$ · TFA

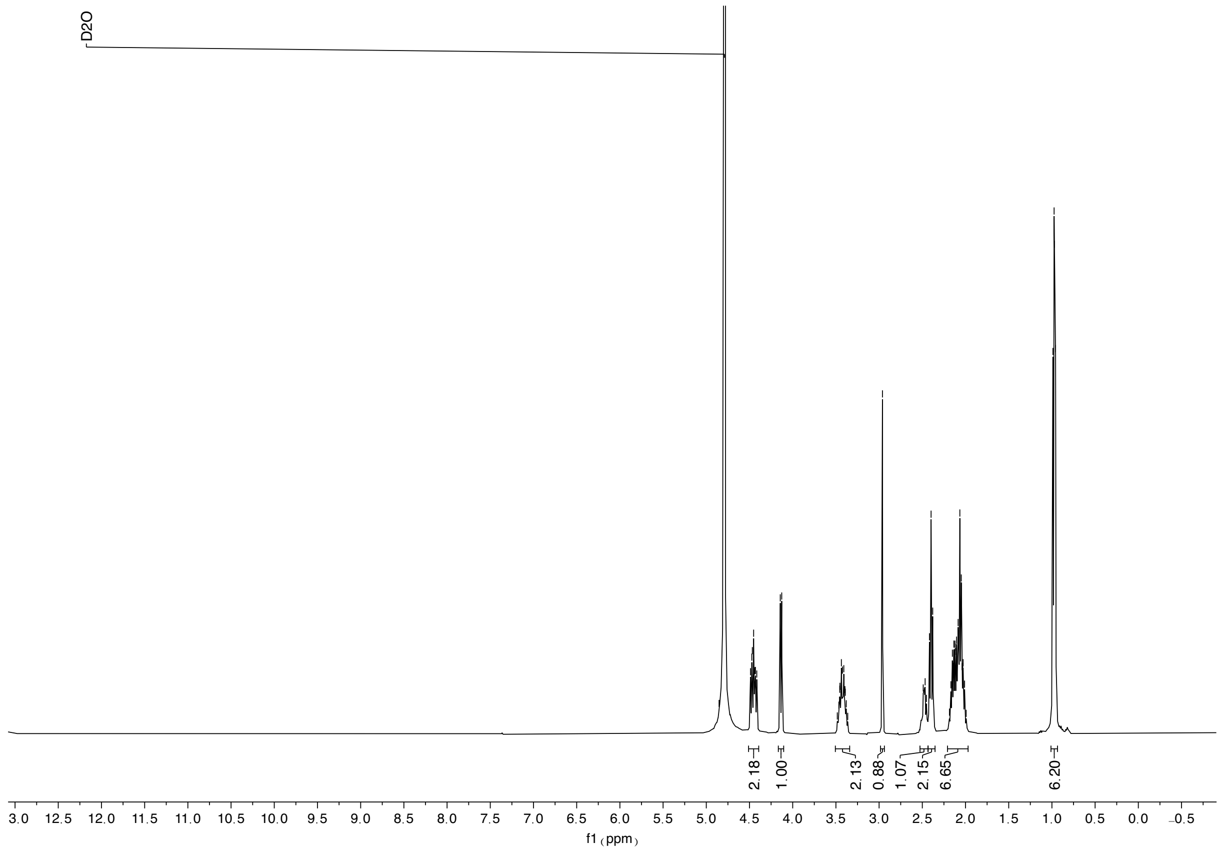

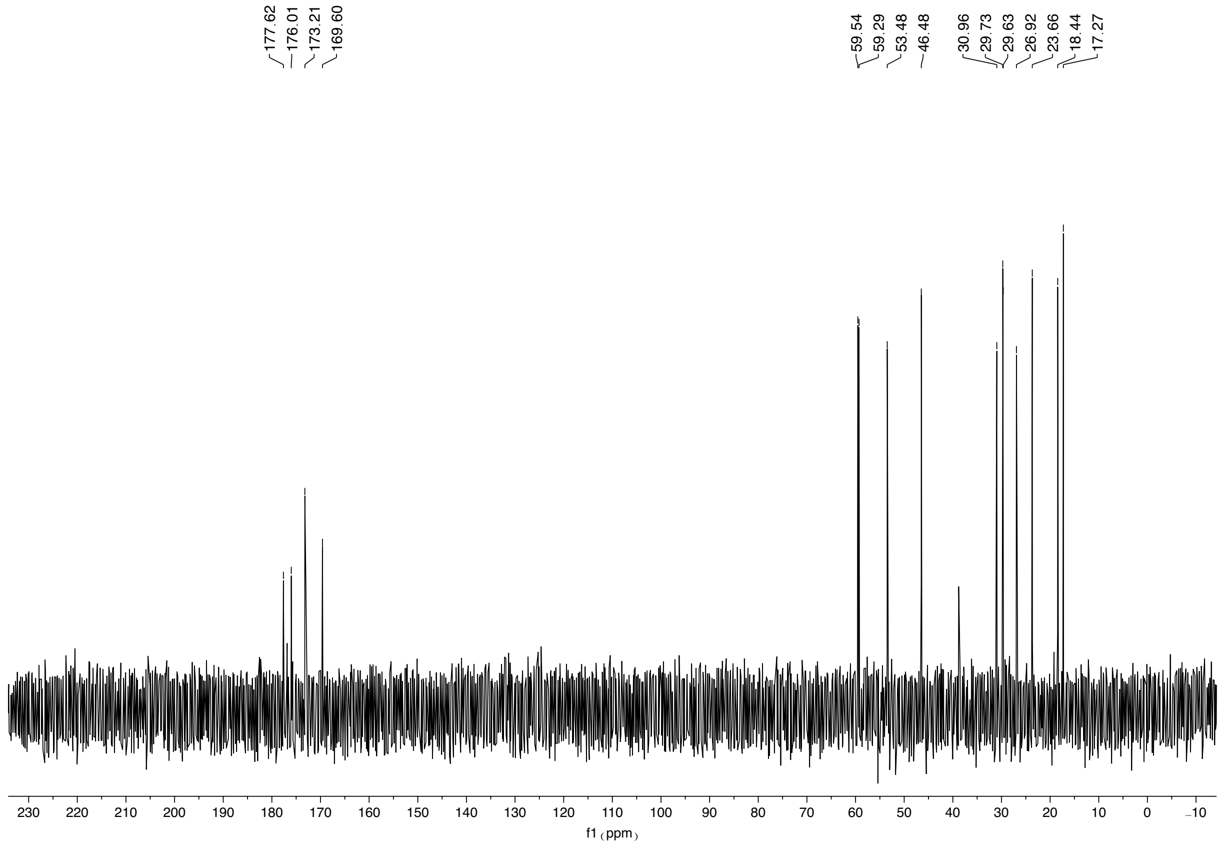

^{1}H and ^{13}C-NMR of H-D-Pro-D-Gln-L-Pro-NH$_2$

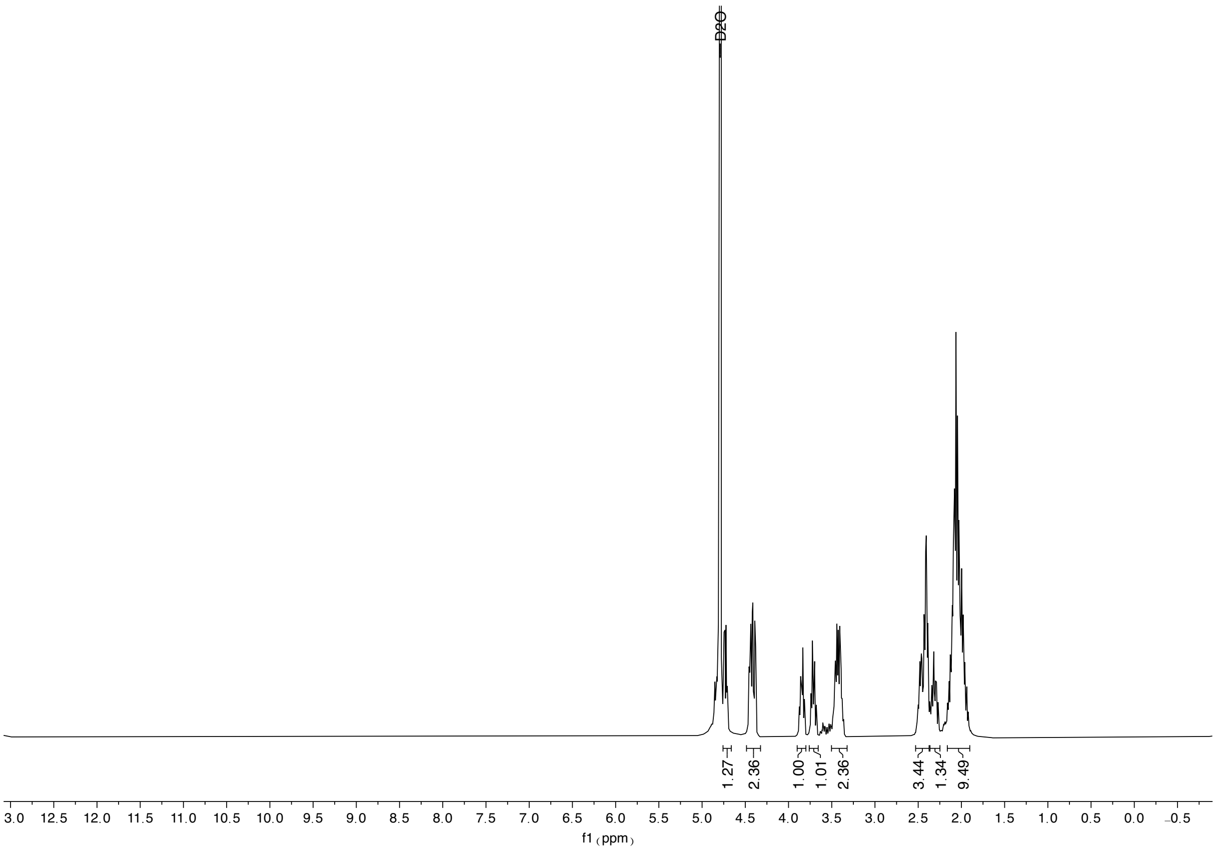

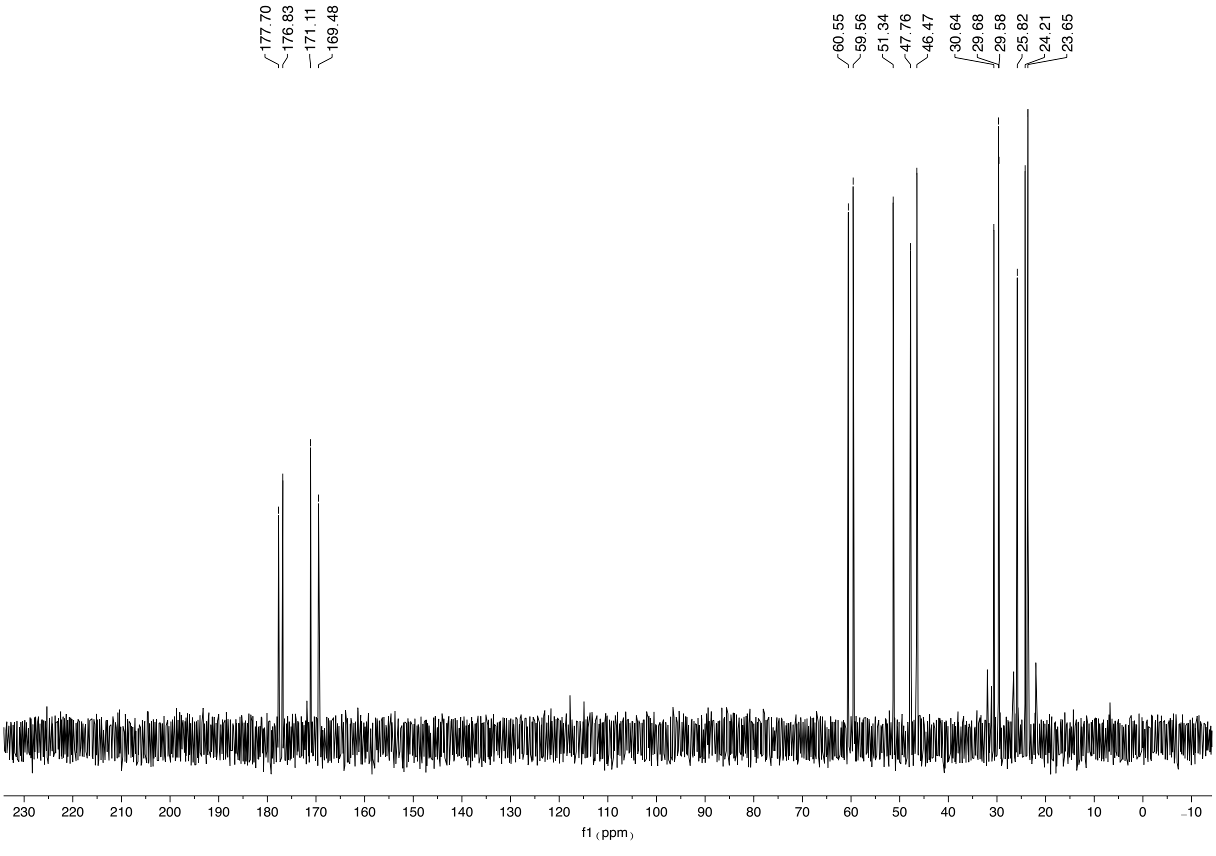

^{1}H and ^{13}C-NMR of H-D-Pro-D-Gln-L-Phe-NH_2 · TFA

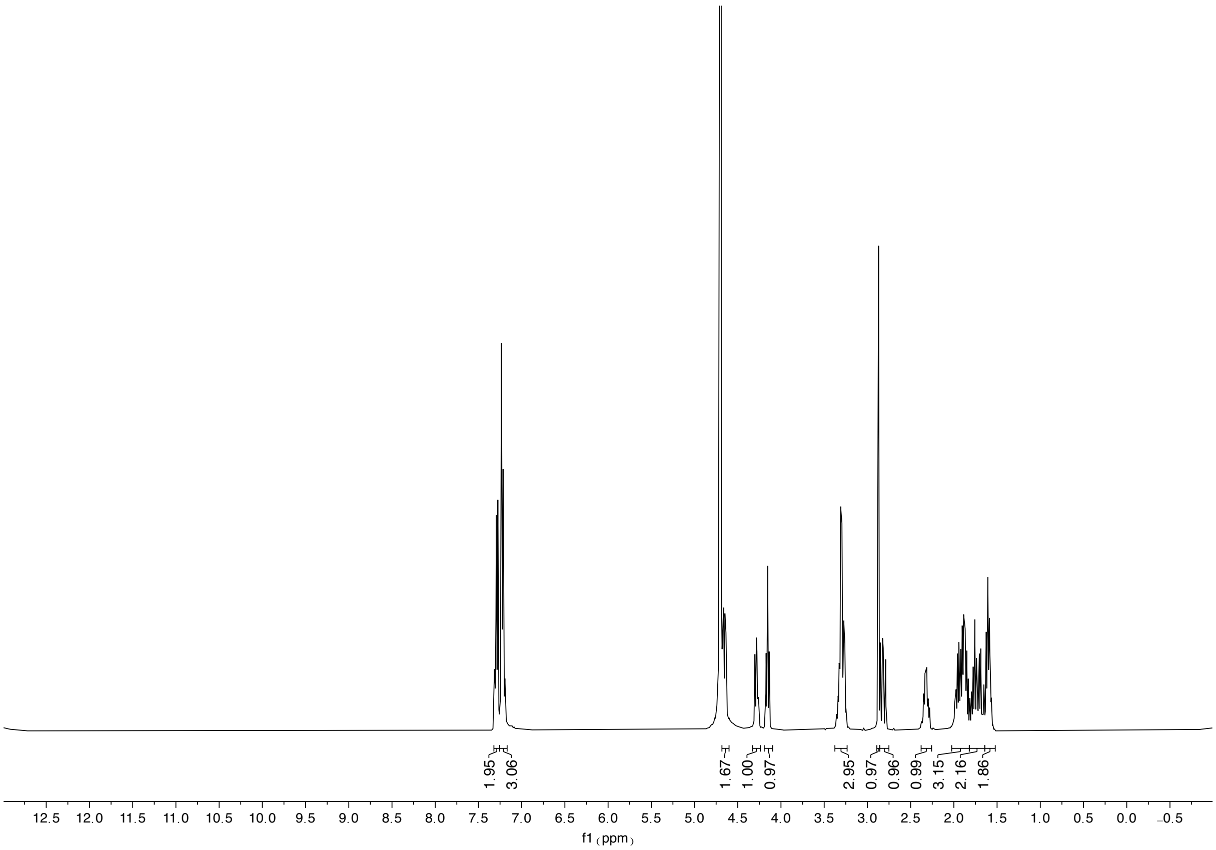

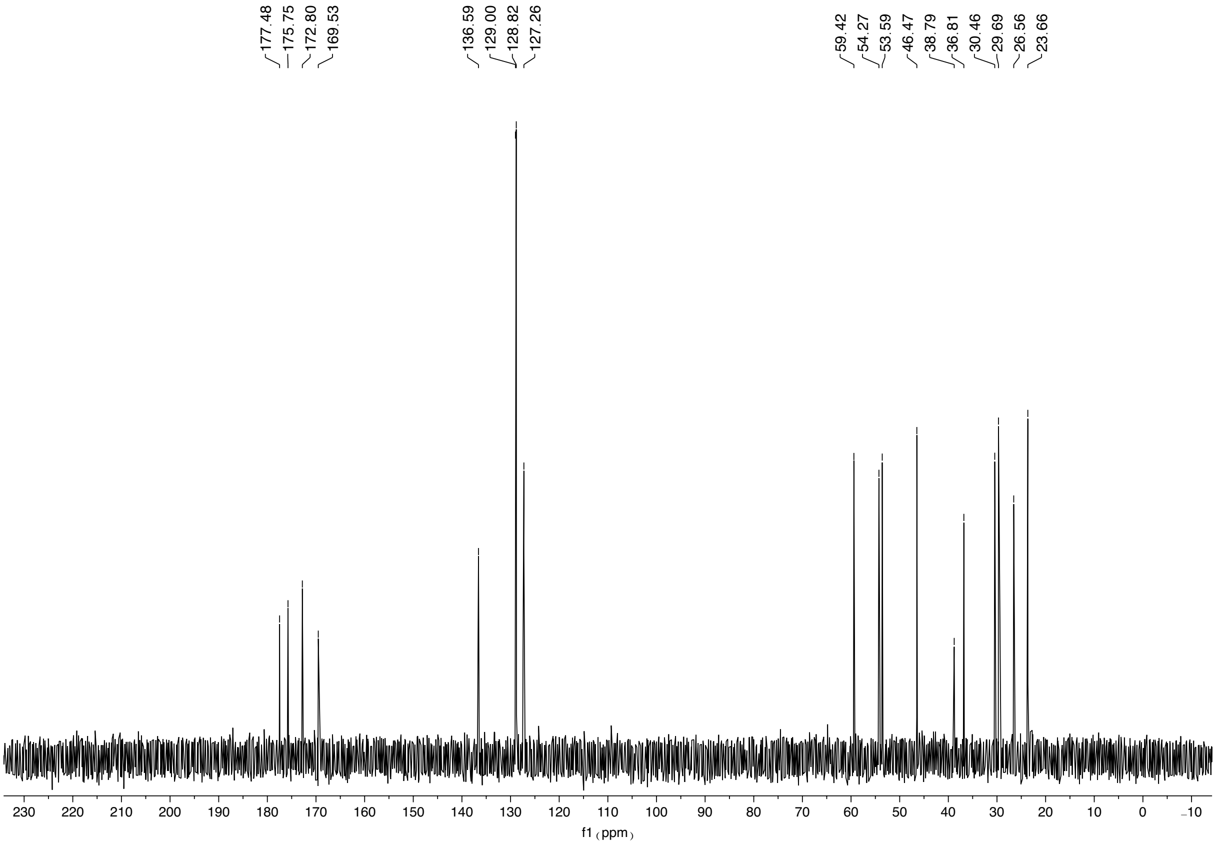

^{1}H and ^{13}C-NMR of H-D-Pro-D-Gln-L-Asn-NH_2 · TFA

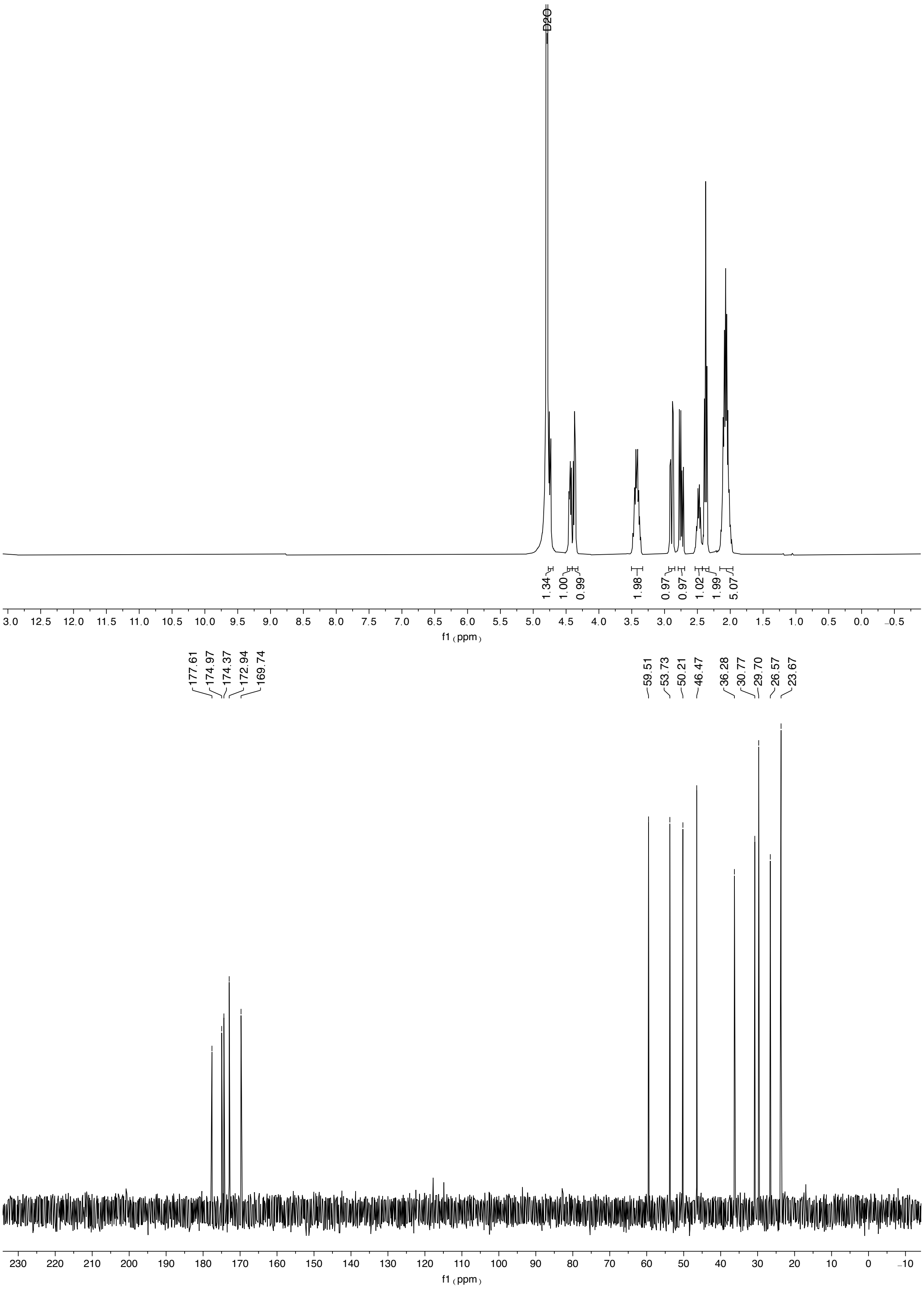

^{1}H and ^{13}C-NMR of H-D-Pro-D-Asn-L-Leu-NH₂

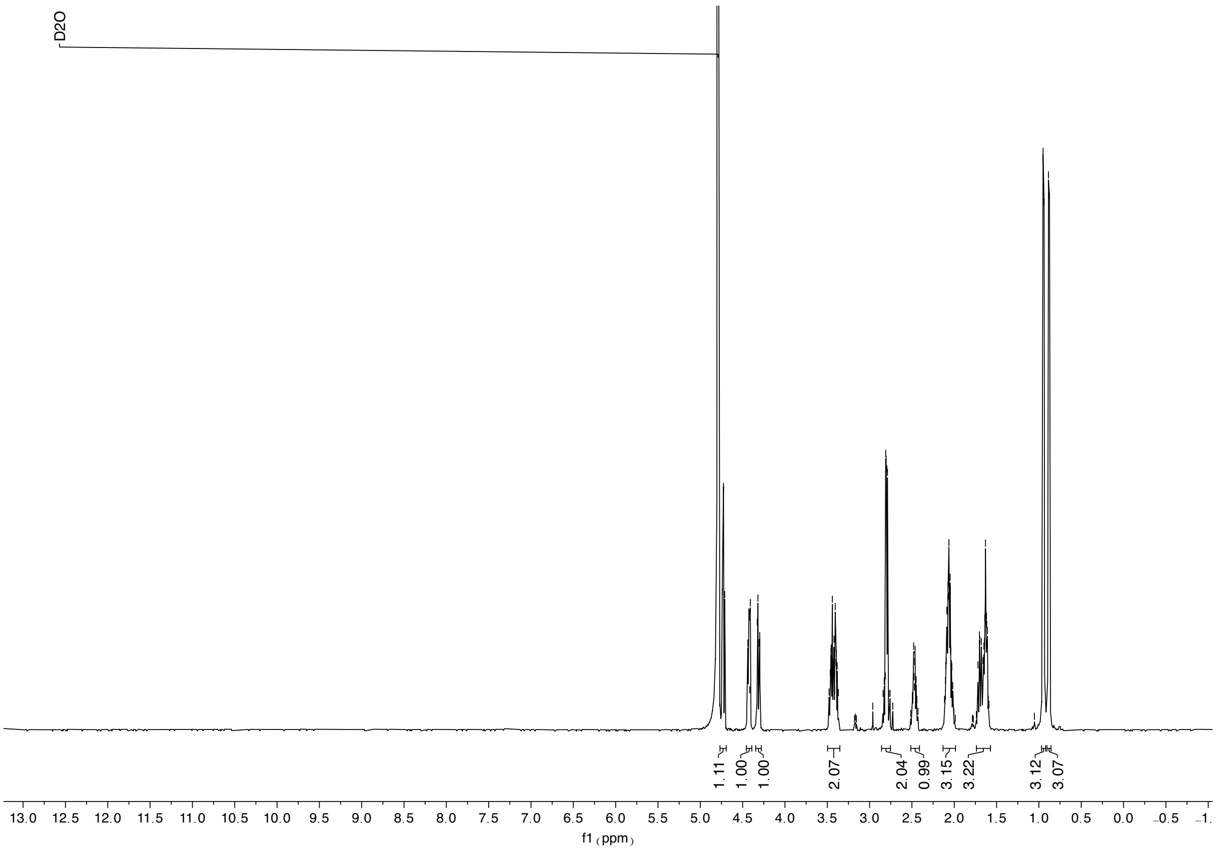

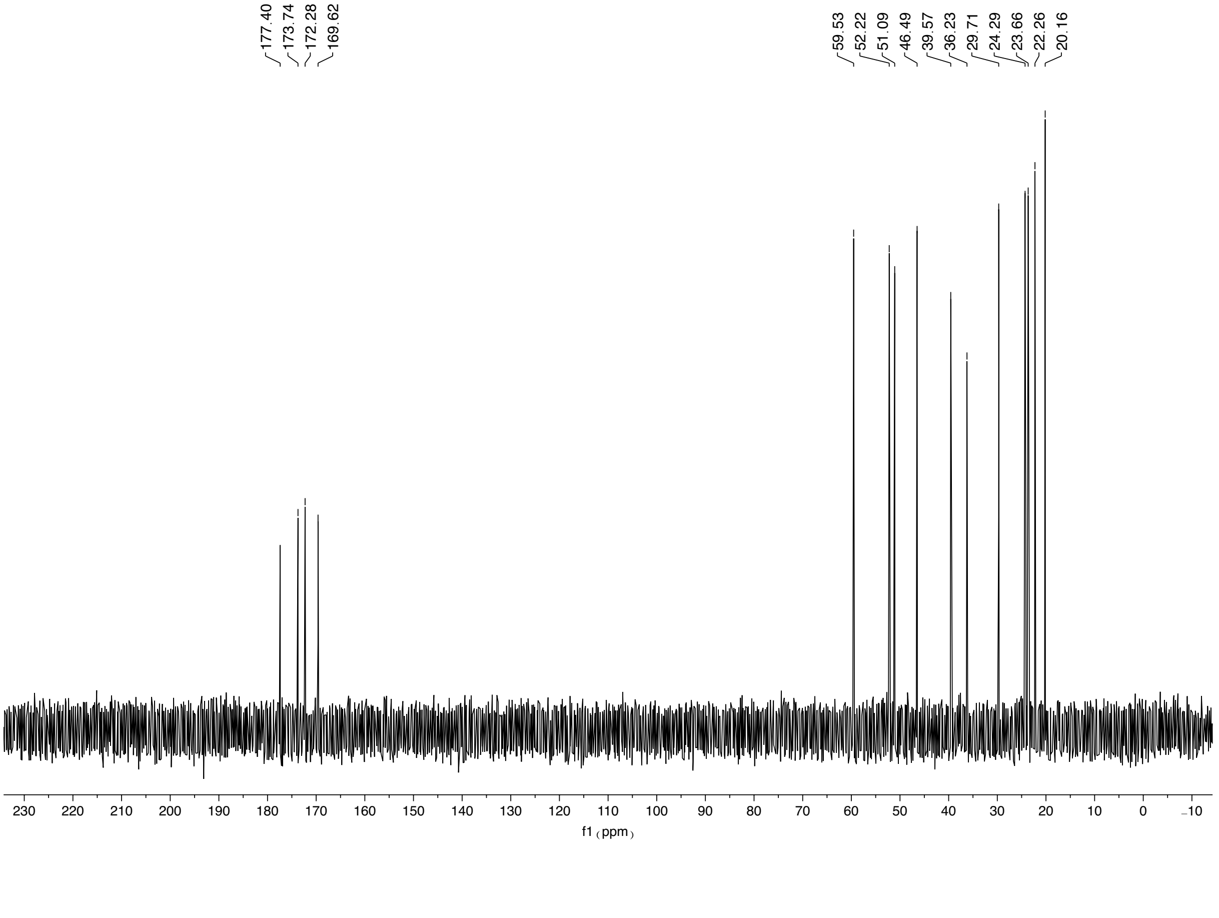

^{1}H and ^{13}C-NMR of H-D-Pro-D-His-L-Leu-NH$_2$ · TFA

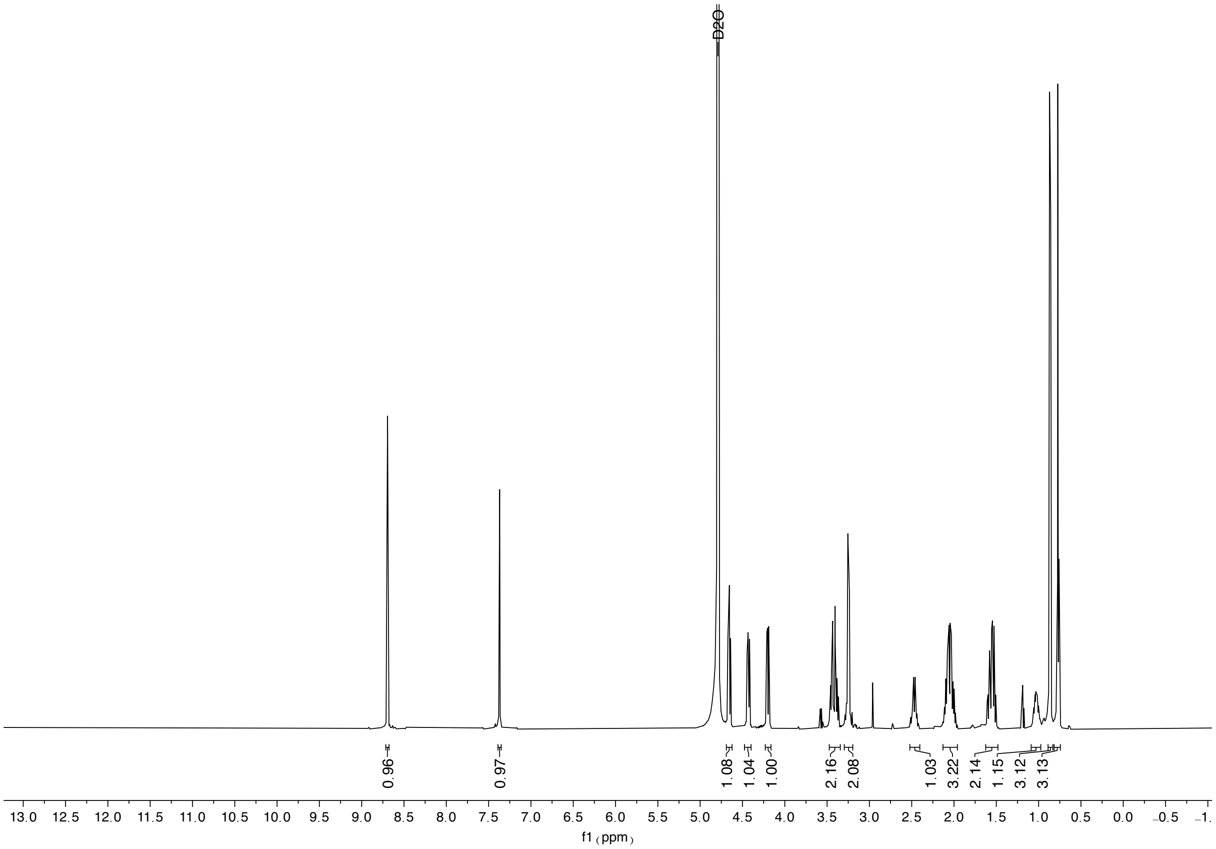

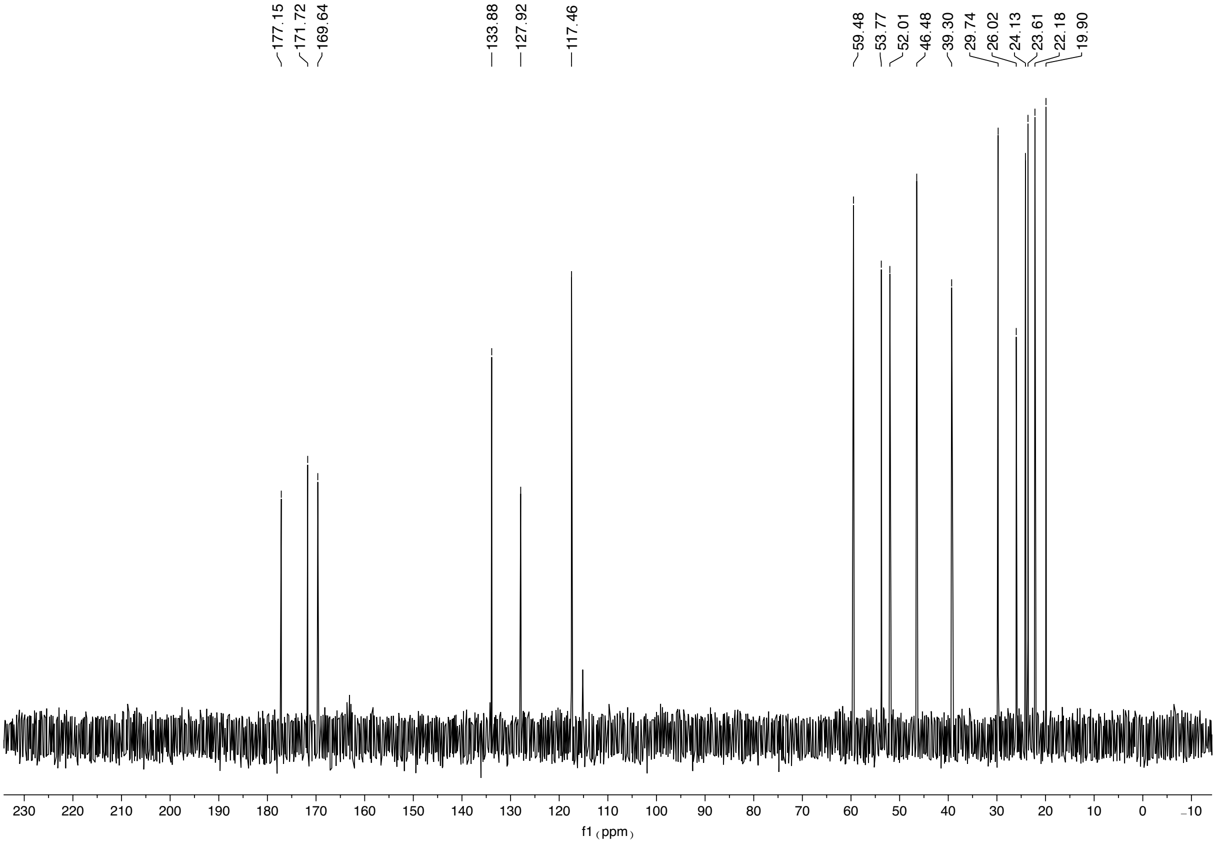

5. References

(1) Krattiger, P.; Kovasy, R.; Revell, J. D.; Ivan, S.; Wennemers, H. Increased Structural Complexity Leads to Higher Activity: Peptides as Efficient and Versatile Catalysts for Asymmetric Aldol Reactions. *Org. Lett.* **2005**, *7*, 1101–1103. https://doi.org/10.1021/OL0500259.

(2) Wiesner, M.; Revell, J. D.; Wennemers, H. Tripeptides as Efficient Asymmetric Catalysts for 1,4-Addition Reactions of Aldehydes to Nitroolefins - A Rational Approach. *Angewandte Chemie - International Edition* **2008**, *47*, 1871–1874. https://doi.org/10.1002/anie.200704972.

(3) Wiesner, M.; Neuburger, M.; Wennemers, H. Tripeptides of the Type H-D-Pro-Pro-Xaa-NH_2 as Catalysts for Asymmetric 1,4-Addition Reactions: Structural Requirements for High Catalytic Efficiency. *Chemistry - A European Journal* **2009**, *15*, 10103–10109. https://doi.org/10.1002/chem.200901021.

(4) Schnitzer, T.; Rackl, J. W.; Wennemers, H. Stereoselective Peptide Catalysis in Complex Environments - from River Water to Cell Lysates. *Chem Sci* **2022**, *13*, 8963–8967. https://doi.org/10.1039/d2sc02044k.

(5) Schnitzer, T.; Wennemers, H. Effect of γ-Substituted Proline Derivatives on the Performance of the Peptidic Catalyst H-DPro-Pro-Glu-NH2. *Synthesis (Stuttg)* **2018**, *50*, 4377–4382. https://doi.org/10.1055/s-0037-1609547.

(6) Schnitzer, T.; Wennemers, H. Influence of the Trans/Cis Conformer Ratio on the Stereoselectivity of Peptidic Catalysts. *J Am Chem Soc* **2017**, *139*, 15356–15362. https://doi.org/10.1021/jacs.7b06194.

(7) Revell, J. D.; Wennemers, H. Investigating Sequence Space: How Important Is the Spatial Arrangement of Functional Groups in the Asymmetric Aldol Reaction Catalyst H-Pro-Pro-Asp-NH2? *Adv Synth Catal* **2008**, *350*, 1046–1052. https://doi.org/10.1002/adsc.200800053.

(8) Schnitzer, T.; Wennemers, H. Thieme Chemistry Journals Awardees – Where Are They Now? A Stereoselective Tripeptide Catalyst for Conjugate Addition Reactions of Acetophenones to Dicyanoolefins. *Synlett* **2017**, *28*, 1282–1286. https://doi.org/10.1055/s-0036-1588964.

(9) Grünenfelder, C. E.; Kisunzu, J. K.; Wennemers, H. Peptide-Catalyzed Stereoselective Conjugate Addition Reactions of Aldehydes to Maleimide. *Angewandte Chemie International Edition* **2016**, *55*, 8571–8574. https://doi.org/10.1002/anie.201602230.

(10) Johansen, T. K.; Villegas Gomez, C.; Bak, J. R.; Davis, R. L.; Jørgensen, K. A. Organocatalytic enantioselective cycloaddition reactions of dienamines with quinones. *Chem. Eur. J.* **2013**, *19*, 16518-16522.

(11) Denmark Lab Chemoinformatics, ccheminfolib, Project ID 8113486, GitLab (2018); https://gitlab.com/SEDenmarkLab/ccheminfolib.

(12) Schrödinger Release 2019-4: MacroModel, Schrödinger, LLC, New York, NY, 2019.

(13) Henle, J.J.; Zahrt, A.F.; Rose, B.T.; Darrow, W.T.; Denmark, S.E. Development of a Computer-Guided Workflow for Catalyst Optimization. Descriptor Validation, Subset Selection, and Training Set Analysis. *J. Am. Chem. Soc.* **2020**, *142*, 11578-11592. https://doi.org/10.1021/jacs.0c04715.

(14) O'Boyle, N. M.; Banck, M.; James, C. A.; Morley, C.; Vandermeersch, T.; Hutchison, G.R. Open Babel: An Open Chemical Toolbox. *J. Cheminforma*. **2011**, *33*, https://doi.org/10.1186/1758-2946-3-33.

(15) Scikit-learn: Machine Learning in Python, *JMLR,* **2011**, *12*, 2825-2830.

(16) F. K. Chollet, GitHub. https://github.com/fchollet/keras.
